# Advancing abdominal surgery recovery implementation: a unified framework for intensified recovery protocols by the EUropean PErioperative MEdical Networking collaborative

**DOI:** 10.3389/fsurg.2026.1827678

**Published:** 2026-05-18

**Authors:** Orestis Ioannidis, Aikaterini Antonia Bourtzinakou, Jose M. Ramirez, Nicolò Fabbri, Javier Martínez Ubieto, Carlo Vittorio Feo, Antonio Pesce, Kristyna Rosetzka, Antonio Arroyo, Petr Kocián, Luis Sánchez-Guillén, Ana Pascual Bellosta, Adam Whitley, Alejandro Bona Enguita, Marta Teresa-Fernandéz, Stefanos Bitsianis, Savvas Symeonidis, Elissavet Anestiadou

**Affiliations:** 1 Fourth Department of Surgery, Faculty of Health Sciences, Medical School, General Hospital “George Papanikolaou”, Aristotle University of Thessaloniki, Thessaloniki, Greece; 2 Institute for Health Research Aragón, Zaragoza, Spain; 3 Department of Surgery, Faculty of Medicine, University of Zaragoza, Zaragoza, Spain.; 4 Department of Surgery, Azienda Unità Sanitaria Locale Ferrara-University of Ferrara, Ferrara, Italy; 5 Department of Anesthesia, Resuscitation and Pain Therapy, Miguel Servet University Hospital, Zaragoza, Spain; 6 Department of Plastic Surgery, Second Faculty of Medicine, Charles University and Motol University Hospital, Prague, Czechia; 7 Department of Surgery, Hospital General Universitario Elche, Universidad Miguel Hernández Elche, Elche, Spain; 8 Grupo Español de Rehabilitación Multimodal (GERM), Zaragoza, Spain; 9 Department of Surgery, Second Faculty of Medicine, Charles University and Motol University Hospital, Prague, Czechia; 10 Department of Surgery, University Hospital Kralovske Vinohrady, Prague, Czechia

**Keywords:** enhanced recovery after surgery (ERAS), EUPEMEN project, perioperative care, perioperative optimization, training and dissemination

## Abstract

**Objective:**

To present the structure, implementation strategy, and early outcomes of the EUropean PErioperative MEdical Networking (EUPEMEN) project, a multinational initiative designed to reduce variability in Enhanced Recovery After Surgery (ERAS) implementation across European surgical centers through a structured educational and protocol-driven framework.

**Methods:**

EUPEMEN was conducted as a 26-month, prospective, multinational implementation initiative funded by the Erasmus + Programme and involving five academic institutions across Spain, Italy, Greece, and the Czech Republic. The project included protocol development, structured education, and dissemination strategies. Seven multidisciplinary procedure-specific Enhanced Recovery modules were developed using a modular design to allow adaptation across different healthcare systems. All materials were translated into five languages to enhance accessibility. A digital learning platform was created to host ten educational modules incorporating multimedia content and knowledge assessment tools. A train-the-trainer Learning Teaching Activity (LTA) was organized to prepare institutional representatives for local and national dissemination and multiplier events took place in participating countries to promote project dissemination. Process evaluation included participation metrics, structured governance monitoring, and documentation of implementation activities within participating hospitals.

**Results:**

Seven standardized Enhanced Recovery protocols covering major general surgical domains were successfully developed and disseminated. The online educational platform provided structured training and interactive assessment to registered users. Ten trainers participated in the LTA and subsequently supported national-level educational activities. Five multiplier events engaged 366 healthcare professionals from multidisciplinary backgrounds. Enhanced Recovery implementation was achieved in all partner institutions with incorporation of monitoring elements for future adherence evaluation. Additionally, the Via RICA pathway was translated into English and Greek, further supporting international dissemination.

**Conclusion:**

The EUPEMEN project represents a coordinated, multilingual, and education-centered multinational strategy for harmonizing Enhanced Recovery implementation across heterogeneous healthcare systems. By integrating structured protocol design, train-the-trainer methodology, and digital educational infrastructure, the project provides a scalable framework for bridging the Enhanced Recovery implementation gap in Europe. Long-term follow-up is required to assess protocol adherence and clinical impact.

## 1 Introduction

Enhanced Recovery After Surgery (ERAS) is a modern approach to perioperative care patients that aims to reduce surgical stress, thus improving patient recovery. Enhanced recovery protocols are designed to ensure that patients are in the best possible condition before surgery, receive optimal surgical and anesthetic management, as well as appropriate postoperative care. Core principals of enhanced recovery protocols include prehabilitation, minimally invasive techniques and early mobilization and feeding (1). Implementation of enhanced recovery relies heavily on close collaboration of all specialists participating in the perioperative process, as well as active engagement of patients and their relatives.

The concept of enhanced recovery was first introduced in the 1990s by Professor Henrik Kehlet and the original protocols were developed for colorectal surgery (2). The protocols have since been shown in several randomized clinical trials and metanalyses to reduce postoperative complications, shorten length of hospitalization, improve clinical recovery parameters and to decrease hospital costs (3). Enhanced recovery protocols have now been developed for a wide range of surgical fields, including foregut surgery, pancreatobiliary surgery, gynecological surgery and urology (4–7).

Despite robust evidence supporting the benefits of the Enhanced Recovery protocols in clinical practice, their systematic implementation remains inconsistent across European hospitals (8). The causes of this variability are multifactorial and differ between healthcare systems. Contributing factors include limited resources (such as time constraints, staff shortages, and absence of a dedicated Enhanced Recovery coordinator), resistance to changing pre-existing patterns and behaviors, logistical incompatibilities, inconsistent training and education, and the absence of systematic tracking of the implementation and lack of feedback (9, 10). These barriers amplify the difficulty of adhering to and even further advancing the practice of Enhanced Recovery protocols for the benefit of patients.

In an effort to address this implementation gap, we present the following collaborative network. EUPEMEN (EUropean PErioperative MEdical Networking) is a European educational initiative project that aims at promoting the dissemination of Enhanced Recovery Protocols across various surgical fields. In a unified region like the European Union, bridging disparities in surgical knowledge and perioperative pathways is a multidimensional challenge that should be addressed by coordinated multinational collaborations. To ensure alignment with European guidelines in the setting of diverse hospital and healthcare settings, different socioeconomic contexts and a multitude of training methodologies, the EUPEMEN project has brought together national clinical leaders who have previously led major change initiatives in their countries and leveraged their expertise to expand the implementation and structured expansion of enhanced recovery protocols throughout Europe.

This overarching main goal will be pursued through the development of a structured educational program, the implementation of enhanced recovery protocols in a significant number of European hospitals, and the collection of medical data that can be used to inform and improve perioperative outcomes. The primary objective of this article is to present in detail the initiatives undertaken by the EUPEMEN network and to report the outcomes achieved through their implementation. By presenting our multicenter experience we aim to provide a reproducible framework for the dissemination and sustainable adoption of Enhanced Recovery protocols across Europe.

## 2 Materials and methods

### 2.1 Study design and reporting framework

This was a multinational, prospective, mixed-methods implementation study designed to evaluate the feasibility, dissemination, and early clinical impact of a standardized Enhanced Recovery educational framework across five European centers. The initiative facilitated multinational collaboration, coordination among participating institutions and the development of shared educational activities across countries. The description of the intervention components was guided by the Standards for Quality Improvement Reporting Excellence (SQUIRE 2.0) guidelines for reporting healthcare improvement initiatives (11).

### 2.2 Consortium design

This initiative was conducted over a 26-month period, spanning from September 2020 to October 2022, with support and funding from Erasmus + Programme of the European Union. *Τ*he consortium consisted of five core collaborative institutions across four European countries (Greece, Czech Republic, Spain, Italy), strategically selected to combine academic expertise with practical clinical implementation capacity (Table 1). In addition, several associated partners and organizations contributed to achieving the project objectives (Table 2).

**Table 1:** List of collaborating institutions and their specific roles. .

Role	Acron	Partner's name	Country	Principal investigator/representative
Coordinator	IISA	The Institute for Health Research Aragon	Spain	Jose M Ramirez
Partner 1 (P1)	AUSLFE	Hospital Presidium of the Azienda Unita Santiaria Locale Hospital Presidium of the Azienda	Italy	Carlo Feo
Partner 2 (P2)	CUNI	The Department of Surgery at the Second Faculty of Medicine of Charles University, Prague	Czech Republic	Petr Kocian
Partner 3 (P3)	UMH	The Miguel Hernandez University of Elche	Spain	Antonio Arroyo
Partner 4 (P4)	GPAP	General Hospital of Thessaloniki “George Papanikolaou”	Greece	Orestis Ioannidis

**Table 2:** List of associated partners and organisations and their contributions. .

Name of the organisation	Country of the organisation	City	Contribution
HCULB: Hospital Clínico Universitario Lozano Blesa	Spain	Zaragoza	Implementation of protocols and contribution in the dissemination of the results.
HUMS: Hospital Universitario Miguel Servet	Spain	Zaragoza	Implementation of protocols and contribution in the dissemination of the results.
Aristotle University of Thessaloniki	Greece	Thessaloniki	Assistance with promoting and disseminating the project and the multiplier event. Placement of the project and the VIA RICA Greek translation under its auspices.
Greek Surgical Society	Greece	Athens	Placement of the project and the VIA RICA Greek translation under its auspices.
Greek Society of Surgical Infections	Greece	Athens	Placement of the project and the VIA RICA Greek translation under its auspices.
Unizar: Universidad de Zaragoza	Spain	Zaragoza	Participation through the Cátedra Médica Perioperatoria (where the IP belongs to).
GERM	Spain	Zaragoza	Support of the project with the VIA RICA.
Hospital Universitario de Elche	Spain	Elche	Involvement in the project with the implementation of the protocols and assistance in the dissemination of the results.
University of Ferrara	Italy	Ferrara	Assistance in the development of protocols and dissemination of the project.
Motol University Hospital	Czech Republic	Prague	Assistance in the implementation of protocols and dissemination of the project.

The five participating institutions were selected based on their expertise and clinical implementation capacity. The Institute for Health Research Aragón (Spain) served as the coordinating partner. The Azienda Unità Sanitaria Locale of Ferrara (Italy) contributed clinical implementation experience. Miguel Hernández University of Elche (Spain) provided academic leadership in dissemination activities. Charles University (Czech Republic) contributed expertise in colorectal surgery and Enhanced Recovery implementation. The General Hospital of Thessaloniki “G. Papanikolaou” (Greece) contributed clinical expertise in perioperative care. Additional details are provided in Table 1 and Supplementary Material 1.

**Figure 1:**
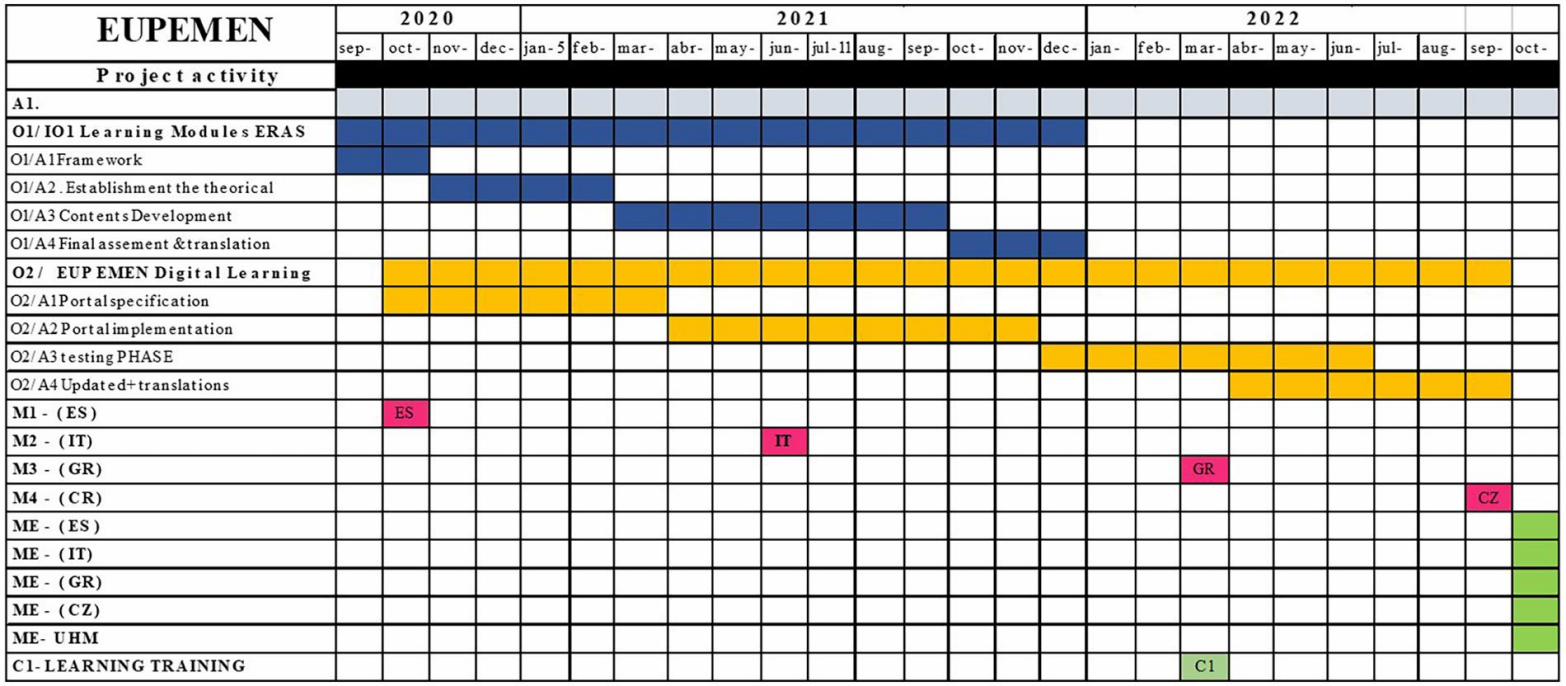
Timetable of the EUPEMEN project.

Tasks and responsibilities among the partners of the EUPEMEN project were distributed as summarized in Table 3. Overall coordination and project governance were undertaken by IISA, including administrative reporting, financial management, and overarching quality control. Dissemination activities were coordinated by CUNI, including the organization of the Dissemination Plan, the creation and distribution of communication materials (leaflet, logo and website, social media and newsletters), as well as the Press Release conducted by all partners. Quality assurance was overseen by UMH, which was responsible for monitoring intellectual output quality reports and evaluation of project processes and products. Development of the EUPEMEN online platform was undertaken by AUSLE, while GPAP organized and hosted the Learning Teaching Activity (LTA) event in Greece. The planned timeline of the project and the corresponding implementation phases are presented in Figure 1.

**Table 3:** Tasks and responsibilities among the partners of the EUPEmen project.

Project management and governance	Category	Description
	Project leadership	IISA led and coordinated the project, ensuring adherence to timelines, budget, objectives, and impact evaluation.
	Partner responsibility	Each partner was responsible for project management and implementation within their own organisation and country.
	Project Management Group:	Established with one representative per partner to oversee coordination and ensure proper project development.
Quality assurance		
	Quality lead	UMH was responsible for quality assurance.
	Key activities	Definition of quality criteria, continuous monitoring of activities, early detection of deviations, and implementation of corrective actions.
Dissemination and exploitation		
	Dissemination lead	CUNI coordinated dissemination and exploitation activities.
	Dissemination plan	A unified dissemination plan was developed and implemented by all partners across participating countries (Supplementary Material 2)
Intellectual Outputs (IOs)		
	IO1 – Protocol Training Manual (lead: IISA):	• Development of modular training manuals covering multiple surgical domains. • Modules and leaders were defined by the Project Management Group: • Bariatric surgery – AUSLFE (Italy) • Oesophageal surgery – CUNI (Czech Republic) • Gastric cancer surgery – GPAP (Greece) • Colon surgery – IISA (Spain) • Urgent abdominal surgery – UMH (Spain) • Hepatobiliary surgery – IISA (Spain) • Preparation of guides and e-learning materials – IISA (Spain)
	IO2 – Digital Learning Platform (lead: AUSLFE):	• Development of an online e-learning training course accessible to target groups. • Creation of a collaborative platform for enhanced recovery protocols. • Translation of the Via RICA protocol from Spanish to English. • Production of educational videos and multiple-choice assessment questions.
Multiplier events		
	Objective	Presentation and dissemination of project results.
	Activities	Five EUPEMEN Local Forums conducted across four countries: • Spain – IISA and UMH • Italy – AUSLFE • Czech Republic – CUNI • Greece – GPAP
Learning, teaching, and training activities	Lead partner: GPAP.	Activity: “Train EUPEMEN Trainers” workshop held in Greece to support implementation, management, and future training of the developed protocols.

Nonetheless all partners were responsible for the following:

- Implementation of project activities at national and institutional level.
- Participation in transnational meetings and joint events.
- Expansion of professional networks and establishment of local stakeholder forums.
- Execution of dissemination activities.
- Financial reporting in accordance with Erasmus + regulations
- Engagement of target groups.
- Coordination of contributions to Intellectual Outputs (IOs).
- Participation in evaluation and other cross-cutting activities.
- Organization of a partner meeting and a multiplier event within their respective countries.

**Figure 2:**
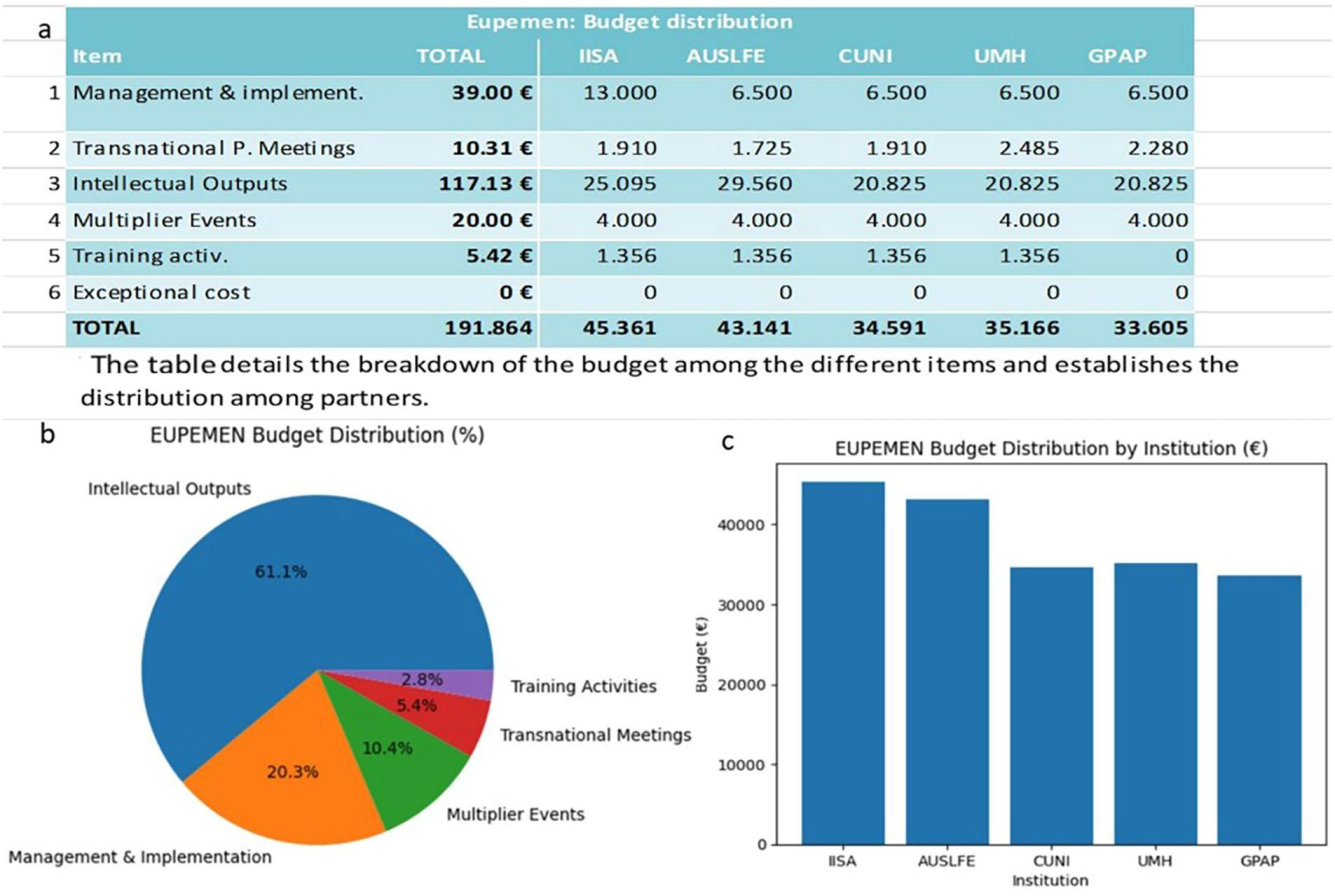
EUPEMEN project budget distribution across activity categories and participating institutions. **(a)** Detailed allocation of the total budget by activity category and institution (EUR). **(b)** Proportional distribution of the budget across activity categories (%). **(c)** Institutional distribution of the total project budget (EUR).

The Erasmus + financial framework was structured around predefined activity categories, including project management and implementation, transnational project meetings, development of intellectual outputs, multiplier dissemination events and training activities. Understandably, the largest portion of the budget was allocated to the development of intellectual outputs, reflecting the central role of protocol standardization and educational material production within the implementation strategy. This activity-based allocation ensured the division of resources based on strategic priorities, as illustrated in Figure 2.

### 2.3 Intervention design and implementation framework

The main objectives - intellectual outcomes (IOs) of the project were defined as:

1. Development of a structured educational programme.
2. Implementation of the Enhanced Recovery protocols across a significant number of European hospitals in a homogenous and standardized manner.
3. Collection of clinical data, including length of hospital stay, morbidity, and mortality of European surgical patients, to facilitate perioperative risk assessment and support complication prevention strategies.

To achieve the aforementioned objectives, all EUPEMEN partners, under the coordination of IISA and led by the correspondent partner, have concluded in the following implementation actions:

1. Development of formal agreements and operational manuals for the standardized implementation of the project.
2. Preparation of a comprehensive manual comprising seven procedure-specific modules (Multimodal Rehabilitation, Bariatric Surgery, Oesophageal Surgery, Gastric Surgery, Colon Surgery, Urgent abdominal surgery, Hepatobiliary Surgery) to be followed by all the target groups.
3. Organization of Learning Training Activities (LTA), based on a train-the-trainer model, enabling participants to independently deliver protocol training within their respective hospitals.
4. Development of the EUPEMEN online platform, hosting the e-learning training course and a collaborative interface to facilitate interaction, discussion, and protocol refinement.
5. Dissemination of the project results through five multiplier events, supported by digital communication strategies and integration within the official project website, accompanied by a structured dissemination plan aimed at promoting the initiative through coordinated press releases and strategic media engagement (Supplementary Material 2).
6. Organization of three face-to-face transnational meetings and one online meeting to ensure protocol harmonization and strategic alignment.

To further ensure the proper development, methodological rigor and structured oversight of the project, a formal Quality Plan was established, defining operational procedures, evaluation criteria and corrective mechanisms. In addition, two governance documents were developed by IISA: the Consortium Agreement and the EUPEMEN Management Guide. The Project Management Group (PMG) was established during the initial meeting on 08 October 2020 to oversee implementation of the work plan and evaluate project outputs (Table 4). Continuous communication and document sharing were facilitated through a cloud-based collaboration platform. In total, fourteen structured meetings, both online in person, were conducted during the project timeline, supporting ongoing monitoring, strategic alignment, and timely corrective action when necessary.

**Table 4:** EUPEmen project management group.

EUPEMEN: Project Management Group		
Partner	Designated representative	Email
IIS Aragón	José Manuel Ramírez	jmirarez@unizar.es
AUSLFE	Carlo Feo	cfo@unife.it, carlo.feo@unife.it
CUNI	Petr Kocian	kocian.cz@gmail.com
UMH	Antonio Arroyo	arroyoic@hotmail.com
GPAP	Orestis Ioannidis	telonakos@hotmail.com

### 2.4 Participant selection for learning teaching activity (LTA) and multiplier events (ME)

Participant selection for the two main activities of the project followed distinct approaches aligned with their respective objectives. The project focused on a multidisciplinary group of professionals essential for effective perioperative surgical care. The direct target group comprised frontline healthcare staff (surgeons, anaesthetists, and nurses) alongside key specialists (such as nutritionists, physiotherapists, and oncologists). To promote continuity and institutional sustainability of Enhanced Recovery implementation, the scope was also expanded to include hospital managers, quality coordinators, and primary care physicians. Beyond the immediate clinical environment, the initiative also engaged indirect target groups, including patients and their families, while involving stakeholders from local and national health authorities to ensure broader adoption and policy-level awareness.

Regarding the Learning Teaching Activity (LTA), which was held in Thessaloniki, Greece and was hosted by GPAP, eligible participants were identified and recruited by each partner institution, according to predefined selection criteria aligned with the project objectives. These criteria included: 1) possession of a university degree relevant to perioperative care (medicine, surgery, anesthesiology, nursing, pharmacy or related disciplines); 2) demonstrated expertise in the field; 3) an established affiliation with the participating institution or project partners; 4) the ability to teach and transfer the knowledge acquired through the activity within their local clinical environment. Each partner nominated two trainers, and the final approval was granted by the institution's principal investigator. A total of 10 trainers attended the LTA. The activity was structured as a multidisciplinary, interactive training workshop, enabling participants not only to receive structured instruction but also to contribute experiential insights, which informed refinements of both the clinical protocols and the e-learning platform.

With respect to the Multiplier Events (MEs), each hosting partner was responsible for developing promotional materials, including announcement posters, and for disseminating invitations through institutional mailing lists, professional networks, social media platforms, and newsletters. These dissemination efforts resulted in a total number of 366 attendees, substantially exceeding the initial target of 200 people. Press releases issued by each partner generated additional media visibility at local and regional levels, extending outreach beyond the professional community and enhancing public awareness of the Erasmus + funded initiative and its broader objectives.

### 2.5 Project evaluation

To objectively evaluate the scope and impact of the initiative, both qualitative and quantitative indicators were employed as measures to assess process performance and overall reach. The evaluation process commenced from the outset of the project, with an initial assessment of the proposal during the first meeting, where specific strengths, weaknesses and recommendations for improvement were identified. The project’s focus of transforming clinical practice through common interdisciplinary guidelines and educational protocols, emphasized the necessity of a robust Quality Plan that could provide methodological rigor and objective evidence regarding the effectiveness of the initiative.

Transnational Project Meetings (TPMs) played a central role in monitoring implementation, coordinating partner activities, and overseeing adherence to the predefined work plan. These meetings functioned both as governance mechanisms and as platforms for promoting collaboration among the participating countries and to foster a cohesive team environment. For this purpose, four meetings were initially planned, one in each participating country. However, due to the numerous restrictions imposed during the COVID-19 pandemic, the first meeting, originally scheduled to take place in Spain, was conducted online in October 2020. The subsequent meetings were successfully held in Italy, Greece and Czech Republic, ensuring continuity of coordination despite the challenging circumstances.

Interim Reports were progress reports constituting the formal project documentation, developed to systematically record the different phases of the initiative. These reports detailed the achievements of the consortium and included financial statements outlining expenditures incurred within defined reporting periods. Overall, two interim reports were prepared during the implementation phase, followed by a final comprehensive report summarizing cumulative outcomes and providing a structured assessment of the overall impact and performance of the initiative.

## 3 Results

### 3.1 Intellectual output 1 - EUPEMEN protocols

The primary aim of the initiative was to develop six standardized general surgery protocols covering a wide range of procedures, spanning the preoperative, intraoperative and postoperative phases. The protocols were designed in a structured, stepwise format to facilitate practical implementation across heterogeneous hospital settings. Seven protocols were ultimately developed. All protocols were translated into English, Spanish, Italian, Czech and Greek and were made accessible through the EUPEMEN website.

All developed protocols within the EUPEMEN framework have either already been published in peer reviewed medical journalsand can be found in Supplementary Material 3 (12–18).

### 3.2 Intellectual output 2 - EUPEMEN online platform

The second Intellectual Output (IO2) comprised the development of an online educational platform with two core pillars: an interactive learning environment and a discussion forum designed to facilitate peer-to-peer exchange within an asynchronous setting. Access to the platform, which was linked through the official EUPEMEN website, required free email registration to create an individual user account.

The educational content provided on the platform focused on providing the knowledge required for the institutional implementation of the developed protocols. The curriculum was structured in ten modules. The first two modules provided foundational theoretical knowledge, while the remaining modules provided a multimedia approach, including video lectures, presentations, and structured instructional materials. The topics addressed were as follows:

1. The Via RICA Pathway (Supplementary Material 4)
2. EUPEMEN Protocols
3. Enhanced Recovery in Adult's Surgery Generalities (https://vimeo.com/791077778) (Supplementary Material 5a)
4. Preoperatory Preparation (https://vimeo.com/791077918) (Supplementary Material 5b)
5. Surgery: Key points. Drains and nasogastric tube (https://vimeo.com/1163550097) (Supplementary Material 5c)
6. Anaesthesia: Key points (https://vimeo.com/791828527) (Supplementary Material 5d)
7. Nursing: Key points (https://vimeo.com/791857669) (Supplementary Material 5e)
8. Nutrition: Key points (https://vimeo.com/791831610) (Supplementary Material 5f)
9. Scientific evidence and recommendation guidelines. Implementations problems, difficulty of adherence and sustainability (https://vimeo.com/784951361) (Supplementary Material 5g)
10. Challenges and future plans for Enhanced Recovery (https://vimeo.com/791077860) (Supplementary Material 5h)

All educational videos corresponding to these modules are provided together as Supplementary Materials 5a–h).

**Figure 3:**
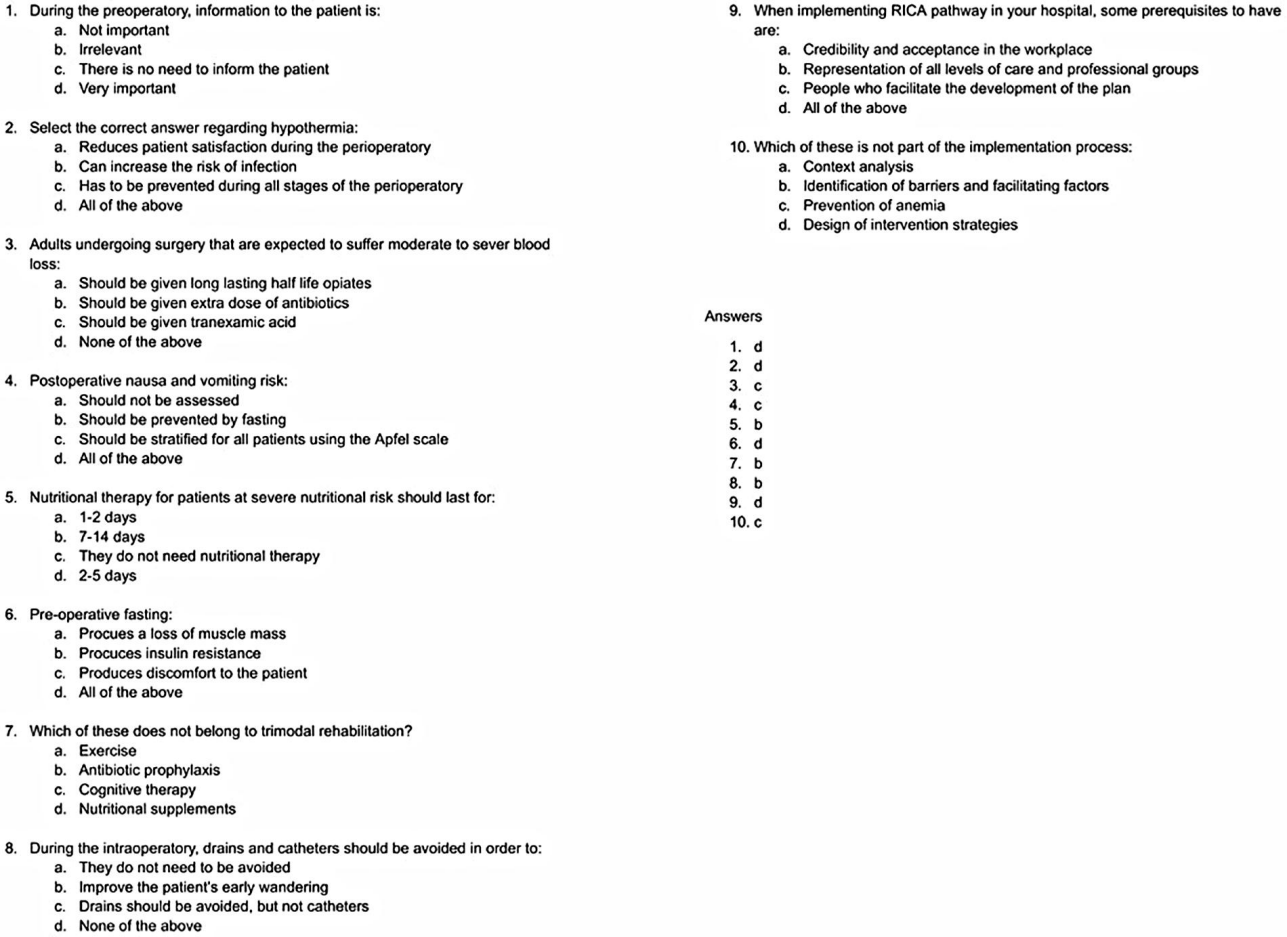
Multiple choice questionnaire EUPEMEN online platform.

Several design elements were incorporated to enhance usability and learner engagement and include: a completion bar that tracked the percentage of covered material for each user. Active recall or retrieval-based learning was also implemented on the platform (19, 20). A short 10-question test was provided after each module (Figure 3). Performance in the multiple-choice assessments indicated adequate knowledge acquisition among participants, although detailed quantitative analysis was not within the scope of the present study.

Concurrently the Protocol Forum, subdivided into categories in accordance with the produced protocols supported the scholarly dialog during the course of this project.

### 3.3 Learning teaching activity (LTA)

This Learning Teaching Activity (LTA) was hosted in Thessaloniki, Greece by GPAP from 5 to 8 May 2022 and was structured as a train-the-trainer workshop. A total of ten participants attended, with each partner institution nominating two representatives. During the three-day training course, each institution's principal investigator, or an appointed representative, explained in great detail the protocols developed within the project, as well as the RICA pathway.

**Figure 4:**
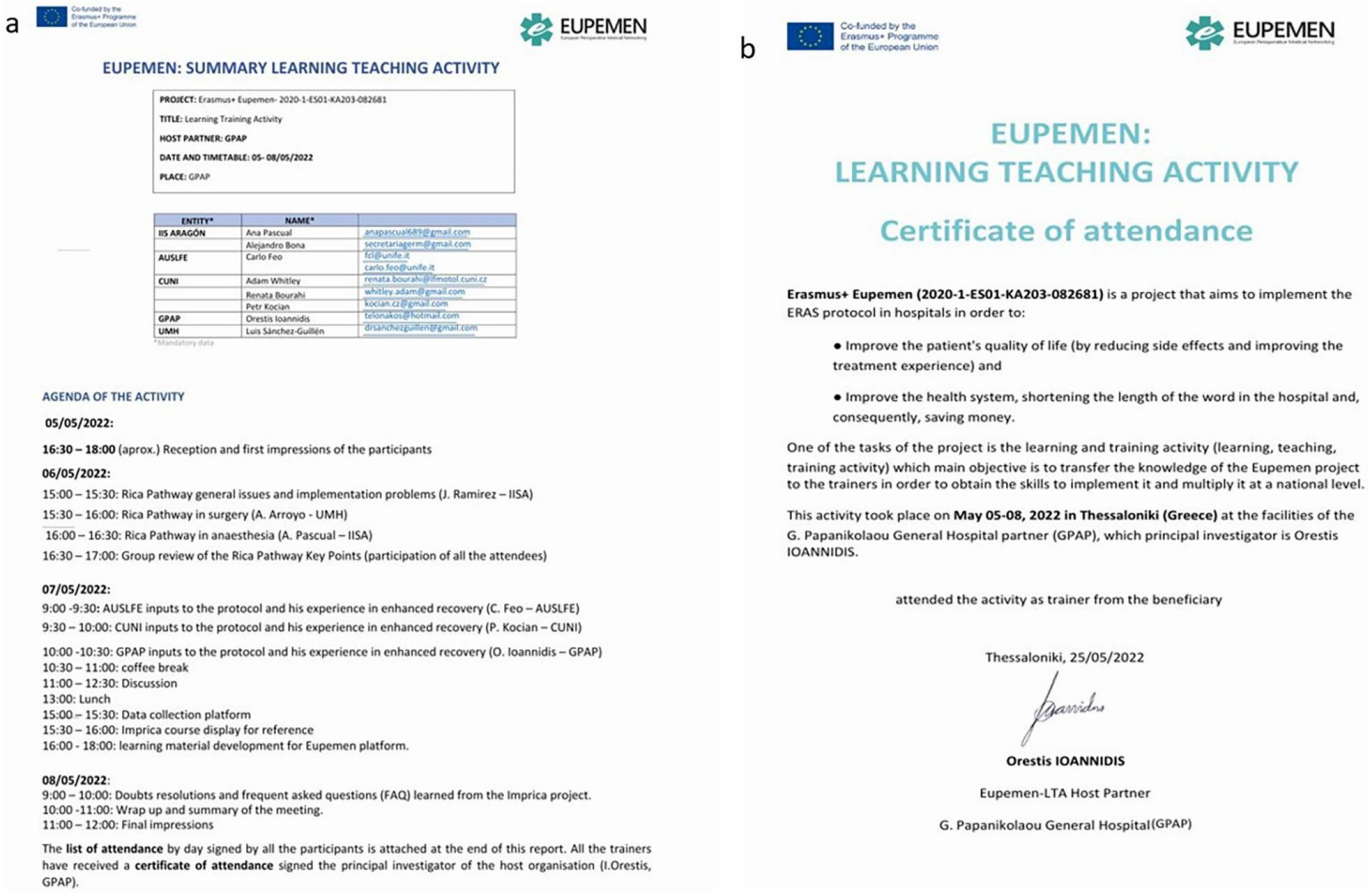
Learning teaching activity. **(a)** Learning Teaching Activity Agenda, **(b)** Certificate of Attendance.

The sessions outlined perioperative management strategies for the respective surgical procedures, addressed procedure-specific considerations and complications, and introduced the database framework designed for standardized data collection across participating hospitals. Upon completing the programme, participants received a certificate of attendance (Figure 4).

### 3.4 Multiplier events

All EUPEMEN partners organized and conducted Multiplier Events (MEs) in their respective countries (Table 5). In total, five distinct events were held across the four participating countries. These dissemination events focused mainly on the presentation of the Enhanced Recovery Protocols Training Programme (IO1) and the EUPEMEN Online Platform (IO2) to third-party institutions and healthcare professionals.

**Table 5:** Multiplier events data.

Entity	Country	Event title	Date	Number of participants
IISA	Spain	Jornada Imprica – Eupemen	28/10/2022	32
AUSLFE	Italy	Il Programma Enhanced Recovery After Surgery (ERAS) in Chirurgia Colorettale. Un approccio multidisciplinare e interprofessionale.	28/10/2022	64
CUNI	Czech Republic	Enhanced Perioperative Care in Colorectal Surgery - EUPEMEN LOCAL FORUM	27/10/2022	75
GPAP	Greece	1*ο Σεμιν*ά*ρ*ιο *Π*ροε*γχ*ειρ*ητ*ι*κ*ής *Φ*ροντί*δα*ς - *Β*ε*λ*τι*σ*τοποίησης της μετεγχειρητικής *Α*νάρρ*ω*σης	25/09/2022	82
UMH	Spain	EL VALOR DE LA ADAPTACIÓN PERIOPERATORIA DEL PACIENTE QUIRÚRGICO. PREHABILITACIÓN Y REHABILITACIÓN MULTIMODAL (ERAS Y EUPEMEN) EN CÁNCER COLORRECTAL	27/10/2022	113
				**Total** 366

**Figure 5:**
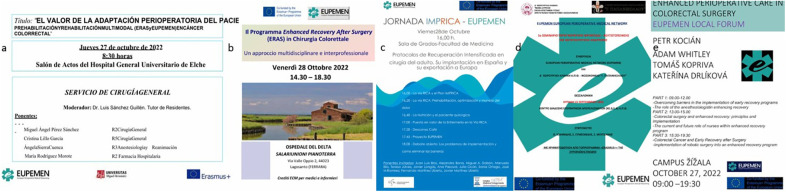
**(a–e).** Posters from the five Multiplier Events.

The MEs consisted structured dissemination activities, including presentations of the developed Enhanced Recovery protocols, their core components, and implementation strategies (Figure 5).

Overall, a total of 366 participants attended the MEs, exceeding the initial target of 200 participants. Participants represented a broad multidisciplinary audience—including surgeons, anesthesiologists, nurses, and allied healthcare professionals (Supplementary Material 6).

### 3.5 EUPEMEN website

**Figure 6:**
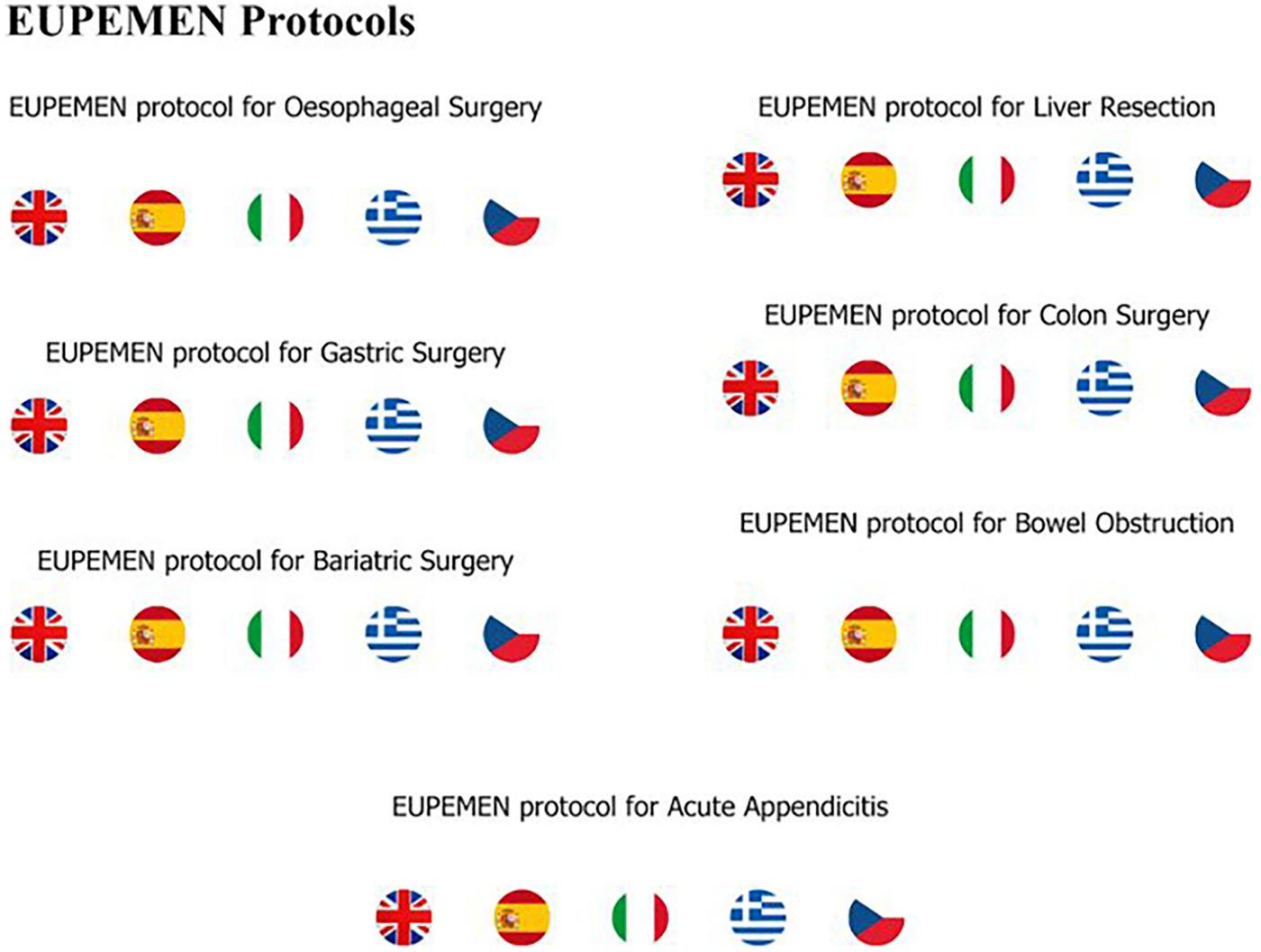
All EUPEMEN protocols available in 5 languages in the EUPEMEN website.

A dedicated EUPEMEN website was developed to function as the project’s central communication and knowledge sharing platform (http://www.eupemen.eu). The platform contained information on the project’s aims and descriptions of the intellectual outputs, with the developed protocols displayed in five different languages (Figure 6).

A direct link to the EUPEMEN Online Platform (http://www.eupemen-learning), featuring the educational content and the discussion forum, was also provided on the official website. Outreach activities were supported through the publication of two newsletters and multiple press releases during the project period.

Following the conclusion of the Erasmus + funding period, the official project website became inactive due to maintenance and financial constraints. However, sustainability is supported through the peer-reviewed publication of the developed protocols, continued academic dissemination, and presentations at international scientific congresses, including the American College of Surgeons (ACS) Clinical Congress 2022, San Diego, CA, USA.

### 3.6 Via RICA - translated versions

An additional outcome of the initiative was the translation of the Clinical Pathway for Intensified Recovery in Abdominal Surgery - Via RICA from Spanish to English and Greek. Via RICA is an interdisciplinary, evidence-based clinical pathway originally developed through a multidisciplinary consensus led by the Spanish Multimodal Rehabilitation Group (GERM). Its translation into English and Greek was performed within the EUPEMEN project.

### 3.7 Implementation of protocols

Beyond protocol development and educational dissemination, the initiative included structured implementation strategies from its inception. As a result, all participating institutions implemented the Enhanced Recovery protocols within their respective hospital settings. Implementation was supported by a monitoring framework designed to track selected perioperative elements.

Due to the limited duration of the project, comprehensive clinical outcome data were not available for formal impact analysis at the time of reporting. However, educational outcomes related to the online platform—such as module completion and performance in multiple-choice assessments—were collected, although these were not the primary focus of the present analysis. The present study therefore focuses on process evaluation and implementation metrics. Future prospective analyses incorporating adherence indicators and patient-level outcomes will be required to assess the long-term clinical impact and sustainability of the EUPEMEN framework.

## 4 Discussion

### 4.1 The implementation gap in enhanced recovery

The implementation of Enhanced Recovery protocols has been consistently associated with significant improvements in perioperative care, including optimized preoperative preparation, reduced postoperative morbidity, shorter hospital stays, and accelerated functional recovery (21). Despite the strength and robustness of the available evidence, adoption remains heterogeneous across European centers (22). International literature attributes this implementation gap to multifactorial barriers, including institutional and organizational constraints, resource variability, resistance to change, and challenges in multidisciplinary coordination and sustained adherence (23).

Within this context, the EUPEMEN project was designed as a structured, multinational initiative, aimed at addressing disparities in Enhanced Recovery uptake across European healthcare systems by consolidating and standardizing Enhanced Recovery knowledge in a structured, accessible format translated in multiple languages. By presenting our multicenter experience, we aim to propose a reproducible European framework for scaling Enhanced Recovery implementation and dissemination through standardized protocols and multidisciplinary, peer-to-peer training.

A novel aspect of this initiative was the formal translation of Enhanced Recovery protocols into multiple languages. By providing translated, validated and context-adapted guidelines, the project facilitated deeper integration of Enhanced Recovery principles into the daily workflow of surgical departments where English is not the primary language.

### 4.2 EUPEMEN protocol design

The primary objective of this initiative was the development of procedure-specific protocols, grounded in evidence-based recommendations, enabling consistent knowledge transfer and practical application across the different hospital settings. Central to this effort was the direct, iterative collaboration among experienced clinicians from multiple specialties.

An important implication for protocol design is that several perioperative elements are broadly transferable across specialties, either because the supporting evidence is strong within individual fields or because the interventions are logical, safe, and not resource intensive. As a result, EUPEMEN adopted a modular structure that preserves a shared core of perioperative Enhanced Recovery elements while allowing procedure-specific adaptations where necessary (24).

Esophagectomy is an operation where procedure-specific protocols are deemed essential rather than optional (25).In this context, the main value of a structured protocol is defining realistic recovery targets -such as early mobilization- and aligning surgical, anaesthetic, and nursing priorities through shared training and consistent implementation across centers (14).

Bariatric surgery illustrates how Enhanced Recovery principles must be applied within a high-comorbidity population. Updated ERAS Society recommendations emphasize meticulous preoperative optimization, opioid-sparing anesthesia to reduce respiratory compromise, and early mobilization to mitigate thromboembolic risk (26).

The EUPEMEN gastrectomy module was developed in accordance with the ERAS Society recommendations for enhanced recovery after gastrectomy, which provide a structured, evidence-based framework across the full perioperative pathway (27). Gastric surgery requires clear operational guidance on high-impact, procedure-relevant decisions, including perioperative nutritional optimization and feeding progression, a clear strategy for gastric decompression/nasogastric tube use, selective use and early removal criteria for drains (28). The resulting protocol functions as a multidisciplinary care pathway that can be taught and reproduced consistently across sites (16).

Colon surgery, where Enhanced Recovery evidence is particularly mature, provides an ideal framework for demonstrating measurable adherence and outcome monitoring (27). The colon module emphasized standardized preoperative preparation, opioid-sparing anaesthesia, early mobilization, early oral intake, and avoidance of unnecessary tubes and drains (18).

Hepatobiliary surgery in its clinical complexity further demonstrates the need for multidisciplinary coordination and reliance on clearly defined criteria. The liver resection module was developed with the 2022 ERAS Society recommendations (29). The module addressed selective drain use and early removal decision rules, analgesia strategies that support mobilization without compromising hemodynamic stability, and nutrition planning tailored to metabolic demands and liver function considerations (15).

Acute appendicitis represents a distinct scenario within emergency surgery for Enhanced Recovery implementation, as it combines elements of emergency surgery with typically short procedural duration and rapid postoperative recovery potential (30). Emerging evidence suggests that structured enhanced recovery principles can and should be applied in uncomplicated cases. In this regard, appendectomy may serve as a pragmatic entry point for integrating enhanced recovery principles into emergency general surgery settings (12).

The application of Enhanced Recovery principles in bowel obstruction presents additional complexity due to physiological instability and variability in operative requirements. Nevertheless, selected elements may contribute to improved functional recovery in urgent surgical contexts (31), highlighting the need for adaptable, modular implementation strategies (13).

### 4.3 Dissemination strategy and hospital implementation

The need for structured educational resources is supported by implementation data showing that insufficient Enhanced Recovery knowledge remains a common barrier in routine practice. In a national survey conducted in France, Clet et al. (32) identified “lack of knowledge” as one of the most frequently reported obstacles to Enhanced Recovery adoption.

Within EUPEMEN, dissemination activities - including multiplier events, media outreach, and the project website - were designed as active implementation tools rather than simple communication outputs. In addition, multiplier events created opportunities for peer discussion, allowing teams to exchange practical adaptations to common constraints.

The three face-to-face transnational meetings and multiple online meetings were central to achieving protocol harmonization across different healthcare systems and professional cultures. Importantly, the meetings also functioned as a mechanism for continuous improvement, as feedback from training delivery and early implementation challenges could be rapidly incorporated into updated manuals and platform content.

Unlike initiatives limited to educational dissemination, EUPEMEN incorporated direct protocol deployment within participating hospitals. Each protocol included a monitoring framework outlining perioperative elements intended for tracking. Although long-term outcome data were not available within the project timeframe, the inclusion of predefined monitoring components represents a structural step toward measurable adherence assessment.

### 4.4 Strengths of the EUPEMEN project

A key strength of the EUPEMEN initiative is its structured multilevel implementation strategy, operating simultaneously at institutional, national, and European levels. At the institutional level, Enhanced Recovery protocols were implemented in five hospitals, supported by a structured train-the-trainer model, which prepared ten trainers and reached nearly 400 healthcare professionals through multiplier events. At the national level, collaboration with established Multimodal Rehabilitation Groups reinforced dissemination and supported sustainability through professional network alignment. At the European level, EUPEMEN established an international collaborative group dedicated to continued harmonization and refinement of perioperative pathways.

### 4.5 Limitations

Although enhanced recovery recommendations are increasingly supported by high-quality evidence, real-world adherence remains the critical determinant of impact. Within the EUPEMEN initiative, the primary challenge was not defining best practices, but ensuring consistent multidisciplinary delivery—particularly in complex elective procedures and urgent surgical settings. However, these measures do not fully mitigate constraints, such as staffing variation, competing priorities, and local resource limits. These contextual factors should be acknowledged when interpreting outcomes and underscore the need for longer follow-up and objective compliance/outcome metrics.

The project unfolded during the COVID-19 pandemic, a period characterized by global disruption of healthcare systems and international mobility. Travel restrictions and institutional constraints necessitated a rapid shift toward fully virtual collaboration, which, while enabling continuity, can introduce well-recognized limitations inherent to remote teamwork (33). Nevertheless, these adaptations were necessary to ensure project continuity during the pandemic period.

Limitations inherent to the funding framework provided by Erasmus + should also be acknowledged. Specifically, the total combined number across all five dissemination events was 366 participants, only 240 of which were ultimately considered eligible for budgetary inclusion. This highlights a broader financial limitation regarding the maintenance and continuation of the results following the conclusion of external funding for the project.

## 5 Conclusion

The EUPEMEN project highlights that a structured, multilingual, and collaborative framework can effectively support harmonized Enhanced Recovery implementation and adoption across diverse European healthcare systems. By integrating standardized protocols, scalable education, and cross-border cooperation, the initiative provides a practical science model for bridging the perioperative Enhanced Recovery implementation gap. Long-term evaluation of adherence and clinical outputs will determine its sustained impact.

## Data Availability

The original contributions presented in the study are included in the article/Supplementary Material, further inquiries can be directed to the corresponding author.

## Supplementary material

Supplementary Material 1 Information Regarding Participating Institutions and Principal Investigators (Information drawn from the original EUPEMEN website).

Supplementary Material 2 EUPEMEN Dissemination Plan and Press Release Strategy (All press releases published from participants throughout the project).

Supplementary Material 3 Detailed EUPEMEN Project Protocols in Italian, Spanish, Czech, and Greek.

Supplementary Material 4 The Via RICA Pathway (in English and Greek).

Supplementary Material 5 EUPEMEN Educational Video Modules (Multimedia Training Content Supporting the Online Learning Platform).

Supplementary Material 6 Agendas of the EUPEMEN Multiplier Events (Agendas in original language and their English translation).

## References

[1] Abeles A Kwasnicki RM Darzi A. Enhanced recovery after surgery: current research insights and future direction. World J Gastrointest Surg. (2017) 9:37–45. 10.4240/wjgs.v9.i2.37 28289508 PMC5329702

[2] Kehlet H. Multimodal approach to control postoperative pathophysiology and rehabilitation. Br J Anaesth. (1997) 78:606–17. 10.1093/bja/78.5.606 9175983

[3] Sauro KM Smith C Ibadin S Thomas A Ganshorn H Bakunda L Enhanced recovery after surgery guidelines and hospital length of stay, readmission, complications, and mortality: a meta-analysis of randomized clinical trials. JAMA Netw Open. (2024) 7:e2417310. 10.1001/jamanetworkopen.2024.17310 38888922 PMC11195621

[4] Low DE Allum W De Manzoni G Ferri L Immanuel A Kuppusamy M Guidelines for perioperative care in esophagectomy: enhanced recovery after surgery (ERAS(®)) society recommendations. World J Surg. (2019) 43:299–330. 10.1007/s00268-018-4786-4 30276441

[5] Melloul E Lassen K Roulin D Grass F Perinel J Adham M Guidelines for perioperative care for pancreatoduodenectomy: enhanced recovery after surgery (ERAS) recommendations 2019. World J Surg. (2020) 44:2056–84. 10.1007/s00268-020-05462-w 32161987

[6] Nelson G Fotopoulou C Taylor J Glaser G Bakkum-Gamez J Meyer LA Enhanced recovery after surgery (ERAS®) society guidelines for gynecologic oncology: addressing implementation challenges - 2023 update. Gynecol Oncol. (2023) 173:58–67. 10.1016/j.ygyno.2023.04.009 37086524

[7] Cerantola Y Valerio M Persson B Jichlinski P Ljungqvist O Hubner M Guidelines for perioperative care after radical cystectomy for bladder cancer: enhanced recovery after surgery (ERAS(®)) society recommendations. Clin Nutr. (2013) 32:879–87. 10.1016/j.clnu.2013.09.014 24189391

[8] Yoon S-H Lee H-J. Challenging issues of implementing enhanced recovery after surgery programs in South Korea. Anesth Pain Med. (2024) 19:24–34. 10.17085/apm.23096 PMC10847003 38311352

[9] Wang D Liu Z Zhou J Yang J Chen X Chang C. Barriers to implementation of enhanced recovery after surgery (ERAS) by a multidisciplinary team in China: a multicentre qualitative study. BMJ Open. (2022) 12:e053687. 10.1136/bmjopen-2021-053687 35288383 PMC8921855

[10] Stone AB Yuan CT Rosen MA Grant MC Benishek LE Hanahan E Barriers to and facilitators of implementing enhanced recovery pathways using an implementation framework: a systematic review. JAMA Surg. (2018) 153(3):270–9. 10.1001/jamasurg.2017.5565. PMID: 29344622 29344622

[11] Ogrinc G Davies L Goodman D Batalden P Davidoff F Stevens D. SQUIRE 2.0 (Standards for QUality Improvement Reporting Excellence): revised publication guidelines from a detailed consensus process. BMJ Qual Saf. (2016) 25:986–92. 10.1136/bmjqs-2015-004411 26369893 PMC5256233

[12] Ioannidis O Anestiadou E Ramirez JM Fabbri N Ubieto JM Feo CV The EUPEMEN (EUropean PErioperative MEdical Networking) protocol for acute appendicitis: recommendations for perioperative care. J Clin Med. (2024) 13:6943. 10.3390/jcm13226943 39598087 PMC11594694

[13] Ioannidis O Ramirez JM Ubieto JM Feo CV Arroyo A Kocián P The EUPEMEN (EUropean PErioperative MEdical Networking) protocol for bowel obstruction: recommendations for perioperative care. J Clin Med. (2023) 12:4185. 10.3390/jcm12134185 37445224 PMC10342611

[14] Ioannidis O Anestiadou E Koltsida A Ramirez JM Fabbri N Ubieto JM Optimizing perioperative care in esophageal surgery: the EUropean PErioperative MEdical Networking (EUPEMEN) collaborative for esophagectomy. Diseases. (2025) 13:231. 10.3390/diseases13080231 40863205 PMC12385772

[15] Ioannidis O Koltsida A Anestiadou E Ramirez JM Fabbri N Ubieto JM Recommendations for perioperative care in liver resection: the EUPEMEN (EUropean PErioperative MEdical Networking) protocol. Medicina (Kaunas. (2025) 61:978. 10.3390/medicina61060978 40572666 PMC12195212

[16] Ioannidis O Anestiadou E Ramirez JM Fabbri N Ubieto JM Feo CV Improving perioperative care in gastric surgery: insights from the EUropean PErioperative MEdical Networking (EUPEMEN) project. J Clin Med. (2025) 14:2108. 10.3390/jcm14062108 40142917 PMC11942800

[17] Ioannidis O Anestiadou E Ramirez JM Fabbri N Ubieto JM Feo CV A phase-based, multidisciplinary enhanced recovery pathway for bariatric procedures: the EUropean PErioperative MEdical Networking (EUPEMEN) collaborative for obesity surgery. J Clin Med. (2026) 15:1706. 10.3390/jcm15051706 41827126 PMC12985827

[18] Pesce A Ramírez JM Fabbri N Ubieto JM Vittorio FC. The EUropean PErioperative MEdical r (EUPEMEN) project and recommendations for perioperative care in colorectal surgery: a quality improvement study. Int J Surg. (2024) 110:4796–803. 10.1097/JS9.0000000000001601 38742840 PMC11325912

[19] Xu J Wu A Filip C Patel Z Bernstein S Tanveer R Active recall strategies associated with academic achievement in young adults: a systematic review. J Affect Disord. (2024) 354:191–8. 10.1016/j.jad.2024.03.010 38461899

[20] Serra MJ Kaminske AN Nebel C Coppola KM. The use of retrieval practice in the health professions: a state-of-the-art review. Behav Sci (Basel). (2025) 15:974. 10.3390/bs15070974 40723757 PMC12292765

[21] Greco M Capretti G Beretta L Gemma M Pecorelli N Braga M. Enhanced recovery program in colorectal surgery: a meta-analysis of randomized controlled trials. World J Surg. (2014) 38:1531–41. 10.1007/s00268-013-2416-8 24368573

[22] ERAS Compliance Group. The impact of enhanced recovery protocol compliance on elective colorectal cancer resection: results from an international registry. Ann Surg. (2015) 261:1153–9. 10.1097/SLA.0000000000001029 25671587

[23] Gramlich LM Sheppard CE Wasylak T Gilmour LE Ljungqvist O Basualdo-Hammond C Implementation of enhanced recovery after surgery: a strategy to transform surgical care across a health system. Implement Sci. (2017) 12:67. 10.1186/s13012-017-0597-5 28526041 PMC5438526

[24] Grant MC Engelman DT. Enhanced recovery after surgery: overarching themes of the ERAS® society guidelines & consensus statements for adult specialty surgery. Perioper Med (Lond). (2025) 14:120. 10.1186/s13741-025-00590-0 41168801 PMC12577217

[25] Perroni G Johnson C Khandhar S Veronesi G Ambrogi V Fernando HC. Implementation of eras for patients undergoing esophagectomy: a narrative review of the current literature and latest evidence. Curr Chall Thorac Surg. (2021) 3:37. 10.21037/ccts-20-105

[26] Stenberg E Dos Reis Falcão LF O'Kane M Liem R Pournaras DJ Salminen P Guidelines for perioperative care in bariatric surgery: enhanced recovery after surgery (ERAS) society recommendations: a 2021 update. World J Surg. (2022) 46:729–51. 10.1007/s00268-021-06394-9 34984504 PMC8885505

[27] Mortensen K Nilsson M Slim K Schäfer M Mariette C Braga M Consensus guidelines for enhanced recovery after gastrectomy: enhanced recovery after surgery (ERAS®) society recommendations. Br J Surg. (2014) 101:1209–29. 10.1002/bjs.9582 25047143

[28] Cai H Liu Z Wei F Yu M Xu N Li Z. 3D printing in spine surgery. Adv Exp Med Biol. (2018) 1093:345–59. 10.1007/978-981-13-1396-7_27 30306494

[29] Joliat G-R Kobayashi K Hasegawa K Thomson J-E Padbury R Scott M Guidelines for perioperative care for liver surgery: enhanced recovery after surgery (ERAS) society recommendations 2022. World J Surg. (2023) 47:11–34. 10.1007/s00268-022-06732-5 36310325 PMC9726826

[30] Di Saverio S Podda M De Simone B Ceresoli M Augustin G Gori A Diagnosis and treatment of acute appendicitis: 2020 update of the WSES Jerusalem Guidelines. World J Emerg Surg. (2020) 15:27. 10.1186/s13017-020-00306-3 32295644 PMC7386163

[31] Wisely JC Barclay KL. Effects of an enhanced recovery after surgery programme on emergency surgical patients. ANZ J Surg. (2016) 86:883–8. 10.1111/ans.13465 26990499

[32] Clet A Guy M Muir J Cuvelier A Gravier F Bonnevie T. Enhanced recovery after surgery (ERAS) implementation and barriers among healthcare providers in France: a cross- sectional study. Healthcare. (2024) 12:436. 10.3390/healthcare12040436 38391811 PMC10887527

[33] Morrison-Smith S Ruiz J. Challenges and barriers in virtual teams: a literature review. SN Appl Sci. (2020) 2:1096. 10.1007/s42452-020-2801-5

