# Supplementary material for: Advancing abdominal surgery recovery implementation: a unified framework for intensified recovery protocols by the EUropean PErioperative MEdical Networking collaborative
Source: Front Surg. 2026 May 18;13:1827678. doi: 10.3389/fsurg.2026.1827678 (PMC13223102; doi:10.3389/fsurg.2026.1827678)
Supplement: Supplementary file 9 [file Presentation1.pdf]

EUPEMEN
European Perioperative Medical Networking

Co-funded by the
Erasmus+ Programme
of the European Union

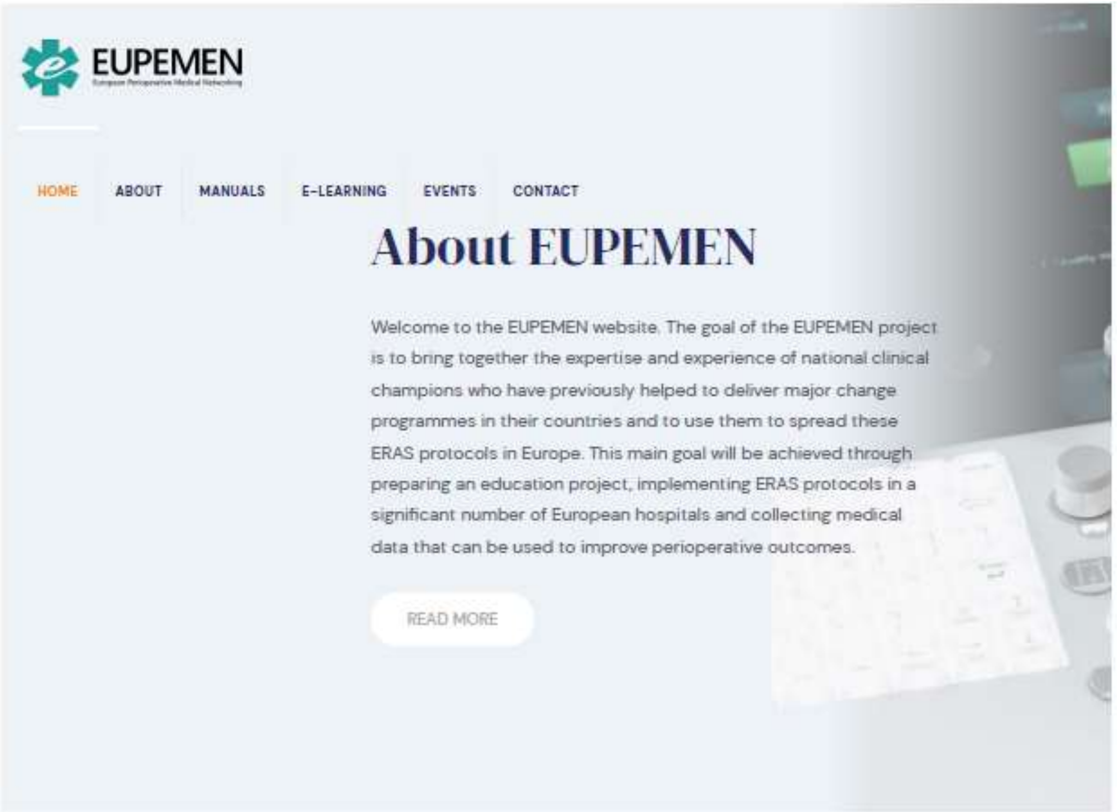

UNIVERSITAS Miguel Hernández

- https://eupemen.eu/

Co-funded by the
Erasmus+ Programme
of the European Union

EUPEMEN

HOME ABOUT MANUALS E-LEARNING EVENTS CONTACT

About us

The project is the result of a collaboration of five institutions in four different European countries

1. The Institute for Health Research Aragon (Spain)

Jose M Ramirez

Javier Martínez Ubieto

2. Hospital Presidium of the Azienda Unita Santiaria Locale (Italy)

Carlo Feo

3. The Miguel Hernandez University of Elche (Spain)

Antonio Arroyo

4. Second Faculty of Medicine of Charles University, Prague (Czech Republic)

Petr Kocian

5. General Hospital of Thessaloniki "George Papanikolaou" (Greece)

Orestis Ioannidis

Co-funded by the Erasmus+ Programme of the European Union

EUPEMEN
European Perioperative Medical Networking

HOME ABOUT MANUALS E-LEARNING EVENTS CONTACT

Contact us

Project coordinator:

Marta Teresa Fernández

Técnico de Gestión de Proyectos Unidad de Proyectos y Gestión Científica

Phone

976 71 64 17

E-mail

mteresa@iisaragon.es

Send us a message

Your name

Your e-mail

Subject

Message

Send a message

Co-funded by the Erasmus+ Programme of the European Union

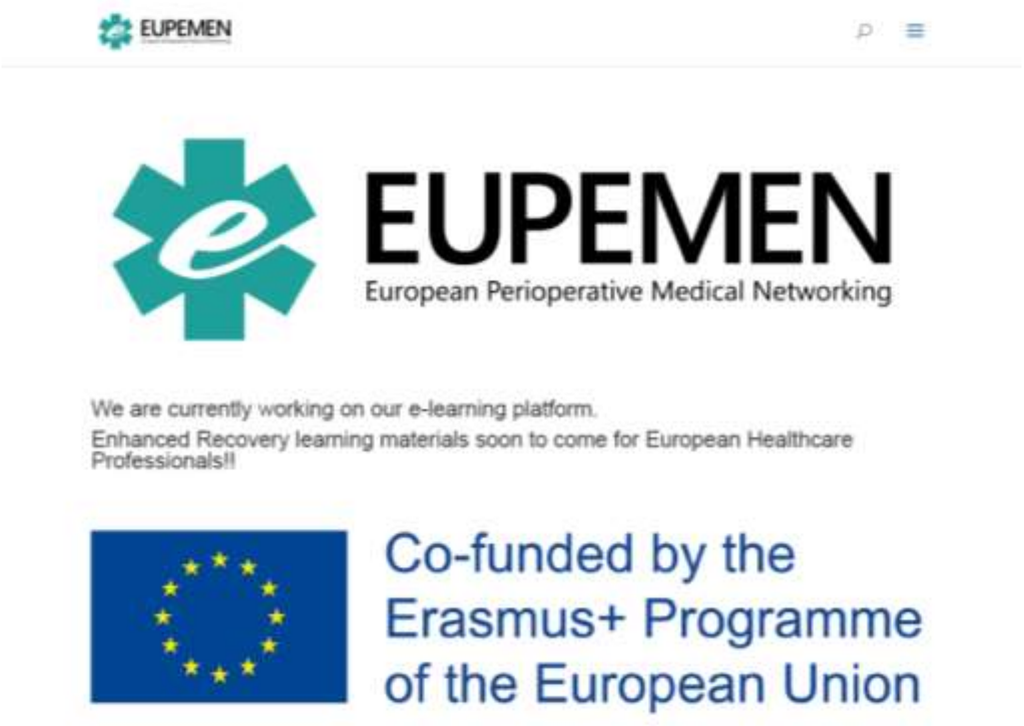

- https://eupemen-learning.com/

Co-funded by the
Erasmus+ Programme
of the European Union

EUPEMEN
European Perioperative Medical Networking

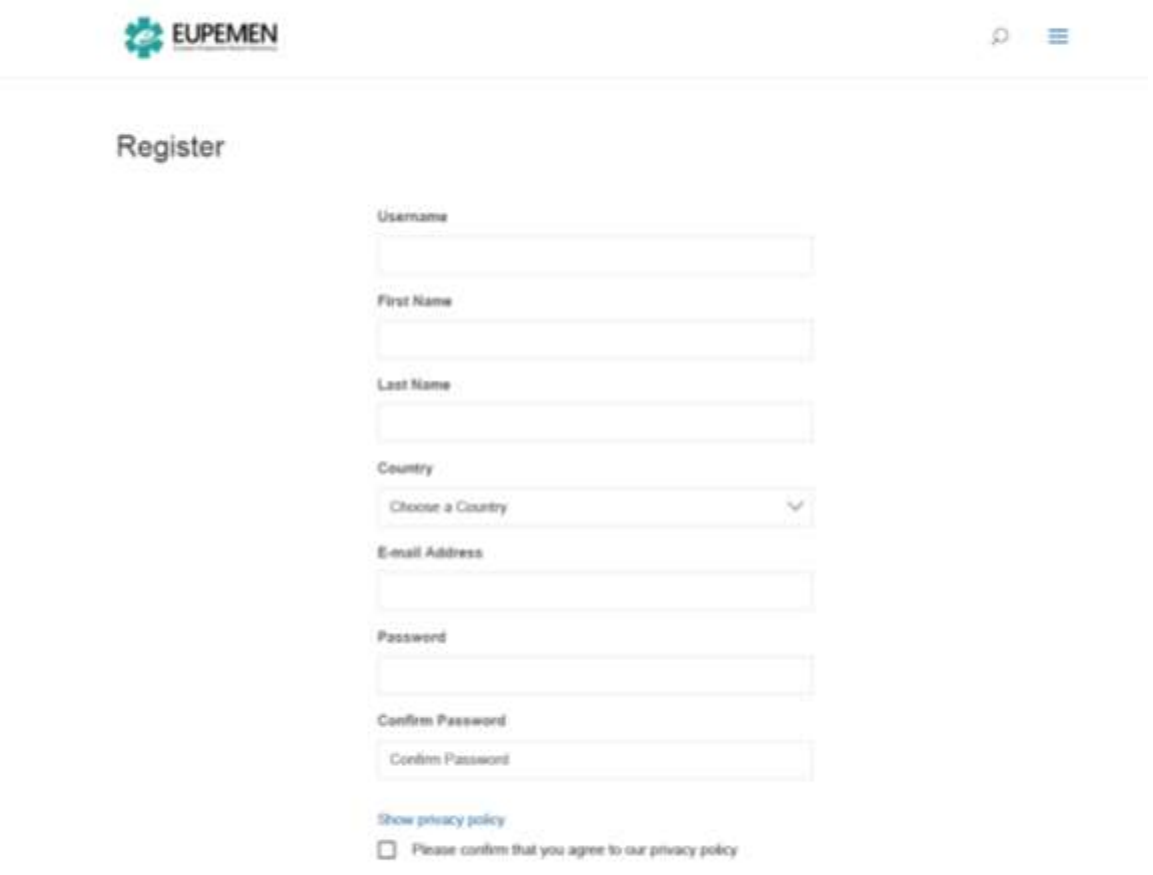

Co-funded by the
Erasmus+ Programme
of the European Union

EUPEMEN
European Perioperative Medical Networking

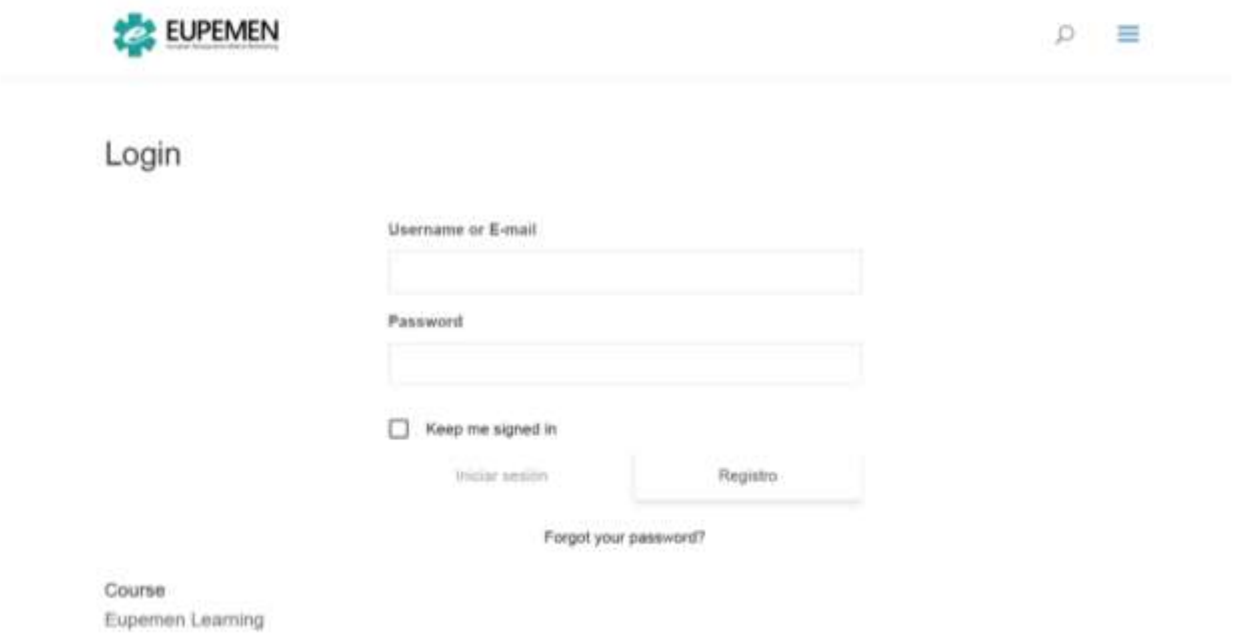

Co-funded by the
Erasmus+ Programme
of the European Union

Συζήτηση

EUPEMEN
European Perioperative Medical Networking

Forums

Home › Forums

Search

Forum	Topics	Posts	Last Post
Abdominal Surgery<br>Acute Apendicitis (1, 0), Bowel Obstruction (1, 0)	2	2	7 months, 2 weeks ago<br>admin
Bariatric	1	1	7 months, 2 weeks ago<br>admin
Colon Surgery<br>Left Hemicolectomy (1, 0), Right Hemicolectomy (1, 0), Total Colectomy (1, 0)	3	3	7 months, 2 weeks ago<br>admin
Gastric surgery	2	2	6 months, 1 week ago<br>Carlo Feo
Liver Resection	1	1	7 months, 2 weeks ago<br>admin
Oesophagectomy	1	1	7 months, 2 weeks ago<br>admin
General Discussion	1	1	6 months ago<br>Carlo Feo

Co-funded by the
Erasmus+ Programme
of the European Union

Online Learning Activity

EUPEMEN
European Perioperative Medical Networking

1. The RICA Pathway
2. Preparatory Preparation
3. RICA Pathway key points: surgery, drains and nasogastric tube
4. RICA Pathway key points: anaesthesia
5. RICA Pathway key points: nursery
6. RICA Pathway key points: nutrition
7. Implementation problems. Difficulties for adherence and sustainability of a fast-track protocol

Co-funded by the Erasmus+ Programme of the European Union
