# Supplementary material for: Advancing abdominal surgery recovery implementation: a unified framework for intensified recovery protocols by the EUropean PErioperative MEdical Networking collaborative
Source: Front Surg. 2026 May 18;13:1827678. doi: 10.3389/fsurg.2026.1827678 (PMC13223102; doi:10.3389/fsurg.2026.1827678)
Supplement: Supplementary file 10 [file Datasheet1.pdf]

Partners

The project is the result of a collaboration of five institutions in four different European countries.

1. **The Institute for Health Research Aragon**

The Institute for Health Research Aragon (IIS Aragon) was created in 2014 by the Government of Aragon and the University of Zaragoza, obtaining the Health Research Centre of excellence accreditation by the National Health Institute Carlos III (ISCIII) in order to facilitate research and innovation in Health Services, generating knowledge and transferring it to the healthcare services of Aragon. More than 1100 affiliated professionals are involved in 100 research groups whose activity is organised in 6 scientific programmes.

Jose M Ramirez

Dr. José Manuel Ramirez, the principal investigator of the Perioperative Surgery and Medicine Research Group of Institute for Health Research Aragon, is the current president of GERM, the Spanish Multimodal Rehabilitation Group. Its foundational objectives included the dissemination, implementation and maintenance of multimodal rehabilitation protocols in the different areas of Surgery. In this sense, worthy of note is the close collaboration that has existed since the beginning of 2013, between the GERM and the Spanish Ministry of Health, Social Services and Equality to develop a care plan aimed at reducing variability in clinical practice.

Javier Martínez Ubieto

Dr. Javier Martínez Ubieto is the main researcher of Muscle Relaxation and Residual Neuromuscular Block Study Group (Anesthesiology group) and has extensive experience in Multimodal Rehabilitation. He participated in the implementation of the Multimodal Rehabilitation in Radical Cystectomy program at the Miguel Servet Hospital from its inception. For this, the corresponding surgical and anesthetic protocols were prepared, which were presented in a clinical session in the departments involved, Anesthesiology and Urology. The results of this implementation were the subject of the doctoral thesis of Dr. Sonia Maria Ortega

Lucea, which was directed by Dr Martínez. The application of the program in radical cystectomy obtained positive results in terms of morbidity and hospital stay and were published in 2013 in the journal Actas Urológicas Españolas. In addition, this study received the Teaching Innovation award in the call for that year, as it is an innovative experience that clearly conditioned the modification of healthcare performance in that area.

2. **Hospital Presidium of the Azienda Unita Santiaria Locale Hospital Presidium of the Azienda**

Unita Santiaria Locale (AUSL) functionally aggregates three hospitals of the province of Ferrara with a view to integration and compatibility with the territorial distribution of the remaining health structures defining an overall organization capable of ensuring optimal integration between hospital and territorial functions. Health care functions are aggregated according to the departmental organizational model, considering the affinity of the different disciplines or the complementarity existing between the specialist branches (Emergency, Surgery, Maternal and Child, and Diagnostic imaging). They are networked ensuring functional integration, management efficiency, technical competence and performance effectiveness. The departments cross the perimeters of the individual hospitals.

SERVIZIO SANITARIO REGIONALE
EMILIA-ROMAGNA
Azienda Unità Sanitaria Locale di Ferrara

Carlo Feo

Carlo Feo is the chief of the Unit of General Surgery of the AUSL of Ferrara and deputy head of the Department of Surgery. He is Professor of Surgery at the University of Ferrara. In 2011, at the UH of Ferrara, Carlo Feo has constituted an Enhanced Recovery Program (ERP) multidisciplinary team including surgeons, anaesthesiologists, and clinical nurses with different and complementary scientific backgrounds to implement an integrate a perioperative care protocol founded on the elements selected on the basis of high-grade evidence of clinical efficacy.

3.The Miguel Hernandez University of Elche

The Miguel Hernandez University of Elche (UMH) is a public and modern university that has begun its journey with enthusiasm and energy, with the resolute purpose of offering the highest quality education and research and is committed to finding solutions to the problems affecting society. It has four campuses located in the province of Alicante, Spain. According to various university quality indicators, the UMH is found among the top Spanish universities in the

parameters of innovation and technological development, in teaching, and in the area of industry, innovation, and infrastructure.

Antonio Arroyo

Antonio Arroyo is a general surgeon with special dedication to colorectal surgery and a Professor at the university, and belongs to the Board of Directors of the GERM and will chair the next national congress on multimodal rehabilitation in October 2020 in Alicante. He is the author of several JCR publications on this topic and researcher in different competitive projects nationwide.

4.The Department of Surgery at the Second Faculty of Medicine of Charles University, Prague

The Department of Surgery at the Second Faculty of Medicine of Charles University is widely regarded as a national leader in colorectal surgery and demonstrates expertise through advanced medical care, leadership in education and clinical research. A full spectrum of minimally invasive surgeries for colorectal cancer, including laparoscopic, robotic and transanal techniques, are performed. In cooperation with the Department of Anaesthesiology and enhanced recovery after surgery protocol (enhanced recovery) was implemented in colorectal surgery in 2016. Since then until January 2020, the enhanced recovery protocol has been used in 500 patients undergoing elective colorectal resections for neoplastic or diverticular diseases aged 18 years and older. The clinical and cost effectiveness of the enhanced recovery protocol has been verified by several studies published in peer-reviewed journals.

Petr Kocian

Petr Kocian is a consultant surgeon at the Department of Surgery at the Second Faculty of Medicine. He was instrumental in setting up the enhanced recovery protocol in patients undergoing colorectal surgery. He performs mini-invasive colorectal surgery and holds

memberships in the most prominent colorectal surgery organizations such as the European Society for Coloproctology and European Association for Endoscopic Surgery. He has more than 30 research papers published by various reputed and peer-reviewed journals.

5.General Hospital of Thessaloniki “George Papanikolaou”

The origins of the General Hospital of Thessaloniki “George Papanikolaou” date back to 1920, when the Greek State founded a Sanatorium for patients with tuberculosis. Over the years, the General Hospital has evolved into a major institution for advanced medical care whilst at the same time providing a strong foundation for clinical and scientific research. Its clinical reputation was built on the treatment of medical conditions notably of the heart and lungs as well as of the hematopoietic system. The General Hospital of Thessaloniki has 650 beds. It employs over 1200 medical doctors, biomedical scientists, nurses and other qualified personnel.

ΓΕΝΙΚΟ ΝΟΣΟΚΟΜΕΙΟ ΘΕΣΣΑΛΟΝΙΚΗΣ
“Γ. ΠΑΠΑΝΙΚΟΛΑΟΥ”

Orestis Ioannidis

Orestis Ioannidis graduated from Medicine at Aristotle University of Thessaloniki, Thessaloniki, Greece. He completed an MSC in Medical Research Methodology and a PhD in experimental surgery and nutrition in 2014. He is responsible for artificial nutrition, clinical nutrition and enhanced recovery protocols in the 4th Surgical Department of the Aristotle University of Thessaloniki, in general hospital "G. Papanikolaou", where he is an attending Surgeon and Academic Fellow. He is a trainer of the Hellenic Surgical Society and integral part of the Nutrition Education Team. He has given a significant number of invited lectures on enhanced recovery protocols and has published over 100 papers in peer-reviewed journals.
