# Supplementary material for: Advancing abdominal surgery recovery implementation: a unified framework for intensified recovery protocols by the EUropean PErioperative MEdical Networking collaborative
Source: Front Surg. 2026 May 18;13:1827678. doi: 10.3389/fsurg.2026.1827678 (PMC13223102; doi:10.3389/fsurg.2026.1827678)
Supplement: Supplementary file 11 [file Datasheet2.pdf]

Co-funded by the
Erasmus+ Programme
of the European Union

EUPEMEN
European Perioperative Medical Networking

EUPEMEN: PRESS RELEASE AND NEWS

PROJECT: Erasmus+ Eupemen- 2020-1-ES01-KA203-082681

DESCRIPTION: This document gathers the press release and / or the news that the Eupemen project is generating.

PERIODIC REPORT: 3 ➔ from 01/09/2020 – 31/10/2022

This document gather all the news generated due to the impact of the Eupemen project in media from all the partners in 4 different countries. Around 40 news have been generated and gathered by all the partners.

https://www.iisaragon.es/el-instituto-de-investigacion-sanitaria-aragon-coordinara-un-erasmus-para-formar-a-profesionales-implicados-en-perioperatorios/

Instituto de Investigación Sanitaria Aragón

INSTITUTO ⌄ GRUPOS DE INVESTIGACIÓN SERVICIOS ⌄ ACTUALIDAD ⌄ EMPLEO iCompras DONAR

EL INSTITUTO DE INVESTIGACIÓN SANITARIA ARAGÓN COORDINARÁ UN ERASMUS+ PARA FORMAR A PROFESIONALES IMPLICADOS EN PERIOPERATORIOS

Inicio > Investigación > El Instituto de Investigación Sanitaria Aragón coordinará un Erasmus+ para formar a profesionales implicados en perioperatorios

Publicado por iisaragon el 14 octubre, 2020 Tags ▾ Categorías ▾

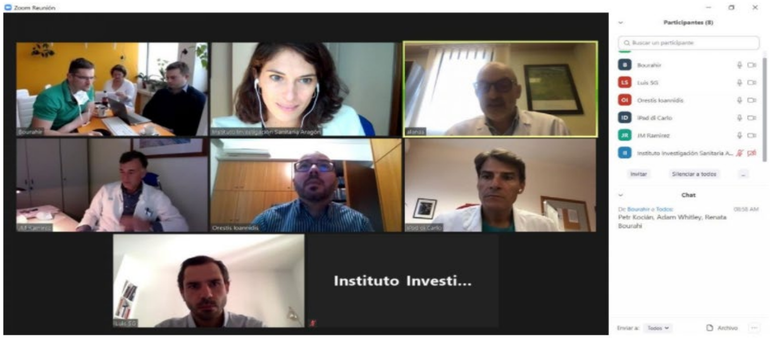

Los grupos de cirugía y medicina perioperatoria y de estudio de relajación muscular y bloqueo neuromuscular residual lideran el proyecto

El plan incluye la participación de cinco socios de cuatro países, que desarrollarán los protocolos que se aplicarán en los hospitales

El Instituto de Investigación Sanitaria Aragón (IIS Aragón) coordinará por primera vez un proyecto Erasmus+ en el ámbito de la educación superior llamado *European perioperative medical networking (Eupemen Project)*, financiado por la Unión Europea. Se trata de un programa para formar a profesionales multidisciplinares directamente implicados en el procedimiento perioperatorio, además de crear una red de docentes con capacidad para enseñar a estos grupos en los hospitales y auditar la correcta aplicación de los protocolos de manera estandarizada y homogénea. El presupuesto asciende a casi 192.000 euros y la duración es de 26 meses, hasta el 31 de octubre de 2022. Este jueves se celebró la primera reunión virtual que sirvió como arranque del proyecto, con la asistencia de todos los socios y en la que el doctor Ángel Lanas, director científico del IIS Aragón, dio la bienvenida a los participantes.

Dos equipos de investigación del IIS Aragón lideran el proyecto: el grupo de cirugía y medicina perioperatoria, liderado por José Manuel Ramírez, y el de estudio de relajación muscular y bloqueo neuromuscular residual, con Javier Martínez Ubieto como investigador principal. En el proyecto también participa la doctora Ana María Pascual Bellosta. El objetivo del proyecto es recopilar y compartir la experiencia de la aplicación de la rehabilitación multimodal con diversos profesionales de hospitales europeos y facilitar su divulgación.

En el plan participan cinco socios de España, Italia, República Checa y Grecia, que están desarrollando los protocolos necesarios con el fin de poder implementar el programa de recuperación en, al menos, cinco hospitales de Europa. A largo plazo, se persigue disminuir los efectos secundarios después de la cirugía para los pacientes y, en consecuencia, lograr una mejoría más rápida; reducir la mortalidad tras las cirugías; y disminuir el tiempo de estancia hospitalaria, con el consiguiente ahorro económico para el sistema sanitario y el aumento de disponibilidad de camas para otros pacientes.

Entre los destinatarios se incluyen no solo los profesionales de la salud que están directamente a cargo de la atención al paciente quirúrgico (cirujanos, anestesistas y enfermeras), sino también a todos aquellos profesionales que, de una u otra forma, están relacionados con el tratamiento interdisciplinario de estos enfermos: nutricionistas, fisioterapeutas, rehabilitadores, digestólogos, radioterapeutas, oncólogos o patólogos. Dado que la eficacia (reducción de estancias hospitalarias y optimización del uso de otros recursos) es una de las ventajas de estos programas, también se beneficiarán del proyecto los gestores de los centros de salud, los responsables clínicos y los coordinadores de calidad. Asimismo, los médicos de atención primaria y los pacientes también tienen un papel muy activo.

Acerca del Instituto de Investigación Sanitaria Aragón

El IIS Aragón es el Instituto de Investigación Sanitaria del complejo hospitalario formado por los Hospitales Docentes y Universitarios "Hospital Clínico Universitario Lozano Blesa" y "Hospital Universitario Miguel Servet" y la Atención Primaria de Salud. A este complejo hospitalario se le asocian a través de distintos instrumentos jurídicos, la Universidad de Zaragoza y el Instituto Aragonés de Ciencias de la Salud.

Los objetivos del IIS Aragón son aproximar la investigación básica y aplicada, clínica y de servicios sanitarios; crear un entorno investigador, asistencial y docente de calidad al que queden expuestos los profesionales sanitarios, los especialistas en formación y los alumnos de postgrado y grado, así como constituir el lugar idóneo para la captación de talento y la ubicación de las grandes instalaciones científico-tecnológicas.

Acerca de Erasmus+

Erasmus+ (2014-2020) es el programa integrado de la Unión Europea (UE) en los ámbitos de la educación y la formación, juventud y deporte, que ofrece oportunidades para todas las personas y en todos los sectores educativos (Educación Escolar, Formación Profesional, Educación Superior y Educación de Personas Adultas). En España, el programa Erasmus+ se gestiona por parte del Servicio Español para la Internacionalización de la Educación (SEPIE), que actúa como Agencia Nacional del programa en los ámbitos de la educación y la formación y que está adscrito al Ministerio de Universidades. El proyecto Eupemen se enmarca dentro de la Acción Clave 203 Asociaciones estratégicas, donde instituciones transnacionales vinculadas con la Educación Superior cooperan en el desarrollo de productos intelectuales innovadores que impliquen una mejora en los sistemas y estructuras de la Educación Superior.

http://www.unife.it/it/notizie/2020/vita/eupenem-project

Università degli Studi di Ferrara

Home / Notizie / 2020 / Vita universitaria / EUPENEM | AUSL Ferrara e Unife partecipano al programma che forma i professionisti del post-operatorio

- Covid-19 | Notizie e info
- Scienza, cultura e ricerca
- Vita universitaria
- Persone
- Unife e le imprese

Notizia

Condividi

EUPENEM | AUSL Ferrara e Unife partecipano al programma che forma i professionisti del post-operatorio

18/11/2020

VITA UNIVERSITARIA

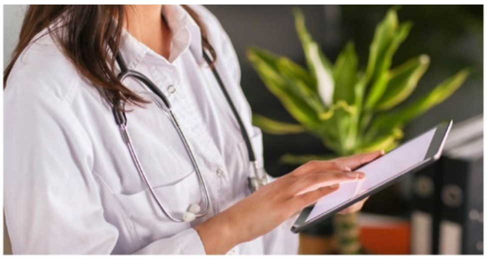

Cinque partner provenienti da **Spagna, Italia, Repubblica Ceca e Grecia** e un **obiettivo** importante **in ambito medico-scientifico: formare i professioni del post-operatorio.**

Questa la mission del **progetto Erasmus+ nel campo dell'istruzione superiore** ***EUropean PErioperative MEdical Networking*** (***EUPENEM Project***), coordinato dall'Istituto ***Aragon Health Research*** (Spagna), a cui partecipa per la prima volta l'**Azienda Unità Sanitaria Locale di Ferrara** e che vede coinvolti **diversi docenti della Facoltà di Medicina, Farmacia e Prevenzione dell'Università di Ferrara.**

Il progetto è iniziato lo scorso 8 ottobre, dopo il primo incontro virtuale e avrà durata di **ventisei mesi** e un budget di circa **192.000 euro**.

EUPENEM si propone di **formare professionisti appartenenti a più discipline**, direttamente coinvolti nelle **cure peri-operatorie** e di creare una rete di **docenti che possa insegnare la corretta applicazione dei protocolli di cura** negli ospedali interessati. I **cinque partner** che partecipano al progetto stanno sviluppando i protocolli necessari per attuare il programma in almeno cinque ospedali europei.

Tre gruppi di ricerca italiani sono attivamente coinvolti nel progetto: il **gruppo dei chirurghi generali dell'Azienda USL di Ferrara**, il **gruppo dei medici dello sport del Centro Studi Scienze Motorie e Sportive dell'Università di Ferrara** e il **programma di Ortogeriatria dell'Azienda Ospedaliero Universitaria di Ferrara**, coordinati, rispettivamente, dai **Professori dell'Università di Ferrar Carlo Feo, Giovanni Grazzi, Gianni Mazzoni e Stefano Volpato**.

Il progetto nasce per condividere e promuovere fra vari professionisti di ospedali europei l'esperienza maturata nell'applicazione di programmi multimodali per **migliorare il recupero post-operatorio**.

Tra gli **obiettivi a lungo termine** la **riduzione delle complicanze** dopo l'intervento chirurgico con un recupero più rapido del paziente, **della mortalità** dopo interventi chirurgici e **della durata della degenza ospedaliera**, con conseguente risparmio economico per il sistema sanitario e aumento della disponibilità di posti letto per altri ricoveri.

Tra i **destinatari del programma** EUPENEM rientrano sia gli **operatori sanitari** che si occupano direttamente della cura del paziente chirurgico (chirurghi, anestesisti e infermieri) sia i **professionisti coinvolti nel trattamento** interdisciplinare di questi pazienti quali i medici di medicina generale, nutrizionisti, fisioterapisti, gastroenterologi, radioterapisti, oncologi e patologi.

A beneficiare del progetto saranno le **dirigenze delle aziende sanitarie**, i **direttori delle attività cliniche** e i **responsabili della qualità**, poiché tra i vantaggi attesi dal programma vi è anche l'efficienza, intesa come riduzione della degenza ospedaliera e ottimizzazione dell'uso delle risorse.

Erasmus+ è un programma dell'Unione Europea nei settori dell'istruzione, della formazione, della gioventù e dello sport che offre opportunità per tutte le persone e in tutti i settori dell'istruzione: Istruzione Scolastica, Formazione Professionale, Istruzione Superiore e Istruzione degli Adulti. In Italia, il programma è coordinato da tre agenzie nazionali coordinate dal Ministero dell'Istruzione, Università e Ricerca - MIUR

https://www.ausl.fe.it/home-page/news/erasmus-per-formare-i-professionisti-del-post-operatorio-azienda-unita-sanitaria-locale-e-universita-di-ferrara-insieme-nel-programma-eupenem-finanziato-dallunione-europea

SERVIZIO SANITARIO REGIONALE
EMILIA-ROMAGNA
Azienda Unità Sanitaria Locale di Ferrara

AZIENDA USL FERRARA

struttura aziendale | area dipendenti | 118 | ufficio stampa | elenco telefonico aziendale | portale fornitori | sistei

tu sei qui: home / home page / news / erasmus+ per formare i professionisti del post-operatorio: azienda unità sanitaria locale e università di ferrara ins dall'unione europea

- Amministrazione Trasparente
- Informazione scientifica sul farmaco
- Ufficio Relazioni con il Pubblico
- Atto aziendale
- Posta elettronica certificata
- Progetti aziendali
- Albo pretorio online
- Formazione e Biblioteca

Bacheca

- Fatturazione elettronica e split payment
- Eventi e Convegni
- Accesso ai documenti
- Autocertificazioni e Autodichiarazioni
- Bandi e concorsi
- Gare ed Appalti
- CUG - Comitato Unico di Garanzia
- Personale medico in convenzione
- Privacy
- Primo Piano Farmaceutica
- Sistema Gestione Ambientale
- Servizio Civile Volontario
- Piano Triennale per la razionalizzazione delle Attrezzature
- Convenzioni Celiachia

Profilo Committente

Vigilanza

- Sicurezza Alimentare

Erasmus+ per formare i professionisti del post-operatorio: Azienda Unità Sanitaria Locale e Università di Ferrara insieme nel programma EUPENEM finanziato dall'Unione Europea

pubblicato il 24/11/2020 08:48, ultima modifica 24/11/2020 08:48

Il progetto, iniziato lo scorso 8 Ottobre dopo il primo incontro virtuale, avrà durata di ventisei mesi e un budget di circa € 192.000 €uro. L'obiettivo è promuovere fra i professionisti di ospedali europei l'esperienza maturata nell'applicazione di programmi per migliorare il recupero post-operatorio.

Erasmus+

Ferrara, 24-11-2020. L'Azienda Unità Sanitaria Locale di Ferrara partecipa per la prima volta ad un progetto Erasmus+ nel campo dell'istruzione superiore chiamato ***EUropean PErioperative MEdical Networking*** (**EUPENEM Project**) coordinato dall'Istituto *Aragon Health Research* (Spagna). Il progetto, iniziato lo scorso 8 Ottobre dopo il primo incontro virtuale, avrà durata di ventisei mesi e un budget di circa € 192.000 €uro.

EUPENEM ha l'obiettivo di formare professionisti -appartenenti a più discipline- direttamente coinvolti nelle cure peri-operatorie e di creare una rete di docenti che possa insegnare la corretta applicazione dei protocolli di cura negli ospedali interessati. Cinque partner provenienti da Spagna, Italia, Repubblica Ceca e Grecia partecipano al progetto e stanno sviluppando i protocolli necessari per attuare il programma in almeno cinque ospedali europei.

Tre gruppi di ricerca italiani sono attivamente coinvolti nel progetto: il gruppo dei chirurghi generali dell'Azienda USL di Ferrara, il gruppo dei medici dello sport del Centro Studi Scienze Motorie e Sportive dell'Università di Ferrara e il programma di Ortogeriatria dell'Azienda Ospedaliero Universitaria di Ferrara, coordinati, rispettivamente, dai professori dell'Università di Ferrara Carlo Feo, Giovanni Grazzi, Gianni Mazzoni e Stefano Volpato.

L'obiettivo del progetto è condividere e promuovere fra vari professionisti di ospedali europei l'esperienza maturata nell'applicazione di programmi multimodali per migliorare il recupero post-operatorio.

Obiettivi a lungo termine sono:

- ridurre le complicanze dopo l'intervento chirurgico con un recupero più rapido del paziente;
- ridurre la mortalità dopo interventi chirurgici;
- diminuire la durata della degenza ospedaliera, con conseguente risparmio economico per il sistema sanitario e aumento della disponibilità di posti letto per altri ricoveri.

Tra i **destinatari del programma** EUPENEM rientrano sia gli operatori sanitari che si occupano direttamente della cura del paziente chirurgico (chirurghi, anestesisti e infermieri) sia i professionisti coinvolti nel trattamento interdisciplinare di questi pazienti quali i medici di medicina generale, nutrizionisti, fisioterapisti, gastroenterologi, radioterapisti, oncologi e patologi.

Dirigenza delle aziende sanitarie, direttori delle attività cliniche e responsabili della qualità beneficeranno anche loro del progetto poiché l'efficienza, intesa come riduzione della degenza ospedaliera e ottimizzazione dell'uso delle risorse, è uno dei vantaggi attesi del programma.

Erasmus+ è un programma dell'Unione Europea nei settori dell'istruzione, della formazione, della gioventù e dello sport che offre opportunità per tutte le persone e in tutti i settori dell'istruzione: Istruzione Scolastica, Formazione Professionale, Istruzione Superiore e Istruzione degli Adulti.

In Italia, il programma è coordinato da tre agenzie nazionali coordinate dal Ministero dell'Istruzione, Università e Ricerca - MIUR http://www.erasmusplus.it/

https://gpapanikolaou.gr/anakoinoseis/2020-12-28/11131/%CF%83%CF%85%CE%BC%CE%BC%CE%B5%CF%84%CE%BF%CF%87%CE%AE-%CF%84%CE%B7%CF%82-%CE%B4-%CF%87%CE%B5%CE%B9%CF%81%CE%BF%CF%85%CF%81%CE%B3%CE%B9%CE%BA%CE%AE%CF%82-%CE%BA%CE%BB%CE%B9%CE%BD%CE%B9%CE%BA/

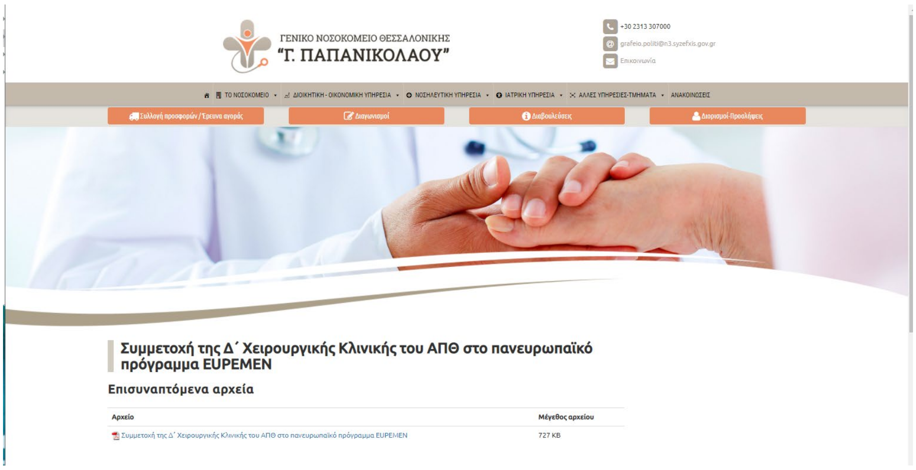

https://www.med.auth.gr/article/i-d-heiroyrgiki-kliniki-sto-paneyropaiko-programma-eupemen

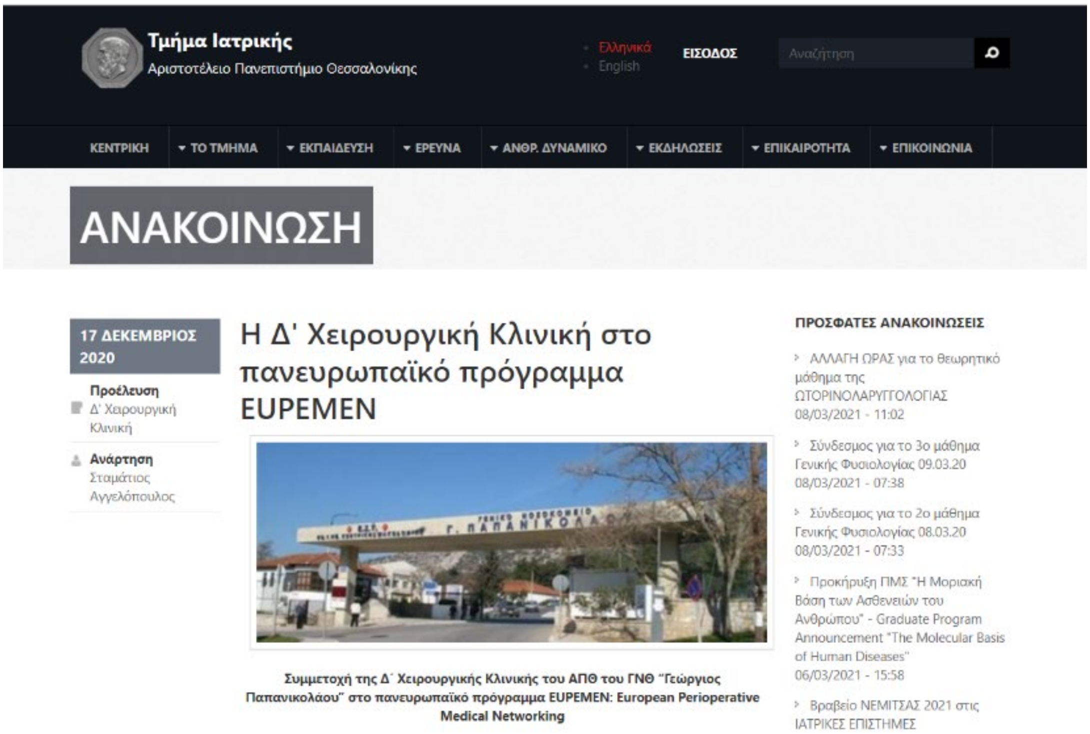

Η Δ΄ Χειρουργική Κλινική του Αριστοτελείου Πανεπιστημίου Θεσσαλονίκης, με έδρα το Γενικό Νοσοκομείο Θεσσαλονίκης "Γεώργιος Παπανικολάου", θα συμμετέχει σε ένα πρόγραμμα Erasmus+ για την εκπαίδευση επαγγελματιών υγείας που εμπλέκονται στην περιεγχειρητική αντιμετώπιση ασθενών. Το πρόγραμμα περιλαμβάνει τη συμμετοχή πέντε εταίρων από τέσσερις χώρες, οι οποίοι θα αναπτύξουν τα πρωτόκολλα που θα εφαρμοστούν στα νοσοκομεία υπό τον συντονισμό του Ινστιτούτου Έρευνας για την Υγεία της Αραγονίας IIS Aragon.

Η Δ΄ Χειρουργική Κλινική του Αριστοτελείου Πανεπιστημίου Θεσσαλονίκης, με έδρα το Γενικό Νοσοκομείο Θεσσαλονίκης "Γεώργιος Παπανικολάου" θα συμμετέχει στην ανάπτυξη για πρώτη φορά σε ένα έργο Erasmus+ στον τομέα της ανώτατης εκπαίδευσης που ονομάζεται EUPEMEN: European Perioperative Medical Networking (ευρωπαϊκή περιεγχειρητική ιατρική δικτύωση), χρηματοδοτούμενο από την Ευρωπαϊκή Ένωση υπό την αιγίδα του Ινστιτούτου Έρευνας για την Υγεία της Αραγονίας IIS Aragon. Είναι ένα πρόγραμμα για την εκπαίδευση επαγγελματιών υγείας διαφόρων ειδικοτήτων που συμμετέχουν άμεσα στην περιεγχειρητική φροντίδα των ασθενών, και επιπρόσθετα για την δημιουργία ενός δικτύου εκπαιδευτών με την ικανότητα να διδάσκουν αυτές τις ομάδες σε νοσοκομεία και να ελέγχουν τη σωστή εφαρμογή των πρωτοκόλλων με τυποποιημένο και ομοιογενή τρόπο. Ο προϋπολογισμός ανέρχεται σε σχεδόν 192.000 ευρώ και η διάρκεια είναι 26 μήνες, έως τις 31 Οκτωβρίου 2022. Την Πέμπτη 8 Οκτωβρίου 2020 πραγματοποιήθηκε η πρώτη διαδικτυακή συνάντηση που σηματοδότησε και την αρχή του έργου, με τη συμμετοχή όλων των εταίρων στην οποία ο Δρ Ángel Lanas, Επιστημονικός Διευθυντής του IIS Aragón, καλωσόρισε τους συμμετέχοντες.

Δύο ερευνητικές ομάδες από το IIS Aragón ηγούνται του έργου: η ομάδα περιεγχειρητικής ιατρικής και χειρουργικής, με επικεφαλής τον José Manuel Ramírez, και η ομάδα μελέτης χαλάρωσης των μυών και αποκλεισμού των υπολειπόμενων νευρομυϊκών ομάδων, με κύριο ερευνητή τον Javier Martínez Ubieto. Ο στόχος του έργου είναι να συλλέξει και να μοιραστεί την εμπειρία της εφαρμογής πολυπαραγοντικής αποκατάστασης με διάφορους επαγγελματίες από ευρωπαϊκά νοσοκομεία και να διευκολύνει τη διάδοσή τους. Πέντε εταίροι από την Ισπανία, την Ιταλία, την Τσεχία και την Ελλάδα συμμετέχουν στο σχέδιο και θα αναπτύξουν τα απαραίτητα πρωτόκολλα προκειμένου να εφαρμόσουν το πρόγραμμα αποκατάστασης σε τουλάχιστον πέντε νοσοκομεία στην Ευρώπη. Όσον αφορά την Ελλάδα, εκπρόσωπός της στο πρόγραμμα Eupemen είναι η Δ΄ Χειρουργική Κλινική του Αριστοτελείου Πανεπιστημίου Θεσσαλονίκης του Γενικού Νοσοκομείου Θεσσαλονίκης "Γεώργιος Παπανικολάου" με επικεφαλής τον κ. Ορέστη Ιωαννίδη, Χειρουργό, Πανεπιστημιακό Υπότροφο.

06/03/2021 - 15:54

Θεωρητικά μαθήματα Ωτορινολαρυγγολογίας - πρόγραμμα
05/03/2021 - 12:50

Προφορική δοκιμασία ΔΔ
05/03/2021 - 12:45

ΟΡΘΗ ΕΠΑΝΑΚΟΙΝΟΠΟΙΗΣΗ, ΚΛΙΝΙΚΗ ΒΙΟΧΗΜΕΙΑ πρόγραμμα μαθημάτων 2020-2021
05/03/2021 - 12:43

TAGS CLOUD

Υποψήφιοι διδάκτορες Υποτροφίες Συνέλευση Συνέδρια Πρωτοετείς ΠΜΣ Ιατρικής ΑΠΘ ΠΜΣ Κρίσεις μελών ΔΕΠ Θέσεις υποψηφίων διδακτόρων Εξετάσεις προπτυχιακού Ενημέρωση προπτυχιακών Εγγραφές σε μαθήματα

Μακροπρόθεσμα, ο στόχος είναι να μειωθούν οι ανεπιθύμητες ενέργειες μετά τη χειρουργική επέμβαση στους ασθενείς και, κατά συνέπεια, να επιτευχθεί ταχύτερη ανάρρωση, μείωση της θνησιμότητας μετά από χειρουργικές επεμβάσεις και μειωμένη διάρκεια παραμονής στο νοσοκομείο, με την επακόλουθη οικονομική εξοικονόμηση για το σύστημα υγείας και την αύξηση της διαθεσιμότητας νοσηλευτικών κλινών για άλλους ασθενείς.

Το πρόγραμμα απευθύνεται όχι μόνο σε επαγγελματίες υγείας που είναι άμεσα υπεύθυνοι για τη χειρουργική φροντίδα ασθενών (χειρουργοί, αναισθησιολόγοι και νοσηλευτές) αλλά και σε όλους εκείνους τους επαγγελματίες που, με τον έναν ή τον άλλο τρόπο, σχετίζονται με τη διεπιστημονική θεραπεία αυτών των ασθενών: διατροφολόγοι, φυσιοθεραπευτές, φυσίατροι, εργοθεραπευτές, γαστρεντερολόγοι, ακτινοθεραπευτές, ογκολόγοι. Δεδομένου ότι η αποτελεσματικότητα (μείωση της παραμονής στο νοσοκομείο και βελτιστοποίηση της χρήσης άλλων πόρων) είναι ένα από τα πλεονεκτήματα αυτών των προγραμμάτων, οι διοικητές των νοσηλευτικών ιδρυμάτων, οι διευθυντές ιατρικής και νοσηλευτικής υπηρεσίας και οι υπεύθυνοι ποιότητας θα επωφεληθούν επίσης από το έργο. Ομοίως, οι γιατροί πρωτοβάθμιας περίθαλψης και οι ασθενείς θα έχουν επίσης πολύ ενεργό ρόλο.

Σχετικά με την Δ΄ Χειρουργική Κλινική του Αριστοτελείου Πανεπιστημίου Θεσσαλονίκης και το Γενικό Νοσοκομείο Θεσσαλονίκης "Γεώργιος Παπανικολάου"

Η Δ΄ Χειρουργική Κλινική λειτουργεί στο Γενικό Νοσοκομείο Θεσσαλονίκης "Γεώργιος Παπανικολάου" από το 1988, δύο χρόνια μετά την ίδρυση της το 1986. Η κλινική υπό την διεύθυνση του Καθηγητή Χειρουργικής Σταματίου Αγγελόπουλου αποτελεί σημείο αναφοράς στην παροχή υψηλού επιπέδου υπηρεσιών υγείας, στην εκπαίδευση και στο πεδίο της έρευνας. Είναι μια κλινική με πολυδιάστατο έργο που εξελίσσεται συνέχεια, με ειδικό βάρος στη διενέργεια χειρουργικών επεμβάσεων σε όλα τα επίπεδα βαρύτητας και με πρωτοποριακές μεθόδους που εφαρμόζονται στην πόλη της Θεσσαλονίκης, με σημαντική παρουσία για το Νοσοκομείο. Το νοσοκομείο "Γεώργιος Παπανικολάου" είναι ένα τριτοβάθμιο νοσηλευτικό ίδρυμα το οποίο διαθέτει τμήματα όλων των ιατρικών ειδικοτήτων (πλην γυναικολογικών, παιδιατρικών και ουρολογικών) και είναι στελεχωμένο με άρτια καταρτισμένο ιατρικό, νοσηλευτικό, παραϊατρικό, τεχνικό και διοικητικό προσωπικό, με τη συμβολή των οποίων παρέχονται με ανθρωπιά και υπευθυνότητα υψηλού επιπέδου ποιοτικές υπηρεσίες υγείας.

Σχετικά με το πρόγραμμα Erasmus+

Το Erasmus+ (2014-2020) είναι το ολοκληρωμένο πρόγραμμα της Ευρωπαϊκής Ένωσης (ΕΕ) στους τομείς της εκπαίδευσης και της κατάρτισης, της νεολαίας και του αθλητισμού, το οποίο προσφέρει ευκαιρίες σε όλους τους ανθρώπους και σε όλους τους εκπαιδευτικούς τομείς (Σχολική Εκπαίδευση, Επαγγελματική Κατάρτιση, Ανώτατη Εκπαίδευση και Εκπαίδευση Ενηλίκων). Στην Ισπανία, το πρόγραμμα Erasmus+ διαχειρίζεται η Ισπανική Υπηρεσία Διεθνοποίησης της Εκπαίδευσης (SEPIE), η οποία ενεργεί ως Εθνική Υπηρεσία για το πρόγραμμα στους τομείς της εκπαίδευσης και της κατάρτισης και είναι συνδεδεμένη με το Υπουργείο Πανεπιστημίων. Το έργο Eupemen αποτελεί μέρος του στρατηγικού πλάνου δράσης 203, όπου διακρατικά ιδρύματα που συνδέονται με την τριτοβάθμια εκπαίδευση συνεργάζονται στην ανάπτυξη καινοτόμων πνευματικών προϊόντων που συνεπάγονται βελτίωση στα συστήματα και τις δομές της τριτοβάθμιας εκπαίδευσης.

Tags: Κινητικότητα

Επισυναπτόμενο αρχείο:

Δελτίο τύπου - Θεσσαλονίκη, Δεκέμβριος 17, 2020

https://www.estense.com/?p=883938

https://lanuovaferrara.gelocal.it/ferrara/cronaca/2020/11/25/news/il-progetto-eupenem-verso-nuovi-protocolli-negli-ospedali-europei-1.39584589

https://it.geosnews.com/p/it/emilia-romagna/fe/ferrara/azienda-unit-sanitaria-locale-e-universit-di-ferrara-insieme-nel-programma-eupenem-finanziato-dall-unione-europea_31738988

https://ne-np.facebook.com/AUSLFe/posts/1736862616477760

https://www.ausl.fe.it/home-page/news/erasmus-per-formare-i-professionisti-del-post-operatorio-azienda-unita-sanitaria-locale-e-universita-di-ferrara-insieme-nel-programma-eupenem-finanziato-dallunione-europea

https://salute.regione.emilia-romagna.it/notizie/ausl-fe

http://www.unife.it/it/notizie/2020/vita/eupenem-project

https://www.lf2.cuni.cz/veda-a-vyzkum/granty-a-projekty/projekty-resene-na-2-lekarske-fakulte

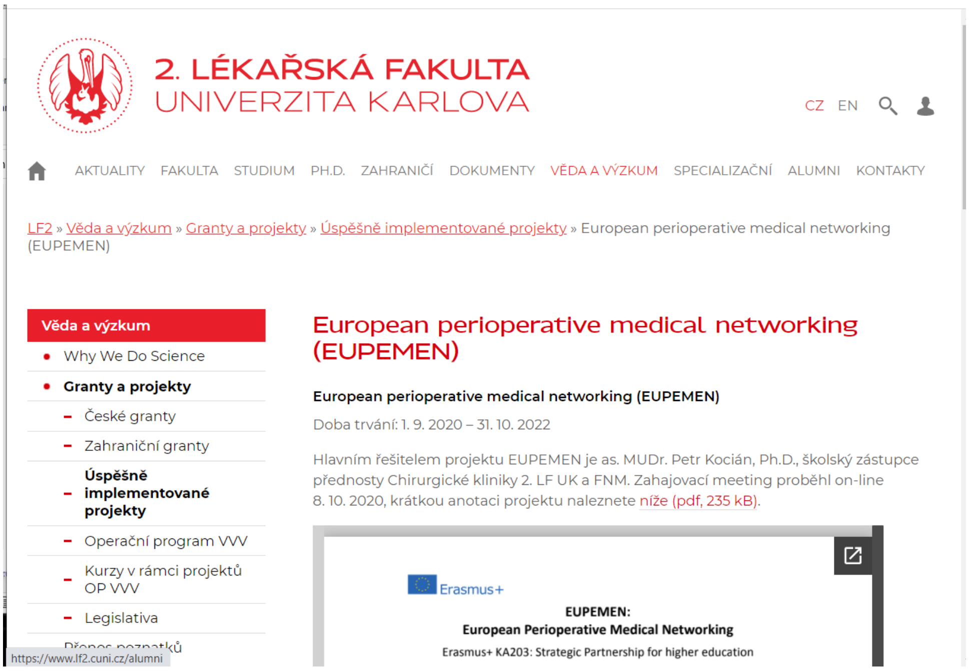

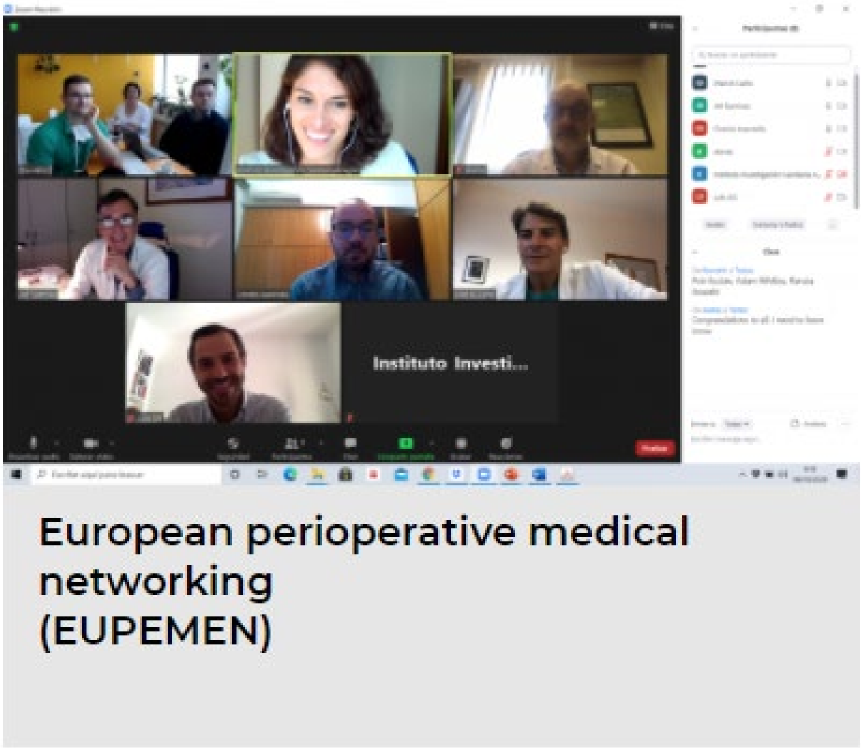

https://www.vivirenelche.com/2021/04/20/elche-cirugia-del-hospital-general-universitario-de-elche-participa-en-un-proyecto-europeo-centrado-en-la-recuperacion-rapida-del-paciente-tras-una-cirugia-abdominal/

< VIVIRENELCHE.COM @ NOTICIAS PRENSA DIGITAL >

NOTICIAS DE PRENSA ELCHE Y COMARCA

INICIO | EDITORIAL | AGENDA | PRENSA | ADMINISTRACIÓN | EMPRESAS | EMPLEO | EDUCACIÓN | CULTURA | OCIO | SER[illegible]

NATURALEZA | DEPORTES | CIENCIA | TÉCNOLOGÍA | AUDIOVISUALES | SUCESOS | COLUMNA LECTOR | CONTRATACION | CONTA[illegible]

ELCHE | Cirugía del Hospital General Universitario de Elche participa en un proyecto europeo centrado en la recuperación rápida del paciente tras una cirugía abdominal

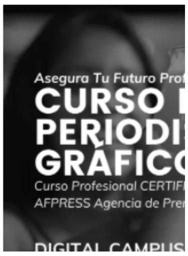

DEBATES: 5 Socios Con Perfil Sanitario Y Universitario De 4 Países Diferentes · Cirugía · Cirugía Abdominal · Disminuir Las Complicaciones Y La Mortalidad · Dr. Antonio Arroyo - Jefe Servicio Cirugía HGUE Y Catedrático UMH · Dr. Luis Sánchez Guillén - Cirujano General HGUE Y Profesor Asociado UMH · Elaboración De Un Proyecto Educativo · Elche · Elx · Evidencia Por Procedimiento · Hospital General Universitario Elche - HGUE · Hospitales · Hospitales De Europa · Procedimiento Perioperatorio · Procedimiento Quirúrgico · Proyecto EUPEMEN · Proyecto Europeo · Recuperación Rápida Del Paciente · Reducir El Estrés Secundario · Rehabilitación Quirúrgica Multimodal · Sociedades Científicas · Universidad Miguel Hernández (UMH) · Universidades

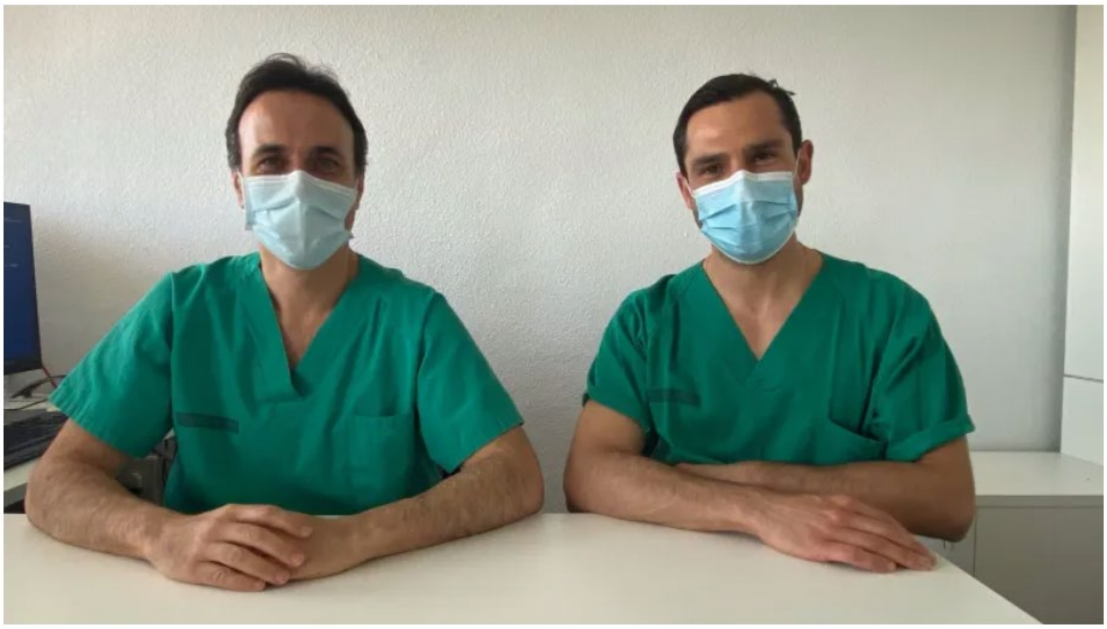

PUBLICADO POR: VIVIR EN ELCHE 20 ABRIL, 2021

- El hospital es uno de los 5 participantes en el estudio junto con otros hospitales, universidades y sociedades científicas de Europa

- El objetivo es implementar la rehabilitación quirúrgica multimodal en los hospitales de Europa

---

El Servicio de Cirugía General del Hospital General Universitario de Elche y la Universidad Miguel Hernández (UMH) participan en un proyecto europeo centrado en la recuperación rápida de pacientes tras una cirugía abdominal, mediante lo que se conoce como rehabilitación quirúrgica multimodal.

Este tipo de rehabilitación implica la aplicación de una serie de medidas y estrategias de procedimiento perioperatorio dirigidas a los pacientes que van a ser sometidos a un procedimiento quirúrgico, con el objetivo de reducir el estrés secundario causado por la intervención quirúrgica y así lograr una mejor recuperación del paciente y disminuir las complicaciones y la mortalidad.

Para implementar la rehabilitación quirúrgica multimodal en los hospitales de Europa, 5 socios con perfil sanitario y universitario de 4 países diferentes han creado el **proyecto EUPEMEN** cuyo objetivo es elaborar una guía con los protocolos a ser puestos en marcha por los especialistas multidisciplinarios involucrados.

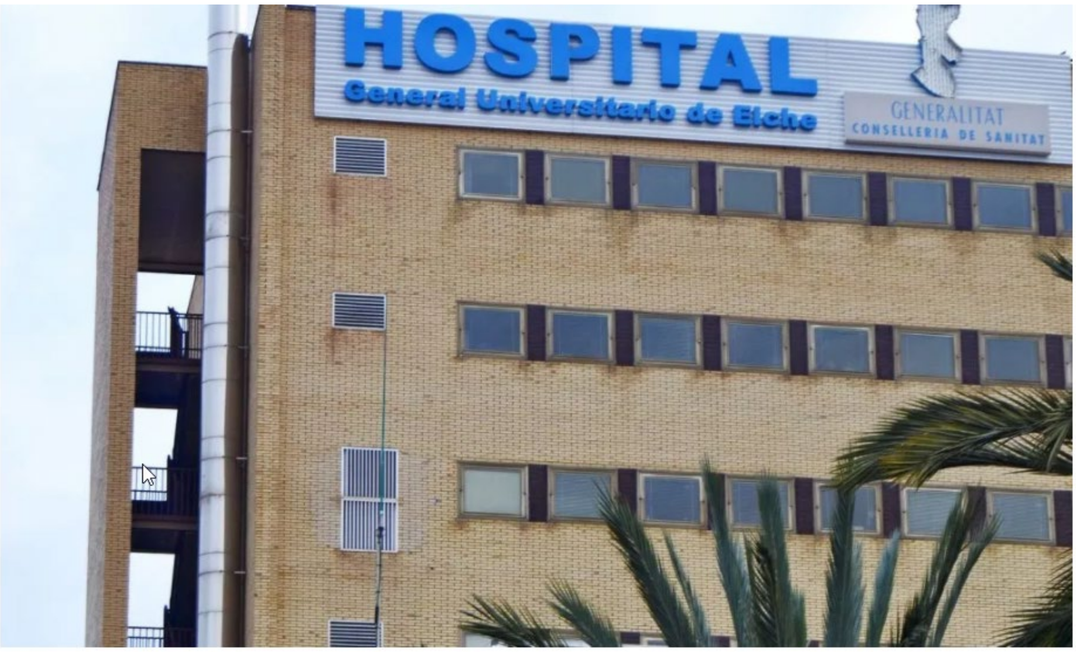

Contexto del proyecto

El **Dr. Antonio Arroyo, jefe de Servicio de Cirugía del Hospital General Universitario de Elche y Catedrático de la UMH** explica que **"La cirugía está indicada para curar o paliar numerosas dolencias físicas; sin embargo, representa un estrés importante que a menudo conduce a efectos adversos no relacionados con los objetivos del tratamiento. Estos efectos adversos tienen profundos impactos negativos en la capacidad de realizar actividades de la vida diaria, lo que posteriormente afecta la calidad de vida".**

"Además, la creciente demanda de cirugía mayor en pacientes de alto riesgo requiere nuevas mejoras que deben incluir un enfoque específico basado en la evidencia por procedimiento y ahí es donde se enmarca este proyecto".

Es necesario destacar que el Hospital General Universitario de Elche forma parte de los miembros fundadores del GERM (Grupo Español de Rehabilitación Multimodal) y fue pionero en la implantación de la rehabilitación multimodal en España, siendo actualmente uno de los Centros de Excelencia y formadores a nivel nacional.

Objetivos generales del proyecto

Entre los objetivos del proyecto destaca el intentar cubrir los vacíos de habilidades existentes de un conocimiento transversal en las actividades perioperatorias con el fin de poner en práctica los protocolos que se desarrollarán para varias cirugías abdominales y así aprender a actuar para los diferentes profesionales involucrados en los procedimientos.

En ese sentido, el **Dr. Luis Sánchez Guillén, cirujano general del hospital ilicitano, profesor asociado de la UMH, miembro de grupos de formación en rehabilitación multimodal en España y participante en varios ensayos clínicos financiados por agencias nacionales sobre el tema**, concreta que **"El proyecto aborda aspectos clínicos relacionados con el manejo perioperatorio de los pacientes, en un intento por homogeneizar este cuidado y mejorar la rehabilitación o recuperación postoperatoria del procedimiento, reduciendo las complicaciones quirúrgicas y mejorando la calidad de vida percibida de estos pacientes"**.

"Para ello se elaborarán unos protocolos cuyo principal objetivo es proporcionar a los profesionales unas recomendaciones basadas en el conocimiento científico y en el consenso de las diferentes sociedades científicas implicadas para implementar y evaluar programas de recuperación mejorada en cirugía abdominal", mantiene.

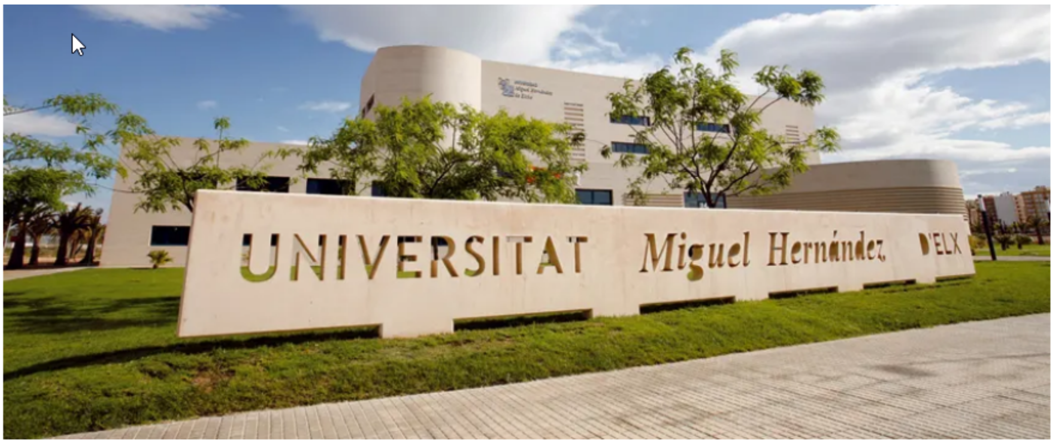

Formación

EUPEMEN intenta enseñar nuevas habilidades para que sean puestas en práctica en todos los profesionales involucrados en las acciones perioperatorias.

Así, el proyecto preparará diferentes materiales didácticos enfocados a la estrecha colaboración de todos los especialistas que participan en el proceso como cirujanos, anestesistas, enfermeras, nutricionistas, estomaterapeutas, fisioterapeutas, rehabilitadores, digestólogos, radioterapeutas, oncólogos y patólogos, así como de centros de salud, gestores y pacientes y familiares, dando un enfoque transdisciplinario para lograr el objetivo del proyecto y obtener el impacto esperado en la mejora de los pacientes.

Asimismo se desarrollará un sistema de aprendizaje que incluya un curso en línea que implique la enseñanza de los profesores con el fin de estar preparados para enseñar a los profesionales multidisciplinares nuevas habilidades para mejorar la recuperación de los pacientes intervenidos. Los asistentes obtendrán un certificado y se evaluará la correcta implementación de las medidas a nivel hospitalario, donde se ponderará el impacto.

Además, periódicamente se evaluará la implantación de los protocolos en los hospitales, con el objetivo de comprobar el éxito de las medidas y comprobar que todas ellas se han implantado correctamente. Las auditorías serán realizadas por un grupo de expertos que se definirá en el proyecto. Se medirán diferentes indicadores con el fin de evaluar la efectividad de la implementación de los protocolos tanto en el grado de implementación y cumplimiento de las recomendaciones por un lado, como en las mejoras en la recuperación del paciente y reducción de la estadía en los hospitales (con la reducción de costos). para los hospitales incluidos) por el otro.

Objetivos específicos del proyecto

Entre los objetivos específicos del proyecto destacan:

- La elaboración de un proyecto educativo (que incluirá un modelo didáctico de los profesores),

- La implantación en un número importante de hospitales europeos de los protocolos de rehabilitación quirúrgica multimodal basados en evidencias de forma homogénea y estandarizada.

- La recolección de datos sobre estancia hospitalaria, morbilidad y mortalidad de pacientes de European Surgical, que una vez analizados, serán de relevante interés para conocer mejor el riesgo quirúrgico de un paciente individual, y así prevenir complicaciones perioperatorias.

Por su parte, el Dr. Arroyo explica que **"En definitiva se trata de un proyecto innovador que modifica la práctica clínica haciéndola más segura, proponiendo el trabajo en equipo y unidades multidisciplinares, promoviendo el talento y la excelencia clínica buscando la mejor calidad asistencial. Es un proyecto diseñado para superar las barreras de resistencia al cambio y fomentar la investigación en medicina perioperatoria que genere un importante incentivo para las nuevas generaciones de profesionales que deben afrontar los retos de la medicina y la práctica clínica del futuro"**.

https://www.puntocomunica.com/temas/proyecto-eupemen/

COMUNITAT VALENCIANA, SALUD Y BIENESTAR

Cirugía del Hospital General Universitario de Elche participa en un proyecto europeo centrado en la recuperación rápida del paciente tras una cirugía abdominal

22 abril, 2021

- **El hospital es uno de los 5 participantes en el estudio junto con otros hospitales, universidades y sociedades científicas de Europa**
- **El objetivo es implementar la rehabilitación quirúrgica multimodal en los hospitales de Europa**

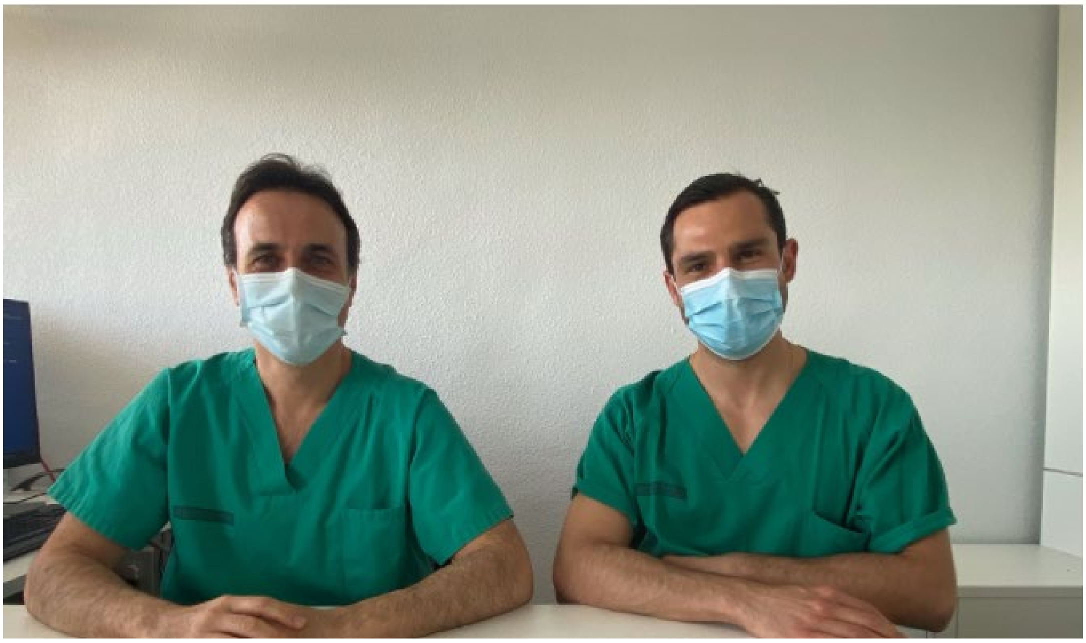

Elche, 2/04/2021

El **Servicio de Cirugía General** del **Hospital General Universitario de Elche** y la **Universidad Miguel Hernández** (UMH) participan en un **proyecto europeo** centrado en la **recuperación rápida de pacientes tras una cirugía abdominal**, mediante lo que se conoce como rehabilitación quirúrgica multimodal.

Este tipo de rehabilitación implica la aplicación de una serie de medidas y estrategias de procedimiento perioperatorio dirigidas a los pacientes que van a ser sometidos a un procedimiento quirúrgico, con el objetivo de reducir el estrés secundario causado por la intervención quirúrgica y así lograr una mejor recuperación del paciente y disminuir las complicaciones y la mortalidad.

Para implementar la rehabilitación quirúrgica multimodal en los hospitales de Europa, 5 socios con perfil sanitario y universitario de 4 países diferentes han creado el **proyecto EUPEMEN** cuyo objetivo es elaborar una guía con los protocolos a ser puestos en marcha por los especialistas multidisciplinarios involucrados.

Contexto del proyecto

El **Dr. Antonio Arroyo**, jefe de Servicio de Cirugía del Hospital General Universitario de Elche y Catedrático de la UMH explica que "La cirugía está indicada para curar o paliar numerosas dolencias físicas; sin embargo, representa un estrés importante que a menudo conduce a efectos adversos no relacionados con los objetivos del tratamiento. Estos efectos adversos tienen profundos impactos negativos en la capacidad de realizar actividades de la vida diaria, lo que posteriormente afecta la calidad de vida".

"Además, la creciente demanda de cirugía mayor en pacientes de alto riesgo requiere nuevas mejoras que deben incluir un enfoque específico basado en la evidencia por procedimiento y ahí es donde se enmarca este proyecto".

Es necesario destacar que el Hospital General Universitario de Elche forma parte de los miembros fundadores del GERM (Grupo Español de Rehabilitación Multimodal) y fue pionero en la implantación de la rehabilitación multimodal en España, siendo actualmente uno de los Centros de Excelencia y formadores a nivel nacional.

Objetivos generales del proyecto

Entre los objetivos del proyecto destaca el intentar cubrir los vacíos de habilidades existentes de un conocimiento transversal en las actividades perioperatorias con el fin de poner en práctica los protocolos que se desarrollarán para varias cirugías abdominales y así aprender a actuar para los diferentes profesionales involucrados en los procedimientos.

En ese sentido, el **Dr. Luis Sánchez Guillén**, cirujano general del hospital ilicitano, profesor asociado de la UMH, miembro de grupos de formación en rehabilitación multimodal en España y participante en varios ensayos clínicos financiados por agencias nacionales sobre el tema, concreta que "El proyecto aborda aspectos clínicos relacionados con el manejo perioperatorio de los pacientes, en un intento por homogeneizar este cuidado y mejorar la rehabilitación o recuperación postoperatoria del procedimiento, reduciendo las complicaciones quirúrgicas y mejorando la calidad de vida percibida de estos pacientes".

"Para ello se elaborarán unos protocolos cuyo principal objetivo es proporcionar a los profesionales unas recomendaciones basadas en el conocimiento científico y en el consenso de las diferentes sociedades científicas implicadas para implementar y evaluar programas de recuperación mejorada en cirugía abdominal", mantiene.

Formación

EUPEMEN intenta enseñar nuevas habilidades para que sean puestas en práctica en todos los profesionales involucrados en las acciones perioperatorias.

Así, el proyecto preparará diferentes materiales didácticos enfocados a la estrecha colaboración de todos los especialistas que participan en el proceso como cirujanos, anestesistas, enfermeras, nutricionistas, estomaterapeutas, fisioterapeutas, rehabilitadores, digestólogos, radioterapeutas, oncólogos y patólogos, así como de centros de salud, gestores y pacientes y familiares, dando un enfoque transdisciplinario para lograr el objetivo del proyecto y obtener el impacto esperado en la mejora de los pacientes.

Asímismo se desarrollará un sistema de aprendizaje que incluya un curso en línea que implique la enseñanza de los profesores con el fin de estar preparados para enseñar a los profesionales multidisciplinares nuevas habilidades para mejorar la recuperación de los pacientes intervenidos. Los asistentes obtendrán un certificado y se evaluará la correcta implementación de las medidas a nivel hospitalario, donde se ponderará el impacto.

Además, periódicamente se evaluará la implantación de los protocolos en los hospitales, con el objetivo de comprobar el éxito de las medidas y comprobar que todas ellas se han implantado correctamente. Las auditorías serán realizadas por un grupo de expertos que se definirá en el proyecto. Se medirán diferentes indicadores con el fin de evaluar la efectividad de la implementación de los protocolos tanto en el grado de implementación y cumplimiento de las recomendaciones por un lado, como en las mejoras en la recuperación del paciente y reducción de la estadía en los hospitales (con la reducción de costos). para los hospitales incluidos) por el otro.

Objetivos específicos del proyecto

Entre los objetivos específicos del proyecto destacan:

- La elaboración de un proyecto educativo (que incluirá un modelo didáctico de los profesores)
- La implantación en un número importante de hospitales europeos de los protocolos de rehabilitación quirúrgica multimodal basados en evidencias de forma homogénea y estandarizada
- La recolección de datos sobre estancia hospitalaria, morbilidad y mortalidad de pacientes de European Surgical, que una vez analizados, serán de relevante interés para conocer mejor el riesgo quirúrgico de un paciente individual, y así prevenir complicaciones perioperatorias

Por su parte, el Dr. Arroyo explica que "En definitiva se trata de un proyecto innovador que modifica la práctica clínica haciéndola más segura, proponiendo el trabajo en equipo y unidades multidisciplinares, promoviendo el talento y la excelencia clínica buscando la mejor calidad asistencial. Es un proyecto diseñado para superar las barreras de resistencia al cambio y fomentar la investigación en medicina perioperatoria que genere un importante incentivo para las nuevas generaciones de profesionales que deben afrontar los retos de la medicina y la práctica clínica del futuro".

Multiplier event - Italy

https://www.ausl.fe.it/novita/comunicati/erasmus-per-formare-i-professionisti-del-post-operatorio-azienda-unita-sanitaria-locale-e-universita-di-ferrara-insieme-nel-programma-eupenem-finanziato-dallunione-europea

https://www.ausl.fe.it/novita/comunicati/eupemen-erasmus-per-formare-i-professionisti-del-post-operatorio

https://www.unife.it/it/eventi/2022/ottobre/seminario-eras

https://www.estense.com/?p=989817

https://www.ausl.fe.it/novita/comunicati/eupemen-erasmus-per-formare-i-professionisti-del-post-operatorio-ampia-partecipazione-al-seminario-al-delta

“Γ. ΠΑΠΑΝΙΚΟΛΑΟΥ”
ΓΕΝΙΚΟ ΝΟΣΟΚΟΜΕΙΟ ΘΕΣΣΑΛΟΝΙΚΗΣ

ΕΛΛΗΝΙΚΗ ΔΗΜΟΚΡΑΤΙΑ

3η ΥΓΕΙΟΝΟΜΙΚΗ ΠΕΡΙΦΕΡΕΙΑ ΜΑΚΕΔΟΝΙΑΣ
ΓΡΑΦΕΙΟ ΔΙΟΙΚΗΤΗ
Τηλ. 2313 307109

Εξοχή 12.9.2022
Αρ. πρωτ. 16142

ΔΕΛΤΙΟ ΤΥΠΟΥ

1ο Σεμινάριο Περιεγχειρητικής Φροντίδας – Βελτιστοποίησης της Μετεγχειρητικής Ανάρρωσης στο πλαίσιο του ευρωπαϊκού προγράμματος «EUPEMEN: European Perioperative Medical Networking» που υλοποιείται στην Δ΄Χειρουργική Κλινική ΑΠΘ του Γενικού Νοσοκομείου «Γεώργιος Παπανικολάου» στις 25 Σεπτεμβρίου 2022.

Στο πλαίσιο του σεμιναρίου θα παρουσιαστεί η πλατφόρμα εξ αποστάσεως μάθησης και η πλατφόρμα καταγραφής που αναπτύχθηκε μέσα από το πρόγραμμα **«EUPEMEN: European Perioperative Medical Networking».** Όλοι οι συμμετέχοντες θα λάβουν δωρεάν ηλεκτρονικά αντίγραφα του εγχειριδίου Εντατικοποίησης της Ανάρρωσης για τη Βέλτιστη Φροντίδα ασθενών στην Χειρουργική Ενήλικων, των εξιδεικευμένων πρωτοκόλλων και του Οδηγού χειρουργικής παχέος εντέρου.
Στο σεμινάριο θα αναλυθούν οι βασικές αρχές της Εντατικοποίησης της Ανάρρωσης για τη Βέλτιστη Φροντίδα στην Χειρουργική Ενήλικων, η πολυπαραγοντική προσέγγιση της με τη συμμετοχή χειρουργών, αναισθησιολόγων και νοσηλευτών και τα εξειδικευμένα πρωτοκόλλα που αναπτύχθηκαν και αφορούν τη χειρουργική του οισοφάγου, του στομάχου, τη βαριατρική χειρουργική, τη χειρουργική του παχέος εντέρου, του ήπατος και την επείγουσα χειρουργική.

Η πραγματοποίηση του σεμιναρίου θα γίνει με φυσική παρουσία την Κυριακή 25 Σεπτεμβρίου 2022 στο Κέντρο Διάδοσης Ερευνητικών Αποτελεσμάτων (ΚΕ.Δ.Ε.Α) του Αριστοτελείου Πανεπιστημίου Θεσσαλονίκης
Ώρα έναρξης 9π.μ.
Οι εγγραφές ξεκινούν στις 8.30π.μ.
Η εγγραφή στο Σεμινάριο είναι ΔΩΡΕΑΝ.

Η διοργάνωση του 1ου Σεμιναρίου Περιεγχειρητικής Φροντίδας – Βελτιστοποίησης της Μετεγχειρητικής Ανάρρωσης στο πλαίσιο του πανευρωπαϊκού προγράμματος «EUPEMEN: European Perioperative Medical Networking» πραγματοποιείται σε συνεργασία με την Δ΄ Χειρουργική Κλινική του Αριστοτελείου Πανεπιστημίου Θεσσαλονίκης του Γενικού Νοσοκομείου Θεσσαλονίκης «Γεώργιος Παπανικολάου» με χρηματοδότηση από το πρόγραμμα “ERASMUS+” της Ευρωπαϊκής Ένωσης

FW: ΠΡΟΣΚΛΗΣΗ - 1ο Σεμινάριο Περιεγχειρητικής Φροντίδας – Βελτιστοποίησης της Μετεγχειρητικής Ανάρρωσης, 25 Σεπτεμβρίου 2022 - ΚΕΔΕΑ - Θεσσαλονίκη

announce-med-faculty@lists.auth.gr <announce-med-faculty@lists.auth.gr>
Tue 9/20/2022 10:45 AM
To: Κατάλογος προπτυχιακών φοιτητών ΕΠΠΣ <announce-medgr-ugrad@lists.auth.gr>;Κατάλογος Εκπαιδευτικού Προσωπικού <announce-med-faculty@lists.auth.gr>

2 attachments (662 KB)
EUPEMEN Agenda_.pdf; Συνημμένο χωρίς τίτλο 00087.pdf;

"Προς το εκπαιδευτικό προσωπικό και τους φοιτητές και φοιτήτριες του Τμήματος Ιατρικής ΑΠΘ

Ο Καθηγητής Σταμάτης Αγγελόπολος, Διευθυντής της Δ΄ Χειρουργικής Κλινικής Α.Π.Θ., σας προσκαλεί στο 1ο Σεμινάριο Περιεγχειρητικής Φροντίδας – Βελτιστοποίησης της Μετεγχειρητικής Ανάρρωσης στο πλαίσιο του ευρωπαϊκού προγράμματος «EUPEMEN: European Perioperative Medical Networking» που υλοποιείται από την Δ΄Χειρουργική Κλινική ΑΠΘ του Γενικού Νοσοκομείου «Γεώργιος Παπανικολάου» στις 25 Σεπτεμβρίου 2022.

Στο πλαίσιο του σεμιναρίου θα παρουσιαστεί η πλατφόρμα εξ αποστάσεως μάθησης και η πλατφόρμα καταγραφής που αναπτύχθηκε μέσα από το πρόγραμμα «EUPEMEN: European Perioperative Medical Networking». Όλοι οι συμμετέχοντες θα λάβουν δωρεάν ηλεκτρονικά αντίγραφα του εγχειριδίου Εντατικοποίησης της Ανάρρωσης για τη Βέλτιστη Φροντίδα ασθενών στην Χειρουργική Ενήλικων, των εξιδεικευμένων πρωτοκόλλων και του Οδηγού χειρουργικής παχέος εντέρου.

Στο σεμινάριο θα αναλυθούν οι βασικές αρχές της Εντατικοποίησης της Ανάρρωσης για τη Βέλτιστη Φροντίδα στην Χειρουργική Ενήλικων, η πολυπαραγοντική προσέγγιση της με τη συμμετοχή χειρουργών, αναισθησιολόγων και νοσηλευτών και τα εξειδικευμένα πρωτοκόλλα που αναπτύχθηκαν και αφορούν τη χειρουργική του οισοφάγου, του στομάχου, τη βαριατρική χειρουργική, τη χειρουργική του παχέος εντέρου, του ήπατος και την επείγουσα χειρουργική.

Η πραγματοποίηση του σεμιναρίου θα γίνει με φυσική παρουσία την Κυριακή 25 Σεπτεμβρίου 2022 στο Κέντρο Διάδοσης Ερευνητικών Αποτελεσμάτων (ΚΕ.Δ.Ε.Α) του Αριστοτελείου Πανεπιστημίου Θεσσαλονίκης

Ώρα έναρξης 9π.μ.

Οι εγγραφές ξεκινούν στις 8.30π.μ.

Η εγγραφή στο Σεμινάριο είναι ΔΩΡΕΑΝ.

Η διοργάνωση του 1ου Σεμιναρίου Περιεγχειρητικής Φροντίδας – Βελτιστοποίησης της Μετεγχειρητικής Ανάρρωσης στο πλαίσιο του πανευρωπαϊκού προγράμματος «EUPEMEN: European Perioperative Medical Networking» πραγματοποιείται σε συνεργασία με την Δ΄ Χειρουργική Κλινική του Αριστοτελείου Πανεπιστημίου Θεσσαλονίκης του Γενικού Νοσοκομείου Θεσσαλονίκης «Γεώργιος Παπανικολάου» με χρηματοδότηση από το πρόγραμμα "ERASMUS+" της Ευρωπαϊκής Ένωσης"

Με εκτίμηση,
Από τη Γραμματεία του Τμήματος Ιατρικής Α.Π.Θ.
Τηλέφωνο Επικοινωνίας: 2310.999.900
E-mail: info@med.auth.gr

Διευκρίνιση ηλεκτρονικού ταχυδρομείου
Οι πληροφορίες που συμπεριλαμβάνονται σε αυτό το μήνυμα είναι εμπιστευτικές και η χρήση τους επιτρέπεται μόνον από τον αναφερόμενο παραλήπτη. Εάν έχετε λάβει το παρόν μήνυμα από λάθος και δεν είστε ο προοριζόμενος παραλήπτης, σας ενημερώνουμε ότι αποκάλυψη, αναπαραγωγή, διανομή ή οποιασδήποτε άλλης μορφής χρήση των περιεχομένων του παρόντος μηνύματος απαγορεύεται. Επίσης παρακαλείσθε να αποστείλετε το αρχικό μήνυμα στην διεύθυνση του αποστολέα, καθώς και στη συνέχεια να διαγράψετε το μήνυμα από το σύστημά σας.
Η επικοινωνία μέσω Internet δεν είναι ασφαλής και επομένως το ΑΠΘ δεν φέρει ευθύνη για οποιαδήποτε θετική ή αποθετική ζημιά που προκλήθηκε από την χρήση του παρόντος ή των συνημμένων του λόγω ιών που έχουν περάσει σε αυτά.

Σας Ευχαριστούμε,
Αριστοτέλειο Πανεπιστήμιο Θεσσαλονίκης

1ο Σεμινάριο Περιεγχειρητικής Φροντίδας Δ΄Χειρουργική Κλινική ΑΠΘ-πρόγραμμα EUPEMEN

Papanikolaou-Library <paplib@gmail.com>
Mon 9/12/2022 2:15 PM

Εξοχή 12.9.2022
Αρ. πρωτ. 16142

ΔΕΛΤΙΟ ΤΥΠΟΥ

1ο Σεμινάριο Περιεγχειρητικής Φροντίδας – Βελτιστοποίησης της Μετεγχειρητικής Ανάρρωσης στο πλαίσιο του ευρωπαϊκού προγράμματος «EUPEMEN: European Perioperative Medical Networking» που υλοποιείται στην Δ΄Χειρουργική Κλινική ΑΠΘ του Γενικού Νοσοκομείου «Γεώργιος Παπανικολάου» στις 25 Σεπτεμβρίου 2022.

Στο πλαίσιο του σεμιναρίου θα παρουσιαστεί η πλατφόρμα εξ αποστάσεως μάθησης και η πλατφόρμα καταγραφής που αναπτύχθηκε μέσα από το πρόγραμμα **«EUPEMEN: European Perioperative Medical Networking».** Όλοι οι συμμετέχοντες θα λάβουν δωρεάν ηλεκτρονικά αντίγραφα του εγχειριδίου Εντατικοποίησης της Ανάρρωσης για τη Βέλτιστη Φροντίδα ασθενών στην Χειρουργική Ενήλικων, των εξιδεικευμένων πρωτοκόλλων και του Οδηγού χειρουργικής παχέος εντέρου.
Στο σεμινάριο θα αναλυθούν οι βασικές αρχές της Εντατικοποίησης της Ανάρρωσης για τη Βέλτιστη Φροντίδα στην Χειρουργική Ενήλικων, η πολυπαραγοντική προσέγγιση της με τη συμμετοχή χειρουργών, αναισθησιολόγων και νοσηλευτών και τα εξειδικευμένα πρωτοκόλλα που αναπτύχθηκαν και αφορούν τη χειρουργική του οισοφάγου, του στομάχου, τη βαριατρική χειρουργική, τη χειρουργική του παχέος εντέρου, του ήπατος και την επείγουσα χειρουργική.

Η πραγματοποίηση του σεμιναρίου θα γίνει με φυσική παρουσία την Κυριακή 25 Σεπτεμβρίου 2022 στο Κέντρο Διάδοσης Ερευνητικών Αποτελεσμάτων (ΚΕ.Δ.Ε.Α) του Αριστοτελείου Πανεπιστημίου Θεσσαλονίκης
Ώρα έναρξης 9π.μ.
Οι εγγραφές ξεκινούν στις 8.30π.μ.
Η εγγραφή στο Σεμινάριο είναι ΔΩΡΕΑΝ.

Η διοργάνωση του 1ου Σεμιναρίου Περιεγχειρητικής Φροντίδας – Βελτιστοποίησης της Μετεγχειρητικής Ανάρρωσης στο πλαίσιο του πανευρωπαϊκού προγράμματος «EUPEMEN: European Perioperative Medical Networking» πραγματοποιείται σε συνεργασία με την Δ΄ Χειρουργική Κλινική του Αριστοτελείου Πανεπιστημίου Θεσσαλονίκης του Γενικού Νοσοκομείου Θεσσαλονίκης «Γεώργιος Παπανικολάου» με χρηματοδότηση από το πρόγραμμα "ERASMUS+" της Ευρωπαϊκής Ένωσης

--
Βαλεντίνη Παπαγεωργίου
Ιατρική Βιβλιοθήκη
ΓΝ "Γ. Παπανικολάου"
Εξοχή Θεσσαλονίκη 57010
Τ.: 2313 307 134

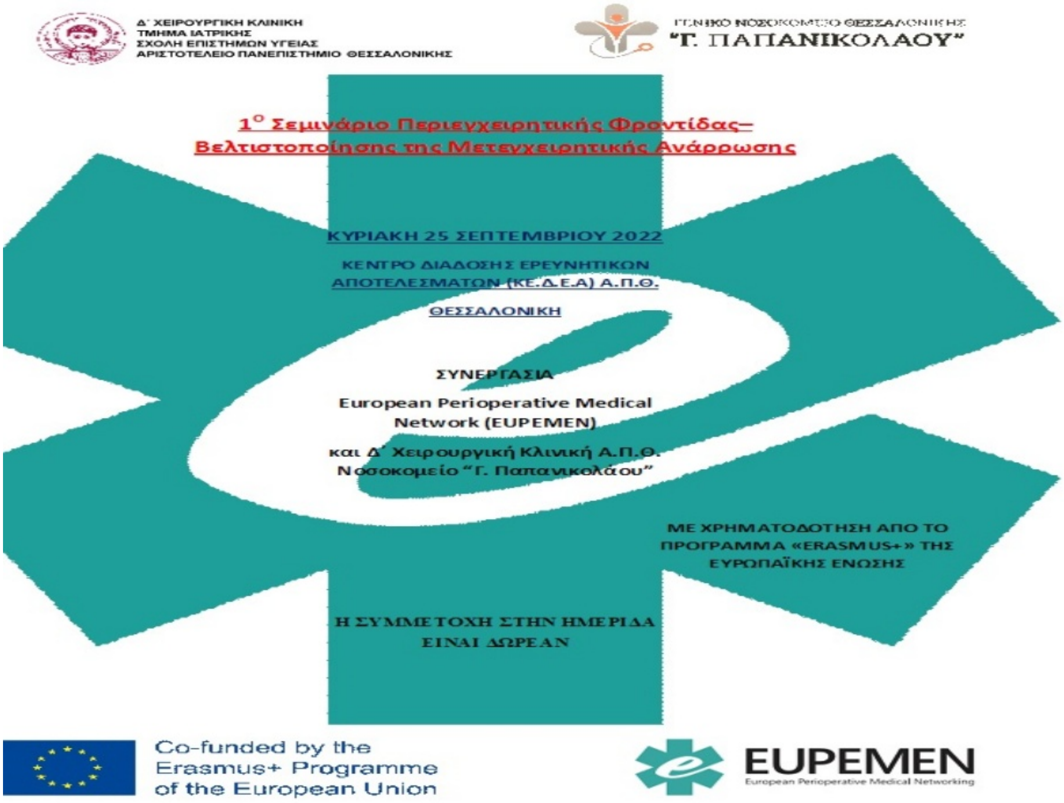

14:45 | 13/09/2022

1ο Σεμινάριο Περιεγχειρητικής Φροντίδας – Βελτιστοποίησης της Μετεγχειρητικής Ανάρρωσης στο πλαίσιο του ευρωπαϊκού προγράμματος «EUPEMEN: European Perioperative Medical Networking» που υλοποιείται στην Δ΄Χειρουργική Κλινική ΑΠΘ του Γενικού Νοσοκομείου «Γεώργιος Παπανικολάου» στις 25 Σεπτεμβρίου 2022.

Στο πλαίσιο του σεμιναρίου θα παρουσιαστεί η πλατφόρμα εξ αποστάσεως μάθησης και η πλατφόρμα καταγραφής που αναπτύχθηκε μέσα από το πρόγραμμα «EUPEMEN: European Perioperative Medical Networking». Όλοι οι συμμετέχοντες θα λάβουν δωρεάν ηλεκτρονικά αντίγραφα του εγχειριδίου Εντατικοποίησης της Ανάρρωσης για τη Βέλτιστη Φροντίδα ασθενών στην Χειρουργική Ενήλικων, των εξιδεικευμένων πρωτοκόλλων και του Οδηγού χειρουργικής παχέος εντέρου.

Στο σεμινάριο θα αναλυθούν οι βασικές αρχές της Εντατικοποίησης της Ανάρρωσης για τη Βέλτιστη Φροντίδα στην Χειρουργική Ενήλικων, η πολυπαραγοντική προσέγγιση της με τη συμμετοχή χειρουργών, αναισθησιολόγων και νοσηλευτών και τα εξειδικευμένα πρωτοκόλλα που αναπτύχθηκαν και αφορούν τη χειρουργική του οισοφάγου, του στομάχου, τη βαριατρική χειρουργική, τη χειρουργική του παχέος εντέρου, του ήπατος και την επείγουσα χειρουργική.

Η πραγματοποίηση του σεμιναρίου θα γίνει με φυσική παρουσία την Κυριακή 25 Σεπτεμβρίου 2022 στο Κέντρο Διάδοσης Ερευνητικών Αποτελεσμάτων (ΚΕ.Δ.Ε.Α) του Αριστοτελείου Πανεπιστημίου Θεσσαλονίκης

Ώρα έναρξης 9π.μ.

Οι εγγραφές ξεκινούν στις 8.30π.μ.

Η εγγραφή στο Σεμινάριο είναι ΔΩΡΕΑΝ.

Η διοργάνωση του 1ου Σεμιναρίου Περιεγχειρητικής Φροντίδας – Βελτιστοποίησης της Μετεγχειρητικής Ανάρρωσης στο πλαίσιο του πανευρωπαϊκού προγράμματος «EUPEMEN: European Perioperative Medical Networking» πραγματοποιείται σε συνεργασία με την Δ΄ Χειρουργική Κλινική του Αριστοτελείου Πανεπιστημίου Θεσσαλονίκης του Γενικού Νοσοκομείου Θεσσαλονίκης «Γεώργιος Παπανικολάου» με χρηματοδότηση από το πρόγραμμα "ERASMUS+" της Ευρωπαϊκής Ένωσης.

ΔΙΑΒΑΣΤΕ ΕΠΙΣΗΣ:

Βασιλακόπουλος: Γιατί ξεκίνησαν χωρίς μέτρα τα σχολεία

Χαλκιδική: Απρόσμενος επισκέπτης στην παραλία της Μεταμόρφωσης! (ΦΩΤΟ)

Οι Γερμανοί του PERMIRA και η περίπτωση της… Γιαννιώτικης "Best Secret"

Δείτε τις ειδήσεις από την Ελλάδα και όλο τον κόσμο στο GRTimes.gr

Ακολουθήστε το GRTimes στο Google News και ενημερωθείτε πριν από όλους

Απόρρητο

Πρόγραμμα EUPEMEN: 1ο Σεμινάριο Περιεγχειρητικής Φροντίδας Δ΄ Χειρουργική Κλινική ΑΠΘ

GRTiMES 1 εβδομάδα πριν

ΝΕΑ ΤΩΡΑ GrTimes | 1ο Σεμινάριο Περιεγχειρητικής Φροντίδας – Βελτιστοποίησης της Μετεγχειρητικής Ανάρρωσης στο πλαίσιο του ευρωπαϊκού προγράμματος «EUPEMEN: European Perioperative Medical Networking» που υλοποιείται στην Δ΄Χειρουργική Κλινική ΑΠΘ του Γενικού Νοσοκομείου «Γεώργιος Παπανικολάου» στις 25 Σεπτεμβρίου 2022. Στο πλαίσιο του σεμιναρίου θα παρουσιαστεί η πλατφόρμα εξ αποστάσεως μάθησης και η πλατφόρμα καταγραφής που αναπτύχθηκε μέσα από το πρόγραμμα «EUPEMEN: European Perioperative Medical Networking». Όλοι οι συμμετέχοντες θα λάβουν δωρεάν ηλεκτρονικά αντίγραφα του εγχειριδίου Εντατικοποίησης της Ανάρρωσης για τη Βέλτιστη Φροντίδα ασθενών στην Χειρουργική Ενήλικων, των εξειδικευμένων πρωτοκόλλων και του Οδηγού χειρουργικής παχέος εντέρου. Στο σεμινάριο θα αναλυθούν οι βασικές &alpha...

Διαβάστε ολόκληρο το άρθρο

Αρχική › Θεσσαλονίκη
› Πρόγραμμα EUPEMEN: 1ο Σεμινάριο Περιεγχειρητικής Φροντίδας Δ΄ Χειρουργική Κλινική ΑΠΘ

Σχετικά Άρθρα

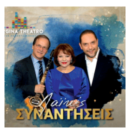

Δ. Ωραιοκάστρου: Εθελοντές αιμοδότες έστειλαν νέο μήνυμα ζωή...
2 ώρες πριν

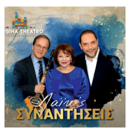

Θεσσαλονίκη: Δράση στον Λευκό Πύργο για την Παγκόσμια Μέρα τ...
2 ώρες πριν

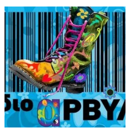

Ράδιο Αρβύλα: Έκαναν απρεπή σχόλια για καρκινοπαθή... και το π...
5 ώρες πριν

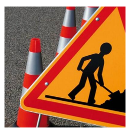

Θεσσαλονίκη: Εργασίες από Δευτέρα στη γέφυρα «Μυτιληνάκια» ε...
5 ώρες πριν

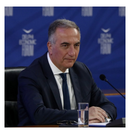

Στ. Καλαφάτης: Παρά την ενεργειακή κρίση, προχωράμε για μια ...
5 ώρες πριν

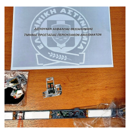

Θεσσαλονίκη: «Στη φάκα» μέλη εγκληματικής οργάνωσης – Παγίδε...
6 ώρες πριν

ΡΟΗ ΕΙΔΗΣΕΩΝ

Έρχεται η 4η σεζόν του You σε δύο μέρη - Πότε είναι η πρεμιέρα στο Netflix
2 λεπτά πριν

Τα βλέμματα στραμμένα στην Ιταλία: Ιχνηλατώντας τις προθέσεις της Μελόνι – Η ακροδεξιά ατζέντα, η «ασάφεια» και οι απόπειρες συμβιβασμού με την Ευρώπη
5 λεπτά πριν

The Crown: Κυκλοφόρησε το πρώτο τρέιλερ της 5ης σεζόν με πριγκίπισσα

ΤΑΣΕΙΣ

1. Χρήστος Πο...
2. Ελενα τοπα...
3. Λιμνη κρεμ...
4. Δημητρα αλ...
5. Αγια ευφρο...
6. Αριστείδης ...
7. Συνταξεισ ο...
8. ΚΩΣΤΑΣ Μ...
9. Εκλογεσ ιτα...
10. Παγιδευμέ...

ΔΗΜΟΦΙΛΗ ΑΡΘΡΑ

Απόπειρα αυτοκτονίας της Δήμητρας Αλεξανδράκη σε ξενοδοχείο της Θεσσαλονίκης
8 ώρες πριν

Μεγάλη φωτιά στη Βιομηχανική Περιοχή Βόλου
23 ώρες πριν

Αναστάτωση από πληροφορίες για εξαφάνιση 8χρονου στο Μαρούσι
17 ώρες πριν

Θλίψη στην ελληνική ιστιοπλοΐα: Πέθανε ο Τέλης Αδαμόπουλος
23 ώρες πριν

Δήμητρα Αλεξανδράκη: Έξαλλη στον αέρα του «Χαμογέλα και πάλι» με

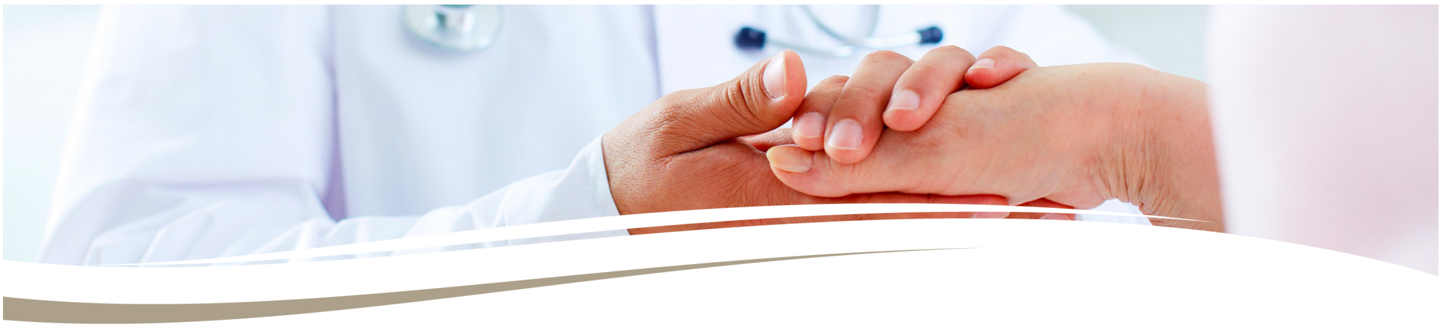

1ο Σεμινάριο Περιεγχειρητικής Φροντίδας Δ΄Χειρουργική Κλινική ΑΠΘ-πρόγραμμα EUPEMEN

Επισυναπτόμενα αρχεία

Αρχείο	Μέγεθος αρχείου
EUPEMEN Agenda_25.9.2022 (https://gpapanikolaou.gr/download-attachment/16131)	295 KB
ΔΕΛΤΙΟ ΤΥΠΟΥ σεμιναριο_Πρόγραμμα EUPEMEN_Δ Χειρουργικη ΑΠΘ (https://gpapanikolaou.gr/download-attachment/16132)	49 KB
σεμιναριο EUPEMEN 25.9.2022_Δ Χειρουργικη ΑΠΘ (https://gpapanikolaou.gr/download-attachment/16133)	122 KB

PREVIOUS ARTICLE
ΠΡΟΣΚΛΗΣΗ ΕΚΔΗΛΩΣΗΣ ΕΝΔΙΑΦΕΡΟΝΤΟΣ ΕΚΠΑΙΔΕΥΤΩΝ ΔΙΕΚ Γ.Ν.Θ. ΠΑΠΑΝΙΚΟΛΑΟΥ (https://gpapanikolaou.gr/anakoinoseis/2022-09-19/16114/%CF%80%CF%81%CE%BF%CF%83%CE%BA%CE%BB%CE%B7%CF%83%CE%B7-%CE%B5%CE%BA%CE%B4%CE%B7%CE%BB%CF%89%CF%83%CE%B7%CF%83-%CE%B5%CE%BD%CE%B4%CE%B9%CE%B1%CF%86%CE%B5%CF%81%CE%BF%CE%BD%CF%84%CE%BF%CF%83-%CE%B5-2/)

NEXT ARTICLE
Διενέργεια Διεθνή ανοιχτού διαγωνισμού προμήθειας αναλώσιμου υλικού ακτινολογικών εργαστηρίων (ΒΤ33/2022) (https://gpapanikolaou.gr/diagonismoi/2022-09-22/16142/%CE%B4%CE%B9%CE%B5%CE%BD%CE%AD%CF%81%CE%B3%CE%B5%CE%B9%CE%B1-%CE%B4%CE%B9%CE%B5%CE%B8%CE%BD%CE%AE-%CE%B1%CE%BD%CE%BF%CE%B9%CF%87%CF%84%CE%BF%CF%8D-%CE%B4%CE%B9%CE%B1%CE%B3%CF%89%CE%BD%CE%B9%CF%83%CE%BC/)

Copyright © 2022 Γ.Ν.Θ. "Γ. ΠΑΠΑΝΙΚΟΛΑΟΥ". All rights reserved.

1ο Σεμινάριο Περιεγχειρητικής Φροντίδας– Βελτιστοποίησης της Μετεγχειρητικής Ανάρρωσης

Προσθήκη σε ημερολόγιο

25 Σεπτεμβρίου 2022

https://isth.gr/wp-content/uploads/2022/09/Eupemen-Δ΄-Χειρουργική-Κλινική-Α.Π.Θ-ΠΡΟΓΡΑΜΜΑ.pdf

ΛΕΠΤΟΜΕΡΕΙΕΣ

Ημερομηνία:

25 Σεπτεμβρίου 2022

© 2021 Ιατρικός Σύλλογος Θεσσαλονίκης

Development and Hosting by **Pointer**

facebook Log In

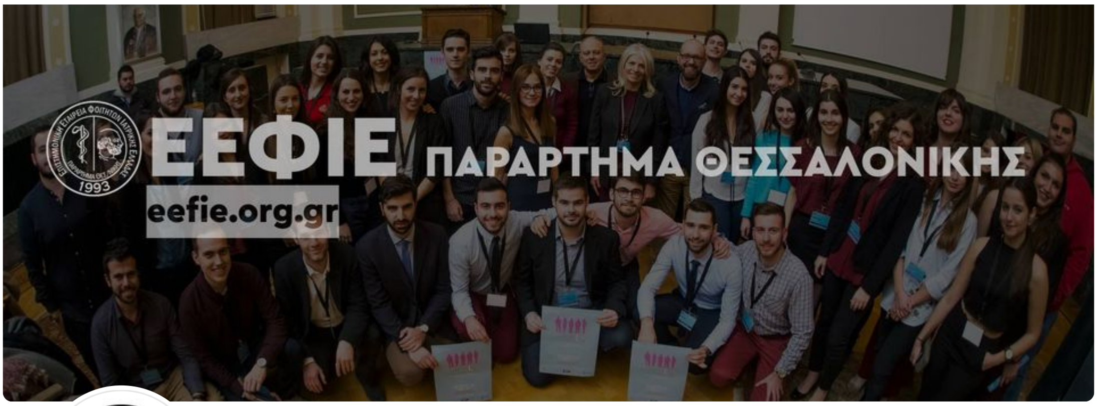

Επιστημονική Εταιρεία Φοιτητών Ιατρικής Ελλάδας - Παράρτημα Θεσσαλονίκης

@eefiethess · Nonprofit organization

Contact us

eefie.org.gr

About

See all

Επιστημονική Εταιρεία Φοιτητών Ιατρικής Ελλάδας - Παράρτημα Θε... Contact us ···

Thessaloníki, Greece

Καλωσήρθατε στην επίσημη σελίδα της ΕΕΦΙΕ Θεσσαλονίκης!
ΣΚΕΨΟΥ, ΔΡΑΣΕ, ΔΗΜΙΟΥΡΓΗΣΕ !

Η Επιστημονική Εταιρεία Φοιτητών Ιατρικής Ελλάδας (Ε.Ε.Φ.Ι.Ε.) ιδρύθηκε τον Σεπτέμβριο του 1993 και αποτελεί Αστική Εταιρεία μη κερδοσκοπική, μη κυβερ... See more

5,096 people like this

5,233 people follow this

455 people checked in here

http://www.eefie.org.gr/

231 099 9030

eefiethessalonikis@gmail.com

Always open

Nonprofit Organization

Photos

See all

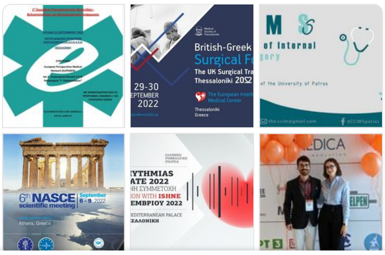

Επιστημονική Εταιρεία Φοιτητών Ιατρικής Ελλάδας - Παράρτημα Θεσσαλονίκης

"Του Σεμινάριο Περιεγχειρητικής Φροντίδας – Βελτιστοποίησης της Μετεγχειρητικής Ανάρρωσης" που διοργανώνεται από Δ' Χειρουργική Κλινική του ΑΠΘ

📍Το σεμινάριο θα πραγματοποιηθεί με φυσική παρουσία, την Κυριακή 25 Σεπτεμβρίου 2022, στο Κέντρο Διάδοσης Ερευνητικών Αποτελεσμάτων (ΚΕ.Δ.Ε.Α) του Αριστοτελείου Πανεπιστημίου Θεσσαλονίκης. ... **See more**

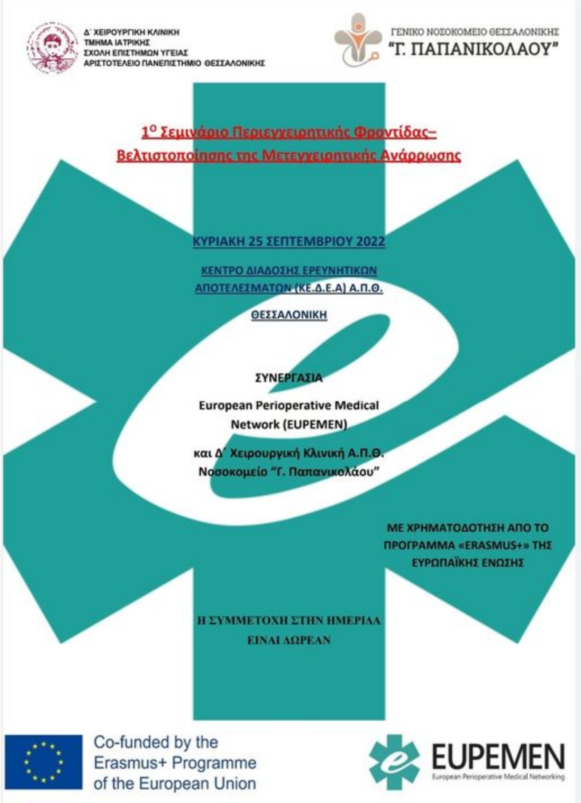

10 · 1 Comment

Like · Comment · Share

See more of Επιστημονική Εταιρεία Φοιτητών Ιατρικής Ελλάδας - Παράρτημα Θεσσαλονίκης on Facebo...

Log In or Create new account

NOTA DE PRENSA

Un proyecto europeo coordinado por el IIS Aragón desarrolla siete protocolos perioperatorios para reducir el estrés del paciente y las complicaciones derivadas de la cirugía

- El programa Eupemen, en el que han participado cinco socios de España, Grecia, Italia y República Checa, busca mejorar tanto la calidad de vida del enfermo como la eficiencia del sistema sanitario

- El objetivo es formar a los profesionales implicados en estos procedimientos y crear una red de docentes que enseñen y auditen su aplicación en los hospitales, para lo que se ha creado una plataforma de aprendizaje con vídeos explicativos en cinco idiomas

Zaragoza, XX de noviembre de 2022.- El proyecto Eupemen (*European perioperative medical networking*), financiado por la Unión Europea a través del programa Erasmus+ y coordinado por el Instituto de Investigación Sanitaria Aragón (IIS Aragón), ha concluido recientemente después de 26 meses de trabajo, durante los que ha desarrollado siete protocolos perioperatorios dirigidos a pacientes que van a ser sometidos a un procedimiento quirúrgico con el objetivo de reducir el estrés secundario causado por la intervención, logrando una mejor recuperación del enfermo y disminuyendo las complicaciones derivadas de la cirugía y la mortalidad. Se trata de una serie de medidas y estrategias basadas en la evidencia científica y enfocadas en la recuperación postoperatoria que abarcan todos los aspectos de la atención al paciente y requieren un manejo multidisciplinario, con la participación de diversos especialistas, para ser implementados en los hospitales con la finalidad de mejorar tanto la calidad de vida del enfermo como la eficiencia del sistema sanitario.

El proyecto, que ha involucrado a cinco socios de España, Italia, República Checa y Grecia, pretende formar a profesionales multidisciplinares directamente implicados en el procedimiento perioperatorio, además de crear una red de docentes con capacidad para enseñar a estos grupos en los hospitales y auditar la correcta aplicación de los protocolos de manera estandarizada y homogénea. Para ello, también se ha creado una plataforma de aprendizaje para la adquisición de los conocimientos necesarios a través de guías y vídeos explicativos que, como los protocolos, está disponible en cinco idiomas: español, inglés, italiano, griego y checo.

Estos resultados se han presentado en una jornada de conclusión celebrada en la Facultad de Medicina sobre 'Protocolos de Recuperación Intensificada en cirugía del adulto. Su implantación en España y su exportación a Europa', en la que han participado el investigador principal del proyecto, José Manuel Ramírez, líder del Grupo de cirugía y medicina perioperatoria del IIS Aragón; Javier Martínez Ubieto, investigador principal del Grupo de estudio de relajación muscular y bloqueo neuromuscular del IIS Aragón; y la doctora Ana María Pascual Bellosta.

"La cirugía está indicada para curar o paliar numerosas dolencias físicas. Sin embargo, representa un estrés importante que, a menudo, conduce a efectos adversos no relacionados con los objetivos del tratamiento y que tienen profundos impactos negativos en la capacidad de realizar actividades de la vida diaria, lo que posteriormente afecta la calidad de vida del paciente", explica Ramírez. "Por eso es importante aplicar programas de atención enfocados en la recuperación postoperatoria a todos los niveles", añade. A largo plazo, el proyecto Eupemen persigue disminuir los efectos secundarios después de la cirugía para los pacientes y, en consecuencia, lograr una mejoría más rápida; reducir la mortalidad tras las cirugías; y rebajar el tiempo de estancia hospitalaria, con el consiguiente ahorro económico para el sistema sanitario y el aumento de disponibilidad de camas para otros pacientes.

Acerca del Instituto de Investigación Sanitaria Aragón

El IIS Aragón es el Instituto de Investigación Sanitaria del complejo hospitalario formado por los Hospitales Docentes y Universitarios "Hospital Clínico Universitario Lozano Blesa" y "Hospital Universitario Miguel Servet" y la Atención Primaria de Salud. A este complejo hospitalario se le asocian a través de distintos instrumentos jurídicos, la Universidad de Zaragoza y el Instituto Aragonés de Ciencias de la Salud.

Los objetivos del IIS Aragón son aproximar la investigación básica y aplicada, clínica y de servicios sanitarios; crear un entorno investigador, asistencial y docente de calidad al que queden expuestos los profesionales sanitarios, los especialistas en formación y los alumnos de postgrado y grado, así como constituir el lugar idóneo para la captación de talento y la ubicación de las grandes instalaciones científico-tecnológicas.

Acerca de Erasmus+

Erasmus+ (2014-2020) es el programa integrado de la Unión Europea (UE) en los ámbitos de la educación y la formación, juventud y deporte, que ofrece oportunidades para todas las personas y en todos los sectores educativos (Educación Escolar, Formación Profesional, Educación Superior y Educación de Personas Adultas). En España, el programa Erasmus+ se gestiona por parte del Servicio Español para la Internacionalización de la Educación (SEPIE), que actúa como Agencia Nacional del programa en los ámbitos de la educación y la formación y que está adscrito al Ministerio de Universidades. El proyecto Eupemen se enmarca dentro de la Acción Clave 203 Asociaciones estratégicas, donde instituciones transnacionales vinculadas con la Educación Superior cooperan en el desarrollo de productos intelectuales innovadores que impliquen una mejora en los sistemas y estructuras de la Educación Superior.

NEWS:

https://www.iisaragon.es/un-proyecto-europeo-coordinado-por-el-iis-aragon-desarrolla-siete-protocolos-perioperatorios-para-reducir-el-estres-del-paciente-y-las-complicaciones-derivadas-de-la-cirugia/

https://www.heraldo.es/noticias/salud/2022/11/09/desarrollan-protocolos-contra-estres-causado-intervenciones-quirurgicas-1611229.html

https://www.ultimahora.es/noticias/comunidades/2022/11/09/1825563/proyecto-europeo-coordinado-por-iis-aragon-realiza-protocolos-perioperatorios-para-reducir-estres-del-paciente.html

https://arainfo.org/el-iis-aragon-ha-coordinado-el-protocolo-europeo-para-reducir-el-estres-y-las-complicaciones-del-paciente/

https://www.aragonhoy.es/sanidad/proyecto-europeo-coordinado-iis-aragon-desarrolla-siete-protocolos-perioperatorios-reducir-estres-paciente-complicaciones-89060

https://www.lavanguardia.com/local/aragon/20221109/8599866/proyecto-europeo-coordinado-iis-aragon-realiza-protocolos-perioperatorios-reducir-estres-paciente.html

https://www.heraldo.es/noticias/salud/2022/11/09/desarrollan-protocolos-contra-estres-causado-intervenciones-quirurgicas-1611229.html

http://www.gentedigital.es/zaragoza/noticia/3500332/un-proyecto-europeo-coordinado-por-el-iis-aragon-realiza-protocolos-perioperatorios-para-reducir-el-estres-del-paciente/

facebook

Log In

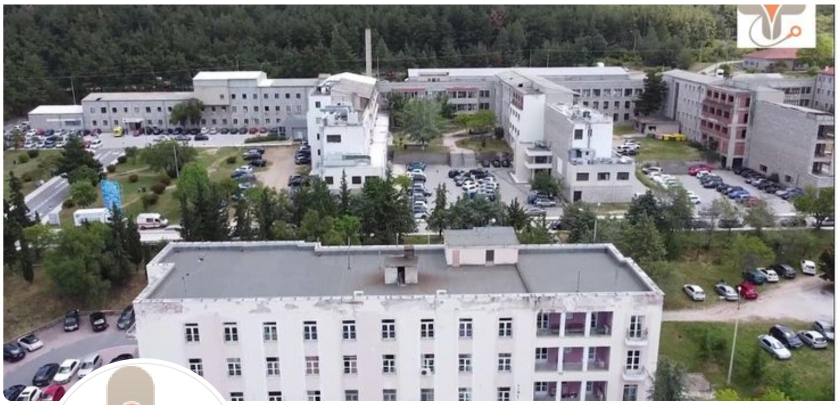

Γενικό Νοσοκομείο "Γεώργιος Παπανικολάου"

4K likes • 33 following

Γενικό Νοσοκομείο "Γεώργιος Παπανικολάου"
3d ·

"Διευρύνει τις υπηρεσίες του το Κέντρο Ημερήσιας Απασχόλησης Ατόμων με διαταραχές μνήμης"
https://www.amna.gr/.../Pagkosmia-Imera-Altschaimer...
#amna.gr #angelafotopoulou

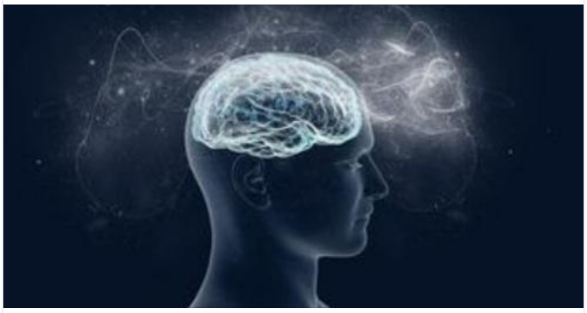

AMNA.GR

Παγκόσμια Ημέρα Αλτσχάιμερ-Διευρύνει τις υπηρεσίες του το Κέντρο Ημερήσιας Απασχόλησης Ατόμων με...

14

Like Comment Share

Γενικό Νοσοκομείο "Γεώργιος Παπανικολάου"
3d ·

"Ενα εξωτερικό ιατρείο μοναδικό στην Ελλάδα που σώζει ζωές στο νοσοκομείο "Γ. Παπανικολάου" #heopinion #EfiVatali
https://www.theopinion.gr/.../ena-exoteriko-iatreio.../...

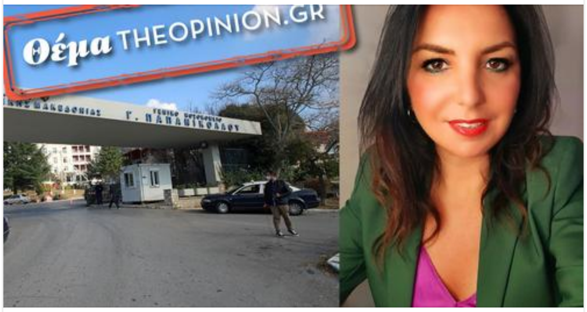

THEOPINION.GR

Ένα εξωτερικό ιατρείο, το μοναδικό στην Ελλάδα, που [illegible]

Connect with Γενικό Νοσοκομείο "Γεώργιος Παπανικολάου" on Facebook

Log In or Create new account

Υβριδικό συνέδριο με τίτλο «Διαχείριση των λοιμώξεων του αναπνευστικού συστήματος στην κλινική πράξη» διοργανώνεται από την Πνευμονολογική Κλινική του Αριστοτελείου Πανεπιστημίου Θεσσαλονίκης που λειτουργεί στο Γενικό Νοσοκομείο «Γεώργιος Παπανικολάου» στις 30 Σεπτεμβρίου 2022 – 1 Οκτωβρίου 2022 στο Κέντρο Διάδοσης Ερευνητικών Αποτελεσμάτων (ΚΕΔΕΑ) του ΑΠΘ.

Το συνέδριο αποτελεί μέρος της σειράς επιστημονικών εκδηλώσεων που διοργανώνει η Πνευμονολογική Κλινική ΑΠΘ στο πλαίσιο… **See more**

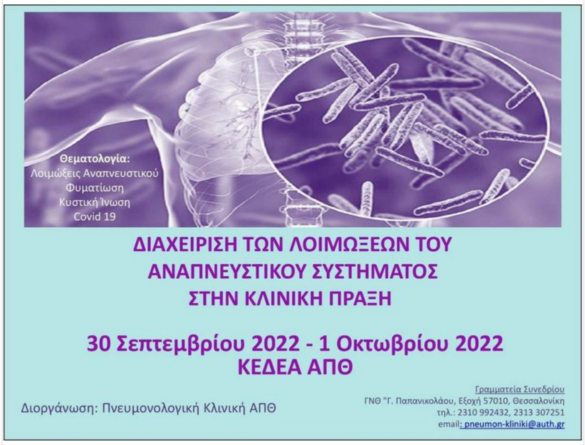

27 · 2 Shares

Like · Comment · Share

Most relevant

Γενικό Νοσοκομείο "Γεώργιος Παπανικολάου"
4d ·

Μια ξεχωριστή διάκριση για το Νοσοκομείο μας στο Διεθνές Συνέδριο της Ευρωπαϊκής Εταιρείας Ογκολογίας (European Society of medical oncology, ESMO 2022, https://www.esmo.org/) για την γιατρό του Γενικού Νοσοκομείου «Γεώργιος Παπανικολάου» κυρία Σοφία Λαμπάκη Sophia Lampaki, MD, PhD ιατρό πνευμονολόγο και ακαδημαϊκή υπότροφο στην Πνευμονολογική Κλινική ΑΠΘ.
Στο συνέδριο ESMO 2022 δημοσιεύθηκε ως ελεύθερη ανακοίνωση η εργασία της Πνευμονολόγου της Πνευμονολογικής Κλινικής ΑΠΘ, … **See more**

206 · 24 Comments · 7 Shares

Like · Comment · Share

Most relevant

Κατερινα Σακαλη
Συγχαρητήρια!!
3d

View 9 more comments

Γενικό Νοσοκομείο "Γεώργιος Παπανικολάου"
4d ·

21 Σεπτεμβρίου 2022: Παγκόσμια Ημέρα για τη νόσο Alzheimer

Για τη φετινή Παγκόσμια Ημέρα για τη νόσο Alzheimer νέα στοιχεία έρχονται να προστεθούν στην υπάρχουσα εικόνα που αφορούν τις επιπτώσεις της πανδημίας COVID 19 και τις νέες προκλήσεις στις οποίες καλείται να ανταποκριθεί το Κέντρο Ημερήσιας Απασχόλησης Ατόμων με διαταραχές μνήμης που ανήκει στο Ψυχιατρικό Νοσοκομείο Θεσσαλονίκης. Οι ιδιαίτερες συνθήκες της πανδημίας στις οποίες έπρεπε να προσαρμοστούν οι ηλικιωμένοι… **See more**

14 · 1 Share

Like · Comment · Share

Γενικό Νοσοκομείο "Γεώργιος Παπανικολάου"
September 12 at 9:06 PM ·

ΟΙ ΝΟΣΗΛΕΥΤΕΣ ΔΙΝΟΥΝ ΟΡΑΜΑ ΕΛΠΙΔΑΣ
Στο πλαίσιο της 1ης Πανελλαδικής Εθελοντικής Δράσης δωρεάς

Connect with Γενικό Νοσοκομείο "Γεώργιος Παπανικολάου" on Facebook

Log In or Create new account

facebook

Log In

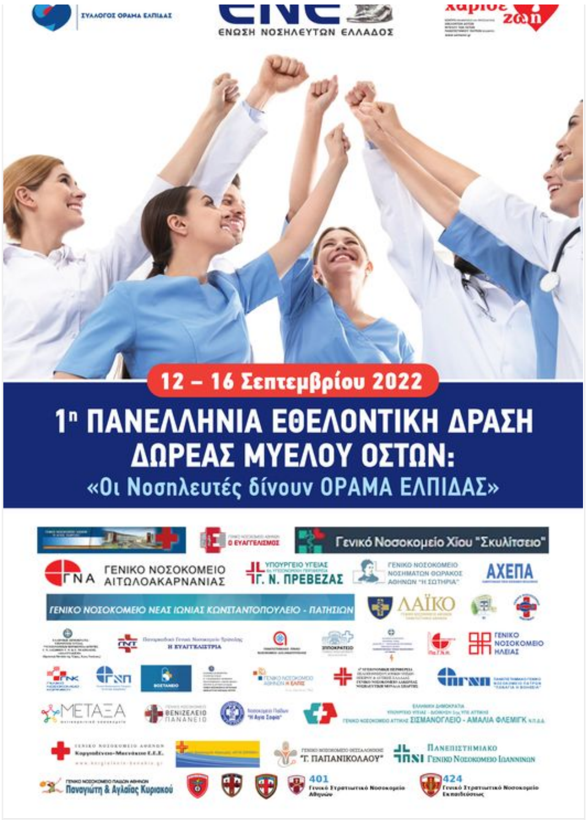

39

4 Shares

Like Comment Share

Γενικό Νοσοκομείο "Γεώργιος Παπανικολάου"

...

Intro

Page · Hospital

gpapanikolaou.gr

Always open

Rating · 3.4 (21 Reviews)

Photos

See all photos

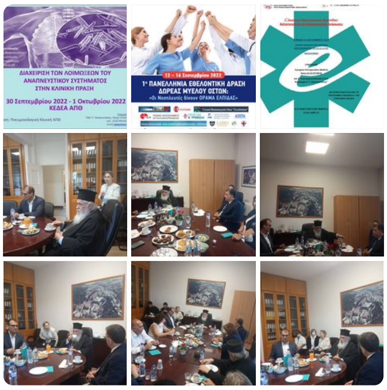

Information about Page Insights Data · Privacy · Terms · Advertising · Ad Choices · Cookies · More · Meta © 2022

Γενικό Νοσοκομείο "Γεώργιος Παπανικολάου"
September 12 at 2:07 PM ·

Το 1ο Σεμινάριο Περιεγχειρητικής Φροντίδας – Βελτιστοποίησης της Μετεγχειρητικής Ανάρρωσης στο πλαίσιο του ευρωπαϊκού προγράμματος «EUPEMEN: European Perioperative Medical Networking» που υλοποιείται στην Δ΄Χειρουργική Κλινική ΑΠΘ του Γενικού Νοσοκομείου «Γεώργιος Παπανικολάου» πραγματοποιείται στις 25 Σεπτεμβρίου 2022 στο ΚΕΔΕΑ.
Στο πλαίσιο του σεμιναρίου θα παρουσιαστεί η πλατφόρμα εξ αποστάσεως μάθησης και η πλατφόρμα καταγραφής που αναπτύχθηκε μέσα από το πρόγραμμα «EUPEM... **See more**

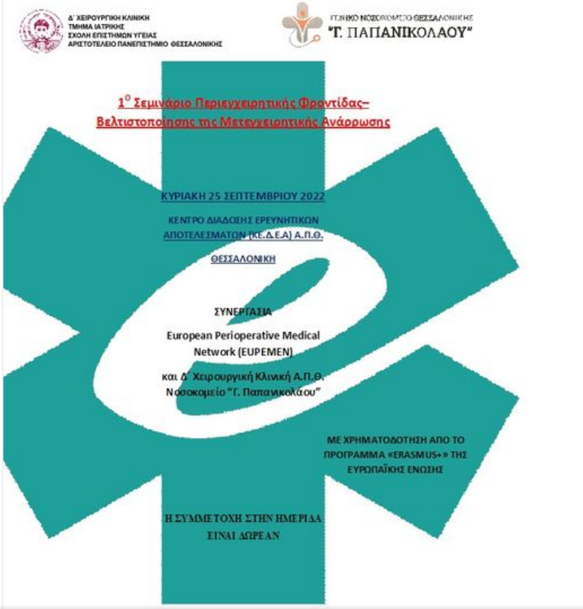

Connect with Γενικό Νοσοκομείο "Γεώργιος Παπανικολάου" on Facebook

Log In or Create new account

facebook

Log In

Γενικό Νοσοκομείο "Γεώργιος Παπανικολάου"

Διήμερη Προγραμματισμένη Αιμοδοσία του Συλλόγου Εθελοντών Αιμοδοτών του Δήμου Προποντίδας "Αλυσίδα Ζωής" στις 2 και 3 Οκτωβρίου από την Αιμοδοσία του Γενικού Νοσοκομείου "Γεώργιος Παπανικολάου"

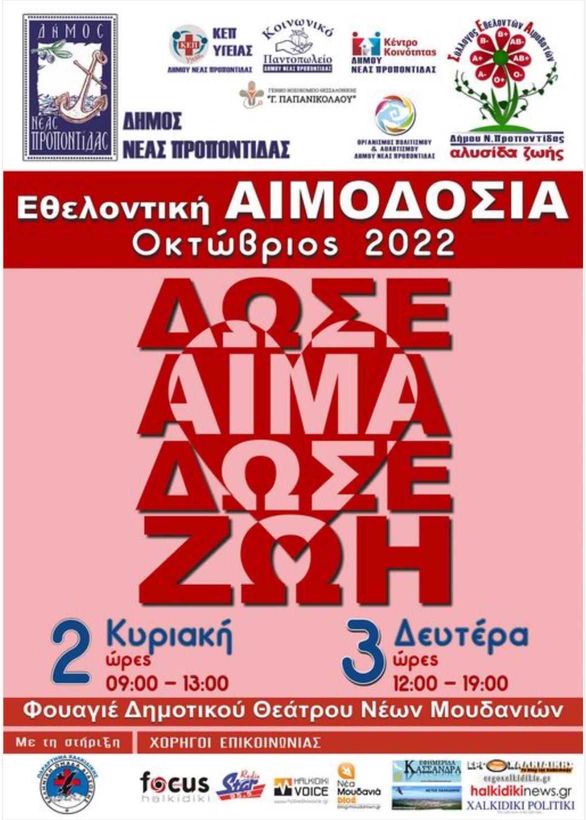

Δήμος Νέας Προποντίδας
September 9 at 8:26 PM ·

📍Διήμερη Προγραμματισμένη Αιμοδοσία του Συλλόγου Εθελοντών Αιμοδοτών του Δήμου μας "Αλυσίδα Ζωής"

✔️Κυριακή 2 Οκτωβρίου 2022
⏰Από 9.00 έως 13.00.... **See more**

16 · 1 Share

Like · Comment · Share

Γενικό Νοσοκομείο "Γεώργιος Παπανικολάου"
September 12 at 10:04 AM ·

Από την Αρχιερατική Θεία Λειτουργία στο Ιερό Παρεκκλήσιο του Νοσοκομείου Παπανικολάου όπου τιμάται το Γενέθλιο της Θεοτόκου και ιερούργησε ο Σεβασμιώτατος Μητροπολίτης Νεαπόλεως και Σταυρουπόλεως κ. Βαρνάβας την Πέμπτη 8 Σεπτεμβρίου 2022.

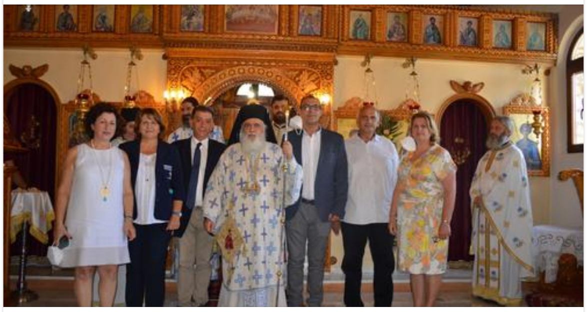

IMNST.GR

Αρχιερατική Θεία Λειτουργία στο Ιερό Παρεκκλήσιο του Νοσοκομείου Παπανικολάου - ΙΕΡΑ ΜΗΤΡΟΠΟΛΗ...

Connect with Γενικό Νοσοκομείο "Γεώργιος Παπανικολάου" on Facebook

Log In or Create new account

Most Relevant is selected, so some comments may have been filtered out.

Γενικό Νοσοκομείο "Γεώργιος Παπανικολάου"
September 8 at 4:07 PM ·

Με την ευκαιρία του εορτασμού του Ιερού Ναού του Νοσοκομείου «Γεώργιος Παπανικολάου» όπου τιμάται το «Γενέσιον της Θεοτόκου» στις 8 Σεπτεμβρίου, μας τίμησε με την παρουσία του ο Σεβασμιότατος Μητροπολίτης Νεαπόλεως και Σταυρουπόλεως κ. κ. Βαρνάβας για την τέλεση της Αρχιερατικής Θείας Λειτουργίας. Στη γόνιμη συζήτηση που ακολούθησε στην αίθουσα συνεδριάσεων στο Κτίριο της Διοίκησης παρουσία του Διοικητή του Νοσοκομείου, των Διευθυντών της Ιατρικής, Νοσηλευτικής, Διοικητικής Υ... **See more**

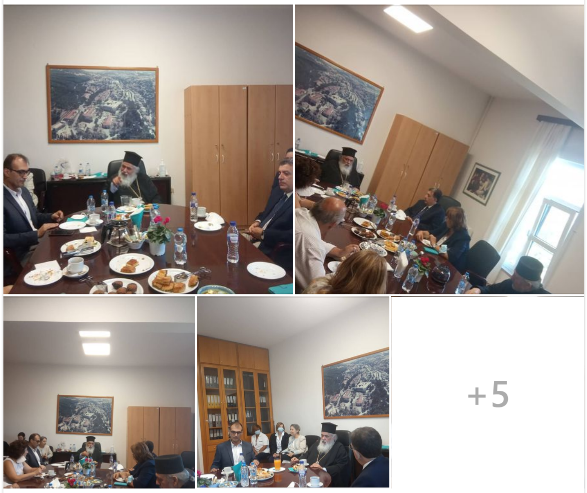

87 2 Shares

Like Comment Share

Most relevant

Γενικό Νοσοκομείο "Γεώργιος Παπανικολάου"
May 30 ·

«Έχεις τη φύση στο χέρι σου» στο Γενικό Νοσοκομείο «Γεώργιος Παπανικολάου» από την Χ.Α.Ν.Θ. και την εταιρεία TITAN την Τετάρτη 1 Ιουνίου 12μ.μ.-2μ.μ.

Για δεύτερη συνεχή χρονιά η εταιρεία TITAN σε συνδιοργάνωση με τη Χ.Α.Ν.Θ., είναι κοντά στο Γενικό Νοσοκομείο «Γεώργιος Παπανικολάου» και τους εργαζόμενους του με την έμπρακτη προσφορά τους και την ενεργό παρουσία τους. Με αφορμή την Παγκόσμια Ημέρα Περιβάλλοντος, Χ.Α.Ν.Θ. και TITAN ανανεώνουν το ραντεβού τους μαζί μας μέσα απ... **See more**

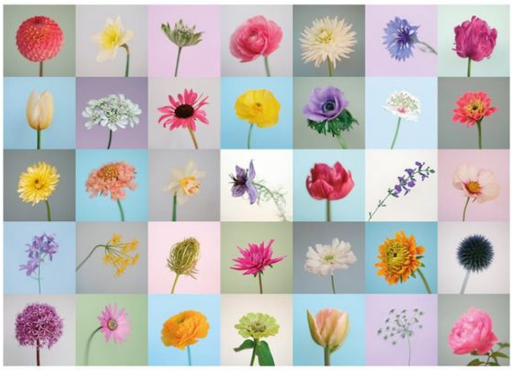

44 2 Shares

Like Comment Share

Most relevant

Most Relevant is selected, so some comments may have been filtered out.

Connect with Γενικό Νοσοκομείο "Γεώργιος Παπανικολάου" on Facebook

Log In or Create new account

Σεπτέμβριος 2022

Δε	Τρ	Τε	Πε	Πα	Σα	Κυ
29	30	31	1<br>15ο Πανελλήνιο Συνέδριο Καρδιομεταβολικών Παραγόντων Κινδύνου	2<br>1ο Ιπποκράτειο Ιατρικό Συνέδριο Πανεπιστημίου Θεσσαλίας<br>3rd Cadaveric Workshop on Thoracolumbar, Lumbar and …<br>9ο Πανελλήνιο Συνέδριο Υπερήχων στη Μαιευτική και Γυναικολογία<br>1ο Ιπποκράτειο Ιατρικό Συνέδριο Πανεπιστημίου Θεσσαλίας<br>Advanced Instability Course	3	4
5	6<br>6th NASCE/UEMS Scientific Meeting – New Frontiers in Open Innovation	7<br>COVID-19 Masterclass: …<br>8th Endoscopic Skull Base Dissection Course	8<br>διεθνές επιστημονικό συ…<br>5ο Πανελλήνιο Επιστημονικό Συνέδριο Μαιευτικής και Γυναικολογίας	9<br>58ο Ετήσιο Συνέδριο της Στοματολογικής Εταιρείας της Ελλάδος<br>Inaugural Forum: The edge of tomorrow – endoscopic car…<br>Επιστημονική Διημερίδα «Συνεργασία Ιατρικών Ειδικοτήτω…	10	11
12	13<br>Η πιθανότητα κακοποίη…<br>12:00 - 17:00<br>ΕΛΙΖΑ_Webinar: «Η πιθανότητα κακοποίησης – παραμέλησης στον παιδιατρικό θάνατο και στην κρίσιμη νόσο»	14	15<br>24ο Πανελλήνιο Θεματικό Συνέδριο Εντατική Θεραπεία & Επείγουσα Ιατρική<br>Advanced ACL Course<br>ΠΑΝΕΛΛΗΝΙΟ ΣΥΝΕΔΡΙΟ ΚΑΡΔΙΟΜΕΤΑΒΟΛΙΚΩΝ ΝΟΣΗΜΑΤΩΝ, ΠΑΧΥΣΑΡΚΙΑΣ ΚΑΙ ΔΙΑΒΗΤΗ<br>13:30<br>Advanced ACL Course	16<br>9ο Πανελλήνιο Συνέδριο Γυναικολογικής Ογκολογίας<br>πανελλήνιο συνέδριο Καρδιομεταβολικών Νοσημάτων, παχυσαρκίας και διαβήτη<br>Αθηροσκληρυντική καρδιαγγειακή νόσος: Σύγχρονοι προβ…	17	18
19	20	21<br>Εκδήλωση Κοινωνικής …<br>08:00 - 17:00<br>Β' Ορθοπαιδική Κλινική ΑΠΘ, 21/9/2022	22<br>What is New in Respiratory Medicine	23<br>16ο Πανελλήνιο Συνέδριο Επιληψίας<br>Διημερίδα ΕΛΛ.Α.ΝΑ. Το ταξίδι του ασθενή<br>2ο ΣΕΜΙΝΑΡΙΟ ΤΗΣ Β' …<br>11:30<br>Ημερίδα στα πλαίσια του προγράμματος HERO Training Program (Erasmus+)	24	25<br>7ο Πανελλήνιο Συνέδριο…<br>1ο Σεμινάριο Περιεγχειρ…<br>09:00<br>1ο Σεμινάριο Περιεγχειρητικής Φροντίδας Δ΄Χειρουργική Κλινική ΑΠΘ-πρόγραμμα EUPEMEN
26<br>7ο Πανελλήνιο Συνέδριο Παιδικής και Εφηβικής Γυναικολο…<br>09:00<br>Υποβοηθούμενη Αναπαραγωγή (IVF) και Αυτοάνοσα Ρευματικά Νοσήματα	27	28<br>18:30<br>«Άνοια & Οικογένεια – Πως συμπεριφερόμαστε στους ανθρώπους με Άνοια» (ΑΚΤΙΟΣ)	29<br>Διαγνωστικοί Αλγόριθμοι & Σύγχρονες Θεραπευτικές Προσεγγίσεις στη Παθολογία	30<br>ΕΤΗΣΙΟ ΜΕΤΕΚΠΑΙΔΕΥΤΙΚΟ ΣΕΜΙΝΑΡΙΟ Υγρών, Ηλεκτ…<br>Παιδιατρικές Ημέρες 2022: Η δυναμική της Νεφρολογίας κ…<br>Διαχείριση των λοιμώξεων του αναπνευστικού συστήματο…<br>SAVE THE DATE 1ο Συνέδριο Περιβαλλοντικής και Κλιματ…	1	2

14.00

Πλ. Αριστοτέλους 4, Θεσσαλονίκη 546 23

2310262300, 2310273755, 2310251960

grammatia@isth.gr

FAX: 2310278880

Πολιτική cookies

Υποβολή Ερωτήσεων στον Υπεύθυνο Προστασίας Δεδομένων του ΙΣΘ

Διαχείριση συγκατάθεσης

Χρήσιμοι Σύνδεσμοι

Επικοινωνία
Περιοδικό "Ιατρικά Θέματα"
Πιστοποιήσεις
Λίστα Ιατρών

Social Media

© 2021 Ιατρικός Σύλλογος Θεσσαλονίκης

Development and Hosting by **Pointer**

ΑΝΑΚΟΙΝΩΣΗ

05 ΣΕΠΤΕΜΒΡΙΟΣ 2022

Προέλευση Δ' Χειρουργική Κλινική

Ανάρτηση Γεώργιος Χαραλαμπίδης

Δελτίο τύπου Eupemen - Δ΄ Χειρουργική Κλινική Α.Π.Θ. - 1ο Σεμινάριο Περιεγχειρητικής Φροντίδας – Βελτιστοποίησης της Μετεγχειρητικής Ανάρρωσης

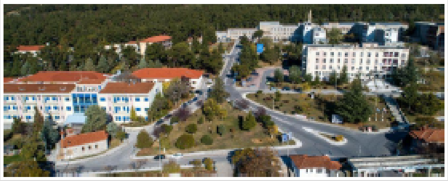

Παρακαλώ βρείτε συνημμένο το δελτίο τύπου που αφορά το 1ο Σεμινάριο Περιεγχειρητικής Φροντίδας – Βελτιστοποίησης της Μετεγχειρητικής Ανάρρωσης που διοργανώνει η Δ' Χειρουργική Κλινική καθώς και το πρόγραμμα της ημερίδας για να ανακοινωθεί στην σελίδα της ιατρικής σχολής

Επισυναπτόμενα αρχεία:

- eupemen agenda .pdf
- 1o seminario periegheiritikis.pdf

ΕΠΙΚΟΙΝΩΝΙΑ

Τμήμα Ιατρικής, Πανεπιστημιούπολη ΑΠΘ, Τ.Κ. 54124, Θεσσαλονίκη

Τηλ: 2310 999 900

Email: info@med.auth.gr

Το περιεχόμενο είναι διαθέσιμο κάτω από Creative Commons Attribution 4.0 International License (http://creativecommons.org/licenses/by/4.0/) .

(http://creativecommons.org/licenses/by/4.0/) - Powered by Drupal (http://drupal.org)

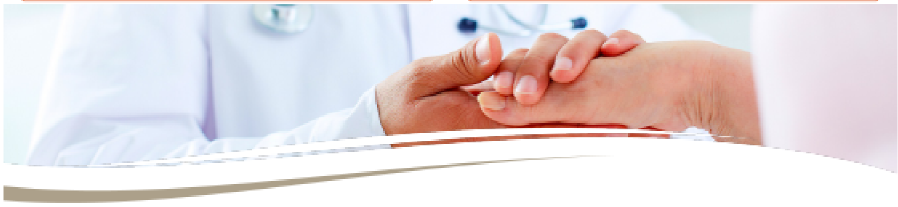

1ο Σεμινάριο Περιεγχειρητικής Φροντίδας Δ΄Χειρουργική Κλινική ΑΠΘ-πρόγραμμα EUPEMEN

Επισυναπτόμενα αρχεία

Αρχείο	Μέγεθος αρχείου
EUPEMEN Agenda_25.9.2022 (https://gpapanikolaou.gr/download-attachment/16131)	295 KB
ΔΕΛΤΙΟ ΤΥΠΟΥ σεμιναριο_Πρόγραμμα EUPEMEN_Δ Χειρουργικη ΑΠΘ (https://gpapanikolaou.gr/download-attachment/16132)	49 KB
σεμιναριο EUPEMEN 25.9.2022_Δ Χειρουργικη ΑΠΘ (https://gpapanikolaou.gr/download-attachment/16133)	122 KB

PREVIOUS ARTICLE
ΠΡΟΣΚΛΗΣΗ ΕΚΔΗΛΩΣΗΣ ΕΝΔΙΑΦΕΡΟΝΤΟΣ ΕΚΠΑΙΔΕΥΤΩΝ ΔΙΕΚ Γ.Ν.Θ. ΠΑΠΑΝΙΚΟΛΑΟΥ (https://gpapanikolaou.gr/anakoinoseis/2022-09-19/16114/%CF%80%CF%81%CE%BF%CF%83%CE%BA%CE%BB%CE%B7%CF%83%CE%B7-%CE%B5%CE%BA%CE%B4%CE%B7%CE%BB%CF%89%CF%83%CE%B7%CF%83-%CE%B5%CE%BD%CE%B4%CE%B9%CE%B1%CF%86%CE%B5%CF%81%CE%BF%CE%BD%CF%84%CE%BF%CF%83-%CE%B5-2/)

NEXT ARTICLE
Διενέργεια Διεθνή ανοικτού διαγωνισμού προμήθειας αναλώσιμου υλικού ακτινολογικών εργαστηρίων (ΒΤ33/2022) (https://gpapanikolaou.gr/diagonismoi/2022-09-22/16142/%CE%B4%CE%B9%CE%B5%CE%BD%CE%AD%CF%81%CE%B3%CE%B5%CE%B9%CE%B1-%CE%B4%CE%B9%CE%B5%CE%B8%CE%BD%CE%AE-%CE%B1%CE%BD%CE%BF%CE%B9%CF%87%CF%84%CE%BF%CF%8D-%CE%B4%CE%B9%CE%B1%CE%B3%CF%89%CE%BD%CE%B9%CF%83/)

Copyright © 2022 Γ.Ν.Θ. "Γ. ΠΑΠΑΝΙΚΟΛΑΟΥ". All rights reserved.

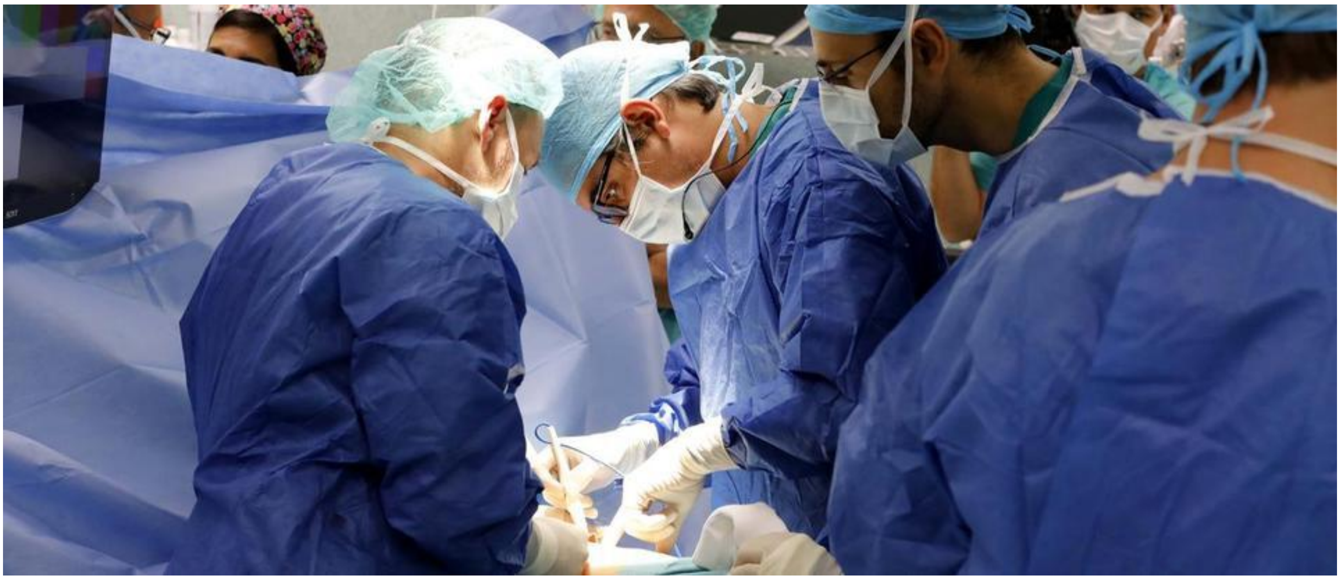

Operación de tórax, pionera en Europa, realizada en el hospital Miguel Servet. / JAIME GALINDO

SANIDAD

Protocolos 'made in Zaragoza' para que el enfermo «se involucre en la recuperación»

Controles de nutrición y movilidad mejoran al paciente tras una operación / Ya se implanta en los hospitales aragoneses y nace con vocación de exportarse a Europa

Eva García

zaragoza | 06·12·22 | 06:00

El manejo tradicional del perioperatorio «ha cambiado bastante» en los últimos años. Y Zaragoza ha sido pionera en su investigación ya que aquí se creó un grupo de trabajo entre el Instituto de Investigación Sanitaria Aragón y el Instituto de Ciencias de la Salud, que importó protocolos del norte de Europa, para proponer después «una guía de recomendaciones prácticas para el paciente quirúrgico, que pretende prepararlo» para que acuda en las mejores condiciones posibles a una

cirugía, que esta tenga lugar de forma positiva y lleve a cabo una «rehabilitación de la mejor forma posible», señala José Manuel Ramírez, investigador principal del Grupo de Cirugía y Medicina Perioperatoria del IIS Aragón.

RELACIONADAS

El personal del Servet clama por la "mala gestión" del Salud y pide sus días de descanso

Esta vía clínica de recuperación intensificada en cirugía abdominal (Rica), que apareció en 2015 y en 2020 se actualizó, se implementó en un principio en pacientes de cirugía de colon pero ahora ya casi en todas las operaciones de cadera, rodilla, hígado, urología, ginecología, traumatología, etc; además hay que tener en cuenta que «cada vez tenemos enfermos más frágiles y más añosos, que son los que más se benefician».

Estas medidas preoperatorias consisten en recomendar ejercicios que mejoren la capacidad funcional, aplicar unos controles y unos suplementos nutricionales para que «vaya en condiciones óptimas al quirófano» y ya allí medidas puntuales que en ocasiones son habituales como «utilizar volúmenes adecuados de fluido y no demasiados opioides», cuenta Ramírez. Y una vez en planta, se les aconseja que «no se encamen, que se movilicen y al día siguiente que coman».

Evidencia científica

En definitiva, estos protocolos buscan que el paciente «se involucre en la recuperación» . Al principio, señala el especialista, pueden estar reticentes pero «como se les explica lo que tienen que hacer» y las mejoras son claras, aceptan. Y es que «todo está basado en la evidencia científica» y refrendado por el Ministerio de Sanidad. La mejoría y la recuperación tras la operación «es más rápida» y el paciente está «menos tiempo en el hospital» porque al haber menos estrés «se recuperan mucho antes y existen menos complicaciones», explica Ramírez.

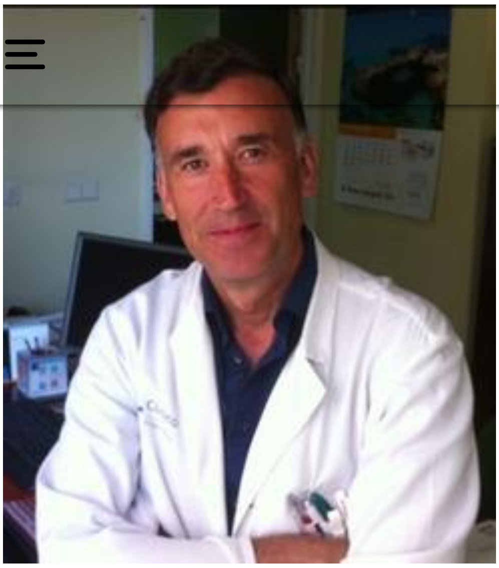

José Manuel Ramírez.

Muchas de estas ideas entran dentro de la lógica, pero «la evidencia científica tiene unos pasos» y cuesta incorporar esos estudios a la práctica habitual en las operaciones.

PUBLICIDAD

Estos protocolos ya se están implementando en los hospitales aragoneses, que «están muy involucrados», en los de Zaragoza y también Alcañiz y Calatayud, pero la idea es que «cada vez se unan más y se beneficien más pacientes», señala el investigador principal del Grupo de Cirugía y Medicina Perioperatoria del IIS Aragón. Además, quieren exportar estas ideas a nivel nacional y «también a nivel europeo», a través del proyecto Eupemen, con la formación de profesionales y la creación de una red de docentes para enseñar a estos grupos en los hospitales y auditar la correcta aplicación de los protocolos de forma homogénea.

Te puede gustar

Nuevo ID. Buzz
Volkswagen

Nuevo Alfa Romeo Tonale 100% online Rápido, fácil y cómodo
Alfa Romeo

La camisa española que no se mancha y no necesita plancha. El mejor regalo de navidad
Sepiia
Compra ahora

El asesino en serie de Castellón, a la criminóloga que le entrevistó en prisión: "No me
