# Supplementary material for: Advancing abdominal surgery recovery implementation: a unified framework for intensified recovery protocols by the EUropean PErioperative MEdical Networking collaborative
Source: Front Surg. 2026 May 18;13:1827678. doi: 10.3389/fsurg.2026.1827678 (PMC13223102; doi:10.3389/fsurg.2026.1827678)
Supplement: Supplementary file 12 [file Datasheet3.pdf]

Co-funded by the Erasmus+ Programme of the European Union

EUPEMEN European Perioperative Medical Networking

EUPEMEN PROTOKOL (CZ)

Instituto de Investigación Sanitaria Aragón

SERVIZIO SANITARIO REGIONALE EMILIA-ROMAGNA Azienda Unità Sanitaria Locale di Ferrara

2. LÉKAŘSKÁ FAKULTA UNIVERZITA KARLOVA

UNIVERSITAS Miguel Hernández

ΓΕΝΙΚΟ ΝΟΣΟΚΟΜΕΙΟ ΘΕΣΣΑΛΟΝΙΚΗΣ "Γ. ΠΑΠΑΝΙΚΟΛΑΟΥ"

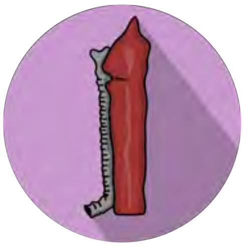

EUPEMEN PROTOKOL

RESEKCE JÍCNU

1	Přednemocniční fáze<br><br>Anesteziolog, Chirurg, Zdravotní sestra, Nutriční terapeut
1.1	**Edukace pacienta**<br>Cílem edukace je poskytnout pacientovi komplexní informace o operaci a obvyklém průběhu hospitalizace včetně podmínek propuštění. Součástí edukace je získání podepsaného informovaného souhlasu.
1.2	**Předoperační vyšetření**<br>Předoperační vyšetření zahrnuje klinické vyšetření pacienta, RTG hrudníku, laboratoř (koagulace, krevní obraz, biochemie) a EKG.
1.3	**Kompenzace chronických onemocnění**<br>Doporučena je optimalizace chronických komorbidit, u nemocných s kardiovaskulárním onemocněním je doporučeno kardiologické vyšetření.
1.4	**Předoperační vyšetření u diabetiků**<br>U všech pacientů je doporučena kontrola hladiny glukózy v krvi a kontrola glykovaného hemoglobinu (HbA1c). U nedostatečně kompenzovaných diabetiků a pacientů s nově diagnostikovaným diabetem je doporučeno vyšetření diabetologem, kompenzace stavu a výkon až v druhé době.
1.5	**Screening a korekce anémie**<br>Sideropenická anémie má být korigována pomocí preparátů intravenózního železa.
1.6	**Nutriční screening**<br>Zhodnocení nutričního stavu je doporučeno provádět rutinně u všech pacientů prostřednictvím screeningových dotazníků, například dotazník MUST (Malnutrition Universal Screening Tool). U pacientů s dysfagií je doporučena tekutá dieta s vyšším obsahem proteinů, u pacientů s afagií umělá výživa dle možností pacienta a zvyklostí pracoviště.
1.7	**Zanechání kouření a redukce konzumace alkoholu**<br>U všech pacientů je doporučeno zanechání kouření a snížení konzumace alkoholu alespoň po dobu jednoho měsíce před operací.
1.8	**Prehabilitace**<br>Je doporučen aerobní a silový trénink přizpůsobený fyzické zdatnosti pacienta.
1.9	**Psychologická konzultace**<br>Jakékoliv psychologické problémy pacienta mají být konzultovány a řešeny s psychologem.
1.10	**Zhodnocení stařecké křehkosti**<br>U pacientů starších 65 let je doporučeno stanovit skóre stařecké křehkosti (Frailty Index, Frail-VIG index).
1.11	**Apfel skóre**<br>U každého pacienta je doporučeno zhodnotit riziko vzniku pooperační nevolnosti a zvracení podle Apfela skórovacího systému.

EUPEMEN European Perioperative Medical Networking
Co-funded by the Erasmus+ Programme of the European Union
Cátedra GERM Medicina Perioperatoria Universidad Zaragoza

1.12	**Klasifikace fyzického stavu nemocného podle ASA**
2	Perioperační fáze
2.1	Časná předoperační fáze<br><br>Anesteziolog, Chirurg, Zdravotní sestra
2.1.1	**Předoperační hygiena**<br>Sprcha / koupel pacienta den nebo ráno před výkonem.
2.1.2	**Mechanická profylaxe tromboembolické nemoci**<br>Je doporučena mechanická profylaxe kompresivními punčochami nebo pomocí intermitentní pneumatické komprese dle individuálních rizik pacienta.
2.1.3	**Farmakologická profylaxe tromboembolické nemoci**<br>Je doporučena aplikace nízkomolekulárního heparinu 2-12 hodin před operací v závislosti, zda je plánována neuroaxiální anestezie či ne.
2.1.4	**Sacharidový nápoj**<br>Je doporučeno podání sacharidového nápoje (12,5 % roztok maltodextrinu) v objemu 800 ml večer před operací a 400 ml 2 hodiny před úvodem do anestezie, pokud není přítomna žádná kontraindikace. U diabetiků je doporučeno podání sacharidového nápoje s antidiabetickou medikací.
2.1.5	**Předoperační lačnění**<br>Je doporučeno podávání lehké stravy do 6 hodin a čirých tekutin do 2 hodin před operací.
2.1.6	**Příprava operačního pole**<br>Odstranění ochlupení při přípravě operačního pole je doporučeno elektrickým strojkem, nikoliv jednorázovou žiletkou.
2.1.7	**Antibiotická profylaxe**<br>Intravenózní antibiotická profylaxe má být aplikována u všech pacientů v intervalu 30-60 minut před provedením incize. Pokud délka operace přesáhne dva poločasy eliminace léku, je doporučeno intraoperačně opakovat profylaktické podání antibiotika.
2.1.8	**Profylaxe regurgitace**<br>U pacientů s evakuační poruchou žaludku je doporučeno předoperační podání prokinetik v kombinaci s dalšími opatřeními k prevenci regurgitace.
2.2	Intraoperační fáze<br><br>Anesteziolog, Chirurg, Zdravotní sestra
2.2.1	**WHO Surgical Safety Checklist**

EUPEMEN European Perioperative Medical Networking

Co-funded by the Erasmus+ Programme of the European Union

Cátedra GERM Medicina Perioperatoria Universidad Zaragoza

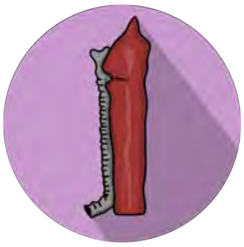

EUPEMEN PROTOKOL

RESEKCE JÍCNU

2.2.2	**Rutinní intraoperační monitorace**<br>Během výkonu je doporučeno provádět rutinně monitoraci vitálních funkcí, monitoraci hloubky celkové anestezie, monitoraci nervosvalové blokády, monitoraci glykémie a neinvazivní hemodynamickou monitoraci.
2.2.3	**Minimálně invazivní přístup**<br>Preferován je minimálně invazivní přístup dle zkušeností pracoviště.
2.2.4	**Derivace moči**<br>Rutinní použití permanentního močového katetru není doporučeno.
2.2.5	**Arteriální katetr**<br>Rutinní použití arteriálního katetru se nedoporučuje. Arteriální katetr je vyhrazen pro pacienty se závažným kardiorespiračním onemocněním.
2.2.6	**Centrální žilní katetr**<br>Rutinní použití centrálního žilního katetru se nedoporučuje u malých resekčních výkon a absenci rizikových faktorů renálního selhání v pooperačním období.
2.2.7	**Úvod a vedení anestezie**<br>Pro úvod a vedení anestezie je doporučeno použití krátkodobě působících anestetik.
2.2.8	**Oxygenace**<br>U všech pacientů je doporučena oxygenace s frakcí kyslíku více než 50 %.
2.2.9	**Tekutinová terapie**<br>Doporučena je cílená tekutinová terapie pomocí hemodynamické monitorace. Pokud není monitorace k dispozici je doporučen restriktivní tekutinový režim dle ideální váhy pacienta balancovanými roztoky rychlostí 1-3 ml/kg/h (laparoskopie) nebo 3-5 ml/kg/h (laparotomie). Krevní ztráta má být hrazena koloidy v poměru 1:1.
2.2.10	**Použití nazogastrické sondy**<br>Preventivní použití nazogastrické sondy není doporučeno. Pokud je nazogastrická sonda zavedena během operace, má být odstraněna před ukončením anestezie.
2.2.11	**Prevence hypotermie**<br>Je doporučen aktivní ohřev pacienta pomocí přikrývek či podložek s proudícím teplým vzduchem a ohřev infuzí.
2.2.12	**Prevence pooperační nevolnosti a zvracení**<br>Je doporučena kombinace antiemetik podle Apfela skórovacího systému.
2.2.13	**Epidurální analgezie**<br>Hrudní epidurální analgezie (TEA) je doporučena u otevřených výkonů. U laparoskopických výkonů TEA doporučena není. U pacientů s kontraindikací k epidurální analgezii, rizikem renálního selhání a koagulopatií je doporučena blokáda břišní stěny (TAP blok) nebo jiné koanalgetické techniky.
2.2.14	**Adjuvantní intravenózní analgetika**<br>Doporučeny jsou nesteroidní protizánětlivé léky, lidokain, ketamin, magnezium sulfát a dexmedetomidin.

EUPEMEN European Perioperative Medical Networking
Co-funded by the Erasmus+ Programme of the European Union
Cátedra GERM Medicina Perioperatoria Universidad Zaragoza

2.2.15	**Perioperační kontrola glykémie**<br>U pacientů s rizikem rozvoje inzulinové rezistence by hladina glykémie neměla překročit 10 mmol/l.
2.2.16	**Dezinfekce operačního pole**<br>Doporučen je alkoholový roztok Chlorhexidine 2 %. Dezinfekce kůže se provádí od středu operačního pole na jeho periferii.
2.2.17	**Použití břišního drénu**<br>Drenáž břišní dutiny nemá být rutinně prováděna.
2.3	Časná pooperační fáze<br>(ARK / JIP ve vybraných případech)<br><br>Anesteziolog, Zdravotní sestra
2.3.1	**Udržení normotermie**<br>U všech pacientů je doporučena monitorace tělesné teploty a aktivní ohřev pacienta k udržení normotermie.
2.3.2	**Multimodální opioidy šetřící analgezie**<br>U všech pacientů je doporučena multimodální opioidy šetřící analgezie s kontrolou bolesti do VAS 3.
2.3.3	**Restriktivní tekutinový režim**
2.3.4	**Časný perorální příjem**<br>Časný perorální příjem má být zahájen čirými tekutinami 6-8 hodin po operaci.
2.3.5	**Dechová rehabilitace**
2.3.6	**Časná mobilizace**<br>Sed na lůžku je doporučen 3 hodiny po operaci.
2.3.7	**Antitrombotická profylaxe**<br>Je doporučena aplikace nízkomolekulárního heparinu 12 hodin po operaci.
2.3.8	**Prevence pooperační nevolnosti a zvracení**<br>Je doporučena kombinace antiemetik podle Apfela skórovacího systému.
2.3.9	**Pokračování v oxygenaci frakcí kyslíku 0,5 po dobu 2 hodin po operaci**
3	Pooperační den 1<br>(ARK / JIP ve vybraných případech)<br><br>Chirurg, Anesteziolog, Zdravotní sestra
3.1	**Časný perorální příjem**<br>Tekutá dieta / Kašovitá dieta dle tolerance pacienta. Při nedostatečné toleranci podání parenterální výživy.
3.2	**Restriktivní tekutinový režim**

EUPEMEN European Perioperative Medical Networking

Co-funded by the Erasmus+ Programme of the European Union

Cátedra GERM Medicina Perioperatoria Universidad Zaragoza

3.3	**Časná mobilizace**<br>Mobilizace pacienta po pokoji (z lůžka na křeslo).
3.4	**Multimodální opioidy šetřící analgezie**<br>U všech pacientů je doporučena multimodální opioidy šetřící analgezie s kontrolou bolesti do VAS 3.
3.5	**Odstranění permanentního močového katetru**<br>Odstranění permanentního močového katetru, pokud byl zaveden.
3.6	**Dechová rehabilitace**
3.7	**Antitrombotická profylaxe**
3.8	**Laboratoř**<br>Kontrola krevního obrazu, CRP a prokalcitoninu.
4	Pooperační den 2<br>(ARK / JIP / Standardní oddělení)<br><br>Chirurg, Anesteziolog, Zdravotní sestra
4.1	**Časný perorální příjem**<br>Kašovitá dieta (pyré, jogurt…)
4.2	**Ukončení infuzní terapie**<br>Ukončení infuzní terapie u pacientů tolerujících perorální příjem.
4.3	**Časná mobilizace**<br>Procházky na krátké vzdálenosti.
4.4	**Multimodální opioidy šetřící analgezie**<br>U všech pacientů je doporučena multimodální opioidy šetřící analgezie s kontrolou bolesti do VAS 3.
4.5	**Odstranění epidurálního katetru**<br>Odstranění epidurálního katetru, pokud byl zaveden. Před odstraněním kontrola koagulačních parametrů.
4.6	**Dechová rehabilitace**
4.7	**Antitrombotická profylaxe**
5	Pooperační den 3<br>(Standardní oddělení)<br><br>Chirurg, Zdravotní sestra
5.1	**Časný perorální příjem**<br>Mixovaná dieta
5.2	**Časná mobilizace**

	Plná mobilizace.
5.3	**Multimodální opioidy šetřící analgezie**<br>U všech pacientů je doporučena multimodální opioidy šetřící analgezie s kontrolou bolesti do VAS 3.
5.4	**Dechová rehabilitace**
5.5	**Antitrombotická profylaxe**
5.6	**Laboratoř**<br>Kontrola krevního obrazu, CRP a prokalcitoninu.
5.7	**Zhodnocení kritérií propuštění pacienta**<br>Pacient bez komplikace či s komplikací zvládnutelnou ambulantně, bez septických projevů, kontrola bolesti perorálními analgetiky, plná mobilizace, tolerance perorálního příjmu a souhlas pacienta.
6	Pooperační den 4, Propuštění a Follow-up<br>Chirurg, Zdravotní sestra, Primární péče
6.1	**Perorální příjem**<br>Šetřící dieta.
6.2	**Časná mobilizace**<br>Plná mobilizace.
6.3	**Multimodální opioidy šetřící analgezie**<br>U všech pacientů je doporučena multimodální opioidy šetřící analgezie s kontrolou bolesti do VAS 3.
6.4	**Dechová rehabilitace**
6.5	**Antitrombotická profylaxe**
6.6	**Laboratoř**<br>Kontrola krevního obrazu, CRP a prokalcitoninu.
7	Propuštění<br>Chirurg, Zdravotní sestra, Primární péče
7.1	**Dokumentace pacienta**<br>Při propuštění má být připravena propouštěcí zpráva a předána do rukou pacienta. V propouštěcí zprávě má být popsán průběh hospitalizace a doporučen další postup péče (dietní a režimová opatření, termíny kontrol).

EUPEMEN European Perioperative Medical Networking
Co-funded by the Erasmus+ Programme of the European Union
Cátedra GERM Medicina Perioperatoria Universidad Zaragoza

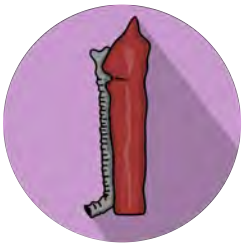

7.2	**Follow-up**<br>Sledování pacienta na chirurgické ambulanci nebo telefonicky dle zvyklostí pracoviště. Zajištění kontroly u praktického lékaře a dalších specialistů dle potřeby pacienta.
7.3	**Nutriční stav**<br>Zhodnocení nutričního příjmu pacienta (kalorie, proteiny, minerály, vitamíny).

EUPEMEN European Perioperative Medical Networking

Co-funded by the Erasmus+ Programme of the European Union

Cátedra GERM Medicina Perioperatoria Universidad Zaragoza

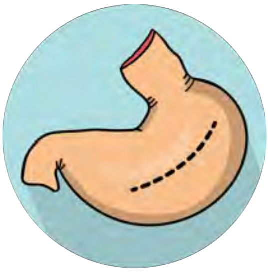

EUPEMEN PROTOKOL

RESEKCE ŽALUDKU

1	Přednemocniční fáze<br><br>Anesteziolog, Chirurg, Zdravotní sestra, Nutriční terapeut
1.1	**Edukace pacienta**<br>Cílem edukace je poskytnout pacientovi komplexní informace o operaci a obvyklém průběhu hospitalizace včetně podmínek propuštění. Součástí edukace je získání podepsaného informovaného souhlasu.
1.2	**Předoperační vyšetření**<br>Předoperační vyšetření zahrnuje klinické vyšetření pacienta, RTG hrudníku, laboratoř (koagulace, krevní obraz, biochemie, nutriční parametry) a EKG.
1.3	**Kompenzace chronických onemocnění**<br>Doporučena je optimalizace chronických komorbidit, u pacientů s restriktivním plicním onemocněním spirometrie, u nemocných s vysokým kardiovaskulárním rizikem nebo již probíhajícím kardiovaskulárním onemocněním kardiologické vyšetření.
1.4	**Screening a korekce anémie**<br>Sideropenická anémie má být korigována pomocí preparátů intravenózního železa.
1.5	**Předoperační vyšetření u diabetiků**<br>U všech pacientů je doporučena kontrola hladiny glukózy v krvi a kontrola glykovaného hemoglobinu (HbA1c). U nedostatečně kompenzovaných diabetiků a pacientů s nově diagnostikovaným diabetem je doporučeno vyšetření diabetologem, kompenzace stavu a výkon až v druhé době.
1.6	**Optimalizace nutričního stavu**<br>Zhodnocení nutričního stavu je doporučeno provádět rutinně u všech pacientů v rámci předoperačního vyšetření. K identifikaci nutričně rizikových pacientů jsou dostupné screeningové dotazníky, například dotazník MUST (Malnutrition Universal Screening Tool). Doporučena je korekce deplece kalcia, železa, vitamínu D a B12. U pacientů s dysfagií je doporučena tekutá vysokokalorická proteinová dieta, u pacientů s afagií parenterální výživa.
1.7	**Zanechání kouření a redukce konzumace alkoholu**<br>U všech pacientů je doporučeno zanechání kouření a snížení konzumace alkoholu alespoň po dobu jednoho měsíce před operací.
1.8	**Prehabilitace**<br>Je doporučen aerobní a silový trénink přizpůsobený fyzické zdatnosti pacienta.
1.9	**Psychologická konzultace**<br>Jakékoliv psychologické problémy pacienta mají být konzultovány a řešeny s psychologem.
1.10	**Apfel skóre**<br>U každého pacienta je doporučeno zhodnotit riziko vzniku pooperační nevolnosti a zvracení podle Apfela skórovacího systému.

EUPEMEN
European Perioperative Medical Networking

Co-funded by the
Erasmus+ Programme
of the European Union

Cátedra GERM
Medicina Perioperatoria
Universidad Zaragoza

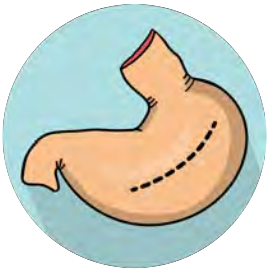

EUPEMEN PROTOKOL

RESEKCE ŽALUDKU

1.11	**Klasifikace fyzického stavu nemocného podle ASA**
2	Perioperační fáze
2.1	Časná předoperační fáze<br>(Přijetí pacienta v den operace)<br><br>Anesteziolog, Chirurg, Zdravotní sestra
2.1.1	**Předoperační lačnění**<br>Je doporučeno podávání lehké stravy do 8 hodin a čirých tekutin do 2 hodin před operací.
2.1.2	**Farmakologická profylaxe tromboembolické nemoci**<br>Je doporučena aplikace nízkomolekulárního heparinu 2-12 hodin před operací v závislosti, zda je plánována neuroaxiální anestezie či ne.
2.1.3	**Mechanická profylaxe tromboembolické nemoci**<br>Je doporučena mechanická profylaxe kompresivními punčochami nebo pomocí intermitentní pneumatické komprese dle individuálních rizik pacienta.
2.1.4	**Sacharidový nápoj**<br>Je doporučeno podání sacharidového nápoje (12,5 % roztok maltodextrinu) v objemu 400 ml 2 hodiny před úvodem do anestezie, pokud není přítomna žádná kontraindikace.
2.1.5	**Premedikace**<br>Aplikace dlouhodobě a krátkodobě působících sedativ (zejména benzodiazepinů) není doporučena.
2.1.6	**Příprava operačního pole**<br>Odstranění ochlupení při přípravě operačního pole je doporučeno elektrickým strojkem, nikoliv jednorázovou žiletkou.
2.1.7	**Antibiotická profylaxe**<br>Intravenózní antibiotická profylaxe má být aplikována u všech pacientů v intervalu 30-60 minut před provedením incize. Volba antibiotika závisí na místním doporučení antibiotického střediska.
2.1.8	**Profylaxe regurgitace**<br>U pacientů s evakuační poruchou žaludku je doporučeno předoperační podání prokinetik v kombinaci s dalšími opatřeními k prevenci regurgitace.
2.2	Intraoperační fáze<br><br>Anesteziolog, Chirurg, Zdravotní sestra
2.2.1	**WHO Surgical Safety Checklist**

EUPEMEN European Perioperative Medical Networking

Co-funded by the Erasmus+ Programme of the European Union

Cátedra GERM Medicina Perioperatoria Universidad Zaragoza

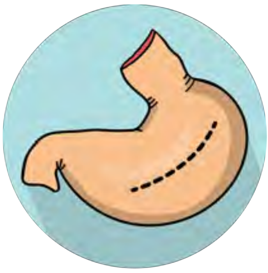

EUPEMEN PROTOKOL

RESEKCE ŽALUDKU

2.2.2	**Rutinní intraoperační monitorace**<br>Během výkonu je doporučeno provádět rutinně monitoraci vitálních funkcí, monitoraci hloubky celkové anestezie, monitoraci nervosvalové blokády a monitoraci glykémie. Doporučena je i neinvazivní hemodynamická monitorace.
2.2.3	**Arteriální katetr**<br>Rutinní použití arteriálního katetru se nedoporučuje. Arteriální katetr je vyhrazen pro pacienty se závažným kardiorespiračním onemocněním.
2.2.4	**Centrální žilní katetr**<br>Rutinní použití centrálního žilního katetru se nedoporučuje. Centrální žilní katetr je vyhrazen pro pacienty se zvýšeným rizikem renálního selhání v pooperačním období.
2.2.5	**Derivace moči**<br>Rutinní použití permanentního močového katetru není doporučeno.
2.2.6	**Úvod a vedení anestezie**<br>Pro úvod a vedení anestezie je doporučeno použití krátkodobě působících anestetik.
2.2.7	**Oxygenace**<br>U všech pacientů je doporučena oxygenace s frakcí kyslíku více než 50 %.
2.2.8	**Tekutinová terapie**<br>Doporučena je cílená tekutinová terapie pomocí neinvazivní hemodynamické monitorace. Pokud není monitorace k dispozici je doporučen restriktivní tekutinový režim dle ideální váhy pacienta.
2.2.9	**Prevence hypotermie**<br>Je doporučen aktivní ohřev pacienta pomocí přikrývek či podložek s proudícím teplým vzduchem a ohřev infuzí.
2.2.10	**Prevence pooperační nevolnosti a zvracení**<br>Je doporučena kombinace antiemetik podle Apfela skórovacího systému.
2.2.11	**Epidurální analgezie**<br>Hrudní epidurální analgezie (TEA) je doporučena u otevřených výkonů. U laparoskopických výkonů TEA doporučena není. U pacientů s kontraindikací k epidurální analgezii, rizikem renálního selhání a koagulopatií je doporučena blokáda břišní stěny (TAP blok) nebo jiné koanalgetické techniky.
2.2.12	**Minimálně invazivní přístup**<br>Doporučen je minimálně invazivní přístup.
2.2.13	**Použití nazogastrické sondy**<br>Preventivní použití nazogastrické sondy není doporučeno. Pokud je nazogastrická sonda zavedena během operace, má být odstraněna před ukončením anestezie.
2.2.14	**Použití břišního drénu**<br>Drenáž břišní dutiny nemá být rutinně prováděna.

EUPEMEN European Perioperative Medical Networking
Co-funded by the Erasmus+ Programme of the European Union
Cátedra GERM Medicina Perioperatoria Universidad Zaragoza

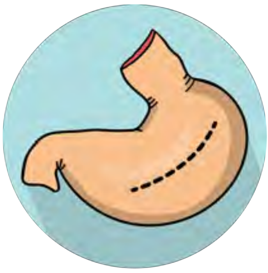

EUPEMEN PROTOKOL

RESEKCE ŽALUDKU

2.3	**Časná pooperační fáze**<br>Anesteziolog, Zdravotní sestra
2.3.1	**Udržení normotermie**<br>U všech pacientů je doporučena monitorace tělesné teploty a aktivní ohřev pacienta k udržení normotermie.
2.3.2	**Multimodální opioidy šetřící analgezie**<br>U všech pacientů je doporučena multimodální opioidy šetřící analgezie s kontrolou bolesti do VAS 3.
2.3.3	**Časný perorální příjem**<br>Časný perorální příjem má být zahájen čirými tekutinami 6 hodin po operaci.
2.3.4	**Časná mobilizace**<br>Sed na lůžku je doporučen 3 hodiny po operaci, chůze 6 hodin po operaci s respektováním nočního klidu pro spánek.
2.3.5	**Antitrombotická profylaxe**<br>Je doporučena aplikace nízkomolekulárního heparinu 12 hodin po operaci.
2.3.6	**Prevence pooperační nevolnosti a zvracení**<br>Je doporučena kombinace antiemetik podle Apfela skórovacího systému.
3	**Pooperační den 1**<br>(Standardní oddělení)<br>Chirurg, Zdravotní sestra
3.1	**Perorální příjem**<br>Tekutá nízkokalorická dieta dle tolerance pacienta.
3.2	**Časná mobilizace**<br>Plná mobilizace.
3.3	**Multimodální opioidy šetřící analgezie**<br>U všech pacientů je doporučena multimodální opioidy šetřící analgezie s kontrolou bolesti do VAS 3.
3.4	**Ukončení infuzní terapie**<br>Ukončení infuzní terapie u pacientů tolerujících perorální příjem.
3.5	**Odstranění permanentního močového katetru**<br>Odstranění permanentního močového katetru, pokud byl zaveden.
3.6	**Odstranění břišních drénů**<br>Odstranění břišních drénů, pokud byly zavedeny.
3.7	**Antitrombotická profylaxe**

EUPEMEN European Perioperative Medical Networking
Co-funded by the Erasmus+ Programme of the European Union
Cátedra GERM Medicina Perioperatoria Universidad Zaragoza

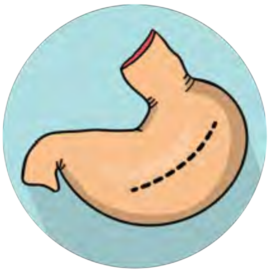

3.8	**Dechová rehabilitace**
4	Pooperační den 2<br>(Standardní oddělení)<br><br>Chirurg, Zdravotní sestra
4.1	**Perorální příjem**<br>Kašovitá dieta (pyré, jogurt...)
4.2	**Časná mobilizace**<br>Plná mobilizace.
4.3	**Multimodální opioidy šetřící analgezie**<br>U všech pacientů je doporučena multimodální opioidy šetřící analgezie s kontrolou bolesti do VAS 3.
4.4	**Odstranění epidurálního katetru**<br>Odstranění epidurálního katetru, pokud byl zaveden. Před odstraněním kontrola koagulačních parametrů.
4.5	**Dechová a pohybová rehabilitace**
4.6	**Antitrombotická profylaxe**
5	Pooperační den 3<br>(Standardní oddělení)<br><br>Chirurg, Zdravotní sestra
5.1	**Perorální příjem**<br>Šetřící dieta
5.2	**Časná mobilizace**<br>Plná mobilizace.
5.3	**Multimodální opioidy šetřící analgezie**<br>U všech pacientů je doporučena multimodální opioidy šetřící analgezie s kontrolou bolesti do VAS 3.
5.4	**Dechová a pohybová rehabilitace**
5.5	**Antitrombotická profylaxe**
5.6	**Laboratoř**<br>Kontrola krevního obrazu, CRP a prokalcitoninu.
5.7	**Zhodnocení kritérií propuštění pacienta**

EUPEMEN European Perioperative Medical Networking

Co-funded by the Erasmus+ Programme of the European Union

Cátedra GERM Medicina Perioperatoria Universidad Zaragoza

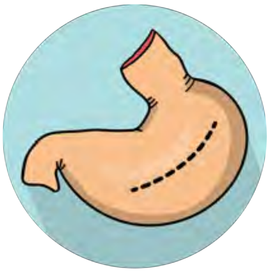

	Pacient bez komplikace či s komplikací zvládnutelnou ambulantně, bez septických projevů, kontrola bolesti perorálními analgetiky, plná mobilizace, tolerance perorálního příjmu a souhlas pacienta.
6	Pooperační den 4<br>(Standardní oddělení)<br><br>Chirurg, Zdravotní sestra
6.1	**Perorální příjem**<br>Šetřící dieta
6.2	**Časná mobilizace**<br>Plná mobilizace.
6.3	**Multimodální opioidy šetřící analgezie**<br>U všech pacientů je doporučena multimodální opioidy šetřící analgezie s kontrolou bolesti do VAS 3.
6.4	**Dechová a pohybová rehabilitace**
6.5	**Antitrombotická profylaxe**
6.6	**Laboratoř**<br>Kontrola krevního obrazu, CRP a prokalcitoninu.
6.7	**Zhodnocení kritérií propuštění pacienta**<br>Pacient bez komplikace či s komplikací zvládnutelnou ambulantně, bez septických projevů, kontrola bolesti perorálními analgetiky, plná mobilizace, tolerance perorálního příjmu a souhlas pacienta.
7	Propuštění<br><br>Chirurg, Zdravotní sestra, Psycholog, Primární péče
7.1	**Dokumentace pacienta**<br>Při propuštění má být připravena propouštěcí zpráva a předána do rukou pacienta. V propouštěcí zprávě má být popsán průběh hospitalizace a doporučen další postup péče.
7.2	**Follow-up**<br>Telefonický kontakt po propuštění. Další péče ve spolupráci s praktickým lékařem.
7.3	**Nutriční stav**<br>Zhodnocení nutričního příjmu pacienta (kalorie, proteiny, minerály, vitamíny).
7.4	**Psychologická konzultace**<br>Konzultace psychologa dle potřeb pacienta. Zhodnocení pooperační kvality života.

EUPEMEN European Perioperative Medical Networking

Co-funded by the Erasmus+ Programme of the European Union

Cátedra GERM Medicina Perioperatoria Universidad Zaragoza

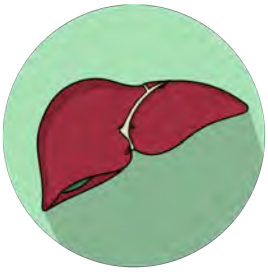

EUPEMEN PROTOKOL

RESEKCE JATER

1	**Přednemocniční fáze**<br><br>Anesteziolog, Chirurg, Zdravotní sestra, Nutriční terapeut
1.1	**Edukace pacienta**<br>Cílem edukace je poskytnout pacientovi komplexní informace o operaci a obvyklém průběhu hospitalizace včetně podmínek propuštění. Součástí edukace je získání podepsaného informovaného souhlasu.
1.2	**Předoperační vyšetření**<br>Předoperační vyšetření zahrnuje klinické vyšetření pacienta, RTG hrudníku, laboratoř (koagulace, krevní obraz, biochemie) a EKG.
1.3	**Kompenzace chronických onemocnění**<br>Doporučena je optimalizace chronických komorbidit, u nemocných s kardiovaskulárním onemocněním je doporučeno kardiologické vyšetření.
1.4	**Předoperační vyšetření u diabetiků**<br>U všech pacientů je doporučena kontrola hladiny glukózy v krvi a kontrola glykovaného hemoglobinu (HbA1c). U nedostatečně kompenzovaných diabetiků a pacientů s nově diagnostikovaným diabetem je doporučeno vyšetření diabetologem, kompenzace stavu a výkon až v druhé době.
1.5	**Screening a korekce anémie**<br>Sideropenická anémie má být korigována pomocí preparátů intravenózního železa.
1.6	**Nutriční screening**<br>Zhodnocení nutričního stavu je doporučeno provádět rutinně u všech pacientů prostřednictvím screeningových dotazníků, například dotazník MUST (Malnutrition Universal Screening Tool).
1.7	**Zanechání kouření a redukce konzumace alkoholu**<br>U všech pacientů je doporučeno zanechání kouření a snížení konzumace alkoholu alespoň po dobu jednoho měsíce před operací.
1.8	**Prehabilitace**<br>Je doporučen aerobní a silový trénink přizpůsobený fyzické zdatnosti pacienta.
1.9	**Psychologická konzultace**<br>Jakékoliv psychologické problémy pacienta mají být konzultovány a řešeny s psychologem.
1.10	**Zhodnocení stařecké křehkosti**<br>U pacientů starších 65 let je doporučeno stanovit skóre stařecké křehkosti (Frailty Index, Frail-VIG index).
1.11	**Apfel skóre**<br>U každého pacienta je doporučeno zhodnotit riziko vzniku pooperační nevolnosti a zvracení podle Apfela skórovacího systému.

EUPEMEN
European Perioperative Medical Networking

Co-funded by the
Erasmus+ Programme
of the European Union

Cátedra GERM
Medicina Perioperatoria
Universidad Zaragoza

EUPEMEN PROTOKOL

RESEKCE JATER

1.12	**Klasifikace fyzického stavu nemocného podle ASA**
2	Perioperační fáze
2.1	Časná předoperační fáze<br><br>Anesteziolog, Chirurg, Zdravotní sestra
2.1.1	**Předoperační hygiena**<br>Sprcha / koupel pacienta den nebo ráno před výkonem.
2.1.2	**Farmakologická profylaxe tromboembolické nemoci**<br>Je doporučena aplikace nízkomolekulárního heparinu 2-12 hodin před operací v závislosti, zda je plánována neuroaxiální anestezie či ne.
2.1.3	**Mechanická profylaxe tromboembolické nemoci**<br>Je doporučena mechanická profylaxe kompresivními punčochami nebo pomocí intermitentní pneumatické komprese dle individuálních rizik pacienta.
2.1.4	**Sacharidový nápoj**<br>Je doporučeno podání sacharidového nápoje (12,5 % roztok maltodextrinu) v objemu 800 ml večer před operací a 400 ml 2 hodiny před úvodem do anestezie, pokud není přítomna žádná kontraindikace. U diabetiků je doporučeno podání sacharidového nápoje s antidiabetickou medikací.
2.1.5	**Předoperační lačnění**<br>Je doporučeno podávání lehké stravy do 6 hodin a čirých tekutin do 2 hodin před operací.
2.1.6	**Příprava operačního pole**<br>Odstranění ochlupení při přípravě operačního pole je doporučeno elektrickým strojkem, nikoliv jednorázovou žiletkou.
2.1.7	**Antibiotická profylaxe**<br>Intravenózní antibiotická profylaxe má být aplikována u všech pacientů v intervalu 30-60 minut před provedením incize. Pokud délka operace přesáhne dva poločasy eliminace léku, je doporučeno intraoperačně opakovat profylaktické podání antibiotika.
2.2	Intraoperační fáze<br><br>Anesteziolog, Chirurg, Zdravotní sestra
2.2.1	**WHO Surgical Safety Checklist**
2.2.2	**Rutinní intraoperační monitorace**<br>Během výkonu je doporučeno provádět rutinně monitoraci vitálních funkcí,

EUPEMEN European Perioperative Medical Networking
Co-funded by the Erasmus+ Programme of the European Union
Cátedra GERM Medicina Perioperatoria Universidad Zaragoza

	monitoraci hloubky celkové anestezie, monitoraci nervosvalové blokády, monitoraci glykémie a neinvazivní hemodynamickou monitoraci.
2.2.3	**Minimálně invazivní přístup**<br>Preferován je minimálně invazivní přístup dle zkušeností pracoviště.
2.2.4	**Derivace moči**<br>Rutinní použití permanentního močového katetru není doporučeno.
2.2.5	**Arteriální katetr**<br>Rutinní použití arteriálního katetru se nedoporučuje. Arteriální katetr je vyhrazen pro pacienty se závažným kardiorespiračním onemocněním.
2.2.6	**Centrální žilní katetr**<br>Rutinní použití centrálního žilního katetru se nedoporučuje u malých resekčních výkon a absenci rizikových faktorů renálního selhání v pooperačním období.
2.2.7	**Úvod a vedení anestezie**<br>Pro úvod a vedení anestezie je doporučeno použití krátkodobě působících anestetik.
2.2.8	**Oxygenace**<br>U všech pacientů je doporučena oxygenace s frakcí kyslíku více než 50 %.
2.2.9	**Tekutinová terapie**<br>V průběhu resekční fáze je doporučeno držet hladinu centrálního žilního tlaku pod 5 cm H2O. Doporučena je cílená tekutinová terapie pomocí hemodynamické monitorace. Pokud není monitorace k dispozici je doporučen restriktivní tekutinový režim dle ideální váhy pacienta balancovanými roztoky rychlostí 1-3 ml/kg/h (laparoskopie) nebo 3-5 ml/kg/h (laparotomie). Krevní ztráta má být hrazena koloidy v poměru 1:1.
2.2.10	**Použití nazogastrické sondy**<br>Preventivní použití nazogastrické sondy není doporučeno. Pokud je nazogastrická sonda zavedena během operace, má být odstraněna před ukončením anestezie.
2.2.11	**Prevence hypotermie**<br>Je doporučen aktivní ohřev pacienta pomocí přikrývek či podložek s proudícím teplým vzduchem a ohřev infuzí.
2.2.12	**Prevence pooperační nevolnosti a zvracení**<br>Je doporučena kombinace antiemetik podle Apfela skórovacího systému.
2.2.13	**Epidurální analgezie**<br>Hrudní epidurální analgezie (TEA) je doporučena u otevřených výkonů. U laparoskopických výkonů TEA doporučena není. U pacientů s kontraindikací k epidurální analgezii, rizikem renálního selhání a koagulopatií je doporučena blokáda břišní stěny (TAP blok) nebo jiné koanalgetické techniky.

EUPEMEN PROTOKOL

RESEKCE JATER

2.2.14	**Adjuvantní intravenózní analgetika**<br>Doporučeny jsou nesteroidní protizánětlivé léky, lidokain, ketamin, magnezium sulfát a dexmedetomidin.
2.2.15	**Perioperační kontrola glykémie**<br>U pacientů s rizikem rozvoje inzulinové rezistence by hladina glykémie neměla překročit 10 mmol/l.
2.2.16	**Dezinfekce operačního pole**<br>Doporučen je alkoholový roztok Chlorhexidine 2 %. Dezinfekce kůže se provádí od středu operačního pole na jeho periferii.
2.2.17	**Použití břišního drénu**<br>Drenáž břišní dutiny nemá být rutinně prováděna.
2.3	Časná pooperační fáze<br>(JIP / Intermediární jednotka)<br>Anesteziolog, Zdravotní sestra
2.3.1	**Udržení normotermie**<br>U všech pacientů je doporučena monitorace tělesné teploty a aktivní ohřev pacienta k udržení normotermie.
2.3.2	**Multimodální opioidy šetřící analgezie**<br>U všech pacientů je doporučena multimodální opioidy šetřící analgezie s kontrolou bolesti do VAS 3.
2.3.3	**Restriktivní tekutinový režim**
2.3.4	**Časný perorální příjem**<br>Časný perorální příjem má být zahájen čirými tekutinami 3 hodin po operaci.
2.3.5	**Dechová rehabilitace**
2.3.6	**Časná mobilizace**<br>Sed na lůžku je doporučen 3 hodiny po operaci.
2.3.7	**Antitrombotická profylaxe**<br>Je doporučena aplikace nízkomolekulárního heparinu 12 hodin po operaci.
2.3.8	**Prevence pooperační nevolnosti a zvracení**<br>Je doporučena kombinace antiemetik podle Apfela skórovacího systému.
2.3.9	**Pokračování v oxygenaci frakcí kyslíku 0,5 po dobu 2 hodin po operaci**

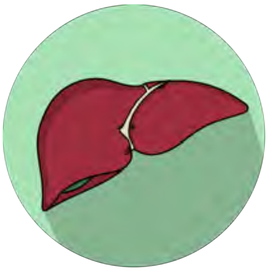

EUPEMEN PROTOKOL

RESEKCE JATER

3	Pooperační den 1<br>(Standardní oddělení)<br><br>Chirurg, Zdravotní sestra
3.1	**Orální nutriční suplementy s vyšším obsahem proteinů**<br>Orální nutriční suplementy jsou doporučeny u pacientů s energetickým příjmem pod 60 % a u pacientů s malnutricí.
3.2	**Časný perorální příjem**<br>Kašovitá dieta / Šetřící dieta dle tolerance pacienta.
3.3	**Časná mobilizace**<br>Mobilizace pacienta po pokoji (z lůžka na křeslo).
3.4	**Odstranění břišních drénů**<br>Odstranění břišních drénů, pokud byly zavedeny.
3.5	**Multimodální opioidy šetřící analgezie**<br>U všech pacientů je doporučena multimodální opioidy šetřící analgezie s kontrolou bolesti do VAS 3.
3.6	**Ukončení infuzní terapie**<br>Ukončení infuzní terapie u pacientů tolerujících perorální příjem.
3.7	**Odstranění permanentního močového katetru**<br>Odstranění permanentního močového katetru, pokud byl zaveden.
3.8	**Dechová rehabilitace**
3.9	**Prevence pooperační nevolnosti a zvracení**<br>Je doporučena kombinace antiemetik podle Apfela skórovacího systému.
3.10	**Antiulcerogenní profylaxe**
3.11	**Antitrombotická profylaxe**
3.12	**Zhodnocení kritérií propuštění pacienta**<br>V případě laparoskopické resekce zhodnocení kritérií propuštění pacienta.
4	Pooperační den 2<br><br>Chirurg, Zdravotní sestra
4.1	**Časný perorální příjem**<br>Šetřící dieta dle tolerance pacienta.
4.2	**Časná mobilizace**<br>Procházky na krátké vzdálenosti.

EUPEMEN
European Perioperative Medical Networking
Co-funded by the Erasmus+ Programme of the European Union
Cátedra GERM Medicina Perioperatoria Universidad Zaragoza

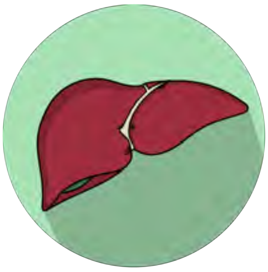

4.3	**Multimodální opioidy šetřící analgezie**<br>U všech pacientů je doporučena multimodální opioidy šetřící analgezie s kontrolou bolesti do VAS 3.
4.4	**Antitrombotická profylaxe**
4.5	**Laboratoř**<br>Kontrola krevního obrazu, CRP a prokalcitoninu.
4.6	**Zhodnocení kritérií propuštění pacienta**<br>Pacient bez komplikace či s komplikací zvládnutelnou ambulantně, bez septických projevů, kontrola bolesti perorálními analgetiky, plná mobilizace, tolerance perorálního příjmu a souhlas pacienta.
5	Propuštění<br><br>Chirurg, Zdravotní sestra, Primární péče
5.1	**Dokumentace pacienta**<br>Při propuštění má být připravena propouštěcí zpráva a předána do rukou pacienta. V propouštěcí zprávě má být popsán průběh hospitalizace a doporučen další postup péče.
5.2	**Follow-up**<br>Telefonický kontakt s pacientem či kontrola na chirurgické ambulanci po propuštění. Další kontroly jsou doporučeny za 1, 3 a 6 měsíců. Ve spolupráci s praktickým lékařem zajištění domácí péče podle potřeby pacienta.

EUPEMEN European Perioperative Medical Networking

Co-funded by the Erasmus+ Programme of the European Union

Cátedra GERM Medicina Perioperatoria Universidad Zaragoza

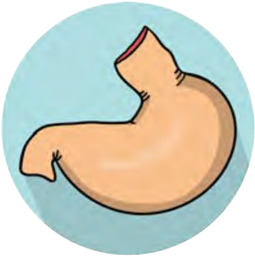

EUPEMEN PROTOKOL

BARIATRICKÁ CHIRURGIE

1	Přednemocniční fáze<br><br>Anesteziolog, Chirurg, Zdravotní sestra, Nutriční terapeut
1.1	**Edukace pacienta**<br>Cílem edukace je poskytnout pacientovi komplexní informace o operaci a obvyklém průběhu hospitalizace včetně podmínek propuštění. Součástí edukace je získání podepsaného informovaného souhlasu.
1.2	**Předoperační vyšetření**<br>Předoperační vyšetření zahrnuje klinické vyšetření pacienta, RTG hrudníku, laboratoř (koagulace, krevní obraz, biochemie, nutriční parametry) a EKG.
1.3	**Gastroskopie**<br>V rámci předoperačního vyšetření je doporučeno provedení endoskopie horní části zažívacího traktu. V případě průkazu Helicobacter pylori je indikována jeho eradikace před plánovanou operací.
1.4	**Kompenzace chronických onemocnění**<br>Doporučena je optimalizace chronických komorbidit, u pacientů s restriktivním plicním onemocněním spirometrie, u nemocných s vysokým kardiovaskulárním rizikem nebo již probíhajícím kardiovaskulárním onemocněním kardiologické vyšetření.
1.5	**Předoperační vyšetření u diabetiků**<br>U všech pacientů je doporučena kontrola hladiny glukózy v krvi a kontrola glykovaného hemoglobinu (HbA1c). U nedostatečně kompenzovaných diabetiků a pacientů s nově diagnostikovaným diabetem je doporučeno vyšetření diabetologem, kompenzace stavu a výkon až v druhé době.
1.6	**Screening spánkové apnoe**<br>U všech pacientů je doporučeno provedení screeningového STOP-BANG testu. Při skóre vyšším než 3 je indikováno vyšetření ve spánkové laboratoři (polysomnografie).
1.7	**Optimalizace nutričního stavu**<br>Je doporučena redukce hmotnosti nízkokalorickou dietu s eventuální medikamentózní terapií. Součástí nutriční přípravy pacienta je korekce deplece kalcia, železa, vitamínu D a vitamínu B12.
1.8	**Zanechání kouření a redukce konzumace alkoholu**<br>U všech pacientů je doporučeno zanechání kouření a snížení konzumace alkoholu alespoň po dobu jednoho měsíce před operací.
1.9	**Prehabilitace**<br>Je doporučen aerobní a silový trénink přizpůsobený fyzické zdatnosti pacienta.
1.10	**Psychologická konzultace**<br>Jakékoliv psychologické problémy pacienta mají být konzultovány a řešeny s psychologem.
1.11	**Klasifikace fyzického stavu nemocného podle ASA**

EUPEMEN European Perioperative Medical Networking
Co-funded by the Erasmus+ Programme of the European Union
Cátedra GERM Medicina Perioperatoria Universidad Zaragoza

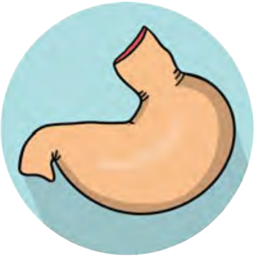

EUPEMEN PROTOKOL

BARIATRICKÁ CHIRURGIE

1.12	**Apfel skóre**<br>U každého pacienta je doporučeno zhodnotit riziko vzniku pooperační nevolnosti a zvracení podle Apfela skórovacího systému.
2	Perioperační fáze
2.1	Časná předoperační fáze<br>(Přijetí pacienta v den operace)<br><br>Anesteziolog, Chirurg, Zdravotní sestra
2.1.1	**Předoperační lačnění**<br>Je doporučeno podávání lehké stravy do 8 hodin a čirých tekutin do 2 hodin před operací.
2.1.2	**Farmakologická profylaxe tromboembolické nemoci**<br>Je doporučena aplikace nízkomolekulárního heparinu 2-12 hodin před operací v závislosti, zda je plánována neuroaxiální anestezie či ne.
2.1.3	**Mechanická profylaxe tromboembolické nemoci**<br>Je doporučena mechanická profylaxe kompresivními punčochami nebo pomocí intermitentní pneumatické komprese dle individuálních rizik pacienta.
2.1.4	**Sacharidový nápoj**<br>Je doporučeno podání sacharidového nápoje (12,5 % roztok maltodextrinu) v objemu 400 ml 2 hodiny před úvodem do anestezie, pokud není přítomna žádná kontraindikace.
2.1.5	**Premedikace**<br>Aplikace dlouhodobě a krátkodobě působících sedativ (zejména benzodiazepinů) není doporučena.
2.1.6	**Příprava operačního pole**<br>Odstranění ochlupení při přípravě operačního pole je doporučeno elektrickým strojkem, nikoliv jednorázovou žiletkou.
2.1.7	**Antibiotická profylaxe**<br>Intravenózní antibiotická profylaxe má být aplikována u všech pacientů v intervalu 30-60 minut před provedením incize. Volba antibiotika závisí na místním doporučení antibiotického střediska.
2.1.8	**Profylaxe regurgitace**<br>U pacientů s evakuační poruchou žaludku je doporučeno předoperační podání prokinetik v kombinaci s dalšími opatřeními k prevenci regurgitace.
2.2	Intraoperační fáze<br><br>Anesteziolog, Chirurg, Zdravotní sestra
2.2.1	**WHO Surgical Safety Checklist**

EUPEMEN European Perioperative Medical Networking
Co-funded by the Erasmus+ Programme of the European Union
Cátedra GERM Medicina Perioperatoria Universidad Zaragoza

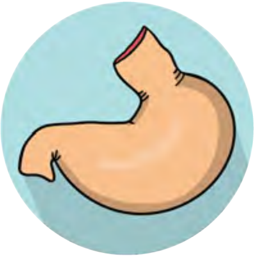

EUPEMEN PROTOKOL

BARIATRICKÁ CHIRURGIE

2.2.2	**Rutinní intraoperační monitorace**<br>Během výkonu je doporučeno provádět rutinně monitoraci vitálních funkcí, monitoraci hloubky celkové anestezie, monitoraci nervosvalové blokády a monitoraci glykémie. Doporučena je i neinvazivní hemodynamická monitorace.
2.2.3	**Arteriální katetr**<br>Rutinní použití arteriálního katetru se nedoporučuje. Arteriální katetr je vyhrazen pro pacienty se závažným kardiorespiračním onemocněním.
2.2.4	**Centrální žilní katetr**<br>Rutinní použití centrálního žilního katetru se nedoporučuje. Centrální žilní katetr je vyhrazen pro pacienty se zvýšeným rizikem renálního selhání v pooperačním období.
2.2.5	**Derivace moči**<br>Rutinní použití permanentního močového katetru není doporučeno.
2.2.6	**Úvod a vedení anestezie**<br>Pro úvod a vedení anestezie je doporučeno použití krátkodobě působících anestetik.
2.2.7	**Oxygenace**<br>U všech pacientů je doporučena oxygenace s frakcí kyslíku více než 50 %.
2.2.8	**Tekutinová terapie**<br>Doporučena je cílená tekutinová terapie pomocí neinvazivní hemodynamické monitorace. Pokud není monitorace k dispozici je doporučen restriktivní tekutinový režim dle ideální váhy pacienta.
2.2.9	**Prevence hypotermie**<br>Je doporučen aktivní ohřev pacienta pomocí přikrývek či podložek s proudícím teplým vzduchem a ohřev infuzí.
2.2.10	**Prevence pooperační nevolnosti a zvracení**<br>Je doporučena kombinace antiemetik podle Apfela skórovacího systému.
2.2.11	**Epidurální analgezie**<br>Hrudní epidurální analgezie (TEA) je doporučena u otevřených výkonů. U laparoskopických výkonů TEA doporučena není. U pacientů s kontraindikací k epidurální analgezii, rizikem renálního selhání a koagulopatií je doporučena blokáda břišní stěny (TAP blok) nebo jiné koanalgetické techniky.
2.2.12	**Minimálně invazivní přístup**<br>Doporučen je minimálně invazivní přístup.
2.2.13	**Operační technika**<br>Přešiti staplerové linie či aplikace tkáňového lepidla neovlivňují riziko dehiscence a není doporučeno je rutinně provádět.
2.2.14	**Kalibrace žaludku u sleeve gastrektomie (tubulizace žaludku)**<br>U vertikální (sleeve) gastrektomie je doporučena kalibrace žaludku silnou sondou zavedenou jícnem do žaludku a umístěnou na stranu malé křiviny žaludku.

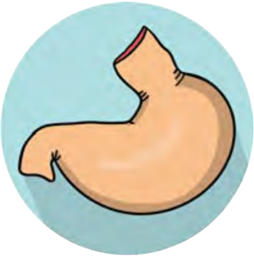

EUPEMEN PROTOKOL

BARIATRICKÁ CHIRURGIE

2.2.15	**Použití nazogastrické sondy**<br>Preventivní použití nazogastrické sondy není doporučeno. Pokud je nazogastrická sonda zavedena během operace, má být odstraněna před ukončením anestezie.
2.2.16	**Použití břišního drénu**<br>Drenáž břišní dutiny nemá být rutinně prováděna.
2.3	Časná pooperační fáze<br>Anesteziolog, Zdravotní sestra
2.3.1	**Udržení normotermie**<br>U všech pacientů je doporučena monitorace tělesné teploty a aktivní ohřev pacienta k udržení normotermie.
2.3.2	**Multimodální opioidy šetřící analgezie**<br>U všech pacientů je doporučena multimodální opioidy šetřící analgezie s kontrolou bolesti do VAS 3.
2.3.3	**Časný perorální příjem**<br>Časný perorální příjem má být zahájen čirými tekutinami 6 hodin po operaci.
2.3.4	**Časná mobilizace**<br>Sed na lůžku je doporučen 3 hodiny po operaci, chůze 6 hodin po operaci s respektováním nočního klidu pro spánek.
2.3.5	**Antitrombotická profylaxe**<br>Je doporučena aplikace nízkomolekulárního heparinu 12 hodin po operaci.
2.3.6	**Prevence pooperační nevolnosti a zvracení**<br>Je doporučena kombinace antiemetik podle Apfela skórovacího systému.
2.3.7	**Terapie spánkové apnoe**<br>U pacientů se syndromem spánkové apnoe je doporučena neinvazivní ventilační podpora s kontinuálním přetlakem v dýchacích cestách (CPAP) ihned po operaci.
3	Pooperační den 1<br>(Standardní oddělení)<br>Chirurg, Zdravotní sestra
3.1	**Perorální příjem**<br>Tekutá nízkokalorická dieta dle tolerance pacienta.
3.2	**Časná mobilizace**<br>Plná mobilizace.
3.3	**Multimodální opioidy šetřící analgezie**<br>U všech pacientů je doporučena multimodální opioidy šetřící analgezie s kontrolou bolesti do VAS 3.

EUPEMEN European Perioperative Medical Networking
Co-funded by the Erasmus+ Programme of the European Union
Cátedra GERM Medicina Perioperatoria Universidad Zaragoza

3.4	**Ukončení infuzní terapie**<br>Ukončení infuzní terapie u pacientů tolerujících perorální příjem.
3.5	**Odstranění permanentního močového katetru**<br>Odstranění permanentního močového katetru, pokud byl zaveden.
3.6	**Odstranění břišních drénů**<br>Odstranění břišních drénů, pokud byly zavedeny.
3.7	**Antitrombotická profylaxe**
3.8	**Dechová rehabilitace**
4	Pooperační den 2 (a následující)<br>(Standardní oddělení)<br><br>Chirurg, Zdravotní sestra
4.1	**Perorální příjem**<br>Tekutá nízkokalorická dieta / Tekutá nízkokalorická dieta s vyšším obsahem proteinů.
4.2	**Odstranění břišních drénů**<br>Odstranění břišních drénů, pokud byly zavedeny.
4.3	**Zhodnocení kritérií propuštění pacienta**<br>Pacient bez komplikace či s komplikací zvládnutelnou ambulantně, bez septických projevů, kontrola bolesti perorálními analgetiky, plná mobilizace, tolerance perorálního příjmu a souhlas pacienta.
5	Propuštění<br><br>Chirurg, Zdravotní sestra, Primární péče
5.1	**Dieta**<br>První dva týdny je doporučena nízkokalorická kašovitá dieta, poté postupně realimentace šetřící dietou po dobu dalších dvou týdnů.
5.2	**Péče o operační ránu**<br>Péče o ránu, odstranění stehů / kožních klipů dle zvyklostí pracoviště.
5.3	**Rehabilitace**<br>Měsíc od provedení operace je doporučeno zahájit aerobní a silový trénink s progresivním zatěžováním pod dohledem odborníka.
5.4	**Antitrombotická profylaxe**<br>Je doporučeno pokračovat s antitrombotickou profylaxí 3 až 4 týdny po operaci.
5.5	**Follow-up**<br>Telefonický kontakt po propuštění. Další péče ve spolupráci s praktickým lékařem.

EUPEMEN European Perioperative Medical Networking

Co-funded by the Erasmus+ Programme of the European Union

Cátedra GERM Medicina Perioperatoria Universidad Zaragoza

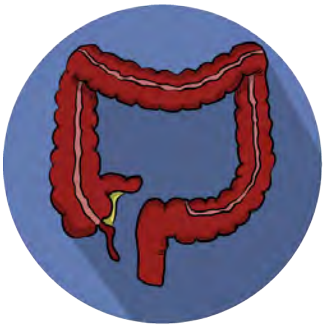

EUPEMEN PROTOKOL

RESEKCE TLUSTÉHO STŘEVA

1	**Přednemocniční fáze**<br>Anesteziolog, Chirurg, Zdravotní sestra, Nutriční terapeut, Stomická sestra
1.1	**Edukace pacienta**<br>Cílem edukace je poskytnout pacientovi komplexní informace o operaci a obvyklém průběhu hospitalizace včetně podmínek propuštění. Součástí edukace je získání podepsaného informovaného souhlasu.
1.2	**Předoperační vyšetření**<br>Předoperační vyšetření zahrnuje klinické vyšetření pacienta, RTG hrudníku, laboratoř (koagulace, krevní obraz, biochemie včetně CRP) a EKG.
1.3	**Zhodnocení stařecké křehkosti**<br>U pacientů starších 65 let je doporučeno stanovit skóre stařecké křehkosti (Frailty Index, Frail-VIG index).
1.4	**Klasifikace fyzického stavu nemocného podle ASA**
1.5	**Apfel skóre**<br>U každého pacienta je doporučeno zhodnotit riziko vzniku pooperační nevolnosti a zvracení podle Apfela skórovacího systému.
1.6	**Kompenzace chronických onemocnění**<br>Doporučena je optimalizace chronických komorbidit, u nemocných s kardiovaskulárním onemocněním je doporučeno kardiologické vyšetření.
1.7	**Předoperační vyšetření u diabetiků**<br>U všech pacientů je doporučena kontrola hladiny glukózy v krvi a kontrola glykovaného hemoglobinu (HbA1c). U nedostatečně kompenzovaných diabetiků a pacientů s nově diagnostikovaným diabetem je doporučeno vyšetření diabetologem, kompenzace stavu a výkon až v druhé době.
1.8	**Screening a korekce anémie**<br>Sideropenická anémie má být korigována pomocí preparátů intravenózního železa.
1.9	**Optimalizace nutričního stavu**<br>Zhodnocení nutričního stavu je doporučeno provádět rutinně u všech pacientů prostřednictvím screeningových dotazníků, například dotazník MUST (Malnutrition Universal Screening Tool). U nutričně rizikových pacientů je doporučena nutriční podpora orálními nutričními suplementy 7 dní před operací a 5 dní po operaci.
1.10	**Zanechání kouření a redukce konzumace alkoholu**<br>U všech pacientů je doporučeno zanechání kouření a snížení konzumace alkoholu alespoň po dobu jednoho měsíce před operací.
1.11	**Prehabilitace**<br>Je doporučen aerobní a silový trénink přizpůsobený fyzické zdatnosti pacienta.

EUPEMEN European Perioperative Medical Networking
Co-funded by the Erasmus+ Programme of the European Union
Cátedra GERM Medicina Perioperatoria Universidad Zaragoza

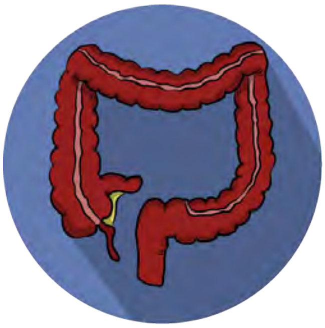

EUPEMEN PROTOKOL

RESEKCE TLUSTÉHO STŘEVA

1.12	**Bezezbytková dieta alespoň 5 dní před operací.**
1.13	**Absence mechanické přípravy střeva kromě případů s plánem provedení perioperační kolonoskopie.**
1.14	**Očistné klyzma**<br>Dvakrát aplikace očistného klyzmatu odpoledne před operací (výkony na levé polovině tračníku).
2	Perioperační fáze
2.1	Časná předoperační fáze<br>(Přijetí pacienta v den operace)<br>Anesteziolog, Chirurg, Zdravotní sestra, Nutriční terapeut, Stomická sestra
2.1.1	**Předoperační hygiena**<br>Sprcha / koupel pacienta den nebo ráno před výkonem.
2.1.2	**Farmakologická profylaxe tromboembolické nemoci**<br>Je doporučena aplikace nízkomolekulárního heparinu 2-12 hodin před operací v závislosti, zda je plánována neuroaxiální anestezie či ne.
2.1.3	**Mechanická profylaxe tromboembolické nemoci**<br>Je doporučena mechanická profylaxe kompresivními punčochami nebo pomocí intermitentní pneumatické komprese dle individuálních rizik pacienta.
2.1.4	**Sacharidový nápoj**<br>Je doporučeno podání sacharidového nápoje (12,5 % roztok maltodextrinu) v objemu 800 ml večer před operací a 400 ml 2 hodiny před úvodem do anestezie, pokud není přítomna žádná kontraindikace. U diabetiků je doporučeno podání sacharidového nápoje s antidiabetickou medikací.
2.1.5	**Předoperační lačnění**<br>Je doporučeno podávání lehké stravy do 6 hodin a čirých tekutin do 2 hodin před operací.
2.1.6	**Příprava operačního pole**<br>Odstranění ochlupení při přípravě operačního pole je doporučeno elektrickým strojkem, nikoliv jednorázovou žiletkou.
2.1.7	**Zakreslení stomie (pokud plánována)**
2.1.8	**Antibiotická profylaxe**<br>Intravenózní antibiotická profylaxe má být aplikována u všech pacientů v intervalu 30-60 minut před provedením incize. Pokud délka operace přesáhne dva poločasy eliminace léku, je doporučeno intraoperačně opakovat profylaktické podání antibiotika.

EUPEMEN European Perioperative Medical Networking
Co-funded by the Erasmus+ Programme of the European Union
Cátedra GERM Medicina Perioperatoria Universidad Zaragoza

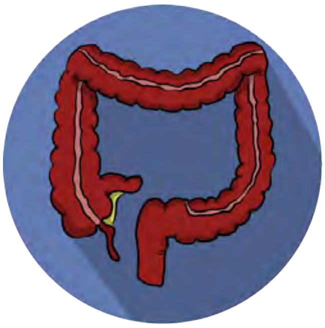

EUPEMEN PROTOKOL

RESEKCE TLUSTÉHO STŘEVA

2.2	Intraoperační fáze<br>Anesteziolog, Chirurg, Zdravotní sestra
2.2.1	**WHO Surgical Safety Checklist**
2.2.2	**Rutinní intraoperační monitorace**<br>Během výkonu je doporučeno provádět rutinně monitoraci vitálních funkcí, monitoraci hloubky celkové anestezie, monitoraci nervosvalové blokády a monitoraci glykémie.
2.2.3	**Minimálně invazivní přístup**<br>Doporučen je minimálně invazivní přístup. Intraabdominální tlak nemá překročit 12 mmHg.
2.2.4	**Derivace moči**<br>Rutinní použití permanentního močového katetru není doporučeno.
2.2.5	**Arteriální katetr**<br>Rutinní použití arteriálního katetru se nedoporučuje.
2.2.6	**Centrální žilní katetr**<br>Rutinní použití centrálního žilního katetru se nedoporučuje.
2.2.7	**Úvod a vedení anestezie**<br>Pro úvod a vedení anestezie je doporučeno použití krátkodobě působících anestetik.
2.2.8	**Oxygenace**<br>U všech pacientů je doporučena oxygenace s frakcí kyslíku více než 50 %.
2.2.9	**Tekutinová terapie**<br>U pacientů s vysokým operačním rizikem a pacientů podstupující operaci spojenou s velkou krevní ztrátou je doporučena cílená tekutinová terapie pomocí neinvazivní hemodynamické monitorace. U ostatních pacientů je doporučen restriktivní tekutinový režim dle ideální váhy pacienta balancovanými roztoky rychlostí 1-3 ml/kg/h (laparoskopie) nebo 3-5 ml/kg/h (laparotomie). Krevní ztráta má být hrazena koloidy v poměru 1:1.
2.2.10	**Použití nazogastrické sondy**<br>Preventivní použití nazogastrické sondy není doporučeno. Pokud je nazogastrická sonda zavedena během operace, má být odstraněna před ukončením anestezie.
2.2.11	**Prevence hypotermie**<br>Je doporučen aktivní ohřev pacienta pomocí přikrývek či podložek s proudícím teplým vzduchem a ohřev infuzí.
2.2.12	**Prevence pooperační nevolnosti a zvracení**<br>Je doporučena kombinace antiemetik podle Apfela skórovacího systému.

EUPEMEN European Perioperative Medical Networking
Co-funded by the Erasmus+ Programme of the European Union
Cátedra GERM Medicina Perioperatoria Universidad Zaragoza

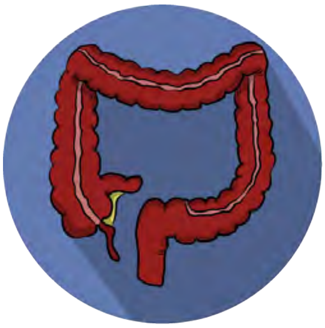

EUPEMEN PROTOKOL

RESEKCE TLUSTÉHO STŘEVA

2.2.13	**Epidurální analgezie**<br>Hrudní epidurální analgezie (TEA) je doporučena u otevřených výkonů. U laparoskopických výkonů TEA doporučena není. U pacientů s kontraindikací k epidurální analgezii, rizikem renálního selhání a koagulopatií je doporučena blokáda břišní stěny (TAP blok) nebo jiné koanalgetické techniky.
2.2.14	**Adjuvantní intravenózní analgetika**<br>Doporučeny jsou nesteroidní protizánětlivé léky, lidokain, ketamin, magnezium sulfát a dexmedetomidin.
2.2.15	**Perioperační kontrola glykémie**<br>U diabetiků je doporučena perioperační kontrola glykémie dle standardu nemocnice. U pacientů s rizikem rozvoje inzulinové rezistence (věk nad 65 let, obezita, operace trvající déle než 1 hodina) by hladina glykémie neměla překročit 10 mmol/l.
2.2.16	**Dezinfekce operačního pole**<br>Doporučen je alkoholový roztok Chlorhexidine 2 %. Dezinfekce kůže se provádí od středu operačního pole na jeho periferii.
2.2.17	**Použití břišního drénu**<br>Drenáž břišní dutiny nemá být rutinně prováděna.
2.3	Časná pooperační fáze<br>(JIP / Intermediární jednotka)<br><br>Anesteziolog, Zdravotní sestra
2.3.1	**Udržení normotermie**<br>U všech pacientů je doporučena monitorace tělesné teploty a aktivní ohřev pacienta k udržení normotermie.
2.3.2	**Multimodální opioidy šetřící analgezie**<br>U všech pacientů je doporučena multimodální opioidy šetřící analgezie s kontrolou bolesti do VAS 3.
2.3.3	**Restriktivní tekutinový režim**
2.3.4	**Časný perorální příjem**<br>Časný perorální příjem má být zahájen čirými tekutinami 6 hodin po operaci.
2.3.5	**Dechová rehabilitace**
2.3.6	**Časná mobilizace**<br>Sed na lůžku je doporučen 3 hodiny po operaci, chůze 8 hodin po operaci s respektováním nočního klidu pro spánek.
2.3.7	**Antitrombotická profylaxe**<br>Je doporučena aplikace nízkomolekulárního heparinu 12 hodin po operaci.

EUPEMEN European Perioperative Medical Networking
Co-funded by the Erasmus+ Programme of the European Union
Cátedra GERM Medicina Perioperatoria Universidad Zaragoza

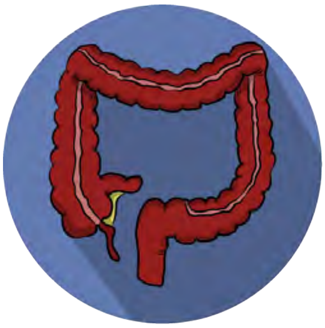

EUPEMEN PROTOKOL

RESEKCE TLUSTÉHO STŘEVA

2.3.8	**Prevence pooperační nevolnosti a zvracení**<br>Je doporučena kombinace antiemetik podle Apfela skórovacího systému.
2.3.9	**Pokračování v oxygenaci frakcí kyslíku 0,5 po dobu 2 hodin po operaci**
2.3.10	**Perioperační kontrola glykémie**<br>U diabetiků je doporučena perioperační kontrola glykémie dle standardu nemocnice. U pacientů s rizikem rozvoje inzulinové rezistence (věk nad 65 let, obezita, operace trvající déle než 1 hodina) by hladina glykémie neměla překročit 10 mmol/l.
3	Pooperační den 1<br>(Standardní oddělení)<br><br>Chirurg, Zdravotní sestra, Stomická sestra
3.1	**Časný perorální příjem**<br>Tekutá dieta / Kašovitá dieta dle tolerance pacienta.
3.2	**Ukončení infuzní terapie**<br>Ukončení infuzní terapie u pacientů tolerujících perorální příjem.
3.3	**Časná mobilizace**<br>Mobilizace pacienta po pokoji (z lůžka na křeslo).
3.4	**Multimodální opioidy šetřící analgezie**<br>U všech pacientů je doporučena multimodální opioidy šetřící analgezie s kontrolou bolesti do VAS 3.
3.5	**Odstranění permanentního močového katetru**<br>Odstranění permanentního močového katetru, pokud byl zaveden.
3.6	**Odstranění břišních drénů**<br>Odstranění břišních drénů, pokud byly zavedeny.
3.7	**Dechová rehabilitace**
3.8	**Antitrombotická profylaxe**
3.9	**Prevence pooperační nevolnosti a zvracení**<br>Je doporučena kombinace antiemetik podle Apfela skórovacího systému.
3.10	**Antiulcerogenní profylaxe**
3.11	**Perioperační kontrola glykémie**<br>U diabetiků je doporučena perioperační kontrola glykémie dle standardu nemocnice. U pacientů s rizikem rozvoje inzulinové rezistence (věk nad 65 let, obezita, operace trvající déle než 1 hodina) by hladina glykémie neměla překročit 10 mmol/l.

EUPEMEN European Perioperative Medical Networking
Co-funded by the Erasmus+ Programme of the European Union
Cátedra GERM Medicina Perioperatoria Universidad Zaragoza

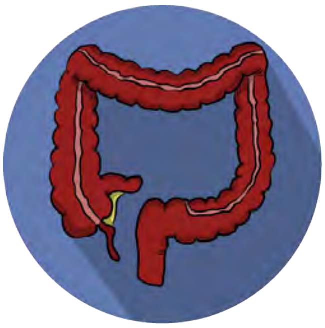

EUPEMEN PROTOKOL

RESEKCE TLUSTÉHO STŘEVA

3.12	**Edukace péče o stomii (pokud přítomna)**
3.13	**Laboratoř**<br>Kontrolní laboratoř včetně CRP.
4	Pooperační den 2<br><br>Chirurg, Zdravotní sestra, Stomická sestra
4.1	**Časný perorální příjem**<br>Kašovitá dieta / Šetřící dieta dle tolerance pacienta.
4.2	**Ukončení infuzní terapie**<br>Ukončení infuzní terapie u pacientů tolerujících perorální příjem.
4.3	**Časná mobilizace**<br>Procházky na krátké vzdálenosti.
4.4	**Multimodální opioidy šetřící analgezie**<br>U všech pacientů je doporučena multimodální opioidy šetřící analgezie s kontrolou bolesti do VAS 3 (preference pouze perorálních analgetik).
4.5	**Odstranění permanentního močového katetru**<br>Odstranění permanentního močového katetru, pokud nebyl již odstraněn.
4.6	**Dechová rehabilitace**
4.7	**Antitrombotická profylaxe**
4.8	**Prevence pooperační nevolnosti a zvracení**
4.9	**Antiulcerogenní profylaxe**
4.10	**Perioperační kontrola glykémie**
4.11	**Pokračování v edukaci stomickou sestrou (pokud stomie přítomna)**
4.12	**Laboratoř**<br>Kontrolní laboratoř včetně CRP.
5	Pooperační den 3<br><br>Chirurg, Zdravotní sestra
5.1	**Časný perorální příjem**<br>Šetřící dieta dle tolerance pacienta.

EUPEMEN European Perioperative Medical Networking
Co-funded by the Erasmus+ Programme of the European Union
Cátedra GERM Medicina Perioperatoria Universidad Zaragoza

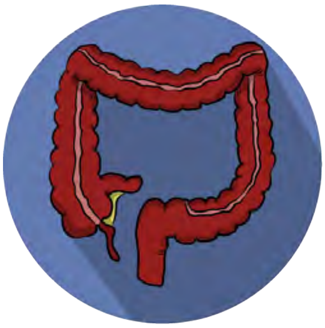

5.2	**Časná mobilizace**<br>Plná mobilizace.
5.3	**Perorální analgetika**
5.4	**Odstranění žilního katetru**
5.5	**Dechová rehabilitace**
5.6	**Antitrombotická profylaxe**
5.7	**Perioperační kontrola glykémie**
5.8	**Laboratoř**<br>Kontrolní laboratoř včetně CRP.
5.9	**Zhodnocení kritérií propuštění pacienta**<br>Pacient bez komplikace či s komplikací zvládnutelnou ambulantně, bez septických projevů, kontrola bolesti perorálními analgetiky, plná mobilizace, tolerance perorálního příjmu a souhlas pacienta.
6	Propuštění<br>Chirurg, Zdravotní sestra, Primární péče
6.1	**Dokumentace pacienta**<br>Při propuštění má být připravena propouštěcí zpráva a předána do rukou pacienta. V propouštěcí zprávě má být popsán průběh hospitalizace a doporučen další postup péče.
6.2	**Antitrombotická profylaxe**<br>Doporučena je prodloužená farmakologická profylaxe po dobu 28 dní od operace.
6.3	**Follow-up**<br>První týden po propuštění telefonický kontakt s pacientem nebo kontrola na chirurgické ambulanci. Další kontroly za 1, 3 a 6 měsíců. Ve spolupráci s praktickým lékařem zajištění domácí péče podle potřeb pacienta.

EUPEMEN European Perioperative Medical Networking

Co-funded by the Erasmus+ Programme of the European Union

Cátedra GERM Medicina Perioperatoria Universidad Zaragoza

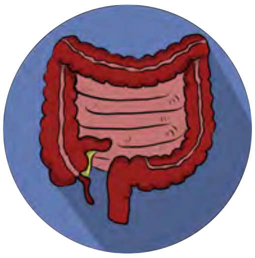

EUPEMEN PROTOKOL

STŘEVNÍ OBSTRUKCE

1	Předoperační fáze<br><br>Anesteziolog, Chirurg
1.1	**Předoperační vyšetření**<br>Klinické vyšetření, nativní RTG břicha a laboratoř včetně CRP.
1.2	**Klinické skórovací systémy**<br>U geriatrických pacientů je doporučeno stanovit skóre stařecké křehkosti: modifikovaný FI (Frailty Index) nebo Frail-VIG Index. K prevenci deliria u pacientů starších 65 let je doporučeno posouzení Beerových kritérií.
1.3	**Normotermie**<br>U křehkých pacientů je v předoperační fázi doporučen aktivní ohřev pomocí přikrývek s proudícím teplým vzduchem.
1.4	**Derivace moči**<br>Rutinní použití permanentního močového katetru není doporučeno.
1.5	**Perioperační kontrola glykémie**<br>U diabetiků je doporučena perioperační kontrola glykémie dle standardu nemocnice. U pacientů s rizikem rozvoje inzulinové rezistence (věk nad 65 let, obezita, operace trvající déle než 1 hodina) by hladina glykémie neměla překročit 10 mmol/l.
1.6	**Antibiotická profylaxe**<br>Antibiotická profylaxe je doporučena u všech pacientů. Volba antibiotika závisí na místním doporučení antibiotického střediska.
1.7	**Nazogastrická sonda**<br>Zavedení nazogastrické sondy je doporučeno u všech u pacientů.
1.8	**Prevence infekce v místě operačního výkonu (IMOV)**<br>Soubor perioperačních opatření k prevenci IMOV (care bundle) je doporučen u všech pacientů.
1.9	**Edukace pacienta**<br>Cílem edukace je poskytnout pacientovi komplexní informace o operaci a obvyklém průběhu hospitalizace včetně podmínek propuštění. Součástí edukace je získání podepsaného informovaného souhlasu.
2	Perioperační fáze
2.1	Intraoperační fáze<br><br>Anesteziolog, Chirurg, Zdravotní sestra
2.1.1	**WHO Surgical Safety Checklist**

EUPEMEN European Perioperative Medical Networking

Co-funded by the Erasmus+ Programme of the European Union

Cátedra GERM Medicina Perioperatoria Universidad Zaragoza

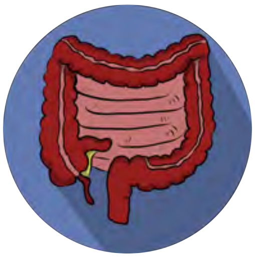

EUPEMEN PROTOKOL

STŘEVNÍ OBSTRUKCE

2.1.2	**Rutinní intraoperační monitorace**
2.1.3	**Operační přístup**<br>Minimálně invazivní přístup je doporučen pouze vybraných případech v závislosti na zkušenosti operatéra. Ve většině případů je preferován otevřený přístup.
2.1.4	**Bleskový úvod do anestezie (crush úvod)**<br>K minimalizaci aspirace je u všech pacientů doporučeno provedení bleskového úvodu do anestezie.
2.1.5	**Perioperační oxygenace**<br>Je doporučeno udržovat frakci kyslíku mezi 0,6 až 0,8.
2.1.6	**Tekutinová terapie**<br>Doporučena je cílená tekutinová terapie pomocí neinvazivní hemodynamické monitorace. Pokud není monitorace k dispozici, je doporučeno podání balancovaných roztoků rychlostí 1-3 ml/kg/h (laparoskopie) nebo 3-5 ml/kg/h (laparotomie).
2.1.7	**Derivace moči**<br>Rutinní použití permanentního močového katetru není doporučeno.
2.1.8	**Udržení normotermie**<br>Je doporučen aktivní ohřev pacienta pomocí přikrývek či podložek s proudícím teplým vzduchem a ohřev infuzí.
2.1.9	**Perioperační kontrola glykémie**<br>U diabetiků je doporučena perioperační kontrola glykémie dle standardu nemocnice. U pacientů s rizikem rozvoje inzulinové rezistence (věk nad 65 let, obezita, operace trvající déle než 1 hodina) by hladina glykémie neměla překročit 10 mmol/l.
2.1.10	**Epidurální analgezie**<br>Epidurální analgezie je doporučena u otevřených výkonů.
2.1.11	**Prevence pooperační nevolnosti a zvracení**<br>Je doporučena kombinace antiemetik podle Apfela skórovacího systému.
2.1.12	**Použití břišního drénu**<br>Drenáž břišní dutiny nemá být rutinně prováděna.
2.1.13	**Antitrombotická profylaxe**<br>Je doporučena mechanická profylaxe kompresivními punčochami nebo pomocí intermitentní pneumatické komprese v kombinaci s farmakologickou profylaxí nízkomolekulárním heparinem dle standardu nemocnice.
2.1.14	**Prevence infekce v místě operačního výkonu (IMOV)**<br>Soubor perioperačních opatření k prevenci IMOV (care bundle) je doporučen u všech pacientů.

EUPEMEN European Perioperative Medical Networking
Co-funded by the Erasmus+ Programme of the European Union
Cátedra GERM Medicina Perioperatoria Universidad Zaragoza

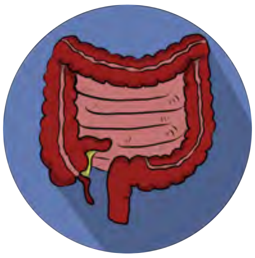

EUPEMEN PROTOKOL

STŘEVNÍ OBSTRUKCE

2.2	Časná pooperační fáze<br><br>Anesteziolog, Chirurg, Zdravotní sestra
2.2.1	**Udržení normotermie**<br>U všech pacientů je doporučena monitorace tělesné teploty a aktivní ohřev pacienta k udržení normotermie.
2.2.2	**Oxygenace**<br>U všech pacientů je doporučeno monitorovat hladinu saturace kyslíkem k prevenci hyposaturace.
2.2.3	**Multimodální opioidy šetřící analgezie**<br>U všech pacientů je doporučena multimodální opioidy šetřící analgezie s kontrolou bolesti do VAS 3.
2.2.4	**Restriktivní tekutinová terapie**
2.2.5	**Perioperační kontrola glykémie**<br>U diabetiků je doporučena perioperační kontrola glykémie dle standardu nemocnice. U pacientů s rizikem rozvoje inzulinové rezistence (věk nad 65 let, obezita, operace trvající déle než 1 hodina) by hladina glykémie neměla překročit 10 mmol/l.
2.2.6	**Časná mobilizace**<br>Vertikalizace pacienta je doporučena 2 hodiny po operaci, chůze 8 hodin po operaci s respektováním nočního klidu pro spánek.
2.2.7	**Nazogastrická sonda**<br>Dle klinického stavu pacienta odstranění nazogastrické sondy 12 hodin po operaci.
2.2.8	**Odstranění permanentního močového katetru**<br>Dle klinického stavu pacienta odstranění permanentního močového katetru 12 hodin po operaci.
2.2.9	**Antitrombotická profylaxe**<br>Je doporučena mechanická profylaxe kompresivními punčochami nebo pomocí intermitentní pneumatické komprese v kombinaci s farmakologickou profylaxí nízkomolekulárním heparinem dle standardu nemocnice.
3	Pooperační den 1<br>(Standardní oddělení)<br><br>Chirurg, Zdravotní sestra
3.1	**Perioperační kontrola glykémie**<br>U diabetiků je doporučena perioperační kontrola glykémie dle standardu nemocnice. U pacientů s rizikem rozvoje inzulinové rezistence (věk nad 65 let, obezita, operace trvající déle než 1 hodina) by hladina glykémie neměla překročit 10 mmol/l.

EUPEMEN European Perioperative Medical Networking
Co-funded by the Erasmus+ Programme of the European Union
Cátedra GERM Medicina Perioperatoria Universidad Zaragoza

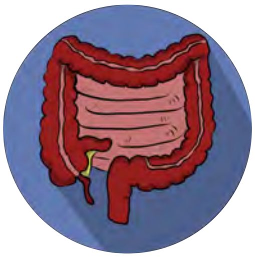

EUPEMEN PROTOKOL

STŘEVNÍ OBSTRUKCE

3.2	**Časná mobilizace**<br>Plná mobilizace.
3.3	**Dechová rehabilitace**
3.4	**Antibiotická terapie**<br>Antibiotická terapie je doporučena v případě bakteriální translokace nebo kontaminace dutiny břišní. Volba širokospektrého antibiotika závisí na místním doporučení antibiotického střediska.
3.5	**Multimodální opioidy šetřící analgezie**<br>U všech pacientů je doporučena multimodální opioidy šetřící analgezie s kontrolou bolesti do VAS 3.
3.6	**Odstranění nazogastrické sondy**<br>Dle klinického stavu pacienta odstranění nazogastrické sondy Při odstranění nazogastrické sondy nasazení tekuté diety.
3.7	**Odstranění permanentního močového katetru**
3.8	**Odstranění epidurálního katetru**<br>Odstranění epidurálního katetru, pokud byl zaveden.
3.9	**Antitrombotická profylaxe**<br>Je doporučena mechanická profylaxe kompresivními punčochami nebo pomocí intermitentní pneumatické komprese v kombinaci s farmakologickou profylaxí nízkomolekulárním heparinem dle standardu nemocnice.
4	Pooperační den 2<br>Chirurg, Zdravotní sestra
4.1	**Perioperační kontrola glykémie**<br>U diabetiků je doporučena perioperační kontrola glykémie dle standardu nemocnice. U pacientů s rizikem rozvoje inzulinové rezistence (věk nad 65 let, obezita, operace trvající déle než 1 hodina) by hladina glykémie neměla překročit 10 mmol/l.
4.2	**Časná mobilizace**<br>Plná mobilizace.
4.3	**Dechová rehabilitace**
4.4	**Multimodální opioidy šetřící analgezie**<br>U všech pacientů je doporučena multimodální opioidy šetřící analgezie s kontrolou bolesti do VAS 3.
4.5	**Odstranění nazogastrické sondy**<br>Dle klinického stavu pacienta odstranění nazogastrické sondy Při odstranění nazogastrické sondy nasazení tekuté diety.

EUPEMEN European Perioperative Medical Networking
Co-funded by the Erasmus+ Programme of the European Union
Cátedra GERM Medicina Perioperatoria Universidad Zaragoza

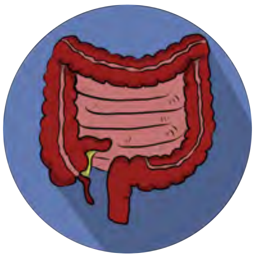

EUPEMEN PROTOKOL

STŘEVNÍ OBSTRUKCE

4.6	**Antitrombotická profylaxe**<br>Je doporučena mechanická profylaxe kompresivními punčochami nebo pomocí intermitentní pneumatické komprese v kombinaci s farmakologickou profylaxí nízkomolekulárním heparinem dle standardu nemocnice.
4.7	**Časné propuštění pacienta**<br>Zhodnocení kritérií propuštění v případě, že nebyla provedena resekce střeva.
5	Pooperační den 3<br>Chirurg, Zdravotní sestra
5.1	**Časný perorální příjem**
5.2	**Časná mobilizace**
5.3	**Dechová rehabilitace**
5.4	**Antitrombotická profylaxe**
5.5	**Zhodnocení kritérií propuštění pacienta**
6	Propuštění<br>Chirurg, Zdravotní sestra, Primární péče
6.1	**Antitrombotická profylaxe**<br>Pokračování v antitrombotické profylaxi je doporučeno dle individuálních rizik pacienta.
6.2	**Antibiotická terapie**<br>Pokračování v perorální antibiotické terapii dle stavu pacientu.
6.3	**Kontrola laboratoře**<br>Pokles CRP minimálně o 50% při propuštění.
6.4	**Follow-up**<br>Telefonický kontakt do 24 hodin od propuštění. Kontrola na ambulanci dle zvyklostí pracoviště. Další péče ve spolupráci s praktickým lékařem.
6.5	**Kritéria propuštění**<br>Pacient bez komplikace či s komplikací zvládnutelnou ambulantně, bez septických projevů, obnovena střevní pasáž, kontrola bolesti perorálními analgetiky, souhlas pacienta.

EUPEMEN European Perioperative Medical Networking
Co-funded by the Erasmus+ Programme of the European Union
Cátedra GERM Medicina Perioperatoria Universidad Zaragoza

EUPEMEN PROTOKOL

AKUTNÍ APENDICITIDA

1	**Předoperační fáze**<br>Anesteziolog, Chirurg
1.1	**Předoperační vyšetření**<br>Klinické vyšetření, ultrazvuk břicha a laboratoř včetně CRP.
1.2	**Klinické skórovací systémy**<br>V rámci diagnostiky je doporučeno stanovit AAS (Adult Appendicits Score) a AIRS (Appendicitis Inflammatory Response Score) skóre. U geriatrických pacientů je doporučeno stanovit skóre stařecké křehkosti: modifikovaný FI (Frailty Index) nebo Frail-VIG Index. K prevenci deliria u pacientů starších 65 let je doporučeno posouzení Beerových kritérií.
1.3	**Normotermie**<br>U křehkých pacientů je v předoperační fázi doporučen aktivní ohřev pomocí přikrývek s proudícím teplým vzduchem.
1.4	**Derivace moči**<br>Rutinní použití permanentního močového katetru není doporučeno.
1.5	**Perioperační kontrola glykémie**<br>U diabetiků je doporučena perioperační kontrola glykémie dle standardu nemocnice. U pacientů s rizikem rozvoje inzulinové rezistence (věk nad 65 let, obezita, operace trvající déle než 1 hodina) by hladina glykémie neměla překročit 10 mmol/l.
1.6	**Antibiotická profylaxe**<br>Antibiotická profylaxe je doporučena u všech pacientů. Volba antibiotika závisí na místním doporučení antibiotického střediska.
1.7	**Prevence infekce v místě operačního výkonu (IMOV)**<br>Soubor perioperačních opatření k prevenci IMOV (care bundle) je doporučen u všech pacientů.
1.8	**Edukace pacienta**<br>Cílem edukace je poskytnout pacientovi komplexní informace o operaci a obvyklém průběhu hospitalizace včetně podmínek propuštění. Součástí edukace je získání podepsaného informovaného souhlasu.
2	**Perioperační fáze**
2.1	**Intraoperační fáze**<br>Anesteziolog, Chirurg, Zdravotní sestra
2.1.1	**WHO Surgical Safety Checklist**

EUPEMEN
European Perioperative Medical Networking
Co-funded by the Erasmus+ Programme of the European Union
Cátedra GERM Medicina Perioperatoria
Universidad Zaragoza

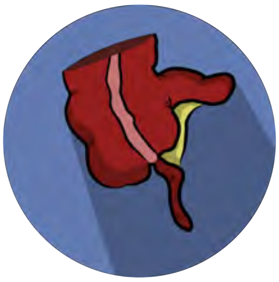

EUPEMEN PROTOKOL

AKUTNÍ APENDICITIDA

2.1.2	**Rutinní intraoperační monitorace**
2.1.3	**Operační přístup**<br>U všech pacientů je doporučen minimálně invazivní přístup.
2.1.4	**Bleskový úvod do anestezie (crush úvod)**<br>K minimalizaci aspirace je u všech pacientů doporučeno provedení bleskového úvodu do anestezie.
2.1.5	**Perioperační oxygenace**<br>Je doporučeno udržovat frakci kyslíku mezi 0,6 až 0,8.
2.1.6	**Tekutinová terapie**<br>Doporučena je cílená tekutinová terapie pomocí neinvazivní hemodynamické monitorace. Pokud není monitorace k dispozici, je doporučeno podání balancovaných roztoků rychlostí 1-3 ml/kg/h (laparoskopie) nebo 3-5 ml/kg/h (laparotomie).
2.1.7	**Derivace moči**<br>Rutinní použití permanentního močového katetru není doporučeno.
2.1.8	**Použití nazogastrické sondy**<br>Preventivní použití nazogastrické sondy není doporučeno.
2.1.9	**Udržení normotermie**<br>Je doporučen aktivní ohřev pacienta pomocí přikrývek či podložek s proudícím teplým vzduchem a ohřev infuzí.
2.1.10	**Perioperační kontrola glykémie**<br>U diabetiků je doporučena perioperační kontrola glykémie dle standardu nemocnice. U pacientů s rizikem rozvoje inzulinové rezistence (věk nad 65 let, obezita, operace trvající déle než 1 hodina) by hladina glykémie neměla překročit 10 mmol/l.
2.1.11	**Prevence pooperační nevolnosti a zvracení**<br>Je doporučena kombinace antiemetik podle Apfela skórovacího systému.
2.1.12	**Použití břišního drénu**<br>Drenáž břišní dutiny nemá být rutinně prováděna.
2.1.13	**Multimodální opioidy šetřící analgezie**<br>U všech pacientů je doporučena multimodální opioidy šetřící analgezie, v případě indikace v kombinaci s infiltrací laparoskopických portů lokálním anestetikem nebo blokádou břišní stěny (TAP blok).
2.1.14	**Antitrombotická profylaxe**<br>Je doporučena mechanická profylaxe kompresivními punčochami nebo pomocí intermitentní pneumatické komprese v kombinaci s farmakologickou profylaxí nízkomolekulárním heparinem dle standardu nemocnice.
2.1.15	**Prevence infekce v místě operačního výkonu (IMOV)**

EUPEMEN
European Perioperative Medical Networking
Co-funded by the Erasmus+ Programme of the European Union
Cátedra GERM Medicina Perioperatoria
Universidad Zaragoza

	Soubor perioperačních opatření k prevenci IMOV (care bundle) je doporučen u všech pacientů.
2.2	Časná pooperační fáze<br><br>Anesteziolog, Chirurg, Zdravotní sestra
2.2.1	**Udržení normotermie**<br>U všech pacientů je doporučena monitorace tělesné teploty a aktivní ohřev pacienta k udržení normotermie.
2.2.2	**Oxygenace**<br>U všech pacientů je doporučeno monitorovat hladinu saturace kyslíkem k prevenci hyposaturace.
2.2.3	**Multimodální opioidy šetřící analgezie**
2.2.4	**Restriktivní tekutinová terapie**
2.2.5	**Perioperační kontrola glykémie**<br>U diabetiků je doporučena perioperační kontrola glykémie dle standardu nemocnice. U pacientů s rizikem rozvoje inzulinové rezistence (věk nad 65 let, obezita, operace trvající déle než 1 hodina) by hladina glykémie neměla překročit 10 mmol/l.
2.2.6	**Časná mobilizace**<br>Vertikalizace pacienta je doporučena 2 hodiny po operaci, chůze 8 hodin po operaci s respektováním nočního klidu pro spánek.
2.2.7	**Časný perorální příjem**<br>Časný perorální příjem má být zahájen čirými tekutinami 4 hodiny po operaci.
2.2.8	**Antitrombotická profylaxe**<br>Je doporučena mechanická profylaxe zahrnující kompresivní punčochy do plné mobilizace pacienta v kombinaci s farmakologickou profylaxí nízkomolekulárním heparinem dle standardu nemocnice.
2.2.9	**Antibiotická terapie**<br>Terapeutické podání antibiotika je doporučeno u komplikovaných apendicitid. Volba antibiotika dle zvyklostí pracoviště.
3	Pooperační den 1<br>(Standardní oddělení)<br><br>Chirurg, Zdravotní sestra
3.1	**Perorální příjem**<br>Kašovitá dieta.

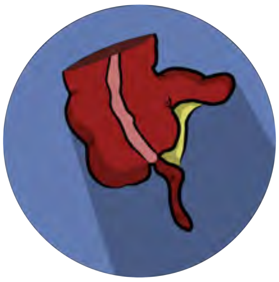

3.2	**Časná mobilizace**<br>Plná mobilizace.
3.3	**Dechová rehabilitace**
3.4	**Perorální analgezie**<br>Ke kontrole bolesti jsou doporučena perorální neopioidní analgetika.
3.5	**Ukončení infuzní terapie**<br>Ukončení infuzní terapie u pacientů tolerujících perorální příjem.
3.6	**Antitrombotická profylaxe**<br>Je doporučena mechanická profylaxe zahrnující kompresivní punčochy do plné mobilizace pacienta v kombinaci s farmakologickou profylaxí nízkomolekulárním heparinem dle standardu nemocnice.
4	Pooperační den 2<br><br>Chirurg, Zdravotní sestra
4.1	**Perorální příjem**<br>Kašovitá dieta / Šetřící dieta.
4.2	**Časná mobilizace**<br>Plná mobilizace.
4.3	**Perorální analgezie**<br>Ke kontrole bolesti jsou doporučena perorální neopioidní analgetika.
4.4	**Ukončení infuzní terapie**<br>Ukončení infuzní terapie u pacientů tolerujících perorální příjem.
4.5	**Antitrombotická profylaxe**<br>Je doporučena mechanická profylaxe zahrnující kompresivní punčochy do plné mobilizace pacienta v kombinaci s farmakologickou profylaxí nízkomolekulárním heparinem dle standardu nemocnice.
4.6	**Časné propuštění pacienta**<br>Zhodnocení kritérií propuštění pacienta.
5	Další dny hospitalizace<br><br>Chirurg, Zdravotní sestra
5.1	**Perorální realimentace**

EUPEMEN European Perioperative Medical Networking

Co-funded by the Erasmus+ Programme of the European Union

Cátedra GERM Medicina Perioperatoria Universidad Zaragoza

5.2	**Mobilizace**
5.3	**Dechová rehabilitace**
5.4	**Perorální analgezie**
5.5	**Antibiotická terapie**
5.6	**Antitrombotická terapie**
5.7	**Zhodnocení kritérií propuštění pacienta**
6	Propuštění<br>Chirurg, Zdravotní sestra, Primární péče
6.1	**Antitrombotická profylaxe**<br>Pokračování v antitrombotické profylaxi je doporučeno dle individuálních rizik pacienta.
6.2	**Antibiotická terapie**<br>Pokračování v perorální antibiotické terapii dle stavu pacientu.
6.3	**Kontrola laboratoře**<br>Pokles CRP minimálně o 50% při propuštění.
6.4	**Follow-up**<br>Telefonický kontakt do 24 hodin od propuštění. Kontrola na ambulanci dle zvyklostí pracoviště. Další péče ve spolupráci s praktickým lékařem.
6.5	**Kritéria propuštění**<br>Pacient bez komplikace či s komplikací zvládnutelnou ambulantně, bez septických projevů, kontrola bolesti perorálními analgetiky, souhlas pacienta.

EUPEMEN European Perioperative Medical Networking

Co-funded by the Erasmus+ Programme of the European Union

Cátedra GERM Medicina Perioperatoria Universidad Zaragoza
