# Supplementary material for: Advancing abdominal surgery recovery implementation: a unified framework for intensified recovery protocols by the EUropean PErioperative MEdical Networking collaborative
Source: Front Surg. 2026 May 18;13:1827678. doi: 10.3389/fsurg.2026.1827678 (PMC13223102; doi:10.3389/fsurg.2026.1827678)
Supplement: Supplementary file 13 [file Datasheet4.pdf]

Co-funded by the
Erasmus+ Programme
of the European Union

EUPEMEN
European Perioperative Medical Networking

EUPEMEN PROTOCOLOS (ES)

iisa Instituto de Investigación Sanitaria Aragón

SERVIZIO SANITARIO REGIONALE
EMILIA-ROMAGNA
Azienda Unità Sanitaria Locale di Ferrara

2. LÉKAŘSKÁ FAKULTA
UNIVERZITA KARLOVA

UNIVERSITAS
Miguel Hernández

ΓΕΝΙΚΟ ΝΟΣΟΚΟΜΕΙΟ ΘΕΣΣΑΛΟΝΙΚΗΣ
"Γ. ΠΑΠΑΝΙΚΟΛΑΟΥ"

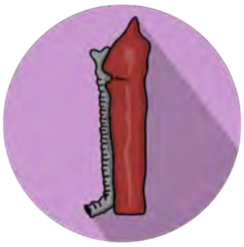

PROTOCOLO EUPEMEN

ESOFAGUECTOMÍA

1	Previo al Ingreso
1.1	**Primera Visita en Consultas**<br>Enfermería, cirugía
1.1.1	Historia clínica y solicitud pruebas complementarias
1.1.2	Con resultados, presentación del caso en el Comité Multidisciplinar de Tumores
1.1.3	Abandono de hábitos tóxicos (tabaco, alcohol, etc) al menos 4-6 semanas antes de la cirugía
1.2	**Segunda Visita en Consultas**<br>Enfermería, cirugía, anestesiología, prehabilitación, psicología, especialidades
1.2.1	Información completa de proceso asistencial a paciente y familiares
1.2.2	Prehabilitación, incluyendo:<br>- Evaluación del estado físico y fragilidad y fisioterapia funcional y respiratoria<br>- Evaluación y soporte psicológico<br>- Cribado, valoración del estado nutricional (Test MUST) y optimización del mismo
1.2.3	En caso de afagia: ingreso y optimización nutricional. Valorar medidas y vías para administración de nutrición artificial según protocolo de cada centro
1.2.4	En disfagia a sólidos: dieta líquida con suplementos proteico-calóricos
1.2.5	Evaluación y optimización de comorbilidades
1.2.6	Evaluación y tratamiento del déficit de hierro y anemia preoperatoria
1.2.7	Escala Apfel (valoración de náuseas y vómitos intraoperatorios)
1.2.8	Evaluación anestésica (ASA). Recomendar valoración odontológica si boca séptica
1.2.9	Firma de consentimientos informados. Entrega de documentación

EUPEMEN European Perioperative Medical Networking
Co-funded by the Erasmus+ Programme of the European Union
Cátedra GERM Medicina Perioperatoria Universidad Zaragoza

PROTOCOLO EUPEMEN

ESOFAGUECTOMÍA

1.3	**Día previo a la Intervención**<br><br>(Si es posible programar el ingreso el mismo día de la cirugía)<br><br>Enfermería
1.3.1	Lista de verificación al ingreso
1.3.2	Iniciar profilaxis tromboembólica según protocolo del centro
1.3.3	Ayuno a sólidos 6 horas y a líquidos claros 2 horas previas a la cirugía
1.3.4	Preparación intestinal en el caso de coloplastia
1.3.5	Baño completo antes de la intervención
2	**Perioperatorio**
2.1	**Preoperatorio Inmediato**<br><br>Enfermería, anestesiología
2.1.1	Lista de verificación preoperatoria
2.1.2	Administración oral de 200-400 ml de una bebida que contenga 25-50 gr de carbohidratos hasta dos horas antes de la intervención, si no existe contraindicación
2.1.3	Colocación de medias compresivas o compresión neumática intermitente, según riesgo tromboembólico y protocolo de cada centro
2.1.4	**Premedicación:**<br>- Profilaxis antibiótica (según protocolo de cada centro)<br>- Si retraso vaciado gástrico: medidas profilácticas de regurgitación según protocolo
2.2	**Intraoperatorio**<br><br>Enfermería, anestesiología, cirugía
2.2.1	Lista de verificación de quirófano

2.2.2	Prevención de la hipotermia. Calentamiento activo y de fluidos
2.2.3	Limpieza de la zona quirúrgica con clorhexidina alcohólica al 2% (si piel intacta) o acuosa si afectación cutánea o mucosas
2.2.4	Monitorización estándar, profundidad anestésica, relajación muscular y temperatura central
2.2.5	Canalización arterial invasiva
2.2.6	Colocación de catéter venoso central
2.2.7	Fluidoterapia guiada por objetivos (FGO), evitando la sobrecarga de fluidos
2.2.8	Profilaxis náuseas y vómitos según escala Apfel
2.2.9	Si cirugía minimamente invasiva (CMI) (recomendable): Valoración individualizada de colocación de catéter epidural torácico T6-T8 combinada con analgesia multimodal ahorradora de opioides
2.2.10	Si cirugía abierta: Colocación de catéter epidural torácico T6-T8 y analgesia multimodal ahorradora de opioides
2.2.11	Uso de agentes anestésicos de acción corta. Valorar relajación muscular profunda
2.2.12	Estrategias ventilatorias:<br>- Ventilación unipulmonar en cirugía abierta y laparoscópica<br>- Valorar ventilación bipulmonar en CMI-posición prona (según experiencia)
2.2.13	Maniobras de reclutamiento alveolar al menos antes de la ventilación unipulmonar y tras ésta<br>- Ventilación de protección pulmonar<br>- Utilizar FiO_2 necesaria para niveles normales de oxígeno en sangre arterial
2.2.14	Extubación precoz
2.2.15	Sondaje vesical: valorar su retirada a las 24-48 horas
2.2.16	SNG: valorar su retirada a las 24-48 horas y en función del débito
2.2.17	Drenajes abdominales y cervicales: No uso sistemático

EUPEMEN European Perioperative Medical Networking

Co-funded by the Erasmus+ Programme of the European Union

Cátedra GERM Medicina Perioperatoria Universidad Zaragoza

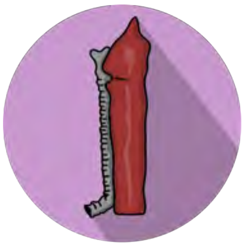

2.2.18	Drenaje torácico: emplear un único drenaje
2.2.19	Valorar colocación catéter alimentación enteral (yeyunostomía, sonda nasoyeyunal …)
2.3	Postoperatorio Inmediato<br><br>Unidad de Reanimación. Valorar Unidad de Cuidados Intermedios en casos seleccionados<br><br>Enfermería, anestesiología, cirugía
2.3.1	Vigilar signos de alerta*
2.3.2	Dieta absoluta
2.3.3	Valorar nutrición enteral (NE) por sonda yeyunal (inicio a las 6 horas de la intervención)<br>- 10 cc/por hora las primeras 8 horas postoperatorias<br>- 20 cc/h las 8-16 h postoperatorias<br>- 30 cc/h las 16-24 h postoperatorias
2.3.4	Nutrición Parenteral Total (NPT) por catéter central hasta cubrir requerimientos por vía enteral
2.3.5	Fluidoterapia intravenosa restrictiva
2.3.6	Inicio de movilización (sentar en cama) a partir de las 6-8 horas tras cirugía
2.3.7	Asegurar buen control del dolor (EVA <3): analgesia combinada
2.3.8	Fisioterapia respiratoria y funcional
2.3.9	Respetar el descanso nocturno
2.3.10	Mantenimiento de FiO_2 0,5% 2 horas tras finalizar la intervención
2.3.11	Profilaxis tromboembólica
2.3.12	Tratamiento de náuseas y vómitos

EUPEMEN European Perioperative Medical Networking

Co-funded by the Erasmus+ Programme of the European Union

Cátedra GERM Medicina Perioperatoria Universidad Zaragoza

2.3.13	**Pruebas control**:<br>- Gasometría arterial<br>- Radiografía de tórax
3	1º Día Postoperatorio<br><br>Unidad de Reanimación<br>Valorar Unidad de Cuidados Intermedios en casos seleccionados<br><br>Enfermería, anestesiología, cirugía
3.1	Dieta absoluta.
3.2	Progresar NE por sonda yeyunal (no superar los 40 ml/h)
3.3	NPT por catéter central
3.4	Fluidoterapia intravenosa restrictiva
3.5	Movilización: sedestación
3.6	Valorar retirada de sondaje vesical
3.7	Asegurar buen control del dolor (EVA <3): analgesia combinada
3.8	Fisioterapia respiratoria y funcional
3.9	Oxigenoterapia según necesidades
3.10	Profilaxis tromboembólica según protocolo de cada centro
3.11	**Pruebas control**:<br>- Analítica sanguínea, incluyendo PCR y procalcitonina<br>- Gasometría arterial<br>- Radiografía de tórax
4	2º Día Postoperatorio<br><br>Unidad de Reanimación- Unidad de Cuidados Intermedios - Sala de Hospitalización<br><br>Enfermería, anestesiología, cirugía

4.1	Dieta absoluta. Valorar inicio de tolerancia oral
4.2	Progresar NE por sonda yeyunal (No superar 100 ml/h.)
4.3	NPT por catéter central
4.4	Fluidoterapia intravenosa restrictiva
4.5	Movilización: inicio de deambulación.
4.6	Asegurar buen control del dolor (EVA <3): analgesia combinada
4.7	Fisioterapia respiratoria y funcional
4.8	Retirada de sondaje vesical
4.9	Retirada de SNG
4.10	Profilaxis tromboembólica
4.11	**Pruebas control**:<br>- Analítica sanguínea incluyendo PCR y procalcitonina<br>- Radiografía de tórax
5	3º Día Postoperatorio<br>Sala de hospitalización<br><br>Enfermería, cirugía
5.1	Dieta líquida / gelatinas / espesante
5.2	Progresar NE por sonda yeyunal (No superar 100 ml /h.)
5.3	Si no se emplea NE: NPT por catéter central
5.4	Valorar retirada de fluidoterapia
5.5	Movilización: deambulación progresiva

EUPEMEN European Perioperative Medical Networking

Co-funded by the Erasmus+ Programme of the European Union

Cátedra GERM Medicina Perioperatoria Universidad Zaragoza

5.6	Valorar retirada de catéter epidural: previo control de coagulación en estado correcto.
5.7	Asegurar buen control del dolor (EVA <3): analgesia combinada
5.8	Fisioterapia respiratoria y funcional
5.9	Valorar retirar drenajes torácicos en anastomosis cervical si incisión cervical normal, radiografía con expansión pulmonar y débito menor de 200 ml/24h y sin productos patológicos (valorar solicitud de estudio bioquímico y recuento celular)
5.10	Profilaxis tromboembólica
5.11	**Pruebas control**:<br>- Analítica sanguínea incluyendo PCR y procalcitonina<br>- Radiografía de tórax
6	4º Día Postoperatorio<br><br>Sala de hospitalización<br><br>Enfermería, cirugía
6.1	Dieta líquida / gelatinas / espesante / purés claros
6.2	NE por sonda (No superar 100 ml /h.)
6.3	Si no se emplea NE: NPT por catéter central
6.4	Movilización: deambulación progresiva.
6.5	Asegurar buen control del dolor (EVA <3): analgesia intravenosa.
6.6	Estimular inspiraciones profundas y el uso de incentivador respiratorio.
6.7	Valorar retirar drenajes torácicos en anastomosis torácica si no hay signos de alerta, TEG sin fuga, radiografía con expansión pulmonar y débito menor de 200 ml/24h y sin productos patológicos (valorar solicitud de estudio bioquímico y recuento celular)

EUPEMEN European Perioperative Medical Networking

Co-funded by the Erasmus+ Programme of the European Union

Cátedra GERM Medicina Perioperatoria Universidad Zaragoza

6.8	Fisioterapia respiratoria y funcional
6.9	Profilaxis tromboembólica
6.10	**Pruebas control**:<br>- Analítica sanguínea incluyendo PCR y procalcitonina<br>- Radiografía de tórax<br>- Valorar realización de tránsito esofagogástrico (TEG) (en anastomosis torácica)
7	5º Día Postoperatorio<br>Sala de hospitalización<br>Enfermería, cirugía
7.1	Progresar dieta (puré, yogurt…)
7.2	Reducir flujo de NE
7.3	Si no se emplea NE: NPT por catéter central
7.4	Movilización: deambulación progresiva
7.5	Asegurar buen control del dolor (EVA <3): analgesia intravenosa.
7.6	Fisioterapia respiratoria y funcional
7.7	Profilaxis tromboembólica
7.8	**Pruebas control**:<br>- Analítica sanguínea incluyendo PCR y procalcitonina
8	6º Día Postoperatorio<br>Sala de hospitalización<br>Enfermería, cirugía
8.1	Progresar dieta. Dieta túrmix

EUPEMEN European Perioperative Medical Networking

Co-funded by the Erasmus+ Programme of the European Union

Cátedra GERM Medicina Perioperatoria Universidad Zaragoza

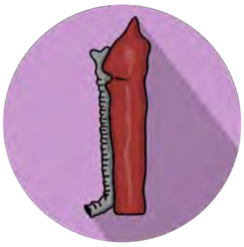

8.2	Retirar NE y NPT
8.3	Movilización: deambulación progresiva
8.4	Asegurar buen control del dolor (EVA <3): analgesia oral
8.5	Fisioterapia respiratoria y funcional
8.6	Profilaxis tromboembólica
8.7	**Pruebas control**:<br>- Analítica sanguínea incluyendo PCR y procalcitonina
9	7º Día Postoperatorio<br>Sala de hospitalización<br>Enfermería, cirugía
9.1	Valoración de posible ALTA si cumple los siguientes criterios: No complicaciones quirúrgicas, no fiebre, dolor controlado con analgesia oral, deambulación, tolerancia oral y aceptación por parte del paciente
9.2	Dieta túrmix / fácil masticación
9.3	Movilización: deambulación progresiva
9.4	Asegurar buen control del dolor (EVA <3): analgesia oral
9.5	Fisioterapia respiratoria y funcional
9.6	Profilaxis tromboembólica
9.7	**Pruebas control**:<br>- Analítica sanguínea incluyendo PCR y procalcitonina
10	Al Alta y Seguimiento<br>Enfermería, cirugía, psicología, especialidades, etención primaria
10.1	Entrega de documentación:<br>- Hoja informativa y de recomendaciones al alta

	- Educación y recomendaciones dietéticas<br>- Hoja de satisfacción
10.2	Valorar control telefónico tras el alta (según protocolo de cada centro)
10.3	Control por su médico de atención primaria
10.4	Control y seguimiento en consultas de Cirugía y otras especialidades si precisa
10.5	Valorar aportes calóricos, proteicos, minerales y vitamínicos según necesidades
10.6	Valorar control en consultas de Psicología. Evaluación calidad de vida postquirúrgica

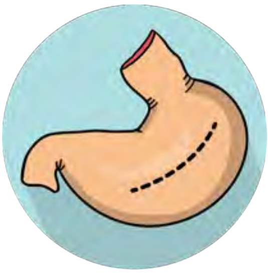

PROTOCOLO EUPEMEN

GASTECTOMÍA

1	Previo al Ingreso
1.1.1	Primera Visita en Consultas<br>Enfermería, cirugía
1.1.2	Historia clínica y solicitud pruebas complementarias
1.1.3	Con resultados, presentación del caso en el Comité Multidisciplinar de Tumores
1.1.4	Abandono de hábitos tóxicos (tabaco, alcohol, etc) al menos 4-6 semanas antes de la cirugía
1.2	Segunda Visita en Consultas<br>Enfermería, cirugía, anestesiología, prehabilitación, psicología, especialidades
1.2.1	Información completa de proceso asistencial a paciente y familiares
1.2.2	Prehabilitación, incluyendo:<br>- Evaluación del estado físico y fragilidad y fisioterapia funcional y respiratoria<br>- Evaluación y soporte psicológico<br>- Cribado, valoración del estado nutricional (Test MUST) y optimización del mismo
1.2.3	En caso de afagia: ingreso y optimización nutricional. Valorar medidas y vías para administración de nutrición artificial según protocolo de cada centro
1.2.4	En disfagia a sólidos: dieta líquida con suplementos proteico-calóricos
1.2.5	Evaluación y optimización de comorbilidades
1.2.6	Evaluación y tratamiento del déficit de hierro y anemia preoperatoria
1.2.7	Escala Apfel (valoración de náuseas y vómitos intraoperatorios)
1.2.8	Evaluación anestésica (ASA). Recomendar valoración odontológica si boca séptica
1.2.9	Firma de consentimientos informados. Entrega de documentación

EUPEMEN European Perioperative Medical Networking
Co-funded by the Erasmus+ Programme of the European Union
Cátedra GERM Medicina Perioperatoria Universidad Zaragoza

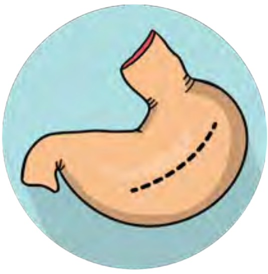

PROTOCOLO EUPEMEN

GASTECTOMÍA

1.3	**Día previo a la Intervención**<br>(Si es posible programar el ingreso el mismo día de la cirugía)<br>Enfermería
1.3.1	Lista de verificación al ingreso
1.3.2	Iniciar profilaxis tromboembólica según protocolo del centro
1.3.3	Ayuno a sólidos 6 horas y a líquidos claros 2 horas previas a la cirugía
1.3.4	Baño completo antes de la intervención
2	**Perioperatorio**
2.1	**Preoperatorio Inmediato**<br>Enfermería, anestesiología
2.1.1	Lista de verificación preoperatoria
2.1.2	Administración oral de 200-400 ml de una bebida que contenga 25-50 gr de carbohidratos hasta dos horas antes de la intervención, si no existe contraindicación
2.1.3	Colocación de medias compresivas o compresión neumática intermitente, según riesgo tromboembólico y protocolo de cada centro
2.1.4	**Premedicación:**<br>- Profilaxis antibiótica (según protocolo de cada centro)<br>- Si retraso vaciado gástrico: medidas profilácticas de regurgitación según protocolo
2.2	**Intraoperatorio**<br>Enfermería, anestesiología, cirugía
2.2.1	Lista de verificación de quirófano

EUPEMEN European Perioperative Medical Networking
Co-funded by the Erasmus+ Programme of the European Union
Cátedra GERM Medicina Perioperatoria Universidad Zaragoza

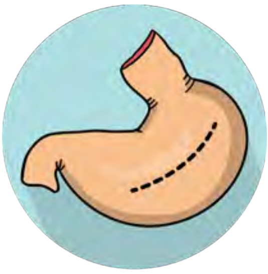

PROTOCOLO EUPEMEN

GASTECTOMÍA

2.2.2	Prevención de la hipotermia. Calentamiento activo y de fluidos
2.2.3	Limpieza de la zona quirúrgica con clorhexidina alcohólica al 2% (si piel intacta) o acuosa si afectación cutánea o mucosas
2.2.4	Monitorización estándar, profundidad anestésica, relajación muscular y temperatura central
2.2.5	Canalización arterial invasiva
2.2.6	No uso sistemático de catéter venoso central, salvo si previsión de NPT
2.2.7	Fluidoterapia guiada por objetivos (FGO), evitando la sobrecarga de fluidos
2.2.8	Profilaxis náuseas y vómitos según escala Apfel
2.2.9	Si cirugía minimamente invasiva (CMI) (preferiblemente): Valoración individualizada de colocación de catéter epidural torácico T6-T8 combinada con analgesia multimodal ahorradora de opioides
2.2.10	Si cirugía abierta: Colocación de catéter epidural torácico T6-T8 y analgesia multimodal ahorradora de opioides
2.2.11	Uso de agentes anestésicos de acción corta. Valorar relajación muscular profunda
2.2.12	Extubación precoz
2.2.13	Sondaje vesical: valorar su retirada a las 24 horas
2.2.14	SNG: si se coloca, retirar al finalizar la intervención
2.2.15	No uso sistemático de drenajes abdominales
2.3	**Postoperatorio Inmediato**<br><br>Unidad de Reanimación. Valorar Unidad de Cuidados Intermedios en casos seleccionados – Sala de hospitalización<br><br>Enfermería, anestesiología, cirugía

EUPEMEN European Perioperative Medical Networking

Co-funded by the Erasmus+ Programme of the European Union

Cátedra GERM Medicina Perioperatoria Universidad Zaragoza

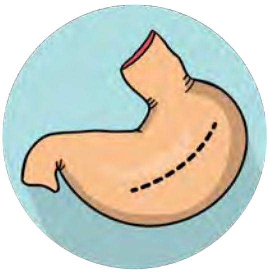

2.3.1	Inicio de la tolerancia oral a partir de las 6-8 horas tras la cirugía
2.3.2	Fluidoterapia intravenosa restrictiva
2.3.3	Valoración del dolor: EVA (conseguir nivel de dolor 0-4)
2.3.4	Inicio de movilización (cama-sillón) a las 6 horas tras cirugía
2.3.5	Asegurar buen control del dolor (EVA <3): analgesia combinada
2.3.6	Fisioterapia respiratoria y funcional
2.3.7	Respetar descanso nocturno
2.3.8	Mantenimiento de FiO_2 0,5% 2 horas tras finalizar la intervención
2.3.9	Fisioterapia respiratoria
2.3.10	Profilaxis tromboembólica
2.3.11	Tratamiento de náuseas y vómitos
3	**1º Día Postoperatorio**<br>Sala de hospitalización<br>Enfermería, anestesiología, cirugía
3.1	Dieta líquida / gelatinas
3.2	NPT hasta completar requerimientos por vía oral
3.3	Fluidoterapia intravenosa restrictiva
3.4	Movilización: cama / sillón / deambulación

EUPEMEN European Perioperative Medical Networking

Co-funded by the Erasmus+ Programme of the European Union

Cátedra GERM Medicina Perioperatoria Universidad Zaragoza

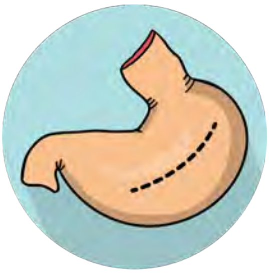

PROTOCOLO EUPEMEN

GASTECTOMÍA

3.5	Asegurar buen control del dolor (EVA <3): analgesia combinada
3.6	Retirar sondaje vesical
3.7	Fisioterapia respiratoria y funcional
3.8	Profilaxis tromboembólica
3.9	**Pruebas control**: Analítica sanguínea, incluyendo PCR y procalcitonina
4	2º Día Postoperatorio<br>Sala de hospitalización<br>Enfermería, anestesiología, cirugía
4.1	Progresar dieta (pures, yogurt)
4.2	Retirar fluidoterapia y funcional
4.3	Movilización: deambulación progresiva
4.4	Asegurar buen control del dolor (EVA <3): analgesia combinada
4.5	Retirar catéter epidural previo control de coagulación en estado correcto
4.6	Fisioterapia respiratoria y funcional
4.7	Profilaxis tromboembólica
5	3º Día Postoperatorio<br>Sala de hospitalización<br>Enfermería, cirugía
5.1	Dieta túrmix

EUPEMEN European Perioperative Medical Networking
Co-funded by the Erasmus+ Programme of the European Union
Cátedra GERM Medicina Perioperatoria Universidad Zaragoza

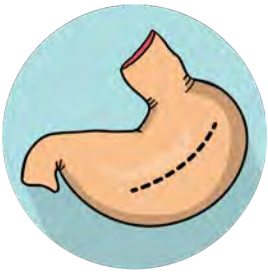

PROTOCOLO EUPEMEN

GASTECTOMÍA

5.2	Movilización: deambulación progresiva
5.3	Asegurar buen control del dolor (EVA <3): analgesia oral
5.4	Fisioterapia respiratoria y funcional
5.5	Profilaxis tromboembólica
5.6	**Pruebas control**: Analítica sanguínea incluyendo PCR y procalcitonina
6	4º Día Postoperatorio<br>Sala de hospitalización<br>Enfermería, cirugía
6.1	Valoración de posible ALTA si cumple los siguientes criterios: No complicaciones quirúrgicas, no fiebre, dolor controlado con analgesia oral, deambulación, tolerancia oral y aceptación por parte del paciente
6.2	Dieta blanda
6.3	Movilización: deambulación progresiva
6.4	Asegurar buen control del dolor (EVA <3): analgesia oral
6.5	Fisioterapia respiratoria y funcional
6.6	Profilaxis tromboembólica (mantener hasta 4 semanas tras la intervención)
6.7	Valorar solicitar analítica sanguínea incluyendo PCR y procalcitonina
7	Al Alta y Seguimiento<br>Enfermería, cirugía, psicología, especialidades, atención primaria
7.1	Entrega de documentación:<br>- Hoja informativa y de recomendaciones al alta<br>- Educación y recomendaciones dietéticas

	- Hoja de satisfacción
7.2	Valorar control telefónico tras el alta (según protocolo de cada centro)
7.3	Control por su médico de atención primaria
7.4	Control y seguimiento en consultas de Cirugía y otras especialidades si precisa
7.5	Valorar aportes calóricos, proteicos, minerales y vitamínicos según necesidades
7.6	Valorar control en consultas de Psicología. Evaluación calidad de vida postquirúrgica

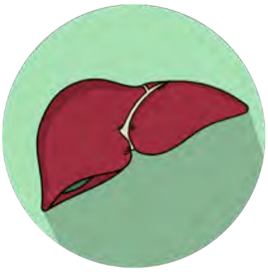

PROTOCOLO EUPEMEN

RESECCIONES HEPÁTICAS

1	Previo al Ingreso<br><br>Enfermería, cirugía, anestesiología, nutrición
1.2	Información completa de proceso asistencial a pacientes y familiares.
1.2	**VALORACIÓN DEL RIESGO ANESTÉSICO-QUIRÚRGICO.**<br>**Optimización nutricional, cardiológica, de anemia y comorbilidad**<br>Evaluación por cardiólogo si patología cardiaca activa de reciente aparición o descompensada<br>Evaluación nutricional:<br>-Test de Cribado nutricional (MUST)<br>Evaluación de la Diabetes Mellitus:<br>-glucemia y HbA1c<br>- si diabetes mal controlada o no diagnosticada previamente remitir a Atención Primaria y/o Endocrinología<br>Evaluación de la anemia (algoritmo manejo preoperatorio anemia)
1.3	Abandonar consumo de tabaco y alcohol al menos UN mes previo a la cirugía
1.4	Analítica Preoperatoria que incluya PCR
1.5	**Firma de Consentimientos Informados**
1.6	Prehabilitación respiratoria y entrenamiento aeróbico adaptado a la capacidad funcional del paciente.
2	Perioperatorio
2.1	Preoperatorio Inmediato<br><br>Si es posible, programar el ingreso el mismo día de la cirugía<br><br>Enfermería, cirugía, anestesiología
2.1.1	Ayuno de 6 horas para sólidos y 2 horas para líquidos claros
2.1.2	Suplemento de bebida carbohidratada 12,5% de maltodextrinas 400 cc / 12 horas (en caso de diabetes administrar junto con medicación antidiabética). Empezando la tarde previa.

EUPEMEN European Perioperative Medical Networking
Co-funded by the Erasmus+ Programme of the European Union
Cátedra GERM Medicina Perioperatoria Universidad Zaragoza

2.1.3	Recomendación fuerte de administración de HBPm 2-12h antes de la cirugía (en función de si se va a realizar anestesia neuroaxial o no).
2.1.4	Baño completo.
2.1.5	Rasurado con maquinilla eléctrica si éste es necesario.
2.1.6	Colocación de medias compresivas o de compresión neumática intermitente.
2.1.7	Administración profiláctica de antibiótico 30-60 min antes de la incisión quirúrgica. En procedimientos prolongados repetir dosis de acuerdo a la vida media de los fármacos.
2.1.8	Analgesia multimodal activa y/o preventiva.
2.2	Intraoperatorio<br>Enfermería, cirugía, anestesiología
2.2.1	Monitorización rutinaria:<br>- EKG<br>- Presión Arterial no Invasiva (PANI)<br>- Pulsioximetría (%Sat O2)<br>- FiO2<br>- Capnografía<br>- Temperatura<br>- Glucemia intraoperatoria<br>- Profundidad anestésica (BI)<br>- Bloqueo neuromuscular<br>- Sondaje vesical.
2.2.2	**Monitorización invasiva:**<br>- Canalización arterial invasiva NO de forma rutinaria (potencialmente en pacientes con alteraciones cardiorrespiratorias graves)<br>- Catéter venoso central *
2.2.3	Inducción y mantenimiento anestésico con agentes de acción corta
2.2.4	Fluidoterapia:<br>- Fase de resección: como objetivo de reducción de

EUPEMEN European Perioperative Medical Networking

Co-funded by the Erasmus+ Programme of the European Union

Cátedra GERM Medicina Perioperatoria Universidad Zaragoza

	sangrado se recomienda mantenimiento de PVC<=5 cmH2O con diuréticos del ASA y Manitol.<br>- En fase de reposición: se recomienda optimización hemodinámica mediante fluidoterapia guiada por objetivos con dispositivos validados.<br>- En caso de no disponer de éstos, serecomienda fluidoterapia restrictiva basada en peso ideal en perfusión continua solución balanceada (1-3ml/kg/h para laparoscopia; 3-5ml/kg/h para laparotomía).<br>- Reponer sangrado con coloides 1:1.
2.2.5	Evitar sonda nasogástrica de manera rutinaria.
2.2.6	Calentamiento activo con manta térmica y calentador de fluidos
2.2.7	Profilaxis de náuseas y vómitos postoperatorios según escala Apfel (según anexo RICA)
2.2.8	Analgesia:<br>- Epidural torácica en cirugía abierta.<br>- En cirugía laparoscópica no se recomienda de rutina.<br>- Pacientes con contraindicación para analgesia epidural, riesgo de fallo renal postoperatorio o de coagulopatía podrían beneficiarse de TAP bilateral u otras alternativas a la epidural.
2.2.9	Evitar niveles de glucemia > 180 mg/dl en paciente de riesgo de desarrollar insulinorresistencia (obesos, ancianos, larga duración quirúrgica)
2.2.10	Desinfección de la piel en círculo de limpio a sucio con clorhexidina en solución alcohólica al 1%
2.2.11	Cirugía mínimamente invasiva (siempre que sea posible)
2.2.12	Evitar drenajes.
2.2.13	** Valorar no poner CVC si resección menor y ausencia de factores de riesgo para insuficiencia renal postoperatoria (recomendable forzar diuresis de forma empírica).*
2.3	Postoperatorio Inmediato<br><br>Enfermería, anestesiología

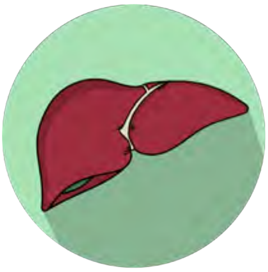

PROTOCOLO EUPEMEN

RESECCIONES HEPÁTICAS

2.3.1	Mantenimiento activo de temperatura.
2.3.2	Analgesia multimodal activa y/o preventiva. Restringir la administración de mórficos.
2.3.3	Fluidoterapia restrictiva.
2.3.4	Inicio de tolerancia oral a partir de 3 horas tras cirugía.
2.3.5	Inicio de movilización a partir de 3 horas tras cirugía.
2.3.6	Dieta líquida/blanda baja en residuo + Suplemento nutricional.
2.3.7	Profilaxis del TVP con HBPM 12 h tras la cirugía
3	1º Día Postoperatorio<br>Enfermería, cirugía
3.1	Suplementación nutricional hiperprotéico en pacientes con ingesta oral <60% de requerimientos energéticos o desnutrición preoperatoria.
3.2	Dieta blanda/normal
3.3	Fisioterapia respiratoria
3.4	Valorar retirada de drenajes, si existen
3.5	Analgesia endovenosa. Evitar la administración de mórficos.
3.6	Movilización activa (cama/sillón/inicio deambulación)
3.7	Si tolerancia oral correcta retirada de líquidos endovenosos.
3.8	Valorar la retirada de Sondaje vesical

EUPEMEN European Perioperative Medical Networking
Co-funded by the Erasmus+ Programme of the European Union
Cátedra GERM Medicina Perioperatoria Universidad Zaragoza

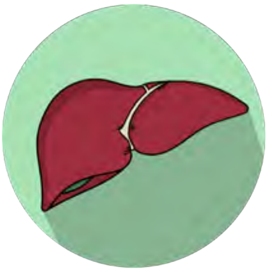

PROTOCOLO EUPEMEN

RESECCIONES HEPÁTICAS

3.9	Profilaxis NVPO. Profilaxis antiulcerosa.
3.10	Profilaxis tromboembólica
3.11	Valorar alta hospitalaria en cirugía laparoscópica.
**4**	**2º Día Postoperatorio**<br>Enfermería, cirugía
4.1	Dieta normal ± suplemento nutricional.
4.2	Analgesia oral ± epidural. NO Mórficos.
4.3	Movilización activa (deambulación).
4.4	Profilaxis de TVP: HBPm +Medias compresivas.
4.5	Valorar alta hospitalaria.
**5**	**3º Día Postoperatorio**<br>Enfermería, cirugía
5.1	Dieta normal ± suplemento nutricional.
5.2	Analgesia oral (AINES + Paracetamol/ Metamizol).
5.3	Movilización activa (deambulación).
5.4	Profilaxis de TVP: HBPm +Medias compresivas.
5.5	Analítica con PCR
5.6	Valorar alta hospitalaria.

EUPEMEN European Perioperative Medical Networking
Co-funded by the Erasmus+ Programme of the European Union
Cátedra GERM Medicina Perioperatoria Universidad Zaragoza

5.7	**VALORAR CRITERIOS DE ALTA**: No complicaciones quirúrgicas, no fiebre, dolor controlado con analgesia oral, deambulación completa, tolerancia oral correcta.
6	Al Alta<br>Enfermería, cirugía, Atención Primaria
6.1	Información personalizada, comprensible y completa
6.2	Control telefónico tras alta
6.3	Seguimiento al alta/continuidad asistencial: 1, 3 y 6 meses tras el alta
6.4	Apoyo domiciliario – Coordinación con Atención Primaria

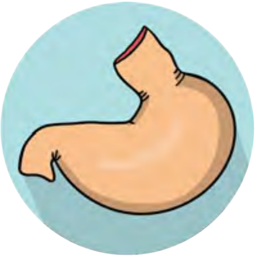

PROTOCOLO EUPEMEN

CIRUGÍA BARIÁTRICA

1	**Previo al Ingreso**<br>Enfermería, cirugía, anestesiología
1.1	**Información** oral y escrita completa al paciente
1.2	Evaluación cardiológica si > 3 FRCV
1.3	Screening de SAHS con el test STOP-BANG. Realizar polisomnografía si puntuación >3
1.4	Espirometría sólo si hay FR pulmonares
1.5	Analítica incluyendo: Hemograma, Coagulación, Bioquímica básica, Perfil Nutricional
1.6	Endoscopia Digestiva Alta y/o estudio esófago-gastroduodenal (si hay exclusión gástrica)
1.7	Screening de H.pylori y erradicación preoperatoria (si hay exclusión gástrica)
1.8	Suprimir tabaco y alcohol al menos 4-8 semanas antes de cirugía
1.9	Corrección de carencias nutricionales preoperatorias (clacio, hierro, vitamina D, Vitamina B12…)
1.10	Optimización preoperatoria de comorbilidades (DM, HTA…)
1.11	Se recomienda la realización de terapia prehabilitadora trimodal con el fin de mejorar la capacidad funcional previa a la intervención
1.12	**Prehabilitación física:** Incentivar ejercicios de fuerza muscular.
1.13	**Optimización nutricional:**<br>- **Pérdida de peso antes de la cirugia (dieta VLCD o productos comerciales)**<br>- **Valorar métodos coadyuvantes (fármacos, balón intragástrico**
2	**Perioperatorio**
2.1	**Preoperatorio Inmediato**

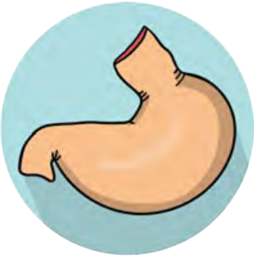

PROTOCOLO EUPEMEN

CIRUGÍA BARIÁTRICA

	Si es posible, programar el ingreso el mismo día de la cirugía<br><br>Enfermería, anestesiología
2.1.1	**Ayuno para líquidos claros 2h y para sólidos 8h**
2.1.2	**Profilaxis antitromboembólica (profilaxis farmacológica según Centro)**
2.1.3	**No añadir premedicación ansiolítica preoperatoria**
2.1.4	Suplemento de bebida carbohidratada (12.5% maltodextrinas 250 cc) 3 horas antes de la cirugía si no existe contraindicación
2.1.5	Colocación de medias compresivas o de compresión neumática intermitente, según riesgo tromboembólico
2.1.6	Evitar en la medida de lo posible la eliminación del vello
2.1.7	**Premedicación:**<br>- Profilaxis antibiótica 30-60 min antes de la incisión quirúrgica.<br>- Si retraso vaciado gástrico: medidas profilácticas de regurgitación.
2.2	Intraoperatorio<br><br>Enfermería, anestesiología, cirugía
2.2.1	Aplicación del listado de verificación quirúrgica (checklist)
2.2.2	Colocar dispositivos de compresión neumática intermitente
2.2.3	No anestesiar al paciente fuera de quirófano
2.2.4	Calentamiento activo con manta térmica y calentador de fluidos
2.2.5	Monitorización rutinaria, incluyendo capnografía, temperatura central, bloqueo neuromuscular, profundidad anestésica con índice biespectral y glucemia
2.2.6	**Monitorización invasiva NO indicada de rutina**

EUPEMEN European Perioperative Medical Networking
Co-funded by the Erasmus+ Programme of the European Union
Cátedra GERM Medicina Perioperatoria Universidad Zaragoza

2.2.7	**Catéter venoso central NO indicado de rutina**
2.2.8	Inducción anestésica en posición de rampa
2.2.9	Minimizar el tiempo entre inducción e intubación
2.2.10	**Uso de relajantes aminoesteroideos** como primera opción (si se dispone de Sugammadex)
2.2.11	Ventilación protectora pulmonar y maniobras de reclutamiento
2.2.12	FiO2: 0.5-0.6
2.2.13	Se recomienda optimización hemodinámica mediante **fluidoterapia guiada por objetivos** con dispositivos validados. En caso de no disponer de éstos, se recomienda fluidoterapia restrictiva basada en peso ideal
2.2.14	**Profilaxis NVPO según escala de Apfel modificada**
2.2.15	Analgesia epidural torácica a todos los pacientes sometidos a cirugía abierta. **En cirugía laparoscópica no se recomienda de rutina**. Pacientes con contraindicación para analgesia epidural podrían beneficiarse de TAP bilateral y/o i**nfiltrar trócares con anestésico local**
2.2.16	El abordaje debe ser laparoscópico siempre que sea posible
2.2.17	**Se recomienda realizar neumoperitoneo mediante aguja de Veress o inserción de trócar óptico**
2.2.18	**No hay evidencia para sobresutura, uso de fundas para grapadoras o pegamentos biológicos para disminuir las dehiscencias**
2.2.19	**La gastrectomía vertical debe calibrarse con sondas**
2.2.20	No sonda nasogástrica (sólo intraoperatoria para vaciar estómago)
2.2.21	No se recomienda dejar drenaje de forma rutinaria
2.3	Postoperatorio Inmediato<br>Enfermería, anestesiología

EUPEMEN European Perioperative Medical Networking

Co-funded by the Erasmus+ Programme of the European Union

Cátedra GERM Medicina Perioperatoria Universidad Zaragoza

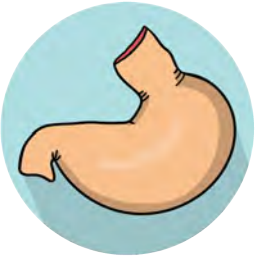

2.3.1	Mantenimiento activo de temperatura
2.3.2	Inicio de tolerancia oral a partir de 6 horas tras cirugía
2.3.3	Inicio de movilización a partir de 6 horas tras cirugía
2.3.4	Analgesia multimodal pautada según intervención. Mínima administración de mórficos. Valorar uso de coadyuvantes
2.3.5	En pacientes SAHS, reinstauración precoz de la CPAP
2.3.6	Control estricto de glucemia manteniendo niveles < 110 mg/dl en no diabéticos, y entre 110-150 mg/dl en diabéticos
**3**	**1º Día Postoperatorio**<br>Sala de hospitalización<br>Enfermería, cirugía
3.1	Dieta líquida hipocalórica según tolerancia
3.2	Movilización activa
3.3	Analgesia I.V.
3.4	Retirada fluidoterapia I.V. si buena tolerancia
3.5	Valorar retirada s. vesical, si la tuviese
3.6	Valorar retirada de drenajes, si existen
3.7	Tromboprofilaxis
3.8	Fisioterapia respiratoria
3.9	Control y cura de HQ

EUPEMEN European Perioperative Medical Networking

Co-funded by the Erasmus+ Programme of the European Union

Cátedra GERM Medicina Perioperatoria Universidad Zaragoza

3.10	Reservar los estudios de imagen para los casos con sospecha clínica de fuga anastomótica
4	2º Día Postoperatorio<br>(Y sucesivos)<br><br>Sala de hospitalización<br><br>Enfermería, cirugía
4.1	Dieta líquida completa hipocalórica o nutrición enteral completa hipocalórica hiperproteica
4.2	Valorar retirada drenajes, si existen
4.3	**Valorar alta domicilio:**<br>Criterios generales de alta: no complicaciones quirúrgicas, no fiebre, no taquicardia ni taquipnea, dolor controlado con analgesia, deambulación completa, adecuada tolerancia oral
5	Al Alta<br><br>Enfermería, cirugía
5.1	Dieta turmix hipocalórica o Nutrición oral completa hipocalórica hiperproteica primeras 1-2 semanas. Después dieta triturada 2 semanas. Dieta sólida tras 1-2 meses de cirugía
5.2	Tromboprofilaxis las primeras 3-4 semanas postoperatorias
5.3	Cura tópica HQ y retirada de puntos/grapas según protocolo.
5.4	Programa de ejercicio que combine entrenamiento aeróbico y de fuerza. Iniciar a partir de 1 mes tras la cirugía a intensidades moderadas progresando a intensidades más elevadas
5.5	**Control domiciliario:**<br>- Control telefónico tras alta<br>- Apoyo domiciliario coordinación con Atención Primaria

EUPEMEN European Perioperative Medical Networking

Co-funded by the Erasmus+ Programme of the European Union

Cátedra GERM Medicina Perioperatoria Universidad Zaragoza

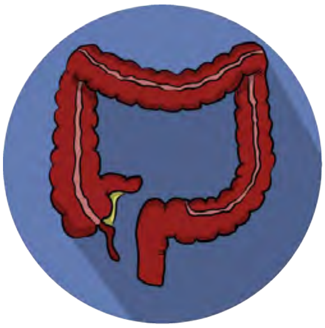

PROTOCOLO EUPEMEN

COLECTOMÍA

1	Previo al Ingreso<br><br>Anestesiología, Cirugía, Enfermería, Nutrición, Estomaterapia
1.1	Información oral y escrita completa al paciente
1.2	VALORACIÓN DEL RIESGO ANESTÉSICO-QUIRÚRGICO. Optimización nutricional, cardiológica, de anemia y comorbilidad<br>Evaluación por cardiólogo si patología cardiaca activa de reciente aparición o descompensada<br>Evaluación nutricional:<br>-Test de Cribado nutricional (MUST)<br>Evaluación de la Diabetes Mellitus:<br>-glucemia y HbA1c<br>- si diabetes mal controlada o no diagnosticada previamente remitir a Atención Primaria y/o Endocrinología<br>Evaluación de la anemia y ferropenia (algoritmo manejo preoperatorio anemia)<br>Evaluación de fragilidad (FRAIL)<br>Evaluación nivel ASA
1.3	Abandonar consumo de tabaco y alcohol al menos UN mes previo a la cirugía
1.4	Suministrar incentivo respiratorio
1.5	No preparación mecánica salvo colonoscopia perioperatoria y previsión de estoma
1.6	Recomendar dieta pobre en residuos (al menos 5 dias previos a la cirugía)
1.7	Valorar la realización de ejercicios de prehabilitación trimodal
1.8	Incluir PROTEINA C REACTIVA (PCR), en la analitica preoperatoria
1.9	Firma de Consentimientos Informados
1.10	Remitir a la Consulta de Estomaterapia si procede

EUPEMEN European Perioperative Medical Networking
Co-funded by the Erasmus+ Programme of the European Union
Cátedra GERM Medicina Perioperatoria Universidad Zaragoza

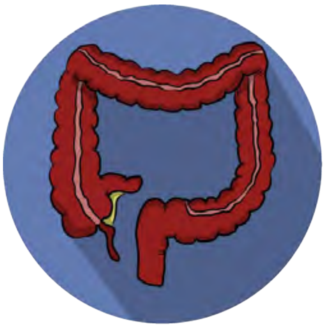

PROTOCOLO EUPEMEN

COLECTOMÍA

2	**Perioperatorio**
2.1	Preoperatorio Inmediato<br>Si es posible, programar el ingreso el mismo día de la cirugía<br><br>Anestesiología, Cirugía, Enfermería
2.1.1	Dieta baja en residuos: al menos 48 horas antes de la cirugía.
2.1.2	Continuar con tratamiento nutricional previo si desnutrición
2.1.3	Ayuno de 6 horas para sólidos y 2 horas para líquidos claros
2.1.4	Suplemento de bebida carbohidratada 12,5% de maltodextrinas 400 cc 2 horas antes de la cirugía (en caso de diabetes administrar junto con medicación antidiabética)
2.1.5	Iniciar profilaxis tromboembólica según protocolo del hospital
2.1.6	No preparación intestinal mecánica (ni antibioterapia oral).
2.1.7	Enema de Limpieza: 2 enemas de limpieza (500 cc SF) la tarde previa a la Cirugía
2.1.8	Baño completo
2.1.9	Rasurado con maquinilla eléctrica si éste es necesario
2.1.10	Colocación de medias compresivas o de compresión neumática intermitente
2.1.11	Administración profiláctica de antibiótico 30-60 min antes de la incisión quirúrgica. En procedimientos prolongados repetir dosis de acuerdo a la vida media de los fármacos
2.1.12	Marcaje de Estoma (si se prevé)
2.2	Intraoperatorio

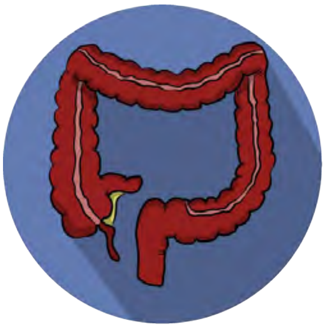

PROTOCOLO EUPEMEN

COLECTOMÍA

	Anestesiología, Cirugía, Enfermería
2.2.1	**Monitorización rutinaria:**<br>EKG, Presión Arteriar no Invasiva (PANI), Pulsioximetría (%Sat O2), FiO2, Capnografía, Temperatura, Glucemia intraoperatoria., Profundidad anestésica (BIS), Bloqueo neuromuscular.
2.2.2	Valorar la incorporación de monitorización hemodinámica no invasiva
2.2.3	Valorar relajación muscular profunda**. Valorar uso de relajantes aminoesteroideos** como primera opción (si se dispone de Sugammadex).
2.2.4	Desinfección de la piel de atrás hacia adelante.
2.2.5	Sondaje vesical
2.2.6	**Completar el LVQ OMS** (mínimo) primera fase
2.2.7	**Monitorización invasiva:**<br>• Canalización arterial invasiva NO de forma rutinaria (potencialmente en pacientes con alteraciones cardiorrespiratrias graves)<br>• Catéter venoso central NO de forma rutinaria
2.2.8	**Inducción y mantenimiento anestésico** con agentes de acción corta
2.2.9	**Oxigenación** FiO2 >50%
2.2.10	**Fluidoterapia:** Se recomienda optimización hemodinámica mediante **fluidoterapia guiada por objetivos** con dispositivos validados. En caso de no disponer de éstos, se recomienda fluidoterapia basada en peso ideal en perfusión continua solución balanceada (3-5ml/kg/h para laparoscopia; 5-7ml/kg/h para laparotomía).
2.2.11	**No sonda nasogástrica de manera rutinaria**.
2.2.12	**Calentamiento activo** según temperatura central, control temperatura ambiente
2.2.13	**Profilaxis de náuseas y vómitos postoperatorios según escala Apfel (según anexo RICA)**

EUPEMEN European Perioperative Medical Networking
Co-funded by the Erasmus+ Programme of the European Union
Cátedra GERM Medicina Perioperatoria Universidad Zaragoza

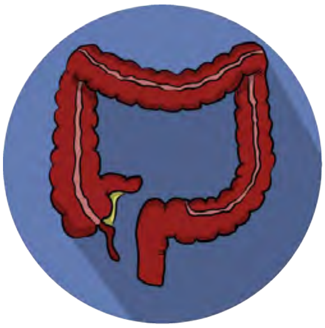

2.2.14	**Analgesia epidural torácica** a todos los pacientes sometidos a cirugía abierta. **En cirugía laparoscópica no se recomienda de rutina**. Pacientes con contraindicación para analgesia epidural podrían beneficiarse de TAP bilateral y/o **infiltrar trócares con anestésico local**
2.2.15	**Limitar el uso de catéteres centrales** a la necesidad de NPT inmediata, necesidad de fluidoterapia de alto flujo u otras indicaciones específicas
2.2.16	En cirugía laparoscópica se recomienda presión de neumoperitoneo entr 8 y 12 mmHg
2.2.17	**Coadyuvantes analgésicos endovenosos:**<br>• AINEs<br>• Lidocaina durante la intervención quirúrgica<br>• Ketamina (si tratamiento con opiáceos mayores)<br>• Sulfato de magnesio<br>• Dexmetomidina
2.2.18	**Evitar niveles de glucemia > 180 mg/dl** en paciente de riesgo de desarrollar insulinoresistencia (obesos, ancianos, larga duración quirúrgica)
2.2.19	**Desinfección de la piel** en circulo de limpio a sucio con clorhexidina en solución alcoholica al 1%
2.2.20	**Cirugía mínimamente invasiva** (siempre que sea posible). En cirugía abierta, incisiones transversas bajas si es posible
2.2.21	**Evitar drenajes**
2.3	Postoperatorio Inmediato<br>Unidad de reanimación – Sala de hospitalización<br><br>Anestesiología, Enfermería
2.3.1	En caso de necesitar la administración de mórficos utilizar sistemas de PCA
2.3.2	Mantenimiento activo de temperatura
2.3.3	Mantenimiento de FiO2 0.5 2 horas tras fin intervención
2.3.4	Valoración del dolor: EVA (conseguir nivel de dolor 0-4)

EUPEMEN European Perioperative Medical Networking

Co-funded by the Erasmus+ Programme of the European Union

Cátedra GERM Medicina Perioperatoria Universidad Zaragoza

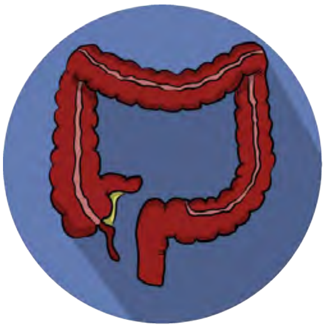

2.3.5	Analgesia pautada según intervención.
2.3.6	Mínima administración de mórficos. AINEs como terapia coadyuvante.
2.3.7	Fluidoterapia restrictiva
2.3.8	Inicio de tolerancia oral 6 horas tras cirugía
2.3.9	Inicio de movilización a las 8 horas tras cirugía
2.3.10	Fisioterapia respiratoria
2.3.11	Profilaxis del tromboembolismo
2.3.12	Control estricto de glucemia manteniendo niveles < 110 mg/dl en no diabéticos, y entre 110-150 mg/dl en diabéticos
3	**1º Día Postoperatorio**<br>Enfermería, Cirugía, Estomaterapia
3.1	Suplementación nutricional en casos seleccionados
3.2	Dieta líquida / semilíquida según tolerancia
3.3	Fisioterapia respiratoria
3.4	Valorar retirada de drenajes, si existen
3.5	Analgesia endovenosa. Evitar la administración de mórficos.
3.6	Movilización activa (cama/sillón/inicio deambulación)
3.7	Si tolerancia oral correcta retirada de líquidos endovenosos.
3.8	Valorar la retirada de Sondaje vesical

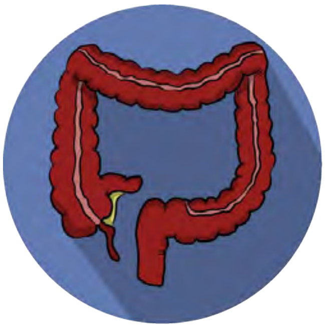

3.9	Profilaxis NVPO. Profilaxis antiulcerosa
3.10	Profilaxis tromboembólica
3.11	Control estricto de glucemia manteniendo niveles < 110 mg/dl en no diabéticos, y entre 110-150 mg/dl en diabéticos
3.12	Cuidados del Estoma e inicio de educación para el manejo del estoma.
3.14	**Analítica con PCR**
4	2º Día Postoperatorio<br>Enfermería, cirugía, estomaterapia
4.1	Valorar retirada de sondaje vesical (si presenta)
4.2	Dieta semiblanda/blanda
4.3	Fisioterapia respiratoria
4.4	Movilización activa (deambulación)
4.5	Analgesia endovenosa. Valorar analgesia oral
4.6	Retirada de líquidos endovenosos (si no se han retirado previamente)
4.7	Profilaxis del tromboembolismo
4.8	Profilaxis NVPO. Profilaxis antiulcerosa
4.9	Control estricto de glucemia
4.10	Cuidados del estoma
5	3º Día Postoperatorio

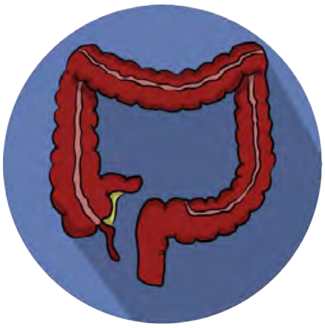

PROTOCOLO EUPEMEN

COLECTOMÍA

	Y resto de hospitalización<br><br>Enfermería, Cirugía, Estomaterapia
5.1	Analgesia oral
5.2	Fisioterapia respiratoria
5.3	Retirada de la vía venosa
5.4	Movilización activa (deambulación)
5.5	Profilaxis del tromboembolismo
5.6	Valorar alta a domicilio a partir de 3º día
5.7	Control estricto de glucemia
5.8	Control del estoma
5.9	**Control analítico de PCR**
5.10	**VALORAR CRITERIOS DE ALTA**<br>Valoración de posible alta si cumple los siguientes criterios: No complicaciones quirúrgicas, no fiebre, dolor controlado con analgesia oral, deambulación completa, tolerancia oral correcta .
6	Al Alta<br><br>Enfermería, Cirugía, Estomaterapia, MAP
6.1	Información personalizada, comprensible y completa
6.2	Mantenimiento de tromboprofilaxis 28 días tras cirugía
6.3	Control telefónico tras alta

EUPEMEN European Perioperative Medical Networking

Co-funded by the Erasmus+ Programme of the European Union

Cátedra GERM Medicina Perioperatoria Universidad Zaragoza

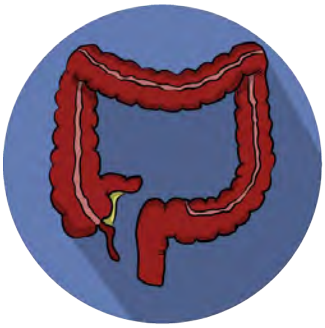

6.4	Seguimiento al alta/continuidad asistencial: 1, 3 y 6 meses tras el alta
6.5	Seguimiento en consulta de Estomaterapia (si procede)
6.6	Apoyo domiciliario-Coordinación con Atención Primaria

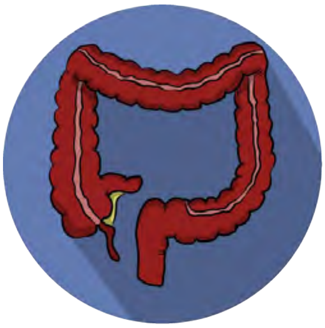

PROTOCOLO EUPEMEN

OBSTRUCCIÓN DE INTESTINO DELGADO

1	Preoperatorio<br><br>Cirugía, Anestesiología, Enfermería
1.1	Valoración preoperatoria habitual, incluyendo PCR en la analítica
1.2	Valorar Scores AIR, AAS, fragilidad (Mfi Frailty, VIG Express), delirium (evaluar criterios de beers)
1.3	Normotermia preoperatoria en pacientes frágiles (mantas calor)
1.4	Control de glucemia perioperaroria:<br>• Si es diabético, protocolo hospitalario de DM y cirugía<br>• Resto: Evitar glucemia > 180 mg/dL en paciente de riesgo de desarrollar insulinorresistencia (obeso, anciano, duración quirúrgica > 1 hora)
1.5	Profilaxis Antibiótica según protocolo del hospital (PROA).
1.6	Colocación de Sonda Nasogástrica
1.7	Paquete de medidas (Bundle) para prevenir la infección de la herida quirúrgica según protocolo IQZ (Infección Quirúrgica Zero).
1.8	Todos los pacientes que cumplan los criterios para entrar en el protocolo serán ampliamente informados, con la entrega de consentimiento informado.
2	Intraoperatorio<br><br>Anestesiología, Cirugía, Enfermería
2.1	Lista de verificación quirúrgica.
2.2	Monitorización rutinaria.
2.3	Inducción anestésica de secuencia rápida.
2.4	Oxigenación con FiO2 de 0,6 a 0,8
2.5	Fluidoterapia: Se recomienda optimización hemodinámica mediante FLUIDOTERAPIA GUIADA POR OBJETIVOS con dispositivos validados (CLEARSIGHT).<br>Si no se dispone, fluidoterapia basada en peso ideal en perfusión continua con solución balanceada:<br>• 3-5ml/kg/h para laparoscopia<br>• 5-7 ml/kg/h para laparotomía

EUPEMEN European Perioperative Medical Networking
Co-funded by the Erasmus+ Programme of the European Union
Cátedra GERM Medicina Perioperatoria Universidad Zaragoza

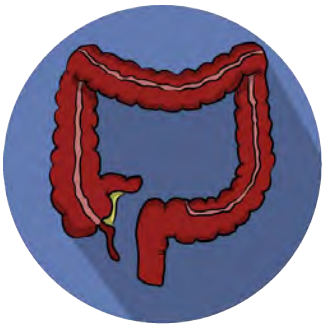

PROTOCOLO EUPEMEN

OBSTRUCCIÓN DE INTESTINO DELGADO

2.6	Control de glucemia perioperaroria:<br>• Si es diabético, protocolo hospitalario de DM y cirugía<br>• Resto: Evitar glucemia > 180 mg/dL en paciente de riesgo
2.7	Cirugía mínimamente invasiva en casos altamente seleccionados: 1º episodio Vs única adherencia, según clínica del paciente y experiencia del cirujano.
2.8	En cirugía abierta, valorar catéter epidural
2.9	Sondaje vesical solo si precisa.
2.10	Sonda nasogástrica
2.11	Protocolo de normotermia: calentamiento activo con manta térmica y calentador de fluidos
2.12	Profilaxis de náuseas y vómitos postoperatorios según escala de Apfel.
2.13	No drenajes
2.14	Analgesia multimodal ahorradora de opioides, incluyendo infiltración de los puertos de laparoscopia con anestésico local o TAP BLOCK.
2.15	Profilaxis tromboembólica según protocolo (medias de compresión o compresión intermitente)
2.16	Paquete de medidas (Bundle) para prevenir la infección de la herida quirúrgica según protocolo IQZ (Infección Quirúrgica Zero)
**3**	**Postoperatorio Inmediato – Día 0**<br>Anestesiología, Enfermería
3.1	Mantenimiento activo de la temperatura.
3.2	Oxigenoterapia con FiO2 adecuada para mantener SpO2 óptimas.
3.3	Analgesia multimodal con mínima administración de mórficos.
3.4	Fluidoterapia restrictiva.
3.5	Control de glucemia perioperaroria:<br>• Si es diabético, protocolo de DM y cirugía<br>• Resto: Evitar glucemia > 180 mg/dL en paciente de riesgo
3.6	Dieta absoluta y SNG (valorar retirada a las 12h tras la intervención si posible)

EUPEMEN European Perioperative Medical Networking
Co-funded by the Erasmus+ Programme of the European Union
Cátedra GERM Medicina Perioperatoria Universidad Zaragoza

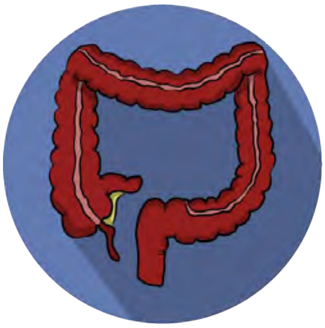

PROTOCOLO EUPEMEN

OBSTRUCCIÓN DE INTESTINO DELGADO

3.7	Valorar retirada sonda uretral
3.8	Inicio de movilización precoz a las 8 horas de la intervención.
3.9	Profilaxis del tromboembolismo a partir de 12 h tras la intervención según protocolo.
3.10	Formulario de Control de Analgesia en Dolor Agudo Postoperatorio al ALTA de la URPA
4	**Postoperatorio Día 1**<br>Cirugía, Enfermería
4.1	Control de glucemia perioperaroria:<br>• Si es diabético, protocolo hospitalario de DM y cirugía<br>• Resto: Evitar glucemia > 180 mg/dL en paciente de riesgo
4.2	Tromboprofilaxis
4.3	Movilización activa (cama/sillón/inicio deambulación)
4.4	Fisioterapia respiratoria.
4.5	Si posible traslocación o contaminación local intraoperatoria asociada: Antibioterapia de amplio espectro según protocolo PROA (día 1)
4.6	Valorar retirada de sonda nasogástrica si la tuviera<br>* Si retirada de SNG: valorar inicio dieta líquida/gelatina/yoghourt según situación clínica +/- retirada de sueroterapia si procede
4.7	Valorar retirada de sondaje vesical si procede
4.8	Valorar retirada de catéter epidural si procede
5	**Postoperatorio Día 2**<br>Cirugía, Enfermería
5.1	Control de glucemia perioperaroria:<br>• Si es diabético, protocolo hospitalario de DM y cirugía<br>• Resto: Evitar glucemia > 180 mg/dL en paciente de riesgo
5.2	Tromboprofilaxis
5.3	Movilización activa (cama/sillón/inicio deambulación)
5.4	Fisioterapia respiratoria

EUPEMEN European Perioperative Medical Networking
Co-funded by the Erasmus+ Programme of the European Union
Cátedra GERM Medicina Perioperatoria Universidad Zaragoza

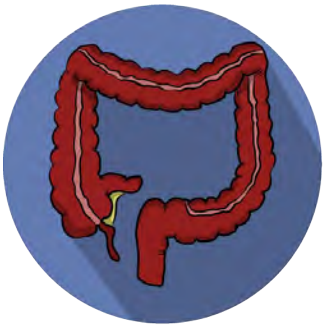

5.5	Analgesia vía oral si procede
5.6	Valorar retirada de sonda nasogástrica si la tuviera<br>* Si retirada de SNG: valorar inicio dieta líquida/gelatina/yogurt según situación clínica +/- retirada de sueroterapia si procede Vs Dieta de transición si se inició dieta día 1
5.7	Valorar alta si cumplen criterios de alta (casos sin resección intestinal)
6	Postoperatorio Día 3<br>Cirugía, Enfermería
6.1	Dieta progresiva
6.2	Movilización activa (deambulación)
6.3	Fisioterapia respiratoria
6.4	Analgesia vía oral
6.5	Tromboprofilaxis
6.6	Valorar alta si cumplen criterios de alta
7	Al Alta<br>Cirugía, Enfermería, MAP
7.1	Mantenimiento de tromboprofilaxis individualizado según riesgos
7.2	Analítica de control con PCR previo al alta
7.3	Valorar control telefónico tras el alta a las 24 horas
7.4	Criterios generales de alta: no complicaciones, no fiebre, dolor controlado con analgesia oral, deambulación completa, aceptación por parte del paciente
7.5	Seguimiento al alta/continuidad asistencial
7.6	Apoyo domiciliario-Coordinación con atención Primaria

EUPEMEN European Perioperative Medical Networking

Co-funded by the Erasmus+ Programme of the European Union

Cátedra GERM Medicina Perioperatoria Universidad Zaragoza

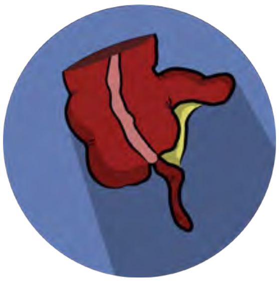

PROTOCOLO EUPEMEN

APENDICITIS AGUDA

**1**	**Preoperatorio**<br>Cirugía, Anestesiología
1.1	Valoración preoperatoria habitual (POCUS), incluyendo PCR en la analítica.
1.2	Valorar Scores AIR, AAS, fragilidad (Mfi Frailty, VIG Express), delirium (evaluar criterios de beers)
1.3	Normotermia preoperatoria en pacientes frágiles (mantas calor)
1.4	Micción voluntaria
1.5	Control de glucemia perioperaroria:<br>• Si es diabético, protocolo hospitalario de DM y cirugía<br>• Resto: Evitar glucemia > 180 mg/dL en paciente de riesgo de desarrollar insulinorresistencia (obeso, anciano, duración quirúrgica > 1 hora)
1.6	Profilaxis Antibiótica según protocolo del hospital (PROA).
1.7	Paquete de medidas (Bundle) para prevenir la infección de la herida quirúrgica según protocolo IQZ (Infección Quirúrgica Zero).
1.8	Todos los pacientes que cumplan los criterios para entrar en el protocolo serán ampliamente informados, con la entrega de consentimiento informado.
**2**	**Intraoperatorio**<br>Anestesiología, Cirugía, Enfermería
2.1	Lista de verificación quirúrgica.
2.2	Monitorización rutinaria.
2.3	PROCEDIMIENTO DE ELECCIÓN: Cirugía laparoscópica o mínimamente invasiva SALVO EXCEPCIONES.
2.4	Inducción anestésica de secuencia rápida.
2.5	Oxigenación con FiO2 de 0,6 a 0,8

EUPEMEN European Perioperative Medical Networking

Co-funded by the Erasmus+ Programme of the European Union

Cátedra GERM Medicina Perioperatoria Universidad Zaragoza

2.6	Fluidoterapia: Se recomienda optimización hemodinámica mediante FLUIDOTERAPIA GUIADA POR OBJETIVOS con dispositivos validados (CLEARSIGHT).<br>Si no se dispone, fluidoterapia basada en peso ideal en perfusión continua con solución balanceada:<br>• 3-5ml/kg/h para laparoscopia<br>• 5-7 ml/kg/h para laparotomía
2.7	Sondaje vesical sólo si precisa (evitar en la medida de lo posible)
2.8	Sonda nasogástrica sólo si precisa (evitar en la medida de lo posible)
2.9	Protocolo de normotermia.
2.10	Control de glucemia perioperaroria:<br>• Si es diabético, protocolo hospitalario de DM y cirugía<br>• Resto: Evitar glucemia > 180 mg/dL en paciente de riesgo
2.11	Profilaxis de náuseas y vómitos postoperatorios según escala de Apfel.
2.12	Evitar drenajes (en la medida de lo posible)
2.13	Analgesia multimodal ahorradora de opiodes, incluyendo infiltración de los puertos de laparoscopia con anestésico local o TAP BLOCK.
2.14	Profilaxis tromboembólica según protocolo (medias de compresión o compresión intermitente)
2.15	Paquete de medidas (Bundle) para prevenir la infección de la herida quirúrgica según protocolo IQZ (Infección Quirúrgica Zero).
**3**	**Postoperatorio Inmediato – Día 0**<br>Anestesiología, Enfermería
3.1	Mantenimiento activo de la temperatura.
3.2	Oxigenoterapia con FiO2 adecuada para mantener SpO2 óptimas.
3.3	Analgesia multimodal ahorradora de opioides
3.4	Fluidoterapia restrictiva.

EUPEMEN European Perioperative Medical Networking

Co-funded by the Erasmus+ Programme of the European Union

Cátedra GERM Medicina Perioperatoria Universidad Zaragoza

3.5	Control de glucemia perioperaroria:<br>• Si es diabético, protocolo de DM y cirugía<br>• Resto: Evitar glucemia > 180 mg/dL en paciente de riesgo
3.6	Sedestación a las 2h como paso previo a ala deambulación
3.7	Inicio de movilización precoz a las 8 horas de la intervención.
3.8	Inicio de dieta según situación clínica (agua): 10-12 h tras la intervención
3.9	Respetar horas de sueño en ingesta y deambulación.
3.10	Profilaxis del tromboembolismo a partir de 12 h tras la intervención según protocolo.
3.11	Antibioterapia según protocolo PROA
3.12	Formulario de Control de Analgesia en Dolor Agudo Postoperatorio al ALTA de la URPA.
4	**Postoperatorio Día 1**<br>Cirugía, Enfermería
4.1	Dieta líquida/gelatina/yoghourt según situación clínica
4.2	Movilización activa (cama/sillón/inicio deambulación)
4.3	Fisioterapia respiratoria.
4.4	Analgesia vía oral si es posible
4.5	Si tolerancia oral correcta, retirar sueroterapia.
4.6	Antibioterapia según PROA.
4.7	Tromboprofilaxis
5	**Postoperatorio Día 2**<br>Cirugía, Enfermería

EUPEMEN European Perioperative Medical Networking

Co-funded by the Erasmus+ Programme of the European Union

Cátedra GERM Medicina Perioperatoria Universidad Zaragoza

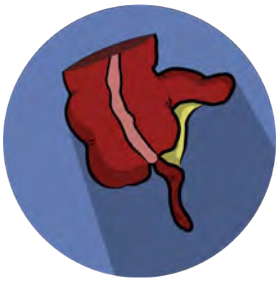

PROTOCOLO EUPEMEN

APENDICITIS AGUDA

5.1	Dieta semilíquida/blanda fácil digestión según situación clínica
5.2	Movilización activa (cama/sillón/inicio deambulación
5.3	Fisioterapia respiratoria
5.4	Analgesia vía oral
5.5	Si tolerancia oral correcta, retirar sueroterapia
5.6	Antibioterapia según PROA.
5.7	Tromboprofilaxis
5.8	Valorar alta si cumplen criterios de alta.
6	Durante el resto de Hospitalización<br>Cirugía, Enfermería
6.1	Dieta progresiva
6.2	Movilización activa (deambulación)
6.3	Fisioterapia respiratoria
6.4	Analgesia vía oral
6.5	Antibioterapia según PROA.
6.6	Tromboprofilaxis
6.7	Valorar alta si cumplen criterios de alta.
7	Al Alta<br>Cirugía, Enfermería, MAP
7.1	Mantenimiento de tromboprofilaxis individualizado según riesgos

EUPEMEN European Perioperative Medical Networking
Co-funded by the Erasmus+ Programme of the European Union
Cátedra GERM Medicina Perioperatoria Universidad Zaragoza

7.2	Antibioterapia según protocolo PROA (valorar ciclo corto si cumple criterios)
7.3	Analítica de control con PCR (valorar reducción pcr 50%) previo al alta.
7.4	Valorar control telefónico tras el alta a las 24 horas.
7.5	Criterios generales de alta: no complicaciones, no fiebre, dolor controlado con analgesia oral, deambulación completa, aceptación por parte del paciente.
7.6	Seguimiento al alta/continuidad asistencial
7.7	Apoyo domiciliario-Coordinación con atención Primaria.
