# Supplementary material for: Advancing abdominal surgery recovery implementation: a unified framework for intensified recovery protocols by the EUropean PErioperative MEdical Networking collaborative
Source: Front Surg. 2026 May 18;13:1827678. doi: 10.3389/fsurg.2026.1827678 (PMC13223102; doi:10.3389/fsurg.2026.1827678)
Supplement: Supplementary file 14 [file Datasheet5.pdf]

Co-funded by the Erasmus+ Programme of the European Union

EUPEMEN
European Perioperative Medical Networking

EUPEMEN Πρωτόκολλο (EL)

Instituto de Investigación Sanitaria Aragón

SERVIZIO SANITARIO REGIONALE EMILIA-ROMAGNA
Azienda Unità Sanitaria Locale di Ferrara

2. LÉKAŘSKÁ FAKULTA UNIVERZITA KARLOVA

UNIVERSITAS Miguel Hernández

ΓΕΝΙΚΟ ΝΟΣΟΚΟΜΕΙΟ ΘΕΣΣΑΛΟΝΙΚΗΣ
“Γ. ΠΑΠΑΝΙΚΟΛΑΟΥ”

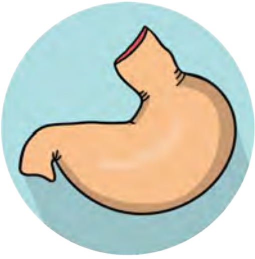

Πρωτόκολλο Eupemen
για Οισοφαγεκτομή

1	Πριν την εισαγωγή<br>Αναισθησιολόγος, Χειρουργός, Νοσηλευτής, Διαιτολόγος
1.1	**Προεγχειρητική συμβουλευτική**<br>Ο ασθενής θα πρέπει να είναι πλήρως ενημερωμένος για τη διαδικασία και την περιεγχειρητική πορεία τόσο προφορικά όσο και γραπτά. Θα πρέπει να λαμβάνεται υπογεγραμμένη ενημερωμένη συγκατάθεση.
1.2	**Ολοκληρωμένη ιατρική αξιολόγηση**<br>Αυτό θα πρέπει να περιλαμβάνει ιατρικό ιστορικό, φυσική εξέταση, ακτινογραφία θώρακα, εξετάσεις αίματος (έλεγχος πήξης, βιοχημικό προφίλ, γενική αίματος) και ηλεκτροκαρδιογράφημα.
1.3	**Ρύθμιση χρόνιων παθήσεων**<br>Όλες οι χρόνιες παθήσεις θα πρέπει να βελτιστοποιούνται πριν από την επέμβαση. Όλες οι περιπτώσεις πρόσφατης έναρξης ή ενεργών καρδιαγγειακών παθήσεων θα πρέπει να αξιολογούνται από καρδιολόγο.
1.4	**Αξιολόγηση Σακχαρώδη Διαβήτη**<br>Τα επίπεδα γλυκόζης αίματος και γλυκοζηλιωμένης αιμοσφαιρίνης (HbA1c) θα πρέπει να εκτιμηθούν. Όλες οι περιπτώσεις φτωχά ελεγχόμενου ή προηγουμένως αδιάγνωστου διαβήτη θα πρέπει να παραπέμπονται στην πρωτοβάθμια φροντίδα ή σε ενδοκρινολογο / διαβητολόγο πριν από τη χειρουργική επέμβαση.
1.5	**Αξιολόγηση και διαχείριση της αναιμίας και της ανεπάρκειας σιδήρου**<br>Η σιδηροπενική αναιμία θα πρέπει να αντιμετωπίζεται ιδανικά με παρεντερική χορήγηση σιδήρου.
1.6	**Διατροφικός έλεγχος**<br>Ο διατροφικός έλεγχος θα πρέπει να γίνεται χρησιμοποιώντας το εργαλείο MUST (Malnutrition Universal Screening Tool). Για περιπτώσεις αφαγίας αξιολογήστε τα μέτρα και τις οδούς χορήγησης τεχνητής διατροφής σύμφωνα με το τοπικό νοσοκομειακό πρωτόκολλο. Για στερεά δυσφαγία χορηγείστε υγρή δίαιτα με συμπληρώματα πρωτεΐνης.
1.7	**Διακόψτε το κάπνισμα και μειώστε την κατανάλωση αλκοόλ**<br>Η χρήση του καπνού θα πρέπει να σταματήσει και η κατανάλωση αλκοόλ θα πρέπει να μειωθεί αμέσως μόλις γίνει η διάγνωση.

EUPEMEN
European Perioperative Medical Networking

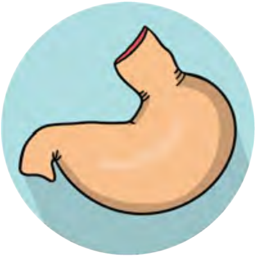

1.8	**Καρδιαγγειακές ασκήσεις**<br>Καρδιαγγειακές και αναπνευστικές ασκήσεις προσαρμοσμένες στη φυσική κατάσταση του ασθενούς.
1.9	**Ψυχολογική συμβουλευτική**<br>Οποιαδήποτε ψυχολογικά προβλήματα μπορεί να αντιμετωπίζει ο ασθενής θα πρέπει να αντιμετωπίζονται πλήρως.
1.10	**Αξιολόγηση ευπάθειας**<br>Για ασθενείς ηλικίας άνω των 65 ετών θα πρέπει να γίνεται αξιολόγηση ευπάθειας.
1.11	**Κλίμακα Apfel**<br>Ο κίνδυνος για μετεγχειρητική ναυτία και έμετο θα πρέπει να αξιολογείται με την κλίμακα Apfel.
1.12	**Αξιολόγηση κατάταξης στην κλίμακα ASA**<br>Ως μέρος της προεγχειρητικής αναισθησιολογικής αξιολόγησης θα πρέπει να εκτιμάται η κατάταξη στην κλίμακα ASA (American Society of Anesthesiologists).
2	Περιεγχειρητικά
2.1	Άμεσα Προεγχειρητικά<br><br>Αναισθησιολόγος, Χειρουργός, Νοσηλευτής
2.1.1	**Προεγχειρητική υγιεινή**<br>Ο ασθενής λαμβάνει οδηγίες να κάνει πλήρες ντους ή μπάνιο το βράδυ ή το πρωί πριν από την επέμβαση.
2.1.2	**Κάλτσες συμπίεσης ή διαλείπουσα πνευματική συμπίεση.**<br>Οι κάλτσες συμπίεσης ή η διαλείπουσα πνευματική συμπίεση πρέπει να τοποθετούνται από την εισαγωγή στο νοσοκομείο.
2.1.3	**Ηπαρίνη χαμηλού μοριακού βάρους**<br>Η ηπαρίνη χαμηλού μοριακού βάρους πρέπει να χορηγείται 2-12 ώρες πριν από την επέμβαση (ανάλογα με το εάν πρόκειται να γίνει νευραξονική αναισθησία ή όχι).
2.1.4	**Πόσιμο διάλυμα υδατανθράκων**<br>Ένα ρόφημα πλούσιο σε υδατάνθρακες (12,5% μαλτοδεξτρίνες) 800 ml πρέπει να χορηγείται το βράδυ πριν από την επέμβαση και 400 ml 2 ώρες

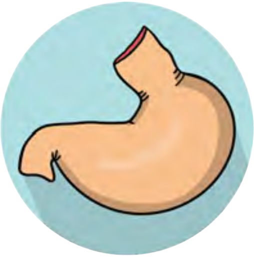

	πριν από την αναισθησία. Για διαβητικούς ασθενείς χορηγήστε το μαζί με αντιδιαβητική αγωγή.
2.1.5	**Προεγχειρητική νηστεία**<br>Νηστεία 6 ωρών για τα στερεά και 2 ωρών για τα διαυγή υγρά.
2.1.6	**Ξύρισμα με ηλεκτρικό ξυράφι**<br>Το σημείο όπου θα γίνει η τομή θα πρέπει να ξυριστεί με ηλεκτρική ξυριστική μηχανή, εάν είναι απαραίτητο.
2.1.7	**Αντιβιοτική χημειοπροφύλαξη**<br>Προφυλακτική χορήγηση αντιβιοτικού 30-60 λεπτά πριν την τομή. Σε παρατεταμένες διαδικασίες επαναλάβετε τις δόσεις ανάλογα με τον χρόνο ημιζωής των φαρμάκων.
2.1.8	**Καθυστερημένη γαστρική κένωση**<br>Για ασθενείς με καθυστερημένη γαστρική κένωση πρέπει να λαμβάνονται προφυλακτικά μέτρα για την πρόληψη της παλινδρόμησης.
2.2	Διεγχειρητικά<br><br>Αναισθησιολόγος, Χειρουργός, Νοσηλευτής
2.2.1	**Λίστα ελέγχου χειρουργικής ασφάλειας του ΠΟΥ (Παγκόσμιου Οργανσιμού Υγείας)**<br>Η λίστα ελέγχου χειρουργικής ασφάλειας του ΠΟΥ θα πρέπει να συμπληρωθεί πριν γίνει η τομή.
2.2.2	**Συστηματική διεγχειρητική παρακολούθηση**<br>Οι ζωτικές λειτουργίες, το FiO2, το βάθος της αναισθησίας, ο νευρομυϊκός αποκλεισμός και η γλυκαιμία θα πρέπει να παρακολουθούνται κατά τη διάρκεια της διαδικασίας. Συνιστάται επίσης η μη επεμβατική αιμοδυναμική παρακολούθηση.
2.2.3	**Ελάχιστα επεμβατική χειρουργική**<br>Προτιμώνται οι ελάχιστα επεμβατικές προσπελάσεις και θα πρέπει να χρησιμοποιούνται όσο το δυνατόν περισσότερο.
2.2.4	**Αποφύγετε τον συστηματικό καθετηριασμό της ουροδόχου κύστης**
2.2.5	**Επεμβατική παρακολούθηση**<br>Δεν απαιτείται συνήθως τοποθέτηση αρτηριακού καθετήρα. Αν και θα πρέπει να χρησιμοποιείται σε ασθενείς με σοβαρές καρδιοαναπνευστικές

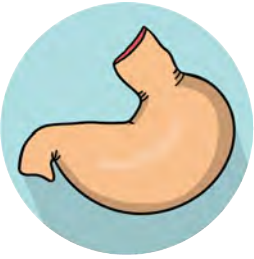

	διαταραχές.
2.2.6	**Κεντρικός φλεβικός καθετήρας**<br>Οι κεντρικοί φλεβικοί καθετήρες δεν απαιτούνται συνήθως για μικρές εκτομές και απουσία παραγόντων κινδύνου για μετεγχειρητική νεφρική ανεπάρκεια.
2.2.7	**Εισαγωγή και διατήρηση της αναισθησίας**<br>Παράγοντες βραχείας δράσης θα πρέπει να χρησιμοποιούνται για την εισαγωγή και τη διατήρηση της αναισθησίας.
2.2.8	**Οξυγόνωση**<br>Οι ασθενείς θα πρέπει να λαμβάνουν οξυγόνο με FiO2 άνω του 50%.
2.2.9	**Χορήγηση Υγρών**<br>Η Αιμοδυναμική βελτιστοποίηση με στοχοκατευθυνόμενη χορήγηση υγρών με τη χρήση συσκευών παρακολούθησης του αιμοδυναμικού προφίλ του ασθενούς, συνιστάται σε ασθενείς υψηλού κινδύνου και σε ασθενείς που υποβάλλονται σε χειρουργική επέμβαση με μεγάλη απώλεια αίματος. Σε όλες τις άλλες περιπτώσεις, συνιστάται περιοριστική χορήγηση υγρών με βάση το ιδανικό βάρος με συνεχή χορήγηση ισορροπημένου κρυσταλλοειδούς διάλυματος (1-3 ml/kg/h για λαπαροσκόπηση, 3-5 ml/kg/h για λαπαροτομία). Η απώλεια αίματος θα πρέπει να αντισταθμίζεται με κολλοειδή 1:1.
2.2.10	**Αποφύγετε το ρινογαστρικό σωλήνα**<br>Οι ρινογαστρικοί σωλήνες δεν πρέπει να χρησιμοποιούνται τακτικά.
2.2.11	**Πρόληψη της υποθερμίας**<br>Η θερμοκρασία θα πρέπει να παρακολουθείται και η νορμοθερμία πρέπει να διατηρείται με ενεργητική θέρμανση (θερμά υγρά, θερμαινόμενη κουβέρτα).
2.2.12	**Προφύλαξη μετεγχειρητικής ναυτίας και εμέτου**<br>Χορηγήστε αντιεμετική θεραπεία σύμφωνα με την κλίμακα Apfel.
2.2.13	**Επισκληρίδιος αναλγησία**<br>Η θωρακική επισκληρίδιος αναλγησία πρέπει να χρησιμοποιείται σε ανοιχτή χειρουργική επέμβαση. Στη λαπαροσκοπική χειρουργική δεν συνιστάται συνήθως. Ασθενείς με αντένδειξη για επισκληρίδιο αναλγησία που έχουν κίνδυνο μετεγχειρητικής νεφρικής ανεπάρκειας ή έχουν διαταραχές πήξης θα μπορούσαν να ωφεληθούν από αμφοτερόπλευρο αποκλεισμό στο επίπεδο του Εγκάρσιου Κοιλιακού Μυός (TAP BLOCK - transabdominal plan blocks), ή άλλες εναλλακτικές λύσεις αντί της επισκληρίδιου αναλγησίας.

2.2.14	**Ενδοφλέβια επικουρικά αναλγητικά φάρμακα**<br>Συνιστώμενα επικουρικά αναλγητικά είναι τα μη στεροειδή αντιφλεγμονώδη φάρμακα, η λιδοκαΐνη, η κεταμίνη, το θειικό μαγνήσιο και η δεξμεντετομιδίνη.
2.2.15	**Παρακολούθηση γλυκόζης αίματος**<br>Αποφύγετε τα επίπεδα γλυκόζης στο αίμα > 180 mg/dl σε ασθενείς που διατρέχουν κίνδυνο να αναπτύξουν αντίσταση στην ινσουλίνη.
2.2.16	**Απολύμανση δέρματος**<br>Το δέρμα πρέπει να απολυμαίνεται από κεντρικά προς περιφερειακά με αλκοολικό διάλυμα χλωρεξιδίνης 2%.
2.2.17	**Αποφύγετε τις παροχετεύσεις**<br>Οι κοιλιακές παροχετεύσεις πρέπει να αποφεύγονται όσο το δυνατόν περισσότερο.
2.3	Άμεσα Μετεγχειρητικά<br>(Μονάδα Μετα-αναισθητικής Φροντίδας / Μονάδα Ενδιάμεσης Φροντίδας σε επιλεγμένες περιπτώσεις)<br><br>Αναισθησιολόγος, Νοσηλευτής
2.3.1	**Διατήρηση της νορμοθερμίας**<br>Η θερμοκρασία πρέπει να μετράται τακτικά και να διατηρείται η νορμοθερμία.
2.3.2	**Αναλγησία με περιορισμό των οπιοειδών**<br>Θα πρέπει να χρησιμοποιείται ενεργητική ή προληπτική πολυπαργοντική αναλγησία. Περιορίστε τη χρήση οπιοειδών. Στοχεύστε σε βαθμολογία πόνου στην οπτική αναλογική κλίμακα (Visual Analog Scale – VAS) μικρότερη από 3.
2.3.3	**Περιοριστική χορήγηση υγρών.**
2.3.4	**Πρώιμη σίτιση**<br>Έναρξη λήψης υγρών από το στόμα 6 - 8 ώρες μετά το χειρουργείο.
2.3.5	**Αναπνευστική φυσιοθεραπεία**
2.3.6	**Πρώιμη κινητοποίηση**<br>Η κινητοποίηση θα πρέπει να ξεκινά 3 ώρες μετά το χειρουργείο και θα

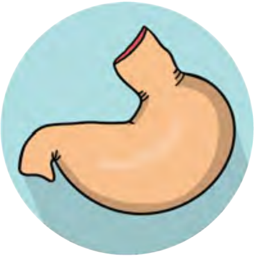

	πρέπει να ξεκινά με κάθισμα στο κρεβάτι.
2.3.7	**Θρομβοεμβολική προφύλαξη**<br>Η ηπαρίνη χαμηλού μοριακού βάρους πρέπει να χορηγείται 12 ώρες μετά την επέμβαση.
2.3.8	**Αντιμετώπιση μετεγχειρητικής ναυτίας και εμέτου**
2.3.9	**Διατήρηση FiO2 (κλάσμα εισπνεόμενου οξυγόνου) 0,5% για 2 ώρες μετά την επέμβαση**
3	1η Μετεγχειρητική ημέρα<br>(Μονάδα Εντατικής Θεραπείας, ενδεχόμενα Μονάδα Ενδιάμεσης Φροντίδας σε επιλεγμένες περιπτώσεις)<br><br>Χειρουργός, Αναισθησιολόγος, Νοσηλευτής
3.1	**Πρώιμη σίτιση**<br>Μια υγρή ή ημιστερεή δίαιτα πρέπει να ξεκινά ανάλογα με την ανοχή. Η ολική παρεντερική διατροφή πρέπει να χορηγείται όταν η από του στόματος δίαιτα δεν είναι ανεκτή.
3.2	**Περιοριστική ενδοφλέβια χορήγηση υγρών.**
3.3	**Πρώιμη κινητοποίηση**<br>Οι ασθενείς θα πρέπει να ενθαρρύνονται να μετακινούνται από το κρεβάτι στην καρέκλα δίπλα στο κρεβάτι.
3.4	**Αναλγησία με περιορισμό των οπιοειδών**<br>Εξασφαλίστε καλό έλεγχο του πόνου. Στοχεύστε σε βαθμολογία πόνου στην οπτική αναλογική κλίμακα (Visual Analog Scale – VAS) μικρότερη από 3.
3.5	**Αφαιρέστε τον ουροκαθετήρα**<br>Εάν έχει τοποθετηθεί ουροκαθετήρας, εξετάστε το ενδεχόμενο αφαίρεσής του.
3.6	**Αναπνευστική φυσιοθεραπεία**
3.7	**Θρομβοεμβολική προφύλαξη**

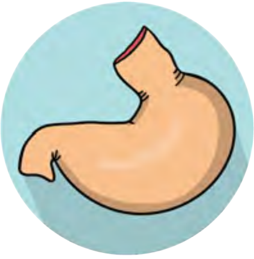

Πρωτόκολλο Eupemen
για Οισοφαγεκτομή

3.8	**Εργαστηριακές εξετάσεις**<br>Θα πρέπει να γίνονται εξετάσεις αίματος συμπεριλαμβανομένης της C-αντιδρώσας πρωτεΐνης και της προκαλσιτονίνης.
4	$2^{η}$ Μετεγχειρητική ημέρα<br>(Μονάδα Εντατικής Θεραπείας - Μονάδα Ενδιάμεσης Φροντίδας - Θάλαμος)<br><br>Χειρουργός, Αναισθησιολόγος, Νοσηλευτής
4.1	**Πρώιμη σίτιση**<br>Αυξήστε την από του στόματος πρόσληψη τροφής. Μπορούν να δοθούν ημιστερεές τροφές όπως πουρές και γιαούρτια.
4.2	**Διακόψτε την την ενδοφλέβια χορήγηση υγρών**
4.3	**Πρώιμη κινητοποίηση**<br>Οι ασθενείς θα πρέπει να μπορούν να περπατούν μικρές αποστάσεις.
4.4	**Αναλγησία με περιορισμό των οπιοειδών**<br>Εξασφαλίστε καλό έλεγχο του πόνου. Στοχεύστε σε βαθμολογία πόνου στην οπτική αναλογική κλίμακα (Visual Analog Scale – VAS) μικρότερη από 3.
4.5	**Αφαιρέστε τον επισκληρίδιο καθετήρα**
4.6	**Αναπνευστική φυσιοθεραπεία**
4.7	**Θρομβοεμβολική προφύλαξη**
5	$3^{η}$ Μετεγχειρητική ημέρα<br>(Θάλαμος)<br><br>Χειρουργός, Νοσηλευτής
5.1	**Πρώιμη σίτιση**<br>Δίαιτα με αλεσμένες τροφές.
5.2	**Πρώιμη κινητοποίηση**<br>Πλήρης κινητοποίηση.

5.3	**Αναλγησία με περιορισμό των οπιοειδών**<br>Εξασφαλίστε καλό έλεγχο του πόνου. Στοχεύστε σε βαθμολογία πόνου στην οπτική αναλογική κλίμακα (Visual Analog Scale – VAS) μικρότερη από 3.
5.4	**Αναπνευστική φυσιοθεραπεία**
5.5	**Θρομβοεμβολική προφύλαξη**
5.6	**Εργαστηριακές εξετάσεις**<br>Θα πρέπει να γίνονται εξετάσεις αίματος συμπεριλαμβανομένης της C-αντιδρώσας πρωτεΐνης και της προκαλσιτονίνης.
5.7	**Αξιολογήστε τα κριτήρια εξιτηρίου**<br>Εξετάστε το ενδεχόμενο εξιτηρίου εάν δεν υπάρχουν χειρουργικές επιπλοκές που δεν μπορούν να αντιμετωπιστούν σε εξωτερικά ιατρεία, δεν υπάρχει πυρετός, πόνος ελεγχόμενος με από του στόματος αναλγησία, πλήρης κινητοποίηση, ανοχή στην από του στόματος λήψη τροφής και αποδοχή από τον ασθενή.
6	4η Μετεγχειρητική Ημέρα, Εξιτήριο και Παρακολούθηση<br><br>Χειρουργός, Νοσηλευτής, Πρωτοβάθμια Φροντίδα
6.1	**Πρώιμη σίτιση**<br>Ελαφρά δίαιτα.
6.2	**Πρώιμη κινητοποίηση**<br>Πλήρης κινητοποίηση.
6.3	**Αναλγησία με περιορισμό των οπιοειδών**<br>Εξασφαλίστε καλό έλεγχο του πόνου. Στοχεύστε σε βαθμολογία πόνου στην οπτική αναλογική κλίμακα (Visual Analog Scale – VAS) μικρότερη από 3.
6.4	**Αναπνευστική φυσιοθεραπεία**
6.5	**Θρομβοεμβολική προφύλαξη**
6.6	**Εργαστηριακές εξετάσεις**<br>Θα πρέπει να γίνονται εξετάσεις αίματος συμπεριλαμβανομένης της C-αντιδρώσας πρωτεΐνης και της προκαλσιτονίνης.

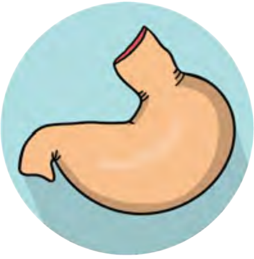

7	Εξιτήριο<br><br>Χειρουργός, Νοσηλευτής, Πρωτοβάθμια Περίθαλψη
7.1	**Ενημερωτικό σημείωμα για τον ασθενή**<br>Παρέχετε στους ασθενείς πληροφορίες για την ιατρική τους κατάσταση και δίνετε συστάσεις για περαιτέρω φροντίδα. Κατά το εξιτήριο στους ασθενείς θα πρέπει να παρέχονται πληροφορίες σχετικά με την πορεία της νοσηλείας τους, το φύλλο συστάσεων, διατροφικές συστάσεις και ερωτηματολόγιο ικανοποίησης.
7.2	**Παρακολούθηση ασθενών**<br>Οι ασθενείς θα πρέπει να παρακολουθούνται σε εξωτερικό ιατρείο ή μέσω τηλεφώνου (σύμφωνα με το πρωτόκολλο κάθε κέντρου). Θα πρέπει να κανονιστεί επίσκεψη στον ιατρό πρωτοβάθμιας περίθαλψης καθώς και σε άλλες ειδικότητες αν χρειαστεί.
7.3	**Διατροφική κατάσταση.**<br>Αξιολογήστε την πρόσληψη θερμίδων, πρωτεϊνών, μετάλλων και βιταμινών ανάλογα με τις ανάγκες

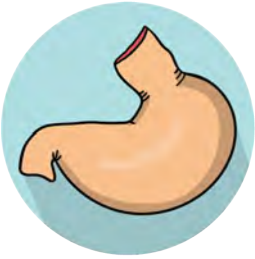

Πρωτόκολλο Eupemen
για Γαστρεκτομή

1	Πριν την εισαγωγή<br><br>Αναισθησιολόγος, Χειρουργός, Νοσηλευτής, Διαιτολόγος
1.1	**Προεγχειρητική συμβουλευτική**<br>Ο ασθενής θα πρέπει να είναι πλήρως ενημερωμένος για τη διαδικασία και την περιεγχειρητική πορεία τόσο προφορικά όσο και γραπτά. Θα πρέπει να λαμβάνεται υπογεγραμμένη ενημερωμένη συγκατάθεση.
1.2	**Ολοκληρωμένη ιατρική αξιολόγηση**<br>Αυτό θα πρέπει να περιλαμβάνει ιατρικό ιστορικό, φυσική εξέταση, ακτινογραφία θώρακα, εξετάσεις αίματος (έλεγχος πήξης, βιοχημικό προφίλ, διατροφικό προφίλ και γενική αίματος) και ηλεκτροκαρδιογράφημα.
1.3	**Ρύθμιση χρόνιων παθήσεων**<br>Όλες οι χρόνιες παθήσεις θα πρέπει να βελτιστοποιούνται πριν από την επέμβαση. Προεγχειρητική σπιρομέτρηση πρέπει να γίνεται σε ασθενείς με περιοριστική πνευμονοπάθεια. Καρδιολογική αξιολόγηση εάν ο παράγοντας καρδιαγγειακού κινδύνου είναι μεγαλύτερος από 3. Όλες οι περιπτώσεις πρόσφατης έναρξης ή ενεργών καρδιαγγειακών παθήσεων θα πρέπει να αξιολογούνται από καρδιολόγο.
1.4	**Αξιολόγηση και θεραπεία σιδηροπενίας και προεγχειρητικής αναιμίας**<br>Η σιδηροπενική αναιμία θα πρέπει να αντιμετωπίζεται ιδανικά με παρεντερική χορήγηση σιδήρου.
1.5	**Αξιολόγηση Σακχαρώδη Διαβήτη**<br>Τα επίπεδα γλυκόζης αίματος και γλυκοζηλιωμένης αιμοσφαιρίνης (HbA1c) θα πρέπει να εκτιμηθούν. Όλες οι περιπτώσεις φτωχά ελεγχόμενου ή προηγουμένως αδιάγνωστου διαβήτη θα πρέπει να παραπέμπονται στην πρωτοβάθμια φροντίδα ή σε ενδοκρινολογο / διαβητολόγο πριν από τη χειρουργική επέμβαση.
1.6	**Έλεγχος για υπνική άπνοια**<br>Έλεγχος για υπνική άπνοια με το τεστ STOP-BANG. Εκτελέστε μελέτη ύπνου εάν η βαθμολογία είναι μεγαλύτερη από 3.
1.7	**Διατροφική βελτιστοποίηση**<br>Αξιολογήστε τη διατροφική κατάσταση με το εργαλείο MUST (Malnutrition Universal Screening Tool). Διορθώστε τις προεγχειρητικές διατροφικές ελλείψεις συμπεριλαμβανομένων του ασβεστίου, του σιδήρου, της βιταμίνης D και της βιταμίνης B12. Για ασθενείς με αφαγία, αξιολογήστε πιθανές οδούς

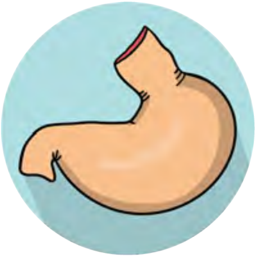

	χορήγησης τεχνητής διατροφής. Για δυσφαγία σε στερεά χορηγείστε υγρές δίαιτες με συμπληρώματα πρωτεΐνης υψηλής θερμιδικής αξίας.
1.8	**Διακόψτε το κάπνισμα και μειώστε την κατανάλωση αλκοόλ**<br>Η χρήση του καπνού θα πρέπει να σταματήσει και η κατανάλωση αλκοόλ θα πρέπει να μειωθεί αμέσως μόλις γίνει η διάγνωση.
1.9	**Φυσική ασκήση**<br>Ασκήσεις καρδιαγγειακής, αναπνευστικής και μυϊκής ενδυνάμωσης προσαρμοσμένες στη φυσική κατάσταση του ασθενούς.
1.10	**Ψυχολογική συμβουλευτική**<br>Οποιαδήποτε ψυχολογικά προβλήματα μπορεί να αντιμετωπίζει ο ασθενής θα πρέπει να αντιμετωπίζονται πλήρως.
1.11	**Κλίμακα Apfel**<br>Ο κίνδυνος για μετεγχειρητική ναυτία και έμετο θα πρέπει να αξιολογείται με τη κλίμακα Apfel.
1.12	**Αξιολόγηση κατάταξης στην κλίμακα ASA**<br>Ως μέρος της προεγχειρητικής αναισθησιολογικής αξιολόγησης θα πρέπει να εκτιμάται η κατάταξη στην κλίμακα ASA (American Society of Anesthesiologists).
2	Περιεγχειρητικά
2.1	Άμεσα Προεγχειρητικά<br>(Προγραμματίστε την εισαγωγή την ίδια ημέρα της επέμβασης, εάν είναι δυνατόν)<br><br>Αναισθησιολόγος, Χειρουργός, Νοσηλευτής
2.1.1	**Προεγχειρητική νηστεία**<br>Θα πρέπει να επιτρέπεται στους ασθενείς να τρώνε στερεά τροφή μέχρι 8 ώρες πριν το χειρουργείο και να πίνουν υγρά μέχρι 2 ώρες πριν το χειρουργείο.
2.1.2	**Ηπαρίνη χαμηλού μοριακού βάρους**<br>Η ηπαρίνη χαμηλού μοριακού βάρους πρέπει να χορηγείται 2-12 ώρες πριν από την επέμβαση (ανάλογα με το εάν πρόκειται να γίνει νευραξονική αναισθησία ή όχι).

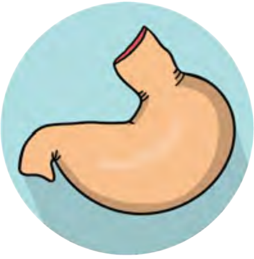

2.1.3	**Κάλτσες συμπίεσης**<br>Τοποθέτηση καλτσών συμπίεσης ή διαλείπουσα πνευματική συμπίεση, ανάλογα με τον κίνδυνο θρομβοεμβολισμού.
2.1.4	**Πόσιμο διάλυμα υδατανθράκων**<br>Ένα ρόφημα με υδατάνθρακες (12,5% μαλτοδεξτρίνες) 400 ml θα πρέπει να χορηγείται 2 ώρες πριν από την αναισθησία εάν δεν υπάρχει αντένδειξη.
2.1.5	**Αποφύγετε την αγχολυτική προφαρμακευτική αγωγή**<br>Μην προσθέτετε προεγχειρητική αγχολυτική προφαρμακευτική αγωγή (προνάρκωση).
2.1.6	**Ξύρισμα με ηλεκτρικό ξυράφι**<br>Το σημείο όπου θα γίνει η τομή θα πρέπει να ξυριστεί με ηλεκτρική ξυριστική μηχανή, εάν είναι απαραίτητο.
2.1.7	**Αντιβιοτική προφύλαξη**<br>Η αντιβιοτική προφύλαξη πρέπει να χορηγείται 30-60 λεπτά πριν από τη χειρουργική τομή. Η επιλογή του αντιβιοτικού θα πρέπει να γίνεται με βάση το τοπικό νοσοκομειακό πρωτόκολλο
2.1.8	**Προφυλακτικά μέτρα για την πρόληψη της γαστρικής παλινδρόμησης**<br>Για ασθενείς με καθυστερημένη γαστρική κένωση πρέπει να λαμβάνονται προφυλακτικά μέτρα για την πρόληψη της παλινδρόμησης.
2.2	Διεγχειρητικά<br><br>Αναισθησιολόγος, Χειρουργός, Νοσηλευτής
2.2.1	**Λίστα ελέγχου χειρουργικής ασφάλειας του ΠΟΥ (Παγκόσμιου Οργανσιμού Υγείας)**<br>Η λίστα ελέγχου χειρουργικής ασφάλειας του ΠΟΥ θα πρέπει να συμπληρωθεί πριν γίνει η τομή.
2.2.2	**Συστηματική διεγχειρητική παρακολούθηση**<br>Οι ζωτικές λειτουργίες, το FiO2, το βάθος της αναισθησίας, ο νευρομυϊκός αποκλεισμός και η γλυκαιμία θα πρέπει να παρακολουθούνται κατά τη διάρκεια της διαδικασίας. Συνιστάται επίσης η μη επεμβατική αιμοδυναμική παρακολούθηση.
2.2.3	**Αποφύγετε τους αρτηριακούς καθετήρες**<br>Δεν απαιτείται συνήθως επεμβατική τοποθέτηση αρτηριακού καθετήρα, αν και θα πρέπει να χρησιμοποιείται σε ασθενείς με σοβαρές καρδιοαναπνευστικές διαταραχές.

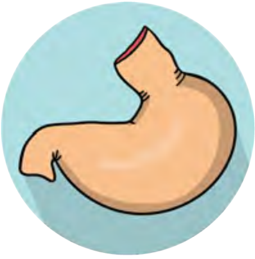

2.2.4	**Αποφύγετε τους κεντρικούς φλεβικούς καθετήρες**<br>Οι κεντρικοί φλεβικοί καθετήρες δεν απαιτούνται συνήθως για μικρές εκτομές και σε απουσία παραγόντων κινδύνου για μετεγχειρητική νεφρική ανεπάρκεια.
2.2.5	**Αποφύγετε τον συστηματικό καθετηριασμό της ουροδόχου κύστης**
2.2.6	**Εισαγωγή και διατήρηση της αναισθησίας**<br>Παράγοντες βραχείας δράσης θα πρέπει να χρησιμοποιούνται για την εισαγωγή και τη διατήρηση της αναισθησίας.
2.2.7	**Οξυγόνωση**<br>Οι ασθενείς θα πρέπει να λαμβάνουν οξυγόνο με FiO2 άνω του 50%.
2.2.8	**Χορήγηση Υγρών**<br>Συνιστάται αιμοδυναμική βελτιστοποίηση με στοχοκατευθυνόμενη χορήγηση υγρών με τη χρήση συσκευών παρακολούθησης του αιμοδυναμικού προφίλ του ασθενούς. Εάν αυτές δεν είναι διαθέσιμες, συνιστάται περιορισμένη χορήγηση υγρών με βάση το ιδανικό βάρος.
2.2.9	**Πρόληψη της υποθερμίας**<br>Η θερμοκρασία θα πρέπει να παρακολουθείται και η νορμοθερμία πρέπει να διατηρείται με ενεργητική θέρμανση (θερμά υγρά, θερμαινόμενη κουβέρτα).
2.2.10	**Προφύλαξη μετεγχειρητικής ναυτίας και εμέτου**<br>Χορηγήστε αντιεμετική θεραπεία σύμφωνα με την κλίμακα Apfel.
2.2.11	**Επισκληρίδιος αναλγησία**<br>Η θωρακική επισκληρίδιος αναλγησία πρέπει να χρησιμοποιείται σε ανοιχτή χειρουργική επέμβαση. Στη λαπαροσκοπική χειρουργική δεν συνιστάται συνήθως. Ασθενείς με αντένδειξη για επισκληρίδιο αναλγησία που έχουν κίνδυνο μετεγχειρητικής νεφρικής ανεπάρκειας ή έχουν διαταραχές πήξης θα μπορούσαν να ωφεληθούν από αμφοτερόπλευρο αποκλεισμό στο επίπεδο του Εγκάρσιου Κοιλιακού Μυός (TAP BLOCK - transabdominal plan blocks) ή άλλες εναλλακτικές λύσεις αντί της επισκληρίδιου αναλγησίας.
2.2.12	**Ελάχιστα επεμβατική χειρουργική**<br>Προτιμώνται οι ελάχιστα επεμβατικές προσπελάσεις και θα πρέπει να χρησιμοποιούνται όσο το δυνατόν περισσότερο.
2.2.13	**Αποφύγετε τα ενισχυτικά συρραπτικών και τις βιολογικές κόλλες**

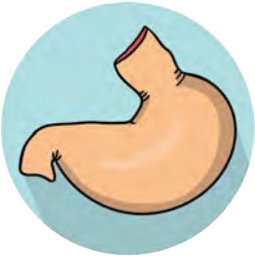

	Οι μέθοδοι ενίσχυσης της γραμμής συρραφής, όπως η χρήση ενισχυτικών συρραφής ή βιολογικών κόλλων, δεν μειώνουν τις διαφυγές σύμφωνα με δεδομένα.
2.2.14	**Βαθμονόμηση της κάθετης (επιμήκους) (sleeve) γαστρεκτομής**<br>Η κάθετη (επιμήκης) (sleeve) γαστρεκτομή πρέπει να βαθμονομηθεί με οδηγούς (probes).
2.2.15	**Αποφύγετε τους ρινογαστρικούς σωλήνες**<br>Οι ρινογαστρικοί σωλήνες συνιστώνται μόνο διεγχειρητικά για να αδειάσει το στομάχι.
2.2.16	**Αποφύγετε τις παροχετεύσεις**
2.3	Άμεσα Μετεγχειρητικά<br><br>Αναισθησιολόγος, Νοσηλευτής
2.3.1	**Διατήρηση της νορμοθερμίας**<br>Η θερμοκρασία πρέπει να μετράται τακτικά και να διατηρείται η νορμοθερμία.
2.3.2	**Αναλγησία με περιορισμό των οπιοειδών**<br>Θα πρέπει να χρησιμοποιείται προληπτική πολυπαραγοντική αναλγησία. Περιορίστε τη χρήση οπιοειδών. Στοχεύστε σε βαθμολογία πόνου στην οπτική αναλογική κλίμακα (Visual Analog Scale – VAS) μικρότερη από 3.
2.3.3	**Πρώιμη σίτιση**<br>Έναρξη λήψης υγρών από το στόμα 6 ώρες μετά το χειρουργείο.
2.3.4	**Πρώιμη κινητοποίηση**<br>Η κινητοποίηση θα πρέπει να ξεκινά 3 ώρες μετά το χειρουργείο και θα πρέπει να ξεκινά με το κάθετο στο κρεβάτι. Η βάδιση θα πρέπει να ξεκινά 6 ώρες μετά την επέμβαση λαμβάνοντας υπόψιν πάντα τις ώρες του νυχτερινού ύπνου.
2.3.5	**Θρομβοεμβολική προφύλαξη**<br>Η ηπαρίνη χαμηλού μοριακού βάρους πρέπει να χορηγείται 12 ώρες μετά την επέμβαση.
2.3.6	**Προφύλαξη μετεγχειρητικής ναυτίας και εμέτου**<br>Χορηγήστε αντιεμετική θεραπεία σύμφωνα με την κλίμακα Apfel.
2.3.7	**Θεραπεία υπνικής άπνοιας**

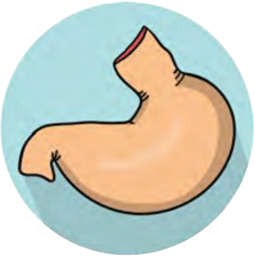

Πρωτόκολλο Eupemen
για Γαστρεκτομή

	Σε ασθενείς με υπνική άπνοια, επαναχρησιμοποιείστε τη Συνεχή Θετική Πίεση των Αεραγωγών (CPAP) το συντομότερο δυνατό.
3	1η μετεγχειρητική ημέρα<br>(Θάλαμος Νοσηλείας)<br><br>Χειρουργός, Νοσηλευτής
3.1	**Πρώιμη σίτιση**<br>Μια υδρική δίαιτα πρέπει να ξεκινά ανάλογα με την ανοχή του ασθενούς.
3.2	**Πρώιμη κινητοποίηση**<br>Οι ασθενείς θα πρέπει να ενθαρρύνονται να περπατούν.
3.3	**Αναλγησία με περιορισμό των οπιοειδών**<br>Θα πρέπει να χρησιμοποιείται ενεργητική ή προληπτική πολυπαραγοντική αναλγησία. Περιορίστε τη χρήση οπιοειδών. Στοχεύστε σε βαθμολογία πόνου στην οπτική αναλογική κλίμακα (Visual Analog Scale – VAS) μικρότερη από 3.
3.4	**Σταματήστε την ενδοφλέβια χορήγηση υγρών**<br>Εάν οι ασθενείς ανέχονται επαρκώς τα υγρά από του στόματος, διακόψτε την ενδοφλέβια χορήγηση υγρών.
3.5	**Αφαιρέστε τον ουροκαθετήρα**<br>Εάν έχει τοποθετηθεί ουροκαθετήρας, εξετάστε το ενδεχόμενο αφαίρεσής του
3.6	**Αφαίρεση παροχετεύσεων**<br>Εξετάστε το ενδεχόμενο αφαίρεσής των παροχετεύσεων, αν υπάρχουν
3.7	**Θρομβοεμβολική προφύλαξη**
3.8	**Αναπνευστική φυσιοθεραπεία**
4	2η μετεγχειρητική ημέρα<br>(Θάλαμος Νοσηλείας)<br><br>Χειρουργός, Νοσηλευτής
4.1	**Πρώιμη σίτιση**<br>Δώστε στους ασθενείς ημι-στερεές δίαιτες (πουρές, γιαούρτια κ.λπ.).
4.2	**Πρώιμη κινητοποίηση**<br>Οι ασθενείς θα πρέπει να ενθαρρύνονται να περπατούν.

EUPEMEN
European Perioperative Medical Networking

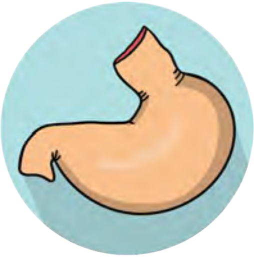

4.3	**Αναλγησία με περιορισμό των οπιοειδών**<br>Θα πρέπει να χρησιμοποιείται ενεργητική ή προληπτική πολυπαργοντική αναλγησία. Περιορίστε τη χρήση οπιοειδών. Στοχεύστε σε βαθμολογία πόνου στην οπτική αναλογική κλίμακα (Visual Analog Scale – VAS) μικρότερη από 3.
4.4	**Αφαιρέστε τον επισκληρίδιο καθετήρα**<br>Ελέγξτε πρώτα τις παραμέτρους πήξης και βεβαιωθείτε ότι έχει αφαιρεθεί ολόκληρος ο καθετήρας.
4.5	**Αναπνευστική και λειτουργική φυσιοθεραπεία**
4.6	**Θρομβοεμβολική προφύλαξη**
5	3η μετεγχειρητική ημέρα<br>(Θάλαμος Νοσηλείας)<br><br>Χειρουργός, Νοσηλευτής
5.1	**Πρώιμη σίτιση**<br>Δίαιτα με αλεσμένες τροφές
5.2	**Πρώιμη κινητοποίηση**<br>Οι ασθενείς θα πρέπει να ενθαρρύνονται να περπατούν.
5.3	**Αναλγησία με περιορισμό των οπιοειδών**<br>Θα πρέπει να χρησιμοποιείται ενεργητική ή προληπτική πολυπαργοντική αναλγησία. Περιορίστε τη χρήση οπιοειδών. Στοχεύστε σε βαθμολογία πόνου στην οπτική αναλογική κλίμακα (Visual Analog Scale – VAS) μικρότερη από 3.
5.4	**Αναπνευστική και λειτουργική φυσιοθεραπεία**
5.5	**Θρομβοεμβολική προφύλαξη**
5.6	**Εργαστηριακές εξετάσεις αίματος**<br>Αυτές θα πρέπει να περιλαμβάνουν C-αντιδρώσα πρωτεΐνη, προκαλσιτονίνη και γενική αίματος.
5.7	**Εξιτήριο**<br>Εξετάστε το ενδεχόμενο εξιτηρίου εάν πληρούνται τα παρακάτω κριτήρια: χωρίς χειρουργικές επιπλοκές, χωρίς πυρετό, πόνος ελεγχόμενος με με από

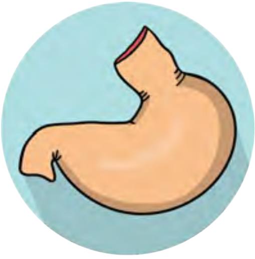

	του στόματος αναλγησία, πλήρης κινητοποίηση, ανοχή στη λήψη τροφής από το στόμα και εάν ο ασθενής συμφωνεί.
6	4η μετεγχειρητική ημέρα<br>(Θάλαμος Νοσηλείας)<br><br>Χειρουργός, Νοσηλευτής
6.1	**Πρώιμη σίτιση**<br>Ελαφρά δίαιτα.
6.2	**Πρώιμη κινητοποίηση**<br>Οι ασθενείς θα πρέπει να ενθαρρύνονται να περπατούν.
6.3	**Αναλγησία με περιορισμό των οπιοειδών**<br>Θα πρέπει να χρησιμοποιείται ενεργητική ή προληπτική πολυπαργοντική αναλγησία. Περιορίστε τη χρήση οπιοειδών. Στοχεύστε σε βαθμολογία πόνου στην οπτική αναλογική κλίμακα (Visual Analog Scale – VAS) μικρότερη από 3.
6.4	**Αναπνευστική και λειτουργική φυσιοθεραπεία**
6.5	**Θρομβοεμβολική προφύλαξη**
6.6	**Εργαστηριακές εξετάσεις αίματος**<br>Αυτές θα πρέπει να περιλαμβάνουν C-αντιδρώσα πρωτεΐνη, προκαλσιτονίνη και γενική αίματος.
6.7	**Εξιτήριο**<br>Εξετάστε το ενδεχόμενο εξιτηρίου εάν πληρούνται τα παρακάτω κριτήρια: χωρίς χειρουργικές επιπλοκές, χωρίς πυρετό, πόνος ελεγχόμενος με με από του στόματος αναλγησία, πλήρης κινητοποίηση, ανοχή στη λήψη τροφής από το στόμα και εάν ο ασθενής συμφωνεί.
7	Εξιτήριο<br><br>Χειρουργός, Νοσηλευτής, Ψυχολόγος, Πρωτοβάθμια Περίθαλψη
7.1	**Ενημερωτικό σημείωμα για τον ασθενή**<br>Παρέχετε στους ασθενείς πληροφορίες για την ιατρική τους κατάσταση και δίνετε συστάσεις για περαιτέρω φροντίδα.

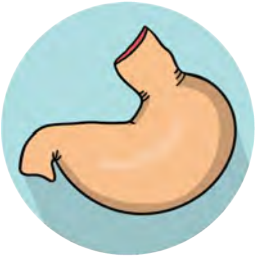

7.2	**Συνεχής φροντίδα**<br>Τηλεφωνικός έλεγχος μετά το εξιτήριο. Συντονισμός υποστήριξης στο σπίτι με την πρωτοβάθμια περίθαλψη
7.3	**Διατροφική κατάσταση.**<br>Αξιολογήστε την πρόσληψη θερμίδων, πρωτεϊνών, μετάλλων και βιταμινών ανάλογα με τις ανάγκες.
7.4	**Ψυχολογική συμβουλευτική**<br>Παραπέμψτε τους ασθενείς σε ειδικούς ψυχολόγους εάν είναι απαραίτητο. Αξιολογήστε την μετεγχειρητική ποιότητα ζωής.

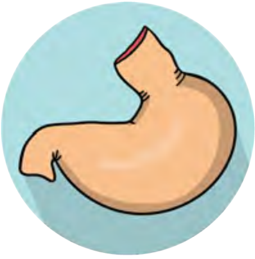

Πρωτόκολλο Eupemen
για Ηπατεκτομή

1	Πριν την εισαγωγή<br>Αναισθησιολόγος, Χειρουργός, Νοσηλευτής, Διαιτολόγος
1.1	**Προεγχειρητική συμβουλευτική**<br>Ο ασθενής θα πρέπει να είναι πλήρως ενημερωμένος για τη διαδικασία και την περιεγχειρητική πορεία τόσο προφορικά όσο και γραπτά. Θα πρέπει να λαμβάνεται υπογεγραμμένη ενημερωμένη συγκατάθεση.
1.2	**Ολοκληρωμένη ιατρική αξιολόγηση**<br>Αυτό θα πρέπει να περιλαμβάνει ιατρικό ιστορικό, φυσική εξέταση, ακτινογραφία θώρακα, εξετάσεις αίματος (έλεγχος πήξης, βιοχημικό προφίλ, γενική αίματος) και ηλεκτροκαρδιογράφημα.
1.3	**Ρύθμιση χρόνιων παθήσεων**<br>Όλες οι χρόνιες παθήσεις θα πρέπει να βελτιστοποιούνται πριν από την επέμβαση. Όλες οι περιπτώσεις πρόσφατης έναρξης ή ενεργών καρδιαγγειακών παθήσεων θα πρέπει να αξιολογούνται από καρδιολόγο.
1.4	**Αξιολόγηση Σακχαρώδη Διαβήτη**<br>Τα επίπεδα γλυκόζης αίματος και γλυκοζηλιωμένης αιμοσφαιρίνης (HbA1c) θα πρέπει να εκτιμηθούν. Όλες οι περιπτώσεις φτωχά ελεγχόμενου ή προηγουμένως αδιάγνωστου διαβήτη θα πρέπει να παραπέμπονται στην πρωτοβάθμια φροντίδα ή σε ενδοκρινολογο / διαβητολόγο πριν από τη χειρουργική επέμβαση.
1.5	**Αξιολόγηση και διαχείριση της αναιμίας και της ανεπάρκειας σιδήρου**<br>Η σιδηροπενική αναιμία θα πρέπει να αντιμετωπίζεται ιδανικά με παρεντερική χορήγηση σιδήρου.
1.6	**Διατροφικός έλεγχος**<br>Ο διατροφικός έλεγχος θα πρέπει να γίνεται χρησιμοποιώντας το εργαλείο MUST (Malnutrition Universal Screening Tool).
1.7	**Διακόψτε το κάπνισμα και μειώστε την κατανάλωση αλκοόλ**
1.8	**Καρδιαγγειακές ασκήσεις**<br>Καρδιαγγειακές και αναπνευστικές ασκήσεις προσαρμοσμένες στη φυσική κατάσταση του ασθενούς.
1.9	**Ψυχολογική συμβουλευτική**<br>Οποιαδήποτε ψυχολογικά προβλήματα μπορεί να αντιμετωπίζει ο ασθενής θα πρέπει να αντιμετωπίζονται πλήρως.

EUPEMEN
European Perioperative Medical Networking

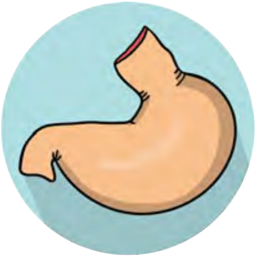

1.10	**Αξιολόγηση ευπάθειας**<br>Για ασθενείς ηλικίας άνω των 65 ετών θα πρέπει να γίνεται αξιολόγηση ευπάθειας
1.11	**Κλιμάκα Apfel**<br>Ο κίνδυνος για μετεγχειρητική ναυτία και έμετο θα πρέπει να αξιολογείται με την κλίμακα Apfel.
1.12	**Αξιολόγηση κατάταξης στην κλίμακα ASA**<br>Ως μέρος της προεγχειρητικής αναισθησιολογικής αξιολόγησης θα πρέπει να εκτιμάται η κατάταξη στην κλίμακα ASA (American Society of Anesthesiologists).
2	Περιεγχειρητικά
2.1	Άμεσα Προεγχειρητικά<br><br>Αναισθησιολόγος, Χειρουργός, Νοσηλευτής
2.1.1	**Προεγχειρητική υγιεινή**<br>Ο ασθενής λαμβάνει οδηγίες να κάνει πλήρες ντους ή μπάνιο το βράδυ ή το πρωί πριν από την επέμβαση.
2.1.2	**Κάλτσες συμπίεσης ή διαλείπουσα πνευματική συμπίεση.**<br>Οι κάλτσες συμπίεσης ή η διαλείπουσα πνευματική συμπίεση πρέπει να τοποθετούνται από την εισαγωγή στο νοσοκομείο.
2.1.3	**Ηπαρίνη χαμηλού μοριακού βάρους**<br>Η ηπαρίνη χαμηλού μοριακού βάρους πρέπει να χορηγείται 2-12 ώρες πριν από την επέμβαση (ανάλογα με το εάν πρόκειται να γίνει νευραξονική αναισθησία ή όχι).
2.1.4	**Πόσιμο διάλυμα υδατανθράκων**<br>Ένα ρόφημα πλούσιο σε υδατάνθρακες (12,5% μαλτοδεξτρίνες) 800 ml πρέπει να χορηγείται το βράδυ πριν από την επέμβαση και 400 ml 2 ώρες πριν από την αναισθησία. Για διαβητικούς ασθενείς χορηγήστε το μαζί με αντιδιαβητική αγωγή.
2.1.5	**Προεγχειρητική νηστεία**<br>Νηστεία 6 ωρών για τα στερεά και 2 ωρών για τα διαυγή υγρά.
2.1.6	**Ξύρισμα με ηλεκτρικό ξυράφι**<br>Το σημείο όπου θα γίνει η τομή θα πρέπει να ξυριστεί με ηλεκτρική ξυριστική μηχανή, εάν είναι απαραίτητο.

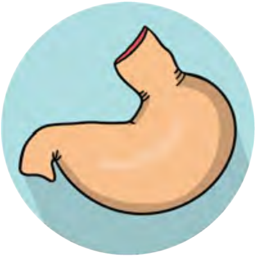

2.1.7	**Αντιβιοτική χημειοπροφύλαξη**<br>Προφυλακτική χορήγηση αντιβιοτικού 30-60 λεπτά πριν την τομή. Σε παρατεταμένες διαδικασίες επαναλάβετε τις δόσεις ανάλογα με τον χρόνο ημιζωής των φαρμάκων.
2.2	Διεγχειρητικά<br><br>Αναισθησιολόγος, Χειρουργός, Νοσηλευτής
2.2.1	**Λίστα ελέγχου χειρουργικής ασφάλειας του ΠΟΥ (Παγκόσμιου Οργανσιμού Υγείας)**<br>Η λίστα ελέγχου χειρουργικής ασφάλειας του ΠΟΥ θα πρέπει να συμπληρωθεί πριν γίνει η τομή.
2.2.2	**Συστηματική διεγχειρητική παρακολούθηση**<br>Οι ζωτικές λειτουργίες, το FiO2, το βάθος της αναισθησίας, ο νευρομυϊκός αποκλεισμός και η γλυκαιμία θα πρέπει να παρακολουθούνται κατά τη διάρκεια της διαδικασίας. Συνιστάται επίσης η μη επεμβατική αιμοδυναμική παρακολούθηση.
2.2.3	**Ελάχιστα επεμβατική χειρουργική**<br>Προτιμώνται οι ελάχιστα επεμβατικές προσπελάσεις και θα πρέπει να χρησιμοποιούνται όσο το δυνατόν περισσότερο.
2.2.4	**Αποφύγετε τον συστηματικό καθετηριασμό της ουροδόχου κύστης**
2.2.5	**Επεμβατική παρακολούθηση**<br>Δεν απαιτείται συνήθως τοποθέτηση αρτηριακού καθετήρα. Αν και θα πρέπει να χρησιμοποιείται σε ασθενείς με σοβαρές καρδιοαναπνευστικές διαταραχές.
2.2.6	**Κεντρικός φλεβικός καθετήρας**<br>Οι κεντρικοί φλεβικοί καθετήρες δεν απαιτούνται συνήθως για μικρές εκτομές και απουσία παραγόντων κινδύνου για μετεγχειρητική νεφρική ανεπάρκεια.
2.2.7	**Εισαγωγή και διατήρηση της αναισθησίας**<br>Παράγοντες βραχείας δράσης θα πρέπει να χρησιμοποιούνται για την εισαγωγή και τη διατήρηση της αναισθησίας.
2.2.8	**Οξυγόνωση**<br>Οι ασθενείς θα πρέπει να λαμβάνουν οξυγόνο με FiO2 άνω του 50%.
2.2.9	**Χορήγηση Υγρών**<br>Η Αιμοδυναμική βελτιστοποίηση με στοχοκατευθυνόμενη χορήγηση υγρών με

	τη χρήση συσκευών παρακολούθησης του αιμοδυναμικού προφίλ του ασθενούς, συνιστάται σε ασθενείς υψηλού κινδύνου και σε ασθενείς που υποβάλλονται σε χειρουργική επέμβαση με μεγάλη απώλεια αίματος. Σε όλες τις άλλες περιπτώσεις, συνιστάται περιοριστική χορήγηση υγρών με βάση το ιδανικό βάρος με συνεχή χορήγηση ισορροπημένου κρυσταλλοειδούς διάλυματος (1-3 ml/kg/h για λαπαροσκόπηση, 3-5 ml/kg/h για λαπαροτομία). Η απώλεια αίματος θα πρέπει να αντισταθμίζεται με κολλοειδή 1:1.
2.2.10	**Αποφύγετε το ρινογαστρικό σωλήνα**<br>Οι ρινογαστρικοί σωλήνες δεν πρέπει να χρησιμοποιούνται τακτικά.
2.2.11	**Πρόληψη της υποθερμίας**<br>Η θερμοκρασία θα πρέπει να παρακολουθείται και η νορμοθερμία πρέπει να διατηρείται με ενεργητική θέρμανση (θερμά υγρά, θερμαινόμενη κουβέρτα).
2.2.12	**Προφύλαξη μετεγχειρητικής ναυτίας και εμέτου**<br>Χορηγήστε αντιεμετική θεραπεία σύμφωνα με την κλίμακα Apfel.
2.2.13	**Επισκληρίδιος αναλγησία**<br>Η θωρακική επισκληρίδιος αναλγησία πρέπει να χρησιμοποιείται σε ανοιχτή χειρουργική επέμβαση. Στη λαπαροσκοπική χειρουργική δεν συνιστάται συνήθως. Ασθενείς με αντένδειξη για επισκληρίδιο αναλγησία που έχουν κίνδυνο μετεγχειρητικής νεφρικής ανεπάρκειας ή έχουν διαταραχές πήξης θα μπορούσαν να ωφεληθούν από αμφοτερόπλευρο αποκλεισμό στο επίπεδο του Εγκάρσιου Κοιλιακού Μυός (TAP BLOCK - transabdominal plan blocks), ή άλλες εναλλακτικές λύσεις αντί της επισκληρίδιου αναλγησίας.
2.2.14	**Ενδοφλέβια επικουρικά αναλγητικά φάρμακα**<br>Συνιστώμενα επικουρικά αναλγητικά είναι τα μη στεροειδή αντιφλεγμονώδη φάρμακα, η λιδοκαΐνη, η κεταμίνη, το θειικό μαγνήσιο και η δεξμεντετομιδίνη.
2.2.15	**Παρακολούθηση γλυκόζης αίματος**<br>Αποφύγετε τα επίπεδα γλυκόζης στο αίμα > 180 mg/dl σε ασθενείς που διατρέχουν κίνδυνο να αναπτύξουν αντίσταση στην ινσουλίνη.
2.2.16	**Απολύμανση δέρματος**<br>Το δέρμα πρέπει να απολυμαίνεται από κεντρικά προς περιφερειακά με αλκοολικό διάλυμα χλωρεξιδίνης 2%.
2.2.17	**Αποφύγετε τις παροχετεύσεις**<br>Οι κοιλιακές παροχετεύσεις πρέπει να αποφεύγονται όσο το δυνατόν περισσότερο.

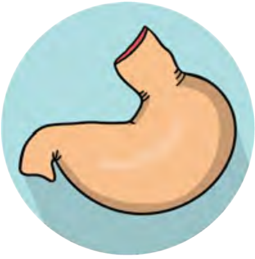

2.3	Άμεσα Μετεγχειρητικά<br>(Μονάδα Μετα-αναισθητικής Φροντίδας / Μονάδα Ενδιάμεσης Φροντίδας σε επιλεγμένες περιπτώσεις)<br><br>Αναισθησιολόγος, Νοσηλευτής
2.3.1	**Διατήρηση της νορμοθερμίας**<br>Η θερμοκρασία πρέπει να μετράται τακτικά και να διατηρείται η νορμοθερμία.
2.3.2	**Αναλγησία με περιορισμό των οπιοειδών**<br>Θα πρέπει να χρησιμοποιείται ενεργητική ή προληπτική πολυπαργοντική αναλγησία. Περιορίστε τη χρήση οπιοειδών. Στοχεύστε σε βαθμολογία πόνου στην οπτική αναλογική κλίμακα (Visual Analog Scale – VAS) μικρότερη από 3.
2.3.3	**Περιοριστική χορήγηση υγρών.**
2.3.4	**Πρώιμη σίτιση**<br>Έναρξη λήψης υγρών από το στόμα 6 - 8 ώρες μετά το χειρουργείο.
2.3.5	**Αναπνευστική φυσιοθεραπεία**
2.3.6	**Πρώιμη κινητοποίηση**<br>Η κινητοποίηση θα πρέπει να ξεκινά 3 ώρες μετά το χειρουργείο και θα πρέπει να ξεκινά με κάθισμα στο κρεβάτι.
2.3.7	**Θρομβοεμβολική προφύλαξη**<br>Η ηπαρίνη χαμηλού μοριακού βάρους πρέπει να χορηγείται 12 ώρες μετά την επέμβαση.
2.3.8	**Αντιμετώπιση μετεγχειρητικής ναυτίας και εμέτου**
2.3.9	**Διατήρηση FiO2 (κλάσμα εισπνεόμενου οξυγόνου) 0,5% για 2 ώρες μετά την επέμβαση**
3	1η Μετεγχειρητική ημέρα<br>(Θάλαμος Νοσηλείας)<br><br>Χειρουργός, Νοσηλευτής

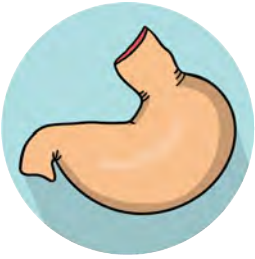

Πρωτόκολλο Eupemen
για Ηπατεκτομή

3.1	**Συμπλήρωμα διατροφής πλούσιο σε πρωτεΐνες**<br>Συμπληρώματα διατροφής πλούσια σε πρωτεΐνες θα πρέπει να χορηγούνται σε ασθενείς με από του στόματος λήψη <60% των ενεργειακών απαιτήσεων ή προεγχειρητικό υποσιτισμό.
3.2	**Πρώιμη σίτιση**<br>Θα πρέπει να δίνεται ημι-στερεή ή κανονική δίαιτα.
3.3	**Πρώιμη κινητοποίηση**<br>Οι ασθενείς θα πρέπει να ενθαρρύνονται να μετακινούνται από το κρεβάτι στην καρέκλα δίπλα στο κρεβάτι.
3.4	**Αφαίρεση παροχετεύσεων**<br>Εξετάστε το ενδεχόμενο αφαίρεσής των παροχετεύσεων, αν υπάρχουν
3.5	**Αναλγησία με περιορισμό των οπιοειδών**<br>Εξασφαλίστε καλό έλεγχο του πόνου. Στοχεύστε σε βαθμολογία πόνου στην οπτική αναλογική κλίμακα (Visual Analog Scale – VAS) μικρότερη από 3.
3.6	**Διακόψτε την ενδοφλέβια χορήγηση υγρών**<br>Εάν οι ασθενείς ανέχονται τα υγρά από του στόματος, διακόψτε την ενδοφλέβια χορήγηση υγρών.
3.7	**Αφαιρέστε τον ουροκαθετήρα**<br>Εάν έχει τοποθετηθεί ουροκαθετήρας, εξετάστε το ενδεχόμενο αφαίρεσής του
3.8	**Αναπνευστική φυσιοθεραπεία**
3.9	**Προφύλαξη μετεγχειρητικής ναυτίας και εμέτου**
3.10	**Προφύλαξη κατά του πεπτικού έλκους**
3.11	**Θρομβοεμβολική προφύλαξη**
3.12	**Αξιολογήστε το εξιτήριο από το νοσοκομείο**<br>Για ασθενείς που υποβλήθηκαν σε λαπαροσκοπικές επεμβάσεις αξιολογήστε τα κριτήρια εξιτηρίου.
4	2η Μετεγχειρητική ημέρα<br><br>Χειρουργός, Νοσηλευτής

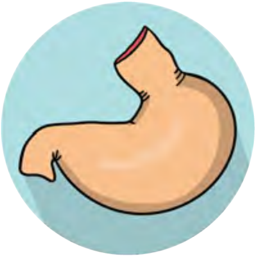

4.1	**Πρώιμη σίτιση**<br>Πρέπει να δίνεται κανονική δίαιτα.
4.2	**Πρώιμη κινητοποίηση**<br>Οι ασθενείς θα πρέπει να μπορούν να περπατούν μικρές αποστάσεις.
4.3	**Αναλγησία με περιορισμό των οπιοειδών**<br>Εξασφαλίστε καλό έλεγχο του πόνου. Στοχεύστε σε βαθμολογία πόνου στην οπτική αναλογική κλίμακα (Visual Analog Scale – VAS) μικρότερη από 3.
4.4	**Θρομβοεμβολική προφύλαξη**
4.5	**Εργαστηριακές εξετάσεις**<br>Θα πρέπει να γίνονται εξετάσεις αίματος συμπεριλαμβανομένης της C-αντιδρώσας πρωτεΐνης και της προκαλσιτονίνης.
4.6	**Αξιολογήστε τα κριτήρια εξιτηρίου**<br>Εξετάστε το ενδεχόμενο εξιτηρίου εάν δεν υπάρχουν χειρουργικές επιπλοκές που δεν μπορούν να αντιμετωπιστούν σε εξωτερικά ιατρεία, δεν υπάρχει πυρετός, πόνος ελεγχόμενος με από του στόματος αναλγησία, πλήρης κινητοποίηση, ανοχή στην από του στόματος λήψη τροφής και αποδοχή από τον ασθενή.
5	Εξιτήριο<br><br>Χειρουργός, Νοσηλευτής, Πρωτοβάθμια Περίθαλψη
5.1	**Έγγραφα κατά το εξιτήριο**<br>Κατά το εξιτήριο στους ασθενείς θα πρέπει να παρέχονται εξατομικευμένες, κατανοητές και πλήρεις πληροφορίες σχετικά με τη νοσηλεία τους στο νοσοκομείο και συστάσεις για την φροντίδα στο σπίτι.
5.2	**Παρακολούθηση**<br>Οι ασθενείς θα πρέπει να παρακολουθούνται τηλεφωνικά ή σε περιβάλλον εξωτερικών ασθενών μετά το εξιτήριο. Ο επανέλεγχος θα πρέπει να κανονιστεί εντός μιας εβδομάδας μετά το εξιτήριο και στη συνέχεια στους 1, 3 και 6 μήνες. Θα πρέπει να κανονιστεί επίσκεψη στον ιατρό πρωτοβάθμιας περίθαλψης και, εάν είναι απαραίτητο, θα πρέπει να συντονιστεί η υποστήριξη στο σπίτι.

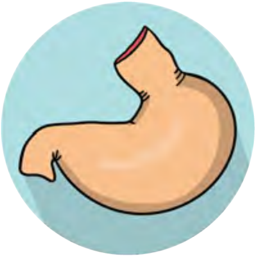

Πρωτόκολλο Eupemen
για Βαριατρική Χειρουργική

1	Πριν την εισαγωγή<br><br>Αναισθησιολόγος, Χειρουργός, Νοσηλευτής, Διαιτολόγος
1.1	**Προεγχειρητική συμβουλευτική**<br>Ο ασθενής θα πρέπει να είναι πλήρως ενημερωμένος για τη διαδικασία και την περιεγχειρητική πορεία τόσο προφορικά όσο και γραπτά. Θα πρέπει να λαμβάνεται υπογεγραμμένη ενημερωμένη συγκατάθεση.
1.2	**Ολοκληρωμένη ιατρική αξιολόγηση**<br>Αυτό θα πρέπει να περιλαμβάνει ιατρικό ιστορικό, φυσική εξέταση, ακτινογραφία θώρακα, εξετάσεις αίματος (έλεγχος πήξης, βιοχημικό προφίλ, διατροφικό προφίλ και γενική αίματος) και ηλεκτροκαρδιογράφημα.
1.3	**Ενδοσκόπηση ανώτερου πεπτικού συστήματος**<br>Η ενδοσκοπική αξιολόγηση του οισοφάγου, του στομάχου και του δωδεκαδακτύλου θα πρέπει να γίνεται ως μέρος της προεγχειρητικής προετοιμασίας. Αυτό θα πρέπει να περιλαμβάνει έλεγχο για ελικοβακτηρίδιο του πυλωρού, το οποίο εάν βρεθεί θα πρέπει να εκριζωθεί πριν από τη χειρουργική επέμβαση.
1.4	**Ρύθμιση χρόνιων παθήσεων**<br>Όλες οι χρόνιες παθήσεις θα πρέπει να βελτιστοποιούνται πριν από την επέμβαση. Προεγχειρητική σπιρομέτρηση πρέπει να γίνεται σε ασθενείς με περιοριστική πνευμονοπάθεια. Καρδιολογική αξιολόγηση εάν ο παράγοντας καρδιαγγειακού κινδύνου είναι μεγαλύτερος από 3. Όλες οι περιπτώσεις πρόσφατης έναρξης ή ενεργών καρδιαγγειακών παθήσεων θα πρέπει να αξιολογούνται από καρδιολόγο.
1.5	**Αξιολόγηση Σακχαρώδη Διαβήτη**<br>Τα επίπεδα γλυκόζης αίματος και γλυκοζηλιωμένης αιμοσφαιρίνης (HbA1c) θα πρέπει να εκτιμηθούν. Όλες οι περιπτώσεις φτωχά ελεγχόμενου ή προηγουμένως αδιάγνωστου διαβήτη θα πρέπει να παραπέμπονται στην πρωτοβάθμια φροντίδα ή σε ενδοκρινολογο / διαβητολόγο πριν από τη χειρουργική επέμβαση.
1.6	**Έλεγχος για υπνική άπνοια**<br>Έλεγχος για υπνική άπνοια με το τεστ STOP-BANG. Εκτελέστε μελέτη ύπνου εάν η βαθμολογία είναι μεγαλύτερη από 3.
1.7	**Διατροφική βελτιστοποίηση**<br>Απώλεια βάρους πριν από την επέμβαση χρησιμοποιώντας δίαιτα πολύ χαμηλών θερμίδων ή εμπορικά προϊόντα. Αξιολογήστε τις συμπληρωματικές μεθόδους απώλειας βάρους (φάρμακα, ενδογαστρικό μπαλόνι). Διόρθωση

	προεγχειρητικών διατροφικών ελλείψεων συμπεριλαμβανομένων ασβεστίου, σιδήρου, βιταμίνης D και βιταμίνης B12.
1.8	**Διακόψτε το κάπνισμα και μειώστε την κατανάλωση αλκοόλ**<br>Η χρήση του καπνού θα πρέπει να σταματήσει και η κατανάλωση αλκοόλ θα πρέπει να μειωθεί αμέσως μόλις γίνει η διάγνωση.
1.9	**Φυσική άσκηση**<br>Ασκήσεις καρδιαγγειακής, αναπνευστικής και μυϊκής ενδυνάμωσης προσαρμοσμένες στη φυσική κατάσταση του ασθενούς.
1.10	**Ψυχολογική συμβουλευτική**<br>Οποιαδήποτε ψυχολογικά προβλήματα μπορεί να αντιμετωπίζει ο ασθενής θα πρέπει να αντιμετωπίζονται πλήρως.
1.11	**Αξιολόγηση κατάταξης στην κλίμακα ASA**<br>Ως μέρος της προεγχειρητικής αναισθησιολογικής αξιολόγησης θα πρέπει να εκτιμάται η κατάταξη στην κλίμακα ASA (American Society of Anesthesiologists).
1.12	**Κλίμακα Apfel**<br>Ο κίνδυνος για μετεγχειρητική ναυτία και έμετο θα πρέπει να αξιολογείται με τη κλίμακα Apfel.
2	Περιεγχειρητικά
2.1	Άμεσα Προεγχειρητικά<br>(Προγραμματίστε την εισαγωγή την ίδια ημέρα της επέμβασης, εάν είναι δυνατόν)<br><br>Αναισθησιολόγος, Χειρουργός, Νοσηλευτής
2.1.1	**Προεγχειρητική νηστεία**<br>Θα πρέπει να επιτρέπεται στους ασθενείς να τρώνε στερεά τροφή μέχρι 8 ώρες πριν το χειρουργείο και να πίνουν υγρά μέχρι 2 ώρες πριν το χειρουργείο.
2.1.2	**Ηπαρίνη χαμηλού μοριακού βάρους**<br>Η ηπαρίνη χαμηλού μοριακού βάρους πρέπει να χορηγείται 2-12 ώρες πριν από την επέμβαση (ανάλογα με το εάν πρόκειται να γίνει νευραξονική αναισθησία ή όχι).
2.1.3	**Κάλτσες συμπίεσης**<br>Τοποθέτηση καλτσών συμπίεσης ή διαλείπουσα πνευματική συμπίεση,

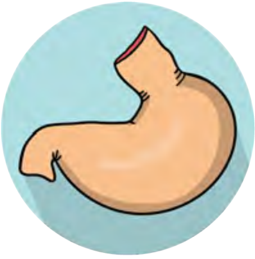

	ανάλογα με τον κίνδυνο θρομβοεμβολισμού.
2.1.4	**Πόσιμο διάλυμα υδατανθράκων**<br>Ένα ρόφημα με υδατάνθρακες (12,5% μαλτοδεξτρίνες) 400 ml θα πρέπει να χορηγείται 2 ώρες πριν από την αναισθησία εάν δεν υπάρχει αντένδειξη.
2.1.5	**Αποφύγετε την αγχολυτική προφαρμακευτική αγωγή**<br>Μην προσθέτετε προεγχειρητική αγχολυτική προφαρμακευτική αγωγή (προνάρκωση).
2.1.6	**Ξύρισμα με ηλεκτρικό ξυράφι**<br>Το σημείο όπου θα γίνει η τομή θα πρέπει να ξυριστεί με ηλεκτρική ξυριστική μηχανή, εάν είναι απαραίτητο.
2.1.7	**Αντιβιοτική προφύλαξη**<br>Η αντιβιοτική προφύλαξη πρέπει να χορηγείται 30-60 λεπτά πριν από τη χειρουργική τομή. Η επιλογή του αντιβιοτικού θα πρέπει να γίνεται με βάση το τοπικό νοσοκομειακό πρωτόκολλο.
2.1.8	**Προφυλακτικά μέτρα για την πρόληψη της γαστρικής παλινδρόμησης**<br>Για ασθενείς με καθυστερημένη γαστρική κένωση πρέπει να λαμβάνονται προφυλακτικά μέτρα για την πρόληψη της παλινδρόμησης.
2.2	Διεγχειρητικά<br><br>Αναισθησιολόγος, Χειρουργός, Νοσηλευτής
2.2.1	**Λίστα ελέγχου χειρουργικής ασφάλειας του ΠΟΥ (Παγκόσμιου Οργανσιμού Υγείας)**<br>Η λίστα ελέγχου χειρουργικής ασφάλειας του ΠΟΥ θα πρέπει να συμπληρωθεί πριν γίνει η τομή.
2.2.2	**Συστηματική διεγχειρητική παρακολούθηση**<br>Οι ζωτικές λειτουργίες, το FiO2, το βάθος της αναισθησίας, ο νευρομυϊκός αποκλεισμός και η γλυκαιμία θα πρέπει να παρακολουθούνται κατά τη διάρκεια της διαδικασίας. Συνιστάται επίσης η μη επεμβατική αιμοδυναμική παρακολούθηση.
2.2.3	**Αποφύγετε τους αρτηριακούς καθετήρες**<br>Δεν απαιτείται συνήθως επεμβατική τοποθέτηση αρτηριακού καθετήρα, αν και θα πρέπει να χρησιμοποιείται σε ασθενείς με σοβαρές καρδιοαναπνευστικές διαταραχές.
2.2.4	**Αποφύγετε τους κεντρικούς φλεβικούς καθετήρες**

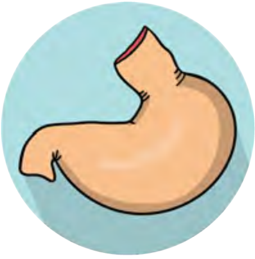

	Οι κεντρικοί φλεβικοί καθετήρες δεν απαιτούνται συνήθως για μικρές εκτομές και σε απουσία παραγόντων κινδύνου για μετεγχειρητική νεφρική ανεπάρκεια.
2.2.5	**Αποφύγετε τον συστηματικό καθετηριασμό της ουροδόχου κύστης**
2.2.6	**Εισαγωγή και διατήρηση της αναισθησίας**<br>Παράγοντες βραχείας δράσης θα πρέπει να χρησιμοποιούνται για την εισαγωγή και τη διατήρηση της αναισθησίας.
2.2.7	**Οξυγόνωση**<br>Οι ασθενείς θα πρέπει να λαμβάνουν οξυγόνο με FiO2 άνω του 50%.
2.2.8	**Χορήγηση Υγρών**<br>Συνιστάται αιμοδυναμική βελτιστοποίηση με στοχοκατευθυνόμενη χορήγηση υγρών με τη χρήση συσκευών παρακολούθησης του αιμοδυναμικού προφίλ του ασθενούς. Εάν αυτές δεν είναι διαθέσιμες, συνιστάται περιορισμένη χορήγηση υγρών με βάση το ιδανικό βάρος.
2.2.9	**Πρόληψη της υποθερμίας**<br>Η θερμοκρασία θα πρέπει να παρακολουθείται και η νορμοθερμία πρέπει να διατηρείται με ενεργητική θέρμανση (θερμά υγρά, θερμαινόμενη κουβέρτα).
2.2.10	**Προφύλαξη μετεγχειρητικής ναυτίας και εμέτου**<br>Χορηγήστε αντιεμετική θεραπεία σύμφωνα με την κλίμακα Apfel.
2.2.11	**Επισκληρίδιος αναλγησία**<br>Η θωρακική επισκληρίδιος αναλγησία πρέπει να χρησιμοποιείται σε ανοιχτή χειρουργική επέμβαση. Στη λαπαροσκοπική χειρουργική δεν συνιστάται συνήθως. Ασθενείς με αντένδειξη για επισκληρίδιο αναλγησία που έχουν κίνδυνο μετεγχειρητικής νεφρικής ανεπάρκειας ή έχουν διαταραχές πήξης θα μπορούσαν να ωφεληθούν από αμφοτερόπλευρο αποκλεισμό στο επίπεδο του Εγκάρσιου Κοιλιακού Μυός (TAP BLOCK - transabdominal plan blocks) ή άλλες εναλλακτικές λύσεις αντί της επισκληρίδιου αναλγησίας.
2.2.12	**Ελάχιστα επεμβατική χειρουργική**<br>Προτιμώνται οι ελάχιστα επεμβατικές προσπελάσεις και θα πρέπει να χρησιμοποιούνται όσο το δυνατόν περισσότερο.
2.2.13	**Αποφύγετε τα ενισχυτικά συρραπτικών και τις βιολογικές κόλλες**<br>Οι μέθοδοι ενίσχυσης της γραμμής συρραφής, όπως η χρήση ενισχυτικών συρραφής ή βιολογικών κόλλων, δεν μειώνουν τις διαφυγές σύμφωνα με δεδομένα.

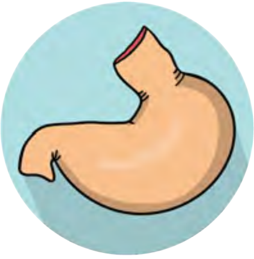

2.2.14	**Βαθμονόμηση της κάθετης (επιμήκους) (sleeve) γαστρεκτομής**<br>Η κάθετη (επιμήκης) (sleeve) γαστρεκτομή πρέπει να βαθμονομηθεί με οδηγούς (probes).
2.2.15	**Αποφύγετε τους ρινογαστρικούς σωλήνες**<br>Οι ρινογαστρικοί σωλήνες συνιστώνται μόνο διεγχειρητικά για να αδειάσει το στομάχι.
2.2.16	**Αποφύγετε τις παροχετεύσεις**
2.3	Άμεσα Μετεγχειρητικά<br><br>Αναισθησιολόγος, Νοσηλευτής
2.3.1	**Διατήρηση της νορμοθερμίας**<br>Η θερμοκρασία πρέπει να μετράται τακτικά και να διατηρείται η νορμοθερμία.
2.3.2	**Αναλγησία με περιορισμό των οπιοειδών**<br>Θα πρέπει να χρησιμοποιείται προληπτική πολυπαραγοντική αναλγησία. Περιορίστε τη χρήση οπιοειδών. Στοχεύστε σε βαθμολογία πόνου στην οπτική αναλογική κλίμακα (Visual Analog Scale – VAS) μικρότερη από 3.
2.3.3	**Πρώιμη σίτιση**<br>Έναρξη λήψης υγρών από το στόμα 6 ώρες μετά το χειρουργείο.
2.3.4	**Πρώιμη κινητοποίηση**<br>Η κινητοποίηση θα πρέπει να ξεκινά 3 ώρες μετά το χειρουργείο και θα πρέπει να ξεκινά με το κάθετο στο κρεβάτι. Η βάδιση θα πρέπει να ξεκινά 6 ώρες μετά την επέμβαση λαμβάνοντας υπόψιν πάντα τις ώρες του νυχτερινού ύπνου.
2.3.5	**Θρομβοεμβολική προφύλαξη**<br>Η ηπαρίνη χαμηλού μοριακού βάρους πρέπει να χορηγείται 12 ώρες μετά την επέμβαση.
2.3.6	**Προφύλαξη μετεγχειρητικής ναυτίας και εμέτου**<br>Χορηγήστε αντιεμετική θεραπεία σύμφωνα με την κλίμακα Apfel.
2.3.7	**Θεραπεία υπνικής άπνοιας**<br>Σε ασθενείς με υπνική άπνοια, επαναχρησιμοποιείστε τη Συνεχή Θετική Πίεση των Αεραγωγών (CPAP) το συντομότερο δυνατό.

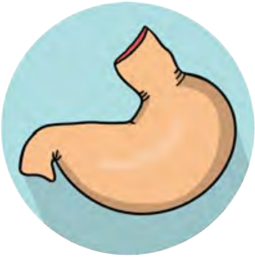

3	1η μετεγχειρητική ημέρα<br>(Θάλαμος Νοσηλείας)<br><br>Χειρουργός, Νοσηλευτής
3.1	**Πρώιμη σίτιση**<br>Μια υδρική υποθερμιδική δίαιτα πρέπει να ξεκινά ανάλογα με την ανοχή του ασθενούς.
3.2	**Πρώιμη κινητοποίηση**<br>Οι ασθενείς θα πρέπει να ενθαρρύνονται να περπατούν.
3.3	**Αναλγησία με περιορισμό των οπιοειδών**<br>Θα πρέπει να χρησιμοποιείται ενεργητική ή προληπτική πολυπαραγοντική αναλγησία. Περιορίστε τη χρήση οπιοειδών. Στοχεύστε σε βαθμολογία πόνου στην οπτική αναλογική κλίμακα (Visual Analog Scale – VAS) μικρότερη από 3.
3.4	**Σταματήστε την ενδοφλέβια χορήγηση υγρών**<br>Εάν οι ασθενείς ανέχονται επαρκώς τα υγρά από του στόματος, διακόψτε την ενδοφλέβια χορήγηση υγρών.
3.5	**Αφαιρέστε τον ουροκαθετήρα**<br>Εάν έχει τοποθετηθεί ουροκαθετήρας, εξετάστε το ενδεχόμενο αφαίρεσής του.
3.6	**Αφαίρεση παροχετεύσεων**<br>Εξετάστε το ενδεχόμενο αφαίρεσής των παροχετεύσεων, αν υπάρχουν.
3.7	**Θρομβοεμβολική προφύλαξη**
3.8	**Αναπνευστική φυσιοθεραπεία**
4	2η μετεγχειρητική ημέρα (και επόμενες μέρες)<br>(Θάλαμος Νοσηλείας)<br><br>Χειρουργός, Νοσηλευτής
4.1	**Πρώιμη σίτιση**<br>Χορηγείστε στους ασθενείς μια υδρική υποθερμιδική πλήρη δίαιτα ή υποθερμιδική υπερπρωτεϊνική πλήρη δίαιτα.
4.2	**Αφαίρεση παροχετεύσεων**

	Εξετάστε το ενδεχόμενο αφαίρεσής των παροχετεύσεων, αν υπάρχουν
4.3	**Αξιολογήστε τα κριτήρια εξιτηρίου**<br>Εξετάστε το ενδεχόμενο εξιτηρίου εάν δεν υπάρχουν χειρουργικές επιπλοκές που δεν μπορούν να αντιμετωπιστούν σε εξωτερικά ιατρεία, δεν υπάρχει πυρετός, δεν υπάρχει ταχυκαρδία ή ταχύπνοια, ο πόνος είναι ελεγχόμενος με από του στόματος αναλγησία, ο ασθενής είναι πλήρως κινητοποιημένος και μπορεί να ανεχθεί μια από του στόματος δίαιτα.
5	Εξιτήριο<br><br>Χειρουργός, Νοσηλευτής
5.1	**Διατροφή**<br>Μια υποθερμιδική δίαιτα με αλεσμένες τροφές ή πλήρης υποθερμιδική υπερπρωτεϊνική από του στόματος δίαιτα συνιστάται για τις πρώτες 1-2 εβδομάδες. Μετά από 2 εβδομάδες ημιστερεή δίαιτα. Η δίαιτα με στερεά πρέπει να ξεκινά 1-2 μήνες μετά την επέμβαση.
5.2	**Περιποίηση τραύματος**<br>Καθημερινή φροντίδα τραύματος και αφαίρεση ραμμάτων ή αγκτήρων δέρματος σύμφωνα με το πρωτόκολλο.
5.3	**Άσκηση**<br>Πρόγραμμα ασκήσεων που συνδυάζει αερόβια άσκηση και ενδυνάμωση. Έναρξη από 1 μήνα μετά την επέμβαση με μέτριες εντάσεις προχωρώντας σε υψηλότερες εντάσεις.
5.4	**Θρομβοπροφύλαξη**<br>Η θρομβοπροφύλαξη συνιστάται για τις πρώτες 3-4 εβδομάδες μετεγχειρητικά.
5.5	**Συνεχιζόμενη φροντίδα**<br>Τηλεφωνικός έλεγχος μετά το εξιτήριο. Συντονισμός υποστήριξης στο σπίτι με την πρωτοβάθμια περίθαλψη.

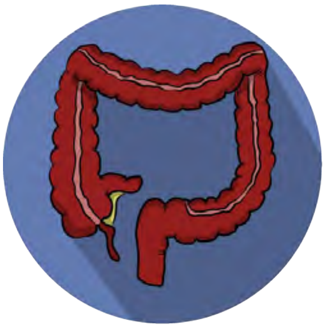

Πρωτόκολλο Eupemen
για Κολεκτομή

1	Πριν την εισαγωγή<br><br>Αναισθησιολόγος, Χειρουργός, Νοσηλευτής, Διαιτολόγος, Ειδικός Στομιών
1.1	**Προεγχειρητική συμβουλευτική**<br>Ο ασθενής θα πρέπει να είναι πλήρως ενημερωμένος για τη διαδικασία και την περιεγχειρητική πορεία τόσο προφορικά όσο και γραπτά. Θα πρέπει να λαμβάνεται υπογεγραμμένη ενημερωμένη συγκατάθεση.
1.2	**Ολοκληρωμένη ιατρική αξιολόγηση**<br>Αυτό θα πρέπει να περιλαμβάνει ιατρικό ιστορικό, φυσική εξέταση, ακτινογραφία θώρακα, εξετάσεις αίματος (έλεγχος πήξης, βιοχημικό προφίλ συμπεριλαμβανομένης της C-αντιδρώσας πρωτεΐνης και γενική αίματος) και ηλεκτροκαρδιογράφημα.
1.3	**Αξιολόγηση ευπάθειας**<br>Για ασθενείς ηλικίας άνω των 65 ετών θα πρέπει να γίνεται αξιολόγηση ευπάθειας
1.4	**Αξιολόγηση κατάταξης στην κλίμακα ASA (American Society of Anesthesiologists)**
1.5	**Κλιμάκα Apfel**<br>Ο κίνδυνος για μετεγχειρητική ναυτία και έμετο θα πρέπει να αξιολογείται με την κλίμακα Apfel.
1.6	**Ρύθμιση χρόνιων παθήσεων**<br>Όλες οι χρόνιες παθήσεις θα πρέπει να βελτιστοποιούνται πριν από την επέμβαση. Όλες οι περιπτώσεις πρόσφατης έναρξης ή ενεργού καρδιαγγειακού νοσήματος θα πρέπει να αξιολογούνται από καρδιολόγο.
1.7	**Αξιολόγηση Σακχαρώδη Διαβήτη**<br>Τα επίπεδα γλυκόζης αίματος και γλυκοζηλιωμένης αιμοσφαιρίνης (HbA1c) θα πρέπει να εκτιμηθούν. Όλες οι περιπτώσεις φτωχά ελεγχόμενου ή προηγουμένως αδιάγνωστου διαβήτη θα πρέπει να παραπέμπονται στην πρωτοβάθμια φρόντιδα ή σε ενδοκρινολογο / διαβητολόγο πριν από τη χειρουργική επέμβαση.
1.8	**Αξιολόγηση και διαχείριση της αναιμίας και της ανεπάρκειας σιδήρου**

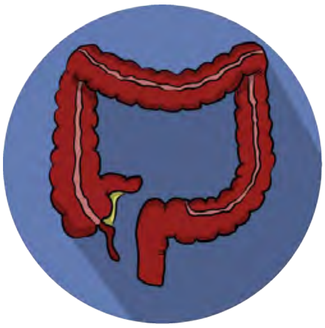

	Η σιδηροπενική αναιμία θα πρέπει να αντιμετωπίζεται ιδανικά με παρεντερική χορήγηση σιδήρου.
1.9	**Διατροφικός έλεγχος**<br>Ο διατροφικός έλεγχος θα πρέπει να γίνεται χρησιμοποιώντας το Malnutrition Universal Screening Tool (MUST). Οι ασθενείς που διατρέχουν κίνδυνο υποσιτισμού θα πρέπει να λαμβάνουν από του στόματος συμπληρώματα διατροφής, κατά προτίμηση ανοσοδιατροφή για μια περίοδο 7 ημερών πριν και 5 ημέρες μετά την επέμβαση.
1.10	**Διακόψτε το κάπνισμα και μειώστε την κατανάλωση αλκοόλ τουλάχιστον ένα μήνα πριν την επέμβαση**
1.11	**Πολυπαραγοντική προαποκατάσταση συμπεριλαμβανομένων αερόβιων ασκήσεων και ασκήσεων αντίστασης**
1.12	**Δίαιτα χαμηλού υπολειμμάτος τουλάχιστον 5 ημέρες πριν την επέμβαση**
1.13	**Χωρίς μηχανική προετοιμασία του εντέρου εκτός από την περιπτώση περιεγχειρητικής κολονοσκόπησης**
1.14	**Κλύσματα Καθαρισμού**<br>Δύο κλύσματα καθαρισμού το απόγευμα πριν την επέμβαση (επεμβάσεις αριστερού κόλου)
2	Περιεγχειρητικά
2.1	Άμεσα Προεγχειρητικά<br>(Προγραμματίστε την εισαγωγή την ίδια ημέρα της επέμβασης, εάν είναι δυνατόν)<br><br>Αναισθησιολόγος, Χειρουργός, Νοσηλευτής, Διαιτολόγος, Ειδικός Στομιών
2.1.1	**Προεγχειρητική υγιεινή**<br>Ο ασθενής λαμβάνει οδηγίες να κάνει πλήρες ντους ή μπάνιο το βράδυ ή το πρωί πριν από την επέμβαση.
2.1.2	**Κάλτσες συμπίεσης ή διαλείπουσα πνευματική συμπίεση.**

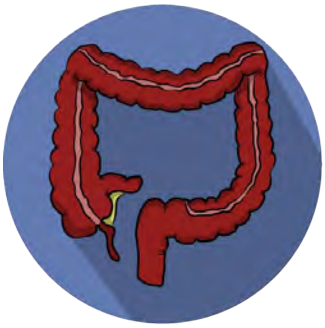

	Οι κάλτσες συμπίεσης ή η διαλείπουσα πνευματική συμπίεση πρέπει να τοποθετούνται από την εισαγωγή στο νοσοκομείο.
2.1.3	**Ηπαρίνη χαμηλού μοριακού βάρους**<br>Η ηπαρίνη χαμηλού μοριακού βάρους πρέπει να χορηγείται 2-12 ώρες πριν από την επέμβαση (ανάλογα με το εάν πρόκειται να γίνει νευραξονική αναισθησία ή όχι).
2.1.4	**Πόσιμο διάλυμα υδατανθράκων**<br>Ένα ρόφημα πλούσιο σε υδατάνθρακες (12,5% μαλτοδεξτρίνες) 800 ml πρέπει να χορηγείται το βράδυ πριν από την επέμβαση και 400 ml 2 ώρες πριν από την αναισθησία. Για διαβητικούς ασθενείς χορηγήστε το μαζί με αντιδιαβητική αγωγή.
2.1.5	**Προεγχειρητική νηστεία**<br>Νηστεία 6 ωρών για τα στερεά και 2 ωρών για τα διαυγή υγρά.
2.1.6	**Ξύρισμα με ηλεκτρικό ξυράφι**<br>Το σημείο όπου θα γίνει η τομή θα πρέπει να ξυριστεί με ηλεκτρική ξυριστική μηχανή, εάν είναι απαραίτητο.
2.1.7	**Σήμανση στομίας (αν αναμένεται)**
2.1.8	**Αντιβιοτική χημειοπροφύλαξη**<br>Προφυλακτική χορήγηση αντιβιοτικού 30-60 λεπτά πριν την τομή. Σε παρατεταμένες διαδικασίες επαναλάβετε τις δόσεις ανάλογα με τον χρόνο ημιζωής των φαρμάκων.
2.2	Διεγχειρητικά<br><br>Αναισθησιολόγος, Χειρουργός, Νοσηλευτής
2.2.1	**Λίστα ελέγχου χειρουργικής ασφάλειας του ΠΟΥ (Παγκόσμιου Οργανσιμού Υγείας)**<br>Η λίστα ελέγχου χειρουργικής ασφάλειας του ΠΟΥ θα πρέπει να συμπληρωθεί πριν γίνει η τομή.
2.2.2	**Συστηματική διεγχειρητική παρακολούθηση**<br>Οι ζωτικές λειτουργίες, το FiO2, το βάθος της αναισθησίας, ο νευρομυϊκός αποκλεισμός και η γλυκαιμία θα πρέπει να παρακολουθούνται κατά τη διάρκεια της διαδικασίας.

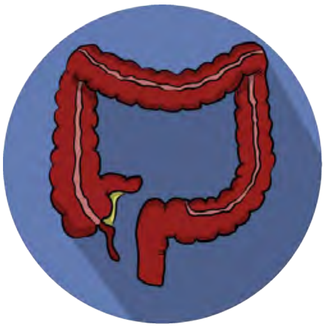

Πρωτόκολλο Eupemen
για Κολεκτομή

2.2.3	**Ελάχιστα επεμβατική χειρουργική**<br>Προτιμώνται οι ελάχιστα επεμβατικές προσεγγίσεις και θα πρέπει να χρησιμοποιούνται όσο το δυνατόν περισσότερο. Συνιστώνται επίπεδα ενδοκοιλιακής πίεσης 8-12 mmHg.
2.2.4	**Αποφύγετε τον συστηματικό καθετηριασμό της ουροδόχου κύστης**
2.2.5	**Επεμβατική παρακολούθηση**<br>Δεν απαιτείται συνήθως τοποθέτηση αρτηριακής γραμμής.
2.2.6	**Κεντρικός φλεβικός καθετήρας**<br>Ο κεντρικός φλεβικός καθετήρας δεν απαιτείται συνήθως.
2.2.7	**Εισαγωγή και διατήρηση της αναισθησίας**<br>Παράγοντες βραχείας δράσης θα πρέπει να χρησιμοποιούνται για την εισαγωγή και τη διατήρηση της αναισθησίας.
2.2.8	**Οξυγόνωση**<br>Οι ασθενείς θα πρέπει να λαμβάνουν οξυγόνο με FiO2 άνω του 50%.
2.2.9	**Χορήγηση Υγρών**<br>Η Αιμοδυναμική βελτιστοποίηση με στοχοκατευθυνόμενη χορήγηση υγρών με τη χρήση συσκευών παρακολούθησης του αιμοδυναμικού προφίλ του ασθενούς, συνιστάται σε ασθενείς υψηλού κινδύνου και σε ασθενείς που υποβάλλονται σε χειρουργική επέμβαση με μεγάλη απώλεια αίματος. Σε όλες τις άλλες περιπτώσεις, συνιστάται περιοριστική χορήγηση υγρών με βάση το ιδανικό βάρος με συνεχή χορήγηση ισορροπημένου κρυσταλλοειδούς διάλυματος (1-3 ml/kg/h για λαπαροσκόπηση, 3-5 ml/kg/h για λαπαροτομία). Η απώλεια αίματος θα πρέπει να αντισταθμίζεται με κολλοειδή 1:1.
2.2.10	**Αποφύγετε το ρινογαστρικό σωλήνα**<br>Οι ρινογαστρικοί σωλήνες δεν πρέπει να χρησιμοποιούνται τακτικά.
2.2.11	**Πρόληψη της υποθερμίας**<br>Η θερμοκρασία θα πρέπει να παρακολουθείται και η νορμοθερμία πρέπει να διατηρείται με ενεργητική θέρμανση (θερμά υγρά, θερμαινόμενη κουβέρτα).
2.2.12	**Προφύλαξη μετεγχειρητικής ναυτίας και εμέτου**<br>Χορηγήστε αντιεμετική θεραπεία σύμφωνα με την κλίμακα Apfel.

EUPEMEN
European Perioperative Medical Networking

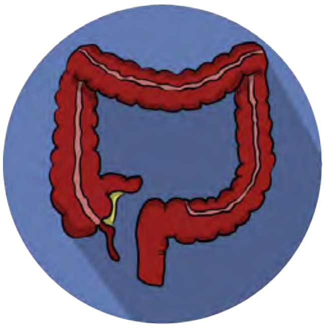

2.2.13	**Επισκληρίδιος αναλγησία**<br>Η θωρακική επισκληρίδιος αναλγησία πρέπει να χρησιμοποιείται σε ανοιχτή χειρουργική επέμβαση. Στη λαπαροσκοπική χειρουργική δεν συνιστάται συνήθως. Ασθενείς με αντένδειξη για επισκληρίδιο αναλγησία που έχουν κίνδυνο μετεγχειρητικής νεφρικής ανεπάρκειας ή έχουν διαταραχές πήξης θα μπορούσαν να ωφεληθούν από αμφοτερόπλευρο αποκλεισμό στο επίπεδο του Εγκάρσιου Κοιλιακού Μυός (TAP BLOCK - transabdominal plan blocks), διήθηση των σημείων εισόδου των λαπαροσκοπικών τροκάρ με τοπικό αναισθητικό ή άλλες εναλλακτικές λύσεις αντί της επισκληρίδιου αναλγησίας.
2.2.14	**Ενδοφλέβια επικουρικά αναλγητικά φάρμακα**<br>Συνιστώμενα επικουρικά αναλγητικά είναι τα μη στεροειδή αντιφλεγμονώδη φάρμακα, η λιδοκαΐνη, η κεταμίνη, το θειικό μαγνήσιο και η δεξμεντετομιδίνη.
2.2.15	**Περιεγχειρητικός γλυκαιμικός έλεγχος**<br>Για τους διαβητικούς ασθενείς χρησιμοποιήστε το τοπικό νοσοκομειακό πρωτόκολλο για διαβητικούς που υποβάλλονται σε χειρουργική επέμβαση. Σε ασθενείς που κινδυνεύουν να αναπτύξουν αντίσταση στην ινσουλίνη, αποφύγετε επίπεδα γλυκόζης στο αίμα υψηλότερα από 180 mg/dL.
2.2.16	**Απολύμανση δέρματος**<br>Το δέρμα πρέπει να απολυμαίνεται από το κεντρο προς την περιφερεια με αλκοολικό διάλυμα χλωρεξιδίνης 2%.
2.2.17	**Αποφύγετε τις παροχετεύσεις**<br>Οι κοιλιακές παροχετεύσεις πρέπει να αποφεύγονται όσο το δυνατόν περισσότερο.
2.3	Άμεσα Μετεγχειρητικά<br>(Μονάδα Αναζωογόνησης / Μονάδα Ενδιάμεσης Φροντίδας)<br><br>Αναισθησιολόγος, Νοσηλευτής
2.3.1	**Διατήρηση της νορμοθερμίας**<br>Η θερμοκρασία πρέπει να μετράται τακτικά και να διατηρείται η

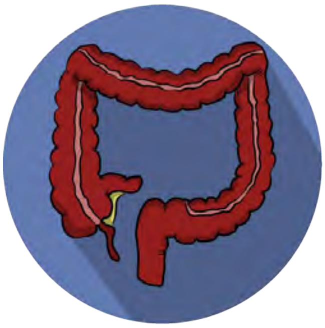

	νορμοθερμία.
2.3.2	**Αναλγησία με περιορισμό των οπιοειδών**<br>Θα πρέπει να χρησιμοποιείται ενεργητική ή προληπτική πολυπαργοντική αναλγησία. Περιορίστε τη χρήση οπιοειδών. Στοχεύστε σε βαθμολογία πόνου στην οπτική αναλογική κλίμακα (Visual Analog Scale – VAS) μικρότερη από 3.
2.3.3	**Περιοριστική χορήγηση υγρών.**
2.3.4	**Πρώιμη σίτιση**<br>Έναρξη λήψης υγρών από το στόμα 6 ώρες μετά το χειρουργείο.
2.3.5	**Αναπνευστική φυσιοθεραπεία**
2.3.6	**Πρώιμη κινητοποίηση**<br>Οι ασθενείς θα πρέπει να κάθονται όρθιοι 3 ώρες μετά την επέμβαση και θα πρέπει να ξεκινούν βάδιση 8 ώρες μετά την επέμβαση λαμβάνοντας υπόψιν πάντα τις ώρες του νυχτερινού ύπνου.
2.3.7	**Θρομβοεμβολική προφύλαξη**<br>Η ηπαρίνη χαμηλού μοριακού βάρους πρέπει να χορηγείται 12 ώρες μετά την επέμβαση.
2.3.8	**Προφύλαξη μετεγχειρητικής ναυτίας και εμέτου**<br>Χορηγήστε αντιεμετική θεραπεία σύμφωνα με την κλίμακα Apfel.
2.3.9	**Διατήρηση FiO2 (κλάσμα εισπνεόμενου οξυγόνου) 0,5% για 2 ώρες μετά την επέμβαση**
2.3.10	**Περιεγχειρητικός γλυκαιμικός έλεγχος**<br>Για τους διαβητικούς ασθενείς χρησιμοποιήστε το τοπικό νοσοκομειακό πρωτόκολλο για διαβητικούς ασθενείς που υποβάλλονται σε χειρουργική επέμβαση. Σε ασθενείς που κινδυνεύουν να αναπτύξουν αντίσταση στην ινσουλίνη, αποφύγετε επίπεδα γλυκόζης στο αίμα υψηλότερα από 180 mg/dL.
3	1η μετεγχειρητική ημέρα<br>(Θάλαμος Νοσηλείας)

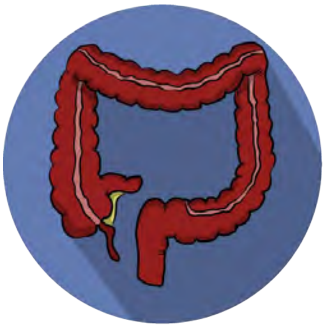

Πρωτόκολλο Eupemen
για Κολεκτομή

	Χειρουργός, Νοσηλευτής, Ειδικός Στομιών
3.1	**Πρώιμη σίτιση**<br>Μια υγρή ή ημιστερεή δίαιτα πρέπει να ξεκινά ανάλογα με την ανοχή του ασθενούς.
3.2	**Αποφύγετε την ενδοφλέβια χορήγηση υγρών**<br>Εάν οι ασθενείς ανέχονται τα υγρά από του στόματος, διακόψτε την ενδοφλέβια χορήγηση υγρών.
3.3	**Πρώιμη κινητοποίηση**<br>Οι ασθενείς θα πρέπει να ενθαρρύνονται να μετακινούνται από το κρεβάτι στην καρέκλα δίπλα στο κρεβάτι.
3.4	**Αναλγησία με περιορισμό των οπιοειδών**<br>Εξασφαλίστε καλό έλεγχο του πόνου. Στοχεύστε σε βαθμολογία πόνου στην οπτική αναλογική κλίμακα (Visual Analog Scale – VAS) μικρότερη από 3.
3.5	**Αφαιρέστε τον ουροκαθετήρα**<br>Εάν έχει τοποθετηθεί ουροκαθετήρας, εξετάστε το ενδεχόμενο αφαίρεσής του
3.6	**Αφαίρεση παροχετεύσεων**<br>Εξετάστε το ενδεχόμενο αφαίρεσής των παροχετεύσεων, αν υπάρχουν
3.7	**Αναπνευστική φυσιοθεραπεία**
3.8	**Θρομβοεμβολική προφύλαξη**<br>Η θρομβοεμβολική προφύλαξη που αποτελείται από κάλτσες συμπίεσης ή διαλείπουσα συμπίεση και ηπαρίνη χαμηλού μοριακού βάρους θα πρέπει να χορηγείται σύμφωνα με το τοπικό νοσοκομειακό πρωτόκολλο
3.9	**Προφύλαξη μετεγχειρητικής ναυτίας και εμέτου**<br>Χορηγήστε αντιεμετική θεραπεία σύμφωνα με την κλίμακα Apfel.
3.10	**Προφύλαξη κατά του πεπτικού έλκους**
3.11	**Περιεγχειρητικός γλυκαιμικός έλεγχος**

EUPEMEN
European Perioperative Medical Networking

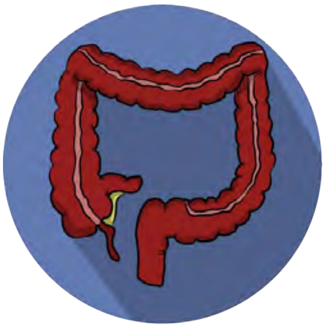

	Για τους διαβητικούς ασθενείς χρησιμοποιήστε το τοπικό νοσοκομειακό πρωτόκολλο για διαβητικούς ασθενείς που υποβάλλονται σε χειρουργική επέμβαση. Σε ασθενείς που κινδυνεύουν να αναπτύξουν αντίσταση στην ινσουλίνη, αποφύγετε επίπεδα γλυκόζης στο αίμα υψηλότερα από 180 mg/dL.
3.12	**Εκπαίδευση φροντίδας στομίας (εάν υπάρχει)**
3.13	**Εργαστηριακές εξετάσεις**<br>Θα πρέπει να γίνονται εξετάσεις αίματος συμπεριλαμβανομένης της C-αντιδρώσας πρωτεΐνης.
4	2η μετεγχειρητική ημέρα<br><br>Χειρουργός, Νοσηλευτής, Ειδικός Στομιών
4.1	**Πρώιμη σίτιση**<br>Πρέπει να δίνεται ημιστερεή ή στερεή δίαιτα.
4.2	**Αποφύγετε την ενδοφλέβια χορήγηση υγρών**<br>Διακόψτε την ενδοφλέβια χορήγηση υγρών εάν δεν έχει γίνει προηγουμένως.
4.3	**Πρώιμη κινητοποίηση**<br>Οι ασθενείς θα πρέπει να μπορούν να περπατούν μικρές αποστάσεις.
4.4	**Αναλγησία με περιορισμό των οπιοειδών**<br>Εξασφαλίστε καλό έλεγχο του πόνου. Στοχεύστε σε βαθμολογία πόνου στην οπτική αναλογική κλίμακα (Visual Analog Scale – VAS) μικρότερη από 3.<br>Εξετάστε το ενδεχόμενο για από του στόματος αναλγησία
4.5	**Αφαιρέστε τον καθετήρα ούρων (αν δεν έχει γίνει προηγουμένως)**
4.6	**Αναπνευστική φυσιοθεραπεία**
4.7	**Θρομβοεμβολική προφύλαξη**
4.8	**Προφύλαξη μετεγχειρητικής ναυτίας και εμέτου**

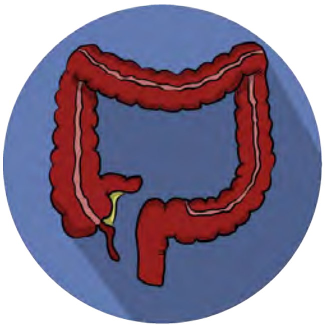

4.9	**Προφύλαξη κατά του πεπτικού έλκους**
4.10	**Περιεγχειρητικός γλυκαιμικός έλεγχος**
4.11	**Συνέχιση με την προηγούμενη εκπαίδευση φροντίδας στομίας (εάν υπάρχει)**
4.12	**Εργαστηριακές εξετάσεις**<br>Θα πρέπει να γίνονται εξετάσεις αίματος συμπεριλαμβανομένης της C-αντιδρώσας πρωτεΐνης.
5	3^{η} Μετεγχειρητική ημέρα<br><br>Χειρουργός, Νοσηλεύτρια
5.1	**Πρώιμη σίτιση**<br>Πρέπει να δίνεται στερεή δίαιτα.
5.2	**Πρώιμη κινητοποίηση**<br>Ο ασθενής πρέπει να είναι πλήρως κινητοποιημένος
5.3	**Από του στόματος αναλγησία**
5.4	**Αφαίρεση της φλεβικής γραμμής**
5.5	**Αναπνευστική φυσιοθεραπεία**
5.6	**Θρομβοεμβολική προφύλαξη**
5.7	**Περιεγχειρητικός γλυκαιμικός έλεγχος**
5.8	**Εργαστηριακές εξετάσεις**<br>Θα πρέπει να γίνονται εξετάσεις αίματος συμπεριλαμβανομένης της C-αντιδρώσας πρωτεΐνης.
5.9	**Αξιολογήστε τα κριτήρια εξιτηρίου**<br>Εξετάστε το ενδεχόμενο εξιτηρίου εάν δεν υπάρχουν χειρουργικές επιπλοκές που δεν μπορούν να αντιμετωπιστούν σε εξωτερικά ιατρεία, δεν υπάρχει πυρετός, πόνος ελεγχόμενος με από του

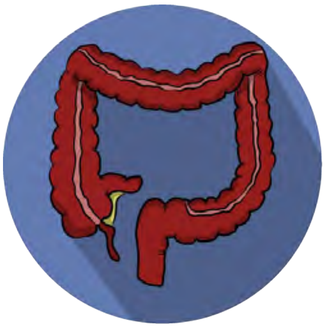

	στόματος αναλγησία, πλήρης κινητοποίηση, ανοχή στην από του στόματος λήψη τροφής και αποδοχή από τον ασθενή.
6	Εξιτήριο<br><br>Χειρουργός, Νοσηλευτής, Πρωτοβάθμια Περίθαλψη
6.1	**Έγγραφα κατά το εξιτήριο**<br>Κατά το εξιτήριο στους ασθενείς θα πρέπει να παρέχονται εξατομικευμένες, κατανοητές και πλήρεις πληροφορίες σχετικά με τη νοσηλεία τους στο νοσοκομείο και συστάσεις για την φροντίδα στο σπίτι.
6.2	**Θρομβοεμβολική προφύλαξη**<br>Η θρομβοεμβολική προφύλαξη θα πρέπει να συνεχιστεί μέχρι τις 28 ημέρες μετά την επέμβαση.
6.3	**Παρακολούθηση**<br>Οι ασθενείς θα πρέπει να παρακολουθούνται την πρώτη εβδομάδα μετά το εξιτήριο σε περιβάλλον εξωτερικών ασθενών ή μέσω τηλεφώνου. Θα πρέπει να προγραμματιστούν περαιτέρω επισκέψεις ελέγχου στους 1, 3 και 6 μήνες μετά το εξιτήριο. Θα πρέπει να οργανωθεί επίσκεψη στον ιατρό πρωτοβάθμιας περίθαλψης και, εάν χρειάζεται, να συντονιστεί η κατ' οίκον υποστήριξη.

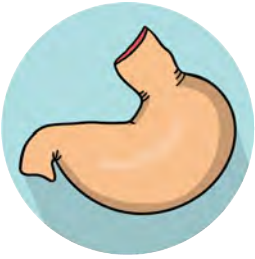

Πρωτόκολλο Eupemen
για Εντερική Απόφραξη

1	Προεγχειρητικά<br><br>Αναισθησιολόγος, Χειρουργός
1.1	**Συστηματική προεγχειρητική αξιολόγηση**<br>Φυσική εξέταση, Ακτινογραφία κοιλίας abdominal X-ray και πλήρης εργαστηριακός έλεγχος συμπεριλαμβανομένης της C-αντιδρώσας πρωτεΐνης
1.2	**Κλινικά συστήματα βαθμολόγησης**<br>Για τους ηλικιωμένους ασθενείς, θα πρέπει να χρησιμοποιούνται βαθμολογίες για τον υπολογισμό της ευπάθειας (frailty), όπως ο τροποποιημένος δείκτης ευπάθειας (modified frailty index) και το VIG Express. Τα κριτήρια του Beers θα πρέπει να αξιολογηθούν για την πρόληψη του παραληρήματος σε ενήλικες άνω των 65 ετών.
1.3	**Νορμοθερμία**<br>Εξασφαλίστε προεγχειρητική νορμοθερμία σε ευπαθείς ασθενείς χρησιμοποιώντας θερμαινόμενες κουβέρτες.
1.4	**Αποφύγετε την τοποθέτηση καθετήρα ούρων**<br>Χρησιμοποιήστε μόνο εάν είναι απαραίτητο.
1.5	**Περιεγχειρητικός έλεγχος γλυκόζης αίματος**<br>Για τους διαβητικούς ασθενείς χρησιμοποιήστε το τοπικό νοσοκομειακό πρωτόκολλο για διαβητικούς που υποβάλλονται σε χειρουργική επέμβαση. Σε ασθενείς που κινδυνεύουν να αναπτύξουν αντίσταση στην ινσουλίνη (παχύσαρκοι και ηλικιωμένοι ασθενείς) και σε χειρουργικές επεμβάσεις που διαρκούν περισσότερο από 1 ώρα, αποφύγετε επίπεδα γλυκόζης στο αίμα υψηλότερα από 180 mg/dL.
1.6	**Αντιβιοτική χημειοπροφύλαξη**<br>Σε όλες τις περιπτώσεις θα πρέπει να χορηγείται αντιβιοτική προφύλαξη και ο τύπος των αντιβιοτικών θα πρέπει να επιλέγεται σύμφωνα με το τοπικό νοσοκομειακό πρωτόκολλο.
1.7	**Ρινογαστρικός σωλήνας**<br>Συνιστάται η τοποθέτηση ρινογαστρικού σωλήνα.
1.8	**Δέσμες μέτρων περιεγχειρητικής φροντίδας**<br>Συνιστώνται δέσμες μέτρων περιεγχειρητικής φροντίδας για την πρόληψη λοιμώξεων του χειρουργικού πεδίου.

EUPEMEN
European Perioperative Medical Networking

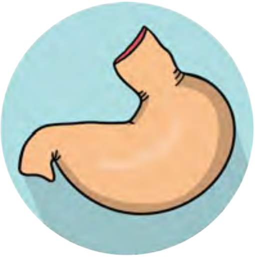

1.9	**Ενημερωμένη συγκατάθεση**<br>Ο ασθενής θα πρέπει να είναι πλήρως ενημερωμένος για την προγραμματισμένη διαδικασία και τις πιθανές επιπλοκές της. Οι ασθενείς που μπορούν θα πρέπει να δίνουν ενυπόγραφη ενημερωμένη συγκατάθεση.
2	Περιεγχειρητικά
2.1	Διεγχειρητικά<br><br>Αναισθησιολόγος, Χειρουργός, Νοσηλευτής
2.1.1	**Λίστα ελέγχου χειρουργικής ασφάλειας του Παγκόσμιου Οργανισμού Υγείας (WHO Surgical Safety Checklist)**
2.1.2	**Συστηματική διεγχειρητική παρακολούθηση**
2.1.3	**Χειρουργική προσπέλαση**<br>Οι ελάχιστα επεμβατικές προσεγγίσεις θα πρέπει να χρησιμοποιούνται μόνο σε εξαιρετικά επιλεγμένες περιπτώσεις σύμφωνα με την εμπειρία του χειρουργού. Στις περισσότερες περιπτώσεις θα πρέπει να προτιμάται η ανοιχτή χειρουργική επέμβαση.
2.1.4	**Ταχεία εισαγωγή στην αναισθησία**<br>Ταχεία εισαγωγή στην αναισθησία θα πρέπει να χρησιμοποιείται για τη μείωση της εισρόφησης του γαστρικού περιεχομένου.
2.1.5	**Περιεγχειρητική οξυγόνωση**<br>Θα πρέπει να χρησιμοποιείται ένα κλάσμα εισπνεόμενου οξυγόνου(FiO2) μεταξύ 0,6 και 0,8.
2.1.6	**Χορήγηση υγρών:**<br>Θα πρέπει να χρησιμοποιείται στοχο-κατευθυνόμενη (goal directed) χορήγηση υγρών με χρήση μη επεμβατικών συστημάτων αιμοδυναμικής παρακολούθησης. Εάν δεν υπάρχουν τέτοια συστήματα, θα πρέπει να χορηγούνται συνεχώς ισορροπημένα διαλύματα ανάλογα με τη χειρουργική προσέγγιση: 3-5 ml/kg/h για λαπαροσκόπηση και 5-7 ml/kg/h για λαπαροτομία.
2.1.7	**Αποφύγετε την τοποθέτηση καθετήρα ούρων**<br>Χρησιμοποιήστε μόνο εάν είναι απαραίτητο.

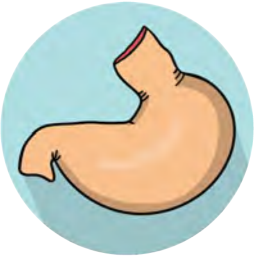

2.1.8	**Διατηρήστε τη νορμοθερμία**<br>Χρησιμοποιήστε θερμαινόμενες κουβέρτες και θερμά υγρά.
2.1.9	**Περιεγχειρητικός έλεγχος γλυκόζης αίματος**<br>Για τους διαβητικούς ασθενείς χρησιμοποιήστε το τοπικό νοσοκομειακό πρωτόκολλο για διαβητικούς που υποβάλλονται σε χειρουργική επέμβαση. Σε ασθενείς που κινδυνεύουν να αναπτύξουν αντίσταση στην ινσουλίνη (παχύσαρκοι και ηλικιωμένοι ασθενείς) και σε χειρουργικές επεμβάσεις που διαρκούν περισσότερο από 1 ώρα, αποφύγετε επίπεδα γλυκόζης στο αίμα υψηλότερα από 180 mg/dL.
2.1.10	**Επισκληρίδιος αναλγησία**<br>Η επισκληρίδιος αναλγησία πρέπει να χρησιμοποιείται σε ανοιχτή χειρουργική επέμβαση.
2.1.11	**Προφύλαξη μετεγχειρητικής ναυτίας και εμέτου**<br>Χορηγήστε αντιεμετική θεραπεία σύμφωνα με την κλίμακα Apfel.
2.1.12	**Αποφύγετε την τοποθέτηση κοιλιακών παροχετεύσεων**
2.1.13	**Θρομβοεμβολική προφύλαξη**<br>Η θρομβοεμβολική προφύλαξη που αποτελείται από κάλτσες συμπίεσης ή διαλείπουσα συμπίεση και ηπαρίνη χαμηλού μοριακού βάρους θα πρέπει να χορηγείται σύμφωνα με το τοπικό νοσοκομειακό πρωτόκολλο.
2.1.14	**Δέσμες μέτρων περιεγχειρητικής φροντίδας**<br>Συνιστώνται δέσμες μέτρων περιεγχειρητικής φροντίδας για την πρόληψη λοιμώξεων του χειρουργικού πεδίου.
2.2	Άμεσα μετεγχειρητικά<br><br>Αναισθησιολόγος, Χειρουργός, Νοσηλευτής
2.2.1	**Ενεργητική διατήρηση θερμοκρασίας.**<br>Η θερμοκρασία του σώματος πρέπει να μετράται τακτικά με στόχο την πρόληψη της υποθερμίας.
2.2.2	**Οξυγονοθεραπεία**<br>Ο κορεσμός οξυγόνου πρέπει να μετράται τακτικά για να αποφευχθεί ο υποκορεσμός.
2.2.3	**Πολυπαραγοντική αναλγησία με περιορισμό των οπιοειδών**

2.2.4	**Περιοριστική χορήγηση υγρών.**
2.2.5	**Περιεγχειρητικός έλεγχος γλυκόζης αίματος**<br>Για τους διαβητικούς ασθενείς χρησιμοποιήστε το τοπικό νοσοκομειακό πρωτόκολλο για διαβητικούς που υποβάλλονται σε χειρουργική επέμβαση. Σε ασθενείς που κινδυνεύουν να αναπτύξουν αντίσταση στην ινσουλίνη (παχύσαρκοι και ηλικιωμένοι ασθενείς) και σε χειρουργικές επεμβάσεις που διαρκούν περισσότερο από 1 ώρα, αποφύγετε επίπεδα γλυκόζης στο αίμα υψηλότερα από 180 mg/dL.
2.2.6	**Πρώιμη κινητοποίηση**<br>Οι ασθενείς θα πρέπει να κάθονται 2 ώρες μετά την επέμβαση και θα πρέπει να ξεκινούν βάδιση 8 ώρες μετά την επέμβαση λαμβάνοντας υπόψιν πάντα τις ώρες του νυχτερινού ύπνου.
2.2.7	**Ουδέν από το στόμα και ρινογαστρικός σωλήνας**<br>Εξετάστε το ενδεχόμενο αφαίρεσης 12 ώρες μετά την επέμβαση.
2.2.8	**Αφαίρεση καθετήρα ούρων**<br>Εάν έχει χρησιμοποιηθεί ουροκαθετήρας, Εξετάστε το ενδεχόμενο αφαίρεσης 12 ώρες μετά την επέμβαση.
2.2.9	**Θρομβοεμβολική προφύλαξη**<br>Η θρομβοεμβολική προφύλαξη που αποτελείται από κάλτσες συμπίεσης ή διαλείπουσα συμπίεση και ηπαρίνη χαμηλού μοριακού βάρους θα πρέπει να χορηγείται σύμφωνα με το τοπικό νοσοκομειακό πρωτόκολλο.
3	1η Μετεγχειρητική μέρα<br>(θάλαμος νοσηλείας)<br><br>Χειρουργός, Νοσηλευτής
3.1	**Περιεγχειρητικός έλεγχος γλυκόζης αίματος**<br>Για τους διαβητικούς ασθενείς χρησιμοποιήστε το τοπικό νοσοκομειακό πρωτόκολλο για διαβητικούς που υποβάλλονται σε χειρουργική επέμβαση. Σε ασθενείς που κινδυνεύουν να αναπτύξουν αντίσταση στην ινσουλίνη (παχύσαρκοι και ηλικιωμένοι ασθενείς) και σε χειρουργικές επεμβάσεις που διαρκούν περισσότερο από 1 ώρα, αποφύγετε επίπεδα γλυκόζης στο αίμα υψηλότερα από 180 mg/dL.
3.2	**Πρώιμη κινητοποίηση**<br>Ο ασθενής πρέπει να είναι πλήρως κινητοποιημένος

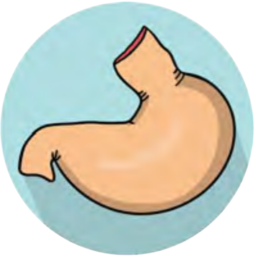

3.3	**Αναπνευστική φυσιοθεραπεία**
3.4	**Αντιβιοτική θεραπεία**<br>Η αντιβιοτική θεραπεία θα πρέπει να χορηγείται εάν πρόκειται για περιπτώσεις βακτηριακής αλλοθέσης ή λοίμωξης της περιτοναϊκής κοιλότητας. Τα αντιβιοτικά ευρέος φάσματος πρέπει να είναι σύμφωνα με το τοπικό νοσοκομειακό πρωτόκολλο.
3.5	**Πολυπαραγοντική αναλγησία με περιορισμό των οπιοειδών**
3.6	**Αφαίρεση ρινογαστρικού σωλήνα**<br>Εξετάστε το ενδεχόμενο αφαίρεσης του ρινογαστρικού σωλήνα.<br>Εάν αφαιρεθεί ο ρινογαστρικός σωλήνας, εξετάστε το ενδεχόμενο να ξεκινήσετε μια υγρή ή ημιστερεή δίαιτα.
3.7	**Αφαίρεση καθετήρα ούρων**<br>Εξετάστε το ενδεχόμενο αφαίρεσης του καθετήρα ούρων.
3.8	**Αφαίρεση επισκληρίδιου καθετήρα**<br>**Ε**ξετάστε το ενδεχόμενο αφαίρεσης του επισκληρίδιου καθετήρα.
3.9	**Θρομβοεμβολική προφύλαξη**<br>Η θρομβοεμβολική προφύλαξη που αποτελείται από κάλτσες συμπίεσης ή διαλείπουσα συμπίεση και ηπαρίνη χαμηλού μοριακού βάρους θα πρέπει να χορηγείται σύμφωνα με το τοπικό νοσοκομειακό πρωτόκολλο.
4	2η Μετεγχειρητική μέρα<br><br>Χειρουργός, Νοσηλευτής
4.1	**Περιεγχειρητικός έλεγχος γλυκόζης αίματος**<br>Για τους διαβητικούς ασθενείς χρησιμοποιήστε το τοπικό νοσοκομειακό πρωτόκολλο για διαβητικούς που υποβάλλονται σε χειρουργική επέμβαση. Σε ασθενείς που κινδυνεύουν να αναπτύξουν αντίσταση στην ινσουλίνη (παχύσαρκοι και ηλικιωμένοι ασθενείς) και σε χειρουργικές επεμβάσεις που διαρκούν περισσότερο από 1 ώρα, αποφύγετε επίπεδα γλυκόζης στο αίμα υψηλότερα από 180 mg/dL.
4.2	**Πρώιμη κινητοποίηση**<br>Ο ασθενής πρέπει να είναι πλήρως κινητοποιημένος

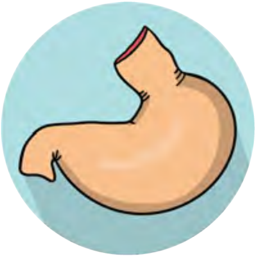

4.3	**Αναπνευστική φυσιοθεραπεία**
4.4	**Από του στόματος αναλγησία**<br>Θα πρέπει να χορηγείται από του στόματος αναλγησία με αποφυγή των οπιοειδών
4.5	**Αφαίρεση ρινογαστρικού σωλήνα**<br>Εξετάστε το ενδεχόμενο αφαίρεσης του ρινογαστρικού σωλήνα.<br>Εάν αφαιρεθεί ο ρινογαστρικός σωλήνας, εξετάστε το ενδεχόμενο να ξεκινήσετε μια υγρή ή ημιστερεή δίαιτα.
4.6	**Θρομβοεμβολική προφύλαξη**<br>Η θρομβοεμβολική προφύλαξη που αποτελείται από κάλτσες συμπίεσης ή διαλείπουσα συμπίεση και ηπαρίνη χαμηλού μοριακού βάρους θα πρέπει να χορηγείται σύμφωνα με το τοπικό νοσοκομειακό πρωτόκολλο.
4.7	**Πρώιμο εξιτήριο από το νοσοκομείο**<br>Αξιολογήστε τα κριτήρια εξιτήριου για περιπτώσεις χωρίς εντερεκτομή.
5	3η Μετεγχειρητική μέρα<br><br>Χειρουργός, Νοσηλευτής
5.1	**Πρώιμη σίτιση**
5.2	**Πρώιμη κινητοποίηση**
5.3	**Αναπνευστική φυσιοθεραπεία.**
5.4	**Θρομβοεμβολική προφύλαξη**
5.5	**Αξιολογήστε τα κριτήρια εξιτήριου**
6	Εξιτήριο<br><br>Χειρουργός, Νοσηλευτής Πρωτοβάθμια περίθαλψη
6.1	**Θρομβοεμβολική προφύλαξη**

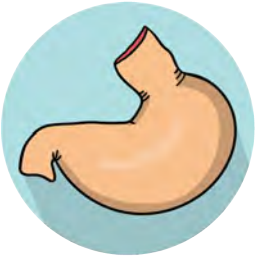

	Συνεχιζόμενη εξατομικευμένη θρομβοπροφύλαξη ανάλογα με τους κινδύνους.
6.2	**Αντιβιοτική θεραπεία**<br>Εξετάστε το ενδεχόμενο να συνεχίσετε τη θεραπεία με αντιβιοτικά μετά το εξιτήριο
6.3	**Εργαστηριακές εξετάσεις αίματος**<br>Εργαστηριακές εξέτασεις αίματος με τουλάχιστον 50% μείωση της C-αντιδρώσας πρωτεΐνης πριν από το εξιτήριο.
6.4	**Παρακολούθηση**<br>Παρακολούθηση μετά το εξιτήριο σε 24 ώρες σε εξωτερικό ιατρείο ή μέσω τηλεφώνου. Προγραμματίστε τους ασθενείς για έλεγχο σύμφωνα με το τοπικό νοσοκομειακό πρωτόκολλο. Συντονίστε την υποστήριξη στο σπίτι με την πρωτοβάθμια φροντίδα, εάν χρειάζεται.
6.5	**Κριτήρια Εξιτηρίου**<br>Γενικά Κριτήρια Εξιτηρίου: χωρίς επιπλοκές που δεν μπορούν να αντιμετωπιστούν σε εξωτερικό ιατρείο, επιστροφή της φυσιολογικής λειτουργείας του εντέρου (τακτικές κενώσεις), χωρίς πυρετό, πόνος ελεγχόμενος με από του στόματος αναλγησία, αποδοχή από τον ασθενή.

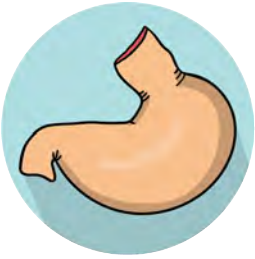

Πρωτόκολλο Eupemen
για Οξεία Σκωληκοειδίτιδα

1	Προεγχειρητικά<br><br>Αναισθησιολόγος, Χειρουργός
1.1	**Συστηματική προεγχειρητική αξιολόγηση**<br>Φυσική εξέταση, υπερηχογράφημα κοιλίας και πλήρης εργαστηριακός έλεγχος συμπεριλαμβανομένης της C-αντιδρώσας πρωτεΐνης.
1.2	**Κλινικά συστήματα βαθμολόγησης**<br>Οι υπολογιζόμενες βαθμολογίες θα πρέπει να περιλαμβάνουν το Appendicitis Inflammatory Response (AIR) Score (βαθμολογία της φλεγμονώδους απάντησης της σκωληκοειδίτιδας) και το adult appendicitis score (AAS) (βαθμολογία σκωληκοειδίτιδας ενηλίκων). Για τους ηλικιωμένους ασθενείς, θα πρέπει να χρησιμοποιούνται βαθμολογίες για τον υπολογισμό της ευπάθειας (frailty), όπως ο τροποποιημένος δείκτης ευπάθειας (modified frailty index) και το VIG Express. Τα κριτήρια του Beers θα πρέπει να αξιολογηθούν για την πρόληψη του παραληρήματος σε ενήλικες άνω των 65 ετών.
1.3	**Νορμοθερμία**<br>Εξασφαλίστε προεγχειρητική νορμοθερμία σε ευπαθείς ασθενείς χρησιμοποιώντας θερμαινόμενες κουβέρτες.
1.4	**Αποφύγετε την τοποθέτηση καθετήρα ούρων**<br>Χρησιμοποιήστε μόνο εάν είναι απαραίτητο.
1.5	**Περιεγχειρητικός έλεγχος γλυκόζης αίματος**<br>Για τους διαβητικούς ασθενείς χρησιμοποιήστε το τοπικό νοσοκομειακό πρωτόκολλο για διαβητικούς που υποβάλλονται σε χειρουργική επέμβαση. Σε ασθενείς που κινδυνεύουν να αναπτύξουν αντίσταση στην ινσουλίνη (παχύσαρκοι και ηλικιωμένοι ασθενείς) και σε χειρουργικές επεμβάσεις που διαρκούν περισσότερο από 1 ώρα, αποφύγετε επίπεδα γλυκόζης στο αίμα υψηλότερα από 180 mg/dL.
1.6	**Αντιβιοτική χημειοπροφύλαξη**<br>Σε όλες τις περιπτώσεις θα πρέπει να χορηγείται αντιβιοτική προφύλαξη και ο τύπος των αντιβιοτικών θα πρέπει να επιλέγεται σύμφωνα με το τοπικό νοσοκομειακό πρωτόκολλο.
1.7	**Δέσμες μέτρων περιεγχειρητικής φροντίδας**

EUPEMEN
European Perioperative Medical Networking

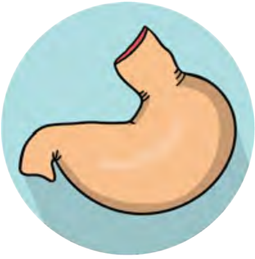

	Συνιστώνται δέσμες μέτρων περιεγχειρητικής φροντίδας για την πρόληψη λοιμώξεων του χειρουργικού πεδίου.
1.8	**Ενημερωμένη συγκατάθεση**<br>Ο ασθενής θα πρέπει να είναι πλήρως ενημερωμένος για την προγραμματισμένη διαδικασία και τις πιθανές επιπλοκές της. Οι ασθενείς που μπορούν θα πρέπει να δίνουν ενυπόγραφη ενημερωμένη συγκατάθεση.
2	Περιεγχειρητικά
2.1	Διεγχειρητικά<br>Αναισθησιολόγος, Χειρουργός, Νοσηλευτής
2.1.1	**Λίστα ελέγχου χειρουργικής ασφάλειας του Παγκόσμιου Οργανισμού Υγείας (WHO Surgical Safety Checklist)**
2.1.2	**Συστηματική διεγχειρητική παρακολούθηση**
2.1.3	**Χειρουργική προσπέλαση**<br>Στις περισσότερες περιπτώσεις θα πρέπει να προτιμάται μια ελάχιστα επεμβατική προσέγγιση.
2.1.4	**Ταχεία εισαγωγή στην αναισθησία**<br>Ταχεία εισαγωγή στην αναισθησία θα πρέπει να χρησιμοποιείται για τη μείωση της εισρόφησης του γαστρικού περιεχομένου.
2.1.5	**Περιεγχειρητική οξυγόνωση**<br>Θα πρέπει να χρησιμοποιείται ένα κλάσμα εισπνεόμενου οξυγόνου(FiO2) μεταξύ 0,6 και 0,8.
2.1.6	**-Χορήγηση υγρών:**<br>Θα πρέπει να χρησιμοποιείται στοχο-κατευθυνόμενη (goal directed) χορήγηση υγρών με χρήση μη επεμβατικών συστημάτων αιμοδυναμικής παρακολούθησης. Εάν δεν υπάρχουν τέτοια συστήματα, θα πρέπει να χορηγούνται συνεχώς ισορροπημένα διαλύματα ανάλογα με τη χειρουργική προσέγγιση: 3-5 ml/kg/h για λαπαροσκόπηση και 5-7 ml/kg/h για λαπαροτομία.
2.1.7	**Αποφύγετε την τοποθέτηση καθετήρα ούρων**

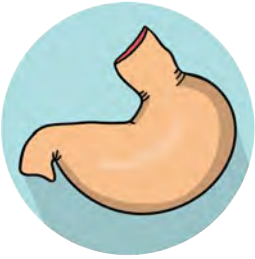

	Χρησιμοποιήστε μόνο εάν είναι απαραίτητο.
2.1.8	**Αποφύγετε την τοποθέτηση ρινογαστρικού σωλήνα**<br>Χρησιμοποιήστε μόνο εάν είναι απαραίτητο.
2.1.9	**Διατηρήστε τη νορμοθερμία**<br>Χρησιμοποιήστε θερμαινόμενες κουβέρτες και θερμά υγρά.
2.1.10	**Περιεγχειρητικός έλεγχος γλυκόζης αίματος**<br>Για τους διαβητικούς ασθενείς χρησιμοποιήστε το τοπικό νοσοκομειακό πρωτόκολλο για διαβητικούς ασθενείς που υποβάλλονται σε χειρουργική επέμβαση. Σε ασθενείς που κινδυνεύουν να αναπτύξουν αντίσταση στην ινσουλίνη (παχύσαρκοι και ηλικιωμένοι ασθενείς) και σε χειρουργικές επεμβάσεις που διαρκούν περισσότερο από 1 ώρα, αποφύγετε επίπεδα γλυκόζης στο αίμα υψηλότερα από 180 mg/dL.
2.1.11	**Προφύλαξη μετεγχειρητικής ναυτίας και εμέτου**<br>Χορηγήστε αντιεμετική θεραπεία σύμφωνα με την κλίμακα Apfel.
2.1.12	**Αποφύγετε την τοποθέτηση κοιλιακών παροχετεύσεων**<br>Χρησιμοποιήστε μόνο εάν είναι απαραίτητο.
2.1.13	**Πολυπαραγοντική αναλγησία με αποφυγή των οπιοειδών**<br>Θα πρέπει να χρησιμοποιείται πολυπαραγοντική αναλγησία με αποφυγή των οπιοειδών συμπεριλαμβανομένης της διήθησης των σημείων εισόδου των λαπαροσκοπικών τροκάρ με τοπικό αναισθητικό ή αποκλεισμός στο επίπεδο του Εγκάρσιου Κοιλιακού Μυός (TAP BLOCK - transabdominal plan block).
2.1.14	**Θρομβοεμβολική προφύλαξη**<br>Η θρομβοεμβολική προφύλαξη που αποτελείται από κάλτσες συμπίεσης ή διαλείπουσα συμπίεση και ηπαρίνη χαμηλού μοριακού βάρους θα πρέπει να χορηγείται σύμφωνα με το τοπικό νοσοκομειακό πρωτόκολλο.
2.1.15	**Δέσμες μέτρων περιεγχειρητικής φροντίδας**<br>Συνιστώνται δέσμες μέτρων περιεγχειρητικής φροντίδας για την πρόληψη λοιμώξεων του χειρουργικού πεδίου.
2.2	Άμεσα μετεγχειρητικά<br><br>Αναισθησιολόγος, Χειρουργός, Νοσηλευτής

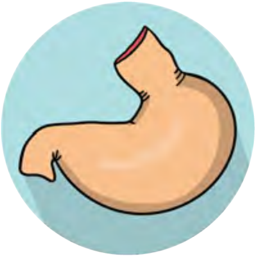

Πρωτόκολλο Eupemen
για Οξεία Σκωληκοειδίτιδα

2.2.1	**Ενεργητική διατήρηση θερμοκρασίας.**<br>Η θερμοκρασία του σώματος πρέπει να μετράται τακτικά με στόχο την πρόληψη της υποθερμίας.
2.2.2	**Οξυγονοθεραπεία**<br>Ο κορεσμός οξυγόνου πρέπει να μετράται τακτικά για να αποφευχθεί ο υποκορεσμός.
2.2.3	**Πολυπαραγοντική αναλγησία με περιορισμό των οπιοειδών**
2.2.4	**Περιοριστική χορήγηση υγρών**
2.2.5	**Περιεγχειρητικός έλεγχος γλυκόζης αίματος**<br>Για τους διαβητικούς ασθενείς χρησιμοποιήστε το τοπικό νοσοκομειακό πρωτόκολλο για διαβητικούς που υποβάλλονται σε χειρουργική επέμβαση. Σε ασθενείς που κινδυνεύουν να αναπτύξουν αντίσταση στην ινσουλίνη (παχύσαρκοι και ηλικιωμένοι ασθενείς) και σε χειρουργικές επεμβάσεις που διαρκούν περισσότερο από 1 ώρα, αποφύγετε επίπεδα γλυκόζης στο αίμα υψηλότερα από 180 mg/dL.
2.2.6	**Πρώιμη κινητοποίηση**<br>Οι ασθενείς θα πρέπει να κάθονται 2 ώρες μετά την επέμβαση και θα πρέπει να ξεκινούν βάδιση 8 ώρες μετά την επέμβαση λαμβάνοντας υπόψιν πάντα τις ώρες του νυχτερινού ύπνου.
2.2.7	**Πρώιμη σίτιση**<br>Οι ασθενείς θα πρέπει να αρχίσουν να πίνουν 4 ώρες μετά την επέμβαση.
2.2.8	**Θρομβοεμβολική προφύλαξη**<br>Η θρομβοεμβολική προφύλαξη που αποτελείται από κάλτσες συμπίεσης ή διαλείπουσα συμπίεση και ηπαρίνη χαμηλού μοριακού βάρους θα πρέπει να χορηγείται σύμφωνα με το τοπικό νοσοκομειακό πρωτόκολλο.
2.2.9	**Αντιβιοτική θεραπεία**<br>Τα αντιβιοτικά πρέπει να χορηγούνται θεραπευτικά για επιπλεγμένη σκωληκοειδίτιδα. Η επιλογή των αντιβιοτικών θα πρέπει να γίνεται σύμφωνα με το τοπικό νοσοκομειακό πρωτόκολλο.

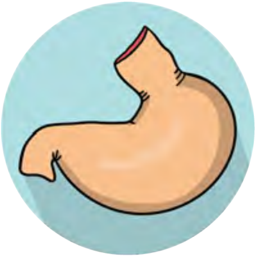

Πρωτόκολλο Eupemen
για Οξεία Σκωληκοειδίτιδα

3	1η Μετεγχειρητική μέρα<br>(θάλαμος νοσηλείας)<br><br>Χειρουργός, Νοσηλευτής
3.1	**Πρώιμη σίτιση**<br>Η σίτιση με ημιστερεή τροφή πρέπει να ξεκινά.
3.2	**Πρώιμη κινητοποίηση**<br>Ο ασθενής πρέπει να είναι πλήρως κινητοποιημένος.
3.3	**Αναπνευστική φυσιοθεραπεία**
3.4	**Από του στόματος αναλγησία**<br>Θα πρέπει να χορηγείται από του στόματος αναλγησία με αποφυγή των οπιοειδών.
3.5	**Αποφύγετε την ενδοφλέβια χορήγηση υγρών**<br>Εάν οι ασθενείς ανέχονται τα υγρά από του στόματος, διακόψτε την ενδοφλέβια χορήγηση υγρών.
3.6	**Θρομβοεμβολική προφύλαξη**<br>Η θρομβοεμβολική προφύλαξη που αποτελείται από κάλτσες συμπίεσης ή διαλείπουσα συμπίεση και ηπαρίνη χαμηλού μοριακού βάρους θα πρέπει να χορηγείται σύμφωνα με το τοπικό νοσοκομειακό πρωτόκολλο.
4	2η Μετεγχειρητική μέρα<br><br>Χειρουργός, Νοσηλευτής
4.1	**Πρώιμη σίτιση**<br>Σίτιση με ημιστερεή / στερεή τροφή.
4.2	**Πρώιμη κινητοποίηση**<br>Ο ασθενής πρέπει να είναι πλήρως κινητοποιημένος
4.3	**Από του στόματος αναλγησία**<br>Θα πρέπει να χορηγείται από του στόματος αναλγησία με αποφυγή των οπιοειδών.

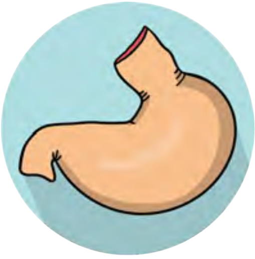

Πρωτόκολλο Eupemen
για Οξεία Σκωληκοειδίτιδα

4.4	**Αποφύγετε την ενδοφλέβια χορήγηση υγρών**<br>Εάν οι ασθενείς ανέχονται τα υγρά από του στόματος, διακόψτε την ενδοφλέβια χορήγηση υγρών.
4.5	**Θρομβοεμβολική προφύλαξη**<br>Η θρομβοεμβολική προφύλαξη που αποτελείται από κάλτσες συμπίεσης ή διαλείπουσα συμπίεση και ηπαρίνη χαμηλού μοριακού βάρους θα πρέπει να χορηγείται σύμφωνα με το τοπικό νοσοκομειακό πρωτόκολλο.
4.6	**Πρώιμο εξιτήριο από το νοσοκομείο**<br>Αξιολογήστε τα κριτήρια εξιτηρίου.
5	Υπολοίπο της νοσοκομειακής νοσηλείας<br><br>Χειρουργός, Νοσηλευτής
5.1	**Πρώιμη σίτιση**
5.2	**Πρώιμη κινητοποίηση**
5.3	**Αναπνευστική φυσιοθεραπεία**
5.4	**Από του στόματος αναλγησία**
5.5	**Αντιβιοτική θεραπεία**
5.6	**Θρομβοεμβολική προφύλαξη**
5.7	**Αξιολογήστε τα κριτήρια εξιτήριου**
6	Εξιτήριο<br><br>Χειρουργός, Νοσηλευτής Πρωτοβάθμια περίθαλψη
6.1	**Θρομβοεμβολική προφύλαξη**

EUPEMEN
European Perioperative Medical Networking

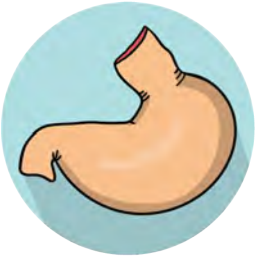

	Συνεχιζόμενη εξατομικευμένη θρομβοπροφύλαξη ανάλογα με τους κινδύνους.
6.2	**Αντιβιοτική θεραπεία**<br>Εξετάστε το ενδεχόμενο να συνεχίσετε τη θεραπεία με αντιβιοτικά μετά το εξιτήριο.
6.3	**Εργαστηριακές εξετάσεις αίματος**<br>Εργαστηριακές εξέτασεις αίματος με τουλάχιστον 50% μείωση της C-αντιδρώσας πρωτεΐνης πριν από το εξιτήριο.
6.4	**Παρακολούθηση**<br>Παρακολούθηση μετά το εξιτήριο σε 24 ώρες σε εξωτερικό ιατρείο ή μέσω τηλεφώνου. Προγραμματίστε τους ασθενείς για έλεγχο σύμφωνα με το τοπικό νοσοκομειακό πρωτόκολλο. Συντονίστε την υποστήριξη στο σπίτι με την πρωτοβάθμια φροντίδα, εάν χρειάζεται.
6.5	**Κριτήρια Εξιτηρίου**<br>Γενικά Κριτήρια Εξιτηρίου: χωρίς επιπλοκές που δεν μπορούν να αντιμετωπιστούν σε εξωτερικό ιατρείο, χωρίς πυρετό, πόνος ελεγχόμενος με από του στόματος αναλγησία, αποδοχή από τον ασθενή.
