# Supplementary material for: Advancing abdominal surgery recovery implementation: a unified framework for intensified recovery protocols by the EUropean PErioperative MEdical Networking collaborative
Source: Front Surg. 2026 May 18;13:1827678. doi: 10.3389/fsurg.2026.1827678 (PMC13223102; doi:10.3389/fsurg.2026.1827678)
Supplement: Supplementary file 15 [file Datasheet6.pdf]

Co-funded by the Erasmus+ Programme of the European Union

EUPEMEN
European Perioperative Medical Networking

EUPEMEN PROTOCOLLI (IT)

Instituto de Investigación Sanitaria Aragón

SERVIZIO SANITARIO REGIONALE EMILIA-ROMAGNA
Azienda Unità Sanitaria Locale di Ferrara

2. LÉKAŘSKÁ FAKULTA UNIVERZITA KARLOVA

UNIVERSITAS Miguel Hernández

ΓΕΝΙΚΟ ΝΟΣΟΚΟΜΕΙΟ ΘΕΣΣΑΛΟΝΙΚΗΣ "Γ. ΠΑΠΑΝΙΚΟΛΑΟΥ"

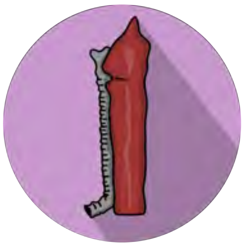

PROTOCOLLO EUPEMEN

ESOFAGECTOMIA

1	Prima del ricovero<br><br>Anestesista, Chirurgo, Infermiera, Dietista
1.1	**Colloquio preoperatorio**<br>Il paziente deve essere pienamente informato sulla procedura e sul decorso perioperatorio sia verbalmente che per iscritto. Dovrebbe essere firmato il consenso informato.
1.2	**Valutazione clinica completa**<br>Ciò dovrebbe includere anamnesi, esame obiettivo, radiografia del torace, esami del sangue (parametri della coagulazione, profilo biochimico, profilo nutrizionale ed emocromo completo) ed elettrocardiogramma.
1.3	**Controllo delle malattie croniche**<br>Tutte le malattie croniche dovrebbero essere ottimizzate prima dell'intervento chirurgico. Tutti i casi di malattie cardiovascolari di recente insorgenza o attive devono essere valutati da un cardiologo.
1.4	**Valutazione del Diabete Mellito**<br>Dovrebbero essere studiati i livelli di glucosio nel sangue e di HbA1c. Tutti i casi di diabete scarsamente controllato o non precedentemente, diagnosticato, dovrebbero essere indirizzati alle cure primarie o dall'endocrinologo prima dell'intervento chirurgico.
1.5	**Valutazione e trattamento marziale in caso di carenza di ferro e anemia**<br>L'anemia da carenza di ferro dovrebbe essere trattata, idealmente, con la somministrazione di ferro per via parenterale.
1.6	**Screening nutrizionale**<br>Lo screening nutrizionale dovrebbe essere effettuato utilizzando il *Malnutrition University Screening Tool* (MUST). Per i casi di afagia valutare misure e vie per la somministrazione della nutrizione artificiale secondo la politica ospedaliera locale. Per la disfagia solida somministrare una dieta liquida con integratori proteici.
1.7	**Smettere di fumare e ridurre il consumo di bevande alcoliche**<br>L'uso del tabacco dovrebbe essere interrotto e il consumo di alcol dovrebbe essere ridotto non appena viene fatta la diagnosi.
1.8	**Esercizio cardiovascolare**<br>Programmare esercizi di potenziamento cardiovascolare e respiratorio adeguati allo stato fisico del paziente.
1.9	**Valutazione psicologica**<br>Qualsiasi problema psicologico il paziente possa avere dovrebbe essere affrontato in maniera completa.
1.10	**Valutazione della fragilità**<br>Per i pazienti di età superiore ai 65 anni dovrebbe essere eseguita una valutazione della fragilità.
1.11	**Apfel score**<br>Il rischio di nausea e vomito intraoperatori dovrebbe essere valutato con il punteggio di Apfel.

EUPEMEN European Perioperative Medical Networking

Co-funded by the Erasmus+ Programme of the European Union

Cátedra GERM Medicina Perioperatoria Universidad Zaragoza

1.12	**Rischio anestesiologico ASA**<br>Nell'ambito della valutazione anestesiologica preoperatoria deve essere calcolato il punteggio ASA.
2	Perioperatorio
2.1	Preoperatorio Immediato<br><br>Anestesista, Chirurgo, Infermiera
2.1.1	**Igiene preoperatoria**<br>Il paziente viene istruito a fare una doccia o un bagno completo la sera o la mattina prima dell'intervento.
2.1.2	**Calze elastiche o compressione pneumatica intermittente**<br>Calze elastiche o compressione pneumatica intermittente dovrebbero essere indossate dal momento del ricovero in ospedale.
2.1.3	**Eparina a Basso Peso Molecolare**<br>L'Eparina a Basso Peso Molecolare dovrebbe essere somministrata 2-12 ore prima dell'intervento chirurgico (a seconda che l'anestesia sia neuroassiale eseguita o meno).
2.1.4	**Bevanda con carboidrati**<br>Una bevanda ricca di carboidrati (maltodestrine al 12,5%) 800 ml dovrebbe essere assunta la sera prima dell'intervento chirurgico e 400 ml 2 ore prima dell'anestesia. Per i pazienti diabetici somministrare in concomitanza la terapia per il diabete.
2.1.5	**Digiuno preoperatorio**<br>Digiuno di 6 ore per i solidi e 2 ore per i liquidi chiari.
2.1.6	**Tricotomia con rasoio elettrico**<br>Il sito in cui verrà eseguita l'incisione dovrebbe essere rasato con un rasoio elettrico, se necessario.
2.1.7	**Profilassi antibiotica**<br>La profilassi antibiotica 30-60 minuti prima dell'incisione chirurgica. Negli interventi prolungati ripetere la dose secondo l'emivita dei farmaci.
2.1.8	**Svuotamento gastrico ritardato**<br>Per i pazienti con svuotamento gastrico ritardato dovrebbero essere adottate misure per la profilassi del rigurgito.
2.2	Intraoperatorio<br><br>Anestesista, Chirurgo, Infermiera
2.2.1	**WHO Surgical Safety Checklist**<br>La checklist dell'OMS per la sicurezza in chirurgia dovrebbe essere completata prima di eseguire l'incisione.
2.2.2	**Monitoraggio intraoperatorio di routine**

		Durante la procedura si dovrebbero monitorare le funzioni vitali, la FiO2, la profondità dell'anestesia, il blocco neuromuscolare e la glicemia.
2.2.3	**Chirurgia mininvasiva**<br>Gli approcci mininvasivi sono preferiti e dovrebbero essere utilizzati il più possibile.
2.2.4	**Evitare la cateterizzazione urinaria routinaria**
2.2.5	**Monitoraggio invasivo**<br>Di routine NON è richiesto un catetere arterioso invasivo, anche se dovrebbe essere usato per pazienti con gravi disturbi cardio-respiratori.
2.2.6	**Catetere venoso centrale**<br>I cateteri venosi centrali NON sono necessari di routine per resezioni minori e in assenza di fattori di rischio per insufficienza renale postoperatoria.
2.2.7	**Induzione e mantenimento dell'anestesia**<br>Anestestici a breve durata d'azione dovrebbero essere usati per l'induzione e il mantenimento dell'anestesia.
2.2.8	**Ossigenazione**<br>I pazienti devono ricevere ossigeno con una FiO2 superiore al 50%.
2.2.9	**Fluidoterapia**<br>L'ottimizzazione emodinamica con fluidoterapia guidata dall'obiettivo (*goal-directed*) utilizzando dispositivi validati è raccomandata nei pazienti ad alto rischio e nei pazienti sottoposti a intervento chirurgico con grandi perdite ematiche. In tutti gli altri casi si raccomanda la fluidoterapia restrittiva in base al peso ideale in perfusione continua, soluzione bilanciata (1-3 ml/kg/h per laparoscopia; 3-5 ml/kg/h per laparotomia). La perdita di sangue dovrebbe essere compensata con colloidi 1:1.
2.2.10	**Evitare il sondino naso-gastrico**<br>Il sondino naso-gastrico NON dovrebbe essere utilizzato di routine.
2.2.11	**Prevenzione dell'ipotermia**<br>La temperatura dovrebbe essere monitorata e la normotermia dovrebbe essere mantenuta mediante riscaldamento attivo (liquidi riscaldati, coperta riscaldata).
2.2.12	**Prevenzione della nausea e vomito post-operatori (PONV)**<br>Somministrare una terapia antiemetica secondo il punteggio di Apfel.
2.2.13	**Analgesia epidurale**<br>L'analgesia epidurale toracica dovrebbe essere utilizzata in chirurgia a cielo aperto. In chirurgia laparoscopica NON è raccomandata di routine. I pazienti con controindicazione per l'analgesia epidurale e che sono a rischio di insufficienza renale o coagulopatia postoperatoria potrebbero trarre beneficio dal blocco del piano addominale trasversale (TAP block) bilaterale o altri metodi alternativi all'analgesia epidurale.
2.2.14	**Coadiuvanti analgesici per via endovenosa**<br>Gli analgesici adiuvanti consigliati sono i farmaci antinfiammatori non steroidei, la lidocaina, la ketamina, il solfato di magnesio e la dexmedetomidina.
2.2.15	**Controllo glicemico perioperatorio**

	Nei pazienti a rischio di sviluppare insulino-resistenza, evitare livelli di glucosio nel sangue superiori a 180 mg/dl.
2.2.16	**Disinfezione cutanea**<br>La cute deve essere disinfettata partendo dalla parte centrale verso la periferia con clorexidina in una soluzione alcolica all'1%.
2.2.17	**Evitare drenaggi addominali**<br>I drenaggi addominali dovrebbero essere evitati il più possibile.
2.3	Postoperatorio Immediato<br>(Terapia intensiva / Unità di Terapia Semintensiva in casi selezionati)<br><br>Anestesista, Infermiera
2.3.1	**Mantenimento della normotermia**<br>La temperatura dovrebbe essere misurata regolarmente e mantenuta.
2.3.2	**Analgesia a risparmio di oppioidi**<br>Dovrebbe essere utilizzata un'analgesia multimodale attiva o preventiva. Limitare l'uso di oppioidi. Puntare a un punteggio VAS inferiore a 3.
2.3.3	**Restrizione dei fluidi per via endovenosa**
2.3.4	**Ripresa precoce dell'alimentazione orale**<br>Inizio dell'assunzione di liquidi per via orale a partire da 6 ore dopo l'intervento chirurgico.
2.3.5	**Fisioterapia respiratoria**
2.3.6	**Mobilizzazione precoce**<br>La mobilizzazione dovrebbe iniziare 3 ore dopo l'intervento chirurgico sedendosi nel letto.
2.3.7	**Profilassi tromboembolica**<br>L'Eparina a Basso Peso Molecolare dovrebbe essere somministrata 12 ore dopo l'intervento chirurgico.
2.3.8	**Terapia della nausea e vomito postoperatori**
2.3.9	**Mantenimento di FiO2 0,5% per 2 ore dopo l'intervento chirurgico**
3	1ª Giornata Postoperatoria<br>(Terapia intensiva / Unità di Terapia Semintensiva in casi selezionati)<br><br>Chirurgo Anestesista, Infermiera
3.1	**Alimentazione precoce**<br>Una dieta liquida o semi-solida dovrebbe essere iniziata come tollerata. La nutrizione parenterale totale dovrebbe essere somministrata quando la dieta orale non è tollerata.

EUPEMEN European Perioperative Medical Networking
Co-funded by the Erasmus+ Programme of the European Union
Cátedra GERM Medicina Perioperatoria Universidad Zaragoza

3.2	**Terapia restrittiva dei fluidi per via endovenosa**
3.3	**Mobilizzazione precoce**<br>I pazienti dovrebbero essere incoraggiati a spostarsi dal letto alla poltrona vicino al letto.
3.4	**Analgesia a risparmio di oppioidi**<br>Garantire un buon controllo del dolore. Puntare a un punteggio VAS inferiore a 3.
3.5	**Rimuovere il catetere vescicale**<br>Se è stato posizionato un catetere vescicale, valutare se può essere rimosso.
3.6	**Fisioterapia respiratoria**
3.7	**Profilassi tromboembolica**<br>La profilassi tromboembolica mediante calze elastiche o a compressione intermittente ed eparina a basso peso molecolare deve essere somministrata secondo la politica ospedaliera locale.
3.8	**Prevenzione della nausea e vomito post-operatori (PONV)**<br>Somministrare una terapia antiemetica secondo il punteggio di Apfel.
4	2ª Giornata Postoperatoria<br>(Terapia intensiva / Unità di Terapia Semintensiva in casi selezionati)<br><br>Chirurgo Anestesista, Infermiera
4.1	**Alimentazione precoce**<br>Aumentare la nutrizione per via orale. Somministrare ai pazienti diete semi-solide (puree, yogurt, ecc.).
4.2	**Stop infusioni endovenose**
4.3	**Mobilizzazione precoce**<br>I pazienti dovrebbero essere in grado di camminare per brevi distanze.
4.4	**Analgesia a risparmio di oppioidi**<br>Garantire un buon controllo del dolore. Puntare a un punteggio VAS inferiore a 3.
4.5	**Rimuovere il catetere epidurale**
4.6	**Fisioterapia respiratoria**
4.7	**Profilassi tromboembolica**
5	3ª Giornata Postoperatoria<br>(Reparto)<br><br>Chirurgo, Infermiera
5.1	**Alimentazione precoce**<br>Dieta frullata.

EUPEMEN European Perioperative Medical Networking

Co-funded by the Erasmus+ Programme of the European Union

Cátedra GERM Medicina Perioperatoria Universidad Zaragoza

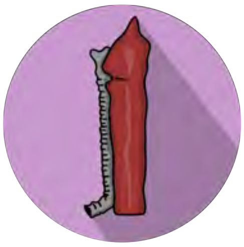

5.2	**Mobilizzazione precoce**<br>Deambulazione completa.
5.3	**Analgesia a risparmio di oppioidi**<br>Garantire un buon controllo del dolore. Puntare a un punteggio VAS inferiore a 3.
5.4	**Fisioterapia respiratoria**
5.5	**Profilassi tromboembolica**
5.6	**Esami di laboratorio**<br>Esami di laboratorio che includano anche la proteina C-reattiva e procalcitonina dovrebbero essere eseguiti.
5.7	**Valutare i criteri di dimissibilità**<br>Considerare la dimissione se NON ci sono complicazioni chirurgiche che non possano essere gestite in regime ambulatoriale, assenza di febbre, dolore controllato con analgesia orale, piena deambulazione, tolleranza all'assunzione orale di cibo e accettazione da parte del paziente.
6	4ª Giornata Postoperatoria, Dinissione e Follow-up<br>Chirurgo, Infermiera, Cure Primarie
6.1	**Alimentazione precoce**<br>Dieta morbida.
6.2	**Mobilizzazione precoce**<br>Deambulazione completa.
6.3	**Analgesia a risparmio di oppioidi**<br>Garantire un buon controllo del dolore. Puntare a un punteggio VAS inferiore a 3.
6.4	**Fisioterapia respiratoria**
6.5	**Profilassi tromboembolica**
6.6	**Esami di laboratorio**<br>Esami di laboratorio che includano anche la proteina C-reattiva e procalcitonina dovrebbero essere eseguiti.
7	Alla dimissione<br>Chirurgo, Infermiera, Cure Primarie
7.1	**Lettera di dimissione**<br>Alla dimissione i pazienti dovrebbero ricevere informazioni sul decorso ospedaliero e un foglio di raccomandazioni, indicazioni dietetiche e un questionario di gradimento.

EUPEMEN European Perioperative Medical Networking
Co-funded by the Erasmus+ Programme of the European Union
Cátedra GERM Medicina Perioperatoria Universidad Zaragoza

7.2	**Follow-up del paziente**<br>I pazienti dovrebbero essere seguiti in regime ambulatoriale o per telefono (secondo il protocollo di ciascun centro). Dovrebbe essere programmata una visita di controllo dal proprio medico di base e di altri specialisti, se necessario.
7.3	**Dieta**<br>Valutare l'apporto calorico, proteico, di sali minerali e vitaminico in base alle esigenze del singolo paziente.

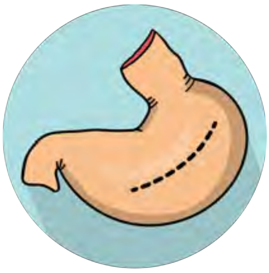

PROTOCOLLO EUPEMEN

RESEZIONE GASTRICA

1	Prima del ricovero<br><br>Anestesista, Chirurgo, Infermiere, Dietista
1.1	**Colloquio preoperatorio**<br>Il paziente deve essere pienamente informato sulla procedura e sul decorso perioperatorio sia verbalmente che per iscritto. Dovrebbe essere firmato il consenso informato.
1.2	**Valutazione clinica completa**<br>Ciò dovrebbe includere anamnesi, esame obiettivo, radiografia del torace, esami del sangue (parametri della coagulazione, profilo biochimico, profilo nutrizionale ed emocromo completo) ed elettrocardiogramma.
1.3	**Controllo delle malattie croniche**<br>Tutte le malattie croniche dovrebbero essere ottimizzate prima dell'intervento chirurgico. La spirometria preoperatoria deve essere eseguita per i pazienti con malattia polmonare restrittiva. Valutazione cardiologica se il fattore di rischio cardiovascolare è maggiore di 3. Tutti i casi di malattie cardiovascolari di recente insorgenza o attive devono essere valutati da un cardiologo.
1.4	**Valutazione e trattamento marziale in caso di carenza di ferro e anemia**<br>L'anemia da carenza di ferro dovrebbe essere trattata, idealmente, con la somministrazione di ferro per via parenterale.
1.5	**Valutazione del Diabete Mellito**<br>Dovrebbero essere studiati i livelli di glucosio nel sangue e di HbA1c. Tutti i casi di diabete scarsamente controllato o non precedentemente, diagnosticato, dovrebbero essere indirizzati alle cure primarie o dall'endocrinologo prima dell'intervento chirurgico.
1.6	**Ottimizzazione nutrizionale**<br>Valutare lo stato nutrizionale con il test del *Malnutrition University Screening Tool* (MUST). Correggere le carenze nutrizionali preoperatorie tra cui calcio, ferro, vitamina D e vitamina B12. Per i pazienti afagici valutare le potenziali vie di somministrazione della nutrizione artificiale. Per la disfagia ai solidi somministrare diete liquide con integratori proteici ipercalorici.
1.7	**Smettere di fumare e ridurre il consumo di bevande alcoliche**<br>L'uso del tabacco dovrebbe essere interrotto e il consumo di alcol dovrebbe essere ridotto non appena viene fatta la diagnosi.
1.8	**Esercizio fisico**<br>Programmare esercizi di potenziamento cardiovascolare, respiratorio e muscolare adeguati allo stato fisico del paziente.
1.9	**Valutazione psicologica**<br>Qualsiasi problema psicologico il paziente possa avere dovrebbe essere affrontato in maniera completa.
1.10	**Apfel score**<br>Il rischio di nausea e vomito intraoperatori dovrebbe essere valutato con il punteggio di Apfel.
1.11	**Rischio anestesiologico ASA**<br>Nell'ambito della valutazione anestesiologica preoperatoria deve essere calcolato il punteggio ASA.

EUPEMEN European Perioperative Medical Networking

Co-funded by the Erasmus+ Programme of the European Union

Cátedra GERM Medicina Perioperatoria Universidad Zaragoza

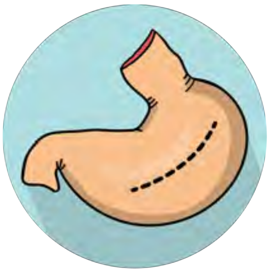

PROTOCOLLO EUPEMEN

RESEZIONE GASTRICA

2	Perioperatorio
2.1	Preoperatorio Immediato<br>(Programma il ricovero lo stesso dell’intervento chirurgico se possible)<br>Anestesista, Chirurgo, Infermiera
2.1.1	**Digiuno preoperatorio**<br>Ai pazienti NON dovrebbe essere consentito mangiare cibi solidi 8 ore prima dell'intervento e bere liquidi 2 ore prima dell'intervento.
2.1.2	**Eparina a basso peso molecolare**<br>L'Eparina a Basso Peso Molecolare dovrebbe essere somministrata 2-12 ore prima dell'intervento chirurgico (a seconda che l'anestesia sia neuroassiale eseguita o meno).
2.1.3	**Calze elastiche o compressione pneumatica intermittente**<br>Posizionare calze elastiche o compressione pneumatica intermittente a seconda del rischio tromboembolico.
2.1.4	**Carico di carboidrati**<br>Una singola bevanda a base di carboidrati (maltodestrine al 12,5%) pari a 400 ml dovrebbe essere somministrata 2 ore prima dell'anestesia, se non vi sono controindicazioni.
2.1.5	**Evitare premedicazione con ansiolitici**<br>NON prescrivere premedicazione con ansiolitici.
2.1.6	**Tricotomia con rasoio elettrico**<br>Il sito in cui verrà eseguita l'incisione dovrebbe essere rasato con un rasoio elettrico, se necessario.
2.1.7	**Profilassi antibiotica**<br>La profilassi antibiotica dovrebbe essere somministrata 30-60 minuti prima dell'incisione chirurgica. La scelta dell'antibiotico dovrebbe essere fatta in base al protocollo ospedaliero locale.
2.1.8	**Misure profilattiche per la prevenzione del rigurgito gastrico**<br>Per i pazienti con svuotamento gastrico ritardato dovrebbero essere adottate misure profilattiche per prevenire il rigurgito.
2.2	Intraoperatorio<br>Anestesista, Chirurgo, Infermiera
2.2.1	**WHO Surgical Safety Checklist**<br>La checklist dell'OMS per la sicurezza in chirurgia dovrebbe essere completata prima di eseguire l'incisione.
2.2.2	**Monitoraggio intraoperatorio di routine**<br>Durante la procedura si dovrebbero monitorare le funzioni vitali, la FiO2, la profondità dell'anestesia, il blocco neuromuscolare e la glicemia. Si raccomanda anche il monitoraggio emodinamico non invasivo.
2.2.3	**Evitare cateterismo arterioso**

EUPEMEN European Perioperative Medical Networking
Co-funded by the Erasmus+ Programme of the European Union
Cátedra GERM Medicina Perioperatoria Universidad Zaragoza

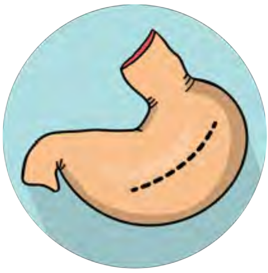

	Il catetere arterioso invasivo NON è richiesto di routine. Anche se dovrebbe essere usato per pazienti con gravi disturbi cardio-respiratori.
2.2.4	**Evitare cateterismo venoso centrale**<br>I cateteri venosi centrali NON sono necessari di routine per resezioni minori e in assenza di fattori di rischio per insufficienza renale postoperatoria.
2.2.5	**Evitare la cateterizzazione urinaria routinaria**
2.2.6	**Induzione e mantenimento dell'anestesia**<br>Anestestici a breve durata d'azione dovrebbero essere usati per l'induzione e il mantenimento dell'anestesia.
2.2.7	**Ossigenazione**<br>I pazienti devono ricevere ossigeno con una FiO2 superiore al 50%.
2.2.8	**Infusioni di liquidi per via endovenosa**<br>Si raccomanda l'ottimizzazione emodinamica utilizzando la fluidoterapia guidata dall'obiettivo (*goal directed*) con dispositivi validati. Se questi non fossero disponibili, si raccomanda una fluidoterapia restrittiva in base al peso ideale.
2.2.9	**Prevenzione dell'ipotermia**<br>La temperatura dovrebbe essere monitorata e la normotermia dovrebbe essere mantenuta mediante riscaldamento attivo (fluidi riscaldati, coperta riscaldata).
2.2.10	**Prevenzione della nausea e vomito postoperatori (PONV)**<br>Somministrare una terapia antiemetica secondo il punteggio di Apfel.
2.2.11	**Analgesia epidurale**<br>L'analgesia epidurale toracica dovrebbe essere utilizzata in chirurgia a cielo aperto. In chirurgia laparoscopica NON è raccomandata di routine. I pazienti con controindicazione per l'analgesia epidurale e che sono a rischio di insufficienza renale o coagulopatia postoperatoria potrebbero trarre beneficio dal blocco del piano addominale trasversale (TAP block) bilaterale o altri metodi alternativi all'analgesia epidurale.
2.2.12	**Chirurgia mininvasiva**<br>Gli approcci mininvasivi sono preferiti e dovrebbero essere utilizzati il più possibile.
2.2.13	**Evitare il sondino naso-gastrico**<br>I sondini nasogastrici sono consigliati solo intraoperatoriamente per svuotare lo stomaco.
2.2.14	**Evitare drenaggi addominali**
2.3	Immediato postoperatorio<br>Anestesista, Infermiera
2.3.1	**Mantenimento della normotermia**<br>La temperatura dovrebbe essere misurata regolarmente e mantenuta.
2.3.2	**Analgesia a risparmio di oppioidi**

Co-funded by the
Erasmus+ Programme
of the European Union

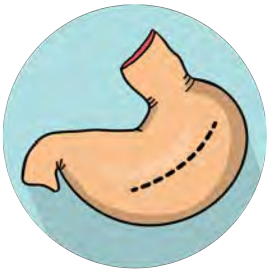

	Dovrebbe essere utilizzata un'analgesia multimodale attiva o preventiva. Limitare l'uso di oppioidi. Puntare a un punteggio VAS inferiore a 3.
2.3.3	**Ripresa precoce dell'alimentazione**<br>Inizio dell'assunzione di liquidi per via orale a partire da 6 ore dopo l'intervento chirurgico.
2.3.4	**Mobilizzazione precoce**<br>La mobilizzazione dovrebbe iniziare 3 ore dopo l'intervento con lo stare seduti a letto. La deambulazione dovrebbe iniziare 6 ore dopo l'intervento chirurgico rispettando le ore del sonno notturno.
2.3.5	**Profilassi tromboembolica**<br>L'Eparina a Basso Peso Molecolare dovrebbe essere somministrata 12 ore dopo l'intervento chirurgico.
2.3.6	**Profilassi della nausea e vomito postoperatori**<br>Somministrare una terapia antiemetica secondo il punteggio di Apfel.
3	1ª Giornata Postoperatoria<br>(Reparto)<br><br>Chirurgo, Infermiera
3.1	**Alimentazione precoce**<br>Dovrebbe essere iniziata una dieta liquida, come tollerata dal paziente.
3.2	**Mobilizzazione precoce**<br>I pazienti devono essere incoraggiati a camminare.
3.3	**Analgesia a risparmio di oppioidi**<br>Dovrebbe essere utilizzata un'analgesia multimodale attiva o preventiva. Limitare l'uso di oppioidi. Puntare a un punteggio VAS inferiore a 3.
3.4	**Stop fluidi per via endovenosa**<br>Se i pazienti tollerano un'adeguata assunzione di liquidi per via orale interrompere la fluidoterapia endovenosa.
3.5	**Rimozione catetere vescicale**<br>Valutare la rimozione del catetere urinario, se presente.
3.6	**Rimozione dei drenaggi addominali**<br>Valuta la rimozione dei drenaggi, se sono stati posizionati.
3.7	**Profilassi tromboembolica**
3.8	**Fisioterapia respiratoria**
4	2ª Giornata Postoperatoria<br>(Reparto)<br><br>Chirurgo, Infermiera
4.1	**Alimentazione precoce**<br>Somministrare ai pazienti diete semi-solide (puree, yogurt, ecc.).

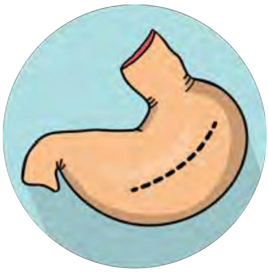

4.2	**Mobilizzazione precoce**<br>I pazienti devono essere incoraggiati a camminare.
4.3	**Analgesia a risparmio di oppioidi**<br>Dovrebbe essere utilizzata un'analgesia multimodale attiva o preventiva. Limitare l'uso di oppioidi. Puntare a un punteggio VAS inferiore a 3.
4.4	**Rimozione catetere epidurale**<br>Per prima cosa controllare i parametri di coagulazione e assicurarsi che l'intero catetere sia rimosso.
4.5	**Fisioterapia respiratoria e funzionale**
4.6	**Profilassi tromboembolica**
5	3ª Giornata Postoperatoria<br>(Reparto)<br><br>Chirurgo, Infermiera
5.1	**Alimentazione precoce**<br>Dieta morbida.
5.2	**Mobilizzazione precoce**<br>I pazienti devono essere incoraggiati a camminare.
5.3	**Analgesia a risparmio di oppioidi**<br>Dovrebbe essere utilizzata un'analgesia multimodale attiva o preventiva. Limitare l'uso di oppioidi. Puntare a un punteggio VAS inferiore a 3.
5.4	**Fisioterapia respiratoria e funzionale**
5.5	**Profilassi tromboembolica**
5.6	**Esami di laboratorio**<br>Questi dovrebbero includere la proteina C-reattiva, la procalcitonina e l'emocromo completo.
5.7	**Dimissione**<br>Considerare la dimissione se: assenza di complicanze chirurgiche, assenza di febbre, dolore controllato con analgesia orale, piena deambulazione, tolleranza all'assunzione di cibo per via orale e se il paziente è d'accordo.
6	4ª Giornata Postoperatoria<br>(Reparto)<br><br>Chirurgo, Infermiera
6.1	**Alimentazione precoce**<br>Dieta leggera.

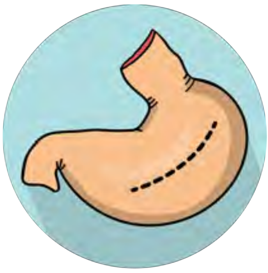

6.2	**Mobilizzazione precoce**<br>I pazienti devono essere incoraggiati a camminare.
6.3	**Analgesia a risparmio di oppioidi**<br>Dovrebbe essere utilizzata un'analgesia multimodale attiva o preventiva. Limitare l'uso di oppioidi. Puntare a un punteggio VAS inferiore a 3.
6.4	**Fisioterapia respiratoria e funzionale**
6.5	**Profilassi tromboembolica**
6.6	**Esami di laboratorio**<br>Questi dovrebbero includere la proteina C-reattiva, la procalcitonina e l'emocromo completo.
6.7	**Dimissione**<br>Considerare la dimissione se: assenza di complicanze chirurgiche, assenza di febbre, dolore controllato con analgesia orale, piena deambulazione, tolleranza all'assunzione di cibo per via orale e se il paziente è d'accordo.
7	Dimissione<br>Chirurgo, Infermiera, Psicologo, Cure Primarie
7.1	**Documentazione clinica del paziente (lettera di dimissione)**<br>Fornire ai pazienti informazioni sullo stato di salute e fornire raccomandazioni per ulteriori cure.
7.2	**Controllo post-dimissione**<br>Controllo telefonico dopo la dimissione. Coordinamento del supporto domiciliare con le cure primarie.
7.3	**Stato nutrizionale**<br>Valutare l'apporto calorico, proteico, minerale e vitaminico in base alle esigenze del paziente.
7.4	**Consulenza psicologica**<br>Indirizzare i pazienti a specialisti in psicologia, se necessario. Valutare la qualità di vita postoperatoria.

EUPEMEN European Perioperative Medical Networking

Co-funded by the Erasmus+ Programme of the European Union

Cátedra GERM Medicina Perioperatoria Universidad Zaragoza

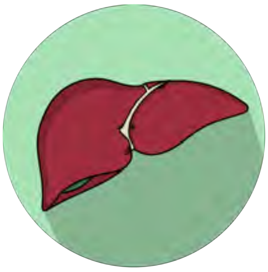

PROTOCOLLO EUPEMEN

RESEZIONE EPATICA

1	Prima del ricovero<br><br>Anestesista, Chirurgo, Infermiera, Dietista
1.1	**Colloquio preoperatorio**<br>Il paziente deve essere pienamente informato sulla procedura e sul decorso perioperatorio sia verbalmente che per iscritto. Dovrebbe essere firmato il consenso informato.
1.2	**Valutazione clinica completa**<br>Ciò dovrebbe includere anamnesi, esame obiettivo, radiografia del torace, esami del sangue (parametri della coagulazione, profilo biochimico, profilo nutrizionale ed emocromo completo) ed elettrocardiogramma.
1.3	**Controllo delle malattie croniche**<br>Tutte le malattie croniche dovrebbero essere ottimizzate prima dell'intervento chirurgico. Tutti i casi di malattie cardiovascolari di recente insorgenza o attive devono essere valutati da un cardiologo.
1.4	**Valutazione del Diabete Mellito**<br>Dovrebbero essere studiati i livelli di glucosio nel sangue e di HbA1c. Tutti i casi di diabete scarsamente controllato o non precedentemente, diagnosticato, dovrebbero essere indirizzati alle cure primarie o dall'endocrinologo prima dell'intervento chirurgico.
1.5	**Valutazione e trattamento marziale in caso di carenza di ferro e anemia**<br>L'anemia da carenza di ferro dovrebbe essere trattata, idealmente, con la somministrazione di ferro per via parenterale.
1.6	**Screening nutrizionale**<br>Lo screening nutrizionale dovrebbe essere effettuato utilizzando il *Malnutrition University Screening Tool* (MUST).
1.7	**Smettere di fumare e ridurre il consumo di bevande alcoliche**
1.8	**Esercizio cardiovascolare**<br>Programmare esercizi di potenziamento cardiovascolare e respiratorio adeguati allo stato fisico del paziente.
1.9	**Valutazione psicologica**<br>Qualsiasi problema psicologico il paziente possa avere dovrebbe essere affrontato in maniera completa.
1.10	**Valutazione della fragilità**<br>Per i pazienti di età superiore ai 65 anni dovrebbe essere eseguita una valutazione della fragilità.
1.11	**Apfel score**<br>Il rischio di nausea e vomito intraoperatori dovrebbe essere valutato con il punteggio di Apfel.

EUPEMEN European Perioperative Medical Networking
Co-funded by the Erasmus+ Programme of the European Union
Cátedra GERM Medicina Perioperatoria Universidad Zaragoza

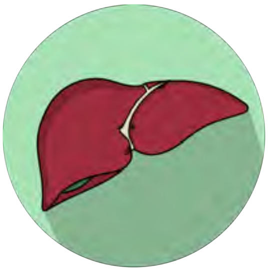

1.12	**Rischio anestesiologico ASA**<br>Nell'ambito della valutazione anestesiologica preoperatoria deve essere calcolato il punteggio ASA.
2	Perioperatorio
2.1	Preoperatorio Immediato<br>Anestesista, Chirurgo, Infermiera
2.1.1	**Igiene preoperatoria**<br>Il paziente viene istruito a fare una doccia o un bagno completo la sera o la mattina prima dell'intervento.
2.1.2	**Calze elastiche o compressione pneumatica intermittente**<br>Calze elastiche o compressione pneumatica intermittente dovrebbero essere indossate dal momento del ricovero in ospedale.
2.1.3	**Eparina a Basso Peso Molecolare**<br>L'Eparina a Basso Peso Molecolare dovrebbe essere somministrata 2-12 ore prima dell'intervento chirurgico (a seconda che l'anestesia sia neuroassiale eseguita o meno).
2.1.4	**Bevanda con carboidrati**<br>Una bevanda ricca di carboidrati (maltodestrine al 12,5%) 800 ml dovrebbe essere assunta la sera prima dell'intervento chirurgico e 400 ml 2 ore prima dell'anestesia. Per i pazienti diabetici somministrare in concomitanza la terapia per il diabete.
2.1.5	**Digiuno preoperatorio**<br>Digiuno di 6 ore per i solidi e 2 ore per i liquidi chiari.
2.1.6	**Tricotomia con rasoio elettrico**<br>Il sito in cui verrà eseguita l'incisione dovrebbe essere rasato con un rasoio elettrico, se necessario.
2.1.7	**Profilassi antibiotica**<br>La profilassi antibiotica 30-60 minuti prima dell'incisione chirurgica. Negli interventi prolungati ripetere la dose secondo l'emivita dei farmaci.
2.2	Intraoperatorio<br>Anestesista, Chirurgo, Infermiera
2.2.1	**WHO Surgical Safety Checklist**<br>La checklist dell'OMS per la sicurezza in chirurgia dovrebbe essere completata prima di eseguire l'incisione.

EUPEMEN European Perioperative Medical Networking

Co-funded by the Erasmus+ Programme of the European Union

Cátedra GERM Medicina Perioperatoria Universidad Zaragoza

2.2.2	**Monitoraggio intraoperatorio di routine**<br>Durante la procedura si dovrebbero monitorare le funzioni vitali, la FiO2, la profondità dell'anestesia, il blocco neuromuscolare e la glicemia. È raccomandato anche il monitoraggio emodinamico non invasivo.
2.2.3	**Chirurgia mininvasiva**<br>Gli approcci mininvasivi sono preferiti e dovrebbero essere utilizzati il più possibile.
2.2.4	**Evitare la cateterizzazione urinaria routinaria**
2.2.5	**Monitoraggio invasivo**<br>Di routine NON è richiesto un catetere arterioso invasivo, anche se dovrebbe essere usato per pazienti con gravi disturbi cardio-respiratori.
2.2.6	**Catetere venoso centrale**<br>I cateteri venosi centrali NON sono necessari di routine per resezioni minori e in assenza di fattori di rischio per insufficienza renale postoperatoria.
2.2.7	**Induzione e mantenimento dell'anestesia**<br>Anestestici a breve durata d'azione dovrebbero essere usati per l'induzione e il mantenimento dell'anestesia.
2.2.8	**Ossigenazione**<br>I pazienti devono ricevere ossigeno con una FiO2 superiore al 50%.
2.2.9	**Fluidoterapia**<br>Durante la fase della resezione è raccomandato il mantenimento della pressione venosa entrale al di sotto di 5 cmH20. Altrimenti, l'ottimizzazione emodinamica con fluidoterapia guidata dall'obiettivo (*goal-directed*) utilizzando dispositivi validati è raccomandata. Se questi non sono disponibili, si raccomanda la fluidoterapia restrittiva in base al peso ideale in perfusione continua, soluzione bilanciata (1-3 ml/kg/h per laparoscopia; 3-5 ml/kg/h per laparotomia). La perdita di sangue dovrebbe essere compensata con colloidi 1:1.
2.2.10	**Evitare il sondino naso-gastrico**<br>Il sondino naso-gastrico NON dovrebbe essere utilizzato di routine.
2.2.11	**Prevenzione dell'ipotermia**<br>La temperatura dovrebbe essere monitorata e la normotermia dovrebbe essere mantenuta mediante riscaldamento attivo (liquidi riscaldati, coperta riscaldata).
2.2.12	**Prevenzione della nausea e vomito post-operatori (PONV)**<br>Somministrare una terapia antiemetica secondo il punteggio di Apfel.
2.2.13	**Analgesia epidurale**<br>L'analgesia epidurale toracica dovrebbe essere utilizzata in chirurgia a cielo aperto. In chirurgia laparoscopica NON è raccomandata di routine. I pazienti con controindicazione per l'analgesia epidurale e che sono a rischio di insufficienza renale o coagulopatia postoperatoria potrebbero trarre beneficio dal blocco del piano addominale trasversale (TAP block) bilaterale o altri metodi

	alternativi all'analgesia epidurale.
2.2.14	**Coadiuvanti analgesici per via endovenosa**<br>Gli analgesici adiuvanti consigliati sono i farmaci antinfiammatori non steroidei, la lidocaina, la ketamina, il solfato di magnesio e la dexmedetomidina.
2.2.15	**Controllo glicemico perioperatorio**<br>Nei pazienti a rischio di sviluppare insulino-resistenza, evitare livelli di glucosio nel sangue superiori a 180 mg/dl.
2.2.16	**Disinfezione cutanea**<br>La cute deve essere disinfettata partendo dalla parte centrale verso la periferia con clorexidina in una soluzione alcolica all'1%.
2.2.17	**Evitare drenaggi addominali**<br>I drenaggi addominali dovrebbero essere evitati il più possibile.
2.3	Postoperatorio Immediato<br>(Terapia intensiva / Unità di Terapia Semintensiva in casi selezionati)<br><br>Anestesista, Infermiera
2.3.1	**Mantenimento della normotermia**<br>La temperatura dovrebbe essere misurata regolarmente e mantenuta.
2.3.2	**Analgesia a risparmio di oppioidi**<br>Dovrebbe essere utilizzata un'analgesia multimodale attiva o preventiva. Limitare l'uso di oppioidi. Puntare a un punteggio VAS inferiore a 3.
2.3.3	**Restrizione dei fluidi per via endovenosa**
2.3.4	**Ripresa precoce dell'alimentazione orale**<br>Inizio dell'assunzione di liquidi per via orale a partire da 3 ore dopo l'intervento chirurgico.
2.3.5	**Fisioterapia respiratoria**
2.3.6	**Mobilizzazione precoce**<br>La mobilizzazione dovrebbe iniziare 3 ore dopo l'intervento chirurgico sedendosi nel letto.
2.3.7	**Profilassi tromboembolica**<br>L'Eparina a Basso Peso Molecolare dovrebbe essere somministrata 12 ore dopo l'intervento chirurgico.
2.3.8	**Terapia della nausea e vomito postoperatori**
2.3.9	**Mantenimento di FiO2 0,5% per 2 ore dopo l'intervento chirurgico**

EUPEMEN European Perioperative Medical Networking

Co-funded by the Erasmus+ Programme of the European Union

Cátedra GERM Medicina Perioperatoria Universidad Zaragoza

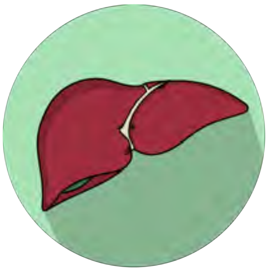

PROTOCOLLO EUPEMEN

RESEZIONE EPATICA

3	1ª Giornata Postoperatoria<br>(Reparto)<br><br>Chirurgo, Infermiera
3.1	**Integrazione nutrizionale ricca di proteine**<br>Integratori nutrizionali ricchi di proteine dovrebbero essere somministrati a pazienti con assunzione orale <60% del fabbisogno energetico o malnutrizione preoperatoria.
3.2	**Alimentazione precoce**<br>Una dieta semi-solida o normae dovrebbe essere iniziata.
3.3	**Mobilizzazione precoce**<br>I pazienti dovrebbero essere incoraggiati a spostarsi dal letto alla poltrona vicino al letto.
3.4	**Rimozione dei drenaggi addominali**<br>Valutare la rimozione del drenaggio addominale, se presente.
3.5	**Analgesia a risparmio di oppioidi**<br>Garantire un buon controllo del dolore. Puntare a un punteggio VAS inferiore a 3.
3.6	**Sospendere infusioni per via endovenosa**<br>Sospendere i liquidi per via endovenosa se il paziente tollera i liquidi per via orale.
3.7	**Rimuovere il catetere vescicale**<br>Se è stato posizionato un catetere vescicale, valutare se può essere rimosso.
3.8	**Fisioterapia respiratoria**
3.9	**Prevenzione della nausea e vomito post-operatori (PONV)**
3.10	**Profilassi anti-ulcera**
3.11	**Profilassi tromboembolica**
3.12	**Valutare la dimissione ospedaliera**<br>Per i pazienti sottoposti a procedure laparoscopiche valutare i criteri di dimissione.
4	2ª Giornata Postoperatoria<br>(Reparto)<br><br>Chirurgo, Infermiera
4.1	**Alimentazione precoce.**<br>Dovrebbe essere somministrata una dieta normale.

EUPEMEN European Perioperative Medical Networking
Co-funded by the Erasmus+ Programme of the European Union
Cátedra GERM Medicina Perioperatoria Universidad Zaragoza

4.2	**Mobilizzazione precoce**<br>I pazienti dovrebbero essere in grado di camminare per brevi distanze.
4.3	**Analgesia a risparmio di oppioidi**<br>Garantire un buon controllo del dolore. Puntare a un punteggio VAS inferiore a 3.
4.4	**Profilassi tromboembolica**
4.5	**Esami di laboratorio**<br>Esami di laboratorio che includano anche la proteina C-reattiva e procalcitonina dovrebbero essere eseguiti.
4.6	**Valutare i criteri di dimissibilità**<br>Considerare la dimissione se NON ci sono complicazioni chirurgiche che non possano essere gestite in regime ambulatoriale, assenza di febbre, dolore controllato con analgesia orale, piena deambulazione, tolleranza all'assunzione orale di cibo e accettazione da parte del paziente.
5	Alla dimissione<br><br>Chirurgo, Infermiera, Cure Primarie
5.1	**Lettera di dimissione**<br>Al momento della dimissione, i pazienti devono ricevere informazioni personalizzate, comprensibili e complete sulla degenza ospedaliera e raccomandazioni per l'assistenza domiciliare.
5.2	**Follow-up**<br>I pazienti devono essere seguiti nella prima settimana dopo la dimissione in regime ambulatoriale o telefonicamente. Ulteriori visite di controllo dovrebbero essere programmate per 1, 3 e 6 mesi dopo la dimissione. Dovrebbe essere organizzata una visita dal medico di base e, se necessario, dovrebbe essere coordinato il supporto domiciliare.

EUPEMEN European Perioperative Medical Networking

Co-funded by the Erasmus+ Programme of the European Union

Cátedra GERM Medicina Perioperatoria Universidad Zaragoza

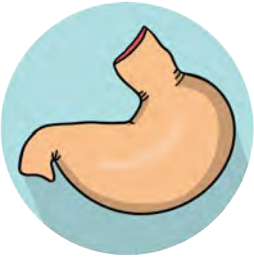

PROTOCOLLO EUPEMEN

CHIRURGIA BARIATRICA

1	Prima del ricovero<br><br>Anestesista, Chirurgo, Infermiera, Dietista
1.1	**Colloquio preoperatorio**<br>Il paziente deve essere pienamente informato sulla procedura e sul decorso perioperatorio sia verbalmente che per iscritto. Dovrebbe essere firmato il consenso informato.
1.2	**Valutazione clinica completa**<br>Ciò dovrebbe includere anamnesi, esame obiettivo, radiografia del torace, esami del sangue (parametri della coagulazione, profilo biochimico, profilo nutrizionale ed emocromo completo) ed elettrocardiogramma.
1.3	**Endoscopia digestiva superiore**<br>La valutazione endoscopica dell'esofago, dello stomaco e del duodeno dovrebbe essere eseguita come parte della preparazione preoperatoria. Ciò dovrebbe includere l'indagine per l'*Helicobacter pylori*, che se trovato dovrebbe essere eradicato prima dell'intervento chirurgico.
1.4	**Controllo delle malattie croniche**<br>Tutte le malattie croniche dovrebbero essere ottimizzate prima dell'intervento chirurgico. La spirometria preoperatoria dovrebbe essere eseguita per i pazienti con malattia polmonare restrittiva. Valutazione cardiologica se il fattore di rischio cardiovascolare è maggiore di 3. Tutti i casi di malattie cardiovascolari di recente insorgenza o attive devono essere valutati da un cardiologo.
1.5	**Valutazione del Diabete Mellito**<br>Dovrebbero essere studiati i livelli di glucosio e di HbA1c. Tutti i casi di diabete scarsamente controllato o non diagnosticato in precedenza devono essere indirizzati alle cure primarie o all'endocrinologo prima dell'intervento chirurgico.
1.6	**Screening per l'apnea durante il sonno**<br>Fare lo screening dell'apnea notturna con il test STOP-BANG. Eseguire la polisonnografia se il punteggio è maggiore di 3.
1.7	**Stato nutrizionale**<br>Perdita di peso prima dell'intervento chirurgico utilizzando una dieta ipocalorica o prodotti commerciali. Valutare i metodi aggiuntivi per la perdita di peso (farmaci, palloncino intragastrico). Correzione delle carenze nutrizionali pre-operatorie tra cui calcio, ferro, vitamina D e vitamina B12.
1.8	**Smettere di fumare e ridurre il consume di bevande alcoliche**<br>L'uso del tabacco dovrebbe essere interrotto e il consumo di alcol dovrebbe essere ridotto non appena viene fatta la diagnosi.
1.9	**Attività fisica**<br>Programmare esercizi di potenziamento cardiovascolare, respiratorio e muscolare adeguati allo stato fisico del paziente.
1.10	**Valutazione psicologica**

EUPEMEN European Perioperative Medical Networking
Co-funded by the Erasmus+ Programme of the European Union
Cátedra GERM Medicina Perioperatoria Universidad Zaragoza

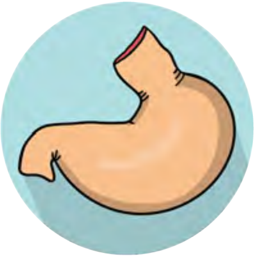

	Qualsiasi problema psicologico che il paziente possa avere dovrebbe essere affrontato in maniera completa.
1.11	**Valutazione ASA**<br>Nell'ambito della valutazione anestesiologica preoperatoria deve essere calcolato il punteggio ASA.
1.12	**Apfel score**<br>Il rischio di nausea e vomito intra-operatori dovrebbe essere valutato con il punteggio di Apfel.
2	Perioperatorio
2.1	Immediato pre-operatorio<br>(Programma il ricovero lo stesso dell'intervento chirurgico se possible)<br><br>Anestesista, Chirurgo, Infermiera
2.1.1	**Digiuno preoperatorio**<br>Ai pazienti NON dovrebbe essere consentito mangiare cibi solidi 8 ore prima dell'intervento e bere liquidi 2 ore prima dell'intervento.
2.1.2	**Eparina a basso peso molecolare**<br>L'eparina a basso peso molecolare dovrebbe essere somministrata 2-12 ore prima dell'intervento chirurgico (a seconda che l'anestesia sia neuroassiale eseguita o meno).
2.1.3	**Calze elastiche**<br>Posizionare calze elastiche o compressione pneumatica intermittente a seconda del rischio tromboembolico.
2.1.4	**Carico di carboidrati**<br>Una singola bevanda a base di carboidrati (maltodestrine al 12,5%) pari a 400 ml dovrebbe essere somministrata 2 ore prima dell'anestesia, se non vi sono controindicazioni.
2.1.5	**Evitare premedicazione con ansiolitici**<br>NON prescrivere premedicazione con ansiolitici.
2.1.6	**Tricotomia con rasoio elettrico**<br>Il sito in cui verrà eseguita l'incisione dovrebbe essere rasato con un rasoio elettrico, se necessario.
2.1.7	**Profilassi antibiotica**<br>La profilassi antibiotica dovrebbe essere somministrata 30-60 minuti prima dell'incisione chirurgica. La scelta dell'antibiotico dovrebbe essere fatta in base al protocollo ospedaliero locale.
2.1.8	**Misure profilattiche per la prevenzione del rigurgito gastrico**<br>Per i pazienti con svuotamento gastrico ritardato dovrebbero essere adottate misure profilattiche per prevenire il rigurgito.

EUPEMEN European Perioperative Medical Networking

Co-funded by the Erasmus+ Programme of the European Union

Cátedra GERM Medicina Perioperatoria Universidad Zaragoza

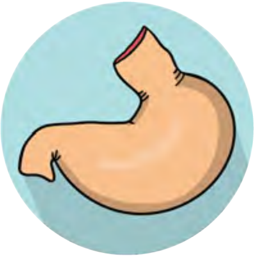

PROTOCOLLO EUPEMEN

CHIRURGIA BARIATRICA

2.2	Intraoperative<br><br>Anaesthetist, Surgeon, Nurse
2.2.1	**WHO Surgical Safety Checklist**<br>La checklist dell'OMS per la sicurezza in chirurgia dovrebbe essere completata prima di eseguire l'incisione.
2.2.2	**Monitoraggio intraoperatorio di routine**<br>Durante la procedura si dovrebbero monitorare le funzioni vitali, la FiO2, la profondità dell'anestesia, il blocco neuromuscolare e la glicemia. Si raccomanda anche il monitoraggio emodinamico non invasivo.
2.2.3	**Evitare cateterismo arterioso**<br>Il catetere arterioso invasivo NON è richiesto di routine. Anche se dovrebbe essere usato per pazienti con gravi disturbi cardio-respiratori.
2.2.4	**Evitare cateterismo venoso centrale**<br>I cateteri venosi centrali NON sono necessari di routine per resezioni minori e in assenza di fattori di rischio per insufficienza renale postoperatoria.
2.2.5	**Evitare la cateterizzazione urinaria routinaria**
2.2.6	**Induzione e mantenimento dell'anestesia**<br>Anestestici a breve durata d'azione dovrebbero essere usati per l'induzione e il mantenimento dell'anestesia.
2.2.7	**Ossigenazione**<br>I pazienti devono ricevere ossigeno con una FiO2 superiore al 50%.
2.2.8	**Infusioni di liquidi per via endovenosa**<br>Si raccomanda l'ottimizzazione emodinamica utilizzando la fluidoterapia guidata dall'obiettivo (*goal directed*) con dispositivi validati. Se questi non fossero disponibili, si raccomanda una fluidoterapia restrittiva in base al peso ideale.
2.2.9	**Prevenzione dell'ipotermia**<br>La temperatura dovrebbe essere monitorata e la normotermia dovrebbe essere mantenuta mediante riscaldamento attivo (fluidi riscaldati, coperta riscaldata).
2.2.10	**Prevenzione della nausea e vomito postoperatori (PONV)**<br>Somministrare una terapia antiemetica secondo il punteggio di Apfel.
2.2.11	**Analgesia epidurale**<br>L'analgesia epidurale toracica dovrebbe essere utilizzata in chirurgia a cielo aperto. In chirurgia laparoscopica NON è raccomandata di routine. I pazienti con controindicazione per l'analgesia epidurale e che sono a rischio di insufficienza renale o coagulopatia postoperatoria potrebbero trarre beneficio dal blocco del piano addominale trasversale (TAP block) bilaterale o altri metodi alternativi all'analgesia epidurale.
2.2.12	**Chirurgia mininvasiva**<br>Gli approcci mininvasivi sono preferiti e dovrebbero essere utilizzati il più possibile.

EUPEMEN European Perioperative Medical Networking
Co-funded by the Erasmus+ Programme of the European Union
Cátedra GERM Medicina Perioperatoria Universidad Zaragoza

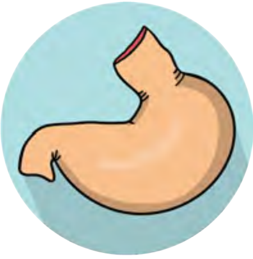

2.2.13	**Evitare dispositivi di protezione delle suturatrici o colle biologiche**<br>I metodi di rinforzo della linea di sutura, quali l'uso di dispositivi di rivestimento delle suturatrici o colle biologiche, NON riducono il rischio di deiscenza secondo le evidenze.
2.2.14	**Calibrare la gastrectomia verticale (sleeve)**<br>La gastrectomia verticale (sleeve) deve essere calibrata con le sonde.
2.2.15	**Evitare sondino naso-gastrico**<br>I sondini nasogastrici sono consigliati solo intraoperatoriamente per svuotare lo stomaco.
2.2.16	**Evitare drenaggi addominali**
2.3	Immediato post-operatorio<br><br>Anestesista, Infermiera
2.3.1	**Mantenimento della normotermia**<br>La temperatura dovrebbe essere misurata regolarmente e mantenuta.
2.3.2	**Analgesia a risparmio di oppioidi**<br>Dovrebbe essere utilizzata un'analgesia multimodale attiva o preventiva. Limitare l'uso di oppioidi. Puntare a un punteggio VAS inferiore a 3.
2.3.3	**Ripresa precoce dell'alimentazione**<br>Inizio dell'assunzione di liquidi per via orale a partire da 6 ore dopo l'intervento chirurgico.
2.3.4	**Mobilizzazione precoce**<br>La mobilizzazione dovrebbe iniziare 3 ore dopo l'intervento e dovrebbe iniziare con lo stare seduti a letto. La deambulazione dovrebbe iniziare 6 ore dopo l'intervento chirurgico rispettando le ore del sonno notturno.
2.3.5	**Profilassi tromboembolica**<br>L'Eparina a Basso Peso Molecolare dovrebbe essere somministrata 12 ore dopo l'intervento chirurgico.
2.3.6	**Profilassi della nausea e vomito postoperatori**<br>Somministrare una terapia antiemetica secondo il punteggio di Apfel.
2.3.7	**Trattamento dell'apnea notturna**<br>Nei pazienti con apnea notturna, ripristinare la pressione positiva continua delle vie aeree (CPAPA) appena possibile.
3	1ª Giornata Postoperatoria<br>(Reparto)<br><br>Chirurgo, Infermiera
3.1	**Alimentazione precoce**<br>Dovrebbe essere iniziata una dieta liquida, come tollerata dal paziente.

EUPEMEN European Perioperative Medical Networking

Co-funded by the Erasmus+ Programme of the European Union

Cátedra GERM Medicina Perioperatoria Universidad Zaragoza

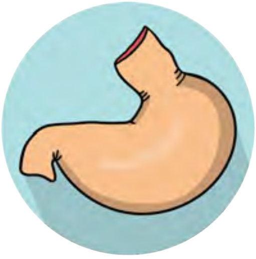

3.2	**Alimentazione precoce**<br>Dovrebbe essere iniziata una dieta liquida, come tollerata dal paziente.
3.3	**Mobilizzazione precoce**<br>I pazienti devono essere incoraggiati a camminare.
3.4	**Analgesia a risparmio di oppioidi**<br>Dovrebbe essere utilizzata un'analgesia multimodale attiva o preventiva. Limitare l'uso di oppioidi. Puntare a un punteggio VAS inferiore a 3.
3.5	**Stop fluidi per via endovenosa**<br>Se i pazienti tollerano un'adeguata assunzione di liquidi per via orale interrompere la fluidoterapia endovenosa.
3.6	**Rimozione catetere vescicale**<br>Valutare la rimozione del catetere urinario, se presente.
3.7	**Rimozione dei drenaggi addominali**<br>Se è stato posizionato un drenaggio addominale, valutarne la rimozione.
3.8	**Profilassi tromboembolica**
4	2ª Giornata Postoperatoria (e successive)<br>(Reparto)<br><br>Chirurgo, Infermiera
4.1	**Alimentazione precoce**<br>Somministrare ai pazienti una dieta completa ipocalorica liquida o una nutrizione completa iperproteica ipocalorica.
4.2	**Rimozione dei drenaggi addominali**<br>Valuta la rimozione dei drenaggi, se sono stati posizionati.
4.3	**Valuta la dimissione**<br>Considera la dimissione se non ci sono complicanze (salvo che possano essere gestite ambulatorialmente), in assenza di febbre, tachicardia o tachipnea, se dolore ben controllato con analgesia orale, il paziente deambula pienamente e tollera una dieta orale e se il paziente è d'accordo.
5	Dimissione<br><br>Chirurgo, Infermiera
5.1	**Dieta**<br>Dieta ipocalorica frullata o nutrizione orale iperproteica ipocalorica completa per le prime 1-2 settimane. Dopo 2 settimane dieta sminuzzata. Dieta solida dopo 1-2 mesi dall'intervento.
5.2	**Medicazione delle ferite**<br>Medicazione quotidiana delle ferite e rimozione di punti e/o graffette cutanee secondo protocollo.

5.3	**Exercizio**<br>Programma di esercizi che combini aerobica e allenamento della forza. Iniziare un mese dopo l'intervento a intensità moderata, progredendo a intensità più elevate
5.4	**Profilassi tromboembolica**<br>Tromboprofilassi raccomandata per le prime 3-4 settimane dopo l'intervento
5.5	**Continuità delle cure**<br>Tromboprofilassi raccomandata per le prime 3-4 settimane dopo l'intervento

EUPEMEN European Perioperative Medical Networking
Co-funded by the Erasmus+ Programme of the European Union
Cátedra GERM Medicina Perioperatoria Universidad Zaragoza

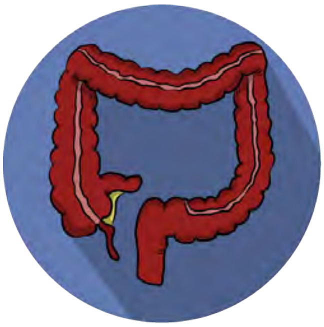

PROTOCOLLO EUPEMEN

RESEZIONE COLICA

1	Prima del ricovero<br><br>Anestesista, Chirurgo, Infermiera, Dietista, Stoma terapista
1.1	**Colloquio preoperatorio**<br>Il paziente deve essere pienamente informato sulla procedura e sul decorso perioperatorio sia verbalmente che per iscritto. Dovrebbe essere firmato il consenso informato.
1.2	**Valutazione clinica completa**<br>Ciò dovrebbe includere anamnesi, esame obiettivo, radiografia del torace, esami del sangue (parametri della coagulazione, profilo biochimico, profilo nutrizionale ed emocromo completo) ed elettrocardiogramma.
1.3	**Valutazione della fragilità**<br>Per i pazienti di età superiore ai 65 anni dovrebbe essere eseguita una valutazione della fragilità.
1.4	**Valutazione del rischio anestesiologico ASA**
1.5	**Apfel score**<br>Il rischio di nausea e vomito intraoperatori dovrebbe essere valutato con il punteggio di Apfel.
1.6	**Controllo delle malattie croniche**<br>Tutte le malattie croniche dovrebbero essere ottimizzate prima dell'intervento chirurgico. Tutti i casi di malattie cardiovascolari di recente insorgenza o attive devono essere valutati da un cardiologo.
1.7	**Valutazione del Diabete Mellito**<br>Dovrebbero essere studiati i livelli di glucosio nel sangue e di HbA1c. Tutti i casi di diabete scarsamente controllato o non precedentemente, diagnosticato, dovrebbero essere indirizzati alle cure primarie o dall'endocrinologo prima dell'intervento chirurgico.
1.8	**Valutazione e trattamento dell'anemia e carenza di ferro**<br>L'anemia da carenza di ferro dovrebbe essere idealmente trattata con la somministrazione di ferro per via parenterale.
1.9	**Screening nutrizionale**<br>Lo screening nutrizionale dovrebbe essere effettuato utilizzando il *Malnutrition University Screening Tool* (MUST). I pazienti a rischio di malnutrizione dovrebbero assumere integratori alimentari orali, preferibilmente immunonutrizione, per un periodo di 7 giorni prima e 5 giorni dopo l'intervento chirurgico.
1.10	**Smettere di fumare e ridurre il consumo di alcol almeno un mese prima dell'intervento**

EUPEMEN European Perioperative Medical Networking
Co-funded by the Erasmus+ Programme of the European Union
Cátedra GERM Medicina Perioperatoria Universidad Zaragoza

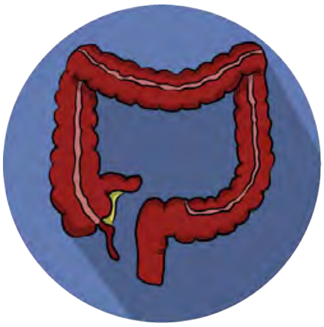

PROTOCOLLO EUPEMEN

RESEZIONE COLICA

1.11	**Preabilitazione multimodale che includa esercizi aerobici e di resistenza**
1.12	**Dieta a basso contenuto di fibre almeno 5 giorni prima dell'intervento chirurgico**
1.13	**Nessuna preparazione intestinale meccanica preoperatoria, esclusa la preparazione alla colonscopia**
1.14	**Clisteri**<br>Due clisteri rettali il pomeriggio prima dell'intervento chirurgico (procedure sul colon sinistro)
2	Perioperatorio
2.1	Preoperatorio Immediato<br>(Programma il ricovero lo stesso dell'intervento chirurgico se possible)<br><br>Anestesista, Chirurgo, Infermiera, Dietista, Stomaterapista
2.1.1	**Igiene preoperatoria**<br>Al paziente viene istruito per fare una doccia completa o un bagno la sera o la mattina prima dell'intervento.
2.1.2	**Calze elastiche o compressione pneumatica intermittente**<br>Calze elastiche o compressione pneumatica intermittente dovrebbero essere indossate dal momento del ricovero in ospedale.
2.1.3	**Eparina a basso peso molecolare**<br>L'Eparina a Basso Peso Molecolare dovrebbe essere somministrata 2-12 ore prima dell'intervento chirurgico (a seconda che l'anestesia sia neuroassiale eseguita o meno).
2.1.4	**Bevanda con carboidrati**<br>Una bevanda ricca di carboidrati (maltodestrine al 12,5%) 800 ml dovrebbe essere assunta la sera prima dell'intervento chirurgico e 400 ml 2 ore prima dell'anestesia. Per i pazienti diabetici somministrare in concomitanza la terapia per il diabete.
2.1.5	**Digiuno preoperatorio**<br>Digiuno di 6 ore per i solidi e 2 ore per i liquidi chiari.
2.1.6	**Tricotomia con rasoio elettrico**<br>Il sito in cui verrà eseguita l'incisione dovrebbe essere rasato con un rasoio elettrico, se necessario.
2.1.7	**Marcatura del sito per la stomia (se prevista)**
2.1.8	**Profilassi antibiotica**

EUPEMEN European Perioperative Medical Networking

Co-funded by the Erasmus+ Programme of the European Union

Cátedra GERM Medicina Perioperatoria Universidad Zaragoza

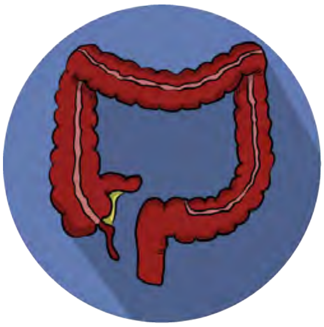

PROTOCOLLO EUPEMEN

RESEZIONE COLICA

	La profilassi antibiotica dovrebbe essere somministrata 30-60 minuti prima dell'incisione chirurgica. Negli interventi prolungati ripetere la dose secondo l’emivita dei farmaci.
2.2	Intraoperatorio<br><br>Anestesista, Chirurgo, Infermiera
2.2.1	**WHO Surgical Safety Checklist**<br>La checklist dell'OMS per la sicurezza in chirurgia dovrebbe essere completata prima di eseguire l'incisione.
2.2.2	**Monitoraggio intraoperatorio di routine**<br>Durante la procedura si dovrebbero monitorare le funzioni vitali, la FiO2, la profondità dell'anestesia, il blocco neuromuscolare e la glicemia.
2.2.3	**Chirurgia mininvasiva**<br>Gli approcci mininvasivi sono preferiti e dovrebbero essere utilizzati il più possibile. Si raccomandano livelli di pressione intraddominale compresi tra 8 e 12 mmHg.
2.2.4	**Evitare la cateterizzazione urinaria routinaria**
2.2.5	**Monitoraggio invasivo**<br>Il catetere arterioso invasivo NON è richiesto di routine.
2.2.6	**Catetere venoso centrale**<br>Il catetere venoso centrale NON è richiesto di routine.
2.2.7	**Induzione e mantenimento dell’anestesia**<br>Anestestici a breve durata d'azione dovrebbero essere usati per l'induzione e il mantenimento dell'anestesia.
2.2.8	**Ossigenazione**<br>I pazienti devono ricevere ossigeno con una FiO2 superiore al 50%.
2.2.9	**Fluidoterapia**<br>L'ottimizzazione emodinamica con fluidoterapia guidata dall'obiettivo (*goal-directed*) utilizzando dispositivi validati è raccomandata nei pazienti ad alto rischio e nei pazienti sottoposti a intervento chirurgico con grandi perdite ematiche. In tutti gli altri casi si raccomanda la fluidoterapia restrittiva in base al peso ideale in perfusione continua, soluzione bilanciata (1-3 ml/kg/h per laparoscopia; 3-5 ml/kg/h per laparotomia). La perdita di sangue dovrebbe essere compensata con colloidi 1:1.
2.2.10	**Evitare il sondino naso-gastrico**<br>Il sondino naso-gastrico NON dovrebbe essere utilizzato di routine.
2.2.11	**Prevenzione dell’ipotermia**

EUPEMEN
European Perioperative Medical Networking
Co-funded by the Erasmus+ Programme of the European Union
Cátedra GERM Medicina Perioperatoria Universidad Zaragoza

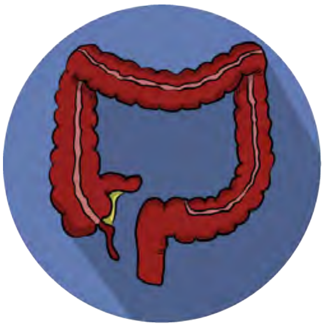

PROTOCOLLO EUPEMEN

RESEZIONE COLICA

	La temperatura dovrebbe essere monitorata e la normotermia dovrebbe essere mantenuta mediante riscaldamento attivo (liquidi riscaldati, coperta riscaldata).
2.2.12	**Prevenzione della nausea e vomito post-operatori (PONV)**<br>Somministrare una terapia antiemetica secondo il punteggio di Apfel.
2.2.13	**Analgesia epidurale**<br>L'analgesia epidurale toracica dovrebbe essere utilizzata in chirurgia a cielo aperto. In chirurgia laparoscopica NON è raccomandata di routine. I pazienti con controindicazione per l'analgesia epidurale e che sono a rischio di insufficienza renale o coagulopatia postoperatoria potrebbero trarre beneficio dal blocco del piano addominale trasversale (TAP block) bilaterale, infiltrazione delle incisioni dei trocar o altri metodi alternativi all'analgesia epidurale.
2.2.14	**Coadiuvanti analgesici per via endovenosa**<br>Gli analgesici adiuvanti consigliati sono i farmaci antinfiammatori non steroidei, la lidocaina, la ketamina, il solfato di magnesio e la dexmedetomidina.
2.2.15	**Controllo glicemico perioperatorio**<br>Per i pazienti diabetici utilizzare il protocollo ospedaliero locale per i diabetici sottoposti a intervento chirurgico. Nei pazienti a rischio di sviluppare insulino-resistenza, evitare livelli di glucosio nel sangue superiori a 180 mg/dl.
2.2.16	**Disinfezione cutanea**<br>La cute deve essere disinfettata partendo dalla parte centrale verso la periferia con clorexidina in una soluzione alcolica all'1%.
2.2.17	**Evitare drenaggi addominali**<br>I drenaggi addominali dovrebbero essere evitati il più possibile.
2.3	Postoperatorio Immediato<br>(Terapia intensiva / Unità di Terapia Semintensiva)<br><br>Anestesista, Infermiera
2.3.1	**Mantenimento della normotermia**<br>La temperatura dovrebbe essere misurata regolarmente e mantenuta.
2.3.2	**Analgesia a risparmio di oppioidi**<br>Dovrebbe essere utilizzata un'analgesia multimodale attiva o preventiva. Limitare l'uso di oppioidi. Puntare a un punteggio VAS inferiore a 3.
2.3.3	**Restrizione dei fluidi per via endovenosa**
2.3.4	**Ripresa precoce dell'alimentazione orale**<br>Inizio dell'assunzione di liquidi per via orale a partire da 6 ore dopo l'intervento chirurgico.
2.3.5	**Fisioterapia respiratoria**

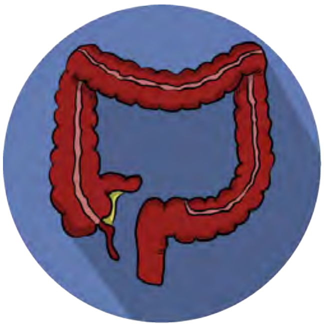

PROTOCOLLO EUPEMEN

RESEZIONE COLICA

2.3.6	**Mobilizzazione precoce**<br>La mobilizzazione dovrebbe iniziare 3 ore dopo l'intervento chirurgico sedendosi nel letto e dovrebbe deambulare 8 ore dopo l'intervento chirurgico, rispettando alle ore del sonno notturno.
2.3.7	**Profilassi tromboembolica**<br>L'Eparina a Basso Peso Molecolare dovrebbe essere somministrata 12 ore dopo l'intervento chirurgico.
2.3.8	**Profilassi della nausea e vomito postoperatori**<br>Somministrare una terapia antiemetica secondo il punteggio di Apfel.
2.3.9	**Mantenimento di FiO2 0,5% per 2 ore dopo l'intervento chirurgico**
2.3.10	**Controllo glicemico perioperatorio**<br>Per i pazienti diabetici utilizzare il protocollo ospedaliero locale per i diabetici sottoposti a intervento chirurgico. Nei pazienti a rischio di sviluppare insulino-resistenza, evitare livelli di glucosio nel sangue superiori a 180 mg/dl.
3	1ª Giornata Postoperatoria<br>(Reparto)<br><br>Chirurgo, Infermiera, Stomaterapista
3.1	**Alimentazione precoce**<br>Una dieta liquida o semi-solida dovrebbe essere iniziata come tollerata.
3.2	**Evitare le infusioni endovenose**<br>Se i pazienti tollerano liquidi per via orale, sospendere l'infusione endovenosa di fluidi.
3.3	**Mobilizzazione precoce**<br>I pazienti dovrebbero essere incoraggiati a spostarsi dal letto alla poltrona vicino al letto.
3.4	**Analgesia a risparmio di oppioidi**<br>Garantire un buon controllo del dolore. Puntare a un punteggio VAS inferiore a 3.
3.5	**Rimuovere il catetere vescicale**<br>Se è stato posizionato un catetere vescicale, valutare se può essere rimosso.
3.6	**Rimozione dei drenaggi addominali**<br>Valutare la rimozione dei drenaggi, se presenti
3.7	**Fisioterapia respiratoria**
3.8	**Profilassi tromboembolica**

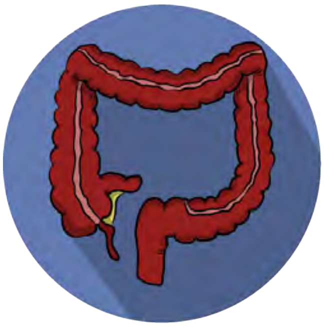

PROTOCOLLO EUPEMEN

RESEZIONE COLICA

	La profilassi tromboembolica consistente in calze elastiche o compressione intermittente ed eparina a basso peso molecolare dovrebbe essere somministrata secondo la politica ospedaliera locale.
3.9	**Prevenzione della nausea e vomito post-operatori (PONV)**<br>Somministrare una terapia antiemetica secondo il punteggio di Apfel.
3.10	**Profilassi antiulcera**
3.11	**Controllo glicemico perioperatorio**<br>Per i pazienti diabetici utilizzare il protocollo ospedaliero locale per i diabetici sottoposti a intervento chirurgico. Nei pazienti a rischio di sviluppare insulino-resistenza, evitare livelli di glucosio nel sangue superiori a 180 mg/dl.
3.12	**Educazione alla cura della stomia (se presente)**
3.13	**Esami di laboratorio**<br>Esami di laboratorio che includano anche la proteina-C reattiva.
4	2ª Giornata Postoperatoria<br>(Reparto)<br><br>Chirurgo, Infermiera, Stomaterapista
4.1	**Alimentazione precoce.**<br>Dovrebbe essere somministrata una dieta semi-solida o solida.
4.2	**Evitare le infusioni endovenose**<br>Sospendere i lquidi endovenosi se non è stato fatto in precedenza.
4.3	**Mobilizzazione precoce**<br>I pazienti dovrebbero essere in grado di camminare per brevi distanze.
4.4	**Analgesia a risparmio di oppioidi**<br>Garantire un buon controllo del dolore. Puntare a un punteggio VAS inferiore a 3. Valutare l'analgesia orale.
4.5	**Rimuovere il catetere urinario (se non già fatto in precedenza)**
4.6	**Fisioterapia respiratoria**
4.7	**Profilassi tromboembolica**
4.8	**Prevenzione di nausea e vomito post-operatori (PONV)**
4.9	**Profilassi anti-ulcera**

EUPEMEN European Perioperative Medical Networking
Co-funded by the Erasmus+ Programme of the European Union
Cátedra GERM Medicina Perioperatoria Universidad Zaragoza

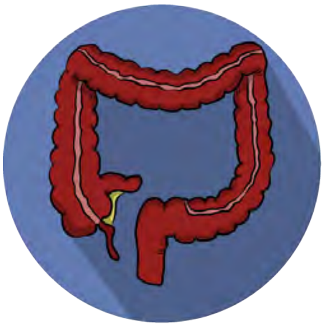

PROTOCOLLO EUPEMEN

RESEZIONE COLICA

4.10	**Controllo glicemico perioperatorio**
4.11	**Continuare con l'educazione della stomia (se presente)**
4.12	**Esami di laboratorio**<br>Esami di laboratorio che includano anche la proteina-C reattiva.
5	3ª Giornata Postoperatoria<br>(Reparto)<br><br>Chirurgo, Infermiera
5.1	**Alimentazione precoce.**<br>Dovrebbe essere fornita una dieta solida.
5.2	**Mobilizzazione precoce**<br>I pazienti devono essere completamente autonomi nella deambulazione.
5.3	**Analgesia orale**
5.4	**Rimozione degli accessi venosi**
5.5	**Fisioterapia respiratoria**
5.6	**Profilassi tromboembolica**
5.7	**Controllo glicemico peri-operatorio**
5.8	**Esami di laboratorio**<br>Esami di laboratorio che includano anche la proteina-C reattiva.
5.9	**Valutare i criteri di dimissibilità**<br>Considerare la dimissione se non ci sono complicazioni chirurgiche che non possano essere gestite in regime ambulatoriale, assenza di febbre, dolore controllato con analgesia orale, piena deambulazione, tolleranza all'assunzione orale di cibo e accettazione da parte del paziente.
6	Alla dimissione<br><br>Chirurgo, Infermiera, Cure Primarie
6.1	**Lettera di dimissione**<br>Alla dimissione, i pazienti dovrebbero ricevere informazioni personalizzate, comprensibili e complete sulla degenza ospedaliera e raccomandazioni per l'assistenza domiciliare.

EUPEMEN European Perioperative Medical Networking

Co-funded by the Erasmus+ Programme of the European Union

Cátedra GERM Medicina Perioperatoria Universidad Zaragoza

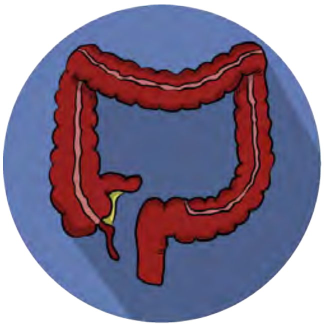

6.2	**Profilassi tromboembolica**<br>La profilassi tromboembolica deve continuare fino a 28 giorni dopo l'intervento chirurgico.
6.3	**Follow-up**<br>I pazienti devono essere seguiti nella prima settimana dopo la dimissione in regime ambulatoriale o telefonicamente. Ulteriori visite di controllo dovrebbero essere programmate per 1, 3 e 6 mesi dopo la dimissione. Dovrebbe essere organizzata una visita dal medico di base e, se necessario, dovrebbe essere coordinato il supporto domiciliare.

EUPEMEN European Perioperative Medical Networking

Co-funded by the Erasmus+ Programme of the European Union

Cátedra GERM Medicina Perioperatoria Universidad Zaragoza

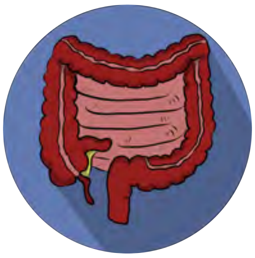

PROTOCOLLO EUPEMEN

OCCLUSIONE INTESTINALE

1	Preoperatorio<br>Anestesista, Chirurgo
1.1	**Valutazione preoperatoria di routine**<br>Esame obiettivo, ecografia addominale e analisi del sangue complete, inclusa la proteina C-reattiva.
1.2	**Sistemi di punteggio clinico**<br>Per i pazienti anziani, devono essere utilizzati punteggi di fragilità come l'indice di fragilità modificato e VIG Express. Si dovrebbero valutare i criteri Beers per prevenire il delirio negli adulti di età superiore ai 65 anni.
1.3	**Normotermia**<br>Garantire la normotermia preoperatoria nei pazienti fragili utilizzando coperte termiche.
1.4	**Evitare il posizionamento di catetere vescicale**<br>Utilizzarlo solo se necessario.
1.5	**Controllo della glicemia perioperatoria**<br>Per i pazienti diabetici utilizzare il protocollo dell'ospedale locale per diabetici da sottoporre a intervento chirurgico. Nei pazienti a rischio di sviluppare insulino-resistenza (pazienti obesi e anziani) e negli interventi chirurgici di durata superiore a 1 ora, evitare livelli di glucosio nel sangue superiori a 180 mg/dl.
1.6	**Profilassi antibiotica**<br>La profilassi antibiotica dovrebbe essere praticata in tutti i casi e la tipologia di antibiotici dovrebbe essere scelta in base alla politica dell'ospedale locale.
1.7	**Sondino nasogastrico**<br>Si raccomanda il posizionamento del sondino nasogastrico.
1.8	**Pacchetti di misure perioperatorie**<br>Sono raccomandate pacchetti di misure perioperatorie per prevenire le infezioni del sito chirurgico.
1.9	**Consenso informato**<br>Il paziente deve essere pienamente informato della procedura programmata e delle sue potenziali complicanze. I pazienti in grado di intendere e di volere devono firmare un consenso informato scritto.
2	Perioperatorio
2.1	Intraoperatorio<br>Anestesista, Chirurgo, Infermiera
2.1.1	**WHO Surgical Safety Checklist**

EUPEMEN European Perioperative Medical Networking
Co-funded by the Erasmus+ Programme of the European Union
Cátedra GERM Medicina Perioperatoria Universidad Zaragoza

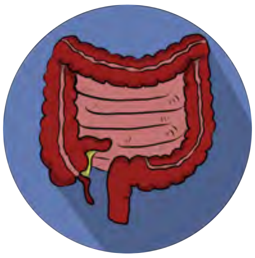

PROTOCOLLO EUPEMEN

OCCLUSIONE INTESTINALE

2.1.2	**Monitoraggio intraoperatorio di routine**
2.1.3	**Approccio chirurgico**<br>Gli approcci mininvasivi dovrebbero essere utilizzati solo in casi altamente selezionati in base all'esperienza del chirurgo. Nella maggior parte dei casi dovrebbe essere preferita la chirurgia a cielo aperto.
2.1.4	**Induzione a sequenza rapida**<br>L'induzione a sequenza rapida per l'anestesia dovrebbe essere utilizzata per ridurre l'inalazione del contenuto gastrico.
2.1.5	**Ossigenazione perioperatoria**<br>Dovrebbe essere utilizzata una frazione di ossigeno inspirato compresa tra 0,6 e 0,8.
2.1.6	**Fluidoterapia**<br>Deve essere utilizzata la fluidoterapia mirata utilizzando sistemi di monitoraggio emodinamico non invasivi. Se tali sistemi non sono disponibili, devono essere somministrate continuamente soluzioni bilanciate in base all'approccio chirurgico: 3-5 ml/kg/h per la laparoscopia e 5-7 ml/kg/h per la laparotomia.
2.1.7	**Evitare la cateterizzazione urinaria routinaria**<br>Usarla solo se necessaria.
2.1.8	**Mantenere la normotermia**<br>Utilizzare coperte termiche e fluidi riscaldati.
2.1.9	**Controllo della glicemia perioperatoria**<br>Per i pazienti diabetici utilizzare il protocollo ospedaliero locale per i diabetici sottoposti a intervento chirurgico. Nei pazienti a rischio di sviluppare insulino-resistenza (pazienti obesi e anziani) e negli interventi chirurgici di durata superiore a 1 ora, evitare livelli di glucosio nel sangue superiori a 180 mg/dl.
2.1.10	**Analgesia epidurale**<br>L'analgesia epidurale deve essere utilizzata in chirurgia a cielo aperto.
2.1.11	**Prevenzione della nausea e vomito post-operatori (PONV)**<br>Somministrare una terapia antiemetica secondo il punteggio di Apfel.
2.1.12	**Evitare drenaggi addominali**
2.1.13	**Profilassi tromboembolica**<br>La profilassi tromboembolica consistente in calze elastiche o a compressione intermittente ed eparina a basso peso molecolare deve essere somministrata secondo la politica dell'ospedale locale.
2.1.14	**Pacchetti di misure perioperatorie**<br>Sono raccomandati pacchetti di misure perioperatorie per prevenire le infezioni del sito chirurgico.

EUPEMEN European Perioperative Medical Networking
Co-funded by the Erasmus+ Programme of the European Union
Cátedra GERM Medicina Perioperatoria Universidad Zaragoza

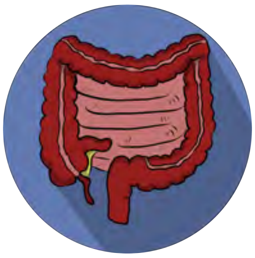

PROTOCOLLO EUPEMEN

OCCLUSIONE INTESTINALE

2.2	Postoperatorio Immediato<br><br>Anestesista, Chirurgo, Infermiera
2.2.1	**Mantenimento attivo della temperatura**<br>La temperatura corporea dovrebbe essere misurata di routine con l'obiettivo di prevenire l'ipotermia.
2.2.2	**Ossigenoterapia**<br>La saturazione di ossigeno deve essere misurata di routine per prevenire la desaturazione.
2.2.3	**Analgesia multimodale a risparmio di oppioidi**
2.2.4	**Fluidoterapia restrittiva**
2.2.5	**Controllo della glicemia perioperatoria**<br>Per i pazienti diabetici, utilizzare il protocollo dell’ospedale locale per diabetici da sottoporre a intervento chirurgico. Nei pazienti a rischio di sviluppare insulino-resistenza (pazienti obesi e anziani) e negli interventi chirurgici di durata superiore a 1 ora, evitare livelli di glucosio nel sangue superiori a 180 mg/dl.
2.2.6	**Mobilizzazione precoce**<br>I pazienti dovrebbero sedersi entro 2 ore dall'intervento chirurgico e dovrebbero iniziare a deambulare 8 ore dopo l'intervento chirurgico, rispettando le ore notturne del sonno.
2.2.7	***Nil per os* e sondino naso-gastrico**<br>Valutare la rimozione a 12 ore dall’intervento chirurgico
2.2.8	**Rimozione del catetere urinario**<br>Se è stato utilizzato un catetere urinario, valutare se rimuoverlo 12 ore dopo l'intervento chirurgico.
2.2.9	**Profilassi tromboembolica**<br>La profilassi tromboembolica consistente in calze elastiche o a compressione intermittente ed eparina a basso peso molecolare deve essere somministrata secondo la politica dell’ospedale locale.
3	1ª Giornata Postoperatoria<br>(Reparto)<br><br>Chirurgo, Infermiera
3.1	**Controllo della glicemia perioperatoria**<br>Per i pazienti diabetici, utilizzare il protocollo dell’ospedale locale per diabetici da sottoporre a intervento chirurgico. Nei pazienti a rischio di sviluppare insulino-resistenza (pazienti obesi e anziani) e negli interventi chirurgici di durata superiore a 1 ora, evitare livelli di glucosio nel sangue superiori a 180 mg/dl.
3.2	**Mobilizzazione precoce**<br>I pazienti dovrebbero deambulare pienamente.

EUPEMEN
European Perioperative Medical Networking

Co-funded by the
Erasmus+ Programme
of the European Union

Cátedra GERM
Medicina Perioperatoria
Universidad Zaragoza

3.3	**Fisioterapia respiratoria**
3.4	**Terapia antibiotica**<br>La terapia antibiotica dovrebbe essere somministrata in caso di traslocazione batterica o contaminazione della cavità addominale. Dovrebbero essere somministrati antibiotici ad ampio spettro secondo la politica ospedaliera locale.
3.5	**Analgesia a risparmio di oppioidi**
3.6	**Rimozione del sondino nasogastrico**<br>Valutare la rimozione del sondino nasogastrico.<br>Se il sondino nasogastrico viene rimosso considerare di iniziare una dieta liquida o semisolida.
3.7	**Rimozione del catetere urinario**<br>Valutare la rimozione del catetere urinario.
3.8	**Rimozione del catetere epidurale.**<br>Valutare la rimozione del catetere epidurale.
3.9	**Profilassi tromboembolica**<br>La profilassi tromboembolica consistente in calze elastiche o a compressione intermittente ed eparina a basso peso molecolare dovrebbe essere somministrata secondo la politica dell'ospedale locale.
4	2ª Giornata Postoperatoria<br>(Reparto)<br><br>Chirurgo, Infermiera
4.1	**Controllo della glicemia perioperatoria**<br>Per i pazienti diabetici, utilizzare il protocollo dell'ospedale locale per diabetici da sottoporre a intervento chirurgico. Nei pazienti a rischio di sviluppare insulino-resistenza (pazienti obesi e anziani) e negli interventi chirurgici di durata superiore a 1 ora, evitare livelli di glucosio nel sangue superiori a 180 mg/dl.
4.2	**Mobilizzazione precoce**<br>I pazienti dovrebbero deambulare pienamente.
4.3	**Fisioterapia respiratoria**
4.4	**Analgesia orale**<br>Dovrebbero essere somministrata analgesia a risparmio di oppioidi per via orale.
4.5	**Rimozione del sondino nasogastrico**<br>Valutare la rimozione del sondino nasogastrico.<br>Se il sondino nasogastrico viene rimosso considerare di iniziare una dieta liquida o semisolida.
4.6	**Profilassi tromboembolica**

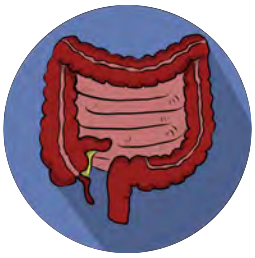

PROTOCOLLO EUPEMEN

OCCLUSIONE INTESTINALE

	La profilassi tromboembolica consistente in calze elastiche o a compressione intermittente ed eparina a basso peso molecolare dovrebbe essere somministrata secondo la politica dell'ospedale locale.
4.7	**Dimissione precoce**<br>Valutare i criteri di dimissibilità nei casi senza resezione intestinale.
5	3ª Giornata Postoperatoria<br>(Reparto)<br><br>Chirurgo, Infermiera
5.1	**Alimentazione orale precoce**
5.2	**Mobilizzazione precoce**
5.3	**Fisioterapia respiratoria**
5.4	**Tromboprofilassi**
5.5	**Valutare criteri di dimissibilità**
6	Alla dimissione<br><br>Chirurgo, Infermiera, Cure Primarie
6.1	**Tromboprofilassi**<br>Continuare tromboprofilassi personalizzata in base ai rischi.
6.2	**Terapia antibiotica**<br>Considerare di continuare la terapia antibiotica al domicilio.
6.3	**Esami di laboratorio**<br>Esami di laboratorio con un calo di almeno il 50% della proteina C-reattiva prima della dimissione.
6.4	**Follow-up**<br>Follow-up in ambulatorio o per telefono a 24 ore dalla dimissione. Invitare i pazienti a un controllo secondo la politica ospedale locale. Coordinare l'assistenza domiciliare con le cure primarie, se necessario.
6.5	**Criteri di dimissibilità**<br>Criteri generali di dimissione: nessuna complicanza non gestibile in regime ambulatoriale, ripristino delle regolari funzioni intestinali, assenza di febbre, dolore controllato con analgesia orale, accettazione da parte del paziente.

EUPEMEN
European Perioperative Medical Networking
Co-funded by the
Erasmus+ Programme
of the European Union
Cátedra GERM
Medicina Perioperatoria
Universidad Zaragoza

PROTOCOLLO EUPEMEN

APPENDICITE ACUTA

1	**Prima del ricovero**<br><br>Anestesista, Chirurgo
1.1	**Valutazione preoperatoria di routine**<br>Esame obiettivo, ecografia addominale e analisi del sangue complete, inclusa la proteina C-reattiva.
1.2	**Sistemi di punteggio clinico**<br>Gli *score* valutati dovrebbero includere il punteggio della risposta infiammatoria dell'appendicite e il punteggio dell'appendicite nell'adulto. Per i pazienti anziani, devono essere utilizzati punteggi di fragilità come l'indice di fragilità modificato e VIG Express. Si dovrebbero valutare i criteri Beers per prevenire il delirio negli adulti di età superiore ai 65 anni.
1.3	**Normotermia**<br>Garantire la normotermia preoperatoria nei pazienti fragili utilizzando coperte termiche.
1.4	**Evitare il posizionamento di catetere vescicale**<br>Utilizzarlo solo se necessario.
1.5	**Controllo della glicemia perioperatoria**<br>Per i pazienti diabetici utilizzare il protocollo dell'ospedale locale per diabetici da sottoporre a intervento chirurgico. Nei pazienti a rischio di sviluppare insulino-resistenza (pazienti obesi e anziani) e negli interventi chirurgici di durata superiore a 1 ora, evitare livelli di glucosio nel sangue superiori a 180 mg/dl.
1.6	**Profilassi antibiotica**<br>La profilassi antibiotica dovrebbe essere praticata in tutti i casi e la tipologia di antibiotici dovrebbe essere scelta in base alla politica dell'ospedale locale.
1.7	**Pacchetti di misure perioperatorie**<br>Sono raccomandate pacchetti di misure perioperatorie per prevenire le infezioni del sito chirurgico.
1.8	**Consenso informato**<br>Il paziente deve essere pienamente informato della procedura programmata e delle sue potenziali complicanze. I pazienti in grado di intendere e di volere devono firmare un consenso informato scritto.
2	**Perioperatorio**
2.1	**Intraoperatorio**<br><br>Anestesista, Chirurgo, Infermiera
2.1.1	**WHO Surgical Safety Checklist**

EUPEMEN European Perioperative Medical Networking
Co-funded by the Erasmus+ Programme of the European Union
Cátedra GERM Medicina Perioperatoria Universidad Zaragoza

PROTOCOLLO EUPEMEN

APPENDICITE ACUTA

2.1.2	**Monitoraggio intraoperatorio di routine**
2.1.3	**Approccio chirurgico**<br>Un approccio mininvasivo dovrebbe essere preferito nella maggior parte dei casi.
2.1.4	**Induzione a sequenza rapida**<br>L'induzione a sequenza rapida per l'anestesia dovrebbe essere utilizzata per ridurre l'inalazione del contenuto gastrico.
2.1.5	**Ossigenazione perioperatoria**<br>Dovrebbe essere utilizzata una frazione di ossigeno inspirato compresa tra 0,6 e 0,8.
2.1.6	**Fluidoterapia**<br>Deve essere utilizzata la fluidoterapia mirata utilizzando sistemi di monitoraggio emodinamico non invasivi. Se tali sistemi non sono disponibili, devono essere somministrate continuamente soluzioni bilanciate in base all'approccio chirurgico: 3-5 ml/kg/h per la laparoscopia e 5-7 ml/kg/h per la laparotomia.
2.1.7	**Evitare la cateterizzazione urinaria routinaria**<br>Usarla solo se necessaria.
2.1.8	**Evitare l'utilizzo di sondino naso-gastrico**<br>Usarlo solo se necessario.
2.1.9	**Mantenere la normotermia**<br>Utilizzare coperte termiche e fluidi riscaldati.
2.1.10	**Controllo della glicemia perioperatoria**<br>Per i pazienti diabetici utilizzare il protocollo dell'ospedale locale per i diabetici da sottoporre a intervento chirurgico. Nei pazienti a rischio di sviluppare insulino-resistenza (pazienti obesi e anziani) e negli interventi chirurgici di durata superiore a 1 ora, evitare livelli di glucosio nel sangue superiori a 180 mg/dl.
2.1.11	**Prevenzione della nausea e vomito post-operatori (PONV)**<br>Somministrare una terapia antiemetica secondo il punteggio di Apfel.
2.1.12	**Evitare drenaggi addominali**<br>Usarli solo se necessario.
2.1.13	**Analgesia multimodale a risparmio di oppioidi**<br>Dovrebbe essere utilizzata un'analgesia a risparmio di oppioidi che includa l'infiltrazione dei siti di accesso laparoscopici con anestetico locale o blocchi del piano per via transaddominale.
2.1.14	**Profilassi tromboembolica**<br>La profilassi tromboembolica consistente in calze elastiche o a compressione intermittente ed eparina a basso peso molecolare deve essere somministrata secondo la politica dell'ospedale locale.
2.1.15	**Pacchetti di misure perioperatorie**<br>Sono raccomandati pacchetti di misure perioperatorie per prevenire le infezioni del sito chirurgico.

EUPEMEN European Perioperative Medical Networking
Co-funded by the Erasmus+ Programme of the European Union
Cátedra GERM Medicina Perioperatoria Universidad Zaragoza

PROTOCOLLO EUPEMEN

APPENDICITE ACUTA

2.2	Immediato Postoperatorio<br><br>Anestesista, Chirurgo, Infermiera
2.2.1	**Mantenimento attivo della temperatura**<br>La temperatura corporea dovrebbe essere misurata di routine con l'obiettivo di prevenire l'ipotermia.
2.2.2	**Ossigenoterapia**<br>La saturazione di ossigeno deve essere misurata di routine per prevenire la desaturazione.
2.2.3	**Analgesia multimodale a risparmio di oppioidi**
2.2.4	**Fluidoterapia restrittiva**
2.2.5	**Controllo della glicemia perioperatoria**<br>Per i pazienti diabetici, utilizzare il protocollo dell'ospedale locale per diabetici da sottoporre a intervento chirurgico. Nei pazienti a rischio di sviluppare insulino-resistenza (pazienti obesi e anziani) e negli interventi chirurgici di durata superiore a 1 ora, evitare livelli di glucosio nel sangue superiori a 180 mg/dl.
2.2.6	**Mobilizzazione precoce**<br>I pazienti dovrebbero sedersi entro 2 ore dall'intervento chirurgico e dovrebbero iniziare a deambulare 8 ore dopo l'intervento chirurgico, rispettando le ore notturne del sonno.
2.2.7	**Alimentazione precoce**<br>I pazienti dovrebbero iniziare a bere 4 ore dopo l'intervento chirurgico.
2.2.8	**Profilassi tromboembolica**<br>La profilassi tromboembolica consistente in calze elastiche o a compressione intermittente ed eparina a basso peso molecolare deve essere somministrata secondo la politica dell'ospedale locale.
2.2.9	**Terapia antibiotica**<br>Gli antibiotici devono essere somministrati per l'appendicite acuta complicata. La scelta degli antibiotici deve essere effettuata in base alla politica dell'ospedale locale.
3	1ª Giornata Postoperatoria<br>(Reparto)<br><br>Chirurgo, Infermiera
3.1	**Alimentazione precoce**<br>Dovrebbe essere iniziata l'alimentazione con cibo semi-solido.
3.2	**Mobilizzazione precoce**<br>I pazienti dovrebbero deambulare pienamente.

EUPEMEN European Perioperative Medical Networking
Co-funded by the Erasmus+ Programme of the European Union
Cátedra GERM Medicina Perioperatoria Universidad Zaragoza

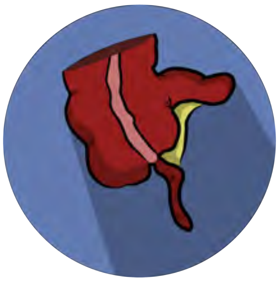

PROTOCOLLO EUPEMEN

APPENDICITE ACUTA

3.3	**Fisioterapia respiratoria**
3.4	**Analgesia orale**<br>Dovrebbero essere somministrata analgesia a risparmio di oppioidi per via orale.
3.5	**Evitare infusioni endovenose**<br>Se i pazienti tollerano i liquidi per via orale, sospendere la fluidoterapia per via endovenosa.
3.6	**Profilassi tromboembolica**<br>La profilassi tromboembolica consistente in calze elastiche o a compressione intermittente ed eparina a basso peso molecolare dovrebbe essere somministrata secondo la politica dell'ospedale locale.
4	2ª Giornata Postoperatoria<br>Chirurgo, Infermiera
4.1	**Alimentazione precoce**<br>Alimentazione con cibo semisolido/solido.
4.2	**Mobilizzazione precoce**<br>I pazienti dovrebbero deambulare pienamente.
4.3	**Analgesia orale**<br>Dovrebbero essere somministrata analgesia a risparmio di oppioidi per via orale.
4.4	**Evitare infusioni endovenose**<br>Se i pazienti tollerano i liquidi per via orale, sospendere la fluidoterapia per via endovenosa.
4.5	**Profilassi tromboembolica**<br>La profilassi tromboembolica consistente in calze elastiche o a compressione intermittente ed eparina a basso peso molecolare dovrebbe essere somministrata secondo la politica dell'ospedale locale.
4.6	**Dimissione precoce**<br>Valutare i criteri di dimissibilità.
5	Altri giorni di ricovero ospedaliero<br>Chirurgo, Infermiera
5.1	**Alimentazione orale precoce**
5.2	**Mobilizzazione precoce**

EUPEMEN European Perioperative Medical Networking

Co-funded by the Erasmus+ Programme of the European Union

Cátedra GERM Medicina Perioperatoria Universidad Zaragoza

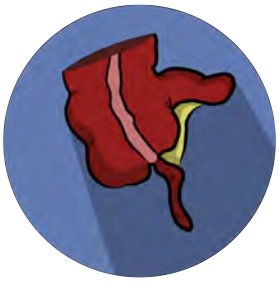

PROTOCOLLO EUPEMEN

APPENDICITE ACUTA

5.3	**Fisioterapia respiratoria**
5.4	**Analgesia orale**
5.5	**Antibioticoterapia**
5.6	**Tromboprofilassi**
5.7	**Valutare criteri di dimissibilità**
6	Alla dimissione<br><br>Chirurgo, Infermiera, Cure Primarie
6.1	**Tromboprofilassi**<br>Continuare tromboprofilassi personalizzata in base ai rischi.
6.2	**Terapia antibiotica**<br>Considerare di continuare la terapia antibiotica al domicilio.
6.3	**Esami di laboratorio**<br>Esami di laboratorio con un calo di almeno il 50% della proteina C-reattiva prima della dimissione.
6.4	**Follow-up**<br>Follow-up in ambulatorio o per telefono a 24 ore dalla dimissione. Invitare i pazienti a un controllo secondo la politica ospedale locale. Coordinare l'assistenza domiciliare con le cure primarie, se necessario.
6.5	**Criteri di dimissibilità**<br>Criteri generali di dimissione: nessuna complicanza non gestibile in regime ambulatoriale, assenza di febbre, dolore controllato con analgesia orale, accettazione da parte del paziente.

EUPEMEN
European Perioperative Medical Networking
Co-funded by the Erasmus+ Programme of the European Union
Cátedra GERM Medicina Perioperatoria
Universidad Zaragoza
