# Supplementary material for: Advancing abdominal surgery recovery implementation: a unified framework for intensified recovery protocols by the EUropean PErioperative MEdical Networking collaborative
Source: Front Surg. 2026 May 18;13:1827678. doi: 10.3389/fsurg.2026.1827678 (PMC13223102; doi:10.3389/fsurg.2026.1827678)
Supplement: Supplementary file 16 [file Datasheet7.pdf]

Εντατικοποίηση της Ανάρρωσης για τη Βέλτιστη Φροντίδα στην Χειρουργική Ενήλικων

CLINICAL PATHWAY

RICA

Recovery Intensification for optimal Care in Adult's surgery

Μετάφραση - Επιμέλεια Ελληνικής Έκδοσης
Ορέστης Ιωαννίδης

MINISTERIO DE SANIDAD

Red Española de Agencias de Evaluación de Tecnologías y Prestaciones del Sistema Nacional de Salud

IACS Instituto Aragonés de Ciencias de la Salud

Clinical Pathway

RICA

Recovery **I**ntensification for optimal **C**are in **A**dult's Surgery

(RICA)

Work Group. Recovery Intensification for optimal Care in Adult's surgery (RICA). Elaboration date: 28/12/2020
Edition: 2021

Edited by: Ministerio de Sanidad

Edited by: Instituto Aragonés de Ciencias de la Salud Edited by: Grupo Español de Rehabilitación Multimodal (GERM)

NIPO: 133-21-077-0

Layout: ARPIrelieve, S. A.

Translation funded by:

EUPEMEN European Perioperative Medical Networking

Co-funded by the Erasmus+ Programme of the European Union

Cátedra GERM Medicina Perioperatoria Universidad Zaragoza

Clinical Pathway
Recovery Intensification for optimal care in Adult's surgery (RICA)

Υπό την Αιγίδα

ΑΡΙΣΤΟΤΕΛΕΙΟ ΠΑΝΕΠΙΣΤΗΜΙΟ ΘΕΣΣΑΛΟΝΙΚΗΣ

ΣΧΟΛΗ ΕΠΙΣΤΗΜΩΝ ΥΓΕΙΑΣ
ΤΜΗΜΑ ΙΑΤΡΙΚΗΣ
Δ΄ ΧΕΙΡΟΥΡΓΙΚΗ ΚΛΙΝΙΚΗ

Διευθυντής: ΚΑΘΗΓΗΤΗΣ ΣΤ. Κ. ΑΓΓΕΛΟΠΟΥΛΟΣ

e-mail:
saggelopoulos@auth.gr, kandylak@auth.gr

ΕΛΛΗΝΙΚΗ ΔΗΜΟΚΡΑΤΙΑ

ΑΡΙΣΤΟΤΕΛΕΙΟ ΠΑΝΕΠΙΣΤΗΜΙΟ ΘΕΣΣΑΛΟΝΙΚΗΣ

ARISTOTLE UNIVERSITY OF THESSALONIKI

FACULTY OF HEALTH SCIENCE
SCHOOL OF MEDICINE
4th SURGICAL CLINIC

Head: Professor ST. K. AGGELOPOULOS

e-mail:
saggelopoulos@auth.gr, kandylak@auth.gr

ΕΛΛΗΝΙΚΗ ΧΕΙΡΟΥΡΓΙΚΗ ΕΤΑΙΡΕΙΑ
Βραβείο Ακαδημίας Αθηνών 1978

Ελληνική Εταιρεία
Χειρουργικών Λοιμώξεων

Συγγραφείς & συνεργάτες

ΣΥΝΤΟΝΙΣΤΗΣ

José Manuel Ramírez Rodríguez Grupo Español de Rehabilitación Multimodal (GERM). Departamento de Cirugía. Hospital Clínico Universitario "Lozano Blesa". Zaragoza.

ΥΠΕΥΘΥΝΟΣ ΜΕΘΟΔΟΛΟΓΙΑΣ

Pedro M. Ruiz López Grupo Español de Rehabilitación Multimodal (GERM). Hospital Universitario 12 de Octubre. Madrid

ΟΜΑΔΑ ΕΠΕΞΕΡΓΑΣΙΑΣ

Alfredo Abad Gurumeta Sociedad Española de Anestesiología y Reanimación (SEDAR). Hospital Universitario Infanta Leonor. Madrid.

Antonio Arroyo Sebastián Asociación Española de Cirujanos (AEC). Grupo de Trabajo de Rehabilitación Multimodal. Hospital Universitario Elche. Universidad Miguel Hernández. Elche. Alicante.

Marcos Bruna Esteban Asociación Española de Cirujanos. Sección de Cirugía Esofagogástrica. Hospital Universitario y Politécnico "La Fe". Valencia.

Alberto Cabañero Sanchez Sociedad Española de Cirugía Torácica(SECT). Hospital Universitario Ramón y Cajal. Madrid.

José María Calvo Vecino Grupo Español de Rehabilitación Multimodal (GERM). Hospital Universitario de Salamanca.

Emilio Del Valle Hernández Asociación Española de Coloproctología (AECP). Hospital General Universitario Gregorio Marañón. Madrid.

José Antonio García Erce Sociedad Española de Hematología y Hemoterapia (SEHH). Banco de Sangre y Tejidos de Navarra. Pamplona.

Alfredo García Fernández Grupo Español de Rehabilitación Multimodal (GERM). Grupo de trabajo ORL. Hospital Universitario 12 de Octubre. Madrid

Manuel García Toro Asociación Española de Enfermería Quirúrgica (AEEQ). Hospital Tierra de Barros. Almendralejo. Badajoz.

**Francisco Guillén Grima**	Sociedad Española de Medicina Preventiva, Salud Pública e Higiene (SEMPSPH). Clínica Universidad de Navarra.
**Ana María Godoy Ramírez**	Sociedad Española de Rehabilitación y Medicina Física (SERMEF). Hospital Regional Universitario. Málaga.
**Carmelo Loinaz Segurola**	Sociedad Española de Nutrición Clínica y Metabolismo (SENPE). Hospital Universitario 12 de Octubre. Madrid.
**Natividad Marcellán Raldúa**	Asociación Española de Enfermería Quirúrgica (AEEQ). Hospital Universitario "Miguel Servet". Zaragoza.
**Juan Antonio Margarit Calabuig**	Sociedad Española de Cirugia Cardiovascular y Endovascular (SECCE). Hospital Universitario de la Ribera. Alzira. Valencia
**Manuel Muñoz Gómez**	Sociedad Española de Transfusión Sanguínea y Terapia Celular (SETS). Departamento de Especialidades Quirúrgicas, Bioquímica e Inmunología. Facultad de Medicina. Universidad de Málaga.
**Carmen Gloria Nogueiras Quinta**	Sociedad Española de Enfermería en Cirugía (SEECir). Hospital Universitario de Fuenlabrada. Madrid.
**Julia Ocón Bretón**	Sociedad Española de Endocrinología y Nutrición (SEEN). Hospital Clínico Universitario "Lozano Blesa". Zaragoza.
**Cristina Ojeda Thies**	Grupo Español de Rehabilitación Multimodal (GERM). Grupo de trabajo en Cirugia Ortopédica y Traumatologia. Hospital Universitario 12 de Octubre. Madrid.
**Dolores Pérez del Caz**	Sociedad Española de Cirugía Plástica, Reparadora y Estética (SECPRE). Hospital Universitario "La Fe". Valencia.
**José Luis Sánchez Iglesias**	Sociedad Española de Ginecología y Obstetricia (SEGO). Hospital Universitario Vall d'Hebrón. Barcelona.
**Victor Soria Aledo**	Programa Cirugía Segura. Hospital Universitario J.M. Morales Meseguer. Murcia
**Jorge Subirá Ríos**	Asociación Española de Urología (AEU). Hospital Clínico Universitario "Lozano Blesa". Zaragoza.

ΕΞΩΤΕΡΙΚΟΙ ΚΡΙΤΕΣ

**Joel Jesús Artiles Ivonnet**	Sociedad Española de Medicos de Atencion Primaria (SEMERGEN). Centro de Salud Barrio Atlántico. Las Palmas.
**José Luis Baquero Úbeda**	Foro Español de Pacientes. Madrid.

**Pedro Javier Cañones Garzón**	Sociedad Española de Médicos Generales y de Familia (SEMG). C.S. Isla de Oza. Madrid.
**Carina Escobar Manero**	Plataforma de Organizaciones de Pacientes (POP). Madrid.
**José María Fernández Cebrián**	Sociedad Española de Patologia Digestiva (SEPD). Hospital Universitario Ramón y Cajal. Madrid.
**Elena Gómez García**	Sociedad Española de Cirugía Oral y Maxilofacial y de Cabeza y Cuello. Hospital Universitario La Paz. Madrid.
**Ana Isabel Hijas Gómez**	Sociedad Española de Epidemiología (SEE). Instituto de Salud Carlos III. Madrid
**Javier Martínez Ubieto**	Grupo Español de Rehabilitación Multimodal. Cátedra GERM. Hospital Universitario “Miguel Servet”. Zaragoza.
**Jesús Puente Alcaraz**	Federacion de Asociaciones de Enfermería Familiar y Atención Primaria (FAECAP). Hospital Universitario de Burgos.
**Manuel Quintana Díaz**	Sociedad Española de Medicina Intensiva, Crítica y Unidades Coronarias (SEMICYUC). Hospital Universitario La Paz. Madrid.
**David Rubal Bran**	Sociedad Española de Medicina Interna (SEMI). Hospital Universitario Lucus Augusti. Lugo
**Julián Carlos Segura Mata**	Sociedad Española de Cirugía Ortopédica y Traumatología. Hospital MAZ. Zaragoza
**Maria Teresa Valle Vicente**	Asociación Española de Cirugía Mayor Ambulatoria (ASECMA). Hospital General Universitario “Gregorio Marañón”. Madrid.
**Marina Varela Durán**	Sociedad Española de Calidad Asistencial(SECA). Área Sanitaria de Pontevedra e O Salnés. Pontevedra.
**Arturo Vilches Moraga**	Sociedad Española de Geriatría y Gerontología (SEGG). Sociedad Española de Medicina Geriátrica (SEMEG). Salford Royal NHS Foundation Trust. Manchester.
**Matilde Zaballos García**	Asociación Española de Cirugía Mayor Ambulatoria (ASECMA). Hospital General Universitario “Gregorio Marañón”. Madrid.

ΣΥΝΕΡΓΑΤΙΚΗ ΟΜΑΔΑ ΓΙΑ ΑΝΑΣΚΟΠΗΣΗ ΤΗΣ ΒΙΒΛΙΟΓΡΑΦΙΑΣ

**José Luis Balibrea del Castillo**	Servicio de Cirugía. Hospital Clinico de Barcelona
**Gloria Bermejo Fernández**	Servicio Medicina Física y Rehabilitación. Fundación Jiménez Díaz. Madrid.

**José Luis Bueno Cabrera**	Servicio de Hematología. Hospital Universitario Puerta de Hierro. Madrid
**Domingo Bustos García**	Servicio de Anestesiología y Reanimación. Complejo Hospitalario Universitario de Salamanca
**Rubén Casans Francés**	Servicio de Anestesiología y Reanimación. Hospital Infanta Elena, Valdemoro, Madrid
**Manuela Elía Guedea**	Servicio de Cirugía Gernal y del Aparato Digestivo. Hospital Clínico Universitario "Lozano Blesa". Zaragoza.
**Oscar Díaz Cambronero**	Servicio de Anestesiología y Reanimación. Hospital Universitario La Fe, Valencia
**Esther García González**	Serviciode ORL. H. Universitario 12 de Octubre. Madrid
**Felícitas García Ortún**	Servicio MedicinaFísica y Rehabilitación. Hospital Universitario Mútua de Terrassa. Terrassa. Barcelona.
**Ignacio Garutti Martínez**	Servicio de Anestesiología y Reanimación. Hospital Universitario "Gregorio Marañón". Madrid.
**Alba Gómez Garrido**	Servicio Medicina Física y Rehabilitación. Hospital Vall d´Hebron. Barcelona.
**Manuel Ángel Gómez Ríos**	Servicio de Anestesiología y Reanimación. Complejo Hospitalario Universitario A Coruña.
**José Luis Gónzalez Rodríguez**	Servicio de Anestesiología y Reanimación. Complejo Hospitalario Universitario de Salamanca.
**Manuel Granell Gil**	Servicio de Anestesia y Reanimación. Hospital General de Valencia.
**Carlos Jericó Alba**	Servicio de Medicina Interna. Hospital Sant Joan Despí-Moisès Broggi. Sant Joan Despí. Barcelona.
**Teresa Júlvez García**	Enfermera quirúrgica. Bloque quirúrgico. Hospital Clínico Universitario Lozano Blesa, Zaragoza.
**Javier Longas Vailén**	Servicio de Anestesiología y Reanimación. Hospital Clínico Universitario Lozano Blesa, Zaragoza.
**Clara Marín Zaldívar**	Servicio de Anestesiología y Reanimación. Hospital MAZ. Zaragoza.
**Lucrecia Mendoza González**	Servicio Medicina Física y Rehabilitación. Hospital de Cabueñes. Gijon.
**Nuria Novoa Valentín**	Servicio de Cirugía Torácica. Complejo Asistencial Universitario de Salamanca (CAUS); Instituto de Investigación Biomédica de Salamanca (IBSAL).

**Sonia Ortega Lucea**	Servicio de Anestesiología, Reanimación y Terapéutica del Dolor. Hospital Universitario Miguel Servet. Zaragoza.
**Mª Azucena Pajares Moncho**	Servicio de Anestesiología y Reanimación. Hospital Universitario y Politécnico la Fe. Valencia.
**José Antonio Páramo**	Servicio de Hematología. Clínica Universidad de Navarra.
**Ana Pascual Bellosta**	Servicio de Anestesiología, Reanimación y Terapéutica del Dolor. Hospital Universitario Miguel Servet. Zaragoza.
**Rosario Vicente Guillen**	Servicio de Anestesia y Reanimación. Hospital Universitario y Politécnico La Fe. Valencia.
**María Rodríguez Pérez**	Servicio de Cirugía Torácica. Clínica Universidad de Navarra. Madrid.
**Enrique Salmerón González**	Servicio de Cirugía Plástica y Quemados. Hospital Universitario y Politécnico La Fe. Valencia.
**Nieves Vanaclocha Saiz**	Servicio de Cirugía Plástica y Quemados. Hospital Universitario y Politécnico La Fe. Valencia.
**Guillermo Tejón Pérez**	Servicio de Anestesiología y Reanimación. Hospital Universitario Valdecilla, Santander.
**Luis Mario Vaquero Roncero**	Servicio de Anestesiología y Reanimación. Complejo Hospitalario Universitario de Salamanca.
**Peter Vorwald**	Servicio de Cirugía General y del Aparato Digestivo. Hospital Universitario Fundación Jiménez Díaz. Madrid.

ΤΕΧΝΙΚΟΣ ΣΥΝΤΟΝΙΣΜΟΣ

Concepción Sánchez Fernández. Subdirección General de Promoción, Prevención y Calidad. Dirección General de Salud Pública, Calidad e Innovación.

Yolanda Agra Varela. Subdirectora Adjunta. Area de Calidad. SG de Promocion, Prevencion y Calidad.

ΔΗΛΩΣΗ ΕΝΔΙΑΦΕΡΟΝΤΟΣ

Όλα τα μέλη της ομάδας εργασίας του Clinical Pathway έχουν κάνει τη δήλωση συμφερόντων και μπορείτε να τη συμβουλευτείτε, κατόπιν αιτήματος, μέσω της Γραμματείας του Grupo Español de Rehabilitación Multimodal – GERM (www.grupogerm.es).

Η αξιολόγηση της συμμόρφωσης με τα κριτήρια ποιότητας αυτού του εγγράφου διενεργήθηκε σε συμφωνία με το πλαίσιο συνεργασίας που

υπογράφηκε από το Ινστιτούτο Υγείας Carlos II, έναν ανεξάρτητο οργανισμό του Υπουργείου Οικονομίας και Ανταγωνιστικότητας, και το Ινστιτούτο Επιστημών Υγείας Aragonese, στο πλαίσιο της ανάπτυξης των δραστηριοτήτων του Ισπανικού Δικτύου για Οργανισμούς Αξιολόγησης Τεχνολογίας Υγείας και Παροχών του Εθνικού Συστήματος Υγείας, που χρηματοδοτούνται από το Υπουργείο Υγείας Κοινωνικών Υπηρεσιών και Ισότητας. Οι συγγραφείς του κειμένου δεν έχουν λάβει κανενός είδους οικονομική αποζημίωση για το έργο τους.

Πρόλογος

Η μετάφραση αυτή έλαβε χώρα στα πλαίσια του πανευρωπαϊκού προγράμματος EUPEMEN: European Perioperative Medical Networking (ευρωπαϊκή περιεγχειρητική ιατρική δικτύωση), το οποίο υλοποιείται στα πλαίσια του έργου Erasmus+ στον τομέα της ανώτατης εκπαίδευσης, χρηματοδοτούμενο από την Ευρωπαϊκή Ένωση υπό την αιγίδα του Ινστιτούτου Έρευνας για την Υγεία της Αραγονίας IIS Aragon. Η Δ΄ Χειρουργική Κλινική του Αριστοτελείου Πανεπιστημίου Θεσσαλονίκης, με έδρα το Γενικό Νοσοκομείο Θεσσαλονίκης “Γεώργιος Παπανικολάου”, συμμετέχει σε αυτό το πρόγραμμα που έχει σκοπό την εκπαίδευση των επαγγελματιών υγείας διαφόρων ειδικοτήτων που εμπλέκονται στην περιεγχειρητική αντιμετώπιση ασθενών και επιπρόσθετα την δημιουργία ενός δικτύου εκπαιδευτών με την ικανότητα να διδάσκουν αυτές τις ομάδες στα νοσοκομεία και να ελέγχουν τη σωστή εφαρμογή των πρωτοκόλλων με τυποποιημένο και ομοιογενή τρόπο. Μακροπρόθεσμα, ο στόχος είναι να μειωθούν οι ανεπιθύμητες ενέργειες μετά τη χειρουργική επέμβαση στους ασθενείς και, κατά συνέπεια, να επιτευχθεί ταχύτερη ανάρρωση, μείωση της θνησιμότητας μετά από χειρουργικές επεμβάσεις και μειωμένη διάρκεια νοσηλείας στο νοσοκομείο, με την επακόλουθη εξοικονόμηση για το σύστημα υγείας και την αύξηση της διαθεσιμότητας νοσηλευτικών κλινών για άλλους ασθενείς.

Με στόχο λοιπόν την ευρεία διάδοση του προγραμμάτος και την μεγαλύτερη του αποδοχή προχώρησα στην μετάφραση αυτού του εγχειριδίου για την Εντατικοποίηση της Ανάρρωσης για τη Βέλτιστη Φροντίδα στην Χειρουργική Ενήλικων, έχοντας πάντα υπόψιν ότι η περιεγχειρητική φροντίδα, περισσότερο από την ίδια την επέμβαση, καθορίζει την έκβαση.

Αναμφισβήτητα η μετάφραση ενός επιστημονικού συγγράμματος αποτελεί σταθμό στη ζωή ενός ιατρού, παρότι συχνά κρύβει δυσκολίες και απογοητεύσεις. Για την υπερπήδηση των παραπάνω εμποδίων η συμβολή του σεβαστού Καθηγητή μου, Διευθυντή της Δ΄ Χειρουργικής Κλινικής του Α.Π.Θ.

κ. Σταμάτιου Αγγελόπουλου υπήρξε καταλυτική και θα ήθελα στο σημείο αυτό να του εκφράσω τις βαθύτατες ευχαριστίες μου.

Ιδιαιτέρες ευχαριστίες εκφράζω επίσης στον φίλο και συνάδελφο χειρουργό κ. Στέφανο Μπιτσιάνη όχι μόνο για την συμμετοχή του σε αυτήν τη μετάφραση αλλά και γιατί οι συνεχείς υποδείξεις, οι πολύτιμες συμβουλές του και η ουσιαστική του βοήθεια καθόλη την διάρκεια της προσπάθειας αυτής αποτελέσαν καθοριστικό παράγοντα στην άρτια και ομαλή ολοκλήρωση της παρούσας μετάφρασης.

Ορέστης Ιωαννίδης
Επίκουρος Καθηγητής Χειρουργικής
Δ΄ Χειρουργική Κλινική Α.Π.Θ.
Γ.Ν.Θ. «Γ. Παπανικολάου»
Θεσσαλόνικη, Έλλαδα

Περιεχόμενα

	Σελίδα
**1. Συνοπτική Παρουσίαση**	13
**2. Εισαγωγή**	16
2.1. Ιστορικό	17
2.2. Αιτιολόγηση και στόχοι	18
2.3. Σε ποιον απευθύνεται;	19
**3. Κριτήρια ένταξης και αποκλεισμού**	21
**4. Μεθοδολογία**	22
**5. Βοηθητική διαδικασία**	26
**6. Συνοπτικός πίνακας συστάσεων**	29
**7. Συστάσεις και πηγές τεκμηρίωσης**	41
7.1. Γενικά	41
7.1.1. Προετοιμασία του ασθενούς (ως εξωτερικού)	41
7.1.2. Προεγχειρητικά	80
7.1.3. Διεγχειρητικά	94
7.1.4. Μετεγχειρητικά	158
7.2. Ειδικά (Κατά Ειδικότητα)	179
7.2.1. Χειρουργική Οισοφάγου	179
7.2.2. Χειρουργική Καρδιάς και Αγγείων	190
7.2.3. Χειρουργική Θώρακα	210
7.2.4. Εγκαύματα	216
7.2.5. Χειρουργική Παχέος Εντέρου	221
7.2.6. Χειρουργική Κεφαλής και Τραχήλου	223
7.2.7. Τραυματιολογία και Ορθοπαιδική Χειρουργική	229
**8. Διάγραμμα δεικτών**	246
8.1. Δείκτες Διαδικασίας	246
8.2. Δείκτες Αποτελεσμάτων	248
**9. Στρατηγική υλοποίησης**	250
**10. Παραρτήματα**	254
10.1. Προφύλαξη μετεγχειρητικής ναυτίας και εμέτου – κλίμακα Apfel	254
10.2. Προεγχειρητική αντιμετώπιση αναιμικών ασθενών	255
10.3. Αλγόριθμος διατροφικού ελέγχου	256
10.4. Προεγχειρητική Διαχείριση	257
10.5. Πληροφορίες για τον ασθενή	258
10.6. Ερωτηματολόγιο ικανοποίησης ασθενούς	264
10.7. Συντομογραφίες	270

1. Συνοπτική Παρουσίαση

Η χειρουργική ταχείας ανάρρωσης (Fast Track Surgery), γνωστή και ως βελτιστοποίηση της μετεγχειρητικής ανάρρωσης (Enhanced Recovery After Surgery - ERAS) εκτός Ισπανίας, είναι μια νέα προσέγγιση στη διαχείριση και φροντίδα των χειρουργικών ασθενών που στοχεύει να διασφαλίσει ότι οι ασθενείς φτάνουν στο χειρουργείο στις καλύτερες δυνατές συνθήκες, λαμβάνοντας την καλύτερη δυνατή αντιμετώπιση κατά τη διάρκεια της χειρουργικής επέμβασης και την συνεχεία έχουν την βέλτιστη ανάρρωση. Υπό αυτή την έννοια, τα πρωτόκολλα εντατικής ανάρρωσης (Intensified Recovery Protocols IRP) καλύπτουν ολόκληρη τη χειρουργική διαδικασία από τη διάγνωση στην επέμβαση και μέχρι την πλήρη επιστροφή στην κανονική δραστηριότητα. Όλα αυτά απαιτούν συντονισμό και ομαδική προσπάθεια όλων των εμπλεκόμενων επαγγελματιών υγείας καθώς και του ίδιου του ασθενούς.

Η αρχική ιδέα γεννήθηκε στη Δανία στις αρχές της δεκαετίας του 1990, από τον καθηγητή Henrik Kehlet. Τα πρώτα πρωτόκολλα εντατικής ανάρρωσης χρησιμοποιήθηκαν στη χειρουργική του παχέος εντέρου. Σε αυτόν τον τομέα επιτεύχθηκε η μείωση των μετεγχειρητικών επιπλοκών, η ταχύτερη ανάρρωση και η μείωση της νοσηλείας στο νοσοκομείο. Έκτοτε, πολυάριθμες μελέτες σε όλους σχεδόν τους τομείς της χειρουργικής κατέληξαν στο συμπέρασμα ότι τα πρωτόκολλα εντατικής ανάρρωσης μπορούν να εφαρμοστούν στην πλειονότητα των χειρουργικών ασθενών και να εφαρμοστούν στις περισσότερες μείζονες χειρουργικές επεμβάσεις, ανεξάρτητα από την ηλικία του ασθενούς. Στην πραγματικότητα, τα οφέλη τους έχουν αποδειχθεί ότι είναι πιο σημαντικά σε ηλικιωμένους ασθενείς λόγω των χαρακτηριστικών εφαρμογής που στοχεύουν στη μείωση του χειρουργικού τραύματος. Τα πρωτόκολλα εντατικής ανάρρωσης αντιπροσωπεύουν μια ουσιαστική αλλαγή στην κλινική πρακτική με τέτοιο τρόπο ώστε η εφαρμογή τους να μην είναι εύκολη και η διατήρηση τους, εφόσον καθιερωθεί, να απαιτεί μόνιμη προσπάθεια.

Έχοντας όλα αυτά υπόψη και χάρη στη στενή συνεργασία μεταξύ της

Ισπανικής Ομάδας Πολυπαραγοντικής Αποκατάστασης (Grupo Español de Rehabilitación Multimodal - GERM) και του ισπανικού Υπουργείου Υγείας, Κοινωνικών Υπηρεσιών και Ισότητας, αναπτύχθηκε ένα σχέδιο ιατρικής και νοσηλευτικής φροντίδας με στόχο τη μείωση της μεταβλητότητας της κλινικής πρακτικής. Το 2015 δημοσιεύθηκε το κλινικό πρωτόκολλο για την εντατική αποκατάσταση στην χειρουργική της κοιλίας (Clinical Pathway for Intensified Recovery in Abdominal Surgery – Via RICA) σε στενή συνεργασία με άλλες επιστημονικές εταιρείες, προκειμένου να προσφέρει ένα διεπιστημονικό έγγραφο συναίνεσης για τη βελτίωση της μετεγχειρητικής ανάρρωσης, τη διατήρηση της ασφάλειας των ασθενών και τη βέλτιστη χρήση των πόρων.

Με την πάροδο του χρόνου, η ανάγκη ενημέρωσης αυτού του εγγράφου με τη συμπερίληψη άλλων χειρουργικών επεμβάσεων εκτός από τη χειρουργική κοιλίας έγινε ξεκάθαρη. Ως αποτέλεσμα, προτείνεται αυτό το νέο πρωτόκολλο, ως ενημέρωση για το RICA 2015, με στόχο να παρέχει στους επαγγελματίες του τομέα της υγειονομικής περίθαλψης συστάσεις βασισμένες σε επιστημονικά δεδομένα με την συναίνεση διαφόρων επιστημονικών εταιρειών. Όλοι εμείς που συμμετέχουμε στην ανάπτυξη αυτού του νέου πρωτόκολλου, το έχουμε κάνει με βαθιά πεποίθηση ότι η χρήση και η εφαρμογή επιστημονικών δεδομένων από επαγγελματίες υγείας βελτιώνουν την κλινική αποτελεσματικότητα και τον έγκαιρο εντοπισμό επιπλοκών. Επιπλέον, με την εναρμόνιση και την ομοιογένεια των θεραπειών, που επιτυγχάνεται χάρη στα συμφωνημένα πρωτόκολλα εντατικής ανάρρωσης, διευκολύνεται η ομαδική εργασία και βελτιώνεται η αποτελεσματικότητα.

Ως εκ τούτου, ο κύριος στόχος αυτού του κειμένου είναι να παρέχει στους επαγγελματίες συστάσεις βασισμένες στην επιστημονική γνώση και στη συναίνεση των διαφόρων επιστημονικών εταιρειών που εμπλέκονται στην υλοποίηση και αξιολόγηση των πρωτόκολλων εντατικής ανάρρωσης σε μείζονες χειρουργικές επεμβάσεις σε ενήλικες. Το έγγραφο χωρίζεται σε ένα γενικό μέρος, το οποίο περιλαμβάνει μια ανασκόπηση των περιεγχειρητικών βημάτων που είναι κοινά για όλες τις επεμβάσεις και ένα ειδικό μέρος για καθεμία από τις ιδιαιτερότητες κάθε ειδικότητας που ενσωματώνεται στο πρωτόκολλο.

Για την προετοιμασία αυτού του κειμένου, έγιναν ανασκοπήσεις εκείνων των σημείων στα οποία δεν υπήρχαν κατευθυντήριες οδηγίες κλινικής

πρακτικής ή σαφή επαληθεύσιμα επιστημονικά δεδομένα.

Σύμφωνα με την ορολογία που προτείνει το GRADE, το κείμενο περιλαμβάνει τον κατάλογο των συστάσεων με βιβλιογραφικές αναφορές, καθώς και το επίπεδο της τεκμηρίωσης και τον βαθμό της σύστασης. Ομοίως, παρέχεται ένας πίνακας δεικτών για τη μέτρηση της διαδικασίας και των αποτελεσμάτων. Για τη μέτρηση της αντιληπτής ποιότητας, έχει σχεδιαστεί ένα ερωτηματολόγιο ικανοποίησης ασθενών. Τέλος, παρέχεται ένα ενημερωτικό κείμενο σχετικά με τη γενική διαδικασία υγειονομικής περίθαλψης για τον ασθενή.

2. Εισαγωγή

Μέχρι πριν από λίγα χρόνια, η περιεγχειρητική αντιμετώπιση για ασθενείς που υποβάλλονταν σε εκλεκτική κοιλιακή επέμβαση συνίστατο σε μια σειρά συνηθειών που αποκτήθηκαν από την πρακτική και όχι σε δεδομένα επιστημονικά αποδεδειγμένα. Στις αρχές του 2000, η μέση μετεγχειρητική νοσηλεία στην Ισπανία μετά από εγχείρηση παχέος εντέρου με αυτές τις οδηγίες θεραπείας ήταν 11,8 ημέρες (95% CI 11,21 έως 12,7)[1]. Μία από τις κύριες βελτιώσεις στον χειρουργικό τομέα τον τελευταίο καιρό είναι η εισαγωγή πρωτόκολλων εντατικής ανάρρωσης. Αυτά τα πρωτόκολλα βασίζονται σε τρεις βασικούς πυλώνες: την εφαρμογή ενός πακέτου περιεγχειρητικών στρατηγικών, τη διεπιστημονικότητα, που νοείται ως η κοινή προσπάθεια και η οργανωμένη συμβολή των διαφόρων εμπλεκόμενων επαγγελματιών υγείας, και την ενεργό συμμετοχή του ασθενούς σε όλη τη διαδικασία.

Λαμβάνοντας όλα αυτά υπόψιν, το 2015 δημοσιεύτηκε το πρωτόκολλο RICA (Intensified Recovery in Abdominal Surgery)[2] στον Οδηγό Υγείας (Guía Salud – OPBE). Είναι ένα έγγραφο συστάσεων κλινικής πρακτικής που ανασκοπεί ολόκληρη την περιεγχειρητική διαδικασία (προεγχειρητική, διεγχειρητική και μετεγχειρητική), και αποτελεί μια πολυπαραγοντική οδό φροντίδας σχεδιασμένη για την επίτευξη πρώιμης ανάρρωσης μετά από τη χειρουργική επέμβαση.

Το Via RICA, υποστηριζόμενο από τα καλύτερα διαθέσιμα επιστημονικά δεδομένα, διατηρεί τις θεμελιώδεις ιδέες των πρωτόκολλων εντατικής ανάρρωσης και στοχεύει να καθοδηγήσει τη λήψη αποφάσεων προς τέσσερις θεμελιώδεις αρχές ποιότητας και ασφάλειας της υγειονομικής περίθαλψης:

1. Οι ασθενείς θα πρέπει να παραμένουν ενημερωμένοι καθ' όλη τη διάρκεια της διαδικασίας και να συμμετέχουν στην πορεία λήψης αποφάσεων.
2. Η προετοιμασία και η ευεξία του ασθενούς θα πρέπει να βελτιστοποιηθούν για να διασφαλιστεί ότι βρίσκονται στην καλύτερη δυνατή κατάσταση για χειρουργική επέμβαση.
3. Ολόκληρο το περιεγχειρητικό στάδιο θα πρέπει να βασίζεται σε

προληπτικές ενέργειες, ώστε οι συστάσεις να είναι προσαρμοστικές και ενσωματωμένες σε όλη την οδό: πριν, κατά τη διάρκεια και μετά την επέμβαση.

4. Οι ασθενείς θα πρέπει να διαδραματίζουν ενεργό ρόλο και να μοιράζονται την ευθύνη για τη βελτίωση της ανάρρωσής τους.

Οι συστάσεις του RICA αντιπροσωπεύουν μια αλλαγή και η αλλαγή είναι πάντα δύσκολη. Ωστόσο, από την πρώτη δημοσίευσή του το 2015, αυτή η προθυμία για αλλαγή φάνηκε από τον έναν σημαντικό αριθμό επαγγελματιών υγείας και από την υποστήριξη αυτής της αλλαγής από το Υπουργείο Υγείας. Σε μόλις τέσσερα χρόνια, έχουν υιοθετηθεί προγράμματα και πρωτόκολλα εντατικής ανάρρωσης από έναν αξιόλογο αριθμό νοσοκομείων στην Ισπανία και ο αριθμός αυτός αυξάνεται σε καθημερινή βάση. Αυτή η τάση μετατρέπει το πρωτόκολλο RICA σε μια τυπική πρακτική.

Μετά από πέντε χρόνια από τη δημοσίευσή του, η ανάγκη για ανασκόπηση και επικαιροποίηση είναι ξεκάθαρη. Εμφανίστηκαν νέες μελέτες και τα πρωτόκολλα επεκτάθηκαν σε όλες σχεδόν τις χειρουργικές ειδικότητες με αποδεδειγμένα οφέλη. Έτσι, δεν προτάθηκε μόνο μια επικαιροποίηση για το RICA 2015, αλλά και μια επέκταση για να συμπεριλάβει αυτές τις νέες ειδικότητες. Το νέο πρωτόκολλο RICA προσφέρει συστάσεις για σχεδόν κάθε ενήλικα ασθενή που υποβάλλεται σε προγραμματισμένη χειρουργική επέμβαση.

2.1. ΙΣΤΟΡΙΚΟ

Τα πρωτόκολλα εντατικής ανάρρωσης συνδυάζουν μια σειρά στοιχείων με στόχο τη βελτιστοποίηση της ανάρρωσης και τη μείωση της ανταπόκρισης στο χειρουργικό στρες. Μετά από προκαταρκτικά ευνοϊκά αποτελέσματα, εισήχθησαν στην κλινική πράξη πριν από περίπου 15 χρόνια, με βάση επαρκή επιστημονικά δεδομένα που προέκυψαν από τυχαιοποιημένες μελέτες. Ξεκινούν στο στάδιο της διάγνωσης και στοχεύουν στην αναγνώριση των ατομικών αναγκών του ασθενούς προκειμένου να βελτιστοποιήσουν τη φροντίδα πριν, κατά τη διάρκεια και μετά τη χειρουργική επέμβαση.

Η συμμετοχή στη αντιμετώπιση της νόσου όλων όσοι εμπλέκονται σε αυτήν, συμπεριλαμβανομένου του ασθενούς και της οικογένειάς του είναι

απαραίτητη. Η υιοθέτηση μιας σειράς μέτρων που αποτελούν το πρωτόκολλο είναι ο κύριος στόχος των μελετών που έχουν πραγματοποιηθεί, επομένως, παρουσιάζεται κάποια μεταβλητότητα λόγω του γεγονότος ότι κανένα από αυτά δεν υιοθετεί όλα τα προτεινόμενα μέτρα. Ωστόσο, υπάρχει αρκετή συναίνεση για να πούμε ότι η εφαρμογή αυτών των πρωτοκόλλων είναι επωφελής για τους ασθενείς, όπως έχει αποδειχθεί στις πρόσφατες μετα-αναλύσεις. Το όφελος που λαμβάνεται από αυτά τα πρωτόκολλα σχετίζεται άμεσα με το ποσοστό συμμόρφωσης σε αυτά. Αυτό αποδείχθηκε σε πρόσφατη μελέτη του Grupo Español de Rehabilitación Multimodal (GERM)[3].

2.2. ΑΙΤΙΟΛΟΓΗΣΗ ΚΑΙ ΣΤΟΧΟΙ

Η αυξανόμενη ζήτηση για μείζονες χειρουργικές επεμβάσεις σε ασθενείς υψηλού κινδύνου απαιτεί περαιτέρω βελτιώσεις που πρέπει να περιλαμβάνουν μια βασισμένη στην τεκμηρίωση, ειδική για την επέμβαση, επικαιροποιημένη και διεπιστημονική προσέγγιση εντός των βάσεων του πρωτοκόλλου ταχείας ανάρρωσης. Η τυποποίηση αυτών των μέτρων είναι επωφελής για τους ασθενείς, τους επαγγελματίες και τα ιατρικά κέντρα. Είναι δυνατόν να πραγματοποιηθεί με τρόπο βασισμένο σε πρωτόκολλα σε κρατικό επίπεδο, όπως αποδείχθηκε από προηγούμενα προγράμματα σε άλλες χώρες με καλά αποτελέσματα.

Αυτό το έγγραφο ασχολείται με κλινικές πτυχές που σχετίζονται με την περιεγχειρητική διαχείριση του ασθενούς, σε μια προσπάθεια ομογενοποίησης της φροντίδας και βελτίωσης της μετεγχειρητικής ανάρρωσης, μειώνοντας τις χειρουργικές επιπλοκές και βελτιώνοντας αισθητά την ποιότητα ζωής όπως την αντιλαμβάνεται ο ασθενής. Για να επιτευχθεί αυτός ο στόχος, είναι απαραίτητο να αλλάξει ο συνήθης τρόπος διαχείρισης αυτών των ασθενών τόσο στο προεγχειρητικό στάδιο όσο και διεγχειρητικά και στην μετεγχειρητική ανάρρωση.

Το εύρος δράσης αυτής της προσέγγισης περιλαμβάνει όλους τους ασθενείς άνω των 18 ετών που υποβάλλονται σε μείζονα χειρουργική επέμβαση.

Τα πρωτόκολλα εντατικής ανάρρωσης πρέπει να τυποποιηθούν τουλάχιστον για τις εκλεκτικές χειρουργικές επεμβάσεις, αλλά για να επιτευχθεί

αυτό, απαιτείται στενότερη συνεργασία μεταξύ χειρουργών, αναισθησιολόγων, διατροφολόγων, νοσηλευτών κ.λπ. προκειμένου να διασφαλιστεί η συμμόρφωση με όλα τα βήματα του πρωτοκόλλου, καθώς έχει αποδειχθεί ότι αυτό αποδίδει τα καλύτερα δυνατά αποτελέσματα.

Ως εκ τούτου, ο κύριος στόχος αυτού του εγγράφου είναι να παράσχει ένα εργαλείο βασισμένο σε επιστημονικά δεδομένα και με τη συναίνεση των διαφόρων επιστημονικών εταιρειών, που μπορούν να χρησιμοποιηθεί για την τυποποίηση της διαδικασίας χειρουργικής περίθαλψης με βάση τις αρχές των πρωτόκολλων εντατικής ανάρρωσης

Όσον αφορά τους ειδικούς στόχους, το έγγραφο στοχεύει να καθορίσει τις κλινικές συστάσεις και τις ευθύνες στις ακόλουθες περιόδους:

- Προεγχειρητική βελτιστοποίηση
- Άμεση προεγχειρητικά
- Διεγχειρητικά
- Μετεγχειρητικά

Καθώς και να:

- Καθορίσει τους δείκτες που χρησιμοποιούνται για τη μέτρηση της ποιότητας της διαδικασίας υγειονομικής περίθαλψης, συμπεριλαμβανομένων των διαφόρων διαστάσεων της: ποιότητα, επιστημονικό-τεχνικό πεδίο, κλινική αποτελεσματικότητα, ποιότητα ζωής και ικανοποίηση ασθενών
- Παρέχει πλήρη γραπτή πληροφόρηση στον ασθενή
- Σχεδιάσει ένα ερωτηματολόγιο για τη μέτρηση της ικανοποίησης των ασθενών
- Προτείνει στρατηγικές για την υλοποίηση των πρωτόκολλων εντατικής ανάρρωσης για διαφορετικές χειρουργικές επεμβάσεις, συμπεριλαμβανομένων των ειδικών πτυχών και ιδιαιτεροτήτων τους.

2.3. ΣΕ ΠΟΙΟΝ ΑΠΕΥΘΥΝΕΤΑΙ;

Αυτή η επικαιροποιημένη έκδοση διατηρεί το εύρος της αρχικής και απευθύνεται όχι μόνο σε επαγγελματίες υγείας που εμπλέκονται άμεσα στη φροντίδα του χειρουργικού ασθενούς όπως χειρουργούς, αναισθησιολόγους και νοσηλευτές, αλλά και σε εκείνους τους επαγγελματίες που κατά κάποιο

τρόπο σχετίζονται με τη διεπιστημονική αντιμετώπιση αυτών των ασθενών, όπως διατροφολόγοι, ειδικοί οστομικών υλικών, φυσίατροι, φυσιοθεραπευτές, γαστρεντερολόγοι, ακτινοθεραπευτές, ογκολόγοι, παθολογοανατόμοι, γηρίατροι και παθολόγοι. Δεδομένου ότι ένα από τα πλεονεκτήματα αυτών των πρωτόκολλων εντατικής ανάρρωσης είναι η οικονομική αποδοτικότητα (μείωση της νοσηλείας στο νοσοκομείο, καθώς και η βελτιστοποίηση των πόρων), πιστεύουμε ότι αυτό το κλινικό πρωτόκολλο μπορεί επίσης να είναι χρήσιμο για τους διοικητές, τους διευθυντές των κλινικών και τους συντονιστές ποιότητας. Τέλος, και λόγω των χαρακτηριστικών των πρωτόκολλων εντατικής ανάρρωσης, στα οποία οι ασθενείς διαδραματίζουν ενεργό ρόλο, πιστεύουμε ότι είναι επίσης χρήσιμο για αυτούς. Περαιτέρω, πιστεύουμε ότι οι επαγγελματίες της πρωτοβάθμιας φροντίδας πρέπει επίσης να επωφεληθούν από αυτό και να το ενσωματώσουν στις διαδικασίες τους καθώς αποτελούν μέρος της ομάδας φροντίδας.

ΒΙΒΛΙΟΓΡΑΦΙΚΕΣ ΑΝΑΦΟΡΕΣ

1. Ruiz P, Alcalde J, Rodriguez E, Landa JI, Jaurrieta E. Proyecto nacional para la gestión clínica de procesos asistenciales. Tratamiento quirúrgico del cáncer colorrectal. I. Aspectos generales. Cir Esp. 2002;71(4):173-80.

2. Grupo de trabajo. Vía Clínica de Recuperación Intensificada en Cirugía Abdominal (RICA). Vía clínica de recuperación intensificada en cirugía abdominal (RICA) Ministerio de Sanidad, Servicios Sociales e Igualdad. Instituto Aragonés de Ciencias de la Salud. 2014 Available from: http://portal.guiasalud.es/contenidos/iframes/documentos/opbe/2015-07/ViaClinica-RICA.pdf

3. Ripollés-Melchor J, Ramirez-Rodríguez JM, Casans-Francés R et al. Association Between Use of Enhanced Recovery After Surgery Protocol and Postoperative Complications in Colorectal Surgery. The Postoperative Outcomes Within Enhanced Recovery After Surgery Protocol (POWER) Study. JAMA Surgery 2019; 154(8):725-736.

3. Κριτήρια ένταξης και αποκλεισμού

Αν και δεν υπάρχουν στοιχεία ότι και άλλοι ασθενείς δεν θα μπορούσαν επίσης να επωφεληθούν από αυτές τις κατευθυντήριες οδηγίες, τα συνιστώμενα κριτήρια για την έναρξη της διαδικασίας είναι τα ακόλουθα.

ΚΡΙΤΗΡΙΑ ΕΝΤΑΞΗΣ

Μείζονες χειρουργικές επεμβάσεις, που δεν είναι πιθανόν να πραγματοποιηθούν με ημερησία νοσηλεία και πληρούν τα ακόλουθα κριτήρια:

- Ηλικία: Πάνω από 18 ετών.
- Οποιοδήποτε ASA.
- Αποδοχή της διαδικασίας.

ΚΡΙΤΗΡΙΑ ΑΠΟΚΛΕΙΣΜΟΥ

- Επείγουσα χειρουργική επέμβαση.
- Σοβαρή γνωστική δυσλειτουργία που καθιστά αδύνατη τη συνεργασία του ασθενούς.
- Παιδιατρικός ασθενής.

4. Μεθοδολογία

Προκειμένου να ενημερωθεί αυτό το έγγραφο, δημιουργήθηκε μια κεντρική ομάδα εργασίας του πρωτοκόλλου RICA. Η διεπιστημονική αυτή ομάδα επαγγελματιών υγείας δημιουργήθηκε από τον τομέα της νοσοκομειακής περίθαλψης των ακόλουθων ειδικοτήτων: γενική χειρουργική, νοσηλευτική, ουρολογία, γυναικολογία, αναισθησιολογία, αναζωογόνηση και θεραπεία του πόνου, ενδοκρινολογία και διαιτολογία – διατροφολογία, αιματολογία και αιμοθεραπεία, προληπτική ιατρική, φυσική ιατρική και αποκατάσταση, πλαστική και επανορθωτική χειρουργική, θωρακοχειρουργική, καρδιαγγειακή χειρουργική, ωτορινολαρυγγολογία, ορθοπεδική χειρουργική και τραυματολογία.

Ομοίως, είχαμε συνεργάτες για την αναζήτηση και την αξιολόγηση συστάσεων. Ως τελικό στάδιο, το έγγραφο εξετάστηκε από μια επιλεγμένη ομάδα εμπειρογνωμόνων που έδρασαν ως εξωτερικοί κριτές. Μεταξύ αυτών των εξωτερικών κριτών εκτός από τους ασθενείς, υπήρχαν ειδικοί στη χειρουργική, την αναισθησιολογία, την πρωτοβάθμια περίθαλψη, την παθολογία, την εντατική ιατρική, τη γηριατρική, τη νοσηλευτική και την προληπτική ιατρική.

Οι επιστημονικές εταιρείες που συμμετέχουν στην ανάπτυξη του πρωτοκόλλου RICA, που εκπροσωπούνται από μέλη της ομάδας εργασίας και εξωτερικούς κριτές, είναι: το Ισπανικό Φόρουμ Ασθενών (Foro Español de Pacientes - FEP), η Πλατφόρμα Οργανώσεων Ασθενών (Plataforma de Organizaciones de Pacientes - POP), η Ισπανική Ένωση Χειρουργών (Asociación Española de Cirujanos - AEC), η Ισπανική Εταιρεία Αναισθησιολογίας, Αναζωογόνησης και Θεραπείας Πόνου (Sociedad Española de Anestesiologia, Reanimación y Terapia del Dolor - SEDAR), η Ισπανική Εταιρεία Ενδοκρινολογίας και Διατροφής (Sociedad Española de Endocrinología y Nutrición - SEEN), η Ισπανική Εταιρεία Παρεντερικής και Εντερικής Διατροφής (Sociedad Española de Nutrición Parenteral y Enteral - SENPE), η Ισπανική Ένωση Κολοπρωκτολογίας (Asociación Española de Coloproctología - AECP), η Ισπανική Ένωση Χειρουργικής Νοσηλευτικής

(Asociación Española de Enfermería Quirúrgica - AEEQ), η Ισπανική Εταιρεία Αποκατάστασης και Φυσικής Ιατρικής (Sociedad Española de Rehabilitación y Medicina Física - SEMERF), η Ισπανική Ένωση Ουρολόγων (Asociacion Española de Urología - AEU), η Ισπανική Εταιρεία Νοσηλευτικής στη Χειρουργική (Sociedad Española de Enfermeria en Cirugia - SEECIR), η Ισπανική Εταιρεία Γυναικολογίας και Μαιευτικής (Sociedad Española de Ginecología y Obstetrica - SEGO), η Ισπανική Εταιρεία Αιματολογίας και Αιμοθεραπείας (Sociedad Española de Hematología y Hemoterapia - SEHH), η Ισπανική Εταιρεία Μετάγγισης Αίματος και Κυτταροθεραπείας (Sociedad Española de Transfusión Sanguínea y Terapia Celular - SETS), η Ισπανική Εταιρεία Προληπτικής Ιατρικής, Δημόσιας Υγείας και Υγιεινής (Sociedad Española de Medicina Preventiva, Salud Pública e Higiene - SEMPSPH), η Ισπανική Εταιρεία Καρδιαγγειακής και Ενδαγγειακής Χειρουργικής (Sociedad Española de Cirugia Cardiovascular y Endovascular - SECCE), η Ισπανική Εταιρεία Πλαστικής, Επανορθωτικής και Αισθητικής Χειρουργικής (Sociedad Española de Cirugía Plástica, Reparadora y Estética - SECPRE), η Ισπανική Εταιρεία Χειρουργικής Θώρακος (Sociedad Española de Cirugía Torácica - SECT), η Ισπανική Ένωση Μείζονος Χειρουργικής Ημέρας (Asociación Española de Cirugía Mayor Ambulatoria - ASECMA), η Ομοσπονδία Συλλόγων Οικογενειακής Νοσηλευτικής και Πρωτοβάθμιας Φροντίδας (Federacion de Asociaciones de Enfermería Familiar y Atención Primaria - FAECAP), Ισπανική Επιδημιολογική Εταιρεία (Sociedad Española de Epidemiología - SEE), η Ισπανική Εταιρεία Γηριατρικής και Γεροντολογίας (Sociedad Española de Geriatría y Gerontología - SEGG), η Ισπανική Εταιρεία Γηριατρικής Ιατρικής (Sociedad Española de Medicina Geriátrica - SEMEG), η Ισπανική Εταιρεία Ιατρών Πρωτοβάθμιας Φροντίδας (Sociedad Española de Médicos De Atención Primaria - SEMERGEN), η Ισπανική Εταιρεία Εσωτερικής Παθολογίας (Sociedad Española de Medicina Interna - SEMI), η Ισπανική Εταιρεία Παθολογίας Πεπτικού (Sociedad Española de Patología Digestiva - SEPD), η Ισπανική Εταιρεία Γενικών και Οικογενειακών Ιατρών (Sociedad Española de Médicos Generales y de Familia - SEMG), η Ισπανική Εταιρεία Εντατικής Ιατρικής, Μονάδων Εντατικής Θεραπείας και Στεφανιαίων Μονάδων (Sociedad Española de Medicina Intensiva, Crítica y Unidades Coronarias - SEMICYUC), η Ισπανική Εταιρεία για την Ποιότητα της Υγείας (Sociedad

Española de Calidad Asistencial - SECA), η Ισπανική Εταιρεία Στοματογναθοπροσωπικής Χειρουργικής και Χειρουργικής Κεφαλής και Τραχήλου (Sociedad Española de Cirugia Oral y Maxilo Facial y de Cabeza y Cuello - SECOMCyC). Συμμετείχαν επίσης μέλη του προγραμμάτος Ασφαλής Χειρουργική (Cirugía Segura) του Παρατηρητήριου Χειρουργικών Λοιμώξεων (Observatorio de Infección en Cirugía - OIC).

Κάθε μία από τις συστάσεις της προηγούμενης έκδοσης του πρωτοκόλλου RICA έχει επαληθευτεί, με χρονικό ορίζοντα για τα στοιχεία από το έτος 2015. Η αναθεώρηση έγινε χωρίς αυτό το φίλτρο για τα νέα στοιχεία που ενσωματώνονται στην παρούσα έκδοση, είτε στην κεντρική ομάδα συστάσεων είτε στις συγκεκριμένες ειδικότητες, μη θεσπίζοντας στις περιπτώσεις αυτές χρονικό περιορισμό για την αναθεώρηση.

Οι ανασκοπήσεις πραγματοποιήθηκαν στο PubMed, το Embase και τη βιβλιοθήκη Cochrane. Οι μελέτες που συμμορφώνονταν με τα κριτήρια ένταξης (καθιερωμένη περίοδος και κριτήρια ένταξης ασθενών) εξετάστηκαν από εμπειρογνώμονες (experts) που ανήκουν και προτάθηκαν από τις διαφορετικές συμμετέχουσες Επιστημονικές Εταιρείες, οι οποίοι καθόρισαν, σύμφωνα με την ορολογία που προτείνει το GRADE[1], το επίπεδο τεκμηρίωσης και την ισχύ της σύστασης. Για την προετοιμασία των συστάσεων, έγινε μια περίληψη για το θέμα με τις βιβλιογραφικές αναφορές που περιλαμβάνονται, τον ορισμό της ίδιας της σύστασης και την καταγραφή του επιπέδου τεκμηρίωσης και του βαθμού σύστασης. Τα κύρια κριτήρια που εξετάστηκαν για την πρόταση των συστάσεων ήταν, κυρίως, η ποιότητα των δεδομένων, η ισορροπία των οφελών και των κινδύνων και η σκοπιμότητα. Αυτές οι συστάσεις παρουσιάστηκαν στις συνεδριάσεις της κεντρικής ομάδας εργασίας του RICA για συζήτηση και συναίνεση.

Οι βιβλιογραφικές ανασκοπήσεις πραγματοποιήθηκαν μέχρι τον Αύγουστο του 2020. Πρόθεση των συγγραφέων και των επιστημονικών εταιρειών είναι να προβούν σε περιοδική επικαιροποίηση αυτού του εγγράφου, για το οποίο θα δημιουργηθεί μια μόνιμη ομάδα αναθεώρησης.

Ο κατάλογος δεικτών έχει διατυπωθεί με βάση εκείνους που καθορίστηκαν στην προηγούμενη έκδοση του πρωτοκόλλου RICA, και έχει επικαιροποιηθεί με τη συναίνεση των συγγραφέων αυτής της έκδοσης.

Το ερωτηματολόγιο για τη μέτρηση της ικανοποίησης των ασθενών έχει επίσης ενημερωθεί, λαμβάνοντας υπόψη τη συμβολή των ασθενών που υποβάλλονται σε χειρουργικές επεμβάσεις με εντατικοποιημένη ανάρρωση, για να βελτιώσει την αναγνωσιμότητα και την κατανόησή του.

Η Κλινική Εντατικοποιημένης Ανάρρωσης για τη Χειρουργική Ενηλίκων θα έχει τα ακόλουθα έγγραφα:

1. Προσωρινό διάγραμμα με όλες τις δραστηριότητες και παρεμβάσεις που εκτελούνται στον ασθενή καθ' όλη τη διάρκεια της υγειονομικής διαδικασίας. Όλες οι ενέργειες και οι διενεργούντες επαγγελματίες πρέπει να είναι καταχωρημένες και υπογεγραμμένες.
2. Φύλλο πληροφοριών ασθενούς.
3. Οδηγίες κατά το εξιτήριο
4. Ερωτηματολόγιο Ικανοποίησης
5. Δείκτες αξιολόγησης.

Το κλινικό πρωτόκολλο αποτελεί μέρος του ιατρικού ιστορικού του ασθενούς κατά τη στιγμή της εφαρμογής του σε οποιοδήποτε ίδρυμα.

Στο τέλος του εγγράφου, ως Παραρτήματα, ομαδοποιούνται τα ακολούθα δεδομένα του κλινικού πρωτοκόλλου για τη διευκόλυνση της συμβουλευτικής και τη βελτίωση της χρησιμότητας του εγγράφου: αλγόριθμοι, συνοπτικός πίνακας συστάσεων, συντομογραφίες.

ΒΙΒΛΙΟΓΡΑΦΙΚΕΣ ΑΝΑΦΟΡΕΣ

1. Alonso P, Rotaeche R, Rigau D, Etxeberria A, Martinez L. La evaluación de la calidad dela evidencia yla graduación de la fuerza de las recomendaciones: el sistema GRADE. (Sede web) A Coruña: Fisterra.com (Actualizada 10 de Octubre de 2019). Disponible en: https://wwww.fisterra.com

5. Βοηθητική διαδικασία

ΠΕΡΙΟΔΟΣ	ΔΡΑΣΤΗΡΙΟΤΗΤΑ	ΥΠΕΥΘΥΝΟΤΗΤΑ
Πριν την εισαγωγή (ως εξωτερικός ασθενής)	Προεγχειρητική εκτίμηση.<br>Ιδιαίτερη προσοχή στον ευπαθή ασθενή.<br>Καρδιολογική εκτίμηση, εκτίμηση αναιμίας και συννοσηρότητων, εάν απαιτείται.<br>Συστάσεις: διακοπή της κατανάλωση αλκοόλ και καπνίσματος.<br>Διατροφική αξιολόγηση και τριτροπική αποκατάσταση με διατροφική βελτιστοποίηση.<br>Πληροφορίες για τον ασθενή και την οικογένειά του.	Χειρουργός + Αναισθησιολόγος + Ενδοκρινολόγος + Νοσηλευτής/τρια
Προεγχειρητικά(κατά προτίμηση χωρίς εισαγωγή)	Έναρξη θρομβοεμβολικής προφύλαξης (αν ο ασθενής εισαχθεί το προηγούμενο απόγευμα, θα γίνει κατά την εισαγωγή).<br>Λουτρό το προηγούμενο βράδυ.<br>Νηστεία πριν την εισαγωγή στην αναισθησία: 6 ώρες για στερεά και 2 ώρες για διαυγή υγρά.<br>Αποφύγετε τις βενζοδιαζεπίνες και τα οπιοειδή με μακράς διάρκειας ημίσεια ζωή σε ηλικιωμένους ασθενείς.	Αναισθησιολόγος + Νοσηλευτής/τρια + Χειρουργός
Προεγχειρητικά	**<u>Άμεσα προεγχειρητικά</u>**<br>Τοποθέτηση καλτσών συμπίεσης ή συσκευών διαλείπουσας πνευματικής συμπίεσης, ανάλογα με τον κίνδυνο θρομβοεμβολής.<br>Χορήγηση πόσιμου διαλύματος υδατανθράκων 2 ώρες πριν την παρέμβαση.<br>Προφυλακτική χορήγηση αντιβιοτικών όταν ενδείκνυται (ή στο χειρουργείο).<br>Χορήγηση 1 δόσης γλυκοκορτικοειδών.<br>Να αποφεύγεται η αποτρίχωση όσο το δυνατόν περισσότερο.	Νοσηλευτής/τρια
	**<u>Διεγχειρητικά</u>**<br>Εφαρμογή τη χειρουργικής λίστας ελέγχου<br>Προτιμάται η περιοχική αναισθησία, εάν είναι δυνατόν.<br>Τοποθέτηση επισκληρίδιου καθετήρα σε ανοιχτή μείζονα χειρουργική κοιλίας.<br>Οξυγόνωση με FiO_2 0,6-0,8.<br>Αιμοδυναμική βελτιστοποίηση μέσω στοχοκατευθυνόμενης χορήγησης υγρών<br>Παρακολούθηση και διατήρηση της γλυκόζης αίματος < 180 mg/dl.<br>Αποφυγή των οπιοειδών όσο το δυνατόν περισσότερο.<br>Σε επεμβάσεις υψηλού αιμορραγικού κινδύνου, αξιολογήστε τη χρήση του τρανεξαμικού οξέος.	Νοσηλευτής/τρια + Αναισθησιολόγος + Χειρουργός

	Εάν χρειάζεται ουροκαθετήρας, αφαιρέστε τον το συντομότερο δυνατό.<br>Αποφύγετε το ρινογαστρικό σωλήνα όσο το δυνατόν περισσότερο.<br>Ενεργή θέρμανση με θερμαινόμενη κουβέρτα και ειδική συσκευή θέρμανσης υγρών.<br>Προφύλαξη μετεγχειρητικής ναυτίας και εμέτου σύμφωνα με την κλίμακα Apfel.<br>Αποφύγετε την συστηματική τοποθέτηση παροχετεύσεων.<br>Διήθηση των σημείων εισόδου των λαπαροσκοπικών τροκάρ ή αποκλεισμός στο επίπεδο του εγκάρσιου κοιλιακού μυός (TAP) ανάλογα με την επέμβαση.<br>**<u>Άμεσα μετεγχειρητικά</u>**<br>Διατηρήστε ενεργά τη θερμοκρασία.<br>Διατηρήστε το FIO_2 στο 0,5 για 2 ώρες μετά την επέμβαση.<br>Προγραμματισμένη αναλγησία ανάλογα με την επέμβαση. Διατηρήστε τη χορήγηση οπιοειδών στο ελάχιστο.<br>Περιορισμένη χορήγηση υγρών.<br>Έναρξη από του στόματος χορήγησης διαίτας 6 ώρες μετά την επέμβαση αναλόγα με την ανοχή.<br>Έναρξη κινητοποίησης στις 8 ώρες μετά την επέμβαση.<br>Προφύλαξη από θρομβοεμβολή.	Νοσηλευτής/τρια + Αναισθησιολόγος
1η Μετεγχειρητική Ημέρα	Συμπληρώματα διατροφής σε επιλεγμένες περιπτώσεις.<br>Κανονική διατροφή ανάλογα με την ανοχή.<br>Αξιολογήστε την αφαίρεση της παροχέτευσης, εάν υπάρχει.<br>Ενεργή κινητοποίηση (κρεβάτι / καρέκλα / βάδιση).<br>Ενδοφλέβια αναλγησία. Αποφύγετε τα οπιοειδή.<br>Εάν υπάρχει καλή ανοχή στην από του στόματος χορήγηση υγρών, διακόψτε την ενδοφλέβια χορήγηση υγρών.<br>Αξιολογήστε την αφαίρεση του ουροκαθετήρα, εάν υπάρχει.<br>Αναπνευστική φυσιοθεραπεία.<br>Προφύλαξη από θρομβοεμβολή.	Νοσηλευτής/τρια + Χειρουργός
1η Μετεγχειρητική Ημέρα	Αξιολογήστε την αφαίρεση του ουροκαθετήρα, εάν υπάρχει.<br>Εξετάστε το ενδεχόμενο αφαίρεσης του επισκληρίδιου καθετήρα, εάν υπάρχει.<br>Κανονική διατροφή<br>Διακοπή ενδοφλέβιας χορήγησης υγρών.<br>Ενεργή κινητοποίηση (περιπατητικός ασθενής).	Νοσηλευτής/τρια + Χειρουργός

	Προφύλαξη από θρομβοεμβολή.<br>Αξιολογήστε την πιθανότητα εξιτηρίου	
Κατά το υπόλοιπο της νοσηλείας	Κανονική διατροφή<br>Αναλγησία από του στόματος.<br>Ενεργή κινητοποίηση (περιπατητικός ασθενής).<br>Προφύλαξη από θρομβοεμβολή.<br>Εκτίμηση του χειρουργικού τραύματος.<br>Αξιολογήστε την πιθανότητα εξιτηρίου	Νοσηλευτής/τρια + Χειρουργός
Κατά το εξιτήριο	Διατήρηση θρομβοπροφύλαξης ανάλογα με το είδος της επέμβασης.<br>Τηλεφωνικοί έλεγχοι μετά το εξιτήριο.<br>Γενικά κριτήρια εξιτηρίου: χωρίς χειρουργική επιπλοκή, χωρίς πυρετό, ο πόνος μπορεί να ελεγχθεί με από του στόματος αναλγησία, πλήρης κινητοποίηση, αποδοχή από τον ασθενή.<br>Παρακολούθηση μετά το εξιτήριο / Πρωτοβάθμια Φροντίδα.<br>Υποστήριξη στο σπίτι – Συντονισμός με Πρωτοβάθμια Φροντίδα.	Νοσηλευτής/τρια + Χειρουργός + Γιατρός Πρωτοβάθμιας Φροντίδας

6. Συνοπτικός πίνακας συστάσεων

Α/Α	Σύσταση	Επίπεδο τεκμηρίωσης	Βαθμός σύστασης
**Προετοιμασία του ασθενούς (ως εξωτερικού)**			
1	Οι ασθενείς θα πρέπει να λαμβάνουν πλήρεις προφορικές και γραπτές πληροφορίες για το τι απαιτείται για τη βελτίωση της ανάρρωσής τους μετά την επέμβαση.	Μέτριο	Ισχυρός
2	Συνιστάται η προεγχειρητική αξιολόγηση της ευπάθειας για τον εντοπισμό ασθενών με υψηλότερο περιεγχειρητικό κίνδυνο	Υψηλό	Ισχυρός
3	Οι ασθενείς με οξεία ή μη αντιρροπούμενη καρδιοπάθεια θα πρέπει να αξιολογούνται από διεπιστημονική ομάδα λόγω του αναισθησιολογικού και χειρουργικού κινδύνου.	Υψηλό	Ισχυρός
4	Η αξιολόγηση της φυσικής κατάστασης του ασθενούς με τη χρήση της ταξινόμησης της Αμερικάνικης Εταιρίας Αναισθησιολογίας ASA συνιστάται σε όλους τους ασθενείς που υποβάλλονται σε χειρουργική επέμβαση.	Υψηλό	Ισχυρός
5	Συνιστάται η διακοπή του καπνίσματος 4-8 εβδομάδες πριν από την επέμβαση για να την μείωση των σχετιζόμενων επιπλοκών.	Υψηλό	Ισχυρός
6	Η κατανάλωση αλκοόλ θα πρέπει να διακόπτεται ένα μήνα πριν από την επέμβαση.	Μέτριο	Ισχυρός
7	Συνιστάται θεραπεία τριτροπικής προαποκατάστασης για τη βελτίωση της λειτουργικής ικανότητας πριν από τη χειρουργική επέμβαση.	Μέτριο	Ισχυρός
8	Συνιστάται διατροφικός έλεγχος (screening) για όλους τους ασθενείς που υποβάλλονται σε μείζονα χειρουργική επέμβαση.	Μέτριο	Ισχυρός
9	Όταν διαπιστωθεί ότι ένας ασθενής διατρέχει κίνδυνο υποθρεψίας, θα πρέπει να διενεργείται πλήρης διατροφική αξιολόγηση (assessment), με τη δημιουργία ενός σχεδίου διατροφικής θεραπείας, την παρακολούθηση της ανοχής και της ανταπόκρισης σε αυτό το σχέδιο.	Μέτριο	Ισχυρός
10	Όλοι οι ασθενείς σε σοβαρό διατροφικό κίνδυνο ή σοβαρή υποθρεψία θα πρέπει να λαμβάνουν διατροφική θεραπεία τουλάχιστον 7–10 ημέρες πριν από την επέμβαση. Η από του στόματος / εντερική οδός θα πρέπει να προτιμάται, εάν είναι δυνατόν.	Μέτριο	Ισχυρός
11	Δεν υπάρχουν επαρκή στοιχεία που να προτείνουν την ανοσοδιατροφή έναντι της χρήσης τυπικών από του στόματος	Χαμηλό	Ασθενής

	συμπληρωμάτων αποκλειστικά κατά την προεγχειρητική περίοδο.		
12	Συνιστάται μόλις ένας ασθενής εισαχθεί στη λίστα αναμονής του χειρουργείου ή από τη στιγμή που τίθεται η χειρουργική ένδειξη, να παρακολουθείται, να μελετάται και να αντιμετωπίζεται επαρκώς η πιθανή εμφάνιση αναιμίας ή οποιουδήποτε αιματολογικού ελλείμματος.	Χαμηλό	Ασθενής
13	Συνιστάται η εφαρμογή προγραμμάτων διαχείρισης αίματος ασθενών σε όλα τα νοσοκομεία και τους χώρους υγείας. Προτείνουμε το πρόγραμμα διαχείρισης αίματος ασθενών να ενσωματωθεί με τα πρωτοκόλλα βελτιστοποίησης της μετεγχειρητικής ανάρρωσης.	Υψηλό	Ισχυρός
14	Συνιστάται να μην προγραμματίζονται για εκλεκτική χειρουργική επέμβαση με κίνδυνο αιμορραγίας οι ασθενείς με αναιμία έως ότου πραγματοποιηθεί η κατάλληλη διαγνωστική μελέτη και θεραπεία.	Υψηλό	Ισχυρός
15	Συνιστάται τουλάχιστον ένας προσδιορισμός της αιμοσφαιρίνης (Hb) σε ασθενείς που υποβάλλονται σε εκλεκτική χειρουργική επέμβαση, τουλάχιστον 28 ημέρες πριν από την επέμβαση ή την επεμβατική διαδικασία (ιδανικά μεταξύ 6-8 εβδομάδων ή από τη στιγμή της χειρουργικής ένδειξης).	Μέτριο	Ισχυρός
16	Συνιστάται σε περιπτώσεις ογκολογικής χειρουργικής επέμβασης, ολόκληρος ο διαθέσιμος χρόνος από τη διάγνωση έως τη στιγμή της επέμβασης να χρησιμοποιείται για την ανίχνευση της αναιμίας και τη διόρθωσή της ή τουλάχιστον τη βελτίωση της συγκέντρωσης της αιμοσφαιρίνης	Μέτριο	Ισχυρός
17	Συνιστάται η προεγχειρητική συγκέντρωση αιμοσφαιρίνης πριν από τη χειρουργική επέμβαση να είναι πάνω από 13 g/dl, ανεξάρτητα από το φύλο.	Μέτριο	Ισχυρός
18	Συνιστάται η ανίχνευση και θεραπεία της περιεγχειρητικής ανεπάρκειας σιδήρου.	Μέτριο	Ισχυρός
19	Συνιστάται η ανίχνευση και αντιμετώπιση της προεγχειρητικής αναιμίας, ακόμη και σε περιπτώσεις επισπευσμένων ή επειγουσών χειρουργικών επεμβάσεων.	Μέτριο	Ισχυρός
20	Η από του στόματος θεραπεία σιδήρου συνιστάται σε περιπτώσεις σιδηροπενίας ή ήπιας-μέτριας σιδηροπενικής αναιμίας εάν μένουν τουλάχιστον 6 εβδομάδες μέχρι την επέμβαση.	Χαμηλό	Ισχυρός
21	Προεγχειρητική θεραπεία με ενδοφλέβιο σίδηρο συνιστάται σε ασθενείς με εκλεκτική χειρουργική επέμβαση με πιθανή αιμορραγία που πάσχουν από σιδηροπενική αναιμία ή/και λειτουργική ανεπάρκεια σιδήρου, για βελτίωση των επιπέδων αιμοσφαιρίνης ή/και μείωση του ρυθμού μετάγγισης.	Μέτριο	Ισχυρός
22	Συνιστούμε την ενδοφλέβια χορήγηση σιδήρου, αντί σιδήρου από του στόματος, σε εκείνες τις περιπτώσεις που αυτό αντενδείκνυται ή που ο	Μέτριο	Ισχυρός

	διαθέσιμος χρόνος μέχρι το χειρουργείο είναι ανεπαρκής		
23	Για τη μείωση της αλλογενούς μετάγγισης αίματος, η χορήγηση ανασυνδυασμένης ανθρώπινης ερυθροποιητίνης (rHuEPO) συνιστάται σε ασθενείς με εκλεκτική ορθοπεδική χειρουργική επέμβαση με κίνδυνο μέτριας-υψηλής αιμορραγίας και μέτριας μη σιδηροπενικής αναιμίας (Hb μεταξύ 10 και 13 g/dl).	Υψηλό	Ισχυρός
24	Η χορήγηση rHuEPO προτείνεται για τη μείωση του ποσοστού μετάγγισης σε αναιμικούς ασθενείς που υποβάλλονται και σε άλλη μείζονα εκλεκτική χειρουργική επέμβαση πέρα από την εκλεκτική ορθοπεδική χειρουργική επέμβαση με μέτριο-υψηλό κίνδυνο αιμορραγίας.	Μέτριο	Ασθενής
25	Η χρήση θρομβοπροφύλαξης συνιστάται σε όλους τους ασθενείς που υποβάλλονται σε μείζονα χειρουργική επέμβαση ή νοσηλεύονται για οξεία ιατρική κατάσταση.	Μέτριο	Ισχυρός
26	Γενικά, συνιστάται η διατήρηση της αντιθρομβωτικής προφύλαξης για τουλάχιστον 7 ημέρες ή έως ότου ο ασθενής είναι περιπατητικός.	Υψηλό	Ισχυρός
27	Σε περίπτωση μείζονος χειρουργικής επέμβασης στην κοιλία, η προφύλαξη θα παραταθεί έως και 4 εβδομάδες μετά την επέμβαση.<br><br>Συγκεκριμένες καταστάσεις:<br>• Ουρολογικές, γυναικολογικές, νευροχειρουργικές επεμβάσεις και επεμβάσεις γενικής χειρουργικής: 8 ημέρες. Εάν ο ασθενής είναι ακινητοποιημένος, θα πρέπει να παραταθεί μέχρι την κινητοποίησή του.<br>• Ουρολογικές, γυναικολογικές και επεμβάσεις γενικής χειρουργικής σε καρκινοπαθείς: 4 εβδομάδες (28 ημέρες)<br>• Σε χειρουργική επέμβαση ισχίου: 4–6 εβδομάδες (28–42 ημέρες)<br>• Σε χειρουργική επέμβαση γόνατος: 3–4 εβδομάδες (21–28 ημέρες)	Μέτριο	Ισχυρός
28	Συνιστάται η πρώιμη κινητοποίηση και η χρήση ελαστικών καλτσών συμπίεσης για τη διάρκεια της περιόδου ακινητοποίησης.	Υψηλό	Ισχυρός
29	Οι κάλτσες συμπίεσης είναι αποτελεσματικές στην πρόληψη της θρομβοεμβολικής νόσου σε χειρουργικούς ασθενείς, μειώνοντας ακόμη περισσότερο τον κίνδυνο όταν συνδυάζονται με φαρμακολογικούς παράγοντες.	Υψηλό	Ισχυρός
30	Οι συσκευές διαλείπουσας πνευματικής συμπίεσης μειώνουν τη συχνότητα της εν τω βάθει φλεβικής θρόμβωσης. Η μέθοδος σε συνδυασμό με φαρμακολογικά μέτρα συνιστάται κυρίως για νευροχειρουργικούς ασθενείς ή/και χειρουργεία με υψηλό κίνδυνο ΦΘΕ.	Μέτριο	Ισχυρός
31	Τα προληπτικά σχήματα περιλαμβάνουν τα νέα από του στόματος άμεσης δράσης αντιπηκτικά (dabigatran - δαβιγατράνη, apixaban -	Υψηλό	Ισχυρός

	απιξαμπάνη, rivaroxaban- ριβαροξαβάνη) ή ΗΧΜΒ (enoxaparin - ενοξαπαρίνη, bemiparin - μπεμιπαρίνη, tinzaparin - τινζαπαρίνη).		
32	Συνιστάται πλήρες λουτρό πριν από την επέμβαση.	Μέτριο	Ισχυρός
33	Στους περισσότερους ασθενείς που πρόκειται να υποβληθούν σε εκλεκτική χειρουργική επέμβαση, η στερεά τροφή θα πρέπει να επιτρέπεται έως και 6 ώρες πριν από την αναισθησία και τα διαυγή υγρά έως και 2 ώρες πριν από την αναισθησία.	Υψηλό	Ισχυρός
34	Σε εκείνους τους ασθενείς με καθυστερημένη γαστρική κένωση και επείγουσα χειρουργική επέμβαση, συνιστάται η νηστεία από τα μεσάνυχτα ή 6–8 ώρες πριν από την επέμβαση.	Μέτριο	Ισχυρός
35	Η από του στόματος λήψη ροφημάτων πλούσιων σε υδατάνθρακες έως και 2 ώρες πριν από την επέμβαση είναι ασφαλής και δεν σχετίζεται με αυξημένο κίνδυνο εισρόφησης.	Μέτριο	Ισχυρός
36	Η από του στόματος χορήγηση 200–400 ml ροφήματος που περιέχει 50 g υδατανθράκων θα πρέπει να επιτρέπεται έως και δύο ώρες πριν από την επέμβαση, καθώς αυτή η θεραπεία βελτιώνει το αίσθημα ευεξίας του ασθενούς και μπορεί να μειώσει την παραμονή στο νοσοκομείο και την αντίσταση στην ινσουλίνη.	Μέτριο	Ισχυρός
37	Σε παχύσαρκους ή/και διαβητικούς τύπου 2 ασθενείς με καλό γλυκαιμικό έλεγχο χωρίς σχετιζόμενες χρόνιες επιπλοκές, θα μπορούσε να εξεταστεί η χρήση ροφημάτων πλούσιων σε υδατάνθρακες 3 ώρες πριν από την επέμβαση. Αυτά μπορούν να χορηγηθούν μαζί με τα συνήθη αντιδιαβητικά φάρμακα του ασθενούς.	Χαμηλό	Ασθενής
38	Συνιστάται να αποφεύγεται η χρήση μακράς διάρκειας ημίσειας ζωής βενζοδιαζεπίνων και οπιοειδών πριν από την εισαγωγή στην αναισθησία σε ασθενείς υψηλού κινδύνου λόγω ηλικίας και συννοσηρότητων.	Χαμηλό	Ισχυρός
**ΠΡΟΕΓΧΕΙΡΗΤΙΚΑ**			
39	Η αντιβιοτική χημειοπροφύλαξη συνιστάται εάν οι πιθανότητες λοίμωξης είναι υψηλές ή εάν οι συνέπειες μιας μετεγχειρητικής λοίμωξης είναι δυνητικά σοβαρές για τον ασθενή (ενδοκαρδίτιδα, ενδοφθαλμίτιδα, λοίμωξη προσθετικού υλικού).	Μέτριο	Ισχυρός
40	Σε καθαρές χειρουργικές επεμβάσεις με παράγοντες κινδύνου για εμφάνιση λοίμωξης, συνιστάται η χρήση αντιβιοτικών που καλύπτουν μικροοργανισμούς του δερματικού μικροβιώματος (Staphylococcus aureus και αρνητικός στην κοαγκουλάση σταφυλόκοκκος) και, σε καθαρές - επιμολυσμένες χειρουργικές επεμβάσεις, συνιστάται η χρήση αντιβιοτικών που καλύπτουν επίσης gram-αρνητικούς βακίλους και εντερόκοκκους καθώς και αναερόβια.	Μέτριο	Ισχυρός
41	Συνιστάται η χορήγηση αντιβιοτικής χημειοπροφύλαξης κατά τη διάρκεια των 120 λεπτών πριν από τη χειρουργική τομή.	Υψηλό	Ισχυρός

42	Συνιστάται η χρήση της ίδιας δόσης για αντιβιοτική χημειοπροφύλαξη με αυτή που χρησιμοποιείται για τη θεραπεία της λοίμωξης, αν και σε παχύσαρκους ασθενείς θα πρέπει να χρησιμοποιείται το προσαρμοσμένο βάρος για τον υπολογισμό της δόσης.	Μέτριο	Ισχυρός
43	Επιπρόσθετη δόση συνιστάται σε περιπτώσεις παρατεταμένων χειρουργικών επεμβάσεων ή εάν υπάρχει σημαντική απώλεια αίματος.	Μέτριο	Ασθενής
44	Συνιστάται να μην παρατείνεται η διάρκεια της αντιβιοτικής χημειοπροφύλαξης πέρα από τη διάρκεια της ίδιας της χειρουργικής επέμβασης.	Υψηλό	Ισχυρός
45	Η χορήγηση μιας εφάπαξ δόσης γλυκοκορτικοειδών συνιστάται γιατί έχει σημαντική επίδραση στη διάρκεια της νοσηλείας στο νοσοκομείο χωρίς να αυξάνει το ποσοστό των επιπλοκών.	Μέτριο	Ισχυρός
46	Η γλυκόζη αίματος θα πρέπει να παρακολουθείται προεγχειρητικά καθώς η διεγχειρητική υπεργλυκαιμία μπορεί να οδηγήσει σε αυξημένες μετεγχειρητικές επιπλοκές, αν και η χρήση εντατικής θεραπείας με ινσουλίνη θα πρέπει να αποφεύγεται λόγω του κινδύνου υπογλυκαιμίας.	Υψηλό	Ισχυρός
47	Η περιεγχειρητική γλυκόζη αίματος θα πρέπει να παρακολουθείται και να αντιμετωπίζεται επαρκώς με ινσουλίνη, αποφεύγοντας τα επίπεδα γλυκόζης στο αίμα > 180 mg/dl.	Μέτριο	Ισχυρός
48	Πιο φιλόδοξοι στόχοι για περιεγχειρητική γλυκόζη αίματος μεταξύ 110 και 140 mg/dl (6,1-7,8 mmol/l) μπορεί να είναι κατάλληλοι σε επιλεγμένους ασθενείς, εάν μπορούν να επιτευχθούν χωρίς σημαντική υπογλυκαιμία.	Χαμηλό	Ασθενής
49	Οι τρίχες δεν πρέπει να αφαιρούνται προεγχειρητικά εκτός εάν είναι απολύτως απαραίτητο. Το συμβατικό ξύρισμα πρέπει να αποφεύγεται, τόσο προεγχειρητικά όσο και στο χειρουργείο.	Χαμηλό	Ισχυρός
50	Σε περίπτωση αφαίρεσης των τριχών, οι ηλεκτρικές ξυριστικές μηχανές μπορούν να χρησιμοποιηθούν όσο το δυνατόν πιο κοντά χρονικά στην επέμβαση αλλά πάντα εκτός χειρουργείου.	Μέτριο	Ισχυρός
**ΔΙΕΓΧΙΕΡΗΤΙΚΑ**			
51	Η χρήση της χειρουργικής λίστας ελέγχου συνιστάται για την πρόληψη των ανεπιθύμητων ενεργειών και της θνησιμότητας που σχετίζονται με την επέμβαση.	Μέτριο	Ισχυρός
52	Η χρήση αλκοολικής χλωρεξιδίνης 2% συνιστάται ως αντισηπτικό για άθικτο δέρμα στο χειρουργικό πεδίο.	Υψηλό	Ισχυρός
53	Συνιστάται η ελαχιστοποίηση της χρήσης βενζοδιαζεπίνων πριν από την εισαγωγή στην αναισθησία και η χρήση υπνωτικών παραγόντων με ελάχιστη υπολειμματική δράση, που επιτρέπουν την ταχεία ανάρρωση μετά την αναισθησία.	Χαμηλό	Ισχυρός

54	Κατά τη γενική αναισθησία, συνιστάται η χρήση προστατευτικού αερισμού, συμπεριλαμβανομένου αναπνεόμενου όγκου 6–8 ml/kg ιδανικού βάρους, η χρήση εξατομικευμένης θετικής τελοεκπνευστικής πίεσης PEEP γενικά άνω των 5 cm H2O και η εφαρμογή χειρισμών στρατολόγησης των κυψελίδων.	Μέτριο	Ισχυρός
55	Σε χειρουργικές επεμβάσεις που απαιτούν αερισμό με έναν πνεύμονα, συνιστούμε τα παραπάνω προστατευτικά μέτρα αερισμού, αλλά μείωση του αναπνεόμενου όγκου στα 4–6 ml/kg ιδανικού βάρους.	Μέτριο	Ισχυρός
56	Η παρακολούθηση του CO_2 με καπνογραφία θα πρέπει να είναι υποχρεωτική σε όλες τις χειρουργικές επεμβάσεις, ιδιαίτερα στη λαπαροσκοπική χειρουργική.	Υψηλό	Ισχυρός
57	Η παρακολούθηση της θερμοκρασίας πρέπει να είναι κεντρική.	Υψηλό	Ισχυρός
58	Το βάθος της αναισθησίας θα πρέπει να παρακολουθείται χρησιμοποιώντας τον διφασματικό δείκτη (BIS).	Υψηλό	Ισχυρός
59	Η χρήση της παρακολούθησης της αλγαισθησίας θα μπορούσε να μειώσει την διεγχειρητική κατανάλωση οπιοειδών σε σύγκριση με την τυπική παρακολούθηση.	Μέτριο	Ασθενής
60	Όταν τοποθετηθεί καθετήρας ουροδόχου κύστης, θα γίνει με τα κατάλληλα άσηπτα μέτρα και, αν είναι δυνατόν, θα αφαιρεθεί 24 ώρες μετά την επέμβαση.	Μέτριο	Ασθενής
61	Η αφαίρεση του ουροκαθετήρα συνιστάται σε 24 ώρες, εκτός από ασθενείς με μέτριο κίνδυνο οξείας επίσχεσης ούρων – άνδρες, επισκληρίδιος αναισθησία και χειρουργική επέμβαση πυέλου – οπότε συνιστάται η διατήρησή του για 3 ημέρες.	Υψηλό	Ισχυρός
62	Η επεμβατική αιμοδυναμική παρακολούθηση δεν ενδείκνυται συνήθως και ο αρτηριακός καθετηριασμός είναι χρήσιμος σε εκείνους τους ασθενείς που παρουσιάζουν σοβαρές καρδιοαναπνευστικές αλλοιώσεις και που μπορεί να παρουσιάσουν μετεγχειρητικά προβλήματα.	Χαμηλό	Ισχυρός
63	Η εισαγωγή κεντρικού φλεβικού καθετήρα (ΚΦΚ) δεν ενδείκνυται τακτικά και περιορίζεται σε ασθενείς με σοβαρές καρδιοαναπνευστικές παθήσεις με πνευμονική υπέρταση ή στους οποίους αναμένεται ότι μπορεί να απαιτήσουν τη χορήγηση αγγειοσυσπαστικών ή ινότροπων φαρμάκων σε συνεχή έγχυση.	Χαμηλό	Ισχυρός
64	Η χρήση ποσοτικής παρακολούθησης του νευρομυϊκού αποκλεισμού είναι απαραίτητη κάθε φορά που χρησιμοποιούνται φάρμακα νευρομυϊκού αποκλεισμού καθ' όλη τη διάρκεια της χειρουργικής επέμβασης.	Υψηλό	Ισχυρός
65	Η χρήση βαθέος νευρομυϊκού αποκλεισμού (PTC 1-2) συνιστάται για τη βελτίωση της απεικόνισης του χειρουργικού πεδίου, τόσο στην ανοιχτή όσο και στη λαπαροσκοπική χειρουργική, και για τη χρήση των χαμηλότερων δυνατών ενδοκοιλιακών πιέσεων στη λαπαροσκόπηση, ευνοώντας την μετεγχειρητική ανάρρωση.	Υψηλό	Ισχυρός

66	Συνιστάται να ελέγχεται η αναστροφή του νευρομυϊκού αποκλεισμού έως ότου ληφθεί λόγος τρένου των τεσσάρων (TOFr) μεγαλύτερος ή ίσος με 0,9 στον προσαγωγό μυ του αντίχειρα κατά τη διάρκεια της αναισθησίας και πριν από την αποδιασωλήνωση για να αποφευχθεί ο υπολειπόμενος νευρομυϊκός αποκλεισμός και να μειωθούν οι αναπνευστικές επιπλοκές.	Υψηλό	Ισχυρός
67	Συνιστάται η αναστροφή του νευρομυϊκού αποκλεισμού με σουγκαμαντέξ αντί για νεοστιγμίνη όταν έχει χρησιμοποιηθεί βρωμιούχο ροκουρόνιο καθώς το πρώτο είναι ταχύτερο και ασφαλέστερο.	Υψηλό	Ισχυρός
68	Συνιστάται η πρόληψη και η αποφυγή της ακούσιας περιεγχειρητικής υποθερμίας.	Υψηλό	Ισχυρός
69	Η θερμοκρασία των ασθενών θα πρέπει να ελέγχεται ώστε να διασφαλίζεται η νορμοθερμία στην περιεγχειρητική περίοδο.	Υψηλό	Ισχυρός
70	Οι ενεργητικές στρατηγικές προθέρμανσης πρέπει να ξεκινούν πριν από τη χειρουργική επέμβαση.	Υψηλό	Ισχυρός
71	Η θερμοκρασία περιβάλλοντος στο χειρουργείο πρέπει να είναι τουλάχιστον 21 °C για ενήλικες ασθενείς.	Υψηλό	Ισχυρός
72	Κατά την περιεγχειρητική περίοδο, η μεγαλύτερη δυνατή επιφάνεια του σώματος θα πρέπει να είναι θερμομονωμένη.	Υψηλό	Ισχυρός
73	Τα ενδοφλέβια υγρά, τα υγρά χειρουργικών πλύσεων και τα παράγωγα αίματος για μεταγγίσεις που χορηγούνται σε δόσεις > 500 ml/ώρα πρέπει πρώτα να έχουν θερμανθεί.	Υψηλό	Ισχυρός
74	Τα διεγχειρητικά μέτρα ενεργητικής θέρμανσης με τη μεταφορά θερμότητας μέσω αγωγής ή συναγωγής ενδείκνυνται για τη διατήρηση της νορμοθερμίας.	Υψηλό	Ισχυρός
75	Η ανάνηψη από την γενική αναισθησία πρέπει να γίνεται σε φυσιολογική θερμοκρασία σώματος.	Υψηλό	Ισχυρός
76	Η χρήση επαρκούς παρακολούθησης (όγκος παλμού – SV ή διακύμανση του όγκου παλμού – SVV) συνιστάται για την καθοδήγηση της διεγχειρητικής χορήγησης υγρών σε ασθενείς σε κίνδυνο.	Υψηλό	Ισχυρός
77	Σε περιπτώσεις όπου υπάρχει πτώση του όγκου παλμού > 10% ή διακύμανση του όγκου παλμού > 10%, ενδείκνυται η αναζωογόνηση με υγρά (δεν υπάρχει προτίμηση μεταξύ κολλοειδών ή κρυσταλλοειδών).	Υψηλό	Ισχυρός
78	Συνιστάται μια μέτρια συνεχής χορήγηση υγρών, που αποδίδει στο τέλος της επέμβασης θετικό ισοζύγιο 1 έως 2 λίτρα για την αποφυγή μετεγχειρητικής οξείας νεφρικής βλάβης.	Υψηλό	Ισχυρός
79	Σε ασθενείς υψηλού κινδύνου, συνιστάται η διατήρηση εξατομικευμένης χορήγησης υγρών με μέτρια θετικό ισοζύγιο και συνεχή παρακολούθηση του όγκου παλμού και της διακύμανσης του όγκου παλμού.	Μέτριο	Ισχυρός
80	Η διεγχειρητική υπόταση χωρίς απόκριση στην παθητική ανύψωση των ποδιών θα πρέπει να αντιμετωπίζεται με αγγειοσυσπαστικά (έλεγχος	Μέτριο	Ισχυρός

	για διακυμάνσεις της αρτηριακής πίεσης, του όγκου παλμού και της διακύμανσης του όγκου παλμού).		
81	Θα πρέπει να επιτευχθεί ένα εύρος μέσης αρτηριακής πίεσης μεγαλύτερο ή ίσο με 65 mmHg.	Υψηλό	Ισχυρός
82	Θα πρέπει να διατηρείται ο καρδιακός δείκτης > 2,5 l/λεπτό/m^2, χρησιμοποιώντας ινότροπα σε περιπτώσεις μη απόκρισης στον όγκο.	Υψηλό	Ισχυρός
83	Προτιμάται η παρακολούθηση με Doppler οισοφάγου ή με μεθόδους που βασίζονται σε πιστοποιημένη ανάλυση περιγράμματος σφυγμού.	Υψηλό	Ισχυρός
84	Το πρωτογενές ενδοφλέβιο υγρό διατήρησης πρέπει να είναι ένα ισορροπημένο ισοτονικό κρυσταλλοειδές διάλυμα.	Υψηλό	Ισχυρός
85	Για τη χορήγηση υγρών κατά την αναζωογόνηση, συνιστάται η χρήση ισορροπημένων κρυσταλλοειδών. 2–3 λίτρα για την αρχική ανάνηψη σε υποογκαιμικό σοκ και αιμοδυναμική παρακολούθηση για καθοδήγηση της πρόσθετης χορήγησης υγρών.	Μέτριο	Ισχυρός
86	Συνιστάται να χορηγείται τρανεξαμικό οξύ σε όλους τους ενήλικες που υποβάλλονται σε χειρουργική επέμβαση και οι οποίοι αναμένεται να έχουν μέτρια έως σοβαρή απώλεια αίματος.	Υψηλό	Ισχυρός
87	Η συμπληρωματική χρήση εισπνεόμενου οξυγόνου δεν συνιστάται σε ασθενείς που υποβάλλονται σε γενική αναισθησία.	Μέτριο	Ασθενής
88	Συνιστάται ελάχιστα επεμβατική χειρουργική, με την προϋπόθεση ότι τα χειρουργικά και ογκολογικά αποτελέσματα δεν διαφέρουν μεταξύ των χειρουργικών τεχνικών.	Υψηλό	Ισχυρός
89	Η εγκάρσια τομή συνιστάται στη ανοιχτή χειρουργική με λαπαροτομία.	Μέτριο	Ισχυρός
90	Συνιστάται η αποφυγή χρήσης παροχετεύσεων σε συστηματική βάση.	Υψηλό	Ισχυρός
91	Δεν συνιστάται η συστηματική χρήση του ρινογαστρικού σωλήνα.	Υψηλό	Ισχυρός
92	Η επισκληρίδιος αναλγησία εντός ενός πρωτοκόλλου συνδυασμένης αναισθησίας θα πρέπει να πραγματοποιείται σε όλους τους ασθενείς που υποβάλλονται σε μείζονα ανοιχτή χειρουργική επέμβαση στην κοιλία.	Υψηλό	Ισχυρός
93	Η τοποθέτηση επισκληρίδιου καθετήρα για έγχυση τοπικών αναισθητικών για αναλγησία σε ανοιχτή μείζονα κοιλιακή χειρουργική επέμβαση πρέπει να πραγματοποιείται στο θωρακικό επίπεδο.	Υψηλό	Ισχυρός
94	Μικρές δόσεις οπιοειδών θα πρέπει να προστίθενται στις δόσεις του τοπικού αναισθητικού που πρόκειται να χορηγηθούν επισκληρίδια σε μείζονες ανοιχτές χειρουργικές επεμβάσεις.	Μέτριο	Ισχυρός
95	Όταν η τοποθέτηση επισκληρίδιου καθετήρα δεν είναι δυνατή σε ανοιχτή μείζονα χειρουργική επέμβαση, η αναλγητική στρατηγική θα πρέπει να εξατομικεύεται, μειώνοντας τη χρήση οπιοειδών και ευνοώντας τη χρήση	Μέτριο	Ισχυρός

	τοποπεριοχικών αποκλεισμών, σπονδυλικής αναλγησίας ή διήθησης των σημείων εισόδου των τροκάρ με τοπικά αναισθητικά, ιδίως λαμβάνοντας υπόψη τον αποκλεισμό στο επίπεδο του εγκάρσιου κοιλιακού μυός.		
96	Η εκτέλεση αμφοτερόπλευρου αποκλεισμού στο επίπεδο του εγκάρσιου κοιλιακού μυός με τοπικά αναισθητικά θα μπορούσε να ωφελήσει εκείνους τους ασθενείς που χρειάζονται ανοιχτή μείζονα χειρουργική επέμβαση στην κοιλιά και που δεν μπορούν να ωφεληθούν από την επισκληρίδιο αναλγησία.	Μέτριο	Ισχυρός
97	Η αναισθησία χωρίς οπιοειδή σε πρωτόκολλα βελτιστοποίησης της μετεγχειρητικής ανάρρωσης μπορεί να είναι μια εναλλακτική λύση στη χρήση ενδοφλεβίων οπιοειδών.	Μέτριο	Ασθενής
98	Η χρήση ενδοφλέβιας λιδοκαΐνης διεγχειρητικά συνιστάται ως συμπληρωματική φαρμακευτική αγωγή για τη μείωση του μετεγχειρητικού πόνου και για τη βελτίωση της αποκατάστασης της εντερικής λειτουργίας στην άμεση μετεγχειρητική περίοδο, αποτελώντας εναλλακτική στην ενδοφλέβια χορήγηση οπιοειδών.	Μέτριο	Ασθενής
99	Η κεταμίνη θα πρέπει να χορηγείται ενδοφλέβια σε εκείνους τους ασθενείς που λαμβάνουν μείζονα οπιοειδή για αναλγησία σε μείζονες κοιλιακές χειρουργικές επεμβάσεις.	Μέτριο	Ασθενής
100	Η χορήγηση διεγχειρητικά θειικού μαγνησίου συνιστάται ως αναλγητικό συμπλήρωμα για τη βελτίωση του ελέγχου του πόνου σε ασθενείς που υποβάλλονται σε κοιλιακή χειρουργική επέμβαση.	Μέτριο	Ασθενής
101	Συνιστάται η ενδοφλέβια διεγχειρητική χορήγηση δεξμεντετομιδίνης, καθώς συμβάλλει στη μείωση του κινδύνου ανεπιθύμητων ενεργειών που σχετίζονται με τα οπιοειδή και βελτιώνει τον έλεγχο του πόνου στην διεγχειρητική και μετεγχειρητική περίοδο.	Μέτριο	Ασθενής
102	Στις ανοικτές μείζονες χειρουργικές κοιλιακές επεμβάσεις θα μπορούσε να περιλαμβάνεται αξιολόγηση της προεγχειρητικής από του στόματος χορήγησης γκαμπαπεντίνης ή πρεγκαμπαλίνης πριν από την επέμβαση για μετεγχειρητικό αναλγητικό έλεγχο.	Υψηλό	Ασθενής
103	Συνιστάται η πολυπαραγοντική διαχείριση του ασθενούς με τη χρήση εναλλακτικών των οπιοειδών φαρμάκων και τεχνικών (θωρακικός επισκληρίδιος καθετήρας, νευρομυϊκοί αποκλεισμοί, ελάχιστα επεμβατική χειρουργική επέμβαση, αποφυγή της συστηματικης χρήσης του ρινογαστρικού σωλήνα και αποφυγή της υπερβολικής ενδοφλέβιας χορήγησης υγρών) για την πρόληψη της εμφάνισης μετεγχειρητικού παραλυτικού ειλεού[1].	Υψηλό	Ισχυρός
104	Ο κίνδυνος μετεγχειρητικής ναυτίας και εμέτου πρέπει να διαστρωματώνεται σε όλους τους ασθενείς χρησιμοποιώντας την κλίμακα Apfel και πρέπει να δίνεται προφύλαξη ανάλογα με τον αναμενόμενο κίνδυνο. Η προφύλαξη με περισσότερα συνδυασμένα φάρμακα μπορεί να	Υψηλό	Ισχυρός

	πραγματοποιηθεί σε χειρουργικές επεμβάσεις στις οποίες η μετεγχειρητική ναυτία και ό έμετος (PONV) ενέχει σημαντικό κίνδυνο επιπλοκών.		
105	Η περιοχική αναισθησία συνιστάται πριν από τη γενική αναισθησία για τη μείωση της συχνότητας εμφάνισης PONV.	Υψηλό	Ισχυρός
106	Η χρήση της προποφόλης συνιστάται για την εισαγωγή και τη διατήρηση της αναισθησίας σε ασθενείς με υψηλό κίνδυνο PONV.	Υψηλό	Ισχυρός
107	Η χρήση του υποξειδίου του αζώτου θα πρέπει να αποφεύγεται σε ασθενείς με υψηλό κίνδυνο PONV ή σε μεγάλης διάρκειας χειρουργικές επεμβάσεις.	Υψηλό	Ισχυρός
108	Η χρήση εισπνεόμενων αναισθητικών θα πρέπει να αποφεύγεται σε ασθενείς με υψηλό κίνδυνο PONV.	Μέτριο	Ισχυρός
109	Συνιστάται η ελαχιστοποίηση της χρήσης των διεγχειρητικών οπιοειδών, και ιδιαίτερα των μετεγχειρητικών.	Υψηλό	Ισχυρός
110	Η αντιεμετική προφύλαξη με μονοθεραπεία θα πρέπει να πραγματοποιείται σε ασθενείς με Apfel 0-1 μόνο σε χειρουργική επέμβαση με υψηλότερο κίνδυνο PONV.	Μέτριο	Ισχυρός
111	Η αντιεμετική προφύλαξη θα πρέπει να πραγματοποιείται ως μονοθεραπεία σε ασθενείς με αξιολόγηση Apfel 2–3 και ως διπλή θεραπεία εάν η χειρουργική επέμβαση έχει υψηλότερο κίνδυνο PONV.	Υψηλό	Ισχυρός
112	Συνιστάται η χορήγηση αντιεμετικής προφύλαξης με διπλή θεραπεία σε ασθενείς με αξιολόγηση Apfel 4 και με τριπλή θεραπεία εάν η χειρουργική επέμβαση έχει υψηλότερο κίνδυνο PONV.	Υψηλό	Ισχυρός
113	Η χρήση περιφερικών ανταγωνιστών υποδοχέων οπιοειδών αποτρέπει την εμφάνιση ειλεού στην μετεγχειρητική περίοδο.	Μέτριο	Ασθενής
**ΜΕΤΕΓΧΕΙΡΗΤΙΚΑ**			
114	Η μετεγχειρητική υποθερμία θα πρέπει να αντιμετωπίζεται με χορήγηση θερμότητας μέσω αγωγής ή συναγωγής έως ότου επιτευχθεί νορμοθερμία.	Υψηλό	Ισχυρός
115	Τα μη στεροειδή αντιφλεγμονώδη φάρμακα (ΜΣΑΦ) θα πρέπει να χρησιμοποιούνται ως επικουρική θεραπεία για τον έλεγχο του πόνου σε ασθενείς που έχουν υποβληθεί σε μείζονα κοιλιακή χειρουργική επέμβαση.	Υψηλό	Ισχυρός
116	Δεν συνιστάται η τακτική χρήση τσίχλας.	Χαμηλό	Ασθενής
117	Σε εγκατεστημένη ναυτία και έμετο, οι εκλεκτικοί ανταγωνιστές των υποδοχέων σεροτονίνης 5-HT3 (ονδανσετρόνη) είναι η θεραπεία εκλογής, ακολουθούμενοι εάν οι ασθενείς δεν ανταποκρίνονται από διαφορετική οικογένεια αντιεμετικών φαρμάκων εκτός από τη δεξαμεθαζόνη.	Υψηλό	Ισχυρός
118	Η χρήση καθαρτικών όπως η δισακοδύλη (στη χειρουργική του παχέος εντέρου), το οξείδιο του μαγνησίου από το στόμα (στην υστερεκτομή), το daikenchuto (ιαπωνικό έγχυμα βοτάνων, στη γαστρεκτομή) και ο καφές (στη χειρουργική του	Χαμηλό	Ασθενής

	παχέος εντέρου) θα μπορούσε να αποτρέψει την εμφάνιση του ειλεού.		
119	Η ανοσοδιατροφή φαίνεται να συνιστάται σε υποσιτιζόμενους ασθενείς που υποβάλλονται σε επέμβαση για καρκίνο του γαστρεντερικού συστήματος, λόγω της μείωσης των λοιμωδών επιπλοκών και μιας πιθανής ελάττωσης της διάρκειας της νοσηλείας.	Χαμηλό	Ισχυρός
120	Η χρήση επισκληρίδιου αναλγησίας συνιστάται κατά τις πρώτες 24–48 ώρες μετά την επέμβαση, όπως και η αφαίρεσή της μετά από αυτήν την αρχική περίοδο ελέγχου του πόνου, μειώνοντας τις συγκεντρώσεις των τοπικών αναισθητικών με τη χρήση επισκληρίδιων οπιοειδών με σκοπό τη μείωση του κινητικού αποκλεισμού ώστε να επιτραπεί η κινητοποίηση.	Υψηλό	Ισχυρός
121	Η χρήση παρακεταμόλης και ΜΣΑΦ συνιστάται για τον μετεγχειρητικό έλεγχο του πόνου με δόσεις διάσωσης οπιοειδών σε σοβαρό μη ελεγχόμενο πόνο σε ασθενείς με επισκληρίδιο αναλγησία ή άλλες τεχνικές τοπικής ή περιοχικής αναλγησίας.	Υψηλό	Ισχυρός
122	Η πρώιμη μετεγχειρητική σίτιση θα πρέπει να ξεκινά το συντομότερο δυνατό, εντός ωρών μετά την επέμβαση στους περισσότερους ασθενείς.	Μέτριο (Υψηλό στην χειρουργική του παχέος εντέρου)	Ισχυρός
123	Συνιστάται η έγκαιρη κινητοποίηση μέσω εκπαίδευσης και ενθάρρυνσης των ασθενών για τη μείωση του αριθμού των ανεπιθύμητων ενεργειών.	Μέτριο	Ισχυρός
124	Συνιστάται προεγχειρητική και μετεγχειρητική αναπνευστική φυσιοθεραπεία.	Υψηλό	Ισχυρός
125	Η από του στόματος χορήγηση αλάτων σιδήρου δεν συνιστάται στην άμεση μετεγχειρητική περίοδο για τη βελτίωση του επιπέδου της αιμοσφαιρίνης και τη μείωση του ρυθμού μετάγγισης.	Μέτριο	Ισχυρός
126	Αντίθετα, η μετεγχειρητική θεραπεία με ενδοφλέβιο σίδηρο προτείνεται για τη βελτίωση των επιπέδων αιμοσφαιρίνης και τη μείωση του ρυθμού μετάγγισης, ειδικά σε ασθενείς με χαμηλές αποθήκες σιδήρου ή/και μέτρια-σοβαρή μετεγχειρητική αναιμία.	Μέτριο	Ισχυρός
127	Η εφαρμογή «περιοριστικών» κριτηρίων για τη μετάγγιση συμπυκνωμένων ερυθρών αιμοσφαιρίων (ΣΕ) συνιστάται (εάν υπάρχουν συμπτώματα ή επίπεδο Hb <7 g/dl), στους περισσότερους νοσηλευόμενους ασθενείς (παθολογικούς, χειρουργικούς ή σε κρίσιμη κατάσταση), χωρίς ενεργό αιμορραγία και που είναι αιμοδυναμικά σταθεροί (συμπεριλαμβανομένων των σηπτικών ασθενών, των ασθενών με αιμορραγία από το ανώτερο γαστρεντερικό και των ασθενών με επιλόχεια αναιμία).	Υψηλό	Ισχυρός
128	Η εφαρμογή «περιοριστικών» κριτηρίων για μετάγγιση ΣΕ (Hb ≤7.5 g/dl) συνιστάται σε καρδιοχειρουργικούς ασθενείς	Μέτριο	Ισχυρός

129	Η εφαρμογή «περιοριστικών» κριτηρίων για μετάγγιση ΣΕ (Hb <8 g/dl) συνιστάται σε ασθενείς με ιστορικό καρδιαγγειακής νόσου που υποβλήθηκαν σε ορθοπεδική επέμβαση ή χειρουργική επέμβαση αποκατάστασης κατάγματος ισχίου.	Μέτριο	Ισχυρός
130	Καθαρίστε το χειρουργικό τραύμα με στείρο ισότονο φυσιολογικό ορό, πόσιμο νερό ή αποσταγμένο νερό.	Μέτριο	Ισχυρός
131	Τοπικά αντιβιοτικά μπορούν να εφαρμοστούν σε χειρουργικά τραύματα με επούλωση κατά πρώτο σκοπό μετά από χειρουργική επέμβαση για την πρόληψη λοίμωξης του χειρουργικού πεδίου.	Χαμηλό	Ασθενής
132	Σε τραύματα με επούλωση κατά πρώτο σκοπό, όποτε είναι δυνατόν, συνιστάται να μην αλλάζεται το επίθεμα κατά τις πρώτες 24–48 ώρες.	Χαμηλό	Ασθενής
133	Η χρήση θεραπείας τραυμάτων αρνητικής πίεσης μπορεί να μειώσει τον κίνδυνο λοίμωξης του χειρουργικού πεδίου και να συντομεύσει την επούλωση σε ανοιχτά χειρουργικά τραύματα, κυρίως σε χειρουργικές επεμβάσεις στην κοιλιά ή στον θώρακα.	Χαμηλό	Ασθενής
134	Οι ασθενείς και οι φροντιστές τους θα πρέπει να λαμβάνουν εξατομικευμένες, κατανοητές και πλήρεις οδηγίες κατά το εξιτήριο. Ο προγραμματισμός του εξιτηρίου και η παροχή επαρκών πληροφοριών για τη φροντίδα μετά το εξιτήριο επηρεάζει τη μέση νοσηλεία και τις επανεισαγωγές.	Υψηλό	Ισχυρός
135	Συνιστώνται έλεγχοι για την εφαρμογή των πρωτοκόλλων βελτιστοποίησης της μετεγχειρητικής ανάρρωσης με σκοπό την αξιολόγηση της κλινικής επάρκειας και αποτελεσματικότητας.	Μέτριο	Ισχυρός

7. Συστάσεις και πηγές τεκμηρίωσης

7.1. ΓΕΝΙΚΑ

7.1.1. ΠΡΟΕΤΟΙΜΑΣΙΑ ΤΟΥ ΑΣΘΕΝΟΥΣ (ΩΣ ΕΞΩΤΕΡΙΚΟΥ)

Ενημέρωση του ασθενούς και του περιβάλλοντός του

Οι πληροφορίες που δίνονται στους ασθενείς και το περιβάλλον τους είναι σημείο κλειδί στη χειρουργική διαδικασία. Οι εκ των προτέρων ενημέρωση, συμβουλευτική και παροχή πληροφοριών ευνοούν την πρόωρη έξοδο από το νοσοκομείο[1] και μειώνουν τη νοσηλεία[2,3]. Ο ασθενής πρέπει να γνωρίζει τις θεραπευτικές επιλογές και να έχει ρεαλιστικές προσδοκίες για τους πιθανούς κινδύνους και τα οφέλη. Η επίτευξη της μέγιστης συνεργασίας με τον ασθενή καθ' όλη τη διάρκεια της θεραπείας είναι ο κύριος στόχος[4,5,6].

Το καλύτερο όργανο μέτρησης για την αξιολόγηση του επιπέδου προετοιμασίας των ασθενών για την επέμβαση είναι η γνώμη τους σχετικά με το πόσο καλά προετοιμασμένοι αισθάνονται[7].

Εκτός από τη συμμετοχή της χειρουργικής ομάδας σε αυτή τη φάση, απαραίτητη είναι και η εμπλοκή του νοσηλευτικού προσωπικού που θα συμμετάσχει αργότερα στη μετεγχειρητική περίοδο. Οι πληροφορίες πρέπει να παρέχονται τόσο προφορικά όσο και γραπτά.

Οι πληροφορίες πρέπει να εξατομικεύονται, προσαρμόζοντάς τις στα χαρακτηριστικά κάθε ασθενή (ικανότητα κατανόησης, πολιτισμικό επίπεδο κ.λπ.). Είναι γνωστό ότι ένα μεγάλο μέρος των λεκτικών πληροφοριών που παρέχονται στους ασθενείς στην προεγχειρητική περίοδο ξεχνιέται. Μερικές φορές λιγότερο από το 25% των παρεχόμενων πληροφοριών απομνημονεύονται, ειδικά αυτές που σχετίζονται με την προεγχειρητική φαρμακευτική αγωγή[8-10].

Η χρήση ενημερωτικών φυλλαδίων είναι ιδιαίτερα χρήσιμη για την επίτευξη της μέγιστης συνεργασίας στα πρωτοκόλλα βελτιστοποίησης της

μετεγχειρητικής ανάρρωσης. Έχει αποδειχθεί ότι αυτές οι πληροφορίες βελτιώνουν την ικανοποίηση των ασθενών και μειώνουν το άγχος και τον μετεγχειρητικό πόνο. Αυτά τα φυλλάδια θα πρέπει να περιλαμβάνουν τα κύρια σημεία της μετεγχειρητικής αποκατάστασης, τα οφέλη που προκύπτουν και τον τρόπο απόκτησής τους, ειδικά αυτά που αναφέρονται στην κινητοποίηση, τη διατροφή και τις αναπνευστικές ασκήσεις. Εάν πρόκειται να γίνει στομία, η επίσκεψη σε ειδικό πριν την επέμβαση βελτιώνει σημαντικά τα αποτελέσματα[11-14].

1. Οι ασθενείς θα πρέπει να λαμβάνουν πλήρεις προφορικές και γραπτές πληροφορίες για το τι απαιτείται για τη βελτίωση της ανάρρωσής τους μετά την επέμβαση.

Μέτριο επίπεδο τεκμηρίωσης. Ισχυρή σύσταση.

ΒΙΒΛΙΟΓΡΑΦΙΚΕΣ ΑΝΑΦΟΡΕΣ

1. Nelson G, Bakkum-Gamez J, Kalogera E, Glaser G, Altman A, Meyer LA et al. Guidelines for perioperative care in gynecologic/oncology: Enhanced Recovery After Surgery (ERAS) Society recommendations-2019 update. Int J Gynecol Cancer. 2019; 29(4):651-668.
2. Gustafsson UO, Scott MJ, Hubner M, Nygen J, Demartines N Francis N, et al. Guidelines for Perioperative Care in Elective Colorectal Surgery: Enhanced Recovery After Surgery (ERAS ®) Society Recommendations: 2018.World J Surg. 2019; 43(3):659-695.
3. Wongkietkachorn A, Wongkietkachorn N, Rhunsiri P. Preoperative needs-based education to reduce anxiety, increase satisfaction, and decrease time spent in day surgery: a randomized controlled trial. World J Surg. 2018, 42(3):666-674.
4. Sugai DY, Deptula PL, Parsa AA, Parsa FD. The importance of communication in the management of postoperative pain. Hawaii J Med Public Health. 2013; 72(6):180-4.
5. Forster AJ, Clark HD, Menard A, Dupuis N, Chernish R, Chandok N et al. Effect of a nurse team coordinator on outcomes for hospitalized medicine patients. Am J Med 2005; 118(10):1148-53.

6. Chan Z, Kan C, Lee P, Chan I, Lam J. A systematic review of qualitative studies: patients' experiences of preoperative communication. J Clin Nurs. 2012; 21(5-6):812-24.
7. Forshaw KL, Carey ML, Hall AE, Boyes AW, Sanson-Fisher R. Preparing patients for medical interventions: A systematic review of the psychometric qualities of published instrument. Patient Educ Couns. 2016; 99(6):960-73.
8. Ronco M, Iona L, Fabbro C, Bulfone G, Palese A. Patient education outcomes in surgery: a systematic review from 2004 to 2010. Int J Evid Based Healthc. 2012; 10(4):309-23
9. Kruzik N. Benefits of preoperative education for adult elective surgery patients. AORN J. 2009; 90(3):381-7.
10. Broadbent E, Kahokehr A, Booth RJ, Thomas J, Windsor JA, Buchanan CM et al. A brief relaxation intervention reduces stress and improves surgical wound healing response: a randomised trial. Brain Behav Immun 2012; 26(2):212-7.
11. Mora M, Shell JE, Thomas CS, Ortiguera CJ, O'Connor MI. Gender differences in questions asked in an online preoperative patient education program. Gend Med 2012;9(6):457- 62.
12. Smith F, Carlsson E, Kokkinakis D, Forsberg M, Kodeda K, Sawatzky R, et al. Readability, suitability and comprehensibility in patient education materials for Swedish patients with colorectal cancer undergoing elective surgery: a mixed method design. Patient Educ Couns 2014;94(2):202-9.
13. Sandberg EH, Sharma R, Sandberg WS. Deficits in retention for verbally presented medical information. Anesthesiology 2012; 117(4):772-9.
14. Lee A, Gin T. Educating patients about anaesthesia: effect of various modes on patients' knowledge, anxiety and satisfaction. Curr Opin Anaesthesiol 2005;18(2):205-8.

Ευπαθής ασθενής

Θα πρέπει να τονιστεί η σημασία της αξιολόγησης της ευπάθειας και των γνωστικών δυσλειτουργιών του ασθενούς, λόγω της επίδρασης που έχουν στα μετεγχειρητικά αποτελέσματα. Ο πληθυσμός γερνάει και ο επιπολασμός της ευπάθειας και των γνωστικών δυσλειτουργιών πλησιάζει το 50% των ασθενών

που αναμένουν χειρουργική επέμβαση. Αν και δεν υπάρχει ιδανική κλίμακα, οποιοδήποτε εργαλείο διαλογής (screening) είναι καλύτερο από κανένα[1-5].

2. Συνιστάται η προεγχειρητική αξιολόγηση της ευπάθειας για τον εντοπισμό ασθενών με υψηλότερο περιεγχειρητικό κίνδυνο

Υψηλό επίπεδο τεκμηρίωσης. Ισχυρή σύσταση.

ΒΙΒΛΙΟΓΡΑΦΙΚΕΣ ΑΝΑΦΟΡΕΣ

1. Dalton A, Zafirova Z. Preoperative Management of the Geriatric Patient: Frailty and Cognitive Impairment Assessment. Anesthesiol Clin. 2018 ;36(4):599-614.

2. Subramaniam S, Aalberg J, Soriano RP, Divino CM. New 5-Factor Modified Frailty Index Using American College of Surgeons NSQIP Data. J Am Coll Surg. 2018 ;226(2):173-181.

3. Wang J, Zou Y, Zhao J, Schneider DB, Yang Y, Ma Y et al. The Impact of Frailty on Outcomes of Elderly Patients After Major Vascular Surgery: A Systematic Review and Meta-analysis. Eur J Vasc Endovasc Surg. 2018;56(4):591-602.

4. Castellví Valls J, Borrell Brau N, Bernat MJ, Iglesias P, Reig L, Pascual L, et al. Colorectal carcinoma in the frail surgical patient. Implementation of a Work Area focused on the Complex Surgical Patient improves postoperative outcome. Cir Esp. 2018;96(3):155-161.

5. Shen Y, Hao Q, Zhou J, Dong B. The impact of frailty and sarcopenia on postoperative outcomes in older patients undergoing gastrectomy surgery: a systematic review and me- ta-analysis. BMC Geriatr. 2017;17(1):188.

Προεγχειρητική καρδιοπάθεια

Οι ασθενείς με πρόσφατης έναρξης ή μη αντιρροπούμενη ενεργό καρδιακή νόσο θα πρέπει να αξιολογούνται από μια διεπιστημονική ομάδα που θα περιλαμβάνει όλους τους γιατρούς που σχετίζονται με τη διαχείριση της περιεγχειρητικής περιόδου, καθώς οι παρεμβάσεις μπορεί να έχουν επιπτώσεις στη χειρουργική και αναισθησιολογική διαχείριση[1,2].

3. Οι ασθενείς με οξεία ή μη αντιρροπούμενη καρδιοπάθεια θα πρέπει να αξιολογούνται από διεπιστημονική ομάδα λόγω του αναισθησιολογικού και χειρουργικού κινδύνου.

Υψηλό επίπεδο τεκμηρίωσης. Ισχυρή σύσταση.

ΒΙΒΛΙΟΓΡΑΦΙΚΕΣ ΑΝΑΦΟΡΕΣ

1. Kristensen SD, Knuuti J, Saraste A, Anker S, Bøtker HE, Hert SD, et al. 2014 ESC/ESA Guidelines on non-cardiac surgery: cardiovascular assessment and management: The Joint Task Force on non-cardiac surgery: cardiovascular assessment and management of the European Society of Cardiology (ESC) and the European Society of Anaesthesiology (ESA). Eur Heart J. 2014;35(35):2383-431.

2. Duceppe E, Parlow J, MacDonald P, Lyons K, McMullen M, Srinathan S, et al. Canadian Cardiovascular Society Guidelines on Perioperative Cardiac Risk Assessment and Management for Patients Who Undergo Noncardiac Surgery. Can J Cardiol. 2017;33(1):17-32.

Εκτίμηση αναισθησιολογικού – χειρουργικού κινδύνου

Εκτός από τον περιεγχειρητικό κίνδυνο που συνεπάγεται κάθε χειρουργική επέμβαση, υπάρχει ένας επιπλέον κίνδυνος που προκύπτει από τη φυσική κατάσταση του ασθενούς πριν από την επέμβαση. Η αξιολόγηση του κινδύνου με βάση την ταξινόμηση ASA εξακολουθεί να είναι μια από τις καλύτερες και απλούστερες κλίμακες για την αξιολόγηση της φυσικής κατάστασης ενός ασθενούς[1]. Ωστόσο, για να εκτιμηθεί ο περιεγχειρητικός κίνδυνος, πρέπει να προστεθούν η ευπάθεια του ασθενούς, η γνωστική δυσλειτουργία και ο χειρουργικός κίνδυνος, λόγω της επίδρασής τους στα μετεγχειρητικά αποτελέσματα. Ο πληθυσμός γερνάει και ο επιπολασμός αυτών των δύο τελευταίων οντοτήτων είναι κοντά στο 50%. Αν και δεν υπάρχει ιδανική κλίμακα για τη μέτρησή τους, οποιοδήποτε εργαλείο ανίχνευσης είναι καλύτερο από κανένα[2,3].

4. Η αξιολόγηση της φυσικής κατάστασης του ασθενούς με τη χρήση της ταξινόμησης ASA συνιστάται σε όλους τους ασθενείς που υποβάλλονται σε χειρουργική επέμβαση.

Υψηλό επίπεδο τεκμηρίωσης. Ισχυρή σύσταση.

ΒΙΒΛΙΟΓΡΑΦΙΚΕΣ ΑΝΑΦΟΡΕΣ

1. Mayhew D, Mendonca V, Murthy BVS. A review of ASA physical status-historical perspectives and modern developments. Anaesthesia 2019; 74:373-9.

2. Dalton A, Zafirova Z. Preoperative Management of the Geriatric Patient: Frailty and Cognitive Impairment Assessment. Anesthesiol Clin. 2018;36(4):599-614.

3. Subramaniam S, Aalberg J, Soriano RP, Divino CM. New 5-Factor Modified Frailty Index Using American College of Surgeons NSQIP Data. J Am Coll Surg. 2018;226(2):173-18.

Συστάσεις στον ασθενή για τοξικές συνήθειες

Η κατανάλωση καπνού και η κατάχρηση αλκοόλ είναι δύο συνήθειες που επηρεάζουν αρνητικά την ανάρρωση του ασθενούς μετά την επέμβαση. Σχετίζονται με αναπνευστικές, μεταβολικές, λοιμώδεις, αιμορραγικές επιπλοκές και επιπλοκές από το χειρουργικό τραύμα[1]. Πριν από την επέμβαση, η υπερβολική κατανάλωση αλκοόλ και το κάπνισμα θα πρέπει να αξιολογούνται χρησιμοποιώντας επικυρωμένα εργαλεία ανίχνευσης[2].

Το κάπνισμα σχετίζεται επίσης με κακή μετεγχειρητική ποιότητα ζωής και κόπωση, και μείωση της μακροχρόνιας επιβίωσης σε ασθενείς που υποβάλλονται σε χειρουργική επέμβαση θώρακος[3]. Στο προεγχειρητικό στάδιο, η παροχή συμβουλών από τη νοσηλεύτρια πριν την εισαγωγή, τα ενημερωτικά φυλλάδια και η θεραπεία υποκατάστασης νικοτίνης είναι πιο πιθανό να είναι αποτελεσματικά για τη διακοπή του καπνίσματος έως και 30 ημέρες μετά την επέμβαση[4]. Αποχή μεταξύ 4-8 εβδομάδων είναι απαραίτητη για τη μείωση των αναπνευστικών επιπλοκών και των επιπλοκών επούλωσης των τραυμάτων[5].

Η κατανάλωση περισσότερων από δύο μονάδων αλκοόλ την ημέρα (20 γραμμάρια αιθανόλης) αυξάνει την διεγχειρητική αιμορραγία και το ποσοστό

μετεγχειρητικών λοιμώξεων. Οι προεγχειρητικές παρεμβάσεις για τη διακοπή της κατανάλωσης αλκοόλ μπορούν να μειώσουν σημαντικά τα ποσοστά των μετεγχειρητικών επιπλοκών[6].

5. Συνιστάται η διακοπή του καπνίσματος 4-8 εβδομάδες πριν από την επέμβαση για να την μείωση των σχετιζόμενων επιπλοκών.

Υψηλό επίπεδο τεκμηρίωσης. Ισχυρή σύσταση.

6. Η κατανάλωση αλκοόλ θα πρέπει να διακόπτεται ένα μήνα πριν από την επέμβαση.

Μέτριο επίπεδο τεκμηρίωσης. Ισχυρή σύσταση.

ΒΙΒΛΙΟΓΡΑΦΙΚΕΣ ΑΝΑΦΟΡΕΣ

1. Gaskill CE, Kling CE, Varghese TK Jr, etal. Financial benefit of a smoking cessation program prior to elective colorectal surgery. J Surg Res. 2017; 215:183-189.
2. Engelman DT, Ben Ali W, Williams JB, Perrault LP, Reddy VS, Arora RC, et al. Guidelines for Perioperative Care in Cardiac Surgery: Enhanced Recovery After Surgery Society Recommendations. JAMA Surg. 2019; 154(8):755-766.
3. Batchelor TJP, Rasburn NJ, Abdelnour-Berchtold E, Brunelli A, Cerfolio RJ, Gonzalez M, et al. Guidelines for enhanced recovery after lung surgery: recommendations of the Enhanced Recovery After Surgery (ERAS®) Society and the European Society of Thoracic Surgeons (ESTS). Eur J Cardiothorac Surg. 2019:55(1):91-115.
4. Low DE, Allum W, De Manzoni G, Ferri L, Immanuel A, Kuppusamy M, et al. Guidelines for Perioperative Care in Esophagectomy: Enhanced Recovery After Surgery (ERAS®) Society Recommendations. World Journal of Surgery. 2019;43(2):299-330.
5. Gustafsson UO, Scott MJ, Hubner M, Nygren J, Demartines N, Francis N, et al. Guidelines for Perioperative Carein Elective Colorectal Surgery: Enhanced Recovery After Surgery(ERAS®) Society Recommendations: 2018. World Journal of Surgery. 2019;43(3):659-95.

6. Shabanzadeh DM, Sørensen LT. Alcohol Consumption Increases Post-Operative Infection but Not Mortality: A Systematic Review and Meta-Analysis. Surgical Infections. 2015;16 (6):657-68.

Προαποκατάσταση (Prehabilitation)

Η χειρουργική προαποκατάσταση χρησιμοποιεί πολυπαραγοντικές πολυτροπικές δράσεις στην προεγχειρητική περίοδο που στοχεύουν να βοηθήσουν τον ασθενή να ξεπεράσει τις οργανικές επιπτώσεις που σχετίζονται με τη χειρουργική «επιθετικότητα», όχι μόνο στην άμεση μετεγχειρητική περίοδο αλλά και μακροπρόθεσμα[1-3]. Αυτό το μοντέλο βασίζεται στην τριτροπική προαποκατάσταση, που αποτελεί το άθροισμα της φυσικοθεραπείας, των συμπληρωμάτων διατροφής πλούσιων σε πρωτεΐνες και της γνωσιακής θεραπείας, με στόχο τη μείωση της κατάθλιψης και του άγχους που σχετίζονται με τη διαδικασία[4]. Δεν υπάρχει συναίνεση για το είδος των ασκήσεων που πρέπει να εκτελούν οι ασθενείς. Τα τρέχοντα δεδομένα υποστηρίζουν τη συμπερίληψη της σωματικής άσκησης και της εκπαίδευσης. Το πρόγραμμα σωματικής άσκησης πρέπει να περιλαμβάνει βελτίωση της αντοχής (αερόμπικ), μυϊκή ενδυνάμωση (περιφερική) και ασκήσεις εισπνευστικών μυών[5-6]. Όσον αφορά την εκπαίδευση, θα πρέπει να διδάσκονται ασκήσεις αναπνευστικής φυσικοθεραπείας και αυτοδιαχείρισης στην άμεση μετεγχειρητική περίοδο[1-5]. Υπάρχει σαφής συναίνεση ότι η προαποκατάσταση του ασθενούς δεν πρέπει να αναβάλλει τη χειρουργική επέμβαση πέραν των 4 εβδομάδων, χρόνος που φαίνεται επαρκής για την επίτευξη βελτίωσης της λειτουργικής ικανότητας πριν από το χειρουργείο[6]. Δεν υπάρχει συναίνεση σχετικά με τα τεστ λειτουργικής αξιολόγησης.

7. Συνιστάται θεραπεία τριτροπικής προαποκατάστασης για τη βελτίωση της λειτουργικής ικανότητας πριν από τη χειρουργική επέμβαση.

Μέτριο επίπεδο τεκμηρίωσης. Ισχυρή σύσταση.

ΒΙΒΛΙΟΓΡΑΦΙΚΕΣ ΑΝΑΦΟΡΕΣ

1. Hughes MJ, Hackney RJ, Lamb PJ, Wigmore SJ, Christopher Deans DA, Skipworth RJE. Prehabilitation Before Major Abdominal Surgery: A Systematic Review and Meta-analysis. World J Surg. 2019;43(7):1661-68.

2. Rosero ID, Ramírez-Vélez R, Lucia A, Martínez-Velilla N, Santos-Lozano A, Valenzuela PL, et al. Systematic Review and Meta-Analysis of Randomized, Controlled Trials on Preoperative Physical Exercise Interventions in Patients with Non-Small-Cell Lung Cancer. Cancers. 2019;11(7):944.

3. Chen X, Hou L, Zhang Y, Liu X, Shao B, Yuan B, et al. The effects of five days of intensive preoperative inspiratory muscle training on postoperative complications and outcome in patients having cardiac surgery: a randomized controlled trial. Clin Rehabil. 2019; 33(5): 913-22.

4. Luther A, Gabriel J, Watson RP, Francis NK. The Impact of Total Body Prehabilitation on Post-Operative Outcomes After Major Abdominal Surgery: A Systematic Review. World J Surg. 2018; 42(9): 2781-91.

5. Milder DA, Pillinger NL, Kam PCA. The role of prehabilitation in frail surgical patients: A systematic review. Acta Anaesthesiol Scand. 2018; 62(10):1356-66.

6. Boden I, Skinner EH, Browning L, Reeve J, Anderson L, Hill C, et al. Preoperative physiotherapy for the prevention of respiratory complications after upper abdominal surgery: pragmatic, double blinded, multicentre randomised controlled trial. BMJ. 2018; 24: 360: j5916.

Προεγχειρητική διατροφική αξιολόγηση

Η προεγχειρητική υποθρεψία σχετίζεται με αυξημένη μετεγχειρητική νοσηρότητα και θνησιμότητα και παρατείνει την παραμονή στο νοσοκομείο για χειρουργικούς ασθενείς[1]. Για το λόγο αυτό, είναι απαραίτητο να διεξαχθεί ένας διατροφικός έλεγχος προ της εισαγωγής για όλους τους ασθενείς που υποβάλλονται σε προγραμματισμένη μείζονα χειρουργική επέμβαση. Συνιστάται η χρήση διατροφικών εργαλείων διαλογής (screening) που περιλαμβάνουν το δείκτη μάζας σώματος (ΔΜΣ – BMI), ακούσια απώλεια βάρους, μείωση πρόσφατης πρόσληψης τροφής και το βαθμό του στρες ή τη σοβαρότητα της νόσου[2,3]. Σε ασθενείς που διατρέχουν κίνδυνο υποθρεψίας, θα πρέπει να διενεργείται πλήρης διατροφική αξιολόγηση (assessment) για τη

διάγνωσή του και την έναρξη της κατάλληλης διατροφικής θεραπείας. Σήμερα, η μεθοδολογία που χρησιμοποιείται για τη διάγνωση του υποσιτισμού βασίζεται στα κριτήρια Global Leadership Initiative on Malnutrition – GLIM (Παγκόσμια Ηγετική Πρωτοβουλία για τον Υποθρεψία), σύμφωνα με τα οποία πρέπει να πληρούνται τουλάχιστον ένα φαινοτυπικό κριτήριο (απώλεια βάρους, ΔΜΣ, μείωση μυϊκής μάζας) και ένα αιτιολογικό κριτήριο (μείωση διατροφικής πρόσληψης / απορρόφησης θρεπτικών συστατικών, φλεγμονώδης κατάσταση)[4]. (Βλ. Παράρτημα 10.3: Αλγόριθμος διατροφικής αξιολόγησης).

Η αλβουμίνη ορού και η προαλβουμίνη ή η C-αντιδρώσα πρωτεΐνη (CRP) αντανακλούν τον βαθμό συστηματικής φλεγμονής και δεν είναι ειδικά για τη διατροφική κατάσταση[3]. Αν και η αλβουμίνη είναι προγνωστικός παράγοντας της μετεγχειρητικής νοσηρότητας και θνησιμότητας, δεν είναι χρήσιμη για τον προσδιορισμό της διατροφικής κατάστασης καθώς τα επίπεδά της μεταβάλλονται αντιστρόφως ανάλογα με τον βαθμό φλεγμονής του ασθενούς και αλλάζουν με την κατάσταση ενυδάτωσης[5].

8. Συνιστάται διατροφικός έλεγχος (screening) για όλους τους ασθενείς που υποβάλλονται σε μείζονα χειρουργική επέμβαση.

Μέτριο επίπεδο τεκμηρίωσης. Ισχυρή σύσταση.

9. Όταν διαπιστωθεί ότι ένας ασθενής διατρέχει κίνδυνο υποθρεψίας, θα πρέπει να διενεργείται πλήρης διατροφική αξιολόγηση (assessment), με τη δημιουργία ενός σχεδίου διατροφικής θεραπείας, την παρακολούθηση της ανοχής και της ανταπόκρισης σε αυτό το σχέδιο.

Μέτριο επίπεδο τεκμηρίωσης. Ισχυρή σύσταση.

ΒΙΒΛΙΟΓΡΑΦΙΚΕΣ ΑΝΑΦΟΡΕΣ

1. Arends J, Bachmann P, Baracos V, Barthelemy N, Hartmut B, Bozzetti F, et al. ESPEN guidelines on nutrition in cancer patients. Clin Nutr. 2017; 36:11-48.
2. Kondrup J, Allison SP, Elia M, Vellas B, Plauth M. Educational and Clinical Practice Committee, European Society of Parenteral and Enteral Nutrition (ESPEN). ESPEN guidelines for nutrition screening 2002. Clin Nutr. 2003; 22:415-21.

3. Jensen GL, Compher C, Sullivan DH, Mullin GE. Recognizing malnutrition in adults: definitions and characteristics, screening, assessment, and team approach. J Parenter Enteral Nutr. 2013; 37:802-7.
4. Cederholm T, Jensen GL, Correia MITD, Gonzalez MC. Fukushima R, Higashiguchi T, et al. GLIM criteria for the diagnosis of malnutrition: a consensus report from the global clinical nutrition community. Clin Nutr. 2019; 38:1-9.
5. White JV, Guenter P, Jensen G, Malone A, Schofield M, Academy Malnutrition Work Group; A.S.P.E.N. Malnutrition Task Force; A.S.P.E.N. Board of Directors. Consensus statement: academy of nutrition and Dietetics and American society for parenteral and enteral nutrition: characteristics recommended for the identification and documentation of adult malnutrition (undernutrition). J Parenter Enter Nutr. 2012; 36:275-83.

Προεγχειρητική διατροφική παρέμβαση

Το όφελος της προεγχειρητικής διατροφικής θεραπείας έχει αποδειχθεί κυρίως σε ασθενείς με διατροφικό κίνδυνο ή σοβαρή υποθρεψία[1]. Η χορήγηση διατροφικής υποστήριξης (από το στόμα, εντερική, παρεντερική) σε ασθενείς με υποθρεψία ή σοβαρό διατροφικό κίνδυνο για τουλάχιστον 7-10 ημέρες πριν από το χειρουργείο σχετίζεται με μείωση των λοιμωδών επιπλοκών και της αναστομωτικής διαφυγής, καθώς και με μικρότερη νοσηλεία στο νοσοκομείο[1-3].

10. Όλοι οι ασθενείς σε σοβαρό διατροφικό κίνδυνο ή σοβαρή υποθρεψία θα πρέπει να λαμβάνουν διατροφική θεραπεία τουλάχιστον 7–10 ημέρες πριν από την επέμβαση. Η από του στόματος / εντερική οδός θα πρέπει να προτιμάται, εάν είναι δυνατόν.

Μέτριο επίπεδο τεκμηρίωσης. Ισχυρή σύσταση.

ΒΙΒΛΙΟΓΡΑΦΙΚΕΣ ΑΝΑΦΟΡΕΣ

1. Jie B, Jiang ZM, Nolan MT, Zhu SN, Yu K, Kondrup J. Impact of preoperative nutritional support on clinical outcome in abdominal surgical patients at nutritional risk. Nutrition. 2012; 28:1022-7.

2. Waitzberg DL, Saito H, Plank LD, Jamieson GG, Jagannath P, Hwang TL, et al Postsurgical infections are reduced with specialized nutrition support. World J Surg. 2006; 30:1592- 1604.
3. Fukuda Y, Yamamoto K, Hirao N, Nishikawa K, Maeda S, Haraguchi N, et al. Prevalence of malnutrition among gastric cancer patients undergoing gastrectomy and optimal preoperative nutritional support for preventing surgical site infections. Ann Surg Oncol. 2015; (Suppl. 3):778-85.

Ανοσοδιατροφή

Η ανοσοδιατροφή αποτελεί αντικείμενο συζήτησης από τη δεκαετία του 1990, ειδικά στη χειρουργική του καρκίνου[1]. Ορισμένες ανασκοπήσεις και μετα-αναλύσεις έχουν δείξει τα ευεργετικά αποτελέσματα της ανοσοδιατροφής αθροίζοντας τα αποτελέσματα των τυχαιοποιημένων ελεγχόμενων μελετών (randomized controlled trials - RCTs) σε όλους τους τύπους ασθενών και εξετάζοντας ολόκληρη την περιεγχειρητική περίοδο. Ωστόσο, άλλες μελέτες δεν βρήκαν πρόσθετο όφελος από τη χρήση της ανοσοδιατροφής έναντι των τυπικών συμπληρωμάτων χρησιμοποιώντας παρόμοιες μεθόδους[2].

Σύμφωνα με τις Κλινικές Κατευθυντήριες Οδηγίες για την Κλινική Διατροφή και την Χειρουργική της ESPEN (European Society of Parenteral and Enteral Nutrition – Ευρωπαϊκή Εταιρία Εντερικής και Παρεντερικής Διατροφής) το 2017, συγκεκριμένα σκευάσματα με ανοσοθρεπτικά συστατικά θα πρέπει να χορηγούνται στο περιεγχειρητικό ή τουλάχιστον στο μετεγχειρητικό στάδιο σε υποθρεπτικούς ασθενείς που υποβάλλονται σε μείζονα χειρουργική επέμβαση για καρκίνο, με ενδιάμεσο βαθμό σύστασης (SIGN, Scottish Intercollegiate Guidelines Network)[3]. Δεν υπάρχουν ξεκάθαρα δεδομένα για τη χρήση της σε σύγκριση με τα τυπικά από του στόματος συμπληρώματα αποκλειστικά στην προεγχειρητική περίοδο.

Συνεχίζουν να δημοσιεύονται μετα-αναλύσεις σχετικά με αυτά τα ευρήματα, με ορισμένα κοινά θετικά αποτελέσματα, αν και με δεδομένα που δεν είναι πάντοτε ισχυρά[4,5].

11. Δεν υπάρχουν επαρκή στοιχεία που να προτείνουν την ανοσοδιατροφή έναντι της χρήσης τυπικών από του στόματος συμπληρωμάτων αποκλειστικά κατά την προεγχειρητική περίοδο.

Χαμηλό επίπεδο τεκμηρίωσης. Ασθενής σύσταση.

ΒΙΒΛΙΟΓΡΑΦΙΚΕΣ ΑΝΑΦΟΡΕΣ

1. Arends J, Bachmann P, Baracos V, et al. ESPEN guidelines on nutrition in cancer patients. Clin Nutr 2017; 36:11-48.

2. Hegazi RA, Hustead DS, Evans DC. Properative standard oral nutrition supplements vs immunonutrition: results of a systematic review and meta-analysis. J Am Coll Surg 2014; 219: 1078-1087.

3. Weimann A, Braga M, Carli F, Higashiguchi T, Hübner M, Klek S, et al. ESPEN guideline: Clinical nutrition in surgery. Clin Nutr 2017; 36: 623-650.

4. Probst P, Ohmann S, Klaiber U, Hüttner FJ, Billeter AT, Ulrich A, Büchler MW, Diener MK. Meta-analysis of immunonutrition in major abdominal surgery. Br J Surg 2017; 104: 1594- 1608.

5. Adiamah A, Skorepa P, Weimann A, Lobo DN. The impact of preoperative immune modulating nutrition on outcomes in patients undergoing surgery for gastrointestinal surgery for gastrointestinal cancer. Ann Surg 2019; 270: 247-256.

Αξιολόγηση και θεραπεία της αναιμίας

Η αναιμία είναι ο κύριος ανεξάρτητος παράγοντας κινδύνου για νοσηρότητα και θνησιμότητα τόσο στις προγραμματισμένες όσο και στις επείγουσες χειρουργικές επεμβάσεις. Αυτή η σχέση ισχύει ακόμα και σε ήπιες περιπτώσεις. Τα χαμηλά επίπεδα αιμοσφαιρίνης σχετίζονται με αυξημένη συχνότητα νοσοκομειακών λοιμώξεων ή λοιμώξεων που σχετίζονται με τη φροντίδα. Η συχνότητα της προεγχειρητικής αναιμίας είναι περίπου 20-30%. Τα επίπεδα αιμοσφαιρίνης σχετίζονται αντιστρόφως ανάλογα με τον κίνδυνο λήψης αλλογενούς μετάγγισης[1-5].

Η μετάγγιση αίματος σχετίζεται, με δοσοεξαρτώμενη επίδραση, με υψηλότερο κίνδυνο νοσοκομειακής λοίμωξης, θρομβοεμβολικών επεισοδίων, επανεπεμβάσεων, επανεισαγωγής, μεγαλύτερης διάρκειας παραμονής στη

ΜΕΘ και νοσηλείας στο νοσοκομείο και ακόμη υψηλότερο ποσοστό μετεγχειρητικής θνησιμότητας. Πολλαπλές μελέτες παρατήρησης και διεθνείς βάσεις δεδομένων - και, πρόσφατα, διάφορες μετα-αναλύσεις που αναλύουν ασθενείς που υποβάλλονται σε επεμβάσεις γενικής χειρουργικής, αλλά κυρίως διαφορετικές χειρουργικές επεμβάσεις καρκίνου του πεπτικού συστήματος (καρκίνος παχέος εντέρου, ορθού, στομάχου και ήπατος), σε ορθοπεδική, αγγειακή και καρδιαγγειακή χειρουργική - δείχνουν μια σχέση μεταξύ μετάγγισης και υψηλότερης νοσηρότητας, θνησιμότητας, επανεπέμβασης και επανεισαγωγής. Επιπλέον, η μετάγγιση αίματος έχει επίσης συσχετιστεί με υψηλότερη συχνότητα υποτροπής του όγκου, ανθεκτικότητας στη θεραπεία και θνησιμότητας που σχετίζεται με την κοιλιακή νεοπλασματική νόσο.

Για αυτούς τους λόγους, διαφορετικοί εθνικοί ισπανικοί (π.χ. SEDAR - Sociedad Española de Anestesiología, Reanimación y Terapéutica del Dolor – Ισπανική Εταιρία Αναισθησιολογίας, Αναζωογόνησης και Θεραπείας του Πόνου) και διεθνείς (π.χ. SABM - Society for the Advancement of Blood Management - Εταιρεία για την Προώθηση της Διαχείρισης Αίματος) οργανισμοί, καθώς και το Συμβούλιο της Ευρωπαϊκής Ένωσης Αναισθησιολογίας (Euopean Society of Anesthesiology – ESA)[6] και το Εθνικό Ινστιτούτο Αριστείας της Υγείας και της Φροντίδας (The National Institute for Health and Care Excellence – NICE), συνιστούν την αξιολόγηση και τη θεραπεία της προεγχειρητικής αναιμίας, ακόμη και την καθυστέρηση ή τον επαναπρογραμματισμό της χειρουργικής επέμβασης για ασθενείς με αναιμία. Τόσο οι ισπανικές κατευθυντήριες οδηγίες («Σεβίλλη»)[4] όσο και οι διεθνείς («Φρανκφούρτη»)[5] συνιστούν ισχυρά έλεγχο (screening) και θεραπεία της προεγχειρητικής αναιμίας, με το υψηλότερο επίπεδο τεκμηρίωσης. Αυτή η σύσταση έχει εγκριθεί από το Υπουργείο Υγείας και Κοινωνικής Πρόνοιας της Ισπανίας από το 2013 στην «Δέσμευση για την Ποιότητα των Επιστημονικών Εταιρειών»

Η Πολιτεία της Δυτικής Αυστραλίας και η Αυστραλιανή Εθνική Αρχή Αίματος έχουν προωθήσει μια εκστρατεία για την ενδυνάμωση των ασθενών, ώστε να γνωρίζουν την ανάγκη να είναι σε φόρμα πριν από τη χειρουργική επέμβαση και τη σημασία της μελέτης και θεραπείας της αναιμίας και της ανεπάρκειας σιδήρου από τους γενικούς ιατρούς και ότι κανένας ασθενής δεν πρέπει να προσέρχεται με αναιμία στο χειρουργείο[1,7].

12. Συνιστάται μόλις ένας ασθενής εισαχθεί στη λίστα αναμονής του χειρουργείου ή από τη στιγμή που τίθεται η χειρουργική ένδειξη, να παρακολουθείται, να μελετάται και να αντιμετωπίζεται επαρκώς η πιθανή εμφάνιση αναιμίας ή οποιουδήποτε αιματολογικού ελλείμματος.

(Αυτή η διαχείριση μπορεί να πραγματοποιηθεί από γενικό ιατρό, οικογενειακό γιατρό, παραπομπή σε ειδικό, τη χειρουργική ομάδα ή τον συντονιστή του περιστατικού, ανάλογα με τον τοπικό οργανισμό[1-3,7])

Χαμηλό επίπεδο τεκμηρίωσης. Ασθενής σύσταση.

ΒΙΒΛΙΟΓΡΑΦΙΚΕΣ ΑΝΑΦΟΡΕΣ

1. Fit For Surgery: Managing Iron Deficiency Anaemia. Resources for general practitioners managing patients undergoing planned surgery. Australia National Blood Authority. https:// www.blood.gov.au/fit-surgery-managing-iron-deficiency-anaemia (último acceso febrero 2020).

2. Supporting Patient Blood Management (PBM) in the EU. A Practical Implementation Guide for Hospitals. Directorate-General for Health and Food Safety. European Commission. 2017. https://ec.europa.eu/health/sites/health/files/blood_tissues_organs/docs/2017_ eupbm_hospitals_en.pdf (último acceso febrero 2020).

3. Ripollés-Melchor J, Jericó-Alba C, Quintana-Díaz M, García-Erce JA. From blood saving programs to patient blood management and beyond. Med Clin (Barc). 2018;151(9):368- 373.

4. Actualización del Documento de Sevilla de Alternativas a la Transfusión Sanguínea. Congreso SETS. Madrid, Junio 2019.

5. Mueller MM, Van Remoortel H, Meybohm P, et al. ICC PBM Frankfurt 2018 Group.Patient Blood Management: Recommendations from the 2018 Frankfurt Consens Conference. JAMA. 2019;321:983-97.

6. European Board of Anaesthesiology (EBA) recommendations for Preoperative anaemia and Patient Blood Management. http://www.eba-uems.eu/resources/PDFS/safety-guidelines/EBA--Preop-anaemia-recommend.pdf (último acceso febrero 2020).

7. Minck S, Robinson K, Saxon B, et al. Patient blood management -- the GP’s guide. Aust Fam Physician 2013;42:291-7.

Προγράμματα διαχείρισης αίματος ασθενών (Patient Blood Management – PBM) για τη διαχείριση της αναιμίας

Στο τμήμα μετάγγισης της πέμπτης έκδοσης των Προτύπων της Επιτροπής Διαπίστευσης Μεταγγίσεων (Standards of the Transfusion Accreditation Committee)[1] της Ισπανικής Εταιρείας Αιματολογίας και Αιμοθεραπείας (SEHH – Sociedad Española de Hematología y Hemoterapia) και της Ισπανικής Εταιρείας Μετάγγισης Αίματος και Κυτταροθεραπείας (SETS – Sociedad Española de Transfusión Sanguínea y Terapia Celular), στο σημείο 4.2.5. αναφέρει ότι «Οι Υπηρεσίες Μετάγγισης πρέπει να προωθούν και να συμμετέχουν στα νοσοκομειακά προγράμματα Διαχείρισης Αίματος Ασθενών (PBM)». Στην 63η Συνέλευση του τον Ιούνιο του 2010, ο Παγκόσμιος Οργανισμός Υγείας (ΠΟΥ) προέτρεψε όλες τις χώρες να ξεκινήσουν προγράμματα διαχείρισης αίματος ασθενών[2]. Αυτή η σύσταση εγκρίθηκε από την Ευρωπαϊκή Επιτροπή από το 2013 και επικυρώθηκε από τις Κατευθυντήριες Οδηγίες για τις Συστάσεις Καλής Πρακτικής στη Διαχείριση Αίματος Ασθενών (Guidelines for Good Practice Recommendations in Patient Blood Management) που δημοσιεύθηκαν τον Απρίλιο 2017[3-4]. Θεωρούμε απαραίτητο να ενσωματώσουμε αυτά τα προγράμματα διαχείρισης αίματος ασθενών σε προγράμματα πολυπαραγοντικής πολυτροπικής προαποκαταστάσης[5-7].

13. Συνιστάται η εφαρμογή προγραμμάτων διαχείρισης αίματος ασθενών σε όλα τα νοσοκομεία και τους χώρους υγείας. Προτείνουμε το πρόγραμμα διαχείρισης αίματος ασθενών να ενσωματωθεί με τα πρωτοκόλλα βελτιστοποίησης της μετεγχειρητικής ανάρρωσης.

Υψηλό επίπεδο τεκμηρίωσης. Ισχυρή σύσταση.

ΒΙΒΛΙΟΓΡΑΦΙΚΕΣ ΑΝΑΦΟΡΕΣ

1. Estándares del Comité de Acreditación de Transfusión (CAT). 5ª Edición. Junio 2019 http://www.catransfusion.es/media/upload/arxius/estandares/ESTANDARES %202019. pdf (último acceso febrero 2020).

2. Global Forum for Blood Safety: Patient Blood Management. World Health Organization.

https://www.who.int/bloodsafety/events/gfbs_01_pbm_concept_paper.pdf (último acceso febrero 2020).
3. Supporting Patient Blood Management (PBM) in the EU. A Practical Implementation Guide for Hospitals. Directorate-General for Health and Food Safety. European Commission. 2017. https://ec.europa.eu/health/sites/health/files/blood_tissues_organs/docs/2017_ eupbm_hospitals_en.pdf (último acceso febrero 2020).
4. Building national programmes of Patient Blood Management (PBM) in the EU. A Guide for Health Authorities. European Commission. 2017. https://ec.europa.eu/health/sites/ health/files/blood_tissues_organs/docs/2017_eupbm_authorities_en.pdf (último acceso febrero 2020).
5. Casans Francés R, J. Ripollés Melchor, J.M. Calvo Vecino. Grupo Español de Rehabilitación Multimodal GERM/ERAS-Spain. ¿Es hora de integrar el manejo sanguíneo del paciente en los protocolos de rehabilitación quirúrgica intensificada? Rev Esp Anestesiol Reanim. 2015;62(2):61-3.
6. García Erce JA, Laso Morales MJ. «Patient blood management» en la Vía Clínica de Recuperación Intensificada en Cirugía Abdominal. Cir Esp 2017; 95(9):552-554.
7. Ripollés-Melchor J, Jericó-Alba C, Quintana-Díaz M, García-Erce JA. From blood saving programs to patient blood management and beyond. Med Clin (Barc). 2018;151(9):368- 373.

Καθυστέρηση ή αναβολή της χειρουργικής επέμβασης σε αναιμικούς ασθενείς

Υπάρχει συσχέτιση μεταξύ προεγχειρητικής αναιμίας και κινδύνου μετεγχειρητικής θνησιμότητας, νοσηρότητας, μετεγχειρητικής ποιότητας και αυξημένου κινδύνου μετάγγισης[1]. Η Ισπανική Εταιρεία Αναισθησιολογίας, Αναζωογόνησης και Θεραπείας του Πόνου (SEDAR)[2] μεταξύ των συστάσεων ΜΗΝ ΤΟ ΚΑΝΕΙΣ (DO NOT DO) του Έργου του Υπουργείου Υγείας Δέσμευση για την Ποιότητα των Επιστημονικών Εταιρειών (Commitment to the Quality of Scientific Societies) κάνει αυτή τη σύσταση: να μην προγραμματίζονται ασθενείς με αναιμία[3]. Χρόνια αργότερα, το Διοικητικό Συμβούλιο της

Ευρωπαϊκής Εταιρείας Αναισθησιολογίας (ESA) και της Εταιρείας για την Προώθηση της Διαχείρισης Αίματος (SABM) έκαναν την ίδια σύσταση[4,5].

14. Συνιστάται να μην προγραμματίζονται για εκλεκτική χειρουργική επέμβαση με κίνδυνο αιμορραγίας οι ασθενείς με αναιμία έως ότου πραγματοποιηθεί η κατάλληλη διαγνωστική μελέτη και θεραπεία.

Υψηλό επίπεδο τεκμηρίωσης. Ισχυρή σύσταση.

ΒΙΒΛΙΟΓΡΑΦΙΚΕΣ ΑΝΑΦΟΡΕΣ

1. García Erce JA, Laso Morales MJ. «Patient blood management» en la Vía Clínica de Recuperación Intensificada en Cirugía Abdominal. Cirugía Española 2017;95(9):552-554.

2. Quecedo Gutiérrez L, Ruiz Abascal R, Calvo Vecino JM, Peral García AI, Matute González E, Muñoz Alameda LE, et al. “Do not do” recommendations of the Spanish Society of Anaesthesiology, Critical Care and Pain Therapy. “Commitment to Quality by Scientific Societies” Project. Rev Esp Anestesiol Reanim. 2016;63(9):519-527. doi: 10.1016/j.re- dar.2016.05.002.

3. Recomendaciones de«nohacer» dela Sociedad Española de Anestesiología, Reanimación y Terapéutica del Dolor. Proyecto «compromiso por la calidad de las sociedades científicas». https://www.mscbs.gob.es/organizacion/sns/planCalidadSNS/pdf/SOCIEDAD_ESP_ ANEST_REA_TE_DOLOR_0K.pdf (último acceso junio 2020).

4. Burns CD, Brown JP, Corwin HL, Gross I, Ozawa SJ, Shander A. Special Report From the Society for the Advancement of Blood Management: The Choosing Wisely Campaign. Anesth Analg. 2019;129:1381-1386. doi: 10.1213/ANE.0000000000004415. PMID: 31517679.

5. Choosing wisely. Society for the Advancement of Blood Management. “Don’t proceed with elective surgery in patients with properly diagnosed and correctable anemia until the anemia has been appropriately treated”. July 23, 2018. http://www.choosingwisely.org/socie-

Προσδιορισμός του επιπέδου της αιμοσφαιρίνης με επαρκές χρονικό πλαίσιο

Ένα χαμηλό επίπεδο αιμοσφαιρίνης σχετίζεται με αύξηση της περιεγχειρητικής νοσηρότητας και θνησιμότητας καθώς και με τον κίνδυνο λήψης αλλογενούς μετάγγισης[1-6]. Η μελέτη και η θεραπεία της προεγχειρητικής αναιμίας είναι ουσιαστικής σημασίας για τη βελτιστοποίηση των κλινικών αποτελεσμάτων των ασθενών που έχουν προγραμματιστεί για καρδιακή[3] και μη καρδιακή[6] χειρουργική επέμβαση. Συνιστάται η ανίχνευση και θεραπεία της προεγχειρητικής αναιμίας, με επαρκή χρόνο για την επαρκή μελέτη και αντιμετώπισή της[2-7].

15. Συνιστάται τουλάχιστον ένας προσδιορισμός της αιμοσφαιρίνης (Hb) σε ασθενείς που υποβάλλονται σε εκλεκτική χειρουργική επέμβαση, τουλάχιστον 28 ημέρες πριν από την επέμβαση ή την επεμβατική διαδικασία[2,4-7] (ιδανικά μεταξύ 6-8 εβδομάδων ή από τη στιγμή της χειρουργικής ένδειξης).

Μέτριο επίπεδο τεκμηρίωσης. Ισχυρή σύσταση.

16. Συνιστάται σε περιπτώσεις ογκολογικής χειρουργικής επέμβασης, ολόκληρος ο διαθέσιμος χρόνος από τη διάγνωση έως τη στιγμή της επέμβασης να χρησιμοποιείται για την ανίχνευση της αναιμίας και τη διόρθωσή της ή τουλάχιστον τη βελτίωση της συγκέντρωσης της αιμοσφαιρίνης[5,6].

Μέτριο επίπεδο τεκμηρίωσης. Ισχυρή σύσταση.

ΒΙΒΛΙΟΓΡΑΦΙΚΕΣ ΑΝΑΦΟΡΕΣ

1. Fowler AJ, Ahmad T, Phull MK, Allard S, Gillies MA, Pearse RM. Meta-analysis of the association between pre-operative anaemia and mortality after surgery. British Journal of Surgery 2015; 102: 1314-24.

2. Muñoz M, Gómez-Ramírez S, Kozek-Langeneker S, Shander A, Richards T, Pavía J, etal. Fit to fly: overcoming barriers to preoperative haemoglobin optimization in surgical patients. Br J Anaesth. 2015;115:15-24. doi: 10.1093/bja/aev165

3. Klein AA, Collier TJ, Brar MS, Evans C, Hallward G, Fletcher SN, Richards T; Association of Cardiothoracic Anaesthetists (ACTA). The incidence and importance of anaemia in patients undergoing cardiac surgery in the UK-the

first Association of Cardiothoracic Anaesthetists national audit. Anaesthesia 2016;71:627-35.
4. Muñoz M, Acheson AG, Auerbach M, Besser M, Habler O, Kehlet H, et al. International consensus statement on the perioperative management of anaemia and iron deficiency. Anaesthesia. 2017;72:233-47.
5. Gustafsson UO, Scott MJ, Hubner M, Nygren J, Demartines N, Francis N, et al. Guidelines for Perioperative Care in Elective Colorectal Surgery: Enhanced Recovery After Surgery (ERAS®) Society Recommendations: 2018. World J Surg. 2019;43:659-95.
6. De Hert S, Staender S, Fritsch G, Hinkelbein J, Afshari A, Bettelli G, et al. Pre-operative evaluation of adults undergoing elective noncardiac surgery: Updated guideline from the European Society of Anaesthesiology. Eur J Anaesthesiol. 2018;35:407-65.
7. Quecedo Gutiérrez L, Ruiz Abascal R, Calvo Vecino JM, Peral García AI, Matute González E, Muñoz Alameda LE, et al. “Do not do” recommendations of the Spanish Society of Anaesthesiology, Critical Care and Pain Therapy. “Commitment to Quality by Scientific Societies” Project. Rev Esp Anestesiol Reanim. 2016;63:519-527. doi: 10.1016/j.redar.2016.05.002.

Επίπεδο αιμοσφαιρίνης 13 g/dl

Το 2004 και το 2006, οι Guralnik και Beutler πρότειναν να αυξηθεί το ελάχιστο επίπεδο αιμοσφαιρίνης (Hb) σε > 12,2 g/dl σε γυναίκες αναπαραγωγικής ηλικίας, σε > 13,7 g/dl σε Καυκάσιους άρρενες ως 60 ετών και σε > 13,2 g/dl σε μεγαλύτερης ηλικίας Καυκάσιους άρρενες[1]. Άλλοι συγγραφείς προτείνουν την αύξηση του στα 13,5 g/dl σε άνδρες και γυναίκες μετά την εμμηνόπαυση[1]. Μια πρόσφατη εθνική επιδημιολογική μελέτη στον μετακινούμενο πληθυσμό δείχνει ότι τα μέσα επίπεδα αιμοσφαιρίνης των γυναικών ηλικίας 50 ετών αυξάνονται σε τιμές κοντά σε εκείνα των ανδρών ηλικίας 70 ετών[2].

Πρόσφατες μελέτες έχουν δείξει ότι η κατάσταση του μεταβολισμού του σιδήρου σε γυναίκες με αιμοσφαιρίνη μεταξύ 12 και 13 g/dl μοιάζει περισσότερο με εκείνες που θεωρούνται αναιμικές (Hb <12 g/dl) παρά σε εκείνες με αιμοσφαιρίνη μεγαλύτερη από 13 g/dl[3,4].

Επιδημιολογικές μελέτες δείχνουν σχεδόν διπλάσιο κίνδυνο μετάγγισης και επιπλοκών σε γυναίκες χωρίς αναιμία αλλά με επίπεδο Hb μεταξύ 12 και 13 g/dl[3,5]. Οι γυναίκες με επίπεδο αιμοσφαιρίνης κάτω από 13 g/dl μπορεί να συνεχίσουν να υφίστανται διακρίσεις σε σύγκριση με τους άνδρες.

Τα διάφορα έγγραφα συναίνεσης (consensus statements) συνιστούν την αύξηση της τιμής της αιμοσφαιρίνης των γυναικών στα 13 g/dl[6,7].

17. Συνιστάται η προεγχειρητική συγκέντρωση αιμοσφαιρίνης πριν από τη χειρουργική επέμβαση να είναι πάνω από 13 g/dl, ανεξάρτητα από το φύλο.

Μέτριο επίπεδο τεκμηρίωσης. Ισχυρή σύσταση.

ΒΙΒΛΙΟΓΡΑΦΙΚΕΣ ΑΝΑΦΟΡΕΣ

1. Rivilla Marugán L, Lorente Aznar T, Molinero Rodriguez M, García-Erce JA. [Anaemia and the elderly: Critical review of its definition and prevalence]. Rev Esp Geriatr Gerontol. 2019;54(4):189-194. doi: 10.1016/j.regg.2019.02.008.

2. García-Erce JA, Lorente-Aznar T, Rivilla-Marugán L. Influence of gender, age and residence altitude on haemoglobin levels and the prevalence of anaemia. Med Clin (Barc). 2019. pii: S0025-7753(19)30125-3. doi: 10.1016/j.medcli.2019.02.002.

3. Butcher A, Richards T, Stanworth SJ, Klein AA. Diagnostic criteria for preoperative anaemia - time to end sex discrimination. Anaesthesia. 2017;72:811-4.

4. Muñoz M, Laso-Morales MJ, Gómez-Ramírez S, Cadellas M, Núñez-Matas MJ, García-Erce JA., Pre-operative haemoglobin levels and iron status in a large multicentre cohort of patients undergoing major elective surgery. Anaesthesia. 2017;72:826-34.

5. Muñoz M, Gómez-Ramírez S, Auerbach M. Stimulating erythropoiesis before hip fracture repair for reducing blood transfusion: should we change the hemoglobin cutoff level for defining anemia in females? Transfusion. 2016;56:2160-3.

6. Muñoz M, Acheson AG, Bisbe E, Butcher A, Gómez-Ramírez S, Khalafallah AA, et al. An international consensus statement on the management of postoperative anaemia after major surgical procedures. Anaesthesia. 2018;73:1418-1431. doi: 10.1111/anae. 14358.7.-Documento consenso

GERM Salamanca (Fit for Surgery. Documento de trabajo. Estudio Delphi. Grupo Español de Rehabilitación Multimodal. III Congreso GERM. Salamanca, abril 2018) (pendiente publicación).

Έλεγχος (screening) για έλλειψη σιδήρου

Η ανεπάρκεια σιδήρου είναι η πιο κοινή αιτιολογία της αναιμίας[1-4]. Είναι η συχνότερη αιτία προεγχειρητικής αναιμίας[1,2]. Έως και το ένα τρίτο των ασθενών χωρίς εμφανή αναιμία έχουν ανεπάρκεια σιδήρου και ένα άλλο τρίτο των ασθενών έχουν επαρκή αποθέματα για να αναρρώσουν μετά από περιεγχειρητική αιμορραγία[1,2]. Επιπλέον, η ανεπάρκεια σιδήρου έχει συσχετιστεί με υψηλότερο κίνδυνο μετάγγισης ή/και νοσοκομειακής λοίμωξης σε χειρουργικές επεμβάσεις αποκατάστασης για κάταγμα ισχίου[5], καρδιοχειρουργικές επεμβάσεις ή εκτομή καρκίνου του παχέος εντέρου[6].

Το Εθνικό Ινστιτούτο Αριστείας της Υγείας και της Φροντίδας (NICE), στα πρότυπα ποιότητας του για μετάγγιση αίματος [QS138], συνιστά την προσφορά συμπληρωμάτων σιδήρου, πριν και μετά την επέμβαση, σε όλους τους ασθενείς με σιδηροπενική αναιμία[7-8]. Είναι επίσης απαραίτητο να εξεταστεί η πιθανότητα παρεντερικής θεραπείας σε περιπτώσεις από του στόματος δυσανεξίας.

18. Συνιστάται η ανίχνευση και θεραπεία της περιεγχειρητικής ανεπάρκειας σιδήρου.

Μέτριο επίπεδο τεκμηρίωσης. Ισχυρή σύσταση.

ΒΙΒΛΙΟΓΡΑΦΙΚΕΣ ΑΝΑΦΟΡΕΣ

1. Muñoz M, Laso-Morales MJ, Gómez-Ramírez S, Cladellas M, Núñez-Matas MJ, García-Erce JA. Pre-operative haemoglobin levels and iron status in a large multicentre cohort of patients undergoing major elective surgery. Anaesthesia 2017;72:826-834. doi: 10.1111/ anae.13840.
2. Muñoz M, Gómez-Ramírez S, Besser M, Pavía J, Gomollón F, Liumbruno GM, et al. Current misconceptions in diagnosis and management of iron deficiency. Blood Transfus 2017;15:422-437. doi: 10.2450/2017.0113-17.

3. De Hert S, Staender S, Fritsch G, Hinkelbein J, Afshari A, Bettelli G, et al. Pre-operative evaluation of adults undergoing elective non-cardiac surgery: Updated guideline from the European Society of Anaesthesiology. Eur J Anaesthesiol. 2018;35:407-65.
4. Althoff FC, Neb H, Herrmann E, Trentino KM, Vernich L, Füllenbach C, at al. Multimodal patient blood management program based on a three-pillar strategy: a systematic review and meta-analysis. Ann Surg 2019;269:794-804.
5. Izuel Rami M, García Erce JA, Gómez-Barrera M, Cuenca Espiérrez J, Abad Sazatornil R, Rabanaque Hernández MJ. Relación entrelatransfusión desangrealogénica, ladeficiencia de hierro y la infección nosocomial en pacientes con fractura de cadera. Med Clin (Barc). 2008;131:647-52.
6. Rössler J, Schoenrath F, Seifert B, Kaserer A, Spahn GH, Falk V, Spahn DR. Iron deficiency is associated with higher mortality in patients undergoing cardiac surgery: a prospective study. Br J Anaesth. 2020;124:25-34. doi: 10.1016/j.bja.2019.09.016.
7. Harju E. Empty iron stores as a significant risk factor in abdominal surgery. JPEN J Parenter Enteral Nutr. 1988;12:282-5. doi: 10.1177/0148607188012003282. PMID: 3392823.
8. "People with iron-deficiency anaemia who are having surgery are offered iron supplementation before and after surgery". Blood transfusion. Quality standard [QS138] Published date: December 2016 https://www.nice.org.uk/guidance/qs138 https://www.nice.org.

Θεραπεία της προεγχειρητικής αναιμίας

Η μελέτη της αναιμίας και η ειδική θεραπεία οποιασδήποτε προεγχειρητικής αναιμίας θα πρέπει να πραγματοποιηθεί το συντομότερο δυνατό[1-5].

Η άμεση περιεγχειρητική θεραπεία της αναιμίας σε ορθοπεδικούς[4,5] ή καρδιοχειρουργικούς ασθενείς, όχι μόνο σχετίζεται με χαμηλότερο ποσοστό μετάγγισης αλλά και με χαμηλότερη συχνότητα ανεπιθύμητων συμβαμάτων και μείωση της νοσηλείας στο νοσοκομείο, επιπλέον των καλύτερων μετεγχειρητικών αιματολογικών παραμέτρων.

19. Συνιστάται η ανίχνευση και αντιμετώπιση της προεγχειρητικής αναιμίας, ακόμη και σε περιπτώσεις επισπευσμένων ή επειγουσών χειρουργικών επεμβάσεων.

Μέτριο επίπεδο τεκμηρίωσης. Ισχυρή σύσταση.

ΒΙΒΛΙΟΓΡΑΦΙΚΕΣ ΑΝΑΦΟΡΕΣ

1. Kotzé A, Harris A, Baker C, Iqbal T, Lavies N, Richards T, et al. British Committee for Stan- dards in Haematology Guidelines on the Identification and Management of Pre-Operative Anaemia. Br J Haematol. 2015;171:322-31. doi: 10.1111/bjh.13623.

2. Muñoz M, Gómez-Ramírez S, Kozek-Langeneker S, Shander A, Richards T, Pavía J, et al. 'Fit to fly': overcoming barriers to preoperative haemoglobin optimization in surgical patients. Br J Anaesth 2015;115:15-24.

3. Muñoz M, Gómez-Ramírez S, Campos A, Ruiz J, Liumbruno GM. Pre-operative anaemia: prevalence, consequences and approaches to management. Blood Transfus. 2015;13:370- 9.

4. Gómez-Ramírez S, Maldonado-Ruiz MÁ, Campos-Garrigues A, Herrera A, Muñoz M. Short-term perioperative iron in major orthopedic surgery: state of the art. Vox Sang 2019;114:3- 16.

5. Muñoz M, Gómez-Ramírez S, Cuenca J, García-Erce JA, Iglesias-Aparicio D, Haman-Alcober S, et al. Very-short-term perioperative intravenous iron administration and postoperative outcome in major orthopedic surgery: a pooled analysis of observational data from 2547 patients. Transfusion. 2014;54:289-99.

6. Spahn D, Schoenrath F, Spahn GH, Seifert B, Stein P, Theusinger OM, et al. Effect of ultra-short-term treatment of patients with iron deficiency or anaemia undergoing cardiac surgery: a prospective randomised trial. Lancet. 2019;393:2201-12.

7. Weltert L, Rondinelli B, Bello R, Falco M, Bellisario A, Maselli D, et al. A single dose of erythropoietin reduces perioperative transfusions in cardiac surgery: results of a prospective single-blind randomized controlled trial. Transfusion. 2015;55:1644-54.

8. Yoo YC, Shim JK, Kim JC, Jo YY, Lee JH, Kwak YL. Effect of single recombinant human erythropoietin injection on transfusion requirements in

preoperatively anemic patients undergoing valvular heart surgery. Anesthesiology 2011;115: 929-37.

Θεραπεία σιδήρου από το στόμα

Η θεραπεία εκλογής για τη σιδηροπενία και την ήπια αναιμία είναι ο συμβατικός από του στόματος σίδηρος εάν υπάρχει επαρκής διαθέσιμος χρόνος και δεν υπάρχει αντένδειξη. Συνιστάται η χορήγηση χαμηλών ημερήσιων δόσεων (40–60 mg) ή μέτριων δόσεων κάθε δεύτερη μέρα (80–100 mg). Δεν υπάρχουν ενδείξεις ότι οι υψηλότερες δόσεις οδηγούν σε μεγαλύτερη απορρόφηση, ενώ σχετίζονται επίσης με υψηλότερο ποσοστό ανεπιθύμητων ενεργειών από το πεπτικό σύστημα[1-7].

20. Η από του στόματος θεραπεία σιδήρου συνιστάται σε περιπτώσεις σιδηροπενίας ή ήπιας-μέτριας σιδηροπενικής αναιμίας εάν μένουν τουλάχιστον 6 εβδομάδες μέχρι την επέμβαση.

Χαμηλό επίπεδο τεκμηρίωσης. Ισχυρή σύσταση.

ΒΙΒΛΙΟΓΡΑΦΙΚΕΣ ΑΝΑΦΟΡΕΣ

1. Muñoz M, Acheson AG, Bisbe E, Butcher A, Gómez-Ramírez S, Khalafallah AA, et al. An international consensus statement on the management of postoperative anaemia after major surgical procedures. Anaesthesia. 2018;73:1418-1431. doi: 10.1111/anae. 14358.

2. Stoffel NU, Cercamondi CI, Brittenham G, Zeder C, Geurts-Moespot AJ, Swinkels DW, et al. Iron absorption from oral iron supplements given on consecutive versus alternate days and as single morning doses versus twice-daily split dosing in iron-depleted women: two open- label, randomised controlled trials. Lancet Haematol. 2017; e524-e533. doi: 10.1016/ S2352-3026(17)30182-5.

3. Moretti D, Goede JS, Zeder C, Jiskra M, Chatzinakou V, Tjalsma H, et al. Oral iron supplements increase hepcidin and decrease iron absorption from daily or twice-daily doses in iron-depleted young women. Blood. 2015;126:1981-9.

4. Muñoz M, Acheson AG, Auerbach M, Besser M, Habler O, Kehlet H, et al. International consensus statement on the perioperative management of anaemia and iron deficiency. Anaesthesia. 2017;72:233-47.
5. Muñoz M, Gómez-Ramírez S, Besser M, Pavía J, Gomollón F, et al. Current misconceptions in diagnosis and management of iron deficiency. Blood Transfus. 2017;15:422-37.
6. Jericó Alba C, Garcia Erce JA. Hiero oral como tratamiento de la ferropenia: ¿debe ser siempre la primera elección? Med Clin (Barc). 2018;151:e27-e28.
7. García Erce JA, Altés A, López Rubio M, Remacha AF; en representación del Grupo Español de Eritropatología de la Sociedad Española de Hematología y Hemoterapia. Management of iron deficiency in various clinical conditions and the role of intravenous iron: Recommendations of the Spanish Erythropathology Group of the Spanish Society of Haematology and Haemotherapy. Rev Clin Esp. 2020;220:31-42.

Ενδοφλέβια θεραπεία σιδήρου

Εάν υπάρχει λίγος χρόνος πριν από τη χειρουργική επέμβαση, αντίσταση ή δυσανεξία στο σίδηρο από το στόμα, αντένδειξη στην χορήγηση του, παρουσία φλεγμονής ή μέτρια-σοβαρή αναιμία ή ταυτόχρονη θεραπεία με ερυθροποιητικούς παράγοντες, η θεραπεία εκλογής για τη σιδηροπενία και την αναιμία που σχετίζεται με ανεπάρκεια σιδήρου είναι η ενδοφλέβια χορήγηση σιδήρου σε υψηλές δόσεις[1-8].

21. Προεγχειρητική θεραπεία με ενδοφλέβιο σίδηρο συνιστάται σε ασθενείς με εκλεκτική χειρουργική επέμβαση με πιθανή αιμορραγία που πάσχουν από σιδηροπενική αναιμία ή/και λειτουργική ανεπάρκεια σιδήρου, για βελτίωση των επιπέδων αιμοσφαιρίνης ή/και μείωση του ρυθμού μετάγγισης.

Μέτριο επίπεδο τεκμηρίωσης. Ισχυρή σύσταση.

22. Συνιστούμε την ενδοφλέβια χορήγηση σιδήρου, αντί σιδήρου από του στόματος, σε εκείνες τις περιπτώσεις που αυτό αντενδείκνυται ή που ο διαθέσιμος χρόνος μέχρι το χειρουργείο είναι ανεπαρκής.

Μέτριο επίπεδο τεκμηρίωσης. Ισχυρή σύσταση.

ΒΙΒΛΙΟΓΡΑΦΙΚΕΣ ΑΝΑΦΟΡΕΣ

1. Banerjee S, McCormack S. Intravenous iron preparations for patients undergoing elective surgery: a review of clinical effectiveness, cost-effectiveness, and guidelines. Ottawa: CADTH; 2019 Mar. (CADTH rapid response report: summary with critical appraisal). https://www.cadth.ca/sites/default/files/pdf/htis/2019/RC1088%20Intravenous%20 Iron%20Final.pdf (último acceso febrero 2020).

2. Calleja JL, Delgado S, del Val A, Hervás A, Larraona JL, Terán Á, et al; Colon Cancer Study Group. Ferric carboxymaltose reduces transfusions and hospital stay in patients with colon cancer and anemia. Int J Colorectal Dis. 2016;31:543-551.

3. Froessler B, Palm P,Weber I, Hodyl NA, Singh R, Murphy EM. The Important Role for Intravenous Iron in Perioperative Patient Blood Management in Major Abdominal Surgery: A Randomized Controlled Trial. Ann Surg. 2016;264:41-6.

4. García Erce JA, Altés A, López Rubio M, Remacha AF; en representación del Grupo Español de Eritropatología de la Sociedad Española de Hematología y Hemoterapia. Management of iron deficiency in various clinical conditions and the role of intravenous iron: Recommendations of the Spanish Erythropathology Group of the Spanish Society of Haematology and Haemotherapy. Rev Clin Esp. 2020;220:31-42. doi: 10.1016/j. rce.2019.09.004

5. Keeler BD, Dickson EA, Simpson JA, Ng O, Padmanabhan H, Brookes MJ, et al; IVICA Trial Group. The impact of pre-operative intravenous iron on quality of life after colorectal cancer surgery: outcomes from the intravenous iron in colorectal cancer-associated anaemia (IVICA) trial. Anaesthesia. 2019;74:714-725.

6. Laso-Morales M, Jericó C, Gómez-Ramírez S, Castellví J, Viso L, Roig-Martínez I, et al. Preoperative management of colorectal cancer-induced iron deficiency anemia in clinical practice: data from a large observational cohort. Transfusion. 2017;57:3040-3048. doi: 10.1111/trf.14278.

7. Schack A, Berkfors AA, Ekeloef S, Gögenur I, Burcharth J. The Effect of Perioperative Iron Therapy in Acute Major Non-cardiac Surgery on Allogenic Blood Transfusion and Postoperative Haemoglobin Levels: A Systematic Review and Meta-analysis. World J Surg. 2019;43:1677-1691. doi: 10.1007/s00268-019-04971-7. PMID: 30824959

8. Blood transfusion. Quality standard [QS138] Published date: December 2016 https:// www.nice.org.uk/guidance/qs138 (último acceso febrero 2020).

Θεραπεία με ερυθροποιητικούς παράγοντες

Η χορήγηση άλφα-ερυθροποιητίνης συνιστάται για τη θεραπεία της μη σιδηροπενικής αναιμίας προεγχειρητικά μετά από ορθοπεδικό χειρουργείο[1]. Διαφορετικές μελέτες καταδεικνύουν το όφελος των σύντομων σχημάτων ή ακόμη και των εφάπαξ δόσεων σε χειρουργικές επεμβάσεις ορθοπεδικής αρθροπλαστικής και χειρουργικής κατάγματος ισχίου[2,3]. Μια πρόσφατη ελβετική μελέτη καταδεικνύει το όφελος, σε συνδυασμό με ενδοφλέβιο σίδηρο και σύμπλοκα βιταμινών, στις καρδιοχειρουργικές επεμβάσεις[4].

Άλλες μετα-αναλύσεις, έγγραφα συναίνεσης (consensus) και κατευθυντήριες οδηγίες από επιστημονικούς οργανισμούς συνιστούν τη χορήγηση ερυθροποιητικών παραγόντων, μαζί με ενδοφλέβιο σίδηρο, σε χειρουργικούς ασθενείς με μη σιδηροπενική αναιμία[5-8].

23. Για τη μείωση της αλλογενούς μετάγγισης αίματος, η χορήγηση ανασυνδυασμένης ανθρώπινης ερυθροποιητίνης (rHuEPO) συνιστάται σε ασθενείς με εκλεκτική ορθοπεδική χειρουργική επέμβαση με κίνδυνο μέτριας-υψηλής αιμορραγίας και μέτριας μη σιδηροπενικής αναιμίας (Hb μεταξύ 10 και 13 g/dl).

Υψηλό επίπεδο τεκμηρίωσης. Ισχυρή σύσταση.

24. Η χορήγηση rHuEPO προτείνεται για τη μείωση του ποσοστού μετάγγισης σε αναιμικούς ασθενείς που υποβάλλονται και σε άλλη μείζονα εκλεκτική χειρουργική επέμβαση πέρα από την εκλεκτική ορθοπεδική χειρουργική επέμβαση με μέτριο-υψηλό κίνδυνο αιμορραγίας.

Μέτριο επίπεδο τεκμηρίωσης. Ασθενής σύσταση.

ΒΙΒΛΙΟΓΡΑΦΙΚΕΣ ΑΝΑΦΟΡΕΣ

1. Kei T, Mistry N, Curley G, et al. Efficacy and safety of erythropoietin and iron therapy to reduce red blood cell transfusion in surgical patients: a systematic

review and meta-analysis, Can J Anesth 2019. doi.org/10.1007/s12630-019-01351-6.
2. Theusinger OM, Kind SL, Seifert B, Borgeat L, Gerber C, Spahn DR. Patient blood management in orthopaedic surgery: a four-year follow-up of transfusion requirements and blood loss from 2008 to 2011 at the Balgrist University Hospital in Zurich, Switzerland. Blood Transfus. 2014;12:195-203.
3. Muñoz M, Gómez-Ramírez S, Cuenca J, García-Erce JA, Iglesias-Aparicio D, Haman-Alcober S, et al. Very-short-term perioperative intravenous iron administration and postoperative outcome in major orthopedic surgery: a pooled analysis of observational data from 2547 patients. Transfusion. 2014;54:289-99. doi: 10.1111/trf.12195.
4. Spahn D, Schoenrath F, Spahn GH, Seifert B, Stein P, Theusinger OM, et al. Effect of ultra-short-term treatment of patients with iron deficiency or anaemia undergoing cardiac surgery: a prospective randomised trial. Lancet. 2019;393:2201-12.
5. French CJ, Glassford NJ, Gantner D, Higgins AM, Cooper DJ, Nichol A, et al. Erythropoiesis-stimulating agents in critically ill trauma patients: a systematic review and meta-analysis. Ann Surg. 2017;265:54-62.
6. De Hert S, Staender S, Fritsch G, Hinkelbein J, Afshari A, Bettelli G, et al. Pre-operative evaluation of adults undergoing elective noncardiac surgery: Updated guideline from the European Society of Anaesthesiology. Eur J Anaesthesiol. 2018;35:407-65.
7. Task Force on Patient Blood Management for Adult Cardiac Surgery of the European Association for Cardio-Thoracic Surgery (EACTS) and the European Association of Cardiothoracic Anaesthesiology (EACTA). Boer C, Meesters MI, Milojevic M, Benedetto U, Bolliger D, von Heymann C, et al. 2017 EACTS/EACTA Guidelines on patient blood management for adult cardiac surgery. J Cardiothorac Vasc Anesth. 2018;32:88-120.
8. Documento Consenso GERM Salamanca (Fit for Surgery. Documento de trabajo. Estudio Delphi. Grupo Español de Rehabilitación Multimodal. III Congreso GERM. Salamanca, abril 2018) (Documento Salamanca Abril 2018).

Θρομβοπροφύλαξη

Η θρομβοεμβολική νόσος είναι μια σημαντική επιπλοκή των μεγάλων χειρουργικών επεμβάσεων σε ασθενείς που δεν λαμβάνουν προφύλαξη, επηρεάζοντας το 20% αυτών που υποβάλλονται σε επέμβαση γενικής χειρουργικής, το 30% σε χειρουργική επέμβαση παχέος εντέρου, μεταξύ 30-50% σε ορθοπεδική επέμβαση, κάταγμα ισχίου και νευροχειρουργική επέμβαση και μεταξύ 0-26% σε χειρουργικές επεμβάσεις κεφαλής και τραχήλου. Η εφαρμογή διαφορετικών μέτρων θρομβοπροφύλαξης έχει δείξει μείωση του θρομβωτικού κινδύνου, με διαφορετικό βαθμό αποτελεσματικότητας και ασφάλειας. Ανάλογα με τον θρομβωτικό κίνδυνο, μπορούν να συνδυαστούν μηχανικές και φαρμακολογικές, υποδόριες ή από του στόματος μέθοδοι[1-5]. Η ελάχιστη διάρκεια πρέπει να είναι 7 ημέρες ή μέχρι την έναρξη της βάδισης. Σε χειρουργικές επεμβάσεις μεγαλύτερου κινδύνου, η προφύλαξη θα πρέπει να διαρκεί από 3 έως 6 εβδομάδες.

Η φαρμακολογική προφύλαξη μειώνει σημαντικά τη συχνότητα εμφάνισης θρομβοεμβολικής νόσου. Η μη κλασματοποιημένη ηπαρίνη (ΜΚΗ – unfractionated heparin UFH) και οι ηπαρίνες χαμηλού μοριακού βάρους (ΗΧΜΒ – low molecular weight heparin - LMWH) είναι εξίσου αποτελεσματικές για την πρόληψη της εν τω βάθει φλεβικής θρόμβωσης και της πνευμονικής θρομβοεμβολής, αν και η χρήση της ΗΧΜΒ προτιμάται έναντι της ΜΚΗ μετεγχειρητικά στις περισσότερες από τις χειρουργικές ενδείξεις, λόγω παρόμοιου αποτελέσματος, αλλά μεγαλύτερης ευκολίας χορήγησης και λιγότερων αιμορραγικών επιπλοκών.

25. Η χρήση θρομβοπροφύλαξης συνιστάται σε όλους τους ασθενείς που υποβάλλονται σε μείζονα χειρουργική επέμβαση ή νοσηλεύονται για οξεία ιατρική κατάσταση.

Μέτριο επίπεδο τεκμηρίωσης. Ισχυρή σύσταση.

26. Γενικά, συνιστάται η διατήρηση της αντιθρομβωτικής προφύλαξης για τουλάχιστον 7 ημέρες ή έως ότου ο ασθενής είναι περιπατητικός.

Υψηλό επίπεδο τεκμηρίωσης. Ισχυρή σύσταση.

27. Σε περίπτωση μείζονος χειρουργικής επέμβασης στην κοιλία, η προφύλαξη θα παραταθεί έως και 4 εβδομάδες μετά την επέμβαση.

Μέτριο επίπεδο τεκμηρίωσης. Ισχυρή σύσταση.

Συγκεκριμένες καταστάσεις:

1) Ουρολογικές, γυναικολογικές, νευροχειρουργικές επεμβάσεις και επεμβάσεις γενικής χειρουργικής: 8 ημέρες. Εάν ο ασθενής είναι ακινητοποιημένος, θα πρέπει να παραταθεί μέχρι την κινητοποίησή του.

2) Ουρολογικές, γυναικολογικές και επεμβάσεις γενικής χειρουργικής σε καρκινοπαθείς: 4 εβδομάδες (28 ημέρες)

3) Σε χειρουργική επέμβαση ισχίου: 4–6 εβδομάδες (28–42 ημέρες)

4) Σε χειρουργική επέμβαση γόνατος: 3–4 εβδομάδες (21–28 ημέρες)

28. Συνιστάται η πρώιμη κινητοποίηση και η χρήση ελαστικών καλτσών συμπίεσης για τη διάρκεια της περιόδου ακινητοποίησης.

Υψηλό επίπεδο τεκμηρίωσης. Ισχυρή σύσταση.

29. Οι κάλτσες συμπίεσης είναι αποτελεσματικές στην πρόληψη της θρομβοεμβολικής νόσου σε χειρουργικούς ασθενείς, μειώνοντας ακόμη περισσότερο τον κίνδυνο όταν συνδυάζονται με φαρμακολογικούς παράγοντες.

Υψηλό επίπεδο τεκμηρίωσης. Ισχυρή σύσταση.

30. Οι συσκευές διαλείπουσας πνευματικής συμπίεσης μειώνουν τη συχνότητα της εν τω βάθει φλεβικής θρόμβωσης. Η μέθοδος σε συνδυασμό με φαρμακολογικά μέτρα συνιστάται κυρίως για νευροχειρουργικούς ασθενείς ή/και χειρουργεία με υψηλό κίνδυνο ΦΘΕ.

Μέτριο επίπεδο τεκμηρίωσης. Ισχυρή σύσταση.

31. Τα προληπτικά σχήματα περιλαμβάνουν τα νέα από του στόματος άμεσης δράσης αντιπηκτικά (dabigatran - δαβιγατράνη, apixaban - απιξαμπάνη, rivaroxaban- ριβαροξαβάνη) ή ΗΧΜΒ (enoxaparin - ενοξαπαρίνη, bemiparin - μπεμιπαρίνη, tinzaparin - τινζαπαρίνη).

Υψηλό επίπεδο τεκμηρίωσης. Ισχυρή σύσταση.

ΒΙΒΛΙΟΓΡΑΦΙΚΕΣ ΑΝΑΦΟΡΕΣ

1. Felder S, Rasmussen MS, King R, Sklow B, Kwaan M, Madoff R, et al. Prolonged thromboprophylaxis with low molecular weight heparin for abdominal or pelvic surgery. Cochrane Database Syst Rev. 2019;3:CD004318. doi: 10.1002/14651858.CD004318.

2. Vivas D, Roldán I, Ferrandis R, Marín F, Roldán V, Tello-Montoliu A, et al. Perioperative and Periprocedural Management of Antithrombotic Therapy: Consensus Document of SEC, SEDAR, SEACV, SECTCV, AEC, SECPRE, SEPD, SEGO, SEHH, SETH, SEMERGEN, SEMFYC, SEMG, SEMICYUC, SEMI, SEMES, SEPAR, SENEC, SEO, SEPA, SERVEI, SECOT and AEU. Rev Esp Cardiol. 2018; 71:553-564.

3. Falck-Ytter Y, Francis CW, Johanson NA, Curley C, Dahl OE, Schulman S, et al: Antithrombotic Therapy and Prevention of Thrombosis, 9th ed: American College of Chest Physicians Evidence-Based Clinical Practice Guidelines. Chest. 2012 Feb;141(2 Suppl):e278S- e325S.

4. Anderson DR, Morgano GP, Bennett C, Dentali F, Francis CW, Garcia DA, et al American Society of Hematology 2019 guidelines for management of venous thromboembolism: prevention of venous thromboembolism in surgical hospitalized patients. Blood Adv. 2019;3: 3898-3944.

5. Afshari A, Fenger-Eriksen C, Monreal M, Verhamme P; ESA VTE Guidelines Task Force. European guidelines on perioperative venous thromboembolism prophylaxis: Mechanical prophylaxis. Eur J Anaesthesiol. 2018; 35:112-11.

Υγιεινή – Λουτρό

Σύμφωνα με την πρώτη έκδοση του Via RICA, το λουτρό το βράδυ πριν από την επέμβαση έχει αποδειχθεί αποτελεσματικό στην πρόληψη της λοίμωξης του χειρουργικού πεδίου.

Η σημασία του μπάνιου ή του ντους το βράδυ πριν την επέμβαση είναι αποδεκτό γεγονός, όπως και η μείωση του αριθμού των βακτηριακών αποικιών λόγω του λουτρού[1-3]. Ωστόσο, σύμφωνα με άλλες κατευθυντήριες οδηγίες κλινικής πρακτικής, τα στοιχεία είναι μέτρια[4].

32. Συνιστάται πλήρες λουτρό πριν από την επέμβαση.

Μέτριο επίπεδο τεκμηρίωσης. Ισχυρή σύσταση.

ΒΙΒΛΙΟΓΡΑΦΙΚΕΣ ΑΝΑΦΟΡΕΣ

1. Webster J, Osborne S. Preoperative bathing or showering with skin antiseptics to prevent surgical site infection. Cochrane Database of Systematic Reviews 2012, Issue 9. Art. No.:CD004985.

2. Kamel C, McGahan L, Polisena J, Mierzwinski-Urban M, Embil JM. Preoperative skin antiseptic preparations for preventing surgical site infections: a systematic review. Infect Control Hosp Epidemiol 2012;33(6):608-17.

3. Kamel C, McGahan L, Mierzwinski-Urban M, Embil J. Preoperative Skin Antiseptic Preparations and Application Techniques for Preventing Surgical Site Infections: A Systematic Review of the Clinical Evidence and Guidelines [Internet]. Ottawa: Canadian Agency for Drugs and Technologies in Health; 2011 (Rapid Response Report: Systematic Review). 2011. Jun. [about. 68 p.]. [cited 2014-11-Mar]. Disponible en: http://www.ca (Otros estudios de interés sobre este tema113,114).

4. Berríos-Torres SI, Craig A, Umscheid MD. Centers for Disease Control and Prevention Guideline for the Prevention of Surgical Site Infection, 2017. JAMA Surg. 2017 Aug 1;152(8):784-791. doi: 10.1001/jamasurg.2017.0904.

Προεγχειρητική νηστεία

Δεν υπάρχουν επιστημονικά στοιχεία που να επιβεβαιώνουν ότι η χορήγηση διαυγών υγρών 2 ώρες πριν από μια εκλεκτική χειρουργική επέμβαση προκαλεί μεγαλύτερο κίνδυνο εισρόφησης, παλινδρόμησης ή νοσηρότητας από τη νηστεία μετά τα μεσάνυχτα καθώς, στους περισσότερους ασθενείς, ο στόμαχος χρειάζεται 60-90 λεπτά για να αδειάσει από υγρά[1,2]. Αρκετές τυχαιοποιημένες ελεγχόμενες μελέτες έχουν δείξει ότι η κατάποση διαυγών υγρών έως και 2 ώρες και ελαφρών στερεών έως και 6 ώρες πριν από την έναρξη της αναισθησίας είναι ασφαλής και βελτιώνει την αίσθηση ευεξίας του ασθενούς[3]. Αυτές οι μελέτες έχουν δείξει ότι δεν υπάρχουν σημαντικές διαφορές σε σχέση με τον γαστρικό όγκο ή το pH του γαστρικού περιεχομένου

κατά τη νυχτερινή νηστεία σε σύγκριση με την κατάποση διαυγών υγρών έως και 2 ώρες πριν από την επέμβαση[3].

Σε ασθενείς με τεκμηριωμένη καθυστερημένη κένωση του στομάχου, διαταραχές της γαστρεντερικής κινητικότητας ή επείγουσα χειρουργική επέμβαση, η χορήγηση διαυγών υγρών 2 ώρες πριν την επέμβαση μπορεί να μην είναι ασφαλής. Υπάρχουν στοιχεία που δείχνουν ότι ασθενείς με σακχαρώδη διαβήτη τύπου 2 χωρίς χρόνιες επιπλοκές[4] και παχύσαρκοι ασθενείς[5] παρουσιάζουν φυσιολογική γαστρική κένωση και η χορήγηση διαυγών υγρών έως και 2-3 ώρες πριν από την αναισθησία μπορεί να είναι ασφαλής.

33. Στους περισσότερους ασθενείς που πρόκειται να υποβληθούν σε εκλεκτική χειρουργική επέμβαση, η στερεά τροφή θα πρέπει να επιτρέπεται έως και 6 ώρες πριν από την αναισθησία και τα διαυγή υγρά έως και 2 ώρες πριν από την αναισθησία.

Υψηλό επίπεδο τεκμηρίωσης. Ισχυρή σύσταση.

34. Σε εκείνους τους ασθενείς με καθυστερημένη γαστρική κένωση και επείγουσα χειρουργική επέμβαση, συνιστάται η νηστεία από τα μεσάνυχτα ή 6–8 ώρες πριν από την επέμβαση.

Μέτριο επίπεδο τεκμηρίωσης. Ισχυρή σύσταση.

ΒΙΒΛΙΟΓΡΑΦΙΚΕΣ ΑΝΑΦΟΡΕΣ

1. Lobo DN, Hendry PO, Rodrigues G, Marciani L, Totman JJ, Wright JW, et al. Gastric emptying of three liquid oral preoperative metabolic preconditioning regimens measured by magnetic resonance imaging in healthy adult volunteers: a randomised double-blind, crossover study. Clin Nutr. 2009; 28:636-41.

2. Lambert E, Carey S. Practice guideline recommendations on perioperative fasting. A systematic review. J Parenter Enteral Nutr. 2016; 40:1158-65.

3. Brady M, Kinn S, Stuart P. Preoperative fasting for adults to prevent perioperative complications. Cochrane Database Syst Rev. 2003; CD00442383.

4. Gustafsson UO, Nygren J, Thorell A, Soop M, Hellstrom PM, Ljungqvist O, et al. Preoperative carbohydrate loading may be used in type 2 diabetes patients. Acta Anaesthesiol Scand. 2008; 52:946-51.
5. Maltby JR, Pytka S, Watson NC, Cowan RA, Fick GH. Drinking 300 mL of clear fluid two hours before surgery has no effect on gastric fluid volume and pH in fasting and non-fasting obese patients. Can JAnaesth. 2004; 51:111-115.

Θεραπεία με ροφήματα υδρογονανθράκων

Η προεγχειρητική νηστεία και το χειρουργικό στρες μπορούν να προκαλέσουν αντίσταση στην ινσουλίνη και μετεγχειρητική υπεργλυκαιμία[1]. Η από του στόματος πρόσληψη υδατανθράκων (12,5% μαλτοδεξτρίνες) σε δόση 800 ml τα μεσάνυχτα και 400 ml 2 ώρες πριν την επέμβαση μπορεί να μετριάσει την καταβολική απόκριση που προκαλείται από τη χειρουργική επέμβαση και τη νηστεία και να μειώσει την μετεγχειρητική αντίσταση στην ινσουλίνη. Επιπλέον, μπορεί να βελτιώσει το αίσθημα ευεξίας του ασθενούς (δίψα, πείνα και άγχος)[1] χωρίς να αυξάνει τον κίνδυνο εισρόφησης[2-4].

Σε ασθενείς που υποβάλλονται σε μείζονα χειρουργική επέμβαση στην κοιλία, μια μετα-ανάλυση και μια συστηματική ανασκόπηση έχουν δείξει ότι, σε σύγκριση με τη νηστεία ή το εικονικό φάρμακο, η θεραπεία με περισσότερα από 45 g υδατανθράκων 4 ώρες πριν από την επέμβαση σχετίζεται με μια μικρή μείωση της νοσηλείας στο νοσοκομείο χωρίς να επηρεάζει το ποσοστό των μετεγχειρητικών επιπλοκών[2,3]. Τα ίδια αποτελέσματα έχουν παρατηρηθεί σε μια πρόσφατη μετα-ανάλυση δικτύου όπου η νηστεία συγκρίθηκε με τη χορήγηση χαμηλής δόσης (<45 g) ή υψηλής δόσης (>45 g) από του στόματος υδατανθράκων έως και 4 ώρες πριν από την επέμβαση, αν και δεν υπήρχαν σημαντικές διαφορές σε σχέση με την αντίσταση στην ινσουλίνη[4]. Η χορήγηση 100 g υδατανθράκων σχετίζεται με χαμηλότερη ανάγκη για θεραπεία ινσουλίνης και χαμηλότερο ποσοστό ασθενών με επίπεδο γλυκόζης στο αίμα > 180 mg/dl, χωρίς διαφορές στην ανάπτυξη μετεγχειρητικών λοιμωδών επιπλοκών[5].

35. Η από του στόματος λήψη ροφημάτων πλούσιων σε υδατάνθρακες έως και 2 ώρες πριν από την επέμβαση είναι ασφαλής και δεν σχετίζεται με αυξημένο κίνδυνο εισρόφησης.

Μέτριο επίπεδο τεκμηρίωσης. Ισχυρή σύσταση.

36. Η από του στόματος χορήγηση 200–400 ml ροφήματος που περιέχει 50 g υδατανθράκων θα πρέπει να επιτρέπεται έως και δύο ώρες πριν από την επέμβαση, καθώς αυτή η θεραπεία βελτιώνει το αίσθημα ευεξίας του ασθενούς και μπορεί να μειώσει τη νοσηλεία στο νοσοκομείο και την αντίσταση στην ινσουλίνη.

Μέτριο επίπεδο τεκμηρίωσης. Ισχυρή σύσταση.

ΒΙΒΛΙΟΓΡΑΦΙΚΕΣ ΑΝΑΦΟΡΕΣ

1. Nygren J, Thorell A, Ljungqvist O. Preoperative oral carbohydrate therapy. Curr Opin Anaesthesiol. 2015; 28:364-9.

2. Smith MD, McCall J, Plank L, Herbison GP, Soop M, Nygren J. Preoperative carbohydrate treatment for enhancing recovery after elective surgery. Cochrane Database Syst Rev. 2014:CD009161.

3. Awad S, Varadhan KK, Ljungqvist O, Lobo DN. A meta-analysis of randomised controlled trials on preoperative oral carbohydrate treatment in elective surgery. Clin Nutr. 2013; 32:34-44.

4. Amer MA, Smith MD, Herbison GP, Plank LD, McCall JL. Network meta-analysis of the effect of preoperative carbohydrate loading on recovery after elective surgery. Br J Surg. 2017; 1 04:187-197.

5. Gianotti L, Biffi R, Sandini M, Marrelli D, Vignali A, Caccialanza R, et al. Preoperative oral carbohydrate load versus placebo in major elective abdominal surgery (PROCY): a randomized, placebo-controlled, multicenter, phase III trial. Ann Surg. 2018;267:623-630.

ΕΙΔΙΚΕΣ ΠΕΡΙΠΤΩΣΕΙΣ:

Θεραπεία με ροφήματα υδατανθράκων σε ασθενείς με διαβήτη και παχυσαρκία

Παρόλο που ο σακχαρώδης διαβήτης (ΣΔ) επηρεάζει το 15% των χειρουργικών ασθενών, υπάρχουν ελάχιστα δημοσιευμένα στοιχεία σχετικά με

τα οφέλη από τη χρήση ροφημάτων με υδατάνθρακες σε παχύσαρκα άτομα ή/και σε άτομα με ΣΔ. Μια πρόσφατη RCT που περιελάμβανε ασθενείς με νοσογόνο παχυσαρκία που υποβλήθηκαν σε βαριατρική χειρουργική (20% με ΣΔ) έδειξε ότι η θεραπεία με από του στόματος υδατάνθρακες είναι ασφαλής, αν και δεν παρατηρήθηκαν διαφορές σε σχέση με τη διατήρηση της άλυπης μάζας, τη νοσηλεία στο νοσοκομείο ή τις μετεγχειρητικές επιπλοκές[1]. Σε ασθενείς με ΣΔ τύπου 2 με καλό μεταβολικό έλεγχο που δεν παρουσιάζουν νευροπαθητικές επιπλοκές και λαμβάνουν τη συνήθη υπογλυκαιμική τους αγωγή, η χορήγηση 50 g υδατανθράκων 3 ώρες πριν από την έναρξη της αναισθησίας είναι ασφαλής, δεν καθυστερεί τη γαστρική κένωση ούτε αυξάνει τον κίνδυνο υπεργλυκαιμίας ή εισρόφησης[2,3].

37. Σε παχύσαρκους ή/και διαβητικούς τύπου 2 ασθενείς με καλό γλυκαιμικό έλεγχο χωρίς σχετιζόμενες χρόνιες επιπλοκές, θα μπορούσε να εξεταστεί η χρήση ροφημάτων πλούσιων σε υδατάνθρακες 3 ώρες πριν από την επέμβαση. Αυτά μπορούν να χορηγηθούν μαζί με τα συνήθη αντιδιαβητικά φάρμακα του ασθενούς.

Χαμηλό επίπεδο τεκμηρίωσης. Ασθενής σύσταση.

ΒΙΒΛΙΟΓΡΑΦΙΚΕΣ ΑΝΑΦΟΡΕΣ

1. Azagury DE, Ris F, Pichard C, Volonte F, Karsegard L, Huber O. Does perioperative nutrition and oral carbohydrate load sustainably preserve muscle mass after bariatric surgery? A randomized control trial. Surg Obes Relat Dis. 2015; 11:920-6.

2. Laffin MR, Li S, Brisebois R, Senior PA, Wang H. The use of a pre-operative carbohydrate drink in patients with diabetes mellitus: a prospective, non-inferiority, cohort study. World J Surg. 2018; 42:1965-70.

3. Gustafsson UO, Nygren J, Thorell A, Soop M, Hellström PM, Ljungqvist O, et al. Pre-operative carbohydrate loading may be used in type 2 diabetes patients. Acta Anaesthesiol Scand. 2008; 52:946-5199.

ΠΡΟΝΑΡΚΩΣΗ

Χρήση ηρεμιστικών και αγχολυτικών φαρμάκων

Η χρήση προνάρκωσης με φάρμακα μακράς διάρκειας ημίσειας ζωής, όπως οπιοειδή ή βενζοδιαζεπίνες, μπορεί να αποτρέψει την πρώιμη μετεγχειρητική ανάρρωση, προκαλώντας καθυστέρηση στην έναρξη της κινητοποίησης και στην ανοχή στα υγρά από του στόματος και πιθανώς παράταση της νοσηλείας στο νοσοκομείο[1,2].

Η χρήση αγχολυτικών με σύντομο χρόνο ημίσειας ζωής στην άμεση προεγχειρητική περίοδο θα μπορούσε να επιμηκύνει τον χρόνο χορήγησης της αναισθησίας[3], καθώς και να καθυστερήσει την μετεγχειρητική ανάρρωση και να αυξήσει τον κίνδυνο γνωστικής δυσλειτουργίας, ιδιαίτερα σε ηλικιωμένους, ευπαθείς ασθενείς και σε εκείνους με σημαντική συννοσηρότητα[4]. Δεν υπάρχουν οριστικά δεδομένα για τη χρήση τους, αλλά σε χειρουργικές επεμβάσεις με βραχεία παραμονή στο νοσοκομείο η χρήση τους σε χαμηλές δόσεις δεν έδειξε καθυστέρηση στην έξοδο από το νοσοκομείο, παρουσιάζοντας μειωμένη συχνότητα μετεγχειρητικής ναυτίας και εμέτου[5].

Αυτή η σύσταση εμφανίζεται στην πρώτη έκδοση του Intensified Recovery Pathway in Abdominal Surgery (RICA) και βασίζεται κυρίως στη συναίνεση των ειδικών[6].

38. Συνιστάται να αποφεύγεται η χρήση μακράς διάρκειας ημίσειας ζωής βενζοδιαζεπίνων και οπιοειδών πριν από την εισαγωγή στην αναισθησία σε ασθενείς υψηλού κινδύνου λόγω ηλικίας και συννοσηρότητων.

Χαμηλό επίπεδο τεκμηρίωσης. Ισχυρή σύσταση.

ΒΙΒΛΙΟΓΡΑΦΙΚΕΣ ΑΝΑΦΟΡΕΣ

1. Jeon S, Lee HJ, Do W, Kim HK, Kwon JY, Hwang BY, et al. Randomized controlled trial assessing the effectiveness of midazolam premedication as an anxiolytic, analgesic, sedative, and hemodynamic stabilizer. Medicine (Baltimore). 2018; 97(35): e12187.

2. Mijderwijk H, Van Beek S, Duivenvoorden HJ, Stolker RJ. Effectiveness of benzodiazepine premedication on recovery in day-case surgery: a systematic review with meta-analysis. Minerva Anestesiol. 2016; 82(4): 438-64.
3. Bucx MJL, Krijtenburg P, Kox M. Preoperative use of anxiolytic-sedative agents; Are we on the right track? Journal of Clinical Anesthesia. J Clin Anesth. 2016; 33:135-40.
4. Maurice-Szamburski A, Auquier P, Viarre-Oreal V, Cuvillon P, Carles M, Ripart et al. Effect of sedative premedication on patient experience after general anesthesia: A randomized clinical trial. JAMA. 2015; 313(9): 916-25.
5. Beydon L, Rouxel A, Camut, N, Schinkel, N, Malinovsky, JM, Aveline C, et al. Sedative premedication before surgery. A multicentre randomized study versus placebo. Anaesth Crit Care Pain Med. 2015; 34(3):165-71.

7.1.2. ΠΡΟΕΓΧΕΙΡΗΤΙΚΑ

Αντιβιοτική χημειοπροφύλαξη

Η λοίμωξη του χειρουργικού πεδίου - ΛΧΠ (Surgical Site Infection - SSI) εξακολουθεί να είναι η δεύτερη κύρια αιτία λοίμωξης που σχετίζεται με την υγειονομική περίθαλψη[1,2] και έχει αποδειχθεί ότι σχετίζεται σαφώς με μεγαλύτερη νοσηλεία, αυξημένη νοσηρότητα και κόστος, καθώς και ότι έχει σαφή αντίκτυπο στην ποιότητα ζωής των ασθενών[3]. Όπως υποδεικνύεται στο πρόγραμμα Zero Surgical Infection, η αντιβιοτική χημειοπροφύλαξη έχει εξαιρετική αποτελεσματικότητα στην πρόληψη των λοιμώξεων του χειρουργικού πεδίου και εξακολουθεί να είναι το κύριο μέτρο πρόληψης και το πιο αποδοτικό από άποψη κόστους[4]. Μεταξύ των διαφόρων δράσεων για την πρόληψη των λοιμώξεων του χειρουργικού πεδίου, η αντιβιοτική χημειοπροφύλαξη είναι ένα από τα πιο αποτελεσματικά μέτρα, αν και η αποτελεσματικότητά του μειώνεται εάν δεν τηρηθούν τα υπόλοιπα μέτρα[5].

Ωστόσο, η ακατάλληλη χορήγηση της αντιβιοτικής χημειοπροφύλαξης όχι μόνο αυξάνει τον κίνδυνο των λοιμώξεων του χειρουργικού πεδίου αλλά σχετίζεται επίσης με αυξημένο επιπολασμό πολυανθεκτικών μικροβίων και με επεισόδια τοξικότητας. Στην Ευρώπη, η χειρουργική αντιβιοτική χημειοπροφύλαξη αντιπροσωπεύει περίπου το 25% των συνταγών αντιβιοτικών, ενώ συντηρείται ασκόπως (για περισσότερες από 24 ώρες) σε περισσότερες από τις μισές περιπτώσεις[6]. Οι πιο πρόσφατες συστάσεις από τον ΠΟΥ[1,2] για την πρόληψη των λοιμώξεων του χειρουργικού πεδίου περιλαμβάνουν τέσσερις συστάσεις που είναι ειδικές για την αντιβιοτική χημειοπροφύλαξη: 1) χορήγηση του αντιβιοτικού πριν από την επέμβαση εάν συνιστάται, 2) χορήγησή του εντός 120 λεπτών πριν από την τομή (με βάση τον χρόνο ημίσειας ζωής του φαρμάκου), 3) διακόψτε τη χορήγηση αντιβιοτικών ακόμα και αν υπάρχουν παροχετεύσεις, 4) μην διατηρείτε την χημειοπροφύλαξη μετά την ολοκλήρωση της χειρουργικής επέμβασης.

Μια προσθήκη σε αυτές τις συστάσεις είναι αυτή που δημοσιεύτηκε το 2017 από τα Κέντρα Ελέγχου και Πρόληψης Νοσημάτων: α) να χορηγείται η αντιβιοτική χημειοπροφύλαξη μόνο σε εκείνες τις χειρουργικές επεμβάσεις στις

οποίες ενδείκνυται, β) σε καισαρικές τομές εγχύστε το αντιβιοτικό πριν την τομή, και γ) να μην διατηρείται η χημειοπροφύλαξη μετά το κλείσιμο του τραύματος[7]. Η αντιβιοτική χημειοπροφύλαξη στη χειρουργική επέμβαση θα πρέπει να οδηγεί σε συγκεντρώσεις αντιβιοτικού στον ορό και στους ιστούς πάνω από τις ελάχιστες ανασταλτικές συγκεντρώσεις των πιο πιθανών μολυσματικών μικροοργανισμών για κάθε επέμβαση τη στιγμή της τομής και να διατηρείται καθ' όλη τη διάρκεια της χειρουργικής επέμβασης[7-9].

Ένδειξη και επιλογή αντιβιοτικού

Στα καθαρά χειρουργεία, η ένδειξη εξαρτάται από το είδος της επέμβασης, τις συννοσηρότητες του ασθενούς και τη χρήση προσθετικού υλικού. Σε καθαρά – επιμολυσμένα και επιμολυσμένα χειρουργεία συνιστάται να χρησιμοποιείτε πάντα αντιβιοτική χημειοπροφύλαξη. Στα ρυπαρά τραύματα δεν λαμβάνεται υπόψη η αντιβιοτική χημειοπροφύλαξη αλλά χορηγείται αντιβιοτική θεραπεία.

Οι κεφαλοσπορίνες πρώτης ή δεύτερης γενιάς είναι τα φάρμακα εκλογής για προφύλαξη λόγω της αποτελεσματικότητας, του αντιμικροβιακού φάσματος, των λίγων ανεπιθύμητων ενεργειών και του χαμηλού κόστους τους, όπως αντικατοπτρίζεται στις μελέτες και στις περισσότερες από τις τρέχουσες κατευθυντήριες οδηγίες[10-14]. Σε περιπτώσεις αλλεργίας στις βήτα-λακτάμες, ιστορικό αποικισμού ή μόλυνσης από ανθεκτικό στη μεθικιλλίνη Staphylococcus aureus ή υψηλό επιπολασμό λοίμωξης του χειρουργικού τραύματος στο νοσοκομείο από αυτόν τον μικροοργανισμό, μπορεί να χρησιμοποιηθεί ένα γλυκοπεπτίδιο. Τέλος, σε χειρουργικές επεμβάσεις παχέος εντέρου ή γυναικολογικές, στις οποίες αναμένεται η παρουσία αναερόβιων μικροοργανισμών και εντεροβακτηρίων, είναι σκόπιμο να επιλέγεται αντιβιοτικό ή συνδυασμός αντιβιοτικών με δράση έναντι και των δύο ομάδων μικροοργανισμών.

39. Η αντιβιοτική χημειοπροφύλαξη συνιστάται εάν οι πιθανότητες λοίμωξης είναι υψηλές ή εάν οι συνέπειες μιας μετεγχειρητικής λοίμωξης είναι δυνητικά σοβαρές για τον ασθενή (ενδοκαρδίτιδα, ενδοφθαλμίτιδα, λοίμωξη προσθετικού υλικού).

Μέτριο επίπεδο τεκμηρίωσης. Ισχυρή σύσταση.

40. Σε καθαρές χειρουργικές επεμβάσεις με παράγοντες κινδύνου για εμφάνιση λοίμωξης, συνιστάται η χρήση αντιβιοτικών που καλύπτουν μικροοργανισμούς του δερματικού μικροβιώματος (Staphylococcus aureus και αρνητικός στην κοαγκουλάση σταφυλόκοκκος) και, σε καθαρές - επιμολυσμένες χειρουργικές επεμβάσεις, συνιστάται η χρήση αντιβιοτικών που καλύπτουν επίσης gram-αρνητικούς βακίλους και εντερόκοκκους καθώς και αναερόβια.

Μέτριο επίπεδο τεκμηρίωσης. Ισχυρή σύσταση.

Χρόνος χορήγησης

Μία από τις θεμελιώδεις πτυχές για τη διατήρηση της αποτελεσματικότητας της αντιβιοτικής χημειοπροφύλαξης είναι η χορήγησή της στον βέλτιστο χρόνο. Στην περίπτωση των βήτα-λακταμών βραχείας ημίσειας ζωής (π.χ. πενικιλίνη και κεφαλοσπορίνες όπως η κεφαζολίνη, η κεφοξιτίνη και η κεφουροξίμη) συνιστάται η χορήγησή τους εντός 60 λεπτών πριν από τη χειρουργική τομή. Στην περίπτωση της βανκομυκίνης, των αμινογλυκοσίδων ή των φθοριοκινολονών, η ενδοφλέβια έγχυση πρέπει να ξεκινά 90 λεπτά πριν από τη χειρουργική τομή, καθώς αυτά τα αντιβιοτικά απαιτούν μεγάλες περιόδους έγχυσης. Στην περίπτωση χειρουργικών επεμβάσεων που απαιτούν ισχαιμία των άκρων, θα πρέπει πρώτα να χορηγείται αντιβιοτική χημειοπροφύλαξη[15,16].

41. Συνιστάται η χορήγηση αντιβιοτικής χημειοπροφύλαξης κατά τη διάρκεια των 120 λεπτών πριν από τη χειρουργική τομή.

Υψηλό επίπεδο τεκμηρίωσης. Ισχυρή σύσταση.

Δόση αντιβιοτικού και διάρκεια αντιβιοτικής χημειοπροφύλαξης

Όσον αφορά τη δόση για αντιβιοτική χημειοπροφύλαξη θα πρέπει να είναι η ίδια με αυτή που χρησιμοποιείται για τη θεραπεία της λοίμωξης. Ωστόσο, και δεδομένου του τρέχοντος επιπολασμού στον πληθυσμό[17-19], οι παχύσαρκοι ασθενείς μπορεί να χρειάζονται υψηλότερες αρχικές δόσεις, αν και οι δόσεις που βασίζονται στο συνολικό σωματικό βάρος τείνουν σε υπερδοσολογία, επομένως θα πρέπει να χρησιμοποιούνται υποκατάστατοι δείκτες του

συνολικού σωματικού βάρους, όπως το ιδανικό βάρος ή το προσαρμοσμένο βάρος. Σε περίπτωση ανάγκης διατήρησης παρατεταμένης δόσης, η προσαρμογή ανάλογα με τη νεφρική λειτουργία μπορεί να είναι μια έγκυρη εναλλακτική λύση[20,21].

Ωστόσο, εάν η επέμβαση υπερβαίνει περισσότερο από 2 φορές τον χρόνο ημίσειας ζωής του αντιβιοτικού ή σε καταστάσεις στις οποίες ο χρόνος ημίσειας ζωής μειώνεται (εγκαύματα, υψηλοί ρυθμοί σπειραματικής διήθησης) ή σε σημαντική αιμορραγία (> 1.500 ml σε ενήλικες ή 25 ml/kg σε παιδιά) θα πρέπει να χορηγηθεί μια επιπλέον δόση[22-24]. Η παράταση της διάρκειας της αντιβιοτικής χημειοπροφύλαξης αντενδείκνυται, καθώς στις περισσότερες χειρουργικές επεμβάσεις, μια εφάπαξ δόση ενός αντιβιοτικού του οποίου ο χρόνος ημίσειας ζωής εξασφαλίζει επαρκή επίπεδα φαρμάκου στον ορό και στους ιστούς κατά τη διάρκεια της χειρουργικής επέμβασης είναι επαρκής.

42. Συνιστάται η χρήση της ίδιας δόσης για αντιβιοτική χημειοπροφύλαξη με αυτή που χρησιμοποιείται για τη θεραπεία της λοίμωξης, αν και σε παχύσαρκους ασθενείς θα πρέπει να χρησιμοποιείται το προσαρμοσμένο βάρος για τον υπολογισμό της δόσης.

Μέτριο επίπεδο τεκμηρίωσης. Ισχυρή σύσταση.

43. Επιπρόσθετη δόση συνιστάται σε περιπτώσεις παρατεταμένων χειρουργικών επεμβάσεων ή εάν υπάρχει σημαντική απώλεια αίματος.

Μέτριο επίπεδο τεκμηρίωσης. Ασθενής σύσταση.

44. Συνιστάται να μην παρατείνεται η διάρκεια της αντιβιοτικής χημειοπροφύλαξης πέρα από τη διάρκεια της ίδιας της χειρουργικής επέμβασης.

Υψηλό επίπεδο τεκμηρίωσης. Ισχυρή σύσταση.

Σημείωση – Ανεπιθύμητες ενέργειες της αντιβιοτικής χημειοπροφύλαξης

Είναι σημαντικό να θυμόμαστε ότι η χορήγηση αντιβιοτικών στη χειρουργική προφύλαξη μπορεί να προκαλέσει ανεπιθύμητες ενέργειες όπως φαρμακευτική αλλεργία[25] (ειδικά στις βήτα-λακτάμες), διάρροια που σχετίζεται με αντιβιοτικά ή/και λοίμωξη από Clostridioides difficile[26,27], ανάπτυξη

αντιμικροβιακής αντοχής[28,29] και οξεία νεφρική ανεπάρκεια σε μείζονες χειρουργικές επεμβάσεις ή/και ταυτόχρονη χορήγηση αμινογλυκοσίδων και γλυκοπεπτιδίων[30,31].

Έτσι, η αλλεργία στις βήτα-λακτάμες θα πρέπει να αποκλείεται τόσο κατά την αναισθησιολογική εκτίμηση όσο και κατά την προεγχειρητική φροντίδα. Ομοίως, όλες οι οδηγίες (γενικές και τοπικές) θα πρέπει να εξετάζουν τη χρήση εναλλακτικών φαρμάκων έναντι των βήτα-λακτάμων σε περίπτωση αλλεργίας.

Η λοίμωξη από C. Difficile είναι μια σοβαρή επιπλοκή που μπορεί να εμφανιστεί με ορισμένα αντιβιοτικά που χρησιμοποιούνται στην αντιβιοτική χημειοπροφύλαξη, όπως οι κεφαλοσπορίνες, οι καρβαπενέμες, οι φθοροκινολόνες ή η κλινδαμυκίνη, ειδικά εάν η διάρκεια της προφύλαξης είναι παρατεταμένη. Η χρήση μεμονωμένων δόσεων βοηθά επίσης στην ελαχιστοποίηση άλλων ανεπιθύμητων ενεργειών που περιλαμβάνουν την αντιμικροβιακή αντοχή.

Σε σχέση με την πιθανότητα ανάπτυξης οξείας νεφρικής ανεπάρκειας λόγω της χρήσης αντιβιοτικών, θα πρέπει να γίνονται διαδοχικοί προσδιορισμοί της κρεατινίνης ορού και ούρων, τόσο προεγχειρητικά όσο και ≥24 ώρες μετά την επέμβαση, σε ασθενείς με μείζονα χειρουργική επέμβαση για έλεγχο του βαθμού νεφρικής λειτουργίας, με ιδιαίτερη προσοχή σε ασθενείς που έχουν λάβει προφύλαξη με αμινογλυκοσίδες ή γλυκοπεπτίδια.

ΒΙΒΛΙΟΓΡΑΦΙΚΕΣ ΑΝΑΦΟΡΕΣ

1. Allegranzi B, Zayed B, Bischoff P, Kubilay NZ, de Jonge S, de Vries F, et al. New WHO recommendations on intraoperative and postoperative measures for surgical site infection prevention: an evidence-based global perspective. Lancet Infect Dis. diciembre de 2016; 16(12):e288-303.

2. Allegranzi B, Bischoff P, de Jonge S, Kubilay NZ, Zayed B, Gomes SM, et al. New WHO recommendations on preoperative measures for surgical site infection prevention: an evidence-based global perspective. Lancet Infect Dis. diciembre de 2016;16(12):e276-87.

3. Badia JM, Casey AL, Petrosillo N, Hudson PM, Mitchell SA, Crosby C. Impact of surgical site infection on healthcare costs and patient outcomes: a systematic review in six European countries. J Hosp Infect. mayo de 2017;96(1):1-15.

4. Proyecto Infección Quirúrgica Zero del Sistema Nacional de Salud. Sociedad Española de Medicina Preventiva, Salud Pública e Higiene. 2016.
5. Koek MBG, Hopmans TEM, Soetens LC, Wille JC, Geerlings SE, Vos MC, et al. Adhering to a national surgical care bundle reduces the risk of surgical site infections. PLoS ONE. 2017;12(9):e0184200.
6. Plachouras D, Kärki T, Hansen S, Hopkins S, Lyytikäinen O, Moro ML, et al. Antimicrobial use in European acute care hospitals: results from the second point prevalence survey (PPS) of healthcare-associated infections and antimicrobial use, 2016 to 2017. Eurosurveillance. 15 de noviembre de 2018;23(46):1800393.
7. Berríos-Torres SI, Umscheid CA, Bratzler DW, Leas B, Stone EC, Kelz RR, et al. Centers for Disease Control and Prevention Guideline for the Prevention of Surgical Site Infection, 2017. JAMA Surg. 1 de agosto de 2017;152(8):784-91.
8. Del Toro López MD, Arias Díaz J, Balibrea JM, Benito N, Canut Blasco A, Esteve E, Horca- jada JP, Ruiz Mesa JD, Vázquez AM, Muñoz Casares C, Del Pozo JL, Pujol M, Riera M, Jimeno J, Rubio Pérez I, Ruiz-Tovar Polo J, Serrablo A, Soriano A, Badia JMl Grupo de Estudio de PA de la Sociedad Española de Enfermedades Infecciosas y Microbiología Clínica (SEIMC) y Asociación Española de Cirujanos (AEC). Executive summary of the Consensus Document of the Spanish Society of Infectious Diseases and Clinical Microbiology (SEIMC) and of the Spanish Association of Surgeons (AEC) in antibiotic prophylaxis in surgery. Cir Esp. 2020 Jul 28;S0009-739X(20)30113-5. doi: 10.1016/j.ci- resp.2020.03.022.
9. Bratzler DW, Dellinger EP, Olsen KM, Perl TM, Auwaerter PG, Bolon MK, et al. Clinical practice guidelines for antimicrobial prophylaxis in surgery. Am J Health Syst Pharm. 1 de febrero de 2013;70(3):195-283.
10. Asensio A. [Surgical site infections: antibiotic prophylaxis in surgery]. Enferm Infecc Microbiol Clin. enero de 2014;32(1):48-53.
11. Townsend TR, Reitz BA, Bilker WB, Bartlett JG. Clinical trial of cefamandole, cefazolin, and cefuroxime for antibiotic prophylaxis in cardiac operations. J Thorac Cardiovasc Surg. octubre de 1993;106(4):664-70.

12. Kreter B, Woods M. Antibiotic prophylaxis for cardiothoracic operations. Meta-analysis of thirty years of clinical trials. J Thorac Cardiovasc Surg. septiembre de 1992;104(3):590-9.
13. Bratzler DW, Houck PM, Surgical Infection Prevention Guidelines Writers Workgroup, Ame- rican Academy of Orthopaedic Surgeons, American Association of Critical Care Nurses, American Association of Nurse Anesthetists, et al. Antimicrobial prophylaxis for surgery: an advisory statement from the National Surgical Infection Prevention Project. Clin Infect Dis. 15 de junio de 2004;38(12):1706-15.
14. Engelman R, Shahian D, Shemin R, Guy TS, Bratzler D, Edwards F, et al. The Society of Thoracic Surgeons practice guideline series: Antibiotic prophylaxis in cardiac surgery, part II: Antibiotic choice. Ann Thorac Surg. abril de 2007;83(4):1569-76.
15. de Jonge SW, Gans SL, Atema JJ, Solomkin JS, Dellinger PE, Boermeester MA. Timing of preoperative antibiotic prophylaxis in 54,552 patients and the risk of surgical site infection: A systematic review and meta-analysis. Medicine (Baltimore). julio de 2017;96(29):e6903.
16. Lizán-García M, García-Caballero J, Asensio-Vegas A. Risk factors for surgical wound infection in general surgery: a prospective study. Infect Control Hosp Epidemiol. mayo de 1997;18(5):310-5.
17. Crawford T, Rodvold KA, Solomkin JS. Vancomycin for surgical prophylaxis? Clin Infect Dis. mayo de 2012;54(10):1474-9.
18. Pea F, Furlanut M, Stellini R, Bonardelli S, Signorini L, Pavan F, et al. Pharmacokinetic - pharmacodynamic aspects of antimicrobial prophylaxis with teicoplanin in patients under- going major vascular surgery. Int J Antimicrob Agents. enero de 2006;27(1):15-9.
19. How should antibiotics be dosed in obesity? - SPS- Specialist Pharmacy Service - The first stop for professional medicines advice [Internet]. [citado 12 de febrero de 2019]. Disponible en: https://www.sps.nhs.uk/articles/how-should-antibiotics-be-dosed-in-obesity/
20. Voigt J, Mosier M, Darouiche R. Systematic review and meta-analysis of randomized controlled trials of antibiotics and antiseptics for preventing infection in people receiving primary total hip and knee prostheses. Antimicrob Agents Chemother. noviembre de 2015;59(11):6696- 707.

21. Pai MP. Treatment of bacterial infections in obese adult patients: how to appropriately manage antimicrobial dosage. Curr Opin Pharmacol. octubre de 2015;24:12-7.
22. Isla A, Trocóniz IF, de Tejada IL, Vázquez S, Canut A, López JM, et al. Population pharmacokinetics of prophylactic cefoxitin in patients undergoing colorectal surgery. Eur J Clin Pharmacol. mayo de 2012;68(5):735-45.
23. Asín-Prieto E, Soraluce A, Trocóniz IF, Campo Cimarras E, Sáenz de Ugarte Sobrón J, Rodríguez-Gascón A, et al. Population pharmacokinetic models for cefuroxime and metronidazole used in combination as prophylactic agents in colorectal surgery: Model-based evaluation of standard dosing regimens. Int JAntimicrob Agents. mayo de 2015;45(5):504-11.
24. Swoboda SM, Merz C, Kostuik J, Trentler B, Lipsett PA. Does intraoperative blood loss affect antibiotic serum and tissue concentrations? Arch Surg. noviembre de 1996;131(11):1165- 71; discussion 1171-1172.
25. Hong J, Krop LC, Johns T, Pai MP. Individualized vancomycin dosing in obese patients: a two-sample measurement approach improves target attainment. Pharmacotherapy. mayo de 2015;35(5):455-63.
26. Carignan A, Allard C, Pépin J, Cossette B, Nault V, Valiquette L. Risk of Clostridium difficile infection after perioperative antibacterial prophylaxis before and during an outbreak of in- fection due to a hypervirulent strain. Clin Infect Dis. 15 de junio de 2008;46(12):1838- 43.
27. Jenkins PJ, Teoh K, Simpson PM, Dave J, Simpson AHWR, Breusch S. Clostridium difficile in patients undergoing primary hip and knee replacement. J Bone Joint Surg Br. julio de 2010;92(7):994-8.
28. Avery CME, Ameerally P, Castling B, Swann RA. Infection of surgical wounds in the maxillofacial region and free flap donor sites with methicillin-resistant Staphylococcus aureus. Br J Oral Maxillofac Surg. junio de 2006;44(3):217-21.
29. Harbarth S, Samore MH, Lichtenberg D, Carmeli Y. Prolonged antibiotic prophylaxis after cardiovascular surgery and its effect on surgical site infections and antimicrobial resistance. Circulation. 27 de junio de 2000;101(25):2916-21.
30. Walker H, Patton A, Bayne G, Marwick C, Sneddon J, Davey P, et al. Reduction in post- operative acute kidney injury following a change in antibiotic

prophylaxis policy for orthopaedic surgery: an observational study. J Antimicrob Chemother. 2016;71(9):2598-605.
31. Bell S, Dekker FW, Vadiveloo T, Marwick C, Deshmukh H, Donnan PT, et al. Risk of postoperative acute kidney injury in patients undergoing orthopaedic surgery - development and validation of a risk score and effect of acute kidney injury on survival: observational cohort study. BMJ. 11 de noviembre de 2015;351:h5639.

Γλυκοκορτικοειδή

Η προεγχειρητική χορήγηση γλυκοκορτικοειδών σχετίζεται με εξασθένηση του μεγέθους της φλεγμονώδους απάντησης στο χειρουργικό στρες και μπορεί να μειώσει τη συχνότητα των επιπλοκών, συμπεριλαμβανομένων και εκείνων λοιμώδους φύσης[1-3]. Οι επιδράσεις της περιλαμβάνουν αγγειοσυστολή και μειωμένη διαπερατότητα των τριχοειδών[4]. Στη μετεγχειρητική περίοδο, μειώνουν την έκκριση παραγόντων οξείας φάσης, όπως η ιντερλευκίνη 6 ή η CRP[5].

45. Η χορήγηση μιας εφάπαξ δόσης γλυκοκορτικοειδών συνιστάται γιατί έχει σημαντική επίδραση στη διάρκεια της νοσηλείας στο νοσοκομείο χωρίς να αυξάνει το ποσοστό των επιπλοκών.

Μέτριο επίπεδο τεκμηρίωσης. Ισχυρή σύσταση.

ΒΙΒΛΙΟΓΡΑΦΙΚΕΣ ΑΝΑΦΟΡΕΣ

1. Taniguchi Y, Kurokawa Y, Hagi T, Takahashi T, Miyazaki Y, Tanaka K, et al. Methylprednisolone Inhibits Tumor Growth and Peritoneal Seeding Induced by Surgical Stress and Postoperative Complications. Annals of Surgical Oncology. 2019;26(9):2831-38.
2. Steinthorsdottir KJ, Kehlet H, Aasvang EK. Surgical stress response and the potential role of preoperative glucocorticoids on post-anesthesia care unit recovery. Minerva Anestesiologi- ca. 2017;83(12):1324-31.
3. El-Sibai K, Rajpal A, Al-Aridi R, Selman WR, Arafah BM. The impact of perioperative dexamethasone administration on the normal hypothalamic

pituitary adrenal response to major surgical procedures. Endocrine. 2017;58(1):134-42.
4. McSorley ST, Horgan PG, McMillan DC. The impact of preoperative corticosteroids on the systemic inflammatory response and postoperative complications following surgery for gastrointestinal cancer: a systematic review and meta-analysis. Crit Rev Oncol Hematol. 2016;101:139-50.
5. McSorley ST, Roxburgh CSD, Horgan PG, McMillan DC. The Impact of Preoperative Dexamethasone on the Magnitude of the Postoperative Systemic Inflammatory Response and Complications Following Surgery for Colorectal Cancer. Ann Surg Oncol. 2017;24(8): 2104-12.

Περιεγχειρητική παρακολούθηση γλυκόζης αίματος

Ο έλεγχος της νορμογλυκαιμίας είναι απαραίτητος για τη μείωση των περιεγχειρητικών λοιμώξεων και τη μείωση των επιπλοκών από την υπεργλυκαιμία. Είναι μία από τις συστάσεις του προγράμματος Zero Surgical Infection του Υπουργείου Υγείας της κυβέρνησης της Ισπανίας[1]. Η χρήση εντατικής θεραπείας με ινσουλίνη πρέπει να αποφεύγεται λόγω του υψηλού κινδύνου υπογλυκαιμίας κατά την περιεγχειρητική περίοδο που μπορεί να οδηγήσει σε αυξημένη θνησιμότητα. Το συναινετικά αποδεκτό εύρος γλυκόζης αίματος πρέπει να είναι μεταξύ 150 και 180 g/dl[2-5].

46. Η γλυκόζη αίματος θα πρέπει να παρακολουθείται προεγχειρητικά καθώς η διεγχειρητική υπεργλυκαιμία μπορεί να οδηγήσει σε αυξημένες μετεγχειρητικές επιπλοκές, αν και η χρήση εντατικής θεραπείας με ινσουλίνη θα πρέπει να αποφεύγεται λόγω του κινδύνου υπογλυκαιμίας.

Υψηλό επίπεδο τεκμηρίωσης. Ισχυρή σύσταση.

ΒΙΒΛΙΟΓΡΑΦΙΚΕΣ ΑΝΑΦΟΡΕΣ

1. Protocolo de Trabajo del IQZ 2017. Disponible en (última consulta 01-06-2020): https:// infeccionquirurgicazero.es/es/documentos-y-materiales/protocolos-de-trabajo

2. Pontes JPJ, Mendes FF, Vasconcelos MM, Batista NR. [Evaluation and perioperative management of patients with diabetes mellitus. A challenge for the anesthesiologist]. Rev Bras Anestesiol 2018;68(1):75-86.
3. Akiboye F, Rayman G. Management of Hyperglycemia and Diabetes in Orthopedic Surgery. Curr Diab Rep 2017;17(2):13.
4. Dhatariya K, Levy N, Hall GM. The impact of glycaemic variability on the surgical patient. Curr Opin Anaesthesiol 2016;29(3):430-7.
5. Barker P, Creasey PE, Dhatariya K, Levy N, Lipp A, Nathanson MH et al. Perioperative management of the surgical patient with diabetes 2015: Association of Anaesthetists of Great Britain and Ireland. Anaesthesia 2015;70(12):1427-40.

Περιεγχειρητική υπεργλυκαιμία

Η υπεργλυκαιμία σχετίζεται με αυξημένη νοσηρότητα και θνησιμότητα σε χειρουργημένους ασθενείς, τόσο διαβητικούς όσο και μη.

Ο βελτιωμένος γλυκαιμικός έλεγχος μειώνει τον κίνδυνο νοσοκομειακών επιπλοκών μετά την επέμβαση[1]. Μια ευρέως αποδεκτή σύσταση είναι η διατήρηση της γλυκόζης αίματος μεταξύ 140 και 180 mg/dl για νοσηλευόμενους ασθενείς. Σε ασθενείς με παρεντερική διατροφή και γλυκόζη στο αίμα μεγαλύτερη από 180 mg/dl, η πρόσληψη γλυκόζης μπορεί να μειωθεί ή/και να αυξηθεί η θεραπεία με ινσουλίνη. Είναι προτιμότερο οι ασθενείς με ασταθή και υψηλά επίπεδα γλυκόζης να αντιμετωπίζονται σε μονάδες αυξημένης φροντίδας ή εντατικής θεραπείας[2].

Η θεραπεία με ινσουλίνη για τη θεραπεία της επίμονης υπεργλυκαιμίας θα πρέπει να ξεκινά σε επίπεδο > 180 mg/dl, με συνιστώμενο εύρος 140–180 mg/dl στους περισσότερους ασθενείς τόσο σε κρίσιμη όσο και σε μη κρίσιμη κατάσταση[3].

Πιο αυστηρά επίπεδα μεταξύ 110-140 mg/dl μπορεί να είναι κατάλληλα σε επιλεγμένους ασθενείς, εάν μπορούν να επιτευχθούν χωρίς σημαντική υπογλυκαιμία. Απαιτείται περισσότερη έρευνα για την ανάπτυξη συστάσεων θεραπείας.

47. Η περιεγχειρητική γλυκόζη αίματος θα πρέπει να παρακολουθείται και να αντιμετωπίζεται επαρκώς με ινσουλίνη, αποφεύγοντας τα επίπεδα γλυκόζης στο αίμα > 180 mg/dl.

Μέτριο επίπεδο τεκμηρίωσης. Ισχυρή σύσταση.

48. Πιο φιλόδοξοι στόχοι για περιεγχειρητική γλυκόζη αίματος μεταξύ 110 και 140 mg/dL (6,1-7,8 mmol/l) μπορεί να είναι κατάλληλοι σε επιλεγμένους ασθενείς, εάν μπορούν να επιτευχθούν χωρίς σημαντική υπογλυκαιμία.

Χαμηλό επίπεδο τεκμηρίωσης. Ασθενής σύσταση.

ΒΙΒΛΙΟΓΡΑΦΙΚΕΣ ΑΝΑΦΟΡΕΣ

1. Kotagal M, Symons RG, Hirsch IB, Umpierrez GE, Farrokhi ET, Flum DR, SCOAP-Ceertain Collaborative. Perioperative hyperglycemia and risk of adverse events among patients with and without diabetes. Ann Surg 2015;261(1):97-103.
2. Weimann A, Braga M, Carli F, Higashiguchi T, Hübner M, Klek S, et al. ESPEN guideline: Clinical nutrition in surgery. Clin Nutr 2017;36:623-650.
3. American Diabetes Association. 15. Diabetes Care in the Hospital: Standards of Medical Care in Diabetes-2019. Diabetes Care. 2019 Jan;42(Suppl 1):S173-S181.

Αφαίρεση τριχών

Οι τρίχες παραδοσιακά θεωρούνταν ότι συνδέονται με έλλειψη καθαρισμού και αυξημένη λοίμωξη του χειρουργικού τραύματος. Επιπλέον, η αφαίρεσή τους επιτρέπει την καλύτερη έκθεση της περιοχής της τομής και διευκολύνει τη ραφή και την τοποθέτηση επιδέσμων.

Μελέτες δείχνουν ότι το προηγούμενο ξύρισμα της περιοχής της τομής έχει προληπτική αποτελεσματικότητα κοντά στο 50% για λοιμώξεις χειρουργικού πεδίου[1].

Τόσο σε μια ανασκόπηση του Cochrane όσο και σε πιο πρόσφατες μετα-αναλύσεις, δεν παρατηρήθηκαν σημαντικές διαφορές στην εμφάνιση λοίμωξης του τραύματος μεταξύ ασθενών στους οποίους είχαν αφαιρεθεί οι τρίχες και εκείνων στους οποίους δεν είχαν[1-5].

Ως εκ τούτου, οι τρέχουσες συστάσεις προτείνουν ότι οι τρίχες του ασθενούς δεν πρέπει να αφαιρούνται πριν από την επέμβαση εκτός εάν είναι απολύτως απαραίτητο, και σε αυτή την περίπτωση θα πρέπει να χρησιμοποιείται ηλεκτρική ξυριστική μηχανή για την κοπή των τριχών, κατά προτίμηση με κεφαλή μιας χρήσης (Project Surgical Infection Zero and Via RICA- έκδοση 2015).

Όσον αφορά τη στιγμή που πρέπει να γίνεται η αφαίρεση των τριχών, δεν υπάρχουν στοιχεία ότι η αφαίρεση κοντά στο χρόνο της επέμβασης μειώνει τις λοιμώξεις, αλλά οι τρέχουσες συστάσεις προτείνουν ότι, σε περίπτωση που αποφασιστεί η αφαίρεση των τριχών, είναι προτιμότερο να γίνεται χρονικά κοντά στην επέμβαση αλλά πάντα εκτός χειρουργείου[6-7].

49. Οι τρίχες δεν πρέπει να αφαιρούνται προεγχειρητικά εκτός εάν είναι απολύτως απαραίτητο. Το συμβατικό ξύρισμα πρέπει να αποφεύγεται, τόσο προεγχειρητικά όσο και στο χειρουργείο.

Υψηλό επίπεδο τεκμηρίωσης. Ισχυρή σύσταση.

50. Σε περίπτωση αφαίρεσης των τριχών, οι ηλεκτρικές ξυριστικές μηχανές μπορούν να χρησιμοποιηθούν όσο το δυνατόν πιο κοντά χρονικά στην επέμβαση αλλά πάντα εκτός χειρουργείου.

Μέτριο επίπεδο τεκμηρίωσης. Ισχυρή σύσταση.

ΒΙΒΛΙΟΓΡΑΦΙΚΕΣ ΑΝΑΦΟΡΕΣ

1. Allegranzi B, Bischoff P, de Jonge S, et al. New WHO recommendations on preoperative measures for surgical site infection prevention: an evidence-based global perspective. Lancet Infect Dis. 2016;16(12):e276-e287. doi:10.1016/S1473-3099(16)30398-X.

2. Dohmen PM, Konertz W. A review of current strategies to reduce intraoperative bacterial contamination of surgical wounds. GMS Krankenhhyg Interdiszip. 2007;2(2):Doc38. http://www.ncbi.nlm.nih.gov/pubmed/20204082.

3. Lefebvre A, Saliou P, Lucet JC, et al. Preoperative hair removal and surgical site infections: network meta-analysis of randomized controlled trials. J Hosp Infect. 2015;91(2):100- 108. doi:10.1016/j.jhin.2015.06.020.

4. Tanner J, Norrie P, Melen K. Preoperative hair removal to reduce surgical site infection. Cochrane Database Syst Rev. 2011;(11):CD004122. doi:10.1002/14651858.cd004122.pub4.
5. Shi D, Yao Y, Yu W. Comparison of preoperative hair removal methods for the reduction of surgical site infections: a meta-analysis. J Clin Nurs. 2017;26(19-20):2907-2914. doi: 10.1111/jocn.13661.
6. Edmiston CEJ, Griggs RK, Tanner J, Spencer M, Seabrook GR, Leaper D. Perioperative hair removal in the 21st century: Utilizing an innovative vacuum-assisted technology to safely expedite hair removal before surgery. Am J Infect Control. 2016;44(12):1639-1644. doi:10.1016/j.ajic.2016.03.071.
7. JBI (Joanna Briggs Institute). Pre-operative hair removal to reduce surgical site infection. Best Practice 2007;11(4).

7.1.3 ΔΙΕΓΧΕΙΡΗΤΙΚΑ

Λίστα Ελέγχου (Checklist)

Τα αποτελέσματα της συστηματικής ανασκόπησης που πραγματοποιήθηκε έως τον Απρίλιο του 2015 στο πρόγραμμα Safe Surgery[1], δείχνουν σημαντική βελτίωση στους δείκτες ασφάλειας των ασθενών (μείωση των ποσοστών ανεπιθύμητων ενεργειών, της θνησιμότητας και της λοίμωξης του χειρουργικού τραύματος), μετά την εφαρμογή της χειρουργικής λίστας ελέγχου.

Λαμβάνοντας υπόψη δημοσιεύσεις του 2015, οι περισσότερες μελέτες δείχνουν επίσης μείωση των ανεπιθύμητων ενεργειών που σχετίζονται με χειρουργική επέμβαση καθώς και της νοσοκομειακής θνησιμότητας, αν και υπάρχει ετερογένεια μεταξύ των διαφορετικών μελετών (χειρουργεία και ειδικότητες, ύπαρξη ταυτόχρονων ομάδων ελέγχου, συνύπαρξη άλλων μέτρων βελτίωσης, κ.λπ.)[2-4].

51. Η χρήση της χειρουργικής λίστας ελέγχου συνιστάται για την πρόληψη των ανεπιθύμητων ενεργειών και της θνησιμότητας που σχετίζονται με την επέμβαση.

Μέτριο επίπεδο τεκμηρίωσης. Ισχυρή σύσταση.

ΒΙΒΛΙΟΓΡΑΦΙΚΕΣ ΑΝΑΦΟΡΕΣ

1. Programa de Cirugía Segura del Sistema Nacional de Salud. Ministerio de Sanidad, Servicios Sociales e Igualdad. 2016.

2. De Jager E, McKenna C, Bartlett L3, Gunnarsson R, Ho YH. postoperative Adverse Events Inconsistently Improved by The World Health Organization Surgical Safety Checklist: A Systematic Literature Review of 25 Studies. World J Surg. 2016 Aug;40(8):1842-58. doi: 10.1007/s00268-016-3519-9.

3. Abbott TEF, Ahmad T, Phull MK, Fowler AJ, Hewson R, Biccard BM, Chew MS, Gillies M, Pearse RM; International Surgical Outcomes Study (ISOS)group.The surgical safety checklist and patient outcomes after surgery: prospective observational cohort study, systematic review and meta-analysis.

Br J Anaesth. 2018 Jan;120(1):146-155. doi: 10.1016/j. bja.2017.08.002. Epub 2017 Nov 23.
4. Biccard BM, Rodseth R, Cronje L, Agaba P, Chikumba E, Du Toit L, Farina Z, Fischer S, Gopalan PD, Govender K, Kanjee J, Kingwill A, Madzimbamuto F, Mashava D, Mrara B, Mudely M, Ninise E, Swanevelder J, Wabule A.A meta-analysis of the efficacy of preoperative surgical safety checklists to improve perioperative outcomes. S Afr Med J. 2016 May 9;106(6). doi: 10.7196/SAMJ.2016.v106i6.9863.

Προετοιμασία του δέρματος και του χειρουργικού πεδίου

Για τη σωστή προετοιμασία της περιοχής της επέμβασης, πρέπει να λάβουμε υπόψη τη σημασία του καθαρισμού του δέρματος με σαπούνι και νερό πριν την εφαρμογή του αντισηπτικού διαλύματος, ακολουθούμενο από ξέβγαλμα και στέγνωμα ολόκληρου του δέρματος, και με αλατούχο διάλυμα σε βλεννογόνους και πληγές.

Η απολύμανση του δέρματος πριν από την οριοθέτηση του χειρουργικού πεδίου πρέπει να πραγματοποιείται με κινήσεις προς τα εμπρός και προς τα πίσω, με τρίψιμο και με τριβή σε οριζόντιες και κάθετες ταινίες. Η αλκοολική χλωρεξιδίνη 2% πρέπει να χρησιμοποιείται για τουλάχιστον 30 δευτερόλεπτα σε όλες τις τομές που γίνονται σε άθικτο δέρμα και είναι σημαντικό να αφήνεται το αντισηπτικό να στεγνώσει για 2 λεπτά για να έχει χρόνο να ενεργήσει[1-2] (Zero Surgical Infection Project).

Τα αλκοολούχα αντισηπτικά είναι εύφλεκτες ουσίες και, ως εκ τούτου, πρέπει να διασφαλίζεται ότι το δέρμα είναι εντελώς στεγνό και ότι δεν υπάρχουν συσσωρευμένες ποσότητες στις πτυχές του δέρματος του ασθενούς ή στις γάζες και τα οθόνια του χειρουργικού πεδίου κάτω από τον ασθενή[3].

Σε επεμβάσεις στον οφθαλμό, στο μέσο ους και στις μήνιγγες και σε εκείνες των οποίων η προσπέλαση είναι δια του βλεννογόνου (στοματικό, ρινικό, ουρηθρικό, κολπικό, πρωκτικό), θα πρέπει να χρησιμοποιείται αραιή υδατική χλωρεξιδίνη (0,5%) ή ιωδιούχος ποβιδόνη (10%), ανάλογα με την περίπτωση[4].

52. Η χρήση αλκοολικής χλωρεξιδίνης 2% συνιστάται ως αντισηπτικό για άθικτο δέρμα στο χειρουργικό πεδίο.

Υψηλό επίπεδο τεκμηρίωσης. Ισχυρή σύσταση.

ΒΙΒΛΙΟΓΡΑΦΙΚΕΣ ΑΝΑΦΟΡΕΣ

1. Privitera GP, Costa AL, Brusaferro S, Chirletti P, Crosasso P, Massimetti G, et al. Skin antisepsis with chlorhexidine versus iodine for the prevention of surgical site infection: A systematic review and meta-analysis. Am J Infect Control. 2017; 45(2):180-189. doi: 10. 1016/j.ajic.2016.09.017.

2. Bratzler DW, Houck PM. Antimicrobial prophylaxis for surgery: an advisory statement from the National Surgical Infection Prevention Project. Clin Infect Dis. 2004 Jun 15; 38(12):1706- 15. doi: 10.1086/421095.

3. Hsieh CS, Cheng HC, Lin JS, Kuo SJ, Chen YL. Effect of 4% chlorhexidine gluconate predisinfection skin scrub prior to hepatectomy: a double-blinded, randomized control study. Int Surg. 2014 Nov-Dec; 99(6):787-94. doi: 10.9738/INTSURG-D-13-00179.1.

4. Darouiche RO, Wall MJ Jr, Itani KM, Otterson MF, Webb AL, Carrick MM. Chlorhexidine-Alcohol versus Povidone-Iodine for Surgical-Site Antisepsis. N Engl J Med. 2010 Jan 7; 362 (1):18-26. doi: 10.1056/NEJMoa0810988.

Εισαγωγή στην αναισθησία και διατήρηση της αναισθησίας

Απαιτείται ένα σταθερό πρωτόκολλο αναισθησίας για να επιτραπεί η ταχεία αφύπνιση. Ο αναισθησιολόγος πρέπει να παρακολουθεί τη θεραπεία με υγρά, την αναλγησία και την αιμοδυναμική σταθερότητα για τη μείωση της μεταβολικής απάντησης στο στρες.

Η χρήση βενζοδιαζεπινών πριν από την εισαγωγή θα πρέπει να είναι μόνο για τη μείωση του άγχους και στη χαμηλότερη δυνατή δόση, με σκοπό τη μείωση των επεισοδίων παραληρήματος και μετεγχειρητικής γνωστικής δυσλειτουργίας, ειδικά σε ηλικιωμένους και με πολλαπλές συννοσηρότητες ασθενείς υψηλού κινδύνου[1].

Επί του παρόντος, χρησιμοποιούνται αναισθητικά και αναλγητικά φάρμακα με ελάχιστη υπολειμματική δράση που επιτρέπουν και την ταχεία ανάρρωση μετά την αναισθησία: προποφόλη, σε συνδυασμό, εάν είναι

απαραίτητο, με ένα οπιοειδές βραχείας δράσης όπως φαιντανύλη, αλφαιντανύλη, σουφαιντανύλη ή έγχυση ρεμιφαιντανύλης.

Η αναισθησία μπορεί να διατηρηθεί με εισπνεόμενα αναισθητικά βραχείας δράσης, όπως το σεβοφλουράνιο ή το δεσφλουράνιο (εισαγωγή στην αναισθησία και αφύπνιση γρηγορότερα από το σεβοφλουράνιο) ή με ενδοφλέβια όπως η προποφόλη. Δεν υπάρχουν στοιχεία για την υπεροχή της ολικής ενδοφλέβιας αναισθησίας με προποφόλη έναντι της αναισθησίας με εισπνεόμενα αναισθητικά, αν και η ολική ενδοφλέβια αναισθησία με προποφόλη μπορεί να είναι ευεργετική σε ασθενείς με ευαισθησία σε μετεγχειρητική ναυτία και έμετο. Δεν υπάρχουν επίσης στοιχεία ότι η ολική ενδοφλέβια αναισθησία με προποφόλη βελτιώνει την ογκολογική πρόγνωση στους ασθενείς σε σχέση με την αναισθησία με εισπνεόμενα αναισθητικά[3].

Αυτή η σύσταση εμφανίζεται και στην πρώτη έκδοση του Intensified Recovery Pathway in Abdominal Surgery (RICA) και βασίζεται κυρίως στη συναίνεση των ειδικών[4].

53. Συνιστάται η ελαχιστοποίηση της χρήσης βενζοδιαζεπίνων πριν από την εισαγωγή στην αναισθησία και η χρήση υπνωτικών παραγόντων με ελάχιστη υπολειμματική δράση, που επιτρέπουν την ταχεία ανάρρωση μετά την αναισθησία.

Χαμηλό επίπεδο τεκμηρίωσης. Ισχυρή σύσταση.

ΒΙΒΛΙΟΓΡΑΦΙΚΕΣ ΑΝΑΦΟΡΕΣ

1. Apfel CC, Korttila K, Abdalla M, Kerger H, Turan A, Vedder I, et al. A factorial trial of six interventions for the prevention of postoperative nausea and vomiting. N Engl J Med 2004;350(24):2441-51.

2. Ren L, Zhu D, Wei Y, Pan X, Liang L, Xu J, et al. Enhanced Recovery After Surgery (ERAS) Program Attenuates Stress and Accelerates Recovery in Patients After Radical Resection for Colorectal Cancer: A Prospective Randomized Controlled Trial. World J Surg 2012;36(2):407-14.

3. Gustafsson UO, Scott MJ, Hubner M, Nygren J, Demartines N, Francis N, et al. Guidelines for Perioperative Care in Elective Colorectal Surgery: Enhanced Recovery After Surgery (ERAS®) Society Recommendations: 2018. World J Surg 2019;43(3):659-95.

4. Grupo de Trabajo. Vía Clínica de Recuperación Intensificada en Cirugía Abdominal (RICA). Madrid: Ministerio de Sanidad, Servicios Sociales e Igualdad. Madrid; 2015.

Προστατευτικός αερισμός των πνευμόνων

Τα πιθανά οφέλη του προστατευτικού αερισμού που παρατηρούνται σε ασθενείς που εισάγονται σε μονάδες εντατικής θεραπείας με σύνδρομο αναπνευστικής δυσχέρειας ενηλίκων είναι λιγότερο εμφανή όταν οι ασθενείς δεν έχουν σοβαρή πνευμονική νόσο και αερίζονται για λίγες ώρες. Ο προστατευτικός αερισμός έχει τρεις θεμελιώδεις πυλώνες που τον υποστηρίζουν: τη χρήση χαμηλών αναπνεόμενων όγκων (Tidal Volume - TV), την εφαρμογή χειρισμών στρατολόγησης των κυψελίδων (Alveolar Recruitment Maneuvers – ARM) και την εφαρμογή θετικής τελοεκπνευστικής πίεσης, (Positive End Expiratory Pressure – PEEP), καλύτερα εξατομικευμένης. Λίγες μελέτες έχουν διερευνήσει την αποτελεσματικότητα της κοινής χρήσης αυτών των χειρισμών στη μετεγχειρητική πρόγνωση σε ασθενείς που υποβάλλονται σε γενική αναισθησία με μηχανικό αερισμό και χωρίς αναπνευστική δυσχέρεια. Μια σημαντική μελέτη έδειξε μείωση των μετεγχειρητικών πνευμονικών επιπλοκών με τη χρήση αυτής της στρατηγικής διεγχειρητικού πνευμονικού αερισμού σε συνδυασμό με την εφαρμογή μετεγχειρητικής συνεχούς θετικής πίεσης αεραγωγών (Continuous Positive Airway Pressure – CPAP) σε σύγκριση με την ομάδα ελέγχου (τυπικός διεγχειρητικός αερισμός και χωρίς CPAP μετεγχειρητικά)[1]. Αν και άλλες μεγάλες τυχαιοποιημένες ελεγχόμενες μελέτες απέτυχαν να καταδείξουν τα οφέλη του προστατευτικού αερισμού[2,3] μια πρόσφατη μετα-ανάλυση έδειξε ότι ο συνδυασμός χαμηλού αναπνεόμενου όγκου και μετρίως υψηλής PEEP (> 5 cm H2O), με ή χωρίς ARM, ήταν ανώτερος από τον συμβατικό μηχανικό αερισμό στη μείωση του κινδύνου καρδιογενούς πνευμονικού οιδήματος. Η εφαρμογή του ARM σε αυτή τη στρατηγική μειώνει τον κίνδυνο ατελεκτασιών[4]. Επιπλέον, στις θωρακοχειρουργικές επεμβάσεις, κατά τον αερισμό ενός πνεύμονα, έχει προταθεί η στρατηγική προστασίας των πνευμόνων, χρησιμοποιώντας αναπνεόμενο όγκο 4-6 ml/kg ιδανικού βάρους, PEEP και ARM, αν και τα

αποτελέσματά της δεν ήταν οριστικά σε σχέση με την επίδρασή της στη μετεγχειρητική έκβαση[5,6].

54. Κατά τη γενική αναισθησία, συνιστάται η χρήση προστατευτικού αερισμού, συμπεριλαμβανομένου αναπνεόμενου όγκου 6–8 ml/kg ιδανικού βάρους, η χρήση εξατομικευμένου PEEP γενικά άνω των 5 cm H2O και η εφαρμογή ARM.

Μέτριο επίπεδο τεκμηρίωσης. Ισχυρή σύσταση.

55. Σε χειρουργικές επεμβάσεις που απαιτούν αερισμό με έναν πνεύμονα, συνιστούμε τα παραπάνω προστατευτικά μέτρα αερισμού, αλλά μείωση του αναπνεόμενου όγκου στα 4–6 ml/kg ιδανικού βάρους.

Μέτριο επίπεδο τεκμηρίωσης. Ισχυρή σύσταση.

ΒΙΒΛΙΟΓΡΑΦΙΚΕΣ ΑΝΑΦΟΡΕΣ

1. Ferrando C, Soro M, Unzueta C, Suarez-Sipmann F, Canet J, Librero J, et al. Network Individualised perioperative open-lung approach versus standard protective ventilation in abdominal surgery (iPROVE): a randomised controlled trial. Lancet Respir Med.2018;6(3):193.

2. Hemmes SN, Gama de Abreu M, Pelosi P, Schultz MJ. High versus low positive end-expiratory pressure during general anaesthesia for open abdominal surgery (PROVHILO trial): a multicentre randomised controlled trial. Lancet.2014;384(9942):495-503.

3. Bluth T, Serpa Neto A, Schultz M J, Pelosi P, Gama de Abreu M, Bobek I, et al. Effect of Intraoperative High Positive End-Expiratory Pressure (PEEP) With Recruitment Maneuvers vs Low PEEP on Postoperative Pulmonary Complications in Obese Patients: A Randomized Clinical Trial. JAMA,2019;321:2292-305.

4. Deng QW. Intraoperative Ventilation Strategies to Prevent Postoperative Pulmonary Com- plications: A Network Meta-Analysis of Randomised Controlled Trials Br J Anaesth. 2020;124:324-35.

5. Liu Z, Liu X, Huang Y, Zhao J.I ntraoperative mechanical ventilation strategies in patients undergoing one-lung ventilation: a meta-analysis. Springerplus.2016; 5:125.

6. El Tahan MR, Pasin L, Marczin N, Landoni G. Impact of low tidal volumes during one lung ventilation. A meta-analysis of randomised controlled trials J Cardiothorac Vasc Anesth 2017;31:1767-73.

Διεγχειρητική Παρακολούθηση

Η παρακολούθηση ρουτίνας θα πρέπει να περιλαμβάνει ηλεκτροκαρδιογράφημα 5 απαγωγών (συνιστώνται οι απαγωγές II και V5), μη επεμβατική αρτηριακή πίεση, παλμική οξυμετρία (% Sat O2), FiO_2, καπνογραφία ($PETCO_2$), θερμοκρασία, ισορροπία χορήγησης υγρών και διεγχειρητική γλυκόζης αίματος.

Παρακολούθηση του CO_2

Η παρακολούθηση του CO_2 με καπνογραφία είναι απαραίτητη σε κάθε επέμβαση με γενική αναισθησία για να εξασφαλιστεί η ανταλλαγή αερίων του ασθενούς και η επαρκής διαχείριση του αεραγωγού και να αποκλειστεί η τυχαία αποδιασωλήνωση[1].

Στη λαπαροσκοπική χειρουργική, όπου γίνεται εμφύσηση CO_2 για τη δημιουργία του πνευμοπεριτοναίου, γίνεται απορρόφηση αυτού του αερίου από τον οργανισμό και η παρακολούθηση του CO_2 μπορεί να αποτελέσει ένδειξη επιπλοκών λόγω υπερκαπνίας[2].

56. Η παρακολούθηση του CO_2 με καπνογραφία θα πρέπει να είναι υποχρεωτική σε όλες τις χειρουργικές επεμβάσεις, ιδιαίτερα στη λαπαροσκοπική χειρουργική.

Υψηλό επίπεδο τεκμηρίωσης. Ισχυρή σύσταση.

ΒΙΒΛΙΟΓΡΑΦΙΚΕΣ ΑΝΑΦΟΡΕΣ

1. Lam T, Nagappa M, Wong J, Singh M, Wong D, Chung F. Continuous Pulse Oximetry and Capnography Monitoring for Postoperative Respiratory Depression and Adverse Events: A Systematic Review and Meta-analysis. Anesth Analg. 2017;125:2019-2029.

2. Frerk C, Mitchell VS, McNarry AF, Mendonca C, Bhagrath R, Patel A et al. Difficult Airway Society 2015 guidelines for management of unanticipated difficult intubation in adults. Br J Anaesth2015;115(6):827-48.1.

Παρακολούθηση της θερμοκρασίας

Η παρακολούθηση της θερμοκρασίας είναι υποχρεωτική για την αποφυγή υποθερμίας ή υπερθερμίας στον ασθενή κατά την περιεγχειρητική περίοδο, παρά τα διαφορετικά εξωτερικά μέτρα για τη διατήρηση της νορμοθερμίας. Ο έλεγχος θερμοκρασίας είναι αξιόπιστος μόνο εάν η μέτρηση γίνεται κεντρικά[1-3].

57. Η παρακολούθηση της θερμοκρασίας πρέπει να είναι κεντρική.

Υψηλό επίπεδο τεκμηρίωσης. Ισχυρή σύσταση.

ΒΙΒΛΙΟΓΡΑΦΙΚΕΣ ΑΝΑΦΟΡΕΣ

1. Urits I, Jones MR, Orhurhu V, Sikorsky A, Seifert D, Flores C, et al. A Comprehensive Update of Current Anesthesia Perspectives on Therapeutic Hypothermia. Adv Ther 2019;36(9): 2223-2232.2.

2. Calvo Vecino JM, Casans Francés R, Ripollés Melchor J, Marín Zaldívar C, Gómez Ríos MA, Pérez Ferrer A, et al. No Intencionada de la SEDAR. Clinical practice guideline. Unintentional perioperative hypothermia. Rev Esp Anestesiol Reanim. 2018;65(10):564-588.

3. Madden LK, Hill M, May TL, Human T, Guanci MM, Jacobi J, Moreda MV, et al. The Imple- mentation of Targeted Temperature Management: An Evidence-Based Guideline from the Neurocritical Care Society. Neurocrit Care. 2017;27(3):468-487.

Παρακολούθηση του βάθους της αναισθησίας – ύπνωσης

Επί του παρόντος, υπάρχουν στην αγορά διαφορετικά συστήματα παρακολούθησης της ύπνωσης για τη μέτρηση του βάθους της γενικής αναισθησίας, υπογραμμίζοντας τα επιστημονικά δεδομένα για τον διφασματικό δείκτη (Bispectral Index) BIS τόσο από τον αριθμό των δημοσιεύσεων, τον

αριθμό των ασθενών που μελετήθηκαν και τη συσσωρευμένη εμπειρία τόσο σε ενήλικες όσο και σε παιδιά[1,2].

Η εισαγωγή και η διατήρηση της αναισθησίας μπορεί να καθοδηγείται από την οθόνη (monitor) BIS, αποφεύγοντας έτσι τα επίπεδα υπερβολικού βάθους ύπνωσης (BIS <30), ειδικά στους ηλικιωμένους, όπου υπάρχουν ενδείξεις ότι η πολύ βαθιά αναισθησία μπορεί να είναι επιβλαβής και μπορεί να αυξήσει τον κίνδυνο μετεγχειρητικής σύγχυσης. Η δόση του αναισθητικού φαρμάκου κατά τη διάρκεια της γενικής αναισθησίας θα πρέπει να προσαρμοστεί ώστε να ληφθούν τιμές BIS μεταξύ 40 και 60[3,4].

58. Το βάθος της αναισθησίας θα πρέπει να παρακολουθείται χρησιμοποιώντας τον διφασματικό δείκτη (BIS).

Υψηλό επίπεδο τεκμηρίωσης. Ισχυρή σύσταση.

ΒΙΒΛΙΟΓΡΑΦΙΚΕΣ ΑΝΑΦΟΡΕΣ

1. Luo C, Zou W. Cerebral monitoring of anaesthesia on reducing cognitive dysfunction and postoperative delirium: a systematic review. J Int Med Res. 2018;46(10):4100-4110.

2. Punjasawadwong Y, Chau-In W, Laopaiboon M, Punjasawadwong S, Pin-On P. Processed electroencephalogram and evoked potential techniques for amelioration of postoperative delirium and cognitive dysfunction following non-cardiac and non-neurosurgical procedures in adults. Cochrane Database Syst Rev 2018;5:Cd011283.3.

3. Oliveira CR, Bernardo WM, Nunes VM. Benefit of general anesthesia monitored by bispectral index compared with monitoring guided only by clinical parameters. Systematic review and meta-analysis. Braz J Anesthesiol. 2017;67(1):72-84.

4. Chhabra A, Subramaniam R, Srivastava A, Prabhakar H, Kalaivani M, Paranjape S. Spectral entropy monitoring for adults and children undergoing general anaesthesia. Cochrane Database Syst Rev. 2016 Mar 14;3:CD010135.

Παρακολούθηση της Αλγαισθησίας (Nociception – Αίσθησης του πόνου)

Επί του παρόντος, υπάρχουν στην αγορά διαφορετικά συστήματα παρακολούθησης του χειρουργικού στρες και της οργανικής απάντησης σε αυτό. Υπάρχει μεγάλη ετερογένεια των συστημάτων παρακολούθησης, με τα κυριότερα να είναι αυτά που βασίζονται στις αποκρίσεις από το αυτόνομο νευρικό σύστημα και στο ηλεκτροεγκεφαλογράφημα – ΗΕΓ (Σύνθετος δείκτης μεταβλητότητας - Composite Variability Index CVI - που προέρχεται από το BIS ή το qCon/qNox). Μέχρι στιγμής, τα πιο ειδικά και ευαίσθητα είναι αυτά που βασίζονται στην ανίχνευση αλλαγών στο συμπαθητικό αυτόνομο νευρικό σύστημα: μέτρηση της διαμέτρου της κόρης του οφθαλμού με αλλοιωμένο αντανακλαστικό της κόρης (Pupillometry), τα πολυπαραμετρικά όπως ο Ολοκληρωμένος Δείκτης Επιπέδου Αλγαισθησίας - Integrated Nociception Level Index (NOL) - (πληθυσμογραφία, θερμοκρασία, επιταχυνσιομετρία και αντίσταση δέρματος – impedance) και ο Δείκτης Χειρουργικής Πληθυσμογραφίας - Surgical Plethysmographic Index (SPI) με βάση τις αλλαγές του παλμικού κύματος πληθυσμογραφίας και ο Δείκτης Αναλγησίας Αλγαισθησίας - Analgesia Nociception Index (ANI) - που εξαρτάται από το ηλεκτροκαρδιογράφημα – ΗΚΓ και την επίδραση του παρασυμπαθητικού στον καρδιακό ρυθμό[1-6].

Η διεγχειρητική κατανάλωση οπιοειδών θα μπορούσε γενικά να είναι μικρότερη αν καθοδηγείται από την παρακολούθηση της αλγαισθησίας σε σύγκριση με την τυπική παρακολούθηση της διακύμανσης του καρδιακού ρυθμού και της αρτηριακής πίεσης[1-2].

Επί του παρόντος δεν φαίνεται να υπάρχουν στατιστικά σημαντικές διαφορές σε σχέση με τις διεγχειρητικές ανεπιθύμητες ενέργειες, τη μετεγχειρητική χρήση οπιοειδών ή αναλγητικών, τον μετεγχειρητικό πόνο και τις μετεγχειρητικές ανεπιθύμητες ενέργειες[2].

59. Η χρήση της παρακολούθησης της αλγαισθησίας θα μπορούσε να μειώσει την διεγχειρητική κατανάλωση οπιοειδών σε σύγκριση με την τυπική παρακολούθηση.

Μέτριο επίπεδο τεκμηρίωσης. Ασθενής σύσταση

ΒΙΒΛΙΟΓΡΑΦΙΚΕΣ ΑΝΑΦΟΡΕΣ

1. Jiao Y, He B, Tong X, Xia R, Zhang C, Shi X. Intraoperative monitoring of nociception for opioid administration: a meta-analysis of randomized controlled trials. Minerva Anestesiol. 2019;85(5):522-530.

2. Meijer FS, Niesters M, van Velzen M, Martini CH, Olofsen E, Edry R, et al. Does nociception monitor-guided anesthesia affect opioid consumption? A systematic review of randomized controlled trials. J Clin Monit Comput 2019. doi: 10.1007/s10877-019-00362-4.

3. Gruenewald M, Dempfle A. Analgesia/nociception monitoring for opioid guidance: meta-analysis of randomized clinical trials. Minerva Anestesiol. 2017;83(2):200- 213.

4. Won YJ1, Lim BG1, Kim YS1, Lee M1, Kim H1. Usefulness of surgical pleth index-guided analgesia during general anesthesia: a systematic review and meta-analysis of randomized controlled trials. J Int Med Res. 2018;46(11):4386-4398.

5. Banerjee S, MacDougall D. Nociception Monitoring for General Anesthesia: A Review of Clinical Effectiveness, Cost-Effectiveness, and Guidelines. Ottawa (ON): Canadian Agency for Drugs and Technologies in Health; 2018 Dec 12. CADTH Rapid Response Reports.

6. Abad-Gurumeta A, Ripollés-Melchor J, Casans-Francés R, Calvo-Vecino JM. Monitoring of nociception, reality or fiction? Rev Esp Anestesiol Reanim. 2017;64:406-414.

Παρακολούθηση της παραγωγής ούρων και τοποθέτηση ουροκαθετήρα

Σε όσες χειρουργικές επεμβάσεις πρέπει να παρακολουθείται η ισορροπία των υγρών ή για λόγους που σχετίζονται με το είδος της επέμβασης, συνιστάται η τοποθέτηση καθετήρα ουροδόχου κύστης. Είναι απαραίτητο να αφαιρεθεί το συντομότερο δυνατό για τη μείωση των ουρολοιμώξεων και τη διευκόλυνση της έγκαιρης κινητοποίησης των ασθενών[1-3].

60. Όταν τοποθετηθεί καθετήρας ουροδόχου κύστης, θα γίνει με τα κατάλληλα άσηπτα μέτρα και, αν είναι δυνατόν, θα αφαιρεθεί 24 ώρες μετά την επέμβαση.

Μέτριο επίπεδο τεκμηρίωσης. Ασθενής σύσταση

ΒΙΒΛΙΟΓΡΑΦΙΚΕΣ ΑΝΑΦΟΡΕΣ

1. Patel DN, Felder SI, Luu M, Daskivich TJ, K NZ, Fleshner P. Early Urinary Catheter Removal Following Pelvic Colorectal Surgery: A Prospective, Randomized, Noninferiority Trial. Dis Colon Rectum 2018;61(10):1180-1186.

2. Alyami M, Lundberg P, Passot G, Glehen O, Cotte E. Laparoscopic Colonic Resection Without Urinary Drainage: Is It "Feasible"? J Gastrointest Surg 2016;20(7):1388-92.

3. Zhang P, Hu WL, Cheng B, Cheng L, Xiong XK, Zeng YJ. A systematic review and meta-analysis comparing immediate and delayed catheter removal following uncomplicated hysterectomy. Int Urogynecol J 2015;26(5):665-74.

Ουροκαθετήρας

Σε μείζονες χειρουργικές επεμβάσεις, ο καθετηριασμός της ουροδόχου κύστης είναι κοινός με σκοπό τον έλεγχο της διούρησης, καθώς και για την αποφυγή παρατεταμένης επίσχεσης ούρων στην ουροδόχο κύστη. Η διατήρηση του καθετήρα κατά τη μετεγχειρητική περίοδο σχετίζεται με ενοχλήσεις για τον ασθενή, καθώς και με ουρολοιμώξεις[1].

61. Η αφαίρεση του ουροκαθετήρα συνιστάται σε 24 ώρες, εκτός από ασθενείς με μέτριο κίνδυνο οξείας επίσχεσης ούρων – άνδρες, επισκληρίδιος αναισθησία και χειρουργική επέμβαση πυέλου – οπότε συνιστάται η διατήρησή του για 3 ημέρες[2,3].

Υψηλό επίπεδο τεκμηρίωσης. Ισχυρή σύσταση.

ΒΙΒΛΙΟΓΡΑΦΙΚΕΣ ΑΝΑΦΟΡΕΣ

1. Wald HL, Ma A, Bratzler DW, Kramer AM. Indwelling urinary catheter use in the postoperative period: analysis of the national surgical infection prevention project data. Arch Surg. 2008 jun;143(6):551-557.

2. Gustafsson UO, Scott MJ, Hubner M, Nygren J, Demartines N, Francis N, Rockall TA, Young-Fadok TM, Hill AG, Soop M, de Boer HD, Urman RD, Chang

GJ, Fichera A, Kessler H, Grass F, Whang EE, Fawcett WJ, Carli F, Lobo DN, Rollins KE, Balfour A, Baldini G, Riedel B, Ljungqvist O. Guidelines for Perioperative Care in Elective Colorectal Surgery: Enhanced Recovery After Surgery (ERAS®) Society Recommendations: 2018. World J Surg. 2019 Mar;43(3):659-695.

3. Carmichael JC, Keller DS, Baldini G, Bordeianou L, Weiss E, Lee L, Boutros M, McClane J, Feldman LS, Steele SR. Clinical Practice Guidelines for Enhanced Recovery After Colon and Rectal Surgery from the American Society of Colon and Rectal Surgeons and Society of American Gastrointestinal and Endoscopic Surgeons. Dis Colon Rectum. 2017 Aug;60(8):761-784.

ΜΗ ΤΥΠΙΚΗ ΠΑΡΑΚΟΛΟΥΘΗΣΗ (NON-ROUTINE MONITORING)

Επεμβατική παρακολούθηση της αρτηριακής πίεσης

Η πρόοδος στην αιμοδυναμική παρακολούθηση έχει επιτρέψει την λιγότερο επεμβατική παρακολούθηση, τη μείωση της συχνότητας τοποθέτησης καθετήρα στην κερκιδική ή μηριαία αρτηρία για τον έλεγχο της συνεχούς αρτηριακής πίεσης και της καρδιακής παροχής, καθώς και άλλων σχετικών δεικτών με αξιόπιστο τρόπο[1-4]. Επί του παρόντος, μόνο οι ασθενείς με υψηλό χειρουργικό κίνδυνο και υψηλό αναισθησιολογικό κίνδυνο με ιστορικό αιμοδυναμικής αστάθειας λόγω ηλικίας και συννοσηρότητων είναι υποψήφιοι για επεμβατική αρτηριακή παρακολούθηση[1-3].

Αυτή η σύσταση εμφανίζεται στην πρώτη έκδοση του Intensified Recovery Pathway in Abdominal Surgery (RICA) και βασίζεται κυρίως στη συναίνεση των ειδικών[5]. Περιλαμβάνεται επίσης στο πρόγραμμα Zero Surgical Infection[6].

62. Η επεμβατική αιμοδυναμική παρακολούθηση δεν ενδείκνυται συνήθως και ο αρτηριακός καθετηριασμός είναι χρήσιμος σε εκείνους τους ασθενείς που παρουσιάζουν σοβαρές καρδιοαναπνευστικές αλλοιώσεις και που μπορεί να παρουσιάσουν μετεγχειρητικά προβλήματα.

Χαμηλό επίπεδο τεκμηρίωσης. Ισχυρή σύσταση

ΒΙΒΛΙΟΓΡΑΦΙΚΕΣ ΑΝΑΦΟΡΕΣ

1. Scheeren TWL, Ramsay MAE. New Developments in Hemodynamic Monitoring. J Cardiothorac Vasc Anesth 2019;33 Suppl 1:S67-s72.

2. Jozwiak M, Monnet X, Teboul JL. Less or more hemodynamic monitoring in critically ill patients. Curr Opin Crit Care 2018;24(4):309-315.

3. Yamada T, Vacas S, Gricourt Y, Cannesson M. Improving Perioperative Outcomes Through Minimally Invasive and Non-invasive Hemodynamic Monitoring Techniques. Front Med (Lausanne) 2018;5:144.

4. Teboul JL, Saugel B, Cecconi M, De Backer D, Hofer CK, Monnet X et al. Less invasive hemodynamic monitoring in critically ill patients. Intensive Care Med 2016;42(9):1350-9.

5. Grupo de Trabajo. Vía Clínica de Recuperación Intensificada en Cirugía Abdominal (RICA). Madrid: Ministerio de Sanidad, Servicios Sociales e Igualdad. Madrid; 2015.

6. Proyecto Infección Quirúrgica Zero del Sistema Nacional de Salud. Sociedad Española de Medicina Preventiva, Salud Pública e Higiene. 2016.

Παρακολούθηση της κεντρικής φλεβικής πίεσης

Η πρόοδος στα μη επεμβατικά συστήματα αιμοδυναμικής παρακολούθησης έχει μειώσει τον αριθμό των ασθενών που χρειάζονται έλεγχο της κεντρικής φλεβικής πίεσης - ΚΦΠ (central venous pressure - CVP). Μόνο σε χειρουργικές επεμβάσεις με υψηλό κίνδυνο αιμορραγίας, με υψηλή πιθανότητα μετάγγισης παραγώγων αίματος, με σημαντικές αιμοδυναμικές αλλοιώσεις που σχετίζονται με υψηλό χειρουργικό κίνδυνο και την ανάγκη για χορήγηση αγγειοσυσπαστικών ή ινότροπων φαρμάκων, και με την ανάγκη για χορήγηση παρεντερικής διατροφής, θα μπορούσε να δικαιολογηθεί η ανάγκη για εισαγωγή κεντρικού φλεβικού καθετήρα και παρακολούθησης της ΚΦΠ[1-4].

Αυτή η σύσταση εμφανίζεται στην πρώτη έκδοση του Clinical Pathway for Intensified Recovery in Abdominal Surgery (RICA) και βασίζεται κυρίως στη συναίνεση των ειδικών[5]. Περιλαμβάνεται επίσης στο πρόγραμμα Zero Surgical Infection[6].

63. Η εισαγωγή κεντρικού φλεβικού καθετήρα (ΚΦΚ) δεν ενδείκνυται τακτικά και περιορίζεται σε ασθενείς με σοβαρές καρδιοαναπνευστικές παθήσεις με πνευμονική υπέρταση ή στους οποίους αναμένεται ότι μπορεί να απαιτήσουν τη χορήγηση αγγειοσυσπαστικών ή ινότροπων φαρμάκων σε συνεχή έγχυση.

Χαμηλό επίπεδο τεκμηρίωσης. Ισχυρή σύσταση.

ΒΙΒΛΙΟΓΡΑΦΙΚΕΣ ΑΝΑΦΟΡΕΣ

1. De Backer D, Vincent JL. The pulmonary artery catheter: is it still alive? Curr Opin Crit Care 2018;24(3):204-208.
2. Joseph C, Garrubba M, Smith JA, Melder A. Does the Use of a Pulmonary Artery Catheter Make a Difference During or After Cardiac Surgery? Heart Lung Circ 2018;27(8):952-960.
3. Youssef N, Whitlock RP. The Routine Use of the Pulmonary Artery Catheter Should Be Abandoned. Can JCardiol 2017;33(1):135-141.
4. Watson X, Cecconi M. Haemodynamic monitoring in the perioperative period: the past, the present and the future. Anaesthesia 2017;72 Suppl 1:7-15.
5. Grupo de Trabajo. Vía Clínica de Recuperación Intensificada en Cirugía Abdominal (RICA). Madrid: Ministerio de Sanidad, Servicios Sociales e Igualdad. Madrid; 2015.
6. Proyecto Infección Quirúrgica Zero del Sistema Nacional de Salud. Sociedad Española de Medicina Preventiva, Salud Pública e Higiene. 2016.

Νευρομυϊκός αποκλεισμός

Η ποσοτική παρακολούθηση του βαθμού του νευρομυϊκού αποκλεισμού (neuromuscular blockade – NMB), μέσω του διεγέρτη περιφερικών νεύρων, που ονομάζεται επίσης "τρένο των τεσσάρων" (“train of four” – TOF), κατά τη διάρκεια ολόκληρης της αναισθητικής διαδικασίας είναι απαραίτητη σε αυτούς τους ασθενείς που λαμβάνουν νευρομυϊκό αποκλεισμό και είναι η μόνη παρακολούθηση που μπορεί να καθορίσει με βεβαιότητα τον χρόνο της αποδιασωλήνωσης. Εκτός από το TOF, μπορεί να προσδιορισθεί ο λόγος TOF (TOFr – TOF ratio), το απλό ερέθισμα και η μετατετανική μέτρηση (post tetanic count – PTC). Παρόλο που μπορούν να χρησιμοποιηθούν και άλλοι μύες του προσώπου, ο προσαγωγός του αντίχειρα αντανακλά με ακρίβεια τη χαλαρή

κατάσταση των φαρυγγικών μυών. Η ποιοτική παρακολούθηση του νευρομυϊκού αποκλεισμού δεν είναι αξιόπιστη[1-6].

64. Η χρήση ποσοτικής παρακολούθησης του νευρομυϊκού αποκλεισμού είναι απαραίτητη κάθε φορά που χρησιμοποιούνται φάρμακα νευρομυϊκού αποκλεισμού καθ' όλη τη διάρκεια της χειρουργικής επέμβασης.

Υψηλό επίπεδο *τεκμηρίωσης. Ισχυρή σύσταση.*

ΒΙΒΛΙΟΓΡΑΦΙΚΕΣ ΑΝΑΦΟΡΕΣ

1. Hristovska AM, Duch P, Allingstrup M, Afshari A. The comparative efficacy and safety of sugammadex and neostigmine in reversing neuromuscular blockade in adults. A Cochrane systematic review with meta-analysis and trial sequential analysis. Anaesthesia. 2018; 73(5):631-641

2. Naguib M, Brull SJ, Kopman AF, Hunter JM, Fulesdi B, Arkes HR et al. Consensus Statement on perioperative use of neuromuscular monitoring. Anesth & Analg 2018;127:71-80.

3. Murphy GS. Neuromuscular monitoring in the perioperative period. Anesth & Analg 2018;126:464-68.

4. Brull SJ, Kopman AF. Current Status of Neuromuscular Reversal and Monitoring: Challenges and Opportunities. Anesthesiology. 2017;126(1):173-190.

5. Naguib M, Brull SJ, Johnson KB. Conceptual and technical insights into the basis of neuromuscular monitoring. Anaesthesia. 2017;72 Suppl 1:16-37.

6. Checketts MR, Alladi R, Ferguson K, Gemmell L, Handy JM, K AA Lein et al. Recommendations for standards of monitoring during anaesthesia and recovery 2015: Association

Βάθος του νευρομυϊκού αποκλεισμού

Ο βαθύς νευρομυϊκός αποκλεισμός είναι πολύ κατάλληλος για χειρουργική επέμβαση στην κοιλία (ανοικτή και λαπαροσκοπική) και για παχύσαρκους ασθενείς. Βελτιώνει τις συνθήκες του χειρουργικού χώρου κατά τη λαπαροσκόπηση και διευκολύνει τη χρήση χαμηλών ενδοκοιλιακών πιέσεων (<10–12 cm H2O), που πιθανότατα οδηγεί σε καλύτερα μετεγχειρητικά

αποτελέσματα. Βελτιστοποιείται εάν χρησιμοποιείται καθ' όλη τη διάρκεια της επέμβασης[1-3]. Για να επιτευχθεί αυτό, η καλή επικοινωνία μεταξύ αναισθησιολόγων και χειρουργών είναι ζωτικής σημασίας[4-5].

65. Η χρήση βαθέος νευρομυϊκού αποκλεισμού (PTC 1-2) συνιστάται για τη βελτίωση της απεικόνισης του χειρουργικού πεδίου, τόσο στην ανοιχτή όσο και στη λαπαροσκοπική χειρουργική, και για τη χρήση των χαμηλότερων δυνατών ενδοκοιλιακών πιέσεων στη λαπαροσκόπηση, ευνοώντας την μετεγχειρητική ανάρρωση.

Υψηλό επίπεδο τεκμηρίωσης. Ισχυρή σύσταση.

ΒΙΒΛΙΟΓΡΑΦΙΚΕΣ ΑΝΑΦΟΡΕΣ

1. Park SK, Son YG, Yoo S, Lim T, Kim WH, Kim JT. Deep vs. moderate neuromuscular blockade during laparoscopic surgery: A systematic review and meta-analysis. Eur J Anaesthesiol. 2018;35(11):867-875.

2. Bruintjes MH, van Helden EV, Braat AE, Dahan A, Scheffer GJ, van Laarhoven CJ et al. Deep neuromuscular block to optimize surgical space conditions during laparoscopic surgery: a systematic review and meta-analysis. Br J Anaesth. 2017:118(6):834-842.

3. Fuchs-Buder T, De Roberts E, Braunaud L. Neuromuscular block in laparoscopic surgery. Minerva Anestesiol 2018;84(4):509-14.

4. Errando-Oyonarte CL, Moreno-Sanz C, Vila-Caral P, Ruíz de Adana-Belbel JC, Vazquez- Alonso E, Ramírez-Rodríguez JM et al. Recomendaciones sobre el uso de bloqueo neuromuscular profundo por parte de anestesiólogos ycirujanos. Consenso AQUILES (Anestesia QUIrúrgica para Lograr Eficiencia y Seguridad) Rev Esp Anestesiol Reanim 2017;64:95- 104.

5. Madsen MV, Staehr-Rye AK, Gätke MR, Claudius C. Neuromuscular blockade for optimising surgical conditions during abdominal and gynaecological surgery: a systematic review. Acta Anaesthesiol Scand. 2015 ;59(1):1-16.

Αναστροφή του νευρομυϊκού αποκλεισμού

Η αναστροφή του νευρομυϊκού αποκλεισμού μπορεί να γίνει με σουγκαμαντέξ (sugammadex) σε οποιαδήποτε φάση βάθους αναισθησίας ή με νεοστιγμίνη για μέτριο νευρομυϊκό αποκλεισμό τουλάχιστον 3 αποκρίσεων TOF, αλλά η αποδιασωλήνωση του ασθενούς θα πρέπει να πραγματοποιείται μόνο αφού ο ασθενής έχει TOFr ≥ 0,9. Ο υπολειπόμενος αποκλεισμός είναι συχνός παρά τη χορήγηση των φαρμάκων αναστροφής του νευρομυϊκού αποκλεισμού (ιδιαίτερα με νεοστιγμίνη) και, χωρίς ποσοτική παρακολούθηση, πολύ μεγαλύτερος στην αυθόρμητη αναστροφή του νευρομυϊκού αποκλεισμού, και σχετίζεται με πνευμονικές επιπλοκές κατά τη μετεγχειρητική περίοδο[1-5].

66. Συνιστάται να ελέγχεται η αναστροφή του νευρομυϊκού αποκλεισμού έως ότου ληφθεί λόγος TOFr μεγαλύτερος ή ίσος με 0,9 στον προσαγωγό μυ του αντίχειρα κατά τη διάρκεια της αναισθησίας και πριν από την αποδιασωλήνωση για να αποφευχθεί ο υπολειπόμενος νευρομυϊκός αποκλεισμός και να μειωθούν οι αναπνευστικές επιπλοκές.

Υψηλό επίπεδο τεκμηρίωσης. Ισχυρή σύσταση.

ΒΙΒΛΙΟΓΡΑΦΙΚΕΣ ΑΝΑΦΟΡΕΣ

1. Kirmeier E, Eriksson LI, Lewald H, Jonsson Fagerlund M, Hoeft A, Hollmann M et al. Post-anaesthesia pulmonary complications after use of muscle relaxants (POPULAR): a multicentre, prospective observational study. Lancet Respir Med. 2019;7(2):129-140.

2. Tajaate N, Schreiber JU, Fuchs-Buder T, Jelting Y, Kranke P. Neostigmine-based reversal of intermediate acting neuromuscular blocking agents to prevent postoperative residual paralysis: A systematic review. Eur J Anaesthesiol. 2018;35(3):184-192.

3. Murphy GS. Neuromuscular Monitoring in the Perioperative Period. Anesth Analg. 2018; 126(2):464-468.

4. Hunter JM. Reversal of residual neuromuscular block: complications associated with perioperative management of muscle relaxation. Br J Anaesth. 2017 D 1;119(suppl_1):i53-i62.

5. Brull SJ, Kopman AF. Current Status of Neuromuscular Reversal and Monitoring: Challenges and Opportunities. Anesthesiology. 2017;126(1):173-190.

Αναστροφή νευρομυϊκού αποκλεισμού από ροκουρόνιο

Ο συνδυασμός ροκουρόνιου – σουγκαμαντέξ (rocuronium – sugammadex) για το νευρομυϊκό αποκλεισμό και την αναστροφή του έχει αποδειχθεί σε διαφορετικές μελέτες. Είναι πολύ πιο γρήγορος, φτάνοντας σε αναλογία TOFr > 0,9 μετά από έντονους, βαθείς και μέτριους αποκλεισμούς, διασφαλίζοντας την ανάρρωση των ασθενών και οδηγεί σε λιγότερες αναπνευστικές επιπλοκές από αυτές που παρατηρούνται όταν η αναστροφή γίνεται με τη νεοστιγμίνη[1-8].

67. Συνιστάται η αναστροφή του νευρομυϊκού αποκλεισμού με σουγκαμαντέξ αντί για νεοστιγμίνη όταν έχει χρησιμοποιηθεί βρωμιούχο ροκουρόνιο καθώς το πρώτο είναι ταχύτερο και ασφαλέστερο.

Υψηλό επίπεδο τεκμηρίωσης. Ισχυρή σύσταση.

ΒΙΒΛΙΟΓΡΑΦΙΚΕΣ ΑΝΑΦΟΡΕΣ

1. Hristovska AM, Duch P, Allingstrup M, Afshari A. The comparative efficacy and safety of sugammadex and neostigmine in reversing neuromuscular blockade in adults. A Cochrane systematic review with meta-analysis and trial sequential analysis. Anaesthesia. 2018;73 (5):631-641.

2. Hafeez KR, Tuteja A, Singh M, Wong DT, Nagappa M, Chung F et al. Postoperative complications with neuromuscular blocking drugs and/or reversal agents in obstructive sleep apnea patients: a systematic review. BMC Anesthesiol. 2018;18(1):91.

3. Carron M, Zarantonello F, Lazzarotto N, Tellaroli P, Ori C. Role of sugammadex in accelerating postoperative discharge: A meta-analysis. J Clin Anesth. 2017;39:38-44.

4. Carron M, Zarantonello F, Tellaroli P, Ori C. Efficacy and safety of sugammadex compared to neostigmine for reversal of neuromuscular

blockade: a meta-analysis of randomized controlled trial. J Clin Anesth. 2016;35:1-12.
5. Abad-Gurumeta A, Ripollés-Melchor J, Casans-Francés R, Espinosa A, Martínez-Hurtado E, Fernández-Pérez C, et al. Evidence Anaesthesia Review Group. A systematic review of sugammadex vs neostigmine for reversal of neuromuscular blockade. Anaesthesia. 2015;70(12):1441-52.
6. Manfred Blobner, Jennifer M Hunter, Claude Meistelman, Andreas Hoeft, Markus W Hollmann, et al. Use of a Train-Of-Four Ratio of 0.95 Versus 0.9 for Tracheal Extubation: An Exploratory Analysis of POPULAR Data. Br J Anaesth.2020;124(1):63-72.
7. Kheterpal S, Vaughn MT, Dubovoy TZ, Shah NJ, Bash LD, Colquhoun DA, et al. Sugammadex versus Neostigmine for Reversal of Neuromuscular Blockade and Postoperative Pulmonary Complications (STRONGER): A Multicenter Matched Cohort Analysis. Anesthesiology. 2020;132(6):1371-1381.
8. Raval AD, Uyei J, Karabis A, Bash LD, Brull SJ. Incidence of residual neuromuscular blockade and use of neuromuscular blocking agents with or without antagonists: A systematic review and meta-analysis of randomized controlled trials. J Clin Anesth. 2020; 64:109818 (On line first: 15th April 2020).

Νορμοθερμία

Η ακούσια περιεγχειρητική υποθερμία μπορεί να επηρεάσει αρνητικά την έκβαση της χειρουργικής επέμβασης και τη μετεγχειρητική κλινική πορεία του ασθενούς, καθώς σχετίζεται με αύξηση της μετεγχειρητικής νοσηρότητας[1-3], με αυξημένη επίπτωση φτωχής επούλωσης, λοίμωξης του χειρουργικού τραύματος, καρδιαγγειακών επιπλοκών, τρόμου και αυξημένης απώλειας αίματος[4,5]. Σχετίζεται επίσης με καθυστέρηση εξόδου από τις μονάδες ανάνηψης και από το νοσοκομείο και κατά συνέπεια με αύξηση του κόστους της διαδικασίας[5].

68. Συνιστάται η πρόληψη και η αποφυγή της ακούσιας περιεγχειρητικής υποθερμίας.

Υψηλό επίπεδο τεκμηρίωσης. Ισχυρή σύσταση.

ΒΙΒΛΙΟΓΡΑΦΙΚΕΣ ΑΝΑΦΟΡΕΣ

1. Torossian A, Bräuer A, Höcker J, Bein B, Wulf H, Horn EP. Preventing inadvertent perioperative hypothermia. Clinical Practice Guideline. Dtsch Arztebl Int. 2015; 112(10): 166-72.

2. Sessler DI. Perioperative thermoregulation and heat balance. Lancet. 2016;387(10038): 2655-2664.

3. Bindu B, Bindra A, Rath G. Temperature management under general anesthesia: Compulsion or option. J Anaesthesiol Clin Pharmacol. 2017;33(3):306-316.

4. Ruetzler K, Kurz A. Consequences of perioperative hypothermia. Handb Clin Neurol. 2018;157:687-697.

5. Calvo Vecino JM, Casans Francés R, Ripollés Melchor J, Marín Zaldívar C, Gómez Ríos MA, Pérez Ferrer A, et al. Clinical practice guideline. Unintentional perioperative hypothermia. Rev Esp Anestesiol Reanim. 2018;65(10):564-588.

Παρακολούθηση της θερμοκρασίας

Η θερμοκρασία θα πρέπει να παρακολουθείται σε όλους τους ασθενείς που υποβάλλονται σε γενική αναισθησία που διαρκεί περισσότερο από 30 λεπτά ή των οποίων η επέμβαση διαρκεί περισσότερο από 1 ώρα, ανεξάρτητα από την τεχνική αναισθησίας που χρησιμοποιείται[1,2]. Ο έλεγχος της θερμοκρασίας επιτρέπει την υιοθέτηση πρώιμων μέτρων για την αποφυγή της υποθερμίας, τα οποία μπορούν να εφαρμοστούν ακόμη και προληπτικά[3], καθώς και την έγκαιρη ανίχνευση και θεραπεία του πυρετού ή/και της υπερθερμίας[4].

69. Η θερμοκρασία των ασθενών θα πρέπει να ελέγχεται ώστε να διασφαλίζεται η νορμοθερμία στην περιεγχειρητική περίοδο.

Υψηλό επίπεδο τεκμηρίωσης. Ισχυρή σύσταση.

ΒΙΒΛΙΟΓΡΑΦΙΚΕΣ ΑΝΑΦΟΡΕΣ

1. Sessler DI. Perioperative thermoregulation and heat balance. Lancet. 2016;387(10038): 2655-2664.

2. Calvo Vecino JM, Casans Francés R, Ripollés Melchor J, Marín Zaldívar C, Gómez Ríos MA, Pérez Ferrer A, et al. Clinical practice guideline. Unintentional perioperative hypothermia. Rev Esp Anestesiol Reanim. 2018;65(10):564-588.
3. Ohki K, Kawano R, Yoshida M, Kanosue I, Yamamoto K. Normothermia is Best Achieved by Warming Above and Below with Prewarming Adjunct: A Comparison of Conductive Fabric Versus Forced-air and Water. Surg Technol Int. 2019;34:40-45.
4. Hooper VD, Chard R, Clifford T, Fetzer S, Fossum S, Godden B, et al. ASPAN's evidence-based clinical practice guideline for the promotion of perioperative normothermia: second edition. JPerianesth Nurs. 2010;25(6):346-65.

Ενεργητική προεγχειρητική προθέρμανση

Οι ενεργητικές στρατηγικές θέρμανσης πρέπει να ξεκινούν 20–30 λεπτά πριν από την επέμβαση, και είναι γνωστές και ως στρατηγικές προθέρμανσης[1,2,,4]. Αυτές οι στρατηγικές θα πρέπει να διατηρούνται κατά τη διάρκεια της διεγχειρητικής περιόδου για να διατηρηθεί η νορμοθερμία[1], ειδικά εάν η διάρκεια της αναισθησίας πρόκειται να είναι μεγαλύτερη από 60 λεπτά[3] και σε εκείνους τους ασθενείς με υψηλότερο κίνδυνο να υποστούν περιεγχειρητική υποθερμία, όπως εκείνοι άνω των 50 ετών ή με υψηλό χειρουργικό κίνδυνο. Φαίνεται ότι αυτές οι στρατηγικές θέρμανσης θα μπορούσαν να σχετίζονται με τη μείωση των λοιμώξεων των χειρουργικών τραυμάτων σε σύγκριση με τη χρήση μη ενεργών μεθόδων.

70. Οι ενεργητικές στρατηγικές προθέρμανσης πρέπει να ξεκινούν πριν από τη χειρουργική επέμβαση.

Υψηλό επίπεδο τεκμηρίωσης. Ισχυρή σύσταση.

ΒΙΒΛΙΟΓΡΑΦΙΚΕΣ ΑΝΑΦΟΡΕΣ

1. Torossian A, Bräuer A, Höcker J, Bein B, Wulf H, Horn EP. Preventing inadvertent perioperative hypothermia. Clinical Practice Guideline. Dtsch Arztebl Int. 2015; 112(10):166-72.

2. Warttig S, Alderson P, Campbell G, Smith AF. Interventions for treating inadvertent postoperative hypothermia. Cochrane Database Syst Rev. 2014 Nov 20;(11):CD009892.
3. Hooper VD, Chard R, Clifford T, Fetzer S, Fossum S, Godden B, et al. ASPAN's evidence-based clinical practice guideline for the promotion of perioperative normothermia: second edition. JPerianesth Nurs. 2010;25(6):346-65.
4. Akhtar Z, Hesler BD, Fiffick AN, Mascha EJ, Sessler DI, Kurz A, et al. A randomized trial of prewarming on patient satisfaction and thermal comfort in outpatient surgery. J Clin Anesth. 2016;33:376-85.

Θερμοκρασία περιβάλλοντος του χειρουργείου

Υπάρχουν παθητικά μέτρα για την πρόληψη της υποθερμίας, μεταξύ των οποίων είναι η θερμοκρασία περιβάλλοντος του χειρουργείου[1]. Η αύξηση της θερμοκρασίας του χειρουργείου προστατεύει τους ασθενείς από ακούσια υποθερμία, τόσο κατά την επέμβαση όσο και κατά την εισαγωγή τους στη μονάδα ανάνηψης[2]. Τα διαθέσιμα στοιχεία δείχνουν υψηλότερες κεντρικές θερμοκρασίες σε εκείνους τους ασθενείς που χειρουργούνται σε χειρουργεία με θερμοκρασία τουλάχιστον 21 ° C[3], αν και μπορεί να απαιτούνται χαμηλότερες θερμοκρασίες σε ορισμένους τύπους χειρουργικών επεμβάσεων.

71. Η θερμοκρασία περιβάλλοντος στο χειρουργείο πρέπει να είναι τουλάχιστον 21 °C για ενήλικες ασθενείς.

Υψηλό επίπεδο τεκμηρίωσης. Ισχυρή σύσταση.

ΒΙΒΛΙΟΓΡΑΦΙΚΕΣ ΑΝΑΦΟΡΕΣ

1. Torossian A, Bräuer A, Höcker J, Bein B, Wulf H, Horn EP. Preventing inadvertent perioperative hypothermia. Clinical Practice Guideline. Dtsch Arztebl Int. 2015 Mar 6; 112(10): 166-72.
2. Gómez-Romero FJ, Fernández-Prada M, Navarro-Gracia JF. Prevention of Surgical Site Infection: Analysis and Narrative Review of Clinical Practice Guidelines. Cir Esp. 2017;95 (9):490-502.

3. Hooper VD. Revisiting the ASPAN evidence-based clinical practice guideline for the promotion of perioperative normothermia. J Perianesth Nurs. 2010;25(6):343-5.

Θερμομόνωση του ασθενούς κατά την διεγχειρητική περίοδο

Παρά το γεγονός ότι αποτελεί παθητικό μέτρο ελέγχου της θερμοκρασίας, η κάλυψη της μεγαλύτερης δυνατής επιφάνειας του σώματος με σεντόνια και μία ή περισσότερες κουβέρτες ή παρόμοια υλικά αποτρέπει την απώλεια της θερμοκρασία του σώματος, συμβάλλοντας με απλό τρόπο στη θερμομόνωση του σώματος του ασθενούς κατά την περιεγχειρητική περίοδο[1-3]. Αυτό το μέτρο πρέπει να αποτελεί μέρος της κανονικής φροντίδας του ασθενούς.

72. Κατά την περιεγχειρητική περίοδο, η μεγαλύτερη δυνατή επιφάνεια του σώματος θα πρέπει να είναι θερμομονωμένη.

Υψηλό επίπεδο τεκμηρίωσης. Ισχυρή σύσταση.

ΒΙΒΛΙΟΓΡΑΦΙΚΕΣ ΑΝΑΦΟΡΕΣ

1. Torossian A, Bräuer A, Höcker J, Bein B, Wulf H, Horn EP. Preventing inadvertent perioperative hypothermia. Clinical Practice Guideline. Dtsch Arztebl Int. 2015; 112(10):166-72.
2. Warttig S, Alderson P, Campbell G, Smith AF. Interventions for treating inadvertent postoperative hypothermia. Cochrane Database Syst Rev. 2014 Nov 20;(11):CD009892.
3. Madrid E, Urrútia G, Roqué i Figuls M, Pardo-Hernandez H, Campos JM et al. Active body surface warming systems for preventing complications caused by inadvertent perioperative hypothermia in adults. Cochrane Database Syst Rev. 2016 Apr 21;4:CD009016.

Θέρμανση των υγρών κατά τη διεγχειρητική περίοδο

Η χορήγηση ενδοφλεβίων υγρών ή οι διεγχειρητικές πλύσεις με υγρά σε χαμηλές θερμοκρασίες θα πρέπει να αποφεύγονται καθώς αυξάνουν τον

κίνδυνο υποθερμίας στην περιεγχειρητική περίοδο. Τα υγρά που χορηγούνται ενδοφλεβίως στον ασθενή θα πρέπει πρώτα να θερμαίνονται, και η σύσταση αυτή ισχύει και για τα υγρά πλύσεων[1,2]. Θα πρέπει να εξετάζεται το ενδεχόμενο θέρμανσης των ενδοφλεβίων υγρών έως και μία ώρα πριν την επέμβαση. Η θέρμανση των ενδοφλεβίων υγρών έχει αποδειχθεί ευνοϊκό μέτρο από άποψη κόστους – αποτελεσματικότητας σε σύγκριση με τη μη θέρμανση, ακόμη και σε περιπτώσεις χαμηλότερου χειρουργικού κινδύνου, χαμηλότερου κινδύνου καρδιακών επιπλοκών και μικρής διάρκειας χειρουργικής επέμβασης[2,3].

73. Τα ενδοφλεβία υγρά, τα υγρά χειρουργικών πλύσεων και τα παράγωγα αίματος για μεταγγίσεις που χορηγούνται σε δόσεις > 500 ml/ώρα πρέπει πρώτα να έχουν θερμανθεί.

Υψηλό επίπεδο τεκμηρίωσης. Ισχυρή σύσταση.

ΒΙΒΛΙΟΓΡΑΦΙΚΕΣ ΑΝΑΦΟΡΕΣ

1. Torossian A, Bräuer A, Höcker J, Bein B, Wulf H, Horn EP. Preventing inadvertent perioperative hypothermia. Clinical Practice Guideline. Dtsch Arztebl Int. 2015; 112(10): 166-72.
2. Campbell G, Alderson P, Smith AF, Warttig S.Warming of intravenous and irrigation fluids for preventing inadvertent perioperative hypothermia. Cochrane Database Syst Rev. 2015 Apr 13;(4):CD009891.
3. Warttig S, Alderson P, Campbell G, SmithAF. Interventions for treating inadvertent postoperative hypothermia. Cochrane Database Syst Rev. 2014 Nov 20;(11):CD009892.

Ενεργητικά μέτρα διεγχειρητικής θέρμανσης

Πρέπει να λαμβάνονται ενεργητικά μέτρα διεγχειρητικής θέρμανσης[1]. Τα μέτρα αυτά πρέπει να εφαρμόζονται όσο το δυνατόν εκ των προτέρων γίνεται[2]. Ανάμεσα στα ενεργητικά συστήματα θέρμανσης του δέρματος, οι πιο αξιολογημένες στρατηγικές είναι η αγωγή θερμότητας και η συναγωγή με ζεστό αέρα, με τις στρατηγικές να είναι οικονομικά αποδοτικές ακόμη και σε ασθενείς με χαμηλότερο χειρουργικό κίνδυνο και μικρή διάρκεια χειρουργικής επέμβασης[3].

74. Τα διεγχειρητικά μέτρα ενεργητικής θέρμανσης με τη μεταφορά θερμότητας μέσω αγωγής ή συναγωγής ενδείκνυνται για τη διατήρηση της νορμοθερμίας.

Υψηλό επίπεδο τεκμηρίωσης. Ισχυρή σύσταση.

ΒΙΒΛΙΟΓΡΑΦΙΚΕΣ ΑΝΑΦΟΡΕΣ

1. Torossian A, Bräuer A, Höcker J, Bein B, Wulf H, Horn EP. Preventing inadvertent perioperative hypothermia. Clinical Practice Guideline. Dtsch Arztebl Int. 2015 Mar 6; 112(10): 166-72. doi: 10.3238/arztebl.2015.0166. PMID: 25837741.

2. Warttig S, Alderson P, Campbell G, Smith AF. Interventions for treating inadvertent postoperative hypothermia. Cochrane Database Syst Rev. 2014 Nov 20;(11): CD009892. doi: 10.1002/14651858.CD009892.pub2. Review. PubMed PMID: 25411963.

3. Calvo Vecino JM, Casans Francés R, Ripollés Melchor J, Marín Zaldívar C, Gómez Ríos MA, Pérez Ferrer A, et al. Clinical practice guideline. Unintentional perioperative hypothermia. Rev Esp Anestesiol Reanim. 2018;65(10):564-588.

Θερμοκρασία κατά την ανάνηψη από την αναισθησία

Πρέπει να διατηρείται υψηλότερη θερμοκρασία από τους 36 ° C καθ' όλη τη διάρκεια της χειρουργικής διαδικασίας, εφαρμόζοντας τα απαραίτητα μέτρα ώστε η ανάνηψη από την αναισθησία να πραγματοποιείται υπό νορμοθερμικές συνθήκες. Η μετεγχειρητική υποθερμία σχετίζεται με μεγαλύτερη παραμονή στη μονάδα μετεγχειρητικής ανάνηψης, επιπλέον του γεγονότος ότι τα ρίγη έχουν περιγραφεί ως αιτία έντονης ενόχλησης μετά την επέμβαση, συγκρίσιμη με τον μετεγχειρητικό πόνο [1-3].

75. Η ανάνηψη από την γενική αναισθησία πρέπει να γίνεται σε φυσιολογική θερμοκρασία σώματος.

Υψηλό επίπεδο τεκμηρίωσης. Ισχυρή σύσταση.

ΒΙΒΛΙΟΓΡΑΦΙΚΕΣ ΑΝΑΦΟΡΕΣ

1. Torossian A, Bräuer A, Höcker J, Bein B, Wulf H, Horn EP. Preventing inadvertent perioperative hypothermia. Clinical Practice Guideline. Dtsch Arztebl Int. 2015; 112(10): 166-72.

2.Bindu B, Bindra A, Rath G. Temperature management under general anesthesia: Compulsion or option. J Anaesthesiol Clin Pharmacol. 2017;33(3):306-316.

3. Warttig S, Alderson P, Campbell G, Smith AF. Interventions for treating inadvertent postoperative hypothermia. Cochrane Database Syst Rev. 2014 Nov 20;(11): CD009892.

Διεγχειρητική χορήγηση υγρών

Η διεγχειρητική χορήγηση υγρών παίζει ουσιαστικό ρόλο στη θεραπεία του χειρουργικού ασθενούς μέσω της σημαντικής επιρροής της στα μετεγχειρητικά αποτελέσματα. Οι βασικοί παράγοντες που πρέπει να ληφθούν υπόψη είναι η παρακολούθηση και οι στόχοι που πρέπει να επιτευχθούν, καθώς και η επιλογή του είδους του χορηγουμένου διαλύματος, ο όγκος και ο χρόνος χορήγησής του. Όλα έχουν αποτελέσει αντικείμενο έρευνας στην πρόσφατη βιβλιογραφία.

Στοχοκατευθυνόμενη χορήγηση υγρών

Ο στόχος της διεγχειρητικής χορήγησης υγρών είναι η διατήρηση της άρδευσης και αιμάτωσης των ιστών με επαρκή όγκο κυκλοφορίας διατηρώντας παράλληλα την ομοιόσταση των ηλεκτρολυτών[1]. Η υποογκαιμία μπορεί να προκαλέσει μεγαλύτερο κίνδυνο υποαιμάτωσης και βλάβης οργάνων ενώ η υπερογκαιμία μπορεί να προκαλέσει διάμεσο οίδημα, διαταραχή της επούλωσης και της πήξης, καρδιοαναπνευστικές επιπλοκές, καθώς και μετεγχειρητικό ειλεό[2]. Επομένως, η προσαρμοσμένη και εξατομικευμένη χορήγηση υγρών που βασίζεται σε καλά καθορισμένα πρωτόκολλα θα πρέπει να είναι ο στόχος για τη βελτιστοποίηση της αποτελεσματικότητάς και την αποφυγή των ιατρογενών επιπλοκών[3].

Όγκος παλμού (stroke volume - SV) και διακύμανση του όγκου παλμού (stroke volume variation - SVV)

Υπάρχει εκτεταμένη βιβλιογραφία που υποστηρίζει την εξατομίκευση της χορήγησης υγρών ή την αιμοδυναμικά στοχοκατευθυνόμενη χορήγηση υγρών με τη χρήση προηγμένης αιμοδυναμικής παρακολούθησης για τη βελτιστοποίηση του όγκου παλμού και τη μείωση της διακύμανσης του όγκου παλμού[2-4].

76. Η χρήση επαρκούς παρακολούθησης (SV ή SVV) συνιστάται για την καθοδήγηση της διεγχειρητικής χορήγησης υγρών σε ασθενείς σε κίνδυνο.

Υψηλό επίπεδο τεκμηρίωσης. Ισχυρή σύσταση.

ΒΙΒΛΙΟΓΡΑΦΙΚΕΣ ΑΝΑΦΟΡΕΣ

1. Makaryus R, Miller TE, Gan TJ. Current concepts of fluid management in enhanced recovery pathways. Br J Anaesth 2018; 120: 376-383.
2. Joosten A, Delaporte A, Ickx B, Touihri K, Stany I, Barvais L, et al. Crystalloid versus colloid for intraoperative goal-directed fluid therapy using a closed-loop system: A randomized, double-blinded, controllled trial in major abdminal surgery. Anesthesiology 2018; 128:55- 66.
3. Kapoor PM, Magoon R, Rawal RS, Mehta Y, Taneja S, Ravi R, et al. Goal-directed therapy improves the outcome of high-risk cardiac patients undergoing off-pump coronary artery bypass. Ann Card Anaesth 2017; 20: 83-9.
4. Bacchin MR, Ceria CM, Giannone S, Ghisi D, Stagni G, Greggi T, et al. Goal-directed fluid therapy based on stroke volume variation in patients undergoing major spine surgery in the prone position: A cohort study. Spine 2016; 41:E1131-7.

Διακύμανση του όγκου παλμού (SVV) και απόκριση σε υγρά

Ο μηχανικός αερισμός θετικής πίεσης προκαλεί κυκλική μείωση του προφορτίου στην αριστερή κοιλία λόγω μείωσης της φλεβικής επιστροφής και είναι πιο έντονη στην υποογκαιμία. Οι αλλαγές στο προφορτίο κατά τη διάρκεια του αναπνευστικού κύκλου οδηγούν σε διακυμάνσεις του όγκου παλμού[1].

Αυτές οι διακυμάνσεις εκτιμώνται με την ανάλυση περιγράμματος σφυγμού (pulse contour analysis). Ως απόκριση σε υγρά ορίζεται γενικά η αύξηση του όγκου παλμού ίση ή μεγαλύτερη από 10%[2].

77. Σε περιπτώσεις όπου υπάρχει πτώση του όγκου παλμού > 10% ή διακύμανση του όγκου παλμού > 10%, ενδείκνυται η αναζωογόνηση με υγρά (δεν υπάρχει προτίμηση μεταξύ κολλοειδών ή κρυσταλλοειδών).

Υψηλό επίπεδο τεκμηρίωσης. Ισχυρή σύσταση.

ΒΙΒΛΙΟΓΡΑΦΙΚΕΣ ΑΝΑΦΟΡΕΣ

1. Kendrick JB, Kaye AD, Tong Y, Belani K, Urman, Hoffman Ch, Liu H. Goal-directed fluid therapy in the perioperative setting. J Anesth Clin Pharm 2019; 35 (Suppl 1): S29-S34.

2. D'Angelo M, Kyle Hodgen R. Wet or Dry? A Review of Intravenous Fluid Administration in Anesthesia Practice. Annu Rev Nurs Res. 2017;35(1):221-239.

Ισοζύγιο υγρών

Η με περιορισμούς (restrictive) τακτική στη χορήγηση υγρών σχετίζεται με σημαντικά υψηλότερο κίνδυνο οξείας νεφρικής βλάβης από τη χωρίς περιορισμούς (liberal) χορήγηση υγρών[1,2]. Ο στόχος του μηδενικού ισοζυγίου μπορεί να είναι πολύ περιοριστικός, επομένως μια μετρίως ελεύθερη τακτική στην χορήγηση υγρών, μπορεί να συνιστάται για την επίτευξη θετικού ισοζυγίου 1 έως 2 λίτρα στο τέλος μιας μείζονας χειρουργικής επέμβασης[3,4].

78. Συνιστάται μια μέτρια συνεχής χορήγηση υγρών, που αποδίδει στο τέλος της επέμβασης θετικό ισοζύγιο 1 έως 2 λίτρα για την αποφυγή μετεγχειρητικής οξείας νεφρικής βλάβης.

Υψηλό επίπεδο τεκμηρίωσης. Ισχυρή σύσταση.

ΒΙΒΛΙΟΓΡΑΦΙΚΕΣ ΑΝΑΦΟΡΕΣ

1. Miller TE, and Myles PS. Perioperative Fluid Therapy for Major Surgery. Anesthesiology 2019; 130:825-32.

2. Myles PS, Bellomo R, Corcoran T, Forbes A, Peytor P, Story D et al. Restrictive versus Liberal Fluid Therapy for Major Abdominal Surgery (RELIEF study). N Eng J Med 2018; 378:2263-74.
3. Brandstrump B. Finding the right Balance. N Engl J Med 2018:378; 2335-6.
4. Miller TE, Pearse RM. Perioperative fluid management: moving toward more answers than questions - A commentary on the RELIEF study. Periop Med 2019;8:2.

Ασθενείς υψηλού κινδύνου

Τα τρέχοντα στοιχεία συνιστούν τη χρήση στοχοκατευθυνόμενης θεραπείας σε ασθενείς υψηλού αναισθησιολογικού κινδύνου και χειρουργικές επεμβάσεις υψηλού κινδύνου. Οι με πολλούς περιορισμούς ή οι χωρίς καθόλου περιορισμούς τακτικές στη χορήγηση υγρών έχουν σοβαρές συνέπειες σε ασθενείς με υψηλότερο χειρουργικό κίνδυνο, επομένως αυτές πρέπει να εξατομικεύονται σύμφωνα με τις αιμοδυναμικές παραμέτρους του όγκου παλμού και της διακύμανσης του όγκου παλμού[1-4].

79. Σε ασθενείς υψηλού κινδύνου, συνιστάται η διατήρηση εξατομικευμένης χορήγησης υγρών με μέτρια θετικό ισοζύγιο και συνεχή παρακολούθηση του όγκου παλμού και της διακύμανσης του όγκου παλμού.

Μέτριο επίπεδο τεκμηρίωσης. Ισχυρή σύσταση.

ΒΙΒΛΙΟΓΡΑΦΙΚΕΣ ΑΝΑΦΟΡΕΣ

1. Miller TE, and Myles PS. Perioperative Fluid Therapy for Major Surgery. Anesthesiology 2019; 130:825-32.
2. Gupta R, Gan TJ. Perioperative fluid management to enhance recovery. Anaesthesia. 2016;71 Suppl 1:40-5.
3. Boland MR, Reynolds I, McCawley N, Galvin E, El-Masry S, Deasy J, McNamara DA. Liberal perioperative fluid administration is an independent risk factor for morbidity and is associated with longer hospital stay after rectal cancer surgery. Ann R Coll Surg Engl. 2017;99(2):113- 116.
4. Bednarczyk JM, Fridfinnson JA, Kumar A, Blanchard L, Rabbani R, Bell D et al. Incorporating Dynamic Assessment of Fluid Responsiveness Into Goal-

Directed Therapy: A Systematic Review and Meta-Analysis. Crit Care Med. 2017;45(9):1538-1545.

Καμία ανταπόκριση στον όγκο

Το τεστ παθητικής ανύψωσης ποδιών που συνοδεύεται από αύξηση της αρτηριακής πίεσης ή του όγκου παλμού επιτρέπει μια απλή και άμεση πρόβλεψη της απόκρισης στα υγρά. Ένα αρνητικό τεστ δείχνει μικρή πιθανότητα απόκρισης. Σε αυτή την περίπτωση, η θεραπεία θα πρέπει να προσανατολίζεται στη χρήση αγγειοσυσπαστικών ή ινότροπων[1,2].

80. Η διεγχειρητική υπόταση χωρίς απόκριση στην παθητική ανύψωση των ποδιών θα πρέπει να αντιμετωπίζεται με αγγειοσυσπαστικά (έλεγχος για διακυμάνσεις της αρτηριακής πίεσης, του όγκου παλμού και της διακύμανσης του όγκου παλμού).

Μέτριο επίπεδο τεκμηρίωσης. Ισχυρή σύσταση.

ΒΙΒΛΙΟΓΡΑΦΙΚΕΣ ΑΝΑΦΟΡΕΣ

1. Futier E, Letirant JY, Guinot PG, Godet T, lorne E, Curvillon P, et al. Effect of individualized vs standard blood pressure management strategies on postoperative organ dysfunction among high-risk patients undergoing major surgery: A randomized clinical trial. JAMA 2017; 318: 1346-57.
2. Bentzer P, Griesdale DE, Boyd J, MacLean K, Sirounis D, and Ayas NT. Will This Hemodynamically Unstable Patient Respond to a Bolus of Intravenous Fluids? Jama 2016; 316:1298-309.

Μέση αρτηριακή πίεση

Υπάρχουν αυξανόμενες ενδείξεις ότι ακόμη και σύντομες περίοδοι διεγχειρητικής υπότασης, που ορίζονται ως μέση αρτηριακή πίεση χαμηλότερη από 65 mmHg, σχετίζονται με μυοκαρδιακή και νεφρική βλάβη. Επομένως, τα επεισόδια διεγχειρητικής υπότασης θα πρέπει να αποφεύγονται για να μειωθεί ο κίνδυνος ισχαιμίας του μυοκαρδίου ή οξείας νεφρικής ανεπάρκειας[1].

81. Θα πρέπει να επιτευχθεί ένα εύρος μέσης αρτηριακής πίεσης μεγαλύτερο ή ίσο με 65 mmHg.

Υψηλό επίπεδο τεκμηρίωσης. Ισχυρή σύσταση.

ΒΙΒΛΙΟΓΡΑΦΙΚΕΣ ΑΝΑΦΟΡΕΣ

1. Salmasi V, Maheshwari K, Yang D, Mascha EJ, Singh A, Sessler DI, et al. Relationship bet- ween Intraoperative Hypotension, Defined by Either Reduction from Baseline or Absolute Thresholds, and Acute Kidney and Myocardial Injury after Noncardiac Surgery: A Retrospective Cohort Analysis. Anesthesiology. 2017; 126(1):47-65.

Καρδιακός δείκτης

Εάν ένας ασθενής έχει βελτιστοποιημένο όγκο (δεν ανταποκρίνεται στα υγρά) και παραμένει υποτασικός με καρδιακό δείκτη μικρότερο από 2,5 l/λεπτό/m^2, θα πρέπει να ληφθούν υπόψη τα ινότροπα[1,2].

82. Θα πρέπει να διατηρείται ο καρδιακός δείκτης > 2,5 l/λεπτό/m^2, χρησιμοποιώντας ινότροπα σε περιπτώσεις μη απόκρισης στον όγκο.

Υψηλό επίπεδο τεκμηρίωσης. Ισχυρή σύσταση.

ΒΙΒΛΙΟΓΡΑΦΙΚΕΣ ΑΝΑΦΟΡΕΣ

1. Calvo-Vecino JM, Ripolles-Melchor J, Mythen MG, Casans-Francés R, Balik A, Artacho JP, et al. Effect of goal-directed haemodynamic therapy on postoperative complications in low-moderate risk surgical patients: A multicenter randomised controlled trial (FEDORA trial). Br J Anaesth 2018; 120:734-44.
2. Ripolles-Melchor J, Chappell D, Espinosa A, Mhyten MG, Abad-Gurumeta A, Bergese SD, et al. Fluid therapy recommendations for major abdominal surgery. Via RICA recommendations revisited. Part III: goal directed hemodynamic therapy. Rationale for maintaining vascular tone and contractility. Rev Esp Anestesiolo Reani 2107; 64: 348-59.

Διεγχειρητική αιμοδυναμική παρακολούθηση

Το οισοφαγικό Doppler είναι επί του παρόντος η μέθοδος που υποστηρίζεται περισσότερο από τα στοιχεία[1]. Ωστόσο, η εισαγωγή λιγότερο επεμβατικής παρακολούθησης με βάση την ανάλυση περιγράμματος σφυγμού κατέστησε δυνατή τη γενίκευση της στοχοκατευθυνόμενης θεραπείας και τη λήψη πιο εκτεταμένων στοιχείων τεκμηρίωσης[2,3].

83. Προτιμάται η παρακολούθηση με Doppler οισοφάγου ή με μεθόδους που βασίζονται σε πιστοποιημένη ανάλυση περιγράμματος σφυγμού.

Υψηλό επίπεδο τεκμηρίωσης. Ισχυρή σύσταση.

ΒΙΒΛΙΟΓΡΑΦΙΚΕΣ ΑΝΑΦΟΡΕΣ

1. Ripollés-Melchor J, Casans-Francés R, Espinosa A, Abad-Gurumeta A, Fedheiser A, Lopez-Timoneda F, et al. Goal directed hemodynamic therapy based in esophageal Doppler Flow parameters: A systematic review, meta-analysis and trial sequential analysis. Rev Esp Anestesiolo Reani 2016; 63:384-405.
2. Xu C, Peng J, Liu S, Huang Y, Guo X, Xiao H, et al. Goal-directed fluid therapy versus conventional fluid therapy in colorectal surgery: A meta analysis of randomized controlled trials. Inter J Surg 2018, 56:264-73.
3. LI MQ, Yang LQ, Zhou I, Liu H. Non-invasive cardiac output measurement: where we now? J Anesth Perioper Med 2018; 5:221-7.

Είδος Διαλύματος

Μεταξύ των κρυσταλλοειδών, τα στοιχεία δείχνουν ότι τα ισορροπημένα διαλύματα με σύσταση ηλεκτρολυτών και οξεοβασική ισορροπία κοντά σε αυτή του πλάσματος είναι προτιμότερα από διαλύματα πλούσια σε χλώριο καθώς τα τελευταία μπορεί να προκαλέσουν υπερχλωραιμία, μεταβολική οξέωση, νεφρική αγγειοσύσπαση και οξεία νεφρική βλάβη[1-4].

84. Το πρωτογενές ενδοφλέβιο υγρό διατήρησης πρέπει να είναι ένα ισορροπημένο ισοτονικό κρυσταλλοειδές διάλυμα.

Υψηλό επίπεδο τεκμηρίωσης. Ισχυρή σύσταση.

ΒΙΒΛΙΟΓΡΑΦΙΚΕΣ ΑΝΑΦΟΡΕΣ

1. Hammond DA, Lam SW, Rech MA, Smith MN, Westrick J, Trivedi AP, et al. Balanced Crystalloids Versus Saline in Critically Ill Adults: A Systematic Review and Meta-analysis. Ann Pharmacother. 2020;54:5-13.

2. Pfortmueller CA, Funk G-C, Reiterer C, Schrott A, Zotti O, Kabon B, et al. Normal saline versus balanced crystalloid for goal-directed perioperative fluid therapy in major abdominal surgery: a double-blind randomised controlled study. B J Anaesth 2018; 120:274-83.

3. Self WH, Semler MW, Wanderer JP, Wang L, Byrne DW, Collins SP, et al. Balanced crystalloids versus saline in non critically ill adults. N Engl J Med 2108; 378:819-28.

4. Semler MW, Self WH, Wanderer JP, Ehrenfeld JM, Wang I, Byrne DW, et al. Balanced crystalloids versus saline in critically ill adults. N England J Med 2018; 378:829-39.

Αναζωογόνηση με χορήγηση υγρών

Για τη χορήγηση υγρών κατά την αναζωογόνηση (resuscitation), συνιστάται η χρήση ισορροπημένων κρυσταλλοειδών: 2-3 λίτρα για την αρχική ανάνηψη σε υποογκαιμικό σοκ και αιμοδυναμική παρακολούθηση για την καθοδήγηση της πρόσθετης χορήγησης υγρών. Αρκετές κλινικές δοκιμές έχουν δείξει ότι η χρήση ισορροπημένων κρυσταλλοειδών αντί για αλατούχο διάλυμα αποτρέπει την ανάπτυξη υπότασης, την ανάγκη για αγγειοσυσπαστικά, τη νεφρική δυσλειτουργία και την ανάγκη για θεραπεία νεφρικής υποκατάστασης, καθώς επίσης μειώνει την θνησιμότητα[1-6].

85. Για τη χορήγηση υγρών κατά την αναζωογόνηση, συνιστάται η χρήση ισορροπημένων κρυσταλλοειδών. 2–3 λίτρα για την αρχική ανάνηψη σε υποογκαιμικό σοκ και αιμοδυναμική παρακολούθηση για καθοδήγηση της πρόσθετης χορήγησης υγρών.

Μέτριο επίπεδο τεκμηρίωσης. Ισχυρή σύσταση.

ΒΙΒΛΙΟΓΡΑΦΙΚΕΣ ΑΝΑΦΟΡΕΣ

1. Semler MW, Self WH, Wanderer JP, Wang L, Byrne DW, Collins SP, et al. Balanced Crystalloids versus Saline in Critically Ill Adults. N Engl J Med. 2018;378:829-839.
2. Semler MW, Kellum JA. Balanced Crystalloid Solutions. Am J Respir Crit Care Med. 2019; 199:952-960.
3. Casey JD, Brown RM, Semler MW. Resuscitation fluids. Curr Opin Crit Care. 2018;24:512- 518.
4. Bampoe S, Odor PM, Dushianthan A, Bennett-Guerrero E, Cro S, Gan TJ, et al. Perioperative administration of buffered versus non-buffered crystalloid intravenous fluid to improve outcomes following adult surgical procedures. Cochrane Database Syst Rev. 2017;Sep21; 9:CD004089.
5. Odor PM, Bampoe S, Dushianthan A, Bennett-Guerrero E, Cro S, Gan TJ, et al. Perioperative administration of buffered versus non-buffered crystalloid intravenous fluid to improve outcomes following adult surgical procedures: a Cochrane systematic review. Perioper Med (Lond). 2018;13;7:27.
6. Reddy S, Weinberg L, Young P. Crystalloid fluid therapy. Crit Care. 2016; 15;20:59.

Τρανεξαμικό οξύ

Συνιστάται η χορήγηση τρανεξαμικού οξέος (προληπτικά ή μετά από την χειρουργική επέμβαση), εκτός εάν αντενδείκνυται λόγω θρομβωτικού κινδύνου ή αλλεργίας. Η ανάγκη του ενδείκνυται σε καρδιοχειρουργικές, ορθοπεδικές (με γενική ή περιοχική αναισθησία), γναθοπροσωπικές, προστατικές και γυναικολογικές επεμβάσεις. Δεν συνιστάται σε περιπτώσεις σοβαρής νεφρικής ανεπάρκειας ή εάν υπάρχει ιστορικό επιληψίας[1-5].

Το τρανεξαμικό οξύ μπορεί να μειώσει την ανάγκη για μετάγγιση αίματος σε ενήλικες που υποβάλλονται σε χειρουργική επέμβαση. Αυτό επιτρέπει την αποφυγή σοβαρών κινδύνων που σχετίζονται με τη μετάγγιση αίματος, όπως λοίμωξη, υπερφόρτωση με υγρά και ακατάλληλες μεταγγίσεις αίματος. Μπορεί επίσης να μειώσει τη διάρκεια νοσηλείας στο νοσοκομείο και το κόστος για το Εθνικό Σύστημα Υγείας[6].

86. Συνιστάται να χορηγείται τρανεξαμικό οξύ σε όλους τους ενήλικες που υποβάλλονται σε χειρουργική επέμβαση και οι οποίοι αναμένεται να έχουν μέτρια έως σοβαρή απώλεια αίματος.

Υψηλό επίπεδο τεκμηρίωσης. Ισχυρή σύσταση.

ΒΙΒΛΙΟΓΡΑΦΙΚΕΣ ΑΝΑΦΟΡΕΣ

1. Blood transfusion Quality standard [QS138] Published date: December 2016. Quality statement 2: Tranexamic acid for adults https://www.nice.org.uk/guidance/qs138/chapter/ Quality-statement-2-Tranexamic-acid-for-adults (útlimo acceso 2020).

2. Xu Y, Sun S, Feng Q, Zhang G, Dong B, Wang X, et al. The efficiency and safety of oral tranexamic acid in total hip arthroplasty: A meta-analysis. Medicine (Baltimore). 2019;98: e17796. doi: 10.1097/MD.0000000000017796.

3. Zhang Y, Bai Y, Chen M, Zhou Y, Yu X, Zhou H, et al. The safety and efficiency of intravenous administration of tranexamic acid in coronary artery bypass grafting (CABG): a meta-analysis of 28 randomized controlled trials. BMC Anesthesiol. 2019;19:104. doi: 10.1186/ s12871-019-0761-3.

4. El-Menyar A, Sathian B, Asim M, Latifi R, Al-Thani H. Efficacy of prehospital administration of tranexamic acid in trauma patients: A meta-analysis of the randomized controlled trials. Am J Emerg Med. 2018;36:1079-1087. doi: 10.1016/j.ajem.2018.03.033.

5. Derzon JH, Clarke N, Alford A, Gross I, Shander A, Thurer R. Reducing red blood cell transfusion in orthopedic and cardiac surgeries with Antifibrinolytics: A laboratory medicine best practice systematic review and meta-analysis. Clin Biochem. 2019;71:1-13. doi: 10.1016/j. clinbiochem.2019.06.015.

6. Ripollés-Melchor J, Abad-Motos A, Díez-Remesal Y, Aseguinolaza-Pagola M, Padin-Barreiro L, Sánchez-Martín R, et al. Association Between Use of Enhanced Recovery After Surgery Protocol and Postoperative Complications in Total Hip and Knee Arthroplasty in the Postoperative Outcomes Within Enhanced Recovery After Surgery Protocol in Elective Total Hip and Knee Arthroplasty Study (POWER2). JAMA Surg. 2020;155:e196024.

Εισπνεόμενο κλάσμα οξυγόνου και κίνδυνος χειρουργικής λοίμωξης

Μέχρι τα τελευταία χρόνια, οι βασισμένες σε τεκμήρια κατευθυντήριες οδηγίες του ΠΟΥ συνιστούσαν υψηλό εισπνεόμενο κλάσμα οξυγόνου (Inspired Oxygen Fraction – FiO_2) 80% για τη μείωση της συχνότητας των χειρουργικών λοιμώξεων σε ενήλικες υπό γενική αναισθησία και ενδοτραχειακή διασωλήνωση. Ωστόσο, πρόσφατες κλινικές μελέτες προειδοποιούν για την απουσία οφελών τόσο στη μείωση των χειρουργικών λοιμώξεων λόγω χρήσης υψηλού FiO_2 (80%) όσο και του ίδιου του μέτρου[1-5].

87. Η συμπληρωματική χρήση εισπνεόμενου οξυγόνου δεν συνιστάται σε ασθενείς που υποβάλλονται σε γενική αναισθησία.

Μέτριο επίπεδο τεκμηρίωσης. Ασθενής σύσταση

ΒΙΒΛΙΟΓΡΑΦΙΚΕΣ ΑΝΑΦΟΡΕΣ

1. de Jonge S, Egger M, Latif A, Loke YK, Berenholtz S, Boermeester M, et al. Effectiveness of 80% vs 30-35% fraction of inspired oxygen in patients undergoing surgery: an updated systematic review and meta-analysis. Br J Anaesth. 2019;122(3):325-334.
2. Chu DK, Kim LH, Young PJ, Zamiri N, Almenawer SA, Jaeschke R, et al. Mortality and morbidity in acutely ill adults treated with liberal versus conservative oxygen therapy (IOTA): a systematic review and meta-analysis. Lancet. 201828;391(10131):1693-1705.
3. Mattishent K, Thavarajah M, Sinha A, Peel A, Egger M, Solomkin J, et al. Safety of 80% vs 30-35% fraction of inspired oxygen in patients undergoing surgery: a systematic review and meta-analysis. Br J Anaesth. 2019;122(3):311-324.
4. Mayank M, Mohsina S, Sureshkumar S, Kundra P, Kate V. Effect of Perioperative High Oxygen Concentration on Postoperative SSI in Elective Colorectal Surgery - A Randomized Controlled Trial. J Gastrointest Surg. 2019 ;23(1):145-152.
5. Cohen B, Schacham YN, Ruetzler K, Ahuja S, Yang D, Mascha EJ, et al. Effect of intraoperative hyperoxia on the incidence of surgical site infections: a meta-analysis. Br JAnaesth. 2018;120(6): 1176-1186.

Χειρουργική προσέγγιση και χειρουργικές τομές - ελάχιστα επεμβατική χειρουργική

Η εισαγωγή της ελάχιστα επεμβατικής χειρουργικής (minimally invasive surgery – MIS, λαπαροσκοπικής και ρομποτικής) έχει βελτιώσει σημαντικά την ευημερία των ασθενών: ο πόνος, το χειρουργικό στρες[1], η κατανάλωση οπιοειδών[2] και η απώλεια αίματος μειώνονται και η πρώιμη κινητοποίηση βελτιώνεται, τα οποία, συνδυαστικά, μειώνουν τη νοσηλεία στο νοσοκομείο[3,4]. Επομένως, εφόσον τα χειρουργικά και ογκολογικά αποτελέσματα δεν διαφέρουν μεταξύ των χειρουργικών τεχνικών, συνιστάται η ελάχιστα επεμβατική χειρουργική[5-7].

88. Συνιστάται ελάχιστα επεμβατική χειρουργική, με την προϋπόθεση ότι τα χειρουργικά και ογκολογικά αποτελέσματα δεν διαφέρουν μεταξύ των χειρουργικών τεχνικών.

Υψηλό επίπεδο τεκμηρίωσης. Ισχυρή σύσταση.

ΒΙΒΛΙΟΓΡΑΦΙΚΕΣ ΑΝΑΦΟΡΕΣ

1. Gustafsson UO, Scott MJ, Hubner M, Nygren J, Demartines N, Francis N, et al. Guidelines for Perioperative Care in Elective Colorectal Surgery: Enhanced Recovery After Surgery (ERAS). Society Recommendations: 2018. World J Surg. 2019; 43(3):659-695.

2. Ljungqvist O, Scott M, Fearon KC. Enhanced Recovery After Surgery: A Review. JAMA Surg. 2017 Mar 1; 152(3):292-298.

3. Obermair A, Janda M, Baker J, Kondalsamy-Chennakesavan S, Brand A, Hogg R, et al. Improved surgical safety after laparoscopic compared to open surgery for apparent early stage endometrial cancer: results from a randomised controlled trial. Eur J Cancer. 2012; 48(8):1147-53.

4. Vlug MS, Wind J, Hollmann MW, Ubbink DT, Cense HA, Engel AF et al. Laparoscopy in combination with fast track multimodal management is the best

perioperative strategy in patients undergoing colonic surgery: a randomized clinical trial (LAFA-study). Ann Surg. 2011; 254: 868-875.
5. Walker JL, Piedmonte MR, Spirtos NM, Eisenkop SM, Schlaerth JB, Mannel RS, et al. Recurrence and survival after random assignment to laparoscopy versus laparotomy for comprehensive surgical staging of uterine cancer: Gynecologic Oncology Group LAP2 Study. J Clin Oncol. 2012; 30(7):695-700.
6. Kalogera E, Glaser GE, Kumar A, Dowdy SC, Langstraat CL. Enhanced Recovery after Minimally Invasive Gynecologic Procedures with Bowel Surgery: A Systematic Review. J Minim Invasive Gynecol. 2019; 26(2):288-298.
7. Li K, Lin T, Fan X, Xu K, Bi L, Duan Y, et al: Systematic review and meta-analysis of comparative studies reporting early outcomes after robot-assisted radical cystectomy versus open radical cystectomy. Cancer Treatment Reviews 2012 Oct; 39: pp. 551-560.

Χειρουργικές Τομές

Όταν απαιτείται ανοιχτή προσπέλαση με λαπαροτομία, η εγκάρσια/λοξή τομή[1,2,3] φαίνεται να ελαττώνει το άλγος και τις πνευμονικές επιπλοκές, αλλά δεν υπάρχουν επαρκή στοιχεία, επομένως η επιλογή της χειρουργικής προσπέλασης εξαρτάται από τον χειρουργό, την εμπειρία και τις προτιμήσεις του, καθώς και χαρακτηριστικά του ασθενούς.

89. Η εγκάρσια τομή συνιστάται στη ανοιχτή χειρουργική με λαπαροτομία.

Μέτριο επίπεδο τεκμηρίωσης. Ισχυρή σύσταση.

ΒΙΒΛΙΟΓΡΑΦΙΚΕΣ ΑΝΑΦΟΡΕΣ

1. Santoro A, Boselli C, Renzi C, Gubbiotti F, Grassi V, Di Rocco G, et al. Transverse skin crease versus vertical midline incision versus laparoscopy for right hemicolectomy: a systematic review-current status of right hemicolectomy. Biomed Res Int. 2014;2014: 643685.
2. Seiler CM, Deckert A, Diener MK, Knaebel HP, Weigand MA, Victor N, et al. Midline versus transverse incision in major abdominal surgery: a randomized,

double blind equivalence trial (POVATI: ISRCTN60734227). Ann Surg. 2009; 249(6):913-20.
3. Brown SR, Goodfellow PB. Transverse verses midline incisions for abdominal surgery. Cochrane Database Syst Rev. 2005; (4):CD005199.

Παροχετεύσεις

Οι παροχετεύσεις έχουν χρησιμοποιηθεί συστηματικά με την πεποίθηση ότι με την εκκένωση του αίματος και των ορώδων συλλογών, θα μπορούσαν να αποφευχθούν οι μετεγχειρητικές λοιμώξεις. Ωστόσο, πολλές μελέτες σχετικά με αυτό το θέμα[1-3] δεν έχουν αποδείξει αυτήν την πεποίθηση, επομένως δεν υπάρχουν στοιχεία που να υποστηρίζουν τη χρήση τους σε συστηματική βάση[4-6].

90. Συνιστάται η αποφυγή χρήσης παροχετεύσεων σε συστηματική βάση.

Υψηλό επίπεδο τεκμηρίωσης. Ισχυρή σύσταση.

ΒΙΒΛΙΟΓΡΑΦΙΚΕΣ ΑΝΑΦΟΡΕΣ

1. Nelson G, Bakkum-Gamez J, Kalogera E, Glaser G, Altman A, Meyer LA, et al. Guidelines for perioperative care in gynecologic/oncology: Enhanced Recovery After Surgery (ERAS) Society recommendations-2019 update. Int J Gynecol Cancer. 2019 May;29(4):651-668.
2. Gustafsson UO, Scott MJ, Hubner M, Nygren J, Demartines N, Francis N, et al. Guidelines for Perioperative Care in Elective Colorectal Surgery: Enhanced Recovery After Surgery (ERAS®) Society Recommendations: 2018. World J Surg. 2019 Mar;43(3):659-695.
3. Denost Q, Rouanet P, Faucheron JL, Panis Y, Meunier B, Cotte E, et al. French Research Group of Rectal Cancer Surgery (GRECCAR). To Drain or Not to Drain Infraperitoneal Anastomosis After Rectal Excision for Cancer: The GRECCAR 5 Randomized Trial. Ann Surg. 2017 Mar;265(3):474-480
4. Carmichael JC, Keller DS, Baldini G, Bordeianou L, Weiss E, Lee L et al. Clinical Practice Guidelines for Enhanced Recovery After Colon and Rectal Surgery From the American Society of Colon and Rectal Surgeons and Society of American Gastrointestinal and Endoscopic Surgeons. Dis Colon Rectum. 2017 Aug;60(8):761-784.

5. Zhang HY, Zhao CL, Xie J, Ye YW, Sun JF, Ding ZH, et al. To drain or not to drain in colorectal anastomosis: a meta-analysis. Int J Colorectal Dis. 2016 May;31(5):951-960.
6. Musser JE, Assel M, Guglielmetti GB, Pathak P, Silberstein JL, Sjoberg DD, et al. Impact of routine use of surgical drains on incidence of complications with robot-assisted radical prostatectomy. J Endourol. 2014 Nov; 28(11):1333-7.

Ρινογαστρικός σωλήνας

Οι διάφορες μετα-αναλύσεις[1-3] έχουν καταλήξει στο συμπέρασμα ότι η τοποθέτηση ρινογαστρικού σωλήνα αυξάνει τον κίνδυνο μετεγχειρητικής πνευμονίας μετά από εκλεκτική χειρουργική επέμβαση στην κοιλία, δεν μειώνει τον κίνδυνο διάσπασης της γραμμής συρραφής ή αναστομωτικής διαφυγής[4-6], καθυστερεί την έναρξη της σίτισης και προκαλεί δυσφορία στην ασθενείς. Ως εκ τούτου, δεν υπάρχουν δεδομένα για την συστηματική του χρήση στη χειρουργική της κοιλίας.

91. Δεν συνιστάται η συστηματική χρήση του ρινογαστρικού σωλήνα.

Υψηλό επίπεδο τεκμηρίωσης. Ισχυρή σύσταση.

ΒΙΒΛΙΟΓΡΑΦΙΚΕΣ ΑΝΑΦΟΡΕΣ

1. Nelson G, Bakkum-Gamez J, Kalogera E, Glaser G, Altman A, Meyer LA, et al. Guidelines for perioperative care in gynecologic/oncology: Enhanced Recovery After Surgery (ERAS) Society recommendations-2019 update. Int J Gynecol Cancer. 2019 May;29(4):651-668.
2. Gustafsson UO, Scott MJ, Hubner M, Nygren J, Demartines N, Francis N, et al. Guidelines for Perioperative Care in Elective Colorectal Surgery: Enhanced Recovery After Surgery (ERAS®) Society Recommendations: 2018. World J Surg. 2019 Mar;43(3):659-695.
3. Carmichael JC, Keller DS, Baldini G, Bordeianou L, Weiss E, Lee L et al. Clinical Practice Guidelines for Enhanced Recovery After Colon and Rectal Surgery From the American Society of Colon and Rectal Surgeons and Society of American Gastrointestinal and Endoscopic Surgeons. Dis Colon Rectum. 2017 Aug;60(8):761-784.

4. Thorell A, MacCormick AD, Awad S, Reynolds N, Roulin D, Demartines N, et al. Guidelines for Perioperative Carein Bariatric Surgery: Enhanced Recovery After Surgery(ERAS) Society Recommendations. World J Surg. 2016 Sep;40(9):2065-83.
5. Weijs TJ, Kumagai K, Berkelmans GH, Nieuwenhuijzen GA, Nilsson M, Luyer MD. Nasogastric decompression following esophagectomy: a systematic literature review and meta- analysis. Dis Esophagus. 2017 Feb 1;30(3):1-8.
6. Rao W, Zhang X, Zhang J, Yan R, Hu Z, Wang Q. The role of nasogastric tube in decompression after elective colon and rectum surgery: a meta-analysis. Int J Colorectal Dis. 2011 Apr;26(4):423-9.

ΑΝΑΛΓΗΣΙΑ

Περιεγχειρητική αναλγησία

Από την έναρξή τους, στα πρωτόκολλα βελτιστοποίησης της μετεγχειρητικής ανάρρωσης, ο έλεγχος του πόνου ήταν βασικό στοιχείο.

Η αναζήτηση για μια αναλγητική μέθοδο που θα προσφέρει υψηλό βαθμό άνεσης στον ασθενή χωρίς να παρεμβαίνει σε άλλα βασικά σημεία των πρωτόκολλων βελτιστοποίησης της μετεγχειρητικής ανάρρωσης, όπως η πρώιμη κινητοποίηση, ο παραλυτικός ειλεός ή η μετεγχειρητική ναυτία και ο έμετος, ή που θα μπορούσε να αυξήσει το ποσοστό των επιπλοκών ή τη μέση νοσηλεία, σημαίνει ότι πολλές περιεγχειρητικές αναλγητικές στρατηγικές έχουν αξιολογηθεί ως μέρος των πρωτόκολλων βελτιστοποίησης της μετεγχειρητικής ανάρρωσης.

Κλασικά, οι περισσότερες μελέτες που πραγματοποιήθηκαν για την περιεγχειρητική αναλγησία έχουν προσφέρει συγκρίσεις μεταξύ της χρήσης ενδοφλέβιας χορήγησης οπιοειδών και τοποθέτησης επισκληρίδιου καθετήρα στο θωρακικό επίπεδο για τη χορήγηση τοπικών αναισθητικών, με ή χωρίς πρόσθετα οπιοειδή, προσφέροντας σαφή υπεροχή του δεύτερου έναντι του πρώτου, σε μείζονα χειρουργική επέμβαση στην κοιλία. Ωστόσο, αν και σήμερα η θωρακική επισκληρίδιος αναλγησία, εξακολουθεί να είναι η τεχνική εκλογής στην ανοιχτές μείζονες χειρουργικές επεμβάσεις κοιλίας, με την ανάπτυξη

ελάχιστα επεμβατικών χειρουργικών τεχνικών, τη διήθηση των σημείων εισόδου των τροκάρ με τοπικά αναισθητικά και την ανάπτυξη καθοδηγουμένων από υπερήχους αναλγητικών τεχνικών αποκλεισμού περιφερικών νεύρων (peripheral nerve block) δεν είναι η πρώτη αναλγητική επιλογή στη λαπαροσκοπική χειρουργική.

Τέλος, θα πρέπει να τονίσουμε τη σημασία των λοιπών επικουρικών τεχνικών στο πλαίσιο των αναλγητικών στρατηγικών βελτιστοποίησης της μετεγχειρητικής ανάρρωσης. Ορισμένα από αυτά είναι πιο συμβατικής χρήσης, όπως τα μη στεροειδή αντιφλεγμονώδη φάρμακα, αλλά άλλα είναι πιο πρόσφατης ή αμφιλεγόμενης χρήσης, όπως η ενδοφλέβια λιδοκαΐνη, η κεταμίνη, το θειικό μαγνήσιο ή η δεξμεντετομιδίνη, τα οποία πρέπει επίσης να λαμβάνονται υπόψη κατά την εφαρμογή ενός αναλγητικού πλάνου δράσης στα πλαίσια των πρωτόκολλων βελτιστοποίησης της μετεγχειρητικής ανάρρωσης. Με βάση τη χρήση αυτών, αναπτύχθηκε η αναισθησία χωρίς οπιοειδή (opioid-free anesthesia – OFA), με στόχο την κατάργηση της χρήσης οπιοειδών στο διεγχειρητικό στάδιο, αντικαθιστώντας τα από έναν συνδυασμό φαρμάκων ή/και τεχνικών ικανών να διατηρήσουν μια σταθερή αναισθησία, αναστέλλοντας τα επώδυνα ερεθίσματα με αποτελεσματικό τρόπο.

Οι διάφορες αναλγητικές μέθοδοι περιγράφονται λεπτομερώς παρακάτω.

Επισκληρίδιος αναλγησία σε ανοιχτή χειρουργική επέμβαση με λαπαροτομία

Υπάρχουν τόσο μετα-αναλύσεις όσο και υψηλής ποιότητας τυχαιοποιημένες κλινικές μελέτες που επιβεβαιώνουν την υπεροχή της επισκληρίδιου αναλγησίας έναντι της ενδοφλέβιας αναλγησίας με οπιοειδή, όσον αφορά την αναλγητική ποιότητα (ιδιαίτερα κατά τις πρώτες 24 ώρες μετεγχειρητικά και όταν ο ασθενής κινητοποιείται) και τη μείωση των μετεγχειρητικών επιπλοκών[1,2].

Η επισκληρίδιος αναλγησία έχει δείξει βελτίωση της ροής του αίματος στο γαστρεντερικό σύστημα, παρέχοντας ένα πιθανό όφελος σε εκείνους τους ασθενείς που υποβάλλονται σε μείζονα χειρουργική επέμβαση στην κοιλία.

Ωστόσο, αυτή η αυξημένη ροή δεν συνοδεύεται από αύξηση της κατανάλωσης οξυγόνου του ασθενούς[1].

Λόγω του αποκλεισμού του συμπαθητικού νευρικού συστήματος που προκαλείται από την τοποθέτηση επισκληρίδιου καθετήρα, η επισκληρίδιος αναλγησία συνοδεύεται από κάποιο βαθμό αιμοδυναμικής αστάθειας, με αποτέλεσμα αυξημένο κίνδυνο υπότασης, η οποία μπορεί να επιλυθεί με αγγειοσυσπαστικά[1].

Η τοποθέτηση επισκληρίδιου καθετήρα σε μείζονες χειρουργικές επεμβάσεις κοιλίας παρουσιάζει καλύτερα αναλγητικά αποτελέσματα από τα ενδοφλέβια οπιοειδή, τόσο με τον ασθενή σε ηρεμία όσο και σε κίνηση, ιδιαίτερα κατά τις πρώτες 24 ώρες μετά την επέμβαση. Η χρήση επισκληρίδιου αναλγησίας μειώνει τον χρόνο ανάκτησης της εντερικής διέλευσης και τη συχνότητα του παραλυτικού ειλεού, υποδηλώνοντας μείωση της νοσηλείας στο νοσοκομείο σε ανοιχτή χειρουργική επέμβαση[2].

92. Η επισκληρίδιος αναλγησία εντός ενός πρωτοκόλλου συνδυασμένης αναισθησίας θα πρέπει να πραγματοποιείται σε όλους τους ασθενείς που υποβάλλονται σε μείζονα ανοιχτή χειρουργική επέμβαση στην κοιλία.

Υψηλό επίπεδο τεκμηρίωσης. Ισχυρή σύσταση.

ΒΙΒΛΙΟΓΡΑΦΙΚΕΣ ΑΝΑΦΟΡΕΣ

1. Salicath JH, Yeoh ECY, Bennett MH. Epidural analgesia versus patient-controlled intravenous analgesia for pain following intra-abdominal surgery in adults. Cochrane Database of Systematic Reviews 2018;8:CD010434.
2. Guay J, Nishimori M, Kopp S. Epidural local anaesthetics versus opioid-based analgesic regimens for postoperative gastrointestinal paralysis, vomiting and pain after abdominal surgery. Cochrane Database of Systematic Reviews 2016;7:CD001893.

Θωρακική επισκληρίδιος αναλγησία

Αν και υπάρχουν λίγες τυχαιοποιημένες κλινικές μελέτες που αξιολογούν τις διαφορές μεταξύ της τοποθέτησης του επισκληρίδιου καθετήρα σε θωρακικό

ή οσφυϊκό επίπεδο, οι υπάρχουσες υποδεικνύουν σαφώς καλύτερη αναλγητική ποιότητα και λιγότερες επιπλοκές και αποκλεισμό κάτω άκρων σε εκείνους τους ασθενείς στους οποίους πραγματοποιείται θωρακική τοποθέτηση του επισκληρίδιου καθετήρα, σε σχέση με εκείνους στους οποίους πραγματοποιείται οσφυϊκή τοποθέτηση του επισκληρίδιου καθετήρα. Αυτά τα δεδομένα υποστηρίζονται επίσης από προοπτικές μελέτες παρατήρησης. Εκτός από όλα τα παραπάνω, οι περισσότερες μελέτες που υποστηρίζουν την τοποθέτηση επισκληρίδιου καθετήρα για αναλγησία σε μείζονες χειρουργικές επεμβάσεις στην κοιλία χρησιμοποιούν σημεία παρακέντησης στον θώρακα για την πραγματοποίησή της[1,2].

93. Η τοποθέτηση επισκληρίδιου καθετήρα για έγχυση τοπικών αναισθητικών για αναλγησία σε ανοιχτή μείζονα κοιλιακή χειρουργική επέμβαση πρέπει να πραγματοποιείται στο θωρακικό επίπεδο.

Υψηλό επίπεδο τεκμηρίωσης. Ισχυρή σύσταση.

ΒΙΒΛΙΟΓΡΑΦΙΚΕΣ ΑΝΑΦΟΡΕΣ

1. Scott AM, Starling JR, Ruscher AE, DeLessio ST, Harms BA, Michelassi F, et al. Thoracic versus lumbar epidural anesthesia’s effect on pain control and ileus resolution after restorative proctocolectomy. Surgery 1996;120(4):688-97292.

2. Pöpping DM, Zahn PK, Van Aken HK, Dasch B, Boche R, Pogatzki-Zahn EM. Effectiveness and safety of postoperative pain management: A survey of 18 925 consecutive patients between 1998 and 2006 (2nd revision): A database analysis of prospectively raised data. Br J Anaesth2008;101(6):832-40293.

Νευροαξονική χορήγηση οπιοειδών

Η χορήγηση μικρών ποσοτήτων οπιοειδών, μαζί με τα τοπικά αναισθητικά που χορηγούνται, στον επισκληρίδιο χώρο, βελτιώνει την αναλγητική ποιότητα του αποκλεισμού που πρόκειται να πραγματοποιηθεί, χωρίς να προκαλεί σημαντική αύξηση των επιπλοκών για τον ασθενή ή να επηρεάζει τα οφέλη της επισκληρίδιου αναλγησίας στην αποκατάσταση της εντερικής κινητικότητας. Ως πιθανή επιπλοκή, αξίζει να αναφερθεί η πιθανή

εμφάνιση κνησμού, αν και η επίπτωση δεν είναι υψηλή. Όσον αφορά το οπιοειδές που χρησιμοποιείται, η μορφίνη, η φαιντανύλη ή η σουφαιντανύλη φαίνεται να είναι εξίσου αποτελεσματικές όσον αφορά την ποιότητα της αναλγησίας. Το επίπεδο επίδρασης των οπιοειδών στον επισκληρίδιο χώρο είναι ανεξάρτητο από το σημείο παρακέντησης που επιλέχθηκε για την τοποθέτηση του επισκληρίδιου καθετήρα[1].

94. Μικρές δόσεις οπιοειδών θα πρέπει να προστίθενται στις δόσεις του τοπικού αναισθητικού που πρόκειται να χορηγηθούν επισκληρίδια σε μείζονες ανοιχτές χειρουργικές επεμβάσεις.

Μέτριο επίπεδο τεκμηρίωσης. Ισχυρή σύσταση.

ΒΙΒΛΙΟΓΡΑΦΙΚΕΣ ΑΝΑΦΟΡΕΣ

1. Guay J, Nishimori M, Kopp S. Epidural local anaesthetics versus opioid-based analgesic regimens for postoperative gastrointestinal paralysis, vomiting and pain after abdominal surgery. Cochrane Database of Systematic Reviews 2016;7: CD001893.

Αναλγησία χωρίς τοποθέτηση επισκληρίδιου καθετήρα

Εάν δεν χρησιμοποιείται επισκληρίδιος αναλγησία, η αναλγητική στρατηγική θα πρέπει να εξατομικεύεται, επιδιώκοντας τη μείωση της χρήσης οπιοειδών με την εφαρμογή διαφορετικών τύπων αποκλεισμών (block), είτε νωτιαίου, είτε τοπικού, είτε περιφερικού είτε διήθηση των σημείων εισόδου των τροκάρ με τοπικά αναισθητικά κλπ[1].

Σε όλες τις άλλες περιπτώσεις, η αναλγητική στρατηγική θα πρέπει να εξατομικεύεται, προσπαθώντας να αποφευχθεί η χρήση οπιοειδών και να ευνοηθεί η χρήση τοποπεριοχικών αποκλεισμών, νωτιαίας αναλγησίας ή διήθησης των σημείων εισόδου των τροκάρ με τοπικά αναισθητικά, ειδικά λαμβάνοντας υπόψη τον αποκλεισμό στο επίπεδο του εγκάρσιου κοιλιακού μυός (Transversus Abdominis Plane – TAP Block)[2].

95. Όταν η τοποθέτηση επισκληρίδιου καθετήρα δεν είναι δυνατή σε ανοιχτή μείζονα χειρουργική επέμβαση, η αναλγητική στρατηγική θα πρέπει να

εξατομικεύεται, μειώνοντας τη χρήση οπιοειδών και ευνοώντας τη χρήση τοποπεριοχικών αποκλεισμών, σπονδυλικής αναλγησίας ή διήθησης των σημείων εισόδου των τροκάρ με τοπικά αναισθητικά, ιδίως λαμβάνοντας υπόψη τον αποκλεισμό στο επίπεδο του εγκάρσιου κοιλιακού μυός.

Μέτριο επίπεδο τεκμηρίωσης. Ισχυρή σύσταση.

ΒΙΒΛΙΟΓΡΑΦΙΚΕΣ ΑΝΑΦΟΡΕΣ

1. Baeriswyl M, Zeiter F, Piubellini D, Kirkham KR, Albrecht E. The analgesic efficacy of transverse abdominis plane block versus epidural analgesia: a systematic review with meta- analysis. Medicine. 2018;97(26).
2. Shahait M, Lee DI. Application of TAP Block in laparoscopic urological surgery: current status and future directions. Curr Urol Rep. 2019;20:20.

Αποκλεισμοί δια μέσου των περιτονιών (interfascial blocks): αποκλεισμός στο επίπεδο του εγκάρσιου κοιλιακού μυός χωρίς δυνατότητα επισκληρίδιου αποκλεισμού

Ο αποκλεισμός στο επίπεδο του εγκάρσιου κοιλιακού μυός μπορεί να θεωρηθεί αποτελεσματική στρατηγική σε αυτές τις περιπτώσεις, με αναλγητική ποιότητα συγκρίσιμη με την επισκληρίδιο αναλγησία αλλά με χαμηλότερο προφίλ κινδύνου καθώς δεν προκαλεί αιμοδυναμικές αλλοιώσεις, διατηρεί την κινητική και αισθητηριακή λειτουργία των κάτω άκρων και μπορεί να χρησιμοποιείται με μεγαλύτερη ασφάλεια σε ασθενείς που λαμβάνουν αντιπηκτική αγωγή. Ωστόσο, δεν έχει καμία επίδραση στον σπλαχνικό πόνο, επομένως πρέπει απαραίτητα να αποτελεί μέρος ενός πρωτοκόλλου πολυπαραγοντικής αναλγησίας που συνδυάζει διαφορετικά φάρμακα ή αναλγητικές τεχνικές με διαφορετικούς μηχανισμούς δράσης. Ομοίως, λόγω της διάρκειας του αποκλεισμού TAP με μία μόνο παρακέντηση, σε εκείνες τις περιπτώσεις όπου αναμένεται η πιθανότητα έντονου πόνου μετά τις πρώτες 24 ώρες, θα πρέπει να λαμβάνεται υπόψη η τοποθέτηση καθετήρα και η συνεχής χορήγηση αναλγησίας στο επίπεδο αυτό[1]. Ωστόσο, ο αποκλεισμός στο επίπεδο του εγκάρσιου κοιλιακού μυός δεν έχει δείξει υπεροχή έναντι της επισκληρίδιου αναλγησίας σε καμία RCT.

96. Η εκτέλεση αμφοτερόπλευρου αποκλεισμού στο επίπεδο του εγκάρσιου κοιλιακού μυός με τοπικά αναισθητικά θα μπορούσε να ωφελήσει εκείνους τους ασθενείς που χρειάζονται ανοιχτή μείζονα χειρουργική επέμβαση στην κοιλιά και που δεν μπορούν να ωφεληθούν από την επισκληρίδιο αναλγησία.

Μέτριο επίπεδο τεκμηρίωσης. Ισχυρή σύσταση.

ΒΙΒΛΙΟΓΡΑΦΙΚΕΣ ΑΝΑΦΟΡΕΣ

1. Grupo de trabajo de la Guía de Práctica Clínica sobre Cuidados Perioperatorios en Cirugía Mayor Abdominal.Guía de Práctica Clínica sobre Cuidados Perioperatorios en Cirugía Mayor Abdominal.Ministerio de Sanidad, Servicios Sociales e Igualdad. Instituto Aragonés de Ciencias de la Salud (IACS); 2016Guías de Práctica Clínica en el SNS.

2. Baeriswy lM, Zeiter F, Piubellini D, Kirkham KR, Albrecht E. The analgesic efficacy of transverse abdominis plane block versus epidural analgesia: a systematic review with meta-analysis. Medicine. 2018;97(26).

3. Shahait M, Lee DI. Application of TAP Block in laparoscopic urological surgery: current status and future directions. Curr Urol Rep. 2019;20:20.

Αναισθησία χωρίς οπιοειδή (Opioid Free Anesthesia – OFA)

Η περιεγχειρητική χορήγηση οπιοειδών υπήρξε για μεγάλο χρονικό διάστημα ένας από τους τρεις πυλώνες της «ισορροπημένης αναισθησίας», που στην πράξη έχει ως στόχους την περιεγχειρητική ανακούφιση από τον πόνο και την προληπτική αναλγησία. Ο πόνος κατά την αναισθησία συνήθως ερμηνεύεται μέσω της αξιολόγησης υποκατάστατων σημείων, όπως η απόκριση του συμπαθητικού νευρικού συστήματος σε χειρουργικά ερεθίσματα. Ωστόσο, η συμβολή της συναισθηματικής εμπειρίας κατά τη διάρκεια μιας ασυνείδητης κατάστασης είναι αμφίβολη και οι αιμοδυναμικές αλλαγές είναι πιθανόν να οδηγήσουν σε σύγχυση πολλές φυσιολογικές διεργασίες. Επομένως, η υπόθεση ότι είναι απαραίτητη η θεραπεία αυτών των υποκατάστατων με οπιοειδή κατά τη διάρκεια της γενικής αναισθησίας μπορεί να είναι εσφαλμένη. Ομοίως, η χρήση οπιοειδών φαρμάκων για τον έλεγχο του πόνου δεν είναι ασφαλής, αλλά συνοδεύεται από διαφορετικές επιπλοκές και ανεπιθύμητες ενέργειες[1].

Με βάση τα παραπάνω, την τελευταία δεκαετία η αναισθησία χωρίς οπιοειδή έχει αρχίσει να αποτελεί εναλλακτική στην κλασική χρήση των ενδοφλεβίων οπιοειδών κατά την διεγχειρητική περίοδο. Η αναισθησία χωρίς οπιοειδή βασίζεται στην ιδέα ότι η πλήρης κατάργηση των οπιοειδών φαρμάκων κατά την διεγχειρητική περίοδο έχει θετικό αντίκτυπο στα αναμενόμενα αποτελέσματα στην μετεγχειρητική περίοδο, αντικαθιστώντας τα πλήρως με συνδυασμούς φαρμάκων ή/και τεχνικών που μαζί μπορούν να επιτύχουν σταθερή διατήρηση της αναισθησίας και να εμποδίσουν αποτελεσματικά τα επώδυνα ερεθίσματα. Τα φάρμακα που έχουν προταθεί για αυτόν τον σκοπό περιλαμβάνουν ανταγωνιστές του υποδοχέα του N-μεθυλο-D-ασπαρτικού οξέος (NMDA) (κεταμίνη, λιδοκαΐνη, θειικό μαγνήσιο), αναστολείς διαύλων ασβεστίου (τοπικά αναισθητικά), αντιφλεγμονώδη (στεροειδή, Μη Στεροειδή Αντιφλεγμονώδη – ΜΣΑΦ, AL) ή άλφα 2 αγωνιστές (κλονιδίνη, δεξμεδετομιδίνη)[2,3].

Προς το παρόν, δεν υπάρχουν ενδείξεις ότι η αναισθησία χωρίς οπιοειδή είναι σαφώς ανώτερη από την κλασική ισορροπημένη αναισθησία με βάση τα οπιοειδή, παρόλο που θα μπορούσε να μειώσει τις καταστάσεις υπεραλγησίας που προκαλούνται από τα οπιοειδή. Ομοίως, η αναισθησία χωρίς οπιοειδή έχει δείξει μείωση των ανεπιθύμητων ενεργειών που προκαλούνται από τα οπιοειδή, όπως η ναυτία και ο έμετος. Στη βαριατρική χειρουργική, υπάρχουν επίσης ενδείξεις για τη χρησιμότητά της στην αύξηση της άνεσης του ασθενούς και στη μείωση των ανεπιθύμητων ενεργειών όπως αποκορεσμούς ή άπνοιες στην άμεση μετεγχειρητική περίοδο[4].

97. Η αναισθησία χωρίς οπιοειδή σε πρωτόκολλα βελτιστοποίησης της μετεγχειρητικής ανάνηψης μπορεί να είναι μια εναλλακτική λύση στη χρήση ενδοφλεβίων οπιοειδών.

Μέτριο επίπεδο τεκμηρίωσης. Ασθενής σύσταση

ΒΙΒΛΙΟΓΡΑΦΙΚΕΣ ΑΝΑΦΟΡΕΣ

1. Mulier JP, Dillemans B. Anaesthetic Factors Affecting Outcome After Bariatric Surgery, a Retrospective Levelled Regression Analysis. Obes Surg. 2019;29:1841-50.

2. Frauenknecht J, Kirkham KR, Jacot-Guillarmod A, Albrecht E. Analgesic impact of intraoperative opioids vs. opioid-free anaesthesia: a systematic review and meta-analysis. Anaesthesia. 2019;74:651-62.
3. Mulier JP, Wouters R, Dillemans B, Deckock MI. A Randomized Controlled Double-Blind Trial Evaluating the Effect of Opioid-Free Versus Opioid General Anaesthesia on Postoperative Pain and Discomfort Measured by the QoR-40. J Clin Anesth Pain Med. 2018;2:2-6.
4. Mulier JP, Dillemans B. Deep Neuromuscular Blockade versus Remifentanil or Sevoflurane to Augment Measurable Laparoscopic Work space during Bariatric Surgery Analysed by a Randomised Controlled Trial. Journal of Clinical Anesthesia and Pain Medicine. 2018;7:2-4.

Διεγχειρητική ενδοφλέβια χορήγηση λιδοκαΐνης

Έρευνα σε άλλους τομείς του πόνου, όπως ο νευροπαθητικός πόνος ή το σύνδρομο σύνθετου περιφερικού πόνου, έχει δείξει ότι η ενδοφλέβια χορήγηση λιδοκαΐνης παράγει μακροχρόνια αναλγητικά αποτελέσματα, αναστέλλοντας την αυθόρμητη δημιουργία νευρικών ώσεων από τα τραυματισμένα περιφερικά νεύρα και τα γάγγλια των οπίσθιων ριζών (νωτιαία γάγγλια) κοντά στις τραυματισμένες νευρικές ίνες, καθώς και καταστέλλοντας τα πολυσυναπτικά αντανακλαστικά στο οπίσθιο κέρας του νωτιαίου μυελού[1]. Αν και ο πόνος στο περιεγχειρητικό περιβάλλον είναι κυρίως φλεγμονώδης, μπορεί επίσης να είναι νευροπαθητικός ή να βασίζεται σε υπεραλγησία. Όλες αυτές οι οντότητες θα μπορούσαν να βελτιωθούν με τη χορήγηση ενδοφλέβιας λιδοκαΐνης σε χαμηλές δόσεις, αν και η αναλγητική της δράση θα περιοριζόταν στις πρώτες 24 ώρες μετεγχειρητικά[2,3]. Επιπλέον, θα μπορούσε να βοηθήσει στην αποκατάσταση της εντερικής λειτουργίας και να αποτρέψει την ανάπτυξη παραλυτικού ειλεού.

98. Η χρήση ενδοφλέβιας λιδοκαΐνης διεγχειρητικά συνιστάται ως συμπληρωματική φαρμακευτική αγωγή για τη μείωση του μετεγχειρητικού πόνου και για τη βελτίωση της αποκατάστασης της εντερικής λειτουργίας στην άμεση μετεγχειρητική περίοδο, αποτελώντας εναλλακτική στην ενδοφλέβια χορήγηση οπιοειδών.

Μέτριο επίπεδο τεκμηρίωσης. Ασθενής σύσταση

ΒΙΒΛΙΟΓΡΑΦΙΚΕΣ ΑΝΑΦΟΡΕΣ

1. Cooke C, Kennedy ED, Foo I, Nimmo S, Speake D, Paterson HM, et al. Meta-analysis of the effect of perioperative intravenous lidocaine on return of gastrointestinal function after colorectal surgery. Tech Coloproctol. 2019;23:15-24.

2. Weibel S, Jelting Y, Pace NL, Helf A, Eberhart LH, Hahnenkamp K, et al. Continuous intravenous perioperative lidocaine infusion for postoperative pain and recovery in adults. Cochrane Database Syst Rev. 2018;6:CD009642.

3. MacFater WS, Rahiri J-L, Lauti M, Su'a B, Hill AG. Intravenous lignocaine in colorectal surgery: a systematic review. ANZ J Surg. 2017;87:879-85.

4. Weibel S, Jokinen J, Pace NL, Schnabel A, Hollmann MW, Hahnenkamp K, et al. Efficacy and safety of intravenous lidocaine for postoperative analgesia and recovery after surgery: a systematic review with trial sequential analysis. Br J Anaesth. 2016;116:770-83.

Διεγχειρητική χορήγηση κεταμίνης

Η κεταμίνη θα μπορούσε να μειώσει τη φλεγμονώδη αντίδραση που εμφανίζεται μετά την επέμβαση, μειώνοντας τα επίπεδα IL6[1]. Η περιεγχειρητική ενδοφλέβια χορήγηση κεταμίνης πιθανώς μειώνει την κατανάλωση μετεγχειρητικών αναλγητικών και την ένταση του πόνου χωρίς να προκαλεί σημαντική αύξηση των ανεπιθύμητων ενεργειών στο επίπεδο του κεντρικού νευρικού συστήματος και πιθανώς μειώνει τη μετεγχειρητική ναυτία και τον έμετο σε μικρό βαθμό, αμφιβόλου κλινικής σημασίας[2,3].

99. Η κεταμίνη θα πρέπει να χορηγείται ενδοφλέβια σε εκείνους τους ασθενείς που λαμβάνουν μείζονα οπιοειδή για αναλγησία σε μείζονες κοιλιακές χειρουργικές επεμβάσεις.

Μέτριο επίπεδο τεκμηρίωσης. Ασθενής σύσταση

ΒΙΒΛΙΟΓΡΑΦΙΚΕΣ ΑΝΑΦΟΡΕΣ

1. Brinck EC, Tiippana E, Heesen M, Bell RF, Straube S, Moore RA, et al. Perioperative intravenous ketamine for acute postoperative pain in adults. Cochrane Database Syst Rev. 2018;12:CD012033.
2. Nielsen RV, Fomsgaard JS, Siegel H, Martusevicius R, Nikolajsen L, Dahl JB, et al. Intraoperative ketamine reduces immediate postoperative opioid consumption after spinal fusion surgery in chronic pain patients with opioid dependency: a randomized, blinded trial. Pain. 2017;158:463-70.
3. Wang L, Johnston B, Kaushal A, Cheng D, Zhu F, Martin J. Ketamine added to morphine or hydromorphone patient-controlled analgesia for acute postoperative pain in adults: a systematic review and meta-analysis of randomized trials. Can J Anaesth. 2016;63:311-25.

Διεγχειρητική χορήγηση θειικού μαγνησίου

Το μαγνήσιο έχει μια ανασταλτική δράση στον νευρώνα μπλοκάροντας τους υποδοχείς NMDA του γλουταμικού οξέος, που είναι ο κύριος διεγερτικός νευροδιαβιβαστής στο κεντρικό νευρικό σύστημα. Έχει ηρεμιστικές και αντισπασμωδικές ιδιότητες, αναστέλλει την έκκριση κατεχολαμίνων και ενισχύει το νευρομυϊκό αποκλεισμό. Μπορεί να μειώσει τη χρήση οπιοειδών ρυθμίζοντας τα αλγαισθητικά ερέθισματα[1-5].

100. Η χορήγηση διεγχειρητικά θειικού μαγνησίου συνιστάται ως αναλγητικό συμπλήρωμα για τη βελτίωση του ελέγχου του πόνου σε ασθενείς που υποβάλλονται σε κοιλιακή χειρουργική επέμβαση.

Μέτριο επίπεδο τεκμηρίωσης. Ασθενής σύσταση

ΒΙΒΛΙΟΓΡΑΦΙΚΕΣ ΑΝΑΦΟΡΕΣ

1. Rodríguez-Rubio L, Nava E, Del Pozo JSG, Jordán J. Influence of the perioperative administration of magnesium sulfate on the total dose of anesthetics during general anesthesia. A systematic review and meta-analysis. J Clin Anesth. 2017;39:129-38.
2. Eizaga Rebollar R, García Palacios MV, Morales Guerrero J, Torres LM. Magnesium sulfate in pediatric anesthesia: the Super Adjuvant. Paediatr Anaesth. 2017;27:480-9.

3. Vicković S, Pjević M, Uvelin A, Pap D, Nikolić D, Lalić I. Magnesium Sulfate as an Adjuvant to Anesthesia in Patients with Arterial Hypertension. Acta Clin Croat. 2016;55:490-6.
4. Sousa AM, Rosado GMC, Neto J de S, Guimarães GMN, Ashmawi HA. Magnesium sulfate improves postoperative analgesia in laparoscopic gynecologic surgeries: a double-blind randomized controlled trial. J Clin Anesth. 2016;34:379-84.
5. Jarahzadeh MH, Harati ST, Babaeizadeh H, Yasaei E, Bashar FR. The effect of intravenous magnesium sulfate infusion on reduction of pain after abdominal hysterectomy under general anesthesia: a double-blind, randomized clinical trial. Electron Physician. 2016; 8:2602-6.

Διεγχειρητική χορήγηση δεξμεντετομιδίνης

Τα αναλγητικά αποτελέσματα της ενδοφλέβιας χορήγησης δεξμεδετομιδίνης και άλλων άλφα-2-αγωνιστών όπως η κλονιδίνη είναι γνωστά από τη δράση τους στο κεντρικό και περιφερικό νευρικό σύστημα[1-4]. Πρόσφατες συστηματικές ανασκοπήσεις σχετικά με τη διεγχειρητική χρήση της ενδοφλέβιας δεξμεντετομιδίνης σε σύγκριση με την ρεμιφαιντανύλη[1] παρέχουν μέτρια δεδομένα για τη μείωση των αναγκών σε οπιοειδή τόσο διεγχειρητικά όσο και κατά τις πρώτες 24 ώρες μετεγχειρητικά, παρουσιάζοντας μικρότερη ένταση πόνου και με μικρότερη ανάγκη για δόσεις διάσωσης (rescue) με οπιοειδή και με μικρότερη συχνότητα[1-3]. Ομοίως, υπάρχουν λιγότερες ανεπιθύμητες ενέργειες που προέρχονται από τη χαμηλότερη χρήση περιεγχειρητικών οπιοειδών, παρουσιάζοντας χαμηλότερη επίπτωση υπότασης, ρίγους, ναυτίας και μετεγχειρητικού εμέτου[1-4].

101. Συνιστάται η ενδοφλέβια διεγχειρητική χορήγηση δεξμεντετομιδίνης, καθώς συμβάλλει στη μείωση του κινδύνου ανεπιθύμητων ενεργειών που σχετίζονται με τα οπιοειδή και βελτιώνει τον έλεγχο του πόνου στην διεγχειρητική και μετεγχειρητική περίοδο.

Μέτριο επίπεδο τεκμηρίωσης. Ασθενής σύσταση

ΒΙΒΛΙΟΓΡΑΦΙΚΕΣ ΑΝΑΦΟΡΕΣ

1. Grape S, Kirkham KR, Frauenknecht J, Albrecht E. Intra-operative analgesia with remifentanil vs. dexmedetomidine: a systematic review and meta-analysis with trial sequential analysis. Anaesthesia. 2019;74:793-800.

2. Wang X, Liu N, Chen J, Xu Z, Wang F, Ding C. Effect of Intravenous Dexmedetomidine During General Anesthesia on Acute Postoperative Pain in Adults: A Systematic Review and Meta-Analysis of Randomized Controlled Trials. Clin J Pain. 2018;34:1180-91.

3. Jin S, Liang DD, Chen C, Zhang M, Wang J. Dexmedetomidine prevent postoperative nausea and vomiting on patients during general anesthesia: A PRISMA-compliant meta analysis of randomized controlled trials. Medicine (Baltimore). 2017;96:e5770.

4. Le Bot A, Michelet D, Hilly J, Maesani M, Dilly MP, Brasher C, et al. Efficacy of intraoperative dexmedetomidine compared with placebo for surgery in adults: a meta-analysis of published studies. Minerva Anestesiol. 2015;81:1105-17.

Περιεγχειρητική χορήγηση νευροτροποποιητών

Η από του στόματος χορήγηση νευροτροποποιητών (neuromodulators) όπως η πρεγκαμπαλίνη ή η γκαμπαπεντίνη θα μπορούσε να οδηγήσει σε σημαντική μείωση στη χρήση οπιοειδών τις πρώτες 24 ώρες χωρίς να προκαλέσει επιβλαβείς επιπτώσεις στους ασθενείς[1-3]. Επιπλέον, θα μπορούσε να έχει ευεργετική επίδραση στον χρόνιο πόνο των ασθενών στους 6 μήνες μετά την επέμβαση. Οι ασθενείς ηλικίας άνω των 65 ετών έχουν περισσότερες ανεπιθύμητες ενέργειες που προέρχονται από τη χρήση της πρεγκαμπαλίνης και επομένως αυτή θα μπορούσε να είναι καλύτερα υποκατάστατο της χρήσης της γκαμπαπεντίνης[4,5]. Μια πρόσφατα δημοσιευμένη μετα-ανάλυση με περισσότερες από 280 κλινικές δοκιμές και 24.000 ασθενείς δεν βρήκε καθόλου σχετικά κλινικά αναλγητικά αποτελέσματα, αν και υπήρχαν στατιστικά σημαντικές διαφορές στις πρώτες μετεγχειρητικές ώρες. Βρέθηκαν μικρότερα ποσοστά ναυτίας και εμέτου και μεγαλύτερος αριθμός οπτικών διαταραχών και ζάλης[6].

102. Στις ανοικτές μείζονες χειρουργικές κοιλιακές επεμβάσεις θα μπορούσε να περιλαμβάνεται αξιολόγηση της προεγχειρητικής από του στόματος χορήγησης γκαμπαπεντίνης ή πρεγκαμπαλίνης πριν από την επέμβαση για μετεγχειρητικό αναλγητικό έλεγχο.

Υψηλό επίπεδο τεκμηρίωσης. Ασθενής σύσταση.

ΒΙΒΛΙΟΓΡΑΦΙΚΕΣ ΑΝΑΦΟΡΕΣ

1. Rai AS, Khan JS, Dhaliwal J, Busse JW, Choi S, Devereaux PJ, et al. Preoperative pregabalin or gabapentin for acute and chronic postoperative pain among patients undergoing breast cancer surgery: A systematic review and meta-analysis of randomized controlled trials. J Plast Reconstr Aesthet Surg.2017;70:1317-28.
2. Liu B, Liu R, Wang L. A meta-analysis of the preoperative use of gabapentinoids for the treatment of acute postoperative pain following spinal surgery. Medicine (Baltimore). 2017;96:e8031.
3. Li S, Guo J, Li F, Yang Z, Wang S, Qin C. Pregabalin can decrease acute pain and morphine consumption in laparoscopic cholecystectomy patients: A meta-analysis of randomized controlled trials. Medicine (Baltimore). 2017;96:e6982.
4.Yao Z, Shen C, Zhong Y. Perioperative Pregabalin for Acute Pain After Gynecological Surgery: A Meta-analysis. Clin Ther.2015;37:1128-35.
5. Eipe N, Penning J, Yazdi F, Mallick R, Turner L, Ahmadzai N, et al. Perioperative use of pregabalin for acute pain - a systematic review and meta-analysis. Pain.2015;156:1284-300.
6. Verret M, Lauzier F, Zarychanski R, Perron C, Savard X, Pinard AM, et al. Perioperative Use of Gabapentinoids for the Management of Postoperative Acute Pain: A Systematic Review and Meta-analysis. Anesthesiology.2020:133: 265-279.

Πρόληψη παραλυτικού ειλεού

Ο παραλυτικός ειλεός είναι μια από τις επιπλοκές που προκαλεί τη μεγαλύτερη δυσφορία στον ασθενή, καθώς και την παράταση της νοσηλείας του στο νοσοκομείο[1].

103. Συνιστάται η πολυπαραγοντική διαχείριση του ασθενούς με τη χρήση εναλλακτικών των οπιοειδών φαρμάκων και τεχνικών (θωρακικός επισκληρίδιος καθετήρας, νευρομυϊκοί αποκλεισμοί, ελάχιστα επεμβατική χειρουργική επέμβαση, αποφυγή της συστηματικής χρήσης του ρινογαστρικού σωλήνα και αποφυγή της υπερβολικής ενδοφλέβιας χορήγησης υγρών) για την πρόληψη της εμφάνισης μετεγχειρητικού παραλυτικού ειλεού[1].

Υψηλό επίπεδο τεκμηρίωσης. Ισχυρή σύσταση.

ΒΙΒΛΙΟΓΡΑΦΙΚΕΣ ΑΝΑΦΟΡΕΣ

1. Gustafsson UO, Scott MJ, Hubner M, Nygren J, Demartines N, Francis N, et al. Guidelines for Perioperative Care in Elective Colorectal Surgery: Enhanced Recovery After Surgery (ERAS) Society Recommendations: 2018. World J Surg 2019; 43(3):659-695.

Προφύλαξη από ναυτία και έμετο

Η μετεγχειρητική ναυτία και ο έμετος (postoperative nausea and vomiting – PONV) είναι η πιο σημαντική αιτία καθυστερημένης έναρξης ανοχής στα υγρά από το στόμα και μπορεί να είναι πιο άβολη για τον ασθενή ακόμα και από τον πόνο. Επηρεάζει το 25–35% όλων των χειρουργικών ασθενών και αποτελεί μια από τις κύριες αιτίες δυσφορίας και καθυστέρησης στην λήψη του ιατρικού εξιτηρίου. Η προφύλαξη πρέπει να είναι ανάλογη με τον εκτιμώμενο κίνδυνο[1-3].

ΜΕΤΡΑ ΠΡΟΦΥΛΑΞΗΣ ΚΑΙ ΑΝΤΙΜΕΤΩΠΙΣΗΣ

Ταυτοποίηση του ασθενούς που διατρέχει κίνδυνο PONV

Ο κίνδυνος PONV θα πρέπει να αξιολογείται σε όλους τους ασθενείς χρησιμοποιώντας μια επικυρωμένη κλίμακα κινδύνου, όπως η απλοποιημένη κλίμακα Apfel, η οποία αξιολογεί τους παράγοντες κινδύνου για PONV: γυναικείο φύλο, ιστορικό PONV ή/και ναυτίας κίνησης, μη καπνιστής, μετεγχειρητική χορήγηση οπιοειδών[1-2]. Ασθενείς ηλικίας κάτω των 50 ετών και εκείνοι με ιστορικό ναυτίας και εμέτου που προκαλείται από χημειοθεραπεία

διατρέχουν αυξημένο κίνδυνο PONV. Όσον αφορά το είδος της επέμβασης, αυξημένος κίνδυνος PONV έχει παρατηρηθεί σε χολοκυστεκτομές, γυναικολογικές επεμβάσεις και λαπαροσκοπικές επεμβάσεις[3].

104. Ο κίνδυνος μετεγχειρητικής ναυτίας και εμέτου πρέπει να διαστρωματώνεται σε όλους τους ασθενείς χρησιμοποιώντας την κλίμακα Apfel και πρέπει να δίνεται προφύλαξη ανάλογα με τον αναμενόμενο κίνδυνο. Η προφύλαξη με περισσότερα συνδυασμένα φάρμακα μπορεί να πραγματοποιηθεί σε χειρουργικές επεμβάσεις στις οποίες η PONV ενέχει σημαντικό κίνδυνο επιπλοκών.

Υψηλό επίπεδο τεκμηρίωσης. Ισχυρή σύσταση.

ΒΙΒΛΙΟΓΡΑΦΙΚΕΣ ΑΝΑΦΟΡΕΣ

1. Apfel CC, Läärä E, Koivuranta M, Greim CA, Roewer N. A simplified risk score for predicting postoperative nausea and vomiting: conclusions from cross-validations between two centers. Anesthesiology 1999;91(3):693-700.
2. Apfel CC, Philip BK, Cakmakkaya OS, Shilling A, Shi Y-Y, Leslie JB, et al. Who is at risk for post discharge nausea and vomiting after ambulatory surgery? Anesthesiology 2012;117(3): 475-86.
3. Apfel CC, Heidrich FM, Jukar-RaoS, Jalota L, Hornuss C, Whelan RP, et al. Evidence based analysis of risk factors for postoperative nausea and vomiting. Br J Anaesth 2012;109(5):742-53.

ΜΕΙΩΣΗ ΤΟΥ ΒΑΣΙΚΟΥ ΚΙΝΔΥΝΟΥ PONV:

Περιοχική αναισθησία έναντι γενικής αναισθησίας

Η μείωση των βασικών παραγόντων κινδύνου για PONV μειώνει τη συχνότητά της[1]. Οι στρατηγικές για την ελαχιστοποίησή της σε ασθενείς σε κίνδυνο περιλαμβάνουν την επιλογή της περιοχικής αναισθησίας έναντι της γενικής αναισθησίας, τη χρήση της προποφόλης στην εισαγωγή και στη διατήρηση της αναισθησίας, την αποφυγή της χρήσης υποξειδίου του αζώτου (nitrous oxide - N_2O) και των πτητικών αναισθητικών, την ελαχιστοποίηση της

χρήσης οπιοειδών διεγχειρητικά και μετεγχειρητικά και την εξασφάλιση επαρκούς ενυδάτωσης[2].

105. Η περιοχική αναισθησία συνιστάται πριν από τη γενική αναισθησία για τη μείωση της συχνότητας εμφάνισης PONV.

Υψηλό επίπεδο τεκμηρίωσης. Ισχυρή σύσταση.

ΒΙΒΛΙΟΓΡΑΦΙΚΕΣ ΑΝΑΦΟΡΕΣ
1. Sinclair DR, Chung F, Mezei G. Can postoperative nausea and vomiting be predicted? Anesthesiology 1999;91:109-18.
2. Veiga-Gil L, Pueyo J, López-Olaondo L. Náuseas y vómitos postoperatorios: fisiopatología, factores de riesgo, profilaxis y tratamiento. Rev Esp Anestesiol Reanim 2017;64(4):223-32.

Ενδοφλέβια αναισθησία έναντι εισπνεόμενης αναισθησίας

Σε ασθενείς με υψηλότερο κίνδυνο ή ιστορικό PONV, έχει αποδειχθεί ότι η ολική ενδοφλέβια γενική αναισθησία αποκλειστικά με προποφόλη μειώνει τη συχνότητα εμφάνισης ναυτίας και εμέτου σε σύγκριση με τη διατήρηση με εισπνεόμενα αλογονωμένα αναισθητικά[1-3].

106. Η χρήση της προποφόλης συνιστάται για την εισαγωγή και τη διατήρηση της αναισθησίας σε ασθενείς με υψηλό κίνδυνο PONV.

Υψηλό επίπεδο τεκμηρίωσης. Ισχυρή σύσταση.

ΒΙΒΛΙΟΓΡΑΦΙΚΕΣ ΑΝΑΦΟΡΕΣ
1. Tramèr M, Moore A, McQuay H. Propofol anaesthesia and postoperative nausea and vomiting: quantitative systematic review of randomized controlled studies. BJA: Br J Anaesth 1997;78(3):247-55.
2. Apfel CC, Korttila K, Abdalla M, Kerger H, Turan A, Vedder I, et al. A factorial trial of six interventions for the prevention of postoperative nausea and vomiting. N Engl J Med 2004;350(24):2441-51.
3. Schraag S, Pradelli L, Alsaleh AJO, Bellone M, Ghetti G, Chung TL, et al. Propofol vs. inhalational agents to maintain general anaesthesia in ambulatory

and in-patient surgery: a systematic review and meta-analysis. BMC Anesthesiol. 2018;18(1):162.

Αποφύγετε τη χρήση υποξειδίου του αζώτου

Σε χειρουργικές επεμβάσεις που διαρκούν περισσότερο από μία ώρα και σε ασθενείς με κίνδυνο PONV, η συχνότητα εμφάνισης ναυτίας και εμέτου αυξάνεται εάν χρησιμοποιείται ισορροπημένη αναισθησία με εισπνεόμενα αλογονωμένα αναισθητικά σε συνδυασμό με οξείδιο του αζώτου[1-6].

107. Η χρήση του υποξειδίου του αζώτου θα πρέπει να αποφεύγεται σε ασθενείς με υψηλό κίνδυνο PONV ή σε μεγάλης διάρκειας χειρουργικές επεμβάσεις.

Υψηλό επίπεδο τεκμηρίωσης. Ισχυρή σύσταση.

ΒΙΒΛΙΟΓΡΑΦΙΚΕΣ ΑΝΑΦΟΡΕΣ

1. Tramèr M, Moore A, McQuay H. Omitting nitrous oxide in general anaesthesia: meta-analysis of intraoperative awareness and postoperative emesis in randomized controlled trials. Br J Anaesth 1996;76(2):186-93.
2. Fernández-Guisasola J, Gómez-Arnau JI, Cabrera Y, del Valle SG. Association between nitrous oxide and the incidence of postoperative nausea and vomiting in adults: a systematic review and meta-analysis. Anaesthesia 2010;65(4):379-87.
3. Peyton PJ, Wu CY. Nitrous oxide-related postoperative nausea and vomiting depends on duration of exposure. Anesthesiology 2014;120(5):1137-45.
4. Sun R, Jia WQ, Zhang P, Yang K, Tian JH, Ma B, et al. Nitrous oxide-based techniques versus nitrous oxide-free techniques for general anaesthesia. Cochrane Database Syst Rev 2015 Nov 6;(11):CD008984.
5. Buhre W, Disma N, Hendrickx J, DeHert S, Hollmann MW, Huhn R, et al. European Society of Anaesthesiology Task Force on Nitrous Oxide: a narrative review of its role in clinical practice. Br JAnaesth 2019;122(5):587-604.
6. Myles PS, Chan MTV, Kasza J, Paech MJ, Leslie K, Peyton PJ, et al. Severe Nausea and Vomiting in the Evaluation of Nitrous Oxide in the Gas Mixture for Anesthesia II Trial. Anesthesiology 2016;124(5):1032-1040.

Αποφύγετε τη χρήση αλογονωμένων αναισθητικών παραγόντων

Η χρήση αλογονωμένων εισπνεόμενων αναισθητικών παραγόντων αυξάνει τη συχνότητα εμφάνισης PONV σε ασθενείς με υψηλότερο κίνδυνο στην κλίμακα Apfel ή ιστορικό PONV σε προηγούμενες χειρουργικές επεμβάσεις με γενική αναισθησία[1-3].

108. Η χρήση εισπνεόμενων αναισθητικών θα πρέπει να αποφεύγεται σε ασθενείς με υψηλό κίνδυνο PONV.

Μέτριο επίπεδο τεκμηρίωσης. Ισχυρή σύσταση.

ΒΙΒΛΙΟΓΡΑΦΙΚΕΣ ΑΝΑΦΟΡΕΣ

1. Sneyd JR, Carr A, Byrom WD, Bilski AJ. A meta-analysis of nausea and vomiting following maintenance of anaesthesia with propofol or inhalational agents. Eur J Anaesthesiol 1998;15(4):433-45.
2. Apfel CC, Kranke P, Katz MH, Goepfert C, Papenfuss T, Rauch S, et al. Volatile anaesthetics may be the main cause of early but not delayed postoperative vomiting: a randomized controlled trial of factorial design. British Journal of Anaesthesia 2002;88(5):659-68.
3. Apfel CC, Korttila K, Abdalla M, Kerger H, Turan A, Vedder I, et al. A factorial trial of six interventions for the prevention of postoperative nausea and vomiting. N Engl J Med 2004;350(24):2441-51.

Μειώστε τη χρήση οπιοειδών

Μία από τις ανεπιθύμητες ενέργειες των οπιοειδών είναι η αύξηση της PONV, ειδικά σε ασθενείς με υψηλότερο κίνδυνο και μεγάλης διάρκειας χειρουργικές επεμβάσεις που απαιτούν μεγαλύτερη αναλγησία. Επομένως, η πολυπαραγοντική αναλγησία με διαφορετικές οικογένειες φαρμάκων, η χρήση τεχνικών περιοχικής αναισθησίας και η τοπική αναισθησία συνιστώνται για τη μείωση της συνολικής δόσης των διεγχειρητικών και μετεγχειρητικών οπιοειδών[1,2].

109. Συνιστάται η ελαχιστοποίηση της χρήσης των διεγχειρητικών οπιοειδών, και ιδιαίτερα των μετεγχειρητικών.

Υψηλό επίπεδο τεκμηρίωσης. Ισχυρή σύσταση.

ΒΙΒΛΙΟΓΡΑΦΙΚΕΣ ΑΝΑΦΟΡΕΣ

1. Roberts GW, Bekker TB, Carlsen HH, Moffatt CH, Slattery PJ, McClure AF. Postoperative nausea and vomiting are strongly influenced by postoperative opioid use in a dose-related manner. Anesth Analg 2005;101(5):1343-8.
2. Guay J, Nishimori M, Kopp S. Epidural local anaesthetics versus opioid-based analgesic regimens for postoperative gastrointestinal paralysis, vomiting and pain after abdominal surgery. Cochrane Database of Syst Rev 2016 Jul 16;7:CD001893.

ΘΕΡΑΠΕΙΑ ΚΑΙ ΑΝΤΙΕΜΕΤΙΚΗ ΠΡΟΦΥΛΑΞΗ ΣΥΜΦΩΝΑ ΜΕ ΤΗΝ ΚΛΙΜΑΚΑ ΚΙΝΔΥΝΟΥ APFEL:

Χαμηλός κίνδυνος (Apfel 0-1)

Η προφύλαξη δεν ενδείκνυται σε όλους τους ασθενείς με Apfel 0-1, εκτός από χειρουργικές επεμβάσεις στις οποίες η PONV ενέχει σημαντικό κίνδυνο επιπλοκών καθώς και σε χειρουργικές επεμβάσεις με υψηλότερο κίνδυνο εμέτου (χολοκυστεκτομές, γυναικολογικές ή λαπαροσκοπικές επεμβάσεις, επεμβάσεις στομάχου και οισοφάγου, νευροχειρουργική κ.λπ.), στις οποίες συνιστάται φαρμακολογική προφύλαξη με μονοθεραπεία[1-3]. Η δεξαμεθαζόνη (4 mg ενδοφλέβια κατά την εισαγωγή στην αναισθησία), η δροπεριδόλη (0,625–1,25 mg ενδοφλέβια στο τέλος της επέμβασης) και η ονδανσετρόνη (4 mg ενδοφλέβια στο τέλος της επέμβασης) έχουν παρόμοια αποτελεσματικότητα[4-6]. Η χρήση δεξαμεθαζόνης ή δροπεριδόλης έχει το πλεονέκτημα ότι διατηρεί την ονδανσετρόνη ως θεραπεία σε περίπτωση αποτυχίας της προφύλαξης.[6]

110. Η αντιεμετική προφύλαξη με μονοθεραπεία θα πρέπει να πραγματοποιείται σε ασθενείς με Apfel 0-1 μόνο σε χειρουργική επέμβαση με υψηλότερο κίνδυνο PONV.

Μέτριο επίπεδο τεκμηρίωσης. Ισχυρή σύσταση.

ΒΙΒΛΙΟΓΡΑΦΙΚΕΣ ΑΝΑΦΟΡΕΣ

1. Wang JJ, Ho ST, Tzeng JI, Tang CS. The effect of timing of dexamethasone administration on its efficacy as a prophylactic antiemetic for postoperative nausea and vomiting. Anesth Analg 2000;91(1):136-9.

2. Henzi I, Sonderegger J, Tramèr MR. Efficacy, dose-response, and adverse effects of droperidol for prevention of postoperative nausea and vomiting. Can J Anesth 2000;47(6):537- 51.

3. Apfel CC, Korttila K, Abdalla M, Kerger H, Turan A, Vedder I, et al. A factorial trial of six interventions for the prevention of postoperative nausea and vomiting. N Engl J Med 2004; 350(24):2441-51.

4. Gómez-Arnau JI, Aguilar JL, Bovaira P, Bustos F, De Andrés J, la Pinta de JC, et al. Recomendaciones de prevención y tratamiento de las náuseas y vómitos postoperatorios y/o asociados a las infusiones de opioides. Rev Esp Anestesiol Reanim 2010;57(8):508-24.

5. Gan TJ, Diemunsch P, Habib AS, Kovac A, Kranke P, Meyer TA, et al. Consensus Guidelines for the Management of Postoperative Nausea and Vomiting. Anesth Analg 2014;118(1):85-113.

6. Veiga-Gil L, Pueyo J, López-Olaondo L. Náuseas y vómitos postoperatorios: fisiopatología, factores de riesgo, profilaxis y tratamiento. Rev Esp Anestesiol Reanim 2017;64(4):223-32.

Μέτριος κίνδυνος (Apfel 2-3)

Ενδείκνυνται μέτρα για τη μείωση των βασικών κινδύνων, καθώς και φαρμακολογική προφύλαξη με μονοθεραπεία. Η φαρμακολογική προφύλαξη με διπλή θεραπεία (δεξαμεθαζόνη και δροπεριδόλη ή ονδανσετρόνη) θα πρέπει να πραγματοποιείται σε χειρουργικές επεμβάσεις στις οποίες η PONV ενέχει σημαντικό κίνδυνο επιπλοκών καθώς και σε χειρουργεία με υψηλότερο κίνδυνο εμέτου. Ο συνδυασμός δεξαμεθαζόνης και δροπεριδόλης έχει το πλεονέκτημα ότι διατηρεί την ονδανσετρόνη για θεραπεία σε περίπτωση αποτυχίας της προφύλαξης[1-3].

111. Η αντιεμετική προφύλαξη θα πρέπει να πραγματοποιείται ως μονοθεραπεία σε ασθενείς με αξιολόγηση Apfel 2–3 και ως διπλή θεραπεία εάν η χειρουργική επέμβαση έχει υψηλότερο κίνδυνο PONV.

Υψηλό επίπεδο τεκμηρίωσης. Ισχυρή σύσταση.

ΒΙΒΛΙΟΓΡΑΦΙΚΕΣ ΑΝΑΦΟΡΕΣ

1. Gan, TJ, Belani KG, Bergese S; Chung FM, Diemunsch P, Habib AS, et al. Fourth Consensus Guidelines for the Management of Postoperative Nausea and Vomiting. Anesth Analg 2020;131:411-448.

2. Gómez-Arnau JI, Aguilar JL, Bovaira P, Bustos F, De Andrés J, la Pinta de JC, et al. Recomendaciones de prevención y tratamiento de las náuseas y vómitos postoperatorios y/o asociados alas infusiones de opioides. Rev Esp Anestesiol Reanim 2010;57(8):508-24.

3. Veiga-Gil L, Pueyo J, López-Olaondo L. Náuseas y vómitos postoperatorios: fisiopatología, factores de riesgo, profilaxis y tratamiento. Rev Esp Anestesiol Reanim 2017;64(4):223-32.

Υψηλού κινδύνου (Apfel 4)

Ενδείκνυται η λήψη μέτρων για τη μείωση των βασικών κινδύνων και η φαρμακευτική προφύλαξη με διπλή θεραπεία. Η φαρμακολογική προφύλαξη με τριπλή θεραπεία (δεξαμεθαζόνη, δροπεριδόλη και ονδανσετρόνη, χορήγηση στο τέλος της χειρουργικής επέμβασης) θα πρέπει να πραγματοποιείται σε χειρουργικές επεμβάσεις στις οποίες η PONV ενέχει σημαντικό κίνδυνο επιπλοκών και σε χειρουργεία με υψηλότερο κίνδυνο εμέτου[1,2].

112. Συνιστάται η χορήγηση αντιεμετικής προφύλαξης με διπλή θεραπεία σε ασθενείς με αξιολόγηση Apfel 4 και με τριπλή θεραπεία εάν η χειρουργική επέμβαση έχει υψηλότερο κίνδυνο PONV.

Υψηλό επίπεδο τεκμηρίωσης. Ισχυρή σύσταση.

ΒΙΒΛΙΟΓΡΑΦΙΚΕΣ ΑΝΑΦΟΡΕΣ

1. Gan, TJ, Belani KG, Bergese S; Chung FM, Diemunsch P, Habib AS, et al. Fourth Consensus Guidelines for the Management of Postoperative Nausea and Vomiting. Anesth Analg 2020;131:411-448.

2. Veiga-Gil L, Pueyo J, López-Olaondo L. Náuseas y vómitos postoperatorios: fisiopatología, factores de riesgo, profilaxis y tratamiento. Rev Esp Anestesiol Reanim 2017;64(4):223- 32.

113. Η χρήση περιφερικών ανταγωνιστών υποδοχέων οπιοειδών αποτρέπει την εμφάνιση ειλεού στην μετεγχειρητική περίοδο.

Μέτριο επίπεδο τεκμηρίωσης. Ισχυρή σύσταση.

ΒΙΒΛΙΟΓΡΑΦΙΚΕΣ ΑΝΑΦΟΡΕΣ

1. Schwenk ES, Grant AE, Torjman MC, SE McNulty, JL Baratta, MD* and ER Viscusi. The efficacy of peripheral opioid antagonists in opioid-induced constipation and postoperative ileus: a systematic review of the literature. Reg Anesth Pain Med. 2017;42:767-777.

7.1.4 ΜΕΤΕΓΧΕΙΡΗΤΙΚΑ

Μετεγχειρητικά μέτρα θέρμανσης

Σε περίπτωση μετεγχειρητικής υποθερμίας, θα πρέπει να χρησιμοποιούνται ενεργητικά συστήματα θέρμανσης του δέρματος έναντι παθητικών συστημάτων[1]. Τα μέτρα αυτά πρέπει να εφαρμόζονται όσο το δυνατόν νωρίτερα[2]. Ανάμεσα στα ενεργητικά συστήματα θέρμανσης του δέρματος, οι πιο αξιολογημένες στρατηγικές είναι η αγωγή θερμότητας και η συναγωγή με ζεστό αέρα, με τις στρατηγικές να είναι οικονομικά αποδοτικές ακόμη και σε ασθενείς με χαμηλότερο χειρουργικό κίνδυνο και μικρή διάρκεια χειρουργικής επέμβασης[3].

114. Η μετεγχειρητική υποθερμία θα πρέπει να αντιμετωπίζεται με χορήγηση θερμότητας μέσω αγωγής ή συναγωγής έως ότου επιτευχθεί νορμοθερμία.

Υψηλό επίπεδο τεκμηρίωσης. Ισχυρή σύσταση.

ΒΙΒΛΙΟΓΡΑΦΙΚΕΣ ΑΝΑΦΟΡΕΣ

1. Torossian A, Bräuer A, Höcker J, Bein B, Wulf H, Horn EP. Preventing inadvertent perioperative hypothermia. Clinical Practice Guideline. Dtsch Arztebl Int. 2015 Mar 6; 112(10): 166-72. doi: 10.3238/arztebl.2015.0166. PMID: 25837741.

2. Warttig S, Alderson P, Campbell G, Smith AF. Interventions for treating inadvertent postoperative hypothermia. Cochrane Database Syst Rev. 2014 Nov 20;(11):CD009892. doi: 10.1002/14651858.CD009892.pub2. Review. PubMed PMID: 25411963.3.

3. Calvo Vecino JM, Casans Francés R, Ripollés Melchor J, Marín Zaldívar C, Gómez Ríos MA, Pérez Ferrer A, et al. Clinical practice guideline. Unintentional perioperative hypothermia. Rev Esp Anestesiol Reanim. 2018 Dec;65(10):564-588. doi: 10.1016/j.redar.2018.07.

006. Epub 2018 Nov 15. English, Spanish. PubMed PMID: 30447894.

Περιεγχειρητική χορήγηση ΜΣΑΦ

Η χρήση των ΜΣΑΦ για τον έλεγχο του πόνου ως επικουρική θεραπεία σχετίζεται με μείωση της κατανάλωσης οπιοειδών και βελτίωση της άνεσης του ασθενούς. Επιπλέον, η χρήση των ΜΣΑΦ θα μπορούσε να είναι ίση, όσον αφορά την αναλγητική ισχύ, με τη διήθηση των σημείων εισόδου των τροκάρ με τοπικά αναισθητικά, και οι εκλεκτικοί αναστολείς της κυκλοοξυγενάσης-2 θα μπορούσαν να έχουν κάποια επίδραση στη βελτίωση της μετεγχειρητικής λειτουργίας του εντέρου[1].

115. Τα ΜΣΑΦ θα πρέπει να χρησιμοποιούνται ως επικουρική θεραπεία για τον έλεγχο του πόνου σε ασθενείς που έχουν υποβληθεί σε μείζονα κοιλιακή χειρουργική επέμβαση.

Υψηλό επίπεδο τεκμηρίωσης. Ισχυρή σύσταση.

ΒΙΒΛΙΟΓΡΑΦΙΚΕΣ ΑΝΑΦΟΡΕΣ

1. Martinez V, Beloeil H, Marret E, Fletcher D, Ravaud P, Trinquart L. Non-opioid analgesics in adults after major surgery: systematic review with network meta-analysis of randomized trials. British Journal of Anaesthesia. 2017;118:22-31.

116. Δεν συνιστάται η τακτική χρήση τσίχλας.

Χαμηλό επίπεδο τεκμηρίωσης. Ασθενής σύσταση.

ΒΙΒΛΙΟΓΡΑΦΙΚΕΣ ΑΝΑΦΟΡΕΣ

1. Gregg Nelson, Jamie Bakkum-Gamez Eleftheria Kalogera, Gretchen Glaser Alon Altman, Larissa A Meyer, Jolyn S Taylor, et al. Guidelines for perioperative care in gynecologic oncology: Enhanced Recovery After Surgery (ERAS) Society recommendation 2019 update. Int J Gynecol Cancer. 2019;0:1-18.
2. de Leede EM, van Leersum NJ, Kroon HM, van Weel V, van der Sijp JRM Bonsing BA; Kauwgomstudie Consortium. Multicentre randomized clinical trial of the effect of chewing gum after abdominal surgery. Br J Surg. 2018;105:820-828.

3. Gustafsson O, Scott MJ, Hubner J, Nygren J, Demartines N, Francis N, et al. Guidelines for Perioperative Care in Elective Colorectal Surgery: Enhanced Recovery After Surgery (ERAS) Society Recommendations: 2018. World J Surg. 2019, 43:659-695.
4. Short V, Herbert G, Perry R, Atkinson C, Ness AR, Penfold C, et al. Chewing gum for postoperative recovery of gastrointestinal function. Cochrane Database Syst Rev.2015;CD006506. pub3.

Θεραπεία της PONV σε ασθενείς με αποτυχημένη προφύλαξη

Θεραπεία εγκατεστημένης PONV: εάν δεν έχει χορηγηθεί προφύλαξη, θα μπορούσε να χρησιμοποιείται χαμηλή δόση ονδανσετρόνης (1 mg ενδοφλέβια). Εάν έχει πραγματοποιηθεί προφύλαξη και έχουν περάσει περισσότερες από 6 ώρες από τη χορήγησή της, θα πρέπει να χρησιμοποιηθεί ένα αντιεμετικό διάσωσης (rescue) από διαφορετική οικογένεια από αυτήν που χρησιμοποιήθηκε για την προφύλαξη (ονδανσετρόνη 1 mg ενδοφλέβια ή δροπεριδόλη 0,625–1,25 mg ενδοφλέβια) εκτός από τη δεξαμεθαζόνη, της οποίας η επανάληψη δεν συνιστάται[1,2].

117. Σε εγκατεστημένη ναυτία και έμετο, οι εκλεκτικοί ανταγωνιστές των υποδοχέων σεροτονίνης 5-HT3 (ονδανσετρόνη) είναι η θεραπεία εκλογής, ακολουθούμενοι εάν οι ασθενείς δεν ανταποκρίνονται από διαφορετική οικογένεια αντιεμετικών φαρμάκων εκτός από τη δεξαμεθαζόνη.

Υψηλό επίπεδο τεκμηρίωσης. Ισχυρή σύσταση.

ΒΙΒΛΙΟΓΡΑΦΙΚΕΣ ΑΝΑΦΟΡΕΣ

1. Gan, TJ, Belani KG, Bergese S; Chung FM, Diemunsch P, Habib AS, et al. Fourth Consensus Guidelines for the Management of Postoperative Nausea and Vomiting. Anesth Analg. 2020;131:411-448.
2. Veiga-Gil L, Pueyo J, López-Olaondo L. Náuseas y vómitos postoperatorios: fisiopatología, factores de riesgo, profilaxis y tratamiento. Rev Esp Anestesiol Reanim 2017;64(4):223-32.

118. Η χρήση καθαρτικών όπως η δισακοδύλη (στη χειρουργική του παχέος εντέρου), το οξείδιο του μαγνησίου από το στόμα (στην υστερεκτομή), το daikenchuto (ιαπωνικό έγχυμα βοτάνων, στη γαστρεκτομή) και ο καφές (στη χειρουργική του παχέος εντέρου) θα μπορούσε να αποτρέψει την εμφάνιση του ειλεού.

Χαμηλό επίπεδο τεκμηρίωσης. Ασθενής σύσταση.

ΒΙΒΛΙΟΓΡΑΦΙΚΕΣ ΑΝΑΦΟΡΕΣ

1. Zingg U, Miskovic D, Pasternak I et al (2008) Effect of bisacodyl on postoperative bowel motility in elective colorectal surgery: a prospective, randomized trial. Int J Colorectal Dis 23:1175-1183.

2. Hansen CT, Sorensen M, Moller C et al (2007) Effect of laxatives on gastrointestinal functional recovery in fast-track hysterectomy: a double-blind, placebo-controlled randomized study. Am J Obstet Gynecol 196(311):311-317.

3. Yoshikawa K, Shimada M, Wakabayashi G et al (2015) Effect of daikenchuto, a traditional japanese herbal medicine, after total gastrectomy for gastric cancer: a multicenter, randomized, double-blind, placebo-controlled, phase II trial. J Am Coll Surg 221:571-578.

4. Muller SA, Rahbari NN, Schneider F et al (2012) Randomized clinical trial on the effect of coffee on postoperative ileus following elective colectomy. Br J Surg 99:1530-1538.

5. Dulskas A, Klimovskij M, Vitkauskiene M et al (2015) Effect of coffee on the length of postoperative ileus after elective laparoscopic left-sided colectomy: a randomized, prospective single-center study. Dis Colon Rectum 58:1064-1069.

Ανοσοδιατροφή (μετεγχειρητικά)

Η ανοσοδιατροφή είναι ένα θέμα που έχει συζητηθεί έντονα από τη δεκαετία του 1990, ειδικά στο πλαίσιο της χειρουργικής του καρκίνου[1]. Ορισμένες ανασκοπήσεις και μετα-αναλύσεις έχουν δείξει τα ευεργετικά αποτελέσματα της ανοσοδιατροφής αθροίζοντας τα αποτελέσματα των RCT σε όλους τους τύπους ασθενών και εξετάζοντας ολόκληρη την περιεγχειρητική περίοδο. Ωστόσο, άλλες μελέτες δεν βρήκαν πρόσθετο όφελος από τη χρήση

της ανοσοδιατροφής έναντι των τυπικών συμπληρωμάτων χρησιμοποιώντας παρόμοιες μεθόδους[2].

Σύμφωνα με τις κλινικές κατευθυντήριες οδηγίες του ESPEN του 2017 για την κλινική διατροφή και τη χειρουργική, συγκεκριμένα διαλύματα με ανοσοθρεπτικά συστατικά θα πρέπει να χορηγούνται περιεγχειρητικά ή τουλάχιστον μετεγχειρητικά σε υποσιτισμένους ασθενείς που υποβάλλονται σε μείζονα χειρουργική επέμβαση για καρκίνο, με μέτριο βαθμό σύστασης (SIGN)[3]. Δεν υπάρχουν σαφείς ενδείξεις για τη χρήση της σε σύγκριση με τα τυπικά από του στόματος συμπληρώματα αποκλειστικά στην προεγχειρητική περίοδο.

Συνεχίζουν να δημοσιεύονται μετα-αναλύσεις σχετικές με το θέμα, με ορισμένα κοινά θετικά αποτελέσματα, με δεδομένα που δεν είναι πάντα υψηλά[4,5].

119. Η ανοσοδιατροφή φαίνεται να συνιστάται σε υποσιτιζόμενους ασθενείς που υποβάλλονται σε επέμβαση για καρκίνο του γαστρεντερικού συστήματος, λόγω της μείωσης των λοιμωδών επιπλοκών και μιας πιθανής ελάττωσης της διάρκειας της νοσηλείας.

Χαμηλό επίπεδο τεκμηρίωσης. Ισχυρή σύσταση.

ΒΙΒΛΙΟΓΡΑΦΙΚΕΣ ΑΝΑΦΟΡΕΣ

1. Arends J, Bachmann P, Baracos V, et al. ESPEN guidelines on nutrition in cancer patients. Clin Nutr 2017; 36:11-48.

2. Hegazi RA, Hustead DS, Evans DC. Properative standard oral nutrition supplements vs immunonutrition: results of a systematic review and meta-analysis. J Am Coll Surg 2014; 219: 1078-1087.

3. Weimann A, Braga M, Carli F, Higashiguchi T, Hübner M, Klek S, et al. ESPEN guideline: Clinical nutrition in surgery. Clin Nutr 2017; 36: 623-650.

4. Probst P, Ohmann S, Klaiber U, Hüttner FJ, Billeter AT, Ulrich A, Büchler MW, Diener MK. Meta-analysis of immunonutrition in major abdominal surgery. Br J Surg 2017; 104: 1594-1608.

5. Adiamah A, Skorepa P, Weimann A, Lobo DN. The impact of preoperative immune modulating nutrition on outcomes in patients undergoing surgery for

gastrointestinal surgery for gastrointestinal cancer. Ann Surg 2019; 270: 247-256.

Μετεγχειρητικός πόνος

Ο έλεγχος του μετεγχειρητικού πόνου επιτρέπει ταχεία ανάρρωση και πολυπαραγοντική αποκατάσταση. Η αναλγησία είναι σημαντική τις πρώτες 24-48 ώρες για να επιτραπεί η έγκαιρη κινητοποίηση, η μείωση του παραλυτικού ειλεού και η νοσηλεία στο νοσοκομείο. Για το λόγο αυτό, είναι σημαντικό να μειωθούν οι συγκεντρώσεις των τοπικών αναισθητικών μέσω της θωρακικής επισκληρίδιου οδού στην μετεγχειρητική περίοδο στις ανοιχτές κοιλιακές επεμβάσεις, ώστε να επιτευχθεί ένας αισθητικός αποκλεισμός των εμπλεκόμενων μεταμερών σε συνδυασμό με τον μικρότερο δυνατό κινητικό αποκλεισμό στα κάτω άκρα[1,2]. Μετά τις πρώτες 48 ώρες, ο επισκληρίδιος καθετήρας πρέπει να αφαιρεθεί για να μειωθεί ο κίνδυνος λοίμωξης και να διασφαλιστεί η κινητοποίηση χωρίς κινητικό αποκλεισμό. Για να επιτευχθεί αυτό, η χρήση εναλλακτικών φαρμάκων όπως τα ΜΣΑΦ και η παρακεταμόλη είναι σημαντική, για την ελαχιστοποίηση της χρήσης ενδοφλέβια οπιοειδών, αφήνοντας τη χρήση τους για τη διάσωση του έντονου μη ελεγχόμενου πόνου[3]. Η μετεγχειρητική χρήση γκαμπαπεντίνης, αναστολέων NMDA όπως η κεταμίνη, καθώς και υψηλές δόσεις οπιοειδών δεν συνιστάται. Σε ορισμένες περιπτώσεις, στις οποίες δεν είναι διαθέσιμες τεχνικές επισκληρίδιου αναλγησίας στις ανοιχτές χειρουργικές επεμβάσεις, μπορεί να εξεταστεί η χρήση λιδοκαΐνης σε συνεχή έγχυση τις πρώτες 24 ώρες ή οι αποκλεισμοί δια μέσου των περιτονιών (interfascial blocks) όπως ο αποκλεισμός στο επίπεδο του εγκάρσιου κοιλιακού μυός (TAP) ή του τετραγώνου οσφυϊκού[4-7].

120. Η χρήση επισκληρίδιου αναλγησίας συνιστάται κατά τις πρώτες 24–48 ώρες μετά την επέμβαση, όπως και η αφαίρεσή της μετά από αυτήν την αρχική περίοδο ελέγχου του πόνου, μειώνοντας τις συγκεντρώσεις των τοπικών αναισθητικών με τη χρήση επισκληρίδιων οπιοειδών με σκοπό τη μείωση του κινητικού αποκλεισμού ώστε να επιτραπεί η κινητοποίηση.

Υψηλό επίπεδο τεκμηρίωσης. Ισχυρή σύσταση.

121. Η χρήση παρακεταμόλης και ΜΣΑΦ συνιστάται για τον μετεγχειρητικό έλεγχο του πόνου με δόσεις διάσωσης οπιοειδών σε σοβαρό μη ελεγχόμενο πόνο σε ασθενείς με επισκληρίδιο αναλγησία ή άλλες τεχνικές τοπικής ή περιοχικής αναλγησίας.

Υψηλό επίπεδο τεκμηρίωσης. Ισχυρή σύσταση.

ΒΙΒΛΙΟΓΡΑΦΙΚΕΣ ΑΝΑΦΟΡΕΣ

1. Salicath JH, Yeoh ECY, Bennett MH. Epidural analgesia versus patient-controlled intravenous analgesia for pain following intra-abdominal surgery in adults. Cochrane Database of Systematic Reviews. 2018;8: CD010434.

2. Guay J, Nishimori M, Kopp S. Epidural local anaesthetics versus opioid-based analgesic regimens for postoperative gastrointestinal paralysis, vomiting and pain after abdominal surgery. Cochrane Database of Systematic Reviews 2016;7:CD001893.

3. Martinez V, Beloeil H, Marret E, Fletcher D, Ravaud P, Trinquart L. Non-opioid analgesics in adults after major surgery: systematic review with network meta-analysis of randomized trials. British Journal of Anaesthesia. 2017;118:22-31.

4. MacFater WS, Rahiri J-L, Lauti M, Su'a B, Hill AG. Intravenous lignocaine in colorectal surgery: a systematic review. ANZ J Surg. 2017;87:879-85.

5. Weibel S, Jokinen J, Pace NL, Schnabel A, Hollmann MW, Hahnenkamp K, et al. Efficacy and safety of intravenous lidocaine for postoperative analgesia and recovery after surgery: a systematic review with trial sequential analysis. Br J Anaesth. 2016;116:770-83.

6. Weibel S, Jelting Y, Pace NL, Helf A, Eberhart LH, Hahnenkamp K, et al. Continuous intravenous perioperative lidocaine infusion for postoperative pain and recovery in adults. Cochrane Database Syst Rev. 2018;6:CD009642.

7. Baeriswyl M, Zeiter F, Piubellini D, Kirkham KR, Albrecht E. The analgesic efficacy of transverse abdominis plane block versus epidural analgesia: a systematic review with meta- analysis. Medicine. 2018;97(26).

Πρώιμη μετεγχειρητική σίτιση

Παραδοσιακά, ήταν κοινή πρακτική να μην ταΐζουμε τους ασθενείς μετεγχειρητικά έως ότου έχουν κένωση ή διέλευση αερίων. Η πρώιμη από του στόματος σίτιση δεν μεταβάλλει την επούλωση της γραμμής συρραφής στο κόλον ή στο ορθό και συντομεύει τη νοσηλεία στο νοσοκομείο, όπως αναφέρεται σε μια συστηματική ανασκόπηση του Cochrane. Πιο πρόσφατες μετα-αναλύσεις δείχνουν προφανή οφέλη σε σχέση με τη μετεγχειρητική ανάρρωση και την επίπτωση των λοιμώξεων. Μια μετα-ανάλυση 15 μελετών (8 από αυτές RCT), με 2.112 ασθενείς που υποβλήθηκαν σε χειρουργική επέμβαση στο ανώτερο γαστρεντερικό, έδειξε σημαντικά μικρότερη νοσηλεία στο νοσοκομείο, χωρίς διαφορές στις επιπλοκές[1].

Η ποσότητα της αρχικής από του στόματος σίτισης πρέπει να προσαρμόζεται στην κατάσταση της γαστρεντερικής λειτουργίας και στην ατομική ανοχή[2].

Συνολικά, υπάρχουν καλές ενδείξεις για τα οφέλη και την ανοχή της πρώιμης σίτισης στην μετεγχειρητική περίοδο της χειρουργικής του παχέος εντέρου. Τα οφέλη είναι λιγότερο σαφή σε ηλικιωμένους ασθενείς με χειρουργική επέμβαση στο ανώτερο γαστρεντερικό και στο πάγκρεας. Δεν υπάρχουν ελεγχόμενα δεδομένα σε ασθενείς με οισοφαγεκτομή.

122. Η πρώιμη μετεγχειρητική σίτιση θα πρέπει να ξεκινά το συντομότερο δυνατό, εντός ωρών μετά την επέμβαση στους περισσότερους ασθενείς.

Μέτριο επίπεδο τεκμηρίωσης (στην χειρουργική του παχέος εντέρου). Ισχυρή σύσταση.

ΒΙΒΛΙΟΓΡΑΦΙΚΕΣ ΑΝΑΦΟΡΕΣ

1. Willcutts KF, Chung MC, Erenberg CL, Finn KL, Schirmer BD, Byham-Gray LD. Early oral feeding as compared with traditional timing of oral feeding after upper gastrointestinal surgery. Ann Surg 2016;264:54e63.

2. Weimann A, Braga M, Carli F, Higashiguchi T, Hübner M, Klek S, et al. ESPEN guideline: Clinical nutrition in surgery. Clin Nutr 2017; 36: 623-650.

Πρώιμη κινητοποίηση

Η ανάπαυση στο κρεβάτι μειώνει τη μυϊκή δύναμη και αυξάνει την αντίσταση στην ινσουλίνη και τον κίνδυνο πνευμονικών και θρομβοεμβολικών επιπλοκών. Τα στοιχεία είναι περιορισμένα σχετικά με το όφελος των παρεμβάσεων πρώιμης κινητοποίησης μετά τη χειρουργική επέμβαση[1,2]. Αν και η κινητοποίηση σχετίζεται με μικρότερη νοσηλεία στο νοσοκομείο, λίγες μελέτες έχουν διερευνήσει τον αντίκτυπο συγκεκριμένων στρατηγικών για την αύξηση της κινητοποίησης σε σύγκριση με το να επιτρέπεται η πρώιμη κινητοποίηση[3]. Υπάρχει μεγάλη διακύμανση στα διάφορα πρωτόκολλα για την εφαρμογή της πρώιμης κινητοποίησης, από κάποια κινητοποίηση στο 24ώρο έως και 8 ώρες την ημέρα μετεγχειρητικά[4]. Η αποτυχία της πρώιμης κινητοποίησης μπορεί να οφείλεται σε παράγοντες όπως ο ανεπαρκής έλεγχος του πόνου, η ενδοφλέβια λήψη υγρών, η χρήση σωλήνων και παροχετεύσεων, η παροχή κίνητρου στον ασθενή και οι προϋπάρχουσες συννοσηρότητες[5]. Θα πρέπει να ενθαρρυνθεί η έγκαιρη κινητοποίηση, αλλά η παροχή πρόσθετων πόρων για την υλοποίησή της πέρα από την ενσωμάτωσή της σε πολυπαραγοντικά πρωτόκολλα βελτιστοποίησης της μετεγχειρητικής ανάρρωσης δεν έδειξε οφέλη[6].

123. Συνιστάται η έγκαιρη κινητοποίηση μέσω εκπαίδευσης και ενθάρρυνσης των ασθενών για τη μείωση του αριθμού των ανεπιθύμητων ενεργειών.

Μέτριο επίπεδο τεκμηρίωσης. Ισχυρή σύσταση.

ΒΙΒΛΙΟΓΡΑΦΙΚΕΣ ΑΝΑΦΟΡΕΣ

1. Castelino T, Fiore JF Jr, Niculiseanu P et al The effect of early mobilization protocols on postoperative outcomes following abdominal and thoracic surgery: a systematic review. Surgery. 2016; 159:991-1003.
2. de Almeida EPM, de Almeida JP, Landoni G, et al Early mobilization programme improves functional capacity after major abdominal cancer surgery: a randomized controlled trial [with consumer summary] British Journal of Anaesthesia 2017;119(5):900-907.
3. Schaller SJ, Anstey M, Blobner M et al Early, goal-directed mobilisation in the surgical intensive care unit: a randomized controlled trial. Lancet. 2016; 388:1377-1388.

4. Fiore JF Jr, Castelino T, Pecorelli N, et al. Ensuring early mobilization within an enhanced recovery program for colorectal surgery: a randomized controlled trial. Ann Surg 2017; 266:223-231.
5. Gustafsson UO, Scott MJ, Hubner M, et al. Guidelines for Perioperative Care in Elective Colorectal Surgery: Enhanced Recovery After Surgery (ERAS_) Society Recommendations: 2018 World J Surg (2019) 43: 659-695. https://doi.org/10.1007/s00268-018-4844-y.
6. Wolk, S., Linke, S., Bogner, A. y col. Use of Activity Tracking in Major Visceral Surgery - the Enhanced Perioperative Mobilization Trial: a Randomized Controlled Trial J Gastrointest Surg 2019; 23: 1218. https://doi.org/10.1007/s11605-018-3998-0.

Αναπνευστική φυσιοθεραπεία

Η εκτέλεση αναπνευστικών ασκήσεων στην προεγχειρητική περίοδο οδηγεί σε μείωση των αναπνευστικών επιπλοκών στην μετεγχειρητική περίοδο[1]. Η χρήση αναπνευστικού σπιρόμετρου – εξασκητή αναπνοών (incentive spirometer) και η εξάσκηση των αναπνευστικών μυών είναι οι πιο μελετημένες τεχνικές. Η χρήση αναπνευστικού σπιρόμετρου – εξασκητή αναπνοών στις κοιλιακές επεμβάσεις, αν και φαίνεται να έχει θετικό αντίκτυπο στη λειτουργία των πνευμόνων και στην κίνηση του διαφράγματος κατά την άμεση μετεγχειρητική περίοδο, δεν έχει δείξει κανένα όφελος στην πρόληψη των μετεγχειρητικών επιπλοκών[2]. Η επιλεκτική εξάσκηση των εισπνευστικών μυών έχει αποδειχθεί ότι μειώνει τον κίνδυνο μετεγχειρητικών πνευμονικών επιπλοκών και τη νοσηλεία στο νοσοκομείο[3,4]. Προεγχειρητικές εκπαιδευτικές συνεδρίες και μαθήματα εξάσκησης που παρέχονται από φυσικοθεραπευτή, με στόχο να καθοδηγήσουν τον ασθενή στην εκτέλεση τεχνικών αναπνευστικής φυσιοθεραπείας και να τον ενημερώσουν για τη σημασία της σχέσης τους με την μετεγχειρητική περίοδο, έχει αποδειχθεί ότι επηρεάζουν τη μετεγχειρητική νοσηρότητα στις κοιλιακές επεμβάσεις, με μείωση των μετεγχειρητικών πνευμονικών επιπλοκών[5]. Είναι απαραίτητος ο συνδυασμός της φυσικοθεραπείας με άλλες παρεμβάσεις όπως η άσκηση και η αγωγή υγείας καθώς και η τροποποίηση της συνήθειας. Οι παρεμβάσεις πρέπει να εξατομικεύονται.

124. Συνιστάται προεγχειρητική και μετεγχειρητική αναπνευστική φυσιοθεραπεία.

Υψηλό επίπεδο τεκμηρίωσης. Ισχυρή σύσταση.

ΒΙΒΛΙΟΓΡΑΦΙΚΕΣ ΑΝΑΦΟΡΕΣ

1. Katsura M, Kuriyama A, Takeshima T, Fukuhara S, Furukawa TA. Preoperative inspiratory muscle training for postoperative pulmonary complications in adults undergoing cardiac and major abdominal surgery. Cochrane Database Syst Rev. 2015; 5;(10):CD010356. doi: 10.1002/14651858.CD010356.pub2.

2. Kalil-Filho FA, Campos ACL, Tambara EM, Tomé BKA, Treml CJ, Kuretzki CH, et al. Physiotherapeutic approaches and the effects on inspiratory muscle force in patients with chronic obstructive pulmonary disease in the preoperative preparation for abdominal surgical procedures. Arq Bras Cir Dig. 2019;32(2): e1439.

3. Kendall F, Oliveira J, Peleteiro B, Pinho P, Bastos PT. Inspiratory muscle training is effective to reduce postoperative pulmonary complications and length of hospital stay: a systematic review and meta-analysis. Disabil Rehabil. 2018; 40(8):864-82.

4. Alaparthi GK, Augustine AJ, Anand R, Mahale A. Comparison of Diaphragmatic Breathing Exercise, Volume and Flow Incentive Spirometry, on Diaphragm Excursion and Pulmonary Function in Patients Undergoing Laparoscopic Surgery: A Randomized Controlled Trial. Minim Invasive Surg 2016;1967532.

5. Karlsson E, Farahnak P, Franzén E, Nygren-Bonnier M, Dronkers J, van Meeteren N, et al. Feasibility of preoperative supervised home-based exercise in older adults undergoing colorectal cancer surgery - A randomized controlled design. PLoS One. 2019;14(7): e0219158.

Αντιμετώπιση μετεγχειρητικής αναιμίας

Παρά το γεγονός ότι είναι κοινή πρακτική, τα στοιχεία δείχνουν ότι η θεραπεία με σίδηρο από το στόμα, σε αναιμικούς ασθενείς με έλλειψη σιδήρου πριν από τη χειρουργική επέμβαση, δεν είναι πιο αποτελεσματική, ούτε είναι

καλύτερα ανεκτή, από το εικονικό φάρμακο για τη θεραπεία της μετεγχειρητικής αναιμίας[1-4].

125. Η από του στόματος χορήγηση αλάτων σιδήρου δεν συνιστάται στην άμεση μετεγχειρητική περίοδο για τη βελτίωση του επιπέδου της αιμοσφαιρίνης και τη μείωση του ρυθμού μετάγγισης.

Μέτριο επίπεδο τεκμηρίωσης. Ισχυρή σύσταση.

126 Αντίθετα, η μετεγχειρητική θεραπεία με ενδοφλέβιο σίδηρο προτείνεται για τη βελτίωση των επιπέδων αιμοσφαιρίνης και τη μείωση του ρυθμού μετάγγισης, ειδικά σε ασθενείς με χαμηλές αποθήκες σιδήρου ή/και μέτρια-σοβαρή μετεγχειρητική αναιμία.

Μέτριο επίπεδο τεκμηρίωσης. Ισχυρή σύσταση.

ΒΙΒΛΙΟΓΡΑΦΙΚΕΣ ΑΝΑΦΟΡΕΣ

1. Muñoz M, Acheson AG, Bisbe E, Butcher A, Gómez-Ramírez S, Khalafallah AA, Kehlet H, et al. An international consensus statement on the management of postoperative anaemia after major surgical procedures. Anaesthesia. 2018;73:1418-1431.
2. Leal-Noval SR, Muñoz M, Asuero M, Contreras E, García-Erce JA, Llau JV, et al. Spanish Expert Panel on Alternatives to Allogeneic Blood Transfusion. Spanish Consensus Statement on alternatives to allogeneic blood transfusion: the 2013 update of the “Seville Document”. Blood Transfus. 2013;11:585-610.
3. Gómez-Ramírez S, Maldonado-Ruiz MÁ, Campos-Garrigues A, Herrera A, Muñoz M. Short-term perioperative iron in major orthopedic surgery: state of the art. Vox Sang. 2019 Jan;114(1):3-16.
4. Laso-Morales MJ, Vives R, Gómez-Ramírez S, Pallisera-Lloveras A, Pontes C. Intravenous iron administration for post-operative anaemia management after colorectal cancer surgery in clinical practice: a single-centre, retrospective study. Blood Transfus. 2018;16:338-342.

ΜΕΤΑΓΓΙΣΗ

Εφαρμογή «περιοριστικών» κριτηρίων μετάγγισης

Υπάρχει γενική συναίνεση (τόσο σε εθνικό όσο και σε διεθνές επίπεδο) ως προς τη σύσταση της εφαρμογής «περιοριστικών» κριτηρίων μετάγγισης έναντι «φιλελεύθερων» κριτηρίων στην πλειονότητα των αιμοδυναμικά σταθερών ασθενών: χειρουργικοί ασθενείς (που υποβάλλονται σε ορθοπεδική και καρδιαγγειακή χειρουργική επέμβαση)[1-13], ασθενείς σε κρίσιμη κατάσταση (τραυματίες, σηπτικοί και παιδιατρικοί)[1,2,4-8], ασθενείς μετά τον τοκετό[14] και ακόμη και σε ασθενείς με αιμορραγία του γαστρεντερικού[15] (μετά από αιμορραγία από το ανώτερο γαστρεντερικό, σταθεροί και με χαμηλό κίνδυνο υποτροπής).

Αυτά τα «περιοριστικά» κριτήρια συνίστανται στη μοναδική κάθε φορά («μία τη φορά») χορήγηση συμπυκνωμένων ερυθρών αιμοσφαιρίων, με επαναξιολόγηση μετά από κάθε μονάδα μετάγγισης σε περίπτωση συμπτωμάτων ή σημείων υποξίας ή αναιμίας ή για διατήρηση της συγκέντρωσης αιμοσφαιρίνης πάνω από 7 g/dl σε βαρέως πάσχοντες ασθενείς[1-8], πάνω από 7,5 g/dl σε καρδιαγγειακούς χειρουργικούς ασθενείς[9-11], ή πάνω από 8 g/dl στην περίπτωση των παραγόντων καρδιαγγειακού κινδύνου[2,3,5-7,12,13].

Η Ισπανική Εταιρεία Αιματολογίας και Αιμοθεραπείας (SEHH) συνιστά «να μην γίνεται μετάγγιση μεγαλύτερου αριθμού συσκευασμένων ερυθρών αιμοσφαιρίων από ό,τι χρειάζεται για την ανακούφιση των συμπτωμάτων της αναιμίας ή για την επιστροφή του ασθενούς σε ένα ασφαλές εύρος αιμοσφαιρίνης (7 έως 8 g/dl σε σταθερούς μη καρδιακούς ασθενείς).»3

Η Ισπανική Εταιρεία Εντατικής Ιατρικής, Μονάδων Εντατικής Θεραπείας και Στεφανιαίων Μονάδων (La Sociedad Española de Medicina Intensiva, Crítica y Unidades Coronarias – SEMICYUC) συνιστά ότι «τα συμπυκνωμένα ερυθρά αιμοσφαίρια δεν πρέπει να μεταγγίζονται σε αιμοδυναμικά σταθερούς, μη αιμορραγικούς ασθενείς σε κρίσιμη κατάσταση, χωρίς καρδιολογική συμμετοχή ή/και συμμετοχή του κεντρικού νευρικού συστήματος, με συγκέντρωση αιμοσφαιρίνης μεγαλύτερη από 7 g/dl».

127. Η εφαρμογή «περιοριστικών» κριτηρίων για τη μετάγγιση συμπυκνωμένων ερυθρών αιμοσφαιρίων (ΣΕ) συνιστάται (εάν υπάρχουν συμπτώματα ή επίπεδο Hb <7 g/dl), στους περισσότερους νοσηλευόμενους ασθενείς (παθολογικούς, χειρουργικούς ή σε κρίσιμη κατάσταση), χωρίς ενεργό αιμορραγία και που είναι αιμοδυναμικά σταθεροί (συμπεριλαμβανομένων των σηπτικών ασθενών, των ασθενών με αιμορραγία από το ανώτερο γαστρεντερικό και των ασθενών με επιλόχεια αναιμία).

Υψηλό επίπεδο τεκμηρίωσης. Ισχυρή σύσταση.

128. Η εφαρμογή «περιοριστικών» κριτηρίων για μετάγγιση ΣΕ (Hb ≤7.5 g/dl) συνιστάται σε καρδιοχειρουργικούς ασθενείς

Μέτριο επίπεδο τεκμηρίωσης. Ισχυρή σύσταση.

129. Η εφαρμογή «περιοριστικών» κριτηρίων για μετάγγιση ΣΕ (Hb <8 g/dl) συνιστάται σε ασθενείς με ιστορικό καρδιαγγειακής νόσου που υποβλήθηκαν σε ορθοπεδική επέμβαση ή χειρουργική επέμβαση αποκατάστασης κατάγματος ισχίου.

Μέτριο επίπεδο τεκμηρίωσης. Ισχυρή σύσταση.

ΒΙΒΛΙΟΓΡΑΦΙΚΕΣ ΑΝΑΦΟΡΕΣ

1. Blood transfusion. Quality standard [QS138] Published date: December 2016 https:// www.nice.org.uk/guidance/qs138 (último acceso febrero 2020).

2. Muñoz Gómez M, Bisbe Vives E, Basora Macaya M, García Erce JA, Gómez Luque S, Leal- Noval SR, et al. Foro de debate: seguridad de las alternativas a la transfusión alogénica en el paciente quirúrgico y/o crítico. Med Intensiva. 2015;39:552-562.

3. Recomendaciones de «no hacer» de la Sociedad Española de Hematología y Hemoterapia. Proyecto COMPROMISO POR LA CALIDAD DE LAS SOCIEDADES CIENTÍFICAS EN ESPA- ÑA. http://www.mscbs.gob.es/organizacion/sns/planCalidadSNS/pdf/SOCIEDAD_ ESP_ HEMATOLOGIA_HEMOTERAPIA_0K.pdf (último acceso febrero 2020).

4. Recomendaciones de «no hacer» de la Sociedad Española de Medicina Intensiva, Crítica y Unidades Coronarias (SEMICYUC). Proyecto COMPROMISO POR LA CALIDAD DE LAS SOCIE- DADES CIENTÍFICAS EN

ESPAÑA. http://www.mscbs.gob.es/organizacion/sns/planCalidad SNS/pdf/SOCIEDAD_ESP_MIC_UNIDCORONARIAS_0K.pdf (último acceso febrero 2020)

5. Carson JL, Guyatt G, Heddle NM, Grossman BJ, Cohn CS, Fung MK, et al. Clinical Practice Guidelines From the AABB: Red Blood Cell Transfusion Thresholds and Storage. JAMA. 2016;316:2025-2035.

6. Mueller MM, Van Remoortel H, Meybohm P, Aranko K, Aubron C, Burger R, et al. ICCPBM Frankfurt 2018 Group. Patient Blood Management: Recommendations from the 2018 Frankfurt Consensus Conference. JAMA. 2019;321:983-97. doi:10.1001/jama.2019.0554.

7. Ripollés Melchor J, Casans Francés R, Espinosa Á, Martínez Hurtado E, Navarro Pérez R, Abad Gurumeta A, et al; EAR Group Anesthesia Evidence Review. Restrictive versus liberal transfusion strategy for red blood cell transfusion in critically ill patients and in patients with acute coronary syndrome: a systematic review, meta-analysis and trial sequential analysis. Minerva Anestesiol. 2016;82:582-98.

8. Gustafsson UO, Scott MJ, Hubner M, et al. Guidelines for Perioperative Care in Elective Colorectal Surgery: Enhanced Recovery After Surgery (ERAS®) Society Recommendations: 2018. World J Surg. 2019;43:659-95.

9. Mazer CD, Whitlock RP, Fergusson DA, Hall J, Belley-Cote E, Connolly K, et al. TRICS Investigators and Perioperative Anesthesia Clinical Trials Group. Restrictive or Liberal Red-Cell Transfusion for Cardiac Surgery. N Engl J Med. 2017;377:2133-2144.

10. Mazer CD, Whitlock RP, Fergusson DA, Belley-Cote E, Connolly K, Khanykin B, Gregory AJ, et al; TRICS Investigators and Perioperative Anesthesia Clinical Trials Group. Six-Month Outcomes after Restrictive or Liberal Transfusion for Cardiac Surgery. N Engl J Med. 2018; 379:1224-1233.

11. Shehata N, Mistry N, da Costa BR, Pereira TV, Whitlock R, Curley GF, et al (Mazer CD). Restrictive compared with liberal red cell transfusion strategies in cardiac surgery: a meta-analysis. Eur Heart J. 2019;40:1081-1088. doi: 10.1093/eurheartj/ehy435.

12. Cortés-Puch I, Wiley BM, Sun J, Klein HG, Welsh J, Danner RL, et al. Risks of restrictive red blood cell transfusion strategies in patients with cardiovascular disease (CVD): a meta-analysis. Transfus Med. 2018;28:335-345.

13. Docherty AB, O‘Donnell R, Brunskill S, Trivella M, Doree C, Holst L, et al. Effect of restrictive versus liberal transfusion strategies on outcomes in patients with cardiovascular disease in a non-cardiac surgery setting: systematic review and meta-analysis. BMJ. 2016;352:i1351.
14. Prick BW, Jansen AJ, Steegers EA, Hop WC, Essink-Bot ML, Uyl-de Groot CA, et al. Transfusion policy after severe postpartum haemorrhage: a randomised non-inferiority trial. BJOG. 2014;121:1005-14.
15. Odutayo A, Desborough MJ, Trivella M, Stanley AJ, Dorée C, Collins GS, et al. Restrictive versus liberal blood transfusion for gastrointestinal bleeding: a systematic review and meta-analysis of randomised controlled trials. Lancet Gastroenterol Hepatol. 2017;2:354-360.

Συστάσεις για τη μετεγχειρητική αντιμετώπιση του τραύματος

Είτε έχει πραγματοποιηθεί σύγκλειση κατά πρώτο σκοπό, είτε επιβραδυμένη σύγκλειση κατά πρώτο σκοπό είτε σύγκλειση κατά δεύτερο σκοπό, τα σημεία κλειδιά για τη φροντίδα του χειρουργικού τραύματος είναι ο επαρκής καθαρισμός, η διαχείριση του εξιδρώματος και η πρόληψη σχετικών επιπλοκών, όπως η λοίμωξη του χειρουργικού πεδίου, η διάσπαση και ο πόνος.

Υπάρχουν πολλές λύσεις για την πλύση και τον καθαρισμό των τραυμάτων και ελάχιστα στοιχεία για τη χρήση τους. Για τον καθαρισμό, συνιστάται η χρήση φυσιολογικού διαλύματος καθώς είναι ισότονο διάλυμα και δεν παρεμβαίνει στη φυσιολογική διαδικασία επούλωσης[1]. Το πόσιμο νερό ή το αποσταγμένο νερό μπορεί επίσης να χρησιμοποιηθεί για τον καθαρισμό των τραυμάτων.

130. Καθαρίστε το χειρουργικό τραύμα με στείρο ισότονο φυσιολογικό ορό, πόσιμο νερό ή αποσταγμένο νερό.

Μέτριο επίπεδο τεκμηρίωσης. Ισχυρή σύσταση.

Χειρουργικά τραύματα με σύγκλειση κατά πρώτο σκοπό

Ορισμένες μελέτες δείχνουν ότι τα τοπικά αντιβιοτικά που εφαρμόζονται σε χειρουργικά τραύματα με επούλωση κατά πρώτο σκοπό πιθανώς μειώνουν

τον κίνδυνο λοίμωξης του χειρουργικού πεδίου σε σχέση με τη μη χρήση αντιβιοτικών και σε σχέση με τα τοπικά αντισηπτικά. Θα πρέπει να ληφθούν υπόψη οι ανεπιθύμητες ενέργειες στο δέρμα και ο πόνος[2].

131. Τοπικά αντιβιοτικά μπορούν να εφαρμοστούν σε χειρουργικά τραύματα με επούλωση κατά πρώτο σκοπό μετά από χειρουργική επέμβαση για την πρόληψη λοίμωξης του χειρουργικού πεδίου.

Χαμηλό επίπεδο τεκμηρίωσης. Ασθενής σύσταση.

Γενικά, συνιστάται ο χειρισμός του χειρουργικού τραύματος όσο το δυνατόν λιγότερο. Επί του παρόντος, δεν υπάρχουν συμπερασματικές μελέτες σχετικά με τη χρήση επιθεμάτων που να υποδεικνύουν ότι η κάλυψη χειρουργικών τραυμάτων με επούλωση κατά πρώτο σκοπό μειώνει τον κίνδυνο εμφάνισης λοίμωξης του χειρουργικού πεδίου ή ότι οποιοδήποτε επίθεμα είναι πιο αποτελεσματικό από ένα άλλο στη μείωση των ποσοστών λοίμωξης του χειρουργικού πεδίου ή στη βελτίωση των ουλών ή του πόνου[3]. Στα τραύματα με επούλωση κατά πρώτο σκοπό, όποτε είναι δυνατόν, το επίθεμα δεν πρέπει να αλλάζεται για τις πρώτες 24–48 ώρες[4].

132. Σε τραύματα με επούλωση κατά πρώτο σκοπό, όποτε είναι δυνατόν, συνιστάται να μην αλλάζεται το επίθεμα κατά τις πρώτες 24–48 ώρες.

Χαμηλό επίπεδο τεκμηρίωσης. Ασθενής σύσταση.

Η εφαρμογή ενός επιθέματος συνδεδεμένου με μια αντλία κενού, γνωστής ως θεραπεία τραυμάτων αρνητικής πίεσης (Negative Pressure Wound Therapy - NPWT), μπορεί να μειώσει το ποσοστό λοίμωξης του χειρουργικού πεδίου σε σύγκριση με τα τυπικά επιθέματα τραυμάτων, σύμφωνα με ορισμένες μελέτες χαμηλής βεβαιότητας που είναι κυρίως μικρές. Υπάρχει ακόμη μεγαλύτερη αβεβαιότητα σχετικά με το εάν η θεραπεία τραυμάτων αρνητικής πίεσης, σε σύγκριση με τα τυπικά επιθέματα, μειώνει τις περισσότερες επιπλοκές που σχετίζονται με τις χειρουργικές τομές, συμπεριλαμβανομένης της θνησιμότητας[5].

ΒΙΒΛΙΟΓΡΑΦΙΚΕΣ ΑΝΑΦΟΡΕΣ

1. Fernandez R, Griffiths R. Water for wound cleansing. Cochrane Database of Systematic Reviews 2012, Issue 2. Art. No.: CD003861. DOI: 10.1002/14651858.CD003861.pub3.

2. Heal CF, Banks JL, Lepper PD, Kontopantelis E, van Driel ML. Topical antibiotics for preventing surgical site infection in wounds healing by primary intention. Cochrane Database of Systematic Reviews 2016, Issue 11. Art. No.: CD011426. DOI: 10.1002/14651858. CD011426.pub2

3. Dumville JC, Gray TA, Walter CJ, Sharp CA, Page T, Macefield R, Blencowe N, Milne TKG, Reeves BC, Blazeby J. Dressings for the prevention of surgical site infection. Cochrane Database of Systematic Reviews 2016, Issue 12. Art. No.: CD003091. DOI: 10.1002/ 14651858.CD003091.pub4.

4. NICE Guideline Updates Team (UK). Surgical site infections: prevention and treatment. London: National Institute for Health and Care Excellence (UK); 2019 Apr. (NICE Guideline, No. 125.) Available from: https://www.ncbi.nlm.nih.gov/books/NBK542473/.

5. Webster J, Liu Z, Norman G, Dumville JC, Chiverton L, Scuffham P, Stankiewicz M, Chaboyer WP. Negative pressure wound therapy for surgical wounds healing by primary closure. Cochrane Database of Systematic Reviews 2019, Issue 3. Art. No.: CD009261. DOI: 10.1002/14651858.CD009261.pub4.

Ανοιχτά χειρουργικά τραύματα

Σε αυτά τα χειρουργικά τραύματα, η έκταση, το βάθος, ο όγκος του εξιδρώματος και ο κίνδυνος λοίμωξης αποτελούν πρόκληση στη διαχείρισή τους.

Αν και η απώλεια της συνέχειας του δέρματος διευκολύνει την πρόσβαση μικροοργανισμών στο σώμα, δεν υπάρχουν μέχρι σήμερα αξιόπιστα στοιχεία σχετικά με τη σχετική αποτελεσματικότητα των αντισηπτικών, των αντιβιοτικών και των αντιβακτηριακών προϊόντων για χρήση σε ανοιχτά χειρουργικά τραύματα[1].

Η NPWT είναι η πιο κοινή εναλλακτική λύση για την διαχείριση των τραυμάτων με επούλωση κατά δεύτερο σκοπό [2]. Μερικές μικρές μελέτες έχουν δείξει την ευεργετική επίδραση της NPWT στη μείωση της μεσοθωρακίτιδας και

της λοίμωξης του τραύματος του στέρνου μετά από στερνοτομή [3]. Μερικές μικρές τυχαιοποιημένες κλινικές μελέτες υποδεικνύουν μικρότερο χρόνο επούλωσης με τη χρήση της NPWT έναντι αλγινικών επιθεμάτων και επιθεμάτων σιλικόνης, χωρίς να είναι όμως οριστικά τα αποτελέσματα λόγω του μεγέθους του δείγματος[4].

133. Η χρήση θεραπείας τραυμάτων αρνητικής πίεσης μπορεί να μειώσει τον κίνδυνο λοίμωξης του χειρουργικού πεδίου και να συντομεύσει την επούλωση σε ανοιχτά χειρουργικά τραύματα, κυρίως σε χειρουργικές επεμβάσεις στην κοιλιά ή στον θώρακα.

Χαμηλό επίπεδο τεκμηρίωσης. Ασθενής σύσταση.

ΒΙΒΛΙΟΓΡΑΦΙΚΕΣ ΑΝΑΦΟΡΕΣ

1. Norman G, Dumville JC, Mohapatra D, Owens GL, Crosbie EJ. Antibiotics and antiseptics for Surgical wounds healing by secondary intention. Cochrane Database of Systematic Reviews 2016, Issue 3. Art. No.: CD011712. DOI: 10.1002/14651858.CD011712.pub2

2. García-Fernández F, Blasco-García M, Rueda-López J, Segovia-Gómez T. Cura avanzada de heridas: terapia de presión negativa, factores de crecimiento plaquetario, sustitutos epidérmicos y apósitos bioactivos. In: García-Fernández F, Soldevilla-Agreda J, Torra Bou J, editors. Atención Integralde las Heridas Crónicas - 2a edición. Logroño:GNEAUPP-FSJJ;2016. p. 531-45.

3. Broadus Zane Atkins, MD, Mary Kay Wooten, MSN, Jean Kistler, NP, Kista Hurley, PA-C, G. Chad Hughes, MD, and Walter G. Wolfe, MD. Does Negative Pressure Wound Therapy Have a Role in Preventing Post-sternotomy Wound Complications? Does Negative Pressure Wound Therapy Have a Role in Preventing Poststernotomy Wound Complications?

4. Dumville JC, Owens GL, Crosbie EJ, Peinemann F, Liu Z. Negative pressure wound therapy for treating surgical wounds healing by secondary intention. Cochrane Database of Systematic Reviews 2015, Issue 6. Art. No.: CD011278. DOI: 10.1002/14651858.CD011278.pub2.

Οδηγίες κατά το εξιτήριο

Το εξιτήριο και η παρακολούθηση των ασθενών πρέπει να προγραμματίζονται και να συμφωνούνται, λαμβάνοντας υπόψη τους ασθενείς και τους φροντιστές τους, ειδικά σε ηλικιωμένους ή εξαρτημένους ασθενείς. Οι οδηγίες προς κάθε ασθενή σχετικά με τη φροντίδα του θα πρέπει να είναι εξατομικευμένες. Κατά το εξιτήριο, πρέπει να διασφαλιστεί ότι ο ασθενής έχει κατανοήσει τη φροντίδα που πρέπει να λάβει και την παρακολούθηση στην οποία θα υποβληθεί. Η χρήση τυποποιημένων εγγράφων πληροφοριών βελτιώνει την κατανόηση από τους ασθενείς των πληροφοριών που λαμβάνουν κατά το εξιτήριο.

Ο ασθενής πρέπει να πάρει εξιτήριο με προγραμματισμένα τα ραντεβού για παρακολούθηση συμπεριλαμβανομένων εκείνων που αντιστοιχούν σε άλλες υπηρεσίες.

Οι εξατομικευμένες οδηγίες εξιτηρίου επηρεάζουν τη μέση νοσηλεία και τις επανεισαγωγές. Οι επαρκείς, κατανοητές και πλήρεις οδηγίες εξιτηρίου βελτιώνουν την ικανοποίηση των ασθενών.

Συνιστάται θεραπεία υποστήριξης κατά το εξιτήριο: φυσικοθεραπεία ή σωματική άσκηση, φροντίδα των στομιών και διατροφή.

Συνιστάται επίσης τηλεφωνική παρακολούθηση εντός των πρώτων 24 ωρών. Η επέκταση της τηλεφωνικής παρακολούθησης μπορεί να είναι σημαντική για ορισμένες παθολογίες.

134. Οι ασθενείς και οι φροντιστές τους θα πρέπει να λαμβάνουν εξατομικευμένες, κατανοητές και πλήρεις οδηγίες κατά το εξιτήριο. Ο προγραμματισμός του εξιτηρίου και η παροχή επαρκών πληροφοριών για τη φροντίδα μετά το εξιτήριο επηρεάζει τη μέση νοσηλεία και τις επανεισαγωγές.

Υψηλό επίπεδο τεκμηρίωσης. Ισχυρή σύσταση.

ΒΙΒΛΙΟΓΡΑΦΙΚΕΣ ΑΝΑΦΟΡΕΣ

1. Shepperd S, Lannin NA, Clemson LM, McCluskey A, Cameron ID, Barras SL. Discharge planning from hospital to home. Cochrane Database Syst Rev. 2013 Jan 31;(1):CD000313.

2. Younis J, Salerno G, Fanto D, Hadjipavlou M, Chellar D, Trickett JP. Focused preoperative patient stoma education, prior to ileostomy formation after anterior

resection, contributes to a reduction in delayed discharge within the enhanced recovery programme. Int J Colorectal Dis. 2012; 27(1):43-7.

Έλεγχοι (Audits)

Τα αποτελέσματα μιας μελέτης στην οποία χρησιμοποιήθηκε ένα οπτικό εργαλείο με τα δεδομένα ελέγχου των διαφόρων επαγγελματιών, βελτίωσαν την τήρηση των οδηγιών της διεγχειρητικής αντιβιοτικής χημειοπροφύλαξης, του ελέγχου της θερμοκρασίας, της στοχοκατευθυνόμενης ενδοφλέβιας χορήγησης υγρών, της προφύλαξης της μετεγχειρητικής ναυτίας και εμέτου και του μετεγχειρητικού περιορισμού υγρών[1].

135. Συνιστώνται έλεγχοι για την εφαρμογή των πρωτοκόλλων βελτιστοποίησης της μετεγχειρητικής ανάρρωσης με σκοπό την αξιολόγηση της κλινικής επάρκειας και αποτελεσματικότητας.

Μέτριο επίπεδο τεκμηρίωσης. Ισχυρή σύσταση.

ΒΙΒΛΙΟΓΡΑΦΙΚΕΣ ΑΝΑΦΟΡΕΣ

1. Bisch SP, Wells T, Gramlich L, Faris P, Wang X, Tran DT, Thanh NX, Glaze S, Chu P, Ghatage P, Nation J, Capstick V, Steed H, Sabourin J, Nelson G. Enhanced Recovery After Surgery (ERAS) in gynecologic oncology: System-wide implementation and audit leads to improved value and patient outcomes. Gynecol Oncol. 2018 Oct;151(1):117-123.

7.2 ΕΙΔΙΚΑ (ΚΑΤΑ ΕΙΔΙΚΟΤΗΤΕΣ)

7.2.1 ΧΕΙΡΟΥΡΓΙΚΗ ΟΙΣΟΦΑΓΟΥ

1. Παροχετεύσεις

Τραχηλικές παροχετεύσεις

Η χρήση τραχηλικής παροχέτευσης μετά από οισοφαγεκτομή δεν έχει αποδειχθεί ότι μειώνει τις τοπικές επιπλοκές στο τραύμα, όπως το αιμάτωμα ή η συλλογή ορώδους υγρού (seroma)[1]. Επιπλέον, δεν υπάρχουν στοιχεία που να υποδηλώνουν ότι η χρήση της μειώνει τον κίνδυνο διάσπασης της αναστόμωσης[1]. Ως εκ τούτου, δεν συνιστάται η συστηματική χρήση τους καθώς δεν παρέχει σημαντικά οφέλη.

1. Οι τραχηλικές παροχετεύσεις μετά από οισοφαγεκτομή δεν έχουν αποδεδειγμένα πλεονεκτήματα σε σχέση με τη μη χρήση τους, επομένως δεν συνιστάται η συστηματική χρήση τους.

Μέτριο επίπεδο τεκμηρίωσης. Ισχυρή σύσταση.

ΒΙΒΛΙΟΓΡΑΦΙΚΕΣ ΑΝΑΦΟΡΕΣ

1. Choi HK, Law S, Chu KM, Wong J. The value of neck drain in esophageal surgery: a randomized trial. Dis Esophagus. 2017:11:40-2.

Θωρακικές παροχετεύσεις

Τα επί του παρόντος διαθέσιμα στοιχεία που να αποδεικνύουν το όφελος από τη χρήση θωρακικής παροχέτευσης μετά από οισοφαγεκτομή είναι εξαιρετικά περιορισμένα και δεν μπορεί να διαπιστωθεί σταθερή σύνδεση[1]. Αυτό συμβαίνει παρά το γεγονός ότι οι περισσότερες από τις δημοσιευμένες κατευθυντήριες οδηγίες και τα κλινικά πρωτόκολλα τις περιλαμβάνουν στις συστάσεις τους, καθώς μπορούν να αποτρέψουν τη συμπίεση των πνευμόνων και να χρησιμοποιηθούν ως οδηγός για την παρακολούθηση της παρουσίας αιμορραγίας ή/και διαφυγών (αέρα, λέμφου ή αναστομωτική). Ωστόσο, η χρήση

τους προκαλεί περισσότερο πόνο που οδηγεί σε χειρότερο αερισμό και κινητοποίηση[2].

2. Συνιστάται η χρήση θωρακικών παροχετεύσεων μετά από οισοφαγεκτομή, αν και καλό είναι να μειωθεί ο αριθμός των παροχετεύσεων και ο χρόνος που παραμένουν (μία μόνο παροχέτευση μπορεί να είναι αρκετή), εάν δεν υπάρχει αναστομωτική διαφυγή, διαφυγή αέρα ή λέμφου (χυλοθώρακας).

Χαμηλό επίπεδο τεκμηρίωσης. Ισχυρή σύσταση.

ΒΙΒΛΙΟΓΡΑΦΙΚΕΣ ΑΝΑΦΟΡΕΣ

1. Low DE, Allum W, De Manzoni G, Ferri L, Immanuel A, Kuppusamy M, et al. Guidelines for Perioperative Care in Esophagectomy: Enhanced Recovery After Surgery (ERAS®) Society Recommendations. World J Surg. 2019; 43(2):299-330.
2. De Pasqual CA, Weindelmayer J, Laiti S, La Mendola R, Bencivenga M, Alberti L, et al. Perianastomotic drainage in Ivor-Lewis esophagectomy, does habit

Τα στοιχεία που προέκυψαν από πρόεκταση και γενίκευση δεδομένων (extrapolated data) από τις πνευμονεκτομές υποστηρίζουν τη χρήση μίας παροχέτευσης θώρακα[1,2], με την ίδια νοσηρότητα, αλλά σημαντική μείωση του μετεγχειρητικού πόνου, του κόστους και της νοσηλείας στο νοσοκομείο σε σύγκριση με την τοποθέτηση μεγαλύτερου αριθμού παροχετεύσεων[3,4]. Επιπλέον, φαίνεται ότι η χρήση παθητικών παροχετεύσεων είναι εξίσου αποτελεσματική με τις ενεργητικές[5].

3. Η τοποθέτηση μιας μονής παροχέτευσης θώρακα συνιστάται σε σχέση με τις πολλές καθώς φαίνεται εξίσου αποτελεσματική, αλλά φθηνότερη και λιγότερο επώδυνη.

Μέτριο επίπεδο τεκμηρίωσης (προεκτάση & γενίκευση δεδομένων). Ισχυρή σύσταση.

4. Συνιστάται η χρήση παθητικής παροχέτευσης (χωρίς αναρρόφηση ή συνεχή αναρρόφηση), καθώς είναι εξίσου αποτελεσματική με μια ενεργητική.

Χαμηλό επίπεδο τεκμηρίωσης. Ισχυρή σύσταση.

ΒΙΒΛΙΟΓΡΑΦΙΚΕΣ ΑΝΑΦΟΡΕΣ

1. Gomez Caro A, Roca MJ, Torres J, Cascales P, Terol E, Castañer J, et al. Successful use of a single chest drain post lobectomy instead of two classical drains: a randomized study. Eur J Cardiothoracic Surg. 2006;29:562-6.

2. Pawelczyk K, Marciniak M, Kacprzak G, Kolodziej J. One or two drains after lobectomy ¿A comparison of both methods in the inmediate postoperative period. Thorac Cardiovasc Surg. 2007;55:313-6.

3. Alex J, Ansari J, Bhalkar P, Agarwala S, Rehman M, Saleh A, et al. Comparison of the inmediate postoperative outcome of using the conventional two drains versus a single drain after lobectomy. Ann Thorac Surg. 2003;76:1046-9.

4. Refai M, Brunelli A, Salati M, Xiumè F, Pompili C, Sabbatini A, et al. The impact of chest tube removal on pain and pulmonary function after pulmonary resection. Eur J Cardiothorac Surg. 2012;41:820-2.

5. Johansson J, Lindberg CG, Johnsson F, von Holstein CS, Zilling T, Walther B. Active or passive chest drainage after esophagectomy in 101 patients: a prospective randomized study. Br J Surg. 1998;85:1143-6.

Υπάρχουν τυχαιοποιημένες ελεγχόμενες δοκιμές που δείχνουν ότι η πρώιμη αφαίρεση της θωρακικής παροχέτευσης, ακόμη και με παροχή 200–300 ml/24ωρο χωρίς διαφυγές (αέρα, αναστομωτική ή λέμφου), είναι ασφαλής και θα μπορούσε να βελτιώσει την μετεγχειρητική άνεση και να μειώσει τη νοσηλεία στο νοσοκομείο[1-4].

5. Η αφαίρεση της θωρακικής παροχέτευσης συνιστάται εάν η παροχή είναι μικρότερη ή ίση με 200–300 ml/24ωρο και δεν υπάρχει διαφυγή εντερικού περιεχομένου ή αέρα ή λέμφου.

Μέτριο επίπεδο τεκμηρίωσης. Ισχυρή σύσταση.

ΒΙΒΛΙΟΓΡΑΦΙΚΕΣ ΑΝΑΦΟΡΕΣ

1. Yao F, Wang J, Yao J, Hang F, Cao S, Qian J, et al. Early chest tube removal after thoracoscopic esophagectomy with high output. J Laparoendosc Adv Surg Tech A. 2016;26:17-22.

2. Novoa NM, Jiménez MF, Varela G. When to remove a chest tube. Thorac Surg Clin. 2017;27:41-6.

3. Hessami MA, Najafi F, Hatami S. Volume threshold for chest tube removal: a randomized controlled trial. J Inj Violence Res. 2009;1:33-6.

4. Findlay JM, Gillies RS, Millo J, Sgromo B, Marshall RE, Maynard ND. Enhanced recovery for esophagectomy: a systematic review and evidence - based guidelines. Ann Surg. 2014; 259:413-31.

Κοιλιακές παροχετεύσεις

Η χρήση κοιλιακών παροχετεύσεων μετά από γαστρεκτομή δεν προσφέρει οφέλη σε σύγκριση με τη μη χρήση τους[1-3].

6. Μετά την οισοφαγεκτομή, η συστηματική χρήση κοιλιακών παροχετεύσεων δεν συνιστάται.

Υψηλό επίπεδο τεκμηρίωσης. Ισχυρή σύσταση.

ΒΙΒΛΙΟΓΡΑΦΙΚΕΣ ΑΝΑΦΟΡΕΣ

1. Wang Z, Chen J, Su K, Dong Z. Abdominal drainage versus no drainage post-gastrectomy for gastric cancer (Review). Cochrane Database Syst Rev. 2015;5:CD008788.

2. Álvarez R, Molina H, Torres, Cancino A. Total gastrectomy with or without abdominal drains. A prospective randomized trial. Rev Esp Enferm Dig. 2005;97:562-9.

3. Kim J, Lee J, Hyung WJ, Cheong JH, Chen J, Choi SH, et al. Gastric cancer surgery without drains: a prospective randomized trial. J Gastrointest Surg. 2004;8:727-32.

2. Οδός χορήγησης και μετεγχειρητική έναρξη διατροφής στην οισοφαγεκτομή.

ΕΝΤΕΡΙΚΗ ΔΙΑΤΡΟΦΗ ΚΑΙ ΧΡΗΣΗ ΚΑΘΕΤΗΡΩΝ ΣΙΤΙΣΗΣ

Η από του στόματος και η εντερική οδοί σίτισης θα πρέπει να επιλέγονται έναντι της παρεντερικής διατροφής σε ασθενείς με νεοπλάσματα του οισοφάγου που χρειάζονται συμπληρώματα διατροφής καθώς είναι οι πιο φυσιολογικές οδοί και σχετίζονται με καλύτερη διατροφική πρόσληψη και λιγότερες επιπλοκές[1,2]. Παρά τη σύσταση για τοποθέτηση καθετήρα νηστιδοστομίας ή ρινονηστιδικού ή ρινοδωδεκαδακτυλικού σωλήνα για παροχή διατροφικής υποστήριξης σε ασθενείς με περιορισμένη από του στόματος λήψη τροφής, αυτού του είδους οι τεχνικές δεν εξαιρούνται από νοσηρότητα και θνησιμότητα και σημαντικά ποσοστά επανατοποθέτησης[3]. Επί του παρόντος, δεν υπάρχουν στοιχεία υπέρ οποιουδήποτε συγκεκριμένου τύπου καθετήρα που θα χρησιμοποιείται για τη χορήγηση επαρκών διατροφικών σκευασμάτων σε αυτόν τον τύπο των ασθενών[4,5].

Η τοποθέτηση νηστιδοστομίας σχετίζεται με ποσοστό θνησιμότητας 0-0,5% και ποσοστό επανεπέμβασης 0-2,9%. Ωστόσο, οι μικρές επιπλοκές είναι πιο συχνές, όπως μόλυνση στο σημείο εισόδου στο δέρμα (0,4–16%), διαρροές (1,4–25%) και γαστρεντερικές ενοχλήσεις (10–39%). Η χρήση του ρινονηστιδικού σωλήνα συνεπάγεται λιγότερες επιπλοκές αλλά συνοδεύεται από μεγαλύτερη ενόχληση και ποσοστό ατυχηματικής αφαίρεσης που κυμαίνεται μεταξύ 20% και 35%.

7. Στην προεγχειρητική αντιμετώπιση ασθενών με δυσφαγία ή αφαγία που υποβάλλονται σε οισοφαγεκτομή, η χρήση εντερικής διατροφής μέσω καθετήρα σίτισης συνιστάται σε εκείνες τις περιπτώσεις με υψηλό κίνδυνο υποθρεψίας και αδυναμία επίτευξης επαρκούς λήψης τροφής από του στόματος για την κάλυψη των διατροφικών αναγκών .

Μέτριο επίπεδο τεκμηρίωσης. Ισχυρή σύσταση.

8. Μετά από οισοφαγεκτομή, συνιστάται η κάλυψη των διατροφικών αναγκών με την από του στόματος ή/και εντερική οδό σίτισης μεταξύ της τρίτης και της έκτης μετεγχειρητικής ημέρας. Η χρήση καθετήρων εντερικής διατροφής θα

πρέπει να γίνεται επιλεκτικά σε ασθενείς που διατρέχουν κίνδυνο ή με διατροφικές απαιτήσεις που δεν μπορούν να καλυφθούν με την από του στόματος λήψη τροφής.

Μέτριο επίπεδο τεκμηρίωσης. Ισχυρή σύσταση.

9. Εάν είναι απαραίτητο, η νηστιδοστομία, οι ρινονηστιδικοί ή οι ρινοδωδεκαδακτυλικοί καθετήρες μπορούν να χρησιμοποιηθούν με την ίδια αποτελεσματικότητα καθώς τα τρέχοντα στοιχεία δεν επιτρέπουν τη σύσταση συγκεκριμένης οδού χορήγησης εντερικής διατροφής.

Μέτριο επίπεδο τεκμηρίωσης. Ισχυρή σύσταση.

ΒΙΒΛΙΟΓΡΑΦΙΚΕΣ ΑΝΑΦΟΡΕΣ

1. Liu K, Ji S, Xu Y, Diao Q, Shao C, Luo J, et al. Safety, feasibility, and effect of an enhanced nutritional support pathway including extended preoperative and home enteral nutrition in patients undergoing enhanced recovery after esophagectomy: a pilot randomized clinical trial. Dis Esophagus. 2020;33:doz030.

2. Chen MJ, Wu IC, Chen YJ, Wang TE, Chang YF, Yang CL, et al. Nutrition therapy in esophageal cancer – Consensus statement of the Gastroenterological Society of Taiwan. Dis Esophagus. 2018;31.

3. Álvarez-Sarrado E, Mingol Navarro F, Rosellón R, Ballester Pla N, Vaqué Urbaneja FJ, Muniesa Gallardo C, et al. Feeding jejunostomy after esophagectomy cannot be routinely recommended. Analysis of nutritional benefits and catheter-related complications. J Thorac Dis. 2019;11:S812-S818.

4. Lorimer PD, Motz BM, Watson M, Truffan SJ, Prabhu RS, Hill JS, etal. Enteralfeedingaccess has an impact on outcomes for patients with esophageal cancer undergoing esophagectomy: an analysis of SEER-Medicare. Ann Surg Oncol. 2019;26:1311-9.

5. Weijs TJ, Berkelmans GH, Nieuwenhuijzen GA, Ruurda JP, van Hillegersberg R, Soeters PB, et al. Routes for early enteral nutrition after esophagectomy: a systematic review. Clin Nutr. 2015;34:1-6.

6. Zheng T, Zhang Y, Zhu S, Ni Z, You Q, Sun X, et al. A prospective randomized trial comparing jejunostomy and nasogastric feeding in minimally invasive McKeown esophagectomy. J Gastrointest Surg. 2020;24(10):2187-96.

Πρώιμη μετεγχειρητική από του στόματος / εντερική διατροφή

Η πρώιμη έναρξη της εντερικής σίτισης κατά τη μετεγχειρητική περίοδο της οισοφαγεκτομής έχει αποδειχθεί ότι είναι ασφαλής[1,2], ευνοώντας τη λειτουργική εντερική αποκατάσταση και μειώνοντας τη νοσηλεία στο νοσοκομείο[3,4].

10. Η πρώιμη έναρξη (το πρώτο 24ωρο) της εντερικής διατροφής μετά την οισοφαγεκτομή συνιστάται καθώς είναι ασφαλής και διευκολύνει τη μετεγχειρητική ανάρρωση.

Μέτριο επίπεδο τεκμηρίωσης. Ισχυρή σύσταση.

ΒΙΒΛΙΟΓΡΑΦΙΚΕΣ ΑΝΑΦΟΡΕΣ

1. Low DE, Allum W, De Manzoni G, Ferri L, Immanuel A, Kuppusamy M, et al. Guidelines for Perioperative Care in Esophagectomy: Enhanced Recovery After Surgery (ERAS®) Society Recommendations. World J Surg. 2019; 43(2):299-330.
2. Giancopuzzi S, Weindelmayer J, Treppiedi E, Bencivenga M, Ceola M, Priolo S, et al. Enhanced recovery after surgery protocol in patients undergoing esophagectomy for cancer: a single center experience. Dis Esophag. 2017;30:1-6.
3. Tomaszek SC; Cassivi SD, Allen MS, Shen KR, Nichols FC 3rd, Deschamps C, et al. An alternative postoperative pathway reduces length of hospitalisation following oesophagectomy. Eur J Cardiothor Surg. 2010;37:807-13.
4. Kingma BF, Steenhagen E, Ruurda JP, van Hillegersberg R. Nutritional aspects of enhanced recovery after esophagectomy with gastric conduit reconstruction. J Surg Oncol. 2017;116: 623-9.

Η πρώιμη έναρξη της από του στόματος σίτισης μετά από οισοφαγεκτομή φαίνεται ασφαλής και εφικτή. Ορισμένες τυχαιοποιημένες ελεγχόμενες μελέτες δείχνουν ότι μια πρώιμη από του στόματος δίαιτα (τις πρώτες 24 ώρες) μετά την οισοφαγεκτομή δεν αυξάνει το ποσοστό των μετεγχειρητικών επιπλοκών[1-5]. Ωστόσο, στην τρέχουσα βιβλιογραφία υπάρχει διαμάχη σχετικά με την αποτελεσματικότητα και την ασφάλεια της χρήσης της σε σύγκριση με μεταγενέστερη έναρξη, σε σχέση με το ποσοστό διάσπασης της αναστόμωσης.

Άλλες οδοί εντερικής σίτισης θα μπορούσαν να ληφθούν υπόψιν και να χρησιμοποιηθούν σε συνδυασμό με την από του στόματος οδό για να εξασφαλιστεί η σωστή διατροφική υποστήριξη κατά τη μετεγχειρητική περίοδο της οισοφαγεκτομής.

11. Η καταλληλότερη οδός για τη χορήγηση εντερικής σίτισης κατά την πρώιμη μετεγχειρητική περίοδο της οισοφαγεκτομής δεν είναι σαφώς καθορισμένη. Από αυτή την άποψη, η έγκαιρη έναρξη της ανοχής στην λήψη τροφής από το στόμα φαίνεται να είναι αποτελεσματική και ασφαλής χωρίς να αυξάνει τον αριθμό των μεγάλων μετεγχειρητικών επιπλοκών.

Χαμηλό επίπεδο τεκμηρίωσης. Ασθενής σύσταση.

ΒΙΒΛΙΟΓΡΑΦΙΚΕΣ ΑΝΑΦΟΡΕΣ

1. Weijs TJ, Berkelmans GHK, Nieuwenhuijzen GAP, Dolmans AC, Kouwenhoven EA, Rosman C, et al. Inmediate postoperative oral nutrition following esophagectomy: a multicenter clinical trial. Ann Thorac Surg. 2016;102:1141-8.

2. Berkelmans GHK, Fransen L, Dolmans-Zwartjes AC, Kouwenhoven EA, van Det MJ, Nilsson M, et al. Direct oral feeding following minimally invasive esophagectomy (Nutrient II trial): an international, multicenter, open-label randomized controlled trial. Ann Surg. 2020;271: 41-7.

3. Sun HB, LI Y, Liu XB, Zhang RX, Wang ZF, Lerut T, et al. Early oral feeding following Mc-Keown minimally invasive esophagectomy. An open-label, randomized, controlled, non inferiority trial. Ann Surg. 2018; 267:435-42.

4. Bolton JS, Conway WC, Abbas AE. Planned delay of oral intake after esophagectomy reduces the cervical anastomotic leak rate and hospital length of stay. J Gastrointest Surg. 2014; 18:304-9.
5. Eberhard KE, Achiam MP, Rolff HC, Belmouhand M, Svendsen LB, Thorsteinsson M. Comparison of “nil by mouth” versus early oral intake in three different diet regimens following esophagectomy. World J Surg. 2017;41:1575-83.

3. Χρήση ρινογαστρικού σωλήνα για αποσυμφόρηση

Παραδοσιακά, η χρήση ρινογαστρικού σωλήνα θεωρείτο υποχρεωτική μετά την οισοφαγεκτομή για την αποσυμπίεση της πυλωροπλαστικής, την αποφυγή της διαστολής της, τη μείωση της αναστομωτικής τάσης και την αποφυγή εμέτου, πόνου και πιθανών εισροφήσεων.

Ωστόσο, υπάρχουν αντικρουόμενα δεδομένα στη βιβλιογραφία σχετικά με τη χρήση του και τον κίνδυνο αναστομωτικών και αναπνευστικών επιπλοκών. Ως εκ τούτου, ορισμένες μελέτες δεν συνιστούν τη χρήση του σε τακτική βάση, καθώς δεν έχουν αποδειχθεί σαφή οφέλη όσον αφορά τη μείωση των επιπλοκών και επιπλέον προκαλεί καθυστέρηση στην έναρξη της από του στόματος ανοχής στην λήψη τροφής και επιμήκυνση της νοσηλείας στο νοσοκομείο[1,2]. Μια πρόσφατη μετα-ανάλυση κατέληξε στο συμπέρασμα ότι η άμεση ή πρώιμη αφαίρεση του ρινογαστρικού σωλήνα δεν αυξάνει τον αριθμό των διασπάσεων της αναστόμωσης, τις πνευμονικές επιπλοκές ή τη μετεγχειρητική θνησιμότητα, μειώνοντας έτσι τη νοσηλεία στο νοσοκομείο[3]. Η πρώιμη αφαίρεση μετά την τοποθέτηση φαίνεται ασφαλής και βελτιώνει την άνεση του ασθενούς, επιταχύνοντας την ανοχή στην λήψη τροφής από το στόμα[4,5].

12. Παρόλο που τα στοιχεία που βασίζονται σε πιο πρόσφατες μελέτες αμφισβητούν τη χρήση του με συστηματικό τρόπο, η χρήση ενός ρινογαστρικού σωλήνα συνιστάται επί του παρόντος μετά την οισοφαγεκτομή.

Χαμηλό επίπεδο τεκμηρίωσης. Ασθενής σύσταση.

13. Εάν τοποθετηθεί, θα πρέπει να ληφθεί υπόψη η έγκαιρη αφαίρεσή του τις πρώτες 48 μετεγχειρητικές ώρες, γεγονός που μειώνει τον μετεγχειρητικό χρόνο νηστείας και τη νοσηλεία στο νοσοκομείο και βελτιώνει την άνεση του ασθενούς.

Μέτριο επίπεδο τεκμηρίωσης. Ισχυρή σύσταση.

ΒΙΒΛΙΟΓΡΑΦΙΚΕΣ ΑΝΑΦΟΡΕΣ

1. Low DE, Allum W, De Manzoni G, Ferri L, Immanuel A, Kuppusamy M, et al. Guidelines for Perioperative Care in Esophagectomy: Enhanced Recovery After Surgery (ERAS®) Society Recommendations. World J Surg. 2019; 43(2):299-330.

2. Menéndez-Jiménez M, Bruna-Esteban M, Mingol M, Vaqué J, Hervás D, Álvarez-Sarrado E, et al. Uso de sonda nasogástrica en pacientes sometidos a esofaguectomía: ¿Un gesto innecesario? Cir Esp. 2020;S0009-739X(20)30158-5.

3. Findlay JM, Gillies RS, Millo J, Sgromo B, Marshall RE, Maynard ND. Enhanced recovery for esophagectomy: a systematic review and evidence - based guidelines. Ann Surg. 2014; 259:413-31.

4. Hayashi M, Kawakubo H, Shoji Y, Mayanagi S, Nakamura R, Suda K, et al. Analysis of the effect of early versus conventional nasogastric tube removal on postoperative complications after transthoracic esophagectomy: a single-center, randomized controlled trial. World J Surg. 2019;43:580-9.

5. Mistry RC, Vijayabhaskar R, Karimundackal G, Jiwnani S, Pramesh CS. Effect of short-term vs prolonged nasogastric decompression on major postesophagectomy complications. Arch Surg. 2012;147:747-51.

4. Εισαγωγή σε Μονάδα Εντατικής Θεραπείας ή Αναζωογόνησης

Η συμβατική μετεγχειρητική αντιμετώπιση μετά από οισοφαγεκτομή περιελάμβανε εισαγωγή συστηματικά σε μονάδα αναζωογόνησης ή Μονάδα Εντατικής Θεραπείας (ΜΕΘ). Με τη διαθεσιμότητα καλύτερου ελέγχου του πόνου, ελάχιστα επεμβατικών προσπελάσεων και έγκαιρης αποδιασωλήνωσης, μεταξύ άλλων μέτρων, είναι δυνατή η μετεγχειρητική διαχείριση ασθενών που υποβάλλονται σε οισοφαγεκτομή σε Μονάδες Ενδιάμεσης Φροντίδας (Intermediate Care Units)[1], χωρίς την ανάγκη

εισαγωγής σε ΜΕΘ, που έχει αποδειχθεί ότι μειώνει τη νοσηλεία στο νοσοκομείο, χωρίς διαφορές στη νοσηρότητα και τη θνησιμότητα ή στο ποσοστό επανειαγωγών[2,3].

14. Η μετεγχειρητική αντιμετώπιση των ασθενών που υποβάλλονται σε οισοφαγεκτομή θα πρέπει να εξατομικεύεται και δεν απαιτεί συνήθως εισαγωγή στη Μονάδα Εντατικής Θεραπείας ή Αναζωογόνησης. Η διαθεσιμότητα μιας Μονάδας Ενδιάμεσης Φροντίδας είναι μια ασφαλής εναλλακτική λύση για ασθενείς χαμηλού κινδύνου.

Μέτριο επίπεδο τεκμηρίωσης. Ισχυρή σύσταση.

ΒΙΒΛΙΟΓΡΑΦΙΚΕΣ ΑΝΑΦΟΡΕΣ

1. Low DE, Allum W, De Manzoni G, Ferri L, Immanuel A , Kuppusamy M, et al. Guidelines for perioeprative care in esophagectomy: Enhanced Recovery After Surgery (ERAS) Society recommendations. World J Surg. 2019;43:299-330.

2. Pisarska M, Małczak P, Major P, Wysocki M, Budzyński A, Pędziwiatr M. Enhanced recovery after surgery protocol in oesophageal cancer surgery: Systematic review and meta-analysis. PLoS One. 2017;12:e0174382.

3. Chen L, Sun L, Lang Y, Wu J, Yao L, Ning J, et al. Fast-track surgery improves postoperative clinical recovery and cellular and humoral immunity after esophagectomy for esophageal cancer. BMC Cancer. 2016;16:449.

35. Wunsch H, Gershengorn HB, Cooke CR, Guerra C, Angus DC, Rowe JW, et al. Use of intensive care services for medicare beneficiaries undergoing major surgical procedures. Anesthesiology. 2016;124:899-907.

7.2.2 ΧΕΙΡΟΥΡΓΙΚΗ ΚΑΡΔΙΑΣ ΚΑΙ ΑΓΓΕΙΩΝ

Προεγχειρητική μέτρηση αιμοσφαιρίνης a1c

Η υπεργλυκαιμία σε νοσηλευόμενους χειρουργικούς ασθενείς σχετίζεται με αυξημένη νοσηρότητα και θνησιμότητα, επομένως πρέπει να αποφεύγεται[1].

Τα αυξημένα επίπεδα αιμοσφαιρίνης A1c (HbA1c – γλυκοζυλιωμένη αιμοσφαιρίνη) συσχετίζονται με κακή ρύθμιση του σακχάρου επομένως, αυξάνουν τον κίνδυνο να εμφανίσει ο ασθενής υπεργλυκαιμία κατά την εισαγωγή. .

Τα βέλτιστα επίπεδα της HbA1c[2] ορίστηκαν ως <7% σε ένα έγγραφο συναίνεσης (consensus) της Αμερικανικής και της Ευρωπαϊκής Ένωσης Διαβήτη (American Diabetes Association - ADA και European Association for the Study of Diabetes - EASD). Έχει αποδειχθεί ότι όσο υψηλότερο είναι το επίπεδο HbA1c, τόσο υψηλότερο η συχνότητα λοίμωξης του εν τω βάθει τραύματος του στέρνου, των ισχαιμικών προβλημάτων και άλλων επιπλοκών[3,4]. Επίσης, επιβεβαιωμένα μη διαβητικοί ασθενείς με αυξημένα επίπεδα HbA1c έχει αποδειχθεί ότι έχουν υψηλότερο κίνδυνο μετεγχειρητικής θνησιμότητας[5].

Ως εκ τούτου, συνιστάται η παρακολούθηση των επιπέδων HbA1c σε όλους τους ασθενείς που υποβάλλονται σε καρδιοχειρουργική επέμβαση.

Εκτός από την επείγουσα χειρουργική επέμβαση, με τιμές > 9% (που υποδεικνύει υποτροπιάζοντα επεισόδια σοβαρής υπεργλυκαιμίας) ή <5% (που υποδεικνύει υποτροπιάζοντα επεισόδια σοβαρής υπογλυκαιμίας), η επέμβαση θα πρέπει να καθυστερήσει[6].

1. Συνιστάται προεγχειρητικός έλεγχος των επιπέδων HbA1c σε όλους τους ασθενείς που υποβάλλονται σε καρδιοχειρουργική επέμβαση για τη διαστρωμάτωση του χειρουργικού κινδύνου.

Μέτριο επίπεδο τεκμηρίωσης. Ισχυρή σύσταση

2. Όταν ο προεγχειρητικός προσδιορισμός της HbA1c είναι <5% ή > 9%, προτείνεται η αναβολή της επέμβασης, εκτός εάν χρειάζεται επείγουσα

χειρουργική επέμβαση, μέχρι να επιτευχθεί επαρκής γλυκαιμικός έλεγχος του σακχαρώδη διαβήτη.

Χαμηλό επίπεδο τεκμηρίωσης. Ασθενής σύσταση.

ΒΙΒΛΙΟΓΡΑΦΙΚΕΣ ΑΝΑΦΟΡΕΣ

1. Palermo NE, Gianchandani RY, McDonnell ME, Alexanian SM. Stress Hyperglycemia During Surgery and Anesthesia: Pathogenesis and Clinical Implications. Curr Diab Rep. 2016; 16(3):33. doi:10.1007/s11892-016-0721-y.

2. Inzucchi SE, Bergenstal RM, Buse JB, Diamant M, Ferrannini E, Nauck M, etal. Management of hyperglycemia in type 2 diabetes: A patient-centered approach. Diabetes Care. 2012; 35(6):1364-79. doi:10.2337/dc12-0413.

3. Umpierrez G, Cardona S, Pasquel F, Jacobs S, Peng L, Unigwe M, et al. Randomized controlled trial of intensive versus conservative glucose control in patients undergoing coronary artery bypass graft surgery: GLUCOCABG trial. Diabetes Care. 2015;38(9):1665-72. doi: 10.2337/dc15-0303.

4. Narayan P, Naresh Kshirsagar, Mandal CK, Ghorai PA, Rao YM, Das D, et al. Preoperative Glycosylated Hemoglobin: A Risk Factor for Patients Undergoing Coronary Artery Bypass. Ann Thorac Surg. 2017;104(2):606-12. doi:10.1016/j.athoracsur.2016.12.020.

5. Hudson CCC, Welsby IJ, Phillips-Bute B, Mathew JP, Lutz A, Chad Hughes G, et al. Glycosylated hemoglobin levels and outcome in non-diabetic cardiac surgery patients. Can J Anesth. 2010;57(6):565-72. doi:10.1007/s12630-010-9294-4.

6. Cosson E, Catargi B, Cheisson G, Jacqueminet S, Ichai C, Leguerrier AM, et al. Practical management of diabetes patients before, during and after surgery: A joint French diabeto- logy and anaesthesiology position statement. Diabetes Metab. 2018;44(3):200-16. doi:10.1016/j.diabet.2018.01.014.

Ρινική εκρίζωση χρυσίζοντος σταφυλόκοκκου (staphylococcus aureus)

Ο χρυσίζων σταφυλόκοκκος είναι το μικρόβιο που ευθύνεται για το μεγαλύτερο μέρος των λοιμωδών επιπλοκών του χειρουργικού πεδίου και των προσθετικών υλικών μετά από καρδιοχειρουργική επέμβαση. Ποσοστό μεταξύ 18% και 30% των ασθενών που υποβάλλονται σε αυτόν τον τύπο χειρουργικής

επέμβασης έχουν ρινικό αποικισμό, που συνεπάγεται κίνδυνο έως και 3 φορές μεγαλύτερο για την εμφάνιση βακτηριαιμίας ή λοιμώξεις χειρουργικών τραυμάτων από S. aureus.

Είναι ευρέως διαπιστωμένο ότι πρέπει να πραγματοποιηθεί εκρίζωση του αποικισμού των φορέων[1,2]. Υπάρχει η δυνατότητα διαλογής τους με καλλιέργεια ή τεχνικές PCR και στη συνέχεια εφαρμογή επιλεκτικής θεραπείας ασθενών με θετικά αποτελέσματα[3], ωστόσο, τα τρέχοντα στοιχεία υποδεικνύουν καθολική εκρίζωση του αποικισμού για πρακτικούς, υλικοτεχνικούς λόγους ή λόγους κόστους-αποτελεσματικότητας, αν και προειδοποιούν ότι μπορεί να οδηγήσει σε εμφάνιση ανθεκτικότητας στα αντιβιοτικά[4,5].

Η θεραπεία εκρίζωσης του αποικισμού θα πρέπει να περιλαμβάνει μουπιροσίνη (mupirocin) ενδορινικά ως τοπικό αντιβιοτικό [6,7], αλλά πάντα μέσα σε ένα συνδυασμό δράσεων μαζί με εκπαίδευση υγιεινής και καθημερινών λουτρών με αντισηπτικά όπως η χλωρεξιδίνη. Πρέπει να γίνεται για 5 ημέρες πριν την επέμβαση.

3. Συνιστάται εκρίζωση του αποικισμού γνωστών ρινικών φορέων Staphylococcus aureus κατα τον προεγχειρητικό έλεγχο καρδιοχειρουργικής επέμβασης.

Υψηλό επίπεδο τεκμηρίωσης. Ισχυρή σύσταση.

4. Η καθολική εκρίζωση του αποικισμού συνιστάται για όλους τους ασθενείς που υποβάλλονται σε καρδιοχειρουργική επέμβαση.

Μέτριο επίπεδο τεκμηρίωσης. Ισχυρή σύσταση.

5. Η θεραπεία εκρίζωσης του αποικισμού συνιστάται να γίνεται με μουπιροσίνη ενδορινικά μαζί με έναν συνδυασμό μέτρων.

Υψηλό επίπεδο τεκμηρίωσης. Ισχυρή σύσταση

ΒΙΒΛΙΟΓΡΑΦΙΚΕΣ ΑΝΑΦΟΡΕΣ

1. Sousa-Uva* M, Head SJ, Milojevic M, Collet J-P, Landoni G, Castella M, et al. 2017 EACTS Guidelines on perioperative medication in adult cardiac surgery. Eur J CardioThoracic Surg. 2018;53(1):5-33.

2. Lepelletier D, Saliou P, Lefebvre A, Lucet JC, Grandbastien B, Bruyère F, et al. Preoperative risk management: Strategy for Staphylococcus aureus preoperative decolonization (2013 update). Med Mal Infect. 2014;44(6):261-7.
3. Kline SE, Neaton JD, Lynfield R, Ferrieri P, Kulasingam S, Dittes K, et al. Randomized controlled trial of a self-administered five-day antiseptic bundle versus usual disinfectant soap showers for preoperative eradication of Staphylococcus aureus colonization. Infect Control Hosp Epidemiol. 2018;39(9):1049-57.
4. Lazar HL, Salm T Vander, Engelman R, Orgill D, Gordon S. Prevention and management of sternal wound infections. J Thorac Cardiovasc Surg. 2016;152(4):962-72. doi:10.1016/j. jtcvs.2016.01.060
5. George S, Leasure AR, Horstmanshof D. Effectiveness of decolonization with chlorhexidine and mupirocin in reducing surgical site infections: A systematic review. Dimens Crit Care Nurs. 2016;35(4):204-22.
6. Sakr A, Brégeon F, Rolain JM, Blin O. Staphylococcus aureus nasal decolonization strategies: a review. Expert Rev Anti Infect Ther. 2019;17(5):327-40.
7. Septimus EJ. Nasal decolonization: What antimicrobials are most effective prior to surgery? Am JInfect Control. 2019;47:A53-7.

Αντιινωδολυτικά φάρμακα στην καρδιοχειρουργική

Η αιμορραγία κατά την περιεγχειρητική περίοδο της καρδιοχειρουργικής επέμβασης είναι μια συχνή επιπλοκή και η εμφάνισή της μπορεί να επιδεινώσει τα αποτελέσματα[1].

Τα αντιινωδολυτικά φάρμακα, όπως το τρανεξαμικό οξύ και το ε-αμινοκαπροϊκό οξύ, είναι ευρέως διαδεδομένα, με προφίλ χαμηλού κινδύνου, υψηλή απόδοση και εύκολη χορήγηση[2-4], έτσι χρησιμοποιούνται ευρέως στην καθημερινή πρακτική. Η χρήση τους συνιστάται από την αρχή της επέμβασης για πρόληψη υπερινωδόλυσης που εμφανίζεται κατά τη διάρκεια της επέμβασης. Τα ισχυρότερα δεδομένα προκύπτουν από χειρουργικές επεμβάσεις με εξωσωματική κυκλοφορία (ΕΣΚ).

Το τρανεξαμικό οξύ έχει αποδειχθεί ότι μειώνει την αιμορραγία, την ανάγκη για επανεπέμβαση και τις απαιτήσεις μετάγγισης[5,6].

Η δοσολογία αυτών των φαρμάκων δεν έχει ακόμη πλήρως τεκμηριωθεί. Υψηλές δόσεις, χωρίς να προσφέρουν όφελος, φαίνεται να σχετίζονται με επιληπτικές κρίσεις ως ανεπιθύμητη ενέργεια, επομένως η μέγιστη συνιστώμενη δόση είναι 100 mg/kg και απαιτεί προσαρμογή σε ασθενείς με νεφρική ανεπάρκεια[7].

6. Για να επιτευχθεί αντιινωδολυτικό αποτέλεσμα, συνιστάται η χρήση τρανεξαμικού οξέος ή έψιλον αμινοκαπροϊκού οξέος κατά τη διάρκεια καρδιοχειρουργικών επεμβάσεων με εξωσωματική κυκλοφορία.

Υψηλό επίπεδο τεκμηρίωσης. Ισχυρή σύσταση

7. Συνιστάται η χρήση τρανεξαμικού οξέος εφόσον σχετίζεται με μείωση της αιμορραγίας, της ανάγκης μετάγγισης και της ανάγκης για επανεπέμβαση.

Μέτριο επίπεδο τεκμηρίωσης. Ισχυρή σύσταση

ΒΙΒΛΙΟΓΡΑΦΙΚΕΣ ΑΝΑΦΟΡΕΣ

1. Dyke C, Aronson S, Dietrich W, Hofmann A, Karkouti K, Levi M, et al. Universal definition of perioperative bleeding in adult cardiac surgery. J Thorac Cardiovasc Surg. 2014 May;147 (5):1458-1463.e1. doi:10.1016/j.jtcvs.2013.10.070

2. Apfelbaum JL, Nuttall GA, Connis RT, Harrison CR, Miller RD, Nickinovich DG, et al. Practice guidelines for perioperative blood management: An updated report by the american society of anesthesiologists task force on perioperative blood management. Anesthesiology. 2015 Feb;122(2):241-75. doi:10.1097/ALN.0000000000000463

3. Henry DA, Carless PA, Moxey AJ, O'Connell D, Stokes BJ, McClelland B, et al. Anti-fibrinolytic use for minimising perioperative allogeneic blood transfusion. Henry DA, editor. Cochrane Database Syst Rev. 2007 Oct 17;4(CD001886). doi:10.1002/14651858. CD001886.pub2

4. Leff J, Rhee A, Nair S, Lazar D, Sathyanarayana S, Shore-Lesserson L. A randomized, double-blinded trial comparing the effectiveness of tranexamic acid and epsilon-aminocaproic acid in reducing bleeding and transfusion in cardiac surgery. Ann Card Anaesth. 2019;22(3):265. doi:10.4103/aca.ACA_137_18

5. Myles PS, Smith JA, Forbes A, Silbert B, Jayarajah M, Painter T, et al. Tranexamic acid in patients undergoing coronary-artery surgery. N Engl J Med. 2017;376(2):136-48. doi: 10.1056/NEJMoa1606424.
6. Taam J, Yang QJ, Pang KS, Karanicolas P, Choi S, Wasowicz M, et al. Current Evidence and Future Directions of Tranexamic Acid Use, Efficacy, and Dosing for Major Surgical Procedures. J Cardiothorac Vasc Anesth. 2020 Mar;34(3):782-90. doi:10.1053/j. jvca.2019.06.042.
7. Hunt BJ. The current place of tranexamic acid in the management of bleeding. Anaesthe- sia. 2015;70:e18-53. doi:10.1111/anae.12910.

Θωρακικές παροχετεύσεις

Μετά από καρδιοχειρουργική επέμβαση, είναι πάντα απαραίτητο να αφήνεται κάποιο είδος παροχετευτικού θωρακικού σωλήνα που επιτρέπει την παροχέτευση της αιμορραγίας που εμφανίζεται πάντα σε μικρότερη ή μεγαλύτερη ποσότητα, διότι διαφορετικά, η συσσώρευση θα μπορούσε να οδηγήσει σε αιμοθώρακα ή καρδιακό επιπωματισμό, οντότητες με επίπτωση από 2 έως 19%[1,2], και οι οποίες έχουν χειρότερη πρόγνωση.

Ωστόσο, οι παροχετεύσεις τείνουν να φράζουν στην κλινική πράξη, φτάνοντας μέχρι και σε ποσοστό 36% των περιπτώσεων με κάποιου βαθμού απόφραξη λόγω σχηματισμού θρόμβου στο εσωτερικό της παροχέτευσης[3].

8. Συνιστάται η διατήρηση της βατότητας των θωρακικών παροχετεύσεων για την αποφυγή μεγάλων επιπλοκών όπως καρδιακός επιπωματισμός ή αιμοθώρακας.

Μέτριο επίπεδο τεκμηρίωσης. Ισχυρή σύσταση.

ΒΙΒΛΙΟΓΡΑΦΙΚΕΣ ΑΝΑΦΟΡΕΣ

1. Balzer F, von Heymann C, Boyle EM, Wernecke KD, Grubitzsch H, Sander M. Impact of retained blood requiring reintervention on outcomes after cardiac surgery. J Thorac Cardiovasc Surg. 2016;152(2):595-601.e4. doi:10.1016/j.jtcvs.2016.03.086.
2. Pompilio G, Filippini S, Agrifoglio M, Merati E, Lauri G, Salis S, et al. Determinants of pericardial drainage for cardiac tamponade following cardiac

surgery. Eur J Cardiothorac Surg. 2011;39(5):e107-13. doi:10.1016/j.ejcts.2010.12.021.
3. Karimov JH, Gillinov AM, Schenck L, Cook M, Kosty Sweeney D, Boyle EM, et al. Incidence of chest tube clogging after cardiac surgery: A single-centre prospective observational study. Eur JCardio-thoracic Surg. 2013;44(6):1029-36. doi:10.1093/ejcts/ezt140.

Για να εξασφαλιστεί η βατότητα των παροχετεύσεων, υπάρχουν διάφορες τεχνικές για τη διάσπαση των θρόμβων που διευκολύνουν την έξοδό τους, οι οποίες μπορούν να πραγματοποιηθούν επί κλίνης από το νοσηλευτικό προσωπικό, όπως η άμελξη (“milking”), η δημιουργία αρνητικής πίεσης με απόφραξη κοντά στον ασθενή και το άδειασμα της παροχετεύσης προς τον συλλέκτη (“απογύμνωση - stripping”) ή συμπίεση πολλών τμημάτων του σωλήνα διπλωμένα στον εαυτό τους ("δίπλωμα βεντάλιας - fan folding").

Κανένας από αυτούς τους τρόπους δεν έχει αποδειχθεί χρήσιμος σε πολλαπλές μελέτες και ανασκοπήσεις, και μάλιστα θα μπορούσαν ακόμη και να προκαλέσουν εσωτερική βλάβη λόγω της αύξησης της αρνητικής πίεσης[1-4], για αυτό θα έπρεπε να αποφεύγονται.

Κάποιοι αποσυνδέουν τις παροχετεύσεις και περνούν έναν καθετήρα μέσα για να τις καθαρίσουν[5], που παραβιάζει το αποστειρωμένο πεδίο ενώ και ο ίδιος ο εισαγόμενος καθετήρας μπορεί να προκαλέσει βλάβη στις εσωτερικές δομές, επομένως ούτε αυτή η μέθοδος πρέπει να εφαρμόζεται.

Επί του παρόντος, συνιστάται, σε περίπτωση χρήσης κλασικών ή αυλακωτών παροχετεύσεων, να αφήσετε την παροχέτευση σε οριζόντια θέση στο κρεβάτι του ασθενούς και μετά κάθετα στο σύστημα συλλογής, να μην κάνετε ελιγμούς που να παραβιάζουν το αποστειρωμένο πεδίο και μόνο εάν είναι απολύτως απαραίτητο, εκτελέστε ήπιες συμπιέσεις του σωλήνα («άμελξη» ή «δίπλωμα βεντάλιας») [2].

9. Δεν συνιστάται η εκτέλεση τεχνικών που να παραβιάζουν το αποστειρωμένο πεδίο των παροχετεύσεων, ή που μπορεί να προκαλέσουν αύξηση της αρνητικής ενδοθωρακικής πίεσης, τόσο επειδή δεν έχουν αποδεδειγμένη αποτελεσματικότητα όσο και λόγω των πιθανών επιπλοκών που μπορεί να προκαλέσουν.

Υψηλό επίπεδο τεκμηρίωσης. Ισχυρή σύσταση.

ΒΙΒΛΙΟΓΡΑΦΙΚΕΣ ΑΝΑΦΟΡΕΣ

1. Day TG, Perring RR, Gofton K. Is manipulation of mediastinal chest drains useful or harmful after cardiac surgery? Interact Cardiovasc Thorac Surg. 2008;7(5):888-90. doi:10.1510/ icvts.2008.185413

2. Halm MA. To Strip or Not to Strip? Physiological Effects of Chest Tube Manipulation. Am J Crit Care. 2007;16(6):609-12. doi:10.4037/ajcc2007.16.6.609.

3. Kirkwood P. Ask the Experts. CriticalCareNurse. 2002;22(4):70-2. doi:10.4037/ccn2002. 22.4.70.

4. Wallen M, Morrison A, Gillies D, O'Riordan E, Bridge C, Stoddart F. Mediastinal chest drain clearance for cardiac surgery. Cochrane database Syst Rev. 2002;2(CD003042). doi:10.1002/14651858.CD003042.pub2.

5. Boyacioğlu K, Kalender M, Özkaynak B, Mert B, Kayalar N, Erentuğ V. A new use of fogarty catheter: Chest tube clearance. Hear Lung Circ. 2014;23(10):e229-30. doi:10.1016/j. hlc.2014.04.255.

Υπάρχουν τρεις κύριοι τύποι παροχετεύσεων: συμβατικές, αυλακωτές ή τύπου Blake® και ενεργής κάθαρσης.

Μεταξύ των πρώτων 2 τύπων παροχετεύσεων, έχουν πραγματοποιηθεί μελέτες χωρίς διαφορές μεταξύ τους, επομένως οποιαδήποτε από αυτές μπορεί να έχει την ίδια αποτελεσματικότητα[1,2]. Τέλος, έχει δημιουργηθεί ένας τύπος παροχέτευσης ενεργής κάθαρσης. Μελέτες που αναλύουν αυτή τη νέα τεχνολογία παρέχουν ενθαρρυντικά αποτελέσματα, αν και όχι απόλυτα αδιαμφισβήτητα[3-6]. Ως εκ τούτου, η χρήση παροχετεύσεων ενεργής κάθαρσης μπορεί να εξεταστεί με σκοπό τη μείωση των επιπλοκών του επιπωματισμού ή του αιμοθώρακα λόγω της δημιουργίας θρόμβων στις παροχετεύσεις.

10. Η χρήση παροχετεύσεων ενεργής κάθαρσης προτείνεται για τη μείωση επιπλοκών όπως καρδιακός επιπωματισμός ή αιμοθώρακας.

Μέτριο επίπεδο τεκμηρίωσης. Ασθενής σύσταση.

ΒΙΒΛΙΟΓΡΑΦΙΚΕΣ ΑΝΑΦΟΡΕΣ

1. Bjessmo S, Hylander S, Vedin J, Mohlkert D, Ivert T. Comparison of three different chest drainages after coronary artery bypass surgery-a randomised trial in 150 patients. Eur J Cardiothorac Surg. 2007;31(3):372-5.

2. Sakopoulos AG, Hurwitz AS, Suda RW, Goodwin JN. Efficacy of Blake drains for mediastinal and pleural drainage following cardiac operations. J Card Surg. 2005;20(6):574-7.

3. Sirch J, Ledwon M, Puski T, Boyle EM, Pfeiffer S, Fischlein T. Active clearance of chest drainage catheters reduces retained blood. J Thorac Cardiovasc Surg. 2016 Mar;151(3):832- 838.e2.

4. St-Onge S, Ben Ali W, Bouhout I, Bouchard D, Lamarche Y, Perrault LP, et al. Examining the impact of active clearance of chest drainage catheters on postoperative atrial fibrillation. J Thorac Cardiovasc Surg. 2017;154(2):501-8.

5. Andersen ND. Active clearance technology to maintain chest tube patency: Practical, innovative, unproven. Vol. 151, The Journal of thoracic and cardiovascular surgery. United Sta- tes; 2016. p. 839-40.

6. Grieshaber P, Heim N, Herzberg M, Niemann B, Roth P, Boening A. Active Chest Tube Clearance After Cardiac Surgery Is Associated With Reduced Reexploration Rates. Ann Tho- rac Surg.2018;105(6):1771-7.

Βελτιστοποίηση της σύγκλισης του στέρνου

Η μέση στερνοτομή είναι η πιο κοινή προσπέλαση στην καρδιοχειρουργική. Η πιο συνηθισμένη και διαδεδομένη σύγκλιση είναι με χαλύβδινα σύρματα λόγω της ευκολίας χρήσης, της ταχύτητας, του χαμηλού κόστους και του σχετικά χαμηλού ποσοστού επιπλοκών. Η σύγκλιση του στέρνου γίνεται κατά προτίμηση με 6 απλά σύρματα, αλλά υπάρχουν κάποιες ενδείξεις ότι θα πρέπει να χρησιμοποιηθούν τουλάχιστον 8 σύρματα και εάν το στέρνο είναι οστεοπορωτικό, καλύτερα σταυρωτά χιαστή σύρματα[1-3].

11. Εάν σύγκλιση του στέρνου γίνεται με σύρματα, προτείνεται η χρήση τουλάχιστον 8 συρμάτων ή/και σταυρωτά χιαστή σύρματα.

Χαμηλό επίπεδο τεκμηρίωσης. Ασθενής σύσταση.

ΒΙΒΛΙΟΓΡΑΦΙΚΕΣ ΑΝΑΦΟΡΕΣ

1. Kamiya H, Al-maisary SSA, Akhyari P, Ruhparwar A, Kallenbach K, Lichtenberg A, et al. The number of wires for sternal closure has a significant influence on sternal complications in high-risk patients. Interact Cardiovasc Thorac Surg. 2012 Oct;15(4):665-70.

2. Mirhosseini SJ, Ali-Hassan-Sayegh S, Mostafavi-Pour-Manshadi SMY, Hadibarhaghtalab M, Lotfaliani MR. Figure-of-Eight Wire Sternal Closure Technique Can Reduce Post-Open Cardiovascular Surgery Chest Re-Exploration and Pain Scores in Diabetic Patients with Severe Obesity (Body Mass Index: 35-40). Int J Clin Exp Med Sci. 2015;1(3):38.

3. Ramzisham ARM, Raflis AR, Khairulasri MG, Ooi Su Min J, Fikri AM, Zamrin MD. Figure-of- eight vs. Interrupted sternal wire closure of median sternotomy. Asian Cardiovasc Thorac Ann. 2009;17(6):587-91.

Η χρήση παραστερνικών διαμήκων ενισχυτικών συρμάτων (Robicsek) δεν έδειξε σαφή πλεονεκτήματα σε ασθενείς υψηλού κινδύνου[1,2].

Η φυσική επέκταση των αρχών στη σταθεροποίηση των οστών που έχουν αποκτηθεί από αλλού στο ανθρώπινο σώμα, οδήγησε στη χρήση άκαμπτων συστημάτων στερέωσης μέσω πλακών τιτανίου που βιδώνονται στο στέρνο ή/και στα πλευρά. Τα στοιχεία δείχνουν καλύτερη και ταχύτερη επούλωση του στέρνου, κάτι που συνεπάγεται μείωση του πόνου, επιτρέπει την πρώιμη ανάρρωση του ασθενούς και συντομεύει τη νοσηλεία στο νοσοκομείο[3-5]. Η χρήση τους ενδείκνυται ιδιαίτερα σε ασθενείς υψηλού κινδύνου, γιατί μειώνει τις επιπλοκές και τη θνησιμότητα[6].

12. Η στερέωση του στέρνου με πλάκες τιτανίου επιτρέπει καλύτερη επούλωση του στέρνου, μείωση του μετεγχειρητικού πόνου και μικρότερη νοσηλεία στο νοσοκομείο. Η χρήση του προτείνεται σε ασθενείς υψηλού κινδύνου, στους οποίους μειώνονται επίσης η θνησιμότητα και οι επιπλοκές.

Μέτριο επίπεδο τεκμηρίωσης. Ασθενής σύσταση.

ΒΙΒΛΙΟΓΡΑΦΙΚΕΣ ΑΝΑΦΟΡΕΣ

1. Schimmer C, Reents W, Berneder S, Eigel P, Sezer O, Scheld H, et al. Prevention of Sternal Dehiscence and Infection in High-Risk Patients: A

Prospective Randomized Multicenter Trial. Ann Thorac Surg. 2008;86(6):1897-904.
2. Pinotti KF, Cataneo DC, Rodrigues OR, Cataneo AJM. Closure of the sternum with anchoring of the steel wires: Systematic review and meta-analysis. J Thorac Cardiovasc Surg. 2018;156(1):178-86.
3. Allen KB, Icke KJ, Thourani VH, Naka Y, Grubb KJ, Grehan J, et al. Sternotomy closure using rigid plate fixation: A paradigm shift from wire cerclage. Ann Cardiothorac Surg. 2018; 7(5):611- 20.
4. Raman J, Lehmann S, Zehr K, De Guzman BJ, Aklog L, Garrett HE, et al. Sternal closure with rigid plate fixation versus wire closure: A randomized controlled multicenter trial. Ann Tho- rac Surg. 2012;94(6):1854-61.
5. Peigh G, Kumar J, Unai S, James DT, Hirose H, Diehl JT, et al. Randomized Trial of Sternal Closure for Low Risk Patients: rigid Fixation versus Wire Closure. Heart Surg Forum. 2017; 20(4):E164-E169.
6. Tam DY, Nedadur R, Yu M, Yanagawa B, Fremes SE, Friedrich JO. Rigid Plate Fixation Versus Wire Cerclage for Sternotomy After Cardiac Surgery: A Meta-Analysis. Ann Thorac Surg. 2018 Jul;106(1):298-304.

Πρόληψη της υποθερμίας στην άμεση μετεγχειρητική περίοδο

Η γενική αναισθησία προκαλεί μεταβολή στη ρύθμιση της θερμοκρασίας του σώματος, προκαλώντας μείωση μεταξύ 1 και 2 °C κατά την πρώτη ώρα, και περαιτέρω πτώση κατά 3,5 ºC εάν δεν ληφθούν τα απαραίτητα μέτρα μετά τις τρεις ώρες αναισθησίας[1]. Επίσης, η έκθεση του δέρματος για παρατεταμένες περιόδους, η χορήγηση μεγάλων όγκων ενδοφλέβιων υγρών και οι πλύσεις του χειρουργικού πεδίου ευνοούν αυτή την κατάσταση.

Ως μετεγχειρητική υποθερμία ορίζουμε την αδυναμία διατήρησης της νορμοθερμίας (≥36 °C) μετά από 2 έως 5 ώρες εισαγωγής στην Μονάδα Εντατικης Θεραπείας[1,2].

Οι επιπλοκές που σχετίζονται με την περιεγχειρητική υποθερμία περιλαμβάνουν την εμφάνιση διαταραχών πήξης που σχετίζεται με μεγαλύτερες ανάγκες μετάγγισης, αύξηση του ποσοστού λοίμωξης του χειρουργικού τραύματος, καθυστερημένο μεταβολισμό φαρμάκων, βραδύτερη

και πιο παρατεταμένη ανάρρωση, ρίγη και θερμική δυσφορία, τα οποία όλα οδηγούν σε μεγαλύτερη νοσηλεία στο νοσοκομείο και αυξημένη θνησιμότητα[3,4].

Η πρόληψη της υποθερμίας πρέπει να πραγματοποιείται με τη χρήση κουβερτών ζεστού αέρα, τη χορήγηση θερμών ενδοφλεβίων υγρών στους 37 °C[5] και η αύξηση της θερμοκρασίας του δωματίου την άμεση μετεγχειρητική περίοδο[6-8]. Μόλις επιτευχθεί η νορμοθερμία, τα προηγούμενα μέτρα θα αποσυρθούν και η παρακολούθηση της θερμοκρασίας του σώματος θα συνεχιστεί.

13. Συνιστούμε τη χρήση κουβερτών με ζεστό αέρα, τη χορήγηση θερμών ενδοφλεβίων υγρών και την αύξηση της θερμοκρασίας δωματίου για την αποφυγή παρατεταμένης υποθερμίας (<36 °C) μετά από εξωσωματική κυκλοφορία και κατά την διάρκεια την άμεσης μετεγχειρητικής περιόδου.

Μέτριο επίπεδο τεκμηρίωσης. Ισχυρή σύσταση.

14. Συνιστούμε την παρακολούθηση της θερμοκρασίας του σώματος κατά την άφιξη του ασθενούς στη Μονάδα Εντατικής Θεραπείας.

Υψηλό επίπεδο τεκμηρίωσης. Ισχυρή σύσταση

ΒΙΒΛΙΟΓΡΑΦΙΚΕΣ ΑΝΑΦΟΡΕΣ

1. Sessler DI. Perioperative heat balance. Anesthesiology. 2000;92(2):578-90.
2. Sessler DI. Perioperative thermoregulation and heat balance. Lancet. 2016;387(10038): 2655-64.
3. Karalapillai D, Story D, Hart GK, Bailey M, Pilcher D, Cooper DJ, et al. Postoperative hypothermia and patient outcomes after elective cardiac surgery. Anaesthesia. 2011;66(9): 780-4.
4. Apfelbaum JL, Silverstein JH, Chung FF, Connis RT, Fillmore RB, Hunt SE, et al. Practice Guidelines for Postanesthetic Care. Anesthesiology. 2013;118(2):291-307.
5. NICE: National Institution for Health and Care Excelence.. Inadvertent perioperative hypothermia overview. NICE pathways. 2017;(February):1-12. Disponible en: http:// pathways.nice.org.uk/pathways/inadvertent-perioperative-hypothermia.

6. Campbell G, Alderson P, Smith AF, Warttig S. Warming of intravenous and irrigation fluids for preventing inadvertent perioperative hypothermia. Cochrane Database Syst Rev. 2015;4(CD009891). doi:10.1002/14651858.CD009891.pub2.
7. Evron S, Weissman A, Toivis V, Shahaf DB, You J, Sessler DI, et al. Evaluation of the temple touch pro, a novel noninvasive coreerature monitoring system. Anesth Analg. 2017;125(1): 103-9.
8. Macias AA, Valedon A, Durán H, Bayter-Marin JE, Rubio J, Cárdenas-Camarena L. Effects of Thermal Protection in Patients Undergoing Body Contouring Procedures: A Controlled Clinical Trial. Aesthetic Surg J. 2018;38(4):448-56.

Βιοδείκτες και στρατηγικές πρόληψης για την οξεία νεφρική βλάβη

Η οξεία νεφρική βλάβη (Acute Kidney Injury - AKI) που σχετίζεται με καρδιοχειρουργική επέμβαση είναι μια επιπλοκή που εμφανίζεται, ανάλογα με την επέμβαση και τον ορισμό της, στο 22-36% των ασθενών[1-4]. Η εμφάνισή της συνεπάγεται αύξηση της νοσηρότητας και της θνησιμότητας και σημαντικό αντίκτυπο στις συνολικές δαπάνες για την υγειονομική περίθαλψη[3].

Οι κλασικοί ορισμοί της νεφρικής βλάβης RIFLE (R - renal risk - νεφρικός κίνδυνος, I –injury - τραυματισμός, F – failure - ανεπάρκεια, L – loss of kidney function - απώλεια νεφρικής λειτουργίας, E – end stage renal disease - νεφρική νόσος τελικού σταδίου)[5], AKIN (Acute Kidney Injury Network - Δίκτυο οξείας νεφρικής βλάβης)[6] και KDIGO (Kidney Disease: Improving Global Outcomes – Νεφρική Νόσος: Βελτίωση παγκόσμιων αποτελεσμάτων)[7] χρησιμοποιούν την αυξημένη κρεατινίνη, η οποία μπορεί να καθυστερήσει την ανίχνευσή της μεταξύ 24 και 72 ωρών σε σύγκριση με νέους βιοδείκτες όπως ο ιστικός αναστολέας της μεταλλοπρωτεάσης-2 (tissue inhibitor metalloproteinase 2 - TIMP-2), η δεσμευτική πρωτεΐνη 7 του αυξητικού παράγοντα τύπου ινσουλίνης (insulin-like growth factor-binding protein 7 - IGFBP7) και η λιποκαλίνη που σχετίζεται με την ουδετεροφιλική ζελατινάση (Neutrophil gelatinase-associated lipocalin - NGAL)[8,9].

15. Προτείνουμε τον προσδιορισμό βιοδεικτών για την έγκαιρη αναγνώριση της οξείας νεφρικής βλάβης σε ασθενείς που διατρέχουν κίνδυνο για να καθοδηγήσει μια στρατηγική πρώιμης παρέμβασης με στόχο τη μείωση της οξείας νεφρικής βλάβης.

Μέτριο επίπεδο τεκμηρίωσης. Ασθενής σύσταση.

ΒΙΒΛΙΟΓΡΑΦΙΚΕΣ ΑΝΑΦΟΡΕΣ

1. Chen S-W, Chang C-H, Fan P-C, Chen Y-C, Chu P-H, Chen T-H, et al. Comparison of contemporary preoperative risk models at predicting acute kidney injury after isolated coronary artery bypass grafting: a retrospective cohort study. BMJ Open. 2016 Jun 27;6(6):e010176. doi:10.1136/bmjopen-2015-010176.

2. Lee CC, Chang CH, Chen SW, Fan PC, Chang SW, Chen YT, et al. Preoperative risk assessment improves biomarker detection for predicting acute kidney injury after cardiac surgery. PLoS One. 2018;13(9):1-13. doi:10.1371/journal.pone.0203447.

3. Hu J, Chen R, Liu S, Yu X, Zou J, Ding X. Global Incidence and Outcomes of Adult Patients with Acute Kidney Injury after Cardiac Surgery: A Systematic Review and Meta-Analysis. J Cardiothorac Vasc Anesth. 2016;30(1):82-9. doi:10.1053/j.jvca.2015.06.017.

4. Chang CH, Lee CC, Chen SW, Fan PC, Chen YC, Chang SW, et al. Predicting acute kidney injury following mitral valve repair. Int J Med Sci. 2016;13(1):19-24. doi:10.7150/ ijms.13253.

5. Van Biesen W, Vanholder R, Lameire N. Defining acuterenalfailure: RIFLE and beyond. Clin J Am Soc Nephrol. 2006;1(6):1314-9. doi:10.2215/CJN.02070606.

6. Mehta RL. From acute renal failure to acute kidney injury: Emerging concepts. Crit Care Med. 2008;36(5):1641-2. doi:10.1097/CCM.0b013e3181701481.

7. Levey AS, De Jong PE, Coresh J, Nahas M El, Astor BC, Matsushita K, et al. The definition, classification, and prognosis of chronic kidney disease: A KDIGO Controversies Conference report. Kidney Int. 2011;80(1):17-28. doi:10.1038/ki.2010.483.

8. Mayer T, Bolliger D, Scholz M, Reuthebuch O, Gregor M, Meier P, et al. Urine Biomarkers of Tubular Renal Cell Damage for the Prediction of Acute Kidney Injury After Cardiac Surgery — A Pilot Study. J Cardiothorac Vasc Anesth. 2017;31(6):2072-9. doi:10.1053/j. jvca.2017.04.024.
9. Vandenberghe W, De Loor J, Hoste EAJ. Diagnosis of cardiac surgery-associated acute kidney injury from functional to damage biomarkers. Curr Opin Anaesthesiol. 2016;30(1):1. doi:10.1097/ACO.0000000000000419.

Για τη μείωση της επίπτωσης της οξείας νεφρικής βλάβης καθ' όλη την περιεγχειρητική περίοδο, είναι απαραίτητο να αποφύγετε τη μείωση του ενδαγγειακού όγκου, να βελτιστοποιήστε την καρδιακή παροχή με αυστηρή διεγχειρητική παρακολούθηση, να αποφύγετε τη χορήγηση νεφροτοξικών φαρμάκων και να αποφύγετε τα επίπεδα γλυκόζης στο αίμα > 180 mg/dl και τις μεγάλες διακυμάνσεις μέσω ελέγχου και έγκαιρης χορήγησης ινσουλίνης[1]. Κατά τη διάρκεια της εξωσωματικής κυκλοφορίας, θα πρέπει να χρησιμοποιείται υπερδιήθηση με μηδενική ισορροπία σε ασθενείς με σπειραματική διήθηση <60 ml/min^2 και επιπλέον να διατηρηθεί η αρτηριακή παροχή οξυγόνου DO_2> 300 ml $O_2/min/m^2$ και να αποφευχθεί ή να μειωθεί η χορήγηση αγγειοσυσπαστικών εάν ο ασθενής έχει μέση αρτηριακή πίεση >70 mmHg[3].

Η μελέτη PREVaki[4] περιγράφει μια σειρά μέτρων (δέσμη χειρουργικής CT KDIGO) που αποτελείται από: αποφυγή νεφροτοξικών παραγόντων, διακοπή των αναστολέων του μετατρεπτικού ενζύμου της αγγειοτενσίνης (Angiotensin-converting enzyme - ACE)) και των Αναστολέων των Υποδοχέων Αγγειοτασίνης II (Angiotensin II Receptor Blockers - ARB) στις πρώτες 48 ώρες μετά την επέμβαση, στενή παρακολούθηση της κρεατινίνης και της παροχής ούρων, αποφυγή της υπεργλυκαιμίας (> 180 mg/dl) κατά τις πρώτες 72 ώρες μετά την επέμβαση, αναζήτηση εναλλακτικών λύσεων αντί του σκιαγραφικού στην ακτινοδιάγνωση, παρακολούθηση με καθετήρα PICCO® ή παρόμοιου για βελτιστοποίηση του ενδαγγειακού όγκου και των αιμοδυναμικών παραμέτρων σύμφωνα με συγκεκριμένο αλγόριθμο. Αυτός ο αλγόριθμος περιελάμβανε καρδιακό δείκτη >3 ml/min ως αιμοδυναμικό στόχο, κατά συνέπεια παρατηρήθηκε μεγαλύτερη χρήση ντοβουταμίνης (9% έναντι 31%) στην ομάδα παρέμβασης, με χαμηλότερο επιπολασμό της οξείας νεφρικής βλάβης. Η εφαρμογή αυτών των μέτρων επιτρέπει, επομένως, τη μείωση της συχνότητας

και της βαρυτήτας της οξείας νεφρικής βλάβης μετά από καρδιοχειρουργική επέμβαση[5].

Στην περίπτωση διάγνωσης της οξείας νεφρικής βλάβης σταδίου 2 κατά KDIGO (διούρηση <0,5 ml/kg/ώρα για περισσότερο από 12 ώρες ή κρεατινίνη διπλάσια της βασικής τιμής), η πρώιμη έναρξη (σε λιγότερο από 8 ώρες) της θεραπείας νεφρικής υποκατάστασης αποδείχθηκε ότι βελτιώνει τα αποτελέσματα[6].

16. Προτείνουμε την εφαρμογή του πακέτου μέτρων νεφρικής προστασίας του Πρωτόκολλου PREVaki για την πρόληψη της οξείας νεφρικής βλάβης που σχετίζεται με την καρδιοχειρουργική επεμβάση.

Μέτριο επίπεδο τεκμηρίωσης. Ισχυρή σύσταση.

ΒΙΒΛΙΟΓΡΑΦΙΚΕΣ ΑΝΑΦΟΡΕΣ

1. Bansal B, Carvalho P, Mehta Y, Yadav J, Sharma P, Mithal A, et al. Prognostic significance of glycemic variability after cardiac surgery. J Diabetes Complications. 2016;30(4):613-7. doi:10.1016/j.jdiacomp.2016.02.010.

2. Matata BM, Scawn N, Morgan M, Shirley S, Kemp I, Richards S, et al. A Single-Center Ran- domized Trial of Intraoperative Zero-Balanced Ultrafiltration during Cardiopulmonary Bypass for Patients with Impaired Kidney Function Undergoing Cardiac Surgery. J Cardiothorac Vasc Anesth. 2015;29(5):1236-47. doi:10.1053/j.jvca.2015.02.020.

3. Magruder JT, Crawford TC, Harness HL, Grimm JC, Suarez-Pierre A, Wierschke C, et al. A pilot goal-directed perfusion initiative is associated with less acute kidney injury after cardiac surgery. J Thorac Cardiovasc Surg. 2017;153(1):118-125.e1. doi:10.1016/j.jtcvs. 2016.09.016.

4. Meersch M, Schmidt C, Hoffmeier A, Van Aken H, Wempe C, Gerss J, et al. Prevention of cardiac surgery-associated AKI by implementing the KDIGO guidelines in high risk patients identified by biomarkers: the PrevAKI randomized controlled trial. Intensive Care Med. 2017;43(11):1551-61. doi:10.1007/s00134-016-4670-3.

5. Gregory AJ, Grant MC, Manning MW, Cheung AT, Ender J, Sander M, et al. Enhanced Re- covery After Cardiac Surgery (ERAS Cardiac) Recommendations: An Important First Step But There Is Much Work to Be

Done. J Cardiothorac Vasc Anesth. 2019;00:1-9. doi:10.1053/j.jvca.2019.09.002.
6. Zarbock A, Kellum JA, Schmidt C, Van Aken H, Wempe C, Pavenstädt H, et al. Effect of Early vs Delayed Initiation of Renal Replacement Therapy on Mortality in Critically Ill Patients With Acute Kidney Injury. JAMA. 2016 May 24;315(20):2190. doi:10.1001/jama.2016. 5828

Πρόληψη παραληρήματος και έγκαιρη διάγνωση

Το παραλήρημα είναι ένα κλινικό σύνδρομο που αποτελείται από μεταβολή της συνείδησης, που σχετίζεται με διάσπαση της προσοχής και της αντίληψης με αποδιοργανωμένη σκέψη, που μπορεί να οδηγήσει σε μια καθολική γενικευμένη γνωστική διαταραχή. Εμφανίζεται - ανάλογα με την ηλικιακή ομάδα - μεταξύ 11,5% και 80% των ασθενών που υποβάλλονται σε καρδιοχειρουργική επέμβαση[1-3].

Αυξάνει τις μετεγχειρητικές επιπλοκές, τη διάρκεια μηχανικού αερισμού, τη νοσηρότητα, τη θνησιμότητα, και έχει συσχετιστεί με μακροπρόθεσμες γνωστικές μεταβολές[4,5].

Είναι πολυπαραγοντικό ζήτημα (πόνος, υποξαιμία, χαμηλή παροχή, σήψη), επομένως απαιτεί διεπιστημονική ομαδική προσέγγιση για την πρόληψη, τη διάγνωση, τη διαστρωμάτωση του κινδύνου και τη θεραπεία.

Η έγκαιρη διάγνωση είναι απαραίτητη για τον προσδιορισμό της υποκείμενης αιτίας και την έναρξη της κατάλληλης θεραπείας[6].

Οι πιο ευρέως χρησιμοποιούμενες κλίμακες πρόβλεψης είναι η "Μέθοδος αξιολόγησης σύγχυσης" (Confusion Assessment Method - CAM-ICU) και «Λίστα ελέγχου παραληρήματος εντατικής θεραπείας» (Intensive Care Delirium Screening Checklist - ICDSC7). Το ICDSC είναι επικυρωμένο από το DSM-IV-TR για την αξιολόγηση του παραληρήματος και είναι η κλίμακα που παρουσιάζει επί του παρόντος υψηλότερη εξειδίκευση και θετική προγνωστική αξία, καθιστώντας το ένα χρήσιμο εργαλείο για τη σωστή του διάγνωση[2].

17. Συστηματική ανίχνευση του παραληρήματος με τη χρήση αξιόπιστων κλιμάκων (ICDSC) συνιστάται στη μετεγχειρητική περίοδο καρδιοχειρουργικής επέμβασης τουλάχιστον μία φορά ανά νοσηλευτική βάρδια για έγκαιρη διάγνωση.

Μέτριο επίπεδο τεκμηρίωσης. Ισχυρή σύσταση.

ΒΙΒΛΙΟΓΡΑΦΙΚΕΣ ΑΝΑΦΟΡΕΣ

1. McPherson JA, Wagner CE, Boehm LM, Hall JD, Johnson DC, Miller LR, et al. Delirium in the cardiovascular ICU: exploring modifiable risk factors.Crit Care Med. 2013; 41(2): 405- 13Järvelä K, Porkkala H, Karlsson S, Martikainen T, Selander T, Bendel S. Postoperative Delirium in Cardiac Surgery Patients. J Cardiothorac Vasc Anesth. 2018;32(4):1597- 602. doi:10.1053/j.jvca.2017.12.030

2. EDA and ADS. The DSM-5 criteria, level of arousal and delirium diagnosis: inclusiveness is safer (European Delirium Association and American Delirium Society). BMC Med. 2014;12(1):141. doi:10.1186/s12916-014-0141-2

3. Krzych ŁJ, Wybraniec MT, Krupka-Matuszczyk I, Skrzypek M, Bolkowska A, Wilczyński M, et al. Detailed insight into the impact of postoperative neuropsychiatric complications on mortality in a cohort of cardiac surgery subjects: A 23,000-patient-year analysis. J Car- diothorac Vasc Anesth. 2014;28(3):448-57. doi:10.1053/j.jvca.2013.05.005

4. Abelha FJ, Luís C, Veiga D, Parente D, Fernandes V, Santos P, et al. Outcome and quality of life in patients with postoperative delirium during an ICU stay following major surgery. Crit Care. 2013;17(5):R257. doi:10.1186/cc13084

5. Maldonado JR. Neuropathogenesis of delirium: Review of current etiologic theories and common pathways. Am J Geriatr Psychiatry. 2013;21(12):1190-222. doi:10.1016/j.jagp. 2013.09.005

6. Bergeron N, Dubois MJ, Dumont M, Dial S, Skrobik Y. Intensive care delirium screening checklist: Evaluation of a new screening tool. Intensive Care Med. 2001;27(5):859-64. doi:10.1007/s001340100909

Η πρόληψη του παραληρήματος μέσω μη φαρμακολογικών στρατηγικών όπως η έγκαιρη κινητοποίηση, η διαχείριση του πόνου, η ελαχιστοποίηση και η στοχευμένη τιτλοποίηση της καταστολής, η αποφυγή της χρήσης βενζοδιαζεπίνων, ο επαναπροσανατολισμός του ασθενούς, η γνωστική διέγερση, η μείωση των προβλημάτων ακοής ή/και όρασης (για παράδειγμα, επιτρέποντας τη χρήση συσκευών όπως ακουστικά βαρηκοΐας ή γυαλιά), η χρήση ρολογιών/ημερολογίων και η προώθηση του κανονικού κιρκάδιου μοτίβου ύπνου-αφύπνισης έχουν δείξει ενθαρρυντικά αποτελέσματα[1,2].

Η φαρμακολογική πρόληψη με αλοπεριδόλη ή κεταμίνη δεν έχει δείξει κλινικά οφέλη σε κλινικές δοκιμές μεγάλης κλίμακας[3,4]. Τα στοιχεία δείχνουν ότι η χρήση άτυπων αντιψυχωσικών, όπως αλοπεριδόλη ή μια στατίνη δεν επηρεάζει τη διάρκεια του παραληρήματος ή τη σχετική νοσηρότητά του[5]. Υπάρχουν ενθαρρυντικά δεδομένα σχετικά με τη χρήση άλφα-2 αδρενεργικών αγωνιστών, όπως η δεξμεντετομιδίνη για την πρόληψη της εμφάνισής του[1].

18. Εάν υπάρχουν παράγοντες κινδύνου για την ανάπτυξη μετεγχειρητικού παραληρήματος ή εάν διαγνωστεί η παρουσία του, συνιστάται η χορήγηση δεξμεντετομιδίνης σε χαμηλές δόσεις (0,2 μg/kg/ώρα).

Μέτριο επίπεδο τεκμηρίωσης. Ισχυρή σύσταση.

ΒΙΒΛΙΟΓΡΑΦΙΚΕΣ ΑΝΑΦΟΡΕΣ

1. Skrobik Y, Duprey MS, Hill NS, Devlin JW. Low-dose nocturnal dexmedetomidine Prevents ICU delirium a randomized, placebo-controlled trial. Am J Respir Crit Care Med. 2018; 197(9):1147-56. doi:10.1164/rccm.201710-1995OC.

2. Blair GJ, Mehmood T, Rudnick M, Kuschner WG, Barr J. Nonpharmacologic and Medication Minimization Strategies for the Prevention and Treatment of ICU Delirium: A Narrative Review. J Intensive Care Med. 2019;34(3):183-90. doi:10.1177/0885066618771528.

3. Avidan; Hannah M. R. MaybrierArbi Ben Abdallah; Jacobsohn E; Vlisides PE. PKO; Veseli. RA. Intraoperative ketamine does not affect postoperative delirium or pain after major surgery in older adults: an international, multicentre, double-blind, randomised clinical trial. Lancet. 2017;390:267-75. doi:10.1016/S0140-6736(17)31467-8.

4. Van Den Boogaard M, Slooter AJC, Brüggemann RJM, Schoonhoven L, Beishuizen A, Vermeijden JW,et al. Effect of haloperidol on survival among critically ill adults with a highrisk of delirium the REDUCE randomized clinical trial. JAMA. 2018;319(7):680-90. doi:10. 1001/jama.2018.0160.

5. Abelha FJ, Luís C, Veiga D, Parente D, Fernandes V, Santos P, et al. Outcome and quality of life in patients with postoperative delirium during an ICU stay following major surgery. Crit Care. 2013;17(5):R257. doi:10.1186/cc13084.

Διάρκεια αντιβιοτικής χημείοπροφύλαξης

Μετεγχειρητικά, θα χορηγηθεί αντιβιοτική χημειοπροφύλαξη κατά τις πρώτες 24-48 ώρες μετά τη χειρουργική επέμβαση. Δεν πρέπει να παραταθεί περισσότερο από 48 ώρες για να αποφευχθεί ο κίνδυνος αντοχής στα αντιβιοτικά ή πρόκληση μόλυνσης από το Clostridium difficile[1-4]. Δεν δικαιολογείται η σύνδεση της διάρκειας της αντιβιοτικής χημειοπροφύλαξης με την διατήρηση καθετήρων, σωλήνων ή παροχετεύσεων θώρακα[3].

19. Στην άμεση μετεγχειρητική περίοδο της καρδιοχειρουργικής επέμβασης η αντιβιοτική χημειοπροφύλαξη συνιστάται για τις πρώτες 24-48 ώρες.

Μέτριο επίπεδο τεκμηρίωσης. Ισχυρή σύσταση.

ΒΙΒΛΙΟΓΡΑΦΙΚΕΣ ΑΝΑΦΟΡΕΣ

1. Gelijns AC, Moskowitz AJ, Acker MA, Argenziano M, Geller NL, Puskas JD, etal. Management practices and major infections after cardiac surgery. J Am Coll Cardiol. 2014;64(4):372-381.
2. Lador A, Nasir H, Mansur N, Sharoni E, Biderman P, Leibovici L, et al. Antibiotic prophylaxis in cardiac surgery: systematic review and meta-analysis. J Antimicrob Chemother. 2012; 67(3):541-50.
3. Edwards FH, Engelman RM, Houck P, Shahian DM, Bridges CR. The Society of Thoracic Surgeons Practice Guideline Series: Antibiotic Prophylaxis in Cardiac Surgery, Part I: Duration. Ann Thorac Surg. 2006;81(1):397-404.
4. Poeran J, Mazumdar M, Rasul R, Meyer J, Sacks HS, Koll BS, etal. Antibiotic prophylaxis and risk of Clostridium difficile infection after coronary artery bypass graft surgery. J Thorac Cardiovasc Surg. 2016;151(2):589-597.e2.

7.2.3 ΧΕΙΡΟΥΡΓΙΚΗ ΘΩΡΑΚΑ

Διαχείριση θωρακικών σωλήνων παροχέτευσης και αναρρόφησης μετά από μείζονα πνευμονική εκτομή

Η διαχείριση των υπεζωκοτικών σωλήνων παροχέτευσης επηρεάζει τη διάρκεια παραμονής τους, την νοσηλεία στο νοσοκομείο, το κόστος υγειονομικής περίθαλψης, την ένταση του μετεγχειρητικού πόνου και την αναπνευστική λειτουργία[1]. Για το λόγο αυτό, αποτελούν έναν από τους ακρογωνιαίους λίθους στους οποίους βασίζονται τα προγράμματα εντατικοποίησης της μετεγχειρητικής ανάρρωσης στη θωρακοχειρουργική.

Έχει παρατηρηθεί ότι όταν οι υπεζωκοτικοι σωλήνες παροχέτευσης αφαιρούνται με οροαιματηρή παροχή κάτω από 450 ml/ημέρα μετά τη θωρακοτομή, οι επανεισαγωγές για υποτροπιάζουσα υπεζωκοτική συλλογή είναι 0,55%, ενώ όταν ο ουδός είναι στα 500ml/ημέρα, η συχνότητα εμφάνισης είναι 2,8%[2].

Επί του παρόντος, συνιστάται η χρήση ενός μόνο υπεζωκοτικού σωλήνα παροχέτευσης, αντί για δύο, καθώς μειώνει την ένταση του πόνου και μειώνει τη διάρκεια της παραμονής του υπεζωκοτικού σωλήνα παροχέτευσης[3] χωρίς να διακυβεύεται η ασφάλεια του ασθενούς εφόσον δεν υπάρχει σημαντικός κίνδυνος αιμορραγίας ή δεν αναμένεται πρόβλημα υπολειπόμενου χώρου.

Η χρήση ψηφιακών συστημάτων για τη μέτρηση της διαφυγής αέρα επιτρέπει τη λήψη αντικειμενικών αποφάσεων σχετικά με την χρονική στιγμή αφαίρεσης του υπεζωκοτικου σωλήνα παροχέτευσης. Μεταφέρονται επίσης εύκολα από τον ασθενή και έχουν το δικό τους σύστημα αναρρόφησης, που διευκολύνει την κινητοποίηση του ασθενούς τις πρώτες μετεγχειρητικές ημέρες. Η τακτική εφαρμογή ψηφιακών συστημάτων συνεπάγεται αύξηση του κόστους και υπάρχουν αποκλίσεις μεταξύ των μελετών σχετικά με τη βελτίωση των αποτελεσμάτων (μείωση της νοσηλείας στο νοσοκομείο και της διάρκειας παραμονής του μετεγχειρητικού υπεζωκοτικου σωλήνα παροχέτευσης)[4].

1. Θεωρείται ασφαλής η αφαίρεση της υπεζωκοτικής παροχέτευσης με ημερήσια οροαιματηρή παροχή έως 450 ml.

Μέτριο επίπεδο τεκμηρίωσης. Ισχυρή σύσταση.

2. Η χρήση μιας μόνο υπεζωκοτικής παροχέτευσης μετά από τυπική ρυθμιζόμενη πνευμονική εκτομή μειώνει την ένταση του μετεγχειρητικού πόνου, χωρίς να επηρεάζεται αρνητικά η κλινική ασφάλεια.

Μέτριο επίπεδο τεκμηρίωσης. Ισχυρή σύσταση.

3. Προτείνεται η χρήση ψηφιακών συστημάτων για τη συντόμευση της διάρκειας παραμονής του μετεγχειρητικού υπεζωκοτικου σωλήνα παροχέτευσης και τη μείωση της νοσηλείας στο νοσοκομείο.

Χαμηλό επίπεδο τεκμηρίωσης. Ασθενής σύσταση.

ΒΙΒΛΙΟΓΡΑΦΙΚΕΣ ΑΝΑΦΟΡΕΣ

1. Refai M, Brunelli A, Salati M, Xiumè F, Pompili C, Sabbatini A. The impact of chest tube removal on pain and pulmonary function after pulmonary resection. Eur J Cardiothorac Surg. 2012;41:820-2.

2. Gao S, Zhang Z, Aragón J, Brunelli A, Cassivi S, Chai Y et al. The Society for Translational Medicine: clinical practice guidelines for the postoperative management of chest tube for patients undergoing lobectomy. J Thorac Dis. 2017; 9:3255-64.

3. Zhang X, Lv D, Li M, Sun G, Liu C. The single chest tube versus double chest tube application after pulmonary lobectomy: A systematic review and meta-analysis. J Cancer Res Ther. 2016;12(Supplement):C309-C316.

4. Aldaghlawi F, Kurman JS, Lilly JA, Hogarth DK, Donington J, Ferguson MK, Murgu SD. A Systematic Review of Digital vs Analog Drainage for Air Leak After Surgical Resection or Spontaneous Pneumothorax. Chest. 2020 Jan 17. pii: S0012-3692(20)30029-5.

Προφύλαξη της μετεγχειρητικής κολπικής μαρμαρυγής (ΚΜ) μετά τη θωρακοτομή

Η χειρουργική θώρακος θεωρείται ότι σχετίζεται με υψηλή συχνότητα εμφάνισης αυτής της αρρυθμίας, φτάνοντας το 24% με αναλογία πιθανοτήτων 9,2 (95% CI 6,7-13) σε σύγκριση με άλλες μη καρδιοχειρουργικές επεμβάσεις[1]. Επιπλέον, ο κίνδυνος είναι αυξημένος σε μεγάλες εκτομές (πνευμονεκτομές) σε σύγκριση με λοβεκτομές ή σφηνοειδείς εκτομές πνεύμονα[2].

Η προεγχειρητική χορήγηση αμιωδαρόνης, αναστολέων διαύλων ασβεστίου, κολχικίνης, στατινών ή μαγνήσιου μπορεί να είναι αποτελεσματική στη μείωση του κινδύνου ΚΜ στην μετεγχειρητική περίοδο της θώρακοχειρουργικής επέμβασης, αλλά προς το παρόν δεν υπάρχουν αρκετές πληροφορίες για να προσδιοριστεί σε ποιον τύπο ασθενή τα οφέλη θα υπερτερούσαν των κινδύνων από την εφαρμογή αυτού του μέτρου προεγχειρητικής προφυλαξης στη θωρακοχειρουργική[3,4].

Τα φάρμακα αποκλεισμού των βήτα αδρενεργικών υποδοχέων (β-αποκλειστές) είναι επίσης αποτελεσματικά στην πρόληψη της κολπικής μαρμαρυγής στη θωρακοχειρουργική επέμβαση, αλλά υπάρχουν αμφιβολίες για αυτή τη στρατηγική λόγω των ανεπιθύμητων ενεργειών του βρογχόσπασμου και της υπότασης. Ωστόσο, υπάρχει μια γενική παραδοχή ότι σε ασθενείς που ήταν προηγουμένως υπό θεραπεία με β-αποκλειστές, δεν πρέπει να διακόπτονται πριν από την επέμβαση, αφού μπορεί να προκληθεί φαινόμενο αναζωπύρωσης (rebound), αυξάνοντας τη συχνότητα εμφάνισης αρρυθμιών και υπέρτασης, και επομένως, συνιστάται η συνέχιση της χορήγησής τους κατά τη διάρκεια της περιεγχειρητικής περίοδού.

4. Προφυλακτική χορήγηση β-αποκλειστών, μαγνησίου, αμιωδαρόνης, αναστολέων διαύλων ασβεστίου, στατίνων ή κολχικίνης μειώνει την πιθανότητα εμφάνισης μετεγχειρητικής ΚΜ.

Μέτριο επίπεδο τεκμηρίωσης. Ασθενής σύσταση.

5. Προτείνεται η ενδοφλέβια χορήγηση μαγνησίου όταν τα επίπεδα είναι χαμηλά.

Χαμηλό επίπεδο τεκμηρίωσης. Ασθενής σύσταση.

6. Συνιστάται η συνέχιση της χορήγησης β-αποκλειστών εάν ο ασθενής λάμβανε την συγκεκριμένη αγωγή και προηγουμένως.

Υψηλό επίπεδο τεκμηρίωσης. Ισχυρή σύσταση.

ΒΙΒΛΙΟΓΡΑΦΙΚΕΣ ΑΝΑΦΟΡΕΣ

1. Polanczyk CA, Goldman L, Marcantonio ER, Orav EJ, Lee TH. Supraventricular arrhythmia in patients having noncardiac surgery: Clinical correlates and effect on length of stay. Ann Intern Med.1998;129(4):279-85.
2. Onaitis M, D'Amico T, Zhao Y, O'Brien S, Harpole D. Risk factors for atrial fibrillation after lung cancer surgery: Analysis of the society of thoracic surgeons general thoracic surgery database. Ann Thorac Surg. 2010;90(2):368-74.
3. Frendl G, Sodickson AC, Chung MK, Waldo AL, Gersh BJ, Tisdale JE, et al. 2014 AATS guidelines for the prevention and management of perioperative atrial fibrillation and flutter for thoracic surgical procedures. J Thorac Cardiovasc Surg. 2014; 148:153-93.
4. Zhang L, Gao S. Systematic Review and Meta-analysis of Atrial Fibrillation Prophylaxis After Lung Surgery. J Cardiovasc Pharmacol. 2016; 67:351-7.

Προσπέλαση της θωρακικής κοιλότητας

Η ελάχιστα επεμβατική θωρακική χειρουργική έχει μειώσει σημαντικά τον πόνο και την μετεγχειρητική νοσηρότητα που σχετίζεται με τη συμβατική χειρουργική θώρακος[1]. Αρκετές μετα-ανάλυσεις έχουν δείξει ότι η θωρακοσκοπική προσπέλαση VATS (Video-assisted thoracoscopic surgery) είναι καλύτερη από τη θωρακοτομή στη μείωση της έντασης του μετεγχειρητικού πόνου, των μετεγχειρητικών επιπλοκών, της νοσηλείας στο νοσοκομείο, της διάρκεια παραμονής των παροχετεύσεων θώρακος και στην ποιότητα ζωής[2-4].

Σε περιπτώσεις που δεν γίνεται θωρακοσκοπική προσπέλαση, για την ελαχιστοποίηση της επεμβατικότητας, έχει συσταθεί θωρακοτομή χωρίς διατομή των μυών (muscle sparing), και έχει προταθεί προστασία των μεσοπλεύριων νεύρων, σωστή διαχείριση του διαστολέα πλευρών και κλείσιμο ή επαναπροσέγγιση των πλευρών ή μεσοπλεύρια ράμματα καθώς αυτές οι τεχνικές μειώνουν τον πόνο μετά τη θωρακοτομή[5-7].

7. Η θωρακοσκοπική προσέγγιση για τη θεραπεία του πρώιμου σταδίου μη μικροκυτταρικού καρκίνου του πνεύμονα είναι προτιμότερη από την κλασική θωρακοτομή.

Υψηλό επίπεδο τεκμηρίωσης. Ισχυρή σύσταση.

8. Συνιστάται θωρακοτομή χωρίς διατομή των μυών σε περιπτώσεις που η θωρακοσκοπική προσπέλαση δεν είναι εφικτή.

Μέτριο επίπεδο τεκμηρίωσης. Ισχυρή σύσταση.

9. Σε αυτές τις περιπτώσεις, συνιστάται η προσθήκη τεχνικών διατήρησης των μεσοπλεύριων νεύρων συμπεριλαμβανομένων των μεσοπλεύριων μυϊκών κρημνών και των μεσοπλεύριων ραμμάτων.

Μέτριο επίπεδο τεκμηρίωσης. Ισχυρή σύσταση.

ΒΙΒΛΙΟΓΡΑΦΙΚΕΣ ΑΝΑΦΟΡΕΣ

1. Cattaneo SM, Park BJ, Wilton AS, et al. Use of video-assisted thoracic surgery for lobectomy in the elderly results in fewer complications. Ann Thorac Surg. 2008; 85:235-6.

2. Yan TD, Black D, Bannon PG, et al. Systematic review and meta-analysis of randomized and nonrandomized trials on safety and efficacy of video-assisted thoracic surgery lobectomy for early-stage non-small-cell lung cancer. J Clin Oncol. 2009; 27:2553-62.

3. Zhang Z, Zhang Y, Feng H, Yao Z, Teng J, Wei D, Liu D. Is video-assisted thoracic surgery lobectomy better than thoracotomy for early-stage non-small-cell lung cancer? A systematic review and meta-analysis. Eur J Cardiothorac Surg. 2013; 44:407-14.

4. Cai YX, Fu XN, Xu QZ, Sun W, Zhang N. Thoracoscopic lobectomy versus open lobectomy in stage I non-small cell lung cancer: a meta-analysis. PLoS One. 2013;8:e82366.

5. Bayram SA, Ozcan M, Kaya F.N., Gebitekin C. Rib approximation without intercostal nerve compression reduces post-thoracotomy pain: a prospective randomized study. Eur J Cardiothorac Surg. 2011;39: 570-4.

6. Li S, Feng Z, Wu L, et al. Analysis of 11 trials comparing muscle-sparing with posterolateral thoracotomy. Thorac Cardiovasc Surg. 2014; 62:344-52.
7. Visagan R, McCormack DJ, Shipolini AR, Jarral OA. Are intracostal sutures better than pericostal sutures for closing a thoracotomy Interact Cardiovasc Thorac Surg. 2012; 14: 807- 15.

7.2.4 ΕΓΚΑΥΜΑΤΑ

Προεγχειρητική και μετεγχειρητική αποκατάσταση

Η αποκατάσταση στον εγκαυματία ασθενή θα πρέπει να ξεκινά έγκαιρα, προοδευτικά και χωρίς διακοπές[1]. Ο σχεδιασμός και η εκτέλεσή της πρέπει να ξεκινήσει ταυτόχρονα με την αρχική εκτίμηση του ασθενούς και πρέπει να εξατομικεύεται, αφού οι στόχοι της επανορθωτικής θεραπείας θα ποικίλλουν σε όλη την εξέλιξη των τραυματισμών[2].

Σε διάφορες μελέτες, έχουν αναφερθεί καλύτερα λειτουργικά αποτελέσματα στις ομάδες που έλαβαν πρωτόκολλα εντατικής αποκατάστασης, τα οποία περιλάμβαναν ενεργητική, παθητική και στατική θεραπεία που εφαρμόστηκε από την αρχή του εγκαύματος σε επιλεγμένες περιπτώσεις που είναι επιρρεπείς σε αγκυλώσεις λόγω ουλής (βαθιά εγκαύματα, εμπλοκή αρθρώσεων και προσώπου)[1-6].

1. Σε ασθενείς που έχουν υποστεί εγκαύματα, ιδιαίτερα σε περιπτώσεις εν τω βάθει εγκαυμάτων που προσβάλλουν περιοχές των αρθρώσεων ή του πρόσωπου, η αποκατάσταση πρέπει να ξεκινήσει από την πρώτη στιγμή του εγκαύματος, περιορίζοντας τις μετεγχειρητικές περιόδους ακινητοποίησης.

Μέτριο επίπεδο τεκμηρίωσης. Ισχυρή σύσταση.

ΒΙΒΛΙΟΓΡΑΦΙΚΕΣ ΑΝΑΦΟΡΕΣ

1. Retrouvey H, Wang A, Corkum J, Shahrokhi S. The Impact of Time of Mobilization After Split Thickness Skin Graft on Lower Extremity Wound Healing-Systematic Review and Meta- analysis. JBurn Care Res. 2018 Oct 23;39(6):902-910.

2. Richard R, Baryza MJ, Carr JA, Dewey WS, Dougherty ME, Forbes-Duchart L, et al. Burn rehabilitation and research: proceedings of a consensus summit. J Burn Care Res. 2009;30 (4):543-73.

3. Jacobson K, Fletchall S, Dodd H, Starnes C. Current Concepts Burn Rehabilitation, Part I: Care During Hospitalization. Clin Plast Surg. 2017 Oct;44(4):703-712.

4. Dodd H, Fletchall S, Starnes C, Jacobson K. Current Concepts Burn Rehabilitation, Part II: Long-Term Recovery. Clin Plast Surg. 2017 Oct;44(4):713-728.
5. Anzarut A, Olson J, Singh P, Rowe BH, Tredget EE. The effectiveness of pressure garment therapy for the prevention of abnormal scarring after burn injury: a meta-analysis. J Plast Reconstr Aesthet Surg. 2009;62(1):77-84.
6. Okhovatian F, Zoubine N. A comparison between two burn rehabilitation protocols. Burns. 2007;33(4):429-34.

Διαχείριση δότριων περιοχών στις μεταμοσχεύσεις δέρματος

Υπάρχει μια μεγάλη ποικιλία επιθεμάτων που διατίθενται για χρήση σε δότριες περιοχές δερματικών μοσχευμάτων μερικού πάχους[1]. Σε αυτές τις περιοχές αναμένεται αυθόρμητη επιθηλιοποίηση στις περισσότερες περιπτώσεις[1]. Επιθέματα που προκαλούν ένα υγρό περιβάλλον στο πεδίο επούλωσης αυτών των τραυμάτων είναι τα πιο αποτελεσματικά, αφού προάγουν και επιταχύνουν την επούλωση των δοτριών περιοχών μοσχευμάτων[1-4].

Ανάμεσα σε αυτά, τα υδροκολλοειδή επιθέματα είναι η πρώτη επιλογή για θεραπεία, δεδομένου του χαμηλού ποσοστού σχετιζόμενων λοιμώξεων που παρουσιάζουν, της ταχείας επιθηλιοποίησης και της μείωση του πόνου (που μετριέται σύμφωνα με την οπτική αναλογική κλίμακα)[2-4].

2. Τα υδροκολλοειδή επιθέματα είναι η πρώτη επιλογή για την επούλωση δοτριών περιοχών δερματικών μοσχευμάτων μερικού πάχους, δεδομένης της αναμενόμενης αυθόρμητης επιθηλιοποίησης, των χαμηλών ποσοστών λοίμωξης που σχετίζονται με τη χρήση τους, και του λιγότερου πόνου που αναφέρεται από τον ασθενή σε σύγκριση με άλλα συχνά χρησιμοποιούμενα επιθέματα.

Μέτριο επίπεδο τεκμηρίωσης. Ισχυρή σύσταση.

ΒΙΒΛΙΟΓΡΑΦΙΚΕΣ ΑΝΑΦΟΡΕΣ

1. Brown JE, Holloway SL. An evidence-based review of split-thickness skin graft donor site dressings. Int Wound J. 2018 Dec;15(6):1000-1009.

2. Demirtas Y, Yagmur C, Soylemez F, Ozturk N, Demir A. Management of split-thickness skin graft donor site: a prospective clinical trial for comparison of five different dressing materials. Burns. 2010 Nov;36(7):999-1005.
3. Brölmann FE, Eskes AM, Goslings JC, Niessen FB, de Bree R, Vahl AC, et al; REMBRANDT study group. Randomized clinical trial of donor-site wound dressings after split-skin grafting. Br J Surg. 2013 Apr;100(5):619-27.
4. Macharia M, Nangole FW. Effects of Dressing Materials on Donor Site Morbidity: A Comparative Study at a Tertiary Hospital in Kenya. Ann Plast Surg. 2019 Jul;83(1):22-25.

Έναρξη διατροφής

Τα εγκαύματα είναι ένας τύπος τραύματος που χαρακτηρίζεται από την πρώιμη ανάπτυξη σοβαρής υπερμεταβολικής απόκρισης[1]. Αυτή η απόκριση οδηγεί στη μαζική και επιταχυνόμενη κατανάλωση μακροθρεπτικών και μικροθρεπτικών συστατικών, τα οποία προάγουν και διατηρούν το σύνδρομο φλεγμονώδους απάντησης και τις μεταβολικές αλλοιώσεις που προκαλούν τα εγκαύματα[2]. Ο προκύπτουσα οξεία υποθρεψία αυξάνει επίσης τον κίνδυνο λοίμωξης[2]. Η λοίμωξη είναι η πρώτη αιτία θανάτου στους σοβαρά εγκαυματίες ασθενείς (αυτούς με έγκαυμα περισσότερο από το 20% της επιφάνειας του σώματος) και προκαλεί καθυστέρηση στην επούλωση[3]. Μάλιστα, έχει εδραιωθεί μια σχέση μεταξύ του συνολικού θερμιδικού και πρωτεϊνικού ελλείμματος και της πιθανότητας επιβίωσης. Οι χειρουργικές επεμβάσεις που απαιτούνται σε αυτούς τους ασθενείς αυξάνουν επίσης τον κίνδυνο υποθρεψίας[1-3].

Για το λόγο αυτό και λόγω των πλεονεκτημάτων της πρώιμης επανέναρξης της σίτισης μετά την επέμβαση (διατήρηση του εντερικού βλεννογόνου, μείωση της βακτηριακής αλλοθέσης, διέγερση του εντερικού λεμφικού ιστού ...), συνιστάται η επανέναρξη της διατροφής το συντομότερο δυνατό, γενικά όχι αργότερα από 3 ώρες μετά την επέμβαση[4-5]. Επιπλέον, λαμβάνοντας υπόψη τα σαφή οφέλη της εντερικής διατροφής έναντι της παρεντερικής διατροφής, η εντερική διατροφή είναι η πρώτη επιλογή, εκτός από τις περιπτώσεις που αντενδείκνυται (π.χ. παραλυτικός ειλεός)[2-6].

3. Σε ασθενείς που έχουν υποστεί εγκαύματα συνιστάται έγκαιρη έναρξη της διατροφής μετά την επέμβαση. Σε αυτούς τους ασθενείς, η εντερική διατροφή είναι η πρώτη επιλογή. Η παρεντερική διατροφή προορίζεται μόνο για περιπτώσεις στις οποίες η πρώτη αντενδείκνυται.

Υψηλό επίπεδο τεκμηρίωσης. Ισχυρή σύσταση.

ΒΙΒΛΙΟΓΡΑΦΙΚΕΣ ΑΝΑΦΟΡΕΣ

1. Moreira E, Burghi G, Manzanares W. Update on metabolism and nutrition therapy in critically ill burn patients. Med Intensiva. 2018 Jun-Jul;42(5):306-316.
2. Rousseau AF, Verbrugge AM, Fadeur M, Struvay A, Lefort H. Nutritional aspects of the management of the severely burned patient. Rev Infirm. 2019 Dec;68(256):28-29.
3. ISBI Practice Guidelines Committee; Steering Subcommittee; Advisory Subcommittee. ISBI Practice Guidelines for Burn Care. Burns. 2016 Aug;42(5):953-1021.
4. Chen Z, Wang S, Yu B, Li A. A comparison study between early enteral nutrition and parenteral nutrition in severe burn patients. Burns 2007;33:708-12.
5. Dylewksi ML, Baker M, Prelack K, Weber JM, Hursey D, Lydon M, et al. The safety and efficacy of parenteral nutrition among pediatric patients with burn injuries. Pediatr Crit Care Med 2013;14:e120-5.
6. Wasiak J, Cleland H, Jeffery R. Early versus late enteral nutritional support in adults with burn injury: a systematic review. J Hum Nutr Diet. 2007 Apr;20(2):75-83.

Θερμοκρασία δωματίου

Σε ενήλικες ασθενείς, συνιστάται γενικά η διατήρηση της θερμοκρασίας δωματίου τουλάχιστον στους 21°C[1].Ωστόσο, οι ασθενείς με εγκαύματα έχουν μεγαλύτερη ευαισθησία στην αναπτύξη υποθερμίας λόγω απώλειας της κάλυψης του δέρματος (το οποίο εμπλέκεται σε μηχανισμούς θερμορύθμισης του σώματος)[1-3].

Επιπλέον, η παρατεταμένη υποθερμία σε αυτούς τους ασθενείς μπορεί να αυξήσει την υπερμεταβολική απάντηση που παρουσιάζεται από το ίδιο το

έγκαυμα. Για το λόγο αυτό, η συνιστώμενη θερμοκρασία δωματίου για ασθενείς με εγκαύματα είναι υψηλότερη (μεταξύ 28 °C και 32 °C)[1-3].

4. Η θερμοκρασία περιβάλλοντος στο χειρουργείο ενηλίκων ασθενών που έχουν υποστεί εγκαύματα(κυρίως εκείνους με εκτεταμένες προσβεβλημένες επιφάνειες) θα πρέπει να είναι μεταξύ 28 °C και 32 °C.

Μέτριο επίπεδο τεκμηρίωσης. Ισχυρή σύσταση.

ΒΙΒΛΙΟΓΡΑΦΙΚΕΣ ΑΝΑΦΟΡΕΣ

1. Kelemen JJ III, Cioffi WG Jr, Mason AD Jr, Mozingo DW, McManus WF, Pruitt BA Jr. Effect of ambient temperature on metabolic rate after thermal injury. Ann Surg 1996;223:406-12.

2. Wilmore DW, Mason AD Jr, Johnson DW, Pruitt BA Jr. Effect of ambient temperature on heat production and heat loss in burn patients. J Appl Physiol 1975;38:593-7.

3. Williams FN, Herndon DN, Jeschke MG. The hypermetabolic response to burn injury and interventions to modify this response. Clin Plast Surg 2009;36:583-96.

7.2.5 ΧΕΙΡΟΥΡΓΙΚΗ ΠΑΧΕΟΣ ΕΝΤΕΡΟΥ

Μηχανική προετοιμασία παχέος εντέρου στη χειρουργική του παχέος εντέρου

Οι προηγούμενες κατευθυντήριες οδηγίες του RICA συνιστούσαν τη μη διενέργεια μηχανικής προετοιμασίας του παχέος εντέρου στην χειρουργική του παχέος εντέρου, εκτός από τις περιπτώσεις που πρόκειται να γίνει χειρουργική επέμβαση ορθού με προφυλακτική στομία εκτροπής, καθώς και όταν πρόκειται να γίνει διεγχειρητική κολοσκόπηση.

Τα τελευταία χρόνια, πολυάριθμες δημοσιεύσεις που βασίζονται στη βάση δεδομένων του Εθνικού Προγράμματος Βελτίωσης Χειρουργικής Ποιότητας του Αμερικανικού Κολλεγίου Χειρουργών (American College of Surgeons National Surgical Quality Improvement Program - ACS NSQIP)[1,2] έθεσαν ξανά αμφιβολίες για τη χρήση τους. Αυτές οι πληροφορίες βασίζονται σε αναδρομικές μελέτες με πολυάριθμα ελαττώματα που εγείρουν την πιθανή ύπαρξη σημαντικής μεροληψίας[3]. Σε κάθε περίπτωση, δεν πρέπει να υποτιμώνται δεδομένου του μεγάλου αριθμού ασθενών που περιλαμβάνονται.

Μια πρόσφατη μετα-ανάλυση που μελετούσε την επίδραση της μηχανικής προετοιμασίας έναντι της μη προετοιμασίας του εντέρου[4] και μια φινλανδική πολυκεντρική τυχαιοποιημένη μελέτη[5], κατέληξαν και οι δύο στο συμπέρασμα ότι η μηχανική προετοιμασία του εντέρου δεν επηρεάζει τη συχνότητα εμφάνισης μετεγχειρητικών επιπλοκών ή λοιμώξεων του χειρουργικού πεδίου.

1. Η μηχανική προετοιμασία του παχέος εντέρου δεν βελτιώνει τα αποτελέσματα, μπορεί να προκαλέσει αφυδάτωση και δεν πρέπει να χρησιμοποιείται συστηματικά σε χειρουργικές επεμβάσεις παχέος εντέρου. Μπορεί να προσφέρει οφέλη στη χειρουργική του ορθού με αναστόμωση.

Υψηλό επίπεδο τεκμηρίωσης. Ισχυρή σύσταση.

ΒΙΒΛΙΟΓΡΑΦΙΚΕΣ ΑΝΑΦΟΡΕΣ

1. Klinger, AL, Green, H, Monlezun, DJ, Beck, D, Kann, B., Vargas, HD, et al. The Role of Bowel Preparation in Colorectal Surgery. Annals of Surgery. 2019;269(4), 671-7.

2. Zorbas KA, Yu D, Choudhry A, Ross HM, Philp M. Preoperative bowel preparation does not favor the management of colorectal anastomotic leak. World J Gastrointest Surg. 2019;11(4):218-28.

3. Beyer-Berjot, L, Slim, K. Colorectal surgery and preoperative bowel preparation: aren't we drawing hasty conclusions? Colorectal Disease.2018; 20(11):955-8.

4. Rollins, KE, Javanmard-Emamghissi H, Lobo, DN. Impact of mechanical bowel preparation in Elective Colorectal Surgery: A Meta-analysis. World J Gastroenterol. 2018;24(4):519- 536.

5. Koskenvuo L, Lehtonen T, Koskensalo S, et al. Mechanical and oral antibiotic bowel preparation versus no bowel preparation for elective colectomy (MOBILE): a multicentre, randomised, parallel, single-blinded trial. The Lancet. 2019; 394(10201):840-8.

7.2.6 ΧΕΙΡΟΥΡΓΙΚΗ ΚΕΦΑΛΗΣ ΚΑΙ ΤΡΑΧΗΛΟΥ

Μετεγχειρητική αντιβιοτική χημειοπροφύλαξη σε χειρουργική κεφαλής και τραχήλου με ελεύθερους κρημνούς

Σε καθαρές – επιμολυσμένες χειρουργικές επεμβάσεις κεφαλής και τραχήλου κατά τις οποίες πραγματοποιείται ελεγχόμενο άνοιγμα της αεροπεπτικής οδού, η παράταση της αντιβιοτικής χημειοπροφύλαξης για περισσότερες από 24 ώρες δεν έχει αποδειχθεί ότι παρέχει πλεονεκτήματα[1]. Ωστόσο, όταν χρησιμοποιούνται ελεύθεροι κρημνοί, ο κίνδυνος λοίμωξης είναι μεγαλύτερος και συνιστάται η παράταση της χορήγησης αντιβιοτικών για 48 ώρες[2,3].

1. Συνιστάται βραχυπρόθεσμη αντιβιοτική χημειοπροφύλαξη (λιγότερο από 3 ημέρες), με αντιβιοτικά ευρέος φάσματος που καλύπτουν Gram+, Gram- και αναερόβια μικρόβια σε μικροχειρουργικές επεμβάσεις αποκατάστασης.

Υψηλό επίπεδο τεκμηρίωσης. Ισχυρή σύσταση.

ΒΙΒΛΙΟΓΡΑΦΙΚΕΣ ΑΝΑΦΟΡΕΣ

1. Chiesa-Estomba CM, Lechien JR, Fakhry N, Melkane A, Calvo-Henriquez C, de Siati D, Gonzalez-Garcia JA, Fagan JJ, Ayad T. Systematic review of international guidelines for perioperative antibiotic prophylaxis in Head & Neck Surgery. A YO-IFOS Head & Neck Study Group Position Paper. Head Neck. 2019 Sep;41(9):3434-3456.
2. Haidar YM, Tripathi PB, Tjoa T, Walia S, Zhang L, Chen Y, Nguyen DV, Mahboubi H, Arm- strong WB, Goddard JA. Antibiotic prophylaxis in clean-contaminated head and neck cases with microvascular free flap reconstruction: A systematic review and meta-analysis. Head Neck. 2018 Feb;40(2):417-427.
3. Patel PN, Jayawardena ADL, Walden RL, Penn EB, Francis DO. Evidence-Based Use of Perioperative Antibiotics in Otolaryngology. Otolaryngol Head Neck Surg. 2018 May;158(5): 783-800.

Συστηματική μετεγχειρητική εισαγωγή σε Μονάδες Εντατικής Θεραπείας

Η συστηματική μετεγχειρητική εισαγωγή ασθενών που υποβλήθηκαν σε ογκολογικές επεμβάσεις κεφαλής και τραχήλου σε ΜΕΘ κατά τις πρώτες 24-48 ώρες είναι περιττή στις περισσότερες περιπτώσεις[1-3] και αυξάνει το κόστος και τη νοσηλεία[2].

Οι περισσότεροι ασθενείς μπορούν να νοσηλευτούν σε θαλάμους με εξειδικευμένο νοσηλευτικό προσωπικό, με τη μετέπειτα μεταφορά στη ΜΕΘ να είναι σπάνια[3].

2. Συνιστάται η μεταφορά των ασθενών που υποβάλλονται σε ογκολογική επέμβαση κεφαλής και τραχήλου σε θαλάμους με νοσηλευτικό προσωπικό εξειδικευμένο στην ωτορινολαρυγγολογία, αποφεύγοντας την εισαγωγή στη ΜΕΘ.

Μέτριο επίπεδο τεκμηρίωσης. Ισχυρή σύσταση.

ΒΙΒΛΙΟΓΡΑΦΙΚΕΣ ΑΝΑΦΟΡΕΣ

1. Yu PK, Sethi RKV, Rathi V, et al. Postoperative care in an intermediate-level medical unit after head and neck microvascular free flap reconstruction. Laryngoscope Investig Otolaryngol. 2018;4(1):39-42.
2. Varadarajan VV, Arshad H, Dziegielewski PT. Head and neck free flap reconstruction: What is the appropriate post-operative level of care?. Oral Oncol. 2017;75:61-66.
3. Panwar A, Smith R, Lydiatt D, et al. Vascularized tissue transfer in head and neck surgery: Is intensive care unit-based management necessary?. Laryngoscope. 2016;126(1):73-79.

Μετεγχειρητική παρακολούθηση ελεύθερων κρημνών

Οι ελεύθεροι κρημνοί που χρησιμοποιούνται για αποκατάσταση στις χειρουργικές επεμβάσεις κεφαλής και τραχήλου θα πρέπει να παρακολουθούνται κάθε ώρα κατά τις πρώτες 24 ώρες μετά την επέμβαση[1]. Η παρακολούθηση θα πρέπει να συνεχίζεται καθ' όλη τη διάρκεια της νοσηλείας, αν και η συχνότητα μειώνεται προοδευτικά[2].

Η παρακολούθηση θα πρέπει να περιλαμβάνει τουλάχιστον κλινική εξέταση από έμπειρο προσωπικό[3]. Θα πρέπει να λαμβάνεται υπόψη η χρήση

άλλων τεχνικών παρακολούθησης, ειδικά σε εκείνους τους κρημνούς που δεν είναι προσβάσιμοι σε άμεση κλινική εξέταση[3,4].

3. Οι ελεύθεροι κρημνοί που χρησιμοποιούνται για αποκατάσταση στις χειρουργικές επεμβάσεις κεφαλής και τραχήλου θα πρέπει να παρακολουθούνται στενά μετεγχειρητικά, τουλάχιστον με άμεση κλινική εξέταση

Μέτριο επίπεδο τεκμηρίωσης. Ισχυρή σύσταση.

ΒΙΒΛΙΟΓΡΑΦΙΚΕΣ ΑΝΑΦΟΡΕΣ

1. Kääriäinen M, Halme E, Laranne J. Modern postoperative monitoring of free flaps. Curr Opin Otolaryngol Head Neck Surg. 2018;26(4):248-253.
2. Disa JJ, Cordeiro PG, Hidalgo DA. Efficacy of conventional monitoring techniques in free tissue transfer: an 11-year experience in 750 consecutive cases.Plast Reconstr Surg. 1999;104:97-101.
3. Chae MP, Rozen WM, Whitaker IS, et al. Current evidence for postoperative monitoring of microvascular free flaps: a systematic review. Ann Plast Surg. 2015;74(5):621-632.
4. Kohlert S, Quimby AE, Saman M, Ducic Y. Postoperative Free-Flap Monitoring Techniques. Semin Plast Surg. 2019;33(1):13-16.

Μετεγχειρητική φροντίδα τραύματος

Η χρήση της θεραπείας με υποβοηθούμενης σύγκλεισης με κενό (Vacuum Assisted Closure - VAC) συνιστάται για τη θεραπεία πολύπλοκων χειρουργικών τραυμάτων μετά από μείζονες χειρουργικές επεμβάσεις κεφαλής και τραχήλου[1], ειδικά σε ασθενείς με σιελογόνο συρίγγιο, πολλαπλές παθολογίες, που έχουν προηγουμένως ακτινοβοληθεί ή σε εκείνους με νεκρούς χώρους που ευνοούν την ανάπτυξη λοίμωξης[2,3]. Αν και η χρήση τους μπορεί να εξεταστεί για το κλείσιμο της δότριας περιοχής των ελεύθερων κρημνών, σε αυτή την περίπτωση δεν φαίνεται να παρέχει αξιόλογα πλεονεκτήματα[4].

Η χρήση αποφρακτικών επιθεμάτων, όπως τα επιθέματα πολυουρεθάνης ή υδροκολλοειδούς, μειώνει τον πόνο και ευνοεί την επούλωση στις δότριες περιοχές μοσχευμάτων δέρματος[5].

4. Η χρήση της θεραπείας VAC συνιστάται σε πολύπλοκα χειρουργικά τραύματα κατά τη μετεγχειρητική περίοδο των μεγάλων χειρουργικών επεμβάσεων κεφαλής και τραχήλου όποτε είναι δυνατόν. Γενικά, η χρήση VAC σε δότριες περιοχές ελεύθερων κρημνού δεν θεωρείται πλεονεκτική.

Μέτριο επίπεδο τεκμηρίωσης. Ισχυρή σύσταση.

ΒΙΒΛΙΟΓΡΑΦΙΚΕΣ ΑΝΑΦΟΡΕΣ

1. Mir A, Guys N, Arianpour K, et al. Negative Pressure Wound Therapy in the Head and Neck: An Evidence-Based Approach. Laryngoscope. 2019;129(3):671-683. doi:10.1002/lary. 27262.

2. Yang YH, Jeng SF, Hsieh CH, Feng GM, Chen CC. Vacuum-assisted closure for complicated wounds in head and neck region after reconstruction. J Plast Reconstr Aesthet Surg. 2013;66(8):e209-e216. doi:10.1016/j.bjps.2013.03.006.

3. Maleki Delarestaghi M, Ahmadi A, Dehghani Firouzabadi F, Roomiani M, Dehghani Firouzabadi M, Faham Z. Effect of Low-Pressure Drainage Suction on Pharyngocutaneous Fistula After Total Laryngectomy [published online ahead of print, 2020 Jun 29]. Ann Otol Rhinol Laryngol. 2020.

4. Halama D, Dreilich R, Lethaus B, Bartella A, Pausch NC. Donor-site morbidity after harvesting of radial forearm free flaps-comparison of vacuum-assisted closure with conventional wound care: A randomized controlled trial. J Craniomaxillofac Surg. 2019;47(12):1980- 1985. doi:10.1016/j.jcms.2019.11.004.

5. Serebrakian AT, Pickrell BB, Varon DE, et al. Meta-analysis and Systematic Review of Skin Graft Donor-site Dressings with Future Guidelines. Plast Reconstr Surg Glob Open. 2018;6(9):e1928.

Διαχείριση τραχειοστομίας

Δεδομένου ότι η διενέργεια τραχειοστομίας σχετίζεται με μεγαλύτερη νοσηλεία στο νοσοκομείο, συνιστάται η αποφυγή της όποτε αυτό είναι ασφαλές[1,2]. Σε ορισμένους ασθενείς, μπορεί να αντικατασταθεί από μια περίοδο 24-48 στοματοτραχειακής διασωλήνωσης[1,2]. Όταν γίνει τραχειοστομία, θα πρέπει να επιχειρηθεί το συντομότερο δυνατό η αφαίρεση της. Το

χειρουργικό κλείσιμο της τραχειοστομίας μπορεί να επιταχύνει την ανάρρωση του ασθενούς[3].

5. Συνιστάται η διενέργεια τραχειοστομίας μόνο όταν είναι απαραίτητη και εάν γίνει να αφαιρείται το συντομότερο δυνατό.

Μέτριο επίπεδο τεκμηρίωσης. Ασθενής σύσταση.

ΒΙΒΛΙΟΓΡΑΦΙΚΕΣ ΑΝΑΦΟΡΕΣ

1. Dort JC, Farwell DG, Findlay M, et al. Optimal Perioperative Care in Major Head and Neck Cancer Surgery With Free Flap Reconstruction: A Consensus Review and Recommendations From the Enhanced Recovery After Surgery Society. JAMA Otolaryngol Head Neck Surg. 2017;143(3):292-303.
2. Bater M, King W, Teare J, D'Souza J. Enhanced recovery in patients having free tissue transfer for head and neck cancer: does it make a difference?. Br JOral Maxillofac Surg. 2017; 55(10):1024-1029.
3. Brookes JT, Seikaly H, Diamond C, Mechor B, Harris JR. Prospective randomized trial comparing the effect of early suturing of tracheostomy sites on postoperative patient swallowing and rehabilitation. J Otolaryngol. 2006;35(2):77-82.

Μετεγχειρητική αναπνευστική φυσικοθεραπεία

Οι πνευμονικές επιπλοκές είναι οι πιο συχνές στην μετεγχειρητική περίοδο των μεγάλων χειρουργικών επεμβάσεων κεφαλής και τραχήλου, με σημαντική επίπτωση στη θνησιμότητα και τη νοσηλεία στο νοσοκομείο[1,2]. Συνδέονται κυρίως με δυσφαγία και εισρόφηση δευτερογενώς σε αυτές τις επεμβάσεις[3]. Ο ρόλος της πρώιμης αναπνευστικής φυσικοθεραπείας στην πρόληψη αυτών των επιπλοκών μετά από μείζονες χειρουργικές επεμβάσεις κεφαλής και τραχήλου έχει μελετηθεί ελάχιστα[4], επομένως η ένδειξη για την πραγματοποίησή της βασίζεται στην προέκταση των αποτελεσμάτων που παρέχει μετά από επεμβάσεις σε άλλες περιοχές[5] και πρέπει να εξεταστεί υπό αυτή την οπτική.

6. Η πνευμονική φυσική αποκατάσταση κατά τη μετεγχειρητική περίοδο μείζονος χειρουργικής κεφαλής και τραχήλου δεν έχει αποδειχθεί ότι παίζει σημαντικό ρόλο στην πρόληψη των πιο συχνών πνευμονικών επιπλοκών μετά από αυτές τις παρεμβάσεις και η χρησιμότητά της είναι αμφίβολη.

Χαμηλό επίπεδο τεκμηρίωσης. Ασθενής σύσταση.

ΒΙΒΛΙΟΓΡΑΦΙΚΕΣ ΑΝΑΦΟΡΕΣ

1. Bhattacharyya N, Fried MP. Benchmarks for mortality, morbidity, and length of stay for head and neck surgical procedures. Arch Otolaryngol Head Neck Surg. 2001;127(2):127-132.
2. Semenov YR, Starmer HM, Gourin CG. The effect of pneumonia on short-term outcomes and cost of care after head and neck cancer surgery. Laryngoscope. 2012;122(9):1994- 2004.
3. Di Santo D, Bondi S, Giordano L, et al. Long-term Swallowing Function, Pulmonary Complications, and Quality of Life after Supracricoid Laryngectomy. Otolaryngol Head Neck Surg. 2019;161(2):307-314.
4. Genç A, Ikiz AO, Güneri EA, Günerli A. Effect of deep breathing exercises on oxygenation after major head and neck surgery. Otolaryngol Head Neck Surg. 2008;139(2):281-285.
5. Worrall DM, Tanella A, DeMaria S Jr, Miles BA. Anesthesia and Enhanced Recovery After Head and Neck Surgery. Otolaryngol Clin North Am. 2019;52(6):1095-1114.

7.2.7 ΤΡΑΥΜΑΤΙΟΛΟΓΙΑ ΚΑΙ ΟΡΘΟΠΑΙΔΙΚΗ ΧΕΙΡΟΥΡΓΙΚΗ

1. ΠΡΟΣΘΕΤΙΚΗ ΧΕΙΡΟΥΡΓΙΚΗ ΓΟΝΑΤΟΣ ΚΑΙ ΙΣΧΙΟΥ[1,2]

Οι αρθροπλαστικές ισχίου και γόνατος είναι αποτελεσματικές χειρουργικές επεμβάσεις που βελτιώνουν την ποιότητα ζωής των ασθενών, αυξάνουν τη λειτουργική τους ικανότητα και μειώνουν τον πόνο. Σύμφωνα με τα δεδόμενα του ισπανικού Υπουργείου Υγείας (RAE-CMBD), το 2018 πραγματοποιήθηκαν περισσότερες από 60.000 αρθροπλαστικές γόνατος και περισσότερες από 50.000 αρθροπλαστικές ισχίου στην Ισπανία[3], οι οποίες προκάλεσαν σχεδόν μισό εκατομμύριο ημέρες νοσηλείας, αποτελώντας την πρώτη και την τέταρτη πιο συχνή χειρουργική επέμβαση στο Εθνικό Σύστημα Υγείας. Ως εκ τούτου, αντιπροσωπεύουν σημαντικό όγκο δραστηριοτήτας και προβλημάτων στη λίστα αναμονής. Η γήρανση του πληθυσμού και οι μεγαλύτερες απαιτήσεις για ενεργητική γήρανση καθιστούν προβλέψιμο ότι οι ενδείξεις για αυτή την επέμβαση θα αυξηθούν σημαντικά τα επόμενα χρόνια. Εκτός από τον υψηλό όγκο τους, έχει αποδειχθεί σημαντική μεταβλητότητα στην ιατρική πρακτική[4]. Τα βασικά της σημεία θα εξεταστούν τώρα.

ΒΙΒΛΙΟΓΡΑΦΙΚΕΣ ΑΝΑΦΟΡΕΣ

1. Joint replacement (primary): hip, knee and shoulder [Internet]. London: National Institute for Health and Care Excellence (UK); 2020. Available from: http://www.ncbi.nlm.nih.gov/ books/NBK561385/.
2. McGrory BJ, Weber KL, Jevsevar DS, Sevarino K. Surgical Management of Osteoarthritis of the Knee: Evidence-based Guideline. J Am Acad Orthop Surg. 2016 Aug;24(8):e87-93.
3. Consulta Interactiva del SNS [Internet]. Available from: https://pestadistico.inteligenciadegestion.mscbs.es/publicoSNS/S.
4. Molko S, Dasí-Sola M, Marco F, Combalia A. El proceso de atención de las artroplastias primarias totales de rodilla y cadera en España: un estudio a nivel nacional. Revista Española de Cirugía Ortopédica y Traumatología. 2019 Nov;63(6):408-15.

Προγράμματα ERAS (Enchaned Recovery After Surgery - Βελτιστοποίηση της αναρρώσης μετά από χειρουργική επέμβαση)

Τα προγράμματα ERAS περιλαμβάνουν παρεμβάσεις στα προεγχειρητικά, διεγχειρητικά και μετεγχειρητικά στάδια για τη διευκόλυνση της ανάρρωσης του ασθενούς, μέσω πολυπαραγοντικών ή «ταχείας διαδρομής» (“fast track”) παρεμβάσεων, οι οποίες ρυθμίζουν καθολικά τη συστηματική απάντηση στη χειρουργική επέμβαση.

Αναπτύχθηκαν εδώ και πολύ καιρό στην κοιλιακή ή γυναικολογική χειρουργική, ενώ η ικανότητά τους να μειώνουν την εμφάνιση μεγάλων επιπλοκών στις προγραμματισμένες αρθροπλαστικές έχει πρόσφατα αποδειχθεί[1-4].

1. Η εφαρμογή προγραμμάτων ERAS ή «fast-track» συνιστάται σε ασθενείς που υποβάλλονται σε αρθροπλαστική ισχίου ή γόνατος.

Μέτριο επίπεδο τεκμηρίωσης. Ισχυρή σύσταση.

ΒΙΒΛΙΟΓΡΑΦΙΚΕΣ ΑΝΑΦΟΡΕΣ

1. Soffin EM, YaDeau JT. Enhanced recovery after surgery for primary hip and knee arthroplasty: a review of the evidence. Br J Anaesth. 2016;117(suppl 3):iii62-72.

2. Wainwright TW, Gill M, McDonald DA, Middleton RG, Reed M, Sahota O, et al. Consensus statement for perioperative care in total hip replacement and total knee replacement surgery: Enhanced Recovery After Surgery (ERAS®) Society recommendations. Acta Orthop. 2020;91(1):3-19.

3. Frassanito L, Vergari A, Nestorini R, Cerulli G, Placella G, Pace V, et al. Enhanced recovery after surgery (ERAS) in hip and knee replacement surgery: description of a multidisciplinary program to improve management of the patients undergoing major orthopedic surgery. Musculoskelet Surg. 2020 Apr;104(1):87-92.

4. Ripollés-Melchor J, Abad-Motos A, Díez-Remesal Y, Aseguinolaza-Pagola M, Padin-Ba- rreiro L, Sánchez-Martín R, et al. Association Between Use of Enhanced Recovery After Surgery Protocol and Postoperative Complications in Total Hip and Knee Arthroplasty in the Postoperative Outcomes Within

Enhanced Recovery After Surgery Protocol in Elective Total Hip and Knee Arthroplasty Study (POWER2). JAMA Surg. 2020 Feb 12;e196024.

Βοηθήματα λήψης αποφάσεων

Τα βοηθήματα απόφασεων μπορούν να είναι χρήσιμα για τη λήψη της κοινής απόφασης για την έναρξη της αρθροπλαστικής γόνατος ή ισχίου, αυξάνοντας την αποτελεσματικότητα των συμβουλευτικών και την ικανοποίηση των ασθενών. Μπορούν να συμβάλουν στην αύξηση του βαθμού γνώσης και συμμετοχής του ασθενούς για την παθολογία του και τη θεραπεία της. Αρκετές μελέτες δείχνουν παρόμοιο ποσοστό χειρουργικής ένδειξης, χωρίς επιπτώσεις στο κόστος αλλά βελτίωση στην ικανοποίηση των ασθενών και στην αποτελεσματικότητα των προεγχειρητικών επισκέψεων[1-3].

2. Τα βοηθήματα λήψης αποφάσεων μπορούν να βελτιώσουν την ευαισθητοποίηση και τη συμμετοχή των ασθενών σχετικά με τη διαδικασία τους και να βοηθήσουν στην κοινή λήψη της απόφασης έναρξης της χειρουργικής επέμβασης.

Μέτριο επίπεδο *επίπεδο τεκμηρίωσης. Ασθενής σύσταση.*

ΒΙΒΛΙΟΓΡΑΦΙΚΕΣ ΑΝΑΦΟΡΕΣ

1. Stacey D, Taljaard M, Dervin G, Tugwell P, O’Connor AM, Pomey MP, et al. Impact of patient decision aids on appropriate and timely access to hip or knee arthroplasty for osteoarthritis: a randomized controlled trial. Osteoarthr Cartil. 2016 Jan;24(1):99-107.

2. Bozic KJ, Belkora J, Chan V, Youm J, Zhou T, Dupaix J, et al. Shared decision making in patients with osteoarthritis of the hip and knee: results of a randomized controlled trial. J Bone Joint Surg Am. 2013 Sep 18;95(18):1633-9.

3. Sepucha K, Atlas SJ, Chang Y, Dorrwachter J, Freiberg A, Mangla M, et al. Patient Decision Aids Improve Decision Quality and Patient Experience and Reduce Surgical Rates in Routine Orthopaedic Care: A Prospective Cohort Study. J Bone Joint Surg Am. 2017 Aug 2;99(15):1253-60.

Προεγχειρητικά προγράμματα αποκατάστασης («σχολεία ασθενών»)

Τα προγράμματα προεγχειρητικής αποκατάστασης είναι ομαδικές συνεδρίες που πραγματοποιούνται 2-6 εβδομάδες πριν από την προγραμματισμένη χειρουργική επέμβαση και διδάσκονται από επαγγελματίες υγείας που συμμετέχουν στη μετεγχειρητική αποκατάσταση. Περιλαμβάνουν: πληροφορίες για τις προγραμματισμένες οδούς, παροχή συμβουλών για στρατηγικές βελτίωσης της ανάρρωσης, διδασκαλία ασκήσεων κατά την προετοιμασία για χειρουργική επέμβαση, συμβουλές για τεχνικές διαχείρισης των καθημερινών δραστηριοτήτων διαβίωσης, και πληροφορίες σχετικά με τη χρήση προσαρμοστικού εξοπλισμού όπως ανασηκωμένα καθίσματα τουαλέτας, βοηθήματα ντυσίματος και βοηθήματα βάδισης όπως περιπατητήρες ή πατερίτσες[1-3].

3. Τα προγράμματα προεγχειρητικής αποκατάστασης θα μπορούσαν να μειώσουν τη μετεγχειρητική νοσηλεία και να βελτιώσουν την πρώιμη μετεγχειρητική λειτουργία.

Χαμηλό επίπεδο τεκμηρίωσης. Ασθενής σύσταση.

ΒΙΒΛΙΟΓΡΑΦΙΚΕΣ ΑΝΑΦΟΡΕΣ

1. Silkman Baker C, McKeon JM. Does preoperative rehabilitation improve patient-based outcomes in persons who have undergone total knee arthroplasty? A systematic review. PM R. 2012 Oct;4(10):756-67.
2. Wang L, Lee M, Zhang Z, Moodie J, Cheng D, Martin J. Does preoperative rehabilitation for patients planning to undergo joint replacement surgery improve outcomes? A systematic review and meta-analysis of randomised controlled trials. BMJ Open. 2016 Feb 2;6(2): e009857.
3. Huang S-W, Chen P-H, Chou Y-H. Effects of a preoperative simplified home rehabilitation education program on length of stay of total knee arthroplasty patients. Orthop Traumatol Surg Res. 2012 May;98(3):259-64.

Αναισθησία και αναλγησία

Τα στοιχεία υποδεικνύουν την αποτελεσματικότητα ενός συνδυασμού νευραξονικής αναισθησίας με ή χωρίς περιφερικούς αποκλεισμούς και ενός

πολυπαραγοντικού πρωτοκόλλου για μετεγχειρητική αναλγησία που δεν περιορίζει τη μετεγχειρητική κινητική λειτουργία ούτε παρατείνει τη νοσηλεία στο νοσοκομείο. Η νευραξονική αναισθησία φαίνεται να μειώνει τη μετεγχειρητική ναυτία και να συντομεύει την νοσηλεία στο νοσοκομείο σε σύγκριση με τη γενική αναισθησία[1,2]. Οι περιφερικοί αποκλεισμοί μπορούν να μειώσουν τον μετεγχειρητικό πόνο, να αποτρέψουν επιπλοκές που σχετίζονται με τη χρήση οπιοειδών και να βελτιώσουν την πρώιμη μετεγχειρητική λειτουργία[3-6].

4. Συνιστάται νευραξονική αναισθησία, σε συνδυασμό με μετεγχειρητικά πρωτόκολλα περιφερικής ή/και πολυπαραγοντικής αναισθησίας.

Μέτριο επίπεδο τεκμηρίωσης. Ισχυρή σύσταση.

ΒΙΒΛΙΟΓΡΑΦΙΚΕΣ ΑΝΑΦΟΡΕΣ

1. Pu X, Sun J-M. General anesthesia vs spinal anesthesia for patients undergoing total-hip arthroplasty: A meta-analysis. Medicine (Baltimore). 2019 Apr;98(16):e14925.
2. Johnson RL, Kopp SL, Burkle CM, Duncan CM, Jacob AK, Erwin PJ, et al. Neuraxial vs general anaesthesia for total hip and total knee arthroplasty: a systematic review of comparative-effectiveness research. Br J Anaesth. 2016 Feb;116(2):163-76.
3. Chan E-Y, Fransen M, Parker DA, Assam PN, Chua N. Femoral nerve blocks for acute postoperative pain after knee replacement surgery. Cochrane Database Syst Rev. 2014 May 13;(5):CD009941.
4. Memtsoudis SG, Cozowicz C, Bekeris J, Bekere D, Liu J, Soffin EM, et al. Anaesthetic care of patients undergoing primary hip and knee arthroplasty: consensus recommendations from the International Consensus on Anaesthesia-Related Outcomes after Surgery group (ICAROS) based on a systematic review and meta-analysis. Br J Anaesth. 2019 Sep;123 (3):269-87.
5. Osinski T, Bekka S, Regnaux J-P, Fletcher D, Martinez V. Functional recovery after knee arthroplasty with regional analgesia: A systematic review and meta-analysis of randomised controlled trials. Eur J Anaesthesiol. 2019;36(6):418-26.

6. Opperer M, Danninger T, Stundner O, Memtsoudis SG. Perioperative outcomes and type of anesthesia in hip surgical patients: An evidence based review. World J Orthop. 2014 Jul 18;5(3):336-43.

Τρανεξαμικό οξύ για μείωση της απώλειας αίματος.

Η χορήγηση ενδοφλέβια ή από του στόματος τρανεξαμικού οξέος σε συνδυασμό με τοπικό (ενδοαρθρικό) τρανεξαμικό οξύ μειώνει τον αριθμό των απαραίτητων μεταγγίσεων αίματος, μειώνοντας τον κίνδυνο μετεγχειρητικών αιματωμάτων και λοιμώξεων και είναι οικονομικά αποδοτική[1-5]. Συνιστάται προσαρμογή της δόσης σε ασθενείς με μειωμένη νεφρική λειτουργία.

5. Συνιστάται η χρήση τρανεξαμικού οξέος από το στόματος ή ενδοφλέβια σε σύγκριση με την τοπική του εφαρμογή για τη μείωση της περιεγχειρητικής αιμορραγίας.

Υψηλό επίπεδο τεκμηρίωσης. Ισχυρή σύσταση.

ΒΙΒΛΙΟΓΡΑΦΙΚΕΣ ΑΝΑΦΟΡΕΣ

1. Xu S, Chen JY, Zheng Q, Lo NN, Chia S-L, Tay KJD, et al. The safest and most efficacious route of tranexamic acid administration in total joint arthroplasty: A systematic review and network meta-analysis. Thromb Res. 2019 Apr;176:61-6.

2. Wu Q, Zhang H-A, Liu S-L, Meng T, Zhou X, Wang P. Is tranexamic acid clinically effective and safe to prevent blood loss in total knee arthroplasty? A meta-analysis of 34 randomized controlled trials. Eur J Orthop Surg Traumatol. 2015 Apr;25(3):525-41.

3. Shin Y-S, Yoon J-R, Lee H-N, Park S-H, Lee D-H. Intravenous versus topical tranexamic acid administration in primary total knee arthroplasty: a meta-analysis. Knee Surg Sports Traumatol Arthrosc. 2017 Nov;25(11):3585-95.

4. Ma Q-M, Han G-S, Li B-W, Li X-J, Jiang T. Effectiveness and safety of the use of antifibrinolytic agents in total-knee arthroplasty: A meta-analysis. Medicine (Baltimore). 2020 May; 99(20):e20214.

5. Sun Q, Li J, Chen J, Zheng C, Liu C, Jia Y. Comparison of intravenous, topical or combined routes of tranexamic acid administration in patients

undergoing total knee and hip arthroplasty: a meta-analysis of randomised controlled trials. BMJ Open. 2019 28;9(1):e024350.

Χρήση μετεγχειρητικών παροχετεύσεων

Οι μετεγχειρητικές παροχετεύσεις χρησιμοποιούνται για τη μείωση των αιματωμάτων και άλλων επιπλοκών στο χειρουργικό τραύμα. Υπάρχουν μετεγχειρητικές παροχετεύσεις που επιτρέπουν την ανάκτηση του αίματος που παροχετεύεται από το χειρουργικό τραύμα για να μειωθεί η ανάγκη για μεταγγίσεις. Ωστόσο, η τακτική χρήση παροχετεύσεων φαίνεται να αυξάνει τη μετεγχειρητική αιμορραγία, χωρίς να παρατηρούνται τα οφέλη που προκύπτουν από τη χρήση τους[1-6]. Οι συλλέκτες αίματος μπορεί να μην είναι οικονομικά αποδοτικοί στους περισσότερους ασθενείς, με επαρκή περιεγχειρητική αντιμετώπιση της αναιμίας[7,8].

6. Η τακτική χρήση παροχετεύσεων αναρρόφησης ή ανάκτησης αίματος για αρθροπλαστικές ισχίου και γόνατος είναι περιττή εάν διασφαλίζεται επαρκής έλεγχος της περιεγχειρητικής αιμορραγίας και της αναιμίας.

Υψηλό επίπεδο τεκμηρίωσης. Ισχυρή σύσταση.

ΒΙΒΛΙΟΓΡΑΦΙΚΕΣ ΑΝΑΦΟΡΕΣ

1. Xu H, Xie J, Lei Y, Huang Q, Huang Z, Pei F. Closed suction drainage following routine primary total joint arthroplasty is associated witha higher transfusion rate and longer postoperative length of stay: a retrospective cohort study. J Orthop Surg Res. 2019 May 29; 14(1):163.
2. Parker MJ, Roberts CP, Hay D. Closed suction drainage for hip and knee arthroplasty. A meta-analysis. J Bone Joint Surg Am. 2004 Jun;86(6):1146-52.
3. Abolghasemian M, Huether TW, Soever LJ, Drexler M, MacDonald MP, Backstein DJ. The Use of a Closed-Suction Drain in Revision Knee Arthroplasty May Not Be Necessary: A Prospective Randomized Study. J Arthroplasty. 2016;31(7):1544-8.
4. Zhou X, Li J, Xiong Y, Jiang L, Li W, Wu L. Do we really need closed-suction drainage in total hip arthroplasty? A meta-analysis. Int Orthop. 2013 Nov;37(11):2109-18.

5. Watanabe T, Muneta T, Yagishita K, Hara K, Koga H, Sekiya I. Closed Suction Drainage Is Not Necessary for Total Knee Arthroplasty: A Prospective Study on Simultaneous Bilateral Surgeries of a Mean Follow-Up of 5.5 Years. J Arthroplasty. 2016 Mar;31(3):641-5.
6. Quinn M, Bowe A, Galvin R, Dawson P, O'Byrne J. The use of postoperative suction drainage in total knee arthroplasty: a systematic review. Int Orthop. 2015 Apr;39(4):653-8.
7. Benjamin JB, Colgan KM. Are Routine Blood Salvage/Preservation Measures Justified in All Patients Undergoing Primary TKA and THA? J Arthroplasty. 2015 Jun;30(6):955-8.
8. Dan M, Liu D, Martos SM, Beller E. Intra-operative blood salvage in total hip and knee arthroplasty. JOrthop Surg (Hong Kong). 2016;24(2):204-8.

Πρώιμη μετεγχειρητική φυσιοθεραπεία

Η πρώιμη μετεγχειρητική κινητοποίηση μπορεί να μειώσει τον κίνδυνο φλεβικών θρομβοεμβολικών επιπλοκών, και επιπλέον, μπορεί να μειώσει τη νοσηλεία στο νοσοκομείο και να βελτιώσει την πρώιμη μετεγχειρητική λειτουργία. Για να επιτευχθεί αυτό, είναι απαραίτητος ο επαρκής μετεγχειρητικός έλεγχος του πόνου[1-4].

7. Συνιστάται κινητοποίηση του ασθενούς την ίδια μέρα ή την επομένη μέρα της επέμβασης.

Υψηλό επίπεδο τεκμηρίωσης. Ισχυρή σύσταση.

ΒΙΒΛΙΟΓΡΑΦΙΚΕΣ ΑΝΑΦΟΡΕΣ

1. Karim A, Pulido L, Incavo S. Does Accelerated Physical Therapy After Elective Primary Hip and Knee Arthroplasty Facilitate Early Discharge? Am J Orthop. 2016 Oct;45(6):E337-42.
2. Bohl DD, Li J, Calkins TE, Darrith B, Edmiston TA, Nam D, et al. Physical Therapy on Postoperative Day Zero Following Total Knee Arthroplasty: A Randomized, Controlled Trial of 394 Patients. J Arthroplasty. 2019 Jul;34(7S):S173-S177.e1.

3. Harikesavan K, Chakravarty RD, Maiya AG. Influence of early mobilization program on pain, self-reported and performance based functional measures following total knee replace- ment. J Clin Orthop Trauma. 2019 Apr;10(2):340-4.
4. Temporiti F, Draghici I, Fusi S, Traverso F, Ruggeri R, Grappiolo G, et al. Does walking the day of total hip arthroplasty speed up functional independence? A non-randomized con- trolled study. Arch Physiother. 2020;10:8.

Μετεγχειρητική εξωνοσοκομειακή αποκατάσταση

Από τα αποτελέσματα που αναφέρθηκαν από τους ασθενείς, δεν έχει αποδειχθεί σημαντική διαφορά όσον αφορά την ποιότητα ζωής, ούτε τη λειτουργική ούτε ως προς το ποσοστό επιπλοκών, όταν η εποπτευόμενη ομαδική ή ατομική αποκατάσταση συγκρίνεται με την αυτοκατευθυνόμενη αποκατάσταση από τον ασθενή[1-4]. Η δυνατότητα αποκατάστασης ως εξωτερικών ασθενών θα πρέπει να προσφέρονται σε ασθενείς με δυσκολία στην εκτέλεση των βασικών δραστηριοτήτων της καθημερινής ζωής, που έχουν λειτουργική έκπτωση που δικαιολογεί την ανάγκη για φυσικοθεραπεία, δεν προοδεύουν επαρκώς με αυτοκατευθυνόμενες ασκήσεις ή παρουσιάζουν γνωστική εξασθένηση.

8. Δεν είναι δυνατό να γίνει σύσταση υπέρ ή κατά της μετεγχειρητικής εξωνοσοκομειακής αποκατάστασης σε σύγκριση με άλλες μεθόδους αποκατάστασης.

Χαμηλό επίπεδο τεκμηρίωσης. Ασθενής σύσταση.

ΒΙΒΛΙΟΓΡΑΦΙΚΕΣ ΑΝΑΦΟΡΕΣ

1. Coulter C, Perriman DM, Neeman TM, Smith PN, Scarvell JM. Supervised or Unsupervised Rehabilitation After Total Hip Replacement Provides Similar Improvements for Patients: A Randomized Controlled Trial. Arch Phys Med Rehabil. 2017;98(11):2253-64.
2. Li D, Yang Z, Kang P, Xie X. Home-Based Compared with Hospital-Based Rehabilitation Program for Patients Undergoing Total Knee Arthroplasty for

Osteoarthritis: A Systematic Review and Meta-analysis of Randomized Controlled Trials. Am J Phys Med Rehabil. 2017 Jun;96(6):440-7.
3. Austin MS, Urbani BT, Fleischman AN, Fernando ND, Purtill JJ, Hozack WJ, et al. Formal Physical Therapy After Total Hip Arthroplasty Is Not Required: A Randomized Controlled Trial. J Bone Joint Surg Am. 2017 Apr 19;99(8):648-55.
4. Florez-García M, García-Pérez F, Curbelo R, Pérez-Porta I, Nishishinya B, Rosario Lozano MP, et al. Efficacy and safety of home-based exercises versus individualized supervised outpatient physical therapy programs after total knee arthroplasty: a sys- tematic review and meta-analysis. Knee Surg Sports Traumatol Arthrosc. 2017 Nov;25(11):3340-53.

1. ΚΑΤΑΓΜΑ ΙΣΧΙΟΥ[1-3]

Υπολογίζεται ότι κάθε χρόνο, περίπου 50.000 κατάγματα του εγγύς μηριαίου οστού συμβαίνουν σε ηλικιωμένους στην Ισπανία, με αυξητική τάση λόγω της γήρανσης του πληθυσμού. Αυτό αντιπροσωπεύει σχεδόν το 3% των δαπανών των νοσοκομείων στην Ισπανία, σύμφωνα με στοιχεία του Υπουργείου Υγείας[4-6]. Οι ασθενείς που επηρεάζονται από κάταγμα ισχίου είναι συχνά ευπαθείς ασθενείς με συννοσηρότητες, με υψηλό κίνδυνο επιπλοκών, λειτουργικής επιδείνωσης και νοσηλείας λόγω του τραυματισμού, επιπλέον του ότι παρουσιάζουν θνησιμότητα περίπου 20-30% ένα χρόνο μετά το κάταγμα.

ΒΙΒΛΙΟΓΡΑΦΙΚΕΣ ΑΝΑΦΟΡΕΣ

1. American Academy of Orthopaedic Surgeons. Management of Hip Fractures in the Elderly [Internet]. 1st ed. Rosemont, IL: American Academy of Orthopaedic Surgeons; Available from: http://www.aaos.org/Research/guidelines/HipFxGuideline_rev.pdf.
2. National Clinical Guideline Centre (UK). The Management of Hip Fracture in Adults [Internet]. London: Royal College of Physicians (UK); 2011. Available from: http://www.ncbi. nlm.nih.gov/books/NBK83014/.
3. Bhandari M, Swiontkowski M. Management of Acute Hip Fracture. N Engl J Med. 2017 Nov 23;377(21):2053-62.

4. Azagra R, López-Expósito F, Martin-Sánchez JC, Aguyé A, Moreno N, Cooper C, etal. Changing trends in the epidemiology of hip fracture in Spain. Osteoporos Int. 2014 Apr;25(4): 1267-74.
5. Instituto de Información Sanitaria. Estadísticas Comentadas: La Atención a la Fractura de Cadera en los Hospitales del SNS [Internet]. Madrid: Ministerio de Sanidad y Política Social; 2010. Available from: http://www.msssi.gob.es/estadEstudios/estadisticas/docs/Estadisticas_comentadas_01.pdf.
6. Sáez López P, González Montalvo JI, Gómez Campelo, P, Ojeda Thies C. Registro Nacional Fracturas de Cadera. Informe Anual 2018 [Internet]. Registro Nacional Fracturas de Cadera; 2019. Available from: http://rnfc.es/wp-content/uploads/2019/11/Informe-Anual-RNFC-2018-1.pdf.

Προεγχειρητική περιφερειακή αναλγησία

Η χορήγηση περιφερικής αναισθησίας με λαγονοπεριτονιακό ή μηριαίο αποκλεισμό μειώνει τον προεγχειρητικό πόνο και μπορεί να μειώσει τη συχνότητα του παραληρήματος και την ανάγκη για οπιοειδή[1-3].

9. Η περιφερική αναλγησία συνιστάται για τον έλεγχο του προεγχειρητικού πόνου σε ασθενείς με κατάγματα ισχίου.

Υψηλό επίπεδο τεκμηρίωσης. Ισχυρή σύσταση.

ΒΙΒΛΙΟΓΡΑΦΙΚΕΣ ΑΝΑΦΟΡΕΣ

1. Fletcher AK, Rigby AS, Heyes FLP. Three-in-onefemoral nerve block asanalgesia for fractured neck of femur in the emergency department: a randomized, controlled trial. Ann Emerg Med. 2003 Feb;41(2):227-33.
2. Foss NB, Kristensen BB, Bundgaard M, Bak M, Heiring C, Virkelyst C, et al. Fascia iliaca compartment blockade for acute pain control in hip fracture patients: a randomized, placebo-controlled trial. Anesthesiology. 2007 Apr;106(4):773-8.
3. Steenberg J, Møller AM. Systematic review of the effects of fascia iliaca compartment block on hip fracture patients before operation. Br J Anaesth. 2018 Jun;120(6):1368-80.

Προεγχειρητική έλξη

Δεν έχουν αποδειχθεί διαφορές μεταξύ των ομάδων κατά τη σύγκριση ασθενών με προεγχειρητική έλξη δέρματος έναντι αυτών χωρίς έλξη, όσον αφορά τη μείωση του πόνου και τις αναλγητικές ανάγκες. Η εφαρμογή έλξης μπορεί να είναι επώδυνη, ειδικά αν πρόκειται για σκελετική έλξη. Η προεγχειρητική έλξη μπορεί να κάνει τη νοσηλευτική φροντίδα δύσκολη και να οδηγήσει στην εμφάνιση ελκών πίεσης[1-3].

10. Η τακτική χρήση της προεγχειρητικής έλξης δεν συνιστάται σε ασθενείς με κατάγματα ισχίου.

Μέτριο επίπεδο τεκμηρίωσης. Ισχυρή σύσταση.

ΒΙΒΛΙΟΓΡΑΦΙΚΕΣ ΑΝΑΦΟΡΕΣ

1. Handoll HH, Queally JM, Parker MJ. Pre-operative traction for hip fractures in adults. Cochrane Database Syst Rev. 2011 Dec 7;(12):CD000168.
2. Endo J, Yamaguchi S, Saito M, Itabashi T, Kita K, Koizumi W, et al. Efficacy of preoperative skin traction for hip fractures: a single-institution prospective randomized controlled trial of skin traction versus no traction. JOrthop Sci. 2013 Mar;18(2):250-5.
3. Tosun B, Aslan O, Tunay S. Preoperative position splint versus skin traction in patients with hip fracture: An experimental study. Int J Orthop Trauma Nurs. 2018 Feb;28:8-15.

Καθυστέρηση της χειρουργικής αντιμετωπίσης

Οι ασθενείς θα πρέπει να χειρουργούνται εντός των πρώτων 48 ωρών μετά την εισαγωγή. Οι διορθώσιμες συννοσηρότητες θα εντοπίζονται και θα αντιμετωπίζονται σε εκείνες τις περιπτώσεις όπου είναι απαραίτητο, όπως αναιμία, αντιπηκτική αγωγή, μείωση όγκου, διαταραχές υγρών και ηλεκτρολυτών, μη ελεγχόμενος διαβήτης, μη ελεγχομένη οξεία καρδιακή ανεπάρκεια, διορθώσιμη καρδιακή ισχαιμία ή αρρυθμίες, οξείες λοιμώξεις του αναπνευστικού συστήματος ή παροξύνσεις χρόνιων πνευμονοπαθειών[1-6]. Μια ασθενής συσχέτιση έχει παρατηρηθεί μεταξύ της πρώιμης χειρουργικής επέμβασης και της χαμηλότερης μετεγχειρητικής θνησιμότητας και μια

ισχυρότερη μεταξύ της πρώιμης χειρουργικής επέμβασης και τόσο του χαμηλότερου ποσοστού επιπλοκών όσο και της μικρότερης νοσηλείας στο νοσοκομείο

11. Συνιστάται η χειρουργική αντιμετώπιση εντός των πρώτων 48 ωρών από την εισαγωγή

Μέτριο επίπεδο τεκμηρίωσης. Ισχυρή σύσταση.

ΒΙΒΛΙΟΓΡΑΦΙΚΕΣ ΑΝΑΦΟΡΕΣ

1. Griffiths R, Alper J, Beckingsale A, Goldhill D, Heyburn G, Holloway J, et al. Management of proximal femoral fractures 2011. Anaesthesia. 2012;67(1):85-98.
2. Shiga T, Wajima Z, Ohe Y. Is operative delay associated with increased mortality of hip fracture patients? Systematic review, meta-analysis, and meta-regression. Can J Anaesth. 2008 Mar;55(3):146-54.
3. Simunovic N, Devereaux PJ, Sprague S, Guyatt GH, Schemitsch E, Debeer J, et al. Effect of early surgery after hip fracture on mortality and complications: systematic review and meta-analysis. CMAJ. 2010 Oct 19;182(15):1609-16.
4. Bretherton CP, Parker MJ. Early surgery for patients with a fracture of the hip decreases 30-day mortality. Bone Joint J. 2015 Jan;97-B(1):104-8.
5. Al-Ani AN, Samuelsson B, Tidermark J, Norling A, Ekström W, Cederholm T, et al. Early operation on patients with a hip fracture improved the ability to return to independent living. A prospective studyof 850 patients. JBone Joint Surg Am. 2008 Jul;90(7):1436-42.
6. Berber R, Boulton C, Moran C. Delay to surgery in hip fracture patients: effect on mortality, length of stay, and post-operative morbidity. Injury Extra. 2010 Dec;41(12):173.

Αναισθητική τεχνική

Τόσο η γενική όσο και η ραχιαία αναισθησία ενέχουν κινδύνους και οφέλη που πρέπει να ληφθούν υπόψη ξεχωριστά. Δεν έχει αποδειχθεί σαφής διαφορά στη θνησιμότητα μεταξύ των δύο τύπων αναισθησίας, αν και οι μελέτες χρησιμοποιούν διαφορετικούς χρόνους παρακολούθησης[1-2]. Ορισμένες

μελέτες υποδηλώνουν διαφορά στο ποσοστό των μετεγχειρητικών επιπλοκών όπως το μετεγχειρητικό παραλήρημα προς όφελος της νευραξονικής αναισθησίας, αν και το επίπεδο καταστολής του ασθενούς και η διατήρηση της εγκεφαλικής αιμάτωσης φαίνεται να έχουν μεγαλύτερη επίδραση[1-4]. Θα πρέπει να χρησιμοποιείται η καταλληλότερη τεχνική για κάθε περίπτωση, παρακολουθώντας την καταστολή και αποφεύγοντας την υπόταση.

12. Τόσο γενική όσο και ραχιαία αναισθησία μπορεί να προσφερθεί σε ασθενείς με κάταγμα ισχίου.

Μέτριο επίπεδο τεκμηρίωσης. Ισχυρή σύσταση.

ΒΙΒΛΙΟΓΡΑΦΙΚΕΣ ΑΝΑΦΟΡΕΣ

1. Van Waesberghe J, Stevanovic A, Rossaint R, Coburn M. General vs. neuraxial anaesthesia in hip fracture patients: a systematic review and meta-analysis. BMC Anesthesiol. 2017 28;17(1):87.
2. Zheng X, Tan Y, Gao Y, Liu Z. Comparative efficacy of Neuraxial and general anesthesia for hip fracture surgery: a meta-analysis of randomized clinical trials. BMC Anesthesiol. 2020 Jun 30;20(1):162.
3. Chen DX, Yang L, Ding L, Li SY, Qi YN, Li Q. Perioperative outcomes in geriatric patients undergoing hip fracture surgery with different anesthesia techniques: A systematic review and meta-analysis. Medicine (Baltimore). 2019 Dec;98(49):e18220.
4. Sieber FE, Neufeld KJ, Gottschalk A, Bigelow GE, Oh ES, Rosenberg PB, et al. Effect of Depth of Sedation in Older Patients Undergoing Hip Fracture Repair on Postoperative Delirium: The STRIDE Randomized Clinical Trial. JAMA Surg. 2018 01;153(11): 987-95.

Μετεγχειρητική αποκατάσταση

Αρκετές μελέτες υποστηρίζουν τα οφέλη των ασκήσεων εντατικής αποκατάστασης σε ηλικιωμένους ασθενείς με κατάγματα ισχίου, με βελτίωση των λειτουργικών αποτελεσμάτων, κινητικότητα και αυτονομία στην εκτέλεση βασικών δραστηριοτήτων της καθημερινής ζωής καθώς και βελτίωση της ποιότητας ζωής. Δεν είναι σαφές τι είδους συστατικά πρέπει να περιλαμβάνει

το πρωτόκολλο αποκατάστασης, αν και έχουν αποδειχθεί βελτιώσεις τόσο με τις ασκήσεις αντίστασης όσο και με τις ασκήσεις ισορροπίας[1-4].

13. Θα πρέπει να προσφέρεται κινητοποίηση την επομένη της επέμβασης και έγκαιρη αποκατάσταση σε ασθενείς που χειρουργούνται για κάταγμα ισχίου.

Υψηλό επίπεδο τεκμηρίωσης. Ισχυρή σύσταση.

ΒΙΒΛΙΟΓΡΑΦΙΚΕΣ ΑΝΑΦΟΡΕΣ

1. Diong J, Allen N, Sherrington C. Structured exercise improves mobility after hip fracture: a meta-analysis with meta-regression. Br J Sports Med. 2016 Mar;50(6):346-55.
2. Mangione KK, Craik RL, Palombaro KM, Tomlinson SS, Hofmann MT. Home-based leg-strengthening exercise improves function 1 year after hip fracture: a randomized controlled study. J Am Geriatr Soc. 2010 Oct;58(10):1911-7.
3. Beckmann M, Bruun-Olsen V, Pripp AH, Bergland A, Smith T, Heiberg KE. Effect of exercise interventions in the early phase to improve physical function after hip fracture - A systematic review and meta-analysis. Physiotherapy. 2020; 108:90-7.
4. Chudyk AM, Jutai JW, Petrella RJ, Speechley M. Systematic review of hip fracture rehabilitation practices in the elderly. Arch Phys Med Rehabil. 2009 Feb;90(2):246-62.

Μετεγχειρητική φόρτιση

Δεδομένης της κακής λειτουργικής εφεδρείας πολλών ασθενών με κάταγμα ισχίου, οποιοσδήποτε προδιαγεγραμμένος περιορισμός φόρτωσης και κινητικότητας μπορεί να θέσει σε κίνδυνο τη μετεγχειρητική φροντίδα και να παρατείνει τη νοσηλεία, καθώς έχει τη δυνατότητα να θέσει σε κίνδυνο την ανεξαρτησία, τον προορισμό εξόδου και τη λειτουργική αποκατάσταση. Διάφορες μελέτες δεν μπόρεσαν να καταδείξουν υψηλότερο ποσοστό μετεγχειρητικών επιπλοκών σε εκείνους τους ασθενείς χωρίς περιορισμένη μετεγχειρητική φόρτιση[1-3].

14. Η φόρτιση συνιστάται σε ασθενείς που υποβάλλονται σε κάταγμα ισχίου.

Μέτριο επίπεδο τεκμηρίωσης. Ισχυρή σύσταση.

ΒΙΒΛΙΟΓΡΑΦΙΚΕΣ ΑΝΑΦΟΡΕΣ

1. Warren J, Sundaram K, Anis H, McLaughlin J, Patterson B, Higuera CA, etal. Theassociation between weight-bearing status and early complications in hip fractures. Eur J Orthop Surg Traumatol. 2019Oct;29(7):1419-27.

2. Pfeufer D, Zeller A, Mehaffey S, Böcker W, Kammerlander C, Neuerburg C. Weight-bearing restrictions reduce postoperative mobility in elderly hip fracture patients. Arch Orthop Trauma Surg. 2019 Sep;139(9):1253-9.

3. Ottesen TD, McLynn RP, Galivanche AR, Bagi PS, Zogg CK, Rubin LE, et al. Increased complications in geriatric patients with a fracture of the hip whose postoperative weight-bearing is restricted: an analysis of 4918 patients. Bone Joint J. 2018;100-B(10):1377-84.

Διεπιστημονική ορθογηριατρική διαχείριση

Θα πρέπει να προσφέρεται πολυεπιστημονική φροντίδα σε ηλικιωμένους ασθενείς που έχουν υποστεί κατάγματα ισχίου, συμπεριλαμβανομένων, εκτός από τραυματολόγους, κλινικών ειδικών (γηρίατρων ή/και κλινικών γιατρών από άλλες ειδικότητες), φυσίατρων, αναισθησιολόγων, νοσηλευτών, θεραπευτών κ.λπ. Έχει αποδειχθεί ότι η ορθογηριατρική πολυεπιστημονική διαχείριση έχει ελαφρά επίδραση στη θνησιμότητα και τη νοσηλεία στο νοσοκομείο, αλλά μεγαλύτερη επίδραση στη λειτουργική αποκατάσταση και την αυτονομία για βασικές δραστηριότητες της καθημερινής ζωής. Η επίδρασή της στο ποσοστό επανεισαγωγής ή νοσηλείας μετά το κάταγμα δεν είναι τόσο ξεκάθαρη[1-5].

15. Η ορθογηριατρική φροντίδα συνιστάται στα πλαίσια μιας διεπιστημονικής ομάδας για ευπαθείς ασθενείς με κάταγμα ισχίου.

Μέτριο επίπεδο τεκμηρίωσης. Ισχυρή σύσταση.

ΒΙΒΛΙΟΓΡΑΦΙΚΕΣ ΑΝΑΦΟΡΕΣ

1. Mukherjee K, Brooks SE, Barraco RD, Como JJ, Hwang F, Robinson BRH, etal. Elderly adults with isolated hip fractures - orthogeriatric care versus standard care: A practice management guideline from the Eastern Association for the Surgery of Trauma. J Trauma Acute Care Surg. 2020 Feb;88(2):266-78.

2. Eamer G, Taheri A, Chen SS, Daviduck Q, Chambers T, Shi X, etal. Comprehensive geriatric assessment for older people admitted to a surgical service. Cochrane Database Syst Rev. 2018 Jan 31;1:CD012485.

3. Moyet J, Deschasse G, Marquant B, Mertl P, Bloch F. Which is the optimal orthogeriatric care model to prevent mortality of elderly subjects post hip fractures? A systematic review and meta-analysis based on current clinical practice. Int Orthop. 2019;43(6):1449-54.

4. Grigoryan KV, Javedan H, Rudolph JL. Orthogeriatric care models and outcomes in hip fracture patients: a systematic review and meta-analysis. J Orthop Trauma. 2014 Mar;28 (3):e49-55.

5. Kammerlander C, Roth T, Friedman SM, Suhm N, Luger TJ, Kammerlander-Knauer U, et al. Ortho-geriatric service - a literature review comparing different models. Osteoporos Int. 2010 Dec;21(Suppl 4):S637-646.

8. Διάγραμμα δεικτών

Γενικοί δείκτες για την ανάλυση της ποιότητας της διαδικασίας φροντίδας παρουσιάζονται σε αυτό το κεφάλαιο. Τα πρότυπα δεν συμπεριλήφθηκαν, καθώς υπάρχουν διαφορετικές χειρουργικές επεμβάσεις στην κοιλιακή χειρουργική με διαφορετικά αποτελέσματα. Επιπλέον, δεν είναι δυνατό να βρεθούν αναφορές για πολλούς από τους δείκτες της διαδικασίας.

*Για αυτούς τους δείκτες απαιτείται ο καθορισμός συγκεκριμένων κριτηρίων.

8.1. ΔΕΙΚΤΕΣ ΔΙΑΔΙΚΑΣΙΑΣ

ΕΠΑΡΚΕΙΑ ΚΑΛΥΨΗΣ

$$\frac{\text{Ασθενείς που πληρούν τα κριτήρια ένταξης στο RICA και έχουν ενταχθεί στο πρόγραμμα}}{\text{Χειρουργημένοι ασθενείς στα πλαίσια του RICA}} \times 100$$

ΕΠΑΡΚΕΙΑ ΤΗΣ ΔΙΑΔΙΚΑΣΙΑΣ

$$\frac{\text{Χειρουργημένοι ασθενείς που πληρούν τα κριτήρια ένταξης στο RICA}}{\text{Χειρουργημένοι ασθενείς στα πλαίσια του RICA}} \times 100$$

ΠΡΟΕΓΧΕΙΡΗΤΙΚΕΣ ΠΛΗΡΟΦΟΡΙΕΣ

$$\frac{\text{Χειρουργημένοι ασθενείς στα πλαίσια του RICA στους οποίους έχουν παρασχεθεί προφορικές και γραπτές πληροφορίες*}}{\text{Χειρουργημένοι ασθενείς στα πλαίσια του RICA}} \times 100$$

ΠΡΟΕΓΧΕΙΡΗΤΙΚΗ ΕΚΤΙΜΗΣΗ

$$\frac{\text{Χειρουργημένοι ασθενείς στα πλαίσια του RICA που έχουν υποβληθεί σε επαρκή προεγχειρητική αξιολόγηση*}}{\text{Χειρουργημένοι ασθενείς στα πλαίσια του RICA}} \times 100$$

ΑΞΙΟΛΟΓΗΣΗ ΔΙΑΤΡΟΦΙΚΟΥ ΚΙΝΔΥΝΟΥ

$$\frac{\text{Χειρουργημένοι ασθενείς στα πλαίσια του RICA που έχουν υποβληθεί σε προεγχειρητική διατροφική αξιολόγηση*}}{\text{Χειρουργημένοι ασθενείς στα πλαίσια του RICA}} \times 100$$

ΠΡΟΕΓΧΕΙΡΗΤΙΚΗ ΑΞΙΟΛΟΓΗΣΗ ΑΝΑΙΜΙΑΣ

Χειρουργημένοι ασθενείς στα πλαίσια του RICA με αιμοσφαιρίνη > 13 g/dl

x 100

Χειρουργημένοι ασθενείς στα πλαίσια του RICA

ΠΡΟΕΓΧΕΙΡΗΤΙΚΗ ΝΗΣΤΕΙΑ ΚΑΙ ΧΟΡΗΓΗΣΗ ΔΙΑΛΥΜΑΤΟΣ ΥΔΑΤΑΝΘΡΑΚΩΝ

Χειρουργημένοι ασθενείς στα πλαίσια του RICA με επαρκή χρόνο νηστείας και προεγχειρητική δίαιτα *

x 100

Χειρουργημένοι ασθενείς στα πλαίσια του RICA

ΠΡΟΦΥΛΑΞΗ ΘΡΟΜΒΟΕΜΒΟΛΗΣ

Χειρουργημένοι ασθενείς στα πλαίσια του RICA με επαρκή προφύλαξη από θρομβοεμβολή*

x 100

Χειρουργημένοι ασθενείς στα πλαίσια του RICA

ΑΝΤΙΒΙΟΤΙΚΗ ΧΗΜΕΙΟΠΡΟΦΥΛΑΞΗ

Χειρουργημένοι ασθενείς στα πλαίσια του RICA στους οποίους έχει χορηγηθεί επαρκής αντιβιοτική χημειοπροφύλαξη*

x 100

Χειρουργημένοι ασθενείς στα πλαίσια του RICA

ΧΕΙΡΟΥΡΓΙΚΗ ΠΡΟΣΕΓΓΙΣΗ

Χειρουργημένοι ασθενείς στα πλαίσια του RICA που έχουν υποβληθεί σε επέμβαση με ελάχιστα επεμβατική προσπέλαση*

x 100

Χειρουργημένοι ασθενείς στα πλαίσια του RICA

ΔΙΑΧΕΙΡΙΣΗ ΥΓΡΩΝ

Χειρουργημένοι ασθενείς στα πλαίσια του RICA με σωστή χορήγηση υγρών στην περιεγχειρητική περίοδο*

x 100

Χειρουργημένοι ασθενείς στα πλαίσια του RICA

ΠΡΟΛΗΨΗ ΥΠΟΘΕΡΜΙΑΣ

Χειρουργημένοι ασθενείς στα πλαίσια του RICA με διεγχειρητική παρακολούθηση της θερμοκρασίας σώματος*

x 100

Χειρουργημένοι ασθενείς στα πλαίσια του RICA

ΡΙΝΟΓΑΣΤΡΙΚΟΣ ΣΩΛΗΝΑΣ

Χειρουργημένοι ασθενείς στα πλαίσια του RICA στους οποίους τοποθετήθηκε ρινογαστρικός σωλήνας

——————————————————— x 100

Χειρουργημένοι ασθενείς στα πλαίσια του RICA

ΑΝΑΛΓΗΣΙΑ

Χειρουργημένοι ασθενείς στα πλαίσια του RICA που έλαβαν επαρκή αναλγησία*

——————————————————— x 100

Χειρουργημένοι ασθενείς στα πλαίσια του RICA

ΔΙΑΤΡΟΦΙΚΗ ΥΠΟΣΤΗΡΙΞΗ

Χειρουργημένοι ασθενείς στα πλαίσια του RICA που έχουν λάβει επαρκή διατροφική υποστήριξη*

——————————————————— x 100

Χειρουργημένοι ασθενείς στα πλαίσια του RICA

ΠΡΩΙΜΗ ΚΙΝΗΤΟΠΟΙΗΣΗ

Χειρουργημένοι ασθενείς στα πλαίσια του RICA που έχουν υποβληθεί σε επαρκή μετεγχειρητική κινητοποίηση

——————————————————— x 100

Χειρουργημένοι ασθενείς στα πλαίσια του RICA

8.1. ΔΕΙΚΤΕΣ ΑΠΟΤΕΛΕΣΜΑΤΩΝ

ΚΛΙΝΙΚΗ ΑΠΟΤΕΛΕΣΜΑΤΙΚΟΤΗΤΑ

Χειρουργημένοι ασθενείς στα πλαίσια του RICA που χρειάζονται επανεπέμβαση λόγω αιμορραγίας

——————————————————— x 100

Χειρουργημένοι ασθενείς στα πλαίσια του RICA

Χειρουργημένοι ασθενείς στα πλαίσια του RICA και χρειάζονται εισαγωγή σε μονάδα εντατικής θεραπείας

——————————————————— x 100

Χειρουργημένοι ασθενείς στα πλαίσια του RICA

$$\frac{\text{Χειρουργημένοι ασθενείς στα πλαίσια του RICA με απρογραμμάτιστη επανεισαγωγή εντός 30 ημερών από την επέμβαση για λόγους που σχετίζονται με αυτήν}}{\text{Χειρουργημένοι ασθενείς στα πλαίσια του RICA}} \times 100$$

$$\frac{\text{Χειρουργημένοι ασθενείς στα πλαίσια του RICA και απεβίωσαν εντός 30 ημερών από την επέμβαση}}{\text{Χειρουργημένοι ασθενείς στα πλαίσια του RICA}} \times 100$$

$$\frac{\text{Χειρουργημένοι ασθενείς στα πλαίσια του RICA και παρουσιάζουν λοίμωξη χειρουργικού πεδίου εντός 30 ημερών από την επέμβαση}}{\text{Χειρουργημένοι ασθενείς στα πλαίσια του RICA}} \times 100$$

ΑΠΟΔΟΤΙΚΟΤΗΤΑ

$$\frac{\text{Χειρουργημένοι ασθενείς στα πλαίσια του RICA που έλαβαν εξιτήριο από το νοσοκομείο σύμφωνα με το πλάνο}}{\text{Χειρουργημένοι ασθενείς στα πλαίσια του RICA}} \times 100$$

ΙΚΑΝΟΠΟΙΗΣΗ ΑΣΘΕΝΩΝ

$$\frac{\text{Χειρουργημένοι ασθενείς στα πλαίσια του RICA και είναι πολύ ικανοποιημένοι με τη φροντίδα που έλαβαν**}}{\text{Χειρουργημένοι ασθενείς στα πλαίσια του RICA}} \times 100$$

**Αυτοί οι δείκτες απαιτούν καθορισμό ρητών κριτηρίων

9. Στρατηγική υλοποίησης

ΙΣΤΟΡΙΚΟ

Από το 2007, μια αφοσιωμένη ομάδα ειδικών από την Ισπανία, πρότεινε να τροποποιηθούν μη ασφαλείς πρακτικές ώστε να πραγματοποιούν τις χειρουργικές επεμβάσεις τους υπό το πρίσμα «Πρώτα μην πονάς» ("primun non nocere"). Η ομάδα αποτελείται από επαγγελματίες υγείας όπως Αναισθησιολόγοι, Χειρουργοί, Νοσηλευτές, Ενδοκρινολόγοι, Διατροφολόγοι, Αιματολόγοι, Φυσίατροι, Ιατροί Προληπτικής Ιατρικής, Εξειδικευμένοι στην Μεθοδολογία, σκοπός των οποίων είναι η πραγματοποίηση μιας ασφαλούς περιεγχειρητικής κλινικής πρακτικής που υποστηρίζεται από την Ιατρική την βασισμένη στην τεκμηρίωση (evidence based medicine).

Ως αποτέλεσμα της εργασίας που πραγματοποιήθηκε, γεννήθηκε η Βελτιστοποίηση της Ανάρρωσης στη Χειρουργική της Κοιλιάς (Vía Clínica de Recuperación Intensificada en Cirugía Abdominal – RICA – Recovery Intensification for optimal Care in Adult's surgery), που υποστηρίζεται από το Ισπανικό Υπουργείο Υγείας, Κοινωνικής Ασφάλισης και Ισότητας (Ministerio de Sanidad, Servicios Sociales e Igualdad) και το Ινστιτούτο Επιστημών Υγείας της Αραγονίας (Instituto Aragonés de Ciencias de la Salud), και υπόκειται στον έλεγχο του Οδηγού Υγείας (Guía Salud). Ενημερώθηκε και αναθεωρήθηκε το 2020, ενσωματώνοντας και άλλες ειδικότητες σε μια προσπάθεια να καλύψει την πλειονότητα των χειρουργικών επεμβάσεων των ενηλίκων.

Ωστόσο, τα εμπόδια που απορρέουν από μια μακρά εδραιωμένη παράδοση, καθώς και η αντίσταση στην αλλαγή, καθιστούν δύσκολη την εφαρμογή κλινικών πρωτοκόλλων ή μονοπατιών και απαιτούν ένα στρατηγικό σχέδιο εφαρμογής. Για το λόγο αυτό, είναι πολύ σημαντικό να γνωρίζουμε τα εμπόδια και να προάγουμε την ηγεσία κατά τη στιγμή της εφαρμογής τους[1,2].

Η εφαρμογή ενός προγράμματος RICA συνεπάγεται την τυποποίηση της φροντίδας και της θεραπείας. Μειώνει τη μεταβλητότητα της κλινικής πρακτικής

και συνηθίζει τους επαγγελματίες σε πρωτόκολλα πρακτικής. Αυτό δημιουργεί μεγαλύτερη ασφάλεια, καθώς περιλαμβάνει την εφαρμογή ορθών συμπεριφορών που οδηγούν στην αποφυγή λαθών ή αποφυγή της λήθης που μπορούν να έχουν επιβλαβείς επιπτώσεις στους ασθενείς μας. Όλα αυτά μεταφράζονται σε καλύτερα κλινικά αποτελέσματα, καλύτερη και ασφαλέστερη ποιότητα φροντίδας και συνολική βελτίωση της ευημερίας του πληθυσμού μας από την άποψη της υγείας.

ΣΤΟΧΟΙ

Με απώτερο στόχο την επίτευξη μιας ομοιόμορφης, συναινετικής και πολυκεντρικής εφαρμογής των προγραμμάτων/πρωτοκόλλων περιεγχειρητικής ιατρικής που προέρχονται από τον κλινικό οδηγό για την Εντατικοποίηση της Ανάρρωσης για τη Βέλτιστη Φροντίδα στην Χειρουργική Ενήλικων - «Recovery Intensification for optimal Care in Adults surgery - RICA» - πιστεύουμε ότι είναι απαραίτητο να δημιουργηθούν στρατηγικές συμμαχίες μεταξύ των επιστημονικών εταιριών και των φορέων που έχουν υπογράψει το παρόν έγγραφο και να αναπτυχθεί ένα ομοιογενές πλάνο υλοποίησης (implementation - πλάνο IMPRICA), με τα ακόλουθα βήματα:

1. ΔΙΑΔΟΣΗ - ΔΙΑΧΥΣΗ

Στόχος: παροχή γνώσεων και βασικών σημείων του οδηγού RICA, ώστε να φτάσει σε ολόκληρο το κλινικό περιβάλλον (ανά ειδικότητα) στην Ισπανία.

Επιθυμητά μέτρα:

- Διεξαγωγή σεμιναρίων σε περιφερειακό και τοπικό επίπεδο.
- Συμμετοχή σε συνέδρια για την ασφάλεια των ασθενών, την αλλαγή αντιμετώπισης, τη μεταβλητότητα της κλινικής πρακτικής.
- Προγραμματισμός ομάδων συζήτησης (discussion panels) και συμποσίων στα προεξαρχόντα εθνικά συνέδρια των εταιρειών που εμπλέκονται στο έγγραφο.
- Παροχή της κατάλληλης τεκμηρίωσης τόσο για τον επαγγελματία υγείας όσο και για τους ασθενείς για τον οδηγό RICA, έγγραφα πληροφοριών για τις διαδικασίες, βίντεο επίδειξης, ...

- Χώρος σε ψηφιακές πλατφόρμες για πληροφορίες (παρουσιάσεις, podcast, webinnars - διαδικτυακά σεμινάρια…).

2. ΣΥΝΑΝΤΗΣΕΙΣ ΚΑΙ ΣΥΝΕΔΡΙΕΣ ΣΕ ΑΥΤΟΝΟΜΕΣ ΚΟΙΝΟΤΗΤΕΣ.

Προκειμένου να εμπλακούν οι διαχειριστές των διαφόρων αυτόνομων κοινοτήτων, θα ήταν επιθυμητό να οργανωθούν συναντήσεις με τη συμμετοχή των ανώτερων διευθυντικών στελεχών των διευθύνσεων υγείας, καλώντας τους διαφορετικούς «ενδιαφερόμενους» της κάθε περιοχής, να υποδείξουν τους διοικητικούς διευθυντές και τους διευθυντές ιατρικής και νοσηλευτικής υπηρεσίας, οι οποίοι είναι υπεύθυνοι για την ποιότητα και την εκπαίδευση. Αυτές οι συνεδρίες θα πρέπει να προωθούνται από τους φορείς λήψης αποφάσεων, κατά προτίμηση από τον ίδιο τον Σύμβουλο ή τον Διευθυντή Προγραμματισμού για την Υγεία.

3. ΕΚΠΑΙΔΕΥΣΗ

Θα πρέπει να εξεταστεί η δυνατότητα διοργάνωσης κατάλληλα διαπιστευμένων και μοριοδοτούμενων σεμιναρίων εκπαίδευσης για διαφορετικούς κλινικούς ιατρούς που ενδιαφέρονται όχι μόνο για τη σωστή εφαρμογή του οδηγού RICA στα κέντρα τους, αλλά και για την επίτευξη των υψηλότερων επιπέδων ποιοτικής φροντίδας.

4. ΕΘΝΙΚΟ ΜΗΤΡΩΟ RICA

Προτείνεται η δημιουργία ενός εθνικού μητρώου RICA για την αξιολόγηση του βαθμού εφαρμογής στα διάφορα νοσοκομειακά κέντρα, καθώς και για την παρακολούθηση των δεικτών ποιότητας που προτείνονται σε αυτόν τον οδηγό.

ΒΙΒΛΙΟΓΡΑΦΙΚΕΣ ΑΝΑΦΟΡΕΣ

1. Giménez-Júlvez T, Hernández-García I, Aibar-Remón C, Gutiérrez-Cía I, Febrel-Bordejé M. Culture of patient safety in directors and managers of a health service. Sanitary Gazette. 2017. 31: 423-426. DOI: 10.1016 / j.gaceta.2017.01.009).

2. Gramlich L, Nelson G, Nelson A, Lagendyk L, Gilmour LE, Wasylak T. Moving enhanced recovery after surgery from implementation to sustainability

across a health system: a qualitative assessment of leadership perspectives. BMC Health Serv Res. 2020 Apr 26; 20 (1): 361. doi: 10.1186 / s12913-020-05227-0. PMID: 32336268).

10. Παραρτήματα

10.1. ΠΡΟΦΥΛΑΞΗ ΝΑΥΤΙΑΣ ΚΑΙ ΜΕΤΑΓΧΕΙΡΙΤΙΚΟΥ ΕΜΕΤΟΥ - ΚΛΙΜΑΚΑ APFEL

ΚΛΙΜΑΚΑ APFEL

ΠΡΟΦΥΛΑΞΗ ΜΕΤΕΓΧΕΙΡΗΤΙΚΗΣ ΝΑΥΤΙΑΣ ΚΑΙ ΕΜΕΤΟΥ

ΜΟΝΤΕΛΟ APFEL ΓΙΑ ΔΙΑΣΤΡΩΜΑΤΩΣΗ ΤΟΥ ΚΙΝΔΥΝΟΥ

ΠΑΡΑΓΟΝΤΕΣ ΚΙΝΔΥΝΟΥ	ΒΑΘΜΟΣ	ΚΙΝΔΥΝΟΣ
Γυναίκες	1	Βάση: 10%
Μη καπνιστές	1	1 βαθμός:20%
Ιστορικό μετεγχειρητικής ναυτίας και εμέτου ή/και ναυτίας κίνησης	1	2 βαθμοί: 40%
Μετεγχειρητική χορήγηση οπιοειδών	1	3 βαθμοί:60%
		4 βαθμοί:80%
Χαμηλού κινδύνου (0-1 βαθμός, 10-20%), μεσαίου κινδύνου (2 βαθμοί, 40%), Υψηλού κινδύνου (3-4 βαθμοί, 60-80%)		

10.2. ΠΡΟΕΓΧΕΙΡΗΤΙΚΗ ΑΝΤΙΜΕΤΩΠΙΣΗ ΑΝΑΙΜΙΚΩΝ ΑΣΘΕΝΩΝ

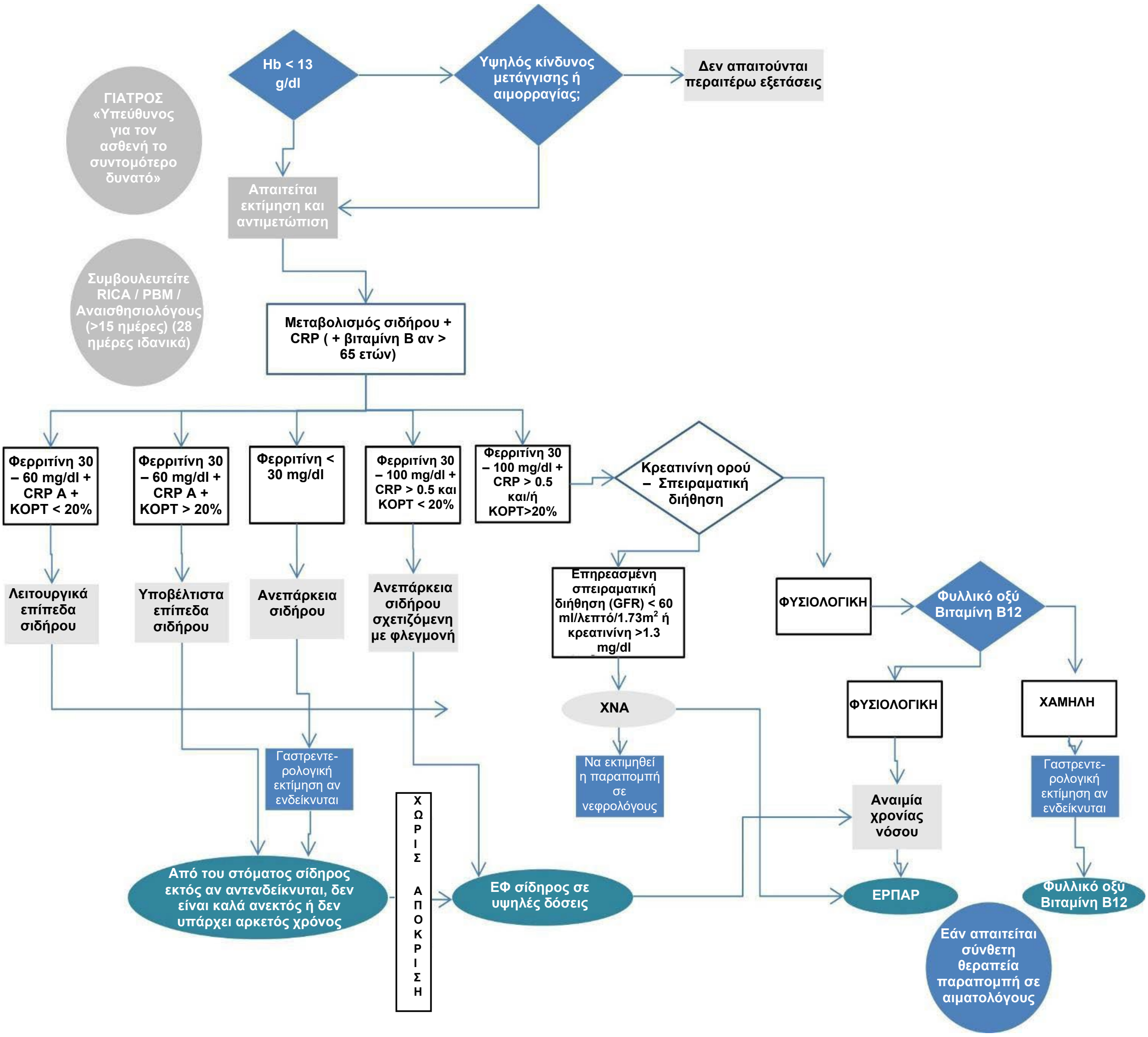

Α: αρνητική

ΕΡΠΑΡ: Ερυθροποιητικοί παράγοντες

ΕΦ: Ενδοφλέβια

ΚΟΡΤ: Κορεσμός Τρανσφερρίνης

ΧΝΑ: Χρόνια Νεφρική Ανεπάρκεια

Hb: Αιμοσφαιρίνη

CRP: C αντιδρώσα πρωτεΐνη (C Reactive Protein)

10.3. ΑΛΓΟΡΙΘΜΟΣ ΔΙΑΤΡΟΦΙΚΟΥ ΕΛΕΓΧΟΥ

Αξιολόγηση Κινδύνου

Κίνδυνος υποθρεψίας

- χρησιμοποιήστε επικυρωμένα εργαλεία ελέγχου

Διαγνωστική αξιολόγηση

Κριτήρια αξιολόγησης

- **Φαινοτυπικά**
  - Ακούσια απώλεια βάρους
  - Χαμηλό BMI
  - Μυϊκή Ατροφία
- **Αιτιολογικά**
  - Μειωμένη πρόσληψη ή αφομοίωση της τροφής
  - Επιβάρυνση από την νόσο / φλεγμονώδης διαδικασία

Διάγνωση

Παρουσία κριτηρίων για την διάγνωση της υποθρεψίας

- Τουλάχιστον 1 φαινοτυπικό και 1 αιτιολογικό κριτήρια

10.4. ΠΡΟΕΓΧΕΙΡΗΤΙΚΗ ΔΙΑΧΕΙΡΙΣΗ

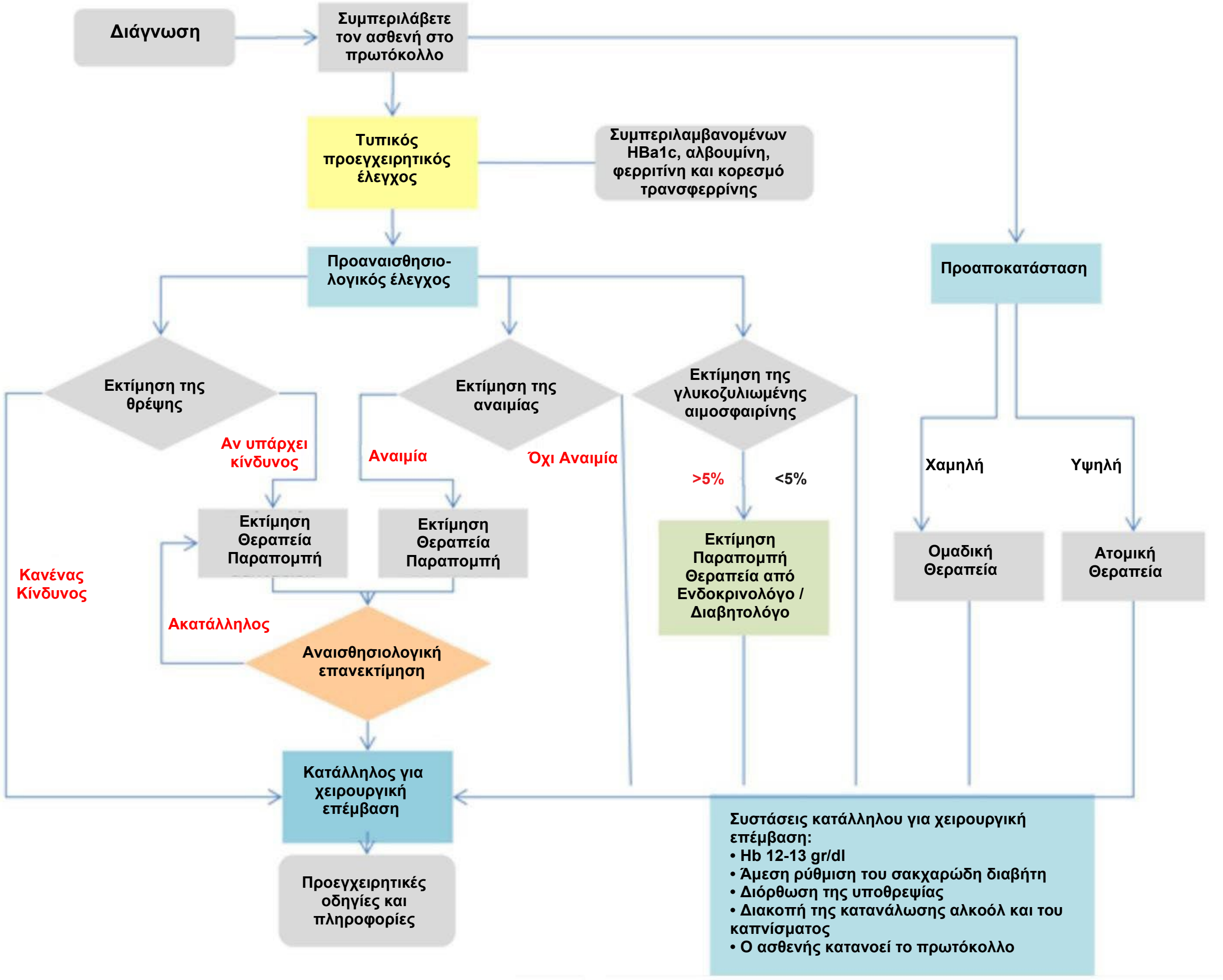

Hb: αιμοσφαιρίνη

Hba1c: γλυκοζυλιωμένη αιμοσφαιρίνη

10.5 ΠΛΗΡΟΦΟΡΙΕΣ ΓΙΑ ΤΟΝ ΑΣΘΕΝΗ

Εντατικοποίηση της Ανάρρωσης για τη Βέλτιστη Φροντίδα στην Χειρουργική Ενήλικων - «Recovery Intensification for optimal Care in Adults surgery - RICA»

1. Εισαγωγή
2. Προετοιμασία στο σπίτι / Πριν την εισαγωγή
3. Κατά τη διάρκεια της νοσηλείας σας στο νοσοκομείο
4. Εξιτήριο

ΕΙΣΑΓΩΓΗ

Αυτός το κλινικό πρωτόκολλο Εντατικοποίησης της Ανάρρωσης για τη Βέλτιστη Φροντίδα στην Χειρουργική Ενήλικων, που ονομάζεται "RICA", στο οποίο συμμετέχετε μέσω της χειρουργικής σας επέμβασης, διαφέρει από την παραδοσιακή θεραπεία. Αποτελείται από την εφαρμογή μιας σειράς μέτρων για την ελαχιστοποίηση των επιπτώσεων και των επιδράσεων που συνεπάγονται οποιασδήποτε χειρουργικής επέμβασης, μειώνει πιθανές επιπλοκές, επιταχύνει την ανάρρωση και μπορεί ακόμη και να μειώσει τη νοσηλεία στο νοσοκομείο.

Η ενεργός συνεργασία σας ως ασθενής αλλά και των μελών της οικογένειάς σας ή των φροντιστών σας, καθώς και η ολοκλήρωση όλων των βημάτων του, είναι απαραίτητα για τη σωστή λειτουργία και επιτυχία αυτού του προγράμματος.

Υπάρχουν τρία βασικά στάδια:

1. *Προετοιμασία πριν από την εισαγωγή.* Από την στιγμή που η ανάγκη για χειρουργική επέμβαση αποφασίζεται με το γιατρό σας.
2. *Κατά τη διάρκεια της νοσηλείας σας στο νοσοκομείο.*
3. *Οδηγίες κατά το εξιτήριο.*

Η ομάδα των επαγγελματιών που θα σας βοηθήσουν σε αυτό το κλινικό πρωτόκολλο είναι εκπαιδευμένη να απαντά σε όλες τις αμφιβολίες σας και να σας καθοδηγεί στην ανάπτυξη κάθε σταδίου του προγράμματος.

ΠΡΟΕΤΟΙΜΑΣΙΑ ΓΙΑ ΤΗΝ ΕΙΣΑΓΩΓΗ

Η εκ των προτέρων προετοιμασία του ασθενούς είναι απαραίτητη και διασφαλίζει ότι ο ασθενής βρίσκεται στην καλύτερη δυνατή κατάσταση, εντοπίζοντας τους ατομικούς κινδύνους κατά την προεγχειρητική περίοδο.

Θα υποβληθείτε σε χειρουργική, αναισθησιολογική και νοσηλευτική συμβουλευτική ώστε να λάβετε όλες τις απαραίτητες πληροφορίες σχετικά με τις λεπτομέρειες της επέμβασής σας και τις διεργασίες που απαιτούν την εκ των προτέρων συνεργασία σας σε αυτό το πρόγραμμα.

Δεδομένου ότι έχετε πάρει την απόφαση να υποβληθείτε σε χειρουργική επέμβαση, πρέπει να δεσμευτείτε ότι θα αποφύγετε τις τοξίνες όπως το αλκοόλ και το κάπνισμα. Είναι σημαντικό να καταλάβετε ότι όλη η προσπάθεια που μπορείτε να καταβάλετε για την ελάττωση αυτών των συνηθειών θα συμβάλει άμεσα σε μείωση πιθανών αναπνευστικών επιπλοκών που μπορεί να προκύψουν κατά τη χειρουργική διαδικασία.

Η χειρουργική επέμβαση μπορεί να αυξήσει τον κίνδυνο αναπνευστικών επιπλοκών. Για να τις αποτρέψετε, ο/η νοσηλευτής/τρια ή φυσικοθεραπευτής/τρια σας θα σας διδάξει πώς να προετοιμάζετε τους αναπνευστικούς μύες σας. Επιπλέον, θα σας διδάξουν πώς να χρησιμοποιείτε το αναπνευστικό σπιρόμετρο – εξασκητή αναπνοών (incentive spirometer), για να σας βοηθήσουν να πραγματοποιήσετε αναπνευστικές ασκήσεις κατά τις ημέρες πριν από την επέμβαση.

Προεγχειρητική διατροφή. Κατά τη διάρκεια της χειρουργικής επέμβασης, θα απαιτηθεί υψηλή ενεργειακή δαπάνη και θα είναι πολύ σημαντικό να υπάρχει μια επαρκής διατροφική κατάσταση για την προαγωγή της επούλωσης και της άμυνας του οργανισμού από λοιμώξεις.

Για να επιτευχθεί καλύτερη προεγχειρητική διατροφική κατάσταση, συνιστάται μια διατροφή πλούσια σε πρωτεΐνες συνδυαστικά με τη σωστή ενυδάτωση, για τουλάχιστον επτά έως δέκα ημέρες πριν από την επέμβαση.

Το βράδυ πριν από την επέμβαση, μπορείτε να καταναλώσετε στερεά τροφή έως και 6 ώρες πριν από τη χειρουργική επέμβαση και διαυγή υγρά (χαμομήλι, χυμό ή διάλυμα ζάχαρης) έως και 2 ώρες πριν από την επέμβαση.

Δεν θα μπορείτε να φάτε ή να πιείτε τίποτα 2 ώρες πριν από τη χειρουργική επέμβαση

Δεν πρέπει να πίνετε αλκοολούχα ποτά. Το αλκοόλ σχετίζεται με μετεγχειρητικές επιπλοκές.

Άσκηση πριν από τη χειρουργική επέμβαση. Η πραγματοποίηση μέτριας έντασης άσκησης πριν από την εισαγωγή θα συμβάλει ευνοϊκά στη μετέπειτα ανάρρωσή σας. Ο/η νοσηλευτής/τρια ή φυσικοθεραπευτής/τρια σας θα σας συμβουλεύσει σχετικά με το είδος της δραστηριότητας που μπορείτε να ακολουθήσετε ανάλογα με τη φυσική σας κατάσταση.

ΚΑΤΑ ΤΗΝ ΝΟΣΗΛΕΙΑ ΣΑΣ ΣΤΟ ΝΟΣΟΚΟΜΕΙΟ

Μετά το χειρουργείο, η ομάδα των επαγγελματιών που θα σας φροντίσουν θα σας υποδείξει ποια θα είναι τα βήματα της ανάρρωσής σας σε καθημερινή βάση. Θυμηθείτε ότι η συνεργασία και η συμμετοχή σας είναι το κλειδί για τη σωστή πρόοδο σας. Μην διστάσετε να κάνετε οποιεσδήποτε ερωτήσεις έχετε ή να ενημερώσετε το προσωπικό για τις ενοχλήσεις που αισθάνεστε.

Για να αποτρέψουμε τις πιθανές επιπλοκές που σχετίζονται με οποιαδήποτε χειρουργική επέμβαση, θα εργαστούμε σε τρεις κύριους τομείς:

1. *Πρώιμη κινητοποίηση*
2. *Πρώιμη σίτιση από του στόματος*
3. *Ασκήσεις αναπνευστικής φυσιοθεραπείας*

ΠΡΩΙΜΗ ΚΙΝΗΤΟΠΟΙΗΣΗ

Όπως εκτιμάται σε αυτό το πρόγραμμα, όσο πιο γρήγορα μπορείτε να κινητοποιηθείτε, τόσο καλύτερα αποτελέσματα θα έχετε, οπότε θα σας ζητήσουμε να σηκωθείτε μετά τη χειρουργική επέμβαση και να περπατήσετε τριγύρω.

Η ιδανική σας εξέλιξη θα ήταν η ακόλουθη:

Την ημέρα της επέμβασης, το νοσηλευτικό προσωπικό θα σας βοηθήσει να σηκωθείτε από το κρεβάτι για να καθίσετε στην καρέκλα σας. Θα πρέπει να προσπαθήσετε να καθίσετε έξω από το κρεβάτι για έως και δύο ώρες. Γνωρίζουμε ότι αυτή είναι μια μεγάλη προσπάθεια και μπορεί να φαίνεται δύσκολο, αλλά θα δείτε πώς η ανάρρωσή σας θα είναι ταχύτερη. Για παράδειγμα: Η χειρουργική επέμβαση παραλύει το έντερο για μεταβλητό

χρονικό διάστημα που μπορεί να συντομευθεί αν σηκωθείτε και περπατήσετε μετά την επέμβαση αλλά επιμηκύνεται εάν ξαπλώσετε.

Την επομένη ημέρα της επέμβασης, θα μπορείτε να κάθεστε στην καρέκλα ανά διαστήματα έως και έξι ώρες, επιπρόσθετα με τη βάδιση μικρών αποστάσεων, περίπου τέσσερα σετ των 60 μέτρων.

Διαδοχικά τις επόμενες μέρες θα συνεχίσετε να περπατάτε και να επιχειρείτε μια σταθερή πρόοδο.

ΠΡΩΙΜΗ ΣΙΤΙΣΗ ΑΠΟ ΤΟ ΣΤΟΜΑ

Σε αυτό το πρόγραμμα εκτιμούμε πολύ τη διατροφή, έτσι ώστε να μπορείτε να ανεχτείτε το φαγητό το συντομότερο δυνατό, με τον ρυθμό που το χρειάζεστε.

Την ίδια μέρα της χειρουργικής επέμβασης, συνιστάται να αρχίσετε να πίνετε το συντομότερο δυνατόν. Αυτό θα γίνει προοδευτικά ξεκινώντας με μικρές ποσότητες και συνεχίζοντας με άλλα είδη εύπεπτων τροφών υπό την προϋπόθεση ότι υπάρχει καλή ανοχή.

Την επομένη της επέμβασης θα αυξήσετε την πρόσληψη υγρών στο 1,5 λίτρο. Μην πίνετε ανθρακούχα ποτά.

Με την προϋπόθεση ότι το ανέχεστε αυτό καλά, τις επόμενες ημέρες, θα προχωρήσετε σε μια πιο στερεή δίαιτα. Θα πρέπει να συνεχίστε να πίνετε υγρά σε τακτική βάση.

ΑΣΚΗΣΕΙΣ ΑΝΑΠΝΕΥΣΤΙΚΗΣ ΦΥΣΙΟΘΕΡΑΠΕΙΑΣ

Σε όλες τις χειρουργικές επεμβάσεις ο κίνδυνος αναπνευστικών επιπλοκών αυξάνεται λόγω ανάπαυσης στο κρεβάτι, δυσφορίας στο σημείο της τομής και άλλων παραγόντων. Ο κίνδυνος μπορεί να προληφθεί μέσω ασκήσεων κινητοποίησης του θώρακα, τις οποίες θα εκτελέσετε με το αναπνευστικό σπιρόμετρο – εξασκητή αναπνοών

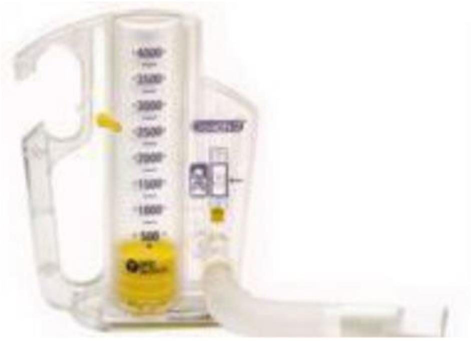

Κάνοντας αυτές τις ασκήσεις θα:

• *Αυξήσετε τον αερισμό των πνευμόνων με σκοπό την πρόληψη των λοιμώξεων του αναπνευστικού*

• *Αυξήσετε τη δύναμη των αναπνευστικών μυών*

• *Αποτρέψετε τη συσσώρευση αναπνευστικών εκκρίσεων*

Περίπου 4-6 ώρες μετά την επέμβαση, μπορείτε να αρχίσετε να χρησιμοποιείτε τη συσκευή. Η συχνότητα χρήσης θα είναι κάθε 2 ώρες για 10 λεπτά κάθε φορά.

ΟΔΗΓΙΕΣ ΚΑΤΑ ΤΟ ΕΞΙΤΗΡΙΟ

Το υψηλό επίπεδο προγραμματισμού πίσω από το κλινικό πρωτόκολλο της Εντατικοποίηση της Ανάρρωσης για τη Βέλτιστη Φροντίδα στην Χειρουργική Ενήλικων σημαίνει ότι πρέπει να προετοιμαστεί όλη η πρακτική υποστήριξη που θα χρειαστείτε στο σπίτι.

Εάν έχετε αμφιβολίες σχετικά με το πως πρέπει να το διαχειριστείτε, συμβουλευτείτε το προσωπικό υγειονομικής περίθαλψης.

Το πλάνο που ετοιμάστηκε για εσάς θα αναθεωρηθεί και θα επικυρωθεί από τους γιατρούς και τους νοσηλευτές που είναι υπεύθυνοι για το εξιτήριό σας από το νοσοκομείο.

Ενδεχομένως, η πιθανή ημερομηνία εξιτηρίου θα σας γνωστοποιηθεί εκ των προτέρων από το γιατρό σας. Αυτό σας διευκολύνει δίνοντάς σας αρκετό χρόνο να έχετε όλα όσα χρειάζεστε έτοιμα για να πάτε στο σπίτι ή στο κέντρο αποκατάστασης εάν απαιτείται.

Το εξιτήριο από το νοσοκομείο βασίζεται σε συγκεκριμένα κριτήρια και στόχους. Όταν τα πετύχετε, θα πάρετε εξιτήριο.

Αυτά τα κριτήρια είναι:

• Αποτελεσματικός έλεγχος του πόνου με από του στόματος αναλγητικά.

• Καλή ανοχή από του στόματος σε υγρά και διατροφή, χωρίς ναυτία ή έμετο.

• Αυτονομία στην κινητοποίηση.

Εάν χρειάζεστε περισσότερες πληροφορίες, μη διστάσετε να ρωτήσετε το γιατρό σας ή τη νοσηλεύτρια του τμήματος.

10.6. ΕΡΩΤΗΜΑΤΟΛΟΓΙΟ ΙΚΑΝΟΠΟΙΗΣΗΣ ΑΣΘΕΝΟΥΣ

ΕΡΩΤΗΜΑΤΟΛΟΓΙΟ ΙΚΑΝΟΠΟΙΗΣΗΣ ΑΣΘΕΝΟΥΣ

(ΕΝΤΑΤΙΚΟΠΟΙΗΣΗ ΤΗΣ ΑΝΑΡΡΩΣΗΣ ΓΙΑ ΤΗ ΒΕΛΤΙΣΤΗ ΦΡΟΝΤΙΔΑ ΣΤΗΝ ΧΕΙΡΟΥΡΓΙΚΗ ΕΝΗΛΙΚΩΝ)

Αγαπητέ/ή ασθενή:

Θα θέλαμε να απαντήσετε σε αυτό το ανώνυμο ερωτηματολόγιο για να μάθουμε τη γνώμη σας για την παρεχόμενη φροντίδα.

Σας ευχαριστούμε για το ενδιαφέρον και την προσοχή σας να συμφωνήσετε να απαντήσετε σε αυτές τις ερωτήσεις βοηθώντας μας να βελτιώσουμε τη δουλειά μας.

Η ιατρική και νοσηλευτική ομάδα

Γενικά Δεδομένα

Ηλικία: **Φύλο:** Άρρεν □ Θήλυ □

Εκπαίδευση: Καμία □ Πρωτοβάθμια □ Δευτεροβάθμια □ Μέση □ Ανώτερη □

Πληροφορίες πριν την επέμβαση

Πώς θα αξιολογούσατε τις πληροφορίες που σας έδωσε ο/η ΧΕΙΡΟΥΡΓΟΣ πριν την επέμβαση;

Πολύ καλές □ Καλές □ Μέτριες □ Κακές □ Πολύ κακές □

Πώς θα αξιολογούσατε τις πληροφορίες που σας έδωσε ο/η ΑΝΑΙΣΘΗΣΙΟΛΟΓΟΣ πριν την επέμβαση;

Πολύ καλές □ Καλές □ Μέτριες □ Κακές □ Πολύ κακές □

Πώς θα αξιολογούσατε τις πληροφορίες που σας έδωσε ο/η ΝΟΣΗΛΕΥΤΗΣ/ΤΡΙΑ πριν την επέμβαση;

Πολύ καλές □ Καλές □ Μέτριες □ Κακές □ Πολύ κακές □

Εγκαταστάσεις και εξοπλισμός

Η οπτική εμφάνιση του χειρουργείου ήταν:

Πολύ καλή □ Καλή □ Μέτρια □ Κακή □ Πολύ κακή □

Ο θάλαμος νοσηλείας στην κλινική ήταν:

Μονόκλινος □ Δίκλινος □ Άλλο □

Ο θάλαμος νοσηλείας σας φάνηκε:

Πολύ άνετος □ Αρκετά άνετος □ Μέτριος □ Όχι πολύ άνετος □ Καθόλου Άνετος □

Πόνος

Ποιο ήταν το μέγιστο επίπεδο πόνου τις πρώτες ώρες μετά την επέμβαση;
(0 = χωρίς πόνο ⇨ 10 = αφόρητος πόνος)

0	1	2	3	4	5	6	7	8	9	10

Ποιο ήταν το μέγιστο επίπεδο πόνου στον θάλαμο μετά την επέμβαση;
(0 = χωρίς πόνο ⇨ 10 = αφόρητος πόνος)

0	1	2	3	4	5	6	7	8	9	10

Μετεγχειρητική σίτιση

Μετά την επέμβαση, παρουσιάσατε ναυτία ή έμετο; ΝΑΙ □ ΟΧΙ □

Όταν σας είπαν ότι έπρεπε να φάτε και να πιείτε, σκεφτήκατε ότι ήταν;
Πολύ νωρίς □ Νωρίς □ Στην ώρα του □ Αργά □ Πολύ αργά □

Πρώιμη Κινητοποίηση

Όταν σας είπαν ότι έπρεπε να σηκωθείτε όρθιος, σκεφτήκατε ότι ήταν;
Πολύ νωρίς □ Νωρίς □ Στην ώρα του □ Αργά □

Όταν σας είπαν ότι έπρεπε να περπατήσετε, σκεφτήκατε ότι ήταν;
Πολύ νωρίς □ Νωρίς □ Στην ώρα του □ Αργά □

Κατά το εξιτήριο

Πώς θα αξιολογούσατε τις πληροφορίες και τις οδηγίες που λάβατε από τον/την ΧΕΙΡΟΥΡΓΟ κατά το εξιτήριο;

Πολύ καλές □ Καλές □ Μέτριες □ Κακές □ Πολύ κακές □ Δεν ενημερώθηκα □

Πώς θα αξιολογούσατε τις πληροφορίες και τις οδηγίες που λάβατε από τον/την ΝΟΣΗΛΕΥΤΗ/ΤΡΙΑ κατά το εξιτήριο;

Πολύ καλές □ Καλές □ Μέτριες □ Κακές □ Πολύ κακές □ Δεν ενημερώθηκα □

Όταν ήσασταν σπίτι, χρειάστηκε να καλέσετε τον αριθμό τηλεφώνου που σας δόθηκε;

ΝΑΙ □ ΟΧΙ □ Δεν μου έδωσαν αριθμό τηλεφώνου □

Παρασχεθείσα Φροντίδα

Πώς θα αξιολογούσατε τη φροντίδα που παρείχε ο/η ΧΕΙΡΟΥΡΓΟΣ;

Πολύ καλή □ Καλή □ Μέτρια □ Κακή □ Πολύ κακή □

Πώς θα αξιολογούσατε τη φροντίδα που παρείχε ο/η ΑΝΑΙΣΘΗΣΙΟΛΟΓΟΣ;

Πολύ καλή □ Καλή □ Μέτρια □ Κακή □ Πολύ κακή □

Πώς θα αξιολογούσατε τη φροντίδα που παρείχε ο/η ΝΟΣΗΛΕΥΤΗΣ/ΤΡΙΑ;

Πολύ καλή □ Καλή □ Μέτρια □ Κακή □ Πολύ κακή □

Πώς θα αξιολογούσατε τη φροντίδα που παρείχε το ΥΠΟΛΟΙΠΟ ΠΡΟΣΩΠΙΚΟ;

Πολύ καλή □ Καλή □ Μέτρια □ Κακή □ Πολύ κακή □

Ικανότητα, επαγγελματικός συντονισμός και έκβαση

Κατά τη γνώμη σας, το επίπεδο της επαγγελματικής ικανότητας του/της ΧΕΙΡΟΥΡΓΟΥ σας φάνηκε;

Πολύ υψηλό □ Υψηλό □ Μέτριο □ Χαμηλό □ Πολύ χαμηλό □

Κατά τη γνώμη σας, το επίπεδο της επαγγελματικής ικανότητας του/της ΑΝΑΙΣΘΗΣΙΟΛΟΓΟΥ σας φάνηκε;

Πολύ υψηλό □ Υψηλό □ Μέτριο □ Χαμηλό □ Πολύ χαμηλό

Κατά τη γνώμη σας, το επίπεδο της επαγγελματικής ικανότητας του/της ΝΟΣΗΛΕΥΤΗ/ΤΡΙΑΣ σας φάνηκε;

Πολύ υψηλό □ Υψηλό □ Μέτριο □ Χαμηλό □ Πολύ χαμηλό

Κατά τη γνώμη σας, το επίπεδο της επαγγελματικής ικανότητας του ΥΠΟΛΟΙΠΟΥ ΠΡΟΣΩΠΙΚΟΥ σας φάνηκε;

Πολύ υψηλό □ Υψηλό □ Μέτριο □ Χαμηλό □ Πολύ χαμηλό

Όσον αφορά τον συντονισμό μεταξύ των επαγγελματιών υγείας, νομίσατε ότι ήταν;

Πολύ συντονισμένοι □ Αρκετά συντονισμένοι □ Μέτρια □ Λίγο συντονισμένοι □ Μη συντονισμένοι

Πώς θα αξιολογούσατε την έκβαση της επέμβασης σας;

Πολύ καλή □ Καλή □ Μέτρια □ Κακή □ Πολύ κακή □

Εάν έπρεπε να υποβληθείτε ξανά σε χειρουργική επέμβαση, θα χρησιμοποιούσατε το ίδιο πρωτόκολλο; ΝΑΙ □ ΟΧΙ □

Συνολική Ικανοποίηση
**Γενικά, πόσο ικανοποιημένος είσαι από την όλη διαδικασία;**<br>Πολύ ικανοποιημένος □ Αρκετά ικανοποιημένος □ Ούτε ικανοποιημένος ούτε δυσαρεστημένος □ Αρκετά δυσαρεστημένος □ Πολύ δυσαρεστημένος □
**Το πιο θετικό για εσάς ήταν:**
**Το πιο αρνητικό για εσάς ήταν:**
**Παρακαλούμε, αναφέρετε τις βελτιώσεις που θα μπορούσαν να συμπεριληφθούν σε αυτό το πρωτόκολλο:**
**Σχόλια - Παρατηρήσεις:**

Ευχαριστούμε για τη συνεργασία σας

10.7. ΣΥΝΤΟΜΟΓΡΑΦΙΕΣ

AKI	Acute Kidney Injury (οξεία νεφρική βλάβη)
ARM	Alveolar Recruitment Maneuvers (χειρισμοί στρατολόγησης των κυψελίδων)
ASA	American Society of Anesthesiology (Αμερικάνικη Αναισθησιολογική Εταιρία)
BIS	Bispectral Index (διφασματικός δείκτης)
BMI	Body Mass Index (δείκτης μάζας ΄σώματος)
CPAP	Continuous Positive Airway Pressure (συνεχόμενη θετική πίεση αεραγωγών)
CRP	C reactive protein (C αντιδρώσα πρωτεΐνη)
CVP	Central Venous Pressure (κεντρική φλεβική πίεση)
DSM-IV-TR	Diagnostic and Statistical Manual of Mental Disorders, fourth edition, text revision (διαγνωστικό και στατιστικό εγχειρίδιο ψυχικών διαταραχών, τέταρτη έκδοση, αναθεώρηση κειμένου)
ERAS	Enhanced Recovery After Surgery (βελτιστοποίηση της μετεγχειρητικής ανάρρωσης)
ESPEN	European Society of Parenteral and Enteral Nutrition (Ευρωπαϊκή Εταιρία Εντερικής και Παρεντερικής Διατροφής)
EUPEMEN	European Perioperative Medical Networking (Ευρωπαϊκή Περιεγχειρητική Ιατρική Δίκτυωση)
FiO_2	Inspired Oxygen Fraction (εισπνεόμενο κλάσμα οξυγόνου)
GERM	Grupo Español de Rehabilitación Multimodal (Ισπανική Ομάδας Πολυπαραγοντικής Αποκατάστασης)
GLIM	Global Leadership Initiative on Malnutrition (Παγκόσμια Ηγετική Πρωτοβουλία για την Υποθρεψία)
GRADE	Grading of Recommendations, Assessment, Development and Evaluation (βαθμολόγηση συστάσεων, αξιολόγηση, ανάπτυξη και εκτίμηση)

Hb	Αιμοσφαιρίνη (Hemoglobin)
HbA1c	Γλυκοζηλιωμένη αιμοσφαιρίνη
ICDSC	Intensive Care Delirium Screening Checklist (Λίστα ελέγχου παραληρήματος εντατικής θεραπείας)
IL	Ιντερλευκίνη
IRP	Intensified Recovery Protocols (πρωτόκολλα εντατικοποιημένης ανάρρωσης)
KDIGO	Kidney Disease: Improving Global Outcomes (νεφρική νόσος: βελτίωση παγκόσμιων αποτελεσμάτων)
LMWH	Low Molecular Weight Heparin (ηπαρίνη χαμηλού μοριακού βάρους)
MIS	Minimal Invasive Surgery (ελάχιστα επεμβατική χειρουργική)
NICE	National Institute for Health and Care Excellence (Εθνικό Ινστιτούτο Αριστείας στην Υγεία και την Φροντίδα)
NMB	Neuromuscular Blockade (νευρομυϊκός αποκλεισμός)
NMDA	N-μεθυλο-D-ασπαρτικό οξύ
NPWT	Negative Pressure Wound Therapy (θεραπεία τραύματος αρνητικής πίεσης)
OFA	Opioid Free Anesthesia (αναισθησία χωρίς οπιοειδή)
PBM	Patient Blood Management (διαχείρηση αίματος ασθενών)
PEEP	Positive end-expiratory pressure (Θετική τελοεκπνευστική πίεση)
PONV	Postoperative Nausea and Vomiting (μετεγχειρητική ναυτία και έμετος)
PTC	Post Tetanic Count (μετατετανική μέτρηση)
RCT	Randomized Control Trial (τυχαιοποιημένη ελεγχόμενη μελέτη)
rHuEPO	Recombinant human erythropoietin (ανασυνδυασμένη ανθρώπινη ερυθροποιητίνη)
RICA	Recovery Intensification for Optimal Care in Adult’s Surgery (Εντατικοποίηση της Ανάρρωσης για τη Βέλτιστη Φρόντιδα στην Χειρουργική Ενηλίκων)

SIGN	Scottish Intercollegiate Guidelines Network (Σκωτσέζικο διακολεγιακό δίκτυο κατευθυντήριων οδηγιών)
SSI	Surgical Site Infection (λοίμωξη χειρουργικού πεδίου)
SV	Stroke Volume (όγκος παλμού)
SVV	Stroke Volume Variation (διακύμανση όγκου παλμού)
TAP	Transversus Abdominis Plane (πλάνο εγκαρσίου κοιλιακού)
TOF	Train of Four (τρένο των τεσσάρων)
TV	Tidal Volume (αποπνεόμενος όγκος)
UFH	Unfractionated Heparin (μη κλασματοποιημένη ηπαρίνη)
VAC	Vacuum Assisted Closure (υποβοηθούμενη σύγκλειση με κενό)
VATS	Video-assisted thoracoscopic surgery (Υποβοηθούμενη με βίντεο θωρακοσκοπική χειρουργική)

ΔΜΣ	Δείκτης Μάζας Σώματος
ΕΡΠΑΡ	Ερυθροποιητικοί παράγοντες
ΕΦ	Ενδοφλέβια
ΕΣΚ	Εξωσωματική κυκλοφορία
ΗΕΓ	Ηλεκτροεγκεφαλογράφημα
ΗΚΓ	Ηλεκτροκαρδιογράφημα
ΗΧΜΒ	Ηπαρίνη Χαμηλού Μοριακού Βάρους
ΚΜ	Κολπική Μαρμαρυγή
ΚΟΡΤ	Κορεσμός Τρανσφερρίνης
ΚΦΚ	Κεντρικός Φλεβικός Καθετήρας
ΚΦΠ	Κεντρική Φλεβική Πίεση
ΛΧΠ	Λοίμωξη Χειρουργικού Πεδίου
ΜΕΘ	Μονάδα Εντατικής Θεραπείας
ΜΚΗ	Μη κλασματοποιημένη ηπαρίνη
ΜΣΑΦ	Μη Στεροειδή Αντιφλεγμονώδη Φάρμακα
ΠΟΥ	Παγκόσμιος Οργανισμός Υγείας
ΣΔ	Σακχαρώδης Διαβήτης
ΣΕ	Συμπυκνωμένα Ερυθρά
ΦΘΕ	Φλεβική Θρομβοεμβολή

ΧΝΑ	Χρόνια Νεφρική Ανεπάρκεια

MINISTERIO
DE SANIDAD
