# Supplementary material for: Advancing abdominal surgery recovery implementation: a unified framework for intensified recovery protocols by the EUropean PErioperative MEdical Networking collaborative
Source: Front Surg. 2026 May 18;13:1827678. doi: 10.3389/fsurg.2026.1827678 (PMC13223102; doi:10.3389/fsurg.2026.1827678)
Supplement: Supplementary file 17 [file Datasheet8.pdf]

CLINICAL PATHWAY RICA

Recovery Intensification for optimal Care in Adult's surgery

MINISTERIO DE SANIDAD

Red Española de Agencias de Evaluación de Tecnologías y Prestaciones del Sistema Nacional de Salud

IACS Instituto Aragonés de Ciencias de la Salud

Clinical Pathway

RICA

Recovery Intensification for optimal Care in Adult's Surgery (RICA)

Work Group. Recovery Intensification for optimal Care in Adult's surgery (RICA).
Ellaboration date: 28/12/2020
Edition: 2021
Edited by: Ministerio de Sanidad
Edited by: Instituto Aragonés de Ciencias de la Salud
Edited by: Grupo Español de Rehabilitación Multimodal (GERM)
NIPO: 133-21-077-0
Layout: ARPIrelieve, S. A.

Clinical Pathway

Recovery Intensification for optimal care in Adult's surgery

(RICA)

Translation funded by:

EUPEMEN European Perioperative Medical Networking

Co-funded by the Erasmus+ Programme of the European Union

Cátedra GERM Medicina Perioperatoria Universidad Zaragoza

Authors & collaborators

COORDINATOR

José Manuel Ramírez Rodríguez Grupo Español de Rehabilitación Multimodal (GERM). Departamento de Cirugía. Hospital Clínico Universitario "Lozano Blesa". Zaragoza.

METHODOLOGIST

Pedro M. Ruiz López — Grupo Español de Rehabilitación Multimodal (GERM). Hospital Universitario 12 de Octubre. Madrid

ELLABORATING GROUP

Alfredo Abad Gurumeta — Sociedad Española de Anestesiología y Reanimación (SEDAR). Hospital Universitario Infanta Leonor. Madrid.

Antonio Arroyo Sebastián — Asociación Española de Cirujanos (AEC). Grupo de Trabajo de Rehabilitación Multimodal. Hospital Universitario Elche. Universidad Miguel Hernández. Elche. Alicante.

Marcos Bruna Esteban — Asociación Española de Cirujanos. Sección de Cirugía Esofagogástrica. Hospital Universitario y Politécnico "La Fe". Valencia.

Alberto Cabañero Sanchez — Sociedad Española de Cirugía Torácica (SECT). Hospital Universitario Ramón y cajal. Madrid.

José María Calvo Vecino — Grupo Español de Rehabilitación Multimodal (GERM). Hospital Universitario de Salamanca.

Emilio Del Valle Hernández — Asociación Española de Coloproctología (AECP).

Hospital General Universitario Gregorio Marañón. Madrid.

José Antonio García Erce — Sociedad Española de Hematología y Hemoterapia (SEHH). Banco de Sangre y Tejidos de Navarra. Pamplona.

Alfredo García Fernández — Grupo Español de Rehabilitación Multimodal (GERM). Grupo de trabajo ORL. Hospital Universitario 12 de octubre. Madrid

Manuel García Toro	Asociación Española de Enfermería Quirúrgica (AEEQ). Hospital Tierra de Barros. Almendralejo. Badajoz.
Francisco Guillén Grima	Sociedad Española de Medicina Preventiva, Salud Pública e Higiene (SEMPSPH). Clínica Universidad de Navarra.
Ana María Godoy Ramírez	Sociedad Española de Rehabilitación y Medicina Física (SERMEF). Hospital Regional Universitario. Málaga.
Carmelo Loinaz Segurola	Sociedad Española de Nutrición Clínica y Metabolismo (SENPE). Hospital Universitario 12 de octubre. Madrid.
Natividad Marcellán Raldúa	Asociación Española de Enfermería Quirúrgica (AEEQ). Hospital Universitario "Miguel Servet". Zaragoza.
Juan Antonio Margarit Calabuig	Sociedad Española de Cirugia Cardiovascular y Endovascular (SECCE). Hospital Universitario de la Ribera. Alzira. Valencia
Manuel Muñoz Gómez	Sociedad Española de Transfusión Sanguínea y Terapia Celular (SETS). Departamento de Especialidades Quirúrgicas, Bioquímica e Inmunología. Facultad de Medicina. Universidad de Málaga.
Carmen Gloria Nogueiras Quinta	Sociedad Española de Enfermería en Cirugía (SEECir). Hospital Universitario de Fuenlabrada. Madrid.
Julia Ocón Bretón	Sociedad Española de Endocrinología y Nutrición (SEEN). Hospital Clínico Universitario "Lozano Blesa". Zaragoza.
Cristina Ojeda Thies	Grupo Español de Rehabilitación Multimodal (GERM). Grupo de trabajo en Cirugia Ortopédica y Traumatologia. Hospital Universitario 12 de Octubre. Madrid.
Dolores Pérez del Caz	Sociedad Española de Cirugía Plástica, Reparadora y Estética (SECPRE). Hospital Universitario "La Fe". Valencia.
José Luis Sánchez Iglesias	Sociedad Española de Ginecología y Obstetricia (SEGO). Hospital Universitario Vall d'Hebrón. Barcelona.
Victor Soria Aledo	Programa Cirugía Segura. Hospital Universitario J.M. Morales Meseguer. Murcia
Jorge Subirá Ríos	Asociación Española de Urología (AEU). Hospital Clínico Universitario "Lozano Blesa". Zaragoza.

EXTERNAL REVIEWERS

Joel Jesús Artiles Ivonnet	Sociedad Española de Medicos de Atencion Primaria (SEMERGEN). Centro de Salud Barrio Atlántico. Las Palmas.
José Luis Baquero Úbeda	Foro Español de Pacientes. Madrid.
Pedro Javier Cañones Garzón	Sociedad Española de Médicos Generales y de Familia (SEMG). C.S. Isla de Oza. Madrid.
Carina Escobar Manero	Plataforma de Organizaciones de Pacientes (POP). Madrid.
José María Fernández Cebrián	Sociedad Española de Patologia Digestiva (SEPD). Hospital Universitario Ramón y Cajal. Madrid.
Elena Gómez García	Sociedad Española de Cirugía Oral y Maxilofacial y de Cabeza y Cuello. Hospital Universitario La Paz. Madrid.

Ana Isabel Hijas Gómez	Sociedad Española de Epidemiología (SEE). Instituto de Salud Carlos III. Madrid
Javier Martínez Ubieto	Grupo Español de Rehabilitación Multimodal. Cátedra GERM. Hospital Universitario "Miguel Servet". Zaragoza.
Jesús Puente Alcaraz	Federacion de Asociaciones de Enfermería Familiar y Atención Primaria (FAECAP). Hospital Universitario de Burgos.
Manuel Quintana Díaz	Sociedad Española de Medicina Intensiva, Crítica y Unidades Coronarias (SEMICYUC). Hospital Universitario La Paz. Madrid.
David Rubal Bran	Sociedad Española de Medicina Interna (SEMI). Hospital Universitario Lucus Augusti. Lugo
Julián Carlos Segura Mata	Sociedad Española de Cirugía Ortopédica y Traumatología. Hospital MAZ. Zaragoza
Maria Teresa Valle Vicente	Asociación Española de Cirugía Mayor Ambulatoria (ASECMA). Hospital General Universitario "Gregorio Marañón". Madrid.
Marina Varela Durán	Sociedad Española de Calidad Asistencial (SECA). Área Sanitaria de Pontevedra e O Salnés. Pontevedra.
Arturo Vilches Moraga	Sociedad Española de Geriatría y Gerontología (SEGG). Sociedad Española de Medicina Geriátrica (SEMEG). Salford Royal NHS Foundation Trust. Manchester.
Matilde Zaballos García	Asociación Española de Cirugía Mayor Ambulatoria (ASECMA). Hospital General Universitario "Gregorio Marañón". Madrid.

COLLABORATING GROUP FOR LITERATURE REVIEW

José Luis Balibrea del Castillo	Servicio de Cirugía. Hospital Clinico de Barcelona
Gloria Bermejo Fernández	Servicio Medicina Física y Rehabilitación. Fundación Jiménez Díaz. Madrid.
José Luis Bueno Cabrera	Servicio de Hematología. Hospital Universitario Puerta de Hierro. Madrid
Domingo Bustos García	Servicio de Anestesiología y Reanimación. Complejo Hospitalario Universitario de Salamanca
Rubén Casans Francés	Servicio de Anestesiología y Reanimación. Hospital Infanta Elena, Valdemoro, Madrid
Manuela Elía Guedea	Servicio de Cirugía Gernal y del Aparato Digestivo. Hospital Clínico Universitario "Lozano Blesa". Zaragoza.
Oscar Díaz Cambronero	Servicio de Anestesiología y Reanimación. Hospital Universitario La Fe, Valencia
Esther García González	Servicio de ORL. H. Universitario 12 de octubre. Madrid
Felícitas García Ortún	Servicio Medicina Física y Rehabilitación. Hospital Universitario Mútua de Terrassa. Terrassa. Barcelona.

Ignacio Garutti Martínez	Servicio de Anestesiología y Reanimación. Hospital Universitario "Gregorio Marañón". Madrid.
Alba Gómez Garrido	Servicio Medicina Física y Rehabilitación. Hospital Vall d´Hebron. Barcelona.
Manuel Ángel Gómez Ríos	Servicio de Anestesiología y Reanimación. Complejo Hospitalario Universitario A Coruña.
José Luis Gónzalez Rodríguez	Servicio de Anestesiología y Reanimación. Complejo Hospitalario Universitario de Salamanca.
Manuel Granell Gil	Servicio de Anestesia y Reanimación. Hospital General de Valencia.
Carlos Jericó Alba	Servicio de Medicina Interna. Hospital Sant Joan Despí-Moisès Broggi. Sant Joan Despí. Barcelona.
Teresa Júlvez García	Enfermera quirúrgica. Bloque quirúrgico. Hospital Clínico Universitario Lozano Blesa, Zaragoza.
Javier Longas Vailén	Servicio de Anestesiología y Reanimación. Hospital Clínico Universitario Lozano Blesa, Zaragoza.
Clara Marín Zaldívar	Servicio de Anestesiología y Reanimación. Hospital MAZ. Zaragoza.
Lucrecia Mendoza González	Servicio Medicina Física y Rehabilitación. Hospital de Cabueñes. Gijon.
Nuria Novoa Valentín	Servicio de Cirugía Torácica. Complejo Asistencial Universitario de Salamanca (CAUS); Instituto de Investigación Biomédica de Salamanca (IBSAL).
Sonia Ortega Lucea	Servicio de Anestesiología, Reanimación y Terapéutica del Dolor. Hospital Universitario Miguel Servet. Zaragoza.
Mª Azucena Pajares Moncho	Servicio de Anestesiología y Reanimación. Hospital Universitario y Politécnico la Fe. Valencia.
José Antonio Páramo	Servicio de Hematología. Clínica Universidad de Navarra.
Ana Pascual Bellosta	Servicio de Anestesiología, Reanimación y Terapéutica del Dolor. Hospital Universitario Miguel Servet. Zaragoza.
Rosario Vicente Guillen	Servicio de Anestesia y Reanimación. Hospital Universitario y Politécnico La Fe. Valencia.
María Rodríguez Pérez	Servicio de Cirugía Torácica. Clínica Universidad de Navarra. Madrid.
Enrique Salmerón González	Servicio de Cirugía Plástica y Quemados. Hospital Universitario y Politécnico La Fe. Valencia.
Nieves Vanaclocha Saiz	Servicio de Cirugía Plástica y Quemados. Hospital Universitario y Politécnico La Fe. Valencia.
Guillermo Tejón Pérez	Servicio de Anestesiología y Reanimación. Hospital Universitario Valdecilla, Santander.
Luis Mario Vaquero Roncero	Servicio de Anestesiología y Reanimación. Complejo Hospitalario Universitario de Salamanca.
Peter Vorwald	Servicio de Cirugía General y del Aparato Digestivo. Hospital Universitario Fundación Jiménez Díaz. Madrid.

TECHNICAL COORDINATION

Concepción Sánchez Fernández. Subdirección General de Promoción, Prevención y Calidad. Dirección General de Salud Pública, Calidad e Innovación.

Yolanda Agra Varela. Subdirectora Adjunta. Area de Calidad. SG de Promocion, Prevencion y Calidad.

DECLARATION OF INTEREST

All members of the Clinical Pathway working group have made the declaration of interests and this can be consulted, upon request, through GERM's Secretary (www.grupogerm.es).

The assessment of compliance with quality criteria of this document has been conducted in agreement with the collaboration agreement signed by Carlos II Health Institute, an independent organisation of the Ministry of Economy and Competitiveness, and the Aragonese Institute for Health Sciences, within the framework of the development of activities of the Spanish Network for Health Technology Assessment Agencies and NHS Benefits, financed by the Ministry of Health Social Services and Equality. The authors of the document have not received any type of economic compensation for their work.

Index

	Page
1. Executive summary	11
2. Introduction	13
2.1. Background	14
2.2. Justification and Objectives	14
2.3. Who is this for?	15
3. Inclusion and exclusion criteria	17
4. Methodology	19
5. Assistance process	21
6. Summary table of recommendations	23
7. Recommendations and sources of evidence	33
7.1. General	33
7.1.1. Patient preparation (outpatient)	33
7.1.2. Preoperative	55
7.1.3. Intraoperative	63
7.1.4. Posoperative	101
7.2. Specific (By specialties)	113
7.2.1. Esophageal Surgery	113
7.2.2. Cardiovascular Surgery	119
7.2.3. Torathic Surgery	131
7.2.4. Burns	134
7.2.5. Colorectal Surgery	136
7.2.6. Major Head and Neck Surgery	137
7.2.7. Traumatology and Orhopedic Surgery	141
8. Indicator Chart	152
8.1. Process indicators	152
8.2. Result indicators	154
9. Implementation Strategy	156
10. Anexes	158

10.1. Postoperative nausaea and vomiting prophylaxis – APFEL scale 158

10.2. Preoperative management for anemic patients 159

10.3. Nutrition screening algorithm 160

10.4. Preoperative management 161

10.5. Patient information 162

10.6. Patient satisfaction questionnaire 166

10.7. Abreviations 170

1. Executive Summary

Fast Track Surgery, also known as Enhanced Recovery or Enhanced Recovery After Surgery (ERAS) outside Spain, is a new approach to the management and care of surgical patients that aims to ensure that patients reach the operating room in the best possible conditions, receiving the best possible treatment during surgery and experiencing best recovery after the process. In this sense, intensified recovery protocols (IRP) cover the entire surgical process from diagnosis to intervention and until full incorporation to normal activity. All of which requires a coordination and teamwork effort of all the healthcare professionals involved as well as the patient him or herself.

The original idea and concept were born in Denmark at the beginning of the 1990s, by Prof. Henrik Kehlet. The first IRPs aimed at Colo Surgery. It was in this field that the reduction of the post-operative complications and a faster recovery, with a decrement in hospital stay were achieved and proved. Since then, numerous studies in almost every surgical area have concluded that IRPs can be applied to most surgical patients, and be implemented in most major surgical procedures, regardless of the patient's age. In fact, their benefits have proven to be more advantageous in elderly patients due to the implementing features aiming to reduce surgical trauma.

With all this in mind and thanks to a close collaboration between the Grupo Español de Rehabilitación Multimodal (GERM) and the Spanish Ministry of Health, Social Services and Equality, a care plan was developed aiming to reduce clinical practice variability. In 2015 The clinical pathway for Intensified Recovery in Abdominal Surgery (Via RICA) was published in close collaboration with other scientific societies to offer an interdisciplinary consensus document to improve postoperative recovery, maintaining patient safety and an optimal resource usage.

Over time, the need to update this document was patent, with the inclusion of other surgical procedures apart from abdominal surgery. As a result, this new pathway is proposed as an update for 2015 RICA, aiming to provide healthcare professionals with recommendations based on scientific evidence in consensus with a wide variety of scientific societies. All of us participating in the development if this new Clinical Pathway have done so with a deep understanding that the use and implementation of scientific evidence by healthcare professionals improves clinical effectiveness and early detection of complications, furthermore, with the harmonization and homogeneity of the treatments, achieved thanks to the agreed PRIs, teamwork is facilitated, and efficiency improves.

Therefore, the main objective of this document is to provide professionals with recommendations based on scientific knowledge and on the consensus of the different scientific societies involved for the implantation and evaluation of PRI in major surgery in adults. The document is divided into a general part, which includes a review of the perioperative steps common to all procedures, and a specific part for each of the particularities of each specialty included in the document.

For the preparation of this document, reviews were made of those points in which there were no Clinical Practice Guidelines or clear acceptance of verifiable scientific evidence.

Following the terminology proposed by GRADE, the document includes the list of recommendations with bibliographic references, as well as the level of evidence and grade of recommendation. Likewise, a table of indicators is provided to measure the process and results. To measure perceived quality, a patient satisfaction questionnaire has been designed. Finally, an informative text is provided on the general healthcare process for the patient.

2. Introduction

Until recently, perioperative treatment for patients undergoing elective abdominal surgery consisted of a series of habits acquired by practice rather than scientifically proven facts. At the beginning of 2000, the mean postoperative stay in Spain after colorectal surgery with these treatment guidelines was 11.8 days (95% CI 11.21 to 12.7)[a]. One of the main improvements within the surgical area in recent times is the introduction of fast-track or Intensified Recovery (IRP) programs. These programs stand on three fundamental pillars: implementing a package of perioperative strategies; interdisciplinarity, understood as the joint effort and organized contribution of the various healthcare professionals involved; and the active participation of the patient throughout the process.

With all this, in 2015 the RICA pathway (Intensified Recovery in Abdominal Surgery)[b] was published in Guía Salud (OPBE), a document of clinical practice recommendations that reviews the entire perioperative process (pre-, intra-, and postoperative) and constitutes a multimodal care pathway designed to achieve early recovery after surgery.

The Via RICA, supported by the best available scientific evidence, preserves the fundamental ideas of the IRPs, and aims to guide decision-making towards four fundamental principles of healthcare quality and safety:

1. To inform the patient during the entire process and involve them in the decision-making course.
2. Patient preparation and well-being optimization, to ensure that they are in the best possible condition for surgery.
3. The entire perioperative stage is based on proactive actions so that recommendations are adaptive and integrated throughout the whole pathway: before, during and after surgery.
4. Patients play an active role and share the responsibility to improve their recovery.

---

a. Ruiz P, Alcalde J, Rodriguez E, Landa JI, Jaurrieta E. Proyecto nacional para la gestión clínica de procesos asistenciales. Tratamiento quirúrgico del cáncer colorrectal. I. Aspectos generales. Cir Esp. 2002;71(4):173-80.

b. Grupo de trabajo. Vía Clínica de Recuperación Intensificada en Cirugía Abdominal (RICA). Vía clínica de recuperación intensificada en cirugía abdominal (RICA) Ministerio de Sanidad, Servicios Sociales e Igualdad. Instituto Aragonés de Ciencias de la Salud. 2014 Available from: http://portal.guiasalud.es/contenidos/iframes/documentos/opbe/2015-07/ViaClinica-RICA.pdf

RICA's recommendations represent a change, and change is always difficult. However, since its first publication in 2015, a willingness to change was shown by a significant number of healthcare professionals and the support of the Spanish Ministry of Health. In just four years, intensive recovery programs and protocols have been adopted by a remarkable number of hospitals in Spain and this number is increasing daily. This trend is turning the RICA pathway into a standard procedure.

After five years of its publication, the necessity to review and update it have been patent. New studies have appeared, and protocols have been extended to almost all surgical specialties with proven benefits. Thus, not only an update on the 2015 RICA was proposed, but also an extension to include these new specialties. New RICA pathway displays recommendations for almost every adult patient undergoing scheduled surgery.

2.1. BACKGROUND

IRPs combine a series of elements aiming to optimize recovery and reduce the response to surgical stress. After preliminary favorable results, they were introduced approximately 15 years ago, based on enough scientific evidence derived from randomized studies. They start at diagnosis stage and aim to acknowledge the patient's individual needs to optimize care before, during and after surgery.

Engagement in the treatment of all of those involved, including the patients and their family is essential. The adoption of a series of measures that form the protocol is the focus of the studies carried out; thus, some variability is shown because none of them adopt all the suggested measures. However, there is enough consensus to say that the implementation of these protocols is beneficial for the patients, as has been proven in recent metanalyses. The benefit obtained from these protocols is related to the percentage of compliance to them. This was demonstrated in a recent study by Grupo Español de Rehabilitación Multimodal (GERM).[c]

2.2. JUSTIFICATION AND OBJECTIVES

The growing demand for major surgery in high-risk patients requires further improvements that must include an evidence-based, procedure-specific, updated and multidisciplinary approach within the foundations of the Fast-Track protocol; the standardization of these measures is beneficial for the patient, the professionals and the centers; And it is possible to carry it out in a protocolized way at state level, as demonstrated by previous projects in other countries with good results.

This document deals with clinical aspects related to the perioperative management of the patient, to homogenize care and improve postoperative recovery, by reducing surgical complications and improving their perceived quality of life. Changing the way these patients are usually managed in the pre-surgical stage and in the intraoperative and postoperative recovery is necessary to achieve this goal.

c. Ripollés-Melchor J, Ramirez-Rodríguez JM, Casans-Francés R et al. Association Between Use of Enhanced Recovery After Surgery Protocol and Postoperative Complications in Colorectal Surgery. The Postoperative Outcomes Within Enhanced Recovery After Surgery Protocol (POWER) Study. JAMA Surgery 2019; 154(8):725-736.

The action range of this approach includes all patients over 18 years of age undergoing major surgery.

Enhanced Recovery Programs must be standardized at least for elective surgery, but to achieve this, a closer collaboration between surgeons, anesthesiologists, nutritionists, nurses, etc. is required to ensure compliance with all the steps of the protocol as this has proved to deliver the best results possible.

Therefore, the main objective of this document is to provide an instrument based on scientific evidence and with the consensus of the different Scientific Societies that can be used to standardize the surgical care process based on the principles of Intensified Recovery.

As specific objectives, it is intended to establish the clinical recommendations and responsibilities in the following stages:

- Preoperative optimization.
- Immediate preoperative.
- Intraoperative.
- Postoperative.

As well as:

- Defining the indicators used to measure the quality of the healthcare process, including its various dimensions: quality, scientific-technical, clinical effectiveness, quality of life and patient satisfaction.
- Providing complete written information to the patient.
- Designing a questionnaire to measure patient satisfaction.
- Proposing a strategy for the implementation of Intensified Recovery Programs for the different surgical procedures, including their specific aspects and particularities.

2.3. WHO IS IT FOR?

This update maintains the scope and is directed not only to healthcare professionals who are directly involved in the care of the surgical patient such as surgeons, anesthetists, and nurses, but to also those professionals who are in some way related to the interdisciplinary treatment of these patients, such as nutritionists, stoma therapists, rehabilitators, physiotherapists, digestive specialists, radiotherapists, oncologists, pathologists, geriatricians, and internists. Since one of advantages of these programs is cost efficiency (reduction of hospital stay, as well as use optimization of resources), we believe that this clinical pathway can also be useful for administrators, clinical managers, and quality coordinators. Finally, and due to the characteristics of enhanced recovery programs, in which patients play an active role, we believe it is also useful for them. We believe that primary care professionals must also benefit from it and incorporate it into their processes as they are part of the caring team.

3. Inclusion and Exclusion Criteria

Although there is no evidence and other patients could also benefit from this guideline, the advised criteria to start the process are the following.

INCLUSION CRITERIA

Major Surgery Procedures, not susceptible to being intervened by CMA and meeting the following criteria:

- Age: Over 18 years old.
- Any ASA.
- Process acceptance.

EXCLUSION CRITERIA

- Urgent surgery.
- Severe cognitive impairment that makes patient collaboration impossible.
- Pediatric patients.

4. Methodology

In order to update this document, a central working group of the RICA pathway was formed by a multidisciplinary team of healthcare professionals from the hospital care field of the following specialties: general surgery; nursing; urology; gynecology; anesthesia, resuscitation, and pain therapy; endocrinology and nutrition; hematology and hemotherapy; preventive medicine; rehabilitation and physical medicine; plastic and reconstructive surgery; thoracic surgery; cardiovascular surgery; otorhinolaryngology, orthopedic surgery and traumatology.

Likewise, we had collaborators for the search and evaluation of recommendations. As a final stage, the document was examined by a selected group of experts who acted as external reviewers, among these external reviewers in addition to patients, there were specialists in surgery, anesthesia, primary care, internal medicine, intensive medicine, geriatrics, nursing, preventive medicine.

With all this, the scientific societies involved in the development of the RICA pathway, represented by members of the working group and external reviewers, are: Foro Español de Pacientes (FEP), Plataforma de Organizaciones de Pacientes (POP), Asociación Española de Cirujanos (AEC), Sociedad Española de Anestesiologia, Reanimación y Terapia del Dolor (SEDAR), Sociedad Española de Endocrinología y Nutrición (SEEN), Sociedad Española de Nutrición Parenteral y Enteral (SENPE), Asociación Española de Coloproctología (AECP), Asociación Española de Enfermería Quirúrgica (AEEQ), Sociedad Española de Rehabilitación y Medicina Física (SEMERF), Asociacion Española de Urología (AEU), Sociedad Española de Enfermeria en Cirugia (SEECIR), Sociedad Española de Ginecología y Obstetrica (SEGO), Sociedad Española de Hematología y Hemoterapia (SEHH), Sociedad Española de Transfusión Sanguínea y Terapia Celular (SETS), Sociedad Española de Medicina Preventiva, Salud Pública e Higiene (SEMPSPH), Sociedad Española de Cirugia Cardiovascular y Endovascular (SECCE), Sociedad Española de Cirugía Plástica, Reparadora y Estética (SECPRE), Sociedad Española de Cirugía Torácica (SECT), Asociación Española de Cirugía Mayor Ambulatoria (ASECMA), Federacion de Asociaciones de Enfermería Familiar y Atención Primaria (FAECAP), Sociedad Española de Epidemiología (SEE), Sociedad Española de Geriatría y Gerontología (SEGG), Sociedad Española de Medicina Geriátrica (SEMEG), Sociedad Española de Médicos De Atención Primaria (SEMERGEN), Sociedad Española de Medicina Interna (SEMI), Sociedad Española de Patología Digestiva (SEPD), Sociedad Española de Médicos Generales y de Familia (SEMG), Sociedad Española de Medicina Intensiva, Crítica y Unidades Coronarias (SEMICYUC), Sociedad Española de Calidad Asistencial (SECA), Sociedad Española de Cirugia Oral y Maxilo Facial y de Cabeza y Cuello (SECOMCyC). También participaron miembros de los programas de Cirugía Segura y del Observatorio de Infección en Cirugía (OIC).

Reviews were carried out in PubMed, Embase, and the Cochrane Library. The studies complying with the inclusion criteria (establishing period and patient inclusion criteria) were examined by the experts proposed by societies belonging to the different participating Scientific Societies, establishing, according to the terminology proposed by GRADE[d], the level of evidence and the severity of the recommendation. To prepare the recommendations, a summary was made on the subject with the bibliographic references including the definition of the recommendation itself and the cataloging of the level of evidence and its severity. The main criteria that were considered for the proposal of the recommendations were, mainly, the quality of the evidence, the balance of benefits and risks and feasibility. Quality of evidence and risk/reward balance as well as feasibility were the main criteria for a recommendation to be considered.

Bibliographic reviews were carried out until August 2020. The intention of the authors and the scientific societies that they represent is to carry out a periodic update of this document, for which a permanent reviewing group will be created.

The list of indicators has been formulated on those established in the previous edition of the RICA pathway, updating it by consensus of the authors of this edition.

The questionnaire for measuring patient satisfaction has also been updated, considering the contributions of patients undergoing intensified recovery surgical procedures, to improve its readability and understandability.

The Intensified Recovery Clinic for Adult Surgery will have the following documentation:

1. Temporary matrix with all the activities and interventions that are performed on the patient throughout the healthcare process. All actions and the performing professional must be registered and signed.
2. Patient Information Sheet.
3. Recommendations at discharge.
4. Satisfaction survey.
5. Evaluation indicators.

The Clinical Pathway is part of the patient's medical history at the time of its application in any institution.

At the end of the document, as Annexes, the following documentation of the Clinical Path is grouped (Temporal matrix, algorithms, summary table of recommendations, abbreviations) to facilitate consultation and improve the usefulness of the document.

d. Alonso P, Rotaeche R, Rigau D, Etxeberria A, Martinez L. La evaluación de la calidad de la evidencia y la graduación de la fuerza de las recomendaciones: el sistema GRADE. (Sede web) A Coruña: Fisterra.com (Actualizada 10 de Octubre de 2019). Disponible en: https://wwww.fisterra.com

5. Assistance Process

PERIOD	ACTIVITY	RESPONSIBILITY
Before admission (outpatient)	Preoperative assessment. Special attention to the fragile patient. Cardiology, anemia, and comorbidity assessment, if required.<br>Recommendations: stop alcohol and tobacco consumption. Nutritional assessment and trimodal rehabilitation with nutritional optimization.<br>Information to the patient and their family.	Surgeon + Anesthesiologist + Endocrinologist + Nursing
Preoperative (preferably without admission)	Start thromboembolic prophylaxis (if the patient is admitted the previous afternoon, it will be done on admission).<br>Shower the night before.<br>Fasting before anesthetic induction: 6 hours for solids and 2 hours for clear liquids.<br>Avoid long and half-life benzodiazepines and opioids in elder patients.	Anesthesiologist + Nursing + Surgeon
Perioperative	<u>Immediate preoperative</u><br>Placement of compression stockings or intermittent pneumatic compression, according to thromboembolic risk.<br>Carbohydrate drink supplement 2 hours before intervention.<br>Prophylactic administration of antibiotics when indicated (or in the operating room).<br>Administration of 1 dose of glucocorticoids.<br>Avoid hair removal as much as possible.<br><u>Intraoperative</u><br>Follow surgical checklist.<br>Local anesthesia preferred, if possible.<br>Use of epidural catheter in open major abdominal surgery.<br>FiO2 oxygenation 0.6-0.8.<br>Hemodynamic optimization through goal-guided fluid therapy (FGO).<br>Monitoring and maintenance of blood glucose <180mg / dl.<br>Avoid opioids as much as possible.<br>In high-risk hemorrhagic surgery, assess the use of tranexamic acid.<br>If urinary catheter needed, remove it as soon as possible.	Nursing<br>Nursing + Anesthesiologist + Surgeon<br>Nursing + Anesthesiologist

PERIOD	ACTIVITY	RESPONSIBILITY
Perioperative	Avoid NG as much as possible.<br>Active heating with thermal blanket and fluid heater. Prophylaxis of postoperative nausea and vomiting according to the Apfel scale.<br>Avoid drainage as a routine.<br>Infiltration of the laparoscopic ports or block of the transverse plane of the abdomen (TAP) depending on the intervention.<br>Immediate postoperative.<br>Actively maintain temperature.<br>Maintain FIO2 at 0.5 2h after intervention.<br>Scheduled analgesia according to intervention. Keep morphic administration to a minimum.<br>Restrictive fluid therapy.<br>Oral tolerance 6 hours after surgery.<br>Start of mobilization at 8 hours after surgery. Thromboembolism prophylaxis.	Nursing<br>Nursing + Anesthesiologist + Surgeon<br>Nursing + Anesthesiologist
Day 1 postoperative day	Nutritional supplementation in selected cases. Normal diet according to tolerance.<br>Assess drainage removal, if applicable.<br>Active mobilization (bed / chair / wandering).<br>Intravenous analgesia. Avoid morphics.<br>If correct oral tolerance, withdrawal of intravenous fluids. Evaluate removal of urinary catheterization, if applicable.<br>Respiratory physiotherapy. Prophylaxis of thromboembolism.	Nursing + Surgeon
Day 2 postoperative day	Evaluate removal of urinary catheterization, if applicable.<br>Consider removal of the epidural catheter, if applicable.<br>Normal diet<br>Withdrawal of intravenous fluids. Active mobilization (wandering).<br>Prophylaxis of thromboembolism.<br>Assess discharge.	Nursing + Surgeon
During the remainder of the hospitalization	Normal diet<br>Oral analgesia.<br>Active mobilization (wandering). Thromboembolism prophylaxis.<br>Surgical wound revision.<br>Assess discharge.	Nursing + Surgeon
At discharge	Maintenance of thromboprophylaxis according to type of surgery.<br>Telephone checkups after discharge.<br>General discharge criteria: No surgical complications, no fever, pain can be controlled with oral analgesia, successful wandering, acceptance by the patient.<br>Follow-up on discharge / Primary Care.<br>Home support-Coordination with Primary Care.	Nursing + Surgeon + MAP

6. SUMMARY TABLE OF RECOMMENDATIONS

No.	Recommendation	Evidence level	Severity
PATIENT PREPARATION (OUTPATIENT)			
1	Patients must receive complete verbal and written information of what is required of them to improve their recovery after surgery.	Moderate	Strong
2	Preoperative assessment of patient frailty is recommended to identify patients at higher perioperative risk.	High	Strong
3	Patients with acute or decompensated heart disease must be multidisciplinary assessed due to anesthetic and surgical risk.	High	Strong
4	Assessment of the patient's physical status using the ASA classification is recommended in all patients undergoing surgery.	High	Strong
5	It is advisable to stop smoking 4-8 weeks prior to surgery, to reduce associated complications.	High	Strong
6	Alcohol consumption should be stopped one month prior to surgery.	Moderate	Strong
7	Trimodal prehabilitation therapy is recommended to improve functional capacity prior to surgical intervention.	Moderate	Strong
8	Nutritional screening is recommended for all patients undergoing major surgery.	Moderate	Strong
9	When a patient at risk of malnutrition is identified, a complete nutritional assessment must be carried out, a nutritional treatment plan must be established, with tolerance monitoring and compliance to it.	Moderate	Strong
10	All patients with severe nutritional risk or severe malnutrition must receive nutritional treatment at least 7-10 days before surgery. Whenever possible, the oral / enteral route will be preferred.	Moderate	Strong

No.	Recommendation	Evidence level	Severity
11	Regarding preoperatory stages, the level of evidence to recommend inmunonutrition versus standard oral supplements is not enough.	Low	Weak
12	It is suggested that as soon as a patient enters the surgical waiting list or when the need of surgical procedure is notified, the appearance of anemia or any blood deficit should be monitored as well as its study and proper management.	Low	Weak
13	The implementation of “Patient Blood Management” Programs is recommended in all hospitals and health areas. We suggest the integration of the PBM program within the ERAS protocol.	High	Strong
14	Avoiding scheduling elective surgery with risk of bleeding in patients with anemia is recommended until proper diagnosis and management.	High	Strong
15	At least one hemoglobin determination is recommended in patients undergoing elective surgery, at least 28 days before surgery or invasive procedure.	Moderate	Strong
16	In surgical oncology, it is recommended to use all the time available between diagnosis and surgery to detect anemia and correct it or, at least, improve hemoglobin concentration.	Moderate	Strong
17	It is recommended that the preoperative hemoglobin concentration before surgery should be above13 g / dl, regardless of gender / sex.	Moderate	Strong
18	Detection and treatment of perioperative iron deficiency is recommended.	Moderate	Strong
19	The detection and treatment of preoperative anemia is recommended, even in cases of urgent surgery.	Moderate	Strong
20	Oral iron treatment is recommended in cases of iron deficiency or mild-moderate iron deficiency anemia, if there is at least 6 weeks until surgery.	Low	Strong
21	Preoperative treatment with intravenous iron (FEEV) is recommended in potentially bleeding elective surgery patients with iron deficiency anemia and / or functional iron deficiency, to improve hemoglobin levels and / or reduce transfusion rate.	Moderate	Strong
22	The administration of intravenous iron is recommended, instead of oral iron, in those cases in which it is contraindicated or the time available until surgery is insufficient.	Moderate	Strong
23	The administration of rHuEPO is recommended in elective orthopedic surgery patients at risk of moderate-high bleeding and moderate non-deficiency anemia (Hb between 10 and 13 g/dL), to reduce the allogeneic transfusion rate.	High	Strong
24	The administration of rHuEPO is suggested to reduce transfusion rate in anemic patients undergoing major elective surgery other than elective orthopedic surgery with a moderate-high risk of bleeding.	Moderate	Weak
25	The use of thromboprophylaxis is recommended in all patients undergoing major surgery or hospitalized due to an acute medical condition.	Moderate	Strong

No.	Recommendation	Evidence level	Severity
26	In general, it is recommended to maintain antithrombotic prophylaxis for a minimum of 7 days or until the patient is ambulation.	High	Strong
27	In the case of major abdominal surgery, the prophylaxis will be extended up to 4 weeks after surgery.<br>Specific situations:<br>• In general, urological, gynecological and neurosurgery surgery: 8 days; in case of immobilization of the patient, it should be prolonged until ambulation.<br>• In general, urological, and gynecological surgery in patients with cancer: 4 weeks (28 days).<br>• In hip surgery: 4-6 weeks (28-42 days).<br>• In knee surgery: 3-4 weeks (21-28 days).	Moderate	Strong
28	Early mobilization and the use of gradual compression elastic stockings are recommended for the duration of the immobilization period.	High	Strong
29	Compression stockings are effective in preventing thromboembolic disease in surgical patients, reducing the risk even more if combined with pharmacological agents.	High	Strong
30	Intermittent pneumatic compression devices decrease the incidence of deep vein thrombosis, the method combined with pharmacological measures being more effective, mainly for neurosurgical patients and / or surgeries with high VTE risk.	Moderate	Strong
31	Prophylaxis regimens include direct acting oral anticoagulants (dabigatran, apixaban, rivaroxaban) or low molecular weight heparins (enoxaparin, bemiparin, tinzaparin).	High	Strong
32	A full bath is recommended prior to surgery.	Moderate	Strong
33	In most patients undergoing an elective surgical procedure, solid food should be allowed up to 6 hours before induction of anesthesia, and clear liquids up to 2 hours before anesthesia.	High	Strong
3. 4	In those patients with delayed gastric emptying and in emergency surgery, it is recommended to fast from midnight or 6-8 hours before surgery.	Moderate	Strong
35	Oral intake of carbohydrate-rich beverages up to 2 hours before surgery is safe and is not associated with increased risk of aspiration.	Moderate	Strong
36	Oral administration of 200-400 ml of a drink containing 50 g of carbohydrates should be allowed up to two hours before surgery since this treatment improves the patient's feeling of well-being and can reduce hospital stay and insulin resistance.	Moderate	Strong
37	In obese and / or type 2 diabetic patients with good glycemic control without associated chronic complications, the use of carbohydrate-rich drinks 3 hours before surgery could be considered. This can be given together with your usual antidiabetic medication.	Low	Weak

No.	Recommendation	Evidence level	Severity
38	It is recommended to avoid the use of long half-life benzodiazepines and opioids prior to induction in patients at high risk due to age and comorbidity.	Low	Strong
	PREOPERATIVE		
39	Antibiotic prophylaxis is recommended if the odds infection rates are high or if the consequences of a postoperative infection are potentially serious for the patient (endocarditis, endophthalmitis, prosthetic infection).	Moderate	Strong
40	In clean surgery with risk factors for infection, it is recommended to use antibiotics that cover microorganisms of the skin microbiota (S. aureus and coagulase negative staphylococci) and in clean-contaminated surgery also gram-negative bacilli and enterococci as well as anaerobes.	Moderate	Strong
41	Antibiotic prophylaxis is recommended for 120 minutes prior to surgical incision.	High	Strong
42	It is recommended to use the same dose of prophylaxis as that used for the treatment of the infection, although in obese patients the adjusted weight should be used to calculate the dose.	Moderate	Strong
43	An additional dose is recommended in cases of prolonged surgeries or if there is significant blood loss.	Moderate	Weak
44	It is recommended not to prolong the duration of antibiotic prophylaxis beyond the duration of the surgery itself.	High	Strong
45	The administration of a single dose of glucocorticoids is recommended because it has a significant impact on the duration of hospital admission without increasing the rate of complications.	Moderate	Strong
46	Blood glucose will be monitored preoperatively, as intraoperative hyperglycemia can lead to an increase in postoperative complications, although the use of intensive insulin therapy should be avoided, due to the risk of hypoglycemia.	High	Strong
47	Perioperative blood glucose should be monitored and treated with insulin, avoiding levels> 180 mg / dl.	Moderate	Strong
48	More ambitious targets for perioperative blood glucose between 110 and 140mg / dL (6.1-7.8 mmol / L) may be appropriate in selected patients if they can be achieved without significant hypoglycemia.	Low	Weak
49	Hair should not be removed preoperatively unless strictly necessary. Conventional shaving should be avoided, both preoperatively and in the operating room.	Low	Strong
50	In the case of hair removal, electric razors can be used as close as possible to the intervention, but always outside the operating room.	Moderate	Strong
	INTRAOPERATIVE		
51	The use of the surgical checklist is recommended for the prevention of adverse events and mortality related to the intervention.	Moderate	Strong
52	The use of 2% alcoholic chlorhexidine is recommended as an antiseptic for intact skin in the surgical field.	High	Strong
53	It is recommended to minimize the use of benzodiazepines prior to induction and to use hypnotic agents with minimal residual effect, which allow rapid recovery after anesthesia.	Low	Strong

No.	Recommendation	Evidence level	Severity
54	The use of ventilation is recommended during general anesthesia protection, which includes a tidal volume of 6-8ml / kg ideal weight, the use of individualized PEEP generally above 5 cm H2O and the application of recruitment maneuvers.	Moderate	Strong
55	In surgeries that require one-lung ventilation, we recommend the above protective ventilation measures, but decreasing the tidal volume to the lung dependent on 4-6mL / kg of ideal weight.	Moderate	Strong
56	CO2 monitoring by capnography should be mandatory in all surgery, especially laparoscopic.	High	Strong
57	Temperature monitoring should be central.	High	Strong
58	The anesthetic depth will be monitored using the bispectral index (BIS).	High	Strong
59	The use of nociception monitoring could decrease intraoperative opioid consumption compared to standard monitoring.	Moderate	Weak
60	When a bladder catheter is placed, it will be done with the appropriate aseptic measures, and, if possible, it will be removed 24 hours after surgery.	Moderate	Weak
61	Removal of the urethral catheter is recommended at 24 h, except in moderate risk of acute urine retention: men, epidural anesthesia, and pelvic surgery, which is recommended for 3 days.	High	Strong
62	Invasive hemodynamic monitoring is not routinely indicated, and arterial cannulation is useful in those patients who present severe cardiorespiratory alterations and who may present problems in the postoperative period.	Low	Strong
63	Central venous catheter (CVC) insertion is not routinely indicated and is limited to patients with severe cardiorespiratory diseases with pulmonary hypertension or in whom it is anticipated that they may require administration of vasopressors or inotropes in continuous infusion.	Low	Strong
64	The use of quantitative monitoring of the block is necessary neuromuscular (BNM) provided that neuromuscular blocking drugs are used throughout the surgical procedure.	High	Strong
65	The use of deep neuromuscular blockade is recommended (PTC 1-2)to improve the visualization of the surgical field, both in open and laparoscopic surgery, and to use the lowest possible intra-abdominal pressures in laparoscopy, favoring postoperative recovery.	High	Strong
66	It is recommended to check the reversal of BNM until a TOF ratio greater than or equal to 0.9 is obtained in the adductor pollicis muscle during the anesthetic discharge prior to extubating to avoid residual neuromuscular block and reduce respiratory complications.	High	Strong
67	It is recommended to perform the reversal of BNM with sugammadex in instead of neostigmine when rocuronium bromide has been used, as it is faster and safer.	High	Strong

No.	Recommendation	Evidence level	Severity
68	It is recommended to prevent and avoid perioperative hypothermia involuntary.	High	Strong
69	Temperature of patients should be controlled to guarantee normothermia in the perioperative period.	High	Strong
70	Active warm-up strategies should be started prior to surgery.	High	Strong
71	Ambient temperature in the operating room should be at least 21 ° C for adult patients.	High	Strong
72	During the perioperative period, the largest possible surface area of the body should be thermally insulated.	High	Strong
73	Infusions, cavity fluid infusions, and blood transfusions given at doses> 500 mL / h should be warmed first.	High	Strong
74	Intraoperative active warming measures are indicated by the administration of convective or conductive heat to maintain normothermia.	High	Strong
75	The removal of general anesthesia should take place at normal body temperature.	High	Strong
76	The use of adequate monitoring (VS or VVS) to guide intraoperative fluid administration in patients at risk.	High	Strong
77	In cases where there is an SV drop> 10% or an SVV > 10%, fluid resuscitation is indicated (there is no preference between colloids or crystalloids).	High	Strong
78	A moderate continuous fluid infusion is recommended, giving a positive balance at the end of surgery of 1 to 2 L. to avoid postoperative acute kidney damage.	High	Strong
79	In high-risk patients, it is recommended to maintain individualized fluid therapy with a moderately positive balance and continuous monitoring of SV or SVV.	Moderate	Strong
80	Intraoperative hypotension unresponsive to lifting Passive legs should be treated with vasopressors (checking for variations in blood pressure, VS and SVV).	Moderate	Strong
81	A mean arterial pressure range greater than or equal to 65 mm Hg should be established.	High	Strong
82	A cardiac index (CI)> 2.5 l / min / m should be maintained^{two}, using inotropes in cases of non-response to volume.	High	Strong
83	Monitoring by esophageal Doppler or validated pulse contour analysis-based methods is preferred.	High	Strong
84	The primary maintenance intravenous fluid should be a balanced, isotonic crystalloid solution.	High	Strong
85	For fluid therapy in resuscitation, the use of balanced crystalloids is recommended, 2-3 liters for initial resuscitation in hypovolemic shock and hemodynamic monitoring to guide the additional administration of fluids.	Moderate	Strong
86	It is recommended that all adults who undergo surgery and are expected to have moderate-severe blood loss be offered tranexamic acid.	High	Strong

No.	Recommendation	Evidence level	Severity
87	The supplemental use of inspired oxygen is not recommended in patients undergoing general anesthesia.	Moderate	Weak
88	Minimally invasive surgery is recommended, provided that the surgical and oncological results do not differ between the surgical techniques	High	Strong
89	Transverse incision is recommended in laparotomic surgery.	Moderate	Strong
90	It is recommended not to use drains on a routine basis.	High	Strong
91	The use of nasogastric tube is not recommended routinely.	High	Strong
92	Epidural analgesia should be performed within combined anesthesia in all patients undergoing major open abdominal surgery procedures.	High	Strong
93	Catheterization of the epidural space for infusion of local anesthetics for analgesia in open major abdominal surgery should be performed at the thoracic level.	High	Strong
94	Small doses of opioids should be added to the local anesthetic doses to be delivered epidurally in major open surgery.	Moderate	Strong
95	When the provision of an epidural catheter is not possible in open major surgery, the analgesic strategy should be individualized, reducing the use of opioids, and favoring the use of locoregional blocks, spinal analgesia, or port infiltration with local anesthetics, especially considering the transverse plane block of the abdomen.	Moderate	Strong
96	Performing a bilateral transverse plane block with local anesthetics could benefit those patients who require open major abdominal surgery and who could not benefit from epidural analgesia.	Moderate	Strong
97	Opioid-free anesthesia in a multimodal setting may be an alternative to the use of intravenous opioids.	Moderate	Weak
98	The use of intraoperative intravenous lidocaine is recommended as an adjunct medication in reducing postoperative pain and improving the recovery of intestinal function in the immediate postoperative period, being an alternative to the use of intravenous opioids.	Moderate	Weak
99	Iv Ketamine should be given to those patients receiving major opioids for analgesia in major abdominal surgery.	Moderate	Weak
100	The use of intraoperative iv magnesium sulfate is recommended as an analgesic adjuvant to improve pain control in patients undergoing abdominal surgery.	Moderate	Weak
101	The use of intraoperative intravenous dexmedetomidine is recommended for contributing to the reduction of the risk of adverse events associated with opioids and improving pain control in the intra and postoperative period.	Moderate	Weak
102	In open major abdominal surgery, a preoperative dose of oral gabapentin or pregabalin could be assessed before the intervention for postoperative analgesic control.	High	Weak

No.	Recommendation	Evidence level	Severity
103	Multimodal management using alternatives to opioids (thoracic epidural catheter, blocks, minimally invasive surgery, avoiding the routine use of nasogastric tube and avoiding an excess of IV therapy fluid) is recommended as measures to prevent the appearance of postoperative paralytic ileus.	High	Strong
104	The risk of postoperative nausea and vomiting should be stratified (PONV) in all patients using the Apfel scale and carry out prophylaxis proportional to the expected risk. Prophylaxis with more combined drugs can be performed in surgeries in which PONV pose a significant risk of complications.	High	Strong
105	Regional anesthesia is recommended over general anesthesia to decrease the incidence of PONV.	High	Strong
106	The use of propofol is recommended for induction and maintenance of anesthesia in patients at high risk for PONV.	High	Strong
107	The use of nitrous oxide should be avoided in patients at high risk for PONV or prolonged surgeries.	High	Strong
108	The use of inhalational anesthetics should be avoided in patients at high risk of PONV.	Moderate	Strong
109	It is advisable to minimize the use of intraoperative opioids and especially postoperative ones.	High	Strong
110	It is advisable to carry out antiemetic prophylaxis in monotherapy in Apfel 0-1 patients but surgery with a higher risk of PONV.	Moderate	Strong
111	Antiemetic prophylaxis should be performed as monotherapy in patients with an Apfel 2-3 assessment and double therapy if surgery with a higher risk of PONV.	High	Strong
112	It is recommended to perform antiemetic prophylaxis in double combination therapy in patients with an Apfel 4 assessment and triple therapy if surgery with a higher risk of PONV.	High	Strong
113	The use of peripheral opioid receptor antagonists is recommended to prevent the appearance of ileus in the postoperative period.	Moderate	Weak
POSTOPERATIVE			
114	Postoperative hypothermia should be treated by administration of convective or conductive heat until normothermia is achieved.	High	Strong
115	Non-steroidal anti-inflammatory drugs (NSAIDs) should be used as adjunctive therapy for pain control in patients who have undergone major abdominal surgery.	High	Strong
116	The use of gum is not recommended routinely.	Low	Weak
117	In nausea and vomiting established selective antagonists 5-HT3 (ondansetron) is the treatment of choice, followed by a different antiemetic drug family if unresponsive except for dexamethasone.	High	Strong
118	Use of laxatives such as bisacodyl (in colorectal surgery), oral magnesium oxide (in hysterectomy), daikenchuto (Japanese herbal infusion, in gastrectomy), coffee (in colorectal surgery) as elements that could prevent the appearance of ileus.	Low	Weak

No.	Recommendation	Evidence level	Severity
119	Inmunonutrition seems advisable in malnourished patients undergoing gastrointestinal surgery for cancer, due to the decrease in infectious complications, and a possible shortening of hospitalization.	Low	Strong
120	The use of epidural analgesia is recommended for the first 24-48 h after surgery and its withdrawal after this initial period of pain control, decreasing the concentrations of local anesthetics with epidural opioids to reduce motor block and allow ambulation.	High	Strong
121	The use of paracetamol and NSAIDs is recommended for the control of postoperative pain with opioid rescues in case of severe pain not controlled with epidural analgesia or with other local or regional analgesia techniques.	High	Strong
122	Early postoperative feeding should be started as soon as possible, within hours after surgery in most patients.	Moderate (high in colorectal surgery)	Strong
123	Early mobilization through patient education and encouragement is recommended to reduce the number of adverse effects.	Moderate	Strong
124	Preoperative and postoperative respiratory physiotherapy is recommended.	High	Strong
125	Oral administration of iron salts is not recommended in the immediate postoperative period to improve the hemoglobin level and decrease the transfusion rate.	Moderate	Strong
126	Postoperative treatment with FEEV is suggested to improve the hemoglobin levels and reduce the transfusion rate, especially in patients with low iron stores and / or anemia moderate-severe post-bleeding.	Moderate	Strong
127	The application of "restrictive" transfusion criteria is recommended of packed red blood cells (CH) (if symptoms or Hb level <70 g / L), in most hospitalized patients (medical, surgical, or critical), without active bleeding and hemodynamically stable (including septic, upper gastrointestinal bleeding and postpartum anemia)	High	Strong
128	The application of "restrictive" transfusion criteria is recommended. of CH (Hb ≤75 g / L) in cardiac surgery patients.	Moderate	Strong
129	The application of restrictive transfusion criteria is recommended of CH (Hb <80 g / L) in patients with a history of cardiovascular disease who underwent orthopedic surgery or hip fracture repair surgery	Moderate	Strong
130	Clean the surgical wound with sterile isotonic saline, potable water, or distilled water.	Moderate	Strong
131	Topical antibiotics can be applied to surgical wounds with primary closure after surgery to prevent surgical site infection.	Low	Weak
132	In wounds with closure by first intention, whenever possible, it is suggested not to lift the dressing during the first 24-48 hours.	Low	Weak

No.	Recommendation	Evidence level	Severity
133	The use of TPN can decrease the risk of surgical site infection and shorten healing in open surgical wounds, mainly in abdominal or thoracic surgeries.	Low	Weak
134	Patients and their caregivers should receive, upon discharge, understandable and complete personalized information. Planning discharge and providing adequate information on post-discharge care influences the mean stay and readmissions.	High	Strong
135	Audits of intensified recovery procedures are advised to assess clinical adequacy and effectiveness.	Moderate	Strong

7. Recommendations and Sources of Evidence.

7.1. GENERAL

7.1.1. PATIENT PREPARATION (OUTPATIENT)

Informing the patient and their environment

INTRODUCTION

Information to the patient and his environment is a key point in the surgical process. Prior advice and information favor early discharge[1] and reduces hospital stay[2,3]. The patient must be aware of the treatment options and have a realistic expectation of the likely risks and benefits. Achieving maximum collaboration and implication by the patient during the whole treatment is the main goal[4,5,6].

The best measurement instrument to assess the level of preparation that patients have for the intervention is their opinion about how well prepared they feel.[7]

Apart from the participation of the surgical team in this phase, the involvement of the nursing staff that will participate later in the postoperative period is also essential. Information must be given both verbally and in writing.

The information must be individualized, adapting it to the characteristics of each patient (comprehension capacity, cultural level, etc.). It is known that a large part of verbal information provided to patients in the preoperative period is forgotten, sometimes remembering less than 25% of the information provided, especially that related to preoperative medication[8-10].

Using informatory brochures or flyers is particularly useful to achieve maximum collaboration in enhancing recovery protocols. It has been demonstrated that this information improves patient satisfaction; reduces anxiety and postoperative pain. These brochures should include the main points of postoperative rehabilitation, the benefits that are obtained and how to obtain them, especially those referring to mobilization, diet, and respiratory exercises. If a stoma is to be performed, a visit to a specialist before the intervention greatly improves results[11-14].

1. Patients should receive complete verbal and written information of what is needed to improve their recovery after surgery.

   Moderate level of evidence. Strong recommendation.

REFERENCES

1. Nelson G, Bakkum-Gamez J, Kalogera E, Glaser G, Altman A, Meyer LA et al. Guidelines for perioperative care in gynecologic/oncology: Enhanced Recovery After Surgery (ERAS) Society recommendations-2019 update.Int J Gynecol Cancer. 2019; 29(4):651-668.
2. Gustafsson UO, Scott MJ, Hubner M, Nygen J, Demartines N Francis N, et al.Guidelines for Perioperative Care in Elective Colorectal Surgery: Enhanced Recovery After Surgery (ERAS ®) Society Recommendations: 2018.World J Surg. 2019; 43(3):659-695.
3. Wongkietkachorn A, Wongkietkachorn N, Rhunsiri P. Preoperative needs-based education to reduce anxiety, increase satisfaction, and decrease time spent in day surgery: a randomized controlled trial. World J Surg. 2018, 42(3):666-674.
4. Sugai DY, Deptula PL, Parsa AA, Parsa FD. The importance of communication in the management of postoperative pain. Hawaii J Med Public Health. 2013; 72(6):180-4.
5. Forster AJ, Clark HD, Menard A, Dupuis N, Chernish R, Chandok N et al. Effect of a nurse team coordinator on outcomes for hospitalized medicine patients. Am J Med 2005; 118(10):1148-53.
6. Chan Z, Kan C, Lee P, Chan I, Lam J. A systematic review of qualitative studies: patients' experiences of preoperative communication. J Clin Nurs. 2012; 21(5-6):812-24.
7. Forshaw KL, Carey ML, Hall AE, Boyes AW, Sanson-Fisher R.Preparing patients for medical interventions: A systematic review of the psychometric qualities of published instrument. Patient Educ Couns. 2016; 99(6):960-73.
8. Ronco M, Iona L, Fabbro C, Bulfone G, Palese A. Patient education outcomes in surgery: a systematic review from 2004 to 2010. Int J Evid Based Healthc. 2012; 10(4):309-23
9. Kruzik N. Benefits of preoperative education for adult elective surgery patients. AORN J. 2009; 90(3):381-7.
10. Broadbent E, Kahokehr A, Booth RJ, Thomas J, Windsor JA, Buchanan CM et al. A brief relaxation intervention reduces stress and improves surgical wound healing response: a randomised trial. Brain Behav Immun 2012; 26(2):212-7.
11. Mora M, Shell JE, Thomas CS, Ortiguera CJ, O'Connor MI. Gender differences in questions asked in an online preoperative patient education program. Gend Med 2012;9(6):457-62.
12. Smith F, Carlsson E, Kokkinakis D, Forsberg M, Kodeda K, Sawatzky R, et al. Readability, suitability and comprehensibility in patient education materials for Swedish patients with colorectal cancer undergoing elective surgery: a mixed method design. Patient Educ Couns 2014;94(2):202-9.
13. Sandberg EH, Sharma R, Sandberg WS. Deficits in retention for verbally presented medical information. Anesthesiology 2012; 117(4):772-9.
14. Lee A, Gin T. Educating patients about anaesthesia: effect of various modes on patients' knowledge, anxiety and satisfaction. Curr Opin Anaesthesiol 2005;18(2):205-8.

Frail patient

INTRODUCTION

The importance of evaluating the frailty and cognitive dysfunctions of the patient should be emphasized, due to the impact they have on postoperative results. The population is getting older, and the prevalence is close to 50% of patients awaiting surgery. Although there is no ideal scale, any screening tool is better than none.[1-5]

2. Preoperative assessment of frailty is recommended to identify patients at higher perioperative risk

High level of evidence. Strong recommendation.

REFERENCES

1. Dalton A, Zafirova Z. Preoperative Management of the Geriatric Patient: Frailty and Cognitive Impairment Assessment. Anesthesiol Clin. 2018 ;36(4):599-614.
2. Subramaniam S, Aalberg J, Soriano RP, Divino CM. New 5-Factor Modified Frailty Index Using American College of Surgeons NSQIP Data. J Am Coll Surg. 2018 ;226(2):173-181.
3. Wang J, Zou Y, Zhao J, Schneider DB, Yang Y, Ma Y et al. The Impact of Frailty on Outcomes of Elderly Patients After Major Vascular Surgery: A Systematic Review and Meta-analysis. Eur J Vasc Endovasc Surg. 2018;56(4):591-602.
4. Castellví Valls J, Borrell Brau N, Bernat MJ, Iglesias P, Reig L, Pascual L et al. Colorectal carcinoma in the frail surgical patient. Implementation of a Work Area focused on the Complex Surgical Patient improves postoperative outcome. Cir Esp. 2018;96(3):155-161.
5. Shen Y, Hao Q, Zhou J, Dong B.The impact of frailty and sarcopenia on postoperative outcomes in older patients undergoing gastrectomy surgery: a systematic review and meta-analysis. BMC Geriatr. 2017;17(1):188.

Patología cardiaca preoperatoria

INTRODUCTION

Patients with recent-onset or decompensated active cardiac disease should be evaluated by a multidisciplinary team involving all physicians related to the management of the perioperative period since the interventions may have implications in surgical and anesthetic management.[1,2]

3. Patients with acute or decompensated heart disease should be assessed by a multidisciplinary team due to anesthetic and surgical risk.

High level of evidence. Strong recommendation.

REFERENCES

1. Kristensen SD, Knuuti J, Saraste A, Anker S, Bøtker HE, Hert SD, et al. 2014 ESC/ESA Guidelines on non-cardiac surgery: cardiovascular assessment and management: The Joint Task Force on non-cardiac surgery: cardiovascular assessment and management of the European Society of Cardiology (ESC) and the European Society of Anaesthesiology (ESA). Eur Heart J. 2014;35(35):2383-431.
2. Duceppe E, Parlow J, MacDonald P, Lyons K, McMullen M, Srinathan S, et al. Canadian Cardiovascular Society Guidelines on Perioperative Cardiac Risk Assessment and Management for Patients Who Undergo Noncardiac Surgery. Can J Cardiol. 2017;33(1):17-32.

Assessment of anesthetic-surgical risk

INTRODUCTION

In addition to the perioperative risk implied in any surgery, there is an extra risk derived from the physical condition of the patient before the intervention. Risk assessment based on the ASA classification continues to be one of the best and simplest scales for assessing patients physical condition[1]. However, to assess perioperative risk, patients' frailty, cognitive dysfunction, and surgical risk must be added due to their impact in postoperative outcomes. The population is getting older and the prevalence of these last two entities is close to 50%. Although there is no ideal scale to measure it, any detection tool is better than none[2,3].

4. Assessment of the patient's physical status using the ASA classification is recommended in all patients undergoing surgery.

High level of evidence. Strong recommendation.

REFERENCES

1. Mayhew D, Mendonca V, Murthy BVS. A review of ASA physical status-historical perspectives and modern developments. Anaesthesia 2019; 74:373-9.
2. Dalton A, Zafirova Z. Preoperative Management of the Geriatric Patient: Frailty and Cognitive Impairment Assessment. Anesthesiol Clin. 2018;36(4):599-614.
3. Subramaniam S, Aalberg J, Soriano RP, Divino CM. New 5-Factor Modified Frailty Index Using American College of Surgeons NSQIP Data. J Am Coll Surg. 2018;226(2):173-18.

Recommendations to the patient on toxic habits

INTRODUCTION

Tobacco consumption and alcohol abuse are two habits that negatively affect the patient's recovery after surgery. They are associated with respiratory, wound, metabolic, infectious, and hemorrhagic complications[1]. Prior to the operation, excessive alcohol consumption and smoking should be assessed using validated detection tools[2].

Smoking is also associated with a poor postoperative quality of life and fatigue and a reduction in long-term survival in patients undergoing thoracic surgery[3]. In the preoperative stage, counseling by the preadmission nurse, information brochures, and nicotine replacement therapy are more likely to be effective for smoking cessation up to 30 days after surgery[4]. Between 4-8 weeks of abstinence seems necessary to reduce respiratory complications and wound healing[5].

Consuming more than two units of alcohol per day (20 grams of ethanol) increases intraoperative bleeding and the rate of postoperative infections. Preoperative interventions to stop drinking alcohol can significantly reduce postoperative complication rates[6].

5. It is advisable to stop smoking 4-8 weeks prior to surgery to reduce associated complications.

High level of evidence. Strong recommendation.

6. Alcohol consumption should be stopped one month prior to surgery.

Moderate level of evidence. Strong recommendation.

REFERENCES

1. Gaskill CE, Kling CE, Varghese TK Jr, et al. Financial benefit of a smoking cessation program prior to elective colorectal surgery. J Surg Res. 2017; 215:183-189.

2. Engelman DT, Ben Ali W, Williams JB, Perrault LP, Reddy VS, Arora RC, et al. Guidelines for Perioperative Care in Cardiac Surgery: Enhanced Recovery After Surgery Society Recommendations. JAMA Surg. 2019; 154(8):755-766.
3. Batchelor TJP, Rasburn NJ, Abdelnour-Berchtold E, Brunelli A, Cerfolio RJ, Gonzalez M, et al. Guidelines for enhanced recovery after lung surgery: recommendations of the Enhanced Recovery After Surgery (ERAS®) Society and the European Society of Thoracic Surgeons (ESTS). Eur J Cardiothorac Surg. 2019:55(1):91-115.
4. Low DE, Allum W, De Manzoni G, Ferri L, Immanuel A, Kuppusamy M, et al. Guidelines for Perioperative Care in Esophagectomy: Enhanced Recovery After Surgery (ERAS®) Society Recommendations. World Journal of Surgery. 2019;43(2):299-330.
5. Gustafsson UO, Scott MJ, Hubner M, Nygren J, Demartines N, Francis N, et al. Guidelines for Perioperative Care in Elective Colorectal Surgery: Enhanced Recovery After Surgery (ERAS®) Society Recommendations: 2018. World Journal of Surgery. 2019;43(3):659-95.
6. Shabanzadeh DM, Sørensen LT. Alcohol Consumption Increases Post-Operative Infection but Not Mortality: A Systematic Review and Meta-Analysis. Surgical Infections. 2015;16 (6):657-68.

Prehabilitation

INTRODUCTION

Surgical prehabilitation uses multimodal actions in the preoperative period aimed at helping the patient overcome the organic repercussion associated with surgical aggression, not only in the immediate postoperative period but also in the long term[1-3]. This model is based on trimodal prehabilitation, understanding this as the sum of physical therapy, nutritional supplements rich in proteins and cognitive therapy, with the aim of reducing the states of depression and anxiety associated with the process[4]. There is no consensus on the type of exercises that patients should perform. Current evidence supports the inclusion of education and physical exercise. The physical exercise program should include stamina buildup (aerobics), muscular strength (peripheral) and inspiratory muscle exercises[5-6]. Regarding education, respiratory physiotherapy and self-management exercises should be taught in the immediate postoperative period[1-5]. There is a definite consensus that the patient's prehabilitation should not defer surgery beyond four weeks, a time that seems adequate to achieve an improvement in functional capacity prior to surgery[6]. There is no consensus on functional assessment tests.

7. Trimodal prehabilitation therapy is recommended to improve functional capacity prior to surgery.

Moderate level of evidence. Strong recommendation.

REFERENCES

1. Hughes MJ, Hackney RJ, Lamb PJ, Wigmore SJ, Christopher Deans DA, Skipworth RJE. Prehabilitation Before Major Abdominal Surgery: A Systematic Review and Meta-analysis. World J Surg. 2019;43(7):1661-68.
2. Rosero ID, Ramírez-Vélez R, Lucia A, Martínez-Velilla N, Santos-Lozano A, Valenzuela PL, et al. Systematic Review and Meta-Analysis of Randomized, Controlled Trials on Preoperative Physical Exercise Interventions in Patients with Non-Small-Cell Lung Cancer. Cancers. 2019;11(7):944.
3. Chen X, Hou L, Zhang Y, Liu X, Shao B, Yuan B, et al. The effects of five days of intensive preoperative inspiratory muscle training on postoperative complications and outcome in

patients having cardiac surgery: a randomized controlled trial. Clin Rehabil. 2019; 33(5): 913-22.

4. Luther A, Gabriel J, Watson RP, Francis NK. The Impact of Total Body Prehabilitation on Post-Operative Outcomes After Major Abdominal Surgery: A Systematic Review. World J Surg. 2018; 42(9): 2781-91.
5. Milder DA, Pillinger NL, Kam PCA. The role of prehabilitation in frail surgical patients: A systematic review. Acta Anaesthesiol Scand. 2018; 62(10):1356-66.
6. Boden I, Skinner EH, Browning L, Reeve J, Anderson L, Hill C, et al. Preoperative physiotherapy for the prevention of respiratory complications after upper abdominal surgery: pragmatic, double blinded, multicentre randomised controlled trial. BMJ. 2018; 24: 360: j5916.

Preoperative nutritional evaluation

INTRODUCTION

Preoperative malnutrition is associated with increased postoperative morbidity and mortality and prolongs hospital stay for surgical patients[1]. For this reason, it is essential to carry out an outpatient nutritional screening for all patients undergoing scheduled major surgery. It is recommended to use nutritional screening tools that include body mass index (BMI), involuntary weight loss, reduction of recent food intake and the degree of stress or severity of the disease[2,3]. In patients at risk of malnutrition, a complete nutritional assessment should be carried out to diagnose it and initiate adequate nutritional treatment. Nowadays, the methodology used to diagnose malnutrition is based on the GLIM (Global Leadership Initiative on Malnutrition) criteria, where at least one phenotypic criterion must be met (weight loss, BMI, reduction in muscle mass) and one etiological criterion (reduction of nutritional intake / nutrient absorption, inflammatory state) [4]. (See annex 10.3: Nutritional evaluation algorithm).

Serum albumin and prealbumin or C-Reactive Protein (PCR) reflect the degree of systemic inflammation and are not specific to the nutritional status[3]. Although albumin is a predictor of postoperative morbidity and mortality, it is not useful for determining nutritional status since its levels are inversely altered with the degree of inflammation of the patient and change with hydration status[5].

8. Nutritional screening is recommended for all patients undergoing major surgery.

   Moderate level of evidence. Strong recommendation.

9. When a patient is identified at risk of malnutrition, a complete nutritional assessment should be carried out, establishing a nutritional treatment plan, monitoring tolerance and response to that plan.

   Moderate level of evidence. Strong recommendation.

REFERENCES

1. Arends J, Bachmann P, Baracos V, Barthelemy N, Hartmut B, Bozzetti F, et al. ESPEN guidelines on nutrition in cancer patients. Clin Nutr. 2017; 36:11-48.
2. Kondrup J, Allison SP, Elia M, Vellas B, Plauth M. Educational and Clinical Practice Committee, European Society of Parenteral and Enteral Nutrition (ESPEN). ESPEN guidelines for nutrition screening 2002. Clin Nutr. 2003; 22:415-21.

3. Jensen GL, Compher C, Sullivan DH, Mullin GE. Recognizing malnutrition in adults: definitions and characteristics, screening, assessment, and team approach. J Parenter Enteral Nutr. 2013; 37:802-7.
4. Cederholm T, Jensen GL, Correia MITD, Gonzalez MC. Fukushima R, Higashiguchi T, et al. GLIM criteria for the diagnosis of malnutrition: a consensus report from the global clinical nutrition community. Clin Nutr. 2019; 38:1-9.
5. White JV, Guenter P, Jensen G, Malone A, Schofield M, Academy Malnutrition Work Group; A.S.P.E.N. Malnutrition Task Force; A.S.P.E.N. Board of Directors. Consensus statement: academy of nutrition and Dietetics and American society for parenteral and enteral nutrition: characteristics recommended for the identification and documentation of adult malnutrition (undernutrition). J Parenter Enter Nutr. 2012; 36:275-83.

Preoperative nutritional intervention

INTRODUCTION

The benefit of preoperative nutritional treatment has been mainly demonstrated in patients at nutritional risk or severe malnutrition[1]. The administration of nutritional support (oral, enteral, parenteral) in patients with malnutrition or severe nutritional risk for at least 7-10 days before surgery is associated with a reduction in infectious complications and anastomotic dehiscence, as well as a shorter hospital stay[1,2,3].

10. All patients at severe nutritional risk or severe malnutrition should receive nutritional treatment at least 7-10 days before surgery. Oral / enteral route will be preferred, if possible.

Moderate level of evidence. Strong recommendation.

REFERENCES

1. Jie B, Jiang ZM, Nolan MT, Zhu SN, Yu K, Kondrup J. Impact of preoperative nutritional support on clinical outcome in abdominal surgical patients at nutritional risk. Nutrition. 2012; 28:1022-7.
2. Waitzberg DL, Saito H, Plank LD, Jamieson GG, Jagannath P, Hwang TL, et al Postsurgical infections are reduced with specialized nutrition support. World J Surg. 2006; 30:1592-1604.
3. Fukuda Y, Yamamoto K, Hirao N, Nishikawa K, Maeda S, Haraguchi N, et al. Prevalence of malnutrition among gastric cancer patients undergoing gastrectomy and optimal preoperative nutritional support for preventing surgical site infections. Ann Surg Oncol. 2015; (Suppl. 3):778-85.

Inmunonutrition

INTRODUCTION

Inmunonutrition (IN) has been at debate since the 1990s, especially in cancer surgery[1]. Some reviews and meta-analysis have shown the beneficial effects of IN by summing the results of RCTs in all types of patients and examining the entire perioperative period. However, other studies have found no added benefit with the use of IN over standard supplements using similar methods[2].

According to the ESPEN Clinical Guidelines on Clinical Nutrition and Surgery 2017, they should be administered. Specific formulas with immunonutrients in the peri- or at least post-operative stage in malnourished patients undergoing major surgery for cancer, with an intermediate grade of recommendation (SIGN, Scottish Intercollegiate Guidelines Network) [3]. And there is no clear evidence for its use compared to standard oral supplements exclusively in the preoperative period.

Meta-analyses continue to appear in this sense, with some common positive results, with evidence that is not always high[4,5].

11. There is insufficient evidence to recommend inmunonutrition versus the use of standard oral supplements exclusively in the preoperative period.

Low level of evidence. Weak recommendation.

1. Arends J, Bachmann P, Baracos V, et al. ESPEN guidelines on nutrition in cancer patients. Clin Nutr 2017; 36:11-48.
2. Hegazi RA, Hustead DS, Evans DC. Properative standard oral nutrition supplements vs immunonutrition: results of a systematic review and meta-analysis. J Am Coll Surg 2014; 219: 1078-1087.
3. Weimann A, Braga M, Carli F, Higashiguchi T, Hübner M, Klek S, et al. ESPEN guideline: Clinical nutrition in surgery. Clin Nutr 2017; 36: 623-650.
4. Probst P, Ohmann S, Klaiber U, Hüttner FJ, Billeter AT, Ulrich A, Büchler MW, Diener MK. Meta-analysis of immunonutrition in major abdominal surgery. Br J Surg 2017; 104: 1594-1608.
5. Adiamah A, Skorepa P, Weimann A, Lobo DN. The impact of preoperative immune modulating nutrition on outcomes in patients undergoing surgery for gastrointestinal surgery for gastrointestinal cancer. Ann Surg 2019; 270: 247-256.

Evaluation and treatment of anemia

INTRODUCTION

Anemia is the main independent risk factor for morbidity and mortality in both scheduled and urgent surgery. This relationship holds even in mild cases. Low hemoglobin levels are associated with an increased incidence of care-associated or nosocomial infections. The incidence of preoperative anemia is around 20-30%. Hemoglobin level is inversely related to the risk of receiving allogeneic transfusion[1-5].

Blood transfusion is associated, with a dose-dependent effect, to a higher risk of nosocomial infection, thromboembolic episodes, reinterventions, readmission, longer ICU, and hospital stay, and an even higher postoperative mortality rate. Multiple observational studies, international data bases, and recently various meta-analysis that analyze patients undergoing general surgery, but especially different digestive cancer surgeries (colon, rectum, gastric and liver carcinoma), in orthopedic, vascular, and cardiovascular surgery show a relationship of transfusion with higher morbidity and mortality, reintervention and readmission. In addition, blood transfusion has also been associated with a higher incidence of tumor recurrence or relapse, refractivity to treatment and abdominal neoplastic disease related mortality.

For these reasons, different National Scientific Societies (SEDAR) and international ones (SABM), the Board of the European Association of Anesthesiology (ESA)[6] and NICE recommend assessing and treating preoperative anemia and even delaying or rescheduling surgery for patients with anemia. All National Consensus Documents (“Sevilla”)[4] or international ones (“Frankfurt”)[5] issue a strong recommendation to screen and treat

preoperative anemia with the highest level of evidence. This recommendation has been endorsed by the Ministerio de Sanidad y Bienestar Social since 2013 in the “Protecto de Compromiso de las Sociecades Científicas con la Calidad”.

The State of Western Australia and the Australian National Blood Authority) have promoted a campaign to empower patients so that they know the need to be fit before surgery and the importance that their general practitioners’ study and treat their anemia and iron deficiency, and that no patient arrives with anemia to operating room[1,7].

12. It is suggested that as soon as a patient enters the surgical waiting list or from the moment the surgical indication is performed, the possible appearance of anemia or any blood deficit is monitored, its study and adequate management.

(This management may be carried out by your general practitioner, family doctor, referral specialist, the surgical team, or the case coordinator, depending on the local organization[1,2,3,7])

Low level of evidence. Weak recommendation.

REFERENCES

1. Fit For Surgery: Managing Iron Deficiency Anaemia. Resources for general practitioners managing patients undergoing planned surgery. Australia National Blood Authority. https://www.blood.gov.au/fit-surgery-managing-iron-deficiency-anaemia (último acceso febrero 2020).
2. Supporting Patient Blood Management (PBM) in the EU. A Practical Implementation Guide for Hospitals. Directorate-General for Health and Food Safety. European Commission. 2017. https://ec.europa.eu/health/sites/health/files/blood_tissues_organs/docs/2017_eupbm_hospitals_en.pdf (último acceso febrero 2020).
3. Ripollés-Melchor J, Jericó-Alba C, Quintana-Díaz M, García-Erce JA. From blood saving programs to patient blood management and beyond. Med Clin (Barc). 2018;151(9):368-373.
4. Actualización del Documento de Sevilla de Alternativas a la Transfusión Sanguínea. Congreso SETS. Madrid, Junio 2019.
5. Mueller MM, Van Remoortel H, Meybohm P, et al. ICC PBM Frankfurt 2018 Group.Patient Blood Management: Recommendations from the 2018 Frankfurt Consens Conference. JAMA. 2019;321:983-97.
6. European Board of Anaesthesiology (EBA) recommendations for Preoperative anaemia and Patient Blood Management. http://www.eba-uems.eu/resources/PDFS/safety-guidelines/EBA--Preop-anaemia-recommend.pdf (último acceso febrero 2020).
7. Minck S, Robinson K, Saxon B et al. Patient blood management -- the GP’s guide. Aust Fam Physician 2013;42:291-7.

Patient Blood Management (PBM) programs for anemia management

INTRODUCTION

In the fifth edition of the Standards of the Transfusion Accreditation Committee[1] of the Spanish Societies of Hematology and Hemotherapy (SEHH) and Blood Transfusion (SETS) it is included in the Transfusion section in point 4.2.5. that "Transfusion Services must promote and participate in hospital Patient Blood Management (PBM) programs." The World Health Organization in its 63rd Assembly in June 2010 urged all countries to launch Patient Blood Management Programs[2]. This recommendation has been

endorsed by the European Commission since 2013, ratified with the Guidelines for Good Practice Recommendations in Patient Blood Management published in April 2017[3-4]. We consider it necessary to integrate these PBM programs into multimodal prehabilitation programs[5-7].

13. The implementation of "Patient Blood Management" Programs is recommended in all hospitals and health areas. We suggest the PBM Program be integrated with the ERAS protocols.

High level of evidence. Strong recommendation.

REFERENCES

1. Estándares del Comité de Acreditación de Transfusión (CAT). 5ª Edición. Junio 2019 http://www.catransfusion.es/media/upload/arxius/estandares/ESTANDARES%202019.pdf (último acceso febrero 2020).
2. Global Forum for Blood Safety: Patient Blood Management. World Health Organization. https://www.who.int/bloodsafety/events/gfbs_01_pbm_concept_paper.pdf (último acceso febrero 2020).
3. Supporting Patient Blood Management (PBM) in the EU. A Practical Implementation Guide for Hospitals. Directorate-General for Health and Food Safety. European Commission. 2017. https://ec.europa.eu/health/sites/health/files/blood_tissues_organs/docs/2017_eupbm_hospitals_en.pdf (último acceso febrero 2020).
4. Building national programmes of Patient Blood Management (PBM) in the EU. A Guide for Health Authorities. European Commission. 2017. https://ec.europa.eu/health/sites/health/files/blood_tissues_organs/docs/2017_eupbm_authorities_en.pdf (último acceso febrero 2020).
5. Casans Francés R, J. Ripollés Melchor, J.M. Calvo Vecino. Grupo Español de Rehabilitación Multimodal GERM/ERAS-Spain. ¿Es hora de integrar el manejo sanguíneo del paciente en los protocolos de rehabilitación quirúrgica intensificada? Rev Esp Anestesiol Reanim. 2015;62(2):61-3.
6. García Erce JA, Laso Morales MJ. «Patient blood management» en la Vía Clínica de Recuperación Intensificada en Cirugía Abdominal. Cir Esp 2017; 95(9):552-554.
7. Ripollés-Melchor J, Jericó-Alba C, Quintana-Díaz M, García-Erce JA. From blood saving programs to patient blood management and beyond. Med Clin (Barc). 2018;151(9):368-373.

Delay or suspension of surgery in anemic patients

INTRODUCTION

There is a correlation between preoperative anemia and risk of postoperative mortality, morbidity, postoperative quality, and increased risk of transfusion[1]. The Spanish Society of Anesthesiology, Resuscitation and Pain Therapy (SEDAR) [2] among its recommendations DO NOT DO of the Project of the Ministry of Health Commitment to the Quality of Scientific Societies makes this recommendation not to program patients with anemia[3]. Years later, the Board of the European Society of Anesthesia and the Society for Advancement of Blood Management (SABM) have made the same recommendation[4,5].

14. It is recommended not to schedule elective surgery with risk of bleeding in patients with anemia until proper diagnostic study and treatment are carried out.

High level of evidence. Strong recommendation.

REFERENCES

1. García Erce JA, Laso Morales MJ. «Patient blood management» en la Vía Clínica de Recuperación Intensificada en Cirugía Abdominal. Cirugía Española 2017;95(9):552-554.
2. Quecedo Gutiérrez L, Ruiz Abascal R, Calvo Vecino JM, Peral García AI, Matute González E, Muñoz Alameda LE, et al. "Do not do" recommendations of the Spanish Society of Anaesthesiology, Critical Care and Pain Therapy. "Commitment to Quality by Scientific Societies" Project. Rev Esp Anestesiol Reanim. 2016;63(9):519-527. doi: 10.1016/j.redar.2016.05.002.
3. Recomendaciones de «no hacer» de la Sociedad Española de Anestesiología, Reanimación y Terapéutica del Dolor. Proyecto «compromiso por la calidad de las sociedades científicas». https://www.mscbs.gob.es/organizacion/sns/planCalidadSNS/pdf/SOCIEDAD_ESP_ANEST_REA_TE_DOLOR_0K.pdf (último acceso junio 2020).
4. Burns CD, Brown JP, Corwin HL, Gross I, Ozawa SJ, Shander A. Special Report From the Society for the Advancement of Blood Management: The Choosing Wisely Campaign. Anesth Analg. 2019;129:1381-1386. doi: 10.1213/ANE.0000000000004415. PMID: 31517679.
5. Choosing wisely. Society for the Advancement of Blood Management. "Don't proceed with elective surgery in patients with properly diagnosed and correctable anemia until the anemia has been appropriately treated". July 23, 2018. http://www.choosingwisely.org/societies/society-for-the-advancement-of-blood-management/ (ultimo acceso junio 2020).

Determination of hemoglobin level with time frame

INTRODUCTION

A low hemoglobin level is associated with an increase in perioperative morbidity and mortality, and the risk of receiving allogeneic transfusion[1-6]. The study and treatment of preoperative anemia is essential to optimize the clinical results of patients scheduled for cardiac[3] and non-cardiac[6] surgery. The detection and treatment of preoperative anemia is recommended with enough time for its adequate study and treatment[2-7].

15. At least one hemoglobin (Hb) determination is recommended in patients undergoing elective surgery, at least 28 days before surgery or the invasive process[2,4,5,6,7] (ideally between 6 8 weeks or from the time of surgical indication)".

Moderate level of evidence. Strong recommendation.

16. It is recommended that in cancer surgery cases the entire time available from diagnosis to the time of surgery be used to detect anemia and correct it, or at least improve hemoglobin concentration[5,6].

Moderate level of evidence. Strong recommendation.

REFERENCES

1. Fowler AJ, Ahmad T, Phull MK, Allard S, Gillies MA, Pearse RM. Meta-analysis of the association between pre-operative anaemia and mortality after surgery. British Journal of Surgery 2015; 102: 1314-24.
2. Muñoz M, Gómez-Ramírez S, Kozek-Langeneker S, Shander A, Richards T, Pavía J, et al. Fit to fly': overcoming barriers to preoperative haemoglobin optimization in surgical patients. Br J Anaesth. 2015;115:15-24. doi: 10.1093/bja/aev165

3. Klein AA, Collier TJ, Brar MS, Evans C, Hallward G, Fletcher SN, Richards T; Association of Cardiothoracic Anaesthetists (ACTA). The incidence and importance of anaemia in patients undergoing cardiac surgery in the UK-the first Association of Cardiothoracic Anaesthetists national audit. Anaesthesia 2016;71:627-35.
4. Muñoz M, Acheson AG, Auerbach M, Besser M, Habler O, Kehlet H, et al. International consensus statement on the peri-operative management of anaemia and iron deficiency. Anaesthesia. 2017;72:233-47.
5. Gustafsson UO, Scott MJ, Hubner M, Nygren J, Demartines N, Francis N, et al. Guidelines for Perioperative Care in Elective Colorectal Surgery: Enhanced Recovery After Surgery (ERAS®) Society Recommendations: 2018. World J Surg. 2019;43:659-95.
6. De Hert S, Staender S, Fritsch G, Hinkelbein J, Afshari A, Bettelli G, et al. Pre-operative evaluation of adults undergoing elective noncardiac surgery: Updated guideline from the European Society of Anaesthesiology. Eur J Anaesthesiol. 2018;35:407-65.
7. Quecedo Gutiérrez L, Ruiz Abascal R, Calvo Vecino JM, Peral García AI, Matute González E, Muñoz Alameda LE, et al. “Do not do” recommendations of the Spanish Society of Anaesthesiology, Critical Care and Pain Therapy. “Commitment to Quality by Scientific Societies” Project. Rev Esp Anestesiol Reanim. 2016;63:519-527. doi: 10.1016/j.redar.2016.05.002.

Hemoglobin level 13 g / dL

INTRODUCTION

In 2004 and 2006, Guralnik and Beutler proposed raising the minimum level of hemoglobin (Hb)> 12.2 g / dL in women of childbearing age, from 13.7 to 60 years and then to 13.2 g / dL. in Caucasian males[1]. Other authors propose raising it to 13.5 g / L in men and women after menopause[1]. A recent national epidemiological study in the mobile population shows that the mean Hb levels of women from 50 years of age increase to values close to those of men from 70 years of age[2].

Recent studies have shown that the status of iron metabolism in women with Hb between 12 and 13 g / dL is more like those theoretically anemic (Hb <12 g / dL) than to those with Hb greater than 133.4.

Epidemiological studies show almost double risk of transfusion and complications in women without anemia, but with an Hb level between 12 and 13 g / dL3.5. Women with an Hb level below 13 may continue to be discriminated against men.

The different consensus documents recommend raising the hemoglobin value of women to 13 g / dL6.7.

17. It is recommended that the preoperative Hb concentration before surgical intervention is above 13 g / dl, regardless of gender / sex.

Moderate level of evidence. Strong recommendation.

REFERENCES.

1. Rivilla Marugán L, Lorente Aznar T, Molinero Rodriguez M, García-Erce JA. [Anaemia and the elderly: Critical review of its definition and prevalence]. Rev Esp Geriatr Gerontol. 2019;54(4):189-194. doi: 10.1016/j.regg.2019.02.008.
2. García-Erce JA, Lorente-Aznar T, Rivilla-Marugán L. Influence of gender, age and residence altitude on haemoglobin levels and the prevalence of anaemia. Med Clin (Barc). 2019. pii: S0025-7753(19)30125-3. doi: 10.1016/j.medcli.2019.02.002.

3. Butcher A, Richards T, Stanworth SJ, Klein AA. Diagnostic criteria for pre-operative anaemia-time to end sex discrimination. Anaesthesia. 2017;72:811-4.

4. Muñoz M, Laso-Morales MJ, Gómez-Ramírez S, Cadellas M, Núñez-Matas MJ, García-Erce JA., Pre-operative haemoglobin levels and iron status in a large multicentre cohort of patients undergoing major elective surgery. Anaesthesia. 2017;72:826-34.

5. Muñoz M, Gómez-Ramírez S, Auerbach M. Stimulating erythropoiesis before hip fracture repair for reducing blood transfusion: should we change the hemoglobin cutoff level for defining anemia in females? Transfusion. 2016;56:2160-3.

6. Muñoz M, Acheson AG, Bisbe E, Butcher A, Gómez-Ramírez S, Khalafallah AA, et al. An international consensus statement on the management of postoperative anaemia after major surgical procedures. Anaesthesia. 2018;73:1418-1431. doi: 10.1111/anae.14358.7.-Documento consenso GERM Salamanca (Fit for Surgery. Documento de trabajo. Estudio Delphi. Grupo Español de Rehabilitación Multimodal. III Congreso GERM. Salamanca, abril 2018) (pendiente publicación).

Screening for iron deficiency

INTRODUCTION

Iron deficiency is the most common etiology of anemia[1-4]. It is the world's most prevalent cause of anemia. It is the most common cause of preoperative anemia[1,2]. Up to one third of patients without apparent anemia have iron deficiency and another third of patients have sufficient reserves to recover after perioperative bleeding[1,2]. Furthermore, iron deficiency has been associated with a higher risk of transfusion and / or nosocomial infection in repair surgery for hip fracture[5], cardiac or colon cancer resection[6].

The NICE, in its quality standards for blood transfusion [QS138] urges the offer of iron supplements, before and after surgery, to all patients with iron deficiency anemia[7-8]. It is also necessary to consider the possibility of parenteral treatment in cases of clear oral intolerance.

18. Detection and treatment of perioperative iron deficiency is recommended.

Moderate level of evidence. Strong recommendation.

REFERENCES

1. Muñoz M, Laso-Morales MJ, Gómez-Ramírez S, Cladellas M, Núñez-Matas MJ, García-Erce JA. Pre-operative haemoglobin levels and iron status in a large multicentre cohort of patients undergoing major elective surgery. Anaesthesia 2017;72:826-834. doi: 10.1111/anae.13840.

2. Muñoz M, Gómez-Ramírez S, Besser M, Pavía J, Gomollón F, Liumbruno GM, et al. Current misconceptions in diagnosis and management of iron deficiency. Blood Transfus 2017;15:422-437. doi: 10.2450/2017.0113-17.

3. De Hert S, Staender S, Fritsch G, Hinkelbein J, Afshari A, Bettelli G, et al. Pre-operative evaluation of adults undergoing elective non-cardiac surgery: Updated guideline from the European Society of Anaesthesiology. Eur J Anaesthesiol. 2018;35:407-65.

4. Althoff FC, Neb H, Herrmann E, Trentino KM, Vernich L, Füllenbach C, at al. Multimodal patient blood management program based on a three-pillar strategy: a systematic review and meta-analysis. Ann Surg 2019;269:794-804.

5. Izuel Rami M, García Erce JA, Gómez-Barrera M, Cuenca Espiérrez J, Abad Sazatornil R, Rabanaque Hernández MJ. Relación entre la transfusión de sangre alogénica, la deficiencia

de hierro y la infección nosocomial en pacientes con fractura de cadera. Med Clin (Barc). 2008;131:647-52.

6. Rössler J, Schoenrath F, Seifert B, Kaserer A, Spahn GH, Falk V, Spahn DR. Iron deficiency is associated with higher mortality in patients undergoing cardiac surgery: a prospective study. Br J Anaesth. 2020;124:25-34. doi: 10.1016/j.bja.2019.09.016.
7. Harju E. Empty iron stores as a significant risk factor in abdominal surgery. JPEN J Parenter Enteral Nutr. 1988;12:282-5. doi: 10.1177/0148607188012003282. PMID: 3392823.
8. "People with iron-deficiency anaemia who are having surgery are offered iron supplementation before and after surgery". Blood transfusion. Quality standard [QS138] Published date: December 2016 https://www.nice.org.uk/guidance/qs138 https://www.nice.org.uk/guidance/qs138/chapter/Quality-statement-1-Iron-supplementation (último acceso febrero 2020).

Treatment of preoperative anemia

INTRODUCTION

The study of anemia and specific treatment of any preoperative anemia should be carried out as soon as possible or as soon as possible[1-5].

The immediate perioperative treatment of anemia in orthopedic[4,5] or cardiac[6,7,8] surgery patients is not only associated with a lower transfusion rate, but also with a lower incidence of adverse effects and a reduction in hospital stay, in addition to better post-operative hematological parameters.

19. The detection and treatment of preoperative anemia is recommended, even in cases of preferential or urgent surgeries.

Moderate level of evidence. Strong recommendation.

REFERENCES

1. Kotzé A, Harris A, Baker C, Iqbal T, Lavies N, Richards T, et al. British Committee for Standards in Haematology Guidelines on the Identification and Management of Pre-Operative Anaemia. Br J Haematol. 2015;171:322-31. doi: 10.1111/bjh.13623.
2. Muñoz M, Gómez-Ramírez S, Kozek-Langeneker S, Shander A, Richards T, Pavía J, et al. 'Fit to fly': overcoming barriers to preoperative haemoglobin optimization in surgical patients. Br J Anaesth 2015;115:15-24.
3. Muñoz M, Gómez-Ramírez S, Campos A, Ruiz J, Liumbruno GM. Pre-operative anaemia: prevalence, consequences and approaches to management. Blood Transfus. 2015;13:370-9.
4. Gómez-Ramírez S, Maldonado-Ruiz MÁ, Campos-Garrigues A, Herrera A, Muñoz M. Short-term perioperative iron in major orthopedic surgery: state of the art. Vox Sang 2019;114:3-16.
5. Muñoz M, Gómez-Ramírez S, Cuenca J, García-Erce JA, Iglesias-Aparicio D, Haman-Alcober S, et al. Very-short-term perioperative intravenous iron administration and postoperative outcome in major orthopedic surgery: a pooled analysis of observational data from 2547 patients. Transfusion. 2014;54:289-99.
6. Spahn D, Schoenrath F, Spahn GH, Seifert B, Stein P, Theusinger OM, et al. Effect of ultra-short-term treatment of patients with iron deficiency or anaemia undergoing cardiac surgery: a prospective randomised trial. Lancet. 2019;393:2201-12.

7. Weltert L, Rondinelli B, Bello R, Falco M, Bellisario A, Maselli D, et al. A single dose of erythropoietin reduces perioperative transfusions in cardiac surgery: results of a prospective single-blind randomized controlled trial. Transfusion. 2015;55:1644-54.
8. Yoo YC, Shim JK, Kim JC, Jo YY, Lee JH, Kwak YL. Effect of single recombinant human erythropoietin injection on transfusion requirements in preoperatively anemic patients undergoing valvular heart surgery. Anesthesiology 2011;115: 929-37.

Oral iron treatment

INTRODUCTION

The treatment of choice for iron deficiency and mild anemia is conventional oral iron if sufficient time is available and there is no contraindication. The administration of low daily doses (40-60mg) or moderate doses every other day (80-100mg) is recommended. There is no evidence that higher doses lead to greater absorption, and, on the other hand, they are associated with a higher rate of digestive adverse effects[1-7].

20. Oral iron treatment is recommended in cases of iron deficiency or mild-moderate iron deficiency anemia if there is at least 6 weeks until surgery.

Low level of evidence. Strong recommendation.

REFERENCES

1. Muñoz M, Acheson AG, Bisbe E, Butcher A, Gómez-Ramírez S, Khalafallah AA, et al. An international consensus statement on the management of postoperative anaemia after major surgical procedures. Anaesthesia. 2018;73:1418-1431. doi: 10.1111/anae.14358.
2. Stoffel NU, Cercamondi CI, Brittenham G, Zeder C, Geurts-Moespot AJ, Swinkels DW, et al. Iron absorption from oral iron supplements given on consecutive versus alternate days and as single morning doses versus twice-daily split dosing in iron-depleted women: two open-label, randomised controlled trials. Lancet Haematol. 2017; e524-e533. doi: 10.1016/S2352-3026(17)30182-5.
3. Moretti D, Goede JS, Zeder C, Jiskra M, Chatzinakou V, Tjalsma H, et al. Oral iron supplements increase hepcidin and decrease iron absorption from daily or twice-daily doses in iron-depleted young women. Blood. 2015;126:1981-9.
4. Muñoz M, Acheson AG, Auerbach M, Besser M, Habler O, Kehlet H, et al. International consensus statement on the peri-operative management of anaemia and iron deficiency. Anaesthesia. 2017;72:233-47.
5. Muñoz M, Gómez-Ramírez S, Besser M, Pavía J, Gomollón F, et al. Current misconceptions in diagnosis and management of iron deficiency. Blood Transfus. 2017;15:422-37.
6. Jericó Alba C, Garcia Erce JA. Hiero oral como tratamiento de la ferropenia: ¿debe ser siempre la primera elección? Med Clin (Barc). 2018;151:e27-e28.
7. García Erce JA, Altés A, López Rubio M, Remacha AF; en representación del Grupo Español de Eritropatología de la Sociedad Española de Hematología y Hemoterapia. Management of iron deficiency in various clinical conditions and the role of intravenous iron: Recommendations of the Spanish Erythropathology Group of the Spanish Society of Haematology and Haemotherapy. Rev Clin Esp. 2020;220:31-42.

Intravenous iron treatment

INTRODUCTION

If there is little time before surgery, resistance or intolerance to oral iron, contraindication to it, the presence of inflammation or moderate-severe anemia or concomitant treatment with erythropoietic agents, the treatment of choice for iron deficiency and anemia associated with iron deficiency is intravenous iron at high doses[1-7].

21. Preoperative treatment with intravenous iron (FEEV) is recommended in potentially bleeding elective surgery patients with iron deficiency anemia and / or functional iron deficiency, to improve hemoglobin levels and / or reduce the transfusion rate.

Moderate level of evidence. Strong recommendation.

22. We recommend the administration of intravenous iron, instead of oral iron, in those cases in which this is contraindicated or the time available until surgery is insufficient.

Moderate level of evidence. Strong recommendation.

REFERENCES

1. Banerjee S, McCormack S. Intravenous iron preparations for patients undergoing elective surgery: a review of clinical effectiveness, cost-effectiveness, and guidelines. Ottawa: CADTH; 2019 Mar. (CADTH rapid response report: summary with critical appraisal). https://www.cadth.ca/sites/default/files/pdf/htis/2019/RC1088%20Intravenous%20 Iron%20Final.pdf (último acceso febrero 2020).
2. Calleja JL, Delgado S, del Val A, Hervás A, Larraona JL, Terán Á, et al; Colon Cancer Study Group. Ferric carboxymaltose reduces transfusions and hospital stay in patients with colon cancer and anemia. Int J Colorectal Dis. 2016;31:543-551.
3. Froessler B, Palm P, Weber I, Hodyl NA, Singh R, Murphy EM. The Important Role for Intravenous Iron in Perioperative Patient Blood Management in Major Abdominal Surgery: A Randomized Controlled Trial. Ann Surg. 2016;264:41-6.
4. García Erce JA, Altés A, López Rubio M, Remacha AF; en representación del Grupo Español de Eritropatología de la Sociedad Española de Hematología y Hemoterapia. Management of iron deficiency in various clinical conditions and the role of intravenous iron: Recommendations of the Spanish Erythropathology Group of the Spanish Society of Haematology and Haemotherapy. Rev Clin Esp. 2020;220:31-42. doi: 10.1016/j.rce.2019.09.004
5. Keeler BD, Dickson EA, Simpson JA, Ng O, Padmanabhan H, Brookes MJ, et al; IVICA Trial Group. The impact of pre-operative intravenous iron on quality of life after colorectal cancer surgery: outcomes from the intravenous iron in colorectal cancer-associated anaemia (IVICA) trial. Anaesthesia. 2019;74:714-725.
6. Laso-Morales M, Jericó C, Gómez-Ramírez S, Castellví J, Viso L, Roig-Martínez I, et al. Preoperative management of colorectal cancer-induced iron deficiency anemia in clinical practice: data from a large observational cohort. Transfusion. 2017;57:3040-3048. doi: 10.1111/trf.14278.
7. Schack A, Berkfors AA, Ekeloef S, Gögenur I, Burcharth J. The Effect of Perioperative Iron Therapy in Acute Major Non-cardiac Surgery on Allogenic Blood Transfusion and Postoperative Haemoglobin Levels: A Systematic Review and Meta-analysis. World J Surg. 2019;43:1677-1691. doi: 10.1007/s00268-019-04971-7. PMID: 30824959

8. Blood transfusion. Quality standard [QS138] Published date: December 2016 https://www.nice.org.uk/guidance/qs138 (último acceso febrero 2020).

Treatment with erythropoietic agents

INTRODUCTION

The administration of alpha-erythropoietin is recommended for the treatment of non-deficiency anemia preoperatively after orthopedic surgery[1]. Different experiences demonstrate the benefit of short regimens or even single doses in orthopedic arthroplasty surgery and hip fracture surgery[2,3]. A recent Swiss experience demonstrates its benefit, in combination with EV iron and vitamin complexes, in cardiac surgery[4].

Different meta-analyzes, consensus documents and guidelines from scientific societies recommend the administration of erythropoietic agents, together with intravenous iron, in surgical patients with non-iron deficiency anemia[5-8].

23. The administration of rHuEPO is recommended in elective orthopedic surgery patients at risk of moderate-high bleeding and moderate non-deficiency anemia (Hb between 10 and 13 g / dL), to reduce allogeneic blood transfusion.

High level of evidence. Strong recommendation.

24. The administration of rHuEPO is suggested to reduce the transfusion rate in anemic patients undergoing major elective surgery other than elective orthopedic surgery with a moderate-high risk of bleeding.

Moderate level of evidence. Weak recommendation

REFERENCES

1. Kei T, Mistry N, Curley G, et al. Efficacy and safety of erythropoietin and iron therapy to reduce red blood cell transfusion in surgical patients: a systematic review and meta-analysis, Can J Anesth 2019. doi.org/10.1007/s12630-019-01351-6.

2. Theusinger OM, Kind SL, Seifert B, Borgeat L, Gerber C, Spahn DR. Patient blood management in orthopaedic surgery: a four-year follow-up of transfusion requirements and blood loss from 2008 to 2011 at the Balgrist University Hospital in Zurich, Switzerland. Blood Transfus. 2014;12:195-203.

3. Muñoz M, Gómez-Ramírez S, Cuenca J, García-Erce JA, Iglesias-Aparicio D, Haman-Alcober S, et al. Very-short-term perioperative intravenous iron administration and postoperative outcome in major orthopedic surgery: a pooled analysis of observational data from 2547 patients. Transfusion. 2014;54:289-99. doi: 10.1111/trf.12195.

4. Spahn D, Schoenrath F, Spahn GH, Seifert B, Stein P, Theusinger OM, et al. Effect of ultra-short-term treatment of patients with iron deficiency or anaemia undergoing cardiac surgery: a prospective randomised trial. Lancet. 2019;393:2201-12.

5. French CJ, Glassford NJ, Gantner D, Higgins AM, Cooper DJ, Nichol A, et al. Erythropoiesis-stimulating agents in critically ill trauma patients: a systematic review and meta-analysis. Ann Surg. 2017;265:54-62.

6. De Hert S, Staender S, Fritsch G, Hinkelbein J, Afshari A, Bettelli G, et al. Pre-operative evaluation of adults undergoing elective noncardiac surgery: Updated guideline from the European Society of Anaesthesiology. Eur J Anaesthesiol. 2018;35:407-65.

7. Task Force on Patient Blood Management for Adult Cardiac Surgery of the European Association for Cardio-Thoracic Surgery (EACTS) and the European Association of Cardiothoracic Anaesthesiology (EACTA). Boer C, Meesters MI, Milojevic M, Benedetto U, Bolliger D, von Heymann C, et al. 2017 EACTS/EACTA Guidelines on patient blood management for adult cardiac surgery. J Cardiothorac Vasc Anesth. 2018;32:88-120.

8. Documento Consenso GERM Salamanca (Fit for Surgery. Documento de trabajo. Estudio Delphi. Grupo Español de Rehabilitación Multimodal. III Congreso GERM. Salamanca, abril 2018) (Documento Salamanca Abril 2018).

Thromboprophylaxis

INTRODUCTION

Thromboembolic disease is an important complication of major surgical procedures in patients who do not receive prophylaxis, reaching 20%, for those undergoing general surgery, 30% for colorectal surgery, between 30-50% in surgery orthopedic hip fracture and neurosurgery and between 0-26% in head and neck surgery The application of different thromboprophylaxis measures has shown a reduction in thrombotic risk, with a different degree of efficiency and safety. Depending on the thrombotic risk, mechanical and pharmacological, subcutaneous, or oral methods may be combined[1,2,3,4,5]. The minimum duration will be seven days or until the start of walking. In those surgeries of greater risk, it should last between three to six weeks.

Pharmacological prophylaxis significantly reduces the incidence of thromboembolic disease. Unfractionated heparin (UFH) and low molecular weight heparins (LMWH) are equally effective for the prevention of deep vein thrombosis and pulmonary thromboembolism, although the use of LMWH is preferred over UFH postoperatively in most patients. Most of the surgical indications, due to a similar effect, but greater ease of administration and fewer bleeding complications.

25. The use of thromboprophylaxis is recommended in all patients undergoing major surgery or hospitalized for an acute medical condition.

Moderate level of evidence. Strong recommendation.

26. In general, it is recommended to maintain antithrombotic prophylaxis for a minimum of 7 days or until the patient is ambulation.

High level of evidence. Strong recommendation.

27. In the case of major abdominal surgery, prophylaxis will be extended up to 4 weeks after surgery.

Moderate level of evidence. Strong recommendation.

<u>Specific situations</u>:

1) *In general, urological, gynecological and neurosurgery surgery: 8 days; in case of immobilization of the patient, it should be prolonged until ambulation.*

2) *In general, urological, and gynecological surgery in cancer patients: 4 weeks (28 days).*

3) *In hip surgery: 4-6 weeks (28-42 days).*

4) In knee surgery: 3-4 weeks (21-28 days).

28. Early mobilization and the use of elastic compression stockings are recommended for the duration of the immobilization period.

High level of evidence. Strong recommendation.

29. Compression stockings are effective in preventing thromboembolic disease in surgical patients, reducing the risk even more when combined with pharmacological agents.

High level of evidence. Strong recommendation.

30. Intermittent pneumatic compression devices reduce the incidence of deep vein thrombosis. The method combined with pharmacological measures is recommended, mainly for neurosurgical patients and / or surgeries with high VTE risk.

Moderate level of evidence. Strong recommendation.

31. Prophylaxis regimens include direct acting oral anticoagulants (dabigatran, apixaban, rivaroxaban) or low molecular weight heparins (enoxaparin, bemiparin, tinzaparin).

High level of evidence. Strong recommendation.

REFERENCES

1. Felder S, Rasmussen MS, King R, Sklow B, Kwaan M, Madoff R, et al. Prolonged thromboprophylaxis with low molecular weight heparin for abdominal or pelvic surgery. Cochrane Database Syst Rev. 2019;3:CD004318. doi: 10.1002/14651858.CD004318.
2. Vivas D, Roldán I, Ferrandis R, Marín F, Roldán V, Tello-Montoliu A, et al. Perioperative and Periprocedural Management of Antithrombotic Therapy: Consensus Document of SEC, SEDAR, SEACV, SECTCV, AEC, SECPRE, SEPD, SEGO, SEHH, SETH, SEMERGEN, SEMFYC, SEMG, SEMICYUC, SEMI, SEMES, SEPAR, SENEC, SEO, SEPA, SERVEI, SECOT and AEU. Rev Esp Cardiol. 2018; 71:553-564.
3. Falck-Ytter Y, Francis CW, Johanson NA, Curley C, Dahl OE, Schulman S, et al: Antithrombotic Therapy and Prevention of Thrombosis, 9th ed: American College of Chest Physicians Evidence-Based Clinical Practice Guidelines. Chest. 2012 Feb;141(2 Suppl):e278S-e325S.
4. Anderson DR, Morgano GP, Bennett C, Dentali F, Francis CW, Garcia DA, et al American Society of Hematology 2019 guidelines for management of venous thromboembolism: prevention of venous thromboembolism in surgical hospitalized patients. Blood Adv. 2019;3: 3898-3944.
5. Afshari A, Fenger-Eriksen C, Monreal M, Verhamme P; ESA VTE Guidelines Task Force. European guidelines on perioperative venous thromboembolism prophylaxis: Mechanical prophylaxis. Eur J Anaesthesiol. 2018; 35:112-11.

Hygiene-Bathe

INTRODUCTION

Bathing the night before surgery has shown its effectiveness in preventing surgical site infection, according to the first edition of the Via RICA.

The importance of bathing or showering the night before surgery is an accepted fact, as is the reduction in the number of bacterial colonies due to bathing[1-3]. However, according to different clinical practice guidelines, the evidence is moderate[4].

32. A full bath is recommended prior to surgery.

Moderate level of evidence. Strong recommendation.

REFERENCES

1. Webster J, Osborne S. Preoperative bathing or showering with skin antiseptics to prevent surgical site infection. Cochrane Database of Systematic Reviews 2012, Issue 9. Art. No.:CD004985.
2. Kamel C, McGahan L, Polisena J, Mierzwinski-Urban M, Embil JM. Preoperative skin antiseptic preparations for preventing surgical site infections: a systematic review. Infect Control Hosp Epidemiol 2012;33(6):608-17.
3. Kamel C, McGahan L, Mierzwinski-Urban M, Embil J. Preoperative Skin Antiseptic Preparations and Application Techniques for Preventing Surgical Site Infections: A Systematic Review of the Clinical Evidence and Guidelines [Internet]. Ottawa: Canadian Agency for Drugs and Technologies in Health; 2011 (Rapid Response Report: Systematic Review). 2011. Jun. [about. 68 p.]. [cited 2014-11-Mar]. Disponible en: http://www.ca (Otros estudios de interés sobre este tema113,114).
4. Berríos-Torres SI, Craig A, Umscheid MD.. Centers for Disease Control and Prevention Guideline for the Prevention of Surgical Site Infection, 2017. JAMA Surg. 2017 Aug 1;152(8):784-791. doi: 10.1001/jamasurg.2017.0904.

Preoperative fasting

INTRODUCTION

There is no scientific evidence to confirm that the administration of clear liquids 2 hours before an elective surgical procedure causes a greater risk of aspiration, regurgitation or morbidity than fasting after midnight, since in most patients the stomach takes between 60-90 minutes to empty liquids[1,2]. Several randomized controlled studies have shown that the ingestion of clear liquids up to 2 hours and light solids up to 6 hours before anesthetic induction is safe and improves the patient's feeling of well-being[3]. These studies have shown that there are no significant differences in relation to gastric volume or the pH of gastric content when night fasting is compared with the ingestion of clear liquids up to 2 hours before surgery[3].

In patients with documented delayed gastric emptying, gastrointestinal motility disorders, or with urgent surgery, the administration of clear liquids 2 hours before surgery may not be safe. There is evidence that shows that patients with type 2 diabetes mellitus without chronic complications[4] and obese patients[5] present normal gastric emptying, and the administration of clear liquids up to 2-3 hours before anesthesia may be safe.

33. In most patients who are going to undergo an elective surgical procedure, solid food should be allowed up to 6 hours before anesthetic induction, and clear liquids up to 2 hours before anesthesia.

High level of evidence. Strong recommendation.

34. In those patients with delayed gastric emptying and in emergency surgery, it is recommended to fast from midnight or 6-8 hours before surgery.

Moderate level of evidence. Strong recommendation.

REFERENCES

1. Lobo DN, Hendry PO, Rodrigues G, Marciani L, Totman JJ, Wright JW, et al. Gastric emptying of three liquid oral preoperative metabolic preconditioning regimens measured by magnetic resonance imaging in healthy adult volunteers: a randomised double-blind, crossover study. Clin Nutr. 2009; 28:636-41.
2. Lambert E, Carey S. Practice guideline recommendations on perioperative fasting. A systematic review. J Parenter Enteral Nutr. 2016; 40:1158-65.
3. Brady M, Kinn S, Stuart P. Preoperative fasting for adults to prevent perioperative complications. Cochrane Database Syst Rev. 2003; CD00442383.
4. Gustafsson UO, Nygren J, Thorell A, Soop M, Hellstrom PM, Ljungqvist O, et al. Pre-operative carbohydrate loading may be used in type 2 diabetes patients. Acta Anaesthesiol Scand. 2008; 52:946-51.
5. Maltby JR, Pytka S, Watson NC, Cowan RA, Fick GH. Drinking 300 mL of clear fluid two hours before surgery has no effect on gastric fluid volume and pH in fasting and non-fasting obese patients. Can J Anaesth. 2004; 51:111-115.

Treatment with hydrocarbon drinks

INTRODUCTION

Preoperative fasting and surgical stress can induce insulin resistance and postoperative hyperglycemia[1].Oral carbohydrate intake (12.5% maltodextrins) at a dose of 800 ml at midnight and 400 ml 2 hours before surgery can attenuate the catabolic response induced by surgery and fasting and reduce postoperative insulin resistance, in addition it can improve the patient's feeling of well-being (thirst, hunger and anxiety) [1] without increasing the risk of aspiration[2,3,4].

In patients undergoing major abdominal surgery, a meta-analysis and a systematic review have shown that, compared with fasting or placebo, treatment with more than 45 g of carbohydrates in the 4 hours before surgery is associated with a small reduction in hospital stay without influencing the rate of postoperative complications[2,3]. The same results have been observed in a recent meta-analysis network where fasting was compared with the administration of a low dose (<45 g) or a high dose (>45 g) of oral carbohydrates up to 4 hours before surgery. although there were no significant differences in relation to insulin resistance[4]. The administration of 100 g of carbohydrates is associated with a lower need for insulin treatment and a lower blood glucose level> 180 mg / dl with no differences in the development of postoperative infectious complications[5].

35. Oral intake of carbohydrate-rich beverages up to 2 hours before surgery is safe and is not associated with increased risk of aspiration.

Moderate level of evidence. Strong recommendation.

36. Oral administration of 200-400 ml of a drink containing 50 g of carbohydrates should be allowed up to two hours before surgery since this treatment improves the patient's feeling of well-being and can reduce hospital stay and insulin resistance.

Moderate level of evidence. Strong recommendation.

REFERENCES

1. Nygren J, Thorell A, Ljungqvist O. Preoperative oral carbohydrate therapy. Curr Opin Anaesthesiol. 2015; 28:364-9.
2. Smith MD, McCall J, Plank L, Herbison GP, Soop M, Nygren J. Preoperative carbohydrate treatment for enhancing recovery after elective surgery. Cochrane Database Syst Rev. 2014:CD009161.
3. Awad S, Varadhan KK, Ljungqvist O, Lobo DN. A meta-analysis of randomised controlled trials on preoperative oral carbohydrate treatment in elective surgery. Clin Nutr. 2013; 32:34-44.
4. Amer MA, Smith MD, Herbison GP, Plank LD, McCall JL. Network meta-analysis of the effect of preoperative carbohydrate loading on recovery after elective surgery. Br J Surg. 2017; 1 04:187-197.
5. Gianotti L, Biffi R, Sandini M, Marrelli D, Vignali A, Caccialanza R, et al. Preoperative oral carbohydrate load versus placebo in major elective abdominal surgery (PROCY): a randomized, placebo-controlled, multicenter, phase III trial. Ann Surg. 2018;267:623-630.

SPECIAL CASES:

Treatment with carbohydrate beverages in patients with diabetes and obesity

INTRODUCTION

Even though diabetes mellitus (DM) affects 15% of surgical patients, there is little published evidence on the benefits of using carbohydrate beverages in obese subjects and / or those with DM. In a recent RCT that included patients with morbid obesity who underwent bariatric surgery (20% with DM), it was shown that treatment with oral carbohydrates is safe, although no differences were observed in relation to the preservation of lean mass, stay hospital or postoperative complications[1]. In patients with type 2 DM with good metabolic control who do not present neuropathic complications and who receive their usual hypoglycemic treatment, the administration of 50 g of carbohydrates 3 hours before anesthetic induction is safe, does not delay gastric emptying nor it increases risk of hyperglycemia or aspiration[2,3].

37. In obese and / or type 2 diabetic patients with good glycemic control without associated chronic complications, the use of carbohydrate-rich beverages 3 hours before surgery could be considered. These can be given together with your usual antidiabetic medication.

Low level of evidence. Weak recommendation.

REFERENCES

1. Azagury DE, Ris F, Pichard C, Volonte F, Karsegard L, Huber O. Does perioperative nutrition and oral carbohydrate load sustainably preserve muscle mass after bariatric surgery? A randomized control trial. Surg Obes Relat Dis. 2015; 11:920-6.
2. Laffin MR, Li S, Brisebois R, Senior PA, Wang H. The use of a pre-operative carbohydrate drink in patients with diabetes mellitus: a prospective, non-inferiority, cohort study. World J Surg. 2018; 42:1965-70.
3. Gustafsson UO, Nygren J, Thorell A, Soop M, Hellström PM, Ljungqvist O, et al. Pre-operative carbohydrate loading may be used in type 2 diabetes patients. Acta Anaesthesiol Scand. 2008; 52:946-5199.

PREMEDICATION

Use of sedative and anxiolytic drugs

INTRODUCTION

The use of premedication with long-half-life drugs, such as opioids or benzodiazepines, can prevent early postoperative recovery, causing a delay in the start of mobilization and oral fluid tolerance, and may prolong hospital stay. talaria.[1,2]

The use of anxiolytics with a short half-life, in the immediate preoperative period, could lengthen the anesthetic delivery time[3], as well as delaying postoperative recovery, increasing the risk of cognitive impairment, especially in elderly, frail patients and in those with significant comorbidity.[4] There are no conclusive data on its use, in short-stay surgeries, its use at low doses, it has not shown a delay in discharge hospital, presenting a decrease in the incidence of postoperative nausea and vomiting.[5]

This recommendation appears in the first edition of the Intensified Recovery Pathway in Abdominal Surgery (RICA) and is based mainly on the consensus of experts.[6]

38. It is recommended to avoid the use of long half-life benzodiazepines and opioids prior to induction in patients at high risk due to age and comorbidity.

Low level of evidence. Strong recommendation.

REFERENCES

1. Jeon S, Lee HJ, Do W, Kim HK, Kwon JY, Hwang BY, et al. Randomized controlled trial assessing the effectiveness of midazolam premedication as an anxiolytic, analgesic, sedative, and hemodynamic stabilizer. Medicine (Baltimore). 2018; 97(35): e12187.
2. Mijderwijk H, Van Beek S, Duivenvoorden HJ, Stolker RJ. Effectiveness of benzodiazepine premedication on recovery in day-case surgery: a systematic review with meta-analysis. Minerva Anestesiol. 2016; 82(4): 438-64.
3. Bucx MJL, Krijtenburg P, Kox M. Preoperative use of anxiolytic-sedative agents; Are we on the right track? Journal of Clinical Anesthesia. J Clin Anesth. 2016; 33:135-40.
4. Maurice-Szamburski A, Auquier P, Viarre-Oreal V, Cuvillon P, Carles M, Ripart et al. Effect of sedative premedication on patient experience after general anesthesia: A randomized clinical trial. JAMA. 2015; 313(9): 916-25.
5. Beydon L, Rouxel A, Camut, N, Schinkel, N, Malinovsky, JM, Aveline C, et al. Sedative premedication before surgery. A multicentre randomized study versus placebo. Anaesth Crit Care Pain Med. 2015; 34(3):165-71.

7.1.2. PREOPERATIVE

Antibiotic prophylaxis

INTRODUCTION

Surgical site infection (SSI) continues to be the second leading cause of infection related to health care in our field[1,2] having been clearly related to longer stays, increased morbidity and costs, as well as a clear impact on quality life span of patients [3]. As indicated in the Zero Surgical Infection project, antibiotic prophylaxis (AP) has outstanding efficacy in the prevention of SSIs and continues to be the main SSI prevention measure and the most cost-effective[4]. Among the various actions for the prevention of SSI antibiotic prophylaxis is one of the most effective measures, although its effectiveness decreases if the rest of the measures are not followed[5]. On the other hand, inappropriate administration of antibiotic prophylaxis not only

increases the risk of SSI but is also associated with increased prevalence of multiresistant germs and episodes of toxicity. In Europe, surgical antibiotic prophylaxis accounts for about 25% of antibiotic prescriptions, being inadequately maintained (more than 24 hours) in more than half of the cases[6]. The latest recommendations from the WHO[1,2] for the prevention of SSI include 4 specific ones about antibiotic prophylaxis: 1) administer the antibiotic before surgery if recommended; 2) do it within the 120 minutes prior to incision (based on the half-life of the drug); 3) stop administering antibiotics even if the drains persist; 4) not maintaining prophylaxis after completion of surgery.

An addition to these recommendations is that published in 2017 by the CDC: a) administer prophylactic antibiotics only in those surgeries in which it is indicated; b) in caesarean sections, infuse the antibiotic before the incision; and c) not maintaining prophylaxis after wound closure[7]. Antibiotic prophylaxis in surgery should obtain serum and tissue antibiotic concentrations above those minimum inhibitory concentrations (MIC) of the most likely contaminating microorganisms for each procedure at the time of the incision and maintained throughout the procedure Surgical[7-9].

Indication and antibiotic choice

INTRODUCTION

In clean surgeries, the indication depends on the type of intervention, the patient's comorbidity, and the use of prosthetic material. In clean-contaminated and contaminated surgeries it is recommended to always use antibiotic prophylaxis. In dirty surgery, antibiotic prophylaxis is not considered but antibiotic treatment.

First or second generation cephalosporins are the drugs of choice for prophylaxis due to their efficacy, spectrum, few adverse effects, and low cost, as reflected in studies and most of the current guidelines[10-14]. In cases of allergy to beta-lactams, a history of colonization or infection by methicillin-resistant Staphylococcus aureus, or a high prevalence in the hospital of infection of the surgical wound by this microorganism, a glycopeptide can be used. Finally, in colon or gynecological surgery, in which the involvement of anaerobic microorganisms and enterobacteria is expected, it is advisable to choose an antibiotic or a combination of antibiotics with activity against both groups of microorganisms.

39. Antibiotic prophylaxis is recommended if the chances of infection are high or if the consequences of a postoperative infection are potentially serious for the patient (endocarditis, endophthalmitis, prosthetic infection).

    Moderate level of evidence. Strong recommendation.

40. In clean surgery with infection risk factors, it is recommended to use antibiotics that cover microorganisms of the cutaneous microbiota (S. aureus and coagulase negative staphylococci) and in clean-contaminated surgery also gram-negative bacilli and enterococci as well as anaerobes.

    Moderate level of evidence. Strong recommendation.

Administration time

INTRODUCTION

One of the fundamental aspects to maintain the effectiveness of antibiotic prophylaxis is to administer it at the optimal time. In the case of short half-life beta-lactams (e.g., penicillin and cephalosporins such as cefazolin, cefoxitin, and cefuroxime) it is advisable to administer them within 60 minutes prior to surgical incision. In the case of vancomycin, aminoglycosides, or fluoroquinolones, the intravenous infusion should begin 90 minutes before the surgical incision, as these antibiotics require long infusion periods. In the case of surgeries that require limb ischemia, administer prophylaxis before applying it[15,16].

41. It is recommended to administer antibiotic prophylaxis during the 120 minutes prior to the surgical incision.

High level of evidence. Strong recommendation.

Antibiotic dose and duration of prophylaxis

INTRODUCTION

Regarding the dose, for prophylaxis it should be the same as that used for the treatment of the infection. However, and given the current prevalence in our population,[17-19] obese patients may require higher initial doses, although dosages based on total body weight tend to overdose, so surrogate descriptors of total body weight should be used, such as ideal weight or adjusted weight. In the event of the need to maintain a prolonged dose, adjustment according to renal function may be a valid alternative[20,21].

On the other hand, if the procedure exceeds more than 2 times the half-life of the antibiotic or in situations in which the half-life is shortened (burns, high glomerular filtration rates), or significant bleeding (> 1,500 mL in adults or 25 mL / kg in children) an additional dose will be administered[22-24]. Prolonging the duration of prophylaxis is contraindicated since in most surgical procedures, a single dose of an antibiotic whose half-life ensures enough serum and tissue drug levels during surgery is adequate.

42. It is recommended to use the same dose of prophylaxis as that used to treat the infection, although in obese patients the adjusted weight should be used to calculate the dose.

Moderate level of evidence. Strong recommendation.

43. An additional dose is recommended in cases of prolonged surgeries or if there is significant blood loss.

Moderate recommendation level. Weak recommendation.

44. It is recommended not to prolong the duration of antibiotic prophylaxis beyond the duration of the surgical intervention itself.

High level of evidence. Strong recommendation.

Note.- Adverse effects of antibiotic prophylaxis

It is crucial to remember that the administration of antibiotics in surgical prophylaxis can generate adverse effects such as drug allergy[25] (especially to beta-lactams), diarrhea associated with antibiotics and / or infection by Clostridioides difficile[26,27], the development of antimicrobial resistance[28,29] and acute renal failure in major surgical procedures and/or concomitant administration of aminoglycosides and glycopeptides[30,31].

Thus, allergy to beta-lactams should be ruled out both in the anesthesia consultation and in preoperative care. Similarly, all guidelines (general and local) should consider the use of alternative drugs to beta-lactams in case of allergy.

C. Difficile infection is a serious complication that can appear with some antibiotics used in antibiotic prophylaxis, such as cephalosporins, carbapenems, fluoroquinolones or clindamycin, especially if the duration of prophylaxis is prolonged. The use of single doses also helps to minimize other adverse effects acquired antimicrobial resistance.

In relation to the possibility of developing acute renal failure due to the use of antibiotics, serial determinations of serum and urinary creatinine will be made, both preoperatively and ≥24h after surgery, in major surgery patients to check the degree of renal function, with special attention to patients who have received prophylaxis with aminoglycosides or glycopeptides.

REFERENCES

1. Allegranzi B, Zayed B, Bischoff P, Kubilay NZ, de Jonge S, de Vries F, et al. New WHO recommendations on intraoperative and postoperative measures for surgical site infection prevention: an evidence-based global perspective. Lancet Infect Dis. diciembre de 2016; 16(12):e288-303.
2. Allegranzi B, Bischoff P, de Jonge S, Kubilay NZ, Zayed B, Gomes SM, et al. New WHO recommendations on preoperative measures for surgical site infection prevention: an evi- dence-based global perspective. Lancet Infect Dis. diciembre de 2016;16(12):e276-87.
3. Badia JM, Casey AL, Petrosillo N, Hudson PM, Mitchell SA, Crosby C. Impact of surgical site infection on healthcare costs and patient outcomes: a systematic review in six European countries. J Hosp Infect. mayo de 2017;96(1):1-15.
4. Proyecto Infección Quirúrgica Zero del Sistema Nacional de Salud. Sociedad Española de Medicina Preventiva, Salud Pública e Higiene. 2016.
5. Koek MBG, Hopmans TEM, Soetens LC, Wille JC, Geerlings SE, Vos MC, et al. Adhering to a national surgical care bundle reduces the risk of surgical site infections. PLoS ONE. 2017;12(9):e0184200.
6. Plachouras D, Kärki T, Hansen S, Hopkins S, Lyytikäinen O, Moro ML, et al. Antimicrobial use in European acute care hospitals: results from the second point prevalence survey (PPS) of healthcare-associated infections and antimicrobial use, 2016 to 2017. Eurosurveillance. 15 de noviembre de 2018;23(46):1800393.
7. Berríos-Torres SI, Umscheid CA, Bratzler DW, Leas B, Stone EC, Kelz RR, et al. Centers for Disease Control and Prevention Guideline for the Prevention of Surgical Site Infection, 2017. JAMA Surg. 1 de agosto de 2017;152(8):784-91.
8. Del Toro López MD, Arias Díaz J, Balibrea JM, Benito N, Canut Blasco A, Esteve E, Horcajada JP, Ruiz Mesa JD, Vázquez AM, Muñoz Casares C, Del Pozo JL, Pujol M, Riera M, Jimeno J, Rubio Pérez I, Ruiz-Tovar Polo J, Serrablo A, Soriano A, Badia JMI Grupo de Estudio de PA de la Sociedad Española de Enfermedades Infecciosas y Microbiología Clínica (SEIMC) y Asociación Española de Cirujanos (AEC). Executive summary of the Consensus Document of the Spanish Society of Infectious Diseases and Clinical Microbiology (SEIMC) and of the Spanish Association of Surgeons (AEC) in antibiotic prophylaxis in surgery. Cir Esp. 2020 Jul 28;S0009-739X(20)30113-5. doi: 10.1016/j.ci-

resp.2020.03.022.

9. Bratzler DW, Dellinger EP, Olsen KM, Perl TM, Auwaerter PG, Bolon MK, et al. Clinical practice guidelines for antimicrobial prophylaxis in surgery. Am J Health Syst Pharm. 1 de febrero de 2013;70(3):195-283.

10. Asensio A. [Surgical site infections: antibiotic prophylaxis in surgery]. Enferm Infecc Microbiol Clin. enero de 2014;32(1):48-53.

11. Townsend TR, Reitz BA, Bilker WB, Bartlett JG. Clinical trial of cefamandole, cefazolin, and cefuroxime for antibiotic prophylaxis in cardiac operations. J Thorac Cardiovasc Surg. octubre de 1993;106(4):664-70.

12. Kreter B, Woods M. Antibiotic prophylaxis for cardiothoracic operations. Meta-analysis of thirty years of clinical trials. J Thorac Cardiovasc Surg. septiembre de 1992;104(3):590-9.

13. Bratzler DW, Houck PM, Surgical Infection Prevention Guidelines Writers Workgroup, American Academy of Orthopaedic Surgeons, American Association of Critical Care Nurses, American Association of Nurse Anesthetists, et al. Antimicrobial prophylaxis for surgery: an advisory statement from the National Surgical Infection Prevention Project. Clin Infect Dis. 15 de junio de 2004;38(12):1706-15.

14. Engelman R, Shahian D, Shemin R, Guy TS, Bratzler D, Edwards F, et al. The Society of Thoracic Surgeons practice guideline series: Antibiotic prophylaxis in cardiac surgery, part II: Antibiotic choice. Ann Thorac Surg. abril de 2007;83(4):1569-76.

15. de Jonge SW, Gans SL, Atema JJ, Solomkin JS, Dellinger PE, Boermeester MA. Timing of preoperative antibiotic prophylaxis in 54,552 patients and the risk of surgical site infection: A systematic review and meta-analysis. Medicine (Baltimore). julio de 2017;96(29):e6903.

16. Lizán-García M, García-Caballero J, Asensio-Vegas A. Risk factors for surgical-wound infection in general surgery: a prospective study. Infect Control Hosp Epidemiol. mayo de 1997;18(5):310-5.

17. Crawford T, Rodvold KA, Solomkin JS. Vancomycin for surgical prophylaxis? Clin Infect Dis. mayo de 2012;54(10):1474-9.

18. Pea F, Furlanut M, Stellini R, Bonardelli S, Signorini L, Pavan F, et al. Pharmacokinetic-pharmacodynamic aspects of antimicrobial prophylaxis with teicoplanin in patients under- going major vascular surgery. Int J Antimicrob Agents. enero de 2006;27(1):15-9.

19. How should antibiotics be dosed in obesity? - SPS- Specialist Pharmacy Service - The first stop for professional medicines advice [Internet]. [citado 12 de febrero de 2019]. Dispo- nible en: https://www.sps.nhs.uk/articles/how-should-antibiotics-be-dosed-in-obesity/

20. Voigt J, Mosier M, Darouiche R. Systematic review and meta-analysis of randomized con- trolled trials of antibiotics and antiseptics for preventing infection in people receiving pri- mary total hip and knee prostheses. Antimicrob Agents Chemother. noviembre de 2015;59(11):6696-707.

21. Pai MP. Treatment of bacterial infections in obese adult patients: how to appropriately manage antimicrobial dosage. Curr Opin Pharmacol. octubre de 2015;24:12-7.

22. Isla A, Trocóniz IF, de Tejada IL, Vázquez S, Canut A, López JM, et al. Population pharmacokinetics of prophylactic cefoxitin in patients undergoing colorectal surgery. Eur J Clin Pharmacol. mayo de 2012;68(5):735-45.

23. Asín-Prieto E, Soraluce A, Trocóniz IF, Campo Cimarras E, Sáenz de Ugarte Sobrón J, Rodrí- guez-Gascón A, et al. Population pharmacokinetic models for cefuroxime and metronida- zole used in combination as prophylactic agents in colorectal surgery: Model-based evalua- tion of standard dosing regimens. Int J Antimicrob Agents. mayo de 2015;45(5):504-11.

24. Swoboda SM, Merz C, Kostuik J, Trentler B, Lipsett PA. Does intraoperative blood loss affect antibiotic serum and tissue concentrations? Arch Surg. noviembre de 1996;131(11):1165- 71; discussion 1171-1172.

25. Hong J, Krop LC, Johns T, Pai MP. Individualized vancomycin dosing in obese patients: a two-sample measurement approach improves target attainment. Pharmacotherapy. mayo de 2015;35(5):455-63.

26. Carignan A, Allard C, Pépin J, Cossette B, Nault V, Valiquette L. Risk of Clostridium difficile infection after perioperative antibacterial prophylaxis before and during an outbreak of in- fection due to a hypervirulent strain. Clin Infect Dis. 15 de junio de 2008;46(12):1838- 43.

27. Jenkins PJ, Teoh K, Simpson PM, Dave J, Simpson AHWR, Breusch S. Clostridium difficile in patients undergoing primary hip and knee replacement. J Bone Joint Surg Br. julio de 2010;92(7):994-8.

28. Avery CME, Ameerally P, Castling B, Swann RA. Infection of surgical wounds in the maxillo- facial region and free flap donor sites with methicillin-resistant Staphylococcus aureus. Br J Oral Maxillofac Surg. junio de 2006;44(3):217-21.

29. Harbarth S, Samore MH, Lichtenberg D, Carmeli Y. Prolonged antibiotic prophylaxis after cardiovascular surgery and its effect on surgical site infections and antimicrobial resistance. Circulation. 27 de junio de 2000;101(25):2916-21.

30. Walker H, Patton A, Bayne G, Marwick C, Sneddon J, Davey P, et al. Reduction in post-operative acute kidney injury following a change in antibiotic prophylaxis policy for ortho-paedic surgery: an observational study. J Antimicrob Chemother. 2016;71(9):2598-605.

31. Bell S, Dekker FW, Vadiveloo T, Marwick C, Deshmukh H, Donnan PT, et al. Risk of posto-perative acute kidney injury in patients undergoing orthopaedic surgery-development and validation of a risk score and effect of acute kidney injury on survival: observational cohort study. BMJ. 11 de noviembre de 2015;351:h5639.

Glucocorticoids

INTRODUCTION

The preoperative administration of glucocorticoids is associated with an attenuation of the magnitude of the inflammatory response to surgical stress, being able to reduce the incidence of complications, including those of an infectious nature.[1-3] Its effects include vasoconstriction and decreased capillary permeability.[4] In the postoperative period, they reduce the secretion of acute phase reactants, such as interleukin 6 or C-reactive protein.[5]

45. The administration of a single dose of glucocorticoids is recommended because it has a significant impact on the duration of hospital admission without increasing the rate of complications.

Moderate level of evidence. Strong recommendation.

REFERENCES

1. Taniguchi Y, Kurokawa Y, Hagi T, Takahashi T, Miyazaki Y, Tanaka K, et al. Methylpredniso-lone Inhibits Tumor Growth and Peritoneal Seeding Induced by Surgical Stress and Posto-perative Complications. Annals of Surgical Oncology. 2019;26(9):2831-38.

2. Steinthorsdottir KJ, Kehlet H, Aasvang EK. Surgical stress response and the potential role of preoperative glucocorticoids on post-anesthesia care unit recovery. Minerva Anestesiologi-ca. 2017;83(12):1324-31.

3. El-Sibai K, Rajpal A, Al-Aridi R, Selman WR, Arafah BM. The impact of peri-operative dexa-methasone administration on the normal hypothalamic pituitary adrenal response to major surgical procedures. Endocrine. 2017;58(1):134-42.

4. McSorley ST, Horgan PG, McMillan DC. The impact of preoperative corticosteroids on the systemic inflammatory response and postoperative complications following surgery for gastrointestinal cancer: a systematic review and meta-analysis. Crit Rev Oncol Hematol. 2016;101:139-50.

5. McSorley ST, Roxburgh CSD, Horgan PG, McMillan DC. The Impact of Preoperative Dexamethasone on the Magnitude of the Postoperative Systemic Inflammatory Response and Complications Following Surgery for Colorectal Cancer. Ann Surg Oncol. 2017;24(8): 2104-12.

Perioperative blood glucose monitoring

INTRODUCTION

Controlling normoglycemia is essential for reducing perioperative infections and reducing complications from hyperglycemia. It is one of the recommendations of the Zero Surgical Infection program of the Ministry of Health of the Government of Spain.[1] The use of intensive insulin therapy must be avoided due to the high risk of hypoglycemia during the perioperative period, which can lead to increased mortality. The consensual range of blood glucose should be between 150 and 180 g / dl. [2-5]

46. Glycemia will be monitored preoperatively, since intraoperative hyperglycemia can lead to increased postoperative complications, although the use of intensive insulin therapy should be avoided due to the risk of hypoglycemia.

High level of evidence. Strong recommendation.

REFERENCES

1. Protocolo de Trabajo del IQZ 2017. Disponible en (última consulta 01-06-2020): https://infeccionquirurgicazero.es/es/documentos-y-materiales/protocolos-de-trabajo

2. Pontes JPJ, Mendes FF, Vasconcelos MM, Batista NR. [Evaluation and perioperative management of patients with diabetes mellitus. A challenge for the anesthesiologist]. Rev Bras Anestesiol 2018;68(1):75-86.

3. Akiboye F, Rayman G. Management of Hyperglycemia and Diabetes in Orthopedic Surgery. Curr Diab Rep 2017;17(2):13.

4. Dhatariya K, Levy N, Hall GM. The impact of glycaemic variability on the surgical patient. Curr Opin Anaesthesiol 2016;29(3):430-7.

5. Barker P, Creasey PE, Dhatariya K, Levy N, Lipp A, Nathanson MH et al. Peri-operative management of the surgical patient with diabetes 2015: Association of Anaesthetists of Great Britain and Ireland. Anaesthesia 2015;70(12):1427-40.

Perioperative hyperglycemia

INTRODUCTION

Hyperglycemia is related to increased morbidity and mortality in operated patients, both diabetic and non-diabetic[1].

Improved glycemic control reduces the risk of hospital complications after surgery[1]. A widely accepted recommendation is to keep blood glucose between 140 and 180 mg / dl for hospitalized patients. In patients with parenteral nutrition and blood glucose greater than 180 mg / dl, glucose intake can be reduced and / or insulin treatment increased. Patients with unstable and high glucose levels are preferable to be treated in critical units[2].

Insulin therapy for the treatment of persistent hyperglycemia should be started at a level> 180 mg/dl, recommending a range of 140-180 mg/dl in most critical and non-critical patients[3]. More

stringent levels between 110-140 mg/dl may be appropriate in selected patients if it can be achieved without significant hypoglycemia. More research is needed to develop treatment recommendations.

47. Perioperative blood glucose should be monitored and adequately treated with insulin, avoiding blood glucose levels> 180 mg / dl.

Moderate level of evidence. Strong recommendation.

48. More ambitious targets for perioperative blood glucose between 110 and 140mg / dL (6.1-7.8 mmol / L) may be appropriate in selected patients if they can be achieved without significant hypoglycemia.

Low level of evidence. Weak recommendation.

REFERENCES

1. Kotagal M, Symons RG, Hirsch IB, Umpierrez GE, Farrokhi ET, Flum DR, SCOAP-Ceertain Collaborative. Perioperative hyperglycemia and risk of adverse events among patients with and without diabetes. Ann Surg 2015;261(1):97-103.
2. Weimann A, Braga M, Carli F, Higashiguchi T, Hübner M, Klek S, et al. ESPEN guideline: Clinical nutrition in surgery. Clin Nutr 2017;36:623-650.
3. American Diabetes Association. 15. Diabetes Care in the Hospital: Standards of Medical Care in Diabetes-2019. Diabetes Care. 2019 Jan;42(Suppl 1):S173-S181.

Hair removal

INTRODUCTION

Hair has traditionally been considered associated with a lack of cleaning and increased infection of the surgical wound, in addition to the fact that its removal allows a better exposure of the incision area and facilitates the suture and placement of dressings.

There are studies that show that not prior shaving of the incision area has a preventive effectiveness close to 50% of surgical site infections[1].

Both in a Cochrane review and in more recent meta-analyses, no significant differences were observed in the appearance of wound infection between patients who had their hair removed and those who had not[1-5].

Therefore, current recommendations suggest that patients' hair should not be removed before the intervention, unless it is strictly necessary, and in that case an electric razor should be used to cut the hair, preferably with a disposable head (Project Surgical Infection Zero and Via RICA-2015 edition).

Regarding the moment in which the hair removal is performed, there is no evidence that the removal close to the time of the intervention reduces infections, but current recommendations suggest that the most appropriate thing, in case of deciding to remove the hair, would be better to do it close to the intervention, but always outside the operating room[6-7].

49. Hair should not be removed preoperatively unless strictly necessary. Conventional shaving should be avoided, both preoperatively and in the operating room.

High level of evidence. Strong recommendation.

50. In the case of hair removal, electric razors can be used as close as possible to the intervention, but always outside the operating room.

Moderate level of evidence. Strong recommendation.

REFERENCES

1. Allegranzi B, Bischoff P, de Jonge S, et al. New WHO recommendations on preoperative measures for surgical site infection prevention: an evidence-based global perspective. Lancet Infect Dis. 2016;16(12):e276-e287. doi:10.1016/S1473-3099(16)30398-X.

2. Dohmen PM, Konertz W. A review of current strategies to reduce intraoperative bacterial contamination of surgical wounds. GMS Krankenhhyg Interdiszip. 2007;2(2):Doc38. http://www.ncbi.nlm.nih.gov/pubmed/20204082.

3. Lefebvre A, Saliou P, Lucet JC, et al. Preoperative hair removal and surgical site infections: network meta-analysis of randomized controlled trials. J Hosp Infect. 2015;91(2):100-108. doi:10.1016/j.jhin.2015.06.020.

4. Tanner J, Norrie P, Melen K. Preoperative hair removal to reduce surgical site infection. Cochrane Database Syst Rev. 2011;(11):CD004122. doi:10.1002/14651858.cd004122.pub4.

5. Shi D, Yao Y, Yu W. Comparison of preoperative hair removal methods for the reduction of surgical site infections: a meta-analysis. J Clin Nurs. 2017;26(19-20):2907-2914. doi: 10.1111/jocn.13661.

6. Edmiston CEJ, Griggs RK, Tanner J, Spencer M, Seabrook GR, Leaper D. Perioperative hair removal in the 21st century: Utilizing an innovative vacuum-assisted technology to safely expedite hair removal before surgery. Am J Infect Control. 2016;44(12):1639-1644. doi:10.1016/j.ajic.2016.03.071.

7. JBI (Joanna Briggs Institute). Pre-operative hair removal to reduce surgical site infection. Best Practice 2007;11(4).

7.1.3 INTRAOPERATIVE

Checklist

INTRODUCTION

The results of the systematic review carried out in the Safe Surgery project[1] until April 2015, show a significant improvement in patient safety indicators (decrease in the rates of adverse events, mortality, and infection of the surgical wound), after the implementation of the surgical checklists.

Considering in publications from 2015, most studies also show a reduction in adverse events related to surgical intervention as well as in hospital mortality, although there is heterogeneity between the studies (surgeries and specialties, existence of concurrent control groups, coexistence of other improvement measures, etc.) different[2-4].

51. The use of the surgical checklist is recommended for the prevention of adverse events and mortality related to the intervention.

Moderate level of evidence. Strong recommendation.

REFERENCES

1. Programa de Cirugía Segura del Sistema Nacional de Salud. Ministerio de Sanidad, Servi- cios Sociales e Igualdad. 2016.

2. De Jager E, McKenna C, Bartlett L3, Gunnarsson R, Ho YH. postoperative Adverse Events Inconsistently Improved byThe World Health Organization Surgical Safety Checklist: A Systematic Literature Review of 25 Studies. World J Surg. 2016 Aug;40(8):1842-58. doi: 10.1007/s00268-016-3519-9.

3. Abbott TEF, Ahmad T, Phull MK, Fowler AJ, Hewson R, Biccard BM, Chew MS, Gillies M, Pearse RM; International Surgical Outcomes Study (ISOS)group.The surgical safety chec- klist and patient outcomes after surgery: prospective observational cohort study, systematic review and

meta-analysis. Br J Anaesth. 2018 Jan;120(1):146-155. doi: 10.1016/j. bja.2017.08.002. Epub 2017 Nov 23.

4. Biccard BM, Rodseth R, Cronje L, Agaba P, Chikumba E, Du Toit L, Farina Z, Fischer S, Go- palan PD, Govender K, Kanjee J, Kingwill A, Madzimbamuto F, Mashava D, Mrara B, Mudely M, Ninise E, Swanevelder J, Wabule A.A meta-analysis of the efficacy of preoperative surgi- cal safety checklists to improve perioperative outcomes. S Afr Med J. 2016 May 9;106(6). doi: 10.7196/SAMJ.2016.v106i6.9863.

Skin and surgical field preparation.

INTRODUCTION

For a correct preparation of the operative field, we must consider the importance of cleaning the skin before applying the antiseptic solution, with soap and water, followed by rinsing and drying whole skin and with saline solution on mucous membranes and wounds.

The disinfection of the skin prior to the delimitation of the surgical field, will be carried out by making forward and backward movements, rubbing and making friction in horizontal and vertical bands, we will use 2% Alcoholic Chlorhexidine in all those incisions that are made on intact skin, it will be done for 30 seconds, it is important to let the antiseptic dry for 2 minutes to give it time to act[1-2]. (Zero Surgical Infection Project).

Alcoholic antiseptics are flammable substances and therefore it must be ensured that it is completely dry and verify that there are no accumulated amounts in the patient's skin folds, or in gauze and drapes of the surgical field under the patient[3].

In interventions on the eye, middle ear, and meninges and those whose approach is a mucosa (oral, nasal, urethral, vaginal, anal), dilute aqueous chlorhexidine (0.5%) or Povidone Iodine will be used, depending on the case. 10% [4].

52. The use of 2% alcoholic chlorhexidine is recommended as an antiseptic for intact skin in the surgical field.

High level of evidence. Strong recommendation.

REFERENCES

1. Privitera GP, Costa AL, Brusaferro S, Chirletti P, Crosasso P, Massimetti G, et al. Skin antisepsis with chlorhexidine versus iodine for the prevention of surgical site infection: A systematic review and meta-analysis. Am J Infect Control. 2017; 45(2):180-189. doi: 10. 1016/j.ajic.2016.09.017.

2. Bratzler DW, Houck PM. Antimicrobial prophylaxis for surgery: an advisory statement from the National Surgical Infection Prevention Project. Clin Infect Dis. 2004 Jun 15; 38(12):1706-15. doi: 10.1086/421095.

3. Hsieh CS, Cheng HC, Lin JS, Kuo SJ, Chen YL. Effect of 4% chlorhexidine gluconate predisinfection skin scrub prior to hepatectomy: a double-blinded, randomized control study. Int Surg. 2014 Nov-Dec; 99(6):787-94. doi: 10.9738/INTSURG-D-13-00179.1.

4. Darouiche RO, Wall MJ Jr, Itani KM, Otterson MF, Webb AL, Carrick MM. Chlorhexidine-Alcohol versus Povidone-Iodine for Surgical-Site Antisepsis. N Engl J Med. 2010 Jan 7; 362 (1):18-26. doi: 10.1056/NEJMoa0810988.

Anesthetic induction and maintenance

INTRODUCTION

A standard anesthetic protocol is required to allow rapid awakening. The anesthetist must

monitor fluid therapy, analgesia, and hemodynamic stability to reduce the metabolic response to stress.

The use of benzodiazepines prior to induction should only be to reduce anxiety and in the lowest possible dose, to reduce episodes of delirium and postoperative cognitive impairment, especially in high-risk, elderly and multipathological patients.[1]

At present, anesthetic and analgesic agents are used with minimal residual effect and that allow a rapid recovery after anesthesia: propofol, combined, if necessary, with a short-acting opioid such as fentanyl, alfentanil, sufentanil or an infusion of remifentanil.[2]

Anesthesia can be maintained with short-acting inhalational anesthetics, such as sevoflurane or desflurane (induction and awakening of anesthetic faster than sevoflurane), such as intravenous ones

like propofol. There is no evidence of superiority of total intravenous anesthesia (TIVA) with propofol versus inhalation anesthesia, although TIVA may be beneficial in patients with susceptibility to postoperative nausea and vomiting. There is also no evidence that TIVA improves oncological prognoses in humans over inhalational anesthesia.[3]

This recommendation appears in the first edition of the Intensified Recovery Pathway in Abdominal Surgery (RICA) and is based mainly on the consensus of experts.[4]

53. It is recommended to minimize the use of benzodiazepines prior to induction and to use hypnotic agents with minimal residual effect, which allow rapid recovery after anesthesia.

Low level of evidence. Strong recommendation.

REFERENCES

1. Apfel CC, Korttila K, Abdalla M, Kerger H, Turan A, Vedder I, et al. A factorial trial of six interventions for the prevention of postoperative nausea and vomiting. N Engl J Med 2004;350(24):2441-51.
2. Ren L, Zhu D, Wei Y, Pan X, Liang L, Xu J, et al. Enhanced Recovery After Surgery (ERAS) Program Attenuates Stress and Accelerates Recovery in Patients After Radical Resection for Colorectal Cancer: A Prospective Randomized Controlled Trial. World J Surg 2012;36(2):407-14.
3. Gustafsson UO, Scott MJ, Hubner M, Nygren J, Demartines N, Francis N, et al. Guidelines for Perioperative Care in Elective Colorectal Surgery: Enhanced Recovery After Surgery (ERAS®) Society Recommendations: 2018. World J Surg 2019;43(3):659-95.
4. Grupo de Trabajo. Vía Clínica de Recuperación Intensificada en Cirugía Abdominal (RICA). Madrid: Ministerio de Sanidad, Servicios Sociales e Igualdad. Madrid; 2015.

Lung protective ventilation

INTRODUCTION

The potential benefits of protective ventilation observed in patients admitted to critical care units with adult respiratory distress syndrome (ARDS) are less evident when patients do not have severe pulmonary disease and are ventilated for a few hours. Protective ventilation has 3 fundamental pillars that support it: The use of low tidal volumes, the application of recruitment maneuvers (MR) and the application of PEEP (better individualized). Few studies have investigated the efficacy of the joint use of these maneuvers on postoperative prognosis in patients undergoing general anesthesia with mechanical ventilation and without distress. An important study showed a reduction in postoperative pulmonary complications with the use of an intraoperative pulmonary ventilation strategy combined with the application of postoperative CPAP compared to the control group (standard intraoperative ventilation and no postoperative

CPAP)[1]. Although other large randomized controlled studies have failed to demonstrate benefits of VP[2,3]. A recent meta-analysis has shown that the combination of low VT and moderately high PEEP (> 5 cmH2O), with or without recruitment maneuvers, was superior to conventional mechanical ventilation in reducing the risk of CPO, the application of MR to this This strategy reduces the risk of atelectases.[4] Also, in thoracic surgery, during one-lung ventilation, a lung protection strategy has been recommended, using a tidal volume PEEP and MR associated of 4-6mL/kg of ideal weight, although results have not been conclusive in relation to its impact on postoperative outcome. [5,6]

54. During general anesthesia, the use of protective ventilation is recommended, including a tidal volume of 6-8 ml / kg ideal weight, the use of individualized PEEP generally above 5 cm H2O and the application of recruitment maneuvers.

Moderate level of evidence. Strong recommendation.

55. In surgeries that require one-lung ventilation, we recommend the above protective ventilation measures, but reducing the tidal volume to the lung dependent on 4-6mL / kg of ideal weight.

Moderate level of evidence. Strong recommendation.

REFERENCES

1. Ferrando C, Soro M, Unzueta C, Suarez-Sipmann F, Canet J, Librero J, et al. Network Individualised perioperative open-lung approach versus standard protective ventilation inabdominal surgery (iPROVE): a randomised controlled trial. Lancet Respir Med.2018;6(3):193.

2. Hemmes SN, Gama de Abreu M, Pelosi P, Schultz MJ. High versus low positive end-expiratory pressure during general anaesthesia for open abdominal surgery (PROVHILO trial): a multicentre randomised controlled trial. Lancet.2014;384(9942):495-503.

3. Bluth T, Serpa Neto A, Schultz M J, Pelosi P, Gama de Abreu M, Bobek I, et al. Effect of Intraoperative High Positive End-Expiratory Pressure (PEEP) With Recruitment Maneuvers vs Low PEEP on Postoperative Pulmonary Complications in Obese Patients: A Randomized Clinical Trial. JAMA,2019;321:2292-305.

4. Deng QW. Intraoperative Ventilation Strategies to Prevent Postoperative Pulmonary Complications: A Network Meta-Analysis of Randomised Controlled Trials Br J Anaesth. 2020;124:324-35.

5. Liu Z, Liu X, Huang Y, Zhao J.I ntraoperative mechanical ventilation strategies in patients undergoing one-lung ventilation: a meta-analysis. Springerplus.2016; 5:125.

6. El Tahan MR, Pasin L, Marczin N, Landoni G. Impact of low tidal volumes during one lung ventilation. A meta-analysis of randomised controlled trials J Cardiothorac Vasc Anesth 2017;31:1767-73.

Intraoperative Monitoring

INTRODUCTION

Routine monitoring should include 5-lead electrocardiogram (EKG) (DII and V5 recommended), non-invasive blood pressure (NIBP), pulse oximetry (% Sat O2), Inspired Oxygen Fraction (FiO2), capnography (EtCO2), temperature, fluid therapy balance and intraoperative blood glucose story.

CO2 monitoring

INTRODUCTION

CO2 monitoring by capnography is necessary in any intervention with general anesthesia to ensure the patient's gas exchange and adequate management of the airway, ruling out accidental extubation.[1]

In laparoscopic surgery where CO2 insufflation is performed to create the pneumoperitoneum, it involves the absorption of this gas by the body and it can be a sign of complications due to hypercapnia.[2]

56. CO2 monitoring by capnography should be mandatory in all surgery, especially laparoscopic surgery.

High level of evidence. Strong recommendation.

REFERENCES

1. Lam T, Nagappa M, Wong J, Singh M, Wong D, Chung F. Continuous Pulse Oximetry and Capnography Monitoring for Postoperative Respiratory Depression and Adverse Events: A Systematic Review and Meta-analysis. Anesth Analg. 2017;125:2019-2029.
2. Frerk C, Mitchell VS, McNarry AF, Mendonca C, Bhagrath R, Patel A et al. Difficult Airway Society 2015 guidelines for management of unanticipated difficult intubation in adults. Br J Anaesth 2015;115(6):827-48.1.

Temperature monitoring

INTRODUCTION

Temperature monitoring is mandatory to avoid hypothermia or hyperthermia in the patient during the perioperative period, despite different external measures to maintain normothermia. Temperature control is only reliable if the measurement is performed centrally.[1-3]

57. Temperature monitoring should be central. High level of

evidence. Strong recommendation. REFERENCES

1. Urits I, Jones MR, Orhurhu V, Sikorsky A, Seifert D, Flores C, et al. A Comprehensive Update of Current Anesthesia Perspectives on Therapeutic Hypothermia. Adv Ther 2019;36(9): 2223-2232.2.
2. Calvo Vecino JM, Casans Francés R, Ripollés Melchor J, Marín Zaldívar C, Gómez Ríos MA, Pérez Ferrer A, et al. No Intencionada de la SEDAR. Clinical practice guideline. Unintentional perioperative hypothermia. Rev Esp Anestesiol Reanim. 2018;65(10):564-588.
3. Madden LK, Hill M, May TL, Human T, Guanci MM, Jacobi J, Moreda MV, et al. The Implementation of Targeted Temperature Management: An Evidence-Based Guideline from the Neurocritical Care Society. Neurocrit Care. 2017;27(3):468-487.

Hypnosis monitoring

INTRODUCTION

Currently, there are different hypnosis monitoring systems on the market to measure the depth of general anesthesia, highlighting the Bispectral Index (BIS) for scientific evidence both by the number of publications, number of patients studied and accumulated experience in both adults and children.[1,2]

Induction and maintenance of anesthesia can be guided by the BIS monitor, thus avoiding levels of excessive depth of hypnosis (BIS <30), especially in the elderly, where there is evidence that too deep anesthesia may be harmful and may increase the risk of postoperative confusion. The dosage of anesthetic medication during general anesthesia should be adjusted

to obtain BIS values between 40 and 60.[3,4]

58. The anesthetic depth will be monitored using the bispectral index (BIS).

High level of evidence. Strong recommendation.

REFERENCES

1. Luo C, Zou W. Cerebral monitoring of anaesthesia on reducing cognitive dysfunction and postoperative delirium: a systematic review. J Int Med Res. 2018;46(10):4100-4110.

2. Punjasawadwong Y, Chau-In W, Laopaiboon M, Punjasawadwong S, Pin-On P. Processed electroencephalogram and evoked potential techniques for amelioration of postoperative delirium and cognitive dysfunction following non-cardiac and non-neurosurgical procedures in adults. Cochrane Database Syst Rev 2018;5:Cd011283.3.

3. Oliveira CR, Bernardo WM, Nunes VM. Benefit of general anesthesia monitored by bispectral index compared with monitoring guided only by clinical parameters. Systematic review and meta-analysis. Braz J Anesthesiol. 2017;67(1):72-84.

4. Chhabra A, Subramaniam R, Srivastava A, Prabhakar H, Kalaivani M, Paranjape S. Spectral entropy monitoring for adults and children undergoing general anaesthesia. Cochrane Database Syst Rev. 2016 Mar 14;3:CD010135.

Nociception Monitoring

INTRODUCTION

Currently, different surgical stress monitoring systems and the response to organic to it exist in the market. There is a great heterogeneity of monitoring systems, highlighting those derived from the autonomic nervous system and those of EEG responses (Composite Variability Index (CVI) derived from BIS or qCon / qNox). So far the most specific and sensitive are those derived from the detection of changes in the sympathetic autonomic nervous system: pupillometry with altered pupillary reflex; multiparametric such as the NOL TM index (plethysmography, temperature, accelerometry and skin impedance) and SPI (Surgical Plethysmography Index) that are based on the changes of the plethysmography pulse wave); ANI (Analgesia Nociception Index) dependent on the ECG and the influence of the parasympathetic on heart rate.[1-6]

Intraoperative opioid consumption could generally be less guided with nociception monitoring compared to standard monitoring of heart rate and blood pressure variation.[1-2]

At the present time there do not seem to be statistically significant differences with respect to intraoperative adverse events, postoperative opioid or analgesic use, postoperative pain, and postoperative adverse events.[2]

59. The use of nociception monitoring could decrease intraoperative opioid consumption compared to standard monitoring.

Moderate level of evidence. Weak recommendation

REFERENCES

1. Jiao Y, He B, Tong X, Xia R, Zhang C, Shi X. Intraoperative monitoring of nociception for opioid administration: a meta-analysis of randomized controlled trials. Minerva Anestesiol. 2019;85(5):522-530.

2. Meijer FS, Niesters M, van Velzen M, Martini CH, Olofsen E, Edry R, et al. Does nociception monitor-guided anesthesia affect opioid consumption? A systematic review of randomized controlled trials. J Clin Monit Comput 2019. doi: 10.1007/s10877-019-00362-4.

3. Gruenewald M, Dempfle A. Analgesia/nociception monitoring for opioid guidance: meta-analysis of randomized clinical trials. Minerva Anestesiol. 2017;83(2):200- 213.

4. Won YJ1, Lim BG1, Kim YS1, Lee M1, Kim H1. Usefulness of surgical pleth index-guided analgesia during general anesthesia: a systematic review and meta-analysis of randomized controlled trials. J Int Med Res. 2018;46(11):4386-4398.

5. Banerjee S, MacDougall D. Nociception Monitoring for General Anesthesia: A Review of Clinical Effectiveness, Cost-Effectiveness, and Guidelines. Ottawa (ON): Canadian Agency for Drugs and Technologies in Health; 2018 Dec 12. CADTH Rapid Response Reports.

6. Abad-Gurumeta A, Ripollés-Melchor J, Casans-Francés R, Calvo-Vecino JM. Monitoring of nociception, reality or fiction? Rev Esp Anestesiol Reanim. 2017;64:406-414.

Monitoring of urine output and urinary catheter placement

INTRODUCTION

In major surgery, urethral catheterization is common to control diuresis, as well as to avoid prolonged retention of urine in the bladder. The maintenance of the catheter during the postoperative period is related to discomfort for the patient, as well as urinary infections.[1-3]

60. The removal of the urethral catheter is recommended at 24 h, except in moderate risk of acute urine retention: men, epidural anesthesia, and pelvic surgery, which is recommended to maintain it for 3 days[2,3].

Moderate level of evidence. Weak recommendation

REFERENCES

1. Patel DN, Felder SI, Luu M, Daskivich TJ, K NZ, Fleshner P. Early Urinary Catheter Removal Following Pelvic Colorectal Surgery: A Prospective, Randomized, Noninferiority Trial. Dis Colon Rectum 2018;61(10):1180-1186.

2. Alyami M, Lundberg P, Passot G, Glehen O, Cotte E. Laparoscopic Colonic Resection Without Urinary Drainage: Is It “Feasible”? J Gastrointest Surg 2016;20(7):1388-92.

3. Zhang P, Hu WL, Cheng B, Cheng L, Xiong XK, Zeng YJ. A systematic review and meta-analysis comparing immediate and delayed catheter removal following uncomplicated hysterectomy. Int Urogynecol J 2015;26(5):665-74.

Urinary catheter

INTRODUCTION

In major surgery, urethral catheterization is common to control diuresis, as well as to avoid prolonged retention of urine in the bladder. The maintenance of the catheter during the postoperative period is related to discomfort for the patient, as well as urinary infections.[1]

61. The removal of the urethral catheter is recommended at 24 h, except in moderate risk of acute urine retention: men, epidural anesthesia, and pelvic surgery, which is recommended to maintain it for 3 days[2,3].

High level of evidence. Strong recommendation.

REFERENCES

1. Wald HL, Ma A, Bratzler DW, Kramer AM. Indwelling urinary catheter use in the postoperative period: analysis of the national surgical infection prevention project data. Arch Surg. 2008 jun;143(6):551-557.
2. Gustafsson UO, Scott MJ, Hubner M, Nygren J, Demartines N, Francis N, Rockall TA, Young-Fadok TM, Hill AG, Soop M, de Boer HD, Urman RD, Chang GJ, Fichera A, Kessler H, Grass F, Whang EE, Fawcett WJ, Carli F, Lobo DN, Rollins KE, Balfour A, Baldini G, Riedel B, Ljungqvist O. Guidelines for Perioperative Care in Elective Colorectal Surgery: Enhanced Recovery After Surgery (ERAS®) Society Recommendations: 2018. World J Surg. 2019 Mar;43(3):659-695.
3. Carmichael JC, Keller DS, Baldini G, Bordeianou L, Weiss E, Lee L, Boutros M, McClane J, Feldman LS, Steele SR. Clinical Practice Guidelines for Enhanced Recovery After Colon and Rectal Surgery From the American Society of Colon and Rectal Surgeons and Society of American Gastrointestinal and Endoscopic Surgeons. Dis Colon Rectum. 2017 Aug;60(8):761-784.

NON-ROUTINE MONITORING

Invasive blood pressure monitoring

INTRODUCTION

Advances in hemodynamic monitoring have allowed us to have less invasive monitoring, decrease the channeling of radial or femoral arteries to control continuous blood pressure and cardiac output, as well as other associated indices in a reliable way.[1-4] Currently, only those patients with high surgical risk and high anesthetic risk with a history of hemodynamic instability due to age and comorbidity are susceptible to invasive arterial monitoring.[1-3]

This recommendation appears in the first edition of the Intensified Recovery Pathway in Abdominal Surgery (RICA) and is mainly based on the consensus of experts.[5] It is also included in the Zero Surgical Infection Project.[6]

62. Invasive hemodynamic monitoring is not routinely indicated, and arterial cannulation is useful in those patients who present severe cardiorespiratory alterations and who may present postoperative problems.

Nivel de evidencia bajo. Recomendación fuerte

REFERENCES

1. Scheeren TWL, Ramsay MAE. New Developments in Hemodynamic Monitoring. J Cardiothorac Vasc Anesth 2019;33 Suppl 1:S67-s72.
2. Jozwiak M, Monnet X, Teboul JL. Less or more hemodynamic monitoring in critically ill patients. Curr Opin Crit Care 2018;24(4):309-315.
3. Yamada T, Vacas S, Gricourt Y, Cannesson M. Improving Perioperative Outcomes Through Minimally Invasive and Non-invasive Hemodynamic Monitoring Techniques. Front Med (Lausanne) 2018;5:144.
4. Teboul JL, Saugel B, Cecconi M, De Backer D, Hofer CK, Monnet X et al. Less invasive hemodynamic monitoring in critically ill patients. Intensive Care Med 2016;42(9):1350-9.
5. Grupo de Trabajo. Vía Clínica de Recuperación Intensificada en Cirugía Abdominal (RICA). Madrid: Ministerio de Sanidad, Servicios Sociales e Igualdad. Madrid; 2015.

6. Proyecto Infección Quirúrgica Zero del Sistema Nacional de Salud. Sociedad Española de Medicina Preventiva, Salud Pública e Higiene. 2016.

Central venous pressure monitoring

INTRODUCTION

Advances in non-invasive hemodynamic monitoring systems have reduced the number of patients requiring central venous pressure (CVP) control. Only when surgeries with a high risk of bleeding, a high possibility of transfusion of blood products, with significant hemodynamic alterations associated with high surgical risk with the need for vasopressor and inotropic drugs, and the need for parenteral nutrition could be justified, the need for their canalization and monitoring PVC.[1-4]

This recommendation appears in the first edition of the Clinical Pathway for Intensified Recovery in Abdominal Surgery (RICA) and is mainly based on the consensus of experts.[5] It is also included in the Zero Surgical Infection Project.[6]

63. Central venous catheter (CVC) insertion is not routinely indicated and is limited to patients with severe cardiorespiratory diseases with pulmonary hypertension or in whom it is anticipated that they may require administration of vasopressors or inotropes in continuous infusion.

Low level of evidence. Strong recommendation.

REFERENCES

1. De Backer D, Vincent JL. The pulmonary artery catheter: is it still alive? Curr Opin Crit Care 2018;24(3):204-208.
2. Joseph C, Garrubba M, Smith JA, Melder A. Does the Use of a Pulmonary Artery Catheter Make a Difference During or After Cardiac Surgery? Heart Lung Circ 2018;27(8):952-960.
3. Youssef N, Whitlock RP. The Routine Use of the Pulmonary Artery Catheter Should Be Abandoned. Can J Cardiol 2017;33(1):135-141.
4. Watson X, Cecconi M. Haemodynamic monitoring in the peri-operative period: the past, the present and the future. Anaesthesia 2017;72 Suppl 1:7-15.
5. Grupo de Trabajo. Vía Clínica de Recuperación Intensificada en Cirugía Abdominal (RICA). Madrid: Ministerio de Sanidad, Servicios Sociales e Igualdad. Madrid; 2015.
6. Proyecto Infección Quirúrgica Zero del Sistema Nacional de Salud. Sociedad Española de Medicina Preventiva, Salud Pública e Higiene. 2016.

Neuromuscular Blockade

INTRODUCTION

Quantitative monitoring of the degree of neuromuscular blockade by means of the “train of four” (TOF) during the entire anesthetic process is essential in those patients receiving neuromuscular blocking drugs (NMB) and is the only monitoring that can determine with certainty the time of extubating. In addition to the TOF, the TOF ratio (TOFr), the simple stimulus and the posttonic count can be measured. Although other muscles of the face can be used, the adductor pollicis accurately reflects the relaxed state of the pharyngeal muscles. Qualitative monitoring of neuromuscular block is not reliable.[1-6]

64. The use of quantitative monitoring of neuromuscular blockade (BNM) is necessary whenever neuromuscular blocking drugs are used throughout the surgical procedure.

High level of evidence. Strong recommendation.

REFERENCES

1. Hristovska AM, Duch P, Allingstrup M, Afshari A. The comparative efficacy and safety of sugammadex and neostigmine in reversing neuromuscular blockade in adults. A Cochrane systematic review with meta-analysis and trial sequential analysis. Anaesthesia. 2018; 73(5):631-641
2. Naguib M, Brull SJ, Kopman AF, Hunter JM, Fulesdi B, Arkes HR et al. Consensus Statement on perioperative use of neuromuscular monitoring. Anesth & Analg 2018;127:71-80.
3. Murphy GS. Neuromuscular monitoring in the perioperative period. Anesth & Analg 2018;126:464-68.
4. Brull SJ, Kopman AF. Current Status of Neuromuscular Reversal and Monitoring: Challenges and Opportunities. Anesthesiology. 2017;126(1):173-190.
5. Naguib M, Brull SJ, Johnson KB. Conceptual and technical insights into the basis of neuromuscular monitoring. Anaesthesia. 2017;72 Suppl 1:16-37.
6. Checketts MR, Alladi R, Ferguson K, Gemmell L, Handy JM, K AA Lein et al. Recommendations for standards of monitoring during anaesthesia and recovery 2015: Association of Anaesthetics of Great Britain and Ireland. Anaesthesia 2016;71:85-93.

Neuromuscular Block Depth

INTRODUCTION

Deep neuromuscular block is very appropriate for abdominal surgery (open and laparoscopic) and for the obese patient. Improves the conditions of the surgical space during laparoscopy and it facilitates the use of low intra-abdominal pressures (<10-12 cm H2O), which probably leads to better postoperative results. It is optimized if used throughout the surgery.[1-3] To achieve this, good communication between anesthesiologists and surgeons is crucial. [4-5]

65. The use of deep neuromuscular block (PTC 1-2) is recommended to improve visualization of the surgical field, both in open and laparoscopic surgery, and to use the lowest possible intra-abdominal pressures in laparoscopy, favoring postoperative recovery.

High level of evidence. Strong recommendation.

REFERENCES

1. Park SK, Son YG, Yoo S, Lim T, Kim WH, Kim JT. Deep vs. moderate neuromuscular blockade during laparoscopic surgery: A systematic review and meta-analysis. Eur J Anaesthesiol. 2018;35(11):867-875.
2. Bruintjes MH, van Helden EV, Braat AE, Dahan A, Scheffer GJ, van Laarhoven CJ et al. Deep neuromuscular block to optimize surgical space conditions during laparoscopic surgery: a systematic review and meta-analysis. Br J Anaesth. 2017:118(6):834-842.
3. Fuchs-Buder T, De Roberts E, Braunaud L. Neuromuscular block in laparoscopic surgery. Minerva Anestesiol 2018;84(4):509-14.

4. Errando-Oyonarte CL, Moreno-Sanz C, Vila-Caral P, Ruíz de Adana-Belbel JC, Vazquez-Alonso E, Ramírez-Rodríguez JM et al. Recomendaciones sobre el uso de bloqueo neuromuscular profundo por parte de anestesiólogos y cirujanos. Consenso AQUILES (Anestesia QUIrúrgica para Lograr Eficiencia y Seguridad) Rev Esp Anestesiol Reanim 2017;64:95-104.
5. Madsen MV, Staehr-Rye AK, Gätke MR, Claudius C. Neuromuscular blockade for optimising surgical conditions during abdominal and gynaecological surgery: a systematic review. Acta Anaesthesiol Scand. 2015 ;59(1):1-16.

Reversal of neuromuscular block

INTRODUCTION

Reversal of neuromuscular block can be done with sugammadex in any phase of depth of anesthesia or with neostigmine from a moderate block of at least 3 TOF responses, but extubating of the patient should only be performed after the patient has a TOFr ≥ 0.9. Residual blockage is frequent despite the administration of reversing drugs (especially with neostigmine) and without quantitative monitoring, much greater in spontaneous reversion of BNM, and is associated with pulmonary complications during the postoperative period.[1-5]

66. It is recommended to check the reversal of BNM until a TOF ratio greater than or equal to 0.9 is obtained in the adductor pollicis muscle during the anesthetic discharge prior to extubating to avoid residual neuromuscular block and reduce respiratory complications.

High level of evidence. Strong recommendation.

REFERENCES

1. Kirmeier E, Eriksson LI, Lewald H, Jonsson Fagerlund M, Hoeft A, Hollmann M et al. Post-anaesthesia pulmonary complications after use of muscle relaxants (POPULAR): a multicentre, prospective observational study. Lancet Respir Med. 2019;7(2):129-140.
2. Tajaate N, Schreiber JU, Fuchs-Buder T, Jelting Y, Kranke P. Neostigmine-based reversal of intermediate acting neuromuscular blocking agents to prevent postoperative residual paralysis: A systematic review. Eur J Anaesthesiol. 2018;35(3):184-192.
3. Murphy GS. Neuromuscular Monitoring in the Perioperative Period. Anesth Analg. 2018; 126(2):464-468.
4. Hunter JM. Reversal of residual neuromuscular block: complications associated with perioperative management of muscle relaxation. Br J Anaesth. 2017 D 1;119(suppl_1):i53-i62.
5. Brull SJ, Kopman AF. Current Status of Neuromuscular Reversal and Monitoring: Challenges and Opportunities. Anesthesiology. 2017;126(1):173-190.

BNM reversal with rocuronium

INTRODUCTION

The rocuronium-sugammadex combination for neuromuscular blockade and its reversal has shown in different studies, it is much faster, reaching the TOF ratio> 0.9 from intense, deep, and moderate blocks, ensuring the recovery of patients, leading to fewer respiratory complications than those reversed with neostigmine.[1-8]

67. It is recommended to perform the reversal of BNM with sugammadex instead of neostigmine when rocuronium bromide has been used, as it is faster and safer.

High level of evidence. Strong recommendation.

REFERENCES

1. Hristovska AM, Duch P, Allingstrup M, Afshari A. The comparative efficacy and safety of sugammadex and neostigmine in reversing neuromuscular blockade in adults. A Cochrane systematic review with meta-analysis and trial sequential analysis. Anaesthesia. 2018;73 (5):631-641.
2. Hafeez KR, Tuteja A, Singh M, Wong DT, Nagappa M, Chung F et al. Postoperative complications with neuromuscular blocking drugs and/or reversal agents in obstructive sleep apnea patients: a systematic review. BMC Anesthesiol. 2018;18(1):91.
3. Carron M, Zarantonello F, Lazzarotto N, Tellaroli P, Ori C. Role of sugammadex in accelerating postoperative discharge: A meta-analysis. J Clin Anesth. 2017;39:38-44.
4. Carron M, Zarantonello F, Tellaroli P, Ori C. Efficacy and safety of sugammadex compared to neostigmine for reversal of neuromuscular blockade: a meta-analysis of randomized controlled trial. J Clin Anesth. 2016;35:1-12.
5. Abad-Gurumeta A, Ripollés-Melchor J, Casans-Francés R, Espinosa A, Martínez-Hurtado E, Fernández-Pérez C, et al. Evidence Anaesthesia Review Group. A systematic review of sugammadex vs neostigmine for reversal of neuromuscular blockade. Anaesthesia. 2015;70(12):1441-52.
6. Manfred Blobner, Jennifer M Hunter, Claude Meistelman, Andreas Hoeft, Markus W Hollmann, et al. Use of a Train-Of-Four Ratio of 0.95 Versus 0.9 for Tracheal Extubation: An Exploratory Analysis of POPULAR Data. Br J Anaesth.2020;124(1):63-72.
7. Kheterpal S, Vaughn MT, Dubovoy TZ, Shah NJ, Bash LD, Colquhoun DA, et al. Sugammadex versus Neostigmine for Reversal of Neuromuscular Blockade and Postoperative Pulmonary Complications (STRONGER): A Multicenter Matched Cohort Analysis. Anesthesiology. 2020;132(6):1371-1381.
8. Raval AD, Uyei J, Karabis A, Bash LD, Brull SJ. Incidence of residual neuromuscular blockade and use of neuromuscular blocking agents with or without antagonists: A systematic review and meta-analysis of randomized controlled trials. J Clin Anesth. 2020; 64:109818 (On line first: 15th April 2020).

Normothermia

INTRODUCTION

Involuntary perioperative hypothermia can negatively affect the outcome of surgery and the postoperative clinical course of the patient, being associated with an increase in postoperative morbidity[1-3], with an increased incidence of poor healing, infection of the surgical wound, cardiovascular complications, tremor and increased blood loss[4,5]. It is also related to a delay in discharge from resuscitation units and from the hospital, and consequently with an increase in the costs of the process[5].

68. It is recommended to prevent and avoid involuntary perioperative hypothermia.

High level of evidence. Strong recommendation.

REFERENCES

1. Torossian A, Bräuer A, Höcker J, Bein B, Wulf H, Horn EP. Preventing inadvertent perioperative hypothermia. Clinical Practice Guideline. Dtsch Arztebl Int. 2015; 112(10): 166-72.

2. Sessler DI. Perioperative thermoregulation and heat balance. Lancet. 2016;387(10038): 2655-2664.

3. Bindu B, Bindra A, Rath G. Temperature management under general anesthesia: Compulsion or option. J Anaesthesiol Clin Pharmacol. 2017;33(3):306-316.

4. Ruetzler K, Kurz A. Consequences of perioperative hypothermia. Handb Clin Neurol. 2018;157:687-697.

5. Calvo Vecino JM, Casans Francés R, Ripollés Melchor J, Marín Zaldívar C, Gómez Ríos MA, Pérez Ferrer A, et al. Clinical practice guideline. Unintentional perioperative hypothermia. Rev Esp Anestesiol Reanim. 2018;65(10):564-588.

Temperature monitoring

INTRODUCTION

Temperature should be monitored in all patients undergoing general anesthesia lasting more than 30 minutes or whose surgery lasts more than one hour, regardless of the anesthetic technique used[1,2]. Temperature control allows the adoption of early measures to avoid hypothermia, which can even be applied preventively[3], as well as the early detection and treatment of fever and / or hyperthermia[4].

69. The temperature of the patients should be controlled to guarantee normothermia in the perioperative period.

High level of evidence. Strong recommendation.

REFERENCES

1. Sessler DI. Perioperative thermoregulation and heat balance. Lancet. 2016;387(10038): 2655-2664.

2. Calvo Vecino JM, Casans Francés R, Ripollés Melchor J, Marín Zaldívar C, Gómez Ríos MA, Pérez Ferrer A, et al. Clinical practice guideline. Unintentional perioperative hypothermia. Rev Esp Anestesiol Reanim. 2018;65(10):564-588.

3. Ohki K, Kawano R, Yoshida M, Kanosue I, Yamamoto K. Normothermia is Best Achieved by Warming Above and Below with Pre-warming Adjunct: A Comparison of Conductive Fabric Versus Forced-air and Water. Surg Technol Int. 2019;34:40-45.

4. Hooper VD, Chard R, Clifford T, Fetzer S, Fossum S, Godden B, et al. ASPAN's evidence-based clinical practice guideline for the promotion of perioperative normothermia: second edition. J Perianesth Nurs. 2010;25(6):346-65.

Active preoperative warm-up

INTRODUCTION

Active warm-up strategies should be started 20-30 minutes before surgery, also known as warm-up strategies[2,4]. These strategies should be maintained during the intraoperative period to maintain normothermia[1], especially if the duration of anesthesia is going to be greater than 60 minutes[3] and in those patients with a higher risk of suffering perioperative hypothermia, do not intentional, such as those over 50 years of age or with a high surgical risk. It seems that these warming strategies could be related to the reduction of surgical wound infections compared to the use of non-active methods.

70. Active warm-up strategies should be started prior to surgery.

High level of evidence. Strong recommendation.

REFERENCES

1. Torossian A, Bräuer A, Höcker J, Bein B, Wulf H, Horn EP. Preventing inadvertent perioperative hypothermia. Clinical Practice Guideline. Dtsch Arztebl Int. 2015; 112(10):166-72.
2. Warttig S, Alderson P, Campbell G, Smith AF. Interventions for treating inadvertent postoperative hypothermia. Cochrane Database Syst Rev. 2014 Nov 20;(11):CD009892.
3. Hooper VD, Chard R, Clifford T, Fetzer S, Fossum S, Godden B, et al. ASPAN's evidence-based clinical practice guideline for the promotion of perioperative normothermia: second edition. J Perianesth Nurs. 2010;25(6):346-65.
4. Akhtar Z, Hesler BD, Fiffick AN, Mascha EJ, Sessler DI, Kurz A, et al. A randomized trial of prewarming on patient satisfaction and thermal comfort in outpatient surgery. J Clin Anesth. 2016;33:376-85.

Operating room temperature

INTRODUCTION

There are passive measures for the prevention of hypothermia, among which is the temperature of the operating room[1]. An increase in the temperature of the operating room protects patients from unintentional hypothermia, both during the intervention and during their admission to the resuscitation unit[2]. The available evidence shows higher central temperatures in those patients operated on in operating rooms with a temperature of at least 21 º C[3], although lower temperatures may be necessary in some types of surgeries.

71. The temperature in the operating room should be at least 21 ° C for adult patients.

High level of evidence. Strong recommendation.

REFERENCES

1. Torossian A, Bräuer A, Höcker J, Bein B, Wulf H, Horn EP. Preventing inadvertent perioperative hypothermia. Clinical Practice Guideline. Dtsch Arztebl Int. 2015 Mar 6; 112(10): 166-72.
2. Gómez-Romero FJ, Fernández-Prada M, Navarro-Gracia JF. Prevention of Surgical Site Infection: Analysis and Narrative Review of Clinical Practice Guidelines. Cir Esp. 2017;95 (9):490-502.
3. Hooper VD. Revisiting the ASPAN evidence-based clinical practice guideline for the promotion of perioperative normothermia. J Perianesth Nurs. 2010;25(6):343-5.

Thermal isolation of the patient during the intraoperative

Despite being a passive measure of temperature control, covering the largest possible body surface area with sheets and one or more blankets or similar materials, prevents losses in body temperature, contributing in a simple way to the thermal insulation of the patient's body in the perioperative period[1,2,3]. This measure should be part of the normal care of the patient.

72. During the perioperative period, the largest possible surface area of the body should be thermally insulated.

High level of evidence. Strong recommendation.

REFERENCES

1. Torossian A, Bräuer A, Höcker J, Bein B, Wulf H, Horn EP. Preventing inadvertent perioperative hypothermia. Clinical Practice Guideline. Dtsch Arztebl Int. 2015; 112(10):166-72.
2. Warttig S, Alderson P, Campbell G, Smith AF. Interventions for treating inadvertent postoperative hypothermia. Cochrane Database Syst Rev. 2014 Nov 20;(11):CD009892.
3. Madrid E, Urrútia G, Roqué i Figuls M, Pardo-Hernandez H, Campos JM et al. Active body surface warming systems for preventing complications caused by inadvertent perioperative hypothermia in adults. Cochrane Database Syst Rev. 2016 Apr 21;4:CD009016.

Fluid warming during intra-operative

INTRODUCTION

The administration of intravenous fluids or irrigation at low temperatures should be avoided since it increases the risk of hypothermia in the perioperative period. Fluids administered intravenously to the patient should be warmed first, this recommendation being applicable to irrigation fluids[1,2]. Warming intravenous fluids should be considered up to an hour before surgery. Warming of intravenous fluids has proven to be a favorable measure in terms of cost-effectiveness compared to not warming even in cases of lower surgical risk, lower risk of cardiac complications, and short duration of surgery[2,3].

73. Infusions, cavity fluid infusions, and blood transfusions given at doses >500 ml / hr. should be warmed first.

High level of evidence. Strong recommendation.

REFERENCES

1. Torossian A, Bräuer A, Höcker J, Bein B, Wulf H, Horn EP. Preventing inadvertent perioperative hypothermia. Clinical Practice Guideline. Dtsch Arztebl Int. 2015; 112(10): 166-72.
2. Campbell G, Alderson P, Smith AF, Warttig S. Warming of intravenous and irrigation fluids for preventing inadvertent perioperative hypothermia. Cochrane Database Syst Rev. 2015 Apr 13;(4):CD009891.
3. Warttig S, Alderson P, Campbell G, Smith AF. Interventions for treating inadvertent postoperative hypothermia. Cochrane Database Syst Rev. 2014 Nov 20;(11):CD009892.

Active intraoperative warming measures

INTRODUCTION

Active intraoperative warming measures should be performed[1]. These measures must be applied as much in advance as possible[2]. Within active skin heating systems, the most evaluated strategies are convective and conductive hot air, with strategies being cost-effective even in those patients with lower surgical risk and short duration of surgery.[3]

74. Intraoperative active warming measures are indicated by the administration of convective or conductive heat to maintain normothermia.

High level of evidence. Strong recommendation.

REFERENCES

1. Torossian A, Bräuer A, Höcker J, Bein B, Wulf H, Horn EP. Preventing inadvertent perioperative hypothermia. Clinical Practice Guideline. Dtsch Arztebl Int. 2015 Mar 6; 112(10): 166-72. doi: 10.3238/arztebl.2015.0166. PMID: 25837741.
2. Warttig S, Alderson P, Campbell G, Smith AF. Interventions for treating inadvertent postoperative hypothermia. Cochrane Database Syst Rev. 2014 Nov 20;(11): CD009892. doi: 10.1002/14651858.CD009892.pub2. Review. PubMed PMID: 25411963.
3. Calvo Vecino JM, Casans Francés R, Ripollés Melchor J, Marín Zaldívar C, Gómez Ríos MA, Pérez Ferrer A, et al. Clinical practice guideline. Unintentional perioperative hypothermia. Rev Esp Anestesiol Reanim. 2018;65(10):564-588.

Temperature in anesthetic eduction

INTRODUCTION

A higher temperature must be maintained at 36 º C throughout the surgical process, applying the necessary measures so that the anesthetic emission is carried out under normothermic conditions. Postoperative hypothermia is related to a longer stay in the postanesthetic resuscitation unit, in addition to the fact that chills have been described as a cause of intense discomfort after surgery, comparable to postoperative pain [1,2,3].

75. The removal of general anesthesia should take place at normal body temperature.

High level of evidence. Strong recommendation.

REFERENCES

1. Torossian A, Bräuer A, Höcker J, Bein B, Wulf H, Horn EP. Preventing inadvertent perioperative hypothermia. Clinical Practice Guideline. Dtsch Arztebl Int. 2015; 112(10): 166-72.
2. Bindu B, Bindra A, Rath G. Temperature management under general anesthesia: Compulsion or option. J Anaesthesiol Clin Pharmacol. 2017;33(3):306-316.
3. Warttig S, Alderson P, Campbell G, Smith AF. Interventions for treating inadvertent postoperative hypothermia. Cochrane Database Syst Rev. 2014 Nov 20;(11): CD009892.

Intraoperative fluid therapy

INTRODUCTION

Intraoperative fluid therapy plays an essential role in the treatment of the surgical patient.co by having an important influence on postoperative results. The essential factors to consider are the monitoring, the objectives to be achieved, as well as the choice of the type of solution, the volume, and the time of its administration. All of them have been the subject of research in recent literature.[1]

Goal-guided fluid therapy

INTRODUCTION

The objective of intraoperative fluid therapy is to maintain tissue perfusion with an adequate circulating volume while maintaining electrolyte homeostasis.[1] Hypovolemia may determine a greater risk of hypoperfusion and organ damage, while hypervolemia may cause interstitial edema, impaired healing, and coagulation. cardiopulmonary complications, as well as postoperative ileus.[2] Therefore, adjusted, and individualized therapy based on well-defined protocols should be the goal to optimize its efficacy and avoid iatrogenesis.[3]

Systolic Volume (SV) and Systolic Volume Variation (SVV)

INTRODUCTION

There is extensive literature that advocates individualizing fluid therapy or goal-directed hemodynamic therapy using advanced hemodynamic monitoring to optimize SV and reduce SVV.[2-4]

76. The use of adequate monitoring (VS or VVS) is recommended to guide intraoperative administration of fluids in patients at risk.

High level of evidence. Strong recommendation.

REFERENCES

1. Makaryus R, Miller TE, Gan TJ. Current concepts of fluid management in enhanced recovery pathways. Br J Anaesth 2018; 120: 376-383.
2. Joosten A, Delaporte A, Ickx B, Touihri K, Stany I, Barvais L, et al. Crystalloid versus colloid for intraoperative goal-directed fluid therapy using a closed-loop system: A randomized, double-blinded, controllled trial in major abdminal surgery. Anesthesiology 2018; 128:55-66.
3. Kapoor PM, Magoon R, Rawal RS, Mehta Y, Taneja S, Ravi R, et al. Goal-directed therapy improves the outcome of high-risk cardiac patients undergoing off-pump coronary artery bypass. Ann Card Anaesth 2017; 20: 83-9.
4. Bacchin MR, Ceria CM, Giannone S, Ghisi D, Stagni G, Greggi T, et al. Goal-direted fluid therapy base don stroke volumen variation in patients undergoing major spine surgery in the prone position: A cohort study. Spine 2016; 41:E1131-7.

Systolic Volume Variation (SVV) and fluid response

INTRODUCTION

Positive pressure mechanical ventilation induces a cyclical reduction in left ventricular is due to a decrease in venous return and more pronounced in hypovolemia. Changes in preload during the respiratory cycle led to variations in SV.[1] These variations are estimated by pulse contour analysis. Fluid responsiveness is generally defined as an increase in SV equal to or greater than 10% .[2]

77. In cases where there is an SV drop> 10% or SVV> 10%, fluid resuscitation is indicated (there is no preference between colloids or crystalloids).

High level of evidence. Strong recommendation.

REFERENCES

1. Kendrick JB, Kaye AD, Tong Y, Belani K, Urman, Hoffman Ch, Liu H. Goal-directed fluid therapy in the perioperative setting. J Anesth Clin Pharm 2019; 35 (Suppl 1): S29-S34.
2. D'Angelo M, Kyle Hodgen R. Wet or Dry? A Review of Intravenous Fluid Administration in Anesthesia Practice. Annu Rev Nurs Res. 2017;35(1):221-239.

Fluid balance

INTRODUCTION

Restrictive policies are associated with a significantly higher risk of acute kidney injury than liberal fluid therapy.[1,2] A goal of 0 balance may be too restrictive, so a moderately liberal fluid regimen may be recommended to achieve a positive balance of 1 to 2 L at the end of major surgery.[3,4]

78. A moderate continuous fluid infusion is recommended, yielding a positive balance at the end of surgery of 1 to 2 l. to avoid postoperative acute kidney damage.

High level of evidence. Strong recommendation.

REFERENCES

1. Miller TE, and Myles PS. Perioperative Fluid Therapy for Major Surgery. Anesthesiology 2019; 130:825-32.
2. Myles PS, Bellomo R, Corcoran T, Forbes A, Peytor P, Story D et al. Restrictive versus Liberal Fluid Therapy for Major Abdominal Surgery (RELIEF study). N Eng J Med 2018; 378:2263-74.
3. Brandstrump B. Finding the right Balance. N Engl J Med 2018:378; 2335-6.
4. Miller TE, Pearse RM. Perioperative fluid management: moving toward mor answers tan questions-A commentary on the RELIEF study. Periop Med 2019;8:2.

High risk patients

INTRODUCTION

Current evidence recommends the use of goal-directed therapy in high-risk anesthetic patients and high-risk surgeries. Very restrictive or very liberal therapies in the administration of fluids have serious consequences in patients with higher surgical risk, so what must be individualized according to the hemodynamic parameters of VS and SVV.[1-4]

79. In high-risk patients, it is recommended to maintain individualized fluid therapy with a moderately positive balance and continuous monitoring of SV or SVV.

Moderate level of evidence. Strong recommendation.

REFERENCES

1. Miller TE, and Myles PS. Perioperative Fluid Therapy for Major Surgery. Anesthesiology 2019; 130:825-32.
2. Gupta R, Gan TJ. Peri-operative fluid management to enhance recovery. Anaesthesia. 2016;71 Suppl 1:40-5.

3. Boland MR, Reynolds I, McCawley N, Galvin E, El-Masry S, Deasy J, McNamara DA. Liberal perioperative fluid administration is an independent risk factor for morbidity and is associated with longer hospital stay after rectal cancer surgery. Ann R Coll Surg Engl. 2017;99(2):113-116.

4. Bednarczyk JM, Fridfinnson JA, Kumar A, Blanchard L, Rabbani R, Bell D et al. Incorporating Dynamic Assessment of Fluid Responsiveness Into Goal-Directed Therapy: A Systematic Review and Meta-Analysis. Crit Care Med. 2017;45(9):1538-1545.

No response to volume

INTRODUCTION

The passive leg elevation test accompanied by an increase in blood pressure or SV allows a simple and immediate prediction of the response to fluids. A negative test has a low probability of response. In this case, therapy should be oriented to the use of vasopressors or inotropes.[1,2]

80. Intraoperative hypotension without response to passive leg raising should be treated with vasopressors (checking for variations in blood pressure, SV and SVV).

Moderate level of evidence. Strong recommendation.

REFERENCES

1. Futier E, Letirant JY, Guinot PG, Godet T, lorne E, Curvillon P et al. Effect of individualized vs standard blood pressure management strategies on postoperative organ dysfunction among high-risk patients undergoing major surgery: A randomized clinical trial. JAMA 2017; 318: 1346-57.

2. Bentzer P, Griesdale DE, Boyd J, MacLean K, Sirounis D, and Ayas NT. Will this the nodynamiclly no dynamically instable patient respond to a bolus of intravenous fluids? Jana 2016; 316:1298-309.

Mean arterial pressure

INTRODUCTION

There is increasing evidence that even short periods of intraoperative hypotension, defined as a mean arterial pressure lower than 65 mmHg, they are associated with myocardial and renal damage, so episodes of intraoperative hypotension should be avoided to reduce the risk of myocardial ischemia or acute renal failure.[1]

81. A range of mean arterial pressure greater than or equal to 65 mm Hg should be established.

High level of evidence. Strong recommendation.

REFERENCES

1. Salmasi V, Maheshwari K, Yang D, Mascha EJ, Singh A, Sessler DI, et al. Relationship between Intraoperative Hypotension, Defined by Either Reduction from Baseline or Absolute Thresholds, and Acute Kidney and Myocardial Injury after Noncardiac Surgery: A Retrospective Cohort Analysis. Anesthesiology. 2017; 126(1):47-65.

Cardiac index

INTRODUCTION

If a patient has an optimized volume (does not respond to fluids) and remains hypotensive with a cardiac index less than 2.5 l / min / m2, inotropes should be considered.[1,2]

82. A CI> 2.5 l / min / m2 should be maintained, using inotropes in cases of non-response to volume.

High level of evidence. Strong recommendation.

REFERENCES

1. Calvo-Vecino JM, Ripolles_melchor J, Mythen MG, Casans-Francés R, Balik A, Artacho JP, et al. Effect of goal-directed haemodynamic therapy on postoperative complications in low-moderate risk surgical patients: A multicenter randomised controlled trial (FEDORA trial). Br J Anaesth 2018; 120:734-44.
2. Ripolles-Melchor J, Chappell D, Espinosa A, Mhyten MG, Abad-Gurumeta A, Bergese SD, et al. Fluid therapy recommendations for major abdominal surgery. Via RICA recommendations revisited. Part III: goal directed hemodinamic therapy. Rationale for maintaining vascular tone and contractility. Rev Esp Anestesiolo Reani 2107; 64: 348-59.

Intraoperative hemodynamic monitoring

INTRODUCTION

Esophageal Doppler is currently the method most supported by the evidence.[1] However, the introduction of less invasive pulse contour analysis-based monitoring has made it possible to generalize goal-guided therapy and obtain more extensive evidence.[2,3]

83. Monitoring by esophageal Doppler or methods based on validated pulse contour analysis is preferred.

High level of evidence. Strong recommendation.

REFERENCES

1. Ripollés-Melchor J, Casans-Francés R, Espinosa A, Abad-Gurumeta A, Fedheiser A, Lopez-Timoneda F, et al. Goal directed hemodynamic therapy based in esophageal Doppler Flow parameters: A systematic review, meta_analysis and trial sequential análisis. Rev Esp Anestesiolo Reani 2016; 63:384-405.
2. Xu C, Peng J, Liu S, Huang Y, Guo X, Xiao H, et al. Goal-directed fluid therapy versus conventional fluid therapy in colorectal surgery: A meta análisis of randomized controlled trials. Inter J Surg 2018, 56:264-73.
3. LI MQ, Yang LQ, Zhou I, Liu H. Non-invasive cardiaca output measurement: where we now? J Anesth Perioper Med 2018; 5:221-7.

Solution type

INTRODUCTION

Among crystalloids, evidence suggests that balanced solutions with electrolytes and a close acid-base balance close to a plasmatic one is preferable to solutions rich in chloride since these can cause hyperchloremia, metabolic acidosis, renal vasoconstriction, and acute renal damage.[1-4]

84. The primary maintenance intravenous fluid should be a balanced isotonic crystalloid solution.

High level of evidence. Strong recommendation.

REFERENCES

1. Hammond DA, Lam SW, Rech MA, Smith MN, Westrick J, Trivedi AP, et al. Balanced Crystalloids Versus Saline in Critically Ill Adults: A Systematic Review and Meta-analysis. Ann Pharmacother. 2020;54:5-13.
2. Pfortmueller CA, Funk G-C, Reiterer C, Schrott A, Zotti O, Kabon B, et al. Normal saline versus balanced crystalloid for goal-directed perioperative fluid therapy in major abdominal surgery: a double-blind randomised controlled study. B J Anaesth 2018; 120:274-83.
3. Self WH, Semler MW, Wanderer JP, Wang L, Byrne DW, Collins SP, et al. Balanced crystalloids versus saline in noncritically ill adults. N Engl J Med 2108; 378:819-28.
4. Semler MW, Self WH, Wanderer JP, Ehrenfeld JM, Wang I, Byrne DW, et al. Balanced crystalloids versus saline in critically ill adults. N England J Med 2018; 378:829-39.

Resuscitation fluid therapy

INTRODUCTION

For fluid therapy in resuscitation, the use of balanced crystalloids is recommended, 2-3 liters for initial resuscitation in hypovolemic shock and hemodynamic monitoring to guide the additional administration of fluids. Several clinical trials have shown that the use of balanced crystalloids instead of saline solution prevents developing hypotension, the need for vasopressors, renal dysfunction and the need for renal replacement therapy as well as reducing mortality.[1-6]

85. For fluid therapy in resuscitation, the use of balanced crystalloids is recommended, 2-3 liters for initial resuscitation in hypovolemic shock and hemodynamic monitoring to guide the additional administration of fluids.

Moderate level of evidence. Strong recommendation.

REFERENCES

1. Semler MW, Self WH, Wanderer JP, Wang L, Byrne DW, Collins SP, et al. Balanced Crystalloids versus Saline in Critically Ill Adults. N Engl J Med. 2018;378:829-839.
2. Semler MW, Kellum JA. Balanced Crystalloid Solutions. Am J Respir Crit Care Med. 2019; 199:952-960.
3. Casey JD, Brown RM, Semler MW. Resuscitation fluids. Curr Opin Crit Care. 2018;24:512-518.
4. Bampoe S, Odor PM, Dushianthan A, Bennett-Guerrero E, Cro S, Gan TJ, et al. Perioperative administration of buffered versus non-buffered crystalloid intravenous fluid to improve outcomes following adult surgical procedures. Cochrane Database Syst Rev. 2017;Sep21; 9:CD004089.
5. Odor PM, Bampoe S, Dushianthan A, Bennett-Guerrero E, Cro S, Gan TJ, et al. Perioperative administration of buffered versus non-buffered crystalloid intravenous fluid to improve outcomes following adult surgical procedures: a Cochrane systematic review. Perioper Med (Lond). 2018;13;7:27.
6. Reddy S, Weinberg L, Young P. Crystalloid fluid therapy. Crit Care. 2016; 15;20:59.

Tranexamic Acid

INTRODUCTION

The administration of tranexamic acid (prophylactic or after surgery) is recommended, except if contraindicated due to thrombotic risk or allergy. It is indicated in cardiac surgery, orthopedic (intravenous or topical), maxillofacial, prostatic, and gynecological. Not recommended in cases of severe kidney failure or if there is a history of epilepsy. [1-5]

Tranexamic acid can reduce the need for blood transfusion in adults undergoing surgery. This avoids serious risks associated with blood transfusion, such as infection, fluid overload, and improper blood transfusions. It can also reduce the length of hospital stays and the cost for the National Health System (*). [6]

86. It is recommended that all adults undergoing surgery and are expected to have moderate to severe blood loss be offered tranexamic acid.

High level of evidence. Strong recommendation.

REFERENCES.

1. Blood transfusion Quality standard [QS138] Published date: December 2016. Quality statement 2: Tranexamic acid for adults https://www.nice.org.uk/guidance/qs138/chapter/Quality-statement-2-Tranexamic-acid-for-adults (último acceso 2020).
2. Xu Y, Sun S, Feng Q, Zhang G, Dong B, Wang X, et al. The efficiency and safety of oral tranexamic acid in total hip arthroplasty: A meta-analysis. Medicine (Baltimore). 2019;98: e17796. doi: 10.1097/MD.0000000000017796.
3. Zhang Y, Bai Y, Chen M, Zhou Y, Yu X, Zhou H, et al. The safety and efficiency of intravenous administration of tranexamic acid in coronary artery bypass grafting (CABG): a meta-analysis of 28 randomized controlled trials. BMC Anesthesiol. 2019;19:104. doi: 10.1186/s12871-019-0761-3.
4. El-Menyar A, Sathian B, Asim M, Latifi R, Al-Thani H. Efficacy of prehospital administration of tranexamic acid in trauma patients: A meta-analysis of the randomized controlled trials. Am J Emerg Med. 2018;36:1079-1087. doi: 10.1016/j.ajem.2018.03.033.
5. Derzon JH, Clarke N, Alford A, Gross I, Shander A, Thurer R. Reducing red blood cell transfusion in orthopedic and cardiac surgeries with Antifibrinolytics: A laboratory medicine best practice systematic review and meta-analysis. Clin Biochem. 2019;71:1-13. doi: 10.1016/j.clinbiochem.2019.06.015.
6. Ripollés-Melchor J, Abad-Motos A, Díez-Remesal Y, Aseguinolaza-Pagola M, Padin-Barreiro L, Sánchez-Martín R, et al. Association Between Use of Enhanced Recovery After Surgery Protocol and Postoperative Complications in Total Hip and Knee Arthroplasty in the Postoperative Outcomes Within Enhanced Recovery After Surgery Protocol in Elective Total Hip and Knee Arthroplasty Study (POWER2). JAMA Surg. 2020;155:e196024.

Inspired Oxygen Fraction and risk of surgical infection

INTRODUCTION

Until recent years, the evidence-based guidelines of the World Health Organization (WHO) recommended a high fraction (80%) of inspired oxygen (FiO2) to reduce the incidence of surgical infection in adults under general anesthesia and tracheal intubation. However, recent clinical trials warn of the absence of benefits in reducing surgical infections

associated to the use of a high fraction of inspired oxygen (FiO2) (80%), as well as this measure itself.[1-5]

87. The supplemental use of inspired oxygen is not recommended in patients undergoing general anesthesia.

Moderate level of evidence. Weak recommendation

REFERENCES

1. de Jonge S, Egger M, Latif A, Loke YK, Berenholtz S, Boermeester M, et al. Effectiveness of 80% vs 30-35% fraction of inspired oxygen in patients undergoing surgery: an updated systematic review and meta-analysis. Br J Anaesth. 2019;122(3):325-334.
2. Chu DK, Kim LH, Young PJ, Zamiri N, Almenawer SA, Jaeschke R, et al. Mortality and morbidity in acutely ill adults treated with liberal versus conservative oxygen therapy (IOTA): a systematic review and meta-analysis. Lancet. 2018 28;391(10131):1693-1705.
3. Mattishent K, Thavarajah M, Sinha A, Peel A, Egger M, Solomkin J, et al. Safety of 80% vs 30-35% fraction of inspired oxygen in patients undergoing surgery: a systematic review and meta-analysis. Br J Anaesth. 2019;122(3):311-324.
4. Mayank M, Mohsina S, Sureshkumar S, Kundra P, Kate V. Effect of Perioperative High Oxygen Concentration on Postoperative SSI in Elective Colorectal Surgery-A Randomized Controlled Trial. J Gastrointest Surg. 2019 ;23(1):145-152.
5. Cohen B, Schacham YN, Ruetzler K, Ahuja S, Yang D, Mascha EJ, et al. Effect of intraoperative hyperoxia on the incidence of surgical site infections: a meta-analysis. Br J Anaesth. 2018;120(6): 1176-1186.

Surgical approach and MIS incisions (minimally invasive surgery)

INTRODUCTION

The introduction of minimally invasive surgery (laparoscopic and robotic) has notably improved the well-being of patients, since pain, surgical stress[1], opioid consumption[2] and blood loss are reduced, early ambulation is improved all of which, combined, decrease hospital stay[3,4]; Therefore, if the surgical and oncological results do not differ from the surgical techniques, MIS is recommended.[5,6,7]

88. Minimally invasive surgery is recommended, provided that surgical and oncological results do not differ from surgical techniques.

High level of evidence. Strong recommendation.

REFERENCES

1. Gustafsson UO, Scott MJ, Hubner M, Nygren J, Demartines N, Francis N, et al.Guidelines for Perioperative Care in Elective Colorectal Surgery: Enhanced Recovery After Surgery (ERAS). Society Recommendations: 2018. World J Surg. 2019; 43(3):659-695.
2. Ljungqvist O, Scott M, Fearon KC. Enhanced Recovery After Surgery: A Review. JAMA Surg. 2017 Mar 1; 152(3):292-298.
3. Obermair A, Janda M, Baker J, Kondalsamy-Chennakesavan S, Brand A, Hogg R, et al. Improved surgical safety after laparoscopic compared to open surgery for apparent early stage endometrial cancer: results from a randomised controlled trial. Eur J Cancer. 2012; 48(8):1147-53.

4. Vlug MS, Wind J, Hollmann MW, Ubbink DT, Cense HA, Engel AF et al. Laparoscopy in combination with fast track multimodal management is the best perioperative strategy in patients undergoing colonic surgery: a randomized clinical trial (LAFA-study). Ann Surg. 2011; 254: 868-875.
5. Walker JL, Piedmonte MR, Spirtos NM, Eisenkop SM, Schlaerth JB, Mannel RS, et al.Recurrence and survival after random assignment to laparoscopy versus laparotomy for comprehensive surgical staging of uterine cancer: Gynecologic Oncology Group LAP2 Study.J Clin Oncol. 2012; 30(7):695-700.
6. Kalogera E, Glaser GE, Kumar A, Dowdy SC, Langstraat CL.Enhanced Recovery after Minimally Invasive Gynecologic Procedures with Bowel Surgery: A Systematic Review. J Minim Invasive Gynecol. 2019; 26(2):288-298.
7. Li K, Lin T, Fan X, Xu K, Bi L, Duan Y, et al: Systematic review and meta-analysis of comparative studies reporting early outcomes after robot-assisted radical cystectomy versus open radical cystectomy. Cancer Treatment Reviews 2012 Oct; 39: pp. 551-560.

INCISIONS

INTRODUCTION

When a laparotomic approach is required, the transverse / oblique incision[1,2,3] appears to reduce pain and pulmonary complications, but there is not enough evidence, so the choice of surgical access depends on the surgeon, his experience and preference, as well as the characteristics of the patient.

89. The transverse incision is recommended in laparotomic surgery.

Moderate level of evidence. Strong recommendation.

REFERENCES

1. Santoro A, Boselli C, Renzi C, Gubbiotti F, Grassi V, Di Rocco G, et al. Transverse skin crease versus vertical midline incision versus laparoscopy for right hemicolectomy: a systematic review-current status of right hemicolectomy. Biomed Res Int. 2014;2014: 643685.
2. Seiler CM, Deckert A, Diener MK, Knaebel HP, Weigand MA, Victor N, et al.Midline versus transverse incision in major abdominal surgery: a randomized, double blind equivalence trial (POVATI: ISRCTN60734227). Ann Surg. 2009; 249(6):913-20.
3. Brown SR, Goodfellow PB.Transverse verses midline incisions for abdominal surgery. Cochrane Database Syst Rev. 2005; (4):CD005199.

Drains

INTRODUCTION

As a routine, drains have been used with the belief that by evacuating blood and serous collections postoperative infections could be prevented, but multiple studies in this regard[1,2,3] have not demonstrated this belief, so there is no evidence to back up its use on a routine basis[4,5,6].

90. It is recommended to avoid using drains on a routine basis

High level of evidence. Strong recommendation.

REFERENCES

1. Nelson G, Bakkum-Gamez J, Kalogera E, Glaser G, Altman A, Meyer LA, et al. Guidelines for perioperative care in gynecologic/oncology: Enhanced Recovery After Surgery (ERAS) Society recommendations-2019 update. Int J Gynecol Cancer. 2019 May;29(4):651-668.
2. Gustafsson UO, Scott MJ, Hubner M, Nygren J, Demartines N, Francis N, et al. Guidelines for Perioperative Care in Elective Colorectal Surgery: Enhanced Recovery After Surgery (ERAS®) Society Recommendations: 2018. World J Surg. 2019 Mar;43(3):659-695.
3. Denost Q, Rouanet P, Faucheron JL, Panis Y, Meunier B, Cotte E, et al. French Research Group of Rectal Cancer Surgery (GRECCAR). To Drain or Not to Drain Infraperitoneal Anastomosis After Rectal Excision for Cancer: The GRECCAR 5 Randomized Trial. Ann Surg. 2017 Mar;265(3):474-480
4. Carmichael JC, Keller DS, Baldini G, Bordeianou L, Weiss E, Lee L et al. Clinical Practice Guidelines for Enhanced Recovery After Colon and Rectal Surgery From the American Society of Colon and Rectal Surgeons and Society of American Gastrointestinal and Endoscopic Surgeons. Dis Colon Rectum. 2017 Aug;60(8):761-784.
5. Zhang HY, Zhao CL, Xie J, Ye YW, Sun JF, Ding ZH, et al. To drain or not to drain in colorectal anastomosis: a meta-analysis. Int J Colorectal Dis. 2016 May;31(5):951-960.
6. Musser JE, Assel M, Guglielmetti GB, Pathak P, Silberstein JL, Sjoberg DD, et al. Impact of routine use of surgical drains on incidence of complications with robot-assisted radical prostatectomy. J Endourol. 2014 Nov; 28(11):1333-7.

Nasogastric tube

INTRODUCTION

The different meta-analisis[1,2,3] have concluded that nasogastric intubation increases the risk of postoperative pneumonia after elective abdominal surgery, does not reduce the risk of suture dehiscence or anastomotic leakage[4,5,6], delays the start of feeding and causes discomfort in patients, therefore, there is no evidence to use them as a routine in abdominal surgery.

91. As a routine, the use of nasogastric tube is not recommended.

High level of evidence. Strong recommendation.

REFERENCES

1. Nelson G, Bakkum-Gamez J, Kalogera E, Glaser G, Altman A, Meyer LA, et al. Guidelines for perioperative care in gynecologic/oncology: Enhanced Recovery After Surgery (ERAS) Society recommendations-2019 update. Int J Gynecol Cancer. 2019 May;29(4):651-668.
2. Gustafsson UO, Scott MJ, Hubner M, Nygren J, Demartines N, Francis N, et al. Guidelines for Perioperative Care in Elective Colorectal Surgery: Enhanced Recovery After Surgery (ERAS®) Society Recommendations: 2018. World J Surg. 2019 Mar;43(3):659-695.
3. Carmichael JC, Keller DS, Baldini G, Bordeianou L, Weiss E, Lee L et al. Clinical Practice Guidelines for Enhanced Recovery After Colon and Rectal Surgery From the American Society of Colon and Rectal Surgeons and Society of American Gastrointestinal and Endoscopic Surgeons. Dis Colon Rectum. 2017 Aug;60(8):761-784.
4. Thorell A, MacCormick AD, Awad S, Reynolds N, Roulin D, Demartines N, et al. Guidelines for Perioperative Care in Bariatric Surgery: Enhanced Recovery After Surgery (ERAS) Society Recommendations. World J Surg. 2016 Sep;40(9):2065-83.

5. Weijs TJ, Kumagai K, Berkelmans GH, Nieuwenhuijzen GA, Nilsson M, Luyer MD. Nasogastric decompression following esophagectomy: a systematic literature review and meta-analysis. Dis Esophagus. 2017 Feb 1;30(3):1-8.
6. Rao W, Zhang X, Zhang J, Yan R, Hu Z, Wang Q. The role of nasogastric tube in decompression after elective colon and rectum surgery: a meta-analysis. Int J Colorectal Dis. 2011 Apr;26(4):423-9.

ANALGESIA

Perioperative analgesia

INTRODUCTION

Pain control has been a key point in enhanced recovery strategies since its inception.

The search for an analgesic method that confers a high degree of comfort for the patient without interfering in other key points of the enhanced recovery strategy such as early mobilization, paralytic ileus or postoperative nausea and vomiting or that could increase the rate complications or average stay, means that many perioperative analgesic strategies have been evaluated to be a part of enhanced recovery strategies.

Classically, most studies carried out on perioperative analgesia offered comparisons between the use of intravenous opiates and catheterization and infiltration of the epidural space at the thoracic level with local anesthetics, with or without added opiates, offering a clear superiority of the latter over the former, in major abdominal surgery. However, although today thoracic epidural catheterization continues to be the technique of choice in open major abdominal surgery, the development of minimally invasive surgical techniques, the infiltration of access ports with local anesthetics and the development of ultrasound-guided peripheral nerve block analgesic techniques, make epidurals not the first analgesic choice for laparoscopic surgery.

Finally, we must point out the importance of adjuvants within enhanced recovery analgesic strategies. Some of them are of more conventional use, such as non-steroidal anti-inflammatory drugs, but others are of more recent or controversial use, such as intravenous lidocaine, ketamine, magnesium sulfate or dexmedetomidine, which should also be considered when implementing an analgesic line of action in an enhanced recovery program. Based on the use of these, opioid-free anesthesia (OFA) is developed, aiming to abolish the use of opioids within the intra-operative stage, replacing them by a combination of drugs and/or techniques capable of maintaining a stable anesthesia, blocking analgesic stimuli in an efficient manner.

The different analgesic modalities are detailed below.

Epidural analgesia in open laparotomy

INTRODUCTION

There are both meta-analysis and high-quality randomized clinical studies that confirm the superiority of epidural analgesia over intravenous opioid analgesia, in terms of analgesic quality (particularly during the first 24 hours postoperatively and when the patient is wandering), and in the reduction of postoperative complications.[1,2]

Epidural analgesia has shown an improvement in gastrointestinal blood flow, providing a potential benefit in those patients undergoing major abdominal surgery. However, this increased flow is not accompanied by an increase in the patient's oxygen consumption.[1]

Due to the sympathetic blockage produced by epidural catheterization, it is accompanied by some degree of hemodynamic instability, resulting in an increased risk of hypotension, which can be resolved with vasoconstrictors.[1]

Epidural catheterization in major abdominal surgery presents better analgesic results than intravenous opiates, both with the patient at rest and in motion, especially during the first 24 hours after surgery. The use of epidural analgesia decreases the time to recover intestinal transit and the incidence of paralytic ileus, suggesting a decrease in hospital stay in open surgery.[2]

92. Epidural analgesia within combined anesthesia should be performed in all patients undergoing major open abdominal surgery.

High level of evidence. Strong recommendation.

REFERENCES

1. Salicath JH, Yeoh ECY, Bennett MH. Epidural analgesia versus patient-controlled intravenous analgesia for pain following intra-abdominal surgery in adults. Cochrane Database of Systematic Reviews 2018;8:CD010434.
2. Guay J, Nishimori M, Kopp S. Epidural local anaesthetics versus opioid-based analgesic regimens for postoperative gastrointestinal paralysis, vomiting and pain after abdominal surgery. Cochrane Database of Systematic Reviews 2016;7:CD001893.

Thoracic epidural

INTRODUCTION

Although there are few randomized clinical studies that evaluate the differences between the implementation at the thoracic or lumbar level of the epidural catheter, the existing ones do clearly indicate a better analgesic quality and fewer complications and lower extremity block in those patients in which a thoracic epidural catheterization is performed, with respect to those with a catheterization at the lumbar level. These data are also supported by prospective observational studies. In addition to all the above, most studies that support the use of epidural catheterization for analgesia in major abdominal surgery use thoracic puncture points to perform it.[1,2]

93. Catheterization of the epidural space for infusion of local anesthetics for analgesia in open major abdominal surgery should be performed at the thoracic level.

High level of evidence. Strong recommendation.

REFERENCES

1. Scott AM, Starling JR, Ruscher AE, DeLessio ST, Harms BA, Michelassi F, et al. Thoracic versus lumbar epidural anesthesia's effect on pain control and ileus resolution after restorative proctocolectomy. Surgery 1996;120(4):688-97292.
2. Pöpping DM, Zahn PK, Van Aken HK, Dasch B, Boche R, Pogatzki-Zahn EM. Effectiveness and safety of postoperative pain management: A survey of 18 925 consecutive patients between 1998 and 2006 (2nd revision): A database analysis of prospectively raised data. Br J Anaesth 2008;101(6):832-40293.

Neuraxial opioids

INTRODUCTION

The supply of small amounts of opiates together with local anesthetics applied epidurally improves the analgesic quality of the block to be performed, without causing a significant increase in complications for the patient or affecting the benefits of epidural analgesia in the recovery of intestinal motility. As a potential complication, it is worth mentioning the possible appearance of itching, although the incidence is not high. Regarding the opioid used, morphine, fentanyl or sufentanil seem to be just as effective in terms of analgesic quality. The effect of opioids at the epidural level is independent of the puncture point chosen for the epidural catheterization.[1]

94. Small doses of opioids should be added to the doses of local anesthetic to be delivered epidurally in major open surgery.

Moderate level of evidence. Strong recommendation.

REFERENCES

1. Guay J, Nishimori M, Kopp S. Epidural local anaesthetics versus opioid-based analgesic regimens for postoperative gastrointestinal paralysis, vomiting and pain after abdominal surgery. Cochrane Database of Systematic Reviews 2016;7: CD001893.

Analgesia without an epidural catheter arrangement

INTRODUCTION

If epidural analgesia is not used, the analgesic strategy should be individualized, seeking to reduce the use of opiates by applying different types of blockades, either spinal, local, regional, or infiltration of ports with local anesthetics, etc.[1]

In all other cases, the analgesic strategy should be individualized, trying to avoid the use of opiates, and favoring the use of locoregional blocks, spinal analgesia, or infiltration of ports with local anesthetics, especially considering the block of the transverse plane of the abdomen.[2]

95. When the provision of an epidural catheter is not possible in open major surgery, the analgesic strategy should be individualized, reducing the use of opiates, and favoring the use of locoregional blocks, spinal analgesia, or port infiltration with local anesthetics, especially considering blockade of the transverse plane of the abdomen.

Moderate level of evidence. Strong recommendation.

REFERENCES

1. Baeriswyl M, Zeiter F, Piubellini D, Kirkham KR, Albrecht E. The analgesic efficacy of transverse abdominis plane block versus epidural analgesia: a systematic review with meta-analysis. Medicine. 2018;97(26).
2. Shahait M, Lee DI. Application of TAP Block in laparoscopic urological surgery: current status and future directions. Curr Urol Rep. 2019;20:20.

Interfacial blocks: transverse plane block without the possibility of epidural block

INTRODUCTION

Block of the transverse plane of the abdomen can be considered an effective strategy in these cases, with analgesic quality comparable to epidural but with a lower risk profile as it does not produce hemodynamic alterations, preserves motor and sensory function of the lower extremities, and can be used more safely in patients on anticoagulant treatment. However, it has no effect on visceral pain, so it must necessarily be part of a multimodal analgesia protocol that combines different drugs or analgesic techniques with different mechanisms of action. Likewise, due to the duration of the TAP block by single puncture, in those cases where the possibility of intense pain is anticipated after the first 24 hours, catheterization and continuous perfusion in space should be considered.[1] However, blockade of the plane of the transverse has not shown superiority to epidural in any RCT.

96. Performing a bilateral transverse plane block with local anesthetics could benefit those patients who require open major abdominal surgery and who could not benefit from epidural analgesia.

Moderate level of evidence. Strong recommendation.

REFERENCES

1. Grupo de trabajo de la Guía de Práctica Clínica sobre Cuidados Perioperatorios en Cirugía Mayor Abdominal. Guía de Práctica Clínica sobre Cuidados Perioperatorios en Cirugía Mayor Abdominal. Ministerio de Sanidad, Servicios Sociales e Igualdad. Instituto Aragonés de Ciencias de la Salud (IACS); 2016 Guías de Práctica Clínica en el SNS.
2. Baeriswyl M, Zeiter F, Piubellini D, Kirkham KR, Albrecht E. The analgesic efficacy of transverse abdominis plane block versus epidural analgesia: a systematic review with meta-analysis. Medicine. 2018;97(26).
3. Shahait M, Lee DI. Application of TAP Block in laparoscopic urological surgery: current status and future directions. Curr Urol Rep. 2019;20:20.

Opioid free anesthesia (OFA)

INTRODUCTION

Perioperative opioid administration has long been one of the three pillars of “balanced anesthesia,” which in practice addressed perioperative pain relief and preventive analgesia as goals. Pain during anesthesia has typically been interpreted through the evaluation of surrogate signs such as the response of the sympathetic nervous system to surgical stimuli. However, the contribution of emotional experience during an unconscious state is questionable, and hemodynamic changes are prone to confound several physiological processes. Therefore, the assumption that it is necessary to treat these substitutes with opiates during general anesthesia may be poorly justified. Likewise, the use of opioid drugs for pain control is not safe but comes with different complications and side effects.[1]

Based on the above, in the last decade “opioid-free anesthesia” (OFA) has begun to become an alternative to the classical use of intravenous opioids during the intraoperative period. Opioid-free anesthesia is based on the idea that the complete abolition of opioid drugs in the intraoperative period has a positive impact on the expected results in the postoperative period, totally replacing them with a

combination of drugs and / or techniques that together can achieve stable anesthetic maintenance and effectively blocking analgesic stimuli. Drugs that have been proposed for this purpose include NMDA receptor antagonists (Ketamine, Lidocaine, Magnesium Sulfate), calcium channel blockers (local anesthetics), anti-inflammatories (steroids, NSAIDs, AL), or alpha 2 agonists (clonidine, dexmedetomidine).[2,3]

At this time there is no evidence that opioid-free anesthesia is clearly superior to classical balanced opioid-based anesthesia, even though it could reduce situations of opioid-induced hyperalgesia. Likewise, opioid-free anesthesia has shown a reduction in the side effects produced by opioids, such as nausea and vomiting. In bariatric surgery there are also indications about its usefulness to increase patient comfort and reduce adverse events such as desaturations or apneas in the immediate postoperative period.[4]

97. Opioid-free anesthesia in enhanced recovery may be an alternative to the use of intravenous opioids.

Moderate level of evidence. Weak recommendation

REFERENCES

1. Mulier JP, Dillemans B. Anaesthetic Factors Affecting Outcome After Bariatric Surgery, a Retrospective Levelled Regression Analysis. OBES SURG. 2019;29:1841-50.

2. Frauenknecht J, Kirkham KR, Jacot-Guillarmod A, Albrecht E. Analgesic impact of intra-operative opioids vs. opioid-free anaesthesia: a systematic review and meta-analysis. Anaesthesia. 2019;74:651-62.

3. Mulier JP, Wouters R, Dillemans B, Deckock MI. A Randomized Controlled, Double-Blind Trial Evaluating the Effect of Opioid-Free Versus Opioid General Anaesthesia on Postoperative Pain and Discomfort Measured by the QoR-40. J Clin Anesth Pain Med. 2018;2:2-6.

4. Mulier JP, Dillemans B. Deep Neuromuscular Blockade versus Remifentanil or Sevoflurane to Augment Measurable Laparoscopic Workspace during Bariatric Surgery Analysed by a Randomised Controlled Trial. Journal of Clinical Anesthesia and Pain Medicine. 2018;7:2-4.

Intraoperative intravenous lidocaine

INTRODUCTION

Research in other areas of pain, such as neuropathic pain or complex regional pain syndrome, has shown that the administration of lidocaine intravenously produces long-lasting analgesic effects, inhibiting the spontaneous generation of impulses from injured peripheral nerves and dorsal node root lymph close to the injured fibers and suppression of polysynaptic reflexes in the dorsal spinal horn.[1] Although pain in the perioperative setting is primarily inflammatory, it can also be neuropathic or based on hyperalgesia. All these entities could be improved by the administration of IV lidocaine at low doses, although its analgesic effect would be limited to the first 24 hours postoperatively.[2,3]. Furthermore, it could help intestinal function recovery and prevent developing paralytic ileus.

98. The use of intraoperative intravenous lidocaine is recommended as an adjunct medication in the reduction of postoperative pain and to improve the recovery of intestinal function in the immediate postoperative period, being an alternative to the use of intravenous opioids.

Nivel de evidencia moderado. Recomendación débil

REFERENCES

1. Cooke C, Kennedy ED, Foo I, Nimmo S, Speake D, Paterson HM, et al. Meta-analysis of the effect of perioperative intravenous lidocaine on return of gastrointestinal function after colorectal surgery. Tech Coloproctol. 2019;23:15-24.
2. Weibel S, Jelting Y, Pace NL, Helf A, Eberhart LH, Hahnenkamp K, et al. Continuous intravenous perioperative lidocaine infusion for postoperative pain and recovery in adults. Cochrane Database Syst Rev. 2018;6:CD009642.
3. MacFater WS, Rahiri J-L, Lauti M, Su'a B, Hill AG. Intravenous lignocaine in colorectal surgery: a systematic review. ANZ J Surg. 2017;87:879-85.
4. Weibel S, Jokinen J, Pace NL, Schnabel A, Hollmann MW, Hahnenkamp K, et al. Efficacy and safety of intravenous lidocaine for postoperative analgesia and recovery after surgery: a systematic review with trial sequential analysis. Br J Anaesth. 2016;116:770-83.

Intraoperative ketamine

INTRODUCTION

Ketamine could reduce the inflammatory reaction that occurs after surgery, decreasing IL6levels.[1] Perioperative intravenous ketamine probably reduces the consumption of postoperative analgesics and the intensity of pain without causing a significant increase in side effects at the level of central nervous system and probably reduces postoperative nausea and vomiting to a small extent, of questionable clinical relevance.[2,3]

99. IV ketamine should be given to those patients on major opioids for analgesia in major abdominal surgery.

Moderate level of evidence. Weak recommendation

REFERENCES

1. Brinck EC, Tiippana E, Heesen M, Bell RF, Straube S, Moore RA, et al. Perioperative intravenous ketamine for acute postoperative pain in adults. Cochrane Database Syst Rev. 2018;12:CD012033.
2. Nielsen RV, Fomsgaard JS, Siegel H, Martusevicius R, Nikolajsen L, Dahl JB, et al. Intraoperative ketamine reduces immediate postoperative opioid consumption after spinal fusion surgery in chronic pain patients with opioid dependency: a randomized, blinded trial. Pain. 2017;158:463-70.
3. Wang L, Johnston B, Kaushal A, Cheng D, Zhu F, Martin J. Ketamine added to morphine or hydromorphone patient-controlled analgesia for acute postoperative pain in adults: a systematic review and meta-analysis of randomized trials. Can J Anaesth. 2016;63:311-25.

Intraoperative magnesium sulfate

INTRODUCTION

Magnesium produces an inhibitory effect on the neuron by blocking glutamate NMDA receptors, which is the main excitatory neurotransmitter in the central nervous system. It has sedative and anticonvulsant properties, inhibits catecholamine secretion, and enhances neuromuscular block. It may reduce opioid use by modulating nociceptive stimulus.[1-5]

100. The use of intraoperative iv magnesium sulfate is recommended as an analgesic adjunct to improve pain control in patients undergoing abdominal surgery.

Moderate level of evidence. Weak recommendation

REFERENCES

1. Rodríguez-Rubio L, Nava E, Del Pozo JSG, Jordán J. Influence of the perioperative administration of magnesium sulfate on the total dose of anesthetics during general anesthesia. A systematic review and meta-analysis. J Clin Anesth. 2017;39:129-38.
2. Eizaga Rebollar R, García Palacios MV, Morales Guerrero J, Torres LM. Magnesium sulfate in pediatric anesthesia: the Super Adjuvant. Paediatr Anaesth. 2017;27:480-9.
3. Vicković S, Pjević M, Uvelin A, Pap D, Nikolić D, Lalić I. Magnesium Sulfate as an Adjuvant to Anesthesia in Patients with Arterial Hypertension. Acta Clin Croat. 2016;55:490-6.
4. Sousa AM, Rosado GMC, Neto J de S, Guimarães GMN, Ashmawi HA. Magnesium sulfate improves postoperative analgesia in laparoscopic gynecologic surgeries: a double-blind randomized controlled trial. J Clin Anesth. 2016;34:379-84.
5. Jarahzadeh MH, Harati ST, Babaeizadeh H, Yasaei E, Bashar FR. The effect of intravenous magnesium sulfate infusion on reduction of pain after abdominal hysterectomy under general anesthesia: a double-blind, randomized clinical trial. Electron Physician. 2016; 8:2602-6.

Intraoperative dexmedetomidine

INTRODUCTION

The analgesic effects of intravenous dexmedetomidine and other alpha-2-agonists such as clonidine are known for their action on the central and peripheral nervous system[1-4]. Recent systematic reviews on the use of intraoperative intravenous dexmedetomidine compared to remifentanil[1] show moderate evidence in the reduction of opioid needs both intraoperatively and during the first 24 postoperative hours, presenting less pain intensity and less need for rescue doses with opioids and with less frequency[1-3]. Likewise, there are fewer adverse effects derived from the lower use of perioperative opioids, presenting a lower incidence of hypotension, chills, nausea, and postoperative vomiting[1-4].

101. The use of intraoperative intravenous dexmedetomidine is recommended as it contributes to reducing the risk of adverse events associated with opiates improves pain control in the intra- and post-operative period.

Moderate level of evidence. Weak recommendation

REFERENCES

1. Grape S, Kirkham KR, Frauenknecht J, Albrecht E. Intra-operative analgesia with remifentanil vs. dexmedetomidine: a systematic review and meta-analysis with trial sequential analysis. Anaesthesia. 2019;74:793-800.
2. Wang X, Liu N, Chen J, Xu Z, Wang F, Ding C. Effect of Intravenous Dexmedetomidine During General Anesthesia on Acute Postoperative Pain in Adults: A Systematic Review and Meta-Analysis of Randomized Controlled Trials. Clin J Pain. 2018;34:1180-91.

3. Jin S, Liang DD, Chen C, Zhang M, Wang J. Dexmedetomidine prevent postoperative nausea and vomiting on patients during general anesthesia: A PRISMA-compliant meta analysis of randomized controlled trials. Medicine (Baltimore). 2017;96:e5770.
4. Le Bot A, Michelet D, Hilly J, Maesani M, Dilly MP, Brasher C, et al. Efficacy of intraoperative dexmedetomidine compared with placebo for surgery in adults: a meta-analysis of published studies. Minerva Anestesiol. 2015;81:1105-17.

Perioperative neuromodulators

INTRODUCTION

The oral administration of neuromodulators such as pregabalin or gabapentin could produce a significant decrease in the use of opioids in the first 24 hours without causing harmful effects in patients[1-3]. Furthermore, it could have a beneficial effect on the patients' chronic pain at 6 months after surgery. Patients over 65 years of age have greater side effects derived from the use of pregabalin and could be a better subsidiary of the use of gabapentin[4,5]. A recently published meta-analysis with more than 280 clinical trials and 24,000 patients found no relevant clinical analgesic effects although there were statistically significant differences in the first postoperative hours. Less nausea and vomiting and a greater number of visual disturbances and dizziness were found.

102. Open major abdominal surgery could assess a preoperative oral dose of gabapentin or pregabalin before the intervention for postoperative analgesic control.

Nivel de evidencia alto. Recomendación débil.

REFERENCES

1. Rai AS, Khan JS, Dhaliwal J, Busse JW, Choi S, Devereaux PJ, et al. Preoperative pregabalin or gabapentin for acute and chronic postoperative pain among patients undergoing breast cancer surgery: A systematic review and meta-analysis of randomized controlled trials. J Plast Reconstr Aesthet Surg. 2017;70:1317-28.
2. Liu B, Liu R, Wang L. A meta-analysis of the preoperative use of gabapentinoids for the treatment of acute postoperative pain following spinal surgery. Medicine (Baltimore). 2017;96:e8031.
3. Li S, Guo J, Li F, Yang Z, Wang S, Qin C. Pregabalin can decrease acute pain and morphine consumption in laparoscopic cholecystectomy patients: A meta-analysis of randomized controlled trials. Medicine (Baltimore). 2017;96:e6982.
4. Yao Z, Shen C, Zhong Y. Perioperative Pregabalin for Acute Pain After Gynecological Surgery: A Meta-analysis. Clin Ther. 2015;37:1128-35.
5. Eipe N, Penning J, Yazdi F, Mallick R, Turner L, Ahmadzai N, et al. Perioperative use of pregabalin for acute pain-a systematic review and meta-analysis. Pain. 2015;156:1284-300.
6. Verret M, Lauzier F, Zarychanski R, Perron C, Savard X, Pinard AM, et al. Perioperative Use of Gabapentinoids for the Management of Postoperative Acute Pain: A Systematic Review and Meta-analysis. Anesthesiology.2020:133: 265-279.

Prevention of paralytic ileus

INTRODUCTION

Paralytic ileus is one of the complications that produces the greatest discomfort in the patient, as well as prolonging their hospital stay[1].

103. Multimodal management by using alternatives to opioids (thoracic epidural catheter, blockages, minimally invasive surgery, avoiding the routine use of nasogastric tube and avoiding an excess of IV therapy fluid) is recommended to prevent the appearance of Postoperative paralytic ileus[1].

High level of evidence. Strong recommendation.

REFERENCES

1. Gustafsson UO, Scott MJ, Hubner M, Nygren J, Demartines N, Francis N, et al. Guidelines for Perioperative Care in Elective Colorectal Surgery: Enhanced Recovery After Surgery (ERAS) Society Recommendations: 2018. World J Surg 2019; 43(3):659-695.

Nausea and vomiting prophylaxis

INTRODUCTION

Postoperative nausea and vomiting (PONV) are the most important cause of delayed onset of oral fluid tolerance and can be more uncomfortable for the patient than pain. They affect 25-35% of all surgical patients and are a major cause of discomfort and delay in medical discharge. Prophylaxis should be proportional to the estimated risk.[1-3]

MEASURES FOR PROPHYLAXIS AND TREATMENT

Identification of the patient at risk of PONV

INTRODUCTION

The risk of PONV should be assessed in all patients using a validated risk scale, such as the simplified Apfel scale, which evaluates risk factors for PONV: female sex, history of PONV and / or motion sickness, non-smoker, administration postoperative morphology.[1-2] Patients under 50 years of age and those with a history of chemotherapy-induced nausea and vomiting are at increased risk of PONV. Regarding the type of surgery, an increased risk of PONV has been observed in cholecystectomies, gynecological surgery, and laparoscopic procedures.[3]

104. The risk of PONV must be stratified in all patients using the Apfel scale and carry out prophylaxis proportional to the expected risk. Prophylaxis with more combined drugs can be performed in surgeries in which PONV pose a significant risk of complications.

High level of evidence. Strong recommendation.

REFERENCES

1. Apfel CC, Läärä E, Koivuranta M, Greim CA, Roewer N. A simplified risk score for predicting postoperative nausea and vomiting: conclusions from cross-validations between two centers. Anesthesiology 1999;91(3):693-700.

2. Apfel CC, Philip BK, Cakmakkaya OS, Shilling A, Shi Y-Y, Leslie JB, et al. Who is at risk for postdischarge nausea and vomiting after ambulatory surgery? Anesthesiology 2012;117(3): 475-86.

3. Apfel CC, Heidrich FM, Jukar-Rao S, Jalota L, Hornuss C, Whelan RP, et al. Evidence-based analysis of risk factors for postoperative nausea and vomiting. Br J Anaesth 2012;109(5):742-53.

DECREASE IN BASAL RISK OF PONV:

Regional anesthesia vs general anesthesia

INTRODUCTION

The decrease in baseline risk factors for PONV lowers their incidence[1]. The strategies to minimize them in patients at risk include the choice of regional anesthesia over general anesthesia, the use of propofol in induction and in maintenance, avoid the use of nitrous oxide and volatile anesthetics, minimize the use of intra- and postoperative opioids and adequate hydration.[2]

105. Regional anesthesia is recommended before general anesthesia to reduce the incidence of PONV

High level of evidence. Strong recommendation.

REFERENCES

1. Sinclair DR, Chung F, Mezei G. Can postoperative nausea and vomiting be predicted? Anesthesiology 1999;91:109-18.
2. Veiga-Gil L, Pueyo J, López-Olaondo L. Náuseas y vómitos postoperatorios: fisiopatología, factores de riesgo, profilaxis y tratamiento. Rev Esp Anestesiol Reanim 2017;64(4):223-32.

Intravenous anesthesia vs inhaled anesthesia

INTRODUCTION

In patients with a higher risk or history of PONV, it has been shown that TIVA intravenous general anesthesia with propofol reduces the incidence of nausea and vomiting compared to maintenance with inhaled halogenated anesthetics.[1-3]

106. The use of propofol is recommended for induction and maintenance of anesthesia in patients at high risk of PONV.

High level of evidence. Strong recommendation.

REFERENCES.

1. Tramèr M, Moore A, McQuay H. Propofol anaesthesia and postoperative nausea and vomiting: quantitative systematic review of randomized controlled studies. BJA: Br J Anaesth 1997;78(3):247-55.
2. Apfel CC, Korttila K, Abdalla M, Kerger H, Turan A, Vedder I, et al. A factorial trial of six interventions for the prevention of postoperative nausea and vomiting. N Engl J Med 2004;350(24):2441-51.
3. Schraag S, Pradelli L, Alsaleh AJO, Bellone M, Ghetti G, Chung TL, et al. Propofol vs. inhalational agents to maintain general anaesthesia in ambulatory and in-patient surgery: a systematic review and meta-analysis. BMC Anesthesiol. 2018;18(1):162.

Avoid using nitrous oxide

INTRODUCTION.

In surgeries lasting more than one hour and in patients at risk of PONV, the incidence of nausea and vomiting is increased if balanced anesthesia is used with inhaled halogenated anesthetics combined with nitrous oxide.[1-6]

107. The use of nitrous oxide should be avoided in patients at high risk for PONV or long-lasting surgeries.

High level of evidence. Strong recommendation.

REFERENCES

1. Tramèr M, Moore A, McQuay H. Omitting nitrous oxide in general anaesthesia: meta-analysis of intraoperative awareness and postoperative emesis in randomized controlled trials. Br J Anaesth 1996;76(2):186-93.
2. Fernández-Guisasola J, Gómez-Arnau JI, Cabrera Y, del Valle SG. Association between nitrous oxide and the incidence of postoperative nausea and vomiting in adults: a systematic review and meta-analysis. Anaesthesia 2010;65(4):379-87.
3. Peyton PJ, Wu CY. Nitrous oxide-related postoperative nausea and vomiting depends on duration of exposure. Anesthesiology 2014;120(5):1137-45.
4. Sun R, Jia WQ, Zhang P, Yang K, Tian JH, Ma B, et al. Nitrous oxide-based techniques versus nitrous oxide-free techniques for general anaesthesia. Cochrane Database Syst Rev 2015 Nov 6;(11):CD008984.
5. Buhre W, Disma N, Hendrickx J, DeHert S, Hollmann MW, Huhn R, et al. European Society of Anaesthesiology Task Force on Nitrous Oxide: a narrative review of its role in clinical practice. Br J Anaesth 2019;122(5):587-604.
6. Myles PS, Chan MTV, Kasza J, Paech MJ, Leslie K, Peyton PJ, et al. Severe Nausea and Vomiting in the Evaluation of Nitrous Oxide in the Gas Mixture for Anesthesia II Trial. *Anesthesiology* 2016;124(5):1032-1040.

Avoid the use of halogenated agents

INTRODUCTION

The use of halogenated inhalation agents increases the incidence of PONV in patients with a higher risk on the Apfel scales or a history of PONV in previous surgical interventions with general anesthesia.[1-3]

108. The use of inhalation anesthetics should be avoided in patients at high risk of PONV.

Moderate level of evidence. Strong recommendation.

REFERENCES

1. Sneyd JR, Carr A, Byrom WD, Bilski AJ. A meta-analysis of nausea and vomiting following maintenance of anaesthesia with propofol or inhalational agents. Eur J Anaesthesiol 1998;15(4):433-45.
2. Apfel CC, Kranke P, Katz MH, Goepfert C, Papenfuss T, Rauch S, et al. Volatile anaesthetics may be the main cause of early but not delayed postoperative vomiting: a randomized controlled trial of factorial design. British Journal of Anaesthesia 2002;88(5):659-68.
3. Apfel CC, Korttila K, Abdalla M, Kerger H, Turan A, Vedder I, et al. A factorial trial of six interventions for the prevention of postoperative nausea and vomiting. N Engl J Med 2004;350(24):2441-51.

Reduce the use of opioids

INTRODUCTION

One of the adverse effects of opioids is the increase in PONV, especially in patients at higher risk and in long-term surgeries that require greater analgesia, therefore, multimodal analgesia with different families of drugs, the use of regional anesthetic techniques and local anesthesia are recommended to reduce the total dose of intra- and postoperative opioids.[1,2]

109. It is advisable to minimize the use of intraoperative opioids, especially postoperative ones.

High level of evidence. Strong recommendation.

REFERENCES.

1. Roberts GW, Bekker TB, Carlsen HH, Moffatt CH, Slattery PJ, McClure AF. Postoperative nausea and vomiting are strongly influenced by postoperative opioid use in a dose-related manner. Anesth Analg 2005;101(5):1343-8.
2. Guay J, Nishimori M, Kopp S. Epidural local anaesthetics versus opioid-based analgesic regimens for postoperative gastrointestinal paralysis, vomiting and pain after abdominal surgery. Cochrane Database of Syst Rev 2016 Jul 16;7:CD001893.

TREATMENT AND ANTIEMETIC PROPHYLAXIS ACCORDING TO APFEL RISK SCALE:

Low Apfel risk 0-1

INTRODUCTION

Prophylaxis is not indicated in all Apfel 0-1 patients, except in surgery with a high risk of complication if PONV and in surgery with a higher emetic risk (cholecystectomies, gynecological or laparoscopic procedures, gastric, esophageal surgery, neurosurgery, etc.) in which pharmacological prophylaxis with monotherapy is recommended.[1-3] Dexamethasone (4 mg iv at induction of anesthesia), droperidol (0.625-1.25 mg iv at the end of surgery) and ondansetron (4 mg iv at end of surgery) have similar efficacy.[4-6] The use of dexamethasone or droperidol has the advantage of reserving ondansetron as a treatment in case of prophylaxis failure.[6]

110. Monotherapy antiemetic prophylaxis should be performed in patients with Apfel 0-1 but surgery with a higher risk of PONV.

Moderate level of evidence. Strong recommendation.

REFERENCES

1. Wang JJ, Ho ST, Tzeng JI, Tang CS. The effect of timing of dexamethasone administration on its efficacy as a prophylactic antiemetic for postoperative nausea and vomiting. Anesth Analg 2000;91(1):136-9.
2. Henzi I, Sonderegger J, Tramèr MR. Efficacy, dose-response, and adverse effects of droperidol for prevention of postoperative nausea and vomiting. Can J Anesth 2000;47(6):537-51.
3. Apfel CC, Korttila K, Abdalla M, Kerger H, Turan A, Vedder I, et al. A factorial trial of six interventions for the prevention of postoperative nausea and vomiting. N Engl J Med 2004; 350(24):2441-51.

4. Gómez-Arnau JI, Aguilar JL, Bovaira P, Bustos F, De Andrés J, la Pinta de JC, et al. Recomendaciones de prevención y tratamiento de las náuseas y vómitos postoperatorios y/o asociados a las infusiones de opioides. Rev Esp Anestesiol Reanim 2010;57(8):508-24.
5. Gan TJ, Diemunsch P, Habib AS, Kovac A, Kranke P, Meyer TA, et al. Consensus Guidelines for the Management of Postoperative Nausea and Vomiting. Anesth Analg 2014;118(1):85-113.
6. Veiga-Gil L, Pueyo J, López-Olaondo L. Náuseas y vómitos postoperatorios: fisiopatología, factores de riesgo, profilaxis y tratamiento. Rev Esp Anestesiol Reanim 2017;64(4):223-32.

Moderate risk (Apfel 2-3)

INTRODUCTION

Measures to reduce baseline risks are indicated, as well as pharmacological prophylaxis with monotherapy. Pharmacological prophylaxis with dual therapy (dexamethasone and droperidol or ondansetron) in surgery with a high risk of complication if PONV and in surgery with a higher emetic risk. The combination of dexamethasone and droperidol has the advantage of reserving ondansetron for treatment in case of prophylaxis failure.[1-3]

111. Antiemetic prophylaxis should be performed as monotherapy in patients with an Apfel 2-3 assessment and double therapy if surgery with a higher risk of PONV.

High level of evidence. Strong recommendation.

REFERENCES

1. Gan, TJ, Belani KG, Bergese S; Chung FM, Diemunsch P, Habib AS, et al. Fourth Consensus Guidelines for the Management of Postoperative Nausea and Vomiting. Anesth Analg 2020;131:411-448.
2. Gómez-Arnau JI, Aguilar JL, Bovaira P, Bustos F, De Andrés J, la Pinta de JC, et al. Recomendaciones de prevención y tratamiento de las náuseas y vómitos postoperatorios y/o asociados a las infusiones de opioides. Rev Esp Anestesiol Reanim 2010;57(8):508-24.
3. Veiga-Gil L, Pueyo J, López-Olaondo L. Náuseas y vómitos postoperatorios: fisiopatología, factores de riesgo, profilaxis y tratamiento. Rev Esp Anestesiol Reanim 2017;64(4):223-32.

High risk (Apfel 4)

INTRODUCTION

Measures to reduce baseline risks and drug prophylaxis with dual therapy are indicated. Pharmacological prophylaxis with triple therapy (dexamethasone, droperidol and ondansetron, administering it at the end of surgery) in surgery with a high risk of complication if PONV and in surgery with a higher emetic risk.[1,2]

112. It is recommended to perform antiemetic prophylaxis in double combinate therapy in patients with Apfel 4 assessment and triple therapy if surgery with a higher risk of PONV.

High level of evidence. Strong recommendation.

REFERENCES

1. Gan, TJ, Belani KG, Bergese S; Chung FM, Diemunsch P, Habib AS, et al. Fourth Consensus Guidelines for the Management of Postoperative Nausea and Vomiting. Anesth Analg 2020;131:411-448.

2. Veiga-Gil L, Pueyo J, López-Olaondo L. Náuseas y vómitos postoperatorios: fisiopatología, factores de riesgo, profilaxis y tratamiento. Rev Esp Anestesiol Reanim 2017;64(4):223-32.

113. The use of peripheral opioid receptor antagonists prevents the appearance of ileus in the postoperative period.

Moderate level of evidence. Weak recommendation.

REFERENCES

1. Schwenk ES, Grant AE, Torjman MC, SE McNulty, JL Baratta, MD* and ER Viscusi. The efficacy of peripheral opioid antagonists in opioid-induced constipation and postoperative ileus: a systematic review of the literature. Reg Anesth Pain Med. 2017;42:767-777.

7.1.4 POSTOPERATIVE

Postoperative warming measures

INTRODUCTION

In case of postoperative hypothermia, active skin warming systems should be used versus passive systems[1]. These measures must be applied as far in advance as possible[2]. Within the active skin heating systems, the most evaluated strategies are convective and conductive hot air, being cost-effective strategies even in those patients with lower surgical risk and short duration of surgery[3].

114. Postoperative hypothermia should be treated by administering convective or conductive heat until normothermia is achieved.

High level of evidence. Strong recommendation.

REFERENCES

1. Torossian A, Bräuer A, Höcker J, Bein B, Wulf H, Horn EP. Preventing inadvertent perioperative hypothermia. Clinical Practice Guideline. Dtsch Arztebl Int. 2015 Mar 6; 112(10): 166-72. doi: 10.3238/arztebl.2015.0166. PMID: 25837741.
2. Warttig S, Alderson P, Campbell G, Smith AF. Interventions for treating inadvertent postoperative hypothermia. Cochrane Database Syst Rev. 2014 Nov 20;(11):CD009892. doi: 10.1002/14651858.CD009892.pub2. Review. PubMed PMID: 25411963.3.
3. Calvo Vecino JM, Casans Francés R, Ripollés Melchor J, Marín Zaldívar C, Gómez Ríos MA, Pérez Ferrer A et al. Clinical practice guideline. Unintentional perioperative hypothermia. Rev Esp Anestesiol Reanim. 2018 Dec;65(10):564-588. doi: 10.1016/j.redar.2018.07.006. Epub 2018 Nov 15. English, Spanish. PubMed PMID: 30447894.

Perioperative NSAIDs

INTRODUCTION

The use of NSAIDs for pain control as adjunctive therapy is associated with a decrease in opioid consumption and an improvement in patient comfort. In addition, the use of NSAIDs could be on an equal footing in terms of analgesic potency with the infiltration of laparoscopic instrument ports with local anesthetics, and selective cyclooxygenase-2 inhibitors could have some influence on improving postoperative bowel function.[1]

115. Non-steroidal anti-inflammatory drugs (NSAIDs) should be used as adjunctive therapy for pain control in patients who have undergone major abdominal surgery.

High level of evidence. Strong recommendation.

REFERENCES

1. Martinez V, Beloeil H, Marret E, Fletcher D, Ravaud P, Trinquart L. Non-opioid analgesics in adults after major surgery: systematic review with network meta-analysis of randomized trials. British Journal of Anaesthesia. 2017;118:22-31.

116. The use of gum is not recommended routinely

Low level of evidence. Weak recommendation.

REFERENCES

1. Gregg Nelson, Jamie Bakkum-Gamez Eleftheria Kalogera, Gretchen Glaser Alon Altman, Larissa A Meyer, Jolyn S Taylor, et al. Guidelines for perioperative care in gynecologic/oncology: Enhanced Recovery After Surgery (ERAS) Society recommendation 2019 update. Int J Gynecol Cancer. 2019;0:1-18.
2. de Leede EM, van Leersum NJ, Kroon HM, van Weel V, van der Sijp JRM Bonsing BA; Kauwgomstudie Consortium. Multicentre randomized clinical trial of the effect of chewing gum after abdominal surgery. Br J Surg. 2018;105:820-828.
3. Gustafsson O, Scott MJ, Hubner J, Nygren J, Demartines N, Francis N, et al. Guidelines for Perioperative Care in Elective Colorectal Surgery: Enhanced Recovery After Surgery (ERAS) Society Recommendations: 2018. World J Surg. 2019, 43:659-695.
4. Short V, Herbert G, Perry R, Atkinson C, Ness AR, Penfold C, et al. Chewing gum for postoperative recovery of gastrointestinal function. Cochrane Database Syst Rev.2015;CD006506. pub3.

Treatment of PONV in patients with failed prophylaxis

INTRODUCTION

Treatment of established PONV: if prophylaxis has not been administered, low-dose ondansetron (1 mg iv) will be used as an option. If prophylaxis has been performed and more than 6 hours have elapsed since its administration, a rescue antiemetic from a different family should be used from that used for prophylaxis (ondansetron 1 mg iv or droperidol 0.625-1.25 mg iv) except for dexamethasone, whose repetition is not recommended.[1,2]

117. In established nausea and vomiting, selective 5-HT3 antagonists (ondansetron) are the treatment of choice, followed by a different antiemetic drug family if unresponsive except for dexamethasone.

High level of evidence. Strong recommendation.

REFERENCES.

1. Gan, TJ, Belani KG, Bergese S; Chung FM, Diemunsch P, Habib AS, et al. Fourth Consensus Guidelines for the Management of Postoperative Nausea and Vomiting. Anesth Analg. 2020;131:411-448.
2. Veiga-Gil L, Pueyo J, López-Olaondo L. Náuseas y vómitos postoperatorios: fisiopatología, factores de riesgo, profilaxis y tratamiento. Rev Esp Anestesiol Reanim 2017;64(4):223-32.

118. Use of laxatives such as bisacodyl (in colorectal surgery), oral magnesium oxide (in hysterectomy), daikenchuto (Japanese herbal infusion, in gastrectomy), coffee (in colorectal surgery) could prevent the appearance of ileus.

Nivel de evidencia baja. Recomendación débil.

REFERENCES

1. Zingg U, Miskovic D, Pasternak I et al (2008) Effect of bisacodyl on postoperative bowel motility in elective colorectal surgery: a prospective, randomized trial. Int J Colorectal Dis 23:1175-1183.

2. Hansen CT, Sorensen M, Moller C et al (2007) Effect of laxatives on gastrointestinal functional recovery in fast-track hys- terectomy: a double-blind, placebo-controlled randomized study. Am J Obstet Gynecol 196(311):311-317.

3. Yoshikawa K, Shimada M, Wakabayashi G et al (2015) Effect of daikenchuto, a traditional japanese herbal medicine, after total gastrectomy for gastric cancer: a multicenter, randomized, double-blind, placebo-controlled, phase II trial. J Am Coll Surg 221:571-578.

4. Muller SA, Rahbari NN, Schneider F et al (2012) Randomized clinical trial on the effect of coffee on postoperative ileus fol-lowing elective colectomy. Br J Surg 99:1530-1538.

5. Dulskas A, Klimovskij M, Vitkauskiene M et al (2015) Effect of coffee on the length of postoperative ileus after elective laparoscopic left-sided colectomy: a randomized, prospective single-center study. Dis Colon Rectum 58:1064-1069.

Inmunonutrition (postoperative)

INTRODUCTION

Inmunonutrition (IN) has been a debated topic since the 1990s, especially in the context of cancer surgery[1]. Some reviews and meta-analysis have shown the beneficial effects of NI by summing the results of RCTs in all types of patients and examining the entire perioperative period. However, other studies have found no added benefit with the use of IN over standard supplements using similar methods[2].

According to the 2017 ESPEN clinical guidelines on clinical nutrition and surgery, specific formulas with immunonutrients should be administered in the peri- or at least post-operative to malnourished patients undergoing major surgery for cancer, with a moderate grade of recommendation (SIGN, Scottish Intercollegiate Guidelines Network)[3]. There is no clear evidence for its use compared to standard oral supplements exclusively in the preoperative period.

Meta-analyzes continue to appear in this sense, with some common positive results, with evidence that is not always high[4,5].

119. Inmunonutrition seems recommendable in malnourished patients undergoing gastrointestinal surgery for cancer, due to the decrease in infectious complications and a possible shortening of hospitalization.

Low level of evidence. Strong recommendation.

REFERENCES

1. Arends J, Bachmann P, Baracos V, et al. ESPEN guidelines on nutrition in cancer patients. Clin Nutr 2017; 36:11-48.

2. Hegazi RA, Hustead DS, Evans DC. Properative standard oral nutrition supplements vs immunonutrition: results of a systematic review and meta-analysis. J Am Coll Surg 2014; 219: 1078-1087.

3. Weimann A, Braga M, Carli F, Higashiguchi T, Hübner M, Klek S, et al. ESPEN guideline: Clinical nutrition in surgery. Clin Nutr 2017; 36: 623-650.

4. Probst P, Ohmann S, Klaiber U, Hüttner FJ, Billeter AT, Ulrich A, Büchler MW, Diener MK. Meta-analysis of immunonutrition in major abdominal surgery. Br J Surg 2017; 104: 1594-1608.

5. Adiamah A, Skorepa P, Weimann A, Lobo DN. The impact of preoperative immune modulating nutrition on outcomes in patients undergoing surgery for gastrointestinal surgery for gastrointestinal cancer. Ann Surg 2019; 270: 247-256.

Postoperative pain

INTRODUCTION

Postoperative pain control allows rapid recovery and multimodal rehabilitation. Analgesia is important in the first 24-48 hours to allow early mobilization, reducing paralytic ileus and hospital stay. For this reason, it is important to reduce the concentrations of local anesthetics through the thoracic epidural route in the postoperative period of open abdominal surgery, to obtain a sensitive block of the involved metameres and with the least possible motor block in the lower limbs.[1, 2] After the first 48 hours, the epidural catheter should be removed to reduce the risk of infection and ensure wandering without motor block. To achieve this, the use of alternative NSAIDs and paracetamol is important, to minimize the use of intravenous opioids, leaving their use for rescues of intense uncontrolled pain.[3] The postoperative use of gabapentin, NMDA blockers such as ketamine, as well as high doses of opioids is not recommended. In some cases, in which epidural analgesic techniques are not available in open surgery, the use of lidocaine in continuous infusion in the first 24 hours or the use of interfacial blocks such as TAP or quadratus lumbar can be considered.[4-7]

120. The use of epidural analgesia is recommended during the first 24-48 h after surgery and its withdrawal after this initial period of pain control, reducing the concentrations of local anesthetics with epidural opioids to reduce motor block and allow wandering.

High level of evidence. Strong recommendation.

121. The use of paracetamol and NSAIDs is recommended for postoperative pain control with opioid rescues in severe uncontrolled pain with epidural analgesia or other local or regional analgesia techniques.

High level of evidence. Strong recommendation.

REFERENCES

1. Salicath JH, Yeoh ECY, Bennett MH. Epidural analgesia versus patient-controlled intravenous analgesia for pain following intra-abdominal surgery in adults. Cochrane Database of Systematic Reviews. 2018;8: CD010434.

2. Guay J, Nishimori M, Kopp S. Epidural local anaesthetics versus opioid-based analgesic regimens for postoperative gastrointestinal paralysis, vomiting and pain after abdominal surgery. Cochrane Database of Systematic Reviews 2016;7:CD001893.

3. Martinez V, Beloeil H, Marret E, Fletcher D, Ravaud P, Trinquart L. Non-opioid analgesics in adults after major surgery: systematic review with network meta-analysis of randomized trials. British Journal of Anaesthesia. 2017;118:22-31.

4. MacFater WS, Rahiri J-L, Lauti M, Su'a B, Hill AG. Intravenous lignocaine in colorectal surgery: a systematic review. ANZ J Surg. 2017;87:879-85.

5. Weibel S, Jokinen J, Pace NL, Schnabel A, Hollmann MW, Hahnenkamp K, et al. Efficacy and safety of intravenous lidocaine for postoperative analgesia and recovery after surgery: a systematic review with trial sequential analysis. Br J Anaesth. 2016;116:770-83.

6. Weibel S, Jelting Y, Pace NL, Helf A, Eberhart LH, Hahnenkamp K, et al. Continuous intravenous perioperative lidocaine infusion for postoperative pain and recovery in adults. Cochrane Database Syst Rev. 2018;6:CD009642.

7. Baeriswyl M, Zeiter F, Piubellini D, Kirkham KR, Albrecht E. The analgesic efficacy of transverse abdominis plane block versus epidural analgesia: a systematic review with meta-analysis. Medicine. 2018;97(26).

Early postoperative feeding

INTRODUCTION

Traditionally, not feeding patients postoperatively until they have a bowel movement or gas transit has been common practice. Early oral feeding does not alter the healing of the sutures in the colon or rectum, and shortens the hospital stay, as reported in a Cochrane systematic review. More recent meta-analyses show apparent benefits in relation to postoperative recovery and the incidence of infections. A meta-analysis of 15 studies (8 of them RCTs) with 2,112 patients undergoing upper gastrointestinal surgery showed a significantly shorter hospital stay with no differences in complications[1].

The amount of the initial oral intake must be adapted to the state of gastrointestinal function and individual tolerance[2].

Overall, we can say that there is good evidence of the benefits and tolerance of early feeding in the postoperative period of colorectal surgery. The benefits are less clear in older patients with upper gastrointestinal and pancreatic surgery. There are no controlled data in patients with esophageal resection.

122. Early postoperative feeding should be started as soon as possible, within hours after surgery in most patients.

Moderate level of evidence (in colorectal surgery). Strong recommendation.

REFERENCES

1. Willcutts KF, Chung MC, Erenberg CL, Finn KL, Schirmer BD, Byham-Gray LD. Early oral feeding as compared with traditional timing of oral feeding after upper gastrointestinal surgery. Ann Surg 2016;264:54e63.

2. Weimann A, Braga M, Carli F, Higashiguchi T, Hübner M, Klek S, et al. ESPEN guideline: Clinical nutrition in surgery. Clin Nutr 2017; 36: 623-650.

Early mobilization

INTRODUCTION

Bed rest decreases muscle strength increases insulin resistance and the risk of pulmonary and thromboembolic complications. The evidence is limited regarding the benefit of early mobilization interventions after surgery.[1,2] Although mobilization is associated with shorter hospital stays, few studies investigate the impact of specific strategies

to increase mobilization compared to allowing early wandering[3]. There is great variability in the different protocols to implement early mobilization from some mobilization at 24 hours to 8 hours per day postoperatively[4]. Failure of early mobilization may be due to factors such as inadequate pain control, intravenous fluid intake, use of tubes and drains, patient motivation, and preexisting comorbidities[5]. Early mobilization should be encouraged, but the allocation of additional resources to implement it beyond integration into multimodal enhanced recovery protocols has not shown benefits[6].

123. Early mobilization through education and patient encouragement is recommended to reduce the number of adverse effects.

Moderate level of evidence. Strong recommendation.

REFERENCES

1. Castelino T, Fiore JF Jr, Niculiseanu P et al The effect of early mobilization protocols on postoperative outcomes following abdominal and thoracic surgery: a systematic review. Surgery. 2016; 159:991-1003.
2. de Almeida EPM, de Almeida JP, Landoni G, et al Early mobilization programme improves functional capacity after major abdominal cancer surgery: a randomized controlled trial [with consumer summary] British Journal of Anaesthesia 2017;119(5):900-907.
3. Schaller SJ, Anstey M, Blobner M et al Early, goal-directed mobilisation in the surgical intensive care unit: a randomized controlled trial. Lancet. 2016; 388:1377-1388.
4. Fiore JF Jr, Castelino T, Pecorelli N et al. Ensuring early mobilization within an enhanced recovery program for colorectal surgery: a randomized controlled trial. Ann Surg 2017; 266:223-231.
5. Gustafsson UO, Scott MJ, Hubner M, et al. Guidelines for Perioperative Care in Elective Colorectal Surgery: Enhanced Recovery After Surgery (ERAS_) Society Recommendations: 2018 World J Surg (2019) 43: 659-695. https://doi.org/10.1007/s00268-018-4844-y.
6. Wolk, S., Linke, S., Bogner, A. y col. Use of Activity Tracking in Major Visceral Surgery-the Enhanced Perioperative Mobilization Trial: a Randomized Controlled Trial J Gastrointest Surg 2019; 23: 1218. https://doi.org/10.1007/s11605-018-3998-0.

Respiratory physiotherapy

INTRODUCTION

Performing respiratory exercises in the preoperative period leads to a reduction in respiratory complications in the postoperative period.[1] Incentive spirometry and training of the respiratory muscles are the most studied techniques. Incentive spirometry in abdominal surgery, although it seems to have a positive impact on lung function and diaphragm excursion during the immediate postoperative period, has not shown benefit in the prevention of postoperative complications.[2] Selective training of the inspiratory muscles has been shown to reduce the risk of postoperative pulmonary complications and hospital stay.[3,4] Pre-surgery educational sessions and training courses given by a physiotherapist, aimed at instructing the patient in the performance of respiratory physiotherapy techniques and in making them aware of the importance of their link with the postoperative period, have been shown to impact post-operative morbidity in abdominal surgery with a decrease of post-operative pulmonary complications[5].

124. Preoperative and postoperative respiratory physiotherapy is recommended.

High level of evidence. Strong recommendation.

REFERENCES

1. Katsura M, Kuriyama A, Takeshima T, Fukuhara S, Furukawa TA. Preoperative inspiratory muscle training for postoperative pulmonary complications in adults undergoing cardiac and major abdominal surgery. Cochrane Database Syst Rev. 2015; 5;(10):CD010356. doi: 10.1002/14651858.CD010356.pub2.
2. Kalil-Filho FA, Campos ACL, Tambara EM, Tomé BKA, Treml CJ, Kuretzki CH, et al. Physiotherapeutic approaches and the effects on inspiratory muscle force in patients with chronic obstructive pulmonary disease in the pre-operative preparation for abdominal sur- gical procedures. Arq Bras Cir Dig. 2019;32(2): e1439.
3. Kendall F, Oliveira J, Peleteiro B, Pinho P, Bastos PT. Inspiratory muscle training is effective to reduce postoperative pulmonary complications and length of hospital stay: a systematic review and meta-analysis. Disabil Rehabil. 2018; 40(8):864-82.
4. Alaparthi GK, Augustine AJ, Anand R, Mahale A. Comparison of Diaphragmatic Breathing Exercise, Volume and Flow Incentive Spirometry, on Diaphragm Excursion and Pulmonary Function in Patients Undergoing Laparoscopic Surgery: A Randomized Controlled Trial. Minim Invasive Surg 2016;1967532.
5. Karlsson E, Farahnak P, Franzén E, Nygren-Bonnier M, Dronkers J, van Meeteren N, et al. Feasibility of preoperative supervised home-based exercise in older adults undergoing colorectal cancer surgery-A randomized controlled design. PLoS One. 2019;14(7): e0219158.

Management of postoperative anemia

INTRODUCTION

Despite being a common practice, evidence shows that treatment with oral iron in anemic, non-iron deficient patients prior to surgery is not more effective, nor is it better tolerated, than placebo for the treatment of post-operative anemia[1-4].

125. Oral administration of iron salts is not recommended in the immediate postoperative period to improve the hemoglobin level and decrease the transfusion rate.

Moderate level of evidence. Strong recommendation.

126 Instead, postoperative treatment with FEEV is suggested to improve hemoglobin levels and reduce the transfusion rate, especially in patients with low iron storage and / or moderate-severe post-bleeding anemia.

Moderate level of evidence. Strong recommendation.

REFERENCES

1. Muñoz M, Acheson AG, Bisbe E, Butcher A, Gómez-Ramírez S, Khalafallah AA, Kehlet H, et al. An international consensus statement on the management of postoperative anaemia after major surgical procedures. Anaesthesia. 2018;73:1418-1431.
2. Leal-Noval SR, Muñoz M, Asuero M, Contreras E, García-Erce JA, Llau JV, et al. Spanish Expert Panel on Alternatives to Allogeneic Blood Transfusion. Spanish Consensus Statement on alternatives to allogeneic blood transfusion: the 2013 update of the “Seville Document”. Blood Transfus. 2013;11:585-610.
3. Gómez-Ramírez S, Maldonado-Ruiz MÁ, Campos-Garrigues A, Herrera A, Muñoz M. Short-term perioperative iron in major orthopedic surgery: state of the art. Vox Sang. 2019 Jan;114(1):3-16.

4. Laso-Morales MJ, Vives R, Gómez-Ramírez S, Pallisera-Lloveras A, Pontes C. Intravenous iron administration for post-operative anaemia management after colorectal cancer surgery in clinical practice: a single-centre, retrospective study. Blood Transfus. 2018;16:338-342.

TRANSFUSION

Application of "restrictive" transfusion criteria

INTRODUCTION

There is a general consensus (both nationally and internationally) in recommending the application of “restrictive” transfusion criteria versus “liberal” criteria in the majority of hemodynamically stable patients: surgical (undergoing orthopedic and cardiovascular surgery) [1,2,3,4,5,6,7,8,9,10,11,12,13], critical (trauma, septic and pediatric) [1,2,4,5,6,7,8], postpartum[14] and even in patients with gastrointestinal bleeding[15] (after upper gastrointestinal bleeding, stable and with low risk of recurrence).

These “restrictive” criteria consist on the unitary (“one at a time”) administration of concentrated red blood cells, with reassessment after each transfused unit, in case of symptoms or signs of hypoxia or anemia, or to maintain the hemoglobin concentration above 7 g / dL in critically ill patients[1,2,3,4,5,6,7,8]; above 7.5 g / dL in cardiovascular surgery patients[9,10,11]; or above 8 g / dL in the case of cardiovascular risk factors[2,3,5,6,7,12,13].

The SEHH recommends “Not to transfuse a greater number of packed red blood cells than the needed to relieve symptoms of anemia or to return a patient to a safe hemoglobin range (7 to 8 g / dl in stable non-cardiac patients).”[3]

The SEMICYUC recommends that "Red blood cell concentrates should not be transfused in hemodynamically stable, non-bleeding critical patients, without cardiological and / or Central Nervous System involvement with a hemoglobin concentration greater than 7 g / dl." [4]

127. The application of “restrictive” criteria for the transfusion of packed red blood cells (HC) is recommended (if symptoms or Hb level <70 g / L), in most hospitalized patients (medical, surgical, or critical), without active bleeding and hemodynamically stable (including septic, upper gastrointestinal bleeding and postpartum anemia).

High level of evidence. Strong recommendation.

128. The application of “restrictive” criteria for CH transfusion (Hb ≤75 g / L) is recommended in cardiac surgery patients.

Moderate level of evidence. Strong recommendation.

129. The application of restrictive criteria for CH transfusion (Hb <80 g / L) is recommended in patients with a history of cardiovascular disease who underwent orthopedic surgery or hip fracture repair surgery.

Moderate level of evidence. Strong recommendation.

REFERENCES

1. Blood transfusion. Quality standard [QS138] Published date: December 2016 https://www.nice.org.uk/guidance/qs138 (último acceso febrero 2020).
2. Muñoz Gómez M, Bisbe Vives E, Basora Macaya M, García Erce JA, Gómez Luque S, Leal-Noval SR, et al. Foro de debate: seguridad de las alternativas a la transfusión alogénica en el paciente quirúrgico y/o crítico. Med Intensiva. 2015;39:552-562.
3. Recomendaciones de «no hacer» de la Sociedad Española de Hematología y Hemoterapia. Proyecto COMPROMISO POR LA CALIDAD DE LAS SOCIEDADES CIENTÍFICAS EN ESPAÑA. http://www.mscbs.gob.es/organizacion/sns/planCalidadSNS/pdf/SOCIEDAD_ESP_HEMATOLOGIA_HEMOTERAPIA_0K.pdf (último acceso febrero 2020).
4. Recomendaciones de «no hacer» de la Sociedad Española de Medicina Intensiva, Crítica y Unidades Coronarias (SEMICYUC). Proyecto COMPROMISO POR LA CALIDAD DE LAS SOCIEDADES CIENTÍFICAS EN ESPAÑA. http://www.mscbs.gob.es/organizacion/sns/planCalidadSNS/pdf/SOCIEDAD_ESP_MIC_UNIDCORONARIAS_0K.pdf (último acceso febrero 2020)
5. Carson JL, Guyatt G, Heddle NM, Grossman BJ, Cohn CS, Fung MK, et al. Clinical Practice Guidelines From the AABB: Red Blood Cell Transfusion Thresholds and Storage. JAMA. 2016;316:2025-2035.
6. Mueller MM, Van Remoortel H, Meybohm P, Aranko K, Aubron C, Burger R, et al. ICC PBM Frankfurt 2018 Group. Patient Blood Management: Recommendations from the 2018 Frankfurt Consensus Conference. JAMA. 2019;321:983-97. doi:10.1001/jama.2019.0554.
7. Ripollés Melchor J, Casans Francés R, Espinosa Á, Martínez Hurtado E, Navarro Pérez R, Abad Gurumeta A, et al; EAR Group Anesthesia Evidence Review. Restrictive versus liberal transfusion strategy for red blood cell transfusion in critically ill patients and in patients with acute coronary syndrome: a systematic review, meta-analysis and trial sequential analysis. Minerva Anestesiol. 2016;82:582-98.
8. Gustafsson UO, Scott MJ, Hubner M, et al. Guidelines for Perioperative Care in Elective Colorectal Surgery: Enhanced Recovery After Surgery (ERAS®) Society Recommendations: 2018. World J Surg. 2019;43:659-95.
9. Mazer CD, Whitlock RP, Fergusson DA, Hall J, Belley-Cote E, Connolly K, et al. TRICS Investigators and Perioperative Anesthesia Clinical Trials Group. Restrictive or Liberal Red-Cell Transfusion for Cardiac Surgery. N Engl J Med. 2017;377:2133-2144.
10. Mazer CD, Whitlock RP, Fergusson DA, Belley-Cote E, Connolly K, Khanykin B, Gregory AJ, et al; TRICS Investigators and Perioperative Anesthesia Clinical Trials Group. Six-Month Outcomes after Restrictive or Liberal Transfusion for Cardiac Surgery. N Engl J Med. 2018; 379:1224-1233.
11. Shehata N, Mistry N, da Costa BR, Pereira TV, Whitlock R, Curley GF, et al (Mazer CD). Restrictive compared with liberal red cell transfusion strategies in cardiac surgery: a meta-analysis. Eur Heart J. 2019;40:1081-1088. doi: 10.1093/eurheartj/ehy435.
12. Cortés-Puch I, Wiley BM, Sun J, Klein HG, Welsh J, Danner RL, et al. Risks of restrictive red blood cell transfusion strategies in patients with cardiovascular disease (CVD): a meta-analysis. Transfus Med. 2018;28:335-345.
13. Docherty AB, O'Donnell R, Brunskill S, Trivella M, Doree C, Holst L, et al. Effect of restrictive versus liberal transfusion strategies on outcomes in patients with cardiovascular disease in a non-cardiac surgery setting: systematic review and meta-analysis. BMJ. 2016;352:i1351.
14. Prick BW, Jansen AJ, Steegers EA, Hop WC, Essink-Bot ML, Uyl-de Groot CA, et al. Transfusion policy after severe postpartum haemorrhage: a randomised non-inferiority trial. BJOG. 2014;121:1005-14.

15. Odutayo A, Desborough MJ, Trivella M, Stanley AJ, Dorée C, Collins GS, et al. Restrictive versus liberal blood transfusion for gastrointestinal bleeding: a systematic review and meta-analysis of randomised controlled trials. Lancet Gastroenterol Hepatol. 2017;2:354-360.

Recommendations for postoperative wound management

INTRODUCTION

Whether a primary closure, a delayed closure, or a closure by second intention has been carried out, the keys to surgical wound care focus on adequate cleaning, management of the exudate, and prevention of associated complications, such as Surgical site infection (SSI), dehiscence and pain.

There are multiple solutions for washing and cleaning wounds and little evidence of their use. For cleaning, the use of physiological solution is recommended since it is an isotonic solution and does not interfere with the normal healing process[1]. Drinking water or distilled water can also be used to clean wounds.

130. Clean the surgical wound with sterile isotonic saline, drinking water, or distilled water.

Moderate level of evidence. Strong recommendation.

Surgical wounds with primary closure

INTRODUCTION

Some studies indicate that topical antibiotics applied to primary healing surgical wounds probably reduce the risk of SSI relative to no antibiotics, and relative to topical antiseptics. Skin adverse effects and pain should be considered.[2]

131. Topical antibiotics can be applied to primary closure surgical wounds after surgery to prevent surgical site infection.

Low level of evidence. Weak recommendation.

In general, it is recommended to manipulate the surgical wound as little as possible. Currently, there are no conclusive studies on the use of dressings indicating that covering surgical wounds with primary intention healing reduces the risk of SSI or that any wound dressing is more effective than another in reducing SSI rates, improving scarring, or pain [3]. In wounds with closure by primary intention, whenever possible, do not lift the dressing for the first 24-48 hours[4].

132. In wounds with closure by primary intention, whenever possible, it is suggested not to lift the dressing during the first 24-48 hours.

Low level of evidence. Weak recommendation.

The application of a dressing, connected to a vacuum pump, known as negative pressure, wound therapy, (NPWT) can reduce the rate of surgical site infection compared to standard wound dressings, according to some low-certainty studies that were predominantly small. There is still greater uncertainty about whether negative pressure wound therapy (NPWT) compared to standard dressings reduces most complications associated with surgical incisions, including mortality.[5]

REFERENCES

1. Fernandez R, Griffiths R. Water for wound cleansing. Cochrane Database of Systematic Reviews 2012, Issue 2. Art. No.: CD003861. DOI: 10.1002/14651858.CD003861.pub3.
2. Heal CF, Banks JL, Lepper PD, Kontopantelis E, van Driel ML. Topical antibiotics for preventing surgical site infection in wounds healing by primary intention. Cochrane Database of Systematic Reviews 2016, Issue 11. Art. No.: CD011426. DOI: 10.1002/14651858. CD011426.pub2
3. Dumville JC, Gray TA, Walter CJ, Sharp CA, Page T, Macefield R, Blencowe N, Milne TKG, Reeves BC, Blazeby J. Dressings for the prevention of surgical site infection. Cochrane Database of Systematic Reviews 2016, Issue 12. Art. No.: CD003091. DOI: 10.1002/14651858.CD003091.pub4.
4. NICE Guideline Updates Team (UK). Surgical site infections: prevention and treatment. London: National Institute for Health and Care Excellence (UK); 2019 Apr. (NICE Guideline, No. 125.) Available from: https://www.ncbi.nlm.nih.gov/books/NBK542473/.
5. Webster J, Liu Z, Norman G, Dumville JC, Chiverton L, Scuffham P, Stankiewicz M, Chaboyer WP. Negative pressure wound therapy for surgical wounds healing by primary closure. Cochrane Database of Systematic Reviews 2019, Issue 3. Art. No.: CD009261. DOI: 10.1002/14651858.CD009261.pub4.

Open surgical wounds

INTRODUCTION

In these surgical wounds, the extension, depth, volume of exudate and risk of infection pose a challenge in their management.

Although the loss of the skin continuity solution facilitates the access of microorganisms to the body, there is no solid evidence evaluated to date on the relative effectiveness of antiseptics, antibiotics, and antibacterial products for use in open surgical wounds.[6]

NPWT is the most common alternative for secondary intention surgical wound management.[7] Some small studies have shown the beneficial effect of NPWT in reducing post-sternotomy mediastinitis and sternal wound infection.[8] Some small randomized clinical trials indicate a shorter healing time in the use of NPWT versus alginate and silicone dressings, without being conclusive due to the sample size.[9]

133. The use of NPWT can reduce the risk of surgical site infection and shorten healing in open surgical wounds, mainly in abdominal or thoracic surgeries.

Low level of evidence. Weak recommendation.

REFERENCES

1. Norman G, Dumville JC, Mohapatra D, Owens GL, Crosbie EJ. Antibiotics and antiseptics for surgical wounds healing by secondary intention. Cochrane Database of Systematic Re- views 2016, Issue 3. Art. No.: CD011712. DOI: 10.1002/14651858.CD011712.pub2
2. García-Fernández F, Blasco-García M, Rueda-López J, Segovia-Gómez T. Cura avanzada de heridas: terapia de presión negativa, factores de crecimiento plaquetario, sustitutos epidér- micos y apósitos bioactivos. In: García-Fernández F, Soldevilla- Agreda J, Torra Bou J, edi- tors. Atención Integral de las Heridas Crónicas-2ª edición. Logroño: GNEAUPP-FSJJ; 2016. p. 531-45.

3. Broadus Zane Atkins, MD, Mary Kay Wooten, MSN, Jean Kistler, NP, Kista Hurley, PA-C, G. Chad Hughes, MD, and Walter G. Wolfe, MD. Does Negative Pressure Wound Therapy Have a Role in Preventing Poststernotomy Wound Complications?. Does Negative Pressure Wound Therapy Have a Role in Preventing Poststernotomy Wound Complications?

4. Dumville JC, Owens GL, Crosbie EJ, Peinemann F, Liu Z. Negative pressure wound therapy for treating surgical wounds healing by secondary intention. Cochrane Database of Systematic Reviews 2015, Issue 6. Art. No.: CD011278. DOI: 10.1002/14651858.CD011278.pub2.

Recommendations at discharge

INTRODUCTION

The discharge and follow-up of patients must be planned and agreed upon, considering patients and caregivers, especially in elderly or dependent patients. The instructions to each patient about their care should be personalized. Upon discharge, it must be ensured that the patient has understood the care he should receive and the follow-up to which he will be subjected. The use of standardized information documents improves patients' understanding of the information received at discharge.

The patient must be discharged with the appointments for follow-up including those corresponding to other services.

Personalized discharge recommendations influence the mean stay and readmissions. Adequate, understandable, and complete discharge information improves patient satisfaction.

Support therapy at discharge is recommended: physical therapy or physical exercise, stomata care and diet.

A telephone follow-up is also recommended within the first 24 hours. The extension of the telephone follow-up can be important for some pathologies.

134. Patients and their caregivers should receive personalized, understandable, and complete information upon discharge. Planning discharge and providing adequate information on post-discharge care influences the mean stay and readmissions.

High level of evidence. Strong recommendation.

1. Shepperd S, Lannin NA, Clemson LM, McCluskey A, Cameron ID, Barras SL. Discharge planning from hospital to home. Cochrane Database Syst Rev. 2013 Jan 31;(1):CD000313.

2. Younis J, Salerno G, Fanto D, Hadjipavlou M, Chellar D, Trickett JP. Focused preoperative patient stoma education, prior to ileostomy formation after anterior resection, contributes to a reduction in delayed discharge within the enhanced recovery programme.Int J Colorectal Dis. 2012;27(1):43-7.

Audits

INTRODUCTION

The results of a study in which a visual tool was used with the audit data of the different professionals, improved adherence to intraoperative antibiotic prophylaxis; temperature control; goal-guided intravenous fluid therapy; prophylaxis of postoperative nausea and vomiting and postoperative fluid restriction[1].

135. Audits of intensified recovery procedures are recommended to assess clinical adequacy and effectiveness.

Moderate level of evidence. Strong recommendation.

REFERENCES

1. Bisch SP, Wells T, Gramlich L, Faris P, Wang X, Tran DT, Thanh NX, Glaze S, Chu P, Ghatage P, Nation J, Capstick V, Steed H, Sabourin J, Nelson G. Enhanced Recovery After Surgery (ERAS) in gynecologic oncology: System-wide implementation and audit leads to improved value and patient outcomes. Gynecol Oncol. 2018 Oct;151(1):117-123.

7.2 SPECIFIC (BY SPECIALTIES)

7.2.1 ESOPHAGEAL SURGERY

1. Drains

Cervical Drainage

The use of cervical drainage after esophagectomy has not been proved to reduce local complications in the wound, such as hematoma or seroma[1]. Furthermore, there is no evidence that suggests that its use reduces risk of anastomotic dehiscence[1], thus, it is not recommended as a routine, since it does not provide significant benefits.

1. Cervical drains after esophagectomy have no proven advantages over not using them, so they are not recommended as a routine.

Moderate level of evidence. Strong recommendation.

REFERENCES

1. Choi HK, Law S, Chu KM, Wong J. The value of neck drain in esophageal surgery: a randomized trial. Dis Esophagus. 2017:11:40-2.

Thoracic drains

Currently available evidence to demonstrate the benefit of using chest drains after esophagectomy is extremely limited and a solid connection cannot be established[1]. Even though most of the published guidelines and clinical pathways include them amongst their recommendations, as they could prevent pulmonary compression and be used as a guide to monitor the presence of bleeding and/or leaks (air, chylous or anastomotic). However, using them causes more pain which results in worst ventilation and mobility[2].

2. The use of thoracic drains after esophagectomy is recommended, although it is advisable to reduce the number of drains and the time they remain (a single drain may be sufficient), if there is no air, anastomotic, or chylothorax leak.

Low level of evidence. Strong recommendation.

REFERENCES

1. Low DE, Allum W, De Manzoni G, Ferri L, Immanuel A, Kuppusamy M, et al. Guidelines for Perioperative Care in Esophagectomy: Enhanced Recovery After Surgery (ERAS®) Society Recommendations. World J Surg. 2019; 43(2):299-330.

2. De Pasqual CA, Weindelmayer J, Laiti S, La Mendola R, Bencivenga M, Alberti L, et al. Perianastomotic drainage in Ivor-Lewis esophagectomy, does habit affect utility? An 11-year single-center experience. Updates Surg. 2020;72:47-53.

Extrapolated evidence from lung resection surgery supports the use of a single chest drain[1,2], with the same morbidity, but a considerable reduction in postoperative pain, cost and hospital stay compared to the placement of a greater number of drains[3,4]. Furthermore, it appears that the use of passive drains is just as effective as active ones[5].

3. The placement of a single chest drain is recommended over several, since it seems just as effective, but cheaper and less painful.

Moderate level of evidence (extrapolated). Strong recommendation.

4. The use of a passive drain (without continuous suction or aspiration) is recommended, as it is just as effective as an active one..

Low level of evidence. Strong recommendation.

REFERENCES

1. Gomez Caro A, Roca MJ, Torres J, Cascales P, Terol E, Castañer J, et al. Successful use of a single chest drain post lobectomy instead of two classical drains: a randomized study. Eur J Cardiothoracic Surg. 2006;29:562-6.

2. Pawelczyk K, Marciniak M, Kacprzak G, Kolodziej J. One or two drains after lobectomy ¿A comparison of both methods in the inmediate postoperative period. Thorac Cardiovasc Surg. 2007;55:313-6.

3. Alex J, Ansari J, Bhalkar P, Agarwala S, Rehman M, Saleh A, et al. Comparison of the inmediate postoperative outcome of using the conventional two drains versus a single drain after lobectomy. Ann Thorac Surg. 2003;76:1046-9.

4. Refai M, Brunelli A, Salati M, Xiumè F, Pompili C, Sabbatini A, et al. The impact of chest tube removal on pain and pulmonary function after pulmonary resection. Eur J Cardiothorac Surg. 2012;41:820-2.

5. Johansson J, Lindberg CG, Johnsson F, von Holstein CS, Zilling T, Walther B. Active or passive chest drainage after esophagectomy in 101 patients: a prospective randomized study. Br J Surg. 1998;85:1143-6.

There are randomized controlled trials demonstrating that early removal of a chest drain, even with outputs of 200-300 ml / 24 hours without any leaks, (air, anastomotic, or chylous), is safe and could improve postoperative comfort and reduce hospital stay[1-4].

5. Removal of the thoracic drain is recommended if the output is less than or equal to 200-300 ml / 24 h and there is no leakage of intestinal material or air or chylous leakage.

Moderate level of evidence. Strong recommendation.

REFERENCES

1. Yao F, Wang J, Yao J, Hang F, Cao S, Qian J, et al. Early chest tube removal after thoracoscopic esophagectomy with high output. J Laparoendosc Adv Surg Tech A. 2016;26:17-22.

2. Novoa NM, Jiménez MF, Varela G. When to remove a chest tube. Thorac Surg Clin. 2017;27:41-6.

3. Hessami MA, Najafi F, Hatami S. Volume threshold for chest tube removal: a randomized controlled trial. J Inj Violence Res. 2009;1:33-6.
4. Findlay JM, Gillies RS, Millo J, Sgromo B, Marshall RE, Maynard ND. Enhanced recovery for esophagectomy: a systematic review and evidence - based guidelines. Ann Surg. 2014; 259:413-31.

Abdominal Drains

The use of abdominal drains after a gastrectomy does not offer benefits compared to not using them[1-3].

6. Following esophagectomy, using abdominal drains as a routine is not recommended.

High level of evidence. Strong recommendation.

REFERENCES

1. Wang Z, Chen J, Su K, Dong Z. Abdominal drainage versus no drainage post-gastrectomy for gastric cancer (Review). Cochrane Database Syst Rev. 2015;5:CD008788.
2. Álvarez R, Molina H, Torres, Cancino A. Total gastrectomy with or without abdominal drains. A prospective randomized trial. Rev Esp Enferm Dig. 2005;97:562-9.
3. Kim J, Lee J, Hyung WJ, Cheong JH, Chen J, Choi SH, et al. Gastric cancer surgery without drains: a prospective randomized trial. J Gastrointest Surg. 2004;8:727-32.

2. Route of administration and postoperative initiation of nutrition in esophagectomy.

ENTERAL FEEDING AND USE OF NUTRITION PROBES

The oral and enteral routes should be chosen preferably over parenteral nutrition in patients with esophageal neoplasms that require nutritional supplements as they are the most physiological routes and those related to a better nutritional intake and fewer complications[1,2]. Despite being recommended to place a jejunostomy catheter or nasojejunal or nasoduodenal tube to provide nutritional support in patients with limited oral intake, these types of techniques are not exempt from morbidity and mortality and considerable repositioning rates[3]. Currently, there is no evidence in favor of any specific type of catheter used for the administration of adequate nutritional preparations in this type of patients[4,5].

Jejunostomy placement is associated with a mortality rate of 0-0.5% and a reoperation rate of 0-2.9%. However, minor complications are more frequent, such as infection at the skin entrance (0.4-16%), leaks (1.4-25%) and gastrointestinal discomfort (10-39%). The use of the nasojejunal tube entails fewer complications but is accompanied by greater discomfort and a dislocation rate that ranges between 20% and 35%.

7. In the preoperative management of patients with dysphagia or aphagia who are undergoing esophagectomy, the use of enteral nutrition through a feeding tube is recommended in those cases with a high risk of malnutrition and inability to achieve an adequate oral intake to meet nutritional requirements.

Moderate level of evidence. Strong recommendation.

8. After an esophagectomy, it is recommended to be able to meet the nutritional requirements by oral and / or enteral route between the third and sixth postoperative day. The use of enteral nutrition tubes should be carried out selectively in patients at risk or with nutritional requirements that cannot be covered by oral intake.

   Moderate level of evidence. Strong recommendation.

9. If necessary, jejunostomy, nasojejunal or nasoduodenal tubes can be used with the same effectiveness since current evidence does not allow recommending a specific route of administration of enteral nutrition.

   Moderate level of evidence. Strong recommendation.

REFERENCES

1. Liu K, Ji S, Xu Y, Diao Q, Shao C, Luo J, et al. Safety, feasibility, and effect of an enhanced nutritional support pathway including extended preoperative and home enteral nutrition in patients undergoing enhanced recovery after esophagectomy: a pilot randomized clinical trial. Dis Esophagus. 2020;33:doz030.
2. Chen MJ, Wu IC, Chen YJ, Wang TE, Chang YF, Yang CL, et al. Nutrition therapy in esophageal cancer-Consensus statement of the Gastroenterological Society of Taiwan. Dis Esophagus. 2018;31.
3. Álvarez-Sarrado E, Mingol Navarro F, Rosellón R, Ballester Pla N, Vaqué Urbaneja FJ, Muniesa Gallardo C, et al. Feeding jejunostomy after esophagectomy cannot be routinely recommended. Analysis of nutritional benefits and catheter-related complications. J Thorac Dis. 2019;11:S812-S818.
4. Lorimer PD, Motz BM, Watson M, Truffan SJ, Prabhu RS, Hill JS, et al. Enteral feeding access has an impact on outcomes for patients with esophageal cancer undergoing esophagectomy: an analysis of SEER-Medicare. Ann Surg Oncol. 2019;26:1311-9.
5. Weijs TJ, Berkelmans GH, Nieuwenhuijzen GA, Ruurda JP, van Hillegersberg R, Soeters PB, et al. Routes for early enteral nutrition after esophagectomy: a systematic review. Clin Nutr. 2015;34:1-6.
6. Zheng T, Zhang Y, Zhu S, Ni Z, You Q, Sun X, et al. A prospective randomized trial comparing jejunostomy and nasogastric feeding in minimally invasive McKeown esophagectomy. J Gastrointest Surg. 2020;24(10):2187-96.

Early postoperative oral / enteral nutrition

The early initiation of enteral feeding during the postoperative period of esophagectomy has proven to be safe[1,2], favoring functional intestinal recovery and reducing hospital stay[3,4].

10. The early start (in the first 24 hours) of enteral nutrition after esophagectomy is recommended since it is safe and facilitates postoperative recovery.

    Moderate level of evidence. Strong recommendation.

REFERENCES

1. Low DE, Allum W, De Manzoni G, Ferri L, Immanuel A, Kuppusamy M, et al. Guidelines for Perioperative Care in Esophagectomy: Enhanced Recovery After Surgery (ERAS®) Society Recommendations. World J Surg. 2019; 43(2):299-330.

2. Giancopuzzi S, Weindelmayer J, Treppiedi E, Bencivenga M, Ceola M, Priolo S, et al. Enhanced recovery after surgery protocol in patients undergoing esophagectomy for cancer: a single center experience. Dis Esophag. 2017;30:1-6.
3. Tomaszek SC; Cassivi SD, Allen MS, Shen KR, Nichols FC 3rd, Deschamps C, et al. An alternative postoperative pathway reduces length of hospitalisation following oesophagectomy. Eur J Cardio-thor Surg. 2010;37:807-13.
4. Kingma BF, Steenhagen E, Ruurda JP, van Hillegersberg R. Nutritional aspects of enhanced recovery after esophagectomy with gastric conduit reconstruction. J Surg Oncol. 2017;116: 623-9.

Early initiation of oral feeding after esophagectomy seems safe and feasible. Some randomized controlled trials show that an early oral diet (in the first 24 hours) after esophagectomy does not increase the percentage of postoperative complications[1-5]. However, in the current literature there is controversy about the effectiveness and safety of its use compared to later initiation in relation to the percentage of anastomotic dehiscence.

Other routes of enteral feeding could be considered and used in combination with the oral route to guarantee correct nutritional support during the postoperative period of an esophagectomy.

11. The most appropriate route for administering enteral feeding during the early postoperative period of esophagectomy is not clearly defined. In this regard, the early initiation of tolerance by the oral route appears to be effective and safe without increasing the number of major postoperative complications.

Low level of evidence. Weak recommendation.

REFERENCES

1. Weijs TJ, Berkelmans GHK, Nieuwenhuijzen GAP, Dolmans AC, Kouwenhoven EA, Rosman C, et al. Inmediate postoperative oral nutrition following esophagectomy: a multicenter clinical trial. Ann Thorac Surg. 2016;102:1141-8.
2. Berkelmans GHK, Fransen L, Dolmans-Zwartjes AC, Kouwenhoven EA, van Det MJ, Nilsson M, et al. Direct oral feeding following minimally invasive esophagectomy (Nutrient II trial): an international, multicenter, open-label randomized controlled trial. Ann Surg. 2020;271: 41-7.
3. Sun HB, LI Y, Liu XB, Zhang RX, Wang ZF, Lerut T, et al. Early oral feeding following McKeown minimally invasive esophagectomy. An open-label, randomized, controlled, non inferiority trial. Ann Surg. 2018;267:435-42.
4. Bolton JS, Conway WC, Abbas AE. Planned delay of oral intake after esophagectomy reduces the cervical anastomotic leak rate and hospital length of stay. J Gastrointest Surg. 2014;18:304-9.
5. Eberhard KE, Achiam MP, Rolff HC, Belmouhand M, Svendsen LB, Thorsteinsson M. Comparison of “nil by mouth” versus early oral intake in three different diet regimens following esophagectomy. World J Surg. 2017;41:1575-83.

3. Use of a decompressive nasogastric tube

Traditionally, the use of a nasogastric tube has been considered mandatory after esophagectomy to decompress the plasty, avoid its dilation, reduce anastomotic tension, and avoid vomiting, pain and possible aspirations.

However, there are conflicting data in the literature regarding its use and the risk of anastomotic and respiratory complications. Thus, some studies do not recommend its use routinely, as clear benefits have not been demonstrated in reducing complications and causing a delay in the onset of oral tolerance, lengthening hospital stay[1,2]. A recent meta-analysis concludes that immediate or early removal of the nasogastric tube does not increase the number of anastomotic dehiscence, pulmonary complications, or postoperative mortality, thus reducing hospital stay[3]. Early removal after placement seems safe and improves patient comfort, accelerating oral tolerance[4,5].

12. Even though the evidence based on more recent studies questions its use in a systematic way, the use of a nasogastric tube is currently suggested after esophagectomy.

    Low level of evidence. Weak recommendation.

13. If it is placed, its early removal should be considered in the first 48 post-operative hours, which reduces post-operative fasting time, hospital stay and improves patient comfort.
    Moderate level of evidence. Strong recommendation.

REFERENCES

1. Low DE, Allum W, De Manzoni G, Ferri L, Immanuel A, Kuppusamy M, et al. Guidelines for Perioperative Care in Esophagectomy: Enhanced Recovery After Surgery (ERAS®) Society Recommendations. World J Surg. 2019; 43(2):299-330.
2. Menéndez-Jiménez M, Bruna-Esteban M, Mingol M, Vaqué J, Hervás D, Álvarez-Sarrado E, et al. Uso de sonda nasogástrica en pacientes sometidos a esofaguectomía: ¿Un gesto innecesario? Cir Esp. 2020;S0009-739X(20)30158-5.
3. Findlay JM, Gillies RS, Millo J, Sgromo B, Marshall RE, Maynard ND. Enhanced recovery for esophagectomy: a systematic review and evidence - based guidelines. Ann Surg. 2014; 259:413-31.
4. Hayashi M, Kawakubo H, Shoji Y, Mayanagi S, Nakamura R, Suda K, et al. Analysis of the effect of early versus conventional nasogastric tube removal on postoperative complications after transthoracic esophagectomy: a single-center, randomized controlled trial. World J Surg. 2019;43:580-9.
5. Mistry RC, Vijayabhaskar R, Karimundackal G, Jiwnani S, Pramesh CS. Effect of short-term vs prolonged nasogastric decompression on major postesophagectomy complications. Arch Surg. 2012;147:747-51.

4. Admission to Intensive Care Unit or Resuscitation

Conventional postoperative management after esophagectomy involved admission as a routine in a post-anesthesia recovery unit (PAR) or Intensive Care Unit (ICU). With the availability of better pain control, minimally invasive approaches and early extubating, among other measures, postoperative management of patients undergoing esophagectomy in Intermediate Care Units[1] is possible, without the need for admission in a PAR or ICU, which has been shown to reduce hospital stay, with no differences in morbidity and mortality or in the readmission rate[2,3].

14. Postoperative management of patients undergoing esophagectomy should be individualized and does not routinely require admission to the Intensive Care or Recovery Unit. The availability of an Intermediate Care Unit is a safe alternative for low-risk patients.

Moderate level of evidence. Strong recommendation.

REFERENCES

1. Low DE, Allum W, De Manzoni G, Ferri L, Immanuel A , Kuppusamy M, et al. Guidelines for perioeprative care in esophagectomy: Enhanced Recovery After Surgery (ERAS) Society recommendations. World J Surg. 2019;43:299-330.
2. Pisarska M, Małczak P, Major P, Wysocki M, Budzyński A, Pędziwiatr M. Enhanced recovery after surgery protocol in oesophageal cancer surgery: Systematic review and meta-analysis. PLoS One. 2017;12:e0174382.
3. Chen L, Sun L, Lang Y, Wu J, Yao L, Ning J, et al. Fast-track surgery improves postoperative clinical recovery and cellular and humoral immunity after esophagectomy for esophageal cancer. BMC Cancer. 2016;16:449.
35. Wunsch H, Gershengorn HB, Cooke CR, Guerra C, Angus DC, Rowe JW, et al. Use of intensive care services for medicare beneficiaries undergoing major surgical procedures. Anesthesiology. 2016;124:899-907.

7.2.2 CARDIOVASCULAR SURGERY

Preoperative measurement of hemoglobin a1c

Hyperglycemia in hospitalized surgical patients is associated with increased morbidity and mortality, so it must be avoided[1].

Elevated hemoglobin A1c (HbA1c) levels correlate with poor glycemic control, therefore, they increase the risk of the patient presenting hyperglycemia upon admission. Optimal HbA1c2 levels were defined as <7% in a consensus document of the American and European Diabetes Associations (ADA and EASD). It has been shown that the higher the HbA1c level, the higher the incidence of deep sternal wound infection, ischemic problems and other complications[3,4].

Known nondiabetic patients with elevated HbA1c levels have also been shown to have a higher risk of postoperative mortality[5].

Therefore, it is advisable to monitor HbA1c levels in all patients who undergo cardiac surgery.

Except in urgent surgery, with values> 9% (indicates having presented recurrence of severe hyperglycemia) or <5% (indicates having presented recurrent severe hypoglycemia), the intervention should be delayed[6].

1. A preoperative control of HbA1c levels is recommended in all patients who undergo cardiac surgery to stratify the surgical risk.

Moderate level of evidence. Strong recommendation.

2. When the preoperative HbA1c determination is <5% or > 9%, it is suggested to postpone the intervention, except if urgent surgery is needed, until adequate glycemic control is achieved.

Low level of evidence. Weak recommendation.

REFERENCES

1. Palermo NE, Gianchandani RY, McDonnell ME, Alexanian SM. Stress Hyperglycemia During Surgery and Anesthesia: Pathogenesis and Clinical Implications. Curr Diab Rep. 2016; 16(3):33. doi:10.1007/s11892-016-0721-y.
2. Inzucchi SE, Bergenstal RM, Buse JB, Diamant M, Ferrannini E, Nauck M, et al. Management of hyperglycemia in type 2 diabetes: A patient-centered approach. Diabetes Care. 2012; 35(6):1364-79. doi:10.2337/dc12-0413.
3. Umpierrez G, Cardona S, Pasquel F, Jacobs S, Peng L, Unigwe M, et al. Randomized controlled trial of intensive versus conservative glucose control in patients undergoing coronary artery bypass graft surgery: GLUCOCABG trial. Diabetes Care. 2015;38(9):1665-72. doi: 10.2337/dc15-0303.
4. Narayan P, Naresh Kshirsagar, Mandal CK, Ghorai PA, Rao YM, Das D, et al. Preoperative Glycosylated Hemoglobin: A Risk Factor for Patients Undergoing Coronary Artery Bypass. Ann Thorac Surg. 2017;104(2):606-12. doi:10.1016/j.athoracsur.2016.12.020.
5. Hudson CCC, Welsby IJ, Phillips-Bute B, Mathew JP, Lutz A, Chad Hughes G, et al. Glycosylated hemoglobin levels and outcome in non-diabetic cardiac surgery patients. Can J Anesth. 2010;57(6):565-72. doi:10.1007/s12630-010-9294-4.
6. Cosson E, Catargi B, Cheisson G, Jacqueminet S, Ichai C, Leguerrier AM, et al. Practical management of diabetes patients before, during and after surgery: A joint French diabetology and anaesthesiology position statement. Diabetes Metab. 2018;44(3):200-16. doi:10.1016/j.diabet.2018.01.014.

Nasal decolonization of staphylococcus aureus

Staphylococcus aureus is the germ responsible for most surgical site and prosthetic infectious complications after cardiac surgery. Between 18 and 30% of patients undergoing this type of surgery have nasal colonization, which implies a risk up to 3 times greater of presenting bacteremia or surgical wound infections by S. aureus.

It is widely established that a decolonization of carriers must be carried out[1,2]. There is the possibility of screening them by culture or PCR techniques and subsequently performing a selective treatment of patients with positive results[3], however, the current evidence points to universal decolonization for practical, logistical or cost-effectiveness reasons, although they warn that it may lead to the appearance of antibiotic resistance[4,5].

Decolonizing treatment should include intranasal mupirocin as a topical antibiotic[6,7], but always within a package of actions together with hygiene education and daily showers with antiseptics such as chlorhexidine. It should be done for 5 days before the intervention.

3. Decolonization of known nasal carriers of Staphylococcus aureus is recommended in the preoperative period of cardiac surgery.

High level of evidence. Strong recommendation.

4. Universal decolonization is recommended for patients undergoing cardiac surgery.

   Moderate level of evidence. Strong recommendation.

5. The decolonization treatment is recommended to be performed with intranasal mupirocin and within a package of measures.

   High level of evidence. Strong recommendation.

REFERENCES

1. Sousa-Uva* M, Head SJ, Milojevic M, Collet J-P, Landoni G, Castella M, et al. 2017 EACTS Guidelines on perioperative medication in adult cardiac surgery. Eur J Cardio-Thoracic Surg. 2018;53(1):5-33.
2. Lepelletier D, Saliou P, Lefebvre A, Lucet JC, Grandbastien B, Bruyère F, et al. Preoperative risk management: Strategy for Staphylococcus aureus preoperative decolonization (2013 update). Med Mal Infect. 2014;44(6):261-7.
3. Kline SE, Neaton JD, Lynfield R, Ferrieri P, Kulasingam S, Dittes K, et al. Randomized controlled trial of a self-administered five-day antiseptic bundle versus usual disinfectant soap showers for preoperative eradication of Staphylococcus aureus colonization. Infect Control Hosp Epidemiol. 2018;39(9):1049-57.
4. Lazar HL, Salm T Vander, Engelman R, Orgill D, Gordon S. Prevention and management of sternal wound infections. J Thorac Cardiovasc Surg. 2016;152(4):962-72. doi:10.1016/j.jtcvs.2016.01.060
5. George S, Leasure AR, Horstmanshof D. Effectiveness of decolonization with chlorhexidine and mupirocin in reducing surgical site infections: A systematic review. Dimens Crit Care Nurs. 2016;35(4):204-22.
6. Sakr A, Brégeon F, Rolain JM, Blin O. Staphylococcus aureus nasal decolonization strategies: a review. Expert Rev Anti Infect Ther. 2019;17(5):327-40.
7. Septimus EJ. Nasal decolonization: What antimicrobials are most effective prior to surgery? Am J Infect Control. 2019;47:A53-7.

Antifibrinolytic drugs in cardiac surgery

Bleeding during the perioperative period of cardiac surgery is a common complication and its appearance can worsen the results[1].

Antifibrinolytic drugs, such as tranexamic acid and Ɛ-aminocaproic acid, are of adequate availability, low risk profile, high profitability, and easy implantation[2-4], so they are widely used in routine practice. Its use is recommended from the beginning of the intervention to prevent hyperfibrinolysis that occurs during the procedure. The greatest evidence is present in surgeries with Extracorporeal Circulation (ECC).

Tranexamic acid has been shown to reduce bleeding, the need for reoperation, and transfusion requirements[5,6].

The dosage of these drugs is not yet fully established. High doses, without providing benefit, seem to associate seizures as a side effect, therefore the maximum recommended dose is 100 mg / kg and requires adjustment in patients with renal failure[7].

6. To obtain an antifibrinolytic effect, it is recommended to use tranexamic acid or epsilon aminocaproic acid during cardiac surgery procedures with ECC.

   High level of evidence. Strong recommendation.

7. The use of tranexamic acid is recommended since it is associated with a decrease in bleeding, transfusion, and the need for reoperation.

   Moderate level of evidence. Strong recommendation.

REFERENCES

1. Dyke C, Aronson S, Dietrich W, Hofmann A, Karkouti K, Levi M, et al. Universal definition of perioperative bleeding in adult cardiac surgery. J Thorac Cardiovasc Surg. 2014 May;147 (5):1458-1463.e1. doi:10.1016/j.jtcvs.2013.10.070
2. Apfelbaum JL, Nuttall GA, Connis RT, Harrison CR, Miller RD, Nickinovich DG, et al. Practice guidelines for perioperative blood management: An updated report by the american society of anesthesiologists task force on perioperative blood management. Anesthesiology. 2015 Feb;122(2):241-75. doi:10.1097/ALN.0000000000000463
3. Henry DA, Carless PA, Moxey AJ, O'Connell D, Stokes BJ, McClelland B, et al. Anti-fibrinolytic use for minimising perioperative allogeneic blood transfusion. Henry DA, editor. Cochrane Database Syst Rev. 2007 Oct 17;4(CD001886). doi:10.1002/14651858.CD001886.pub2
4. Leff J, Rhee A, Nair S, Lazar D, Sathyanarayana S, Shore-Lesserson L. A randomized, double-blinded trial comparing the effectiveness of tranexamic acid and epsilon-aminocaproic acid in reducing bleeding and transfusion in cardiac surgery. Ann Card Anaesth. 2019;22(3):265. doi:10.4103/aca.ACA_137_18
5. Myles PS, Smith JA, Forbes A, Silbert B, Jayarajah M, Painter T, et al. Tranexamic acid in patients undergoing coronary-artery surgery. N Engl J Med. 2017;376(2):136-48. doi: 10.1056/NEJMoa1606424.
6. Taam J, Yang QJ, Pang KS, Karanicolas P, Choi S, Wasowicz M, et al. Current Evidence and Future Directions of Tranexamic Acid Use, Efficacy, and Dosing for Major Surgical Procedures. J Cardiothorac Vasc Anesth. 2020 Mar;34(3):782-90. doi:10.1053/j.jvca.2019.06.042.
7. Hunt BJ. The current place of tranexamic acid in the management of bleeding. Anaesthesia. 2015;70:e18-53. doi:10.1111/anae.12910.

Thoracic drains

After cardiac surgery, it is always necessary to leave some type of drainage that allows evacuation of bleeding, as it always occurs in a smaller or greater quantity, since otherwise, its accumulation could lead to hemothorax or cardiac tamponade, entities with an incidence from 2 to 19% [1,2], and which carry a worse prognosis.

However, drainage tubes tend to coagulate in clinical practice, reaching up to 36% with some degree of obstruction due to internal coagulation[3].

8. Maintaining patency of the thoracic drains is recommended to avoid major complications such as cardiac tamponade or hemothorax.

   Moderate level of evidence. Strong recommendation.

REFERENCES

1. Balzer F, von Heymann C, Boyle EM, Wernecke KD, Grubitzsch H, Sander M. Impact of retained blood requiring reintervention on outcomes after cardiac surgery. J Thorac Cardio- vasc Surg. 2016;152(2):595-601.e4. doi:10.1016/j.jtcvs.2016.03.086.
2. Pompilio G, Filippini S, Agrifoglio M, Merati E, Lauri G, Salis S, et al. Determinants of pericardial drainage for cardiac tamponade following cardiac surgery. Eur J Cardiothorac Surg. 2011;39(5):e107-13. doi:10.1016/j.ejcts.2010.12.021.
3. Karimov JH, Gillinov AM, Schenck L, Cook M, Kosty Sweeney D, Boyle EM, et al. Incidence of chest tube clogging after cardiac surgery: A single-centre prospective observational stu- dy. Eur J Cardio-thoracic Surg. 2013;44(6):1029-36. doi:10.1093/ejcts/ezt140.

To ensure the permeability of the tubes, there are several techniques to break up the clots and facilitate their exit, which can be performed at the bedside by nursing staff, such as milking, creating negative pressure by occlusion near the patient and emptying the tube towards the collector (stripping) or compressing several segments of the tube folded on itself (“fan folding”). All these maneuvers in multiple studies and reviews have not shown to be useful, and they could even cause internal damage due to the increase in negative pressure[1-4], so they should not be performed.

There are also those who disconnect the tubes and pass a catheter inside to clean them[5], which breaks the sterile field and the inserted probe itself can cause damage to internal structures, so this maneuver should not be performed either.

Currently it can be recommended, in case of using classic or grooved drains, to leave the tube in a horizontal position on the patient's bed and then vertically to the collecting system, do not perform maneuvers that break the sterile field and only if essential, perform gentle compressions of the tube (“milking” or “ fan folding ”) [2].

9. Performing maneuvers that break the sterile field of the drains is not recommended as that may cause negative intrathoracic hyper pressure, both because they have not demonstrated efficacy, and because of the possible complications that they may cause.

High level of evidence. Strong recommendation.

REFERENCES

4. Day TG, Perring RR, Gofton K. Is manipulation of mediastinal chest drains useful or harmful after cardiac surgery? Interact Cardiovasc Thorac Surg. 2008;7(5):888-90. doi:10.1510/icvts.2008.185413
5. Halm MA. To Strip or Not to Strip? Physiological Effects of Chest Tube Manipulation. Am J Crit Care. 2007;16(6):609-12. doi:10.4037/ajcc2007.16.6.609.
6. Kirkwood P. Ask the Experts. CriticalCareNurse. 2002;22(4):70-2. doi:10.4037/ccn2002.22.4.70.
7. Wallen M, Morrison A, Gillies D, O’Riordan E, Bridge C, Stoddart F. Mediastinal chest drain clearance for cardiac surgery. Cochrane database Syst Rev. 2002;2(CD003042). doi:10.1002/14651858.CD003042.pub2.
8. Boyacioğlu K, Kalender M, Özkaynak B, Mert B, Kayalar N, Erentuğ V. A new use of fogarty catheter: Chest tube clearance. Hear Lung Circ. 2014;23(10):e229-30. doi:10.1016/j.hlc.2014.04.255.

There are three main types of drains: conventional, grooved or Blake® type and active cleaning.

Among the first 2 types of drainage, studies have been carried out with no differences between them, so that any of them can have the same utility[1,2]. Finally, a type of drainage with active cleaning has been developed. Studies that analyze this new technology provide encouraging results, although not fully conclusive[3-6]. Therefore, the use of active cleaning drains can be considered with the intention of reducing the complications of tamponade or hemothorax due to coagulation of the drains.

1. The use of active cleaning drains is suggested to reduce complications such as cardiac tamponade or hemothorax.

Moderate level of evidence. Weak recommendation

REFERENCES

9. Bjessmo S, Hylander S, Vedin J, Mohlkert D, Ivert T. Comparison of three different chest drainages after coronary artery bypass surgery-a randomised trial in 150 patients. Eur J Cardiothorac Surg. 2007;31(3):372-5.
10. Sakopoulos AG, Hurwitz AS, Suda RW, Goodwin JN. Efficacy of Blake drains for mediastinal and pleural drainage following cardiac operations. J Card Surg. 2005;20(6):574-7.
11. Sirch J, Ledwon M, Puski T, Boyle EM, Pfeiffer S, Fischlein T. Active clearance of chest drainage catheters reduces retained blood. J Thorac Cardiovasc Surg. 2016 Mar;151(3):832-838.e2.
12. St-Onge S, Ben Ali W, Bouhout I, Bouchard D, Lamarche Y, Perrault LP, et al. Examining the impact of active clearance of chest drainage catheters on postoperative atrial fibrillation. J Thorac Cardiovasc Surg. 2017;154(2):501-8.
13. Andersen ND. Active clearance technology to maintain chest tube patency: Practical, innovative, unproven. Vol. 151, The Journal of thoracic and cardiovascular surgery. United States; 2016. p. 839-40.
14. Grieshaber P, Heim N, Herzberg M, Niemann B, Roth P, Boening A. Active Chest Tube Clearance After Cardiac Surgery Is Associated With Reduced Reexploration Rates. Ann Thorac Surg. 2018;105(6):1771-7.

Optimization of sternal closure

Median sternotomy is the most common approach in cardiac surgery. The most usual and widespread closure is by means of cerclage with steel wires due to the ease of use, speed, low cost, and a relatively low rate of complications.

The closure of preference is with 6 simple wires, but there is some evidence that at least 8 wires should be used, and if the sternum is osteoporotic, it is better with crossed wires[1-3].

2. If the sternal closure is performed with wires, it is suggested to use at least 8 wires and / or crossed wires.

Low level of evidence. Weak recommendation.

REFERENCES

15. Kamiya H, Al-maisary SSA, Akhyari P, Ruhparwar A, Kallenbach K, Lichtenberg A, et al. The number of wires for sternal closure has a significant influence on sternal complications in high-risk patients. Interact Cardiovasc Thorac Surg. 2012 Oct;15(4):665-70.

16. Mirhosseini SJ, Ali-Hassan-Sayegh S, Mostafavi-Pour-Manshadi SMY, Hadibarhaghta- lab M, Lotfaliani MR. Figure-of-Eight Wire Sternal Closure Technique Can Reduce Post-Open Cardiovascular Surgery Chest Re-Exploration and Pain Scores in Diabetic Patients with Severe Obesity (Body Mass Index: 35-40). Int J Clin Exp Med Sci. 2015;1(3):38.
17. Ramzisham ARM, Raflis AR, Khairulasri MG, Ooi Su Min J, Fikri AM, Zamrin MD. Figure-of- eight vs. Interrupted sternal wire closure of median sternotomy. Asian Cardiovasc Thorac Ann. 2009;17(6):587-91.

The use of parasternal longitudinal reinforcing wires (Robicsek) has not shown a clear advantage in high-risk patients[1,2].

The natural extension of principles in bone stabilization learned elsewhere in the human body, leads us to the use of rigid fixation systems by means of titanium plates screwed to the sternum and / or ribs. The evidence points to a better and faster healing of the sternum, which entails a reduction in pain and allows an early recovery of the patient and shortens the hospital stay[3-5]. Its use is especially indicated in high-risk patients because it reduces complications and mortality[6].

3. Sternal fixation with titanium plates allows better sternal healing, a reduction in postoperative pain, and a shorter hospital stay. Its use is suggested in high-risk patients, in whom mortality and complications are also reduced.

Moderate level of evidence. Weak recommendation

REFERENCES

18. Schimmer C, Reents W, Berneder S, Eigel P, Sezer O, Scheld H, et al. Prevention of Sternal Dehiscence and Infection in High-Risk Patients: A Prospective Randomized Multicenter Trial. Ann Thorac Surg. 2008;86(6):1897-904.
19. Pinotti KF, Cataneo DC, Rodrigues OR, Cataneo AJM. Closure of the sternum with ancho- ring of the steel wires: Systematic review and meta-analysis. J Thorac Cardiovasc Surg. 2018;156(1):178-86.
20. Allen KB, Icke KJ, Thourani VH, Naka Y, Grubb KJ, Grehan J, et al. Sternotomy closure using rigid plate fixation: A paradigm shift from wire cerclage. Ann Cardiothorac Surg. 2018; 7(5):611-20.
21. Raman J, Lehmann S, Zehr K, De Guzman BJ, Aklog L, Garrett HE, et al. Sternal closure with rigid plate fixation versus wire closure: A randomized controlled multicenter trial. Ann Tho- rac Surg. 2012;94(6):1854-61.
22. Peigh G, Kumar J, Unai S, James DT, Hirose H, Diehl JT, et al. Randomized Trial of Sternal Closure for Low Risk Patients: rigid Fixation versus Wire Closure. Heart Surg Forum. 2017; 20(4):E164-E169.
23. Tam DY, Nedadur R, Yu M, Yanagawa B, Fremes SE, Friedrich JO. Rigid Plate Fixation Versus Wire Cerclage for Sternotomy After Cardiac Surgery: A Meta-Analysis. Ann Thorac Surg. 2018 Jul;106(1):298-304.

Prevention of hypothermia in the immediate postoperative period

General anesthesia produces an alteration in the regulation of body temperature, producing a decrease between 1 and 2 ° C during the first hour, further dropping to 3.5ºC if no measures are taken after the third hour of anesthesia[1]. Also, the skin

exposed for prolonged periods and the infusion of large volumes of intravenous fluids and irrigation of the surgical field favors this situation.

We define postoperative hypothermia as the impossibility of maintaining normothermia (≥36 ° C) after 2 to 5 hours of admission to the Critical Unit[1,2.]

Complications related to perioperative hypothermia include the appearance of coagulopathy associated with greater transfusion needs, an increase in the surgical wound infection rate, delayed drug metabolism, slower and longer recovery, chills, and thermal discomfort. All of this leads to longer hospital stays and increased mortality[3,4].

The prevention of hypothermia should be carried out using warm air blankets, the infusion of warm intravenous fluids at 37 °C[5] and the elevation of the room temperature in the immediate postoperative period[6-8]. Once normothermia has been reached, the previous measures will be withdrawn, and the monitoring of body temperature will continue.

4. We recommend the use of warm air blankets, the infusion of warm intravenous fluids, and raising the room temperature to avoid sustained hypothermia (<36 ° C) after ECC and in the early postoperative period.

   Moderate level of evidence. Strong recommendation.

5. We recommend monitoring body temperature upon arrival of the patient to the Critical Care Unit.

   High level of evidence. Strong recommendation.

REFERENCES

24. Sessler DI. Perioperative heat balance. Anesthesiology. 2000;92(2):578-90.
25. Sessler DI. Perioperative thermoregulation and heat balance. Lancet. 2016;387(10038): 2655-64.
26. Karalapillai D, Story D, Hart GK, Bailey M, Pilcher D, Cooper DJ, et al. Postoperative hypothermia and patient outcomes after elective cardiac surgery. Anaesthesia. 2011;66(9): 780-4.
27. Apfelbaum JL, Silverstein JH, Chung FF, Connis RT, Fillmore RB, Hunt SE, et al. Practice Guidelines for Postanesthetic Care. Anesthesiology. 2013;118(2):291-307.
28. NICE: National Institution for Health and Care Excelence.. Inadvertent perioperative hypothermia overview. NICE pathways. 2017;(February):1-12. Disponible en: http://pathways.nice.org.uk/pathways/inadvertent-perioperative-hypothermia.
29. Campbell G, Alderson P, Smith AF, Warttig S. Warming of intravenous and irrigation fluids for preventing inadvertent perioperative hypothermia. Cochrane Database Syst Rev. 2015;4(CD009891). doi:10.1002/14651858.CD009891.pub2.
30. Evron S, Weissman A, Toivis V, Shahaf DB, You J, Sessler DI, et al. Evaluation of the temple touch pro, a novel noninvasive coreerature monitoring system. Anesth Analg. 2017;125(1): 103-9.
31. Macias AA, Valedon A, Durán H, Bayter-Marin JE, Rubio J, Cárdenas-Camarena L. Effects of Thermal Protection in Patients Undergoing Body Contouring Procedures: A Controlled Clinical Trial. Aesthetic Surg J. 2018;38(4):448-56.

Biomarkers and Prevention Strategies for Acute Kidney Damage

Acute kidney injury (AKI) associated with cardiac surgery is a complication that appears depending on the procedure and its definition in 22-36% of patients[1-4]. Its appearance entails an increase in morbidity and mortality and a significant impact on total healthcare spending[3].

The classic definitions of kidney injury RIFLE (R-renal risk, I-injury, F-failure, L-loss of kidney function, E-end-stage renal disease) [5], AKIN (Acute Kidney Injury Network) [6] and KDIGO (Kidney Disease: Improving Global Outcomes) [7] use increased creatinine, being able to delay its detection between 24 and 72 h compared to new biomarkers such as tissue inhibitor of metalloprotease-2 (IGFBP7), insulin-like growth factor binding protein 7 (TIMP-2) and lipocalin associated with neutrophil gelatinase (NGAL) [8,9].

15. We suggest the determination of biomarkers for the early identification of AKI in patients at risk to guide an early intervention strategy with the aim of reducing AKI.

Moderate level of evidence. Weak recommendation

REFERENCES

1. Chen S-W, Chang C-H, Fan P-C, Chen Y-C, Chu P-H, Chen T-H, et al. Comparison of contemporary preoperative risk models at predicting acute kidney injury after isolated coronary artery bypass grafting: a retrospective cohort study. BMJ Open. 2016 Jun 27;6(6):e010176. doi:10.1136/bmjopen-2015-010176.
2. Lee CC, Chang CH, Chen SW, Fan PC, Chang SW, Chen YT, et al. Preoperative risk assessment improves biomarker detection for predicting acute kidney injury after cardiac surgery. PLoS One. 2018;13(9):1-13. doi:10.1371/journal.pone.0203447.
3. Hu J, Chen R, Liu S, Yu X, Zou J, Ding X. Global Incidence and Outcomes of Adult Patients with Acute Kidney Injury after Cardiac Surgery: A Systematic Review and Meta-Analysis. J Cardiothorac Vasc Anesth. 2016;30(1):82-9. doi:10.1053/j.jvca.2015.06.017.
4. Chang CH, Lee CC, Chen SW, Fan PC, Chen YC, Chang SW, et al. Predicting acute kidney injury following mitral valve repair. Int J Med Sci. 2016;13(1):19-24. doi:10.7150/ ijms.13253.
5. Van Biesen W, Vanholder R, Lameire N. Defining acute renal failure: RIFLE and beyond. Clin J Am Soc Nephrol. 2006;1(6):1314-9. doi:10.2215/CJN.02070606.
6. Mehta RL. From acute renal failure to acute kidney injury: Emerging concepts. Crit Care Med. 2008;36(5):1641-2. doi:10.1097/CCM.0b013e3181701481.
7. Levey AS, De Jong PE, Coresh J, Nahas M El, Astor BC, Matsushita K, et al. The definition, classification, and prognosis of chronic kidney disease: A KDIGO Controversies Conference report. Kidney Int. 2011;80(1):17-28. doi:10.1038/ki.2010.483.
8. Mayer T, Bolliger D, Scholz M, Reuthebuch O, Gregor M, Meier P, et al. Urine Biomarkers of Tubular Renal Cell Damage for the Prediction of Acute Kidney Injury After Cardiac Sur- gery—A Pilot Study. J Cardiothorac Vasc Anesth. 2017;31(6):2072-9. doi:10.1053/j.jvca.2017.04.024.
9. Vandenberghe W, De Loor J, Hoste EAJ. Diagnosis of cardiac surgery-associated acute kid- ney injury from functional to damage biomarkers. Curr Opin Anaesthesiol. 2016;30(1):1. doi:10.1097/ACO.0000000000000419.

To reduce the incidence of AKI throughout the perioperative period, it is necessary to avoid intravascular volume depletion, optimize cardiac output with strict monitoring, and avoid administering nephrotoxic drugs,

avoid blood glucose levels > 180 mg/dl and large fluctuations through control and early administration of insulin[1]. During ECC, ultrafiltration with zero balance should be used in patients with glomerular filtration <60 ml/min[2], maintain DO2> 300 ml O2/min/m2 and avoid administering (or reduce the administration) vasopressors if the patient has mean blood pressure >70 mmHg3.

The PREVaki[4] trial describes a series of measures (KDIGO CT surgery bundle) consisting of: avoid nephrotoxic agents, interruption of ACE inhibitors and ARBs in the first 48 h after surgery, close monitoring of Cr and urinary output, avoid hyperglycemia (> 180 mg / dl) during the first 72 h after surgery, consider alternatives to contrast in radiodiagnosis, monitoring with a PICCO® catheter or similar with an optimization of intravascular volume and hemodynamic parameters according to a specific algorithm. This algorithm included a cardiac index>3 ml/min as a hemodynamic objective, consequently a greater use of dobutamine (9% vs 31%) was observed in the intervention group, with a lower prevalence of AKI. The application of these measures allows, therefore, to reduce the frequency and severity of AKI after cardiac surgery.

In the case of a KDIGO stage 2 AKI diagnosis (diuresis <0.5 ml / Kg / h for more than 12 h or Cr twice the baseline), early initiation (in less than 8 h) of renal replacement therapy has been shown to improve the results [6].

16. We recommend the application of the package of renal protection measures of the PREVaki protocol to prevent AKI associated with cardiac surgery.

Moderate level of evidence. Strong recommendation.

REFERENCES

10. Bansal B, Carvalho P, Mehta Y, Yadav J, Sharma P, Mithal A, et al. Prognostic significance of glycemic variability after cardiac surgery. J Diabetes Complications. 2016;30(4):613-7. doi:10.1016/j.jdiacomp.2016.02.010.
11. Matata BM, Scawn N, Morgan M, Shirley S, Kemp I, Richards S, et al. A Single-Center Randomized Trial of Intraoperative Zero-Balanced Ultrafiltration during Cardiopulmonary Bypass for Patients with Impaired Kidney Function Undergoing Cardiac Surgery. J Cardiothorac Vasc Anesth. 2015;29(5):1236-47. doi:10.1053/j.jvca.2015.02.020.
12. Magruder JT, Crawford TC, Harness HL, Grimm JC, Suarez-Pierre A, Wierschke C, et al. A pilot goal-directed perfusion initiative is associated with less acute kidney injury after cardiac surgery. J Thorac Cardiovasc Surg. 2017;153(1):118-125.e1. doi:10.1016/j.jtcvs.2016.09.016.
13. Meersch M, Schmidt C, Hoffmeier A, Van Aken H, Wempe C, Gerss J, et al. Prevention of cardiac surgery-associated AKI by implementing the KDIGO guidelines in high risk patients identified by biomarkers: the PrevAKI randomized controlled trial. Intensive Care Med. 2017;43(11):1551-61. doi:10.1007/s00134-016-4670-3.
14. Gregory AJ, Grant MC, Manning MW, Cheung AT, Ender J, Sander M, et al. Enhanced Recovery After Cardiac Surgery (ERAS Cardiac) Recommendations: An Important First Step—But There Is Much Work to Be Done. J Cardiothorac Vasc Anesth. 2019;00:1-9. doi:10.1053/j.jvca.2019.09.002.
15. Zarbock A, Kellum JA, Schmidt C, Van Aken H, Wempe C, Pavenstädt H, et al. Effect of Early vs Delayed Initiation of Renal Replacement Therapy on Mortality in Critically Ill Patients With Acute Kidney Injury. JAMA. 2016 May 24;315(20):2190. doi:10.1001/jama.2016.5828

Delirium prevention and early detection

Delirium is a clinical syndrome consisting of an alteration of consciousness, associated with a deficit of attention and perception with disorganized thinking, which can lead to a global cognitive disorder. It appears - depending on the age group – in between 11.5 and 80% of patients who undergo cardiac surgery[1-3].

Increases postoperative complications, duration of mechanical ventilation, morbidity, mortality, and has been associated with long-term cognitive changes[4,5].

It is multifactorial (pain, hypoxemia, low output, sepsis), therefore it requires an interdisciplinary team approach for prevention, diagnosis, risk stratification and treatment. Early detection is essential to determine the underlying cause and start appropriate treatment[6].

The most widely used current prediction scales are the "Confusion Assessment Method" or CAM-ICU and the "Intensive Care Delirium Screening Checklist" or ICDSC7. The ICDSC is validated by the DSM-IV-TR for the evaluation of delirium, and it is the scale that currently presents higher specificity and positive predictive value, making it a useful tool for its correct identification[2].

17. Systematic detection of delirium using validated scales (ICDSC) is recommended in the postoperative period of cardiac surgery at least once per nursing shift for early detection.

Moderate level of evidence. Strong recommendation.

REFERENCES

1. McPherson JA, Wagner CE, Boehm LM, Hall JD, Johnson DC, Miller LR, et al. Delirium in the cardiovascular ICU: exploring modifiable risk factors.Crit Care Med. 2013; 41(2): 405-13Järvelä K, Porkkala H, Karlsson S, Martikainen T, Selander T, Bendel S. Postoperati- ve Delirium in Cardiac Surgery Patients. J Cardiothorac Vasc Anesth. 2018;32(4):1597- 602. doi:10.1053/j.jvca.2017.12.030
2. EDA and ADS. The DSM-5 criteria, level of arousal and delirium diagnosis: inclusiveness is safer (European Delirium Association and American Delirium Society). BMC Med. 2014;12(1):141. doi:10.1186/s12916-014-0141-2
3. Krzych ŁJ, Wybraniec MT, Krupka-Matuszczyk I, Skrzypek M, Bolkowska A, Wilczyński M, et al. Detailed insight into the impact of postoperative neuropsychiatric complications on mortality in a cohort of cardiac surgery subjects: A 23,000-patient-year analysis. J Car- diothorac Vasc Anesth. 2014;28(3):448-57. doi:10.1053/j.jvca.2013.05.005
4. Abelha FJ, Luís C, Veiga D, Parente D, Fernandes V, Santos P, et al. Outcome and quality of life in patients with postoperative delirium during an ICU stay following major surgery. Crit Care. 2013;17(5):R257. doi:10.1186/cc13084
5. Maldonado JR. Neuropathogenesis of delirium: Review of current etiologic theories and common pathways. Am J Geriatr Psychiatry. 2013;21(12):1190-222. doi:10.1016/j.jagp. 2013.09.005
6. Bergeron N, Dubois MJ, Dumont M, Dial S, Skrobik Y. Intensive care delirium screening checklist: Evaluation of a new screening tool. Intensive Care Med. 2001;27(5):859-64. doi:10.1007/s001340100909

The prevention of delirium through non-pharmacological strategies such as early mobilization, pain management, minimization, and targeted titration of sedation, avoid the use of

benzodiazepines, reorientation of the patient, cognitive stimulation, reduction of hearing and / or visual impairment (for example, allowing the use of devices such as hearing aids or glasses), use of clocks / calendars and the promotion of the normal circadian sleep-wake pattern have shown promising results[1,2].

Pharmacological prevention with haloperidol or ketamine has not shown clinical benefits in large-scale clinical trials[3,4]. Evidence suggests that the use of atypical antipsychotics, haloperidol, or a statin does not affect the duration of delirium or its related morbidity[5]. There are encouraging data on the use of alpha-2 adrenergic agonists, such as dexmedetomidine in the prevention of its appearance[1].

18. If there are risk factors for the development of postoperative delirium or if its appearance is detected, it is recommended to administer dexmedetomidine at low doses (0.2 μg / kg / h).

Moderate level of evidence. Strong recommendation.

REFERENCES

7. Skrobik Y, Duprey MS, Hill NS, Devlin JW. Low-dose nocturnal dexmedetomidine prevents ICU delirium a randomized, placebo-controlled trial. Am J Respir Crit Care Med. 2018; 197(9):1147-56. doi:10.1164/rccm.201710-1995OC.

8. Blair GJ, Mehmood T, Rudnick M, Kuschner WG, Barr J. Nonpharmacologic and Medication Minimization Strategies for the Prevention and Treatment of ICU Delirium: A Narrative Review. J Intensive Care Med. 2019;34(3):183-90. doi:10.1177/0885066618771528.

9. Avidan; Hannah M. R. MaybrierArbi Ben Abdallah; Jacobsohn E; Vlisides PE. PKO; Veseli. RA. Intraoperative ketamine does not affect postoperative delirium or pain after major surgery in older adults: an international, multicentre, double-blind, randomised clinical trial. Lancet. 2017;390:267-75. doi:10.1016/S0140-6736(17)31467-8.

10. Van Den Boogaard M, Slooter AJC, Brüggemann RJM, Schoonhoven L, Beishuizen A, Vermeijden JW, et al. Effect of haloperidol on survival among critically ill adults with a high risk of delirium the REDUCE randomized clinical trial. JAMA. 2018;319(7):680-90. doi:10. 1001/jama.2018.0160.

11. Abelha FJ, Luís C, Veiga D, Parente D, Fernandes V, Santos P, et al. Outcome and quality of life in patients with postoperative delirium during an ICU stay following major surgery. Crit Care. 2013;17(5):R257. doi:10.1186/cc13084.

Duration of antibiotic prophylaxis

After the intervention, antibiotic prophylaxis will be administered during the first 24-48 h after surgery. It should not be prolonged more than 48 hours to avoid the risk of antibiotic resistance or causing infection by Clostridium difficile[1-4]. It is never justified to link the duration of prophylaxis to the remaining of catheters, tubes, or chest drains[3].

19. In the immediate postoperative period of cardiac surgery, antibiotic prophylaxis is recommended for the first 24-48 h.

Moderate level of evidence. Strong recommendation.

REFERENCES

12. Gelijns AC, Moskowitz AJ, Acker MA, Argenziano M, Geller NL, Puskas JD, et al. Management practices and major infections after cardiac surgery. J Am Coll Cardiol. 2014;64(4):372-381.

13. Lador A, Nasir H, Mansur N, Sharoni E, Biderman P, Leibovici L, et al. Antibiotic prophylaxis in cardiac surgery: systematic review and meta-analysis. J Antimicrob Chemother. 2012; 67(3):541-50.
14. Edwards FH, Engelman RM, Houck P, Shahian DM, Bridges CR. The Society of Thoracic Surgeons Practice Guideline Series: Antibiotic Prophylaxis in Cardiac Surgery, Part I: Dura- tion. Ann Thorac Surg. 2006;81(1):397-404.
15. Poeran J, Mazumdar M, Rasul R, Meyer J, Sacks HS, Koll BS, et al. Antibiotic prophylaxis and risk of Clostridium difficile infection after coronary artery bypass graft surgery. J Thorac Car- diovasc Surg. 2016;151(2):589-597.e2.

7.2.3 TORATHIC SURGERY

Manejo de los tubos de drenaje torácico y la aspiración tras resección pulmonar mayor

INTRODUCTION

Management of chest drainage tubes and aspiration after major lung resection

INTRODUCTION

The management of pleural drainage tubes (PDT) influences their duration, hospital stay, health costs, intensity of postoperative pain and respiratory function[1]. For this reason, they represent one of the cornerstones on which the ERAS programs in thoracic surgery are based.

It has been seen that when PDTs are removed with a serum-hematic output below 450 mL/day after thoracotomy, readmissions for recurrent pleural effusion are 0.55%, while with a threshold of 500mL/day, the incidence is 2.8% [2].

Currently, the use of a single PDT is recommended, instead of two, since it decreases the intensity of pain and shortens the duration of PDT[3] without compromising patient safety if there is no significant risk of bleeding, or a residual space problem is anticipated.

The use of digital systems to measure air leakage allows objective decisions to be made to choose when to remove the PDT, they are also easily transportable by the patient and have their own suction system, which facilitates the mobilization of the patient in the first post-operative days. The routine implementation of digital systems implies an increase in costs, and there are discrepancies between the studies regarding the improvement of the results (reduction in hospital stay and the duration of postoperative PDT) [4].

1. It is considered safe to remove the pleural drainage with a daily serum-hematic output of up to 450 ml.

   Moderate level of evidence. Strong recommendation.

2. The use of a single pleural drain after a standard regulated lung resection attenuates the intensity of postoperative pain, without negatively affecting clinical safety.

   Moderate level of evidence. Strong recommendation.

3. The use of digital systems is suggested to shorten the time of the PDT and the hospital stay.

   Low level of evidence. Weak recommendation.

REFERENCES

1. Refai M, Brunelli A, Salati M, Xiumè F, Pompili C, Sabbatini A. The impact of chest tube removal on pain and pulmonary function after pulmonary resection. Eur J Cardiothorac Surg. 2012;41:820-2.
2. Gao S, Zhang Z, Aragón J, Brunelli A, Cassivi S, Chai Y et al. The Society for Translational Medicine: clinical practice guidelines for the postoperative management of chest tube for patients undergoing lobectomy. J Thorac Dis. 2017; 9:3255-64.
3. Zhang X, Lv D, Li M, Sun G, Liu C. The single chest tube versus double chest tube application after pulmonary lobectomy: A systematic review and meta-analysis. J Cancer Res Ther. 2016;12(Supplement):C309-C316.
4. Aldaghlawi F, Kurman JS, Lilly JA, Hogarth DK, Donington J, Ferguson MK, Murgu SD. A Systematic Review of Digital vs Analog Drainage for Air Leak After Surgical Resection or Spontaneous Pneumothorax. Chest. 2020 Jan 17. pii: S0012-3692(20)30029-5.

Prophylaxis of post-thoracotomy postoperative atrial fibrillation (AF)

Thoracic surgery is associated with a high incidence of this arrhythmia, reaching 24% with an OR of 9.2 (95% CI 6.7-13) compared to other non-cardiac surgeries[1]. Furthermore, the risk is increased in major resections (pneumonectomies) compared to lobectomies or wedge lung resections [2].

Preoperative administration of amiodarone, calcium channel blockers, colchicine, statins, or magnesium may be effective in reducing the risk of AF in the postoperative period of thoracic surgery, but at present time, there is not enough information to determine in what type of patients the benefits would outweigh the risks of implementing this preoperative prophylactic measure in thoracic surgery[3,4].

Beta adrenergic receptor (BB) blocking drugs are also effective in preventing AF in thoracic surgery, but there are doubts about this strategy due to the side effects of bronchospasm and hypotension. However, there is a consensus that in patients who were previously under treatment with BB, they should not be suspended before the intervention, since a rebound phenomenon can be triggered, increasing the incidence of arrhythmias and hypertension, therefore, it is recommended to continue administering them during the perioperative period.

4. Prophylactic administration of beta-blockers, magnesium, amiodarone, calcium antagonists, statins, or colchicine reduces the probability of developing postoperative AF.

   Moderate level of evidence. Weak recommendation.

5. It is suggested to replace magnesium intravenously when the levels are low.

   Low level of evidence. Weak recommendation.

6. It is recommended to continue administering beta-blockers if the patient is being treated with them previously.

   High level of evidence. Strong recommendation.

REFERENCES

1. Polanczyk CA, Goldman L, Marcantonio ER, Orav EJ, Lee TH. Supraventricular arrhythmia in patients having noncardiac surgery: Clinical correlates and effect on length of stay. Ann Intern Med.1998;129(4):279-85.

2. Onaitis M, D'Amico T, Zhao Y, O'Brien S, Harpole D. Risk factors for atrial fibrillation after lung cancer surgery: Analysis of the society of thoracic surgeons general thoracic surgery database. Ann Thorac Surg. 2010;90(2):368-74.
3. Frendl G, Sodickson AC, Chung MK, Waldo AL, Gersh BJ, Tisdale JE, et al. 2014 AATS guidelines for the prevention and management of perioperative atrial fibrillation and flutter for thoracic surgical procedures. J Thorac Cardiovasc Surg. 2014; 148:153-93.
4. Zhang L, Gao S. Systematic Review and Meta-analysis of Atrial Fibrillation Prophylaxis After Lung Surgery. J Cardiovasc Pharmacol. 2016; 67:351-7.

Thoracic cavity approach

Minimally invasive thoracic surgery has significantly reduced pain and perioperative morbidity associated with conventional thoracotomy surgery[1]. Several meta-analyses have shown that the VATS approach is better than thoracotomy in reducing the intensity of postoperative pain, perioperative complications, hospital stay, duration of chest drains and quality of life[2,3,4].

In cases where VATS is not performed, to minimize aggression, thoracotomy without muscle section -muscle sparing- has been advised, protection of the intercostal nerves, management of the rib separator and rib closure or re-approximation or intracostal sutures have been recommended. since these techniques reduce post-thoracotomy pain[5,6,7].

7. The thoracoscopic approach for the treatment of early-stage non-small cell lung cancer is preferable to the classical thoracotomy.

   High level of evidence. Strong recommendation.

8. A thoracotomy without muscle section is recommended in cases where the VATS approach is not feasible.

   Moderate level of evidence. Strong recommendation.

9. In these cases, it is recommended to add intercostal nerve preservation techniques including intercostal muscle flaps and intracostal sutures.

   Moderate level of evidence. Strong recommendation.

REFERENCES

1. Cattaneo SM, Park BJ, Wilton AS, et al. Use of video-assisted thoracic surgery for lobectomy in the elderly results in fewer complications. Ann Thorac Surg. 2008; 85:235-6.
2. Yan TD, Black D, Bannon PG, et al. Systematic review and meta-analysis of randomized and nonrandomized trials on safety and efficacy of video-assisted thoracic surgery lobectomy for early-stage non-small-cell lung cancer. J Clin Oncol. 2009; 27:2553-62.
3. Zhang Z, Zhang Y, Feng H, Yao Z, Teng J, Wei D, Liu D. Is video-assisted thoracic surgery lobectomy better than thoracotomy for early-stage non-small-cell lung cancer? A systematic review and meta- analysis. Eur J Cardiothorac Surg. 2013; 44:407-14.
4. Cai YX, Fu XN, Xu QZ, Sun W, Zhang N. Thoracoscopic lobectomy versus open lobectomy in stage I non-small cell lung cancer: a meta-analysis. PLoS One. 2013;8:e82366.
5. Bayram SA, Ozcan M, Kaya F.N., Gebitekin C. Rib approximation without intercostal nerve compression reduces post-thoracotomy pain: a prospective randomized study. Eur J Cardiothorac Surg. 2011;39: 570-4.

6. Li S, Feng Z, Wu L, et al. Analysis of 11 trials comparing muscle-sparing with posterolateral thoracotomy. Thorac Cardiovasc Surg. 2014; 62:344-52.
7. Visagan R, McCormack DJ, Shipolini AR, Jarral OA. Are intracostal sutures better than pericostal sutures for closing a thoracotomy Interact Cardiovasc Thorac Surg. 2012; 14: 807-15.

7.2.4 BURNS

Preoperative and postoperative rehabilitation

INTRODUCTION

Rehabilitation in the burned patient should begin early, progressively and with no interruptions[1]. Its planning and execution must begin at the same time as the initial assessment of the patient and must be individualized, since the objectives of the rehabilitative treatment will vary throughout the evolution of the injuries[2].

In various studies, better functional outcomes have been reported in the groups that received intensive rehabilitation protocols, which included active, passive, and postural therapy applied from the onset of the burn in selected cases susceptible to develop scar contractions (deep burns, involvement of joint areas and face)[1-6].

1. In patients who suffer burns, especially in cases of deep burns that affect joint areas or the face, rehabilitation should begin from the initial moment of the burn, limiting postoperative immobilization periods.

Moderate level of evidence. Strong recommendation.

REFERENCES

1. Retrouvey H, Wang A, Corkum J, Shahrokhi S. The Impact of Time of Mobilization After Split Thickness Skin Graft on Lower Extremity Wound Healing-Systematic Review and Meta-analysis. J Burn Care Res. 2018 Oct 23;39(6):902-910.
2. Richard R, Baryza MJ, Carr JA, Dewey WS, Dougherty ME, Forbes-Duchart L, et al. Burn rehabilitation and research: proceedings of a consensus summit. J Burn Care Res. 2009;30 (4):543-73.
3. Jacobson K, Fletchall S, Dodd H, Starnes C. Current Concepts Burn Rehabilitation, Part I: Care During Hospitalization. Clin Plast Surg. 2017 Oct;44(4):703-712.
4. Dodd H, Fletchall S, Starnes C, Jacobson K. Current Concepts Burn Rehabilitation, Part II: Long-Term Recovery. Clin Plast Surg. 2017 Oct;44(4):713-728.
5. Anzarut A, Olson J, Singh P, Rowe BH, Tredget EE. The effectiveness of pressure garment therapy for the prevention of abnormal scarring after burn injury: a meta-analysis. J Plast Reconstr Aesthet Surg. 2009;62(1):77-84.
6. Okhovatian F, Zoubine N. A comparison between two burn rehabilitation protocols. Burns. 2007;33(4):429-34.

Management of skin graft donor areas

There is a wide variety of dressings available for use in partial skin graft donor sites[1]. In these areas, spontaneous epithelialization is expected in most of

cases[1]. Dressings that induce a humid environment in the healing bed of these wounds are the most effective since they promote and accelerate the healing of graft donor areas.[1-4]

Within these, hydrocolloid dressings are the first choice for cure, given the low rate of associated infection they present, the rapid epithelialization and the reduction of pain (measured according to the Visual Analogue Scale)[2-4].

2. Hydrocolloid dressings are the first choice for healing partial skin graft sites, given the expected spontaneous epithelialization, the low infection rates associated with their use, and the lower patient-referred pain compared to other commonly used dressings.

Moderate level of evidence. Strong recommendation.

REFERENCES

1. Brown JE, Holloway SL. An evidence-based review of split-thickness skin graft donor site dressings. Int Wound J. 2018 Dec;15(6):1000-1009.
2. Demirtas Y, Yagmur C, Soylemez F, Ozturk N, Demir A. Management of split-thickness skin graft donor site: a prospective clinical trial for comparison of five different dressing materials. Burns. 2010 Nov;36(7):999-1005.
3. Brölmann FE, Eskes AM, Goslings JC, Niessen FB, de Bree R, Vahl AC, et al; REMBRANDT study group. Randomized clinical trial of donor-site wound dressings after split-skin grafting. Br J Surg. 2013 Apr;100(5):619-27.
4. Macharia M, Nangole FW. Effects of Dressing Materials on Donor Site Morbidity: A Comparative Study at a Tertiary Hospital in Kenya. Ann Plast Surg. 2019 Jul;83(1):22-25.

Nutrition start

Burns are a type of trauma characterized by the early development of a severe hypermetabolic response[1]. This response leads to the massive and accelerated consumption of macro and micronutrients, which promote and maintain the inflammatory response syndrome and the metabolic alterations that cause burns[2]. The resulting acute malnutrition increases the risk of infection as well[2]. Infection is the first cause of death in the severely burned patient (one with more than 20% of the burned body surface) and induces a delay in healing[3]. In fact, a relationship has been established between the total caloric and protein deficit and the probability of survival. The surgical interventions required in these patients also increase the risk of malnutrition[1-3].

For this reason, and due to the benefits of the early restart of feeding after surgery (maintenance of the intestinal mucosa, reduction of bacterial translocation, stimulation of intestinal lymphoid tissue ...), it is recommended to restart nutrition as soon as possible, generally no later than 3 hours after surgery[4-5]. Additionally, given the clear benefits of enteral nutrition over parenteral nutrition, enteral nutrition is the first choice, except in those cases in which it is contraindicated (e.g., paralytic ileus)[2-6].

3. In patients suffering from burns, an early start of nutrition is recommended after surgery. In these patients, enteral nutrition is the first choice, so parenteral nutrition is reserved for cases in which the former is contraindicated.

High level of evidence. Strong recommendation.

REFERENCES

1. Moreira E, Burghi G, Manzanares W. Update on metabolism and nutrition therapy in critically ill burn patients. Med Intensiva. 2018 Jun-Jul;42(5):306-316.
2. Rousseau AF, Verbrugge AM, Fadeur M, Struvay A, Lefort H. Nutritional aspects of the management of the severely burned patient. Rev Infirm. 2019 Dec;68(256):28-29.
3. ISBI Practice Guidelines Committee; Steering Subcommittee; Advisory Subcommittee. ISBI Practice Guidelines for Burn Care. Burns. 2016 Aug;42(5):953-1021.
4. Chen Z, Wang S, Yu B, Li A. A comparison study between early enteral nutrition and parenteral nutrition in severe burn patients. Burns 2007;33:708-12.
5. Dylewksi ML, Baker M, Prelack K, Weber JM, Hursey D, Lydon M, et al. The safety and efficacy of parenteral nutrition among pediatric patients with burn injuries. Pediatr Crit Care Med 2013;14:e120-5.
6. Wasiak J, Cleland H, Jeffery R. Early versus late enteral nutritional support in adults with burn injury: a systematic review. J Hum Nutr Diet. 2007 Apr;20(2):75-83.

Operating room temperature

In adult patients, maintaining a room temperature of at least 21 ° C1 is generally recommended. However, burn patients have greater susceptibility for hypothermia due to loss of skin coverage (implicated in body thermoregulation mechanisms)[1-3].

Furthermore, sustained hypothermia in these patients may increase the hypermetabolic response presented by the burn itself. For this reason, the recommended room temperature for burned patients is higher (between 28 ° C and 32 ° C)[1-3].

4. The ambient temperature in the operating room of adult patients suffering from burns (mainly those with extensive affected surfaces) should be between 28 ° C and 32 ° C.

   Nivel de evidencia moderado. Grado de recomendación fuerte.

REFERENCES

1. Kelemen JJ III, Cioffi WG Jr, Mason AD Jr, Mozingo DW, McManus WF, Pruitt BA Jr. Effect of ambient temperature on metabolic rate after thermal injury. Ann Surg 1996;223:406-12.
2. Wilmore DW, Mason AD Jr, Johnson DW, Pruitt BA Jr. Effect of ambient temperature on heat production and heat loss in burn patients. J Appl Physiol 1975;38:593-7.
3. Williams FN, Herndon DN, Jeschke MG. The hypermetabolic response to burn injury and interventions to modify this response. Clin Plast Surg 2009;36:583-96.

7.2.5 COLORECTAL SURGERY

Preparación mecánica de colon en cirugía colorrectal

Colon mechanical preparation in colorectal surgery

In the previous RICA guidelines, the non-mechanical preparation of the colon was recommended in colorectal surgery, except for when rectal surgery with a derivative stoma is to be performed, as well as when an intraoperative colonoscopy is to be performed.

In recent years, numerous publications based on the database of the American College of Surgeons National Surgical Quality Improvement Program (ACS NSQIP®)[1,2]

have again sowed doubts about their use. This information is based on retrospective studies with numerous flaws and raises the possible existence of important bias[3]. In any case, it should not be underestimated, given the high number of patients included.

In a recent meta-analysis studying the effect of mechanical preparation vs. no bowel preparation[4] and a Finnish multicenter randomized study[5], both concluded that mechanical bowel preparation does not affect the incidence of postoperative complications or surgical site infection.

1. Mechanical colon prep does not improve results, may cause dehydration, and should not be used routinely in colon surgery. It can offer benefits in rectal surgery with anastomosis.

High level of evidence. Strong recommendation.

REFERENCES

1. Klinger, AL, Green, H, Monlezun, DJ, Beck, D, Kann, B., Vargas, HD, et al. The Role of Bowel Preparation in Colorectal Surgery. Annals of Surgery. 2019;269(4), 671-7.
2. Zorbas KA, Yu D, Choudhry A, Ross HM, Philp M. Preoperative bowel preparation does not favor the management of colorectal anastomotic leak. World J Gastrointest Surg. 2019;11(4):218-28.
3. Beyer-Berjot, L, Slim, K. Colorectal surgery and preoperative bowel preparation: aren't we drawing hasty conclusions? Colorectal Disease.2018; 20(11):955-8.
4. Rollins, KE, Javanmard-Emamghissi H, Lobo, DN. Impact of mechanical bowel preparation in Elective Colorectal Surgery: A Meta-analysis. World J gastroenterol. 2018;24(4):519- 536.
5. Koskenvuo L, Lehtonen T, Koskensalo S, et al. Mechanical and oral antibiotic bowel prepa- ration versus no bowel preparation for elective colectomy (MOBILE): a multicentre, rando- mised, parallel, single-blinded trial. The Lancet. 2019; 394(10201):840-8.

7.2.6 MAJOR HEAD AND NECK SURGERY

Postoperative antibiotic prophylaxis in head and neck surgery with free flaps

In clean-contaminated head and neck surgery, in which controlled opening of the aerodigestive tract is performed, prolonging antibiotic prophylaxis for more than 24 hours has not been shown to provide advantages[1]. However, when free flaps are used, the risk of infection is greater and it is recommended to prolong the administration of antibiotics for 48h[2,3]

1. Short-term antibiotic prophylaxis (less than 3 days) is recommended, with broad spectrum antibiotics covering Gram+, Gram- and anaerobes in microsurgical reconstruction procedures.

High level of evidence. Strong recommendation.

REFERENCES

1. Chiesa-Estomba CM, Lechien JR, Fakhry N, Melkane A, Calvo-Henriquez C, de Siati D, Gonzalez-Garcia JA, Fagan JJ, Ayad T. Systematic review of international guidelines for pe- rioperative antibiotic prophylaxis in Head & Neck Surgery. A YO-IFOS Head & Neck Study Group Position Paper. Head Neck. 2019 Sep;41(9):3434-3456.

2. Haidar YM, Tripathi PB, Tjoa T, Walia S, Zhang L, Chen Y, Nguyen DV, Mahboubi H, Armstrong WB, Goddard JA. Antibiotic prophylaxis in clean-contaminated head and neck cases with microvascular free flap reconstruction: A systematic review and meta-analysis. Head Neck. 2018 Feb;40(2):417-427.
3. Patel PN, Jayawardena ADL, Walden RL, Penn EB, Francis DO. Evidence-Based Use of Perioperative Antibiotics in Otolaryngology. Otolaryngol Head Neck Surg. 2018 May;158(5): 783-800.

Routine postoperative admission to Intensive Care Units

The routine post-operative admission of patients who underwent oncological head and neck surgery procedures in Intensive Care Units during the first 24-48 hours is unnecessary in most cases[1-3] and increases costs and hospital stay[2].

Most patients can be treated in wards with specialized nursing staff, with subsequent transfer to the ICU being infrequent[3].

2. Transfer patients undergoing head and neck Oncological surgery to wards with nursing personnel specialized in otolaryngology is recommended, avoiding admission to the ICU.

Moderate level of evidence. Strong recommendation.

REFERENCES

1. Yu PK, Sethi RKV, Rathi V, et al. Postoperative care in an intermediate-level medical unit after head and neck microvascular free flap reconstruction. Laryngoscope Investig Otolaryngol. 2018;4(1):39-42.
2. Varadarajan VV, Arshad H, Dziegielewski PT. Head and neck free flap reconstruction: What is the appropriate post-operative level of care?. Oral Oncol. 2017;75:61-66.
3. Panwar A, Smith R, Lydiatt D, et al. Vascularized tissue transfer in head and neck surgery: Is intensive care unit-based management necessary?. Laryngoscope. 2016;126(1):73-79.

Postoperative monitoring of free flaps

The free flaps used in the reconstruction of head and neck surgeries should be monitored every hour during the first 24 hours after surgery[1]. Monitoring should continue throughout the hospital stay, although the frequency decreasing progressively[2].

Monitoring should include at least a clinical examination by experienced personnel[3]. The use of other monitoring techniques should be considered, especially in those flaps that are not accessible to direct clinical examination[3,4].

3. Free flaps used in reconstruction of head and neck cancer surgeries should be closely monitored postoperatively, at least by direct clinical examination.

Moderate level of evidence. Strong recommendation.

REFERENCES

1. Kääriäinen M, Halme E, Laranne J. Modern postoperative monitoring of free flaps. Curr Opin Otolaryngol Head Neck Surg. 2018;26(4):248-253.

2. Disa JJ, Cordeiro PG, Hidalgo DA. Efficacy of conventional monitoring techniques in free tissue transfer: an 11-year experience in 750 consecutive cases.Plast Reconstr Surg. 1999;104:97-101.
3. Chae MP, Rozen WM, Whitaker IS, et al. Current evidence for postoperative monitoring of microvascular free flaps: a systematic review. Ann Plast Surg. 2015;74(5):621-632.
4. Kohlert S, Quimby AE, Saman M, Ducic Y. Postoperative Free-Flap Monitoring Techniques. Semin Plast Surg. 2019;33(1):13-16.

Post-operative wound care

The use of VAC (Vacuum Assisted Closure) therapy is recommended for the treatment of complex surgical wounds after major head and neck surgery[1], especially in patients with salivary fistula, multiple pathologies, previously irradiated or in those with dead spaces that favor development of infection[2,3]. Although its use can be considered in the closure of the free flap donor site, in this case it does not seem to provide appreciable advantages[4].

The use of occlusive dressings, such as polyurethane or hydrocolloid dressings, reduces pain and favors healing in skin graft donor areas[5].

4. The use of VAC (Vacuum Assisted Closure) therapy is recommended in complex surgical wounds during the postoperative period of major head and neck surgery whenever possible. In general, the use of VAC in free flap donor sites is not considered advantageous.

Moderate level of evidence. Strong recommendation.

REFERENCES

1. Mir A, Guys N, Arianpour K, et al. Negative Pressure Wound Therapy in the Head and Neck: An Evidence-Based Approach. Laryngoscope. 2019;129(3):671-683. doi:10.1002/lary.27262.
2. Yang YH, Jeng SF, Hsieh CH, Feng GM, Chen CC. Vacuum-assisted closure for complicated wounds in head and neck region after reconstruction. J Plast Reconstr Aesthet Surg. 2013;66(8):e209-e216. doi:10.1016/j.bjps.2013.03.006.
3. Maleki Delarestaghi M, Ahmadi A, Dehghani Firouzabadi F, Roomiani M, Dehghani Firouzabadi M, Faham Z. Effect of Low-Pressure Drainage Suction on Pharyngocutaneous Fistula After Total Laryngectomy [published online ahead of print, 2020 Jun 29]. Ann Otol Rhinol Laryngol. 2020.
4. Halama D, Dreilich R, Lethaus B, Bartella A, Pausch NC. Donor-site morbidity after harvesting of radial forearm free flaps-comparison of vacuum-assisted closure with conventional wound care: A randomized controlled trial. J Craniomaxillofac Surg. 2019;47(12):1980-1985. doi:10.1016/j.jcms.2019.11.004.
5. Serebrakian AT, Pickrell BB, Varon DE, et al. Meta-analysis and Systematic Review of Skin Graft Donor-site Dressings with Future Guidelines. Plast Reconstr Surg Glob Open. 2018;6(9):e1928.

Tracheostomy management

Given that performing a tracheostomy is associated with a longer hospital stay, it is recommended to avoid it whenever it is safely to do so[1,2]. In some patients, it can be replaced by a 24-48 period of orotracheal intubation[1,2]. When tracheostomy is done,

decannulation should be attempted as soon as possible. Surgical closure of the tracheostomy can accelerate the recovery of the patient[3].

5. It is recommended to perform tracheostomy only when it is essential, and if it is performed, proceed to remove it as soon as possible.

Moderate level of evidence. Weak recommendation

REFERENCES

1. Dort JC, Farwell DG, Findlay M, et al. Optimal Perioperative Care in Major Head and Neck Cancer Surgery With Free Flap Reconstruction: A Consensus Review and Recommendations From the Enhanced Recovery After Surgery Society. JAMA Otolaryngol Head Neck Surg. 2017;143(3):292-303.
2. Bater M, King W, Teare J, D'Souza J. Enhanced recovery in patients having free tissue transfer for head and neck cancer: does it make a difference?. Br J Oral Maxillofac Surg. 2017; 55(10):1024-1029.
3. Brookes JT, Seikaly H, Diamond C, Mechor B, Harris JR. Prospective randomized trial comparing the effect of early suturing of tracheostomy sites on postoperative patient swallowing and rehabilitation. J Otolaryngol. 2006;35(2):77-82.

Postoperative pulmonary physical therapy

Pulmonary complications are the most frequent in the postoperative period of major head and neck surgery, with a significant impact on mortality and hospital stay[1,2]. They are mainly associated with dysphagia and secondary aspiration to these interventions[3]. The role of early pulmonary physical therapy in the prevention of these complications after major head and neck surgery has been scarcely studied[4], so the indication to perform it is based on the extrapolation of the results it provides after interventions in other territories[5] and must be valued from this perspective.

6. Pulmonary physical rehabilitation during the postoperative period of major head and neck surgery has not been shown to play a relevant role in the prevention of the most frequent pulmonary complications after these interventions, and its usefulness is doubtful.

Low level of evidence. Weak recommendation.

REFERENCES

1. Bhattacharyya N, Fried MP. Benchmarks for mortality, morbidity, and length of stay for head and neck surgical procedures. Arch Otolaryngol Head Neck Surg. 2001;127(2):127-132.
2. Semenov YR, Starmer HM, Gourin CG. The effect of pneumonia on short-term outcomes and cost of care after head and neck cancer surgery. Laryngoscope. 2012;122(9):1994-2004.
3. Di Santo D, Bondi S, Giordano L, et al. Long-term Swallowing Function, Pulmonary Complications, and Quality of Life after Supracricoid Laryngectomy. Otolaryngol Head Neck Surg. 2019;161(2):307-314.
4. Genç A, Ikiz AO, Güneri EA, Günerli A. Effect of deep breathing exercises on oxygenation after major head and neck surgery. Otolaryngol Head Neck Surg. 2008;139(2):281-285.
5. Worrall DM, Tanella A, DeMaria S Jr, Miles BA. Anesthesia and Enhanced Recovery After Head and Neck Surgery. Otolaryngol Clin North Am. 2019;52(6):1095-1114.

7.2.7 TRAUMATOLOGY AND ORTHOPEDIC SURGERY

1. Knee and hip prosthetic surgery[1,2]

Hip and knee arthroplasties are effective surgical procedures that improve the quality of life of patients, increase their functional capacity and reduce pain. According to data from the Spanish Ministry of Health (RAE-CMBD), in 2018 more than 60,000 knee arthroplasties and more than 50,000 hip arthroplasties were performed in Spain[3], which generated almost half a million hospital stays, being the first and fourth most frequent surgical procedure in the National Health System. Therefore, they represent a significant volume of activity and waiting list problems. The aging of the population and the greater demands on active aging make it foreseeable that the indications for this surgery will increase significantly in the coming years. In addition to its high volume, ana significant variability in medical practice has been shown[4]. Its key points will be addressed now.

REFERENCES

1. Joint replacement (primary): hip, knee and shoulder [Internet]. London: National Institute for Health and Care Excellence (UK); 2020. Available from: http://www.ncbi.nlm.nih.gov/books/NBK561385/.
2. McGrory BJ, Weber KL, Jevsevar DS, Sevarino K. Surgical Management of Osteoarthritis of the Knee: Evidence-based Guideline. J Am Acad Orthop Surg. 2016 Aug;24(8):e87-93.
3. Consulta Interactiva del SNS [Internet]. Available from: https://pestadistico.inteligenciadegestion.mscbs.es/publicoSNS/S.
4. Molko S, Dasí-Sola M, Marco F, Combalia A. El proceso de atención de las artroplastias primarias totales de rodilla y cadera en España: un estudio a nivel nacional. Revista Española de Cirugía Ortopédica y Traumatología. 2019 Nov;63(6):408-15.

ERAS programs (enhanced recovery after surgery, enhanced recovery after surgery)

ERAS programs include interventions in the pre-operative, intraoperative, and post-operative stages to facilitate patient recovery, through multimodal or “fast-track” interventions, which globally modulate the systemic response to surgery.

Developed some time ago in abdominal or gynecological surgery, its ability to reduce the appearance of major complications in scheduled arthroplasties has recently been demonstrated[1-4].

1. The implementation of ERAS or “fast-track” programs is recommended in patients undergoing hip or knee arthroplasty.

Nivel de evidencia moderado. Grado de recomendación fuerte.

REFERENCES

1. Soffin EM, YaDeau JT. Enhanced recovery after surgery for primary hip and knee arthroplasty: a review of the evidence. Br J Anaesth. 2016;117(suppl 3):iii62-72.
2. Wainwright TW, Gill M, McDonald DA, Middleton RG, Reed M, Sahota O, et al. Consensus statement for perioperative care in total hip replacement and total knee replacement surgery: Enhanced Recovery After Surgery (ERAS®) Society recommendations. Acta Orthop. 2020;91(1):3-19.

3. Frassanito L, Vergari A, Nestorini R, Cerulli G, Placella G, Pace V, et al. Enhanced recovery after surgery (ERAS) in hip and knee replacement surgery: description of a multidisciplinary program to improve management of the patients undergoing major orthopedic surgery. Musculoskelet Surg. 2020 Apr;104(1):87-92.
4. Ripollés-Melchor J, Abad-Motos A, Díez-Remesal Y, Aseguinolaza-Pagola M, Padin-Barreiro L, Sánchez-Martín R, et al. Association Between Use of Enhanced Recovery After Surgery Protocol and Postoperative Complications in Total Hip and Knee Arthroplasty in the Postoperative Outcomes Within Enhanced Recovery After Surgery Protocol in Elective Total Hip and Knee Arthroplasty Study (POWER2). JAMA Surg. 2020 Feb 12;e196024.

Decision-making aids

Decision aids can be helpful in making the shared decision to start knee or hip replacement, increasing the efficiency of consultations and patient satisfaction. They can contribute to increase the degree of knowledge and participation of the patient about his pathology and its treatment. Several studies show a similar surgical indication rate, with no impact on costs, but improving patient satisfaction and the efficiency of preoperative visits[1-3].

2. Decision aids can improve patient awareness and participation about their process and be helpful in sharing the decision to start surgery.

Moderate level of evidence. Weak recommendation

REFERENCES

1. Stacey D, Taljaard M, Dervin G, Tugwell P, O'Connor AM, Pomey MP, et al. Impact of patient decision aids on appropriate and timely access to hip or knee arthroplasty for osteoarthritis: a randomized controlled trial. Osteoarthr Cartil. 2016 Jan;24(1):99-107.
2. Bozic KJ, Belkora J, Chan V, Youm J, Zhou T, Dupaix J, et al. Shared decision making in patients with osteoarthritis of the hip and knee: results of a randomized controlled trial. J Bone Joint Surg Am. 2013 Sep 18;95(18):1633-9.
3. Sepucha K, Atlas SJ, Chang Y, Dorrwachter J, Freiberg A, Mangla M, et al. Patient Decision Aids Improve Decision Quality and Patient Experience and Reduce Surgical Rates in Routine Orthopaedic Care: A Prospective Cohort Study. J Bone Joint Surg Am. 2017 Aug 2;99(15):1253-60.

Preoperative rehabilitation programs ("patient schools")

Pre-operative rehabilitation programs are group sessions that take place 2-6 weeks prior to the scheduled surgery, taught by healthcare professionals who will participate in the post-operative recovery, which include: information on the planned routes, counseling on strategies to improve recovery, teaching exercises in preparation for surgery; advice on techniques for managing activities of daily living and information on the use of adaptive equipment such as raised toilet seats, dressing aids, and walking aids such as walkers or crutches[1-3].

3. Pre-operative rehabilitation programs could reduce post-operative stay and improve early post-operative function.

Low level of evidence. Weak recommendation.

REFERENCES

1. Silkman Baker C, McKeon JM. Does preoperative rehabilitation improve patient-based outcomes in persons who have undergone total knee arthroplasty? A systematic review. PM R. 2012 Oct;4(10):756-67.

2. Wang L, Lee M, Zhang Z, Moodie J, Cheng D, Martin J. Does preoperative rehabilitation for patients planning to undergo joint replacement surgery improve outcomes? A systematic review and meta-analysis of randomised controlled trials. BMJ Open. 2016 Feb 2;6(2): e009857.

3. Huang S-W, Chen P-H, Chou Y-H. Effects of a preoperative simplified home rehabilitation education program on length of stay of total knee arthroplasty patients. Orthop Traumatol Surg Res. 2012 May;98(3):259-64.

Anesthesia and analgesia

It is indicated to use a combination of neuraxial anesthesia with or without regional blocks, and a multimodal protocol for postoperative analgesia that does not limit postoperative motor function or prolong hospital stay. Neuraxial anesthesia appears to reduce post-operative nausea and shorten hospital stay, compared to general anesthesia[1,2]. Peripheral blocks can reduce postoperative pain, prevent complications associated with the use of opiates, and improve early postoperative function[3-6].

4. Neuraxial anesthesia is recommended, combined with post-operative regional and/or multimodal anesthesia protocols.

Moderate level of evidence. Strong recommendation.

REFERENCES

1. Pu X, Sun J-M. General anesthesia vs spinal anesthesia for patients undergoing total-hip arthroplasty: A meta-analysis. Medicine (Baltimore). 2019 Apr;98(16):e14925.

2. Johnson RL, Kopp SL, Burkle CM, Duncan CM, Jacob AK, Erwin PJ, et al. Neuraxial vs general anaesthesia for total hip and total knee arthroplasty: a systematic review of comparative-effectiveness research. Br J Anaesth. 2016 Feb;116(2):163-76.

3. Chan E-Y, Fransen M, Parker DA, Assam PN, Chua N. Femoral nerve blocks for acute postoperative pain after knee replacement surgery. Cochrane Database Syst Rev. 2014 May 13;(5):CD009941.

4. Memtsoudis SG, Cozowicz C, Bekeris J, Bekere D, Liu J, Soffin EM, et al. Anaesthetic care of patients undergoing primary hip and knee arthroplasty: consensus recommendations from the International Consensus on Anaesthesia-Related Outcomes after Surgery group (ICAROS) based on a systematic review and meta-analysis. Br J Anaesth. 2019 Sep;123 (3):269-87.

5. Osinski T, Bekka S, Regnaux J-P, Fletcher D, Martinez V. Functional recovery after knee arthroplasty with regional analgesia: A systematic review and meta-analysis of randomised controlled trials. Eur J Anaesthesiol. 2019;36(6):418-26.

6. Opperer M, Danninger T, Stundner O, Memtsoudis SG. Perioperative outcomes and type of anesthesia in hip surgical patients: An evidence based review. World J Orthop. 2014 Jul 18;5(3):336-43.

Tranexamic acid to reduce blood loss.

Intravenous or oral tranexamic acid combined with topical (intra-articular) tranexamic acid reduces the number of necessary blood transfusions, being able to reduce the risk of postoperative bruising and infections, as well as being cost-efficient[1-5]. Dose adjustment is recommended in patients with impaired renal function.

5. The use of oral or intravenous tranexamic acid is recommended compared to topical to reduce perioperative bleeding.

High level of evidence. Strong recommendation.

REFERENCES

1. Xu S, Chen JY, Zheng Q, Lo NN, Chia S-L, Tay KJD, et al. The safest and most efficacious route of tranexamic acid administration in total joint arthroplasty: A systematic review and network meta-analysis. Thromb Res. 2019 Apr;176:61-6.
2. Wu Q, Zhang H-A, Liu S-L, Meng T, Zhou X, Wang P. Is tranexamic acid clinically effective and safe to prevent blood loss in total knee arthroplasty? A meta-analysis of 34 randomized controlled trials. Eur J Orthop Surg Traumatol. 2015 Apr;25(3):525-41.
3. Shin Y-S, Yoon J-R, Lee H-N, Park S-H, Lee D-H. Intravenous versus topical tranexamic acid administration in primary total knee arthroplasty: a meta-analysis. Knee Surg Sports Traumatol Arthrosc. 2017 Nov;25(11):3585-95.
4. Ma Q-M, Han G-S, Li B-W, Li X-J, Jiang T. Effectiveness and safety of the use of antifibrinolytic agents in total-knee arthroplasty: A meta-analysis. Medicine (Baltimore). 2020 May; 99(20):e20214.
5. Sun Q, Li J, Chen J, Zheng C, Liu C, Jia Y. Comparison of intravenous, topical or combined routes of tranexamic acid administration in patients undergoing total knee and hip arthroplasty: a meta-analysis of randomised controlled trials. BMJ Open. 2019 28;9(1):e024350.

Use of postoperative drains

Postoperative drains are used to reduce bruising and other complications in the surgical wound. There are postoperative drains that allow recovering the blood drained from the surgical wound, to reduce the need for transfusions. However, the routine use of drains seems to increase postoperative bleeding, without observing the benefits derived from their use[1-6]. Blood collectors may not be cost-effective in most patients, with adequate perioperative management of anemia[7,8].

6. It is not necessary to routinely use suction or blood recovery drains for hip and knee arthroplasties if adequate control of perioperative bleeding and anemia is performed.

High level of evidence. Strong recommendation.

REFERENCES:

1. Xu H, Xie J, Lei Y, Huang Q, Huang Z, Pei F. Closed suction drainage following routine primary total joint arthroplasty is associated with a higher transfusion rate and longer postoperative length of stay: a retrospective cohort study. J Orthop Surg Res. 2019 May 29; 14(1):163.
2. Parker MJ, Roberts CP, Hay D. Closed suction drainage for hip and knee arthroplasty. A meta-analysis. J Bone Joint Surg Am. 2004 Jun;86(6):1146-52.

3. Abolghasemian M, Huether TW, Soever LJ, Drexler M, MacDonald MP, Backstein DJ. The Use of a Closed-Suction Drain in Revision Knee Arthroplasty May Not Be Necessary: A Prospective Randomized Study. J Arthroplasty. 2016;31(7):1544-8.
4. Zhou X, Li J, Xiong Y, Jiang L, Li W, Wu L. Do we really need closed-suction drainage in total hip arthroplasty? A meta-analysis. Int Orthop. 2013 Nov;37(11):2109-18.
5. Watanabe T, Muneta T, Yagishita K, Hara K, Koga H, Sekiya I. Closed Suction Drainage Is Not Necessary for Total Knee Arthroplasty: A Prospective Study on Simultaneous Bilateral Surgeries of a Mean Follow-Up of 5.5 Years. J Arthroplasty. 2016 Mar;31(3):641-5.
6. Quinn M, Bowe A, Galvin R, Dawson P, O'Byrne J. The use of postoperative suction drainage in total knee arthroplasty: a systematic review. Int Orthop. 2015 Apr;39(4):653-8.
7. Benjamin JB, Colgan KM. Are Routine Blood Salvage/Preservation Measures Justified in All Patients Undergoing Primary TKA and THA? J Arthroplasty. 2015 Jun;30(6):955-8.
8. Dan M, Liu D, Martos SM, Beller E. Intra-operative blood salvage in total hip and knee arthroplasty. J Orthop Surg (Hong Kong). 2016;24(2):204-8.

Early post-operative physiotherapy

Early post-operative mobilization may reduce the risk of venous thromboembolic complications, in addition, it might reduce hospital stay and improve early postoperative function. To achieve this, adequate postoperative pain control is necessary[1-4].

7. Mobilization of the patient is recommended on the same day or the day after surgery.

High level of evidence. Strong recommendation.

REFERENCES

1. Karim A, Pulido L, Incavo S. Does Accelerated Physical Therapy After Elective Primary Hip and Knee Arthroplasty Facilitate Early Discharge? Am J Orthop. 2016 Oct;45(6):E337-42.
2. Bohl DD, Li J, Calkins TE, Darrith B, Edmiston TA, Nam D, et al. Physical Therapy on Postoperative Day Zero Following Total Knee Arthroplasty: A Randomized, Controlled Trial of 394 Patients. J Arthroplasty. 2019 Jul;34(7S):S173-S177.e1.
3. Harikesavan K, Chakravarty RD, Maiya AG. Influence of early mobilization program on pain, self-reported and performance based functional measures following total knee replacement. J Clin Orthop Trauma. 2019 Apr;10(2):340-4.
4. Temporiti F, Draghici I, Fusi S, Traverso F, Ruggeri R, Grappiolo G, et al. Does walking the day of total hip arthroplasty speed up functional independence? A non-randomized controlled study. Arch Physiother. 2020;10:8.

Postoperative outpatient rehabilitation

With the results reported by patients in hand, no significant difference has been demonstrated regarding quality of life, neither functional nor in terms of the rate of complications, when supervised group or individual rehabilitation are compared with self-directed rehabilitation by the patient[1-4]. Outpatient rehabilitation resources should be offered to patients with difficulty in performing basic activities of daily living, or if they have a functional impairment that justifies the need for physical therapy, or if they do not progress adequately with self-directed exercises, or if they present cognitive impairment.

8. It is not possible to establish a recommendation for or against postoperative outpatient rehabilitation, compared to other rehabilitation modalities.

Nivel de evidencia bajo. Grado de recomendación débil.

REFERENCES

1. Coulter C, Perriman DM, Neeman TM, Smith PN, Scarvell JM. Supervised or Unsupervised Rehabilitation After Total Hip Replacement Provides Similar Improvements for Patients: A Randomized Controlled Trial. Arch Phys Med Rehabil. 2017;98(11):2253-64.
2. Li D, Yang Z, Kang P, Xie X. Home-Based Compared with Hospital-Based Rehabilitation Program for Patients Undergoing Total Knee Arthroplasty for Osteoarthritis: A Systematic Review and Meta-analysis of Randomized Controlled Trials. Am J Phys Med Rehabil. 2017 Jun;96(6):440-7.
3. Austin MS, Urbani BT, Fleischman AN, Fernando ND, Purtill JJ, Hozack WJ, et al. Formal Physical Therapy After Total Hip Arthroplasty Is Not Required: A Randomized Controlled Trial. J Bone Joint Surg Am. 2017 Apr 19;99(8):648-55.
4. Florez-García M, García-Pérez F, Curbelo R, Pérez-Porta I, Nishishinya B, Rosario Lozano MP, et al. Efficacy and safety of home-based exercises versus individualized supervised outpatient physical therapy programs after total knee arthroplasty: a systematic review and meta-analysis. Knee Surg Sports Traumatol Arthrosc. 2017 Nov;25(11):3340-53.

2. FRACTURED HIP[1-3]

It is estimated that each year around 50,000 proximal femur fractures occur in older people in Spain, with a growing trend due to population aging; this represents almost 3% of hospital spending in Spain, according to data from the Ministry of Health[4-6]. Patients affected by hip fracture are often fragile patients with comorbidities, with a high risk of complications, functional deterioration, and hospitalization due to the injury, in addition to presenting a mortality of around 20-30% one year after the fracture.

REFERENCES

1. American Academy of Orthopaedic Surgeons. Management of Hip Fractures in the Elderly [Internet]. 1st ed. Rosemont, IL: American Academy of Orthopaedic Surgeons; Available from: http://www.aaos.org/Research/guidelines/HipFxGuideline_rev.pdf.
2. National Clinical Guideline Centre (UK). The Management of Hip Fracture in Adults [Internet]. London: Royal College of Physicians (UK); 2011. Available from: http://www.ncbi.nlm.nih.gov/books/NBK83014/.
3. Bhandari M, Swiontkowski M. Management of Acute Hip Fracture. N Engl J Med. 2017 Nov 23;377(21):2053-62.
4. Azagra R, López-Expósito F, Martin-Sánchez JC, Aguyé A, Moreno N, Cooper C, et al. Changing trends in the epidemiology of hip fracture in Spain. Osteoporos Int. 2014 Apr;25(4):1267-74.
5. Instituto de Información Sanitaria. Estadísticas Comentadas: La Atención a la Fractura de Cadera en los Hospitales del SNS [Internet]. Madrid: Ministerio de Sanidad y Política Social; 2010. Available from: http://www.msssi.gob.es/estadEstudios/estadisticas/docs/Estadisticas_comentadas_01.pdf.

6. Sáez López P, González Montalvo JI, Gómez Campelo, P, Ojeda Thies C. Registro Nacional Fracturas de Cadera. Informe Anual 2018 [Internet]. Registro Nacional Fracturas de Cadera; 2019. Available from: http://rnfc.es/wp-content/uploads/2019/11/Informe-Anual-RNFC-2018-1.pdf.

Pre-operative regional analgesia

Administering regional anesthesia by fascia iliac or femoral block reduces preoperative pain and may reduce the incidence of delirium and the need for opioids[1-3].

9. Regional analgesia is recommended to control preoperative pain in patients with hip fractures.

Nivel de evidencia alto. Grado de recomendación fuerte.

REFERENCES

1. Fletcher AK, Rigby AS, Heyes FLP. Three-in-one femoral nerve block as analgesia for fractured neck of femur in the emergency department: a randomized, controlled trial. Ann Emerg Med. 2003 Feb;41(2):227-33.
2. Foss NB, Kristensen BB, Bundgaard M, Bak M, Heiring C, Virkelyst C, et al. Fascia iliaca compartment blockade for acute pain control in hip fracture patients: a randomized, placebo-controlled trial. Anesthesiology. 2007 Apr;106(4):773-8.
3. Steenberg J, Møller AM. Systematic review of the effects of fascia iliaca compartment block on hip fracture patients before operation. Br J Anaesth. 2018 Jun;120(6):1368-80.

Preoperative traction

No differences between groups have been demonstrated when comparing patients with preoperative skin traction versus without traction, in terms of pain reduction and analgesic needs. The application of traction can be painful, especially if it is a skeletal traction. Preoperative traction can make nursing care difficult and facilitate the appearance of pressure ulcers[1-3].

10. The routine use of preoperative traction is not recommended in patients with hip fractures.

Nivel de evidencia moderado. Grado de recomendación fuerte.

REFERENCES

1. Handoll HH, Queally JM, Parker MJ. Pre-operative traction for hip fractures in adults. Cochrane Database Syst Rev. 2011 Dec 7;(12):CD000168.
2. Endo J, Yamaguchi S, Saito M, Itabashi T, Kita K, Koizumi W, et al. Efficacy of preoperative skin traction for hip fractures: a single-institution prospective randomized controlled trial of skin traction versus no traction. J Orthop Sci. 2013 Mar;18(2):250-5.
3. Tosun B, Aslan O, Tunay S. Preoperative position splint versus skin traction in patients with hip fracture: An experimental study. Int J Orthop Trauma Nurs. 2018 Feb;28:8-15.

Surgical delay

Patients should be operated on within the first 48 hours after admission. Correctable comorbidities will be identified and treated in those cases where necessary,

such as anemia, anticoagulation, depletion volume disorders, fluid and electrolyte disorders, uncontrolled diabetes, uncontrolled acute heart failure, ischemia or correctable cardiac arrhythmias, acute respiratory tract infections, or exacerbations of chronic lung diseases[1-6].A weak association has been observed between early surgery and lower postoperative mortality, and a stronger one with a lower rate of complications and shorter hospital stays.

11. Surgical management is recommended within the first 48 hours of admission.

Nivel de evidencia moderado. Grado de recomendación fuerte.

REFERENCES

1. Griffiths R, Alper J, Beckingsale A, Goldhill D, Heyburn G, Holloway J, et al. Management of proximal femoral fractures 2011. Anaesthesia. 2012;67(1):85-98.
2. Shiga T, Wajima Z, Ohe Y. Is operative delay associated with increased mortality of hip fracture patients? Systematic review, meta-analysis, and meta-regression. Can J Anaesth. 2008 Mar;55(3):146-54.
3. Simunovic N, Devereaux PJ, Sprague S, Guyatt GH, Schemitsch E, Debeer J, et al. Effect of early surgery after hip fracture on mortality and complications: systematic review and meta-analysis. CMAJ. 2010 Oct 19;182(15):1609-16.
4. Bretherton CP, Parker MJ. Early surgery for patients with a fracture of the hip decreases 30-day mortality. Bone Joint J. 2015 Jan;97-B(1):104-8.
5. Al-Ani AN, Samuelsson B, Tidermark J, Norling A, Ekström W, Cederholm T, et al. Early operation on patients with a hip fracture improved the ability to return to independent living. A prospective study of 850 patients. J Bone Joint Surg Am. 2008 Jul;90(7):1436-42.
6. Berber R, Boulton C, Moran C. Delay to surgery in hip fracture patients: effect on mortality, length of stay, and post-operative morbidity. Injury Extra. 2010 Dec;41(12):173.

Anesthetic technique

Both general and spinal anesthesia carry risks and benefits, which must be considered individually. A clear difference in mortality between the two types of anesthesia has not been demonstrated, although the studies use different follow-up times[1-2]. Some studies suggest a difference in the rate of postoperative complications such as postoperative delirium in favor of neuraxial anesthesia, although the level of sedation of the patient and maintenance of cerebral perfusion seems to have a greater influence[1-4]. The most appropriate technique should be used for each case, monitoring sedation and avoiding hypotension.

12. Both general and spinal anesthesia can be offered in patients with hip fracture.

Moderate level of evidence. Strong recommendation.

REFERENCES

1. Van Waesberghe J, Stevanovic A, Rossaint R, Coburn M. General vs. neuraxial anaesthesia in hip fracture patients: a systematic review and meta-analysis. BMC Anesthesiol. 2017 28;17(1):87.
2. Zheng X, Tan Y, Gao Y, Liu Z. Comparative efficacy of Neuraxial and general anesthesia for hip fracture surgery: a meta-analysis of randomized clinical trials. BMC Anesthesiol. 2020 Jun 30;20(1):162.

3. Chen DX, Yang L, Ding L, Li SY, Qi YN, Li Q. Perioperative outcomes in geriatric patients undergoing hip fracture surgery with different anesthesia techniques: A systematic review and meta-analysis. Medicine (Baltimore). 2019 Dec;98(49):e18220.
4. Sieber FE, Neufeld KJ, Gottschalk A, Bigelow GE, Oh ES, Rosenberg PB, et al. Effect of Depth of Sedation in Older Patients Undergoing Hip Fracture Repair on Postoperative Delirium: The STRIDE Randomized Clinical Trial. JAMA Surg. 2018 01;153(11):987-95.

Postoperative rehabilitation

Several studies support the benefit of intensive rehabilitation exercises in older patients with hip fractures, with an improvement in functional results, mobility, and autonomy to carrying out basic activities of daily life as well as improving quality of life. It is not clear what type of components the rehabilitation protocol should include, although improvements have been demonstrated with both resistance and balance exercises[1-4].

13. Mobilization should be offered the day after surgery and early rehabilitation to patients operated on for hip fracture.

Nivel de evidencia alto. Grado de recomendación fuerte.

REFERENCES

1. Diong J, Allen N, Sherrington C. Structured exercise improves mobility after hip fracture: a meta-analysis with meta-regression. Br J Sports Med. 2016 Mar;50(6):346-55.
2. Mangione KK, Craik RL, Palombaro KM, Tomlinson SS, Hofmann MT. Home-based leg-strengthening exercise improves function 1 year after hip fracture: a randomized controlled study. J Am Geriatr Soc. 2010 Oct;58(10):1911-7.
3. Beckmann M, Bruun-Olsen V, Pripp AH, Bergland A, Smith T, Heiberg KE. Effect of exercise interventions in the early phase to improve physical function after hip fracture - A systematic review and meta-analysis. Physiotherapy. 2020; 108:90-7.
4. Chudyk AM, Jutai JW, Petrella RJ, Speechley M. Systematic review of hip fracture rehabilitation practices in the elderly. Arch Phys Med Rehabil. 2009 Feb;90(2):246-62.

Postoperative load

Given the poor functional reserve of many hip fracture patients, any prescribed limitations of load and mobility management can significantly compromise post-operative care and prolong hospital stay, as well as having the potential to compromise independence, discharge destination, and functional recovery. Various studies have been unable to demonstrate a higher rate of postoperative complications in those patients with no restricted post-operative load[1-3].

14. Loading is recommended in patients undergoing hip fracture.

Moderate level of evidence. Strong recommendation.

REFERENCES

1. Warren J, Sundaram K, Anis H, McLaughlin J, Patterson B, Higuera CA, et al. The association between weight-bearing status and early complications in hip fractures. Eur J Orthop Surg Traumatol. 2019 Oct;29(7):1419-27.

2. Pfeufer D, Zeller A, Mehaffey S, Böcker W, Kammerlander C, Neuerburg C. Weight-bearing restrictions reduce postoperative mobility in elderly hip fracture patients. Arch Orthop Trauma Surg. 2019 Sep;139(9):1253-9.
3. Ottesen TD, McLynn RP, Galivanche AR, Bagi PS, Zogg CK, Rubin LE, et al. Increased complications in geriatric patients with a fracture of the hip whose postoperative weight-bearing is restricted: an analysis of 4918 patients. Bone Joint J. 2018;100-B(10):1377-84.

Multidisciplinary orthogeriatric management

Multidisciplinary care should be offered to older patients affected with hip fractures, including, in addition to traumatologists, clinical specialists (geriatricians and / or clinicians from other specialties), rehabilitators, anesthetists, nurses, therapists, etc. It has been shown that orthogeriatric multidisciplinary management has a slight effect on mortality and hospital stay, but more in favor of functional recovery and autonomy for basic activities of daily life. Its influence on readmission rate or hospitalization after the fracture is not so clear[1-5].

15. Orthogeriatric care is recommended within a multidisciplinary team for patients with a fragility hip fracture.

Moderate level of evidence. Strong recommendation.

REFERENCES

1. Mukherjee K, Brooks SE, Barraco RD, Como JJ, Hwang F, Robinson BRH, et al. Elderly adults with isolated hip fractures- orthogeriatric care versus standard care: A practice management guideline from the Eastern Association for the Surgery of Trauma. J Trauma Acute Care Surg. 2020 Feb;88(2):266-78.
2. Eamer G, Taheri A, Chen SS, Daviduck Q, Chambers T, Shi X, et al. Comprehensive geriatric assessment for older people admitted to a surgical service. Cochrane Database Syst Rev. 2018 Jan 31;1:CD012485.
3. Moyet J, Deschasse G, Marquant B, Mertl P, Bloch F. Which is the optimal orthogeriatric care model to prevent mortality of elderly subjects post hip fractures? A systematic review and meta-analysis based on current clinical practice. Int Orthop. 2019;43(6):1449-54.
4. Grigoryan KV, Javedan H, Rudolph JL. Orthogeriatric care models and outcomes in hip fracture patients: a systematic review and meta-analysis. J Orthop Trauma. 2014 Mar;28 (3):e49-55.
5. Kammerlander C, Roth T, Friedman SM, Suhm N, Luger TJ, Kammerlander-Knauer U, et al. Ortho-geriatric service-a literature review comparing different models. Osteoporos Int. 2010 Dec;21(Suppl 4):S637-646.

8. Indicator Chart

En este apartado se exponen indicadores generales para el an9álisis de la calidad del proceso asistencial. No se han incluido estándares pues, dentro de la cirugía abdominal, existen diversos procedimientos quirúrgicos con resultados diferentes. Asimismo, en muchos de los indicadores de proceso no es posible encontrar references.

8.1. PROCES INDICATORS.

COVERAGE QUALITY

$$\frac{\text{Patients that comply with inclusion criteria and have been included in RICA}}{\text{Patients in RICA}} \times 100$$

PROCEDURE QUALITY

$$\frac{\text{Number of patients that comply with RICA's inclusion criteria}}{\text{Patients in RICA}} \times 100$$

PREOPERATORY INFORMATION

$$\frac{\text{Patients in RICA that were given verbal and written information*}}{\text{Patients in RICA}} \times 100$$

PREOPERATORY ASSESMENT

$$\frac{\text{Patients in RICA with adequate preoperatory screening*}}{\text{Patients in RICA}} \times 100$$

NUTRITIONAL RISK SCREENING

$$\frac{\text{Patients in RICA with adequate nutritional screening*}}{\text{Patients in RICA}} \times 100$$

PREOPERATORY ANEMIA ASSESMENT

$$\frac{\text{Patients in RICA with hemoglobin > 13 g/dl}}{\text{Patients included in a RICA protocol}} \times 100$$

PREOPERATORY FASTING AND CARBOHYDRATE DRINK

$$\frac{\text{Patients in RICA with adequate preoperatory fasting time and diet*}}{\text{Patients included in a RICA protocol}} \times 100$$

TROMBOEMBOLISM PROPHYLAXIS

$$\frac{\text{Patients in RICA with adequate thromboembolism prophylaxis*}}{\text{Patients included in a RICA protocol}} \times 100$$

ANTIBIOTIC PROPHYLAXIS

$$\frac{\text{Patients in RICA with an adequate antibiotic prophylaxis prescription*}}{\text{Pacientes included in a RICA protocol}} \times 100$$

SURGICAL APPROACH

$$\frac{\text{Patients in RICA undergoing minimally invasive surgical approach*}}{\text{Patients included in a RICA protocol}} \times 100$$

FLUID MANAGEMENT

$$\frac{\text{Patients in RICA with adequate perioperative fluid therapy*}}{\text{Patients included in a RICA protocol}} \times 100$$

HYPOTHERMIA PREVENTION

$$\frac{\text{Patients in RICA with intraoperatory body temperature monitoring*}}{\text{Patients included in RICA pathway}} \times 100$$

NASOGASTRIC TUBE

$$\frac{\text{Patients in RICA with nasogastric tube}}{\text{Patients included in RICA pathway}} \times 100$$

ANALGESIA

$$\frac{\text{Patients in RICA with adequate analgesia*}}{\text{Patients included in a RICA pathway}} \times 100$$

NUTRITION SUPPORT

$$\frac{\text{Patients in RICA with adequate nutrition support*}}{\text{Patients included in RICA pathway}} \times 100$$

EARLY MOBILISATION

$$\frac{\text{Patients in RICA with adequate postoperative mobilisation*}}{\text{Patients included in RICA pathway}} \times 100$$

8.2. RESULT INDICATORS

CLINIC EFECTIVENESS

$$\frac{\text{Patients in RICA requiring reoperation due to bleeding}}{\text{Patients included in RICA pathway}} \times 100$$

$$\frac{\text{Patients in RICA requiring transfer to ICU}}{\text{Patients included in RICA pathway}} \times 100$$

$$\frac{\text{Patients in RICA requiring unscheduled hospital admission related to surgery within the first 30 postoperative days}}{\text{Patients included in RICA pathway}} \times 100$$

$$\frac{\text{Deceased patients in RICA within the first 30 postoperative days}}{\text{Patients included in RICA pathway}} \times 100$$

$$\frac{\text{Pacientes in RICA developing SSI* within the first 30 postoperative days}}{\text{Patients included in RICA pathway}} \times 100$$

**SSI: Surgical Site Infection*

EFICIENCY

$$\frac{\text{Patients in RICA discharged from hospital according to plan*}}{\text{Patients included in RICA pathway}} \times 100$$

PATIENT SATISFACTION

$$\frac{\text{Patients in RICA answering 'very satisfied' with the care received*}}{\text{Patients included in RICA pathway}} \times 100$$

**Defining specific criteria is needed for these indicators.*

9. Implementation Srategy

BACKGROUND

Since 2007, a committed group of specialists from our country, proposed to modify unsafe practices to undertake their surgical processes from the perspective of "primun non nocere". This Group is made up of healthcare professionals such as Anesthesiologists, Surgeons, Nurses, Endocrinologists / Nutritionists, Hematologists, Rehabilitators, Preventivists, Methodologists whose purpose is to carry out a safe perioperative clinical practice supported by Medicine Based on Scientific Evidence.

As a result of the work carried out, the Enhanced Recovery Pathway in Abdominal Surgery (RICA in Spanish) was born; sponsored by the Spanish Ministry of Health, Social Security and Equality and the Aragonese Institute of Health Sciences, and audited by Guía Salud; updated in 2020, incorporating other specialties in an attempt to cover most adult surgical procedures.

However, the obstacles stemming from a long-established tradition, as well as resistance to change, make the implementation of clinical protocols or pathways difficult, requiring a strategic plan for it. For this reason, it is particularly important to know the barriers and strengthen leadership when implementing them[1,2].

The implementation of a RCIA program implies the standardization of care and therapy; It reduces the variability of clinical practice and accustoms professionals to a protocolized practice, which generates greater security since it involves assuming orthodox behaviors in order to avoid errors or forgetfulness that can have deleterious effects on our patients. All this translates into better clinical results, better and safer quality of care, and an overall improvement in the well-being of our population in terms of health.

OBJECTIVES

With the goal of achieving a uniform, consensual and multicenter implementation of perioperative medicine programs/protocols stemming from the clinical pathway for Recovery Intensification for optimal Care in Adults surgery -“RICA”- we believe it is very necessary to generate strategic alliances between scientific societies and signatory agencies of this document and develop a homogeneous implantation plan (IMPRICA plan), with the following steps:

1. DISSEMINATION

Objective: distribution of knowledge and key points of the RICA pathway, so that it reaches the entire clinical environment (by specialties) of our country.

Desirable measures:

- Conducting seminars at the regional and local level.
- Participation in Conferences on Patient Safety, Change Management, variability of clinical practice.
- Planning of discussion panels and symposia in the outstanding National Congresses of the Societies involved in the document.
- Distribution of the appropriate documentation for both the professionals and the patients on the Via RICA, information documents on the procedures, demonstrative videos, ...
- Space on digital platforms for information (presentations, podcasts, webinars…).

2. MEETINGS AND SESSIONS IN AUTONOMOUS COMMUNITIES.

To involve the managing agents of the different autonomous communities, it would be desirable to organize meetings where senior managers of health ministries participate, inviting the different "stakeholders" of the area, to highlight the directors of management, medical and nursing management, those responsible for quality and training. These sessions should be promoted by the decision-making Bodies, preferably the Counselor or Director of Health Planning himself.

3. TRAINING

The possibility of organizing appropriately accredited training courses for different clinicians interested not only in the proper implementation of the Via RICA in their centers, but also in achieving the highest levels of quality care should be considered.

4. RICA NATIONAL REGISTRY

The creation of a National RCIA Registry is proposed to assess the degree of implementation in the different hospital centers, as well as to monitor the quality indicators proposed in this way.

1. Giménez-Júlvez T, Hernández-García I, Aibar-Remón C, Gutiérrez-Cía I, Febrel-Bordejé M.Culture of patient safety in directors and managers of a health service. Sanitary Gazette. 2017. 31: 423-426. DOI: 10.1016 / j.gaceta.2017.01.009).
2. Gramlich L, Nelson G, Nelson A, Lagendyk L, Gilmour LE, Wasylak T. Moving enhanced recovery after surgery from implementation to sustainability across a health system: a qualitative assessment of leadership perspectives. BMC Health Serv Res. 2020 Apr 26; 20 (1): 361. doi: 10.1186 / s12913-020-05227-0. PMID: 32336268).

10. Anexes

10.1. POSTOPERATIVE NAUSA AND VOMITING PROPHYLAXIS – APFEL SCALE

APFEL SCALE

POSTOPERATIVE NAUSA AND VOMITING PROPHYLAXIS

APFEL MODEL FOR RISK STRATIFICATION

RISK FACTORS	SCORE	RISK
Woman	1	Base line: 10%
Non smoker	1	1 point: 20%
Previous PONV or kinetosis history	1	2 points: 40%
Postoperative opioid usage	1	3 points: 60%
		4 points: 80%`
Low risk (0-1 point, 10-20%); moderate (2 points, 40%); high (3-4 points, 60-80%)		

10.2. PREOPERATIVE MANAGEMENT FOR ANEMIC PATIENTS

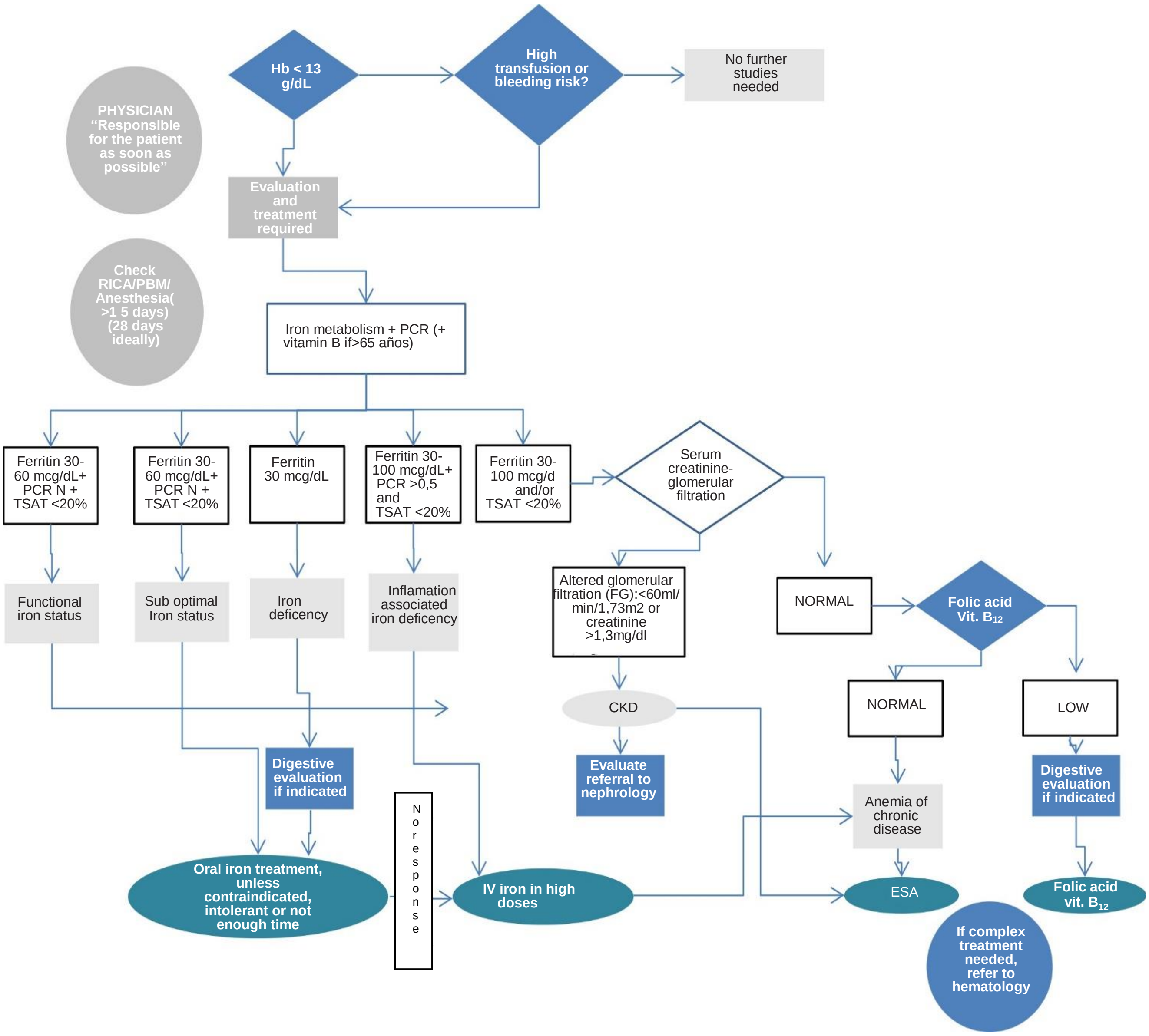

Hb: Hemoglobin
CKD: Chronic Kidney Disease
PCR: Polymerase Chain Reaction
TSAT: Transferrin Saturation
ESA: Erythropoiesis Stimulating Agents

10.3. NUTRITIONAL SCREENING ALGORITHM

Risk assesment ↓

Malnutrition risk

- Use validated screeining tools

Diagnostic evaluation ↓

Evaluation criteria

- Phenotypic
  - Voluntary weight loss
  - Low BMI
  - Muscle atrophy
- Etiologic
  - Low intake or assimilation rate
  - Disease/inflamatory process

Diagnosis ↓

Malnoursihment diagnosis acording to criteria

- At least 1 phenotypic and 1 etiologic criteria

10.4. PREOPERATIVE MANAGEMENT

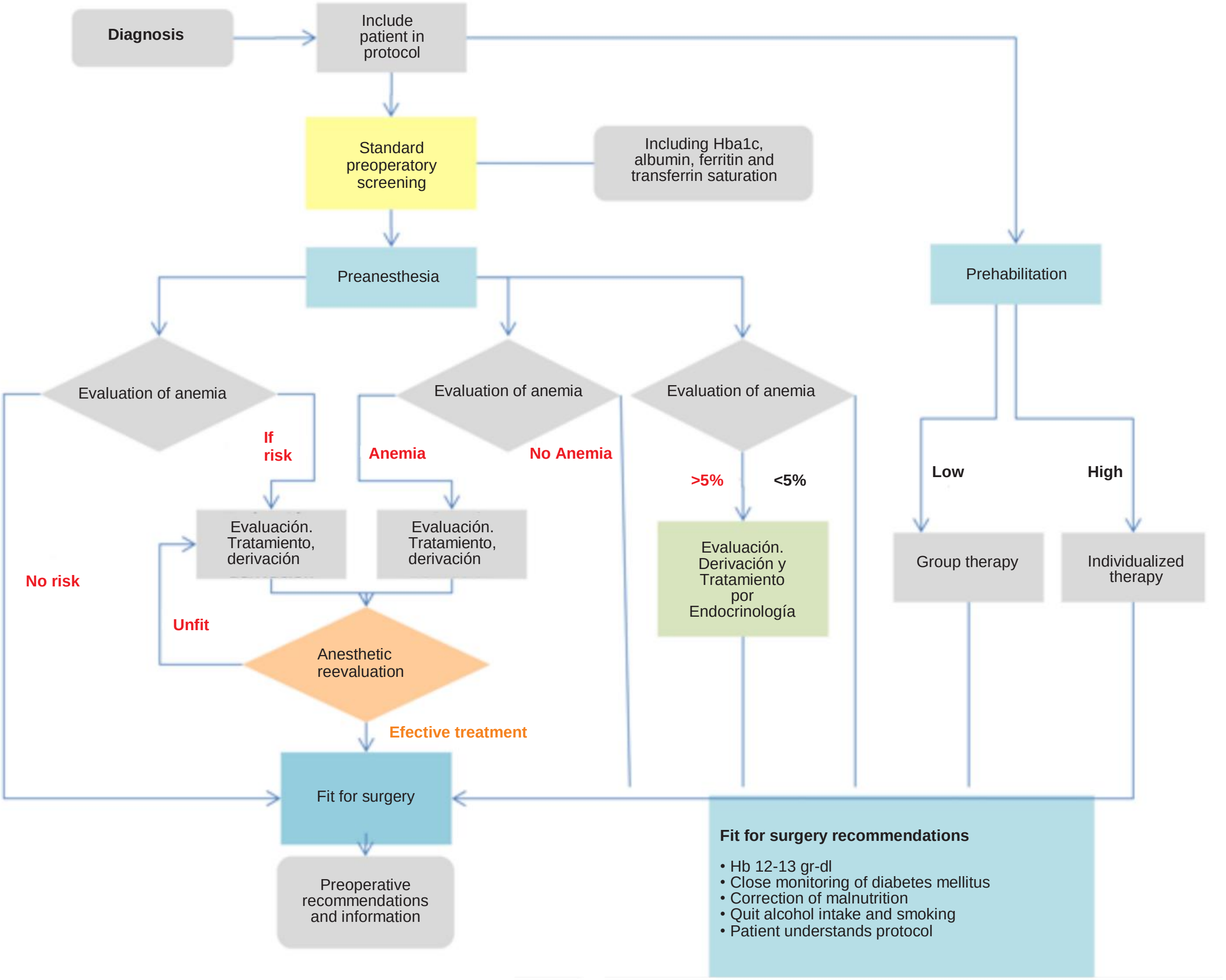

10.5. PATIENT INFORMATION

Recovery Intensification for optimal Care in Adult's surgery (RICA)

1. Introduction
2. Preparation at home / Pre-admission
3. During your hospital stay
4. Home discharge

INTRODUCTION

This Clinical Pathway for Recovery Intensification for optimal Care in Adult's surgery, called "RICA", in which you take part during your surgical intervention, is different from traditional treatment. It consists of the application of a series of measures to minimize the impact and repercussions that any surgical intervention implies, reduces possible complications, speeds up recovery and can even reduce hospital stay.

Your active collaboration as a patient and that of your family members or caretakers, as well as the completion of all its steps is essential for the proper functioning and success of this program.

There are three main stages:

1. *Preparation prior to admission. Since the need for a surgical intervention is decided with your doctor.*
2. *During your stay in the hospital.*
3. *Recommendations at discharge.*

The team of professionals that will assist you throughout this Clinical Pathway is trained to answer all your doubts and guide you in the development of each stage of the Program.

PREPARATION FOR ADMISSION

Prior preparation of the patient is essential and ensures that the patient is in the best possible condition, identifying personal risks in the preoperative period.

You will go to surgery, anesthesia, and nursing consultations to receive all the information necessary about the details of your intervention and the tasks that require your prior collaboration in this program.

Since you have made the decision to undergo surgery, you must commit to avoiding toxins such as alcohol and tobacco. It is important that you understand that all the effort you can put into reducing these habits will directly revert to a reduction in possible respiratory complications that you may suffer during the surgical process.

Surgery can increase the risk of respiratory complications. To prevent them, your nurse/physical therapist will teach you how to prepare your respiratory muscles. In addition, they will teach you how to use the incentive spirometer, to help you carry out respiratory exercises during the days prior to the intervention.

<u>Preoperative nutrition</u>. During the surgery, a high energy expenditure will be required, and it will be particularly important to have an adequate nutritional state to promote healing and the body's defense against infections.

To achieve a better preoperative nutritional status, a rich in proteins diet is recommended in addition to proper hydration, at least seven to ten days prior to surgery.

The night before the intervention, you can have solid food up to 6 hours before surgery and clear liquids (chamomile, juice, or sugar solution) up to 2 hours before surgery.

You will not be able to eat or drink anything 2 hours before surgery

You should not drink alcoholic beverages; alcohol is related to post-operative complications.

Exercise prior to surgery. Practicing moderate exercise before admission will contribute favorably to your later recovery. Your nurse will advise you on what type of activity you can practice depending on your physical condition.

DURING YOUR HOSPITAL STAY

After the surgery, the team of professionals who will take care of you will indicate what the steps of your recovery should be on a day-to-day basis. Remember that your collaboration and involvement is key in the proper progress of your evolution, do not hesitate to ask any questions you have or to let people know what discomfort you have.

To help prevent the possible complications associated to any surgery, we will work on three main fields:

1. *Early mobilization*
2. *Early oral feeding*
3. *Respiratory physiotherapy exercises*

EARLY MOBILIZATION

As valued in this program, the sooner you can move, the better results you will achieve, so we will ask you stand after surgery and wander before usual.

Your ideal progression would be the following:

Day of the intervention, the nursing staff will help you get out of bed to sit in your chair. You should try to sit out of bed for up to two hours. We know that this is a great effort, and it may seem hard, but you will see how your recovery will be faster. For example: The surgery paralyzes the intestine for a variable time that can be shortened if you get up and walk after the intervention and lengthened if you lie down.

The day after the intervention, you will be able to sit on the chair at intervals for up to six hours, in addition to walking short distances, around four sets of 60 meters.

Successive days You will continue to walk and attempt a steady progression.

The surgery paralyzes the intestine for a variable time that can be shortened if you stand up and walk after the intervention and lengthened if you lie down.

EARLY ORAL FEEDING

In this program we value nutrition very much, so that you can tolerate food as soon as possible, at the rate you need it.

On the same day of surgery, it is recommended to start drinking as soon as possible, it will be done progressively starting with small amounts and continue with other types of easy to digest foods provided there is good tolerance.

The day after the intervention, you will increase your fluid intake to 1.5 liters. Do not drink carbonated drinks.

The following days provided you are tolerating well; you will evolve to a more solid diet. Continue to drink liquids on a regular basis.

RESPIRATORY PHYSIOTHERAPY EXERCISES

In all surgery the risk of respiratory complications is increased due to bed rest, discomfort at the incision site, and other factors. The risk can be prevented through chest mobilization exercises, which you will perform with the incentive spirometer.

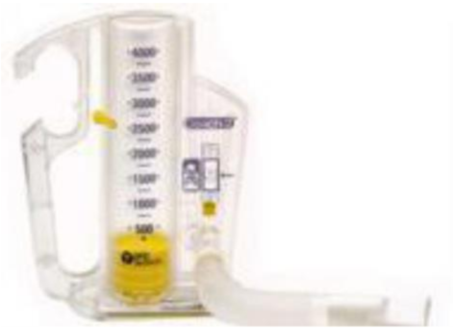

By doing these exercises you will:

- *Increase lung ventilation to prevent respiratory infections*
- *Increase the strength of the respiratory muscles*
- *Prevent respiratory secretions from accumulating*

Approximately 4-6 hours after the intervention, you can start using the incentive. The frequency of use will be every 2 hours for 10 minutes each time.

RECOMMENDATIONS AT DISCHARGE

The high level of planning behind RICA pathways means that all the practical support that you will need at home must be prepared.

If you have any doubts in handling, consult with healthcare personnel.

The planning prepared for you will be reviewed and validated by the doctors and nurses responsible for your discharge from the hospital.

Possibly, your probable discharge date will be communicated to you in advance by your doctor, this makes it easier for you to have everything you need ready to go home, or health center if required, with enough time.

Your discharge from the hospital is based on specific criteria and goals. When you achieve them, you will be discharged.

These criteria are:

- *Effective pain control with oral analgesics.*
- *Good oral tolerance to liquids and diet, without nausea or vomiting.*
- *Autonomy in mobility.*

If you need more information, do not hesitate to ask your doctor or the Unit nurse.

10.6. PATIENT SATISFACTION QUESTIONNAIRE

PATIENT SATISFACTION QUESTIONNAIRE

(RECOVERY INTENSIFICATION PROGRAM FOR ADULT'S SURGERY)

Dear patient:

We would like you to answer this questionnaire with the purpose of knowing your grade of satisfaction with the assistance given.

We would like to thank you for your interest and attention by accepting to answer these questions, helping us to improve our care.

The healthcare team.

General Data

Age: Gender: Male □ Female □

Education: None □ Primary □ Secondary □ Further □ Higher □

Information Before Surgery

How would you rate the information given to you by the SURGEON before surgery?

Excellent □ Good □ Average □ Bad □ Terrible □

How would you rate the information given to you by the ANESTHETIST before surgery?

Excellent □ Good □ Average □ Bad □ Terrible □

How would you rate the information given to you by the NURSES before surgery?

Excellent □ Good □ Average □ Bad □ Terrible □

Facilities and equipment

The visual appearance of the operating room was:

Excellent □ Good □ Average □ Bad □ Terrible □

Your room when taken to ward was:

Single □ Double □ Other □

Your room was:

Very comfortable □ Quite comfortable □ Average □ Uncomfortable □ Not comfortable at all □

Pain

What was your maximum level of pain during the first hours after surgery? (0 = no pain ⇨10 = unbearable pain)

0	1	2	3	4	5	6	7	8	9	10

Once at ward, what was your maximum level of pain after surgery? (0 = no pain ⇨10 = unbearable pain)

0	1	2	3	4	5	6	7	8	9	10

Postoperative feeding

After surgery, did you experience any nausa or vomiting? YES □ NO □

When you were told you had to eat and drink, you thought it was:

Too early □ Early □ On time □ Late □ Too late □

Early Mobilisation

When you were told you had to stand up, you thought it was:

Too early □ Early □ On time □ Late □

When you were told you had to walk, you thought it was:

Too early □ Early □ On time □ Late □

At Discharge

How would you rate the information and recommendations you received from the SURGEON at discharge?

Very good □ Good □ Average □ Bad □ Very Bad □ I was not informed □

How would you rate the information and recommendations you received from the NURSES at discharge?

Very good □ Good □ Average □ Bad □ Very Bad □ I was not informed □

Once at home, did you have to call the phone number you were given?

YES □ NO □ I was not given any □

Care given

How would you rate the care given by the SURGEON?

Excellent □ Good □ Average □ Bad □ Terrible □

How would you rate the care given by the ANESTHETIST?

Excellent □ Good □ Average □ Bad □ Terrible □

How would you rate the care given by the NURSES?

Excellent □ Good □ Average □ Bad □ Terrible □

How would you rate the care given by the REST OF THE STAFF?

Excellent □ Good □ Average □ Bad □ Terrible □

Expertise, healthcare coordination and outcome

How would you rate the SURGEONS' professional expertise?

Very highy □ High □ Average □ Low □ Very Low □

How would you rate the ANESTHETIST'S professional expertise?

Very highy □ High □ Average □ Low □ Very Low □

How would you rate the NURSES' professional expertise?

Very highy □ High □ Average □ Low □ Very Low □

How would you rate the professional expertise of the REST OF THE STAFF?

Very highy □ High □ Average □ Low □ Very Low □

Regarding coordination between healthcare professionals, you thought they were:

Very coordinated □ Quite coordinated □ Average □ Poorly coordinated □ Not coordinated □

How would you rate the outcome of your surgery?

Excellent □ Good □ Average □ Bad □ Terrible □

If you had to undergo surgery again, would you use this same protocol? YES □ NO □

General Satisfaction

Generally speaking, how satisfied are you with the whole process?

Very satisfied □ Quite satisfied □ Not satisfied nor unsatisfied □ Quite unsatisfied □ Not satisfied □

The best thing for you was:

The worst thing for you was:

Please, list the improvements you would include in this protocol:

Other comments:

Thanks for your collaboration.

10.7. ABBREVIATIONS

NSAIDs	Non-Steroidal Anti-Inflamatory Drugs
BIS	Bispectral Index
NMB	NeuroMuscular Blockade
MAS	Major Abdominal Surgery
MIS	Minimally Invasive Surgery
CVC	Central Venous Catheter
DXA	Dual-energy X-ray Absorptiometry
AKI	Acute Kidney Injury
RCT	Randomized Controlled Trial
EKG	Electrocardiogram
ESA	Erythropoiesis Stimulating Agents
$EtCO_2$	End-Tidal Carbon Dioxide.Capnography
VAS	Visual Analogue Scale
GDFT	Goal-Directed Fluid Therapy
FiO2	Fraction of Inspired Oxygen
LMWH	Low-Molecular-Weight Heparin
UFH	UnFractioned Heparin
CI	Cardiac Index
IMPRICA	IMPlementation of RICA pathway
CKD	Chronic Kidney Disease
SSI	Surgical Site Infection
PCP	Primary Care Physician
PONV	Post Operative Nausa and Vomiting
OEBP	Other Evidence-Based Products
NIAP	Non-Invasive Arterial Pressure
MBP	Mechanical Bowel Preparation
IRP	Intensified Recovery Program
IR	Intensified Recovery
RICA	Recovery Intensification for optimal Care in Adult’s surgery

OSAS	Obstructive Sleep Apnoea Syndrome
NGT	NasoGastric Tube
TOF	Train Of Four
ABT	Allogenic Blood Transfusion
TSAT	Transferrin SATuration
PAR	Post-Anesthesia Recovery unit
SV	Stroke Volume
VVS	Stroke Volume Variation

MINISTERIO
DE SANIDAD
