# Supplementary material for: Advancing abdominal surgery recovery implementation: a unified framework for intensified recovery protocols by the EUropean PErioperative MEdical Networking collaborative
Source: Front Surg. 2026 May 18;13:1827678. doi: 10.3389/fsurg.2026.1827678 (PMC13223102; doi:10.3389/fsurg.2026.1827678)
Supplement: Supplementary file 18 [file Datasheet9.pdf]

Co-funded by the Erasmus+ Programme of the European Union

EUPEMEN
European Perioperative Medical Networking

EUPEMEN: SUMMARY MULTIPLIER EVENT (ME)

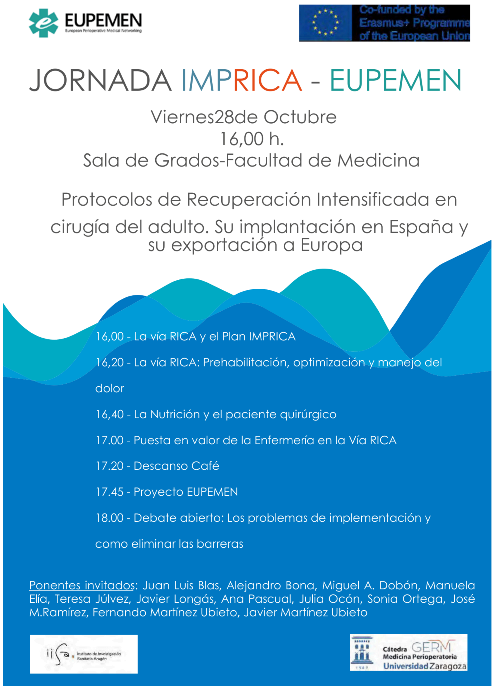

PROJECT: Erasmus+ Eupemen- 2020-1-ES01-KA203-082681

TITLE: Jornada Imprica - Eupemen

HOST PARTNER: IISA

DATE AND TIMETABLE: 28/10/2022 (16:00 – 19:00)

PLACE: Sala de Grados-Facultad de Medicina

The AGENDA of the event was as followed:
Seminar IMPRICA - EUPEMEN.
Intensified Recovery Protocols in adult surgery. Its implementation in Spain and its export to Europe.
- The RICA pathway and the IMPRICA Plan
- The RICA pathway: Prehabilitation, optimization and pain management
- Nutrition and the surgical patient
- Valuation of Nursing in the Vía RICA
- Coffee break
- EUPEMEN Project
- Open debate: Implementation problems and how to remove barriers

Invited speakers: Juan Luis Blas, Alejandro Bona, Miguel A. Dobón, Manuela.Elía, Teresa Júlvez, Javier Longás, Ana Pascual, Julia Ocón, Sonia Ortega, José.M. Ramirez, Fernando Martinez Ubieto, Javier Martinez Ubieto

Co-funded by the Erasmus+ Programme of the European Union

EUPEMEN
European Perioperative Medical Networking

Il Programma *Enhanced Recovery After Surgery* (ERAS) in Chirurgia Colorettale

Un approccio multidisciplinare e interprofessionale

Venerdì 28 Ottobre 2022
14.30 – 18.30

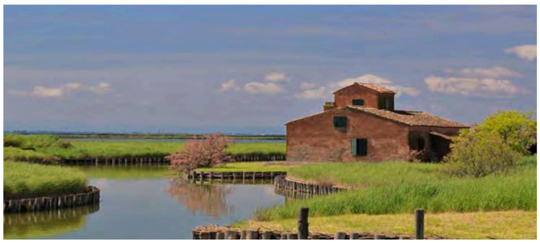

OSPEDALE DEL DELTA
SALARIUNIONI PIANOTERRA
Via Valle Oppio 2, 44023
Lagosanto (FERRARA)

Crediti ECM per medici e infermieri

PROJECT: Erasmus+ Eupemen- 2020-1-ES01-KA203-082681

TITLE: Il Programma Enhanced Recovery After Surgery ((ERAS)) in Chirurgia Colorettale

Un approccio multidisciplinare e interprofessionale.

HOST PARTNER: AUSLFE

DATE AND TIMETABLE: 28/10/2022

PLACE: OSPEDALE DEL DELTA, SALA RIUNIONI PIANO TERRA

Via Valle Oppio 2, 44023, Lagosanto (FERRARA)

The AGENDA of the event was as followed:

- Registration of participants
- Presentation of the course (Erminio RIGHINI, MD)
- Why an ERAS programme? (Carlo FEO, MD, Prof)
- Pre-operative preparation (Nicolò FABBRI, MD - Lisa UCCELLATORI, RN)
- Patient management in the operating room (Luca BAGNOLI, MD)
- Postoperative management in hospital (Filippo ADVANCE, RN)
- The coach for respiratory gymnastics (Luisa GRAZIANO, PT)
- Results of the ERAS program in the Province of Ferrara (Carlo FEO, MD, Prof)
- The EUropean PErioperative MEdical Network project (EUPEMEN) (Antonio PESCE, MD, PhD)
- Discussion
- Closing of works

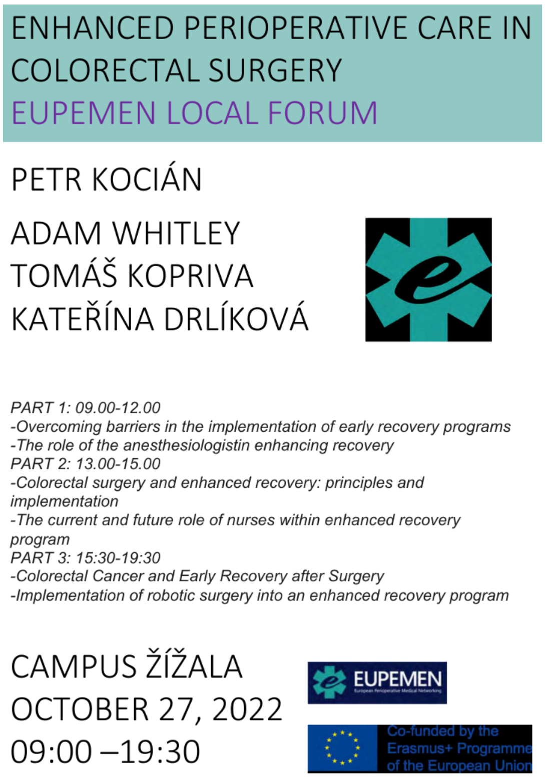

PROJECT: Erasmus+ Eupemen- 2020-1-ES01-KA203-082681

TITLE: ENHANCED PERIOPERATIVE CARE IN COLORECTAL SURGERY

HOST PARTNER: CUNI

DATE AND TIMETABLE: 27/10/2022 (9:00 – 19:30)

PLACE: CAMPUS ŽÍŽALA

AGENDA OF THE EVENT

ENHANCED PERIOPERATIVE CARE IN COLORECTAL SURGERY - EUPEMEN LOCAL FORUM

PART 1: 09.00-12.00

- Overcoming barriers in the implementation of early recovery programs
- The role of the anesthesiologist in enhancing recovery

PART 2: 13.00-15.00

- Colorectal surgery and enhanced recovery: principles and implementation
- The current and future role of nurses within enhanced recovery program

PART 3: 15:30-19:30

- Colorectal Cancer and Early Recovery after Surgery
- Implementation of robotic surgery into an enhanced recovery program

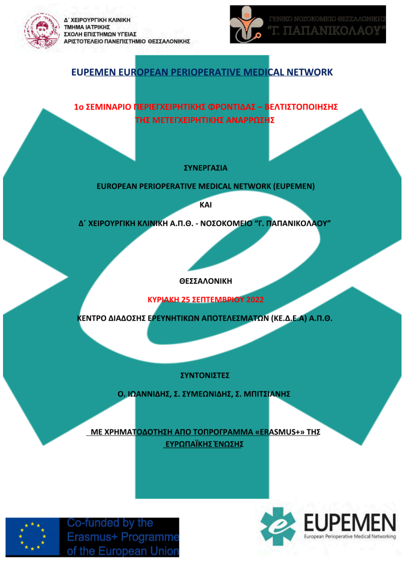

PROJECT: Erasmus+ Eupemen- 2020-1-ES01-KA203-082681

TITLE: 1o Σεμινάριο Περιεγχειρητικής Φροντίδας – Βελτιστοποίησης της Μετεγχειρητικής Ανάρρωσης

HOST PARTNER: GPAP

DATE AND TIMETABLE: 25/09/2022

PLACE: Thessaloniki

AGENDA OF THE EVENT
08:30 – 09:00 Arrival – Registration
09:00 – 09:10 Opening – Introduction: S. Angelopoulos
09:10 - 09:30 EUPEMEN project: objectives and implementation - Distance learning platform and recording platform: O. Ioannidis
09:30 – 10:30 Basic Principles of Optimization of Postoperative Resuscitation
Chair: E. Kotidis, S. Symeonidis
1. Principles and philosophy of Postoperative Resuscitation Optimization Programs: O. Ioannidis
2. Prehabilitation programs: V. Foutsitzis
3. Controversial issues in era protocols: a. Non-steroidal anti-inflammatory drugs and anastomosis, b. Mechanical bowel preparation: N. Ouzounidis
10:30 – 11:30 Multifactorial approach to optimization of postoperative resuscitation
President: M.G. Pramateutakis, O. Ioannidis
1. Constitutive elements of protocols in surgery: S. Symeonidis
2.Constitutive elements of the Protocols in Anesthesiology: D. Konstantinidis
3. Constitutive elements of Nursing Protocols: M. Demirtzoglou
11:30-12:00 Rest
12:00 – 14:00 Specialized protocols
Chairman: I. Mantzoros, N. Ouzounidis
1. Protocol in esophagectomy: S. bitsianis
2. Protocol in gastrectomy: S. Symeonidis
3. Protocol in Bariatrics: E. Kotidis
4. Colectomy protocol: M.G. pramateutakis

5. Protocol on hepatectomy: I. Mantzoros
6. Protocols in emergency surgery: O. Ioannidis
14:00 - 14:30: End – Conclusions – Observations – Discussion – Delivery of certificates

Título: “**EL VALOR DE LA ADAPTACIÓN PERIOPERATORIA DEL PACIE**
PREHABILITACIÓNYREHABILITACIÓNMULTIMODAL (ERASyEUPEMEN)ENCÁNCER COLORRECTAL”

Jueves 27 de octubre de 2022
8:30 horas
Salón de Actos del Hospital General Universitario de Elche

SERVICIO DE CIRUGÍAGENERAL

Moderador: Dr. Luis Sánchez Guillén. Tutor de Residentes.

Ponentes:

- - -
-

Miguel Ángel Pérez Sánchez	R2CirugíaGeneral
Cristina Lillo García	R5CirugíaGeneral
ÁngelaSierraCuenca	R3Anestesiologíay Reanimación
María Rodríguez Morote	R2 Farmacia Hospitalaria

PROJECT: Erasmus+ Eupemen- 2020-1-ES01-KA203-082681

TITLE: “EL VALOR DE LA ADAPTACIÓN PERIOPERATORIA DEL PACIENTE UIRÚRGICO. PREHABILITACIÓN Y REHABILITACIÓN MULTIMODAL (ERAS Y EUPEMEN) EN CÁNCER COLORRECTAL”

HOST PARTNER: UMH

DATE AND TIMETABLE: 27/10/2022 (8:30)

PLACE: Salón de Actos del Hospital General Universitario de Elche

AGENDA OF THE EVENT

“The Value of Perioperative Optimization of the Surgical Patient. Multimodal Prehabilitation and Rehabilitation (ERAS and EUPEMEN) in Colorectal Cancer.”

Moderator: Dr. Luis Sánchez Guillén

Invited speakers:

- Miguel Ángel Pérez Sánchez R2 Cirugía General
- Cristina Lillo García R5 Cirugía General
- Ángela Sierra Cuenca R3Anestesiología y Reanimación
- María Rodríguez Morote R2 Farmacia Hospitalaria
